# A machine learning technique for identifying DNA enhancer regions utilizing CIS-regulatory element patterns

**DOI:** 10.1038/s41598-022-19099-3

**Published:** 2022-09-07

**Authors:** Ahmad Hassan Butt, Tamim Alkhalifah, Fahad Alturise, Yaser Daanial Khan

**Affiliations:** 1 Department of Computer Science, School of Systems and Technology, University of Management and Technology, Lahore, Pakistan; 2 Department of Computer, College of Science and Arts in Ar Rass, Qassim University, Ar Rass, Saudi Arabia

**Keywords:** Computational biology and bioinformatics, Classification and taxonomy, Computational models, Machine learning

## Abstract

Enhancers regulate gene expression, by playing a crucial role in the synthesis of RNAs and proteins. They do not directly encode proteins or RNA molecules. In order to control gene expression, it is important to predict enhancers and their potency. Given their distance from the target gene, lack of common motifs, and tissue/cell specificity, enhancer regions are thought to be difficult to predict in DNA sequences. Recently, a number of bioinformatics tools were created to distinguish enhancers from other regulatory components and to pinpoint their advantages. However, because the quality of its prediction method needs to be improved, its practical application value must also be improved. Based on nucleotide composition and statistical moment-based features, the current study suggests a novel method for identifying enhancers and non-enhancers and evaluating their strength. The proposed study outperformed state-of-the-art techniques using fivefold and tenfold cross-validation in terms of accuracy. The accuracy from the current study results in 86.5% and 72.3% in enhancer site and its strength prediction respectively. The results of the suggested methodology point to the potential for more efficient and successful outcomes when statistical moment-based features are used. The current study's source code is available to the research community at https://github.com/csbioinfopk/enpred.

## Introduction

In cellular biology, regulation of transcription is performed to recruit elongation factors or RNA polymerase II initiation. This is mainly achieved at specific sequences of DNA by binding transcriptional factors (TFs). Transcription initiation sites are harbored by promoter regions which are the most studied sites in DNA^1^. Some DNA sequences have multiple transcription factor binding sites and are near or far away from promoter regions. Such DNA segments are denoted as enhancers^2,3^.The transcription of genes is boosted by enhancers which influence various cellular activities such as cell carcinogenesis and virus activity, tissue specificity of gene expression, differentiation and cell growth, regulation and gene expression and develop relationship between such processes very closely^4^.

Enhancers can be a short (50–1500 bp) segment of DNA and situated 1Mbp (1,000,000 bp) distance away from a gene. Sometimes they can even exist in different chromosomes^5,6^. On the other hand, promoters are located near the start of the transcription sites of a gene. Due to this fact of locational difference between promoters and enhancers, the task of enhancer’s prediction is highly difficult and challenging than promoters^7^. Many human diseases like inflammatory bowel disease, disorder and various cancers have been linked to this genetic variation in enhancers^8–11^.

A DNA segment characterized as the first enhancer, reported 40 years ago, increased the transcription of β-globin gene during a transgenic assay inside the virus genome of SV40 tumor^12^. Scientific research during recent past has discovered that enhancers have many subgroups such as weak and strong enhancers, latent enhancers and poised enhancers^13^. Prediction of enhancers and their subgroups is an interesting area of research as they are considered important in disease and evolution. In higher classification of eukaryotes, transcription factor repertoire, diverse in nature, binds to enhancers^14^. This process of binding orchestrates many cellular events that are critical to the cellular system. Some of the events that are coordinated through this binding are maintenance of the cell identity, differentiation and response to stimuli^15,16^.

In the past, purely experimental techniques were being relied upon for the prediction of enhancers. Pioneering works in enhancer prediction was proposed in^4,17^. The former was to use combinations such as transcription factor, P300^18^, with enhancers to identify them. This method would usually under-detect or miss the concerned targets. This has resulted in high failure rates because all enhancers do not have transcription factor occupations. The latter was to utilize DNase I hypersensitivity for enhancer predictions. Hence, this led to a high false-positive rate as many other DNA segments, which were non-enhancers, were detected incorrectly as enhancers. Although, genome-wide mapping techniques of histone modifications^1,19–23^ could improve the aforesaid deficiencies in the prediction of promoters and enhancers, but they are time consuming and expensive.

Several bioinformatics tools have been developed for rapid and cost effective classification of enhancers in genomics. CSI-ANN^21^ used data transformations efficiently to formulate the samples and predict using Artificial-Neural-Network (ANN) classifications. EnhancerFinder^1^ incorporated evolutionary conservation information features into sample formulation combined with a multiple kernel learning algorithm as a classifier. RFECS^23^applied random forest algorithm for improvements in detection methods. EnhancerDBN^24^ used deep belief networks for enhancer predictions. BiRen^25^ increased the predictive performance by utilizing deep learning based method. By utilizing these bioinformatics tools, enhancer detection can be achieved by the research community. Formed using many different large sub-groups of functional elements, enhancers can be grouped as weak, strong, inactive and poised enhancers. iEnhancer-2L^26^,the first ever predictor to detect enhancers and identify their strengths and was based on sequence information only. Pseudo K-tuple nucleotide compositions (PseKNC) based features were incorporated into iEnhancer-2L. It has been used in many analysis related to genomics increasingly. Furthermore, many other methods, such as EnhancerPred^27^ and EnhancerPred_2.0^28^, were introduced to improve the performance by incorporating other features based on DNA sequences. iEnhancer-5Step^29^ was recently developed using the hidden information of DNA sequences infused with Support Vector Machine (SVM) based predictions. Recently, iEnhancer-RD^30^ combined features and utilized recursive feature elimination algorithm for feature selection with deep neural network for enhancer identification. Similarly, ES-ARCNN^31^ used reverse complement method of data augmentation with residual Convolution Neural Network (CNN) to predict enhancer strength. iEnhancer-GAN^32^ also implemented CNNs to identify enhancers with strength using deep learning frameworks and combination of word embedding techniques. iEnhancer-XG^33^ utilized XGBoost classifier as base classifier and five feature extraction methods namely, K-Spectrum Profile, Mismatch K-tuple, Subsequence Profile, Position-Specific-Scoring-Matrix (PSSM) and Pseudo dinucleotide composition (PseDNC) to classify enhancers and their strength. iEnhancer-KL^34^ also implemented Position specific Nucleotide Composition and Kullback–Leibler (KL) method with several machine learning models. Enhancer-IF utilized comprehensively explored heterogeneous features with five commonly used machine learning algorithms. These five methods were extensively trained using 35 baseline models having seven encodings. This integration of five meta–models enhanced the overall performance of prediction model. BERT(bidirectional encoder representations from transformers)^35^ and 2D CNN based models were used with the contextualized word embedding for capturing the semantics and context of the words for representing DNA sequences. This opened a new avenue in biological sequence modeling. iEnhancer-MFGBDT^36^ used gradient boosting decision tree by fusing multiple features which included k-mer, k-mer with reverse compliments, second-order moving components etc. compared to other state of the art methods, this was an effective and intelligent tool to identify enhancers. iEnhancer-ECNN^37^ used one hot encoding methods and k-mers for data transformation and convolution neural networks (CNN) for identifying enhancers and classify their strengths. An ensemble deep recurrent neural network based method^38^ was also used to identify enhancers and their strength. These deep ensemble networks were generated from six types of dinucleotide physiochemical properties. These properties outperformed other features and achieved better performance and efficiency. This method proved to be better and has the potential to improve performance of biological sequential modeling using shallow machine learning models. However, improvement in the performance of the aforementioned predictors is still required. Specifically, the success rate of discriminating strong and weak enhancers is not up to the expectations of the scientific community. The current study is initiated to propose a method which would deal with this problem.

## Materials and methods

### Benchmark dataset

The benchmark dataset of DNA enhancer sites, originally constructed and used in recent past by iEnhancer-2L^26^, was re-used in the proposed method. In the current dataset, information related to nine different cell lines (K562, H1ES, HepG2, GM12878, HSMM, HUVEC, NHEK, NHLF and HMEC) was used in the collection of enhancers and 200 bp fragments were extracted from DNA sequences. The annotation of chromatin state information was performed by ChromHMM. The whole genome profile included multiple histone marks such as, H3K27ac H3K4me1, H3K4me3, etc. To remove pairwise sequences from the dataset, CD-HIT^39^ tool was used to remove sequences having more than 20% similarity. The benchmark dataset includes 2968 DNA enhancer sequences from which 1484 are non-enhancer sequences and 1484 are enhancer sequences. From 1484 enhancer sequences, 742 are strong enhancers and 742 are weak enhancers for the second layer classification. Furthermore, the independent dataset used by iEnhancer-5Step^29^ was utilized to enhance the effectiveness and performance of the proposed model. The independent dataset included 400DNA enhancer sequences from which 200 (100 strong and 100 weak enhancers) are enhancers and 200 are non-enhancers. Table 1 includes the breakdown of the benchmark dataset. The details of the above mention dataset is provided in the Supplementary Material (see Online Supporting Information S1, Online Supporting Information S2 and Online Supporting Information S3).

**Table 1:** Breakdown of the benchmark datasets of DNA enhancers and non-enhancers.

DNA samples	Benchmark dataset^26^	Independent dataset^29^
Non-enhancers	1484	200
Enhancers	1484	200
Overall	2968	400
**Breakdown of strong and weak enhancers dataset**		
Strong enhancers	742	100
Weak enhancers	742	100
Total enhancer	1484	200

It is not always simple to understand the semantics of a piece of data, which in turn reflects the difficulty of developing biological data models. It can be difficult to come to a consensus about the data in a given domain because different people will emphasize different features, use different terminology, and have different perspectives on how things should be seen. The fact that biosciences are non-axiomatic and that different, though closely related communities have very different perspectives on the same or similar concepts makes the situation even more difficult. Biological data models, however, can be useful for creating, making explicit, and communicating precise and in-depth descriptions of data that is already available or soon to be produced. It is hoped that the current study will increase the use of biological data models in bioinformatics, alleviating the management and sharing issues that are currently becoming more and more problematic.

In statistical based prediction models, the benchmark dataset mostly includes training datasets and testing datasets. By utilizing various benchmark datasets, results obtained are computed from fivefold and tenfold cross-validations. The definition of a benchmark dataset is used in Eq. (1):

1 $$\left\{\begin{array}{l}D={D}^{+}\cup {D}^{-}\\ \\ {D}^{+}= {D}_{strong}^{+}\cup {D}_{weak}^{+}\end{array}\right.$$

where ${D}^{+}$ contains 1484 enhancers and ${D}^{-}$ contains 1484 non-enhancers. ${D}_{strong}^{+}$ contains 742 strong enhancers, ${D}_{weak}^{+}$ contains 742 weak enhancers and U denotes the symbol of “union” in the set theory.

### Feature extraction

An effective bioinformatics predictor is the need of researchers in medicine and pharmacology to formulate the biological sequence with a vector or a discrete model without losing any key-order characteristics or sequence-pattern information. The reason for this fact, as explained in a comprehensive state-of-the-art review^40^, that the existing machine-learning algorithms cannot handle sequences directly but rather in vector formulations. However, there exists some possibility that all the sequence-pattern information from a vector might be lost in a discrete model formulation. To overcome the sequence-pattern information loss from proteins, Chou proposed pseudo amino acid composition (PseAAC)^41^. In almost all areas of bioinformatics and computational proteomics^40^, the Chou’s PseAAC concept has been widely used ever since it was proposed. In the recent past, three publicly accessible and powerful softwares, ‘propy’^42^, ‘PseAAC-Builder’^43^ and ‘PseAAC-General’^44^ were developed and the importance and popularity of Chou’s PseAAC in computational proteomics has increased more ever since. ‘PseAAC-General’ calculates Chou’s general PseAAC^45^ and the other two software generate Chou’s special PseAAC in various modes^46^. The Chou’s general PseAAC included not only the feature vectors of all the special modes, but also the feature vectors of higher levels, such as “Gene Ontology” mode^45^, “Functional Domain” mode^45^ and “Sequential Evolution” mode or “PSSM” mode^45^. Using PseAAC successfully for finding solutions to various problems relevant to peptide/protein sequences, encouraged the idea to introduce PseKNC (Pseudo K-tuple Nucleotide Composition)^47^ for generating different feature vectors for DNA/RNA sequences^48,49^ which proved very effective and efficient as well. In recent times a useful, efficient and a very powerful webserver called ‘Pse-in-One’^50^ and its recently updated version ‘Pse-in-One2.0’^51^ were developed that are able to generate any preferred feature vector of pseudo components for DNA/RNA and protein/peptide sequences.

In this study, we utilized the Kmer^52^ approach to represent the DNA sequences. According to Kmer, the occurrence frequency of ‘n’ neighboring nucleic acids can be represented from a DNA sequence. Hence, by using the sequential model, a sample of DNA having ‘w’ nucleotides is expressed generally as Eq. (2)

2 $$\mathbf{S}={Y}_{1}{Y}_{2}{Y}_{3}\dots {Y}_{v}\dots {Y}_{w}$$

where ${Y}_{1}$ is represented as the first nucleotide of the DNA sample **S,** ${Y}_{2}$ as the second nucleotide having the 2nd position of occurrence in DNA sample S and so on so fourth ${Y}_{w}$ denotes the last nucleotide of the DNA sample. ‘w’ is the total length of the nucleotides in a DNA sample. The ${Y}_{v}$ nucleotide can be any four of the nucleotides which can be represented using the aforementioned discrete model. The nucleotide ${Y}_{v}$ can be further described using Eq. (3)

3 $${Y}_{v}\in \left\{A \left(adenine\right) \; C\left(cytosine\right) \; G\left(guanine\right) \; T(thymine)\right\}$$

Here $\in$ is the symbol used to represent the set theory ‘member of’ property and 1 ≤ *v* ≤ *n*. The components that are defined by the aforementioned discrete model utilize relevant nucleotides useful features to expedite the extraction methods. These components are further used in statistical moments based feature extraction methods.

#### Statistical moments

Statistical moments are quantitative measures that are used for the study of the concentrations of some key configurations in a collection of data used for pattern recognition related problems^53^. Several properties of data are described by different orders of moments. Some moments are used to reveal eccentricity and orientation of data while some are used to estimate the data size^54–59^. Several moments have been formed by various mathematicians and statisticians based on famous distribution functions and polynomials^60–62^. These moments were utilized to explicate the current problem^63^.

The moments that are used in calculations of mean, variance and asymmetry of the probability distribution are known as raw moments. They are neither location-invariant nor scale-invariant. Similar type of information is obtained from the Central moments, but these central moments are calculated using the centroid of the data. The central moments are location-invariant with respect to centroid as they are calculated along the centroid of the data, but still they remain scale-variant. The moments based on Hahn polynomials are known as Hahn moments. These moments are neither location-variant nor scale-invariant^64–67^. The fact that these moments are sensitive to biological sequence ordered information amplifies the reason to choose them as they are primarily significant in extracting the obscure features from DNA sequences. These features have been utilized in previous research studies^54,59–61,68–73^ and have proved to be more robust and effective in extracting core sequence characteristics. The use of scale-invariant moment has consequently been avoided during the current study. The values quantified from utilizing each method enumerate data on its own measures. Furthermore, the variations in data source characteristics imply variations in the quantified value of moments calculated for arbitrary datasets. In the current study, the 2D version of the aforementioned moments is used and hence the linear structured DNA sequence as expressed by Eq. (2) is transformed into a 2D notation. The DNA sequence, which is 1D, is transformed to a 2D structure using row major scheme through the following Eq. (4):

4 $$d= \lceil\sqrt{z}\rceil$$

where the sample sequence length is ‘z’ and the2-dimensional square matrix has ‘$d$’ as its dimension. The ordering obtained from Eq. (4) is used to form matrix M (Eq. 5) having ‘m’ rows and ‘m’ columns.

5 $$M= \left[\begin{array}{c}\begin{array}{cc}{N}_{1\to 1}& {N}_{1\to 2}\\ {N}_{2\to 1}& {N}_{2\to 2}\end{array}\\ \begin{array}{cc}\vdots & \vdots \\ {N}_{k\to 1}& {N}_{k\to 2}\end{array}\\ \begin{array}{cc}\vdots & \vdots \\ {N}_{m\to 1}& {N}_{m\to 2}\end{array}\end{array}\begin{array}{c}\begin{array}{cc}\cdots & {N}_{1\to j}\\ \cdots & {N}_{2\to j}\end{array}\\ \begin{array}{cc}\cdots & \vdots \\ \cdots & {N}_{k\to j}\end{array}\\ \begin{array}{cc}\cdots & \vdots \\ \cdots & {N}_{m\to j}\end{array}\end{array}\begin{array}{c}\begin{array}{cc}\cdots & {N}_{1\to m}\\ \cdots & {N}_{2\to m}\end{array}\\ \begin{array}{cc}\cdots & \vdots \\ \cdots & {N}_{k\to m}\end{array}\\ \begin{array}{cc}\cdots & \vdots \\ \cdots & {N}_{m\to m}\end{array}\end{array}\right]$$

The transformation from M matrix to square matrix M’ is performed using the mapping function ‘Ʀ’. This function is defined as Eq. (6):6

If the population of square matrix M’ is done as row major order then, $i=\frac{x}{m}+1$ and $j=x mod m$.

Any vector or matrix, which represents any pattern, can be used to compute different forms of moments. The values of M’ are used to compute raw moments. The moments of a 2D continuous function $A\left(j, k\right)$ to order (j + k) are calculated from Eq. (7):

7 $${A}_{jk}= \sum_{a}\sum_{b}{a}^{j}{b}^{k}f(a,b)$$

The raw moments of 2D matrix M, with order (j + k) and up to a degree of 3,are computed using the Eq. (7). The origin of data as the reference point and distant components from the origin are assumed and utilized by the raw moments for computations. The 10 moment features computed up to degree-3 are labeled as ${M}_{00}$,${M}_{01}$, ${M}_{10}$, ${M}_{11}$,${M}_{02}$, ${M}_{20}, {M}_{12}$,${M}_{21}$, ${M}_{30}$ and ${M}_{03}.$

The centroid of any data is considered as its center of gravity. The centroid is the point in the data where it is uniformly distributed in all directions in the relations of its weighted average^74,75^. The central moments are also computed up to degree-3, using the centroid of the data as their reference point, from the following Eq. (8):

8 $${\mu }_{jk}= \sum_{a}\sum_{b}{(a-\overline{a })}^{j}{\left(b-\overline{b }\right)}^{k}f(a,b)$$

The degree-3 central moments with ten distinct feature sare labeled as ${\mu }_{00}$, ${\mu }_{01}$, ${\mu }_{10}$, ${\mu }_{11}$,${\mu }_{02}$,${\mu }_{20}$,${\mu }_{12}$, ${\mu }_{21}$, ${\mu }_{30}$&${\mu }_{03}.$ The centroids $\overline{a }$ and $\overline{b }$ are calculated from Eqs. (9) and (10):

9 $$\overline{a }= \frac{{M}_{10}}{{M}_{00}},$$

10 $$\overline{b }= \frac{{M}_{01}}{{M}_{00}}$$

The Hahn moments are computed by transforming 1D notations into square matrix notations. This square matrix is valuable for the computations of discrete Hahn moments or orthogonal moments as these moments are of 2D form and require a two-dimensional square matrix as input data. These Hahn moments are orthogonal in nature that implies that they possess reversible properties. Usage of this property enables the reconstruction of the original data using the inverse functions of discrete Hahn moments. This further indicates that the compositional and positional features of a DNA sequence are somehow conserved within the calculated moments. M’ matrix is used as 2D input data for the computations of Orthogonal Hahn moments. The order ‘m’ Hahn polynomial can be computed from Eq. (11):

11 $${h}_{m}^{x,y}\left(i, N\right)={(N+y-1)}_{m}{(N-1)}_{m}\sum_{j=0}^{m}{(-1)}^{j}\frac{{(-m)}_{j}{(-i)}_{j}{(2N+ x+y-m-1)}_{j}}{{(N+y-1)}_{j}{(N-1)}_{j}}.\frac{1}{j!}$$

The aforementioned Pochhammer symbol ( ) was defined as follows in Eq. (12):
12

And was simplified further by the Gamma operator in Eq. (13):13

The Hahn moments raw values are scaled using a weighting function and a square norm given as in Eq. (14):

14 $$\widetilde{{h}_{m}^{x,y}}\left(i, N\right)= {h}_{m}^{x,y}\left(i, N\right)\sqrt{\frac{\rho \left(i\right)}{{k}_{m}^{2}}}, m=0, 1, \dots ,N-1$$

Meanwhile, in Eq. (15),

15 $$\rho \left(i\right)= \frac{\Gamma(x+i+y)\Gamma(y+i+1){(x+y+i+1)}_{N}}{(x+y+2i+1)m!(N-i-1)!}$$

The Hahn moments are computed up to degree-3for the 2-D discrete data as follows in Eq. (16):

16 $${H}_{uv}= \sum_{b=0}^{N-1}\sum_{a=0}^{N-1}{\beta }_{ab}\widetilde{{h}_{u}^{x,y}}\left(b,N\right)\widetilde{{h}_{v}^{x,y}}\left(a,N\right), m,n=0, 1,\dots , N-1$$

The 10 key Hahn moments-based features are represented by ${H}_{00}$, ${H}_{01}$, ${H}_{10}$, ${H}_{11}$,${H}_{02}$,${H}_{20}$,${H}_{12}$,${H}_{21}$,${H}_{30} \mathrm{and} {H}_{03}$. Matrix M’ was utilized in computing ten Raw, ten Central and ten Hahn moments for every DNA sample sequence up to degree-3 which later are unified into the miscellany super feature vector (SFV).

#### DNA-position-relative-incident-matrix (D-PRIM)

The DNA characteristics such as ordered location of the nucleotides in the DNA sequences are of pivotal significance for identification. The relative positioning of nucleotides in any DNA sequence is considered core patterns prevailing the physical features of the DNA sequence. The DNA sequence is represented by D-PRIM in (4 × 4) order. The matrix in Eq. (17) is used to extract position-relative attributes of every nucleotide in the given DNA sequence.

17 $$SD-PRIM= \left[\begin{array}{c}\begin{array}{cc}{N}_{1\to 1}& {N}_{2\to 1}\\ {N}_{1\to 2}& {N}_{2\to 3}\end{array}\begin{array}{cc}{N}_{3\to 1}& {N}_{4\to 1}\\ {N}_{3\to 2}& {N}_{4\to 2}\end{array}\\ \begin{array}{cc}{N}_{1\to 3}& {N}_{2\to 3}\\ {N}_{1\to 4}& {N}_{2\to 4}\end{array}\begin{array}{cc}{N}_{3\to 3}& {N}_{4\to 3}\\ {N}_{3\to 4}& {N}_{4\to 4}\end{array}\end{array}\right]$$

**Figure 1:**
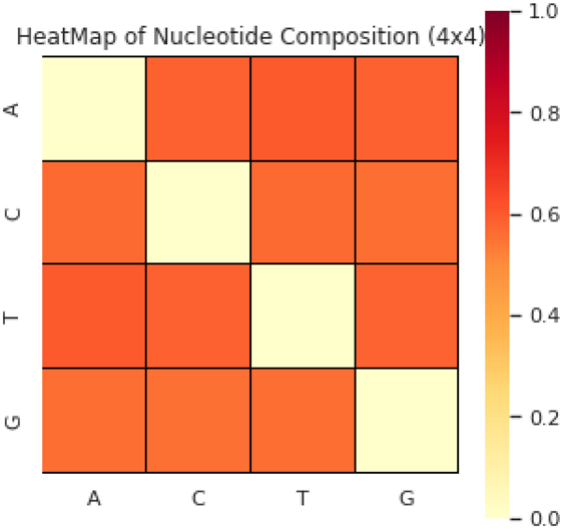
The heatmap of nucleotide composition based PRIMs.

**Figure 2:**
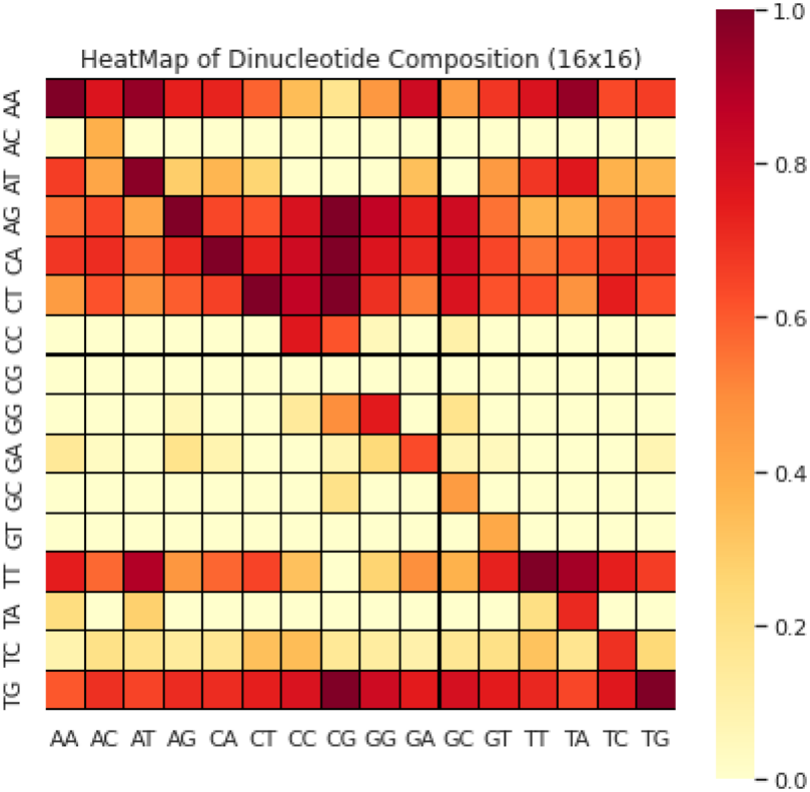
The heatmap of dinucleotide composition based PRIMs.

**Figure 3:**
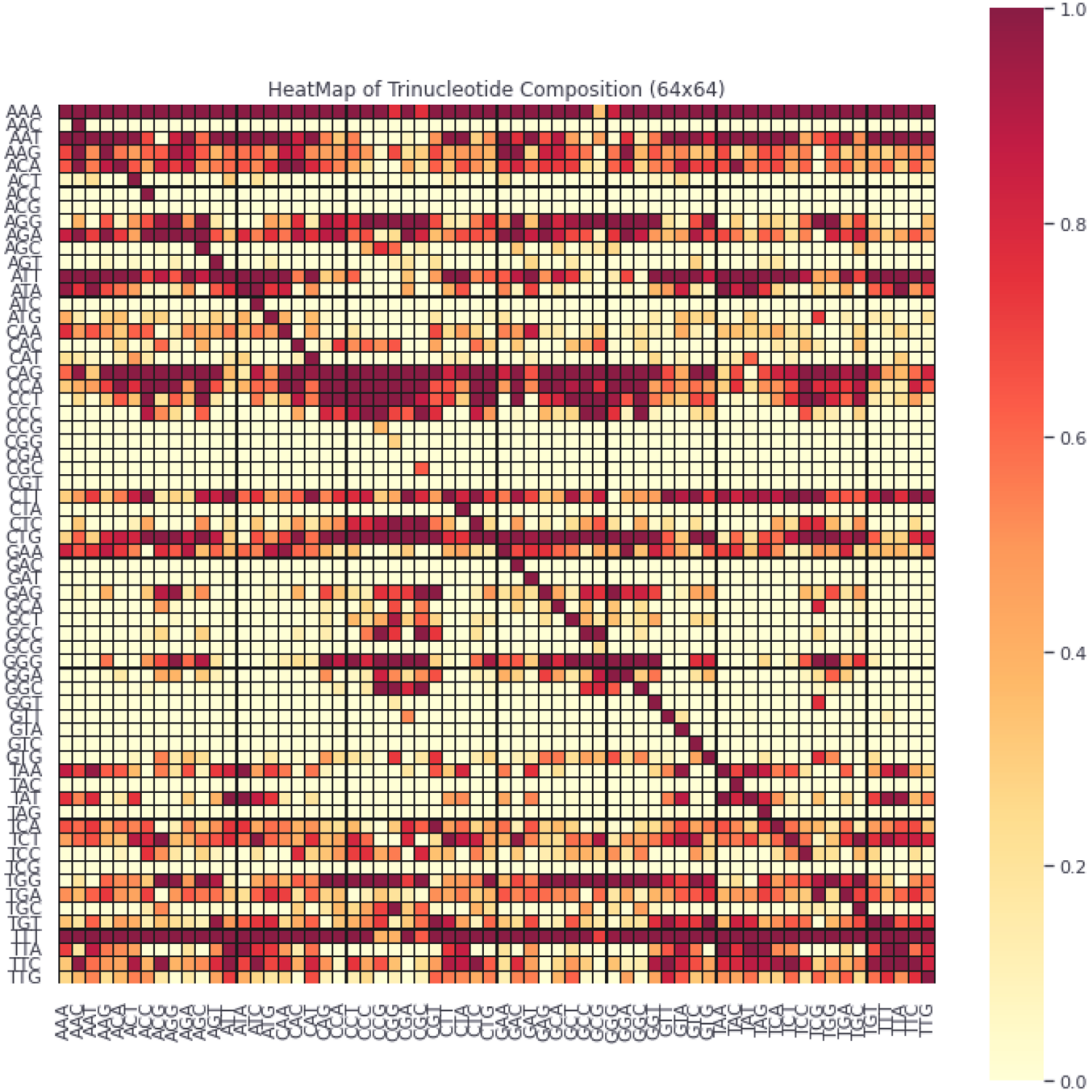
The heatmap of trinucleotide composition based PRIMs.

The position occurrence values of nucleotides are represented here using the notation ${N}_{x\to y}$. Here the indication score of the $y$th position nucleotide is determined using ${N}_{x\to y}$ with respect to the xth nucleotide first occurrence in the sequence. The nucleotide type ‘$y$’substitutes this score in the biological evolutionary process. The occurrence positional values, in alphabetical order, represented as four native nucleotides. The S_D-PRIM_ matrix is formed with 16 coefficient values obtained after successfully performing computations on position relative incidences. Similarly, S_D-PRIM16_^68^and S_D-PRIM64_^68^were constructedhaving16 × 16 and 64 × 64 valuable coefficient features respectively. The 2D heatmaps of these matrices are shown in Figs. 1, 2 and 3. These heatmaps are based on the summation of nucleotide, dinucleotide and trinucleotide composition PRIMs.

30 raw, central and Hahn moments (10 raw, 10 central & 10 Hahn), up to degree-3, were computed using the 2D S_D-PRIM_ matrix through which 30 features were obtained with 16 unique coefficients and were further incorporated into the miscellany Super Feature Vector (SFV).

#### DNA-reverse-position-relative-incident-matrix (D-RPRIM)

It often happens in cellular biology that the same ancestor is responsible for evolving more than one DNA sequence. These cases mostly outcome homologous sequences. The performance of the classifier is hugely affected by these homologous sequences and hence for producing accurate results, sequence similarity searching is reliable and effectively useful. In machine learning, accuracy and efficiency is hugely dependent on the meticulousness and thoroughness of algorithms through which most pertinent features in the data are extracted. The algorithms used in machine learning have the ability to learn and adapt the most obscure patterns embedded in the data while understanding and uncovering them during the learning phase. The procedure followed during the computation of D-PRIM was utilized in computations of D-RPRIM but only with reverse DNA sequence ordering. The position occurrence values of nucleotides are represented here using the notation ${N}_{x\to y}$. Here the indication score of the $y$ ^th^position nucleotide is determined using ${N}_{x\to y}$ with respect to the xth nucleotide first occurrence in the sequence. The nucleotide type ‘$y$’ substitutes this score in the biological evolutionary process. The occurrence positional values, in alphabetical order, represented as 4 native nucleotides. This procedure further uncovered hidden patterns for prediction and ambiguities between similar DNA sequences were also alleviated. The 2D matrix D-RPRIM was formed with (4 × 4) order having16unique coefficients. It is defined by Eq. (18):

18 $$SD-RPRIM= \left[\begin{array}{c}\begin{array}{cc}{N}_{1\to 1}& {N}_{2\to 1}\\ {N}_{1\to 2}& {N}_{2\to 3}\end{array}\begin{array}{cc}{N}_{3\to 1}& {N}_{4\to 1}\\ {N}_{3\to 2}& {N}_{4\to 2}\end{array}\\ \begin{array}{cc}{N}_{1\to 3}& {N}_{2\to 3}\\ {N}_{1\to 4}& {N}_{2\to 4}\end{array}\begin{array}{cc}{N}_{3\to 3}& {N}_{4\to 3}\\ {N}_{3\to 4}& {N}_{4\to 4}\end{array}\end{array}\right]$$

Similarly, 30 raw, central and Hahn moments (10 raw, 10 central & 10 Hahn), up to degree-3, were computed using the 2D S_D-RPRIM_ matrix through which 30 features were also obtained with 16 unique coefficients and they were also incorporated into the miscellany Super Feature Vector (SFV).

#### Frequency-distribution-vector (FDV)

The distribution of occurrence of every nucleotide was used to compute the frequency distribution vector. The frequency distribution vector (FDV) is defined as in Eq. (19):19

**Figure 4:**
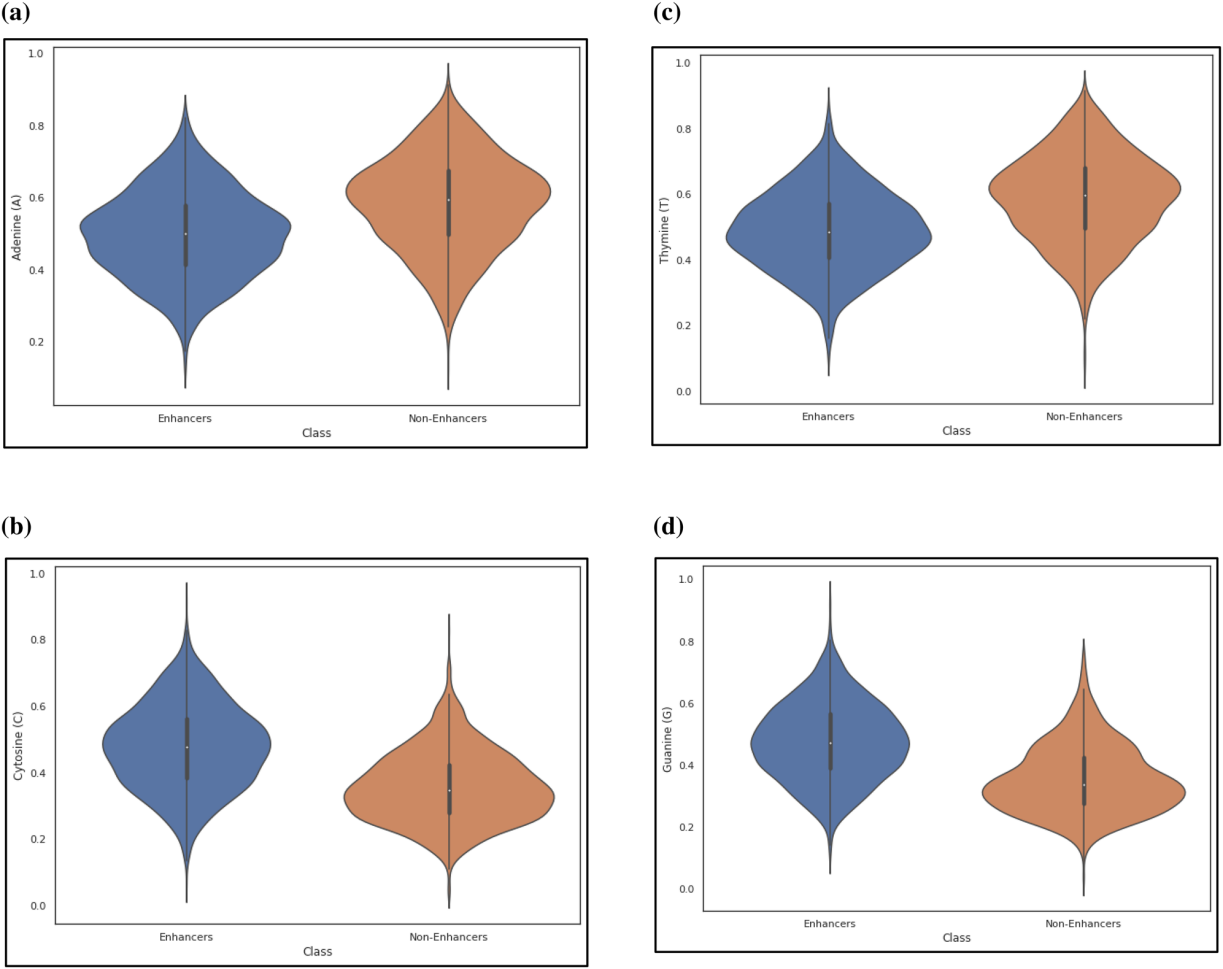
(**a**) The violin plot of nucleotide adenine (A) composition. (**b**) The violin plot of nucleotide cytosine (C) composition. (**c**) The violin plot of nucleotide thymine (T) composition. (**d**) The violin plot of nucleotide guanine (G) composition.

**Figure 5:**
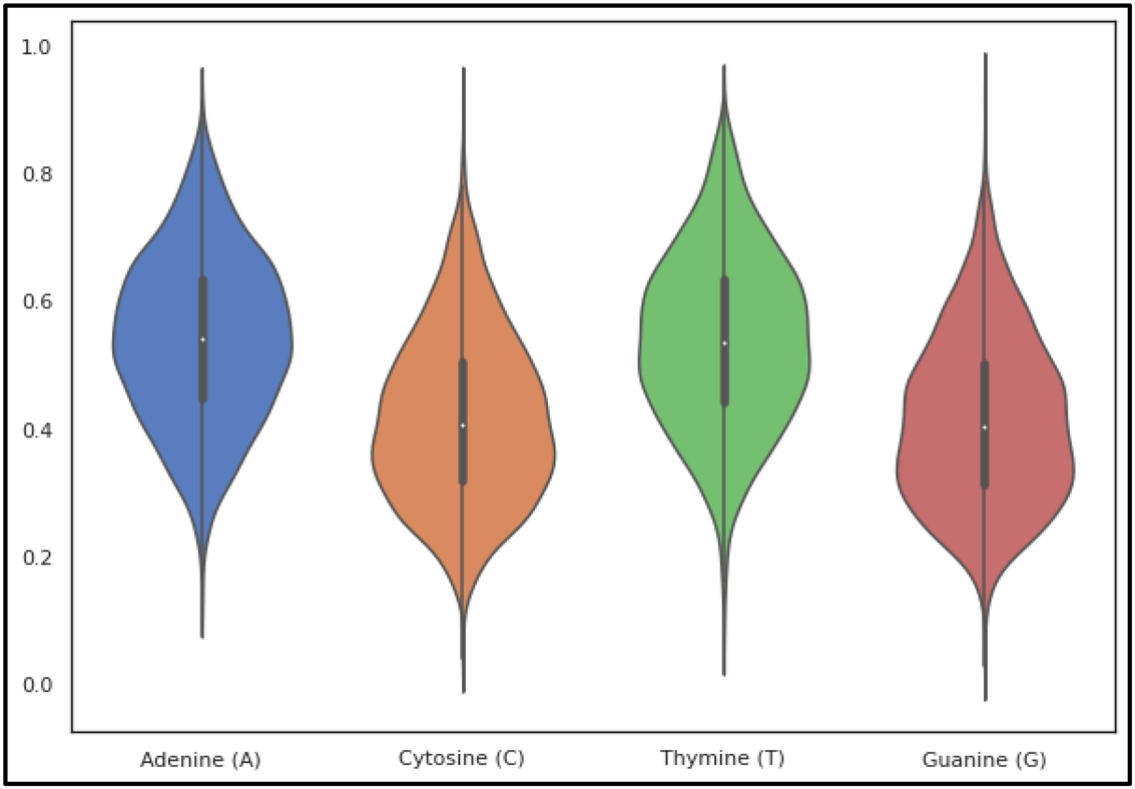
The violin plot of all four nucleotide compositions.

Here ${\rho }_{i}$ is the frequency of occurrence of the *i*th (1 ≤ *i* ≤ 4) relevant nucleotide. Furthermore, the relative positions of nucleotides in any sequence are highly utilized using these measures. The miscellany Super Feature Vector (SFV) includes these four features from FDV as unique attributes. The violin plots of nucleotide composition and overall frequency normalization is shown in Figs. 4a–d and 5.

#### D-AAPIV (DNA-accumulative-absolute-position-incidence-vector)

The distributional information of nucleotides was stored using frequency distribution vector which used the hidden patterns features of DNA sequences in relevance to the compositional details. FDV does not have any information regarding relative positional details of relevant nucleotide residues in DNA sequences. This relative positional information was accommodated using D-AAPIV with a length of four critical features associated with four native nucleotides in a DNA sequence. These four critical features from D-AAPIV are also added into the miscellany Super Feature Vector (SFV).20

Here ${\alpha }_{i}$ is any element of D-AAPIV, from DNA sequence ${S}_{j}$ having ‘n’ total nucleotides, which can be calculated using Eq. (21):

21 $${\beta }_{i}=\sum_{j=1}^{n}{S}_{j}$$

#### D-RAAPIV (DNA-reverse-accumulative-absolute-position-incidence-vector)

D-RAAPIV is calculated using the reverse DNA sequence as input with the same method used using D-AAPIV calculations. This vector is calculated to find the deep and hidden features of every sample with respect to reverse relative positional information. D-RAAPIV is formed as the following Eq. (24) using the reversed DNA sequence and generates four valuable features. These four critical features from D-RAAPIV are also added into the miscellany Super Feature Vector (SFV).22

Here ${\alpha }_{i}$ is any element of D-RAAPIV, from DNA sequence ${S}_{j}$ having ‘n’ total nucleotides, which can be calculated using Eq. (23):

23 $${\beta }_{i}=\sum_{j=1}^{n}{Reverse(S)}_{j}$$

**Figure 6:**
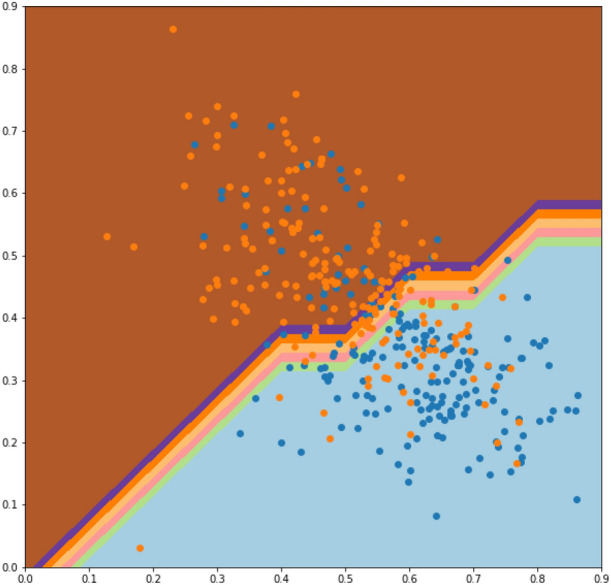
The feature visualization scatter plot of features extracted and used in the proposed study.

After calculating all possible features from the aforementioned extraction methods, the Super Feature Vector (SFV)was constructed, for further processing in classification algorithm. The proposed model has used extracted features with more robustness to noise and effective against the sensitive DNA Enhancer sites as shown in Fig. 6. All the combined features efficiently differentiate from Enhancers and Non Enhancer sites.

### Classification algorithm

#### Random forests

In the past, ensemble learning methods have been applied in many bioinformatics relevant research studies^76,77^ and have produced highly efficient outcomes in measures of performance. Ensemble learning methods utilize many classifiers in a classification problem with aggregation of their results. The two most commonly used methods are boosting^78,79^ and bagging^80^ which perform classifications using trees. In boosting, the trees which are successive, propagate extra weights to points which are predicted incorrectly by the previous classifiers. The weighted vote decides the prediction in the end. Whereas, in bagging, the successive trees do not rely on previous trees, rather, each tree is constructed independently from the data using a bootstrap sample. The simple majority vote decides the prediction in the end.

**Figure 7:**
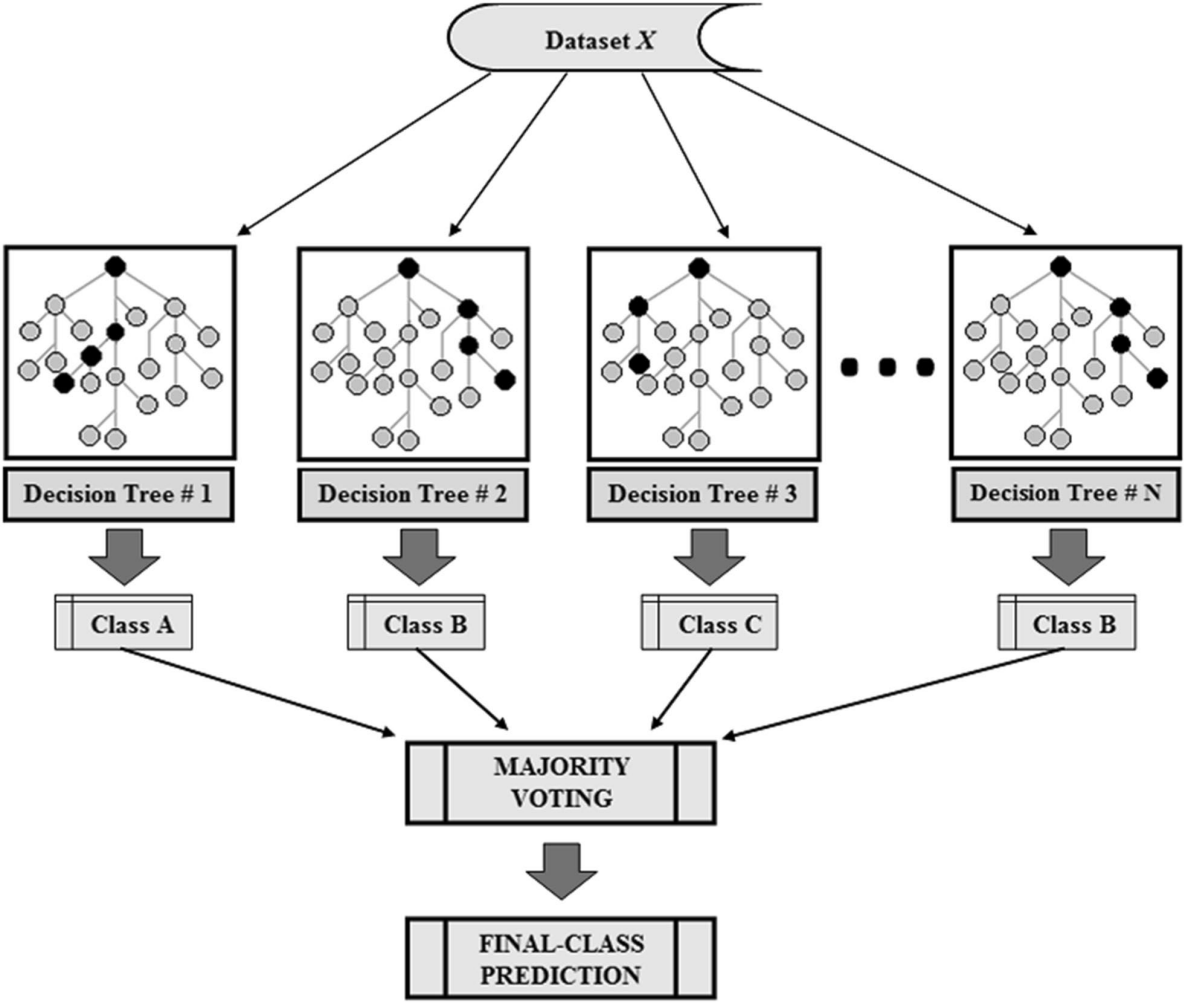
The structure of the random forest classifier.

In bioinformatics and related fields, random forests have grown in popularity as a classification tool. They have also performed admirably in extremely complex data environments. A random sample of the observations, typically a bootstrap sample or a subsample of the original data, is used to build each tree in a random forest. Out-of-bag (OOB) observations are those that are not included in the subsample or the bootstrap sample, respectively. The so-called OOB error can be produced, for instance, by using the OOB observations to estimate the random forest prediction error. The OOB error is frequently used to gauge how well the random forest classifier predicts outcomes and aids in identifying model uncertainties. The OOB error has the benefit of using the entire original sample for both building the random forest classifier and estimating error. In order to add more randomness to bagging, Leo Breiman^81^ constructed random forests. The random forests changed the construction of the classification trees by adding the construction of each tree from the data using a different bootstrap sample. The splitting of each node, in standard classification trees, is performed by dividing each node equally among all the variables. However, in random forests, the splitting of each node is performed by choosing the best among a subset of predictors which are chosen randomly at that node (Fig. 7 shows the structure of the random forest classifier). As compared to many other classifiers, such as support vector machine, discriminant analysis and neural networks, this counterintuitive strategy perform very well and is robust against overfitting^76^.

#### Algorithm: supervised learning using random forest

**Figure 8:**
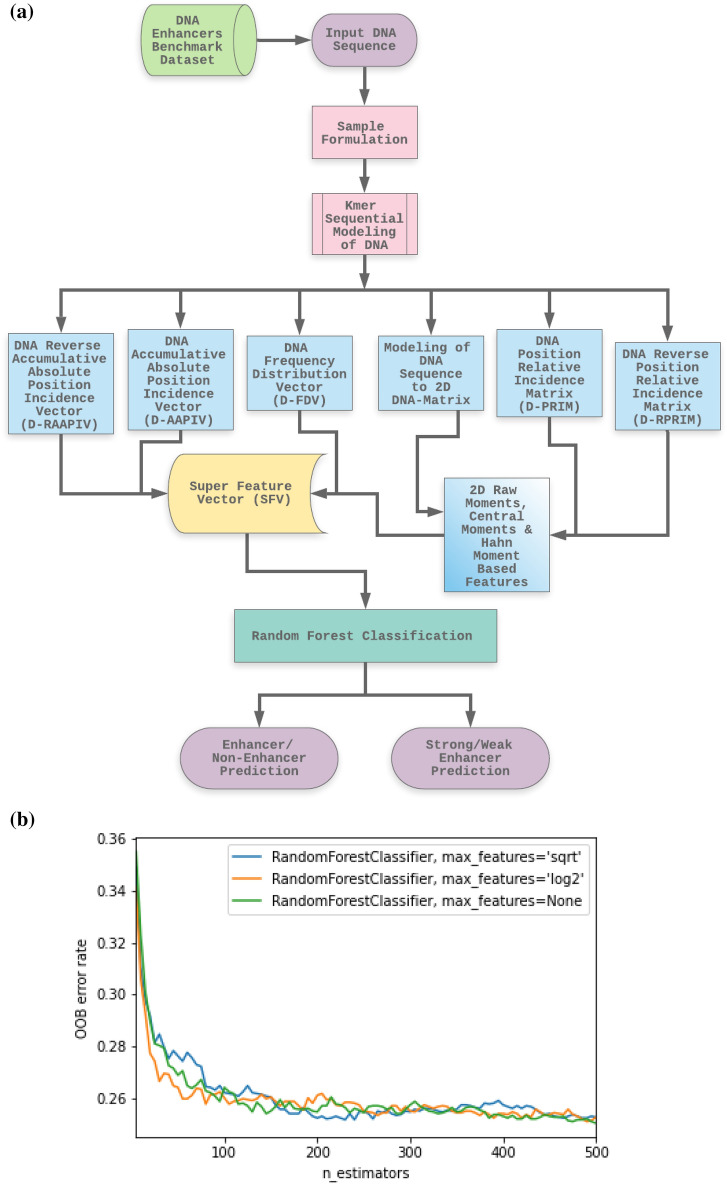
(**a**) The Flowchart of the overall proposed method. (**b**) The OOB error rate stabilization during training estimator trees.

Scikit-Learn^82^ library using python was implemented for random forest classifier for fitting the trainings and simulations in our proposed method. The number of trees was increased from the default parameter value of 10 to 100. The number of trees parameter value was optimized to 100 using hyper parameter tuning methods and optimal value for the parameter was searched using the successive halving technique in scikit-learn^82^ library. The searching space for the parameter “n_estimators” in random forest classifier was (5–500) which was optimized to 100 after successful halving. One of the key findings observed during the experimentation process was that forest with more than 100 trees minimally contribute to the accuracy of the classifier, but can enhance the overall size of the proposed model substantially. Figure 8a illustrates a flowchart to show the overall process of the proposed method.

#### Out-of-bag estimation

It is frequently asserted that the OOB error is a neutral estimator of the true error rate. Every observation is "out-of-bag" for some of the trees in a random forest because each tree is constructed from a different sample of the original data. Then, only those trees can be used for which the observation was not used in the construction to derive the prediction for the observation. Each observation is given a classification as a result, and the error rate can be calculated using these predictions. The resulting error rate is referred to as OOB error. Breiman^81^ was the first to propose this process, and it has since gained widespread acceptance as a reliable technique for error estimation in “Random forests”. Each new tree is fitted from a bootstrap sample of the training observations ${z}_{i}= {x}_{i}, {y}_{i}$ when training the random forest classifier using bootstrap aggregation. The average error for each ${z}_{i}$ calculated using predictions from the trees that do not contain ${z}_{i}$ in their respective bootstrap sample is known as the out-of-bag (OOB) error. This makes it possible to fit and validate the random forest classifier while it is being trained. The OOB error is calculated at the addition of each new tree during training, as shown in the plot below. A practitioner can roughly determine the value of n estimators at which the error stabilizes using the resulting Fig. 8b. The scikit-learn^82^ library was used to process the out of bag error estimation.

### Ethical approval

This article does not contain any studies involved with human participants or animals performed by any of the authors.

## Experiments and results

For the assessment and verifications of the model and to analyze its performance, some methods are used to evaluate them. These methods evaluate the classifiers using inspection attributes which are based on the outcomes of classification assessments and estimates.

### Cross-validation

####  k-fold cross validation

K-fold cross validation (KFCV) technique is most commonly used by practitioners for estimation of errors in classifications. Also known as rotation estimation, KFCV splits a dataset into ‘K’ folds which are randomly selected and are equal in size approximately. The prediction error of the fitted model is calculated by predicting the *k*th part of the data which is dependent on other K − 1 parts to fit the model. The error estimates of K from the prediction are combined together using the same procedure for each k = 1, 2, … , K.

Although the generalization performance of any classifier is mostly estimated using unbiased approximations in jackknife tests, two drawbacks exists in this test, firstly, the variance is high as estimates used in all the datasets are very similar to each other, secondly, its calculative expensive as n estimates are required to be computed, and the total number of observations to test is n in the dataset. The fivefold and tenfold cross validation tests are proven to be a good compromise between computational requirements and impartiality.

In the KFCV tests, the selection of ‘K’ is considered as a significant attribute. To testify errors in prediction models, cross validations (K = 5 and K = 10) tests have been used in many research studies. 5-Fold and 10-Fold tests proved to have accurate results in our proposed model and proved to be much better than state-of-the-art methods. These results are listed in Tables 4, 5 and 6.

### Evaluation parameters

The problems of binary classifications use metrics such as Accuracy (Acc), Sensitivity (Sn), Specificity (Sp) and Mathew’s Correlation Coefficient (MCC)for measuring the proposed prediction model quality and efficiency. These metrics are defined in the following Eq. (24):

24 $$\left\{\begin{array}{l}Sn= \frac{TP}{TP+FN}\\ Sp= \frac{TN}{TN+FP}\\ \begin{array}{l}Accuracy= \frac{TP+TN}{TP+TN+FP+FN}\\ \begin{array}{c}\begin{array}{l}MCC= \frac{TP\times TN-FP\times FN}{\sqrt{(TP+FP)(TP+FN)(TN+FP)(TN+FN)}}\end{array}\end{array}\end{array}\end{array}\right.$$

Here true-positives (TP), TN (true-negatives), FP (false-positives) and FN (false-negatives) represent the outcomes from the cross validation tests. Unfortunately, the conventional formulations from the above mentioned metrics in Eq. (24) lack in intuitiveness and due to this fact, understanding these measures especially MCC, many scientists have faced difficulties. To ease this difficulty, the above conventional equations were converted by Xu^83^ and Feng^84^ using Chou’s four intuitive equations which used the symbols introduced by Chou^85^. The symbols that define these equations are; ${\mathrm{Y}}^{+}, {\mathrm{Y}}^{-},{\mathrm{Y}}_{-}^{+}\mathrm{ and }{\mathrm{Y}}_{+}^{-}$.The description of these symbols is defined in Table 2.

**Table 2:** Description of symbols used to define these equations.

Symbols	Description of symbols
${Y}^{+}$	The total number of true enhancers
${Y}_{-}^{+}$	The total number of true enhancers incorrectly predicted as non-enhancers
${Y}^{-}$	The total number of true non-enhancers
${Y}_{+}^{-}$	The total number of non-enhancers predicted as enhancers

From the above correspondence in Table 2, we can define Eq. (25):

25 $$\left\{\begin{array}{l}TP={Y}^{+ }-{Y}_{-}^{+}\\ TN= {Y}^{-}-{Y}_{+}^{-}\\ \begin{array}{c}FP= {Y}_{+}^{-}\\ FN= {Y}_{-}^{+}\end{array}\end{array}\right.$$

From the above correspondence in Table 2, we can define Eq. (26):

26 $$\left\{\begin{array}{l}Sn= 1- \frac{{Y}_{-}^{+}}{{Y}^{+ }}\\ Sp= 1-\frac{{Y}_{+}^{-}}{{Y}^{- }}\\ \begin{array}{l}Accuracy=1- \frac{{Y}_{-}^{+} + {Y}_{+}^{-}}{{Y}^{+ }+ {Y}^{-}}\\ \begin{array}{l}\begin{array}{l}MCC= \frac{1-\left(\frac{{Y}_{-}^{+}}{{Y}^{+ }} + \frac{{Y}_{+}^{-}}{{Y}^{-}}\right)}{\sqrt{\left(1+ \frac{{Y}_{+}^{-} - {Y}_{-}^{+}}{{Y}^{+ }}\right)\left(1+ \frac{{Y}_{-}^{+} -{ Y}_{+}^{-}}{{Y}^{-}}\right)}}\end{array}\end{array}\end{array}\end{array}\right.$$

The above Eq. (26) has the same meaning as the Eq. (24) but it is more easy to understand and intuitive. Table 3 defines the detail description of these equations.

**Table 3:** Description of equations used Eqs. (26).

When	Then	Description
${Y}_{-}^{+}=0$	Sn = 1	None of the enhancer is predicted as a non-enhancer
${Y}_{-}^{+}={Y}^{+}$	Sn = 0	All of the enhancers were incorrectly predicted as non-enhancers
${Y}_{+}^{-}=0$	Sp = 1	None of the non-enhancer is incorrectly predicted as an enhancer
${Y}_{+}^{-}={Y}^{-}$	Sp = 0	All of the non-enhancers are incorrectly predicted as enhancers
${Y}_{-}^{+}+{Y}_{+}^{-}=0$	ACC = 1, MCC = 1	None of the enhancers and none of the non-enhancers were incorrectly predicted
${Y}_{-}^{+}={Y}^{+} \mathrm{and}$ ${Y}_{+}^{-}={Y}^{-}$	ACC = 0, MCC = −1	All of the enhancers and all of the non-enhancers were incorrectly predicted
${Y}_{-}^{+}=\frac{{Y}^{+}}{2} \mathrm{and }{Y}_{+}^{-}=\frac{{Y}^{-}}{2}$	ACC = 0.5, MCC = 0	The overall prediction is not a better than any other random prediction outcome

The set of metrics used in above Table 3 are not applicable to multi-labeled prediction models rather they are only useful for single labeled-systems. A different set of metrics exists for multi-labeled-systems which have been used by various researchers^86–88^. The comparison of existing classifiers with proposed method is mentioned in Tables 4, 5 and 6.

**Table 4:** Comparison of state-of-the-art methods with the proposed method using 5-fold cross validation tests.

Layer	Classifiers	Sn (%)	Sp (%)	ACC (%)	MCC	AUC
1	iEnhancer-2L	78.09	75.88	76.89	0.54	0.85
	iEnhancer-2L-Hybrid	75.33	80.39	77.86	0.558	–
	EnhancerPred	72.57	73.79	77.39	0.464	–
	iEnhancer-EL	75.67	80.39	78.03	0.561	–
	iEnhancer-5Step	81.1	83.5	82.3	0.65	–
	Proposed method	84.90	88.21	86.56	0.7319	0.93
2	iEnhancer-2L	62.21	61.82	61.93	0.24	0.66
	iEnhancer-2L-Hybrid	71.02	60.64	65.83	0.318	–
	EnhancerPred	62.67	61.46	68.19	0.2413	–
	iEnhancer-EL	69	61.05	65.03	0.315	–
	iEnhancer-5Step	75.3	60.8	68.1	0.37	–
	ES-ARCNN	72.78	59.57	66.17	0.3263	–
	Proposed method	81.54	63.06	72.30	0.4537	0.80

**Table 5:** Comparison of classifiers for predicting enhancers using tenfold cross validations.

Layer	Classifier	Sn(%)	Sp(%)	ACC(%)	MCC	AUC	AUPR
1	KNN	69.81	72.90	71.36	0.4275	0.89	0.80
	Naïve Bayes	67.59	69.47	68.53	0.3712	0.78	0.72
	AdaBoost	72.30	73.31	72.80	0.4569	0.89	0.80
	SVM	70.68	78.43	74.56	0.4933	0.84	0.82
	Probalistic NN	72.04	72.97	72.51	0.4507	0.81	0.84
	Random forest	86.53	96.90	91.72	0.8398	0.87	0.97
2	KNN	58.77	54.05	56.41	0.1285	0.58	0.57
	Naïve Bayes	58.35	59.56	58.95	0.1793	0.62	0.61
	AdaBoost	63.46	57.15	60.31	0.2079	0.63	0.66
	SVM	69.94	55.68	62.80	0.2598	0.66	0.66
	Probalistic NN	76.95	44.34	60.64	0.2261	0.64	0.69
	Random forest	80.49	93.97	87.23	0.7519	0.82	0.93

**Table 6:** Independent tests based comparison of state-of-the-art methods with the proposed method.

Layer	Classifiers	Sn (%)	Sp (%)	ACC (%)	MCC	AUC
1	iEnhancer-2L	71	75	73	0.46	0.80
	EnhancerPred	73.5	74.5	74	0.48	0.81
	iEnhancer-EL	71	78.5	74.75	0.496	0.82
	iEnhancer-5Step	82	76	79	0.58	0.87
	iEnhancer-ECNN	78.5	75.2	76.9	0.537	0.83
	iEnhancer-RD	81.0	76.5	78.8	0.576	0.84
	Proposed method	78.10	81.05	79.50	0.5907	0.93
2	iEnhancer-2L	47	74	60.5	0.2181	–
	EnhancerPred	45	65	55	0.1021	–
	iEnhancer-EL	54	68	61	0.2222	–
	iEnhancer-5Step	74	53	63.5	0.28	–
	iEnhancer-ECNN	79.1	56.4	67.8	0.368	–
	iEnhancer-RD	84.0	57.0	70.5	0.426	–
	ES-ARCNN	86	45	65.5	0.3399	–
	Proposed method	68.29	79.22	72.5	0.4624	–

## Results and discussions

**Figure 9:**
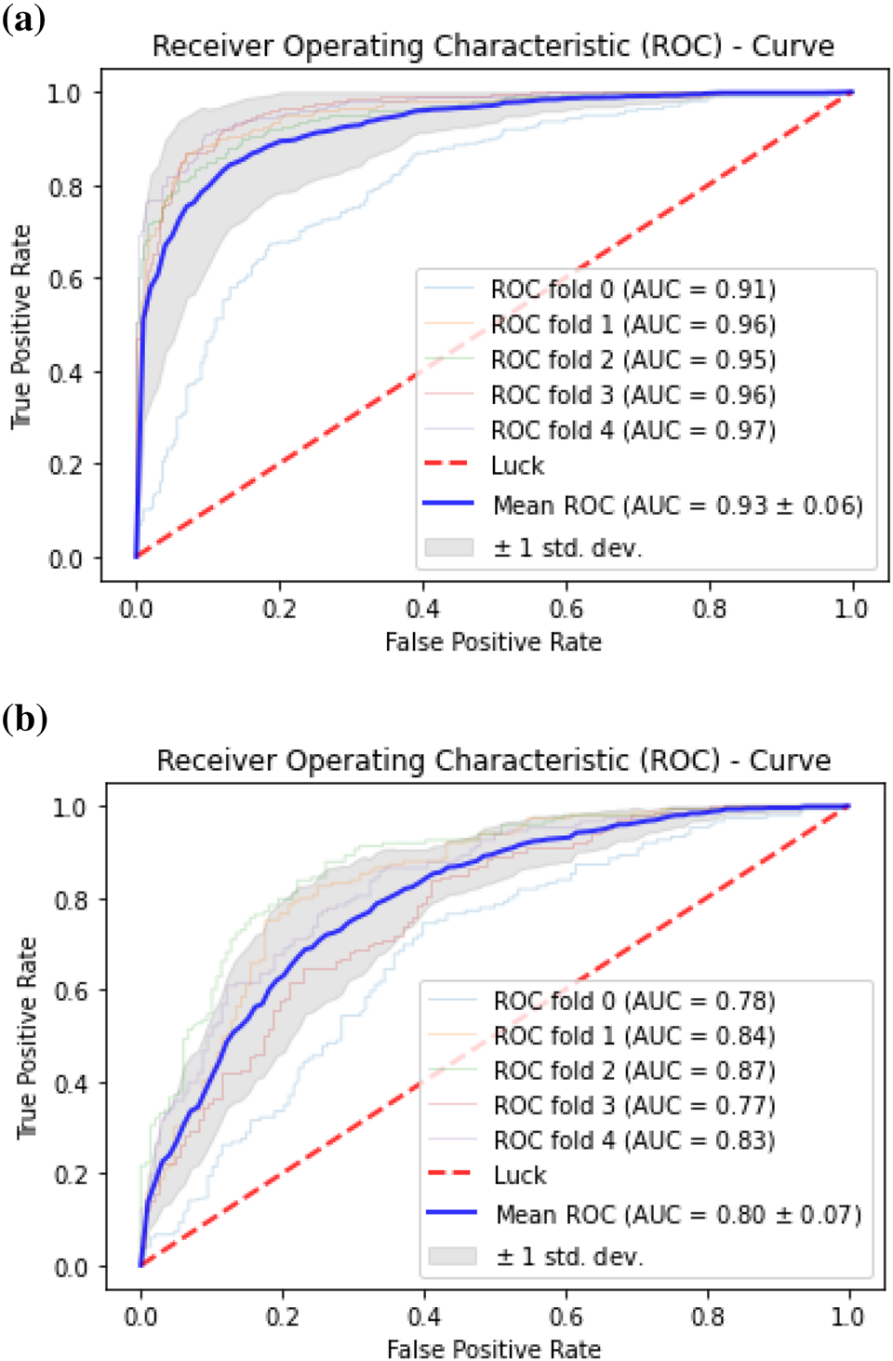
(**a**) ROC curve of fivefold cross validation tests for enhancers. (**b**) ROC curve of fivefold cross validation tests for enhancer strengths.

**Figure 10:**
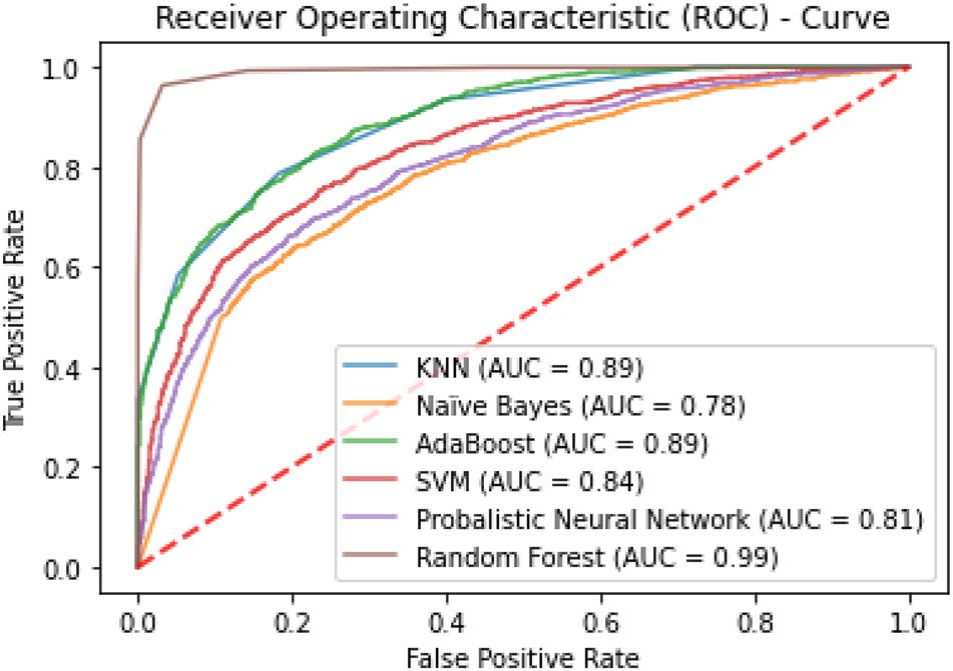
10 fold test ROCs comparison of classifiers for enhancer site prediction.

**Figure 11:**
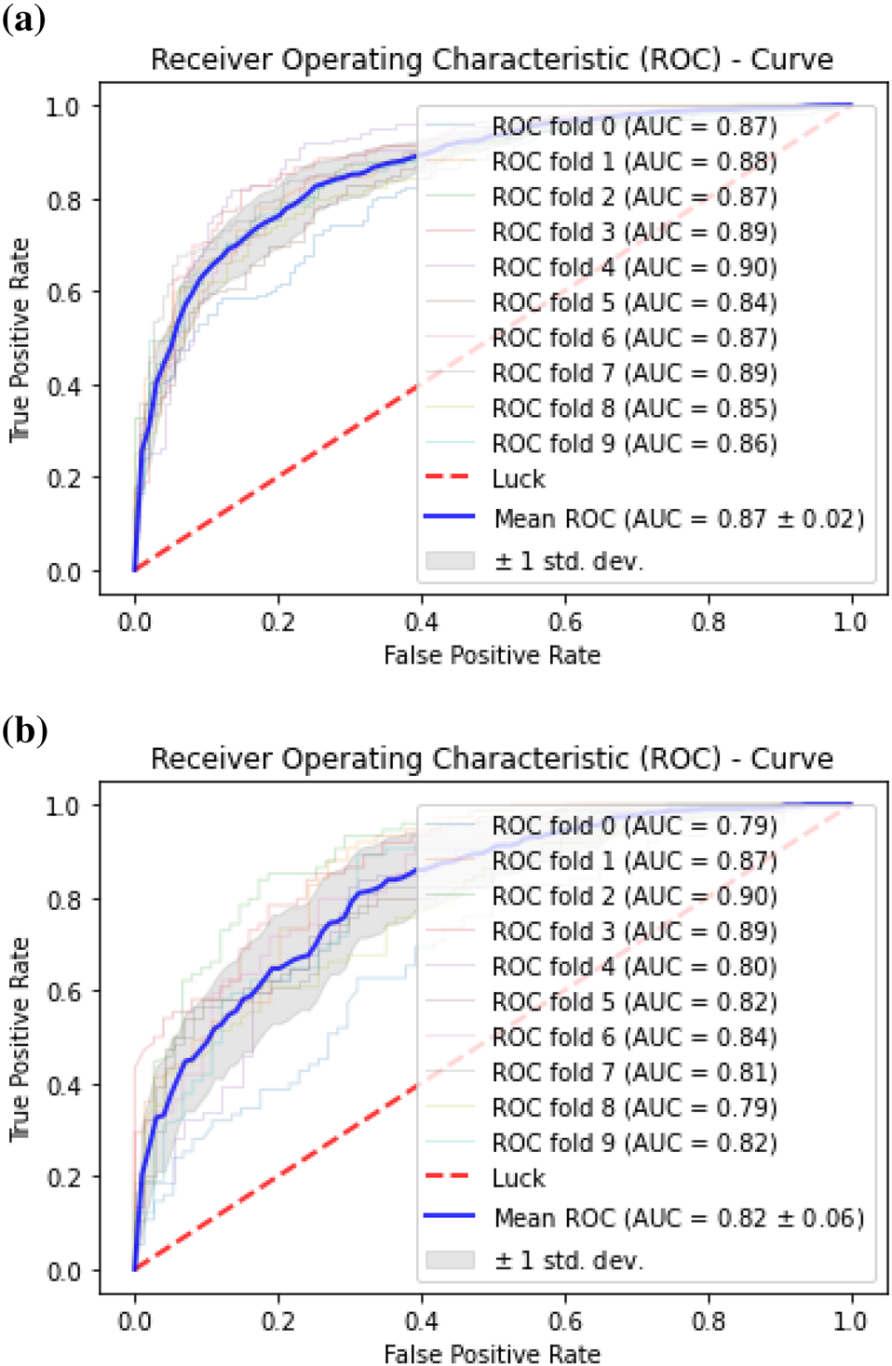
(**a**) ROC curve of tenfold cross validation tests for enhancers using (random forest). (**b**) ROC curve of tenfold cross validation tests for enhancers strength (random forest).

**Figure 12:**
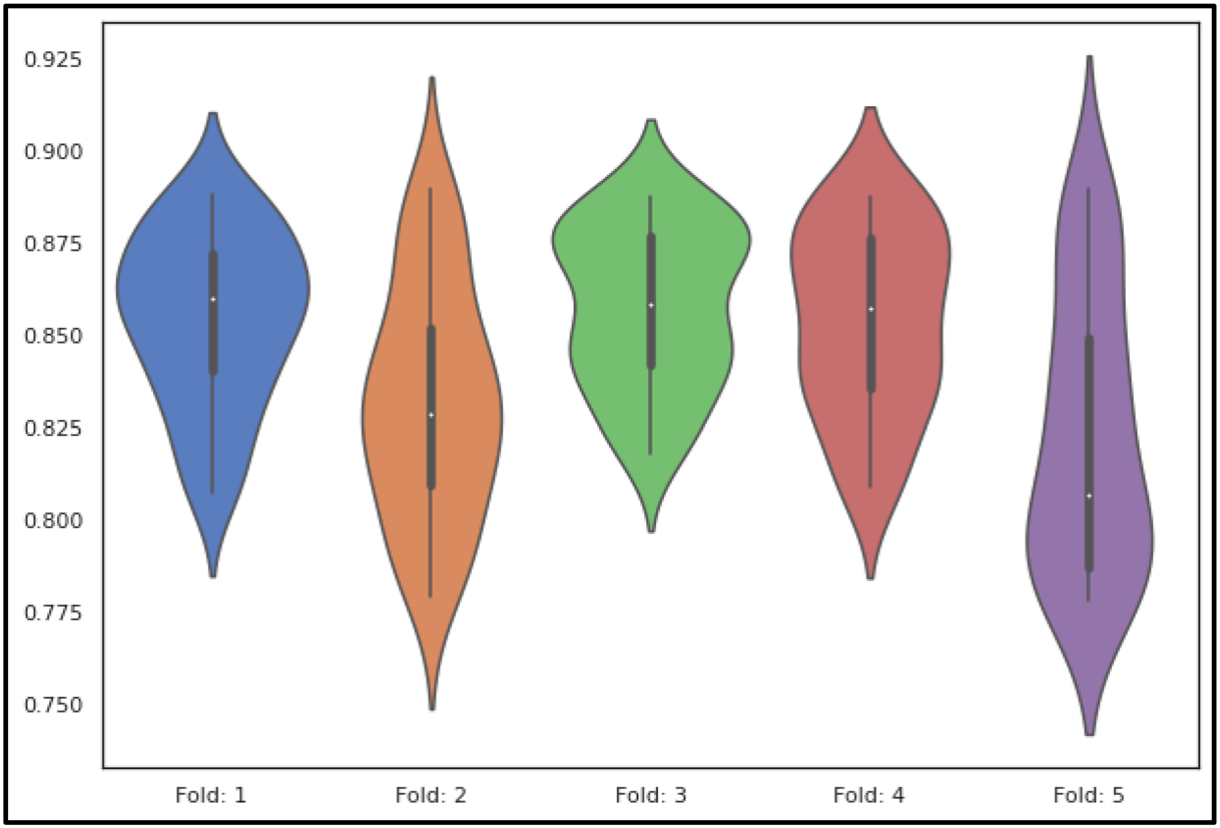
Violinplot of fivefold cross validation for enhancers (random forest).

**Figure 13:**
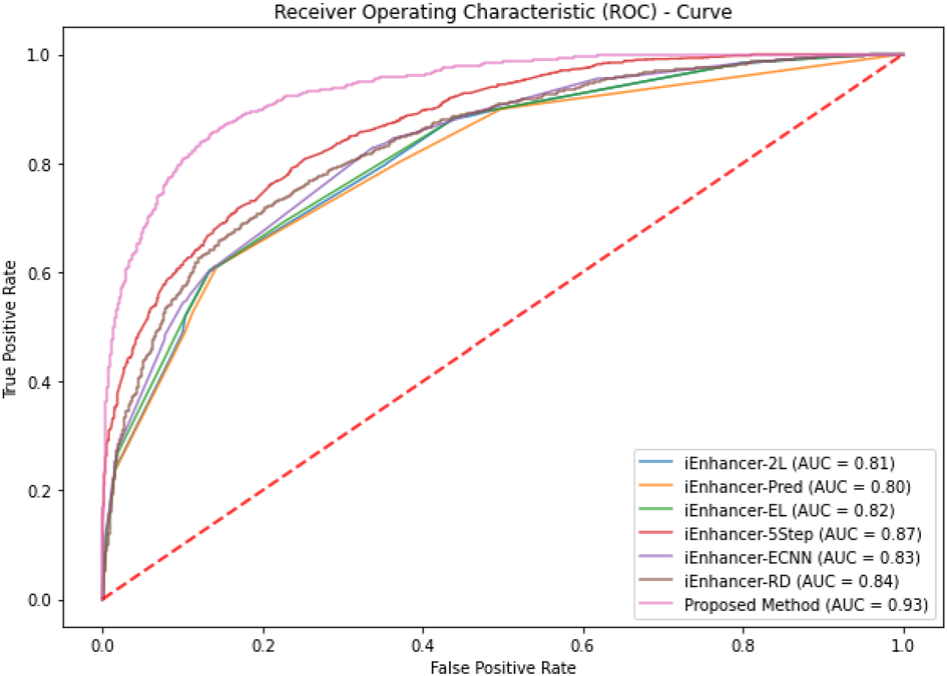
The ROCs of state of art methods using independent tests for enhancer prediction.

**Figure 14:**
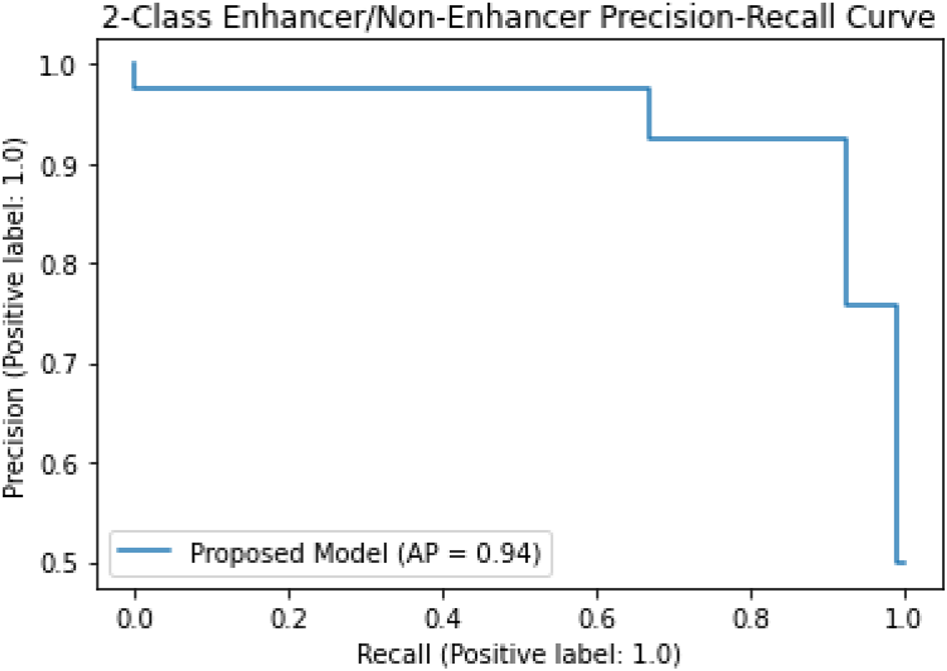
The PR curves of random forest using jackknife tests for enhancer site prediction.

**Figure 15:**
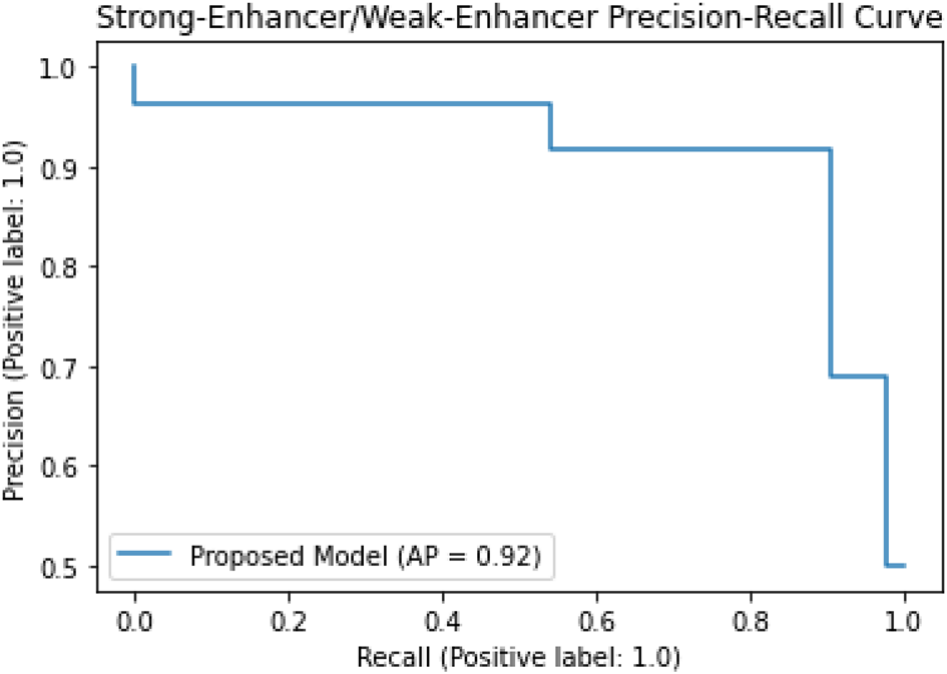
The PR curves of random forest using jackknife tests for enhancer strength prediction.

The classification algorithms with their predictions results using benchmark dataset are shown in Tables 4, 5 and 6. iEnhancer-EL^89^ and iEnhancer-2L^26^ produced better outcomes using ensemble classifiers and achieved accuracy of 78.03% and 76.89% respectively in which they were successful in predicting strong enhancers with accuracy of 65.03% and 61.93% respectively. Whereas EnhancerPred^27^ achieved 80.82% accuracy and used SVMs which produced slightly better results in predicting strong enhancers with 62.06% accuracy. Similarly, iEnhancer-2L-Hybrid^90^ and iEnhancer-5Step^29^ improved the accuracy results with their prediction model and acquired77.86% and 82.3% accuracies respectively with identifying the strong enhancers with 65.83% and 68.1% accuracies respectively. In contrast, 91.68%and 84.53%accuracy was achieved in predicting enhancers and their strength respectively by the currently proposed method after utilizing obscure features from statistical moments and random forest classifications using 5-Fold cross validation tests (see Table 4 and Fig. 9 for ROCs). Furthermore, tenfold cross-validation test was also conducted using random forest classifier on benchmark dataset and obtained the accuracy results are listed in Table 5. The ROCs of  10-fold cross-validation tests are shown in Figs. 10 and 11. The violin plots of 5 fold cross-validation tests are shown in Fig. 12. In addition to cross validation tests, an independent test was also performed using the independent dataset. The comparison of proposed model and state-of-the-art methods using independent dataset is listed in Table 6 and ROC is shown in Fig. 13. Furthermore, jackknife test was also performed on these datasets. A detailed comparison of some selected machine learning algorithms using jackknife test is mentioned in Table 7. The Precision-Recall (PR) curves for enhancer and their strength prediction is also labeled in Figs. 14 and 15 respectively. The proposed method is based on the feature sets that are evaluated using Hahn moments which are easier for the random forest based classifier to classify the feature vectors in acute time and are very efficient as compared to previous methods which were not able to produce better results on the computational cost of training and testing using classification process.

**Table 7:** Jackknife test comparison of machine learning algorithms for predicting enhancers and their strengths.

Layer	Classifier	Sn (%)	Sp (%)	ACC (%)	AUC
1	KNN	70.89	78.77	74.83	0.86
	Naïve Bayes	67.58	69.33	68.46	0.79
	Gaussian Naïve Bayes	71.63	71.09	71.36	0.90
	Random forest	75.26	97.43	86.35	0.95
**2**	KNN	70.35	53.23	61.79	0.76
	Naïve Bayes	57.95	59.43	58.69	0.67
	Gaussian Naïve Bayes	69.67	52.02	60.84	0.69
	Random forest	68.86	97.17	83.01	0.92

### Web-server

As observed in past studies^91–95^, the development of a web-server is highly significant and useful for building more useful prediction methodologies. Thus, efforts for a user friendly webserver have been made in past^72,96–99^ to provide ease to biologists and scientists in drug discovery. The software code which has been developed for the proposed method is accessible at https://github.com/csbioinfopk/enpred which is developed using Python, Scikit-Learn and Flask. The webserver to the current study will be provided for the research community in near future.

## Conclusion

In the proposed research, an efficient model for predicting the enhancers and their strength using statistical moments and random forest classifier is developed. In recent past, many methods were proposed to predict enhancers, but our method has proved to be better in accuracy than the existing state-of-the-art methods. Our method achieved accuracies of91.68% and 84.53% for enhancer and strong enhancer classifications using 5 Fold tests on a benchmark dataset which is currently the highest and accurate classification method for prediction of enhancers and their strength.

## Supplementary Information

Supplementary Information 1.

Supplementary Information 2.

Supplementary Information 3.

## Data Availability

The Online Supporting Information S1 (https://github.com/csbioinfopk/enpred/blob/master/static/Supp-S1.pdf) provides sequence information of DNA Enhancer and non-Enhancer sites used for training, Online Supporting Information S2 (https://github.com/csbioinfopk/enpred/blob/master/static/Supp-S2.pdf) provides sequence information of DNA Strong enhancer sites and Weak enhancer sites, and Online Supporting Information S3 (https://github.com/csbioinfopk/enpred/blob/master/static/Supp-S3.pdf) provides sequence information of DNA Sample sequences used for Independent Tests. The GitHub repository provide access to all the data necessary with relevant accession numbers to substantiate the study's findings.
