# Supplementary material for: A machine learning technique for identifying DNA enhancer regions utilizing CIS-regulatory element patterns
Source: Sci Rep. 2022 Sep 7;12:15183. doi: 10.1038/s41598-022-19099-3 (PMC9452539; doi:10.1038/s41598-022-19099-3)
Supplement: Supplementary file 1 — Supplementary Information 1. [file 41598_2022_19099_MOESM1_ESM.pdf]

Online Supporting Information S1: The following are the sequences of **2,968** DNA sample sites in the training dataset out of which **1,484** are DNA Enhancer sites and **1,484** are DNA Non-Enhancer sites. See the text of the paper for further explanation.

I. 1,484 DNA Enhancer Sites

```
>chrx_48897056_48897256
cacaatgtagaagcagagacacaggaaccaggcttggtgatggctctcaggggtcacagtctgat
gggggacacactggaggtcagtctggtgggggagttttagcctttggtccttatggtgaagccta
gatttgagcctgttcacatattaagtggagatgctattgttcagctctgcaaggggggggtttgtc
ctatt

>chr12_6444339_6444539
gccctcacattccctggcccatcccctccacctcaaaatttacaaacacccggggttggcaagga
caaatggggaggagagtaaaagggaaaggttggggatcctgcatgggtagacgggggacaaagtt
gagacgggggcaggagtgggcaaaggtgtgcaggacataggcaaatagagctggccaaggctagc
gaccc

>chr12_6444939_6445139
gagcaggaggccagtcaccctgagtcagccacggggagacgctgcagctgtaagctgtgcaggcc
cggaagttcaagcctcagccgcaaacaggggaagccacacacagaaacacaacagcagcccccgc
aggtggaggcgtgtgcccctgctcagaggacaacaaccttcccctgcccgcaccagccaacaccg
tgcca

>chr12_6445139_6445339
cctctgctgagaacaggactggggcttccagggcaacaggaagggtttggggtggtgatgggaag
aacttcccagtcctgaaagtgtggttcctttccccgaagaagccactggagcacctaaggtcact
ggtactttgaatcccatccagggggcttgaggcagagtcatgatgaagaactgggggacgccagc
cccag

>chr12_6445339_6445539
acagccttaaagggagcttttcagggacctctggccagtgggggatgagcctccctggagggagg
gagggagagaggctttctcaggccacgtgttcccttctctttccagcactctgggggttaagtca
gatccgggaaagggaaatcttttcccagcaactccgccccagtgactcaggccagagggagggag
accta

>chr12_6641339_6641539
tgcagtgcctaggctccagatcagtgcttctgaaccgggggcaattttgtctgccagaggacatc
tgacaacacctggggcctgttttgttgtcatagcctataggggaagaatgctaccagcatttgtg
ggaagaggccagggatgtggctcaacatcctgcagtgcacaggatggcccctcaacaaagaatca
cacgg

>chr12_6645339_6645539
gtggcatagtggggtggtgaataccatgtacaaagcttgtgcccagactgtgggtggcagtgccc
cacatggccgcttctcctggaagggcttcgtatgactgggggtgttgggcagccctggagccttc
agttgcagccatgccttaagccaggccagcctggcagggaagctcaagggagataaaattcaacc
tcttg

>chr12_7052339_7052539
cctgtcgtcccagcgacttgggaggttgaggcaggagaatcgcttgaacccgggaggtggaggtt
atggtgagccaagatccccccccagctgcactccagcttggcgacagagcaagactccgtctcaaa
aacaaaaaacaaagccggttatgaagcgggggtggggtgggctagttttaataggtccaggcgat
tagta

>chr12_46780933_46781133
taaaaattttcccatttcatttatactccagtttcagttccgttgattcaaagttttgggactta
ggacaagtttgctagcttctctgagcctcagtttttttaaaccgttaaataggaataacagcatgc
tgagtgccaagaattaaagaaaaatttatgtgaaaatatagattagaaagaaaactaatataaat
gcagg
```

>chr12_53772933_53773133
actctgcatattggtcagctcagtattaacttattcggtgagtgctgtcaccagatctcgtcccg
cctgcattcccagggcttgcagcgacattgaggcatctgcccgcctgtccgaccacccgggaggg
gggtaagatttgagaggtactttataggggcagttaaatgaagacgcaaacaagtcctagtgttg
atgcg

>chr12_109085071_109085271
ggcgaagatattcaacttggcaggagcagatgatttgctacatgtggttataaggtggcttctgg
gcaaaaccgtgactccagaaatgtgttcatgacaccttgagtttatttcagagtaatgtgtgttc
agatacctttctttaccggagatacagcagctttcccagactggggttaccatgtttacacactg
agggc

>chr12_109085871_109086071
tggctggcagctcgtgacataatttggaagtcattgttctgcacctggtgccctcccccctgcatc
ctccacccctcccagtcctgtcccccagcttttcacggcccatacacttggcagttgaaggtgct
ccaggcatcagacaatctatttcaggaggcagtgaatgggcatcccacgacatgtcaggacactg
gctgg

>chr12_122884047_122884247
aagaaaatcacatctctacacatcagaccctttccagtccattttgccccatccacaaaatgaaa
atgccactttatttactaaatctaaaaattctgttgggaggcaaaggagagttgaagaatttgca
atgaatttaacctgccaggagagggagtttaacaccaagcttccagaaacaggctgcgtggtagc
tacaa

>chr12_122884847_122885047
aaacaaaaaagtcaactatccaatgtcacggaaaagtaaccaaaaaaaaaaaaattatgggcctg
cttagccccaatttaaaaacatcacatctcctggctgaactgagcttaaggtccttggacgggct
tcatttcccacgtactggctgtttctagagccaggccccatacgctactgccacgcgcagcatac
tgcca

>chr11_6627824_6628024
atgctgccagaaggaaaaggggtggaattaatgaaactggaaggttgtggtgctggtttgaggag
taaagtatgggggccaaagttggctatatgctggatatgaagaggggggttaattccttgcaggtc
ttcttgagatagaagtccaggccctgaggtggcaggcagcctgatagtgaacagaacccttgtgc
ccata

>chr11_9587224_9587424
ggcatttttttaacctgtgtttcattttcatctgtgaaatgtgaataaaaaataactctcttacaga
gttcccgtaaagattaaataagtatgtaaagcatctgggtcagtgcctatcatatagttggcacc
aaaatattctcccctctcccagctcctgcaaaggcaccccagctctttgcagcacttagggcctt
tctca

>chr11_46576424_46576624
atcaattctcaatcaccaaaagcttatccaagtagaagcaaaagacatggctaggatatgtacat
aagacaaatgccggtcaggcatggtgggaggccaaggtgggaaggttgcttgagtccaggaggtc
aagactacagtgagccatgattgcaccgctgcactcttcagcccaggtaatagagtgagaccctg
tctta

>chr11_47428824_47429024
ttgtatgggcccggtggggtgagatggacagaaagcttcagaagacaagctgggccaccagggag
aagttggcggtctaaggaaggccaacagtgcaacatgaagtgggacaggcaccagaggcagccct
gagagctgagtgtgctctcaagagccccagcagaggggaaagtgacagagtaaatggttggagct
gccat

>chr11_61738824_61739024
gccaaggagcacaggaggaaatgaaggtttcggggaaagcccttggggaaggcctgcttggtttt
ggactagatgtgctgagtttccaggcttccgggatgtccaggggggacaaagccagcatgaagctc
tcggacccactggagggaagtcggtctgctgcagggagtaggtttcctgaatcagtgttggctcc
atgct

>chr11_62320824_62321024
ctcccctccccagtggggagcagggagggagggggctgaggaagcggggaacaccacctcctcacc
cctttcttggaatctgcttttctcctgtcctttccaacttcctctgcctttcccccagcttcctg

gggaaagagagaccagcctgggtttccctctctcctctgcttacagttccctaagtccagttgaa
ggaga

>chr11_62321224_62321424
cgggtgcaggaagcctttagatgcctcgctctccctgcccctccacacttggtatgctgtgtcca
ccacccttatccctcagcatttgagggcagcatcctcctgctcagctttggagcagaagtgactc
agtgagtgactgcaaggattgtggaaaagctcggatgcacccttgataagtcacttcaactctct
gggct

>chr11_62647224_62647424
cccaactggcactgtttaggaacaaggagttcctgagagtgttggcaccccacggtgttgatgtc
cggtagttccctgctgtgaaccctgtctctgccccacttcaatccaaggaccctgtggagggaat
tctaggcttcttcaaagcctctgcagtaccagctgcctccaaggcctaacatagtgaagtgatag
ggaga

>chr11_65187024_65187224
gaaaccacagagctgacctggcttcagaacaagatgtggggctccaggcacccgggagaccagtg
accctgattggcaatagccgcaagaaagggcttggagacaggggccaggcgcggtggctacccct
gtaatcgcagcactttgggaggtcaaggcaggtggatcacttgaggtcaggagttggagaccagc
ctggc

>chr11_118783790_118783990
ctctgggaccctcgatgtggggacttccaggcttagggggccttcccatggccctggttcctagt
agctctccacacttcccccgactctgtgccaaaggagaccatgcaaatcagccatttttgcttgg
gaggttttccctgggagtttccagccccaccccacctgcagccctagggtaatgtcagacaaccc
cagct

>chr11_118783990_118784190
ccacaggaccagggaaactcctggtcccagacctttctagtctgggattgttgaacagggtactg
gggggtgatcggcggttttgcccattctggcccactgtcccttctggagcctgcacagacaagtg
gcattgcttggacaggcctgaggtcacctcacttctcttggctcctacagtcattgtcagtgtgg
ccggg

>chr10_74014594_74014794
tttgcataggggcattaccactggacttgggctcagagcaagtgtttatgagataaatgaggtgc
cgtctcagaatcacagagcactcctctacctttgaccttctgcaggctttcactggcttctaaga
gccaagaaccttctccgcccccacccagctctctctcccctgcaatctctccatgaacagcattg
tttta

>chr10_82219420_82219620
ccagctgttgggcctgtgctcctgtacactgcacggccttgagaggagttcggagccctaacatc
caggagagaggccccacagcagtggaaggaaatgggcctctcccgaatctcttgtttgtaccccg
aggtctgagtggtgatcctggggatgctatgggactctcagcagtaggagtgtgtctgtccccag
tctgg

>chr10_103911010_103911210
gggaccacaggtgcatgccactacacctggctaattttaaaatttttttgtagagataggggggt
ttcactgtgttgtccaggctggcctcttaactcctgggctcaagggatcctcccacctaggcctt
ccaaagtgttgagataacagttgtgagccactatgcctggccaattttatataggagagggggggt
gtgtt

>chr10_105667810_105668010
cgggaggcgggggttgcagtgagccaagatcacaccactgcactccagcgtgggccacagagcga
gactccatctcaaaaaaaaaaaaaaaattagcacatgaacatttgtcctagagaatgcgaggtca
ttctggtcttgactgtagccttccaatattctaggtctaagctcgagttgctaccaagtgttctg
tgcac

>chr17_7478276_7478476
tgatctggttcccattgtgtaactgtgttgattgggaaggtagtttgtgagccatgaaatgcttg
gttcattggttgcttattgacctcattaacctaggacttgaatatcccaaagggtatgctcttta
ccacattcaactcctaatttatttgtttaggttatgatgtgattgctcaagcccaatctgggact
gggaa

>chr17_7482676_7482876
aactgccaagcaagtttcgctgagtttgacacatggatccctgtggatcaactgccctaggactc
cgtttgcacccatgtgacactgttgactttgccctgatgaagcagggccaacagtcccctaactt
aattacaaaaactaatgactaagagagaggtggctagagctgaggcccctgagtcaggctgtggg
tggga

>chr17_38269674_38269874
gtgggcggaacgcgctgaaagttttggcggggctagcagagggagaaaaatacccaaatttgagt
tgtgatttttaataggggggaaaaaaaaaaggcaaggagggtgtgtcctaccctctgagaggcccc
cgcagaggggagcaggagagcagcacactagcatcctgctcgcggaagcctggaacgcaggacat
ggttt

>chr17_38270074_38270274
acccagatgaatcctctttccctggggagtggccaaaacaagtctcctctttttctacctactt
ctgccccactgttagctggaaaacgtagctttatacagtcataaagctgcaagggaccttaaaga
tgatttatttccattttatttttggtctgggaaaaccaagcccagtcaagtgaaaagtcttgct
gcatc

>chr17_42173274_42173474
ctgctgggttccaagaccttgggtccagtccccacatggacaatgggccgagtggctcagggagt
gcacgaagttccaatgggcttatgggagggctaggtctccacttctttgtacctacacacagttc
tcaggtcactgcatgtcactcctcaccactgccctgtggttgccaggacaactgggcaaacacca
cacca

>chr17_57923018_57923218
agggatcctatcccttcccaacaaggccaagagttcacctgaggtaatgcatttgccttggggggc
gaggccttgaattgcagcaaataacctgtgtgtatttgcatatttaaccagtactcttaagaagg
attatacaaaaacctgttccagacagctgcttactcattgcctttaaaattgcgtggtgctggga
ttggc

>chr16_2301999_2302199
cccggcctcctgccccctttgggaacaggatcagagctgcaggtttagtcaccgaaatgggggtgg
gggcagcagaacccagcggtaagaacaggccacccgccttgctcagcacaagggcaagtgcacaa
aggccggaccgcagtggcaccttttatttgcagagaaatgccgaaagataagaatacagtctgt
tcccg

>chr16_9141899_9142099
attttcagacttcaaactcatcccagcaatgtagaggaagctgatgagggaaagctccctgcagc
gtgggctgtgcccccaggagcccctgagtttaaattcagtctctgctacttactaatcacaggat
gttggcaaatcgctgagcctgcatccccccatctgtcaaatgggggtgctaaggcctacctggca
agatt

>chr16_9142099_9142299
ccttcatctgatcagtgccaggcgctggggagatgatagtaaatgacacagacaaaattcctgcc
cccacaaagcttacattctggcgaggaaagacaataatgaacaaatctatatgtacaacgtaagg
ccagctggtggggagcatggtgaagaaaaattaaacgggtgaggtgtagaagtaagaggaggtaa
gagga

>chr16_21514099_21514299
gggaagggagtacccacgaccaccaaaccctgtcctctgcaatgtgaaatgtgctttgaagtcct
ctcaccctgaggcttgcccgccttctgctggtgaaagaagctgggggctaggcgcggtgggtcac
gcctgtaatcccagcactttgggaggccgaggtgggcggatcatctgaggtgaggagttccagac
cagcc

>chr16_53132499_53132699
gctttttttctttgtctcttgtaaaccaacatcagcaatgagcaggccacctcaagtgtgactcaa
tcaacgcgaggcaggaggcatgcaaaacaaaacagaaaactcctggcagccttaatatagcgatt
tttctagcaatctacatgactatccaggcccaatgcaccccattacatatgcaaaacatctgacg
tttgt

>chr16_67275699_67275899
ggttgggtatgcactggccaacacaggctgaagcagcacagacccagaaaggtcaggcccaccac
gctgtcctgcctccttgcatgctgtaatcccaagagagcagagaatgtccctctccttccaggac

acctgcatccatttggggaagggtcctgaaggttaaatagtccaccttcttttttttttgatat
ggagt

>chr16_87421899_87422099
gaagacattctgaagactgcagtctattcagtgtcaacccctaaatcctcctgaccaaatccttt
ctgagaatagctccctgcactcctcttgagcaatacttgtttgtttacttgctctcaagagcaaa
gcgatgacaccacgatggataggacactgggcctatagtggtgcagacgacaaagccggtggcct
ctctc

>chr16_87812299_87812499
ccacaggcgtgtaccactacgcccggctaattttgtaatattttgtacagagatggggtctcatt
atgctgctcaggctggtgacaaactcctgggctcaagcgacccacccacctcggcccccaaagt
ggtgggatcacagacgtgagccagcgctccctgtcagcgtttcattccctcttcatatcacatca
cttgg

>chr16_87887299_87887499
cctagccggcttctggagagctttctggattcgcaagaggccggctgattgcatcactcgctcat
tcatgcccggagggaggcggggagtgggtgtggccaagccgcactcgaggctgcctctccccaaa
agggagagaggcacgctgccaccactgaactccagacaccagacaggcgctgcggagacactgag
cactg

>chr15_58624108_58624308
agcatctccctcctaattccctgtgatgtgataattctaagacccacttcctggagcaagaagaa
gctaagatccctcagcctggctgatgtggttttgaaagtggaagagggcaggaagtgttgtggtc
actcccacccagctgtgggcctcttcccagagccctcctcaccagatgctgcaaactcagactgc
aaatc

>chr15_89181596_89181796
gacccaaggaagtgtggccgggctggtggggcctccccgaaggcctgcttctccttcaccctcac
agagtcagccccactggaggcagagctcattgaggcatctggaacccacggaggcctgtaagggc
tccctcgctgctccttgccttccctgggttggaagtgggggctagccactcccaccacaagtttc
tcctg

>chr15_90943596_90943796
ctttgtcctattgtagtttatattttaattaattccttttccgtctccattcttgttaatagct
taaatgatctctttttagttcatgaagaagagtgtggcacaaaagatcctttcaaggtgaaaaaa
aggagcaggtagccaagatagttgggttacatagtttaccacttccctttataatccgtaggtgt
tgaaa

>chr14_61569847_61570047
tctgtgggtaatattgcactttgtaaacacttggtactcaataaattccagcacacatatggaag
caatggtgccaactagctggattgaaagtactcgatacccagtgtccagagcattggctaatgga
gaggaagaggttccctccacttctgactatgggtgtgcggcacaagcccctggcgggcctcacac
accct

>chr14_61570047_61570247
acttagcacctgtcaatctcctgtcctctgtgaaagagcctgcatttctccctgttgggcccgcc
acttctccaggatttttcagtgctgtctgtccaagtctttcttctgctggaagtgtgggaatgg
gaagtcagctctagccatggctacaaataattcacctttccagcagcctgcgggttttcttctta
ccggt

>chr14_61570247_61570447
acaacagtgagtctcattctcagaatctctgaggcctggccagtgaggcccctattcttcttccc
ctggcagaggccagctctgatggtgtccccacccaaagggcagacacccatgtgatttcagtggt
tcggaggaataggaacggaatggaagggtgaggtcttttctgctgaagctcaaagcactgtggat
acagg

>chr14_74208847_74209047
aactagaagtgcgtgtctgcagaagcaagacagagaaaggaagaatgtgcgccagcctggcaagc
ccacgtgcctccaggggggctgcgagcccctccagtgggcgctcacacacactcacgcctcccttc
atcacagccctgtctccttgtgagccacagcacgggcttgaggcctggagcaagggaaggtccac
gtggc

>chr14_77422047_77422247
caaacaaaaccgagaaagtctctgccctcaagcttttattgaaggagggaaatagacaaaagaga
agcaaacaaacaaaaaaggtaaaatcagagggtggtaaaagctatgaaggaaataaagcagtata
actgcttaaggagcagggacctctgcaggtggatggcaagggaaggtctctctgcagagacgaca
ttgac

>chr19_4374400_4374600
cagggccagaaggacctctcctgtgctgacagcaaagtcaccagcgggagtccagcccacactgc
ctttctgatgaggcctccagctctggctgctgactttaaaccctaaggaaccctgtgcagggcct
gcatcccggaaggcctccaccagcccgaacctggcccgtccgccctagatggggcaatcggcgtt
tctcc

>chr19_4375000_4375200
cctggggctcccatcaccactaccagtctgcccaggaggcagcagagcactgagcccgacctgct
cccctcgggcacctacgagacccacctgcccctccatgcgtgggggagaaggggtgccctctgct
gtggaaagctgggacctactgtctgtggtgcgaggctgaccccacagagctccaggacaaccccg
agagg

>chr19_4375200_4375400
agtggacagagccagccgggccctccagccccccacctggcccagctgccgcctctgggcagatgt
ctccacttgataaacaggacagctaagggcctaggagagacatgcctggcccaaggccagacagc
aggagtggctaggaaggtcaagggatccccactgcaggccccaccaaccctggctctgcctggaa
tgcag

>chr19_6738600_6738800
ttgcccaactgcaatcagacattttctagctcagaagccaccagcaggtggggccaggtctcagc
agagacatcagtcccagtgtgggctcccaaggctctcctcctgggctcagctgtatctctctgtt
ctgggagcctgggtgctctgtgtaaggggaacgtatacccaagggccccccctgcaagacgcccag
ccttg

>chr19_13275200_13275400
tccagatggggactgcagggagggctgacccccctcccgcatacaccaggcgggcaaggtggagcg
cccctcctcggaaattgccccccgcccaagatgcgtcagtgacagctgtgcctcctcagcccgca
cccagccggtttccttttcctgtgcacactctcccgatgggctggccttcagcctggagagactc
cacgg

>chr19_13275400_13275600
ccccttggaaggggctccccagagggtcttcctggacgagggggaggggacaggctgaagaacaa
tggggcttctgtggctgcaggacgccaggcacttcctgttaaccctttcctcggggtgcagctgc
agggagcacctcccaggtcccgccttgtgggaaggggcagaggcagcctgaggggaaatcttgtt
gcggg

>chr19_14629600_14629800
ctacccagtcctttccccgggaggcgatgaacctgctcgcccttgtctcgtggggcctgcggtgg
gctcccgcccggtgacgccggacgaactacctaacctgtgtctgcctaacccaggggcccccttc
tatctacaacatggcaataagagaacctatcaggccgggcgcggagcctcatgcctgtaatccca
tcact

>chr19_18393600_18393800
gcatgcacccctccagctccccacctctgtctgaatggagcccacaccctcctttccctgacctc
cttcccctcactctctctgatccttggcctcccagttgcaggactaatcgtacctctgggccttt
gcacgtgctatgctctctgcttggtactcccttcccccagtgcaaagatgtgtcttcaggaagtg
cccca

>chr19_39173960_39174160
cccctgcctctcctctttctcgcccccccgccctcctccagagaaagcaggtggtgagggctctct
aacccagacaggacttgctgttcagccccagcttgaaattgatttcctccagccctccttgagcc
aggccccgtgaacagaggaggggtctgagccaggcctcctaggccattggtgggagggagaatga
gacag

>chr19_39174360_39174560
tgaaatctgtcatcatgcccatataaggcaaactccttggattactttttaccttggcagaatca
caggaatgtcaaggtaaccaggccacctcagcatagctctgattctcacccggtcacctgacttg

cccgccctcccccatgactcacccagtggctcagcatggggcctgctgcactgtggctgctggaa
tctgc

>chr19_39174760_39174960
tcccccagcattcagagccttatctgtaaaattagagcacgcagaacaaaccctggtacacacta
agtgctcaataaatgtgagtcatttgtgtaattatagtagggtaggccttatgccccacccagtc
ataaaaatgcctcttgctgttgttggttcaggctcagccccttagtggctttcatggggcaggtt
gacat

>chr19_39175960_39176160
tttaggtaactcttaacaccacgaattagttgcctgctggcaaacttcaaactccaagcctgaaa
tttctggttggagtcacctcccagatggactcagggattcattcacccccctcatccagtgctcac
ggagcctctgtgaggtgcaggcctggagaggagccctgtggcctcagcaaagggcaggcaggctg
gtgag

>chr19_39177160_39177360
agcctctgctgctgccctaattttaaaactgccttttgggagtgtaagtttcctctgttaaaggt
agttatttcaaggtaggcctcaccatctcctcctcctggtgagaagctctgcctggagggctgag
cactgcctcccgctctgtgggccccacctgccttgggttgagacctatctcttcctggactctgt
gtggg

>chr19_39893160_39893360
cacacatactgttcacacaaccgggtacacatgacggctagcccaggcccgccgcccacgcagtc
gcaggccagggtacacacggccagcgccgcccttggccacgcccgctcaggcccaggcaatcgcg
ccaggcgcacacacggcgcgagcacacgtgccgcgggtacacgatccccagctcgcccgcgcact
cggcc

>chr19_39924960_39925160
ccttccccagttctgaccactttggatcagcttctggtattgggcccagtccttctccccaacta
ggctggcaggtccatgaaggcaagaccttaagtcatctggatcactgctatgtccccaaaactca
acagagggtacacatacaaatccttcaaatacagtgacagccaataacagtaacattcaacatct
gctac

>chr18_3596800_3597000
agcaaagccattcgctgtgatctctgattgtgcagtgtcatgtcctgtcaccagagccccctcgt
gtttgatgttggccaatgccgccagcatgatctagcaggccaaatcctaatctaccattctctga
caccagctggtcccctgggtcgtccacccgatgtcccccattctccccacttggcctcccccaca
ggctc

>chr18_3602800_3603000
aggtcaaagacgttattaagtatcgactctgaaaaagcaactataaatactgcaaatcgactaag
ccagaggacgcaaatatatcctgcgtgttgtgtgctggactgtggttgagacttcatggtcgtgc
tctgggctcatgggggcagctgactgctcataattaagattgctgttgccgctgcctgtgttcca
aagag

>chr18_3606200_3606400
tcattcctgagccttggtgccttgacgagtcaccacgctcaggaagaaagctgcaactcgccagg
atgaaactgggccccgcttcttagaacccttccccgcttccttcccctgccagaaccacactaaa
gaattcgctgctctcaaagacagacgttttcattacttccttacattatagagtttgggcttaga
taacc

>chr18_20839802_20840002
gcagcttcagccccttgtaagccctgccccctctcaggactgccctgaggcctctccagtcctcc
ccacactcagagatctgtggggaagctccgcccagccacactccttgggataatactagccggtt
ctgcctgattccttttcccccggagccagcctagggggcccgggactcctctagtgagccttgac
tgtta

>chr18_20840202_20840402
tgcaaatgggttttgttttccttggcattttgttgttccgatcacagatttccctgccagggtgt
ctgtggttatcagctgcaggctcagcttggggcggccgcttcacacaagccactctgtaccacgt
gccctaccttagtgacgggagtaaggagcttccttcccctccatgtcattccttcctgttccctt
cattc

>chr22_30592400_30592600
aggtatcctgtcactgggtgctgagctgccggcacgcctcctatcaccattgccatggccagcgt
catcaccatcgcggtggccagcgtcagcgtttgacaaaggaagtactcagttacaattaccttcc
tgttggccagcagctcagataattgctttgcaatgtgtgtgagaacctcataaattaaaccaaga
aacag

>chr22_30592600_30592800
aggggccaggtctgggctgaagggaagtgtcaagtggaggaggcagtgatgcaaggctacctttg
aagtcactttgtttgtgaaaagtctgctgtgctctcttaaggaaactccactggaatgaggtttc
tgtgcatgtggggtttagggctattgtttttggctctgactttcacctcaagccctagctaggac
atgta

>chr22_35772600_35772800
agagtgctaggattacaggcgtgagccaccacaattggcccagtctatggatttttaaaaaataa
cttgggcttgtcttccttgctgaagaaggctgttttccagcctgtcacacagcagttaggctgta
gaccctcactggagcccctgccctgcagaatcgagcacatgttctttctgggtctgtgtggggtg
tgggt

>chr22_35773000_35773200
ctgcccaaaccacttctgtttcctgaaggcgccttgggaatgctgagtcgcgatttcctcatccc
ctcgtgcagctgcatttctgctgcgtcatgtttgggagggggggactcgcggaaacaaaggggaagg
cggattttgctagattttgctgagtcaccagtgcctcctcagcttctctttaggtgggaggtgaa
agggc

>chr22_36726454_36726654
ttggtctgttatcttgtgtggcctccaataaccttacaaggtattgtattgtaatgtacaaagca
ggtattatcatcattgttttgccatctttccacacagcggttcttctggatttgttcctgatctc
agaggcatctcttctgggcagtggggtggttagaattcttagcaaccagtgggctctttggccta
ctcag

>chr22_36727054_36727254
cttcccaaattcctgagcaagggacagtgggtggtggagacagggttcctcagagcacagtttga
caaccactagtccatgtgtctggagcagttctgcaaaggaggaagccacagccgagagcgctgtg
gccctgggttccgctcttccccacgcaatgcctggccccttgacctctctgggaatgaagggact
gctct

>chr22_36727254_36727454
gaggatggaaggaaatggtgttcctgtcgtacacggtgtgaggcagatgcccagacatgccgtcc
tcacccgcctgctgtctcggagctcccaactgagcaattccctggcaccgccacgagaaagtctt
tcataaaagttacaaattcaggaaccaggccattttctccctggatttcattttcaaccgaaaat
gtcct

>chr22_36727454_36727654
gaggccaagcccatgcagtgagggcatctgagctgatgtggccatctacctgtggacaggtgttt
ccagccagctcagcaggctgggcccaggagggaggaaggggaacctgaccgcaggtatttcatcc
caggtgcagacttctgctgggggcttaagcctcacttcctctgtgagaggagctgcagctcactc
ttgtc

>chr20_10492200_10492400
cagtaacagcaagcacaaatgactaatttaagaaaagcagtacagcagctggacgtgttccatct
tctttgatgctcaccacctccctgtgaggaaagtactcttactaccccagcttcgcagatgacac
ttggaaaacgtaaggacccacagttaggtggccagactgagagcagagtgctgagtcaccacacg
gtgct

>chr20_30160339_30160539
ggcaccgtcagtctgacagcagggctggctcctgcagtgtgaatgaagcggtcttgtgctcgact
gtgaagggggggttaggacctgcttcccagggcgggtccagctttctgagggaaagccttcagagc
ctccatcaggccggcaccaggtaccagttaagcactgggcagggaacagaagtggtcaaacgccg
cttgc

>chr20_30160539_30160739
cgcgacttgaaactgctgaataaatggataatgtcaagcgctatggcttagggcagtagtacctc
cggttcctacgatcgtgtctggggtccctgaggagcaaggcaagagtcggccattcaggaggtca

gagttcagctgttcttgcacaagcagggaagctgagtcaccccaaacacgggcaagactagcggt
ggtat

>chr20_45946593_45946793
taacctctctgtgcctcagagagtttaccactttacaagtggtaccttgtaaagttatcgaagaa
gtcaatgagttaattcacgtaaagctcttagtataatacctggcaaatagtaagcacctattcaa
tggtaactaatgggtaactgtattttttttaaccaatcactcttcctccttttcttgcttctctt
ccacc

>chr20_45977593_45977793
tgaggtgctagaactttccaaacattcaggctgacactgaagtggtaagaagctgggcatttact
ccagaagcaattccatcagactgtcgttccacaggaagaaatgggtgcatcaccaattgatatgg
ctggaattttccatttggaagactcaacgccaggacaggaaggaggctggggaagagagaatgtc
ctgtc

>chr20_45977793_45977993
ctggggaagagagaatgttccgtggtctgggactgtgacaacgcaaaaggcccaaaaagacagcc
tcatgtctcgaaccctagtataaggagaagattttaggagagttccacgttaggaggagaggact
taggatgggagctagatagaatggaaaagattatagttcagagttaaattataggggcttggaga
cgaag

>chr20_45978793_45978993
taatcaatgctggagcagaagagagagggaaaaaaaagttaagctgttttatctttccaagtatt
acaagtgggcaccataatcacccaactttagcaatagcaccaccagctttagcacagcatttcaa
gcacaacaatttaagaaaaccatctttttcacagatcagcaaaccatcctttcaaaaggaacccg
tgcac

>chr20_45988593_45988793
actgcataatctgtgttttcttttaggaggtagaccagtttttttgctttttttgtttttttgataca
gtgtctcgctctgtcgcccaggcttgagtgcagtggcatcatcacaactcaatgctgcaacctga
agacccaccctttttgaggtgggttttcagtattgaatgatggcgctgaaggaaacttttagatta
gactc

>chr21_35320130_35320330
gcctccccccaaccctattgtaaaatgtcaagtgtttcagttttgagaaaaaagaaagctgaaga
aaccatgactcatgacatgggctgacaaacaacgatctgaaggtatgctggtgagtcacactgtg
gagctctgcctttgtgttgtggtgagggccggggctggaaggagtgtcatgggaccagccagtaa
aattc

>chr21_35320330_35320530
cactactctccgaagccgattgggaggcaatttaaaaaccctgggctttttccagccccagtgta
gtggatgactctgggtcccctcaggtatgtcgatctgtatgggaaatcaggaagaatgattatta
ataactggaaaaaaaaaaacctgttagtatctttccatgcccaatgccatgctgaaccagccctgc
ctgct

>chr7_44679475_44679675
agcagctcagcagtaccggtatgaaccagttttttttctgactgcctccagcatgagctgaatttc
cgtctgtgcagttatcctcagccaattgaaaatcacctggagttgtattccaagcacaaaagaag
gtcagagagtggaggcccgatgatcatgatcgccctgtctccagggcctaggctggaaggagtcc
tgcag

>chr7_44679675_44679875
cctttgtggctcaggaccagagagctgaccttgaccctgaccttgtgatcccaggcatcagtggc
tggaaattcctttcattttattgttgagcccagaagcgcccagctctctttggcaaggttaagct
agggtaagaggcactgttactagagtgaccagagttctttaagcgtcgctctgctattactcagt
taacc

>chr7_104585764_104585964
ttccccagggactgggcctagctcaatcgggaaaagtcagcatccaaaagtcctcaatgacgcag
aggaagccaggagaaaacagtcaatggctagtcatcactgattcgtggtcacaggcgttagaaca
ggaaggatctgcacagatcagcctctcccacccctcactttcctgataaaggaactaaagtccgg
aaagt

>chr6_7146401_7146601
aataaaacactttgtggagcctgttgtgtgagaagcaggcagcggctgtgggcctcagccttgcc
atctgtttctagagcagtctgtggcccgcttaccatgccctctgtcatgtaacataggccccatt
cagcagcagcattcttgaatgtgcatttatgtctctgtgggaccgggggtagatggcctgagagg
gggaa

>chr6_26025821_26026021
ggagtccaagaactgagccagagtacaccagagtatgtagtggagtacagctttctcattcttta
gtagggctgtgtagagaaatactctattttatggatgtatagatcactctagtcctttctgatga
aaactttacagttgccactcatttactattacaaacaatgctgcactacattatgtttcagtttt
tagcc

>chr6_26026221_26026421
caaagatgctgaatatttggctgacttctaaaaatctggtcacctaactttgacacttcctaggt
cgtacagtttgaaactttcacaattaaatcagttttggaatttacaatttaaggcaggaatagaa
gactattgggttgacaggtacagtgaagatgcaagtcatctgagaatcttacatcagagggggct
tccat

>chr6_26122621_26122821
tgttctagacttgaataaaaagataggtagactacgtctaacccttcatcttaaaatctttacct
ggaaaagaccatgagtaaaatacttaagggaatgtggaatttcccaggccacaaagcggcctgca
gttgtctaggaagggagagtcctctaggagatacagtgtatgtgctaagtttaatgacgtctcct
cttca

>chr6_31706021_31706221
gattccatgcctctgtgtgggtgtgaattccctcatggtgaccctcaaaatctgcacacaggacc
ccttcccattgaggggaggggatcaaaacaactctacttctcagggtcctctcctgttccaactg
gtctgtgtccaagagaagccttaggtaaatggggccagcttgaagatcaaacaggtttggcagcc
tctcc

>chr6_35437822_35438022
cggggtcttagagagggtgctgcttggaggtcacttacatgattgattcaagtgcgtttctggcc
tgccacatttgaggtgtgccttggtgaccaggttctaagcaatgccaatggcttcctctctctat
cagccaagaagtatgatgcgtttttggcctcagagtctctgatcaagcagattccacgaatcctc
ggccc

>chr5_79542844_79543044
cagaacactaagaaaacactgggtctttacaattaggcaggtatggccacttaaagtactaaaaa
tagcttggtcaaagaattacatccaatggctagttctgtagacatatcagcttatatgaagactt
ccttttaacatcaagttatcacagatgatctggccacatactgctctgaaataaaagtagacaa
tgcca

>chr5_133838901_133839101
taggctttagatttttggtgctaaaaccagtacagtcccaggcaaaccaggacagaacagttgg
tcaccctaggtagtaatcattaacccagagagggccaggcttcaggagccagctggccagggttc
aggtgtggcccccagggccccctccaggcattgccttgtccttgtggtttgtggtagggccttgt
cccat

>chr5_179246594_179246794
gcttggccagcacctgtctgtaggccacagcctctgccagcacgcccctctgtgtcccctgcccc
tgtctgcaaggcagtggctccagcaggccctggggcattttccactctccaccgccggatgcagg
gagaggcctgaaccctctccacagggctgctctgggcagggtggaagccttgcccacttcggagc
cctcc

>chr5_179246994_179247194
cttgcctgggaggctccagagggtgaccactcgtcctgcccctctccttgccccagttctggcgg
acaggttactctggtggcataaagcagtgtttcttccttcctagctgaggaggctgttggctgac
ccccttggctgcccacaaggccaacgggcctgagcccccacagggccatgggcattacctgctga
attga

>chr5_179247194_179247394
ggagcccataaggagtcacttggaccacagtgaacacttggcgaccactgacactcaggagacct
tagctggtcctccagcacctctcaactccactcctactaaactgggaacttctctggtgctcagg

ccagagtcggggtccgtcaccgagtatgctatgcgctgcccatcaccgaggatgccatgcgctgt
aagag

>chr5_179247394_179247594
ggctgccaccgcggcaggctgaccatggcagggtcggaacagcaacctgagagccagcttgttct
ggccagcagtgcccactgggcgacctagcagcctcctgatatgggggctgtgtccccctctccct
gcactgggtacccccaactgaggatattgctgagtcatggccaggcccaagcctgggaggggcga
ggggc

>chr3_39192396_39192596
tactcctctggagaggcgggggaagtgcccctggttctcatgttcctctgttccacctcggaatc
cagaggctaagaggcagaggcaggagatgaaacaaaaagactgagccagagacaaggagaggcca
agagggaatgaaacatagtgagaatctaagaaaagtgtcagacagtgagtcagaggtggggggag
gggcc

>chr3_39192796_39192996
caggcagccctcctgccaccacacacaggcactgacaacccccactcaactggccacagttatc
cacacgctcaggtcacactgtgcaaatccaggaaaggccacctcacccctgggcaccaagtaacc
aaaggctgtttttgagaagtgggggcggggcttgaaaacgtcacagcaactcctctgtccacctc
agcag

>chr3_39192996_39193196
tcctaccagtgccccaggcctcaacccaagccagtcttagggagggggcacctttagctgcctcc
atccccttcaggaatctgaagaatgtgtcctttaacctcagagccccaggccttggccagtccc
cccaaaacacttggggtcacatgacccctgtccctcaggaacttcctgggagaaggggagggcac
ctccc

>chr3_39193796_39193996
gggcacggcaagccacagaaggcttaagtagggagtgacaatgccagatttgcattttaaaaacg
tggctcagcaccgggagtggagggggtgtgaccggttggaggtgctgccagggccaggagagatg
acaggtcaagggcataggagaaaggcagataccaagatgtggagaaggaagagggcagggctgtc
aagga

>chr3_49066196_49066396
gtctgcagtgaagtcgatgtacccagggagaatgagaaagtcactgcgagggaagggagtgaatt
gggagtaaagctctcagcttagggcagcatgagactccatccccatgtaccaatcaacccgtaac
gcatgtgagcagagagtggcactgacagagtctgtgtgtcacaggcacagcacaaggtggaaatg
ggtac

>chr3_52028760_52028960
cacgcactcagacaaggccactccttaccctaagtgccactgccaagcaagggagatagccaaac
tgccctcaggaggcaggttacaccctgaccagagggttacacttcaggctgtggagaccaaggaa
agctttattatgggtctggtagggtagaaaaaggaattgcaggctttgggtgggccagttaggct
gtgct

>chr3_53303760_53303960
ctaattttgtattttagtatagacgggtttttgtcatgttggccaggctgatcttgaactcct
gacctcaggtgatccgcctgtctgcatcaatattttaaataaagatgttaatgctccacaggttg
tgcgtctttccccacgtgagtccacattcttttctaccctaggattactactggcacagaggaag
ttgaa

>chr3_141086710_141086910
taattaccaagcctcatgccaggcactgagatataaatatcccctttaaagagtggtcatgtctt
aaatccaggaagagctagttcatgttggcacccagggtagggagcgaagcccagggccacagcac
agaggcagaggagaatagacactgaccaccaggagctgcccggggatggtgacaaaagtgccttc
cacct

>chr3_177077106_177077306
gcgcccagtgcccccaggctgcgacagccaagtcggtgggcgcgcatggcggggcttgaaggaaa
ggtccagagccggtgggaaaggccacgccccggggtagagcgcttccttccagatcccggccttt
aactgcaattccgttttaagggcaaccctgcccggttcacacctagccttgaagatgacagattg
cactg

>chr3_177077306_177077506
tgagcaatttgcagggtgagaacgggagcctacgactccgcacctgcactgccagagccataaat
gcagcaaatcatggcctataaaatgtcaccactgctgaagtgcaattttccaggggtcttctgca
gagggaactccaccacggagagcgaccctcggctctgcagcctaagaggaaggcccctgcagggt
ggggg

>chr2_43446296_43446496
gccctcacacacatccacacatacttacacacagacacacccatgtataccaacacacacgcccc
tgcgtacacaccaacccccacatcacaacagacacacccacacccaggcacaccccaaagatcca
catacctacatagcaacagatacacccaaacccacaccagcactcacacacatccacacacactt
acaca

>chr2_70370696_70370896
tgccccagcagttggagcagtttggtgagagagtggcacaaagctgacactggtacatggccctg
gccactagttgttaagttccacccatctccagcactgccctccccatgagtgacagtgaaaggtg
ggagggacgttacaggactgtatcaggcattagacctgctactccaagtgcagtctggtgcagtc
tgcag

>chr2_87785885_87786085
ctaaagagcttagctaggcatggtggctcacgcctgtaatcccagcactttggaaggtcgaagtg
ggatgattgcttgagcccagaagtttgagaccggcctgggcaacatagtgagacctcatctccac
acacacacatgcgcgtacacacacacgagcgcgcatgtgtgtgcacacacacacaaattagccag
gtgtg

>chr2_101620168_101620368
ctgcctgcacacttcaggcccctgctctttaagcatagatgaagtcaatagcattgaatgatctg
ttccaaaaacatgtcctgtcttaagacatgacatttaatcaaatttagtattttgattgaggact
tcagaaagtggattatgcatcttcttgtcctaagtgttatgctaattgctgggcattgcacactg
gtggg

>chr2_178029954_178030154
actccttcctcctaaccagcttcagctttcttcttccgaccacagactctccaagattttgcttt
tctcatcctatccttccctcttcttgtacaagcgaacactgtccttccagactcttccatggctg
cagaagctgtagggaggagccctcggccagccagacaaaatgcacgtaatattgctcactttagg
aaaag

>chr2_202022955_202023155
cctgcaccacacagctgtctgctcacagtcatgtgacaggggcttccccagccagaacctccagg
gagcacaaataaaggaaagagctggaaggcacagtacagcatttgttctgtccaagccttacata
tcagcatgggcagggagaatgtggtgaagttttctttcaaggactttgtttgataaggaaggaca
tttat

>chr1_8933213_8933413
ggcttggagtctacagacttttggtgatgataacgtgttcacgcatgttgaccactctagtgggg
atgctgacaatggggggctgcgcatgtgcagaggcagtgggtactcatgggtttatgtacctct
gcaccttccgcctgcttctgctgtgaacctaaaactgctctaaaaaagttgatgagacagggaga
gagag

>chr1_23882213_23882413
gaaggcaagaacatgagctttgaagctagaggaagctggggttcctaaactctgctctgctactt
tcttgctgtgtgaactgtttcttgctgtgtgccactttcttgctgggtgatcaagttattcagcc
tgagtctttttctataaaccaaggaatgttacccatcacggtggtttgtgaggattagaacaaa
aatgg

>chr1_23883213_23883413
agaacaggttaagaaccactgatcaggctgggagtttggttggcaatgccagaggttctcctcct
cccccatgcccctgttcaccccagcaagctcaccagggttaaggcactaaaggtgatgacgtgac
ctgtttgttatgaaatattgaagtaattcatgccagggaccaacacacagatgagatcatacat
aaaca

>chr1_27113613_27113813
atcctgagaaacaagaaatccaccaacggagaaggatttatcatctgtttcctttaaggagggtg
caaagttgttctaacagtttccgttacaagggtacaaactggccgctccccccaatagacggttt

tgttgagttcgcctctttttttttcttcttttttttggccccacttcctatgagagaaactgcttg
ggtga

>chr1_36838813_36839013
ggtgtcaccaggtctttgctgggtgaagggtgggtgctccctgctccgtgctgaggccgcttccc
tgtggcaagtctctgcaggtatgtctttttttcattatcatttgatgggcacccaccgcttgcca
gaaaccattcccagtgtttcccagattctctctttaactcgcaccactttctgatgtggattctc
ccctt

>chr1_36839213_36839413
ggaggcggatagagaggaggattagtaatccctcgatttcagccaggactccctgacgacaaaga
ttcatgcatgtgctgtcatcttcactgctccaaaacagagcgatgattttgttcatttttaaaac
gcaggacatgtatagctccaatttgtcaagaatacataatatcacatcatttaatgcataatatc
acatc

>chr1_36839813_36840013
cctcctcaccccactgccccgaggaagtccccactgggcttttctgtgacctcctttggaggctg
caaagaaagcctagaagctaaaggcccctggccaagggggtgcatcccagagaaattctggaaga
aaagtctcagggctgtgcacacagaggtagagagaagctttctctgcaggctctcctggggaaaa
aatac

>chr1_36840613_36840813
gagaaagcagcaagcaaccgcagaacaactggttgattctaaaagccaccattctgtccactgtg
tgacctggacacatgcttctctgctctgcagtcttctcatctggaggacagggacagcgctactc
ccaaaaagggttcccttcaggattgccagggcccagtacataggaggcactagacgcttaactta
gaaat

>chr1_36853413_36853613
actcttgctggctctccaactctaggcttacggcagacggtgctgccataacaaagtgacgcctg
tcacaacagcatgagatggtggcttgacaaggtataggcagatctgcctatcacagaaggaggga
aagagaccttcaacccccaggggcttcctcaggcttctgaaccctcgtctccattactggttggc
tctgg

>chr1_37943213_37943413
tggggaggctggcccagccctgccttctgtcctggggcaggagcagcctagctggagtcttctac
acacacatcctatcagtgtctgcttagcccggctggggaccctgaccttacaggcaaagagaac
tggaatgggggcagggatgtctggttttcctgtgccagcagcgcttcctccagtgcagtgaaaga
gacta

>chr1_45272213_45272413
gacctgccggtcgggcctcatcaatgctgggcaggcagcctcggggccgcaccggtgagggtttc
cgcccggagtctttggcattctcctcctcctggcgtcccgggggcagaggcctgctggccatgga
cctgctggctggaggaaccacactcagggtggccttcaccaggcatctgtctacccaccccagcc
tcttg

>chr1_45272813_45273013
tcatcttccacccagatctactctagggtggctggcacaaagatctcccctacacctggcccagg
ctgagggcagatgactcatgaagccccccttccaccttctgcctttggcctctgatcccacttcct
ccagaatcagttctgagatggctgccctctgctcccgaaactccctcaggcacctgagtcacccg
caggc

>chr1_45273213_45273413
gcccctgagtcatcagggagagcggccgacagggtcttgcagcttagaggcaaacttcctaagcg
caggcaacagaggggcagatgtagagcaaggctgtggtcagaaaggctttgtcctaccccaggac
tcttgagccagggcccattttctccccttgtggaatggccagatgactggccattcgtgaacaag
tcaca

>chr1_45274013_45274213
ctagggcaggctcagcttcgggggctaagaccctcgtatggcgcttaatgcccctctaagggctg
aagttttccctgggcctgcccgggagatgggtggagtctcttctcaggccagctccagtttccat
ggaaacccacaggcttcccaccccgtcggctgcctgggcagactcccagccagggcctctttcgc
cgccg

>chr1_45274813_45275013
gcccttccttctttggactttggcccctggcagaagaaaatgattctcagcccctgggatcctta
gagatggtgggtcatgattcccctcctccctccactcactcagcttggtattgaggcctcactgc
tagaagaagaggaacagatgacctgttctgggaactgaggcttcacaagaagaacaaagctccag
agatt

>chr1_150539776_150539976
agatacagcgacttccatcctgtttcatacctgaccccaactacccagctttgaagtggggcaag
gggcaaggaagcaggtagcccagcactcctccccgccatcaccaggctcctctgcagagctgttg
ttccttaaagctgacaacaagccacctcatttcaaaacacacacacacaacctcattttgacaca
aagtt

>chr1_150540176_150540376
ttcccatagcccttcacctgaggtgtgtgccccactttccttagatttgggctctcatgcaaaag
gaaataaacaaggtatcctcccctcaggaaagttcagtaacaaagcagtgaaaccgaacagctgc
tcccttgcagcaggccagctgtctcccctccccccatttccaccaagggccggagacggtgggaa
ctgct

>chr1_150540376_150540576
ccaggcaacctaaaggatggcgttgtgagctgagaaaggcaggcctgatagatctgggcatagga
gcttcaggaataaaatgcatgtgaatgcggtgtttcccagggagaattaaccgcattctcgcctt
caaatctgaatacctgagagtcataaccagcccagtagggtagactatgaaatgtatagcaaaat
tttca

>chr1_150540576_150540776
aaaccagctcctcctaccagacaagaaaattaacatttttagggcacagaactgaaagcatttt
tactgaaatctaatccaggatgaaacctaacagttaaacattcttttccttctctgcttctttgg
ctctctatcccagcacaccttgtcccaggctaacttgagtaaacatacacatgtgaccacatgag
ggcgg

>chr1_156074376_156074576
tcgtccatcctccactcctggatccattcaccaaggggcctgagtgaggtcccacccccctaagcc
acacaggtcctctgcttctcccccgctacatctcactgggaccccagcctggcaacggctagtgc
gtcgtcggtgctcagttagccccaacatctctttcctttcctccaacaggaaacacaccttccac
ctcta

>chr1_156093776_156093976
acctggctttgtcttagggaaggacagctgggagcctagtggctctggtaggggatctgagaggc
ctcagaccctaggcatatttggctgtttggcaggtgtcacgcccaagggaagcgtgtggaagcag
agccatgcctgctgtgggtgcacatgcccgcgtgagggagtcggggtgtttcatcctggggcacc
tgtgg

>chr1_156096576_156096776
ctcaagaggccactgggatgcagccactcctgtgcttggggaacctggaggatgcaagggaaagg
actggcactctgctggcacagcacccggcctggggcaggacacgggcgaagccagggtctcccct
gtgagcactagaggatttcccgacccctgcccgggtattgtgtgcctgagcatgagtcacctgag
gggcc

>chr1_156717976_156718176
cttctcacttgatggaatttctaacccttcacccaaaagaggagaaatcccttctacagtgacac
ctctatccctcaagctacaaagaaaatgtcctttcttacatttggaagtggagggcagttcttcc
tagatgtagaggttatgaggtctcagggcagccctgtaccccaatacagacatctgtcttccagg
tggtg

>chr1_161089376_161089576
ttgtcacgtgctgctgaacagcaaaggtgagactttataaccctttagcctgcctcttcccatgc
tcttctacctagattctccagaattgtttctcaactcttatttccttgacccaaggatttagggg
tggtcctacttcagttcctagcctataaactatctcctccttgggaggagtaagcaaggcttcta
gaaca

>chr1_234747377_234747577
gattggtaggtggagagaaaaaaaaaatcatgctgacgtttctctctagtcgttgctattgaaaa
taccaggttgcttcaaaggattaaccacaaggaagaggaaaaacacccttagagaaacagtggag

ggagctgcttaaaacacatgaaaaaacctgttcgaaaatggcacagaaaacgtaatgtcagacct
aaatg

>chr9_131903379_131903579
ttttgggaacctgggccaggggagggaaatgagggggataaaatgacaggttttttttgacttaa
atcttaaaagttttgtcacagttgcccttactagagaagtaagaggcctgccaggagtgggtcac
caggtggcagcccatggctcaagagtgggggctcctgagggctcttgtggtgaactgatgcccct
ttttg

>chr9_136215979_136216179
ttttagtgggtgtaaagtggtcgcagtccttaatttgtgtctcttagagacgggggcaatgatac
atgcttcttgctttcattgggagttgctgagcgagcattcagctcaatatggtagtggccttgaa
ttcagcttagccatctggaaacaagtacagtagcagtgtcgcagcgaggtactaggactgcaatt
ctgct

>chr13_99137399_99137599
agacagagccgactcatcaagacaggggaactgcagttgagaaagagtaattcacgcagagccag
ctgtgcaggagaccagagttttattattactgaaatcagtctctccgagcattcggggagaattt
ttaaggataagctggtggggtaggggaagccagtgagccagaagtgctgattggtaagagatgaa
atcac

>chr12_6446539_6446739
aggaggctttaaagtgtgacttcctgtgctggggctgcagagtggggaggcgacgctgagatcgg
ccttgtggtctgaccccgatccagccaccttgcagggacccaattagtcttcctttccccctcat
tcatgtgtctgttcatttaatgtcaaggtgttgggggaggtcgtggcagcttctcctccaagggc
ccagt

>chr12_6999739_6999939
tgtatgctagcatggaagggaattgtttcttcggttgagtgcctaaagtgcttgatggcctttca
gatggatcatgtcatacagaaaggaagtatcttaaaacactacaatctagtttaagacataaaac
agtagaatcgctgtcagcgtcacaagatacaaacgaagggctcagaactgggaccaagagaacat
agtct

>chr12_45626733_45626933
gagagggagcaggtacatctgttccctgcactccatcttttcctcttgaggaagggagaggcagg
gagctagaagccaacgggcctctggattctcagccagaaatggctctccagacttctactctact
ggctgtgggatgtgactcattcctgtctggaggaagagtttaacctgacttcggctgggagtcac
aacca

>chr12_45627933_45628133
aaatgtgttcttaagttggaagagaatgtgtgaggaaaattcactcctatctcatttacagaaa
ttcagattttaattgtcctgctgtaattgtgaaactgaactgcacattcattctgaggattattg
cactgatgagtttactgatgagtgtgacactggttcggagggaactgtttgcagactctccctat
gtata

>chr12_45628333_45628533
atttgttcaggccccttttccaagttcccagggaagttactgacactgatgttggtttcttttaa
gaatgactcatgatttttcgagcctggtttccactcttagtcatcttccctgcctgagttaaaac
aggctttaggctctcactgaattccagccttgacagaacccccccacccccacccctgggcttttg
attcc

>chr12_45628733_45628933
tctgtgttgctcttgtcagtgtttactatcagtccagcagtaacctttcagttctaataatttgg
cttgtccttttcctcctcagttgttcaaactcctatctcagaaacatgcctaggacctcagtctc
tttagttctttggattctgtgttgctcttgtcagtgtttcctaatcaccctcttgccctggaaat
aaagt

>chr12_45628933_45629133
cctctccaatgtggtgctacttctcctccttcctgtagcagactctggttgtggcctgtctgtgc
ctacagatttagtgacaggagctgtacttttctgttcccttctttagctgtcatactggtggtat
cttaaatcttagccctaaattgtgatgaaactgttgtattttcacaagtcttccttagtcatatt
gtgct

>chr12_45629333_45629533
acctagttgagtggcatgacatctgagcagaactagcaaggaggcttggtggtatgccgcctttt
aactccagcacctgtggctcttgagcattctcctcattcttcagtatggaaaatggatattttct
cagtcttgtgcaaaaacagcttcaagaaaagtcccctagtcaaaaattccagtatttggaagtgt
accag

>chr12_56520133_56520333
tggcacctgggcagcagaattgtcagtgggcggggacccaccctgtttacttgtgttggtaactg
cattgcagctgctgatcctgtggctcctgggccccagctgggacaagaacttcctgaggatgaag
ttttatcccctatataatttctgatgatctggatgaggtgaggaagggagaagtttggaaaagaa
cctcc

>chr12_76337933_76338133
tgaatggggggacctgctaagagcagagaaaatccccccagggatcattttaggccactgcttata
aaagaacaaaaataaaatgggaggaactccagaaccaaagtcctagagtccctatttgcttgtga
atgaaagagactagtatgtaaataagagctttgcataagccagacattctttcaagtccagttta
tctgc

>chr12_76338133_76338333
agtatgagtcacttcctttttaaaagcaagaactaaaagagaaagaggcacatagtcattaccca
gcacaaaccttcttaacagaccttttcttcctattcaaattgcaagaaggtaaaagactcttgct
ctaacaaaacagcaacatcattttgcatatagtgtatacactgtttggccagttaataccctcct
tcagc

>chr12_109083471_109083671
ctcaaagaacagttaaaagatttccacattgttcaaacccctacctaatattcatctaagttttc
attttaggaaaaaatgagtatcaaaatcactttatgtgcccaaatttcaatcataacttattttt
ggctttgggtgatacatactgagaattcttcaactcagcaaggagaatgttaagtcacacatgtg
ctcag

>chr12_109086271_109086471
gatcacacagaaaagcactgtcaggagactgccattccctctcaagtagcccatgaaagaaggca
cttatcagcacggcgcctggcacttaaaggaagccctcaacaaatgtgaactgctgcgatttcat
cactgtcactgtgtcctcacaaggcatggtatagaccaagtactttccaaacctaagacaattct
ctgag

>chr12_120637017_120637217
gaagtttatgggagacaatcacctttcagcaccattctccaggaagagggagacgggcactgggg
agaaaggacaaaactgccaggggaactgacttctccagagctgggttgttttccaggtgccctcg
gatggccttgcgcatcatggtgttcttgcccatcagcaccacagccttcccgcgaagggacatgc
ggatc

>chr12_125402647_125402847
ttccccatttctccctcccaccagcccctggcatccaccattctactttctgtctctatgaattt
gacaactcatgtgacctcatataataagctttgtttaagggtaaaataaaatttaatagttgaaa
aagcactttttttcaaagagtatttctaggaagctccaattcctgacaccaacctttaacaataac
aactc

>chr11_9385424_9385624
aaaggaaagaaagagaaaatacaaacaaaagctgagatttgctgagttcctaccatgtgctacgc
aatgccaacaacaaggtggcatggaataatcctcatgtctgttaagtgggtgggggtaggagtgg
gggtaggagtaggggagtgtcacacatctgcaaacggtaacgttgaacttccaaagtaggtccat
cgggc

>chr11_9587424_9587624
tattctgacctgtattaagcaaatgtgcagctggtaggataagatgcttgaacgtagtagacact
caatagaggactgccaagcagagttgatttctgtagttagaaactagttcatccttaccagtaat
tttttttttcttgtatacactcccaatcggcttatcggcttatcatgtcacacacaactggttac
tctct

>chr11_9587624_9587824
acccaactagatactggatgaggtcatctctcaatcctgtctgagctgaacaaggccttttatgg
aactttaaagttgactcaccactgagtcatcaaaacacagcaatacctaaacacgctcctgttgc

taacaaaagaccagcatgggatgtctgtcttacctcctttccctcccctaatgagacacggcccc
ttgac

>chr11_9587824_9588024
tcactggagaagagtcatcacgtcttttgtgctgcagtaatctgaacgaagccagtttaaataat
cacaatgtggccagcaaacaactggcagtccctgtggtcattcaggtcatttcctgtgtatgtca
ctaacccaaaaggagaggcaattaatcaatcacctgaatatttaataagtgcctgcattctgttg
ggcac

>chr11_9588024_9588224
tcacagttccctctgtttatgacatttctgtgaagtgaacttttcagttattgaaactcattaaa
aatatagagaacagtgttgcgggaagtcagggaccccaaacggagggaccacattagaacagcaa
cctttgtgcctctgtaaaggaaataaaaaaaggaatattggagaaaatgggactattcagttcca
ttcca

>chr11_47290224_47290424
agatctgggatgtgcacgaatgactgttctgtccccatatttctgttttcttggccggatggct
gaggcctggtggctgcctcctagaagtggaacagactgagaagggcaaacattcctgggagctgg
gcaaggagatcctcccgtggcattaaaagagagtcaaagggttgcgagttttgtggctactgagc
agtgg

>chr11_57093424_57093624
aacacaccaaggctacaaagaaagcatgaggacatggtttccaaagtcagttccctttattaaat
cattttcacagaccagaaatacaaaatgaaagtagaaataggccccaggtaggagctacaggcct
ggcctcacatgacccctgctccagcaacttgaacaggacaagcagcagctacatccttaaggtcg
ggaaa

>chr11_61582224_61582424
ttggtgcgtttttacagagcactgattggtgcattttacaaacctctagatacagaaaagttctc
taggtccccattcgacccatcaactccagctggcttcacctctcagggctatgagacttccgtcc
cttcttgcgggtctttcaataagtccctttcctctcttagctagggttctccaaaaaccacgaag
aggaa

>chr11_62320024_62320224
aagaaatgactgttaacccttcttagcaccatctgggatctcccagaccccgcctccaccccata
cacacaccagcccagggattagcatccctgccaaacaggcccttctccattcacttccgtcactt
cctaatctgtcattgtccaccccacccccccaaaagcaaatctatctggtgattctggaatccaaa
catta

>chr11_62433824_62434024
gtggcgacatagaccaagtgacaccctccaaccgtgcccacaggctcactggctgatagttgcct
ggcggaccgctgtctctgggatcggctgcatgcccagcctcgtttgggcactgtccccaccttcg
actggttctttggatacgacgaagtccaggggctcctactgccattgctgcaggaggcacaggct
gccag

>chr11_62434024_62434224
tcctctgcgagtgctggatgtgggctgtgggacttccagcctatgtacaggcctctacaccaaat
ctccacacccagtggatgtgctgggggtggacttttctcctgtggctgtggcccacatgaatagc
ctcctggagggtggcccaggccaaacacctctatgccctggacaccctgcctcaagcctccactt
catgc

>chr11_62650424_62650624
actcgtggttctccactcaggttgacactgtggccaccaaggtgaaggtgagtgttggagctgat
ggctggtggaagtcagatgctggggctgggacagtcctttcacagcaagccctgtagacccagcc
tgactccagctgagggtccctttgctccttaggaccaaggaggaatgcctcctcctatagccaaa
ggata

>chr11_65187224_65187424
caatgtggtaaaaccctgtctctactaaaaatacaaaaaaaaaaaaaaaaaaattagccaatgtgg
tggtgtaagcctatagtcccagctactcgggaggctgaggcaggaaaatcacttgaacccaggag
ggagggtgcagtgagcagagatcacaccactgcactccagcctgggcaacagagtgagactccat
caaaa

>chr11_65187624_65187824
acttgttttgatctagagaaatctgctgacaccggccttcctgctctcaccctccagctgggcag
aggtatttttagacttatttgtagaggaagagagcagaacccaggcccaatgtgtaccctcctta
ctgtgggcacagctttggccccttaaggcaggacagagatatccatgtggttgtctaccctcagc
tcatc

>chr11_65256424_65256624
atggactgactgttcaggttcaaggggtcttgacctggctcctgaagttgggggctcccactgc
caggagagtggccacgactgtggggagcagtggagggaggtcagtgcaagtagggaaagtaggga
tcttgctgccacaaacagtgggggtgggggtggggggggcaggaacagaggaggctggactgcat
gaggt

>chr11_65259024_65259224
tgtgggaatgggaacttgctacctagaggtttattccactcaaacactgggtggctctgatccac
tattaccagttcaggagaaggccctgtcattctgcgggcttctgttttaatccggcattgttgag
aaacacaccccgacaaagtgcctgaaaccttgtggccgctaccccccttgtcccaggagatgtgtg
tctgt

>chr11_67055424_67055624
gagggttgagaaggcactgtcatggaattaagtgccagtggggagctgtgttcaagatggcagaa
atcaggagaggctggaaccaagaaagtcctgtcggggttgagtttggagagggctcagggaaggg
accgcctctgagaaaaggggtggccagggagggctgcagtagtggtgagcaggtgagctctgggc
attca

>chr11_67121624_67121824
gtcccaagtcgtgtccggcccgccccaccagtcaggaagattccatactgagtaggaaggcagag
caagggtctggtccccccaccacaatgtgtttggatccctagttcttcactggcaaaatctgccc
tgtctctcacaataatgtgagtctgagcagaaaatattaataataaacctaacactcattgcacg
tacca

>chr11_71814552_71814752
gtctctaaactggcagtagtggccatcgtgaattgtgttatgcgttctcgtggccactaggccac
ttttctgggccattgagcgaactcgtaccaaatgccctcgtatgcccttgtgatatactcttcta
aatgccatgcgaggggcgactgggatccaggcctgatgctccagatcgtacggtctgggtgcaca
cacac

>chr11_118783390_118783590
ctgctcctagctgcccccacgttgggagcctctgcttggctcaccagctggcccagcagtgtcac
tgcctagctcaaaaccaccccaaagaggcagtgaggaaatgttttggagggttcatgctcctgag
ctgccagggccccctgtgatgacgtgaaagcccaggaccctgctggggacaacagggcgaacgag
atcct

>chr10_105668010_105668210
tctgggcactgtcaccaacccccggacttcccagtttgtgacctctcccagcagaccctgtgcct
tggtgcccagaggtctggctgctggaatgcccctgaatggaggcacccttgcagtgctgcacaat
gtcctcagcagctacacagtaccgcctgggccgctggccttgtttcagctgcgggatgagtcagt
gcgag

>chr17_7486476_7486676
cctcgccaggcctcggctccccggagactcttgggggtctgggccccaagggtgattcaggtgct
gcccttccccgacctgggctcgcctcccccacctcctcctctgcttcactgtcccgggcccagca
gttgccggcgcactcatgctccgaggcctgagccaaccggaggcgagacaagcacagggccctgc
gcgca

>chr17_17740675_17740875
gaccaggatccaagttataggggtcccaggcctgctgtgttgtgagcaggcctgatgtaactgct
gggccagttacatcactctgcctcaattttcctcatttgtaacatgggcctaatgatgctttctc
acgggcaggctgggaggctagtgacaatgtggaggggagcatgacctgtgagatcagcctgcccc
ctctc

>chr17_38267874_38268074
agaggtggggagtgagttctcagtgccctgagtcccttttagagagaaaggttgaggctggactc
attggcttgtgaaggacttgcagcttcagagcacatgacttctgggtcacccttctgctcagggt

catccctgatgacccaaggtgtcctctggaggcttgttaactcaatctccctccttacgcagggg
ctgtg

>chr17_38268274_38268474
cctcaacattctagtttgatcgcttttgggctgtttcccttgaaaacacgtgccccagcaaggat
tgaaaaactacctattggtactatgctcactacctgggtgacaggttcagtcataccccaaactt
caccattacacaatatacccttgtaacaaacctgcatatgtaccccctgaatctaaaataaaagt
tgaaa

>chr17_38269874_38270074
ctgccagaaagagctgagccaggccaggctgcggagctggaagcctctgtggctccacgctggct
tcccatcccccatcctcccggagaaggcaaaggtggcctctccaactgtggtgtcctttcagtgt
cacaggactggggtaataccagtcacacaactccagtctctatttacctttccgagagcttcagg
aaacc

>chr17_42173474_42173674
gcagggagccccaagcccagcccaagccccacaaagtctccagccaggaaggggaaggcaggata
ccactgcctgggaaggcggaagtgagagaggcaggccaacccattcctgtttctcttctacttct
ttctccaaagaaagccctcactctcctcgctacagcccagggaggtcacgaggggctgggaagac
tcctg

>chr17_42173674_42173874
tggcaaagtggcccactccagcccaggcctgagaaaaaaggaccccgaaatccttctggctacca
gtatcttctgccttcatgagctggcctctctccagaccacccccccagcattaagaggcgagagag
ggaaaaaaaaaacaaaaaacaagttttggttttttttttgttttttttttttttggagatggagtt
ttgct

>chr17_57914418_57914618
ccaggcatggtgacaagcgcctgtaatcccagctacttgggaggctgaggcaggagaatcacttg
aaccctggaggtagaggttgcagtgagccgagatcaggccattgcactccagcctgggggacaga
gtgagactctgtctcaaaaaaaaaaaaagtgaagtgaagtgaatttgagtcagtgtacttaatagg
acaag

>chr17_57919418_57919618
cggtgtgatacccttctttttcagctgttcgtgccttcctttcttgtatccaccaaagtggagac
aaatacatgatctcaaagatacacagtacctacttaattccagctgatgggagaccaaagaattt
gcaagtggatggtttggtatcactgtaaataaaaagagggcctgggaattcttgcgattccatct
ctact

>chr16_14595499_14595699
aatgtgccaccatgttattaaagccatcatggtgagagttcctgggcaggcagcccgctggaatg
ctgagtcatctgttcccccgctcacagctggccctcctccccagcacatgctctgcccacaagca
cactctttggggaatgcattaggaagcagctacctgaatgtatcctgaaaatcacagacaaggca
ctaaa

>chr16_14595699_14595899
cacacacccattggagcctgttctgtcacaactcaagccagagtgcaggcataaatcactgccgt
ttctggaaagagcacaggcacaccttgcactcagctctctagaggccttagctaaatgctgttta
gattatttttaaaaaatgtggttttgactggttcaggatttaaaagagttaacaaacatcatatg
agcag

>chr16_87422099_87422299
ttccaatcagaaagaggagaggagggagaggccgaggaggcctgaggcctggagcaaattctccg
cgtgacttgccgtccatattgagtgtggcaaaaaataaccccgatgctttctgtaggtcaggtta
atgttttgtgattcatttgacaggttcttacacacagctagacagcatgatttacagccacttac
atcac

>chr16_87812499_87812699
gtctgtggtccaggtgcgctgggggcagcgcaggccgggttcaggagagctcggctcagcagcag
ccgatggagctttcctgggggctcctacctggccgccagctccagaagccaagccaggggctctt
cgagacatggtcccaccagaggagagaccgagcctcggagaaatgaacgtcctaggccatgggac
tcgga

>chr16_87812899_87813099
accgcagtatgcgaaggttgcatcaccctcgcggggtctaagaggaaggatggggaccagcccgc
aggcgggcctgagcctcctggcaaagccctttgtcaacgtcaacagaagcatctggcgtcctttc
tggtcgcggacgtcagagctggctgctccggcactcagccgggtggttggtccccaggcagccgc
tgggg

>chr15_93374396_93374596
aagcttttggactgagatatgcgtaagcaaggcaaaaagatcccatagtccaggaatatcaacca
gtccagtttcccaaggaaactaaaagtaactgaaaatgagaagggtgccatataggaaaattgaa
ggtggggcataattattaatacactgctttggaaatgctggtatggaagattcatatatggactt
caaag

>chr14_74208447_74208647
cactttcacccagcacccagaaggggagtaatttcctcctccatttccttcctcagctcctgggc
cttgaagagagtaagagaccctcctcgtgtgcagcctttgtcttttcatatatgaagctggaggg
agggagaggcacagagactaggagggcatccaagtcaccctcacccccagcaagaggggagtggg
gggat

>chr19_4374800_4375000
caccctgcctggcccagctccacatgggtgtgggggaggctgcgggctgcgcctggtggccgtat
ggggcctaggctggccagtgctgcccaatggtgggggctccctcaccgcccgtctggcagaagcc
atgggccacacgctgtcccagcacatggtggcagggcggtgacctgggctcggctctaatgaccg
gcagc

>chr19_4375600_4375800
ggctcctctcccaggaacaaaccacacccagagctccagcccgagcgaacacatctacatgaaaa
tcagcctcatgcatcatgcattgtaatttctcatcttggggaccaggcaggagggctgtgtgaca
tacaactggtaaaatcagtttccgatgaaaacttataaaaggaaaaagccaacccacaaacaaat
gaggc

>chr19_4375800_4376000
gataaatcccctgaggctccgcctggcagggaaacaccagctctgcagcactgagcacaggctgg
tgttcccactgggggcagcatggaggatctgcgtccagggggcagagtgtgatgacatcacccac
atgcacaggaacgataaggctttcttaaaaaacgatcttttctcaacagctcctcaaaaggagct
gaggc

>chr19_8067000_8067200
ctcagccttcttgtccctaaagaacccacgtggttctttgaacacacgatgttctctgaacactg
cgatgttcccacctccgggcttggctgtatcctaaaaagccccaccctattgtctttgcctggtt
agctcctccccaccgctactgcctcctctcctccaggaagcattctgggctctcctgtgcccacc
caggt

>chr19_13276000_13276200
ctaccaagaggacacggcagtctaggagctccctaaggggcaggagtgggctgggagccttggaa
acggtttgtttctcctgggagccctgagaagtcatgttgggggaattaactgctgtgttaattga
ggcttcaagccaagtcccctctgtttacacagagagtctgagaaatgccctcggaagattccgag
agcct

>chr19_13957200_13957400
tctttgggatggctttggggccaggcgattacacttcctaagcctcagttttgtcggctgaaaaa
tggggctgaaaatgcgcaatttgcagttgttaccaggctttgaggtgagacgtgtgatgtgctga
tcgcaggtagaaagagggtaagtgatacaccaggtacacctggatcaagggaccctgacaggccc
agccg

>chr19_13958800_13959000
gtaggggtgccacagagcctaggctgcgctgggggactggggaagcgcatcccatgagaaaggcc
cttagctgggtcaccaggaacccagggaaccatagtgtccaaccacccccaccaatcccgtgggg
gaggggggttcaagttctgagagttgagggactgcagccaacatgcctgggttccaatcgagggtc
accca

>chr19_18475400_18475600
gtgacagagcgagactctgtctcaaaacaattggacttaagatggcatgtctttgttttagggcc
aaaaatgaggccccccttgcttaaggcatggggtccgtggccaggcaacggggtgggactgctgcc

ccagatgtggccatgggggtcgtggtcctttcaacaccaagctatgagctgtcgccatggagacc
agggc

>chr19_18475800_18476000
atgtcccggcatgtctgagcaggaggaattccgggcggccaaagggttttatgtagattgctttt
ggacactcgcccaggagtcaggagttgatttttatctcacttcctggtaaccttgaacttaccag
gcagggatgagatgacgtaaacttcctcagatgatgtaagttcagccacatcttgctgcccatta
gggag

>chr19_18476000_18476200
agaggacgacccccacagtgaaagtaattgagtgaaatgcacgttctggaaatcagtttctattt
tggcctggaggaatgtggctatactgcttccctgagtgtgacacagttggaatctaatcttgttg
gtttcccaacatctaggtttttgtttgtttgtttgtttttatgatagggtcttgttctgttgacc
aggct

>chr19_18484800_18485000
tgggtgaggctgtcacctctcccttccctgtatggtgctgataagggcgtggggaggcagagaca
ggcagagatagacatgatgagagacaggcagaaatggagggagagccagggagagagagacacaa
agagaaaaagtggaaattctccagatattttgtttagggtgaggacacggagacacagagaggga
cttag

>chr19_39175560_39175760
cttaacccacccaaatggctttttgtttgtttgtttgtttgtttgagacggaatctcgctcttta
gccagggtggaaggcagtggcgtgatctcagctcactgcaacctctgcctcctgggttcaagcga
ttctcctgcctcagactcccaagtagctgtgattacaggcacatgccaccaagcccagctaattt
ttgta

>chr19_39175760_39175960
tttttagtagagatagggtttcaccatgttgaccgggatggtctcgaactcctgacctcgtgatc
cacccaccttggcctcccaaagtgctaggattacaggtgtgagccaccgtgcttggccaacccac
ccaaattgtgtaccctacaccatcctagtgcttgggagccaacacccactcacaggaggctaagg
gcaag

>chr19_39176560_39176760
tcgcatattcacccttccaccagcagggcccagtttacggatcatttgaatactagcaaaaaaac
aagccatctgctggagagattgaacagagggagaaacaggagtgtttgccctgcttccagacacc
cctttcctgtggcagttcctgcacctggcaagatctactggggaattcccggggtcccactagct
cagga

>chr19_39176960_39177160
acttggctatctttttgaaagatcaaagtttaaaccagatgatctgtagtcccccacccccaccc
tgaaacctgagcttagctgtaagcattgaaagtaaatggggtgtttgtagctcaccctccctgtt
ctccaggtgaagggccccgtgtgcctgatcatttcatagcaaaatgctagatggggccagaggag
gcccc

>chr19_46221360_46221560
ggtaccccacctgtccagtcgcatccctccccactcctccgccccgcaggcaataccccagtgca
ccctggtgggcacctgatctggcatacatagaatactcaggacccatctgatccccggtaggagt
gatcgtctccacggtatggatgaagaaactgacgcagggcaggtctcctgaccacaaggtcacac
agagc

>chr19_46221560_46221760
tggagtgtgagctggagcagggatatgaagccagggaatctgatttcctagtcactgtgccctac
tgtttcactatatattccctggggtttcagctcgaccacccctcctctaagaagctgacaccccc
aatccccctaggccaggtcaggcttcacccagggcgtccatgcggtgggctcattgctgtctatt
cctca

>chr19_47611360_47611560
ctgctgaataaataccaattgccagaaatctcaggagtcatcctttttttcttagctgtgctgcca
aggcacaggttaagcgcctggctcaattcagctactttgcttgctattcactctgtcactcaact
ctcggtgtgaatcggacccagttgctctggcactagaattactgttcgtggggaccaaaattaag
ttctc

>chr19_47612360_47612560
tgcaacatccattaatttgatttttaactaatactggtacatcagtctgggggaaaaaaggcag
tactgtgacatttcaaaaacagattaccaaaatacagtttttgacttaattactggggcttctag
gtcttaatggatgagatttctttgagaatggaaaacaataaaccgttgtgttgacatgcctgtga
gcttt

>chr19_47612960_47613160
tgcagtttcctcatctacatgtgcgtgacctttttttccttgtaagaaggaaaaaaacaaaacggt
tcttttggagagggccagggttctctaagtatgtcagggatcaaaagtgggaaactgggaaaatg
caacttcaacgtgttttgtcttttaacggaacttatgcttgtaaaacacatcagaagtacagtgt
ttggt

>chr19_49120588_49120788
agaggcggccggttggtgcgactcataaacaacctcttcaacacaacctggttgaatgtggagtt
ggttcttctggccagaaacctgtataactggagggacgggaagacagtgagaagctgggacagcc
tgctcccccgcagccttccagtgaaggggcaaacacatgatgatctggactaaaataatctttat
ccctt

>chr18_3603000_3603200
aacacaagaggacagagctaaggtgtaggaatgcggaagaacttgcagaaactagagctagtctt
cggattgaaatgaattaccaataaagggttgggaaatggatcaaggagagggaagcgttttgatg
gtccctgctccgaggttcagcccatttgcagttgctgataaccttgtcaggtcaccagataaagg
caagt

>chr18_3606000_3606200
gtcgggacattaagaaaaaaaaaggtctcgctgtggtttgctattagatggagagatattcagat
ttcatttttaaaatgaaagtgaaagattaaagaattttggcatcctgaccctttgtagaggccat
tcctcagaggcacagtgtgcagacaaaactatcctagaggtgccaaccagaacaagctctaatgt
ctgtc

>chr22_36726054_36726254
aatgtgaacatacatgtatgtacatagccatgcacacacacggatgtatgcacaggcacacacac
ttcaaattccgaaaccccagagccaaattcagtgtctcctctccatccacccgagcgtccctagc
tctcccacttctccagccttccccgtccaattcctgaagccaatttaccactgtcaacacagcct
aacca

>chr22_36726854_36727054
aacgggaggaggccgccagggacaagcctcttctgggacacgagccccctgccagatgaaagtct
agccgtggtaggcgcctgaacccactgccccagcccctctcttggtgagggtggggtagagatgg
ggttatgagtcactctgcagtagttttcagacttttcaaaaagcagcggcagtccttcctcagat
aaaat

>chr22_36767254_36767454
cagaatgtgactggtatttgaggacagggccttggaagaggtaattaagttaaaatgaggtcctt
cgggtaggccctaatcccatggctggtgtccttataagacgaggaagagacaccaggcgtgtgcg
tgcacagagcgaagaccatgtgaggacacagttggcagacggccatctatgagccaaggagagag
ggctc

>chr22_38576654_38576854
ttccactcctttcaattctgcctatgacccaacagtaaaatcatgtatttgtacagggccatact
ggttataaagctttaccttctacattttctcattcaaattttgaacctcatggcagacttacgag
ggcaggaggtacagttcccctgtcacagatgaggaaactgagacttggacagaaaacaggttcca
aactc

>chr20_10492000_10492200
ccatgccatggggctcacactgccctagggatactgtcagggatgttgaactccatgagactgtc
tgttcacctgactcccaggcagacagtaaggcgatcaggcttttccttaagttgagctgaaagct
gaaatctgcctccttgaaacctacctccagctgttttaattttgccagcagagtatgtccagctc
ttcac

>chr20_45946793_45946993
tcctccccgactaccgccccccactccaaaaacacagcttgctttctggaaggagcatgttaaat
caattccacaagttcaaagctatggaaaacaccggcctgtgtagactggagaccagaatcagccc

aggacaggatttctgcatttcaatcagactgaaaatgccaggtttcccccagtaactgtgaaggc
agggt

>chr20_45977993_45978193
aaaggtagttgggtttaaatgagaatattaacacatttagccatctgattttgaaccccgttaac
aatttcatttacaccgcatctgtaaagaaagcctgtgacacaaatcaccaaagaaacaactgaca
tgttgtcacaaatggttttcatcaggagaaagcagcctgctaaatttaaacacatcgcttatatg
caata

>chr20_45978593_45978793
aaaaaacccacttgaaggatttctgcagcgatgaaaacattcaaaggtgtattgatcatggagga
aggctgcattcaagggaaccattatttggcttgtaacagcctagaggagctgaatacttcaggca
ggaatgctgctgagagacgaggaaaatgcaaactagttatgtggtggctatttgcttctgagttt
taaga

>chr20_45988193_45988393
ctggggcaggcctacgaagggctggtcctgggcttcatggaatgtggctgataagcaggaaataa
aaccaaatgttgcaggtctcaatctagaaatccattcagcagctcctcttaaagccccactgcag
tgagtatcccagcagttacttctaaattagtgctttacatagactgattcctgaagtaggtggca
ttgtt

>chr20_45988793_45988993
attcaaatgcccctcattacagattgaggcccagaggaagcgaccctctccctcccaacctccag
caagatgcaagatgtcaaggggggaacactttgccatctctaaaattaggcttttatattccctga
aaccagtggttcaaccatgggggggatttgcccttcagggggcatttggcaatgcttggagacgtg
tgggt

>chr20_52239793_52239993
caatgggtggttattactaacaccagtaggaaaaaatatcacaggagcttcatgacaaatcctta
ggcagggtttaggtctcactaatttagaatatgcctggtaagcactggttaaaaattgatgatcc
cgggaagtctttctcaaacgatttttgtaaaatacctaggagaccttgaactgtctgttacggag
gtttc

>chr21_35319930_35320130
gatacatagcccaggggtattcccattcctccacgacacaaatgacaacttccttgagctctgat
ggcttgccaggtggctgatggcgtggagaaccttgctcatgtttacctgttcttgcatccattac
tcacctctgcccacgtgcccccacacttgcacacaaagccacagagtggcttaaaaaaaaaaaat
gaaaa

>chr7_1083274_1083474
ggcgtgggccgtggtgagcctctccctgccctcccgctggaatagaaagctgatcccgacgtccc
actccgacccacggtcaggaagccgtcctgtccaggaaggtggctgaggtacatccctgaacaca
gtaggccacagcatcatgcagcggagggccagtggccgctattcttcctgctggtgttccactcg
gcccg

>chr7_44678475_44678675
tgggtggtcaatgactcagcctagtcttgatttatcccccaggatggggacagacagcaggggag
agatctcaggttgagatgagtgtacagtaagggctgggagtatcagaaggggaaacgctctccca
gatagtgacaaagctaagcgaccatgtgaagggagaggacaaacatcccatggaaagaagggtag
ctgcc

>chr7_44678675_44678875
aggcctgcgggaaggaaggagcatgatgctggggcatgggagcaagtggacagggagggctctgc
aggtcccctggaacctaaagccaggggttatggaaggcagatccagattcaggtgttcagaggat
gaggcaggggtgggtagtttaaggagctggagaagaggtatctccttggggagtggaagatgtta
gagag

>chr7_100143464_100143664
ggaggaagggggctctcgtttctaaattgcttcttttggccagcagtggcctctctgtgattcag
aaagaaaaactctgactcataggccatgtaactaggccccccttcctaggctgcagtgttgccttg
tagagaattctccctgcccccagactgtggctctggttgtacaaaatccagtctacagacatcaa
ggtca

>chr7_104585964_104586164
acttgctcagagtcctactgccagtgggtgtgaaagccagaaccaccaccctcattgccttctgt
atacatagctttaacagtttgcaaacactcttatatgctttaatttcatctgatcctggactgag
agcaaatatttgtatttcaaccaaaaggccaaatgctcagtttcctcatctgagaggataataag
agtac

>chr6_7146801_7147001
ttaaaagccagtttgggtctgatataagttctgcttcgtgtttgtgtccattcactccgtaaaca
taccatatatagtcatgtttgagctcactataacacacattgcaagggagccgctcatagtgcac
acatcctttggctgttagaagcagactcacataggctatttctggtgtgtcgtggctttcttctt
tcttt

>chr6_7147001_7147201
tttttttttggtggggaggaggtggtgggggagggagaggaaaaggagctctttttccttccat
cttcacactggttggaagatgtttctgattctagtttgtctggcactctgaaagctaaggaactg
tattgtcatggcagaagaaacaattctctgtgtgcatctgttttagaatgaaaccgtacttttct
tagta

>chr6_26026021_26026221
atctcatctggtctcagcatttatttatctgctgatgactcacacatttttatctccagctcaga
cctctcacccgaactcacttattcaactgcctattcactatatcctcctgtcaggagaagcttgt
tagcaaagtaaccagaaaacaaagttgatttttttttgctgaagtagccatcaatatatttgttac
taaat

>chr6_29932421_29932621
ggacacaaaacgtccccaaagcattcaagatccaacaacaagaagaggagactccctctgcattt
ctgcagaggctcagggatcaagtgaaaaaatattcaggattaaatccagaggacccagtagggca
aggccttttaaaggttaattttgtaactaaaagctgatgtaatattactaagaaactgcaaaaga
ttaac

>chr6_35438022_35438222
aggtttaaataaggcaggaaagttcccttccctgctcacacacaacgaaaacatggtggccaaag
tggatgaggtgaagtccacaatcaagttccaaatgaagaaggtgagtgggtctggcgggttgcta
tgggtgaaggtgttggcagggtctaaatcttatccaagtctctaaatatgccagtaagagcaccc
accag

>chr6_43141422_43141622
tccagagatcctgataggacaccctgccctcagagtcattagagacgaaggtcattatgggaatg
ggggaatgacatcactgtataattctttttccaacttctcaaggaaggtagagataaaaagtttg
ctgacctgcccatctccctcctcacccctcaggcctatgagctgtccacgctgacagggacacag
gtgct

>chr5_172191995_172192195
acacatgaatgaatgaatgaatgaacgaatgagtgaatgaatgcgtgggcctcctgcctagcttt
aaaagccacattcaaggaaaaggtggctatgagcttatcttccttggcagagcagttttgtccac
tgtggcgctagcagagagcacaccctttgttctccacggggtggaagacacctcctcggtgggca
gaggg

>chr5_179246794_179246994
gggaaggatcattcacacctgtggaccagcccctgctgtgcgcacacccacacatcaccttcgca
cctgactggccccatccagccactcttgcctccctctgggtttcctcccctgggaggtttctcca
gctcctgcaagccctgggctgaaatggcatgagttggacccagcaggttctgacctcctactcac
aggac

>chr5_180668994_180669194
cctcagcaatgctgagtaacttatttaaagtgagggaggccaaacatcttctgtaggtgtgctct
gctttccagttcccaaatggactggatctcccctgggaaaccagccaattgcatccacctcactt
ctgcccagattcttcccaaggccccagagctaagtgagcagctacttgcgttgtgagatcccaga
ggcgc

>chr4_38673805_38674005
ttcagagggaaagggagatgattgtggaactcctactataagctaagtgctcttatgttagctcc
ttaggcttgacataaaaacttcattcagggaacaagcccgatctcctgtggggagtggggaggga

gccctctcaggaaggctgaggcctgcctgtctgtccttcccagtactggtggcggtgtgggacgc
accac

>chr3_39193396_39193596
gaaactgcacctgacaatgactgcttcctccacctaagtggggccttcttccctctctcattcat
tcattcaacaaatacacactaagctcacatatgtgccaaacactgtggcaggcactcgcgcatgc
agggagcaagacttatacaactgctgccctcttgaagctctgaattgagcagggaggacaaatag
agtgg

>chr3_48507396_48507596
tggcaccactgccctggtccttccagctgcctgtcactggtatgatggccccggtgcattgtgcc
accagcaggccacagctgtggatcttggaaggcctctggggtcccccgggagcaggggagtgggt
gtgggggggaacggatggtggtgagagggacagaccaggcaggctgacgagcagggcgggcctgg
ctcac

>chr3_52028560_52028760
agtttgtgattcactccacggaacagagagcaaaagtaaagggaaggaacagactggggacatgc
tcagctctcccctagaccctccagccttctcagctaatccttagaacccccctgacacaggaagag
aagctgaccccaactccagcactgggtcagatggaggtcataaggctggtcagtgtgggaatgtg
ccctg

>chr3_53304160_53304360
aggcctggggctggagaggcctggaactcctcggaggtaagtgccagtgtgaggccagccaagcc
ctgccgggcctgctttccttgtgcataaagaaaacatgccgagtcattgtgacttgcacttttttg
actagtgcggtcaggctctgtgtggcactgccgtaggcctgggtacctcctagtgtgaggagcag
gagct

>chr3_171855906_171856106
cctttaagactttaggcctctttcttccaagagctctctcagaccactgcctgacttttccctga
attctggaggagcaggtagaggtagcactacacagtctggttatatgcgtaaacctttccttagc
tctcgaattagatgtgaattcagttgtgacaggaaacagtgtatcttcaccctgctccccagcca
cacag

>chr2_43446496_43446696
cagacacactcacatccaaacacagccatacccacagaccctccccccacatagcctccacatgc
agaagcacacagatccacacaccagtactcccctagggcgacatcacacatgcagacccctgaac
acctgcagacggagcaacaccacaaacctctgcacacccacagacagaaaaggacgcagaaacac
gtact

>chr2_87785085_87785285
ctagaacgtgacctgaatgtgaactagacattcgttttcctaaaccccatgagacccaggtattc
ttgacatgtgtagagaggattgaagggggcccgaggctgtctgtcctctctccgtgggcctggctc
tccttggaccaaatcgaatgcatgggaacacaccccccagttgtctttttccttcggtttatata
attcc

>chr2_101620368_101620568
aaaaaaggattgttactgcctagtttaagggttggtttaggctggttttaggaggacataccatc
tacagtaagtcttcactgagctttaagcacactggcctactgtatgctgtaaaaacttggttttg
ttacataagacattgcttaaagtcagtttccaggagcctatcatcgacattgaggatttctcaca
tgtac

>chr2_112221329_112221529
tcactatgttgcccaggccggtctcaaacttctgggctcaagcaatcatcccacttcgaccttcc
aaagtgctgggattacaggcgtgagccaccatgcctagctaagctctttagaaatgatttactcc
catttttgaagtttgccatgtcacagaaaatgagaccccactgagagattcaaatgtgcgggaa
gaaag

>chr2_112221729_112221929
acccacacaggctgggtttcagtttcaccagggagcaaacaccattatgcaaattattttccttg
ctcaactccccatcccccatcactggcttacatattcattcttcactctgaagttaatgtacaca
agaggccaactaaggatttgcatcctatttactttaaaatgcccaggtgcaattttgaaacaaga
ttctc

>chr2_219270156_219270356
atatgctaggcccaccagctaagtgtgtgtgggggtctctacgccagctcatcagtgcctcctt
gcccatccttcaccggtgcctttgggggatctgtaggaggtgggaccttctgtggggtttgggga
tctccaggaagcccgaccaagctgtccccttcccctgtgccaacccatctcctacagccccctgc
ctgat

>chr2_219270756_219270956
cacacctagagggctgttggtggagggaggggctgcccggccctcgacacttcaggtgggaaggg
cagcgtcagagcacaaatttgagcctccaggctgtgctcgtctacgtcttcccgcctcgggtatg
tggtctgcaaaatggagatgtgccctattggcaggactaattaagtgcctggacacagacgacag
gatac

>chr1_8773013_8773213
atggaggactatagtttattcttcccagccaaggattaatgtccctagggaaaaggccaaaaagg
tactcatataactagaaaagcacattatcctaaaataagacttgttataaaatctgaatcataaa
tcttacaagatacctttcaacattaaataggaactaatttagtaactggaggaggaacagtttgt
ctttt

>chr1_8933413_8933613
agatgaaaggttctgagcaagtccatcatttcatgtttttttgaaaaggattaagacacacaagag
aaagttccattcatttcaatttcaaaatgtccaactcccaacaatcttctagcatctggggtctt
ttgttgcttaaagttcattaatatccaagtagaaagttccacccattgactaagcaaaccactct
tactg

>chr1_24307213_24307413
cccggttggtccaagttaatgtcctgcttttattttgccttaaatttcccgtttggtccaggtta
tgccctgacctgcgaaagaaggatttacagcttgtattgagtgtgtaaaggaaggaggtgtgtgc
ccttgcgcagagctgggcctgtctctccagtgttttcagcctgcctttgcccctcacccacaccc
tagcc

>chr1_27049613_27049813
tggctggggagagaagggacaatgagaatgatttatgctggtcattttatgtaccagggtacata
gggtgatttttttggtggaagcctagtctgtcctaagatattactcctgagatctaagtgagcca
cgtgcagctggcaggaagctgaaagctgttggttgggagaggccttaatttttacacttgcccct
gcctg

>chr1_27050013_27050213
tctcggctctttctgggttatcaggcgccagtccaagtcctcccaggcagccagcttcatggcca
gagattacgagcactaaccttcctggcaggggcggtgtggcttcgagtttatgcagattagccat
gtgtttctcacttcaaggcgttccacaggaaaagccttttcctgtgtttggctctcagtgggcgg
ttccc

>chr1_27113213_27113413
tgccacgttactcacaggcctctggagctgagaacaagtgcgtggcactgaaaaacctgggggaa
gggccatgaaactccatttttttccccccttctccaacacacaacgactaaatatgatttgcctttt
ctccaccttgcacacaacagatatctgaaggcaatttaggtctcagggcctgaaatgagttacag
agtca

>chr1_28835413_28835613
gtaggtaaactgatttttttttttttaacagggagggtttgacaatctttggcagacttggagcaaa
agattgaggtgcatttcatgcctccttttgagagtcttgctctgtcgcccaggctgtagtgcagt
ggcgcaatcttggctgcaacctcagcctcccaagtagctgggattacaaacataagccaccacgc
ccagc

>chr1_36839413_36839613
tccaaatgctgcactttattgccttggggggttataagtgaactggctgcttctaaccagagctct
ctttgtataggatgaccccacccccctgaggcctgctctgctcccagcctgtgggagagtacctgc
tgcagccagagaggaagtgactcagcccagtgggaagtgggaggggcggtcagcaagagggccct
gcgcc

>chr1_36852413_36852613
tgtctttccagctgtcccctgggtcactctatatccatgtgtgggcacacgtgtctaagcttgtg
tgcactgctgggttttcctagggtgtgtggccacttgtgtgtatgtgcatttgtcatctccacat

aggcagtgccaggacacaagagctgatatgagtgaagtctggctgcctctacctgtgtcctttgt
ctctg

>chr1_36852613_36852813
aatgcctggtgcccactgagctgctgatagggctgactaacaagagtaatcaggacagctcccac
cgttgtgccacactctgcatggagcacttgagacagtattaggtaggaggctgtttacagctgag
gaagccagtgctcagagaaggaaattaatgtgcccaaacacacacagctaataatggcagggcta
ggatt

>chr1_37943813_37944013
gggacctgacctattgtggcaggtcagggaaggcttctcagaggcagtgacatctcacaagggtt
ctgaaggaagagcaggagtttgccagcagcattgtttctcccgttcgccacctgtctctcataac
acactttgttcaaactcaattttgggagctttgttctcccaaataaattctggatcttctgttag
agaca

>chr1_38465213_38465413
tgccagttcagagtaggtggggatggggaaggggtgcttctgggaaaccaggaatcccacatgtt
tctttctgtatttaactcggggtaggaagaggtcaggaaaaaaaccaggaagcagaagttgtttt
cttatctaggccctagcagatctagggggagggggcttttcctgggtcagtgactcatttcctgt
cccag

>chr1_53791212_53791412
aggcaggagggaaagatgtgctagagacatagactcagtcagtctagttctgccacgcacaagac
acaaagccaactttgggttaggtctgaaagtgaacgggagggtgactggcaaacagtcaaaaacc
aaaacgattcaacagagagggtgcctgtccaagtgtcccgtgacccttcctgtgcagtgtggggc
tgtgg

>chr1_150536376_150536576
gctgtgctgagaggcctgggaagagccctgtgctgggggaggaaggctaggttgggtcggcatgt
ttgtgtgctgtactcttctgagccttacctgaatctcttgagatagctccactgaactttgagtg
aagaggaaaagaaactgggggaaagagggtgggattataaaaaaaggagcagactgtacccctgg
gatcc

>chr1_150541176_150541376
cagtgacctaccttaggtgatgctcttaaaagagcaactttattcaaaccaggcctgccaatgcc
aactccttcagtctagatccaaagatggctatgatcctgagattgtgtgtttagggtcaccactt
taacatgatacaggaagcaattgagtcttttctacttcctaaacacagtccatccagttgtgtgt
cattt

>chr1_156074976_156075176
tgctcctctggggcgggcctgtgggctggcttagggatagcagacaggggaatctggggagttgg
actaatgtggatctagaagggaggtggttgggctgcacgatgccttaagactcctcccagctctg
aagttctacttctaggcaatgaatgatgaaacgctggcccaagtgcagtcccttccccatcccac
agggc

>chr1_156092576_156092776
acggtgaccttgggctgcccaccttctacccttccagcacccatactggctcccccaacctgcgg
ctgggctgggaggaggtcttggcccctaccaatcccttaaggaaggggaaagagtttgggaaggg
gagtcctcccttcacccctgcctcccccaagttgtgagagaggaagccggaatcctgcctgctga
agcca

>chr1_156093376_156093576
actatcttcttaggccatccctccctcctccaggcactacttcttttgctctatccccaagcccc
accccctgcatttttgtgacaacaccggaatgatttctagagagagaggccaggaagaaggaaagt
ggcacttggcaggagaccttgcagggggcggctggtgaggaagccagccgcccattgtccaggac
cccag

>chr1_156099776_156099976
agggcacccagactgggtcctgagaaaggaaagggtcaatattgtgcctggtcatccttgtctga
ggtccctctgagctctaaccagactttccttccccacagtcccacatgtgtaaaagggactagga
gaggtgaccagtacctttggggctcagatcgagaagtgctagggacatgtgggccatgagcttag
ttgtc

>chr1_156718176_156718376
cctttgtatccaggctgtgactcaaaacaagtgagtcaaggctaactgggtcagggtggggccaa
atagtgctggggaccccacggtacccccctccactagccagcaataggggaactcggctacctctt
aaccattccagatccaggcaagttacttattcttaggccttgcatttgcccgattccatcagaca
ggcct

>chr1_161089176_161089376
aggtatcagggaaatagcgagggagaggtagaatctttgttggacagtgtccctgggttgccaga
tatgagggtagagccttgagaagtcagtgaagagttgtcagtgtcccttccccccagggaatgtg
gactctggctgtccttgggtggtttccatgagcgtggccaagactgggagcagactcagaaaatc
tacaa

>chr1_234747577_234747777
tttttacaggctaaccttgaatgtcttttggggatttccatagaaggtgcgcctgttttaaaggt
ctctgcatctgttcctggatcttgccctgcaattgcaatcctgcctcaccctctccaagctggga
gaatggggcctgtccagatccttcccgggagtagctggtgccagcgggtttggtcactcccaggc
cctgc

>chr1_234859577_234859777
gcttcataaggaagcacgagctggggcagttggaaaccacatccctgtgagtgctgattacagcc
tactaaggaaaatgagatttttatctcacaggaattcagttataaaaataaaatacagcgtctgg
cttgattcagctaaatgtacaagcactagggtgtttttaatttaacagagaagctggacaagaag
aatga

>chr1_234859977_234860177
cagttggaggagagaggagaaaaacaactcggagctgtgctaatcctgtttgctgccctgaaggg
aatggaaggcggccaaggcagggagttctgggaaaccatataagggtgccaaacaagcattgccg
ccaacattaggaactgacagctttgttctttgtaacttggctgcccgccagaggcctcatttcag
ctctg

>chr9_91932380_91932580
ctgctagaggctgtgtagtgaactgagacccaccaggacagaaatctgaaaaaactggagctgca
cctgccttacttgcttgaaatacttcgtattcaatggtagagttaacgtctgtgcaagggacaga
ggaaacctacaatttgcctcaacctaatccccattcctcaaatagcctgttttgctgacattttc
tgttc

>chr9_131903179_131903379
ctgccctcctggaggagagagcctggggtgattctgagtgaagcgagtggtccaagacaaggccc
ccagggaccagagtgggctttgtctctgttgggtgcctcgggctcttgcctggctgtggtggagc
ctgggagagaagaggaggggagggaggagcctgttgtccttgctcctgaacatgggtgtttggat
gggct

>chr9_131903579_131903779
gagagcgtttctggcttatcttttctccttcaaaaaataacccagtctcccctgacacccgcatg
tttctagaaccttccaagtctctgtttctttgccagattcagcctctttgcagagtcttccttct
gcagcatgggtctggggtacagagccctgttcccctgtaaatgctgcctatgagctgggggctg
tgtcg

>chr9_131903779_131903979
aggggaattatgaactgcttggttaataaatacaaatgtgctggacttgcggtgtcagggggata
tgctcaggcacacaggcgtccctctgtggtttctgacgtcatgggaacttcatgctggctgcaga
atctttctcttccgtcctggagggtcagactctttggcaagagggaggaccaggaatggagccgt
ccttg

>chr9_136008579_136008779
cctcagggcctaatcctggggctcctcctttcggagccagggtgggcagggctacccggggcagt
gggcactgagcttgctggaaaggccatcgagggactgctgacacagtcaggctgcaggaggagca
gcaccagctgccacgcagaaatgcaggagtcccgaggaggtggtgctggcaggaggggagcagca
gggcc

>chr8_95449024_95449224
tggcttcacaagctttagcaaatcaagcttttcggataaggtaaaattggaagatctagcctttc
ctaccattgttcttttcaaattaagtaggcagtttctgaaaccaccatgcagccttcagctctgc

tcagctcttttcacaaacagaaaaagatcaggcttaactaggctagcctcatttaggcccacgcc
tacag

>chr8_103800824_103801024
gccacttgctaacggtgtgccctaagggtagttccataacctctctgagcttcgattttgtcttc
tgtgcaaaatagggctgaagcacctcacaagattatcacagagatactctgctactggactgtct
tccttcccttctcagctctctaatgaacgctctggcccacattttgggttgaggcctcccaggca
ggccc

>chr8_103801024_103801224
acccgctttgttcctgccctcttccctccctgttcacctagaagggacccaagagcagctcccag
gaaggcctgccccatcagagctggctagcaaggggcctgcggggttgctgagaagacaccactgc
attctcacccagtgacctttccccagggctcctagggagtgggtgctcacatctccccttcccag
ggtac

>chr8_103801624_103801824
ggagacattgctcaaagagaggcccagggccttttcccagccccgggggccgccctggagccag
gcctcagaggtctcagctctggagtccctgccagctgcttctccagaatttgcttctctgccttc
tcctactggggtggagatgaagagtatctaggatggccagttggagcagataacaatacaggatg
accag

>chr13_99136999_99137199
cctgatccttctggggacgtgcaagcaacaacagaacatccagagctctctacccagagcaaacc
ccgcctcaggaccaaggccgcctccttcctggagaggaagatggagaaataacacagccttcccg
cttccctccaactcattcctggtagcagacgcgctgggtggcaaggctacccagacacaaggctc
accag

>chr13_99137199_99137399
agggcactcaggaaaccagctcaagtccttattgaggtcactgcgtctcaaatacccgggctggg
cctgtgactcacacaccagcaccaacaaccaggggggtgggatattgcatcaggaaaagctccact
ctgggtggaacctgcttttcaacctcattgggtgtgtcaccgcccagtgggttcaccttgcccgc
tgcct

>chr12_14410733_14410933
agagaaaatccagcatggacttcgcagaaagaactggatttggcctggactcgctggagcctaaa
gctgctgccagtagtggcaaagtcagatgaaatataaggcctggagcgtggacagtggcctcttc
cagaaagagaaatcacacagcactgtctctgtgccataatccagtccgcattttctgtcttgctg
ctcct

>chr12_31901933_31902133
aatatggtggttcaaaattagtatttttcctctttgtaggggaaatcacccaaaagtaacaatca
gaactgttaccacctgactggttcttgcccactgcacagataaaaccaaaacactgagccagcag
gagttgcagcagagaaagagtttgattattgcaaggcaatagagtgaggaggacgggatacattt
cccag

>chr12_46776533_46776733
tttgttgtgtaaccatttgtcacttctacatggatgaggtcaacgaaatcctcaaagttgctgtc
tcaggaataaattttgttcgtggttgggaaaacgaagtggagaatgttatgggatagagaaaata
gcttattttctcagtggttaagtgggagtgggtaaaacacaattggccttgcatgtgctttgttt
aaacg

>chr12_109085271_109085471
ttctgctggcagcaattgtcagcctgcttagcacagactggcacgctccacaaacccagtaaaga
gttaaacgttctttgcacttgactcagtggcttctcttcaccacacactcccagatcccaccatt
ctctgagccacccgtgcagccaatcaaagcaaaggaaggaaattatcctggccggctgacagcca
cagcg

>chr12_109085471_109085671
ctctgatggccatatatggtaaaaaccactgtcagagccctgtcaggcctgcgggccggctgctg
tggacatatcagtgaactgcagcagcaggagaagctggccaaggaaacgcacaaaggctgcctgc
tcatctccaggggaggggggtggggtggaaaccatcatctgccagggcacacattcagtgagtcac
agcca

>chr12_109085671_109085871
ctcactgtcatcaaaaatagccccggggtacattgggaagttccttctgccaggactttgtgctc
cattgttaactgggggtgagcagggtgcccaccccacccctcccagcaaaactgtggataacaca
gtcactaggagaaaatgaaaaggcccttctgtggcacttcatgtggtaatgaggccaggccgtgc
tcgtg

>chr12_109086071_109086271
ctctgaacaaaatcctctgcccctaacaccaaagctaccaagtgccaaatgtccacatctgtaca
aagggctaccggcactgccactgttgtctaaaacagagtaccacaatgtgtagaaatggaaattg
cctgtcagcaatgcacagtaaggcagacatgcagccaatgcaaatccagcaggtgctggcaaaga
ctgtg

>chr12_109232071_109232271
cgtgttttccagaacagaagtggccctgtttcgtgcccagagcagaagagaacgatgaagagctc
tgctctcccaggtcttcctggtctgtgtgtgtccaggttttgagggcctctcacatacacggctc
tggaccacgtaagatctaattttagcattttcctgctcggagaccacatgtttggaacagcaggg
gctga

>chr12_122883847_122884047
gtcctgttttgggtatcaaatccaaatcctgtaagcaaaaggtcaaggattcagaagtgaaagga
aagactgtggctctaactttgccaaagtgaaaagtacagacctctgccatcagtgaagaaagaac
ccagatctgataagagcttcaaacaggctcagcacctcctggcacacatagagcggcatcacgtt
gcccc

>chr12_122884247_122884447
cgtccagaaacccaggtttcaacgcggccctgcttcaagacccaaaggaaattcagcaccaggcg
ggtgaagtgacttacctgtgcacagctgcactcgcagacttcggagcccccacctaattctaaaa
tgctgtatgtccatgaacttaataacagaaggtcaggggagagacgttctgagagcaacggaaat
ctatc

>chr12_122884447_122884647
ttaagataaagatgtttctgaggctgattttcaagtcaaacttgagagagaaattcaaaggcta
tgttctcatatcatattcctgggtttgggaggttcattcacagcttaatcctgtcccctgaagtc
acggttacgtaagtatgctgtgtcacttgctgtccccacccccaacacaggagatgcagtgtaac
ctgag

>chr12_125402847_125403047
cctgcggtattttttttccttgtcatgcactagtagaaatatttggctttgtttctttgtttttt
gaaacagtatctcgctctgtcatgcagtggtatgatcacggctcactctgcagcctggatgttcc
ggatcaggtgatcctcccacctcagccttctgtgtaggtaggactacaggtatgcgccaccacac
ctaat

>chr12_125403047_125403247
ttttgcatttttttgtagagaggagggtttcgcactttgggaggctgaggccggtggatcacctga
ggtcaggtgttcgagaccaacctgtccaacatgctgaaactccatctctactaaaaatacaaaaa
ttatcaggtgtggtagctcacgcctgtaatcccagctactccagaggctgaggcaggagaatcac
ttgag

>chr11_355600_355800
accgtctggaaatgctgcccagtggatttcagcctcattttacccagctcccaatcaagatggaa
ttgctctggttcaaacgcctagcttcgccagctagggggtgaaccggccacagatccccggaggc
caacagaagacagagcccctgggtcagaggcaaggacggttgacttctcaggcagggaggtggcc
aaggg

>chr11_46576224_46576424
tgtaaagaacaaagggagctaagtcaaactgaagtcacacagggctgctgatgaggagaaaatgc
cagtcaattgtgtttttcattcctcttttaaaaaggaagaagaaacaagtttcccaatctgtccc
ttaaagtaaagcagactgagacaaaggttaccaacaatagtctttgtgataccgtcacacagtcc
ccaaa

>chr11_47290424_47290624
agccctcgctaacactgtgctgtgtctgaagatcatgctgaccccacaaacggatgggcctgggg
gccactttgcacagggttctccagagccctgcccatcctgcctccaccacttcctgtttttccca

cagggccccaagaaaaattctccactgtcactctgtggtatggccataaatgcctcagcgccgcc
tcact

>chr11_57093224_57093424
taactgggagaaacttcctaatcagatcagctctcatttctagactctcaggtggcccatccggg
cagaggcgggtgaaaaccaatgctcgtccagccgggaacagaacaggatgtacctcgagacttgc
ttcccggctggccaggaaggccctgcccagtctgaccagagcagtaaacaccaccaataaaagtg
ggagc

>chr11_57093624_57093824
gtaagatgaggatttggatcctgcattgccctgcctcccaccctatctctccccaaattataaac
agccatccttgggaagcagcagagttaagacgtctccccactgccctagtgacatacacaccaac
aggagagcatgttcagatggcacagaatccagggactgcatttcatgaggagaaactggtaccaa
aatat

>chr11_62320424_62320624
ttcaagcccaaccagaacccaagttaaagctggttagagaacaagagacatgaggctgtctccag
agtaccgtctgggaggaaagaaggcaaagaaagactgtccaggcaggtggaagggaaagaaattg
ggcagggagagtggggcaggtggacacgatgggtcatggctggatttcaggcagagttaggggtg
tggga

>chr11_62320624_62320824
atgctgtggggcaataagaggagatgcagacctgagattaaggaaaagaagcgacaagacatgag
gctgtgaaggaaggagggagggagagagagaaaacagaagagctcagcactgagctaaggactgg
gcgggctgagttgtaaacaggagccgagggagggagttggagtaaacggagcagccaacgcccca
gccag

>chr11_62323824_62324024
aaggatccagcaagccatggagagcccagcggggatgggaagggcaagagcccttgctgcagtga
ctcaggcctccctggggcagggcgggggacagaaagaactccagatgtgcttcccctttcctgcc
tcacatctgccgttagaaacttccactgttaacctctcccctacccaccacagcagagaagccct
tgcat

>chr11_62650224_62650424
atctgtttcaatggaatgaattttgcccgggttcaaattcaagcttttgtaccaagtaatacata
ggaaacactcagtgacttgcatttgtgatttccttattttcccttaactcatagactgtctcatg
attgcgtcttcctccgttgttttaggcatccgtgtcattctggaccttactcccaactaccgggg
tgaga

>chr11_65259224_65259424
gggatgtgggccacacttgcatgaggaagaatgaatatacgtgtgtgtgtgtgtgtgtgtgtgtg
agcatgctcatggaggatgtttaatcagcttcatgctgggtgtgccaggccaatacaaacatctg
ccagggacctgaagcatgactgaaaagatcagggacacaagaacgcggctgcgactccctgagag
cttca

>chr11_71751152_71751352
ccgtctggtccaagtccaaagagtttcctgaggtcggggcaaaccagtttggtccatctaaaggg
actagcttgggctgaagcctcctcttcacacagaccactccatctttgtagcctagagtttggca
cagaaccccaaagccctggagaggaacaagggagggattacagaggaaaccaagcttctctggtt
tcttc

>chr11_118783190_118783390
gctcccccaccccgagtgacgcgagggtaggagctagcagcctaagggttgctaagggttaaagt
ggacttgatcctagctggggggagggagctgaggaaggctcagatgcccagagcctccaggtatga
cacaccggctctgctccacacacaactaccacaggtagggcaggcgttcacacatccagtggcaa
cctgc

>chr10_74014794_74014994
gcttcctctgctctgggtcctgggaaggggcttggcctccagaaggggccagatgccctggcctc
tcaccccaggcctggaagttagcctggcctgctagcgggcagcccaaaagagccagcagagaacc
agggcacgctcccaggaacaccagggctggtagcaagccgacctcctcccgcggaggctggggtt
tggcc

>chr10_103911610_103911810
tcttctgcagcagaggcaggagtctggaggctgggatggtatcagaaagggcctccctgggtatt
ggaggcagtgaagatgaagggcgagaatccattggccgtgccagtacgccatgggggcggttggg
agatcttgtagaacaactgttcgagcccgcttgtccactcattactctggaatccgagtcccagc
agggg

>chr17_2299850_2300050
gcaccccaccacccgctcagcatccctgctgaaagcccagcaggaaggcaaggcccactcaggag
ctggctgccaacaggggaaggggaggcacctccatctcccgcttgcctcgtcctctcccaggcgc
caccattctggttccctggctacggggctggcccaggcccttggagtctgcagactccaggccag
ggagc

>chr17_7486676_7486876
acccggcacctaaggaggcctgcccggtgcagactctcctgctcccaccggcgcccttccctcta
gagacgctgagagaacgggagctagtagcgcccccacccaacgccacctcggagactccggctcc
ttctctctcaacttcgaacaatacaaagtgtgctaggagaagacaagatggcgcccagcaggagg
agcgg

>chr17_17740475_17740675
agtcattcattttatccagcctcgcgcacaatggcagcggcctctggccggctgctgcgggaacg
cgggcaagccgagcccccctctctgggcctctgtttctccagcaccaagcaaggaagccgagtgga
agccccaggcctgggcccatcccaaacttcatttcctcatttcctagatttgactctttagaaca
agaca

>chr17_17744275_17744475
agatgacgctcgtggtctcattccagcttgggctctgtggtgagctgggcgagttctttccagcc
ctctctgagcctccatggccagctttgcccctggaggctcacacccatgtgggcacctgcaggct
tatcatgagtcactgaacacaccctctaccaaagagctacttgaaccccaaagattgctgcacag
tgagg

>chr17_27135474_27135674
actgtaaactactaagggttaaactgattgagagaaaatttgcctaagcaaccattctttaggaa
gagttcagtaactcaccaacaataaattctacatcctcatctgagtggtaaacagattacagact
ccatcaagtcctctactttcaggattcagactcatagcaaacaacttcctcaaaagattcagctt
ccaag

>chr17_27135674_27135874
agtcaaaccctgggggaggggattgtgcttaaacacacaaaatattgtctctgacacctgactga
aaaagctgaaaagctaaaaactaccatcacccacatctccatatgaatgcatccattgttctaaa
taagtctctgacaattgacagagcattctcagagtctcctgtgcctctaatattttctcacctag
agtaa

>chr17_33390687_33390887
tcatctcaggaaccaagagcagcggctttcatgtgaagtgaatggtgccgcctggggatgtatct
ttggtaagcaagattgcaacacagcagggagtttacccagggcagtgctgactcacgctgggcaa
aatttgtacttcccaaacaactcagcctgaaccaaccgcagtcaactgcaacctaggaaagaaga
ggcag

>chr17_33390887_33391087
ctcactgattgttttaaatcaagtgctgattactgggggcaggtaaacctcattcccccactact
ggtaacttggccacctccagagcccttacccaaaaagttccagtggccagtttctaaaatttcat
ctgagaacaggctggggctggggccagcaagattactgaacatagcaggttcacagctctgaaga
tttcc

>chr17_33391087_33391287
ccaggaggctgataaataagtcactttgcatttacaaattagaacatctgggtcagtggactcag
ggaacttgcccagtcatcattagccagtcaaatcattagggtgaatttttgtcagttctaaccag
gctcattgttctttttttcccactaggcctcctctgacctctctatttcttcagacaactcaagtt
ctctc

>chr17_38270274_38270474
ctctttccacgacataggcattcccttcaaagaatgtaggtcatagaaatggaattctgccattc
tataagccttagaggggtggtgaaagtattggcttttaactctttccatactcttagccagaagg

caaaaagaatggtcattatctcattagggacagaattgcttagggaggcccgacactgtggctca
atccg

>chr17_42172674_42172874
tgcccgctcccatttctcaacaccgtgccccatgcaaagcctcatactagaagagaaactgaggc
acagaggttggttctaggactgacagtgcccttggtaaccccaggagaggctggtggagaccttc
tggatatctctaccctctgtccccttctggggcctcatccctatctggggcagactgggcaggag
ggaga

>chr17_57864418_57864618
caaaattaagcaattttaagtggtcttgatggattctgttacaggatattttgactattcgcaca
gttaggtgtcggtaccaaaggtcacttacttgcactttgttcatcaagaaaaggtagcacttaga
gtaattctggagttgtgatgtctaagcacacttagcatgatttatagtggaattcttcgtgcccc
ctttt

>chr17_57864618_57864818
acttgctttctctagaagcatttactttattttgaactcttttgccacctcactcgcagacttta
atctttctccctttttaatttcttctatttctttaactcattcccatattatcaacaaggaaagat
agttgcaatgtgatgcaagctgttaaatgaatggccagtgggaaacttgtcttctcaacatacat
tccca

>chr17_57923218_57923418
aggtgccaactgtttgctcattctctttgacccactagccttgcagtttgctgtgtgggtgttga
tactcctaaattaaagcagggaaggggtggttttagatggcttttaacacctgctctccacccaa
gcaccctggattcatttgatatctggattctaattctgtagtcaggagactggcccaaactgtgg
cccag

>chr17_57924018_57924218
ggaggatctttctgtgaacttgctttgatgacctccacccaaagccctttgaggaacagttttgt
ttattcaaggagcccttggcaggtcactcctcccctaccacaaatctaatccaaatgcgacattt
tcctttgaggccatctatcccttttgggggacaaaacacatcagcttttcccttcagaccacttt
tgact

>chr16_2202999_2203199
cccatgagcgtgtggtgaagagggaggacggggagaagctggccaaggtgagtcagggcaggggg
gtggtgagggggtgcccctggaggctgcgagcctaggctgtccccgccaggccaccacctggctg
gcagcagctgtttacaggagtatggactgcccttcatggagaccagcgccaagacgggcctcaac
gtgga

>chr16_4665999_4666199
tatagctagcctcctagctctcctcactcaatctctcatcgcccactcgtggcatgggtccttcc
tagcctggctctgggcctgccccactgtagccccatgaccctctggacggcgcctgggacaggct
ggcggggccagcagcctctggtgcttcggggtcccccctcccagcagttctcccgctttcccacgc
aaaat

>chr16_20877499_20877699
gcgagctgaattatctcaaaacaaggactgtaccatgtggagggcctggtttccagatttgtgtt
cctaaagactagaatgctgaaagaaacaaaaggcccaaacttacacaaaccccacatgctcacag
actggcaactctaattcccaggcacgctgactcaccagggtttactgctcagctcctgctgctga
gtgag

>chr16_56965299_56965499
acgcgttttgaggggggactccagtgtgagcaggactgtggctgacttgtcaatcatcattagggt
gatgtcccaaacgttcaagtctgaggcccagggaaattggcaacagctgcctggatgcctccatc
ctcccagcccaaggaaaatctggccttgctccttcgttgtctttcttaggagcacgtggagttct
cctag

>chr16_56965499_56965699
gtgggcttgcactcggggtcgcccagccgttctttgagggttcgagtgggtaggcttcccctgta
ggacgggcttccccgtaggtttccacgtggggccacagaggcaggaaaaggggaagtgggcactg
acacctggggtgaaaaagggtgggggtgcggctttgtgaggaaagaatttgcagagactgctaaa
ctggg

>chr16_87421699_87421899
catggtggaacctcagcccgttccacctggtgttacacactcatttaggaaacagctgcgggtcg
cgtgactgatttgtcagtgctttaccacgcgttctactttgcagattcttgaagctgaacaggcc
agacttccctggggaggaagacactgaacatacagcctgctctagctacttattccctggattct
ccagg

>chr16_87886899_87887099
gccgcctgcctatctctcagaaattaaccaccccgctacaattctgtggatgccagagcggagac
ggctactacacaccaggaaagtctggttgaaaaatgtctgaaagtcagtgttttcgctgctattc
ctaaaataaaatacttgaaattccggaacttccactcctcacccaaactgaagaggtgattcttc
acctc

>chr16_87887099_87887299
ttcccagggctcccagggacatggagatgttaccagtccagagctctccagtccaaccctcaggg
ctcagcagggtcaggtcagtgggtctaggcagctccttgtccgctgcacagggcaaggggcaggt
cctggcttgccagtcccatttccttatggttgggggagcacgaccataaaagaaactgagcctc
gggga

>chr15_90943796_90943996
tagatgtggccatgaaaaaccagtgttaatcgggcagctgagtgtatggattgtccaccagtcaa
ggttggggaaaaaatctggctgggctaagtgtggcccgttagtttgacctcatttggcatgtatt
tcttctggtcttacaggtcagagcaactgtttttatatattttaatgtccctaatgacaaaatag
taaaa

>chr15_90943996_90944196
atacatttgaggacccaagttggcatgttctaggaatttcattttccggcttagtgtggtgacct
gcctctgattgctgtacaattccttctgcttcacagtgtcacatctggggataccagttggtagt
ggaacctgttttggacatttttaagccttgtggtatctgatggactcttggttgtttctagctgg
tgcta

>chr15_90944196_90944396
aataaaccccttgacaagaagtatttgtgtaagaaagcgctggtaggcaaagtgagaacatccgg
aagtgattcatattactgtggttgtttgcttaactcaggacttggttgcctagagaccatatcgt
tgagttagccgagtgaagtgccagtggaacctggagggcctgtggcctaccggatcaaatcaggg
ctctg

>chr14_23476760_23476960
gggccccgaggattcccaggcaaaatcaacagagtttagttttgctttgctttggaaggccaagc
caacgatatatatgtgcgaggaattctctcctttcttaggaagaaaaagcaagaacagtggatat
gaccctacgttcctggatgtagaaatcaaatagcctaggaaatcagggatacgggctctgcactg
ttcag

>chr14_61569647_61569847
agcacaaagcaaatagggcttgggtgggctcagggacaactaaaggggttggatgactaactccc
ttttctgtgaccatccctgcctcttgtttactccactgaaaaagggcgtagggaggtcagttgcc
gctgctgatctgcacaaaggtcaaaggcagagccaccgaaagtagtctgctcctgtttttgaagc
tttct

>chr14_74208647_74208847
ttggacaagaagtgcagagctgcgggctgcagaaacaacctttctcttgcttttaaacccgcatt
ccctgacagccaacctaattcagagcaggaacccaaaagctaaaccctgcactgaaaatcaccct
gggcacccccatgaagctgcagggtgggactgtctggctccgcctctccgagggaacatcaagcc
cacag

>chr14_77422247_77422447
attcatgctgagacctgggccagccatgcaaagaagggggtgtaggcagaagtgccgcagtggg
aacaggctagaagttgggcctggtgctgggacagtcccagttcaggcatgtcctcctggtgtcgt
ttttaatagctcatcctttcacaatcaagagtggctcatgccggagatgagttacccgcaatgag
gggct

>chr14_77422447_77422647
ggatttattctaagggcagagggaagccatcacaggaaatgatgtgattgatgttttgaaaagaa
ctcgctggctgttccgtgaagaataaattagaggggcaggagggaagaagtctgcctggggcggc

tgcagcaacccaggcgtgctgcgaggtgccctttgactagattccagtgggggctttcgggcgag
cagaa

>chr14_105147555_105147755
aaagcaggggtagggaagggccctcccagcgccccactgtaataggggcctcatcaatgccccat
gctcactgaataaagcactgccagcgaaaggtgaaaagaggaacaaagaacattctcctggacgc
cacccacagaaagccacgtgcaggcttggccctcaccttggggaccttggacacggagctggtta
tgtca

>chr14_105147755_105147955
catctggctctcagagctggggcagcgtctaggaggcctgatgtagaaagcactcagctaagccc
tagttaccggcacacgggcaccagcgccccctctcagcaaactccacgtcttatgaaattagcac
tggatttccacttcaattggaaaatatggccaaggtaagagatcaggaacgacaaggccaagctg
gggta

>chr19_4375400_4375600
tcagggcaggtggaaggggaggggcaacgccccagggctgggcgaggaatctgggagcaggtggg
aagctcatgctaccttcccaggagacgggaggacaggcctgccaaccaggggggctcagctatgtg
ggacacacgctcccacccttgcccttgcacagggtttcatccaaacatggagatgccagtgagtg
cctgg

>chr19_4376200_4376400
tcccttccatttctgaaggacttccatttctttgtgactgtgggccaccatgggcatgagtcacc
tgtcaccagagcctggtgtccctgtgcagctgtgccaggtgggcagaggagacaggtgaaggcac
attcctcagcgcccgctcacagctctgcccaccgtgcattctgtagagaacctcagcgccctcct
ccctg

>chr19_13275600_13275800
aattaactgttgtgttaattgaggcttttgtgagtgtacttaggaggggtgaggtgaatcacacc
ttttctcacttgatggagttacagggctttctcctctattgctgagggctgtgcagtcagggacg
gggcagggctagcctgggagaagcctgtgcactgagctcagagacgttcaaggcttttccccaga
taggt

>chr19_18393400_18393600
ccaactccaggggctgggctgatgaggcaggaatgccgggtataattattacatgcttgttacca
ctatcatggctgttagggcttaatgagccactattaagactggctgggaagggggtgtgagggag
gagagatggacccaccccaccaatactcacaagggatccttaggacatctggatctgacctacag
cctct

>chr19_18476200_18476400
ggagtacagtagtgtgatcatggatcactgcagcctctacctcctgggctcaagtgatcctccca
tctcagcctcctgagtagctgggactacaggcatgcaccaccacacctggctaatttttaaattt
tttatacagacagggtttcaccatgttgcccaggctggtctcgaactcctggactcaaactgatc
ctcct

>chr19_18485000_18485200
ccagctgaggatgacgatttgggatgttaggtcacccaggtgctgtgctgtgtaactcaggcaca
gagctgccctgttccgagcctcagtttacccctgtgactcagagcagatctccatacccccatca
cagggctgtgtatccgagctcagccagagcctggcatgcagtaggtgcttcataaatacacactg
tggac

>chr19_34662760_34662960
acagaaaccctggttcacggaattgatccaagctacatactgataattgggattagtgagttaca
atggcgttgcaagccatgaaactggttcagaccggctgaatgagcgggacaggaaggggctgaaa
cacttgagtccttgtgagagccccagctccaggggcagcctcgggggaagctccccgggcgatga
ctgcg

>chr19_39155960_39156160
ccttctttgaaaaggggcatcatcctatcctcagtttgaggattagacgtgatagtgcgcaggaa
gcactctggtgcctggtacattgtaagtgctcaataaatgcagcttcccaaaatgatttcaatgg
cacatggtcctgcatccaggtgggatctgctacttgagtctccccctcaaaggggcctgctgaaa
tacgc

>chr19_39156560_39156760
caggctgccctctcttcagttggtgtgtgatgtagaagcaggaagggcttcccactgataaccct
aaaaataactgcccagaaatgctgtcagatggggccaagaggtgcacaggagcctgtgcccagct
gccgacctggaacgtgggactttctagagggcttggaggagccctgccccttccaggcctcttcc
caaca

>chr19_39157160_39157360
gcatctccagttgtgggatccacgtgccaggagaacacgaagtacacacaggtccgcagtgggag
cccgctcggagacacctccccgccatcacggctgggactgtgcatatccaaacacttcttgcctc
tgagatgagcagttttcttccccttgactgtgccagggttcaattagcctgggcattaattaaaa
cttgt

>chr19_39174160_39174360
ccatttggcccacaggccaccaactttttcccccccttctctaaaagacacacaatggcagttggg
ctgcacctttgtgagtcaccggtgatagcccatcataaattgttatttcctatatttccagagca
gctttatcgggcgttgcctgtgcagtccgggaaccgatttggtatggggtcagttaacatgctgt
cttgt

>chr19_39174560_39174760
caaggacctcctgggactgcccttggttttgtgtcttccttagagtagatcaaatgagaggaacc
atgtgaagtagctcacacagggcctaacagagcaaacacaagacgtggaaagcagacttggtgag
gcttgagtttagtttggggggttgcgggggtcccagctctgctgcttaacagccctgtggccatgg
gtata

>chr19_39175160_39175360
tgtcctttgacgtcatacccacaccgctcctggagacagccaccccttcatgccagccccaggaa
ggcttgtaggtggggcgagccagggtgagagtgtagccctgttcctcggccagggcagatgtttc
accatttttaatggagcaattatgagtcagaggtttcagtctctactggttcctcccgatcctat
aatta

>chr19_39176160_39176360
cacccaacctctggatcgaggggactcaattatgcggataaaggcccctaatcctctcagtgctc
caaactcactctgcttctgaaagggatcttcgtaccctgggttcagagagtaggaagcccgcagg
gaccgagtatgcatgaaacaagcccaagtctgtgggggctgtacatggtcaagatcacagatttc
cagac

>chr19_39176360_39176560
caagccagctccaccctagctgggatgcccaccctgagacacccctctctgctttcactggaata
ggatggccctgacttggccgggtggactgttgaatcagagcctctcaaaacaggaaaaaggaaaa
ggatcaccaagcaggagtttcagttgtcaaagacaaaacccttaccacgattaaagattactcca
gggcc

>chr19_39176760_39176960
cgggtgtgtagcacttgcctaaataacaatcccatcagtgccctgtctgaccttttctccccaaa
tgccaaaggcctcttgtctggatgaacaaatccctgggcggatttgtccagactaaaacgttaat
tctaaagactgggccccttaggcactcgctaagattccaggccacactgtctcaaggccagaatt
ggctt

>chr19_39892760_39892960
cacaggggagggcagtcattccctcacccaggcacctgcgcggagttacacacacttagtcactg
cagatcccacagtggctcagagctacgcacagatatgcaggtggggatgaaaatctcatgttctt
aagaatgccgcctcagagacaaattgcagtacagttacaacagagcatccaccccacacccagag
atggc

>chr19_39892960_39893160
caaagtacacacggtacagtcgatcacacaggcggtttcacacattgattcacacagccaagtca
tgcgggattgtatgcacacagccttctcacaggaaactgggcacactcgtgtccctcacgttccc
ccactgcatacccaggcacacaggctctcgcagccccagatgccaaggcgcacacctttgcaccc
gcaag

>chr19_47611960_47612160
aaaccacagcagtggcttactaaaatagctggctctacttagagaaaactatggcttataaagaa
atcaaaaatttaaaccttcagagcagagcaaaattgtacctgggggagaggacaggccatgaatt

agtcattttggggaaccgcatttttttcttttaaagagcaaatttgaggggaattaaaaaaataa
aggag

>chr18_3596600_3596800
ggaagaattacaagatcaggcactgctgtctgtctgttccacggatgtaaccacagcacacgcgt
ggctcacggtactagtgtgataaatgcttgttacatgaaggcgtgaacagggatgagaagagact
tcctggagaaacaaaaggactaacaatcaggaaggggaggtgatcggggcaggagtaaagtggac
acctc

>chr18_3603200_3603400
cttgtcaccctcaggcacctggctgcgaagtaataaaacggttgccttatcatctaaaggtggag
tgagtcaaacctcagcccagggtaattatagtaaactgaaattgcctgtcctttacaaaaaaaaa
ggcggagggagagagaaaggaaaagggcttaaggcaggaaattgtgtttgtctttctccttggat
ttaaa

>chr18_20840002_20840202
ggtaagagacaggaagcagacaagccaagaggttgctgcagctgcccccaggaggaaacgggcag
cagggagtgtggcccagcccccactgtacccctccaggggcccgagcccttgccagcccaatgac
accttgaagtcaccacttttcctttcttgcccgctaataaaacctatttaaacaggaaattcata
ccaaa

>chr22_30592000_30592200
aaacaaacagagagactgccctttctgtaaactcaccgaatgtcaaatattgttcactcgcatgg
cattggcttactaagccactacagagccacttccctcacgtaatcccctcaatccaaaggcactt
caacttgaagaggatcaactgaaataatacacatttcctctgatgattggacaagaggcagccga
tggaa

>chr20_30160139_30160339
tcgttgtaacaacactttgaggtacctacatcattcttacgatggggaaactgaggcagagaccg
taagggcgggcccagggggcatgtgaacccagagtgtctggcttccaagactcgtattcaccgtg
gtgccgtttgacatgatcaggataatggatccctggttccttttgggggcagcattggagaaggg
ggttt

>chr20_30307139_30307339
actcctacccttaaggatctcccagccgcagtctccaggataattacccgatgccaccacagtag
ccagtcggtgcagcttggaacaggataatacctgagaggaaggggggcgggtaccttctacccagg
ctcaaaggatcctgagtcaaatgttctttactccctggccttagggagagaatgtgtgctcagaa
atggt

>chr20_30307739_30307939
gtccctcctgtaatcagctgaacaaatatttgccccaaaagagaatcaaagcaggagggaacagg
agaggtcactgcacaaaccaacaggaactatttaatcatttatcccccctagagagttcatttaca
gaatggctttcaagtctagtcagctagctcaagagaacccaccacctttacctcccctgacccca
cccca

>chr20_45978193_45978393
atacaacggtgatttattgaaggttatttcagcactaatgctttctattccaacttaaggagaaa
acattctcaactcagaaacactctgcctgaaacatacacaggttgtccaactggatgctgttgat
gggttctggcttggggtgactggaaatacatcttaacaaaaagccccatgcagccctcggagatg
ggtag

>chr20_45987993_45988193
cacttgatcacttgaaatgttttctggggcaaagagcaggaagctgctggcttattctggcagct
ctgctctgtttgaggaatgctgttggcattcgggtaggtctgaggttatctggaaaacagcaatt
cggctggggacagacacaaaagtatggtggcagatggaatcctgggaggaacacagattgaaaag
aaagc

>chr20_48782993_48783193
aggaccacattttgcttgtttgttcatgtgatcgatgatgatggacacttaggttgtttccactt
tttagctatcgtaaacactgttgctaatggaatatgggtgtacaaatatttcttcaagatcttgc
tttcaattcttttgggtatatacccagaagtggaactgctgcatcatatggtaatttctgtgttt
aattt

```
>chr20_49434993_49435193
tctctctctctcactcaaccccaagtgcttcctgaggcggccatcttgatctcgtccttccctcg
agatggttttattccgcagaggagccaggagtggaccacaaggtggcactctcgggcaccgatg
cagggagccgttcagcggggacgctagactatatttaactttcctcagctctgcaccgtcccctg
tgcaa

>chr21_35319730_35319930
ctttttttcttatcttacgtcgcagtgtactttgtctcttgtaactcctgcaggatttttcttaa
agccttacatggtgctgttatcacgtggcacaaaatgaagccgacctctttgtgtgtgggaatag
agggagcattacactgctgggatgcaacagatggagggtggaaagctgtccagcaccttgtctct
tccaa

>chr21_35320530_35320730
tctgcaagccagcgaggctgctggattttctcgaagggtattgagttgctaagcaacagcactgg
cggcgcctatcatgcccagcgatgtcaatgcctgagtgtggcctgcagagcacatttgctcccct
ccccctcgcctcctctcgaccccttccccccactacatgagaacattaatttgcatctcagggac
aggag

>chr21_35320930_35321130
cttgaagtgtttttttcctgtctgcccatccagcatcttatttatgccccattctgtccctctct
ctcgttgcctttgttctgtggtctcctgggtgatggagttattgtttgtgtgtagggatgtgtgg
ataaaagggagctgatttctagaggtgagatggccctgtggttaacggttggaggctgctgttga
caagg

>chr7_44679275_44679475
gggcttgctgttgttgggtgtgggggctcacccagtcctggagacagaatgggctctttgtctga
aggtggggcagggtgctcccccagtggtgtgggaactgagccaactggagagtgaagtgggatgc
atggtgtaagatgaataagggaaaggctgcagaagttgcattcccagcattttccttcccgggga
ttccc

>chr7_100143264_100143464
ctggttagaaataccagaatgaatttgctgtgattacctcttttgggaggaacctgagagttatg
ttggggagctgggtgcaagtgggagagctgggcctgtttaattccaggccagtgactcacatctg
gtccgctgcatgaccctggtgtgtacccagtgaaatgcagcacacctaggccacatggttttagc
tacca

>chr6_7147201_7147401
tcttaacatcacatgcattttgtagtttatggtctccagtctccagctgtttttggagcaccttc
taactttgagagggtgagctctagcctgtaaaatggactgtgggtggctcgtggagaaggtgccc
tggtgtgcttttctgtgtcctctctggattctccctgagctgtccacctctgaagcctgcttcac
cttca

>chr6_26025621_26025821
agcaaactgatagaatttggagaggtggttaggcatcccgggaatgagaaacagcccgaagctgc
cactatcacaggctttggcattgctagaagttaacgtggcacttacagctaggccgtggtgttct
gttgaacaaactatttgacagagcacagagcatgtaagtggtgaggccagttgagttagccaaga
aaaga

>chr6_29932221_29932421
gaaaaagacgggaataattagaagggcagccataatcatttgggagagacagcagcatcctcctg
ggtaaggagtcctgccagctaagcagaaattcccaaatgcagatcctggatgggataataatgac
cccagggatcgggtccaaatgcaagaccatagggagctaataattagagggattatgcagtccac
tcata

>chr6_30582221_30582421
gaactaatcaagtgcagaacgtgatacagcactgaatacagtttatccccaaactgagaggtggg
aatgagggcgatttagaagaaagtcctaaaagtacccaccttcccccgattctcattacacaaag
cgaccaaatgcaggaggcccactggttcctaagcagaaatggcacacttcagtgtcattaggccc
gttta

>chr6_33558022_33558222
cagggtgtgggtggagcctggagagggctgaaggctttctcctggacttcctggccagagcccat
ggtgtccttgactcagaccccgagctggggccgggtggaagggcccatctggactgcccccctga
```

gtcagccaggaggcagccttgtgaccagatgactcacctgtgccctaattgccctgttaatcatt
tcctc

>chr6_34625022_34625222
ctgcaaaatatgttttggagggaagaaactatttgtgcactcaggacaattgccaattctcatcc
ccagcttgtgtcatatttcaccctttggctctgccatcaggataggactttaagaccactgcagt
aggaacactagtaagtgggaaagtcaagctacccaacttaggcagatccattcctatcctcaaat
ctcct

>chr6_34625622_34625822
caacaagtaattcttcatgaaatggagctggctaaggtgatgggaaagggaaaacattgacccag
cattcagtgcagtctgaaagtttaaacctccagattccttcagtttttctgctcaaaaaagtttt
aaatctaatttcagatggttaaaagtaccctgaagaacaaacagaggcaacgttaacagtgataa
aaagc

>chr6_44205022_44205222
gaggtcaggttggcagtgtccccccaaacccttggggaccaactgtttcctggggcaggttggca
ctgagggtggaaatgggcagcgcccaactccctctggccccaacctcatcaatggccttatgtcc
gggctggacccctccagcccatctcggtatgacctggcttcctgtgtagatgctctcggggagag
gacgt

>chr6_74289279_74289479
ggggatttgccagaggcggtgcctcccttccccagcagggcatgtgctccagatcttgtgccact
tgacatgcagagcctgggtgcaacctgcagaaaccatgtgggctgtgccccagccttggggtggg
tgtggacctggtctccctggggatactggaagggcctctgggagatatttagggcgcagcctgga
catct

>chr5_180669194_180669394
agggttccatcccaggagcctgagagggcaaactggccatctgaggagataaccacatcactaac
aaagtgggagtgaccccgcagagcacgctgtggaattccatagttggtctcatccctggtcagtt
tccacatgatgatggtcttatctaaaggaggtaaaacagaagaaaaatcagtgaggaacccgcag
cccgt

>chr3_39192196_39192396
aggctcagatacgcactgcggctctgcctaagggatcgccacagaaagagaacttaaggtgagga
ggttatgacctccaagcctacagacagggaaacagagacacagggaagctacggtcatgctgcag
gttctagtgatgaacaatcccctccaaatctgcagagcgggaatgaaatgagaaattacctcctc
cctga

>chr3_53303960_53304160
ccactgtccccctttggttggatgtttgcaatacttctgcttttatactgcaacaaacaatactt
gtgctttccctccatctagcactgtgcctccaggagagagggttttttctggtcgcccctgcag
acatgaccctgcttgtccctggccaatcccagggccagactggattgttggccctctgactcacc
ctaga

>chr3_69101910_69102110
aaagcacaacctctctctcaaaatagtagtaaactcattgctgcagtcaaaatacccctaatact
gtggtctgtcggtgtcccctgcacaaattcctatgttgaaacctaatctccaatgggacagtatt
aaggggagcctttaggagatgattagatgatgatggagccctcataaatggggttagtgccctta
taaga

>chr3_133291510_133291710
tcaaggccccaagggtatttaaatgggctttaaacccatttaaacctggctttcgcctgggcatt
ttggtctccacccctatgataagacagtcccctccttttatcagggatagaagctaaggataaac
tgtatcatcatctagactcaagtcagggcgttaagcaatgtgctgagcagctaataatgttgact
ctctt

>chr2_26981296_26981496
tggcagggacgtggagcctcactgtaggtcacaccctggcgtgaagggctgatcagccacccccat
ggtgtccacatggttgtctctgcctggggtgaccctgctttctgcacctggagcaatcctgctcc
tccttcaagtccctactcaggtctcctctgaattccagttctacagggctactgtgaaaaccaag
gccac

>chr2_70313096_70313296
actgtggggacccaaattatcccttgatactgccccccctattatccactattaagctttaaaaag
gcgagagattaaatattgtgcccacattttacagtcattcgcgttcccgtttcaaattaaaggga
ggaaaacgcgttaaaattaggcctccgaccttcagaccagcctgtgggcttttttaagtcataaa
actcg

>chr2_87784885_87785085
ctaacaaatttatttatcatgacaggaaaggaaagcatagctttgtgcaccccatcccacaccct
ccaccccctacaaattttactaacactgtttctgggttcagaattttttgcctgagttggtacc
tcaaatcacttgagttaaactgcaaatgaatcacacataatgcagataaaatgacttcatgccct
aattt

>chr2_112222129_112222329
gagagtggacagacagcctcgggccccttcaatcctcgctacacatgtcaagaatacctgggtct
catggggtttaggaaaacgaatgtctagttcacattcaggtcacgttctagaaattagggcatga
agtcattttatctgcattatgtgtgattcatttgcagtttaactcaagtgatttgaggtaccaac
tcagg

>chr2_202022755_202022955
gctttagccattttcttctgttcttttaggtaaggattttctgccccagacctcagattcccat
ttccttcctagcttctctgctgttatagctcagtctcttccaagaccatgcaagattcagaccct
agtaggaggagatggaggaactaggacttaaacccacatcttgtgattccaaggctggttcctct
tttgc

>chr2_216980155_216980355
ggatgcttgtctaggcggcagatacctgcccagccagtatgctacgcatgcttgaatccagcagc
aggcacccagacaccgttagtccatgtacaactgggaattgttgtgttgtttacagactaaagga
ttaaacagaccctacaaaaatgaagaatgacagaattccattatctctccatacagcactcttcc
ccatc

>chr2_219270356_219270556
cccctgctggctgggggcagctcccaggatatcctgccttccaactgtttctgaagcccctcctc
ctaacatggcgattccggaggtcaaggccttgggctctccccagggtctaacggttaaggggacc
cacataccagtgccaaggggggatgtcaagtggtgatgtcgttgtgctcccctcccccagagcggg
tgggc

>chr1_8772413_8772613
taacttctaacgtttacatcatctgattaattccaacaataaaatctaaacactgatcatcccct
ccaatcttcagagcaagctgactctacagtgaagaatacgagagccctatcagctgactctggta
tgtttaaacgattataatgtaaagcatgttcattctcttaagtgaaaggaaatgactgccaaatg
cttcc

>chr1_8772613_8772813
ttcacaatctctccagaatagggcaggtcctttaatgtgagattttcctgacattccaattgtag
cagacattgaacaaacagagacgcattaaaagccgacctggttctattatcttttattgaaggcc
tagcaaagatcagacatgagccctgtgctatttacatttttttggtgcattaatcacttttatact
caagg

>chr1_23881813_23882013
gacatgaatttgaattctggctttgttacttagaagccgtgtggccaccggcaagttacttcgcc
tgttctgagctagttcatttataaaataaagacacctcccaaatgtctgtatttcacagccgatg
cgatcagctctgaatccgatcgcatgcgacaatttgcacaagacactcagcagtgaatcttgtag
ttaag

>chr1_23882613_23882813
attagcttaataatacctcgtaggtgtgtgatgcatttcaaagaactttcccaccattatcttct
gtcctgggaggcagcagggcacagaaggagcatggctcagagaggtggaatgcttgctcaaggtc
actcggcagatgaccaccgcagccctttttagccttccacggtactgaaacttccgagcaaacgg
gaggc

>chr1_27113413_27113613
agagatacagtcccaggcccccacagactgatgtgggctggtcttcgcctggagaggtagcaacc
atctctttgttcccacccacatgagtagccgttgcagaagataaacccctccctgagccaggctg

tggccagaggctaacccggcccctggcttcgtttggagggcagataactgaaaccacataaggct
ttgga

>chr1_36839013_36839213
ctattattgaaaaagcaaagcctaaagactgtggcccaggttgaggctctggtgagggcttttcc
actgagtctgaatgacagccatccctgaagaaggcatgcaccctctccccgggagcttggtggct
atgagcaatctccttaggaacccattggaagccatttgctttgtgctcaggacagatgctgacag
gctgg

>chr1_36852813_36853013
caaatcaagtctgatggattccaaaaaacttggctttgaatccctgctgcatcacttacaagctg
ggtgactatgtgcaaattgcccaatcttccccttctgtccaaacagggataacgcttggggatc
actgtgaggactcggagttatagaaacagcttttttgcaagacccagcgagcactcatactcatct
cagtt

>chr1_38465013_38465213
ctgtctgtctggatgtacttgcgtccatacagggactcgttgcattttgcacagtcaaatgactc
gctcatggtggcaaggggagagaaccctgttaggagcacaaggtggaactggggtcacacagaga
ggctgtggcttggctcctggcaaagaggcctctgcattcaaaacacagccaagagacccggcata
ggaaa

>chr1_45273013_45273213
tttggcttctgagttctgtcctggctgcacagccctggccccgctggcctgaagggcaagtggga
ggggaaagacctgtgttgtggagttgccatgggttgctagagcaaacactcctccctcccaggat
ccctccctccagtctggctctggcttaacctgagagcattgttgctgtgagcatgtgctgagcat
ggggt

>chr1_45273813_45274013
gacaactcatgtgactcctcctctggcagggatgggactgtcccctcccaaacaagccaggtcgg
ggccctgacccatctatgggggcgaggattttccctgttgagtgggggtttccccggcctggcta
accggatcctgaccctgtgtgcctgtggaggagggaagggttaggagggcaaggagtttgaactc
tgaac

>chr1_110546277_110546477
gactggcatgtgtttatataatattggtggtgggactgagaaaagaagtatggacaggtcaggca
actaggtaggtagtagatagctaaataggcattgaaacagtctgggtgtgtgttacattctgttg
caaattgtggtgttcaaaagattgggaagccctttctgtcctacttactgcctttttttctaact
tctta

>chr1_150536176_150536376
actcaagagggtgtttcagggctgagtctgaaggaagaaaggatttgagggaggaatgtataaga
ttagtccaaaacaaggtatgcacgagatatggcagtcagatgccagggagccatcagctctgcca
gctgaaggggaggtgcagagttggctggaagctggccagagcagagcatttctcctgtgagcact
ggaga

>chr1_156073976_156074176
caagaggtgcgtgtatggaggggtatagctcagcctcccagctcgggtggggagcggtggctcag
gcctgtgtaggctggcttttgttggggaggagcctggaagggcctgcagctactggcctccctcc
tccttcctccttgcttagcaactgttgtcgtctggtaaatatttgccccaacaggatctggggct
ggagc

>chr1_156075576_156075776
ttaggtaggatgaacaactgtccctattttagcattaaaagtcccttatctggccaggcacggtg
gctcacgcctgtaatcccagcactttgggaggccgaggcaggcggatcacgaggccaggagaccg
agaccatcctggctaacacggtgaaaccccgtctctacttaaaaaaaaacagaaaacaaaaaaca
aaaaa

>chr1_156093176_156093376
gactgcctaggtcctccctcacttcttctcctgaccttggggtgtggctcccactctctcccagt
gtcctcagggttaataactatgtgccaccagatagagagttaaggggctgctgaattggcttctt
gtgaagggaatcccctaaatgtccctcgttttggtcactggcctccctcccgccccccttcaggac
attct

>chr1_156093976_156094176
gcttttgaggtgtatgatattcagaacttcacaggttggggtttggggaaggctcaaggggcttc
taagtccctggaacagctgcccccctcagttcctctctctctctcttttttttttttgagatggag
tctcgctctgttgcccaggctagaatgcagtggcgcgatcttggctcactgcaaactccgcctcc
tgggt

>chr1_156095976_156096176
cctgtgtgctgcctggcaatggggaactctgagggctggtgagcagggctgctgaggagtgggtc
taaggagtccctgcagggctgggccagctcctccacctcccctttgtcttcccctcccacttgtt
atttttagctacagtgtctgtccctcttgcttctcccccagattgggagaggaaacggaggcctc
tccct

>chr1_156099576_156099776
cgactggttatagctaaagctttacccactttgaggagcagggaggcttaaagctggggcccaga
tggacctggaggcctgggatccacatctggaaccagatgctgaggctatggtagatgggtagggc
tcagccttctcccagggcacggatgaggcaggagggagggaggcagggacccctctgttcagtgc
agatc

>chr1_156717776_156717976
gaatgggattaaaaatagcatgaggcacaccagatatgccagccctgctgacttctagctacata
aaggatgggtctagagggaagagagaggaagacaggggcctgagcagataggcatctccccccaa
agcatcatcccacctggctccctcacctagagtataggcacttgtggggaagggccaggggaggt
gtgct

>chr1_161171376_161171576
gaaagacgttagggaaaagaggctgacgcaagacggaagtggggtgtgaggcatctctaagggcg
cttcctgggtctgtgattctgtaggttcgggaagcctggaggatgtcagggtcacccacagagga
cctccactcccccatctgggatgaggtggctcctacacaccccgacactcccctccgcaccacca
atgcc

>chr1_234859377_234859577
tgtgtggaaccgtaggggcttccctggacaagaacgtgtgtccatgtgtgtgtgcatacatgtat
aatggtctttgtgtgcacatcctctgtgtgtgtgtgtgtgtgtgtgtgtgtgtgtgtgtgtgtat
gagtgagcaatgtgtgaccggcctcgggccctttaaacctgaacaggagccaagttcatttctca
gcgga

>chr1_234860177_234860377
cggcatgattaccctggacggccgccagccccgcgctgagagctccttttcttctctccgagctt
ttttttttttttcctctttccctctgccaaacatgcagcagttaccatggcagtggaaagtaaagt
tttaaaaaaacacaacaaagtagagaggcacgcctttatccagatgtgccacatgagcagataga
aaagg

>chr1_234860377_234860577
cagttcacctattcatcggggcctttgactgcagaagaaaacactggggcactggctgtcaagtt
cgggagggaggtgactccaggataaacagagcagcgagcagccctggctgcagacagagaggggc
tggctcccaggccctattttccttccctctctctctcccttcctggggcattgtggagtgcgtaa
tgtga

>chr1_234860577_234860777
aacggaaaatgtcatgatttccactgggaacgtgtccacggttttgagaaagctccagaaactca
ggtccataaagcactgaggggaggaggaagggagggaggagaatgggcggtcacagtgccctctg
gtcaccaaggtgaacccagcgagcaggagccagtacgtggctgtggcccaagcctctgcctgctg
ctgga

>chr9_99182979_99183179
actgggcagaaggtgattccaggagcaaagctgaggtcataccagatctttgcttgtttggctcc
attttcatgcaatgaagaagctgtctgaagtgtatggatcatattcctgatgtctttgttctgtc
tcagatattaacagcccataatgtcagaggcctgtgaaccagagcaactccattttaaaaaggag
ctggg

>chr9_132176379_132176579
tccttggtttccaggcgctgtaggccaggagaggagggggcaggaaccccggcccagcggtccca
gacgttttcgaagcgacagaccctgggcccagcgcggggacgcggaggggacacgggctagcagc

cctgccctccggagctcaggcccagtccgggcttcccaggccccggggctgccctcccggagccc
gacct

>chr9_132176579_132176779
cccctgggggtgggggtgctgccccggggccccccttcctgggtttcctggtccttttgttcacgt
atccccctccccggattgaaccagcaaagagcacaaagcagaaaatacatccttgccgcccgcag
gctccctcatcccgcagaaaactctgaaacagtccacacccgtcccaaagttgagagaaacctgg
acgtt

>chr9_136008779_136008979
atgcacccctcctcccacccacgtggtccctggccctgcctctgccgccagcctgggggagagca
gggtctttgttccctgctggccaggccgccacgtgatgcgccacatcactgtcactgcctggaac
gaagactgccatggacagccgaggcgctggggcctgggggtgaggccagttggaggagctccacc
tgcag

>chr8_95448824_95449024
gattgggtctctttttgctgcaatagctcctgtttggcatattctttgagccttttataaagggt
gcgtaggccctgtgctgttcgaggagggcggtctactccaattgcattatagttaactgctatga
tatcccaacatctattcttttctactattactgaatgtttattagtgtgttcttcgagaattttc
acata

>chr8_95449224_95449424
gggagggtttattaaaattcctccagcttaagctgacgcaggttcaggaaatctgcttaagccaa
gcctgacctacataaagcttctcactgaagaagacaggaaggcacaagcaaaacacacttgtgta
taaagggaacagctcgagacaggatttaatacacaaaggtctagagataagcatggaacacagct
aaaaa

>chr12_6444539_6444739
agcttggatgactgtcaggtcagagctgagggcgtgctggggagtcctggagccccctgcacacc
atggcattctggaaggtgtgctttgcagtctcaggccaaccccacagcagctaaatggcttgtcc
tgttccggcatcagtgccaggaaagcaaagccaggagtgaatatttagctctaacttggactttg
atctc

>chr12_6446939_6447139
aaaaaatgtcttggtcagaaagtgtcttatcctcccctggtgtcctctcagctcccagaaccaga
cctccagactgggagcagataaatatggatcaactgatcgatctaatgatacatctgtcttccac
tctagtagaagccagtgattcccaaagagggaggtgaggagacagcacccctggtaggaatatat
ttatc

>chr12_45628533_45628733
cacttcctagctcagctacattccagtctctggaaccaattctgactccaggcaggcaagatgag
acaattcagcatttactatcagtccagcagtaacctttcagttctaataatttggcttgtccttt
tcctcctcagttgttcagactcctttctcagaaacatgcctaggacctcagtctctttagttctt
tggat

>chr12_45629533_45629733
gagtccaaaaagttgagaactactcctggctttttccttccctccctcacactgacggcattcct
gaactagcatgcctgtctgtctctacatttctgggtgactgcctcatcccaggtggccttcctcc
agctgtgagctgggtgtgttcccaggtatttaatccaccccttggggcaagttcttccaggcagcc
tccag

>chr12_56520333_56520533
ataggtcaccctctccattcccccattctcaggaacctccttgcctactccagtgcttggcaaat
ttcagtttatagagaggtttctctctaaggttcatccatgttccttctgtagttctgctttatca
gttggtgagcaggtcactcataattcctccaatttccttgtttcccttttctacctgcttacctc
tggga

>chr12_56694733_56694933
tgctttgtgagctgtcagttctgtgtgtaatggcaggaaatccaagtcttaactgcgcccttact
atgaaggctttccgagccagccacagcttctgggtcatgggcattagtctcactgccactctggt
cacgtaaccccacacagcagctcaccccactttagccacagattaggagccgcagactagcccct
catcg

>chr12_76337733_76337933
ctgctttgttgaactctgtgaaggaaatcataggatgtaaatatagctccatctagacagacaag
aagacatgagataccagtggtgggatgtctgagattctgaggagttagaaatgaaggagaggaaa
tttttataccctctctctggtccctggtcttatatttatgaaattcctactttcagcaaagaaaa
aagtc

>chr12_109083271_109083471
ggattcagactgctttctacacggtttcccttatctaatccaactcaggcagctgtctcacttcc
agtacttttccatctcctgatctgcagtcctctatgcctattatccagactggttacaccacccg
aagatgtaagggcctaagagcccccaccccatcctggggaaggcaaacaaagatactttacatga
cctga

>chr12_109084671_109084871
aaactggttgtctaagccctcgtagtaagcaaatgacattggagattgatctactgaaaccctct
gtgaacgctgaagagctccagggctaacccttcccattgcagctctgcattggtttaatccacag
gtctcagaatgaaaatccaccctccttggatggtggagatgataaatagaaaaatattacaactt
ttttt

>chr12_109084871_109085071
ggggggtgggagggggctaatgccttgggctccatggctgagtcagttccacacaagcaagaggac
tgcagtatagactcaattcagtcagccagaaatgaaacccaagtcaccgttcctccaagaaagca
gacatgtgtcccatcacatgccctctcagctcattcttccccagactcaactcaggtcccacccc
taatg

>chr12_109086471_109086671
ctgacagaatgaaagccctgtcaagttactgtttttgttattaaactctctaatcccagagaaag
aatgcaaaggattcagtgtccattagaccacagctttttttttttttttttttttgagacagagt
cttgctctgtcgcccaggctggagtgcaatggtgctatctcagctcactgcaacctctgcctcct
gtgtt

>chr12_118558417_118558617
gaatttgggggaaataaaggattactagggaaatgaatgggtcaggaactagaagagaaattaac
ttgtaaatagatgtctttgaaatgtgaatgagcctgacattatcttatgaaaggatctgttcagg
tgtggttaccttctgtgtcttcctttctgcaataaagagataacagagagggaaagaaagaacaa
ttgtt

>chr11_355400_355600
gggaacagatggctcagaacacaggaagttaatttaactgcagtccagggaggatggcattctgg
aagccgagtcggcctgaggtctaaggggcggagggaactccaagcttggcctggagctcagggaa
gggagctcgcgtggacttcaggagcccggtgggtgcttcctgtgcacaaacgggcagggtctgag
ctccc

>chr11_355800_356000
aaccgcttctgcggcggcttcccaagccccagctcccgcagaaggcctggagggggcccggggacc
cgtgccacgcagtgagtggcggcaggacacgagcccgggggcgcagcttcctggacactctgccc
ccagacactggctgccacgcgggacggtgaccagcgctgccctcggcgcccgggaggcctcccag
ggctg

>chr11_6628024_6628224
cttttgccccccgagtttggcctgccctggcgccacaggcagtactggaagctgataagtcaggg
cacaaattaaaggtctaggaggcaagaccaactgataaacagggggcgtaatgcttcctggggat
aatcccatctcaaggaaaagcttttggctatttctgtgttagcagatgcgagcaagttgtggagg
aaaat

>chr11_6628424_6628624
tttccttttatggccccaggctgtcaggcaggcccagctccaaaccaattgttttgttttgtttg
tatactttactttataaaatacatatttcttggaaaagaaaaaaatcaggtatagttttaatccc
aaaacagccattctcattttcttctgttttctatgcacaggattgctaagtaggggaatcctgct
ttgca

>chr11_6628624_6628824
tggtggtttgttatctacctcatcagtagtcaaaagcatttctccttttcaacataaacccttcc
aaaacatcatttaaattccattccatttttagatgttagtttcagtaccagagccaaatagtaag

tttgttccctcttctataagttgtggcctgccttctccaagacacccttctcccccactaacatg
cactg

>chr11_6628824_6629024
aacagctgctgtcaatccattccctcaagaaacttaaaggtctaacagacaagacagtccattaa
cagatttttacaatccagtgcaacaagtgctgaagtaggggggacatataagatgtggtggaaaac
agaaaggaccaatagatgttgcagaaggggtgcaagagaaaccaggggaagagcactaccaccta
aggaa

>chr11_47429024_47429224
gggaggctgatgagggtcagagacttaagagacctgtactcagtcgctatctctgcccaagtgag
tacccacatgccccttcctgaatcttctcctgtactggctgatttccagagaagaatacaaactc
gaaacgccaagccagactcccactgctgttcaggacctagacgtgagaagtggtatcacatactc
tggct

>chr11_62321024_62321224
tcaggctgagcagggaaggaagggcctaggctgcccagagcctgagtcaccacatagttgaggtt
cctgtcactcgaagtcccgcctctgttacactccgtgccctgtgcccgccccaggacttggatcg
ctacatctgaccctggccccagggctggctccacttccacgctccttaccaagctacccttacca
agcta

>chr11_62324024_62324224
cccacccccatctagaggccaacggcccagccttcctggggccacaaccctctcgtcttgaggct
cctcctcctgcttgtgggtactggccctagcccaagtgcaagcactgcttcaaacaacaacctgg
ccacccttactctgcaccgcgtggcgagggctggaggcaggatgtgcagatgactcactcttcct
gcagg

>chr11_65186824_65187024
cagctccagaactagacccgtggagaatggagggctggggccctgcacattctcaagcagtagta
gcaacactgtcattgtcctgcccttggtataagcctgaagaaccaccctttcttacccggcttct
gctccccttttccaggggctcctgtgggaaatcccgggtacttcctgggactggtctgtctatat
gtttg

>chr11_65420624_65420824
aaagcatcggtgggccgggtagatctttacctggcaccacacattccccaagagaagcacacact
ccctgggccacccaaggaatttggccccatcacatctttcgaacatcgtttctcttacctctgac
gctggctagtccctctttgacatccactctcagctgatctaagacctggagctaatggggcctct
tggaa

>chr11_65420824_65421024
gactcctgaaactgctccatccccacagccagcgggctctactaggctctcagtgtgctaggact
cttgccttagctgcccgttttcatccactggacggtaactcggggtttccaagggcattctcaga
ctgcaagctctcagaggacagtgcacgcattcacccagctctctaccaccaatgtctggcctgat
gtgac

>chr11_67055624_67055824
gaagccttcgggctgctaccaaccccaggacctcacagattgacaccatagcgtggggtggcaaa
gtgaccccagatggcacaggcgccatgtgactacagggagaaggcagggtaaaaatggcaatcag
gccttccagcccagagtctcagaactgaggacgccctacagcaggaagtgatgctcaagctagct
ggccc

>chr11_71750952_71751152
ccgagatctcacctggcaggttacccagaggggccaagccaccttgccctaggctaataagctat
aaaaacagcataggaaatactttgctggtacatcccctgcctctgagaagataaaaggcaagagg
accagtggctccaagagacgccatacagagaaaaacaatctttctcaattcctggggagtcctgg
gtaga

>chr11_118783590_118783790
tggcctctggctcagggagatacagcgcccgagacgacagggtcccaaatcccctttctcctggg
tgtcaggacccctcagtcctcagaagctctctccaccactcaggtcacttcctcttcctgaggtg
gtgactcagcgggcagggaggtcaaggattcgtagcccagcaggaaatctgctcaaggaccaagg
tgctg

>chr10_74081194_74081394
tcctcccaaaagagtcagagacccctgcaccctcagccgaccccaggcatgacagaccccgcctc
atcccacagtgcacaaggagatcacactcagatctgggccccagtctggcgtctgccccaggcca
gctgcatgaccttgggcaggtgattgaatgtttttgggtctccatttttccacttgtgaaatggg
gatag

>chr10_74081394_74081594
cgatgcctactttgtaaagctgttgtgaggctggggaaaatgagaacgatatcaagtgttcaca
gtagaggccagcactgttgtgagcggcagctcctctccctcccctcccctccccctttccctatc
acaggtggggccaggtggaatgtcacagtaagtccttgggatagaaaggtctactgacatattca
aagtg

>chr10_103911410_103911610
gtgaactctgatcccggaggaagaagggttattttcaacaatctcttcagcttgaatagattgtt
gctgtgtgtgattcaggtttggtgcatgacacctttcctcttcatggagctgacctaaggcggaa
tataagggagatgggaggggcagcttgggaaatcaacacctcccaccctcacgacggataacccg
aagga

>chr10_105668210_105668410
caggaagcacccccagcacaggcctgcggtgagtcaggggaagcagaacccagaacagcagggag
cggccacctggcagatggcccaaaagccatattgtgcctgaggacagcaaggagaggggcagttc
tccacaagaattaaagttgcaaaagcctctctactctcaataatttcagcagtcagatgcttttt
atttt

>chr10_105668410_105668610
cccaaagcccacagatgtttcatgctcctcagacactcactcagagggaaatgcttactggagaa
gaggcaatcatagtgccttaatctttttataatgggaaaagaggttaagtgaagctcctttagac
acaggcttaatatctagcctggttctagggccaggattttaaagtcctcaaacagtctcatttat
ttgat

>chr17_27073273_27073473
gctgggtgagccattgctctgcctgagccatgccaggcatgtgtgactggccctggctggggtca
gcaagggcatgtcgacagaaagaagcatgccaacaggctccttgggtgctgacggccaggtaggg
tttggtgcaggagaaggcgggcagctagcaggaggggacggtcaccaggcttcagaagcccactg
agcag

>chr17_38267474_38267674
agcttctgaaataggtgctttccctctctcccactataagccacagtgtttaggatgaccactgc
ttccagattgtgtgatttgccctggcaaggaaatcctattgtgggggggaggaaatgaggcagtcg
tgacacaaagtgatggatgagccttttgcttagcagagagagagagagaaagccggcaggctgtgca
gaact

>chr17_41560874_41561074
taaaacaagagaaggcaatctactgcctactggcttgtcaagggtcacttgagatcggacgacag
gaaccacaaagaagtgaggccgggaatttgcggatccacagtaaagcaagtttgtctttttccag
acgtgcattaagattccttttttcaagtgttctaaagcgcaggcgcaggctgttttgcggttgcct
cccgg

>chr17_57914618_57914818
cattaggcagtgttgcaagtacatatcggaatctctttggctggctctaagaaagagtttgaact
tatttacctccttagccctatgtaacaggtaagaaactaaaaggtacagaaaatagagatgtttg
atttttctaagttgccccaagctaccgtttttaaaaacgcctgcaagcatgtctaaaacaggagc
ctgtt

>chr17_57923818_57924018
tgactcagaggacctagagggagggcttgaacacactccagcactgtttctacaatttagccttt
atttgcattggaaaccacattcctgaattcttgagggggcaggctctggcttattctgggcaact
gacttcaagtgggacccctgagctacctatgtaagcaaagagtcagccatctctggagagttacc
aggga

>chr17_57924218_57924418
gaagcagtgatcaacattgcttaattgtgttccagtctgtttccagcggggaaaaaatgctttag
gggagtgaggcagttagggatttgaaaacagggatggtgccgcctgaaggtggggaatgacttag

atcactgtcttcctttggctcctcaccatgttgtgtggaggaaacgaaaagcagctcaagttcac
tcctc

>chr16_21513899_21514099
ccctggggtgcgcgcgcacacaggcgcgcccttggattcacgtgtgccagttttcccactttcta
cggggcggggtggaaagtgaaaatagtcagatcgacaggtggatatccaccttcccaacctccac
ctgaaatgtgcccatcgagtcctagcaccttttaccatttcttcccaatgaaaaaaactaaacga
tggaa

>chr16_29607099_29607299
aaaatagtaagatcgagaggtggatatccaccttcccaacctccacctgaaatgtgcccatcgag
tcctagcaccttttaccatttcttcccaatgaaaaaaactaaacgatgtaagggaagggagtacc
cacgaccaccaaaccctgtcctctgcaatgtgaaatgtgctttgaagtcctctcaccctgaggct
tgccc

>chr15_58624308_58624508
cagaagcctgcagccttccggggattggctccctgcgaagataaccctgtccttcaacaaaccct
cgcttcctatttgcattcctaattggcatcttctattgcttttcctggtgacttcatttttcact
cttggctaaaaatgggtctctgatgatttattctatcctgggtgttgacaagctgaagaagttgt
gtggg

>chr15_89181396_89181596
atctggggtaggaggcaagcgtgggtaacgctcagcttattgttggatctcaccacagtgaaaga
caagcacttccctcattgaaagttgatttcttttttgccccagttgtaactcatttgacattcac
taaataccaagccctccctatctggcaggcactgttctaagagctcaaagtcctagcctgggaga
gtgtg

>chr15_93460796_93460996
caaacaagcaaaaagaaaaaaaaaggtgtgaccagtcagtcttctttgcttttccatcttaagaca
catagaagtgactgagaaggaatagttatgcaatgacatcggtttaataatcagttcaactgaag
aatgtatgtaaataagggcagtgatacctaacgctgatagttgatagtgtggattgcataattca
gactg

>chr15_93460996_93461196
tggggtgatatcttacacacgcacatacacacacacactcttgggctttgtcttctttataggta
aggattaggagaattttgacatatgcctgacacaaaagtgctccgtaaatattagctattgtatc
actggcaggaagttagcctggaaataagttttatggaattccagtgtagattgctttgtgattgt
tttgc

>chr14_23789760_23789960
gttttgagactttacctcgccagcaaagggggggccagtctgttagcggtgcagattggaggggtg
acattggaagctgtccaggaaaaagaaaatggaactggggagcagaaggcctacgcaagagggcg
ggacagacaggacttgtgactagtagctctggactgaggaatcctccctgctttctggtgcggga
gagct

>chr14_55570247_55570447
taaaggtgaatgcagccccagtgccagcacataaggccagtcagtgctatacagggtcagcttag
ccctgggctgacccttaagatcaggaaaaagttatggggcagtgacttctcagcaagtcttttcc
atgtttctggtgtcttcgaattcctagctgaacagaatttatcaaagcagaaggtagctgtttat
acccc

>chr14_55570447_55570647
agtttacccaggcaaagagaaatgctttggaattgagatattttcagggcatgagtggtttagat
ctctgaaactggaagacgaaaatccaggttattagagatcagcagcacctgagaggcaggcttcc
tggcacctgaagactcacagacaagccttggtagcctggcttgttttccttgcagttcaaaacca
tttct

>chr14_75725447_75725647
cggggaggtcaccctgacaattatggccaactgacggtggagggagtggtctcggtttggccctc
agtctggagtcacagtctggattcacaggcagcgcagagagatgttggctcaggacctgaggctg
aggcgtgctgggacagcctagggggtgtcctggctttccaggggctggggaaccctctctggccc
ggggg

>chr19_1270600_1270800
tactaaaacaaatttaaaaaaattatccaggcgtggtggtgtacacctacagtcccagtttcttg
ggaggctgaggtaggaggattgcttgagcccaggaggtggaggctgcagtgacccctgcactcca
gtctgggtgacagttagacacactgcccccgaccccaaaaaaataagtcctaggtatgagtttga
gataa

>chr19_1415400_1415600
ttttttttttgagaactgcttgaactttaattccaaaggattgtgtaaatgagcatttttccggt
cggttccagccaggcttgggggcccttccttcatggggtgggggctgctgctggctcctgctggg
tgccgggccctgctggcagggatgtgggcggggctggccctgactcagcgcgggcacagcctatg
gagag

>chr19_3983600_3983800
aaagcctccagagactccgaccctcccctcctcacttctgcccctgctccaggggaaatgaagag
ccttagccagagtccctcaccaatgtaccaacccaggacaaaaagcagttggggtcaccgtactc
tgcgctcccccctactcatatcggggccagaaactgacatggaatgaacatcatccagtgcaacac
aggag

>chr19_5968200_5968400
cctctctacacggaagcttacaggaaggaaatccaagaactacagaatttatgctgaggcagcag
agcaaaactgatgatactggagagcgcgggctctggtctttgtgtgtgttcaaacctgccctggc
cacttacttgcggttgggatcccagataagacaatgtacctctggcacaatgtcttcatttgcat
atcag

>chr19_5968400_5968600
ggctgttatttcaccccaggcagcttcggcaaggatgaaatgaaatgctgcacatgccgtgtttg
gggaagtggttggcacacaggatgattccacactggcactggtagcattattatgctcggaagat
tgcaaacatattgatgccaccttaaagcataggccttggattcaaggacagctgccagtgcccgg
caacg

>chr19_6738000_6738200
gacggacttcctcttcttgagccagggatgtggctcccagccagagctgaggcttttggtaaaca
cacgcatctgttcaggcctgacctgttttcctaatcttcctggtccacagagaggcctaaagctg
gcctcaggttaaagtttgaccctaggatgacctatgaccccaggctgagaccccagccttaggct
aagcc

>chr19_6738400_6738600
ctgactccagattggacctgaccccttaccgggttgctaatccttctggttaagcctctggcctc
tatcccctatccccacggtggtgctgacctggactgaaccccaattccagtttgagtctctgagc
agaggtgaactcagaactttgctaggctgagcccccatttgagactagccacagaactcactggg
ttcaa

>chr19_6738800_6739000
tctgaggctgcagagaaagcgccaaagcccggagagaatgggagtgggaatccccagtgcctcct
ctcctctttcattccagcacctcaaagggatttacaaggtgtgaacagacttttatgggagaggt
gacagacttcccttctccagggcccagatggcccagatgtctgggggaggggcggagtcggaatc
tcagg

>chr19_8066800_8067000
cctcaagcggttcagccattacggggccacctcagtactgcaacagcctgtgactggtttctcct
tgtctccccccagcctctccaccagggtgagcagtgaactaaaactgctcagtagccacccctgg
cctacctgcagggtaacagccaagccctctgcccagtggacacctgagcactcctgccaccacta
cccat

>chr19_8067200_8067400
ccctttttctgggcttccacatcagccagggcataattctcacagcactcatcagagtgggctgag
atgacctacacacatcatgcacactctccagagcctagcgggggcccaggacacagcagcggagc
agggagagtcgttcagaggagggagcgagcctcacttccctcccacaggctggcttccgttggtc
ttccc

>chr19_11253600_11253800
ccgtctcttccttggggaaactgaggcccaggttgctggggtggaggggatgtggagtgacaggg
cagggcaatggctcctcggccccctccgggaacaaagccaggtcattcctgtggggagggagcgc

caaccccagggttgggggggcacgggccctgggtcaggggtacagataagcctgggctcccaga
accct

>chr19_11253800_11254000
tgtcacagcggcacccctgccccatgcctgctttctctccagggcactcagagctgcagtttgca
ggacgagagggagacgtgcccaatccccccatcccaggacgctcagccgtgcgtgggctgggccc
actccactcccggcgcccacagttgccccagtgaaccggccagtggaggcatcctgtcccgtgtg
cggga

>chr19_11254000_11254200
aaggaatgaccgccaaggtgacagcagcgcttagccaaccctggcgctgacacctgacttgcaaa
ctgctccctgcctgaggcttcaaggcaggcgagaggctttgtgtgtgtgacttcctccccgaagg
gacactggcaacagctggagacatttcctgttgttaaggctgagggggagctgctggcatttggt
gggtg

>chr19_13275800_13276000
tacaccaggatctccagggcatggacagggatggggtcggggaacccaggcatcctaggtgaatc
caaggaacagccatgtctgcaaaccactgttctagctgccttgctgggcttacctaggggccacg
atccctgagtcagttcccttggcagcctgggtctcagtttcccttggcttgtagaagttctttca
ggggc

>chr19_13957400_13957600
gcccacaggtcaccccagctcacatgcagggctgcctgctgggccccatggcaacgccacacaca
gagacatcaaacaggccctgccgaggaagtcccacgtcactgcggttagagtggctcctcccagc
ccagcccccggctggcggcagtgacccccatgccagtcagggcccctgcctcctgttgccctggc
gacca

>chr19_13957600_13957800
gctgtgttatggactgagccgggatcccaccacatccctgccccctcaccagggcaccagccact
ccccacatccggagaaggacattacacggtggccccagctcaccgtgacaaactcaattgcctgg
tgtctggagcaccaggaaagactttgtctacctacataatggttcagaagggcagagaggtccca
gggat

>chr19_18393200_18393400
ggctcagagaggtggagtgaccatcccccgagagttagctaggagtcacagcagggcccgggctg
tgactaggcctgagctcttgtccacccttagaaccctagaacctgggagtccaggcccctgcgca
gccccttccttccgccgcccacgttttaaccccaggtctgccagggctgtgatgtcctgggcgag
ggtgc

>chr19_18484400_18484600
cctacaggaaataggatttctgtactgggcatttgaaccccattcccttgtttctgctgaccccc
agcagtctcaatatcccctacttcctcttcccagaaccaccctccccaaagcccttatcgatgct
tggaggattcctatgcatccctcaaaatccaaggtttgggaaagatgctgcagtggtctcaagac
atcct

>chr19_18484600_18484800
cagtgtgatgacagcctggggtgggggtaagggggcaaagtacagcaccagaaatcccaactgag
tccccccacacgtcttccaccttcccaccctctctttcgggttctggctccctgcctccaggcaa
tcaccagtttttaccaggagtgcctcagacctgcccggacatgtccaagtcccctagaagcaggg
agggg

>chr19_18485200_18485400
agtagaattatgatgggagcgagcgtgtgaactgggttgctaatgtggagcgtgaagagggaggc
ccagggttactcggccaatgggttccgggcagcaagcacgtgggggaagcctcagctgaccactt
cctggccagtttatgcaggaactcgcacagccggtccctgaggctgagcccaccacttccgcccc
ccatg

>chr19_34662560_34662760
ggatatattgtgtatacataacggagaggtgtagtcagaacagatgtggaagcaagacagggcaa
ggctgactcacggcttttagtctgagcagctgagtaaaaatggtaccgtttacatcaaataggga
acggtggtgaaggagcaggttagaggggaaacagggagcatggctttggacccttaacgtttgag
attcg

>chr19_39156360_39156560
cagagttacaggccctgaacaattttgtgttgggatgtgccataggcagtgccaggaaggttctt
tcatgagtaatttaacttgttactaagtttgctatcagccgggggctctttccccgggttccct
tctttccctgggtccccttccccgcaaagcaaagtccaactcaagttagaaacatcccacagcct
ggagt

>chr19_39173560_39173760
agtcacattactccgtgttatggtacccatccctgtctccttagcttgtttttgtctgtattggc
tcttccactagactgtaagttgcatgagggcagggatgtctgtttaatcccagtgctcaggatag
tgtatggctcgtgatagatgcctagtacattttaaaatgagaacgaatgaagtttgggagaggct
cagag

>chr19_39173760_39173960
cagtgagtctccccctgttgggggactggggagtgcctgggaggggctatctggtgccagcggt
ttggagtggctgggatgactctggaatcctgtgaggcccagtcagtttctttggttctcatgcag
tgcagtgccctcagacctaaccattttgttccgtgctggcttgaaagggtctgcctccctcccag
tgcag

>chr19_39174960_39175160
ggtacccagagtcctcctcggtcctcttcccatggtgtagtgagagcactcaggaccacctaggc
ctttcctagaaaactgaacccacaccttcccagtgctgccccaccctggtccccaccccctgca
ggacaaaccactcctcccttgttttggggccaggagtcagatctgcccctgagagcagcaggggc
ccctt

>chr19_39175360_39175560
ctctttggctatagaatcctattttgatctcttcttttcttttctcttctttctctctctgtggt
catggtcaggtttttcttttttttaaatcctcccaagacactgctaatgttgtctgtctcatgcat
ccaaggaatctgagatggactgaatattgtcaagggaaaaaaaaagagacccccaaatccagagt
gattt

>chr19_39888760_39888960
gttaatgtgtttctccccatggtcctatttctctcactctgacctctctctcttagtcccctta
gctgtcttctatccccagctcctaactgggactctgtgtctatgcaggggccagcacccctggg
ttatctggggctaagggaagggacttcatttccaggggccacagccaagcccagagtcccccagc
ggctc

>chr19_45959160_45959360
ctgggagaaaccccagccacatacctggccgctgacatcacccggccagggcacccccggcagcc
tagacaagctgactgaatcacaggcggaattcagccaccccgggcacgtggcctgctgtgacccc
ccgcaacacccccgagtggccgtctggctgcgggggttgggccgggcacacaggggtcagtgagg
gggca

>chr19_45959360_45959560
tggggcctgagtcagggacagggtggctacagccagagaccacccagccacaggcgtccatgtgg
ggcaggaaggagaaagtttgggaaggagagcctgtggggaggccctggcgggtgaggaggaagca
cgtgtgggtgtgacggggaggctgcggcttgtgggcagcggctgggcgacccacaggggtgggat
ggggt

>chr19_47611560_47611760
tagaatctggtttgacttgatagctatccacggagacctggaaatagtttcattagcaattttca
ccagaggtatttactgtaactggatctgacctctatggagagatgtttctacaggcccaatagcc
ataaaaatgtaacaaaaggaagtgaaggaccctcagacagccatttctctcccatcagggaagaa
ggtaa

>chr19_47612760_47612960
ttcaaataagtgcaattcattttgggctgacccagtgccctggataatgtgttttgttcggttcg
tgcttcagacatttcttgcaggatatcgtagtttggagatttcttcctcccgtaactgattttac
atacctaggatcaccactctacggcagttgtgaacatattaagaggaagaaaacaagtggcctat
tgaat

>chr22_30591800_30592000
ctgtctggaaatataaaggctcacattggctttggccaccaaagagaggaaacattctgcaatcc
ttaaaaactcatggccgaagggggtggggagaactgtttgagccttgtacttacaaagtcaaaag

ggaaaacatgtgtttcacatttgtcttgggatgggaaagaaaaacttttcctttcacttaatctt
ggttt

>chr22_30592200_30592400
tcaggtttccaaacctataacccctggtttcagccggagacttgaggcaatacagcttcagtcat
gactcaggattttgcgagtgaaagcaagcacgtcaagtgatgtaagaaaattcccccattcctcc
aacccactggcttcctcctccctggagaattcagtcagacagcattgtgttgggggtgggggaga
aacag

>chr22_35772800_35773000
gtggtcaggacaccgtctgtgaccttatttactggggacgtcccctgcttgaggagaatatccag
gcaaggtctcctcactctccttagccaccatccagcttggccaagaggcctccccatgattcctc
ccacgccagggcctcgcctttgtttttcactgttaggggctggcgagtcactgacccgcccccct
ccctg

>chr22_35773200_35773400
agctttaatggtaggcaggaggaagtgaaacttctagaaaacggcagaagcctcttgtttgtctt
ttctaagtctgtgcctttcccctgctgggtcccccagctcccaccctgtgaagtccacctcaatc
tgcccctgatttaatgttgcaatccaccgaggccttcttatgcttcacatctctctgacttctgc
atctg

>chr22_36725054_36725254
cttctgcccactggtcattcaggagggaaaaggaagcccagacagaaagaagaaaaaacaaacct
cacacccaacaccaatggcagctgcccctgccctgggccgcagcttcctcccagcccccctaagca
tcaacggggtgggggcgcagaggggctgggtcactctcattcacaaacacaccccacaccctgct
ctctg

>chr22_36725254_36725454
atgtggaaactactcaggaggcaagctctaggggaggcaagccttactccccccttgcctttctca
aaaaacacgagtccttcaagcctgcacagcgaggacagagattagagacctgcatcctcgactag
acagacgggaagacagaagggagggaggcggcaggaaccacagcctggggaagacagagcaattt
cattg

>chr22_36725654_36725854
taaaaggaaacattccccaacccttccagaggaaccgttttcccagggctgtccctaggatggct
ggaattcctctaattgtgtgaggaggagtcagaggctctgctgctgaacaacaggaagcagagcc
cccaaacacactcgagggcatgcacacagaggcgcacgcacgcacaagggcatggacacacacat
agaca

>chr22_36725854_36726054
cgcaagcatctgtgcatacacagtcacatatggaggtaggtgtacagaggtacgtgtgcacagaa
atacatgcgcacacacgtgtgcaaagccatatacatagatccacacaaggcacacagctgcacac
gcattcacagatgcatatccacaagcacaagtgcaaacacaggcacgcaaggcacatgtattcac
aggtg

>chr22_36726254_36726454
taccttaaagaaatctggatttgttctgccaggaacattcatctggacaaatgctttctgaaagg
caaactgctgggaggcctcctcattgcatctcttaccgcactgcttagtatcctaaatccaccgg
ccggatgcaccacgaatcgtaccatggccccatgcaaccgaaaaggtctttccaatgctcaggaa
gggtt

>chr20_30307939_30308139
gttatctgacctctattgacttgacccccttgccttttacaagagctggcacagacgagttgaaa
ttgcaaagaaatgcaaaatgagagaggggggtggggttccagggcaaactcaggaggtctcttgct
cagtggtaaagtgaagtgcctgccacaccctaggcccagcctatagacagttgcattcttgacat
tcctg

>chr20_45946393_45946593
gagtagggcaaccaaccatgtgcttagctgggtcccgggcaaaccaggacacataggtcaaggca
tatgctctcatgggccaggttgcaaacgacagagaggcaggatgccacagtggtggggaatcagc
tttctggcgtcaaaatgcatgcagaacctggctgtgctctcccctgcagtgtgaccgtaaaaagg
caact

>chr20_45946993_45947193
cagaagcagaggcctgctctacagcccttttcgtaatcaccagtcctccccttggcctacttcaa
acatgaccacacactgctgctcccccagaagcagcaaagcagagcatggttaatcacttcaaagc
gtttcccaaagacacagagcaggtacccaaacaggcctcccttccccaggaatatttggggccct
cccca

>chr20_45947193_45947393
accagtaattacatcataaagagacaaaagtccggaatttcctcctggcagagcatagaagtaag
aaatcaggagcaaaggttgggggtggggaggaagcaaaattgtcctcgcagcagagaatgcctaa
gccaggaggaacttgtaaatgaaaaccgctcgcattagcaaatgattatttcctccaacgctgac
cgtct

>chr20_45947393_45947593
cttagacccacagaaactcgaggaccggtctgcaccctggctgacttgggcctgccagagctgcc
gggctgagccctctcctccagaggactgcactggcctgaactgtgcttataaagaatctctgcaa
atcaacaggaacttctcatccagagccgggatagggagggctggaatggaaatgaggctgtggaa
tgaaa

>chr20_45978393_45978593
acagttaacttcaaaggaacaaatagcaaccttgatcttttctagcaactagaattctgtttccc
tctctagcatgaagtatgtgaaattctaaacttcttgaaaccgatcccatattttgagtaataga
gaaaacgctaatctgtgatccaactgaaatctacagtggtgagggaaacaattatgcagaactga
gccaa

>chr20_45987793_45987993
ctgtttgtttctgcgggtgctgctttgaggggaacaaacaggactgtggggttggtggcagatct
cccatgcagctaagtccacgaaccaatgaggaaataaaatatgccgctggtgtgcgtggtggatt
ttaacctgggattaaagcttgttgcataagcagctacagcatcctccccgcctccctccctctct
ctgat

>chr20_48782193_48782393
tgattttgactcccaggggacatttggcaatgtctggagaaatttttggttgtcacaactggagg
tggtgatttactggcatctattgggtagaagccacggatgctgctaaacatcctacagaggacgg
ttcccccgctgcctgacaacaaagaatgacccagtccaggatgtcaatggggccaaggttagaaa
tcctg

>chr20_48782393_48782593
acctgtaagtaaactgaagaggcatgcctttttctgttgcaatggagctcccgtggatctacaat
tcaccccagcaattccatttccccccacctatgtcacaaaatcccacctctgtaggcacagggag
gtccagccaagtgtgtttaggatcgctggaaactggaaccaacctgcatgcccgttaggaggaga
cagat

>chr20_49434793_49434993
aagagttttgcagcctggctgacctggtgagtggctgcctggaaggcgtgggtttaggcccaggc
cagacttcaggcccttgctggagttttctgcggaagccattttggtggtgagttagcaaccagga
ggaatggggcagcggccagcgctgagaggaaagccacgtgcaaacactccctcctctctctctct
ctcgc

>chr21_45148172_45148372
ttgatcagggtgaagcagtcacatgatctctcctgtcctctttcctcattctggtttcctaaagt
ggtcagaggtctctgcaaaataagctaaagggagggttctgctgggtgggtggcttcggcttctg
tgcagcgctcaccctgctctcccctggaacgcctggaactagggtggtcaggctggggtggagga
gctcc

>chr7_44679875_44680075
ttattaataccctgcctggtgactgctgtgtgtaaattctgcctgagcccacatctctccagtgg
gaatatgattgacattccaggtgctctaagtaagttggaaaagcccacagagagcctcagtaaaa
gttagctatacctatccctgctgctattggtgggttttttttgttttgttttgttttactatttt
aattg

>chr7_100143064_100143264
acacatacaagtggcttcagctgccaaataattcagaatacttcatatgccctttaggtgccctg
tgaaaaagaaactgaagtggataaagacaacctgactggctggaggcttgagtcctagtaataat

gtactttccattggaaaaacttactctattctgagaaaataaattgatctctgttcctccccagc
acttc

>chr6_7146601_7146801
agcaggagatgggaggggaggaggcgaggtggggacggagaaggaagtgtgtgcacacgcagaca
gcctgggagggcagtgaggtggagtcactgcaactcggtgccagcaagtcaggagacattactgc
tggaagtatccacagttagctctgacttaggggacgtcttgtggccataaatgtggaggaaccga
gctct

>chr6_7147401_7147601
gactgccagggcaagacatgcagcttctgcagaactcatggcagccgttttccacttggccgagc
tgggtctgtgaagcagagaggaatcagtaataggaaagaaatgtagttgtttttttcccccttag
aatacctaccatactgcatttcagcttggagtgcgcagcatgaggcatttgtggttcagaaaaga
ggtct

>chr6_34625222_34625422
ttcacacctttccaacttatcccacagtcaaagcttatttgtatgtgtattatacaggtaattca
gccagagaaagattaagaaacatttttaaaccccaaactgaaactgaaagcagcgaactggaatc
ctggcaggaagctggagaggatgacaaagcaaattcatttcccaagtacaaggcagaaagtggac
tttag

>chr6_34625422_34625622
cccgagtgatctaacccatcaagcaggtacacagctacaaatgtggggtacacagcttctcagag
ttcagagaatttaaattactggcccactaggctgagcaacagcaggcatagaccaaacagctcca
attcagtgtcagaaagtagacatgatagcaggtattcctgttcaaaacaacatactcagctatta
ttagc

>chr5_180668794_180668994
gaagagccaagtgacagagaatcccttcagaattgcaggacatgtcctcactcccacttggggat
tggctgacaatgccatctgtcatcaccaggaccctctaattccacctgccaattacctgagcttt
caaggtaaaacatgttcaaactgctgcccactcagaacttttgcacctggcaaaaatgttcagcc
acact

>chr3_141086910_141087110
ttccgcatctctttcaataccactgcaactccttgagtaaggtgggcaccacacagaagaagggg
gatggcagaggggcagtgagaggtccccagggaaacaccctcttcttcttccctgcttctctgac
agagcacggccatgcctctgccatgcaggagggtgatgtcctttggggaggccttgttgaatctg
tctgg

>chr3_156807106_156807306
gtttcttggcagcacgttggggacactgcgccatgagaacagagccacagaatgttaaaggcaca
gcctccttcgagggcatttgcataatgcctcccgtttacagatgaggaaattgaggcccaaagag
gtgatgggaaaggcccaaagctgcatttctggcttctggcagctgggaccagatccagctcgtat
tgaca

>chr3_171858506_171858706
catgaggctgggttacgtcaataccaaactatgttgctttcctaggaggttttaagtccctctca
agggaggtactcagggtatatgctgcttcctgcctttctccttcttcctgtctcagttatcttga
tttttatgtttattcttatatttacataagaacggaatgttaaacccttagataaaactgtcacc
caatg

>chr3_177078106_177078306
tatcgatacatgcttcattttgccttcgggtgcacttataaatcatccttaagtgacctctattt
ctaccaaagacatccacttggtctttaagaagcattcttagtgatcctcacaaaggaaaaaaaaa
aaaaggagatatcttctgtgacaggaagccaaaggctgattttcttttttttttttttgagactc
ttgga

>chr2_26981096_26981296
gcctagtaggtctcagtcttattttacccagcccctattcaagatagagttgctctcgttcaaat
gcttctgacgattcctctgtatttttttcagaattcctgtctctttgggccctgataccttagcc
tggggctcactgctacctggaagcactaatgcaatcctagttttctcccctaaagcccattctag
ccagg

>chr2_43448296_43448496
ttggcctcgggcttcctctcccctgctgctgaggccttcagtcaggggctggcatctgggagaac
cttgaaactgtttcccatcccctttggcagcaagcaacaagcaccaaaacgctccagttaacacc
agtgcaatcatcgttaacgtgaaacagctgcccgaggcctggcgtatccaccagaccgccccctc
ccctc

>chr2_70312896_70313096
tctagactaaaaatgaacacttaattgtggtaaactattaataagattcaccggccaactgtcct
atgaaaaagtcagaggcactgtagtctcttcgtagctctcgttctctccagcacaacagcccatg
ctaggaagtctagaaaagcagtaacttatctcattccttaaattaaacagaggtttctaaatgct
ttggt

>chr2_70313296_70313496
cggaggtggaaggccccggggaggaagaggggtgcattctagagctttcgggccgaccccaattt
ctcgttggcgacgaatgctaaccacgtgtcgccattttgtgttcaggaaacatggcggccgcccc
aagggtaaggaacaggggaggcggagtagcgccacgttagccgctttccctgagagattgtgaag
cacgt

>chr2_70370496_70370696
ggctcagagtcatgcccaaggccgcgcataaaagagtggcagactggggtttcaaacccaggctc
taaactgtgaacctgcaagatggtgggagtggggaagacagtgaaaagttgtgcagggaattcac
agaactacagaacccactttgtacttctggaactgctgagtggggaactagttctaaggaacaag
gttag

>chr2_87785685_87785885
ggcgtctggccaggagcaggggctggggacagcaagtgtgaaaccagctgaagcacctgcagctc
aggcgggctgcaggctccctgctctcccccctgtgaagaagcacacattgttctttcttcccgcac
atttgaatctctcagtggggtctcattttctgtgacatggcaaacctcaaaaaatgggagtaaat
cattt

>chr1_10447813_10448013
tgcatagagggctgttcttgtagaacttgtgctcatgctttgatttgggatttggggagttaggg
caagccagaaagtttttctggtggataataatgtgggttgactttcttaagcattttaagccaag
cacttgagtttctaacaactaaaaagctaagtcagcctgacacagctctagcgcgccctggcttg
attct

>chr1_23882413_23882613
aagataaaatagtctggaacctggcaggcacttccataggtgattatgacagaagacactatcct
ctctgggagttatctgacctagggccaaattgctttcactctctaatgactaatggattggcatg
ctggtctttcttttgtcctggccacctgaacaactcagtcctgggagtcctgacaggcaatttcc
ttgga

>chr1_23882813_23883013
aacatgctgtggcagaaaagggtataagacgttgagctcaatgcctggctctactgcagttcatg
gtgtaatgtggggcctaggacctcagaaccgctgccttagaatcagcttcctttacagatgcaga
aactggggctcaagagaggcaaagtccctagcctgaagttatacaggtaagaggtgacagagcag
ggatt

>chr1_23883013_23883213
tatgtccctttatggtcgtctctaaagccagcacagcacacctaaaaaacaatccaagatagacc
caacccaggccttgtaggtggtttggggcaagggttcctcacatgaggattgaagtctgctattg
ctctgcactgtgtgtgtatttttttaagagagtgttcatatcttccttcagatcagagtgaccag
gacac

>chr1_27113013_27113213
agatttttgagagaatttgatgtggacagagggaaccctgagaatgcatgtctttttttccccttg
tgaatttacagagcaaggacagttcatttctcagttttgtctttgcagagagagtgctgcttgga
gcctcatggtggacttctagagattcaagtcacagccttaggccccagtcacaagtgtgtcccca
ggctt

>chr1_28835213_28835413
ctacagcttcccagagtcctgtggacaatgactggggagacaaaccatgcaggaaacatatctag
tatactagattttaagttgaagtaggatcttcaggagtctaatcattatttcttttcttttagga

gagaagacgatctgcacttcgcattttggcattgacatttaattttagggtcctttatatagaag
ggaga

>chr1_32644813_32645013
tttggaagaaggacattgaggcccagagagagaacagaacgtccagccacacagcaaatccgtga
tgaagttgggactggagtatgggtctcctgagtctcagcccaggactctatccctcttcccgagt
cctcggagttcccggatggagtcacatttgttcacggccagggaggaaggtttgatggaggcctg
cagga

>chr1_36840413_36840613
tggcctgctctggctcatttggactagaagaggagattgcaacaagggaggtcctggcatgggca
tgtgactaagagtcccttcagctccaaccatcagttgttgctttgtgcagtgactcagtcctgga
ataaggcctgccatggggtcccatgtggagttcaagttcttaccacccatgatagcccaaatcag
gcaag

>chr1_37943613_37943813
cagtgctccaagatactagcctaggatcatgtgcccggagggaggcaggtgggcggatacatgcg
cacactgcatacctgagacaaggagccaggcctgggatgacccagctgccatggttttgagaggc
aggccagaaggcccaaggaagggggtgatgtgatgggggaagcactgggcattgtgaagacaca
gacca

>chr1_38465413_38465613
ccagcgtctagcctccactgtcccgactgtccccagcgtgggagaagcaggggcagggagccacc
ccactctactccttatgagcagtactcccaccctctcctgactcccaggaagggactttaggaat
atggataaaggaacccttaaccagagctctggagtacagaacttagagacgggaatgggctgtgc
ctgcc

>chr1_38465813_38466013
ctcatccccccacccccgctgtgtcacaccagcttggacagccttggcctgtaacatctgcagca
gctgtggcggcacagatggccagatgtcaggttttgccagcaatggggagaaaaaaggaaaatga
agtgtcgggggaggcagaggggaactggagacagactgggagtgcagtgtacccccctgccacctc
ctgct

>chr1_38466013_38466213
aacatctggcaaaaccccaggatctttccatcccctggggtcatgatttccctcctactgcctct
gggagagaaggtctctcccacctttccgatttctcagggtttgggactagggagctgaaaggag
agagagcaagagccactgaagacattttctccccaatcaggccccatcttgggtggggagtagga
caagg

>chr1_45272413_45272613
ctcagtgatgtcaagggtttttaccttctaccccccttcttacctgtgttttagaacaagtggatc
agatagtcccaggctgcctgggttcctaggtgctgagaaggttaaaggctgttagacacatgagc
aggggacaggaggctgctaggactgggatcctcggctagtccttgccgactgagctccttctctg
ggcac

>chr1_45273613_45273813
gaggcccactggctcagctggtaggcaccaggccttataaagtggcagtgactcggcagtgaacc
ccgagtccagcctcgtgacctttgctgacccaagtgtcctggggcttgtccctagctggctacac
ctctagtacctaggtttccccaaagttacatttcagagatggcagaggctgtgacacctctgcca
gggct

>chr1_90372412_90372612
ccaggctgcatatagtttgcttttcctgttcgttgcattccaacctgtcaacggctattttctga
aggtctgtgggggctcatgtagaaaaggaataggtctgggtggaggccaagaatctgaatttgta
tcatgtaccctagctcatcttaattcaagtgttagtctgtaagaaacagaagaatgggtaactca
actcc

>chr1_110546677_110546877
ctggtattgcctcacctcttctcgccgcgagcattgacagaagatctagttgagtcctactttga
aaggagtattgcttctgactgtggaattggattctagctgtgtgtgtgttatatcctgttactgt
tgagacacagaggcgggagtcggcgggggaagatatgtgctgagattagtcagcctgccctccct
ctccc

>chr1_145455843_145456043
caaaaatagctgtaatcatttttttgtaaataaggacaagatcaaggtcaagtgtgggaaagacag
gacctatgtccttgtgaacatgtgtgaacagacacaatcatgtccttgtggcctgaggaccagag
gctcagaagtcagctgtactgcagggggcgtaggctgttctctacaatttactttgcattgccct
acaga

>chr1_150536576_150536776
ctaggctaaaaggccctccccttgatgagacaggcagagatcatctcccctggaacaaggtcacc
cttcctctctcccatcgctacattcctctcctccttactgttgactattggttgcctgcctcttt
catcacaaaatgagactcctctttagctttagtagagggttgggcaaactttttctctacaggag
tagat

>chr1_150540976_150541176
atagcaatagttgagtctcccacgggaacaggatcagggagagtggaagccaccattcagtatct
tagggttccagttaaataacttttggaagctcttattaaaattctgttctgcagacagcatagtg
ctcttaggcaaaaccttggttcctttccctgactatgtaaaacctagagggtgaaggaccagtga
ggctc

>chr1_150541376_150541576
atacccagtccagacttatttagaccaaagagcctttttttcctatcagtgttttccaaatttta
tcttaagaggcagggtcttgttatgttgcccatgctggagagcagtggctattcacaggtgcaat
tctactactgaggagcacaggagttttaaccagctctatttccaacctaggcaggttcacccctа
cttgg

>chr1_154943576_154943776
cagaggcagctgggggtggaaaagggaggagatctcagagctcagcatttcccttcctgtcctgg
ttggacactcgaattcccccaccctctctatgcccgaaagctggctggccccagcccaggccacc
cccaaccgctaccccatacagtaagcctgggccattagcatcctctgctacagcagctgtggact
ggagc

>chr1_154943976_154944176
ctcatagccaagcactgtcctgtctcccctttccacagggcttgttaacacagcacactgtcaag
ggtgagaatgaggcaatcaggtccccactcccactccaaaccaccccaattcacatccctcctct
tacagggccctaagccccaccaaagagcacagcatccttcacaccagggaaggagcctggctgga
ggagg

>chr1_155953376_155953576
tacgaccctctctgctgtttggagaaggcctctgggcctaaagccaataaggactgaatttcctg
ggatttcatcagccccatcccatgagctaatcccaaacccagagccaggaggatgtgtcagctag
agattttcaggaccacaaagcagagcctgatatcttaaagggaccattacttgcttacacatcca
cactg

>chr1_156075176_156075376
ggagaccccagccatccctactctacttggcagccccccctccccactcctcctgagagtgcccct
tgctccaccccagcggccaggaaatctcctgcagattcaccaccaccctcctggctgggagttca
ctttcctagttgacctcctggcctgagggccagaggagagctttcaacggggaccttgaggagtg
tgagg

>chr1_156092776_156092976
ggaataattctggctgagatcccaggcccggcaggggcgctgagtcatggtagagggcagagtgg
agagtggacaggagaccctaagcttgtccagtcagaaaagcagaggctgaggggtggccttttct
tgagaactacattcaagttgcagcaagaaggacagtggtctgaatttgacggggacaaatggaag
ggaga

>chr1_156096376_156096576
ctggggagccggacttccttgtcccaccaggcacagctcttcagacccctgccttgggtcacatt
tgcaagtgccaactctcatttctaccttattcttttcctctctgttcccctccccacccccctctc
ttccctctttctgagatcagatttgccagtgatgggaagagttagaaacaggatgcccagcccctt
ctcgc

>chr1_161171576_161171776
ggcggccttggcatcctattctttttttgttttggtttggcgggagctctcagacccgcccagccc
cacttaggctcctttctccaatctcaatttatgacatctggaaattagctggctttcccaactcc

tgtcttttggattcagtgatgggaaagtaattggcaaagcctggggctaccctataagggcaggg
ctcag

>chr9_35728600_35728800
atctcaatcacacagcccccacacagatcccatcacgactgcattatcttctcctagggagccca
tcctatgcacacagcttttgcacagataacctccataacaagcacacccggcacccagtgtctct
cctcccattatgtagacattctccattcacccagcaagcattccttgagggccagctgtggggaa
gaccc

>chr9_130834379_130834579
ttacctgtaaaggagcacaagttagtcctgtggggaacgtttgaagtcttggagagaagatctgc
ctttttgtaaataactgggcaacaggaagacatcctgcctcttgtaccccccataccacttcctgt
tgaattagcattagtattaactgcctacagaaaaagcagtgctgaggctttgcagaactagcagg
agccg

>chr9_131901579_131901779
gaaatctcaaagtagccactaggtggcaggagaggcacactgaacttggagagggtttggtgaca
tttatttgaggcagcagaaggaacagggaggggagggcgtgcctagagttgttggctgttccgca
ccttctccacaggtccgggttttcactttgggtctaggctcttgggcatggtgttcaacagtaga
cccta

>chr9_131901779_131901979
ggaggagtgtgcccaggagccggggtggctgcagcaagggcccatcttgccacgtggccgctggt
tgcagcacacgttgtgttggttctccagagcgcccaccctcttccacctcggagcagtgagcagc
atttgcagtccctagttggtgagtggcctggcctagctcactggggacctggaggcttgcatgg
agttc

>chr9_136216179_136216379
gtacttcgtggcaccttggcttcttgttagatgaggaaaagcatcgtgctctttgttctcaggtg
tttgtgtgcagatgatgtaaaagaatatttgctatctgagagatggtgatgacattttaaaccac
caagatcgctgatgcaccaacacccttcctagtggccccagacatgaacttgacatggaatttga
gcctc

>chr12_6444739_6444939
tgccagcttcatttctggttttccctgggcttcattttttttttctactataagagacactttgt
gtaaagataagagggtgtcctcaatgtctgttaaacactggggagcaactgactcaccagatcac
agaaaccctagggcagggaaggagggtcaggcagcccacagccacccagcctcaccaactccaca
tttcg

>chr12_6446739_6446939
tgagatcccatctccctcccgtgaagccaccattgacgatgtctgtttcctccaccccggctcct
tagacccatagtccctgtgaactcgaagcacgtgaactgaccctatctacttttggctcttatca
taatatggttatgttgggtgacagatatgatctccccaaagtgaccacgaaccttataagatgag
agatc

>chr12_6641139_6641339
tgtctttgtttcatctttcacattagcccagtttcatgcagcagagagagggttatcagtgcaga
gagagatgagtgagcccagagtcctagggcctgtcccgggatggcagatgagcttcctgccccgt
cactgccacctttcccctctcaacctctggaccctgcacagtgaccagacagcctctctggggag
aatta

>chr12_6641539_6641739
cccacaatgtcaatagcgtcacagttgagaaaacctgctctagaccaagggttgctttctgccgt
gtgcctcaccccacccccactcgtgttccctaatcccatctccaaaggttggcagcagaccggcc
caggctcgtggaagttcagatcatgatcccctccagctctgcaggagacaagacctgtctcccag
cattc

>chr12_31902133_31902333
atccacctcaccaagaattcagaggctaaggtttttaaggatgatttggcaggtagggcattagg
aaacagatgttgtggattggttgggggatgaaatcataggagtgttgaaactgtcttcatgtgct
gagtcatttcctgcaggggagtggtcagaggaccggttgagtcagttcctttgttgcagtcacgg
tcact

>chr12_45628133_45628333
aggagttcatgtgtaataatttatttctccagaaggtcacctttttttcctactgattttttttta
ctggtaaaaatattgcaaacagatattgttagtctcgtttggtaactgaaagagctctgagaacg
gaaaagttagagtgacctatccaaatgaaggagcagttcagtgttggtgcaggatattagattcc
aaaat

>chr12_45629133_45629333
gtctgttctaagtagggctgtgaataagagtggggtacagtttcctgcaataagagatgggaggc
ttaacttcagcttggcacgtaagaaagatcaaatgtttactgcaatgtgggaaaaccacctcctg
ggcataatagaaatccaggcctggcagtaaacaattctattcaaaagcagcttctcagggctgag
gaatg

>chr12_46776333_46776533
gactgattaaggcacctgcctccggttccatgtcacttcgtcttgggacacgtggcgctaatgcc
acttttcaaccccccaccccattcagcgacattaacccgggcgacagtccagggctaaacagttg
ctgaaacagttaaatgtgtgttttattgaggtgaaccacgtacacaacaaccccctttgagtgaa
acaat

>chr12_76338333_76338533
agagggaaatgaacaagtgagattaacaggcagatgagatgatacaaagcaagcacaggtccagc
cagggttaacctgacacaaaggaatctgaataacaagaagcctttgaatgactcaggtaaggttt
atctcctccaaaaggaagttaacaccatgttactcacagtagcaaaattactcatgaactttgga
ggaag

>chr12_76338533_76338733
tctcagagaaggctatggtcagaaaatggaaaaggaaacaagccacaggctcatcttcagatgag
gactctgcacagggcttgacctttcaatcgaagaacaaccacagtagtattctgagatggccaag
tgtatgggaaagtgactgcttctctcccactccaatgtctactgtaagtcttgttcagccccgag
acaga

>chr12_109231871_109232071
gttatttttaaatctccacttaattcgattttggtaaaacacgacctgtaattttctttatcg
gtaggtaataaaagcttcagatgattttactgatcactggtatggcatatttcatgactttgcc
ctttcatctcttgcatagttttaccctcaccaagcaagaccttccctgcctcagcactgtttgcc
ctctt

>chr12_122884647_122884847
ctcctcggcttgagccactgaaaacacttacacaacgctgggacctttccaaattgacttgtttc
cccttaagaaagggcaagctatttgatttcttccttccacttcattcacattcatatttctgta
tatacagaagagactgaaaaagataaataagattgaagggcatttgcaaattttcttcaattctt
gagta

>chr12_125402447_125402647
taacctatttgggctttgtttgtgtcgtcgtttggttttgtcttaaaattgtggtaaaatacaca
taaaatttaccattcttaagtgtacatttctgtagcatgaagtacattcagtattgtgcatcagc
catcaccaccaagccatttctggagctcttttcgtcttgcaaaacaaactctgtccccatgaaaa
actga

>chr11_6627624_6627824
ccattaggttctacactatttctctttaatactttgcaaagtcatttgttccatttttcatataa
ttctcaaagggtaccctgataaagacagtaagcactgttactgttttgttggtgaggaaatagag
gcttaggcagttgcccagtgggcctagcatctaggaagactctagctgagtctaggaagactcta
ggaag

>chr11_46575824_46576024
taaaaaataaaaaaaaaccgaccttccgacctcctgttactcaagaggaacaataaaatacaggtc
acaagtactgtatgactcagaaaactgtgatgcaacctcagattcctacaggattcagaaagggg
aaagagtagctacaaatatcaataccaagaaaaaaatcaaaggatcaggattaccaaacaacatt
tctat

>chr11_46576024_46576224
caatttccagggacctaaacacactaacagctcaaacaagagaaagctttactgatgggaagaag
tcagagggagtaaaacaattacacaatctctctttcatctgaagaaaaatatttgctgttgcttt

aaaaggataagggacttctctcctgatatgctcttaccagccacctgagaagagattttactctg
ttccc

>chr11_61739024_61739224
tggctaaacatgcatgtagctgcagtgaacatcgcagcaagactgagggacagcttgtgctgtag
aaggtcattgggaggaggctggctgggaacaatggaagggaaatctattggtgggggagtgtgag
tgtgtgtgtgtgtttatgattgtttatatgattgcttgtgtccgttccctactttctggtaactt
gaggt

>chr11_61739224_61739424
gtcagaactgcaatgtctaagcctttagattcttccgaagctgtggcctggtcttgggacctttg
ttgaccagggttctacctcccccaccctccagctcagctcctcgtgacttggaggcagcagagag
tttggctggtctcccaggacagccgcctcccagggccaggagtggcgggagggacctcagatgag
agaag

>chr11_62320224_62320424
tttcctaagcaattctcaagccccaaacccttaaccctagccgaaaatataaagccaagtaaaat
catccttccccagctcaattcaaatcccagcctgtatttaaacttcaagcttcaaataaggagtt
cctgaatttcttagatcagagagctttgatacacagaaactctccaattacactcagtcctaatg
aaatc

>chr11_62389824_62390024
atcttcaccttattcatccatccagtcagtcacgccacccaatagctgttgctcaatacttatta
cacgaatgaaatctaaggcccaccacatgatcctctgggtgtctctcctggaatcctcactataa
tactttgagatagataagaaaccaaagttgacaaaaattatgtcattgccacagggcagttttgt
gtcct

>chr11_65187824_65188024
tctccttaaatacattcctttcccgagaaaaacgagctgtgtggaacttggaggccagcaaagct
tctacataaggaccctctgaaacagtgcccttttgtccttgaaaacccagggtggcaaaggcagc
tccagatacaagggcacaaaggagacttggttccctttgtgacagggaagcctatgctttacaga
atcct

>chr11_65255224_65255424
cctgaccagggtggaataaccaggaggcaggcgcacgtgaccgtttccatggtgacctcccttag
caaccctgactggtcctgtttatgaacgctggaaaattccaaccagcctctgtcctgattttttt
acctactctgcctccgcctgctccaatgcaggggtgaagtcactgccaacgtttaaccctttccc
cacgt

>chr11_65255424_65255624
gggcctggctccagtcagatcgagggtctgggagggctgcagggatctctcagtcgccaatggtt
gcagacaatccagtaagccacagctgttacttgctgcactgaaccctgcctgcgctgagtaccga
aagcaggagggaaggtagccacatctcttgagcccggcgctgtcctaagctcctgacatccccac
aggcc

>chr11_65255624_65255824
tgagaaaggggcaccacaaacaggaaccaggctgagactgagcctgtggaccctggtagcaaggg
cgccctctctctaagaagccctctgtcatgctaccgatgttgcagaacacctggggcggagtagg
gagtgccaaagcgcaggggatgagtgcacagcagggagacttctatggtctgcaggtgagctgaa
ggcag

>chr11_65255824_65256024
gcccctgaggaggtggacatttaggctgaatggaggacagggaggtagcaggacctgctgtctcc
aggacccctaagtttctaggccagagctgacggctggtgggtgcagtcagccaggctccagggaa
ggctgctccaggcagggctgagtcttcacggttcagcttttagaactggccaggttggaattctg
gctgg

>chr11_65256024_65256224
gggcagacagggagggcaaaccacaaacacttctgcctgatctggctggggaatccacagagacc
accagctgggactggggtgagcctctcagagcccctagcaccagcccaaggctgcatgggctgac
tgtgtgggcggatgctgcctaagtcatgtgcagagccgtcctgagctgaggcagaactataagac
atgaa

>chr11_65256224_65256424
ctctgttttcaagacaggtgacaatgacaaacagcaataaaagccacacaccatagctcaggata
acaagggctgagatgcgtttggacaatgagaagaaaataaaaattaaaaaaaacagctgattcac
caaatgaagggcacagtcccaggcgtgctggtgacgtcactgggcagggaaggctgcaggaatct
gcagc

>chr11_65257024_65257224
ggagggcaaagaggaggagtaacagcctcctccgtcacagcagtactcgagtcacatcagctcat
caccaatgtgcctcccccagagtaagggcagcaggggcggaagaagcccaggggcacctaaatat
tgtgggttcagcacttctgctctccagaaatgatccaggtccactgttttctcaggggggagtatt
ttata

>chr11_65257224_65257424
tgtctatctctagaagcttccaaggggaaaaagtgacttcaaggggaaagaggagctgggaagac
cttggcagctccttccccttgcccggccttcctttccctactctgggctccaggctccaccccac
ctcctgttaggccttcctgaacagcagcagcagctgggaatgcaaaatgacatccctatgttgtc
cctgc

>chr11_65257424_65257624
cctttgtccagggtgagagggacccttcctcggtggagttgacacagacctgcctctctctcatg
tctgtactgggagtgagcctgaggcctccctgccaaccggactggaaactctggaatccccctttg
tccccctttgatcctcagaggagcgagggagccctgttaacaaacacatagtcatccgagaaaatg
taggc

>chr11_71814752_71814952
agatgatccagatgaccagtacctagtcggggaacacagagggtgtatcgcccagctgggctggg
ggtgaatacacagagggtgtattctccacctgggctggggatctgcgacttacaggaagaaggga
ccttatactaccttcacatgctgacgcgcagcggagataaatcctggtagaggagatagaagcca
cctca

>chr11_118782990_118783190
gaggtacgagaaagcaaagggatggggctctgggaacacgtcaccgtgcctccctcttaggggggt
taggtggggaggggtagcaagctcacaaacagggagcaggtgcgagtcacctgagtggaaggcca
ggcgcataccaggagaatgaatcggaggagcggaagaggcggcttgacaaagggcctgagatatc
catcc

>chr10_76969594_76969794
gggcctaatgcaggaggtcagtctaaactaacggtctcccatatacattttatctaatagctctg
tcacccttcctgcacacccggggaactcgggtgcggcattctaacgcctgctcagcatgattcca
cattttctccaggcccataatccagaggaggttgggcccggagcaggcttgcctcgccaagctcc
ggctc

>chr10_103911210_103911410
cccatgaggatccttatctttcctgagcggcattagtgggaggtagccaaggggtagtgggtagg
attgaagggattgctaactgctaatatctggaatgtggcagaacttggaattaacttggtaggtc
tgagcattgatactggagttactcatgacaaaggcattgatactggagttactcatgacaaaggc
agagt

>chr17_7482476_7482676
gctctgtgaatgacaatgctgactggagtgctgcccctctgtaaagggctgggtgtggatggtca
caagcccctcacatgcctcagccaagaggaagtagtacaggggtcagcccagaggtccaggggaa
aggagtggaaaccgatttccccaccaagggaggggcctgtacctcagctgttcccatagctactt
gccac

>chr17_17656075_17656275
tccttctttccggccgattcctcacccccctcaggaggctctccacacagaccgtgacctgccctg
cctggcactcctctgcccagcgtttatatctctttttgtgcactctccgcccctcatcacacgtgt
gcctcagggcagggccctgtctgtggcctggcacacagtaggtgctcggtttgtgttgttgaatg
cctcc

>chr17_17744075_17744275
agccccccctgccccccctgcccccctgcctggccctgcatatccttcactgctgtgggagccaagc
tgggacctgcccaggttatggcagaggggccaggctgtgggaagagactgctcagagcctgagtc

tcccctcccccacttctccatctgtatataatggggtcagatgactgttgacatgggtgattctg
gtcct

>chr17_27073473_27073673
tgcccagagtcttagctgtgcttgctggacattttccttgccccagcttctcttccttgtgtgt
gtggtgtagtagagcctgtctgagctgtggagtcagacacacatagccaggtgctgcttcacctg
taagcctcttcgtttcatctgtaaaatgggggcattccaccccttctcagaggcaggtggcaagg
gccgg

>chr17_27135274_27135474
ccaaccatttgatctaggagagaaattaataacaatgccagcccaggttaccaaaagctaaattg
tcctggatgaagagagaaccatacatcattccagtcctttattttgagccactgaacaacacaca
tgcatatccaacaaggattcttaaaggacagtatcattagaaagacagctctggccttcactgag
aacag

>chr17_38267674_38267874
gagtggctcttgggtccaaagggcttccccttggccaagtcccaccccaaagcccaggaagtcca
cagcttggcgcccagtcaaggcaaaaacattcctcaggactctgacaggctggggggatggttccc
accacgaggatgggggaggggaaggaaacacgcgggggaggaaatgaccacaaagaagggggtga
gggta

>chr17_38268074_38268274
gttgagcttgggagcccctgagtggctaaaagctggaattgcagttgtgctgggggaggagccca
tcgggtctccctctgcctctccacctccgcccctccctttccctctccctaagcccaggactctg
gaaatagcacctggtaatgggagcgctgacagatgctgagttataggagagcaaatgtcctggct
agagt

>chr17_62224468_62224668
gcgttcactacatggacattcgatgaatgaaggtgtcccgaattaacattatactagacaatgaa
gggtgtaaactgcagcccaggagcaccactgccctacccccctcagttacgaactttcactggatt
ctttaaatgtgggctgaagataatggcacatagtttgaaggatgtagtgaggatggctttgtagc
cttag

>chr17_62224668_62224868
gactgggcagtgatcaacgcagtaaatgaaagctgcgattgccaggtgagggccccggtgctctg
tactcattttattgtgcccctatctgaacaggattccgtagaaatgacttagatgcttatctagt
atttgacattgagatatttattttgtgaaacaatgcaagcgattttaaatccccacatactattt
gatta

>chr16_2202799_2202999
ctggtctgctgcctcccacaggcctggctgaccgagatccacgagtacgcccagcacgacgtggc
gctcatgctgctggggaacaaggtgggaggcccggctgtcctcacctgggccacagggcagggca
ggtgagggggcaggggccaaccatgggccagctttcaccaagaccctgtgcctgggccaggtgga
ctctg

>chr16_4665799_4665999
ctagggaccctgaacccctctgtgattggcatcgctctgtgttaaatagtaggcacgcccttaag
agaggggtctctaggaattttggcccaaatcggctggtctaggctgaaaggcccggcacactgta
ggcgccgacaaatactcgttcagtgaatgaatgaatgagccacttaaaagctccgccgcacgtca
ccgcc

>chr16_14595299_14595499
aaaaaaaaaaaaaaaaaacagaaagaaaaagagaatgaatgttaaaatgtaggcagaggcatgaa
aaatagaaaagaaagtcagcttgcagagccaagtttatatcttaccccaccctgatgtggttggc
caacagctgatagagggcttttcggtaaagtaggtagaaacaaaccccaccaaccctcaggttac
caggc

>chr16_53132699_53132899
gtaacaagcccttaacccctggctgttattcttatttccaaactggggtgcatcattagataaat
gcagaaatcaagaaccgtcgctcctagtcaagcgtctgtggctcagttctcagagccaggccaaa
ggagtaattatagctttcctctatagtgtacggggcaggaggctgatttaataaaggaagcctac
tgtct

>chr16_53132899_53133099
gtctccagcctggctctgactcatggaacgagggagtcccagagcactacccacagagggtgggt
ggtgagcagctgagggtgggaggtgagggtggccgctagcaaggcctgggcccagagccctgaga
atgaggaaactccctgcttggccaggaggaacctgcagctggagccagaggaagtgaggctcaga
cagag

>chr16_87421499_87421699
actgctcaaaagaactagtcacactaactggaaagaagggaaaatattcaaactttgaacaagag
gattgcagtttaaaataagcgggctgatggagcctccacagttgcacacgttaacagtcccagaa
tggagcaaaagctataaacacagcagtcctacagaatttgtttagaattaaacaaacaggcaaac
aaaaa

>chr16_87812699_87812899
cccctcaccccacccgccacacctgccaacttcccggcctgcagggtggcaggaagcctgaggca
ttattcagcttccttagggcccgttggctgatgcaacacagctcagaagttgagtccaagcacca
agcccgcaatgtctgcgtcggccgccaggggtcactcctcccgcgcagaaggcggccttggagcc
tcgca

>chr16_87886699_87886899
cgtgggctcctcttctctctttccccttgggagtaacgctcagaaaggtctggccagccaaggcc
ttgaggactccagtggcttttccccagctgaacaaatcctgtggaatcttacaatgtaagcaaca
gaccccgaggaaagacatccatgctaattaactaatgacaccggaaacgtcctgaggtgcgggac
gggct

>chr15_41575508_41575708
agcattaaatttttccctaggtgttgtgctacccctggattaaaagcctgggcgcggtggctca
tgcctgtaatcccagcacttcgggagaccgaggcgggtggatcacctgaggtcaggagttcgaca
ccagtctggccaacgtagtgaaatctcgtctctactaaaaatacaaaaattagctgggtgtggtg
gcatg

>chr15_44092308_44092508
ggagacgtccggaagagaacactgacctccaggttgcggtaaatgcaaatgccctgtgaaggagc
tttgtccttagggcctcaacactgcggccactcaggctgttctctccagattagggggcagtttg
tctatctggccccctgtggattagcattctttattggtttaggattagacgtcattcaggttgaa
cggag

>chr15_66124946_66125146
tggttaacatttaactaataatactgacgtatgttggtttagcccagatgctggcagcttttaat
tctccaaactcctggcctgaatactgatcatgtttcctcaggctaaaagccagcaaaaagaatca
cctggccaaacatccccttcagtctgctttccttagaatggcctcccaggggagctctctgggc
aaaca

>chr15_90944396_90944596
ctccttaatttttctcctgaagtctcctagcaatctgattgatctctgtggtttgacagtgcgtg
aattaaaaaaatcttaaacatattaatgtttaagaaagatctggttaatggtttattttgaagag
taagagtttacttgctgaagtttgaatgttgggttaaatttttttaaaaaaatagttgtatgttg
ggaag

>chr15_90944596_90944796
aattagttatttcaataaggcattttattctagaaacctcagtactttaaggcagctaaaggaaa
gccaaaataatcccccaaaacaaaaaaccaaaaacctgcaatttgcaaggcagtcataatctaaa
atattttctgtctgtcccaaagtcagtgtattccccttgcctctccccctcttcctcattgtcat
gatta

>chr14_61569247_61569447
gggaagggaagtgagctcctttagcagttcaaggtttccctcccactctgggacagggtggtaga
ggtatgagagtagagactgctgagagaatatggtctctttcattgtgctctgttttctcctttg
gcctcatggggaggcatggacgggtgtcatcaaagggtgacttgaggaagtaggagagcaaatgg
cttct

>chr14_61569447_61569647
ccctggttctcagaagtctaggtccctcacctggctcttatccacaatgcctgtgctgttggtaa
tagttcacgtgcacatgtctcctccagttaggttgaaaaaaatcctgctggctcgcactccaaca

tgggctgctttgtatgtctccctggcaaggcacacagcaaatattggttgttgcctttggaggcc
aggtt

>chr14_61570847_61571047
ccatgcagcagggaagcgggtgtctgctgcaaatctccccgtgtggggggatgggaagaacaatg
aggctgaacagacacaaagggagctgtcctgtgcccagacagccccagtgacatcttcaggccca
gccaggatcctcccttcccaaaatggagcctgtttgctcccagtattagtagggctctttcgatt
gcaaa

>chr14_75725247_75725447
aagttctgctgcttttaaagtataaaagataaattcaaagttccttatgcttctcctttctggga
gaattctgggttttactccaacagtgggaaagttttctaagctggttttgctcctgtctcatggg
caaggcagtttggtcggtggctgttctgtgggctgctgtctggaggagacggtcggtggagacgg
tcggt

>chr14_77421847_77422047
tcttatccatagatcctgcacaggaacacccccacgcaggccctggccctctccttgttatgtgt
ccctgaggctgtcctcccaggacttggttcactttcaggtctgccacccattggtgcattcattt
ctgtatttaatgttactgagcaccaactatgtcccacacactcttccaggtactggggatacagc
agagc

>chr19_1265000_1265200
gaggtggggctccggagcccccagaccttttcccagtttcagaaactaggtggctgcatcccca
gcgtccacgcaggcgagcctaagcctcggtcagctttagggtgtccgtgggccatgcagggtctt
gggcctttcacagacacctgatctgcacgtcacagacacgcatgggtgcacgtcactacacgtcc
gccac

>chr19_1265200_1265400
atgtgcttggctggtctgcgccccagcagaaggggtcacctatgtctctgcagtatcctgtgtac
tgaaagagcaaacaggtgtgtgaccccagggcaggtcggggtccgtcctgtccccagcctgtcta
ctgcgtgtgcctgtggcaggcagggtctccggatgccgagtgcatggtgtatgactttgtgtcca
gtggg

>chr19_3983800_3984000
cttcccagcgtcccaagcactagaacaccctcccctcccaggtgtgacagccaaacctctccaga
tattgtaggagtgggggcaagtcccccagggagggagaaccacggagttaagccccctcctcaag
aaaaccttccagctcgcagtaccccgaatccctgtgcctctccatcctgtctcgctggactgaac
ctcac

>chr19_4374600_4374800
cgggacagcctcctccctggcctcctggccctcgtctgcattgagaggcttggcctctggtccgc
atgctgcccttcccgtgctgtggccttgcagcccggcctctcccctgctgtccccccttggctctg
gcctggcccctgggcccctgagtcacccctgaggtgactcagcagtccttggaaacgcatgccga
ggacg

>chr19_4376000_4376200
aatgcatagccctgcaggaagagactggaaatggtcttggagagagaactgcggggcatggtgac
agcggtgagaggctgcaggtgcacagatctcaacgaggggccgtgagttccctcactccctgaga
gctgaggagacttgcccgaaagcccagagcccttcctgagacacagaaaccatcttcaccatctc
taaga

>chr19_6738200_6738400
ttttattccagagtgaggcaattggagccccacccccagattccaggctagaccggactccgggc
tgaactctgaccccaggtgaactccctctctgcaagttgagtcctaacttcagcctgaaactctg
acaccccctcgtcccccacaccacacactgaggcttaatctctggtttaggccaccgatctggac
tagcc

>chr19_13957000_13957200
agtagctgggattacaggcgtgcaccaccatgctcggctaatttttctatttttagtagagacag
ggtttcaccatattggccaggctgatcttgaactcctgacttcaagtgatccgcccgcctcagcc
tcccaaagtgctgggatgacaggtgtgagccacagcgccaggccctacaccccagctttctagct
ggatg

>chr19_13957800_13958000
ctgtggggactcccagccaggtgcctgaaatccccggggaagggtcccagtattgctcaagggca
ctggtggggcaggtcagacaggaatagaccccaggcctggcccagcccccacctcaggaatgact
caccacccacccagctcccgatgacatcaaggcctggcctgccaccctgacctggccccaggcca
gaact

>chr19_18485400_18485600
gtcccagccctgctgactcatactcagctgccacggtccgctttccctgctgagcacgggggtgg
gggcgggggggaagagggggagggaagggcacctgaccgaggccagaggcccagaaaggacagaa
ggtcatggctggggagaagcaggcaggagaaccgagtcttggccttaactggctggcaacgtaaa
acctg

>chr19_39156160_39156360
agctcagtgggaaaccaaaacatacacccctttcctccagtgctccccaggaagcgccaagtcct
gagcccagccagcaacttcgaagaagtgccctccaacaagtggcagtgggcacttgagcaagtga
cttaacgcagccctctccccagcccctgcatccatctgggcctcattatctgggaggtggcttct
tgttt

>chr19_39177360_39177560
gagtgcaggctcttccccttggggagaacccagttctttgacgtataatctgagtggtttgggtt
ggttggttggttggttggttggtttcccatgtgtgggatggctccggaagtctgtttgagaacag
aggcaggctcagatgggagcagctcctaccccgggccgcctatccccctttaccctggggtttct
tttag

>chr19_39925160_39925360
acaaaggaaatctcagaatttttattaaattctaggccaggcatggtgatcacacctgtaatccc
agtccaggcagaaggatcacatgagaccaaggaccagcctgggcaataaagtgagaccccacgtc
tacagaaaaatatttctcaaatgagccaggcgtggtgatgtgcctgtagtcttggcaaccgaaag
gctga

>chr19_47611760_47611960
aagatctaagagcaagaagcagaggtgtaggcaccgttaaataaattactaaccaggcccaatat
gaaattgctctacattcaactgtatggaaacgcaagagtcaaaaccgttcatctttaacttgcca
ggcagttgaacagccaggtccacaggtacaaaacaattctcccctgggatcagccttcctggctg
tggaa

>chr22_36725454_36725654
ctggcccctaacgacagtgtggcaaccgataagggtttggaacaaacatttttgtcatcccagcc
ccctgccctcccccactgcgtctccctgtggtctgccccagctgtgggcatgtttagtcaccggc
tctactagcaggcaagaatgtacctgcgagtgctggcaagtcgtgagcagccaacggcccaatgc
tacca

>chr22_36767054_36767254
tcaaagcaattaaacaaccggtagagcaccgtcctggagggctcctcccccagatctgaaggcat
ccacagaatgtccctggatcagagaaccctctgctgacacaaacacactcccctactcccttctt
ccctgttacagattgaattgtgtccctcccaaaaatatccattatgttggagccctaactcccag
taccg

>chr22_38576854_38577054
caggcagaaactctaacaaaaacaatgcattcaaaaacaatgcattcagcccaagggtgcagcct
caacgttgccaaaccgaacctgcctgacatcctgttgggcgggtaagaggcaggtgagggaaaga
cccagaagctagggggaggtattcgttggggtcatgtgggtagcccattaaaaatacggccacag
gtccc

>chr20_30307339_30307539
tctgatataactggctcttccacacacatcccctctgttcaccaagaggaagtgacatgtggtat
gttgtccaacaggctctgatgattcttaagcaaagagatggaagatggaatttcaaccccatgga
gatctaataaacttacccagagttgctgtgtctaagacctctttcatatgcaccactgggcagct
ggcaa

>chr20_30307539_30307739
ttctctggtaggggtagccttctgcagaggctggctagcagaccaggcacctctcccagactgcc
ttttgtttatagctggcaacaacccacctggtattaggaccactggccaaagacaatagtcaggc

aaagtgggcaagcagcctcaatccctacttggagatgcctcagagggtgtgtctgcagtactgga
atcct

>chr20_45988393_45988593
agaaacctggaactaagcccagagaggtgaagtgagctgcccaaggtcacacagctgggaagtgc
cagagatcagatgtccaccccgttagcacttcacatccctaaactctgtaccttctcctggctct
aaagaaagaagcaaaagcaaagccgaaccaagcacagcagaaacaatgaggctgaaacctacgaa
cagca

>chr20_45988993_45989193
tgtcacagttagggaggttgtattaccggtatccagtgggtagaggccaaggatgatgcaaaaca
tcctataagtacacaagaccagccccgccacaacaaggaagtatctgatccaaaatgtccaaggt
tgggaaaccctggctttaagccctttaaggaaagggaagaaaacagtccaatcattctgcctctg
gctag

>chr20_49435193_49435393
acatggagacagccaggaaatggcttcaggcctgcgaggcctaggaggagcctcttgcccaggcc
acagcccgtggtggagttgggcatcgagttccttcctgaatccttctgttctctctgcttttacc
tcctactctgtctggttggcccaaatctactagcctggctgaggaggaagatggaagacaatggc
ccaga

>chr21_35320730_35320930
ctgtgccaggtgattctgtgtataaaacagcgcagtgcagcccctgaagcacaaatattgttttg
ggttgcaaggcggtgcatgtgacgtagttgaaaatacagactcatttgtgtatactccccacgta
aggagggtaggcttcatctaaaagcctgcgtgtcaaatactcctttctaatacatcctggtgtgc
tctgc

>chr21_45147572_45147772
ggagggagccagcactaggtggagctctgccagccggggcacagggaggaacagcatccaggagg
agggtgcagcgggtgtgaggccctgtggagcagactctggagctagaataaggtggcatttttgc
tgcacgctgtgggggtggacggggcccgaggtcacaccagggagcccatattgggtcaggaatgc
tgctt

>chr6_30582421_30582621
tctccaagttactcttgcaagcccttgtgtctttcccatctccctctacacacatatatacatac
acacacgctcacacacatcctcaaagcttcccagtcttaggtttgcctgtttttcacccctggc
agctgaagtggggaaaaattacaagcagttgtgatgagtgaaggaaagtgaaaataaaaactggt
tctat

>chr6_52382641_52382841
gtccaccctaatctcagccaccctagactgggagaatgtaacggtgggcagtggtgagggggtgg
gaggaggtggcgaggacagagagaacatgatcttggaaatcagacaaactttcctttttcctctc
cctcttcctttccacaaagacctattctgtgagaggcactgggctaactgttgaaacatggagat
aaaag

>chr6_52382841_52383041
acaccacccccggttggaaaacatacagtgcagcagggagactaacagacagatgtacgattaca
ctacagacgggcagcgctcctgagaagcacatacagtgagccacagcaggcgagggccccactct
ggctagggtggaggttgtgggtaaactgttcactggtcagggtgaacagaacggcattggaactc
gggga

>chr6_74289479_74289679
ataccatgcactggcaaacatacaaacaacaaaaaaacagggtattctatatattctattgattt
ttaaatgttggctgcagctgctacattcctcgagagtaactgagtcccacacaagaaaccctaag
ctcaacagattctctattagatgattgcatttttttcagaaacaatctcctaaccaccaggcctct
ggcca

>chr6_74289679_74289879
atttcttttctgagttcctgttcctgccagaaagggcaaattcattatcctgtgaggtccaagct
ggcctcttggaggtacagcaaagacttttacccttggcctggaagcaaaggctagccatgtggcg
cccttgggctttttttttttttttctagaaagttttctggtgagttaaaaaaacgtgctcagagt
cctac

>chr5_133839101_133839301
tggggctggccctctgcaaggagatgccccctcctccatgcaggaaaacctctgtggtctgggag
ctctcaaaaggcaggaagttgaatcatcaactccggtaacgtctctgtcagcctgtgggtctgcc
cagggagctcagggctccaggcccagggggctggctctgcgctttgtttgctgctttctctgaac
aggaa

>chr5_172192195_172192395
gagcagggctgctggtctgcacctacaggctgttctcaaacaggctcaccccgcagagctctgga
aacgaggtgagcagccctcccacctgctctccagaaagcaacctgcagagaaaaacagcccgggt
tgacctggatccacccggcgggcgcggggaggggccgcagcggcattgtttgcctctgtgtatgg
caacc

>chr5_179246394_179246594
atggcgagaaagcaaaggagctccttcttgggggtgagtggggcgccttgagcgcttcctcaaag
ctatgttcccagagccacaggccttccttgtgtccctcaccctgctcagaccgggccatagccgg
gggctggggcaggaaagccggccctcggcgggggccacgtggctctcaggcgcctgggctgctga
gtcac

>chr3_39192596_39192796
ctcagcagctacccaggaatcaagatcctgaccatggcagtgttcccagggtgagcacagccccc
tccctagggtcctggaggaaggaaaagcagttcccaggagctggctcagacagaaataccacacc
gccgaggaaccccagagacaggccctgaatctgagagccaggcttcttctgggatctgagccctg
acaat

>chr3_39193196_39193396
agcacctctagcccccactgaaaaaccagcctgggtggccctgagagggcagaccgaaagccttg
gcacctcctccccaaccatccctctgaaggagagctgggggaggggacggctcctcggctttggc
ctccaggacctggcactggataggtgccaaatgtttgctggctacatgaggcacttgttcctgca
gaagg

>chr3_39193596_39193796
ggtgctgggatggagggtctgaagggtgggacagggtacggctggggtgaaggtaggtctctccg
aggaaggaatgtaggcagagttccagataggggctccgcatgggtaaggaacaggaggagggacc
agcacagtgtgatcggaggaggggcagcccgcgaggctgagcctgggctccagggcaggctttac
ctcga

>chr3_52086760_52086960
ctttcaggaaggataaaggtacccactagccaggcccagcctggcgaagcctgcttccctctctc
tattcccctccctcgttgggggagttcaaatgctgaggcagcacccctagccctcttgacccagg
gggtggtgggcagggctgagaagctccaggcagccccagttgcagcccctcccccaggcctcagc
ttcct

>chr3_53304360_53304560
gctttccagcaacgccctacagcccctgtcaactcctgcccaggggcgctggacaggctgggctg
ggcaggggcggtgctggtgacagacaggacactcttaggggctcctcctgcacagtctgcaggac
gcagtagggagagatcttcatccttgtccccttttctggtacctcttgggtttccctgagcaagg
ctggg

>chr3_133291710_133291910
ccctccatcccccctatcttgtaagaaatagtatacccactttacagattagggacttgacgatag
gatttattgaacttgaccttggtaaacataatccagagggtggagttcagacaaaatccctttct
acctaccaaccagcctatgaaagatcttttagtaaatgggacctaaacttcaggctcactgctcc
tctca

>chr3_171858306_171858506
acattccatacgctgacctgagaaaccccttttacagacagcagcttgctgctgagtaactggaag
tttaccagcaggtggtttccaccagggccggaaaatactgacgggcccagagcagaaagtccagg
ttatggaaggaatgagagcaaaggctgtgattgtacgtgcaactgtcatcttgctgggattgtgt
aatgt

>chr3_177077906_177078106
tactttccagagtacctggcattatctaagagaggagcactccggctgcccaagaaagggcctga
actgggagcttggcggcctcggggaggggcggctgctcagagccaggcctttccctagtggggag

ggcatctgcaaggcacaaattagaaacagactatgagcttttgcttcactaaatgcagatgcact
ttaga

>chr3_183903106_183903306
agaccaattggaggaatattcacgttaaatataaagcagtagcatcattttggaggaaattccta
acttcccgagggaatcggtgaaaggaaatagccttttattggcatttattgagtgcagagaagtc
tgctcagggtgtaggtagggaataatttatacaggccacgttgtcctggttttcttgccctttgg
cggtg

>chr2_27603896_27604096
cgcgacgcccgccaggccggcctaccagcagctcgctctgggctctcctatacacgcgcaggaca
cacacgagcacacgcacacactcacatggggctcgccacatgctacagatgggaccaagtcggg
ggccacggcagcagaaaaaaaaggacacgatgcgtacgattaggttttggcctttagtctgaaaa
agtgt

>chr2_43446096_43446296
gcaacacttacagctgctcaccccccacagagccacacacctacccacacacagcagcagctaca
cacaggcacacccaaacccactcccatagatcacacacacacacacacacacacacacacacacc
cagactcagacataccccaaggacccacatacctacctacacagcaacagacatacccaaacccc
cacca

>chr2_43448096_43448296
aatgaatgtggcttaccccc aacattccagctgatcaaaagtacttgtttagaaaaataagttta
ggaatgaagtggaaacaaggcctttgaaatgcagcttaacctaaatagcacattttagacttttc
ctccccactgcacattctttatggtgtaggggccagggagcctgcagtcttcacccctcctgagc
cctcc

>chr2_70359496_70359696
gcttcagcacagcctggggaaggaagccctgccagcaggcagcgccaggccaagtgtaccctctt
tccttatccctgacttagaaaaacaaaaccgataggcaaatccactcatcggcatttctgaatcc
agttgttaaccaatcctcttcctgccctttactctccttttcttccttttttccagaaaatcctgg
aaagc

>chr2_112221529_112221729
aacaatgtgtgcttcttcacagggggagagcagggagcctgcagcccgcctgagctgcaggtgct
tcagctggtttcacacttgctgtccccagcccctgctcctggccagacgccggcctctgtgggcg
gcccggagggcaccgtgcgttggtgcaggccttcgtgggcagcagcgctgacgtttggaaacaag
gcccc

>chr2_112221929_112222129
ctttcagaagggcaatcacgaatataaagtagagaattagtagcagtgtcaagagtaaagccccg
aggctaataccaaacttctaacataaacggagtgttgaaaggcacatgccaggaattatataaac
cgaaggaaaaagacaactgggggtgtgttcccatgcattcgatttggtccaaggagagccaggc
ccacg

>chr2_219270556_219270756
gggggtgaatatggttggcctgcatcaggtggccttcccatttaagtgccttctctgtgactga
gagccctagtgtgatgagaactaaagagaaagccagacccctatcctgcttctgtggttattgcg
ggggacttcagcaagtggggtgtgtgccttgcacctgcggctgccgtgggcccccccccccccgc
ttcag

>chr1_8772813_8773013
gctttagaaaaattcaatggattttttttttttaatgaaaaacaaaataaaaaaaaaacacaggat
gtaaccacagctatattggtctttttctcaggaaggtcagaaaagctttaaaaacaggaagtctg
tgctataaggttgagaaatgtttgttttaacagcctaaggcgtcatggttgcagtttgcagtcag
ctgct

>chr1_23881613_23881813
gcagagtggagcccttggaagcagcgctgcgggaaagagcgcggcaggaggttctcaggtttagc
ctggggcatggggaagagtcgtggccacgaggggtgatgaggccgggaagacagataattgaagg
ttgtccggcgcgtccacagctagagagctataactggctttctgaaaagggcaccatttctggtg
tcaca

>chr1_23882013_23882213
gaagcctcgtggatactttatgcgccttatagtttatccgaacaacacacatggaagggaggtac
cgacatccccctttcacagatgagggggagtcctaggtctttcattccacaagcgctccctggga
caagcacccccctggggaccaagcatgtgccaggctttgggaatggcgagggaaaagagccaattt
gaaga

>chr1_27049813_27050013
gaatgccctatcacaggggagggtcccatactggaactggatcaacagctgtcgcctgtccctgg
aatcacaggaagcggaggacggggtgcctttgtatagggagctctgaagccagacaatagtttgt
gctggcggggccatgtggcgagtcatgtgacctggggctgttttctgctggggtccttacccatc
tgttc

>chr1_36839613_36839813
cagcctgctcccccagcacatcctctgccaccgctgcctttcctcagcaggactccaatgtgctg
agcacagcaggcctgggggccaggcagggcctgaaagtccagggtctgcagcttggtgacctagc
tctagtctcctccaggaagcaaggttcatgagttagccccacataaggagactgcgtaatcagtg
acatc

>chr1_36840013_36840213
atggagttagagaccggtccccatagccctgaaccagaccggctgctcctgggagcaaagggctt
caccagcagtcacactgagagccaccagagcagaatccagggcagcattgccagccctgccacca
gctctcccctcccaagggcctccggaagcagctgatgtgacaacaaataatctgtcactgtgtgt
gggtg

>chr1_36840213_36840413
tcagaggaggaagggacagacttacaagctctttctctgggccgtactggcttggcctctccatc
cagagaggtagcctgggaaagcagccaagcaccctggcttagggatgtttgcggtggccccttgg
ctcttcagggtgccttctgccatgcgcagtgacggcagactgagtcactggcaggtcttccctgt
catac

>chr1_36853013_36853213
gaagggcttcaatgaggtgggtgaccaaaagacaggcataaagaaggccccagcccagcaagctc
cactctctgacaaaacaaaaagcctagggattccggaagccctgactctgacaaaaatgagttct
gggttttccagagctctaattatcagctagatgaatcactctgcgctgacaccaccattcctacc
cccag

>chr1_36853213_36853413
gagactctcagctgattgaaaatccaggattttaacaaaggagaggaggaggaacagaaagctag
gaggagaagaagtaatgaatagaaaccaaaggtgccagccagaggaaatgagaaactgtcatcag
gacactaccatgaggagaacgctgggacacacacacacacacacacacacactcacactcacgct
gacat

>chr1_36853613_36853813
cttctctttgtcaccaaatcgcaaggcctaaatctggcaaattttgagtgtcagacctacaccct
gtggctaggtaccaccactgtggtgaacaaaggcacctcttatactctgtcatcagtatgatgcc
aagagtgctgggaaacagaatagcacagcagttaagatcattgagttggagtcacacagacctga
gttca

>chr1_37943413_37943613
aagctagctgtagggggggcactccaggcctcatgtgctctaccaggaggagggctctgatagcgc
ccactgatcagtttttccatctgctgagggaatattcaggtgctagggctgctgagaaccgactt
gtcccagccaagattttcagtgcccacaggcagtgcactgccctgagagccctggcatcccatta
gttcc

>chr1_38465613_38465813
cctcctgatccagaaaacagtggaatgctagagaggtgctggactaatgaatggtagaagaggcc
agtgtgagaggttgggccttccctcccggggaatcagccaagcaatgggaacttcagaggctcag
cttaggttcaagcaggaaggaaatcctgcaagctggacacagccagtcttccctagctagaccct
ctgcc

>chr1_38466813_38467013
ccagggcatgaggccaccaggggccagggtctttagagctggaactcctgggctgggggctatgg
tgtagagaagatactcctctttctcagccccccttcctgccactcaggcccaggtttcctctggcc

caacatctgaggattgcctgggctgtcagccacggggcagcagggggccacacaggtggaatgcg
tccac

>chr1_45272613_45272813
tatggaagttagccaggtacgctcggagtcttagagactgccatttctcccttccacccgacttc
tctggaaggagaggcaggcctctgcctcagcctggactgtggaggcagagcctccaacccctaac
caggaaggactgacagactcagctccttcaaccccacagatccttccagccctgggggtgttgaa
tccag

>chr1_45273413_45273613
gaggcctaggttagggggagatcttgaggctgcgggggtggtggctcaggaatccagagtctggg
cccacaggaaggaacacgtcatggctctgttgtctccccatcggcctcgacaaggacaaacattt
ccgctcccggcgcagagttagggtggggcttggatgacttcctcagttcagggctatgtgcgggg
gaggg

>chr1_53791412_53791612
gcctgtccacctgtcgccctcctccctcagaccccgtcagtgctccctggctggcagggctggcc
tgtcttggagggcgccccagcgcctggcacgcagcagatgctcccgaatgatgaatgaatgactg
cccgggtctatgtctctaggagagccctgtttttctgttcccggagctacatttcttccaccgtg
acaac

>chr1_90372212_90372412
tggaaaaacctgcctgtgtgttgtgccaagttgtgttagccactccatgttattcttagatgggc
tctggagtatgaccagcctgtgtgcatgcatttgtagtaggcactggataagaactggagtaatg
actacagaagtgaacggtcacagctctaacctgctgagtcatctcagctcagccttgctagcaag
gcctc

>chr1_110546477_110546677
tgtttacttctagttggcagttgcaggcctcgattactcattgactggagcactttaggagataa
tagtgaagtgaagtcagtgaagtgtttagtcttacaggaagagaggggagtagctcacaagggtc
aaactactctttggcagagtttttcaagctgtgtgttccttaccttttaagtgactcagcagttt
ggggc

>chr1_110546877_110547077
ccaagaaactcaggccccatctggggagagtgagaacttaagaaacttgagacagggaaggagag
ccgggagcagccaaaacctgctaagtctcagaagactggtttctgcacaagggaatggaagaagt
gactggaggtggctgtggattcaactctggcctgagattgattcccttgagataagttgttttct
ggtgg

>chr1_145455643_145455843
acaaagggaagaaccaaaagagaaactgcaactcagccacacactggtttctgtaactggagaac
aggatatttaggggggacttgctctgcacagacatacctttcaaagtggcatctccagagtctgga
agaggagggagtatggtgaataagtcagctcctcctccctcctcatacctggttttcttgccttt
cccca

>chr1_145456043_145456243
tgaggccttgaatattccttggctgggctcgattgtgaatatgtgtcccaccatgggggtagaag
ggaagggacaaaggacaagcgggctgtagggctgtaaccctccagtgtgtcacaccagacccggc
ttagttaagcatttccttcattcctccagccctctgtcaccaggcctcacccacaacagagagac
agata

>chr1_150539976_150540176
gtttccctctttctgtttctctgctttgcctcatgagggatttctgagaaatgaaaaaaagtcac
ctcccacagccgcagcctgcagttttgtttttggttttgtttttacagatgaagaagagggggg
tggaggaaaggagtataccgcttaagcatctagtattaatccaaaactctgcaaagtaagagaaa
tgctc

>chr1_154943376_154943576
tagggctatccaagcccctctcccctgccctatacaaacaactagtaatctggggagaagtaaag
tgggggtgggagggaaggatggaagagtgggacggaaaggaaggagataggaggtctgggtcact
caaaaccggacacacgcaggccaaagggggcaggaagccaggcaggagtacaggggaatgaggga
ggaaa

>chr1_154943776_154943976
tagcgaagtggggatgggaagtaagtggtcccaatggatgcagagcagggccaagaggaagagca
aagctggtgaaaaaaggggaataaagttcaacctggatgatacatcaggaagctaccaggcaaga
agggaacaagaggacatgctggtcacggcttgttctctacgtatgccatctaccacaccaagacc
gatgg

>chr1_154944176_154944376
tggaagaaacagttctccaagagaagtagaaactacaggccaggaagggacagagagatcagaag
acacatctgcgtcaataaggacaggagaaaaataagtacaggagaaaggggtgtcaggagaagat
gggttgaagctggacacagggaacagtcccaaacccattcctgccacccacggcttcctgccact
gctct

>chr1_156074576_156074776
aaccttagctccaccaagctccaaggggagagaagagagggcatatgggaatgttttgctggacc
ccatactgcactcccaggccaggaagctctgcatcaggaagccagcaccattttcacctcctctg
gggtaggactgaggggaccatggccaagaggaaacagatgccccccttagctcctccctgggtagc
ctgag

>chr1_156074776_156074976
cgggccagggcctgagagcatgccagttttagcctgtcttcctgtccttcccagccagacccctc
tcatcctcctcccccagtggttttctcattacctgtcactgacggagagcccctcagaggtcaag
gccaaagtgaggtgggctcttggcatgctgtgcaagcttggagctcacccggcaaagagggccaa
tgggg

>chr1_156075376_156075576
gctgcaggggctgtggactggagtggattcaccagggagcaaaggaaagtgaagtttcagggcct
ctcagttgcacgggccctttcaagacccgaagaggagccctgccaaactttccacatgatcccat
gtccataacatttgcaaaaagatcattttgtatttcttaaagagggcctccccacaaaattgtgt
gcact

>chr1_156092376_156092576
aaagggcaactcgttttcgatgcctctcccttctggacggtggaaagggctgtgtcatagagtag
gaacgggagatgcggcacaggaatggctcccattgacccgggttggggggctagggcgaaggccta
ggagaggcagaactgttaccttagagctggccaggattagagaacagtgcctggaaccgggggga
ggggc

>chr1_156092976_156093176
taggacacatgagttcctttaggtctggctcaggggagctagacttcatttcaaggggtctaggt
tctgggcagttgagaaggaggctatttggggtcaccaaggctcccctttcttcccaaagctctaa
cactgccaccttctgctggctaggagagagctgtgtcttctgaggctagagctggaatgcagtga
gacca

>chr1_156093576_156093776
tgccctggcctccggcctcaggcttctcctgcctctgtacaatgccacgttgatacgcccagcag
ctgtgactcaggcctggccccctgccaggcccagcacttctactggagttgcgtctgaacatgtc
aacaggcttcctatccctctctcagcaccagttctccccacttcagcccctccctctgcctggaa
ttaaa

>chr1_156096176_156096376
ccgggcctagcctgttgcccccagcaaccgggcccaaacaggcctgtggccggccctggcttcca
tatctggcatcagagttgggctgagcagggtgactcagagggtgggtcagcgcctggcccggtgc
ccacctagcccctttgctgtgctggtgcctttcttccccaaacagccccaagggcccgggcctgc
tgcag

>chr1_156099976_156100176
aggctcctcagagggagggaagcttggccaaagggaagtgagtagagtccagggagaaggctaag
taaggccctgtgtgggaaggggcaggagacaaaggtacccctgtctctttgggaaagaatgggag
gagagagagggaaaagcattcatatcacggggtagagctctgcccttggccccaggcacgttcct
gagcc

>chr1_156717576_156717776
gtgggtgaaagttcagaaataaggagaaatgaggttctttgcagaagcacagactgcaaagtggc
tacctggccaccgtctcgtgccaccccagcttcccatggaaagcacactcagaggcagatagctg

aatggtgtggcaggggacccaggaggccaccagctgcccagccccagcagtgggggagaaacaaa
agaac

>chr1_234859777_234859977
cctaaaacattgcatttaaagaaagtcagaggcgagggggaggctacaggaagtgagtgggagta
gagatgtgtgtgtgagagagagagagagagagagaaatggagggagagggagggaggaagggaggag
ggaggtggagagagagagagaaaaaaaccaccctgctggcaaatgaatgcagcagggatgatgtcat
tggaa

>chr9_35073000_35073200
gtattagtctagtcctattgtggtaacccatacacccatcttctcccaaaacctggagatcttct
aagacgccatcaccaccctgacacttcttgctccttctcaacgctgcctccaaccacccctactc
tgaactttctgactagcaccccactcctaccccattgtcactgcaccctctggtaaccccaatcc
cacag

>chr9_35073200_35073400
cctttgcagtttctcagcgcttgtgcctttcttcctcccgtcattaatattaataccttataata
aggcactcaaatccagagaagtcaaagggcaaccacagaacacacagccaattcagtagaaccca
aattagcaagaaaactgcatatgctgggacttgaaaatccttgggccccaaataacaggggtccc
tgtta

>chr9_91932580_91932780
cttaaatccacttgcactttcctccaagcctttgagtgagaggaaagttcctcctccatctattg
aaatccactctgcattcttccctgtacttttggtcaacctcctatccctgccgcactttatacat
ttctctcaatatttaacgaatttgctctgtgtagaactttttttttctcgtgaaaattatactat
tccat

>chr9_99183179_99183379
taaaatgaggctgaaacctactgggctgcattcccagacggttaaggcattctaagtcacagcat
tagataggaggtcagcacaaaatacaggtcataaacacattgctgataaaacaggttgcagtaaa
ggaaccggccaaaactcaccaaaaccaaaatggccatgagagtgacctgtggtcctcctcaactg
ctaca

>chr9_136008979_136009179
cctccccaccgcaactctggctgccttcaggagctgggcctagtgagggccatacatggcacaaa
catttccagctactgtgcatgaagcttacgacgaccctgaccccaggcttctcccaggacacacg
acctaccagtggtcccacgatgctctgcccagcacgccgggtggcctgggataatgcacctggcc
acaca

>chr8_103801224_103801424
ctttgggtggtgcacaaataaccagttactcagctcatgtccctgaattggcaggacagccttgt
aggccccagggacattcagtgacctcagtctcagagaatgtgggggaattccatgagaggcagga
ctgcacagcccagcctgaagggaaggtcagggcaggctggggagttgacagagccagctgaggtg
tgggg

>chr8_103801424_103801624
tggtgtactgtaccccctgggtgtcagggtggcccccacaagcccagggaagacagccagctgcgt
ggtctgcaggccaggcgcacttgcggatgttgccacactgagcctgcctctcccgcactggggac
tgcccccagaagcctgaattgttttcaaggcttcctactgcagctgctgggccccgcctgtttcc
tcccg

>chr2_87930085_87930285
aaacaaacaaaaaagaaggtaatcagtgtggatgaggatgtggaaaagttggaaacttcagacat
tgccgttgagaatggaaaatgatacagctgttttggaaaacagtttaatgtttccttaaaaagtt
gaatagagaattagcatatgacccagcaattccatttttatgtttgtaccccaaagaattgaaag
caagg

>chr2_88301085_88301285
attcaaaataaaataatgagtacacgttttggaataacgtcctattaatgaaaagaatgggatcc
attttgctgggataacattttgcctagttgaggttccaatgtaaaatcttctcagatcgagggca
gtaaaaaagctggatttgatctgcaggtggtagtgtgccggccctccaagccctatctatggggt
ggagg

>chr3_126780310_126780510
acatccttgccagcatttggtgtttttttttttttaattagcatactgttcttgttttatagctg
tagcatttctctcatttctcgaaaactattaattagaatttttggtaaatttccttgtgtttcct
gctgtaatgttggtttctttaatttccttctctatgtttactttgttttctgtctctcaagctgg
ggctt

>chr3_171979306_171979506
tggcttttattttaaatttaatttgctcaaccagacacacagtgtagcagacgctattaccgtat
ttcagtttttggtgctgtagtgagactttgaaattctaggaattgagtagataacacaagtgtga
tgtgggaaaggtccctgggaaacaacagtgattctattaggctggtgcacaaataattgtgggtt
ttgcc

>chr3_176244106_176244306
gcctctttcttggatcaagttcatgatgccagatacttttctcttttgtgataaggtctcttttc
ctcattcctcatgtaaatgggcctttggcaagtctgtccctccggatcaccagatatgtcttaat
cctgcttagactcaaattcatatttaaactgggactcaagccagatgcatttttcaatgtcctta
ttcat

>chr4_114130551_114130751
agatgcaaatctcccccacaggacagctttgcagggctacttctttttgtaggccctctgaacgg
ccatctcaaaatatgtcaaagaagaatatttgttatcgaaccaaactggggcccactcgccctgt
atagcaataccagatatccacaccaaggttttcagcgggagaaaggaaggtgtttattggcaggg
tgcct

>chr5_172834994_172835194
ctttctggttgttagttttccttctaacagacaggaccctcagctgcaggtctgttggaataccc
tgccgtgtgaggtgtcagtgtgcccctgctgggggtgcctcccagttaggctgctcgggggtca
ggggtcagggacccacttgaggaggcagtctgcccgttctcagatctccagctgcgtgctgggag
aacca

>chr7_70005464_70005664
cggggaagcacacagtcgtctctgggtttctggtgaaagtccattgtgcctgctcagggctcct
tggtgcaggagcagtcaagccttcctggaggttgaagatcacgtgctgtgtttggtggctcccca
cttgggtttccatcgattcatggtattggtttgggaggtgctcacatcctcctgcaagtaccctg
tagtg

>chr7_107615764_107615964
caccgacactggccagcaatgagaccagtagaaaaatcagtatagctgtcacaaattccctcatt
ttgagagccagctgggtcacacgtacatcctgagaagaatgcaaagcaccaggttaaaatcagga
ggaaactcaatgtatctttgtataaatacatatattcccttcctccctctaaatctcacaaaagt
cgact

>chr7_152109267_152109467
cttgaggcaagagtttgagaccagtctggccaacatggtgaaacccgatctctactaaaaatcca
aaaattagccgggcatcgtggcaggaacctgtaatcccagctactcaggaggctgaggcaggaga
atggcttgaacccgggaggcggaggttgcagtgagctgagatcctgccactgcactccagcctgg
gcgac

>chr9_132698579_132698779
ttttattccctttcctctaagaatgcactcttttgcccacagacaggatgcctgtaatcattatt
cagtgagcagcaacctgcagcagctcctcctgactggcagatgggcctggcggccacccagaggc
tggggacacagcaagaatccagcacagcaccgatcccgattccctcctccccaaactacctgagc
catgg

>chr12_45048333_45048533
aggatgtcagttcctattggaatgcagctattccacaaccagattcctgacccacagacacagta
aggaaacactcttcaagcagtcagtgtggctgtgtctcaccccaacagaagcacacaggagagat
gaggcttctgatttgggggaagaaactctatctcagggcccaaaaccatgacttgtcatgaatgt
gacac

>chr13_35509600_35509800
atgggtttcactgtgctagccaggatggtctcaatctcctgacctcgtgatccgcctgcctcggc
ctccaaaagtgctgggattacaggcgtaagccaccgcgccttgccttagatcactttcatctggg

tgggaacatgaatatattcatgaaaacggcacttccactggcaagaacttcagtaggggagatga
gaagg

>chr16_88291899_88292099
acagaggaagggatttcaagaccacctgctgacatttgaggattttgctcaaggaaggttcctgg
aagggaggggccactcccatgagacagcacagctcagcagagctgaatggctcacccccccaccc
ccccagcttacccccctacattgccgatggccttctctgccagggataggggccctggagccatcg
gctaa

>chr17_18312675_18312875
tatacgaggaggtcatgttaagtattccacaacaaattaaagagatgatgtccatgaagttatct
atgctactctatataacagatggaaaaagcttgggtaatattcagtgcggatactgacttgtgtt
cctgactcataggcggtgctcagacacatttaacccactgtctgacagaataaatgaacgtgtcc
tgtaa

>chr17_71087405_71087605
aaaacacaaaaattacttgggcgtcgtggtgtgtgcctgtagtcccagtgcctgtagtcccagct
actcgggaggctgaggcaggagtaatcgcttgaacccgggaggcggaggttgcagtgagccgaga
tcgcgccactgcactccagtccgggtgacagagcaaggctctgtctcaaaaaaaaaataaaaataa
ataaa

>chr22_28076200_28076400
tacaatggaagctccccacagggtaggaactttttccacccgttcactactatctcagagcttag
cccactgcctggcacatagtagatgctcaataagtacttgctcaatgagctgtattcgctggggt
aacattcgatgctctaacaggtaagccctaagttctcagtggcttaacacaatcattttgttctc
tgcct

>chrx_137749134_137749334
aatagtcataataacgagtaaacagcaggtttgaaacatggtatgcatagttattccaaatgtac
atatatatctttatgtaatatatatgtattttatatatgtacacatatatgtgtatacatatatt
atgctatatcatatattaggttatatatgtatattatgtatttatacatatatataaaatacgta
tatat

>chr1_17662413_17662613
ctgaaggggaacaggtgtggtcagaggccccagctctgctgtccgaactgtagccagacttcctg
ggggccgtggaggagggatgtcttgaacctgtgtctcctctgaaggacgggaagaggggctcaca
ctatgggtgcacacagtcccctggggaaacgacctgcccattcagggccaggctgggtgccccaa
ccccg

>chr1_19679413_19679613
ctggcctaccaccagccgcctacactggggcacgagtgggcacagggcatagcctgatgtgccca
cggaagggacccctgcgaggtgccagcaagccaaagctggcggccttctccagatgggccgtggg
aaacaggaagacaagggtgcgcccggggccacacacagggaagggcagctgccgctgctgccggg
gtgag

>chr1_44018613_44018813
tgtctaggcctttgggactgctgctggtatgtgggggctgggagagagggaggagtctcggttcc
tggccggagccccggggtggatggtggtgccatcactgagatggagagcaggggggagggacaact
ctcagggagagctggagctcttcccagcagctctccagcacgcctttttcctggagcttggaatt
gatgt

>chr1_55279212_55279412
aatggatgaaaaggagctgggcagtagggtgtggcaggtgcatggtgcaagagactaaccccgcc
ccgacccgctggttggggaaagactgtctgtttccaccagcccttgtcaggggggccttgaggct
ggcccggcctccggcctccgggatgtctgcacagcaggatagtaagtgatgccccattgcttttc
tctgg

>chr1_226661977_226662177
tgcagatacacccagaaacagtgcttcaccagccatccaggcatccctcaatccaggcaagttaa
cacctaaaattaaccatcacagttactaatatgggaaaattggaggactactaataaaactcaga
ttctcagcttcttttgatgattggaagatctgacatcactgtgcccagctagctgaatgataata
gttgt

>chr4_688200_688400
cttaaatatctgctagccataataaataaatgaatgtacttttttttcttagctcccacaagtta
gcctaaatatttgccctggcatacttatactggtcccagcaagcattaggtcacagtctgttcct
cttccttatttgaaggtgtttttacctttctcagcattccacaaattacttcctccttcctttgt
tctcc

>chr4_81383176_81383376
ttaccaatctaaatcctttggccatcctgggattcccaaagggcagtaaccatgctaatttaatc
caaccttggatccccaacacttagtgcagtacaagacaaatgggcattcaaaaccatagtctcaa
atgagaagataaatgcgttttctctcttgtattttcaacatcttccttttcactgacactttccc
cttag

>chr5_115200301_115200501
tcctgctgaagaaaaccgtgtctatatgttgagcatgacttcacaggatttatgacacaaccaat
caaagaaatcatggaagagattgtgaatgtagcaaaaaaggtgggagaatgaagggtttcaagat
aagggtttcagagaaatctaagagcgcatagcacaacagagtaattagcagaacacattttgatg
gagat

>chr5_129492101_129492301
tttgcattttcaaagtgttttgtctactattaattagcatttcttcgcagcatacctgtgagatg
ggtcaatatcaccattttacagatcagaaaagtggaatgacaaaggctgagtgatttgaatgaag
gactataacaagtcaaaggcagccaaggatggatttgtggcaccagcctgggagttcaaacatat
catgc

>chr5_139021216_139021416
ggatcaggggatatgaagggggtgtcccctctttagtatttcaagggaagtagatagttacagcc
ctaccattccccattaggccatttgaaagggaaaaaagttacttccaacaataaacagttccatc
taaccacagccccactgctgcactctcccttgctgtagtctctgggcctctggacacagttgcag
tctct

>chr6_9086201_9086401
attcaagttttattcaaccagttcttaaaaatgagttctaaacaaatagtgattttgcattagc
ctgcaggaggtcatctcttacaatggattcatagtgctttgccggtgatgagaattctcacctcc
actgggttgtgcatcaggaatgacctcgtcactctgctgtatcacagatgcacccccataatggg
agaac

>chr6_143377107_143377307
ttgctggatgtgttgggtttggatacatgcacacgaagagagagcattccaagcttgatgaggag
cactggtaaatgcacttctagtactagagagtgacggagatgttttgcatctgtatgagatcatg
caagactcacgactcatctagaccctaattttctaacacttttcaatgaccctccatagaatcc
tcggt

>chr7_15821475_15821675
taatagacatgaagccagccatgtcacctcaaatcagtctccttgaaggcatgtagttagcggtt
ttacaaagggcacttgctgctgataggtaggatggagacaaaatcataagttgttctcttgagta
tagttgtttctgggtggggccacggcaatgggagtcagcaagtacagatggagtcactgtgtcca
catac

>chr7_30394075_30394275
ttccttcttaatgtgcagtctcaaatgaataattaaagggaagaaccacttcttatagaatgaat
ttttctgcagggtactcttacagataatgtacacaatcaaatttgttggttggtttttataggag
gaatgggcatatcaaacaaatacttaactttagaaaatgtccctgcctttctgtttctttcagaa
tttac

>chr8_7441190_7441390
cctgaaattacacgtctactttctttcccaggctggcgctgagatgggcaggtgctgcagcagcc
cggctggaagcgatgcagcatccaggacgacggaggaaggggcagagagggacctccgctttcca
ggctgccttttatactgcctctggtcacctgacatggaacgtaccctaacctaatcagttacctg
tacct

>chr9_33415400_33415600
tctactcccgccctgcacgcccctacccacaaccctggcccctagttgagcctctcactcctttc
acagcagctgaggcaacccagccaagagcctcagtgagatgggtttgggtaaggtggaaccttaa

cccccagactcccaacctcatcctgttttcagcacccatgagactggctctagcacccccctgctg
gcagc

>chr9_132268579_132268779
ttctgcgagatgttaccattggggaaactgagtgaaggcgggtacctgggatccctctgttttct
tttttgttgttgttgttattttgagatgaagtttccctcttgttgcccaggctgggtgatccacc
tgcctcggcctcccaaagtgctgggattacacgcgtgagccactgtgcccagcctctctgtttta
tttct

>chr10_48431194_48431394
agctcactccggtggtagcggtggtgtatctgcagcatctggggttgaccgatggatggggtgaa
agttcaccaataatgttgcacccccccatccccaagctagaaaatgccctaggaaccaagctccc
aggactcagtggcgccacgccctgaaccttgctttccccatctgcctcacagggacaacagcctc
catct

>chr12_1953139_1953339
agagcagggcagccccactgacttagtgagtactgcagcccctaatgaaaccacccgtctgggtg
gtttatgaagctttcacagacatgcaggataaatagaagaatcttagagatggactcttctgatc
tcatatttggaattggggctagaatcccatgctgggtttttaaactccaactttgatggctggct
aggga

>chr14_67665647_67665847
taaacactgagctattacctctaactcttaatacattcgagaagctgctcagctgatttcacaga
ggggtgacaagggaggacaacagacacttcaaaaggcaagaagaggatggcaggagctcttttgc
catggctaccaggccgatctccgagcggaaccaggatgccactgtgtacgtggggggcctgaatg
agaag

>chr15_40530308_40530508
gcggaagactgaagactcacagcctggacttttaaccaaacaccacatggcttcgctacagaaga
aagttagaaagttttcaaaaataaaatgtagagccaagtgccaccttggatataagggactgggg
gacgggaagaggcaagaactcccaggcttccctctggctgtcaatagctagtgggtcctggcagt
gggag

>chr16_19328299_19328499
ccagagtcatcactatttgctttacaatagaaagaatccaccagtcacatgggcctgtcaaagcc
aagacttctggggagcattggtctgcattaataatacaatagatccactagggtgagtctccagc
tgcgctgtatgtgtggatgccagggagagattgggagaagccctgcaggagccctcagagactcc
atatg

>chr16_84592099_84592299
gtttggaaatttagcacaagtgttatcactgttccccgatcactttgtaagctgccttttatacc
tgcctttatgttctggggtttaatagagccatcacagcttgtcttgcctgtgcgtgcacgcagtg
gtgtggcctgtcctccgcagtgacaccggacatgggaactgctggggatgaaccaaggagcaaag
aattc

>chr17_77025805_77026005
gacgaacaaagcccctcactgctaggaaacgggttctctctggctcgcctaattccagcctggat
gaagtgccaccctgctgggctccctaccacctgtgctgttcctggtcctaacacatcccatacca
tagctgccagggactctactgcccaagggaaagggcatcttgttccgttcgtcccaagaatccag
ctccc

>chr19_10196200_10196400
gttgccatctctgtgtccccaggctgggccagagccccaccacctaagggctccctgatgtgccc
tgtactgggttagaattgtccgtcccacggggttcttaaccattgcagattccctggatccctga
gacagccggattctaccgggctactagagaccaagaatctgtatttttctcaagcaccgctcccc
ccaac

>chr22_26966000_26966200
aaagaaagacagaaagaaagaaagaaagaaggaaagaaagaaagaaagaaaagaaagagagaaaa
aaattaattaatcacactttgtcaaatgctgaggaatggtgttgctggctggggtatgctacccg
ccccactccaccccacacactttttttttcccggctgctgcagaggaacacacaagactctttgca
ttcat

>chr2_85683889_85684089
tgaccaggggcagacttagctgtgtaaggctggtccagactcctccagcagtcacatagatcagc
tttaatgtgcccacgaatccctgggcttcttgtttaaatgcacattccagtgggtgagcctggga
taagcgggcgatctgtgtttctaacaagctcccaggtgatactgatgaccacactttgagtaacg
aggat

>chr2_135583330_135583530
aacggcgcaccacgagattatatcccgcacctggcttggagggtcctatgcccacggagtcttgc
tgattgctagcacagcagtctgagatcaaactgcaaggctgcagcgaggctgggggaggggcgcc
cgccattgcccaggcttgcttaggtaaacaaagcagcctggaagctcaaactaggcggagcccac
cacag

>chr3_183544706_183544906
taggaataattctgggtgccttcaggaggtttagtgtggtagtgagggacggaaggaagtgtaat
tgaactgacaaggccaggtcagcatgagagggaggcaggctggggaaagctggactcgccacctg
ttgtgagggagggtggttgggccaggtgggaatgtaggtcacgggggaggagggttcagctgagg
tagca

>chr5_714800_715000
acactgagccatcttgtttcaatcactaatcaagctctgccctgggagcttgctctttgctgtgg
tttggacatctgacccttccaaaccttatgttgaaatttgatctccattgttggagatggggcct
cataggacgtgtttgtgtcatggggctggacccccctatgaacagattaatgccctccctctgaag
ggagt

>chr5_128734901_128735101
cctccacaaagagctgtcacatgactttggcacctcccaattcttcccccctgtcactcagctttt
agacagaagctggctttttctgtagtcctttcttcacctagcgcatgcctgaagcctggaagct
ttgctgcaggcttaaattcttaagtatgtaagaggttatagccattcgaggaaaaataccaaata
ggcaa

>chr6_15208221_15208421
gaggatcactagagccagagagattgaggctgcagtgagctatgacctcaccactgcactccagc
ctgggctacagaacaagacccagtctcaaaaaaaaaaaaaaaaagagaaaagaaaaaaaagagga
ctgagtcagccttttcacttattatccctctggccctcattcctccttttggtttgagaactaag
gtttt

>chr6_73839479_73839679
cttagtaatgtgctgctctatttccctcactcctagtaagttctgtagtaataagcagaagctct
tcagaatgtacacctcacggaagcagaggtacccagcatccgggctattgacatgagatttgaga
atgccaactaacctcaggtgcatgaccaggttacaccgccaccccacctgagccctgttcagaga
ctcac

>chr6_133289107_133289307
agtagtgacacatggaaagataaagctatttattctttttagaataatggttagcaaactacagc
ctggccaaaatccagccattcccctggtttcataaataaactttattggagcacagtcatgttc
atttgaaaacatagtgtttatgactttcatgattcaatggcagaggtgagtatttgaggcagaga
ccgtt

>chr6_142469707_142469907
cgctgtgactacatttgctgcatttttggagcttacatacaatggaggtagagagtttcaacaat
atagagtctatcatacatagagtattaaaagcttatgaagacaaatagagcaaggaaaaggaata
agagtatcaggagtagtggtggcagggagtgcagatgggatttcagtttcatagtggtcaggaac
acttc

>chr7_139654931_139655131
ttagtacctggctgtgttctggttgaccacagctgcctgaggctcgaaggtggaagaggctgtgc
cctcctctcctcccaggtctttgtttagacgccaggtcttcgtgtctcctcccaccctctgccgt
cactgctcagagtgccccatccaggagcccatctccctccacctccccccagatctgcagaagct
cctct

>chr10_83660820_83661020
catggtgaagtcgggagctccagatagaaatccaaagagagagaaagacaggggcagggaaggta
cagtgtggaataatgtggactgtctcacatgacacttaagaggtcatgtgatgctagtgactttg

ctgatgtgtcaaggctagggctgagaggctgtgtccagcactgtcatctccactacctgaggcag
aagca

>chr12_117556417_117556617
ctgggcgtcaagagtgaaacttcatctaaaaaaaaaaaacaaagagttctagagttctaaaactg
tgatgtgcataacattcccctggagcctctggtgtcgcaggaagggttggggactctggggtcag
tacctgttagaaacccccaggtgatggtgaggttggggtgagcaggatggcaaaatactctgccc
cccac

>chr12_131912647_131912847
gcataacaaggcacatcttctggaggtgtaacacggcatagccacagcggctctatgggcagaga
accctcagaccctgcagcaaaggacactgtgctctgcctggattccctcagcacctgggtgagct
acccacccttggtctcccctccagtgggcctcacaccagcgcccatccctgtctaggcacagacc
atgtg

>chr13_108215199_108215399
aaaaaattaccttccaggcatttcccagttcccatgaggagtttcctctgtgttccacttcctaa
ctgccaaagttgacttttgaaatgttggagctgagtccttcttaaaacttacatgagtaggcttt
ttgaattcagaggttggaaggcttcttcagatgaacaagttaatgtaagtaaaataggtttaatg
agttt

>chr14_78121247_78121447
gctggctctgagccatcctggagatataggctggcttaaacctggaagggcctcctggctggccc
tgggataacataaagcaggctaagggacttaaaggcatgtgggaacacagggagcttgggaggtt
ttgctctttgcttttgaggtttctaagccaggaacaacaggatcagatctgtgttctggaaagca
ggttc

>chr16_52344499_52344699
ctatcactgcaggacagtgcccctaaacttagtggcttaaaacaacaaacacgtattttctcaga
gtttcttaggcactaggatctgggcacaacttaattggatgtctttgtctcagggcctttcacag
gactgcatcaaagtgtggcaggctgggactgcagtcatcttcaggttggatgggacaagatggac
ttcca

>chr17_35969487_35969687
caggccccacctcgccgcggaccgggcgatggggcggggcctcgcgcccacgtgactcccccggc
cctcggggaggcgtggcgcgcccacggcacgtgacccggcgccgcaggggtcccccagattcaat
tctagctctcgggctggtggggcggtcgcgggcgcctctccaagggagctgattcattgtctggt
aggcc

>chr17_55859601_55859801
tttggaaagaggaaacatccggctcctcctccctcacatctctgcttctgggtctgggcttgtgg
gtcccagcttgggctaaggagggccatgctccgggtgggagcagctgtgtccccaccccctcctt
cccatgcgacttgaggccctctggtcaggactggagctgtcacatgcagccagcagctgtggtgg
cgcca

>chr19_709000_709200
gcccccgcgccgcgcagccccggccgccaggccttagcccgccccggcccccgccaggccgcgtc
cccctcccctcccctcccccgcgcgccacccgcgcccgcccccgcccggcaccgcggacccaccc
ggacctcggcggggagatggagtgagtaggcgcgctcgggccgcggggctgggggcccggagctc
cggga

>chr20_12576800_12577000
actcaagctgatttagtaaataggaaatatataggcttgcaggagctaaaaattcaggctgccag
tctgtctttagtcactatgaactccatcagctcaaatgagatcccgagggtccagcttttctctc
tttctcttgcagctcatttcccctgtgttgatatttctctcatggccacaaatggattctgatgc
tccag

>chr21_33286729_33286929
aaaaaaaaaaaaaaaaaaaagacccggccctagcccgcagtgaatgaatgctagccctttgttct
tacattttactcttctgcagggaatcccgtagaattgtaggatgctcagatggtctattagcct
gctcaggctgccacacaacaaaataccatggagtgggtggcttaaaacaacagaaattcatgtttca
cagtc

```
>chr21_38164930_38165130
atgaggcacctgctcagagaactgaagttactcatccagggtcacatggctgtcaagaggcatgc
ccagtacctatgacaaacattcatccccaagtaacgaagccaacaacctgtgtctcactccagaa
ccacagagctgttacaacacgtggtgcctcctgagccagcagccaggggcaagaacgagaggatg
caggg

>chr22_49079288_49079488
gaggccaagaggctctcttggccaaagtgagtttacctgaagggacgggctgaggccagagccag
gtcgttgggccacagaccgtgcactcgctggttcctccacggctcaggaacagcctcccactgcc
aggacagcctaggaggctgggccagcaccttcattcagttcaacgctttctatcacagaactgtt
ttgaa

>chrx_78621744_78621944
tagtttaaattacaacactggacctgcttcagatctctaaatagagggaaggggatgttgttcaa
caagaaaaaaatttaaaaataattgaaaagtatcctcaaatttattttacaagagaagagtgtt
ttctttaggaaataccactctaaaaatatacaaggctcttgcagtaaaagggcacagtgaacacc
caaca

>chr1_59168412_59168612
tatttaaaattccctctaatcctgcaaccctaggacttgttgaaagactcatttctcaccaagta
gtttgaaagcagaatgaataattcagaattagtgtttcatgaaacttgtagcattcctactgtgc
ttggtactgatgcattgaatgagattgactcagtgtctgccctcataaaatcaatcaaatgtgca
atttc

>chr1_180209177_180209377
aaacaaaaagaacacttcatccagaaggcagttaaaaagcaaagggagttaaaaagcaaaagcct
cacaggaggcacatcaagctggcacatcaaagtacaaactttacaaaatattaatctggtctcag
agcatcgttaaataaacattattcccttttaaaattattataatagaggtaggggctcactatgt
tgccc

>chr2_84540689_84540889
gtctcaccgggctgtccttgatagaggtagcctgtttgtcatgttaatgaagagaaaatttccag
ttctgagttccacaaagactgttctcccccacctacagctaaagagggagaaaaatgtcagcagg
taaggggtagaattggagtgaaccacttccttacctttgggagcagctggcctaatgtcaatgtc
aaggg

>chr3_57193160_57193360
tttttaaagacacatttacatttatgcactcaggacttcaagtgtgaattctgttcaacagact
aaattctgagcaagataatgggccatctcaaagattccccccttggatttgtttcttcaagactg
gccctagctttaatctgcagtaagactatctgtttcaataggatgtgatcacttgagggcttgtg
tcttt

>chr4_3305802_3306002
gctccatgaggccggaattgaggcatttaatgtgtgtgtccctgatgctcagcctcttggtttct
ttggatttgaaatgttaaaacctcaagaagtgacatttattaaaaacccacgaaaaactatgcat
gatgacaaaatgctgacctgggacattttatgtattgtacttttcagttcagcctaatgtattat
tcaaa

>chr6_42151622_42151822
agtccctttaagtacagctgaccctttcaaaggcagaaagaccacagcctgcagcgtatttgctg
ccatttttcctgttgacaccactggtgaggatgccttggggaggaggaggccagggtcctcccca
tgaacgcccctcagcatcccggaagttatagaattattgctgccccctgcccccaaaactcctg
ccttt

>chr7_6413875_6414075
gcgccgagctgacaggttcctcttaaagccctgagtcccagggaagggacctgcaggcccggcca
cgcccaggccgctttagcgcgccaagatggcggctgccacgccccgcggccggagccgagtgggc
ccgagcgcttccgagcattcccgaagtccagagaaactccgggagcggcgcgggcgggagcggcg
cgggc

>chr7_21579075_21579275
gtttgggtggacatgagtcccattaaaacttggaacaggggtaaatgtcgtatttccaagagaag
gaaaaggtgaatggacactggggacagttagcagtttctgctacatcatcttcttttaaaaaaat
```

ctagctattatcacagccctatgtgatttttgtctcatatttcttctttgtcctcatctcttgct
attct

>chr9_99835179_99835379
gagatgtgagctggggtctgcagggtgcgtgtgtgtgagcgatgtgagctggggtctgcacagtg
tgtgtatgtgagctatggaaactggagtctgcagggtgtgtgtatatgaacaatggaagctgggg
tctgcagggtgtgtttatgtgagtgatgaaagtgccaccctgatgtgtgtttctgctctagaata
gcttc

>chr9_102485179_102485379
gaggcaggggggaagtagcatgtcagaagaaactgtgaaaacagtgggataaatcgggaatattac
aaaaagaatagacccaccctactatgggactgtggttcagagctagctctccaagtgtgaaacca
caaaaggcagctatcagagagtgattcttcacatgcacccccctctgtgcacagatgtggaggggc
agtga

>chr10_47694794_47694994
agttcattccctaaaacaagctcggatgaagcttccaacttggctgctgctacatctgcttttaa
atgattagaatattccctaactactgggtctaattgcagactggaatatgcagtgggcctatgtt
tcccccatgttcttgagtaggaacttctaatacctgattgttgtgttcatgaacaaccaaggtaa
aaggt

>chr11_12482824_12483024
ttgtatctacatttcctatgtaattagccagcttaccaggtcaagttctatgttctccccaaggc
cgttacaaatgctcagggcatgtggacacacacagatgcacacccacgcacaaaggcatggcact
tcttctctaagtaggagcagacttttaaaccaaggaaatggttctcattctcaatctaaaaagat
aacag

>chr11_122394790_122394990
tatagaaacaccatgtcacaaagcttttcagacaaagacgtagtggggggtgagttcattgggtgc
agaatagaatattttgatgggttgcatccaacccatttcccaccccccaactcctacatgctctg
ataaaagtcagatctgtaaagaacttcaaaggctccccagtctgacaaacagtaataaaacaaga
aatcg

>chr12_97855669_97855869
cttctggggaaggagagttccatctcttattttaactaaatgacaagctactttttgtttttcta
ttccttgtcttctagtattgctaaacaatcaaatgcaaggtattttgtcttctttcactccttaa
aagaaaatcaatcaattaatacacaaagataaaagtgagaaacctactgtaatctactaaggtcc
aggaa

>chr15_41058908_41059108
caggagctactggggctcagccttgttgctacagaactgcaggacagccagggcagtgaggggcc
tggaggcttattttttttttgaaatggagtctctgtcgcccaggctggagtgcggtggtgcaatca
tggctcactgcagcctcagcctcctcctacctggaagcctgggtggggagcaagcccagggaggg
cggca

>chr17_41996474_41996674
gacacctctccaggcctctgacttagctagatctccaccatgtgactccaccatagactccatgc
cttcttcttttgcaaaccctcggacacccaaacacctaccaatttcagccctttctgtgcaggtg
ccggagcccaggaagcacacatcaaggctcgcttgccagcagggtgctgccaataaaatgtagtc
acatg

>chr2_85683689_85683889
ctcagctgcatcatcagtcctggagcacctgacgggtgctggagcacacagcataatccccacaa
acaaccccttgacttgacttccctttccactctcctctcaccgcccagtccccagctctgtcagc
ctcactccccgctgatggccaagcttgtctcagagttgcaggtaaccttggcatttgtgccaaac
gttgc

>chr2_184375955_184376155
gatgcctgtgtgtttcccatctgaaagacaaaactgcctgtggttttggtttgtttccccccctgc
ccaaggacctgcaacagtccctggaccctgctgataggaatggttgtgctcactgatgcagcagc
agaaatacctctcactcaagaaccctcaatggtccctggaccctgctgattgaagtagttgcact
cacca

>chr3_78946510_78946710
acacatcaaaagcaatttccaggcaggtagcatgtttagaagtcaggacttggaatcgctcttat
atatccctacatatcccaagtagttagcatctttgtcattcggtagatatgttcatcagttgagt
ttacagaactttaaaaaacagttaattacaatcttaaattaatgagataccttttgaaaggactt
gtgtg

>chr5_126664501_126664701
tcacacttcgttgaagggaaatacttctcacatagataaagcaatgagacaaggaaataattgcc
atgctcatttgttacagccaaatatattaaagattttttgatggatgaggaaaacactagctgt
atattacacggcttcaactgtaaaacagggacaataaaaactactacttcttagttaagttgctg
taagg

>chr5_141122816_141123016
ttctccctggtagcagcatggcagattctgaagtcacaaaaactcctcgctcatcctgagagtag
agaactctacccaagttactgatctacaggccaagccacgtcgatcttctgtccccactgtcagt
gcttatgcaccctgggcagaggcctacactgccttttcggtaaaggcagctggcccctagccctc
cggat

>chr6_24693421_24693621
aaattgtcatccattaagattgtctgtttttctttgtgcttctgtagacagtcttcttttggggg
tggctcacgtgtttatgtacacagattttctcataccatatgtcacactcctgtgcttgaggccc
attacagacacccttactgagccagagacatacattcctttatctgtttagcagaggcagtggag
gcagt

>chr7_100241064_100241264
ctctgaagcccctccccacctcatcctcccctcctcggtctgtcccctttcatattcagagggag
agtatcctcttcatatcattcaatcaataattcattcattcaacaagtattattgaacagcagtc
tctgccctgagctaggccctgttcctgagctgggggggatggcacgcacagccccaggccaggcaa
cctgc

>chr10_14372194_14372394
cagccaaaatccagacttagggagcaaaaggctcaaggggaagataggccacctttcacacaggg
tggggaggaggtcagggaagacagggtgggaactgtgcttagggaggaaaaaaaaaaaaaagaaga
aggaaaataggaagctgcaggttatttaaaagtgagccctccctagcttcgcaaagcggcacaga
caggg

>chr11_6496824_6497024
cttcattatatgctagcctctctgcctgggatgctcttccctttaccctgcctgtttgtcatcta
ctcacccttcaggtctcagcttttttttttttttttttgagatggagtcccgctctgttgcccag
gctggagtgcagtggcgcaatcttggctccactgcaagctccgcctcccgggttcacaccattct
cctgc

>chr11_113731790_113731990
agatcgcaccactgcattccactccagcctgggcgacaaagcgagactacacctcaaaaaaaaaa
aaaaaaccacctctgtctggacaacagagatggttgctaacctttccaacctccataaaacaagg
aatggccctcaaaagccttggtatttttcagtttttcttccagcaacaatgccctgctgatcca
attta

>chr13_40153000_40153200
tcatggataagtcaacattcaaaccaggccttaaaaggcaaataggtttggggtctgtgaagaga
ggagagaaaggtgaagggaacagcgttcagcaaaggcacaacaatgttattctaatcttatgtaa
cctctcgtgtaactagtgcacaagaaacatcagaagattcagtgtggctaaagtagaataaacaa
gaccg

>chr14_74881447_74881647
atgctcgtgttgactctagattcctggtaatagaataacaacaggatagagggcctcctttttca
gctggggcagactaagtcatgtcacgccaaaccgccaataacagctagaaaagtggtgtgtggtg
ggtgggtaatatgaaggcttcaaggaatcaccaagggagtaagaactggagggtcagaatgccaa
gatcc

>chr15_50350308_50350508
tcctggggcatccctaatgatctgtttgcctcctatctctgctggctagagctcatctatgcctc
ctccttttaaatcaactggggatctagggagtcagccctcagctttggcccacgaattcgtctta

tcctgagctttagggatcttgtatgatcagagtgggcatttctttgagttgaggtgggggctggc
ttgtt

>chr17_32297287_32297487
gaaccagtggggtagttggatacctttcctcattctcgaagcccaggcagcaccccacttaaact
tctggagctgtggatgcatcttgaccaggccacgctctccttggtctctgcctagtgggatgctg
cacttggaacacatcccagagacaggcagtgcccacacccagctgtgaaacacactgctcccctg
ctgag

>chr17_77834205_77834405
gataccatgctctggcagctgagggtaattacgggggctgcgtccttgaggacgggtctgcgcat
gtgcttctcgcggggctggtggcccagccggggcggcctcggtgccgtcagctgctcggtgagat
gtgaagggccggggctccgcgtggggccttccgtgctgcagcagaggcctgggattggaatgcga
aatcc

>chr18_10087200_10087400
gggacacagaggctgttttcacaagacttacagggtggcatgggaatgcagctttgtaaacaggt
gatttcagcacccaccgtgagtctcaatagagaccatgaatctgcacagggcaaggaagcgctca
ctcagcatacgtatgttaggggcaagttatagaatggtttttagagttacaatccttatttaatg
gtgaa

>chr18_57246820_57247020
atttatgacaacggttctacatcagacaacacacaattccagagggccctcggcggttcctacat
gctctatggtggcagccacctgcctggagcatgccaacagaacggaatgttaggctcttactgcg
ttagtgtctctgtccctgagatctgtcctggactgtccctgaacagcagcattcaattccttgtc
tcagg

>chrx_21858879_21859079
aaatgagacttttccttctatcatttaaacttgtaaagtgcaggaggttacatagcgtagtcctt
agatccagggctgggcttctagccaatgtgaccttgggaaaattgctatctgtgccttggtttct
catctttaaatagagataatagtaaggatctcctaagattacagagagaattacattagtgtatc
cacat

>chr9_115610379_115610579
aaagtctcactgtgcccttcgccttttgatgtgtcatatcaatgggatgcagatccctataaag
ttctgtgagaagttcagatgatgactctcttctgcttgtcactcaatgatggaggggcctctctg
cagacgccggcttggtctcttggcctctttaccccactgccggctcgggcctcatctgacagcca
taggc

>chr15_35312708_35312908
agttgcttcagtcacaatcaatggtcatttaactgcgtctaaggaggtcaggtcttaggtgcagg
ccttttgtttggctgatttgctttgcagatttgggtccaaggcatgcaaatgtccctgtttagga
tcacaaaggagtcactggggagccaccagcattctctccactacatttcacatcttctccagact
taata

>chr9_129109179_129109379
gaacttggtggcaagctcagaagcaaggttagagtggacctgcaaggtaagtgggaggtgacaac
gagaggttgagagtgcagatggcctttgaagaggggaggccagggcatagcgggagctagaggat
gccatgaccccagctagaggatgccatgactccctttgttaaaggtaggaaagtgtcgaatgtgt
ctagt

>chr19_7445600_7445800
acactgattttgtttagccaagacacattactgccctctgctggaaaacctgtcataatgtctca
tatacaagcccagagtgtcaaggacaggtgggctcttagacggggtacctggtgtatgcctgtac
tcggtagcactggggtaggtgagtgggagaagaggctgccgacccaggtatctgtcccctctgat
cctgc

>chr12_117539417_117539617
gtctgatgtgggggataggaagtggcattctctggacatcagcccctcctgcttggcacagttgc
tctggtggggacaaaagccctgggacaaggagaagggcacatggcttcacattgtcccttggtga
tctcaaggaagcccctggtatctagtatgaagggagtctgtcctacccgatcacctgtctaccct
gaccc

>chr4_76994976_76995176
ttttactcaaaattttgtttgtgagatttatccacattgttatatatggtcagagtttgttcgtt
ttcattgctatataaaatttcacttacagtctaaccattctactgttgatggatatttggtttcc
agtttggggggttattatgaatagtgctactatcaacattctattaatgtattttggtgtatgtat
gcgtg

>chr17_14227075_14227275
atttagcaaacccaggaaaacattgtctttggagaggtttgagcttgattaatctccaaaacgtg
ctaatggggcaatagagagctcagctcttgttctctccttcaggtggtgtttgcctgtgagctta
gtgccctatgttcccagcccagggaattggaaattgattcatttgggaaagtaacagcttaaagg
tcttt

>chr19_38984760_38984960
atagccccacagcaccctagcctgggcaacagagcaagacactgtctctaaaagaaaaaaaggaa
aacaatctgctagaatctgcctgctcccagcaggtggagggcgcaggtggtagtaactgggaaaa
cttctggaacaggggggccccttccacattgttctggtccaaggccccatgtgccgacctgccctg
catgg

>chr6_79290681_79290881
cagctattaaacaatgttctccctccaacaaacctagtcctctgcactgtgctgtgtgatgctgg
ggccatggcttaccaaattccactgatcctttgacaaggaggcttcctgttaggttctgccacca
taaaggtactagagaggggactgcaaggctggaggaggaactgaaatactcctttttgctttgct
tcctg

>chr14_50275850_50276050
ctaaaaatactcttaccatgtgattcaacaatattgatgtttggtatttaccaaaatgaactgaa
aacttatatctgtacaaaaacctgcacagatgtttatagtagctttattcatattgccaaaacat
gggggcaaccaaattcagtagattaatggataaactgtggttcatccagacaactgaatattatt
caggg

>chr1_197780977_197781177
catgcttattcaatttgtttcttctttctctcagactggttaaaggccaaaacttgcccttccta
tttgtgtcctcatgttctatgtatttatttgaatcatccaaagaatgttaagtatcatgctctcc
tcctatcctgtactttcttacctctgggccttcttgggtaacaattaactcatactgaattgtac
tatat

>chr2_61808696_61808896
attgcaccaacttaaatcccaccagcagtgtataacatatccttaccagcatgtgttgttatatg
atttttttcctttttgactatctgaaggttataaagtggtgtttccttttggtttaaattttca
gttctctgattactaataaagttgtgtgcatttttttcatatgtttattgaccattcaagtccctt
ctatg

>chr20_25768600_25768800
acgttgcaaatgtttgttctaaatcccacactctgaagtcagaaagacctgggttagccctcagc
cactgacggttgcttgctatgtgaccttggacaagtcactgcccttctctgagcctcaacaccca
catttacacaggggaagaataatacctccctcttgggctgctgtgaagcagagctaatgcgtaca
ttgcc

>chr21_28642129_28642329
ttttcagacttgtctccttccctccctctcccttgagtcactagtcctggagcttcttgaggttg
ctgaatcaccacttatctgcaggcctacttgacctcagcatccttattgacagtattcctctgct
tgaggatcctttcttttccatgtcatgtacccatgcatattcatctttcaatatctgcatctaat
aggtt

>chr22_50111996_50112196
ttgaaccagaagcccagccctgcacctcccctccacttccctttcttctcctttttcttcctttc
tccttcctggcaaactgcagaaataccttgctatgagctaggcctggactcctatctgtgcagcc
actcacagtggtcagttgcatccgtcagccactgctgcataacaagcaacccacggtaagtggcc
tgaag

>chr10_35814794_35814994
cttctgtttaaatatttatgttcagactgtggccaggtaatgtgtaaggtcttgaatatgctcat
ggacactgaacctctgcattctatttttacagtgtcgctcttgcaatatattatcacatcaaaaa

caggtatgtggatggttgtgtgctaaaaacatcatacgttatcttctgttatgttgttcctattt
tttca

>chr2_78193492_78193692
cctccttcactatgggtaagcttccaccttccattcctccttcttctcccttagcctgtgttctc
aaaaacttaaaacctcttcaactcacacctgacctaaaacttaaatgacttattttcttctgcaa
tgctgcttgaccccaatacaaactcgacagtagttccaaatagccaggaaatggcactttcaatt
tttcc

>chr3_62905560_62905760
taggacagaagttctcaaaatgtggtgccccaaccagggttatcagcatcacctgggaaccgaat
agagatgcaaattcttgagccacatcccaggcctacttttagaaactctggaggcgggctcacct
tagtcctacaagcactcctggtgatttcaatgcactcatctttgagaaccactgttctggggtgt
tctga

>chr11_13025224_13025424
tcaaagcccatatcaattcagagagccacatttcaagtattcaacagccccatatgcctagtggc
tactgtattggacagagcagccctaaggattaagactgcaaaagatctatgatgtgctgaaaggc
atcttctctatttatattaaagtgattaagcaaccacagtctgaatttttaagtacctccttcac
cctcc

>chr18_45502202_45502402
gcctgacctcaggcaattgcagagacgttaccaggcaggccccaagggaaaagctgctctggcac
accagagttgcagtacagcctggaggaagttgcagtcaaggactcagccccgagggttaagtagc
acccgcattgttactcttctcttagatttcagagatgttttccctgccctgcttcctatcttatc
ttgat

>chr19_29481560_29481760
ggccagacagtgtcaatagcaccagagcactctaacacctggcaagcttcaccctgagccagggc
ccccaccagccagcgggagaatctgtcatcgtgacaagccaacctgccttccgcctgtgctgctt
cactggcctgatggttagggaaggaaatttgatctctccttcctgcccagttcctcttaactgca
ggccc

>chr4_188952406_188952606
tagggaaagggaactcacaatgagtcatagacacaaatgttaaaataaaactagtaaacatctag
aaaaaaaaaacgaaagaaaaggctgggtgcggtggctcatgcctgtaataccaacattttgggag
gccaaggtgggcagatcacttgaggtcaggagtgcgagaccagcctggccaacatggtgaaaccc
catct

>chr13_84505399_84505599
atatgctaaacaaggggtgtattattcatgcctccccattttagacatatagggtaacttcctga
cattgccatggcatttgtaaagtgtcatggcactggtgagagtgtagcagtgaggacggccagag
gttattctcatcgccattttggttttggtgggatttagccaacttctttactgcaacctgtttta
tcagc

>chr6_32983222_32983422
cctttggctgggaactgctcacttcccttagaactttcccctcccgtctcctgacttctctaaat
gccagagttccaacctctgtctcctgggaaattctaagctaaaatcactcttcctttatatctgc
agatagtttgaaatttatacatcaaaaaaagtaactttaaaaatatataactggtctcattacac
tctgg

>chr2_51000696_51000896
aaacattggtgcagtgcactagctaagttcagtgaactcccaagctaatcctgtgttctcagaag
ctataagcgccaatctcttcacctttgctcaggggggatgagctcaccacagaaactggcctgttt
tttgacctcaaaatccagttgggacagggttgggggatggcagaagcaatgaaagggaaggtttc
caggc

>chr2_212528755_212528955
tgaaccaatgaattggcaattaccacttccaaggtgaatttgaatttgaatggcagaatattact
ttaccttttgaggatctgtcacttcacagagtgagttaagcttatttcttgacctgcaggaaatt
gtagcaacatctgcaaattgatgttttcttggcaggaaatgatgtcgcttgcttaatttcttcag
tatcc

>chr8_22638855_22639055
cggggctgggagtggggaggagggacacgggggtgggctccaggcttttccccacacttcctcag
ggatcgctttacataaccagggctgcttagtcaccgctttttactttttttttggaaacaatttt
ctcagcatcacagctcccgtctccatcccttactagtcaccggtgctagggaagtccctttctc
ccgtt

>chr1_71054612_71054812
attttaaatcaggtaagcggcctctgtttactctcttctccaacctccctcactatccctcaacc
tctttctcctttcaatcttggtgccacacttcaatctctcccttctcttaatttcaattcctttc
attttctggtagagacagaggagacaggttttatccgtggacccaaaactccggcgccggtcacg
gacta

>chr10_1463400_1463600
ttacagtcaaagcacagcctgtgcacctgcccgtcggttccatagtgtttgtctgctgcttggtt
tgtgggacaggtggctggtcaggatctgcaggacctaaccccacccacgctcagctgtgaggaga
ttaaagggtctcagtgtctcagggtcaggttccaggctggcttcttgcgatttcacagaagtcac
taaat

>chr19_24221760_24221960
tctcttgtccaaatgcctatgcatgtggcaagggtgcctttggcttgcttttctgccctcctaaa
cctaaatctgcagttccaaattttgagtccagctaatagggttctccattctgtgttggagcagt
cttcatgtggggatttctcctctacttttcctctcctcactgtaacacaatatggagccaagtta
atggt

>chr11_22360424_22360624
gtttttgtcctttactacacaggatttatcttctttacagttaagaaatggccagcccctgctca
aacagcgtttcttttggtgcttatagataccccgaattaaattcctttgatgcagacaaatgaat
ataaaacacaaccatctaaaaaaataaccaagaaattccaaagagtaatggtttatatatatata
tatgt

>chr1_160780576_160780776
gacttgagatatgattcaccctgaggcaaaattcttcttcagttgtgaacctgcaaaaccagaga
agttatctgcctccaaaattcaatggtggaacaggtatggaatagacattgccattccaaaagag
agacattgggaagaagaaaaaagtcacaggtcccaaacaagtccaaaacctaacagggtaaattc
catta

>chr20_61075205_61075405
aacggctgtgtgtgtgtgatgggccggagcccgggggagtcaggagctcagactcaagtgaagct
cagagtgaacaggaagggtcacagccgcaccgtcccccaacccccaggcggtcagaatccagagc
gtgggcccagggctgcccctgcccgtcggtccccggagaaaacgcccggtgtgtgcggagcttca
ccacc

>chr12_67966533_67966733
ctggatctctgccactctgacttggccactccatttagaggcctcttgttgcagggctttggtca
ggagctcttttataggcctgcccatgcaaagagtatagttatcatgtgaagaaagttttctctga
cccctgagggtacagaaaagaaaaggcttgtgtgagtactgagtgccaatgaacctgttaaaaag
atgtt

>chr2_35641096_35641296
aattgtcttatgagttgtatggagttctatattttgtacatgctagctcgttgagttgcagctaa
atgtgctgcctgtgaggcactgtgctggcagtggtgaggaaataaatcattgctcagtgggaatg
atgaaggtacctgtgtcacaaggaataagattcatcccttttatgaatgctttataagttgtaaa
gttct

>chr4_123617950_123618150
ggcaagaggcttatttagcttttaaaaagatttacatacatctcaatgggggcaaagaaagaatt
cacaattacaagtgttcttaagtaatagaaagcagaaggatgattaccagaggctgagaagtata
gtgggggggaagggggttagttaatgagtattaaaaaaaaatgaataagacctagtatttgataac
acaac

>chr14_55141050_55141250
tttgcatgaggttctctaaaaaagtaagactgtaaaagcagcttgttcaaaacttgacctcttcg
gcatgcccaagaatttgtgaaacaaaagagaaggcaatggataattgattctgttctctaaaggc

ccttctgacactggtcaggtactggtgtggtttctactggtgtagaaactggtgtagatactggt
gtaga

>chr11_117009990_117010190
gctggaattacttgcgtgagccactgcgcccgggcgcctcttttttttgaggtgaagtttcgct
cttcttgcttaggctggagagcaatggtggatctcggctcactgcaacttttgccttccaggttc
aaacgattctcctgcctcaacctccgaagtagctgggattacaggcatgtgccaccacgcccagc
taatg

>chr17_48154001_48154201
atgcgggcagccttcgtgtcagagcagcagcagaagctgagcaggtggctgtgggccgccggccg
cgttaatcggccaagggtgggacggggcctcattaactggcagggtgggggcggggcctcatggc
aaggcgagcccagggacagggctttagcgggaacaggtgggatggtcagaaacggggctttctcc
tccag

>chr5_129061501_129061701
tcttggctgaggggaggggtccattcaattagttgggggacttagaattttattgttggtttaca
tggtgaaattatctcatgtgtacccaagtacagagaagattgtactttccttaggtagatttaat
tgatgaatatcactacggcaatggaacaaattttgctttcagagaccttggggcttattttttgg
tgggg

>chr2_47905696_47905896
cccagtcctgagaaaacaagaaattcaaaatctaagctgttggaagaataaattattgtgagctt
taaagaatgattaaaagcctgagtcccatgacaggcagctataacctaggcagttgcaaacttta
ttttctctgattatggattaagccttcttccttgcctacattgttttgtaaaatgctgtaaatgg
ctgaa

>chr5_78984844_78985044
tttgagcagtgggaagccattgaggggttttaaccacaacttgatccggggaaaggtttggggca
tttgggccagtttcagacaaaacttagctttttgctaacgtgggggaaattgctcctcctcaagg
tttaagatgagtggagataaagtttcctggctcaataacagggaccactgagcaagaaaatacac
agtat

>chr14_82863247_82863447
taatccccagacagttccaagtttttggttcattatcacatttcatttggagtctcctcccata
aggcattctcacccaacactagctgcacagtttttctcaaaatatctttcacaagaggcttcaga
gctctgataacttcacacaggagacagttgggtgttgcagttaaaaacggtgcccatggacttca
agggg

>chr1_242375177_242375377
gcttaaagctagtggagcctatgtttggcaggctcaattaagggacctaatgcaaactgttgtat
cccaaaacccatggttccctgaagaaggcatgctagacgtaaagctctggaacaagtggggagaa
atcttaaacattgcatgcaagggcaaggtcccagtatcatctttaatgctatgggccttagttag
ggcag

>chr1_228107977_228108177
aactacacacatctatctcgatttagtggtttcaaacaactaagcagatcagaggcagggttagg
agggaatatgtcagggacggaaggcggggccctcccagactggggcttggccgagagcggggagc
tgcagccggggaggtggcagggatggagaaaataataattattatattagttaaataatcataat
aattt

>chr8_5423192_5423392
gcttttacgtatgttactctgagaacaccctcttttatttgtggattagcatagagtttccataa
tccatggggcctcctgtttgtgtggaagtggttagtgtgtcctctgatttacaatcccctgtggt
ttctgtagccctgttttcctactgttgccttctttgccataagcctcccacgatcacctcccctt
ccaag

>chr2_188430755_188430955
taaggtggggcagggcatattcacttcttttgtgattcttcagttacttcaggccatctgggcgt
atatgtgcaggtcacaggggatgcgatggcttggcttgggctcagaggcctgacagaaagcataa
aatgagaactatagaaaaagtacatagtgtagaaataaaataaaatttcaggactatctaaattt
agtat

>chr2_194212955_194213155
cacagaggctgcagctcatggagggtgcagtgggcgggctttttgtagtctccttttctctcatg
gagtcatttagaagttttccttagtgctgttctgatatttaggaagagaaagatataaagatgct
tcctaggatgaaaataaactgaacaggtttcctgtagtctcacctggacctctcctgcaatccaa
aacca

>chr3_150820710_150820910
taagtgtaagtcaattaactggacttcagaggacagaaaccaaggcttatgtttgtgttctctgc
cctcagatcacctagcacactagcttgttaagtactctgtgaaaaatgatttaaaggaaaaattt
gttatgtaagattgcaatgcaagaactatatggcatacctcagcaataagggatctaaagagaaa
actat

>chr4_135545550_135545750
actaatcttccttccaaaggtttactgctcaaggctacacttaagaaccttcctcctcaaagtga
gttaccattaaattcctgtgcaactgtttgggagattgttttggttggaattacagtggtataat
gactgcagtgctcattgcctctgtgcataacacttgcaagttctttagtccttattagctctcaa
acact

>chr5_6143200_6143400
gtttccaaacaagatcacattccgaggtgccagggattaagactgcagcatatgaatggggtttg
agggaacacaattcagcctgtagcagtaggtattcttattgctatttttttgtataaagaaatcaa
cagtatcagccagggcccacttaggaaaacagaaatcacaccaattattataacggagagaattt
agtat

>chr6_136827507_136827707
caaaccacacttgaaagtcacctgtagggaagggaagagaaatgtggggagggaagggcattggt
aggagtttttagaggacacaatccacagttttagataattcaggatattttaaggtttataagct
ttgggaaatttctggaaacaataaaagattatttatcaggggggcatctgctcttccagcctcagt
actgg

>chr6_151423107_151423307
agtttcctagaaaactgtgacaagcaaagcaataacacacgtcgagaaatatctgatcaagcggg
aaatcttctgactgtcggggatctctagtaagatctcttggaatgaagtgcactgtgtatccaaa
actattttccagcgccagtgaagttgctcttacctaaaacaaatgggtttatgctagtttccacc
aagga

>chr7_29322475_29322675
aaataggaatcgtgaaagaagcaacagagaaagatgaaaaagattggagaagaagaagaaaatga
aaacagagaaaaggaacaatatcagcaggtggtggatggcccgagtggtagaggaagtgaaagcc
aaatagcaaaagtggcagcaaagaggacagctggccagtaggagagaaaagagaagccacctctc
ctgga

>chr9_27354000_27354200
tggatgatctgtcacgtctgtgtctgtgtcagtgtgccaggctccccctccctgctggagaccaa
gtgaaatggcatgtgtttgcctgggttcccaggctgccagccatagcggctgaacagcaggtccc
aatgggagccatgaggcaagctctgggggtggatggatgtgctggtcttcaccaggctagccagg
gtgac

>chr10_77298594_77298794
aaacatccccaggcaatgaccttctgctagcctcatgtgatattcacattctacagtactgaatg
ccaaattttctgtcttggaatagagtggtacaggttgcatgttcaagtttagaatcagcagggtg
ccatccctatattagtgttgcttgcatgcaattgagtgaaagctttttagaggatttcctggcag
aatgg

>chr11_2024424_2024624
cacgatccctgttcctgctggaggccctgttgggagggtctccctctggaccttgggctcaagtt
ctttggggtccaagtcatgaccactgcagaacagagatttctctttgcctccaagtatccactgt
cctacgtgctatgggagctgtcatcggtggtgtccctgggattaggctcccagccatgcatgggc
tccta

>chr11_68868624_68868824
gtgagatgaggtggaagtgagcagagggggctcctggcagggctgcgagcctcctacctctgggt
cccacatcggtgctgggtcccaatgcccaccttgtctcaggggtgacgtcatcaccttttgaaga

agccccagatggtgttgaatagcgatcgccacccccagtgtgcaccagggaaggagcatcgtaga
cctag

>chr14_96448247_96448447
ggcgagggggataaatggtccccacctcaaagaccatgaagggttaaaggaaatggtatttgtg
agctccttagcagagtaacaggcacatcaaaagtgctcagaaaatctgtactcaaaaccccaata
cccacagcctgaggagccaattactctcctcttgttctctctaaaaccccccttcaatcaggctga
cacct

>chr15_74556147_74556347
tgcaacagtgtggttatatgtgtgtctggatgaatctggatggtggtgtatgccttgcagtgcac
aagtatgtatagatcgtgtgtgtggctctgtgtgtatgcatgcatgtatttatgtggctggtggg
aagttagaggttatgatgagtggacctgagctgggatcctgccctgggctctacatacttggggt
aacag

>chr15_90011796_90011996
atccagaaacagccagtttagcaagatacctagttaacttaagacaatttcagcaggctttacaa
aagtttggaactcaaaggccccaccataccgggaactaaccagcaacccaaaatcaggctaaggg
ataaggtacttgttaaaacatagaaggagggatcacctgctcaacaattgcaaccccaatggaag
ggacc

>chr17_3875851_3876051
atgtcagtggtgaaggggaagtggcgggggcagcgtcttcccgcagagagaacagaaaacagtgg
cggggttgccctgctgctgccccactttggtttgtaaattggtttgagtcaaaatgcaaacagcc
ttgcaggcttgggaacctcaccttcctcttcttcggggtcacctgggcctggtggacaggtgtcc
caggt

>chr17_35118487_35118687
tggatgtgtgagtaggtgtgtatagtgagtgtgctggtgtgtgacaaagtgtggatgtgtgaata
ggtgcgtgtagtgagtgctggtgtgtggagtgtgagtgtggatgtgtgagtaggtgtgtatagtg
agtgtgccagtgtgtgacggagtgtgagtatggatgtgtgaatagatgtgtatagcgagtgagtg
tatat

>chr20_50214993_50215193
gctcccaggtgacagtgtcaatggcgctttatgtaagttctttccagattgatgaaccaagaaaa
aggtcaactgaatccttatgcttaaggtttacaaactcaggagagtttcttgtatagtaactcta
gtttcttgtttgcttatttctttgtcttccccaccacaactaagatgacctccgagaagtaccca
ggcgc

>chr20_60140805_60141005
cctggcagaggagaagcctccttccacgtagggaacagcatctgcctgcagagggctgctgccag
gagccagccgaggctacatgcctgcctggacgctgtcatgaggttgctgccggggttgctcagct
gcctggggtgagggtgtctgcatccaccccctctgtcctcatggggttctccagccccatgtaca
ctgtt

>chr20_61865355_61865555
ccccggtgcactgcgggcagactgggaaccaggcagcaaatccacccagcttgaggcccacgtcc
tcctgaaagggccccaggcccacacccggggttttgctgaagtgaccaaaggtaaccgctgtgct
gagggggcggtggaggacagggagggggcgaaggcgaggtgggcgctggaggacgtgggggtgaa
ggcga

>chr22_46241536_46241736
tagaggggaaggcttctcagcttgatactgagtccaagtatttgcagagtgattattttttaata
ccttaacaatggatgtgaagatttcaaaaatacttatttttgcaatcttaaaacagtcggctctc
tgtatccacagattgaaccaactggggattgaaaatactcaggaaaaataaaatagtagtacaac
aacaa

>chrx_8672400_8672600
agattgtcttcagctgcccaacctccccttcccaagtgtaaaccctcctgccctgccccacacca
actctccttctttcctgcaattcaatgctccccataccattggcttatacacacccatcaaaatg
agaagaaagccaatagccacagagcctattttgaattgggaattaatatcaatacattgcgttga
gcaag

```
>chrx_45763456_45763656
ccatgacacaggcactgaggagcaggagtcaacccgtgaaagctggcaccaggacgggtctgtcc
ttcaagggaaaagacatagcccctgtgggagaagccatccaaggcagggaggggtggtctagctt
tcccctccgaaccattccttccattggctgcacccaggcagctgacacaggagcttactcagaaa
actgg

>chr2_181010755_181010955
agtcttctcttacctaggatacaaggatgtatatccatggtttttctcactgaattcaaattaat
tgcctgtgatataagtttatatttatgccaagatacacaaggttactttttatcctgcttaact
ctagaaaataattaagattatctaagataaaataattatataataaagccgctgctataaaaata
gatac

>chr3_23787796_23787996
ataagactccaacgctcaatagcccacccagctaataataataataatgataataaaaagtctat
gtggaaacctgtgataatccaaataactaattctagccattaatttcagccttctccctcactgg
gagattcgagagctgcgaaacaccaagaaataaatcactgagcttcgcttctcacagtctaccgt
ctggt

>chr3_191640106_191640306
atttgtcctcctgcccatttttgattgcattgcttaataaggattgagtttggagagtcctgtat
ctattatgggtaacgtccttcaccaggtgtatgttctctgaaaatggtggttgtaagcattgacc
tgctaggtggctaattaagggattggcttgggagcttgcctctgttttgcccacctcagtgcttt
ctcat

>chr5_169691222_169691422
ttcagacatactgaagaccctgaagaccaattgccccaaattgcaattctttcttcctaaataaa
acaaatttagagattttcttctacgtttttattttgactttgatatggtccatgtgggctgacaa
ttgttagttaactcgcctttctttcccttccttccacacccaggcacagttagtgccctatggcg
aatgg

>chr6_18485421_18485621
ctctcagggctaagatacatacctcattggaatgggcatagctgtggctcaccagatagtggagg
agtccctgaaataccttgatgaacttgtaggaaatctttcctctgaggtgctgagaaagccattg
acagggacatgctgtgctttgcaattcattgcaatgttacctgagggtttgttggggaaggtatt
ggcca

>chr6_34109022_34109222
gctgtccaggctctcctgggactcaccatcatcaccaccaatgggcgaccagtgccttctcggga
gaactgagagccttagctagacaccacggcctgcctgtcagcatggctcggccccaaatggcgtg
ggctggcattatgggctggcatttcccaattaccacagataatatctaccttaattgaatggggt
ccaga

>chr6_129517307_129517507
ttgccaagaatacctatagtctagtttaagatgcacattaggagagtctgctgacataatagcat
ctggcttctaaaagtaatctctggtaaactcttttgttttgttgttcttgcttgactttccattc
tacttcacggatctcaaattaaagctatagatttgaagaaggaaactgatatttgagatttgtag
gacag

>chr8_129476018_129476218
gtttccaaacagctagaaacatccttttccctgaataatatccatgttaggctttcagagctgag
tgagtaataagatactagggagagtcctggataacaggtaagtggagttggaaccacttccagag
caaaggataaatgctcttacttggctcataggcctgacatgacatgaacaaaaaaccagagtttt
ctaat

>chr9_2213600_2213800
agtgggcatatttgcttataagctgttaagcagggtattggaaggcctctatggaacttcactta
tcagtgatattttgcttaacctaactacatgccaggtatgtagttcagtgacacttaaggacaaa
ggtggagtcactccccaaacttaacatcaaaacaactctctaagaagcaagcctgtggacataag
aaacc

>chr9_102062179_102062379
atctccacattatcagcatgacttctctgagtcatgttagcccctgcatcctccccttcaggcag
caggatgtcctcacaaaacagggccattgacaacacaggtgtgcaatttcaattcagtacaattt
```

gattcaaaaagatgtgccctttatgctggcgctggacagagaaggggagaagaaactttcttggc
tgggc

>chr13_20260800_20261000
aaaattcaaaaatatagtttatgtataaatagtatttgaaggctgttgatttttattaggtattc
tttctaaagtgcaacagtgtaataaagattatttctagatctcaaggaggatctgaatgatggct
ataatgaagggcctatttaggtaacttttaagtttgaggaataagagaatggggttagtccactt
agggt

>chr14_48734050_48734250
ttgagatctagaacagaataatgggttgtggagggaggtattgaggataggagagtatatgggtt
cggcaccacggggtgtataggcaaaacaatttagttgataaggcacagatcctgaactaatctgt
aagacttgtccagtttttggacaggtaaaatggggaattgtaaggagagtttataggctttaaa
agacc

>chr14_70758247_70758447
ctcaacctagcagagccaaagagatacttgtatgatgtaagtcagattatgtcattcttctgctc
aaaactctccaataaatagctcaccattcccttcagagaaaagccaaaggacttaaaatggccaa
caaagcctatgatctgcacccccctcctccctctacctcacttcatctttctccctgactcactt
ggttt

>chr15_60412708_60412908
agattctcttcataacccacttttgagctaagaaagccattcatcccaataacaatagctggcct
ttattgagtgattgccatgcaccaagctctgtgtgtgtcacttacaaatacaggcagccttgctc
tcaagttcacacacttaatactaccctagaaaaattctcttaacataaaatgcaagtcaatctgc
agagc

>chr16_710199_710399
aagcacagaggccccccgcagggctccccagcacctcctcagggttgcttttggtcgccaaaggt
gatggctctggcccttgggcgcctgtcccctggaggcaacttcatcccacacccccacccctgcc
ctccgctggcctccagacgggactctgtggtggcctcctgctgacctgccttcccctctcaccca
gcagc

>chr16_3048399_3048599
gacccctggctctgccccacacctccttctgcctctcagcccttctagcctctgcccccagtgtt
gccacactcatctctgctttccctctccaggacacggccatgaaaatgggccctggccaggtgag
gccactgggaccaggggtgggggcccaggaggaagggagagggaggctgctggaagacggagggg
cctcc

>chr16_29923299_29923499
agctgctgctcctcccggggagatgtcaccatggggatgaggcacagcggggacagcgtctccct
tttttgctggggaagaagcggaaggtggttaacagcacaaccaaaggccacgtactcccccactt
aaaagccttcaaaagctggttgggaacaactccagactcctcagtggtccgcagggctctgcagg
ctcca

>chr22_42777256_42777456
tggcaaggcccctgctcccagaagctggtttcccaggatgattttctcaagcaagtgctgacaga
gcaggagtcaagcgggggtggggttggaggctgaaacccattgaaggggtcaacttcctagaggc
aggcagggatgcctgggggtgccagacgtggctgagatgtttctgcagaggggaggttgggaaga
cctag

>chrx_62975075_62975275
ccactgacatacccgccgaccaataacagatgccgcagtagcacgttcgctgcccaggggcggga
cttgcgctggcgtgctagccgcgaccgccaatcagagcctcaaggctcccaagcaagctcgctct
ccccagaccggcgagagtcaccagccaccagttctggtttgggggtggggagcttcttgggggcg
gatag

>chr1_3270340_3270540
ctgcctctgtgtcttgtcaccaggccttaccctggggacccctgctcccagcggagccagtagtg
atgacaggcgcagctgggagcagcttggtagacctagggggtctttctagaagccaagggggccc
ttggcacacacatgtggatgcagggctgcccacccaacactgctgagcccacacaggcccaagag
aaaag

>chr1_12125613_12125813
gcagacctttataaatgcatgtccatatttatattgttttaatagacatatatatagttttccat
atactataccctacagtacatatggttcttcatttggctttataatgtctatgaggcatcttggt
gagctttccatgctgaaacttacagatcctctgcctatcttttaaaatgctatgtgatcttttgc
caaat

>chr1_114566077_114566277
agtggaaagcccatttgcataataagattagagtggggtggccagtgtcccctcctgctatgtaa
acctcacacttggtccaaccaatctgtgggccatatgtaaatcagacaccgtctcctcaagcctg
tctataaaatccggtgcactccagtgcactctgctgcaggccggaagtcccatttgggcacccct
tactc

>chr3_191389306_191389506
cacctttgaaacatgggtgaataatcagagaggtgtccctgcaatgattaaacaccaagggaagg
ctgccttcccagtccgtgaccggcgccggagttttgggtccacggataaaacgtgtctcctttgt
ctctcctagaaaatgaaaggaattgaaattaagagaacggagagattgaagagtggtgccaagat
tgaaa

>chr5_34545243_34545443
tgttactggcagcaaatccatacggatctgcggtaacctcaattcttgcctcctcagaagaaaga
attcaccaagaagagaccaaggcaagttttagagcaggagtgaaagtttattaaaaagctttaga
gcaggaaggaaagtacacttgtaagcaggcaacttgagagatcaatgtgccatttgacttttgac
ttgga

>chr5_143116607_143116807
tgactgcagcgtccaccagaattcctggagggcttgttaaaactgattactgggttccatcttca
aagattctggtcagtagatctggggtgaggcctgagaatttgcatttctaagacactccccacga
atgctaatgcagtttgagcatcacagcactagagttagctgttggatcctttcctctggggagtt
ggatg

>chr5_176202194_176202394
ctcgtggttcgtttgaggcccgtcttcccaccaacaccacacagagctttttgagggctgagaat
aaacccaagtcatctacgtctctccaggaacacgtgtttattaaatgaatgaatgaatgaatgaa
tgaacgaacaaaccagaagctgggtgacggtcgctgtcatggagtagaaagaaagactcttgggt
taggg

>chr5_176369594_176369794
aaattagctgggcatggtggcaggcacctgtagtcccagctactcgggaggctgaggcaggagaa
tggtgtgaacccaggaggcggagctttcagtgagccaagatcgcaccactgcactccagcctggg
cgacagagcgagactccgtcttaaaaaaaaaaaaaaaaaaaaaaggttgggccaggtgcagtggg
tcaca

>chr6_168733551_168733751
taaagttacgggagaggatgtcaaaaataacatgaaatagaggaaatctgtggccactcaatgca
aggggtagagctcagtaatgtgtgaggtcactagcaatagaaaattagcaccaaaatcttgttca
ttgtgaggaccctaagtgattgagaggaaattaaaatacagaaggaataatttctttacatgtga
attat

>chr7_137888860_137889060
gtaacaaaattgcacttgtaccttataaatttttacaagcaaaaaataaaataaaataaaatata
aaataaaacaaaataaatgaatgtgatgatggcacctgagtcacgtgaaagacagctgtaaccta
ggcagccgtaaactttgtttctctgtttatagattagccttttaccttgcctatattgttttgta
aaatg

>chr9_45734404_45734604
cccctttaaagaacgccaccgctgagaactgaccctcacatctcccttcctgaatttctttaggg
aaagcaacaaccatccctgtaagccatgggttttcatgcggaggaaggagagggctagttgtctg
agagggaaactgcttaaggctgcagactaggaaatgggattccaggtttaagatagctcctttct
ggacc

>chr9_128191379_128191579
ccacacggcctcctccgagaacagacgcctctgccaggccatgtggagcgcaggtgtgcggggcc
ggccgctcttggattaaagtcacacacacacacacacacacacacacgccaggtgcatacactta

aactcacgtgcatgtacacacatgcagaaactcacaacacatacactcacacatggttacatgca
cacac

>chr10_104951210_104951410
ccccataaaacaggagaaatagcctagagaaagattaatgagcacctctgtgcctaacattctat
tgtaatctggctttctttaggaagatgtgatgtcttctggttaagggtggcaggtactataaggg
aatactatctatataaatcttcttacaattacaaacttctgaaaacttgtagctgatggtaaatt
ttcat

>chr11_94391152_94391352
tcagtgagtctactttagctagttttgatccttgttcaaaaacagatacatattttaggttgtt
gctatttgtttatacttgaatggggataaattctatgtgctttatcaagagctttagatatttaa
agagcaagacagtctgagtgcccagcgagaaagcctggttatgttcagctcctcggtctctcgct
ctggt

>chr11_126906790_126906990
catattttcactgacttgctctatgctaaacagctttttctagtacaatttgagccatgagtgg
aggtttcaagcaagtcagtgaagagtttttgaaacaggctggagtgcttcagtcctttcttttca
ttttacttctggtgtaatagagctcttcaagagtttcatttcaggggatgctgagtattaggaga
gggct

>chr12_77018069_77018269
ctcctgaaatccaagttcccagatgccagccaagggccggctttgtaagcaggcctttccaagga
taagcagttagggctgctacctcaactttttgttgcacagacctgcacagggtttgtctgtgcag
gtttctttgttttgttttatttgtattttacttagttgccatttttttaaatattaagatatttca
ctcag

>chr12_79268269_79268469
tcaataagatagttccccaggaatgaaaatgcaataatgtttgcaaattgtctggtgccctgtta
ctatatggtagatttgaccacctttgctcctctttctctttttttattttcagaaattctttcac
ttgtaaaaatgccacctgactcaaaatgcttaagcaagaaaagggattttggtgctcatgtgacc
ataaa

>chr13_73109199_73109399
cggatggtgcttccaggactcacagcttcccttccctctggcccttctttatgccctggtgtttt
tcagattcagtatctctattaaaacttcctactcctctttcctggccttgtctccctctcttaac
ccctacaaaggactgaaactggctgtcagctctggggcttgccacagtcatagcagcctttgtta
acaag

>chr14_69096047_69096247
ctccaggggggtttgcaaaataagaaccccaaaacccacctctctttcccattttcttctttaaag
gtagagaaagcactgcatttcacattgggtcttggaaacccaaagctagacacaaaatgtgggcg
ttagcagctctgaaaagtgtgtgcttggtcttaacgtttattcttaaaaaggaggtgttcatacc
ccagc

>chr16_1299199_1299399
ctcttcctcccccgacctctgacccttggcccctgacctgcatgagcccctccagctccctcctc
ccagaggggtggagggcgatggccacagcctaggcctggaggcagaccccgctccaggcctgcac
agccccggctcgcaccccagtgcagcctcttctccccgtggggaaactgaggcagagagggcgcc
gtgac

>chr16_8981099_8981299
tattgacccattattgccccaacctacagatgcggaagctgaggctgagtaactagatcagcgct
ggagacataggtctgtcctcagggttcagcatcagggaggaaggcacagaggtgaggggtgcatg
tgtgtgtggctgaaggcctggaaggaagggtgtgtgtttatgtgtgtacgcgcatgtgtgtggct
gaagg

>chr17_35200087_35200287
atttggcatctggttcaagtttggtttatttagagcccaggtcggcgggcttttacatattaaag
gtgcattttaaaagagcattttctattctaatttggggggagccaagctctttagggctttcggga
gcggtgtccacctgatttacatttcccccattggagccgagccacagacgcacattagaatgtaa
ataat

```
>chr18_33072202_33072402
aaaacagcaaaaattcaaaatgcaacctcaatatccaataataaagtcaattaaatatgagtagt
taattccttggaatataatgcagtcaatagtcataaaagactatttgacttttaaaagtctaatt
gagaagactatcaacacatagaaatgttcacaaaataatgctaagtgaaaaacggggcacgtaat
actat

>chr20_60091005_60091205
tccctcacccctcctgcctggtttcctggctgctccagggtagaatgcagaacttctccagcccc
atcttcccctgggctctggaacgccccccaatacgattccaccgcaggcaggcagctccctcac
cacaggggctcgcctcgtgctctgcgttgcactggcagaggggcagcacaggacttaattctgca
gccag

>chr21_31232529_31232729
aggctatctactctaagccttttgactgctggaaacctcctagctgcagcttgtgagccctgggc
agagttacagcctgctgctgttactgccaacctgggggagtgacagcataattggatgcctctct
gtcttttggctcaagacaagggcaaggaagaatgcccacctgcagggaggaagtagagtccagaa
gacag

>chr22_30281000_30281200
gaccagcacagtggtacagccccgcttgtcgttaatgaccagccataaaaaaccctgagtttctc
agctcataggccaggttcttcagtggttgtctagtttaaggctggtcattagaaatattatggac
tacctaaattcctcgaactcatgcaggacctggtgaattaaaatctctgaggatagagcctgggg
atctg

>chrx_92084144_92084344
cccactcctgcccgccagagaacaaaccccctttgactgtaattttcctttacctacccaaatcc
tataaaatggccccaccgttatctccctttgctgactctcttttcggactcagcccacctgcacc
caggtgaaataaacagccatgttgctcacacaaagcctgtttggtggtctcttcacacggacgcg
catga

>chr1_164562176_164562376
cacacaatccttagatttttttttttttttaatttgagacagagtcttgctctgtagcccaggc
tggagtgcagtgccgtgatcttggctcactgcaacctctgtctcctgggttcaagggattctcat
gcctcagcctcccaagtaactggcattacaggtgtgtgccaccacacccagctaatttttgtatt
tttaa

>chr2_114430330_114430530
tcacatgaatattgatatccgaaactccctaggcctaagcatccatttgcccagtcaagaatttc
aattctcacagtttttctcctctgctccacatattcctgggaatcaccaggatgtgtcttacatg
tgccaacagtgagcctgggctgcctctcaccctgagtctgtgttgctgtggaggcaccactctgc
agcct

>chr2_131268730_131268930
ctctccagctgcatcccatttctctgttcccctgtagagcaaaatggctccaaagcgtggtctcg
tcttaccttgaacacccctcctccctctcgctatgtgagctactccagttcagctctccacctct
gcactaccctgaacctgctcctgccggcagcaatggcctatggagaagtgcagcccccagctgta
agaca

>chr3_19034596_19034796
aataaggctataattgtttcaaaaggttgacacacacttaaactaggtatcttggatttctttga
gtttaaagccctagtagaacagacaactgaatgtttttaagtatgtggtgcatttgctgtttttc
tttcattattgttagggtttgatggcaatgttattgtcagtatggtccaacagaactctctacaa
tgatg

>chr4_92488377_92488577
tgcactatgatctgagagtatgtttggtgtgattacatttcttttacatttgttgaggattgttt
tatgttcaattatgtggttgattttaaagtatgtgccatgtggtgatgagaagaatgtatatttt
gttgttttaaggtggagagttatgtaaaggtctatccaatccatttggtccaatgttgaattcag
gtctt

>chr4_159182550_159182750
agggatttttttgcaaacaccaaagactctacatgctgtactattttatgtaaatatgtacctt
ttctatggctattgcaaaaattctaaatacctaatcattgcttatcttcgatgctttgtaagact
```

gatttacctcctgaaaagatgattcaagatcataaataaaaggaagaaaatgtcattttccttaa
ccttt

>chr5_89600644_89600844
ccttatttgtagataatattaatggtgataagaatagccaggcttacaagaatcatttccaaact
gcactcaaactgaactccaagtctgcaaaagcaatattgtaccttccccacccccacagaaaaat
aacaatagatcacaaccaaagataccagcaggtgtgcaacatgcaaatggcaagcatgaaaactg
aagtg

>chr6_144965507_144965707
aataccagggtattccaaatgaatgatttttatctgactcattaagaagtgaaaataaatatttt
attatacatgtaatatgatttagttttacaaactgttaatatcataataatctcaagaagttcta
aaaatggagataattatgacagccataaaatataaagtatatggaattatatatttggctatata
ttagt

>chr7_27473475_27473675
ggattaaaaggaatgccattcagttgaggcagcatcttaagacctggggacgtctgtccccatgt
aaaacttctggtgaatgatttaaactatctctactaggccaacatccttttaagctaagaaaacc
acattctaatctagacatttgatataaaaattagatttcatgaaatagcataaacgtattgtcgt
tagct

>chr7_143583067_143583267
cggcttctctacaaatatgatttctccctgtggagaactttgttgctagttctgatccatgttat
ttgagtggaacacgagacttgaccatggagcaggaactggctttatgcctgaccaggtcctgcac
tcgcatctactcatctaacctggagagctcaactgtaaaacctggaggaaccactcatctctttt
tatgg

>chr10_60775594_60775794
agctcacttaacttaaaagttcaaggtggtattatctttaggcaagcaggatccaggagtttgaa
taattccattgcagatgctgtgttctctattgctagtctctgctttatcctccattagccccgtt
tttttcaggttttcctttgtcatgacaaaatgtttgtcaatagatctaaattagtcttagacatt
ccaat

>chr11_132202190_132202390
caggttccgggggtggtgagcgcatgtacctgtggggtgggctgcgcagactggatatggtcaca
tagtgccagccactcaccagcctgagatggagatgatgatgatggagagcttcagaggggctgct
caggcttttcccagatatactacaagcaagagaatggctggcaggcacttggtggcctttttctg
tcttc

>chr13_54707199_54707399
aatgtcatcagttaaggcaggaactggccatctggatgtgtacgtgcaggtcacaggggatatga
tggcttagcttgggctcagaggcctgacagttttcccttagggcttctgtcaggccagcatttta
atatgcttgacatttttgactttccagtaaatgtttaaaagaaattgtaaactaaatttagctga
tgaat

>chr14_103813847_103814047
gcacaaaggccgtcacaaccttccacaaataatacttctacaaggacatctacccagcaactgcc
tgtccaaccttggaccggcgtcggccttgttattgaccttcatggccaaggctgattatctcaaa
acaattatgtaatcctcctcacttttcctttaaaagctattctctccctttaccttcctaaagta
cacac

>chr15_84145796_84145996
agtcacctctgctaatgccttacttggccatcacacaccaaactcttctatccagatattatagt
actggggaagaatgactgtggtatgtttgcttggatggggactctcatttacttggagggaggaa
ctcaagaagcatttttgggttgcaggttgtctggcattttgctgtgcatttcattgtaatttctc
accag

>chr16_76920099_76920299
aagtactcacagataaaagggtgccatagctgcaacttgctctataaatggccaaatgactcgga
aaataaagcatgtgtgtgtgtgaagagagagattgaaagagaaaggaaggggaacagggagagag
agaataaggcagatggaactactactaaaataggctctatacatatgtatatatagtggtgcttg
gaaca

>chr17_27923074_27923274
gccgccacagaaacagctcggctttcctcaagcactcaggagccaggtttccctggtcttggctt
ggcatagctgcctcttcttcactccaagaaagccccgcagaggccctccgtgggcaggcttggca
gtgcagctggctgaggaggggccagcatggccaccttcagagcaggttaatgggctttgctcag
cccca

>chr20_32262139_32262339
gcagccccgcgcacgccatggcgccgccacccgctcgggctcggctgcggttgctgccgaccctg
gacgccgcggcggactcggtgtggctagaggccgccccttggcgccggcgccgacgcgcgggctc
aggccccgcccccgccccgcccccgcggacgccgggttccctcgcctcaaggtccaactccagcg
ccgcg

>chr21_35231530_35231730
atcagcagttcctcaggatcactccgggaagctggccctaggctggtcgtattttcttccaactt
tagtttgcagaggcagctccagggccaggagtcaagagacctgggtttggattctgacttactca
ctgcctttccttttttgagccttagtttccccatctgtaaaatgaaagggttggattcgatcatca
caaag

>chrx_89032944_89033144
cagaccaaaaaggggaagctacctatattactgagacaaatatcatgtatgtttttatatgtgct
ctgaagaggatccttattgcctgtcactgcttgactgaggctgctgaaaatcaaatacaaaacct
catcctttgagtgtgtgaattacaatacaggttgaactcaaagcctcacagcatgtctactgtta
aagta

>chrx_115947772_115947972
taaagtcccttctgttatcagactgtattgaggtgggaaggctaaactgaggaattatgtctgac
agaagggaagaaatgactgcggtggccttctcagaacctgtaggaaaggcctctacctatccagt
gaaagtatctacctagactaagaggtattttagttatctgactcagggcatgttgagtaaagcta
atttg

>chr1_57822612_57822812
ctttaagagacttactttaatccctttgtgcttcaactatacattggaaacaataatgaccctat
cctatagggtcactgcatggattaactaagttcatcctgtgaagtcattagaacagtgcctggca
cacagtacatgttcaacacatgttatattttatcaccaatacactctagcacataataggtgtcc
aaaca

>chr2_150172354_150172554
ttctgaaaaccctgtgaaatggactggttcgaacgggcagtttataacatgatacttttagattg
gaaggcccagtgaggtgaggatcttgaagtactcactccctgagaagttattctcaaaatgaaaa
cagtttaaataggtggacaggaacaggtatttatgaagcaaagagttaggctaatttttgcttgg
tctca

>chr2_207096955_207097155
gataaatgtttgagatgatggatatgctaattatcccgatctaatcactctacatatgtattgca
acatcactatgtaccccataaatatatacaattattatttgtcaattaacaaaataaaatttagt
ttacaaaaacacagaaaaacaaaaatccttcatggcttctctctttttcctgtctctcactttac
ccacc

>chr2_236666061_236666261
agccttgtggcttattagggcttcctctgttcacacgctgtgtacatcgtcctggtggatgggtg
tttatctggtagagtagttcctggtctaggcagatccatgtcagtcctttgacctcattgcccct
ggctttttatgtttgtgctgcagttaacattctggaaagcacttttatagaagccattccatttg
tcttt

>chr2_236757061_236757261
aacactgggagctattcagctgcctgtaccatatcagaacatgcttaagaaaaagagggaaataa
ttattgtctcaacaaagatttgggtttagcaaaaactggaaataattcacagtgaaactaataat
ccattgattgtgatgtccatggtgagatcattactctcgagaataatactggtaatttagtttgc
tcgga

>chr3_115833910_115834110
ttaaaatggaatttttagtctagctcaggttttatgaaattataaaatgaaaactaaatatggct
tacatttggggaatttatttttttcttgaaataagccaatacatttgcattactttcaatgcagaa

tgaagacagcaaagctgaaaggggctttaataataaagggtaaaggccagaatttgttgagcgtc
tattt

>chr3_195537003_195537203
tggggaggtgtagggaggtggtggcagtttcctttgccctcagcctagtcctgaaacatcccgag
gtggaagggtccgcttcaccagcatggcttctcttgcagcctttaaacggcactcgccttctgac
tcactcccaggaagctcctcaaatatgcgcccaagaaatattttccctcggcgactgttcttagc
ctgct

>chr4_91654377_91654577
taacaaggatgagatgatatctctttgtagttttgatttgcatttctctgatgattagtgatgtt
gagcaccttttgatatatgtgtttcccatttgtatatattctttagataaatgtctattcagatg
ttttggccatttttaaattgcattgttaatttttttgtcctatagagttgtttgagctccttatat
attct

>chr4_183004606_183004806
agaaatgtgttattgatcacctaattttttatttttttttcagtcttgcatttgctttttcaactg
tagaagtgcaagaagtggtaaatgtgccgcattttagacgttaaaggcattgttaacagatttga
cacaaaagataatgttatgagatgcaaacgacagaaatttacatgtcaaatcacatacttaatat
ttatt

>chr5_72779844_72780044
ttctgccagctgttgaatatatctattcttattacacatttggaaactggggaaatgatcacagg
agggtttaacaatccactgtgagatgaacctgagttaaaactccattgagccaggcgtcgtggct
cacgcctttaaccccagcactttgggaggctgaggcgagcagatcacctgaggtcaggagttcga
ggcca

>chr6_11993414_11993614
aactaaaaagaggggagttgagcttacacactaaatgcaaaaacccttcatatttcttaaccttt
tctaggctggaataccgtttgctaagtacacacactcttgtgagtgcatacacacgcatacacag
acacatatgccctaggcagacaactagaagtcttctccagggcatttgacaagcgcaatagaaaa
gatct

>chr7_13789275_13789475
ttgtttgtggtgttgttgttgttgttgtttttagttctggaaaatacctttagaaatgtatacat
atgccagacacctaggacttctggaacctggcaaaccaccttttttttaggaacaatgccatcata
acagatgggtgttagtgtaggcatgccagggaatcaaaatgtacatcacgttccactacaagggg
gcaga

>chr7_127657564_127657764
ggacaagtcaggaaaagattgcctagctgactcttccctggtctcagttccgaatgaaaagcctg
ccatgcggccttcagcatctgctactccccgtgccacacctgatcccatctcaagtcatttcaca
ccagcagagggaagccctgttccaactccagcagcagcaccactcggggagggggggcaaactct
ttacc

>chr9_8750600_8750800
ggcatgtaagcctgaacctgaatgacaagaagaaatgcagaaaagcttcccatgtgaaggcacag
actgcccagggcagaggctgggatgcaggaatgagcttggttgtggctggagcccagagattccc
agcagagaatgataccaggcagactgggaaaataaagtgggcccaggttgcacagggcctgtaag
caaca

>chr9_80639980_80640180
atggacatttatttttgtttttaaagcccaaattcatgaccacctaggatgtgttaactcatttc
atcagtatgcctaggtccttttactacatgttagatgagaaatcagacagatttttacatatcgg
taatgtaacaaatcaaaggtgattgttgaacaacttcctataattttattttttacatatcggta
atgta

>chr9_87771380_87771580
ttatccctttaaagcagggtgggtgactggacatgggaagcagcagagatctgaccagtacacct
gccttcctcacttggattcctaagcccacctgagttgtcatccagtcccctgttagaacccttgt
agccgcagctgtgttctataactacagcaatgtctgaggatacttcgctttatgtgcataaagga
gagag

>chr14_33634249_33634449
agtttagcagagtgtaagtcaggtttggggcagaaggaaaaatcagtcaggataaatgggtcagc
attcatcatttgttcaacaaatacttattgtactaggtgccaggcattggactcggcctgtcgga
aagcactgggtgatttatttttgttgggttgactccctggtgggattattcacaaacaacctttc
gatgt

>chr15_70923546_70923746
tggaaagaggtttgcggccagtatttatccagggtaggtatttctagttagctcttagcaactcc
ggggaaaggggcatttttctctgccaatatttgcatgtccattcagaaaagaactccaactggcc
ttgcttggatgttcacaccccttaactagtccctgtggccagggacagggcatcctggtttggagt
gagtg

>chr22_32607800_32608000
gctgaaactcctggagtcagggctaggttaatgggaaggttcccaggacactgcactctagggta
accagcttctccctggagggagagggtactgtctgcatcaccccatggtatcaccaagcaatcag
tgttgagtcaactcccccagggagaccaggtatccctgtccatgaaaattcctcctagtggcata
aatgc

>chr5_118739101_118739301
ctgtaatgtagtacaatatcaaaaccaggaattgacactggtatgttggtgctagactacagact
tccttagttttcactgttgtataaatggaaccatacagttgtaatcttttcagattggcttttt
ttactaagcataatacccttgagacatagccaggttgttgcatgtatcagtaattgattcctttt
tattg

>chr2_188423155_188423355
gtgttgaagtgttggggcagtgaaaatttttggggggtgatatggcgagagaatgggtgatgttt
atcagggctgcttcaagcgggattaggggcggcgtgggaacctagagtgggagagattaagctga
agggaggtcttgtggtaaggggtgatattgtggggatgttagaagaaacatttgtcgtatagaat
gattg

>chr16_70694299_70694499
ggacctggggacctgaaggtggatggggacacagctcctggcttctcctggtgctgccctcactg
tccccccgcctaaaggggggtactgagcctcctgtggcccgcagcagtgagggcacagctgtgggt
tgcaggggagacagccagcacggcgtggccattctatgaccccccagcctggcagactggggagc
tgggg

>chr5_162236222_162236422
cagaaaatgacagacaatcccctgacaaaatacatcagcctcaatttctaaaaattatatggtga
tcaaataaaacatttctgtgacctagatctaactcagagtcaagaagataaaatcctgacctaat
gttcagccattttattttattttatagatagcaaaccaataccatcttgttgggggaagtcaggg
acccc

>chr4_152474350_152474550
atcaatgattaatgacctgcatttttatttataatatcacatggttttatatttcataaacaatt
acaagaaaagtacatataattaaatcttaatttgctaatttaaattgtataaaaacagggaatat
atacaactaaatttgtgggtattccttgtattattgatcagtatctgccatatgaaagggaagaa
acaaa

>chr18_34768602_34768802
tccagaactgtctccttcagtaagtttatgcctcaccacagtcctcaccacaggtttccctaatt
cttttgtctttttcctttggatagaaatatttttgtgaacagcaaaatgtttctactgctgtatc
aatgttaggtcacatggcattgacttttattcaaactggaagatttcatagcaggattataacat
tatat

>chr19_17958000_17958200
ccagccccagtcgagtttaaaataaggcttcagtggcagtggcgccccactgcaatcccagctac
tcagggggctgaggcagcaggatagcttgaggccaggggttagagacctgcctgaacaacatagc
aagactcccatctccaaaacaacaaaaaaaatcttaacagaatattattattctgtaatactgtaa
caaaa

>chr7_155017267_155017467
tgtgagtgactacggaagacaaggtggactccaaacccgacgctatgatccactcgagcctcgtc
cagggaatttatggagcacgtgggacctccctgccatccagaagcacagaggaggtgcgtcccca

caacaatgccaggaacacacagcggggaattctctgacatcgagaggcttccagtgaggggcact
ggctg

>chr2_9533749_9533949
cctcccgcagaagaagcctgcgccggggtaagccacccccagccagctcggccatccgtgctcct
gccctctgacctcacctgcccagggctctgcaggaatgtttgcaatccccagggctcctactcca
gcgtgggcgccacataactaattcatcggggctgaagggtcttaaaaataactggaggattttta
gactg

>chr4_105613351_105613551
ctgctcgggggtcaggggtcagggacccacttgaggaggcagtctgcgggttctcagacctccag
ctgcgtgctgggagaaccactgctctcttcaaagctgtcagacagggacatttaagtctgcagag
gttactgctgtctttttgtttgtctgtgccctgcccccagaggtggagcctacagaggcaggcag
gcctc

>chr1_10879813_10880013
ggctgctctcgggctggtcagggcccagttaggaccccagagtcaagctccttccagggggcact
gggaggggcggggatagagggactcgtgggtgagctgccaggcgtgggccaggctggccagcgga
aggagaggtacagacctcccacgccgagggtgcccacacaggccccaactctggtcctcggcagc
cagcc

>chr16_12322699_12322899
ggcgagtcaagagccagagggacttcattgcaaagctggtgatgcggggcaggctgcctccctgc
agtgaccacacgagggagcctccaccgagtcttctgactcctgctgctctccctaagtgagtgtc
tagaatgcgcttttctcccccactcccaaatgccttgcagggcttgcttgccctcattccttcac
acaga

>chr13_89894999_89895199
ccttgtgatttcctaggcctgtctttactttaatcccctaatcctgtcatctcgtaagcctagga
ggatgtatgtcacctcaggaccctgtgatgattgcgttaactgcacaaactgtagagcatgtgtg
tttgaacaatatgaaatctgggcaccttgaaaaaagaacaggataacagcaatgtttagggaaca
agaga

>chr14_63863647_63863847
caataaaagtaataatatgaataatagctaatacacatatggcacttactgtgatcatcaggcaa
ttttccaagcaatctacatattaatcctcccaatgagatagttttgtccccctttttacagaagag
gaaactggggcacatagggttaagtaacgtgcccacggtctcacagctaataggtggtaaagcta
gaatt

>chr9_124710179_124710379
atagctacaaagcctggttatgctggtgccgtgcttttggaggatggaaggagattgtaaattga
aaaggaataaagaaaactaccatttactgactgtctactctgcaactaggcactgagctagacac
tctcagacctggcactagaattgacacttactgaataactattacatgtcaagccctattccagg
ccctt

>chr6_56658041_56658241
tcttagagttgaaaatggtattatgattatgtaaagaggatgtccttattctgacaacacaaata
cttaattatttagagacaaaaatgtcatgatgtctaaacttaaattttaatggttcagtaaaaaa
aactgagaaagcacaaattcagcaaaatgttaacactcagtgaactaaggtgacaggtatacata
gggtt

>chr9_139642979_139643179
ctccaggccctggagacccgtttatatggctctgaatttctccgtgtgggtggcactaactgcca
ggaaccctcctggcctcctgcaaaattacccaggggagcactgagcagtggcagcctctgagccc
agatcagaccagctgcacagccaggagagggtctggagtgtgtggatcaaccatactatactgat
cccct

>chr17_41440874_41441074
cagcaggcagcaccggcccgctcccggagccctccatcctgcgctttcccaaacccccatcaaaa
caccaacccagtctcctccctctcaagccattttccaacctgcctgggagccactctgatctccc
agaacacctcattttgtgaattataaacctttttcacaccttctcggtccctgtgtgatgtcacca
gtctc

>chr12_67948933_67949133
actgggaggtgtctcccagtcaggatacatgggggccagggaaccacttgaggagtcagtctgac
ccttagcagagcttgagcgctgtgctgggagatctgctctcttcttagccatcaggcagggacgt
ttaagtctgctgaagctgcacccacagccgccccttcccccaggtactctgtcccagggagatgg
gggtt

>chr2_61867296_61867496
gtgaaaaaccccagggtatataccccagacaatgcaactgcttcactattgtcattgtacccatc
ttacctcacttaagagttagtgtcacacttacacacacttaagagttgtgtaaaaacagaaggtg
ttgggcaccataaagacactaaatactgactggggagagaacacaggaagtataaggcttttctt
cctcc

>chr6_34106022_34106222
cccgagccccttggggttcagcaaaaagagccaggccaagagctccctcccaccttcctctcagc
tcccaaccttccctgtgttcccaccagcacttccttcatctccattcacctttgtctctgcaggg
cccacccagcccaggtgctcaggagcgctctagaattgtgaatggctgtgtggagtctggtttca
attcc

>chr7_148096667_148096867
tccaaggtcatactaagataaccatttattaaactctttggctgtttttttcctaaactgtaataa
actccccctctccaacattcaaaataatagtaaataattctctctgaaattaacacagtgttcct
attattgctttcacaacactaaaaagaaatagaactaacggagtcgaactgtggggcattctggc
aggca

>chr7_97000464_97000664
agtggtcagggaaggtgacatttgagtagagacttgattgaaaggtaaggcaagtcctgtgaaac
tctggggctaagagtgtttcatgcagagaaaaggaaatgtaaagacctcgagcacaactgagctt
tgcacatttagggggcagcaaggccagctagggggagtgatagagaaggggtagggtcagagaat
caagg

>chr5_492200_492400
agggaggtcagggccgggctgtgagggaggggagggaggtcagggccgggctgtgagggagggga
gggaggtgtggggactcgtgggggaggggagggatgtcggggcagggcctgttggggagggaggt
cagcggggactcgtggaggaagggaggaaggtgtggggactcacaggggaggggagggaggtcgg
ggtgg

>chr13_52001199_52001399
catttactcctgaaaaacagcaccaacagaacagatataatatacaatgttggataacatgactc
ttaagcaaaggatatcatacaggtggaattatacaactccataaaaagtaataggtcataccaac
accagccacaagccaaacaggaattatgtacacatatctctaatgtggaaattaatgacacattc
ataca

>chr6_37789422_37789622
gtaactcattgtaagacatatgtcctaaagaaagtcgctgccagtggcagctaatgctcaggatg
tatcaggtctctttcctggcaacacgtttatgtccttcggactgttaccatgaatggaatcttta
tcgaaagacttatatcaaggcttagaaactaaggaaatggaatagccttttacctttatttattt
attta

>chr4_183215006_183215206
actaattcgcactgaggtatgaatgaggcttaaccctcccaggggacatttgcattttaaaagag
gagcttgttaacccaaatgcctcgacaatgacaattagtggcatgtgtggggagatatattttgg
gaggttgggtctttcctgcgctccctgcgtcctaggccatcacaggcccgggtaaacttcaggat
ttcct

>chr20_61848555_61848755
cagtgacagcagggacggcaatggagtcccaagacggctccaagcctggacatccgcatcagtgg
ggtagcagacagcctctcacactgctgcaggaggaggtggaaactgcccccagcaggccggtcag
ccacagagaggactgctgctccaagttgctccaggtctccctggagccctgcctcgcctaaagct
gggga

>chr12_22850333_22850533
gtcttcttagaatctagcatcccagcagcttttgaagtcccaccagtgtatagtcttaaaaatgt
gtgtccataacattgaaacaagcattaaacgattttatctctctagatgagtttcagccaaagcc

tataattgtttttctcagactattgcatggaagtcaataaaacccaagccaagcattattgactt
ttgat

>chr2_184896155_184896355
taaaattagagtgcgttttaatattgtaaaaacccaacagaagttacatgggatataatattcta
agcctgagctcttaagtggtgtacgtctttcttcagtaaggtcaattggcagagaacaaaaatgc
agagctcaggagaatgaattcgtagctttttctcccatctggacatggagttacagctcagtcct
ttgct

>chr9_135358979_135359179
ggggccacactgtgaaatgggatgcatccagtttcatggtttcagacatgcttccccagctccca
ccatactgtcagaacagcgagactcagatggaaacggccttcactatccttcccagcccagggcc
cactccccaccccatgcccacctcagcacagtctcagaagagagcacagtctcctcaagactgag
ccacg

>chr8_126429618_126429818
gagtagctgggactacaggcgtgtgccaccacgccctgctaattttaatttttttgtagcaacg
ggggaggggggggtctcactatgttgcccaggttggtcttaaactcctggacttgagggatcctc
tcgcctcagcctcccaaagtgctgggattacagtatgagccactgcgtccggcccactggctccc
agtct

>chr17_25817073_25817273
gatggtaaggtgtcgggcaagtgtgtggactctgggatggattgcccaggtccattcctctctac
ctgttatcagcatcctggtgggaccttacagaaataacttctaagtctccgtttctgcatttgta
caagggggctgttgagaggatcaaatgggattatccttaaaaagttaagcatatagtaaatgtta
tgtaa

>chr12_45165333_45165533
atggctgtaattactatggctttaacgatttggccttggcattcctgcctttggattttatcctc
tctgtttaactccagcacactattttaatggtccaggcaggaaattaagcccctcctcccaatgc
tttgtttatgtagattcaactaaagaaaaggcttggtttcttctgacttctttcccagaacaaca
accat

>chr2_139406130_139406330
aaaaccagttttatttgtttctgggtcttgtcttataattgtaactgtaataggttcattgtttg
atgcacacagcaagtcaatatgtggagacaccgagttgcagtaaagagatttaatcatagggcca
ctgcatgaggagaggggaggaaacctcaagtccatctctctgaggaattttgggttagggattta
agggc

>chr13_100805599_100805799
gagtttagattagaggagattcatcctaaattgaagtacttgggaaagatccagtggtagatgtg
aaacttggagtccatcttctataataggtggtatctgaggacaataaaacaagtgtggatttaag
tttaaacaaagacttaaatgtattctttttaaaggattgtgaagtactcaatttctttcttacag
aagaa

>chr19_7865200_7865400
cacacagaccaagcggagaaaggaatttcttgaccctctttttgtgcctgtttgaagtcagccct
tattttttcccagtctttcaaatccatggagctgtgttccgggaaccaaggagaatattgttcta
ttagctgaaacagggacagaagattttgggtgctcacgtttaaccacagtggcacaataactgcc
gcacc

>chr2_15783349_15783549
ttttctgcctcaccaaccatggggaatgtgagctgggggggaaaagtctgttaaagctggtcatt
catttcagggggccaaggagcgattctgaaggagcaaagtctcctcatgctcttggccttttgat
ctactcaggctctgtggcaggaagacagggaggaatccctgatcacccacagccctccccgctgc
tgtta

>chr6_88641881_88642081
aggtagagaaattggggtctaaatcctaagactcatgctcccttctgaaaaaattgtatctgcaa
agatcagcagccaagtgaagaatactaaccatatttattaacatctcagcaagcttccaattaca
acaatactaccccacttgccctgtgaaaagtgtctctggttaatttgaaacctagcatgtggctt
aggct

>chr2_200848355_200848555
ccatctccttccaatctcccttcaacagtgctggattcaattaactgttgactttgttgattaaa
gtaattcatattcaaatttgaggaggtattgaataataccgttggtagctgtgttgtagaggagt
gtagaattccctaagaccttctggcatatctgaagaagggaagggttgaaataggagcagctctc
tctgc

>chr2_206148755_206148955
ttaaactatgcaattaaatcaagaaagcagattattctagtagctgacattaacgtttgaaaaga
gacaggattagctgtacttagtcacatataaactgcacaaagcgaacaaaggggaaaaaaattat
ttcaccaagcttcccttgaaagaaaatcgttccatcttgcacctctaatttttcagcttaattct
gttac

>chr2_208274755_208274955
gacttggcatggcaatggcgagaagtgaacagattcaagcaataatgaggaaacagaatccctgc
cttccttcaaccgtgggaatagaaatgatgcacagcttggggggaggaggagtgagacatttcat
tttttgcctaatattcttcaaacacaaattatcattccacattttcccttagaaaatcaccatct
cccat

>chr3_46422196_46422396
tgtgtgctaataaattcacaaaagccaacatattggagtcactcagggagttttacaaatagtga
ggttaaatccaacctcaaatagttctgattcgatctgcctgcattgctgccctgtggttccccac
tgtagaagctccccaggtgattctaagtgtagccaagtctgagaaatactgcctaaagcctgttg
gactg

>chr3_56155960_56156160
atggatcccacctacagctcagccccactccagggcagagacacactactgcacaactggcaact
gacaaaggacttcaccaagactattttggctttgccaaaatggatgaaaggtcacatagtaacat
agtctggttcttaaaaataagtttgtaatggcagaaacattggaaacaacctgtttatctatagg
ggaat

>chr4_13649102_13649302
tgaaataatttcctatatcctcttttaactcacttctaataggataacacatctatttttctca
ttctgaattctggatgattttattgttgagggaagacagtagatattgcatatctactttctttt
ttctgttttgtgtgtgtgtgtgtgtgtgtgtgtgtgtgtgtgtgtgtttacagttttatttaaacac
aaaac

>chr4_87370176_87370376
gctcaggcggtaatgcaagcaactgtaaatacagaggaggatcgctggctcgccacccccaccgc
accacccccacctcacgaccctgctcacctcctgctgtgcagcctagttcctgtaccagtctgtg
gccccaggggttggggacccgtgttctaactgactgtggtttgttccttgttttccaagtgtcat
tttta

>chr4_134017750_134017950
caggaaatgtccacacaaaaaagcaagaatagctaggaaaagtctatttctaacatcaataagag
gagaatcaggatgttggtaagcagtaagcaagttctgcaaaaacagttattgtaataataaatat
tcatgtgtacctgttccctcaggtagtatacgctcaccccacggtagacaaaactaagtcccatc
tagtt

>chr4_148547550_148547750
ttcactgtctcagtcataattttgcaaaggcagtttcactaataaatgaggaataaggtggaagt
tacaagccaaggtaaatggaggtagagtgatcagtgtagacatacaacataagaatgggtgcatt
gcctcctatgcagtacaggaccatttgtacaacgtcctgacaaattttgtctctatttacaccct
tgaaa

>chr6_84927681_84927881
ccaagtctaatggaacaagagacgtggcaaagaaaacagtaaccattggaggttaccagagaagc
agcagtattcacagagcaaagttaaatgtgaattacaactctgctttgaaggaattatataagat
taattggggggggtataccaagacaggaaaatccaagaatatttccatgaggaagtaacatttctg
ctaag

>chr6_107424507_107424707
tcacaagctctgctcctcctgggtggacacccagcacccagatgcctagcatgccacagacattc
ctctcgcctacctgactataggctacgtaggcgctgccacaactacccaaccaccacagcaccca

cctcctaaaggctgctgtgagcattccctttgcctcctggactctggagaacttctcattctgac
attct

>chr8_38889243_38889443
gggcgctctgattttagaattttcagcttttctgctctggtttctccccatctttgtggtttta
tctacctttggtctttgatgctgatgacctacagatggggttttggtgtggatgtcctttttgtt
gatgttgatgctattcctttctgtttgttggttttccttctgacagtcaggtccctcagctgcag
atctg

>chr9_107696779_107696979
ctaagaacatgcacccaaggtggttggggtgcagcttggtttttagggagatatgagacttcaat
caaataagtttaagaaatacattggttcagttcaaaaaggcagggcaacttgaagtgggggcttc
taggttatagatagatttaaaatttttctggttgacaattggttgagtttatctaaagacctggg
atcaa

>chr10_34703394_34703594
acatcctcgggtcaaaaccctaaggtttctataaaagctgaatctactgaccacagttaacaaca
atctggctgggcgtgggctcacacctgtaatcccagcattttgggaggccaagatgggccaatca
cttgagcccaggagttcaagaccagcctgggcaacacagcgagacccagtctctataaaaaaaaa
aatta

>chr11_133004590_133004790
tgcaaaggagcccagcccacatcaagcgagatgagccaccatcctgctacgaccaccaggtggtg
ctgttctccctgcacccaaagtcattcatgccaggagggcgcagagagctccctgcaccccagct
acaaggcagcaggaaacaccagtaaggttccttggctcagagctgttgtgcataattacagccac
tttct

>chr12_45525333_45525533
ctaactggaaggcataattaggaatgacttcactacagtctttcagtaggtgggactctggctgg
tcttagagaatggcttcattgtggaaggcagcagatttctgcacagctgagtagaaacagtgcag
gagggaccagggcttccatcacagtcactctagtgacatggttggggaaggaggaagtcagagtc
tcctg

>chr12_116719017_116719217
aaagcttagacaaatgtagagagaagtaggactctcatacactgctggtgggaatataaattaac
accatagctttggatgtccgttcaactagcatcaaaatctataatgcacacagcctttgacccag
ctgttcgacttctagaagtttatccaacagagatacttgtacgattgtataaagacatgcagtgc
aggga

>chr17_55989201_55989401
ttcttaagtgattcctcattaaccaggaggcattcctcatgtcccaggtgaactgaatctgtttc
attctcaggcaccccagtgacattcacaaacagacttctaattattcactcgctcattcactggc
agtcaaaaaacacttactgagcacctactgtatgtcaggatgcgcaagataaaaacctcttttct
tcaca

>chr18_59470220_59470420
taaaaaacaaaccatgagctaatagttcaatcttcttatgtaaatatgagcacttaatttatagg
ctggttggttggtttgctttgggccagcctttgattatcacttcaaaccagaaacgaaaggagta
agtcaaaatagtgaattatcattcaaggccagtattcccccaactgtgttctgctagacatcaat
agaca

>chr19_38326960_38327160
aagaatatcttaacgcaatgtatctgcctgatattactgctacctactctacatcaaatccttct
ccttcctcttcccctccttccatccgaatgaaattctgatttggcttagggcgatcatggaccc
agaaacttcagcaacatagagtactactatagtactccaaggctggcatggccctgtggctctct
gagct

>chr20_16349200_16349400
gctggtatctttataaaggtctgagaacttgtggtaaaaatatcccagtccattgtttccttctg
ttctgatatttaccagtgactccgatgagctcatttttttagcatccaactcttcctcaccttgc
tgctcactataaaactcagacgccagtacgctctgcggggcactgcaggatgctctctcctcatg
ggaaa

>chrx_153298806_153299006
atgcggagggaagagcaaggaaaaaggtgtggtccaggcagagccatacatccccttcctgccgg
tggagaagagcaccctacctattctagtgtgggaggcttccccgttcttttctggtgaccgaagc
agctccttctctacacatcactttggcccggtggggtctcgtcagaaatggccaccgtgaggccc
cacac

>chr1_19332213_19332413
tagtgttctcgtgatagtgaataagtctcacgagatctgatgattttataagaagaaacccccttt
ggcttggttttcattttgtcttgtctgctaccatgtaagacatgccttttgccttctgccatgat
tgtgaggcctccccagccacatagaactgtgagtccattaaacctctttttctttataaattacc
cagtc

>chr1_56840812_56841012
atctgggtaaaaccactaagtaatagaactgcacatgggttatttaaatccataatactcacttt
gagttagatgaacagaaaggagcttgccttctctgcttaattcttgcttcctcctcccccaaccc
cagcccatcctgtcaaaaaaaaaaaaaaagaaagaaagaaaaagaaaaagaaaagatactgcctg
ctcct

>chr1_64410012_64410212
aagaatcccagctggttaaatttagggtgcggaaagagatccaatcggcagataatccctggcca
tgggcccacagcattttcatttgattgtgcattggatacattttcattcattttaagcccttgcc
tttcagtattttgttccttggagacaggcaaactagtatcaatagctctcttagcctatgtcaaa
tggtt

>chr1_247550977_247551177
tagaggaattgcagtggagaaagtgtaattcacgcagagccagctgtgcaggggaacggagtttc
gttgttactcaaatcagtctccccaaacatttgggcagcagagtttttaaggacagcttggtggg
tgtgaggaagccagtgagccaggagtgctgattggtcagagattaaatcatagggaattgaagct
gtcct

>chr2_106886968_106887168
ggccaggagactctcaggaatgtgagtcctgctaacccttaaagggacacttacagagcaagagt
agcaacagtgagaagagggtggggtgtcccgtcacagcccttagcacatgtccgcagagcaaagc
taggtggctcccagccctcggggcagcaacacaggcccgcacctccactgccacagctatgctct
cagct

>chr2_240362063_240362263
aaggagctggtggtcagaattcctcagcaggtgaacagaaagccaggaaaacatcttcccaggga
ccttgcccagccccaggtgcagctgttgttctggaaaattactgcactgcatttcagaagcatct
ctaatccccaaatctgtcatcgccttgcccaccctggcccagcttttgtaaaagggcaaagagaa
gagca

>chr3_116868310_116868510
ctaacactaattcagtggtaattctccgcattgcacaaatgagaaagctgaagctcagagaaatt
catttacttgcccaaatttctactttctacttccctatgaatctgtaaccagaaaaaatgaatag
tttgtacttattttattctcattgtccatagtttaaaaaaaaaaatgtgtaatctaccagctgcc
ctgag

>chr4_149036550_149036750
aatcaactagaattttatttctaggtgataaagacctaatgaaaattcaccttgggatattaaca
ttctgattttgaaactgctcttactttcacttcttaattacctatttatttgctaatttcccata
actggtgtaaataattatttactttggtcatcagcacttggagacttggagatggtgctgagtat
tgtgt

>chr4_150105350_150105550
ctgattttatagctgcttgctcagaagcactggtgacgactaagacttgcaagtggcatctgaag
tgggggcagtattgtggagcctgagtccttaactcaggatttgatgctatctccaggtagatag
tgttagaagtgagttaaattataggacaccggccgggcgtggtggctcatgcctgtaatcccagc
acttt

>chr5_167155022_167155222
ctggccccaagtaacatgccaggtaacagtttcaagcctgggttacttgaaaagaaggctgagta
ggttggtgggaactgggaccagtcttatgatggaggaatatgctgttgctattcttgaaattctt

ttcctccaccccacttcatcccctaccccttgttgttggcaagatttatcttcaggtatccaggg
aagat

>chr7_100805080_100805280
tagctgggcataatggtgcacacttgtagtcccagctactcaggaggttggggtaggaggatcac
ttgagcccaggaagtcgaggttgcagtgagctgtgattgcaccactgcactccagcctgggcaac
atagtgagaccctgtctcaaaaaaaaaaaaaaaaaaaaagttaagaagtggctttagccagcatg
tctct

>chr7_146110867_146111067
aaggggactttcaaaataagcctttaaaaggaaagcacttttttacctatatttttcttttcttt
tttttctttttttaaaagttgagcttataagatcatgcctggtgaagctgtgttcatccactcctc
tcagcattagtggaagaattagagtattaacgaccttatagaccaggtattttgtagcctccttt
acagt

>chr9_27671600_27671800
tcttgggtaaatgagctgtttcttgggacattataaatcatatttggaatcaagtttattttcaa
gctgcttttgaagtctcgatgctgtgctaactgaacagaaaatgttaaatgtttgagggagtagt
gcaagctctcaaatgctaacggcatgagctggacttaaaaaaacaaatgggatttataccctctg
aggca

>chr11_44978024_44978224
taaggcctgccactcctggaccacccccaccccagtgaagccatacagtttggggacagtaagtc
acctgcggaccgacaggaacatctgacaaatagattctagtttataaatctctttagagtgtatg
agtctcaaagcctacttgggggcaggaaaatgttagaaacacaaattctcatttccacctcagac
aactg

>chr11_88162152_88162352
tatttgcaactgaagcaactgtgctttatgtcagataagataaagattaaaaaggagaaaaagtg
acatgttctcacagagctgttctaaatgtggtctgaagaacaacagcaatagcagtactgccttg
gaactggatagaaaagaaaattcttgggccccatctgagacctgctgaatctctgcgggggcaag
ctagg

>chr14_57325447_57325647
gcacgaagccctgtgcttgttactctatgtgcctattgcatagtgaccaagctgtcggggcgtgg
gagaaaggtatgaggtgaggctacagagttttactggccttgaagatcatggtaaggggttgagg
ttatattctttttttcttccccgcaaaagacaccattgttccaatttgttattcagtatagtcaga
gttag

>chr14_73401447_73401647
tctgtctcccatattaaacaatcggtgcctccaagccaggtagcacagcttattatctctgccca
ctccccagtgcctcacatggagtgggcgctttgtaaatgtgtgtggagtagtaagtggaaataac
ccagagcagaaagaagctacctgggacttaaatgtaaagtagccacaggttttgttttgctttgc
ttcag

>chr18_52882602_52882802
atgctaatgggggcaccaaatgaaatatttctcctggaaagttccctgggcaagttgggaagaga
atctgcttttcctcctggctatgccaagtgctgtgtggcctccaggaattcagaaagagaaactt
ccacatcgtgttaggtgctggcagagaggataaatgaacacatcttcattaattcgtttctgcct
attgg

>chr19_7211800_7212000
accacaggcaagagggaacgaaaacacttcttctgccaaatagagctttggagggaatatcggtt
tgtctggggcaggactgggtcacgggctacacgccttctccttgtattctcacgaattcaacacc
tggtaatgccggggccgtttcccaagtcacagactcagactctggggtggataattcccccatca
ctgtt

>chr20_4031000_4031200
ttctcttccaactcactctgctcctgatgtgattctcaacacagcagccagattaatcctttaga
ggctgagaccatgttcctcctctgctggaaaccctccaatgttccaacagctcccagcccactct
aagtataagccacagtccttacaatgtctgtgaggccctaggtgatctccatcatacttccctcc
tatca

>chr22_42813856_42814056
cggtgtctgggagggcccccgccccgtccccgggcttggctgtttgttttgagtcttcaactcct
ctcctgtgcctccccacccccacagaactgcaagcgcagacatctcatctgggcagcgccgtgga
tccctgcttagtctgcccaccctgctttacaggagactgtgttttgggggggtgacacaccctcca
tccct

>chr1_81717612_81717812
aagaggatgacctcccctgatagaggaagaccattattcctgtcaagagtagctttgctcccctg
caagaacctgcaaacagctcaaaaaaaaaaaaaaaaaaaaaaacccaaaaccaataaaagtctat
aatgtaaaggactccaggttaagtaatttcatttctcttgtctttgtctaaaattgtaaaaatta
cctga

>chr1_181561577_181561777
cctctgcttgtcctgcctcactacagctcagctccctcctccagtgaccggtgggggtgggggg
tggggaaacaatgaaaagtaggggaaaggtaatttatttcagtgtttacagagggtttgcgtgta
attagcagtttatttctgtgacacagctggcctcctcagcttggaacctgactttgaaatgctct
taaag

>chr2_15268349_15268549
gcctttaattatccttttaatgctaatatccttacttttgcagcagaattattaatgtgattgac
agcgggggactactccagacctactggggctgttacatgatatggaatagatgcaccataatacc
tttccaagatctgacaaattctaaattccaaagcacatctgttctccagggttttggctaaggga
ttggg

>chr2_118309530_118309730
atcttaccttggtccctccctctaagaggcatgtaaatggaacagggactgcaaaggacttctct
ttgttcttagctctcccctgagggcaagcacatttttgcaacacaggttaggctctcttttgcct
ttccaggaatgtgcacatacacgtgcatatacacacacatacactcacacatttgtgactttcta
tttac

>chr4_38123605_38123805
tcaagcacattcctgcattgtgcacccaaatgatctcccgatttaagacccccctgtgtctcacag
aagcttctggggctgaactttctccggccttggagggttggacgctttgaatgggaggagtggtg
gtgagtggagcatctctggcagcaggcatttgggagtctctggcaggaatcaatcagcgtagtct
ccaaa

>chr4_102248977_102249177
acatttcaataaatatatattaacaaaatgagaactgtcaacaaaacactctagttgtatgaacg
ttactcaatttttcatctacatggtactggacacctttcataaacaacaaccaaaaaaaatata
tttgaagttcagggtccacaaaaatatcaccagggaaaaaaagaagaaaaagaaaatatgaacct
aagct

>chr4_123746750_123746950
tcttggagacttaagaacaaatgcacttatgcatggccttttgaaacctaatgtttctacaagga
ggcacgtccatacttgcaactccttctagaagcttccccaaatctcctgcctccccacgctgagt
tatccgatgtctgaaatgtcacagcacttagtcttactcttctatggcctactttctactgctat
ttgtg

>chr5_54987643_54987843
aaagtaggcctgcatagtttgaaaaagcccccccaaatgacttgacatatattattcccctataac
aacaagctccttcctccacctaagtttagttagaccccagttccactaaaaggataagcgaacca
tcatactttcagtccaaagcagttattaaaattatgtgatttttattgccacctagtggaagaga
tagaa

>chr8_10195190_10195390
gaaccttatactcctcctctctcggcagctggtgcttccctgttcttcctcagccatgctggggc
tcactgcagcctcctctgctcaactcgcctgggtgcagccatcttcaagaaaacattattaaagc
aatattattagtatgcagcaaggtgccttctttctgctaaatcagtttgtaaagtggtcctcaca
aatgc

>chr9_77102580_77102780
gtagctgcagttagctgtatagtttttatttcaaggaaagaaatgtggcgataaactccaaggat
gagctctcaccaaattgtagaatctcaagggaaaacacagattcccatctactcctttaagcaat

agaaaagataaaaacgttttgtactaagaaatgatctgattttggtgagtgtgttgaaaagaatg
tagta

>chr12_52685533_52685733
aaagtgtcttgccagcagggcattccttcatctgcaggcattggggagggaaggggaaggcagag
gagaggacgagaaggaggagtagagcctggcgaggggaagagaagggaggccaggtgctgggaga
aactgggaggaggagacctgtgtgttaagtgtgatgtggggagaggggtttcaggaaaggctgag
gcatt

>chr12_111471017_111471217
agataataacaacagaaatccctattccaaaggacctgaggccccgccctcggcccccgcccgta
aagaaccctgagctactactcagacgaagccccccctccccgccaataataataatataatggcgc
ggaaactgtgggcggtggtggtgacatttcccacgactcagtggcgcccccgggcggctcccacc
ctccc

>chr13_54784999_54785199
ttgtggttggcaacactttgcacatattttcacatattgttggtaagagaatggaatgtgcccca
gggtgactttgctggggaacaccactgaaagcttgcacctgatttcttctaacctttccctatgt
gccttttccctttgctgatttgaaacagttccctttcattgtatacaccataatcataagtacaa
caatt

>chr14_99610047_99610247
cttcaccatgttggccatggttggccaggctagtctcgaactcctgacctcgggtgatctgccta
ccttggcctcccaaagtgctgggattataggcatgagccaccgtgcctagccaaaaatttgcatc
tctaacaagcttcaagctgtggccttggccaggggatcaggcattcagactcactaacctgaatg
ttcct

>chr15_49683508_49683708
caagcatgaaaataagggaaaaggcttgagtcccttcaagagaaattccaggcatatagctagcc
tcgagaagtaaatcagcaactcaataagcaagaaggacaagatgtttggttccctatagaaacta
aagatgacacatgtccctgagttgtttttcagaaacccagacccccatcagatggaaaaggccaa
ccaca

>chr16_52535899_52536099
tccctgcctccgcaagtaaaacttgagccatgccaatgagggaaaaagtggccgcgatttcccag
gtggcttagtaatacacagttttctgacacactgcaactccattctttcctaaaagttccttcca
agcctgtaggcactgtaagacaaacagctaagcacttcctgagtgcctactatttgccaggctgt
atgca

>chr18_35105602_35105802
ttagggttccttggtgactggctgatgggtctacagtatttcctcaaacttccatgacaagagaa
tgggattcatgaacatttacattgatcagacattatgctgaagggaggaaatggtactttctccc
tttaaagaaggatcctgcattttacaaaatcgaaaataattattaaaaacccctttcacaagatc
ccttg

>chr18_43185602_43185802
aatattaaagatttggttattaattaaacttgaccaaggttaaacagctggtaggcagcagagct
aaatttagatcccaggtctcctcactcccaatgcaatgatttttcttctgatttgccatgtcaga
gaacacctggtccctccattttatcctctcccagaaagataggttatcaggagcctagcatggaa
cagca

>chr19_5689800_5690000
tgcccaccaccacgcctggctaatttttttgtatttttagtagagacggggtttcaccgtattagg
ctggtctcgagctcctgacctcgtgatccacatgcctcgacctcccaaagtgctgagattacagg
cgtgagccaccgcgcccggcctaatgggcgttttaacagttcatacagtttggtattattattcc
cgctc

>chr20_57987605_57987805
ctcatcagtaaacaccagcaagccaccagctgctaccaccgtgtcctgcaagagtccgaccccaa
attcctctccctggagcccctccttcctgaagcccctttcagagggatagaatgttttctttacc
agttagcactcaggagtaaagtgcctgggaacacaaatgaaagggttactctctttgcagcattt
tgctg

>chr1_53056212_53056412
gaatcatacatcagttcattcctcaaattctcactgcgctccaaccaagtaccaggccccatact
agataccggggacttagagatgaataaaaccctacctcattcctcaaagaactcacagcatagca
ggatggacagatttcttcattcatccaagaaatatgtattgagtgcccatcaatagtacaatggt
taaac

>chr2_36523696_36523896
tcagggagatggtcaaatagtgttttcagtagtcttttaacagtcattacattactcacgttagc
gaagtgcctcggtttaaaataaatagctgcacaacctgatcataaagaagtactatatgatcatt
tgcagaggaggcaatctggtgagtacacctttcactaatgtttcaggcaacaaattttacctaaa
tacaa

>chr3_132911110_132911310
gtattgaaactagtttactcctcttattttggacacattgagtaggagatttgagcagtactttt
ctgaggagttgtccagcaggcatgtgggaagctcagggaggtgtccgtagttagaggcatcacta
atgcagaggtaactaagatttgagagttttgaagtaattgcctagggagagtgtttagaggaaga
aaaga

>chr3_148721510_148721710
ccatgctgctggaacccgggccacagtttgaggagcactattataggggaacattcctttggct
gagatgctggggctcatgtctaaaccagaattaactcactgccctgttatccacagtagacaagc
cagctgccagcaaactgtggaagagcacgcaaagtacacaagcattgaacaaatgcacagattta
aaaag

>chr4_5786899_5787099
cccattcttccttcttagaacgcatgggaggcctggaggtgcatcagcctttctctgccctagag
gcagcctgcgtgagcctggtgggctccagatggtggagcagtctcagacagccctgagcccctgg
cagtgctgttaagccattaccgcagccctgaaccgcctgcccctacatgcttattgtcagaaaca
agccc

>chr5_155351222_155351422
atctagtcatagcaaacctaggaggaaatactcatcactaatgcacaaaagaagacagaacaagg
aggaggaggacctgaatggggacaaaggcacctgaattcatcaaatagtttattgtcagcacag
aggaagggatgtcttgctatagcaggagaactttcatcacaatggatcatcagcctcttggcacc
tgttt

>chr5_174973994_174974194
gtagagacggggtttcaccgcgttagccaggatggtctcgatctcctgacctcgtgatccacccg
cctcggcctcccaaagtgctgggattacaggcgtaagccaccgcacccggccccaggtgtttata
tctattgggagacggcctttccctggcgccggctgccaccaagtattattttagagacacagttc
acaac

>chr8_34895858_34896058
atccagctatcatgctccaaaatatttgcccacactaaaacctgcactcaaatgtgtatagcagc
tttattcataatcaccaaacacaggaagcaaccaaaatgtgctccaatatatgaatagataagca
agatgtaatacatttaaacaattgaatgtttttcagcattaaaaggaaacaagctatcgaaccac
aagaa

>chr8_56044446_56044646
tgttacagtgagcatgctgtttacacatggcctgtgcacagtcttaatgagagccattctgggga
taaaatgatcagatgtctgccctagaggagcagtttcatatggcaggtgtagcgcagagcccgaa
gggtgtggcactaccagatcattggtcaggccatgggaatgtccaggtaaccagcagtcagcagt
catta

>chr9_109521179_109521379
aggcaagtcatagaatacaaggaaatacttactaatcataaggaactgacattcacaatacatga
agaactcttaaactcaacaacaacaaaaaggcaaacaacctgattttaaaataggcaaagggctt
gaatcaacatttctcccaagaagatatacacatggccaataagcacatgaaaaaaatgctcagta
ccatt

>chr9_138993979_138994179
ccagagggaaagtggccagcagggctgcaggtgaggccctccgtccctctgggctacgtgggcgg
gccggcacccctgactcccaggagtgaccaggaaggcatttggcccctttcagaacaagtcactg

gcccctgctcatctggggctcctgttctagtagcaggggtggcaggctggttaaaaaagaaatcc
aggtc

>chr10_88626620_88626820
cagcaagttatttatctccttgaacctatcttttctctgtaagtaggaatgattttacttactgt
aaggatcacagagatgggataatgcacgtaaaactaatttgtaagttataaaaatgacagtgaca
atgggatgctaagcaggtaccattaaataccaccacatatctgagtttctctgatttaatctgca
caaaa

>chr12_40500533_40500733
tccactcatgtaacgggtaattcccagaaatagaaagaaaactcaagaccctgatttgtgattcg
gcaggcagtctccatttccccgccccaacatgctaaccaaaaggtaggatagcttctctcctgag
aatccccactgttgccctccatgaggcactgttttaacagcctcatttacttgaaacaaagcaaa
gataa

>chr12_113950017_113950217
cagagctaggggtggggcttgaatggtggggactctgcctggggaggaggcaagacccttgaatg
caggaagttcactctcactttcccctctggctggtgaccaggctttcttctctctgggcctcagt
ttccctatctgcaagtagggaggcagtttaaatgcctgtaaggtggagagggagggagttactgt
gactt

>chr13_108867199_108867399
ctgacgcctcccagacccccccacctgacgcccctcaggccccccgcctgacgtcccagacaccc
ccacgaccggatccccagaccccaatctgacgccccacttcccctacttgactccccacacat
cccccgcctgacgaccccccgacccccgcctgacgtcccagacaccacccatctgatcccccaga
ccccc

>chr13_115070298_115070498
tcgcttccgagcgcgagagtgtggcggaaacaggaggatctgcaaggcagaaggttcggggactg
gtcctgagaagagggaagaggcagagtgagtcactgcacgcacctggcctccatggacgagcaag
ggcatcccagaaacgtgtaaatgaccccgagtgtgactgggaaggagaacttattccttaccagg
aaact

>chr14_102411647_102411847
cttgcaaatacgaatcccgcctctatccctttgagatgtatatctatctccttcagctcaggagt
gtctttctttaggacctgaaagccgtccttttgaaatgtaatcatgaggaaggttagactcctcc
agtctctgtgggaagacagaatcctaacttctataattgacagctgggctaatcacatgacattg
accaa

>chr20_30511939_30512139
cattcctctcacctccaccccgggcctggccaccatcctcttcctcctagacaaccacgcccgcc
ccctcactggtgcctgccgctgctctgcccacttcactccaccccaacgtggcagccagagagga
gacacccttccatggcttcccatggattgtgagacaaaaccccaaaactgcatgggacctgacct
ctgcc

>chr20_38724786_38724986
cctggactacagctatatcttgttgccacccttcttcccaatccaaagcctcctttgcatcctcc
tcttgtatcccccaccttaacccacaagtataagatacctctactccctccttggcgacccttac
catctcattaaaacctaatcacctttaccccactcaacaccaatatcccatcccacagtgcgctt
taaaa

>chr1_82130812_82131012
tctgcatggcattttcaaaaaaatcttcaaagacagatggataaactgattatttagaattagta
gagtttgccaagtaagcatgtttatgaagaccatttgaggtagagaacatgcaaagatacattat
acaaacagcgtaagtacctggaaatcacagaggaagtgtcctgatatcagttgcattttccttta
aaaat

>chr1_110031277_110031477
ttccagattcccccatcccaaaggagggttttggccccatgggggaatgaaacaaagacacatga
acacagctgcaaatatgcccacctctctccctgccccccaacttgcccgggccaaagcaccctgc
cgtccctcaccccttctcagttcatacctactccccagctctccactctgctgtattctagatcc
actca

>chr1_164056176_164056376
tctttccctgatatctctgggcatctgtgaggaagaatttcatgttacacagactcagtctgatt
ttcatccataacctcataggacagattggcacctaactggtgctcagctcctgcaatcacagcct
ctcattgcataatgagttgctgttgctcttcacattctgttttacccaaaatgataacataattc
tgctt

>chr1_248056977_248057177
gttctcagatctccagctgcatgctgggagaaccactgctctcttcaaagctgtcagacagggac
atttaagtctgcagaggttactgctgtctttttgtttgtctgtgccctgcccccagaggtggagc
ctacagaggcaggcaggcctccttgagctgtggtgggctccacccagttcgagcttccaggctgc
tttgt

>chr2_80109692_80109892
caggggaaaagcatggcctggagctatagaaatggatgtcacccttacccctcccaggaagctta
gtgtgttaggcagttgtacctcccagggctggctgctgcccctccgccaaggagcataaatggct
tagacagcaggcagccacagctgtggtgctggtcactactccccctgggagcttggcaggcttaa
gcagg

>chr3_65376360_65376560
atggttaaaaaaaattaccagtatttttaaaaatcaagagaccacttaaaaagcatttaatttct
tctcattagcccaaaatcccagtcaaacatgtttaagttctgttgcatatcagatttgtgctcag
agcacatcacaatctccactcggagaattaatgagacagcagctcttatctaccattccttttaa
ctgaa

>chr4_140774150_140774350
tttataaataggaacacacagtacacactttactactaccctcttgtgttacagacaagacctga
cattacagaatgactaggtgaccagaaccagaaagtgattcagttacagaaccacagtcagccct
agtatccacacattccagggaactctacacttctcatttctgcttcccttatacatacagactaa
ggaca

>chr6_38443822_38444022
ccatttggcaggctggaaaaaattaatttggggttgccattgtgtttttcactctgtataattta
accgttaaatatcaaaggttcactgaagtaaatgaaagtagtttttaagcttaacggttagacac
tttaaaaattgttttcaaggcagcacagcacaaaggccagaatcatctgatcacaaattcctccc
aggga

>chr6_41540422_41540622
ccagccagaggcagacaaaggaagtttggagtttgtttgttttggctgcccctcaccacaatcca
aattaaaccagaatttgctagtacctcctccatcctgcccagccagcgcttggtttctcttccct
gtgcagagcccagtttcaggtctggtacattcaggttctgcttttgccactgtgtcccgttgggc
aaatc

>chr7_89778864_89779064
tcctttttctggcctagagcataatacttcagccctgggggccccactcacctggtattctatgt
atatggtgccccctgcagcagccctgggctcattacaagtaacactgattcttaggtgttctatg
ccttttttaccaatctctttagaggaagcagatgaacatttagaattttaacagggaatgtttacc
tgagg

>chr10_1563600_1563800
ccaaaaataacattctaaggccccctaagcttctgaatgcacttcctccccagccagggctcttt
taaaatttaacctgagagactgtttgaggcaatgttgggaagtcggggtcagacatgcctcatga
tgcctctccggcattaacagcaacacagaccttaagtctgataagaaacatttacagcctcttct
ctctg

>chr12_64453733_64453933
aagtcctttttttctgcttggggaaatagcacccccaaaagacatggttgcaggcccatttacttg
atgattgttttagggcattgcttcttagacttttaatgtgcatatgaatcacctgtggatccttt
ttaatcttcagattctgaaataggtcggggtggaactcaggatcctgcatttcttttttttttctt
tttcc

>chr12_81243469_81243669
cttttctccacccccaccattcctcaaacatattgaactctccaagttacctaggtctctgcaca
tgccgtttactctgctaggaatgcccttccacctggcagactcttattcatccaaatttaatatg

atgataaaaggtcaaattcaaatattgtctcctctattaaatgttttctgcctccctcaagcaga
gtgag

>chr13_53480999_53481199
ttctcatccagcttaaaaagagttaagtgagaaaagcaaggtgcaggaaagcgtgtataagaaga
tctcagttttgtaaaacagaccatgacaagctgctcaaactccgcatgtgtacgtaaagggctat
actggattacacacagatgaaaatctgggacacaggctgagttcttaaaacttactgccgggagc
ggtgg

>chr13_113273999_113274199
aaaagaggcccacgtgtggcaggagaacagggaatgagggtagccaagggataaggcagaagcaa
aagaacagcaggtgcagccagttctgggcaggatcaggcagcactcaggccacagcctcactcct
gaaataacgagactgtctccacttcagcctctgattggccatgagctgcctccacttcagcctct
gattg

>chr16_52295899_52296099
tgaggtgctgcctgcaaggacacagaagggtcagaagaaacaggtataaaataagaaccaacttc
attggcccttccctagcacaacaatcatgtcagtagactggaccagtgattccaacacaatggga
aaggttgggattaagaagcgttctgttcccatttagttcttttcctgtgtctctcagcctcagtt
gttcg

>chr18_37227602_37227802
acaggttgtttgttctaatcctatggataagtctttccctcattttagaatgttgcttcacatta
atgtgttgaccagtacttagctgaatacttgagtgcctcttgtcttagtccatttgtgttgctgt
aaagaaatgcttcaggctaggtaatttactaagagcaaggtttatttggtttacaattctgcagg
cagta

>chr18_46509602_46509802
tccatggcaggaaaaacatctgccaaagacattcccttaagatgtcaccataaaaaggcttaagg
tattagttctctctagggcattttcagacagttcttggggaatagctgtttgtaaaaagagctca
ccatttactggcctttttttttttttttttgatcacaccttctaaagtataagctgttggtaga
aaagc

>chr22_18808600_18808800
gcttgaaagctctatctgtgcctgcacttcttggtccaaaaggcgcaggggggatgggggagaggc
accggggtcatggtgcaggctcagctgttgagcctcattccccaaacacaatgtgacaggtatca
atagttctggagttgatcatttcacagaaaggaaactgctgtcaacctgcccaaggccacacagc
tagtc

>chrx_104878144_104878344
aaatcaattaatcatattgactgactttcctagttcaaaaagagaaagataagtgatagtatctg
aaaagagtacaaaaaggaaaggtacattttcccattctaataatcaggcctacaattgctctcat
taactctaagactaagccccctgcccacttacctccagtcaccacaatccctaagtaaacaattt
gccat

>chr12_105051670_105051870
agcctggacccgaataaagacacgctttatttccccttcagttgacggaggggggattttgaaggt
agcaggggaaacagagtgagcagtgttcagggacgcagcccaccttcctgggtagttttccactg
caacagacccgccgaggccagccagccaaggatactcagagttttacaagcatgctgtgtggact
ggggc

>chr2_67556696_67556896
gataattgaaaaatgaaatggagaacctcttaaaatggcaatgtatttggaatatccagggagtt
cttgaaatgtgaattctaaggaaatgggtagtacttaagaagaaaagattaactcaaactcacta
tttcaagtgataaaactgcaagtgaatcacaaagggatggagctgccaatgcctgcagaggaaaa
ccaaa

>chr15_67841546_67841746
taggtgttctatgcttattcaattaagctgtctcagtatactaattttcatgtgttttcaacttt
atagatttatttaagcattgatgtcagattatatacccttttttagatttttgttgtaggctagat
taactttatttcggtgtcattaagtatgttttctgttgcttaagagtaaaggttgataagaactg
tttgt

>chr12_131433047_131433247
caaggcagcgctcttctttctttcgcctattaaactttctgctcttaaacctactccttgtgtgt
ccatgtctttgatttccttagcaggagacaacaaaccccggggatttccccagacaaacgatgct
gcttcattgggactaacttacgcgagtgtctcgttcttggttctctgagagcagagcccagtgcc
aacac

>chr3_193902106_193902306
agagtgagggaagtctatctccctgacaattccctaaagtgatttattgtggaggtcacaatagt
ccctgtttggacctgttacagtttgctaagctccactaagcttaggtagtaggagatccttcatt
ggaacacttgtgttgaagggaggaggaggaggagaggaagaagccgccactgctttgtttttagc
tctag

>chr6_6169801_6170001
aaggttggtcatcctagactagtagttgtcaataggaggcaatgttatccccaggggacatttgg
caatgtctggagaactttttgattgtcacaactggggtggagggtgggttactactggaatctgg
tggataggggccaaaaatgcagttaaacatcctacaatgcacaggatagtcccacaacaaagtat
ccagc

>chr7_74899664_74899864
tatgtcacacctcaagcagatttgcagtcccttgacagcagcctcatctggcacataaaggacct
ctgcctctctgcatggcttcccagggccccggagaacagtgaggctgccttggcctccacctgct
gctccagagggcagcacagccccggactccatctccccagcccctgtgggaaggggggacgttcct
ggagt

>chr12_8680133_8680333
ccctggctccgcaggggaaaagcacagcctgaagctacagaaatgggtgcttcccttccgccgcc
cagggagctttgcatgttaagcagctgtgagtcccagtgctggctactgcccctccctcaaagag
cgcaaacggcttagacagcaggcagccacagccagtgctggtcgccctccgcccgcgctccccca
ccccc

>chr8_145690992_145691192
tcgggaggagctcggggaccagaaaacgctctgtttgggtgggtgcgtgcgtgggcatagtggtt
tggaaggagcgtgatgaggggagaagggccggggcggggctgcgaggggcggggtctgggcgggg
ctgcgaggggcggggggtctgggcggggctgagggagataaggtttaggcggggctgagagcggc
gggtc

>chr1_113067277_113067477
ggggttgggcatctttgaagcaatgttggataacaagaaagagatgcttccttttcactctttgc
cctccctgtcagcctgagcacaaccatgaggttacacacacacacacagaggtgtacatatacag
acacatagagaacttctctcaggctgcataggagttctgctcatcctcctctccccaacaattaa
aaaaa

>chr14_92803247_92803447
gcatcacctcccgtacctggaggaacgcttgctagcaaccactgtgtcttgctcattggctgtcc
aaggggaaagagaaaataaacacctcctaagattaccaggtacaatagtaagtaattaggctttg
gggatttatctaggagccagtgctcaactagggctagggctggatcagattacttggatttttt
tttta

>chr12_119712417_119712617
gaaagagcacactgtaccacacgcccactggggcttcgggagctataaacattcaaccctagatg
ctgctgtggggtcggagcccgcgctccccacgacctgctcatctgcatgctccctctaggggttt
aagctgtggggcactgaagaagagagccacacccccatcgcacaccccgagaggggggataaggga
atttt

>chr1_65862012_65862212
tctgctcaaaatgcctccccatttactcagcatgaaaaccaaagtccttgaaatggcctgtgta
gcctctgtatgctctagcatctcagtgcccgtgacctcatttcccattctttcccactttactct
ctgcattccagctgtactggcctctttgctgttgcactggctgtttcctctgcctcatactcttc
cccag

>chr8_17770720_17770920
taattataggttggatggtgccttcctgtccatctggtactaaacagtgtaaaattgtaacactc
tgataaacttcagatgccatgcctattccaattaaacccatagaagctctctgtttgggccatgt

ttttggccactgatccagggctgtaacagaaatattagcgcctgtatcgacaagtccttcaaaga
gtttt

>chr11_113755190_113755390
cccaagagacggaggttgcagtgagccaagattgcatcattgcattccagcctgggaaacagagc
gagactcctgggggtggcactgcagctgagttgacaaagatagggaaagctgaggggaaaaatgg
gagttttagcactgctaagtttagcttaaagctgcctccttacatactttaagttcagcccaaag
gtttc

>chr17_75966005_75966205
actagaatgctttagttaatggggtaagctagaagtaagctgcctttgataactcatgtaagagc
agcatatgaatggatgataactgtctttcttccatttaattcagctacttcctattcaatctcaa
atctcagttggaaagcaatttccctcacttgacccatttttcttctgcattatatctttcttctg
gttct

>chr15_68585746_68585946
acactgcttatttataaaatgagaataatgattatatgtacatattcagacatcaaaatttaatg
acattctattttgagaagggaagacaatgctgaagaaagtaaaactgttgattgaaatgctgaca
gggtggagaacgaatttgaagacagtggaataaactatgaactatgctaatggtaattagatttt
ttgtt

>chr3_75683510_75683710
ctgactggggcttctgcctggggttttgcaaagagctacttatgaatatagtctctccagattcc
ttgtttcaaaggaagtgagcatgagctagcaagtgtagcaaccccacagctgataaacaactttg
tcttggttttaaaccatcacatcttcatttcacattggaataaagtaagtgaaacctgctacccc
agcct

>chr9_72437380_72437580
aaactacactgggaaacagaacatcctcttttagcctcatatagggcttacctcatagctctcaa
gcaaaacacttagctcttctgtgactctgttaccccacagagatttgagtcataccataaatcac
cactagaagttattgttaaagagaaagactttttgcaaagggacagtgcttctgctctctgctgc
accac

>chr1_4131140_4131340
ctggattttgatgaccttgatagttgtttggcatactggccagatattttgtagaatttccgtca
atgagggtttgtctgatgtttttctcatgattagactggcgctatgtagttttggaaggaagact
gcaggggtgaagtgcccttctcattgcattgcatccaggatgcctattctcaacatgcctcatta
ctact

>chr16_22627899_22628099
ctataaaatggggactaaagtcctgttgagacatacaacgaatggtaacagagtaagagtcattc
ccctgagcctagtggcagtcatggcttatgctggaattgagacacacaaaggagtccagacacat
gatccaaggtcactttaaggagggccccaatcccatgctcctgtttctaaatgccaattagtgaa
atttc

>chr4_25216902_25217102
tccaggtgcatgatacaagatgtcagtggatctaccattctggggtctgaaggatggtggccctc
ttctcacagctccacttgacagtgccctaatggggactctgtgggggctccaacctcacatttcc
cctctgcattgccctaggagaggctctccgtgagagctccattcctgcagcagacttttgcctag
acatt

>chr6_106901707_106901907
gaggccagttggtatacctgggagaggcggaggcttgaagccgcagatgaactgggatcccagtg
tagggatggagccggagcagggagaagaagggcaggaacttgagagagcaaggagaggcagagtg
tggttcaggcgaccctgtctctaagtgtcactggaatggggtcagccaggcgctgtctctactct
ctact

>chr22_48583336_48583536
gaggcctgtctccttggcagtgctattttagtccttgtaaacacagccaccagggaacaaaggcc
atcagagcgctcagcctggccccacggaaacctctccaggaggccattctgcctgctcactccaa
gggctctgccttcaggtctgccgcctgtgccgccccctgcccgctcctcctgtgcttgaaatctc
tgttc

>chr17_9124875_9125075
tggtcttctgtgtctgcatcatactgcggggatttgacccgagtccacgtggctcaaagggaagc
tctctgggcatcagacaagggctgggagctccccggtgcatttccagtgttccgccaggctgtag
cggggccgggagtctgcgctgtaaacggctcaccactcattcccatgcaccggaagttccgctgc
ccaga

>chr2_8364749_8364949
gtgtcactgtgtgacacctgtgtgagtgtctaaaccccgccatccctgctacgcctgccttcagt
tctctgttccctctccggatggtaagctctgtgagggcaggtgtattagttccccagaaaagcag
ggcaaataggatgggagggagagagaggggaaagaaaagagagagagagagaggttttaaggaat
tggct

>chrx_108427544_108427744
aaaactagtttttatctcatgttatttccccgttaaccatttttgcagcatgtgaatgttagaca
ttctttcaatcacctattccattgccccaagaaattgctagctaggaaaccctgaatttgtactt
tcaaagggatgccttcccagatgaaacaagttaaataatttgtagaactcagatctaaacactgt
tattt

>chr7_114465164_114465364
gtctaccttgaggaagtctgggatcagtttacagagtaggcagagaaaatgaacatgaagggatg
aacttgtatctaaacaggttgttctgaccctgtgagctctggagcaccttggattttgcagttct
aatgcttgtcccactagtttttgttgtagtattctgaagcattggctctacttgtaacacaaata
aataa

>chr2_178012354_178012554
agcaaagatatttttaagaaatacctctgtgactactacacaatttccatatactccaagctaca
tttgtgctaaagatgtttttgttggaccatcgcgtatccctgaagagataaacctggggagattt
ttagaattccatactgaacccccctcccattattatgatagcagaggatggaaatgggaataagaa
tgctg

>chr2_122444130_122444330
gaaatgtagcaggacgagccacagacgaaactcctcagacaccgaattaaagaaggaagaggttt
ttattcggccgggagcatcagcagactcatgtcttaagagccgagctctccgaaaaagaaattct
tggcctttttaaaggcttacaactttaaggggtccatgtgaaagggtcgtgataaatcgagcaag
cgtgg

>chr2_21512295_21512495
tgggcatgtcccagttaccccttggggactttcttaatgttatcatggcaacatacaattaagtc
agattacatttttatatgcagggagaggtgctaagatactattctaaattattcatcagttcac
ttttcacaaaacaatcccagatatgactttaagatgtgcaatattaaaaaaaaaaaaaaccagata
atttc

>chr13_60118999_60119199
gctatcagagagtcaggaagttgcaagaacttcagaaaatgtcagagatgactatagctatctga
agcatcatagcagaggatttgcaaagaaacaccctgaggctgctggaaaatttcatttttgctga
aatctgcaaaattgcctactgctgctaaaggcaataatatagtttgtgcctttcttatgccttcc
aaatc

>chr1_117492077_117492277
aaaactctggctactattactgccacgtgtccctgtgggcacccggacacaacaggagctggcac
aaagtggcagaggccgtgtcttccccagctggtgtgggtgtgacctggctaggtgagtggtttgg
agaatgactcttaacctcttcagcttaacccctcctcggagttatctgaaggagttatctcagga
ggctc

>chr3_20435996_20436196
tcaccacccccctgtgctccctcccatgaggggtggagcatggtggacctaagcaaacagtgtttg
ctcctgacagcaggtttctgcacttcctcactgtagttcttgcactcgttccctttttgtgaagga
ttgagcagggcaggctgagtacatggggcaccgctgtcatgaatcccacaaaggggtcaagaaaa
tatcc

>chr7_121128164_121128364
ccagtagaagaagtgaccctcaaaattatggtttacaactgcattgggcattaaccagtttcgca
tttaacccagcaatgttatcctcctgttaatatctagcttttcaaatgatctttgcaccggtcat

gacatctgactttgacatgtattcattttctgtttccataagtcataattttgcctttgtgaaaa
tgatt

>chr17_14293075_14293275
tgcctctcccccctcccccaacgcattacagccatccccttctctcctctcaagtatgtgacgagt
gactggcctcccatgtttataaggctgctctgcaatgcgattgctcctccaggactttattaatg
ccatttacgattcccgtaataactaaaccacactgttctccaaacaccaggccttggagaagtca
ttacc

>chr1_241800377_241800577
gaagagcatactcacctgaaagtctttaggctaaagcaacaatactgctaaactttctaatattc
atcattgcacagttttgagctctggttttcactactgttaaacctactgaaatataaatcagtac
aagctgtacctgtctagcccaagatgactaataatcctggatactggaaaaacaagccattcaat
tggaa

>chr2_171537154_171537354
gtaataggttgcaaatgagtttggttgaaatgtgagaatagctttgtaactgaaaaacagaagta
ataccagcaatgtggagaagctcacataagaagctcagacattagcaatagcaaaatcagagcgt
gaaattcagattagccctccccaggctgtgagaggaagggggtattgcaagacttggcatggcta
aaggt

>chr20_21919200_21919400
acctttggcatactggtgactaacagagaactgaaaggaaatgtaactatctgaccatggttggc
tcatccagtgaactaatatgaataagagaggacatggtaggtttcccttgttgttcatgtttttt
agaatgccgttgccttctttctgtgtttggagtaagttccggtttggatgaaaagggtggcttct
acagg

>chr7_41995675_41995875
atcccctgccagctggctgcctccctttcctctgggaactcagtgaatcacacctccgcagcgcg
tttggctcttgcaagccctgaagggctggaggcttgtgcctgccagctccgaggagctgcagccc
aacttctgagcatggatcatgtcgggaccctggaatttctgagcacaggaggctttccagctgca
gggga

>chr18_3048200_3048400
attgccctccgtcctctccctcccgtccctcttgctctgtttggagtctaaagtagaagcatagg
aagtgggctagaaggcagcacatcccagggccgtctgcgacttctctgcatgcagagacagatgg
cagtctctgcaattactccccatcatttcctccagcgatgctcccagtcagactccaggaagatg
ccaga

>chr11_8890824_8891024
ccttctggcccaattaggggataacatttcccatacatacacttgaacaattttcttcccccta
tcttggggagtttttccaaaggtaatatttgccagatagcctgggcccagtggctcacgcctgta
attccagcactttgggaggcccaggcgggtggatcacctcaaatcaggactttgagaccagcctg
gccaa

>chr12_50275933_50276133
ctgtgtccagctctgctcagctgcagggctgggagtggcccccactgtgctgagaattccccaaa
ggctacggtgggagagggcaggatgggcagacgagagaccagggggtgctgggtgctagttatgg
gataggctggagctaggggaccacagagtccgcaggcctgagggtcaggacgaggtcagcatgtt
tctga

>chr5_49845643_49845843
catattatgaagttctggtttggtatatgcaagattttgactgtttgttcactctatcattatga
agtatttccctttgtcacttttaatgtattccatccctgacttcaccaatgattccaagttctgt
atacagggtttggattcttggctcctctttctatgcaggtgctgaggccttagccaccatgaatt
tgcta

>chr2_169391154_169391354
cagtaggatcactcctgtatagggttcctgacaacccctgttggacggtctcaccctgttgggtg
gcatggggaacaacaggacccgtttaacaaagcactttgtcccttggtggaaggggtgtgcctca
ctgtggggaaacacagtcatctgggctgcccagattcctcagaactaccaggaggaaaggctaag
tctgc

>chr11_76158552_76158752
atgtaagccatgctcactttttcaaattatattttactatcaataataaaacacattttatgctt
gtaatttattatttagtgtttcttttcccttacagcagcctttctcaaccagcattgagggaaag
aattaaaccctaatgctaaggtatccattgtatgtaaagaactaacttcgccctgtgcatctaga
gtgct

>chr4_92191177_92191377
attatacttttgccgcagagatattaatgtctttgattacaccagctccagatgtagctaagggt
gctttttgtaaattctgaataattctgaattctgaaaaatcacttggcctcaagcttttggataa
agagttacttaagaattttcttatgtttatatttccattccatgtctaacactgttttgcttttt
gatgt

>chr4_180067206_180067406
agcaatggaatgctatgcaccctcaaaaaaaagaagaaatcctgtaatttgtgacaacatgatta
aacctgaaagacattatactaagtgaagacaggagaaataataaaagttcaagagactcagtgta
caacatagtgactaaatcaagtgaattaacatatgtattacctcacatacttatcattgttttgt
ggtga

>chr4_184722606_184722806
atgggacatgccatcttttggggtattctgacataagaaattgagagtgtgctaaatgctttgag
gactctttgttttgagtgaacgagaagcagttacttctgtcttgtgagctggtaggggaggtttc
ccaggtatgaggcttgaacttgatcttgtgcagatggagatgggggtggggcgggagttcctgca
gagca

>chr6_20881421_20881621
tggaccccatcctcagagtctgcgattcagtaggtctggagtagagcttgagaatttgcatttct
gtcaggttccaagataatatgatgttgctggtctgggactacactccaagaaacactgaaaacag
actttgctttcacctttgatttatgaatccctctttgccatatataatcatagcatatattttgt
atatg

>chr6_108026507_108026707
gcagagcgttgccatcaatctcctggtgggataggatcagaagaaagataaacccagaagatggt
cagagtcatcgaacagtggctaaggttaaggtgtggtcaaggggtgtgctcagggcatatggatt
gatttaggcagcatattctcttggtcctggggcaaggtcttttaaaatattaaagcaaacaaata
ctgtg

>chr10_99628210_99628410
caggcctgttcccagagaggggctcatcagactgtggggacaacggctccagcctcttaggtggg
ggctgggcagtcccctctgggtggttctcatgtttgttgtcactgccgctaagggctgcagtgag
ctgtgtgcagcctggactcactccctctgctggaacctgggccgtgtgtgggttgccacaagtga
gcgtg

>chr11_95026552_95026752
aggagtatgggagaggaaactgatagacagttggggccagtttttggtttttttttttttttttt
gagagagagttttgctcttgttgcctaggctggagtgcaatggtgccatctcaactcacggcaac
ctccgcctcccgggttcaagcaattctctcgcctcagcctccctggtaccttggattacaggtat
gcgcc

>chr16_4238999_4239199
ggaggaggagaacactttctggtgaagccaagggggaggtggcagccttaccattcaggaaacctt
tgtgccctcctccactggagccctcagttatctaaggcagctgcgtggatgcccaggggaatgag
gcattgaggtgctgaggagagagcagttctttaagtttcagaggacaccacgccttcctgggcgg
ctgag

>chr16_11759699_11759899
cacaacgccacggccagctaacttttctttttagagataggagtctcactacatttccagggct
gttctcaaactcctgagctcaagccatccccctgccttggcattccccaaagtgctgagattaca
ggtgtgagccaccgcacccggcctcatctccatttctcagacgaaaaaactgaggcacagaaacg
ttcgg

>chr16_49573299_49573499
ctcatcggctgcattcagctatagctttctggtggcacagcacatgaacagaagtgcagcctcgg
agtcagattgcacaggttcaaatctcccaccaccatccactagcagtgggatcctggaaccccac

ctccccgggccaatgttccccaccatgtaaaatgagaatgattgtagtgcctacatcataactgt
tggga

>chr16_61973699_61973899
attgtaaatagctgtcactaacggactatctgccatttccagggacataagcattatttgtcaca
tggcacagataagaagctgagactcaaagaatgtaagaaacccatactagagcacatagactcca
agtgaaggggacaggattttagctagaaatacctggctcacaaaacatcaaaccacttccaacac
atctc

>chr17_7614475_7614675
aagatggttgctttgtccaccagaggtcaagttcacctctctggtgctgtagttcccagctcctt
cctgattttctaatcgctccttctggggaacaggaagttgatattgccatggtggcggggtatg
ccgtcacctcagtagttttactgtaaaagggaaatttgaacaacaaaaaccaaaaaaaaataaaaa
taaaa

>chr19_55652188_55652388
acaaaatgggcatccggctgaagaagtaacatttgggctctcagggcaggggaggcacccaccgc
cagcttagcctgacgttcgcgttccttctcagttctgaagcgctgttgctcggctctctctgacc
ggcgccgctcctgggaaacggagaagcataaggggggtgcagggacgtacccccagtcttccttcc
ccaga

>chr22_17943000_17943200
gtgtcctgaaagccagagaagaaattattttaagatagaatcaacttaaatgctgcttttaaact
tagtatgatggaggctgagaaccatttgttaaatttggcttggttgcagtacaatggtgggaatg
aaaatttgtttgcatcactaatactggcattaagattttgtatagaagtttttgttaccatctct
gaggc

>chr22_40640254_40640454
gcgtgtttcacacctagacagaggcacacagtctgactccaaagaaatccagcataagacctaag
gaaatttcagtaaaatctcctccttggttgtgtggattttgcatattaggcttttatatctttat
acccagaggtgcagaaggagaaaggctaatatttgaatgcagacaaagtcagtgacaacatgtag
gtaaa

>chr1_10899813_10900013
tcaggcccgtagggattgctcagtggcacagagcaggtaacagtccccaggcctcgcctaacaca
gaaagtgctcagccagctctttcgtcaatagatgagcaaagcgtggatggggcaaaatgagcgtc
ggctgcccagtctgctcagatcttcaggtggggtctcagtgatagggtcctcagcactgctgaga
tggca

>chr1_108067277_108067477
cgctctggcaggaaaccgttccttcctcatgtgcacagggaccacttcccagtccactcttgtta
acgttataacaaaagcttatgtgtgaccacttttaagggaggggacatattctttcaggacagtg
atttgttaggtatgaaataaataattattgatcaactgcttaaccacccaagtaagacatttgta
atctt

>chr5_68090044_68090244
atcacaatttgcagttcatgttggaaaatgaatttcacttaccaaggagaagtgcaaaattggta
ttatttaagatagttctaccctcacaaagcggggagtcaccctcactcccccacagccaggacag
ccaatgtgcctttagatttagaaatgggctggtctgtcacacgtgcactttctttctcttgtcag
caaag

>chr6_2908401_2908601
aggagatgagccttgaaacgtatgttgaggtcaaatcgaaaaggcctgtatgcaaacctttgaag
cccagagctgactcccttccatgatcacctggtttcagctataatgggcttggcactagcctccc
atcctctgcccatcttcttgttctcaaataatcttggttctacagaagaggatgtagagaggaga
agggg

>chr6_71469479_71469679
atagtgaattgattggtgtctatattggtaaatccttgacgttcgacacagtgaagatataatac
tggaaacaacagctgtaataactttactgcttaataagtgtttgccctatgccaagccctgacca
tggcattttatgtacattatctttttatttctcaaaataactcggtgacatagagcataatatt
ttctt

>chr6_71707879_71708079
gagccaagtggtctggctcagcaggtcccacccccatggagcccagcaaattaagatccactggc
ttgaaattcttgctgccagcacagcagcggtctgagattgatcttcacagcagcggtctgagatt
gacctgggatgctcgagcttggtgggggaaggggtgtccatcactgttgaggcttgagtaggcag
tttta

>chr6_151936707_151936907
aggctaaggagaataaagaaagggccagaaacatgatagaagtggtaaccagtgaaatgaagaca
ctaaaaaaatctctggaagaagcagaaaagagagaaaagcaggtgggtctaaatctggtgatgaa
accttgtttaggaaatgacaggacaattcaagagcttgatattaatacttattcatgcatttctt
tttaa

>chr6_157193708_157193908
cctcccaaagtgctgagccacctcccaaggtgcgagccaccactcctggcctaacccatcggttt
tcagtctgtttggtttattggcttgctaagctttctttaataatttctattcagtaggacagagc
cttccagggaccagaaaaaggggaaataggtatctgagaaataacaaggtctcttcagcaccctt
ttcca

>chr7_30489875_30490075
agagtcactggctgagagtacagagaacagactccaggttttccaattcccaggctagggcagcc
ttcgacccccctgcaccccatgtccccatgcaattatttacactctgacactggtgtgacaggcac
tgggagtcagttccaaatccctactgctttttagaaatgcatgagtcaagtaatggcaaatttaa
ccccc

>chr7_139672531_139672731
gttattaacacaccactcattttgggaggggaatatcatcaagtcccagccaggatgtaaacccc
acgactaggaagcctcgtccgctgggtaccttggtaggtcccagggccgagcacagtgcctggca
cgtgagaaataggtgttgagaaaagttatttcactcttccttttctaactggaaaccaactccaa
gcaca

>chr8_144368825_144369025
gtggtgctctgtccaccttcgggggtgtccctggtgcccagcgcagcaggagtgaatgtcctgag
cctgagccagttctccttgactgcacagcaccaaggtgccagtcagtgctggtcaggctggccgc
taggtcttggtgcacccgccacttcctaccacccttgcattttctcctcttagggccactggtgt
gtctc

>chr9_117954379_117954579
cacagagatgttgcatatttgaagccagctatgcatttgaatgttctctttggaactgttatccg
ttatcattgtgagtttggagcaatgccacttgcctggatttcttcctttgtttcctacctggcaa
acctaatggtattccctaaactccaaatatctgcacatcatttccctgtttataacatttcattc
tatcc

>chr10_88183820_88184020
taccttagcccaaactcctcagagaaatgcatttgaactttctttccatctcctcgttcggcaac
cctataattaaacctctttatctgctgcaacccagagaatattaacattctcagggtattaactt
gctgggtacatcaggtaacagacctgttaacggtttcaatcccactgttgggtatctacccaaaa
gaaag

>chr11_116331190_116331390
ccttgggagtggccttggggctgttccagcagggaattccaagtgccaggtaggaggtaggccag
agcggggcctctaaagcacaaccccagagcaaggaccctgttacctaggtcttcaagtggtagca
gcctaaagagcaggtgtttactggtgttcatacagcactggcaaatagggagagacacagactga
cagag

>chr11_133584190_133584390
gcacaggtgtccatcattggccacataactagcgctgccattggttgcacactcatctctcctgc
agtgcaggagcatctcaaggcagtggacgtgccttactcatggctccaccacagcacccagcaca
gacctggcaaggggtaggctcacagctgaattttgaatgccgatgcctttcgtctcatggctttg
tattc

>chr12_118553617_118553817
agttttagaacaggaatgcaaggaaataaagtacacgtggaagagggccagtagggaccttgaga
gattcaagtgcatggtttgacctttgacttgtggttttatatgttggcagtctgcatgtacaatg

gcctgccagcacttgggaggggctgcatgtgagtgtgtttactgaagctatatgtgtgctcactt
gaggt

>chr13_94212999_94213199
cagagcgagactccgcctccaaaaaaaaaaaaaaaaagaattttgctcaatatccagcataaccct
gttaattttgtgggaacataataaaagctactttgataacaaagcagaaaaatcagctgtttgtc
ttcttgcgcatgtcaaaccttaaatttggctgtgttttctgctctcttccccagtaacttcattt
ttaag

>chr13_111179799_111179999
aggagtcagaagaatgtgccctgctgccagtctgagggtcaaagtgcgagagctacccgggtcac
ctccctcttctgggaaaagccagcctgggaatgctgttgtttgggcgacagagccaccctgttgg
caagcagcttgtgctctggaattcagcacctgaagggcaacattcctcccttccgcacgcctacc
agggg

>chr18_53019402_53019602
ttcacaaggcagtcaccaactaaaacaaagggtccttgttttaaattaagacagtctttttgatt
cctaaatagctcaacaaagtggcatgtcttctagtgaaagcagttggtcaaatatgacccataag
ctggaaaatcaaaaattcaaacctcccctttccaccgccatatagctaaatatcataaagagaac
ctctc

>chr19_51556588_51556788
gcaaccagagtgataatccgatttcagatattcctcccctgttgacactctccaggagctccccc
actacaatgggagaaagaccacctccctgactacagctaaaaagattccgaatatttggctccag
ccaaatcctccagtctcattccctacccagtcactcgttcatcattactgtacagtcatgctggc
ctgcc

>chr22_35159600_35159800
ccccttgtaaggaagagcatgtctatgtctctatacagttccccatttcacccagaatcaaagcc
acagcctggaagtggtgcagagagcctcacaggctgtgcccacccccatttcctcttaatctcct
ctcttactctctccttcactcactctactctagccacagggagctccttgctgcgtctcaaaaca
cacca

>chr1_65326812_65327012
tcttggcactcaacggtgaatggctaaatcggcattattcctgcttaggagaaaagaaacagagg
cagtgtggttcatctcatagcagacttgctctagaaggtgaaactcccggattttaatctaggaa
taatcccaggcccagtgaagagtttgctagattaagcctagggagctctgagagttggagagagc
agagt

>chr1_91604012_91604212
agcagggtcttaggctgggattgaaacaagggtggccactggaccagactagtttggggcagcca
gcaaaggcagctaatcctgggattggacacatgtatgagatggtcctatctgaggagctctgccc
gggagactacttcaagcaagtttgaatgaggagccagaggaagagccagctgtcagtagggagaa
gaaag

>chr1_205407777_205407977
ccagctgcagtgctggccccaacctcagccctgggtgaagaaggggctgccggcttggaggcaga
ggacaactggacctccactcagaacactttgaccttgggaccccacccatgttcccaaagccagg
gctcaagatatctagtatccctctggagccaagggagttctcccgggggaggaagctctctggat
gggct

>chr2_1771393_1771593
ggcacgtgccgcaccggatccccagagtccgagcccgccccaccccccacgccctccgtggggct
ggtgcggcctcccaggtgctggtcccccagcacttaccactaagccgccgcacctgcttccttct
tattcaccctggccctccctctgcctctccacaccaccaggcttgagttaactcacacctccttc
ctctg

>chr2_97612273_97612473
gtggccacaggagatgctctggagttagaagagccagagctaagctgacaggggactcaagagct
tgtttctgatccttcctaatggggagcgcatcagagacaacgtggcaagaagcaaaggagagtgg
ggcatcccctctgcctcagcggaggagacacataccactgcagggagcaggacaagggaagcctc
tgttc

```
>chr2_189318555_189318755
aaggtttccagcaggaaaagatattctaggttacatgtgtgaattttgctagttctgtagatgct
aatgcttcaggtgcttttggcctttctttctttccactgcactcttggctccccagcctggacct
ggtcactccatctggtaatgatgcagggtttttctcagccactttgccacctggggacccgtatg
gctgg

>chr4_80475976_80476176
gagttaagagtggcagtttggggatagcaccaggagatatcagctgtgatggcttggaaaaacag
tgtaaactggcagtgtaaacaagagcagggcatgtatgagtagttgagaatggtgaataggagta
tgactagacagaagatagtagggatgacaagttttctggggcacagtccaagttggtctggtgtc
tggaa

>chr4_120518752_120518952
ttacatgcttaatctaataatagcatcactgctccaaaaactacgcagttggaattcccttcaga
gaatatggaatatccacactggaaaagctcatcttttgatgagctttgaaggtcatttcttttta
aaaaaataaaaaataaattagtcacagtaaagctgtgaatgaagtgattacactagattccatca
attgg

>chr5_67486644_67486844
ccctccacccacacacattcacttaccctacttatatgtgaatattgcaattaagatggcaaaag
gcaggcagcagcgcgagcacatgtggccatgatgaatgcaaatgttaaagattaacctttttaa
aaaaagcaggtttacttaattttgaaaagtatcatatttgcaaagctaatttagtatttttaatt
cccac

>chr5_175302794_175302994
ggatcaggcagcaggattttgggcagactcgggaatttcagataaagaatcccagcaggctgaa
gaagagctacttagagccacagggatgtaagcattgagaagactcagtggtgagtagaggtactt
tgcaaactggtggaggggtggcagtggtaaacaatgtctgatgaatgcatgctggcaagcagagg
tggcc

>chr6_160663010_160663210
tttacttccatcacctggcttccctaagctgtttctatggacctgtttgtgctgttccacaacaa
tctcccctgtagccattgggtaatccctgtcttgcaattcaattttctgttctcaccatggcctc
tgtattttaccccagcctgtaagtgtgagaggtgaactgctgggcctggctggacctttacgtgt
tctca

>chr7_43641075_43641275
taaggtagaattggaaattgatccagaaaagaccacctgaaatttctgtctccaaaatctcctca
ttcttgcaaaacctgctcttcagcctttcatcctgggacccatttcagttcttccagtcttcacc
cggcttctgactttttcttccctttctttccttctcctctttagtagttaattcaattcctct
cttac

>chr7_148999267_148999467
tagactggccgtcaagagagtcagggatgagctcacaggagcatgacgcttgtgctcggtctcaa
agaatgagtaaatgtcttcagggactctccaaggcgtgaatcccgtccaaaggcctcagccaccc
acctccgccgccacctctgctaccatcctgcctcaaacatcatgctccacagccaggccttcccc
tggct

>chr9_10331400_10331600
cttccatctgaaagcccagacactgtggagcagaggcaaaccatttccccattccctgtctgaat
tccctgattcacagtttttttgtgttttaactagtcttgaagtggttagttaaatagtaagagat
ggttcataaagaatttggtgctgaaagtggagcagtcagagttgcatctgctttcctgctcttgt
gttat

>chr9_126126379_126126579
agagatggcagctgatgaagagagatttgggaagctggttccatgcatctttctcatcgttccag
tgtctcagcgtctgggtgtattagtgtacatgccagtgcaggtcacattcgtccatgtgtctcct
agttccaagttctgttttgcatctgtaaagatctgccagtacctgggggtgtctgcagtctgagt
gcatg

>chr11_44212824_44213024
aagaggtgagcaaggcattttggccaaaggccagcacattgtcagaataatacacacttattctt
ttggtatctcctcctacctttgagtgaacaggttcatccagtgtatttgaaaattgcattgacag
```

ccaaggttttgaaggcatagctgcgatagatgagctttgcctcttctccagctctgttgctggat
gcctg

>chr11_70020952_70021152
agctccctgtcccatggcagtaaagaaaagagtgccatcccattccagcaggtacagctaaaaaa
agaaaagaaaaaagaaaagaaaagaaaagaaaagaagagtgcagcccacactgagtcctcacaaa
ggcctgatgctcaggcagccgacagtaccccacccccgcccgccacctgccacatcacagtgcgc
gtgtg

>chr15_60852308_60852508
ttttgctccctatgagattgtattaaatttagatctctccagaacatttgactcccttcttaagg
agatttatgttgaaaattctcacatagccacaaaacgcagtccattttatattaaaatgtgagtg
tatttctaaaatttttttcttttgttttgtttgtgttctcaagaatgctttctaaaacaaactcat
gtgac

>chr15_76400745_76400945
ttaaagatcattcatcccagtgcttttcaaactgtaacgtcttataaatctcctggggatcctgt
taagctacagattcctgattcactgggcctgggatggggtctgaggatttgcatttctaatgagc
tcccaggtgatgccagtgctgctgctggacccgggaccgcactttgaaaagcaaggatctaagaa
atcaa

>chr17_17527075_17527275
ttaggtgggacgtgctgggggggctaggccgtaggagcgggatggtgagaagaggcgatctggagg
gatcatggaggcatcgcccccaggatttgcagcgactgggccaggggggggatgagggattgcaag
gagtggggaggactcgggtttcaggcctgagcaagtgggacaatgggggtgccaatgtgaggtgg
tggag

>chr17_19078607_19078807
tccgggggccggctctgggccttcacacatgctgttctctccacctggaacacacttcgctttct
caaacgccacctcccatttatttttcagcatacagttaaaaaataaactcctgggaaacctgcct
ttatccagctggtaaagttctcctgtaggttcccacagcacccgctgcctcagtctcctccccat
cctgt

>chr18_44352602_44352802
agtcagaggtggcgctgtgtgaggaggccccaggaccctactgggatgacactggtaactccgag
gacaagcttggctaggagagcaagagagagcacaggccatatggcatgaaaagaacatgtgacca
gagacaggacctggatttttggttcagtttacactgtcagcatgctgtgtggccatgaacaagtt
gcttt

>chr19_28658760_28658960
aggtccatttttattgtcctctctgcacctggtgcctagcacagagcccagcatattcacatgtgc
tgaagaattatctagcttcttaagcatcatataaaataagcattattagtcatagaaaaagtata
agctccaattcactggtttagcgatgcctattttcctaggtttcttttctgattcctagatgtga
ttgac

>chr1_100900612_100900812
cctgtgtctgtggaactatccgggtgatgccaatacacactaaagttttagaggccctggtctag
agagagagagtatggagtgagaagaattttggtctagagttgaaccttgagaaggtttgactttt
aaagactgggaagtgaaggatgactttgcaaaggaggttggaagtagtgtttagagggtagaagg
aaaat

>chr1_113369877_113370077
acagtgttactatgacaccaggaaaacttagaactttgtgtgaaatagactggccagcattagag
gtgggttggccatcagaaggaagcctggacaggtcctttgtttcaaaggtatggcacaaggtaag
ccagggcacccagaccagttcctgtacatagacacttggttacagctggttttagatgcccccca
cagtg

>chr1_117553077_117553277
gtgtgtggttttcccaaggccttgtccgcagctctccataggttgctagtagcaacagcattcaa
caaagctgtgagagttctccctgtgttgtctctgcttgcaggcagaacagtaatttgacaagaaa
gctggtaccataggtgataagtcattaacttagaaatgataattctcaaactaccattctctagc
atatt

>chr1_208351977_208352177
tgggaacccaatttggaaggtatcgaatgagggacaatgtaaacacctgtatccaggaggatgcg
aggagaagaagagttccaataactcacccttaggggagtggtttgcaaaatgtggtactcagatc
aacagcatcaacatcaactggaaacttacaaaatcagcatttggaaaatacaaattctcaggctc
catct

>chr2_125083930_125084130
gcatttgtgtgtgtgcctgtgccaacatgtgatagtcgtgggcagaaactccaaaagtaaagcat
aaagcttttctcatatgtacccccaactatgattaaaaagcctgtgcttgacagagaaaaaaaca
aaataatattttattagaaagtaagtttattaaagcagctaagaacatgggctctggaggcagat
tcctt

>chr3_71095510_71095710
gttctgatgagtgtagaactaatttgctcaaaaatcaacagatgaatgcaaaaagaaaaaaaaag
gaaactaaacaaagttttcctttttataagagaaagtggttgatttctatcgtttacagaaaaat
aaacctgaatttatccctagatattaaaacatggcatttataaagcattactaagatttaaaata
gcaca

>chr3_72745110_72745310
cttagaatgttgacaatttcctgctaagattctggaggcagcaacagaaagttgacagtgtgagt
tttggagatagcctgtctgcctcagctctcagccctcagcagccaggggagggggtgggaggag
tgggtaacttatgccctgtgcctcggtttcctcatacctaaaacggagagggcagcagaacctcc
ctccc

>chr4_62254805_62255005
tggatttgtatattagaaagataactctggaagcagcatgcatgatcatgtgactggaggcaggt
ggattacccaggaaatatttgcaataactcaggtgaggtatgagaaagatccagtttaacacaac
aacaatggggattgagtgggagtaacacatttaagattatttagaaggcagttgataggtcttag
tgact

>chr6_114649907_114650107
aattactacttgtcagtgtaatctattttagggaactctactttgaatgataaaagatgggcttg
atattgagagggtgggataggcaaggtgactgcttacttccccagatcagaaactcactctttct
tatgatgaatgagtacctttattgtgatgatgtcttatctgaattattctgtatttctttagtgc
atgag

>chr6_144258107_144258307
gttattgctccttcataaaacactgagtagaaaggaggcttcttaggacagaggggacttgttcc
tttttatttttaacaaaaaaaagaacctctgagaatcctgtcccctacgatgtggtcaaaact
aagccatggtgtggtgtgcccatatccataaacttgtgtatctccatacctaacaacactgctgg
gctca

>chr7_25946875_25947075
ggaaagccattgcagctccatccacacacaaagtgggcagtgagcctgccctgccaagaggcagc
tgctgtctgttttggggaaggctcccgcatctttgctcagcagggagtcaccttctctcttttgc
ctcccactggccacctctgcaacctgctcacacaaccaattgaaactttctggcaagacatgaaa
tgtgc

>chr8_7616790_7616990
ggtgcagtgtcaaaggggacgcgactggttccaaagctcgagaagaccatggggtcacttgggct
acatgagaaaacgccccagtgtgctggttcatcattccgactcctgcctgtctcttcccgtccaa
ggaacatggaccctaagtcgtgcaggtgcggatgaccatgggcagaattaggggccgtggcacaa
aagtt

>chr10_73336994_73337194
ctgttagttccagctcctgccccttctccatcagtgctgcagagagaatgagtaggggtgtgggc
agagccattccaagcaggaattgccaggcacagtgctgtgtgggccatggttcctagggccatgg
cggcctcctgagttgccaccgtcatcccccgagatacctgctttcatgatttatgctttcatccc
taaag

>chr11_11684024_11684224
atgccgtgcaccctatatttgcaggtgaaaatcagacttgtcccctgcaacactcccaggattcc
aaggacagaatgcctggagtcagtcgtaccaggctttctataccctctagcctcgccttcctctt

gcaacaaaaagactcacttggaattgatctacactgttgccttgcaaagtaactagcagcccata
atgtt

>chr11_45394224_45394424
gcccatgcttgccctggctctgccgggaagacagtaggtgctttgcttcctgagaaaggtcagga
agaggcgtggctcctccagctgtacagacgaccaggccagatccacatggccccgtttggtgctt
ggatgtctggtaaactgcctgccaaggaggaacgcaggtaggacaagaaaactgtgggtggtgaa
acgca

>chr11_45676224_45676424
cctggcctccctctacctctgcttagtaaaaatccaagcaaaccttggacacaggagaccacgat
ctatcatttctgttagaaagcaggcaagttggtgagctttcactcccaggagaaagctcagattt
cattatcagtgaacttgagatgagggcacagactgggagcagtgaacagctctatattggaaaac
tgtaa

>chr13_74547199_74547399
acagcaatatacaaaaagcagaaatgtaaaacccatttagctttctgaatatgttttgccccagg
aaatgcataaaataactttgttatttaacactgcagatgcacctgtgggcaccaaagtactctta
gttatttaaaataattaccctgtggagaaatcttaaggtctttaataaataaaaatctgtgcaat
agggc

>chr14_95723047_95723247
tctaggctgggatgggagagaaattgccaaatgtagggccctggtccacattgcaccgggaaaac
agacatggggaaactggggatggcaggagaagagctgtcagtggacagggccactaagcctcctg
ggtcaggggcagaattggagcaggagcttgaaggccccatgcggtgttagcagatagggaaaagg
agagg

>chr1_34944213_34944413
aagccgccctacttcctgctcctttgatgttgggctgagccacgtgacttgctttacccagtggt
gtcccactgggggactcactccacatctgagcagagcctttcaatgtgctttctggttgagctc
tttctcccttgagcttctgccctctgccacaagaacagcatgctgcatatgtgggctgccccttc
agctg

>chr2_52248896_52249096
ggagtgggaagctagaaagtcatgtgtcctgatcatgcctttgagccataatattagccttaaac
tgacttgtccctaacattttgtggtatggggcaatttagcccctaccttttaaaagcacaatagg
agaacttcctgttatttgaaacagatgtagtcctatcaagcaaccagcaatctgtaatgtgctac
aataa

>chr2_118607530_118607730
gctgatggttcagttacaggctctggggctaaatcccctggtcctggctaccctgaacttcacgc
agctctgagtaggggagtaaattagcctgcaggggggaatgtgggtaattctcgacctcattacag
ccctcctttccaatttcgccttcctacgagcagcttaaaatgcagaagagatcagctgagacaca
cactc

>chr2_154260954_154261154
aagcaagttgtatttcctgttctttgctgttagaagctttctaatttcacctaattgtgattttt
ttcaaaagattttttttttactttagaaaaaaaatctaggaaaagtatttcccaattttctgcta
ttattaagtaaaattaaggtattagtctacatcatttctctctgataataaatttggaatccagg
aagga

>chr2_213617555_213617755
cagattaagagacaaatcatcattgaatacacagtttattacagttccaaagaggaaggagcaca
ccatgtcatgcagggccacaccagggtcgatcaagagggagaaagagcaagggaaaagcatgagc
aggagcctttattgtaggttccttggaaaaggcaaagcaaggcagagtaggcaggtttaggattg
gctaa

>chr3_14133199_14133399
ctcttttactaatcctcctcagcacagaccctttacgggtgtcggctgggggacggtcaggtct
ttcccttcccacgaggccatatttcagactatcacatggggagaaaccttggacaatacctggct
ttcctaggcagaggtccctgcggccttccgcagtgtttgtgtccctgggtacttgagattaggga
ggggt

>chr4_79843376_79843576
atcctcatgtttacaacgcaggttttcccttgtatttccattcttatttaaagctcagtggtgaa
aaaccaagtacacaaaggaatgcctgaggatctgtgggatggattgtttaggccagggtttctca
ccctttcttctttcccaacacacgtgacgagtggtacaatcccttctctgattcaccgtggaagt
gattc

>chr4_139792150_139792350
ttagcacatgttcgatgtaaaggaacatccttctccattgaaagagaaattttatttctctggga
gaaaaaattacaacgtgtagtcactaggatgcattatgaacctgttattgcgtgtgggatttatg
atcaaggtctcttctcttccgcaaggattcttcagctaagggcggaattacagatttcagcgccc
acaaa

>chr6_138437307_138437507
aatcagcataattttttttttttttttttttgagacagggtctcactttcttacccaggctggag
tgcagtggtgcaatcacggctgactgcagtgactgcagcctcgacctcctagggctcaagtgatc
ctcccttctcagcctcctgagtagctgggactacaggtgtgcaccaccacgtcctgctaattttt
atatt

>chr8_3896792_3896992
tccaaattccttcatgatgtgtactttctatgtaaaccaacgatatcaaatgtgtttgttttgga
ctatttgctgctgaaagctcttcaccccactctctctcctggctttgggaaggattagaaaaatc
tggttatataattttcaggatcacattaaaaaatcggatttcctagtcttgaaaccttacatttc
ttctc

>chr8_54230447_54230647
tgtgtatatatgtgtgtgtgcatttatctttatccagaccatagcatttttaaatgagaaaatac
tctttataaaggaataagattgttaaagtaaatgaaaatggaaacacgacctgaagaatccctga
gcatacaaagccagccgggcctcctcgtgacctcaaccttgcttgatttgcaaatataagtgaaa
cttaa

>chr10_26055194_26055394
tgaaatatctgtttcacaagtaaagcaattttaccccaccctactcaccctagatgaatgcctg
atcatctttctgttaaagtgtagtcccctgcacatggatgctcttaccagaacaccagtgctgtc
atttacattccatgaggaacccaggaatttaaccaagccttcgcggaggctctgaagccagccct
accat

>chr10_50135194_50135394
aacctctcgttaagtacttctgctgttgccaaacagcagccgtctctatgtgctggtcaaatgat
gcctcccccgaaccctccccccctgcttccacacacatcctcagacccatgctaagaggtacatca
tgcccgtgtcttcataaagtgacctctcctttgccatttcagtcatccgcaacactctcctttgt
ggtag

>chr11_84101552_84101752
taggggagaggtcaggaaaaactgtatgagtttcgaatggcaaggagaccgcaggtggtaaacag
gaatgacctaaacctgagcagcatctatggaatgtgaagcctgggcccaaagggggagcacttgg
ataaattatagagtctggtgatcacctggtcttagaggctgtgagaaagagaagttaaagctaaa
attcc

>chr11_127639390_127639590
attttgtggtaaggggtgatattgtggggttttaagaagaaatatttgtcatttagaattactgg
tgatggcctggatacagttttgtatgaattgaaaaactaaacggaataagagaaggagaaaaaca
ggtgttaaaggactaagaattgggagtacctaggacatctaattagagagtacctaaggaggttc
agcat

>chr12_125281847_125282047
aagcagcgtcctgttgagtcccccgtccccatgccctcccccagtttcaggagcagaaaagtggc
cacacggaagcagcttccaacacacgttcgtttttcaaggctaaaggccaacaggcagcaagctg
atctcctgatccccctccccaacgtgcccagctcaaaaaacaaagaaataaaggccacttcctct
ccccg

>chr14_65048047_65048247
tttagaacaatgaatgcagttagagttcagtcctcagtctccccttatctgaggtctacatgcca
gtggacccatttggtgggggtctgagtttctgaaaaacaacgcagggacatatgttaaaatgtta

tttttactttatatagggaaccaaacatcttgtgactctaacttccttggctgtcttttaagcta
ctgtt

>chr15_61436508_61436708
gtcccaaactggtaaaaaaggagctgagccttttagtttttggatgtagggtattccgaggaaag
gggcgtgacttggccaggggagtgtgttcagcgaaggctgagttccttctgctacccacatgcac
agcaactgaggactgagaacttgggtcctacagagggatctgggaagctcagcacagctgccatg
actgt

>chr16_84170299_84170499
gcccccactagacacccctcacctcttgtcaggggagcgccatcgtgtgcaagtaccttaaacgt
gtgttaatattattgttgctatgtatattttaaaatcacagagcatcaggggggctgatggcat
tcaaggtctggtcctcagccttgatggatagaaacccaagttcttcattccttagaggcagctca
agaca

>chr18_25358802_25359002
atatttaagcatctgcaaatacctagagcatgtttctgtatcatgaataaaaattataaattctt
agtataggacatgggctgtctgtaaggggtgcaataaaaacagaaaagaaccacgaatggcccca
atagcctagtcaaattaaatcagaatgtgagctttttgttgttattgtttaccatcaacttactt
ttgaa

>chr22_28605800_28606000
gatctgaaaacacacagactgcctcctcaagtgggttcctaacccctgagtagtctaactgagag
acacctcccagtaggggccgactgacacctcatacagacaggtgcccctctgagacgaagcttcc
agaggaaggatcaggcagcaacatttgctgtactgcaatatttgctgttcggcagcctccaatgg
agata

>chr22_46515336_46515536
ggctttgggtagcaccccgcaggcccagcagtagagctcatatggcctggggtcacctccggggg
gaggcaccctccagtgcccagccaggggcaggctgagtgcgggcaccggagaggccagctaacag
caggaagcccagccctcgagtgattgccccctgcagccctaatacatgacagaggggcctcgggg
tggca

>chr12_128643647_128643847
aaacaaatgttttataaagtcagtaaatatagaaatagcattataagcatattatagtgtcctgg
aggaaaacacccgccagaagaatcaaaagctccaaagtctaaaagaggctgcagaagcacagatg
tgaacttgaagagggtggaatgacgacttttgaatttcatcataaccttagatttaattagttgc
cacat

>chr19_10198000_10198200
aggaccgggtcacgggagggagagcaagagggggacccgacccgttcatggtgactgaccccccc
tctgaaacaggagggaccaggcattgtcggtccataactcatcacccaatctcactttttcacct
ctccatcatttgtccggccagggagcctgcaaacagtaggcgctcattatgtgctgattgtattc
aatgg

>chr3_47118196_47118396
actcattcaaaagaaaaaaaagtattccaagtaaggagaacagtatgttcaaggacatgaagagg
tgaatatgtatgagaagactgatgtgcctcatgcactgagtgtgtaaagagaaactgcctgctag
gcaagaagtttagacagatacggaattactttctctatcatacactaaaggctttgttcttagag
taatg

>chr17_71511805_71512005
cttcttgggaagaaggggctaagctgggggaccccaccctgcagccctgctcccgaggaaccacc
ttttcaagaatctggcttgaagatcccagtgggagaagataaattgggtttacatcccatttcca
tgctaactagagtttgaccttatgcaaattcacctcctgggccttacctgtctcatcagtgaaac
agatg

>chr12_32240333_32240533
aaaagtctcaggtgctcattaaagagaaagcacttgtagcagctgccccaagactgggctttctg
attagcatgctgggtggcttcctgacggtagaggggaatccctaagaaggacatttgacccaccc
acacccgcatattgcaaaacacacagcttctgaaatggtaaatggctggctcaatatctttgcag
tccca

>chr7_119393364_119393564
tggtgaggttacagtcctagtcattcagttaagcactaatttcagtgttcctatgaagaatttttg
cagatgtcactaaggcacaacagcttctgcctgggcccagggagctccatcctgctcccgatcgc
ttcttgaatactgtgaacaatagctttctctaatgcccatatgttctatcctgtctatgatcttt
ggtcc

>chr2_58724496_58724696
ttctgcggtgaactctttctctctgtaatccttccccttttacctggtcacctcttacttgacc
ttagtcagcagttcattcttcacttcccctgggaagcctttgattcctcaaaggcacataatgct
cttcgatggtctcacatgacatcttgaaccacagtttgtagaatgcctttcttggtttaattagt
tgatc

>chr5_120277901_120278101
tcaagtttagggaaattgctcagaatcaatcttagctaattcttgacttattttactctaatttg
ttcttcttatatttacactcttcactctgtagtttttgctttatattatttagttcttttgaatt
aggtcctgccgagttgaagctcagtcatttgatcacagatgtcactctctgctcatcacagtgat
cttga

>chr17_79899309_79899509
gcagccgtcgcgcatagcagggtggccagcatggcgaaggcggcggtgaagttgccccaggagag
ccgcaggcagccgtggagccgtgtgaactcacaggccaccaccagcgcagagacggcgaagcaga
agccccaggcggccatgcagaaggtgccctggacgcccgcaaagccaccccggtgggccaccagg
ctgaa

>chr15_83376146_83376346
ccccagtctcattccagacaccagaccaacttggactgtgccccagaaaacttgtcatccctact
atcttctgactagtcatactcctattcaccatactcaactactcatacatgccctgctcttgttt
acactgccggtttacactgtttctccaaaccattacagctgatatctcctggtgctatccccaaa
ctgcc

>chr3_39619996_39620196
atgaattgaaaaactaaatggaataagagaaggagaaaaacaggtattaaaggtctaagaattgg
gaggacctacgacatctaattagagtgcctaaggaaattcagcatagtcctgtcagcaaagatta
tttatttacttcaagagttaagagtggcagtttggggatagcaccaggagatatcagctgtgatg
gcttg

>chr15_93824396_93824596
ctagggatttctcctaagaaataattaggcaaagagccaagagttatgcacaaggatgtgtgcc
tcaggagtgtttataatacagctcaattggaaacacttttctctctgtccaggattaaaaaaatg
aaggcatgttatctcatggaatagtatagggtcattgtaaatgatgatgtagatttatatttgtg
tatgg

>chr14_96362047_96362247
ccttgggaaggtgtttccggcccatttttttcttgctttgaaccacagcacgtgctgttgccccc
actggaacactctcccgcttctcttcctccttcagatctcagcccgctgtcgccttctctctgac
cctggtcccttccactgcaggctgttgctccggtgtctctgggctgctccaccacgtggtgcctg
cccat

>chr19_12249400_12249600
acagaacacaaatattttaaaaaaagttatccatgaccttgtgaaaagatgataaaccactaaaa
tactgcatctatgtgaaagaaaggatgaagaaatatactctacaataaagaaagttgatacagga
ggctgaggcaggagaatcacttgaacccgggaggcagaggttgcaatgagccaagatcatgccat
tgcat

>chr21_20926729_20926929
gaaaaatgctaataagactcatgctgagaaaagctctcactcatcagggtgcatcattgcagaca
ttaagctgagactgtgaattgctattagccaaaactggagaaagactaacgagaatagtgccaga
aaatacttcgtggcactgtttgaattgctgtctgaggcaggaggtttgttgtattgtttttgaaa
tgatt

>chr8_728600_728800
tgccctgtgtcagtgtacccctgttggggggtgcctcccagttaggctgctcaggggtcaggggt
caggcacccacttgaggaggcagtctgccccttctcagatctccagctgcgtactgggagaacca

ctgctctcttcaaagctgtcagacagggacatttaagtctgcagaggttactgctgtctttttgt
ttgtt

>chr14_104320447_104320647
cttcttctgcagcatttccccagatcgcccagggatgacgcatctcaccacattcctccagactg
cccagacctgggtcacgcatcacaatctgccaccttggagctgtctatgtacctgttgccccagg
ggccagcaggctccatgaacaggtgtcgcctggtttcttccatgtacccacccaggtccaggccc
tggtg

>chr13_79856399_79856599
tcctctgctttgcaccaaactgaaaacactcattttcatgtaatttatggattccttcagctttt
tactcccgttaaacttaattactcgaagaaaaatttctcatgtaaaaacattcctttgggagatc
ttcaaatcccaaataaaagaagtaggtgtctaagcaggtaagtccaatatgaaaaattgaggaaa
gatta

>chr2_231769756_231769956
acaaacatgtcccaccctgagccggtcctctggagccctccttaaccactccaggcccactggcc
tcaaggcttctccactgccctcagggaaacccctccacccactcccagaactgtgctttggctgg
gggctcacacagcgatcactgggtcattggacatcagttttccccagtcagggtcattccgtctg
aaatg

>chr2_54612096_54612296
agatggggacggcactctctgccagaaaaccagtctcagcaaacttgctgagttaaaactatcag
atttaagctaaaaacaactatatttccaaaatatgcagcctgtgcaccatgggaaaaggaagact
atccgtacctagggagtagataagtaataaatagtctcatactcttttaaggagagcaatgcccc
aggct

>chr13_56227399_56227599
ttgtccatactttagatgaactgaatcaagcctactatactcactgacccagctaaatgtatact
ttgcaaaggagtaataagaaatgtcctgtgtggtgtgtgtggccatgtgccaatgtgaataaatg
gtgtgttgagcgtagagtggtagctagactttagaatttgcttgagggtattagtttgaataaat
gttta

>chr14_65434647_65434847
tctccctgcttcaagtcttaaccccttcttccagccaacctcattagtaccctactcaaaacccc
tcctaccattgctaaggctcatgttccacctccttggccaggctctcaatgccctctgccaactg
gtccctgcattcattcattaatttaacaacaggcattgaatctgcctggccctgtactagggact
ataaa

>chr2_1240049_1240249
tagtctcccagcattttgaagtgtaaaaattaatttattatagcagacatccaggaccaaaggcc
ccacgggaagcgatggatcctgagggacaggtgctccccaggaggaccaggaaaggtgatcagaa
agggctgtgcgaaggtggaggcagagctgcctgccctccccatgcaggggacctgcttcctctgg
cttct

>chr12_7403733_7403933
gccaagatggccgaataggaacagctccggtctacagctcccagcgtgagtgacgcagaagacgg
gtgatttctgcatttccatctgaggtaccgggttcatctcactagggagtgccagacagtggggg
caggacagtgggtgcagcacaccgtgtgccagccaaagcagggtgaggctttgcctcactgggaa
tcgca

>chr21_18840329_18840529
tttacagaaaaagcttatctagcctcttacatgaggaccactatttgtttttcttaaacagagag
taacaacttcagggctgcaaaatacctttgagattacctaattcaccctcttcattttacagata
tgcacccaaggtctggtacgcttttactaggtcagtgtttgggctgaagaaaacagggttgggtt
gctga

>chr12_96380469_96380669
ctaccaattcgacagtttttcttccccaattttctagtcgggaccaactctaatcctactccttt
ttacaggactcctaagtcctgtaaaaagattcctaagacagcacagagcttgacacacccagatc
cctgggtgctctcaggcaagtcactcaacttccgggctgtgtgtatctgtaaattgcagttcttt
caaag

>chr11_61991824_61992024
agcttgtattgctatctcatggctaaggttccaagctactgaatcttcatttatgtgtgtgtata
catgtccagatgtgtttatttgtatgtacacttattgttacatgttgtgtctaccaattggctta
taagggcatcataaattaagtaaataagtctaagtaattttcaagctcacatgacctaagtatga
cttta

>chr1_162369176_162369376
aagggtacccctgtaagggcttacgtctgtacctacaggcgggctttgctatcttaaggactat
cggaagtggacttctgatgggaatgcaaaaatgcaattaaaaaaaattccaactgttattttaag
ttcaggggtacatgtgcaggatgtacaggtttgttacataggtaaacgtgtgccgtagtggtttg
ctgca

>chr18_56436820_56437020
attcagaaacgggaacaaggctatttgaaaactttgcaatgttatggcagaaataatctttgcag
gatgtaaatgaaaagttccaagcaattacgacaaatttttgagcaattaatgcactgggtcttta
gccttcaatgagctggaaagctgagcaagacagaacttgaacccctaataattataacagctaat
actta

>chr11_94165952_94166152
cagagcgagactccgtctcaaaaaacaaaacaaaacaaaacgaaacaaaacaaaacaaaaaccct
tatctgagttctgaattcacacattcaattgcctataacttcccttcatgtaagtttctaggcat
ctcaaacttttcacatctaaagtggaacttttaattatatgcctatcattccctacaacacatct
gtcct

>chr12_43865133_43865333
tcctacgcccacggagtctcgctgtttgctagcacagcagtctgagatcaaactgcaaggcggca
gcgaggctggggggaggggcgcccgccattgcccaggcttgcttaggtaaacaaaggagccaggaa
gctcgaactgggtggagcccaccacagctcaaggaggcctgcctgcctctgtaggctccacctct
ggggg

>chr8_10405990_10406190
cgatgggctgtggcaccgcggggctgcactgggtcctgggagctgggagacggtttccctcactc
tgaggctggggtcactttggaccctttccttcctgacacggtgagccaggaatctgggccaacac
cttagccccggccattccccctgcctgcaagtctcctgcggaagcagacgaggagctgttgggac
agaag

>chr7_128678364_128678564
atggacaaagcaaaaaataacagatgctggtgaggttgcagagaaaaagaaatgcttacacactg
ttggtgggagtgtaaactagttcagccattgtggaaagcaatgtggcgattccttgaagagctaa
aaacagaactaccattcgacccagcaatcccattactggatgtatacccaaaggaatataaattg
ttcta

>chr13_110969599_110969799
ttgcaccacactcacacatacacacagatgcacacacacacgcacatacacatagatgcaaatac
acagagacacacacttgcacccacacacacacagaaacacatgtacacacacataaacacacatg
ctcatgcgtgtacacacacacacacttagccggaggacgctcctggtttctttcttcatgggagc
agcca

>chr5_174967394_174967594
tctctggctggtcccacctgggctcactcatgtagctgcattcagttgccagcagttccgagatg
gcctcagccacatgcttggtagtaggtgctggctgttggctggggcacctcagtgctcctcccca
cggcctctcctcccgcaaaaggccaaactggctttcttgcacagtggcctcaggacagcaataca
ccagg

>chr21_44167331_44167531
gagagccctgtagcaatgtggggaccctgggaccagtctcagttaagctggaacttccccggatg
actggggagaggagctgcaaaaggcagggtgggggtggggggccttcccgagatggcagcccagc
cagaggccagcgttcttgcctctgggaagagaataaaggccccagacagggtggagctagctcag
ccccg

>chr8_27509681_27509881
gctgagaccaaggaatctgggggtcctgcaggtgagacccagggatctgaggtttggttcttct
catacacattagggtttgtagtagttgacctaggtcacagcacacagccagaggcaagtcacttc

tgccttccagatgctgacagattgtggtaaacttgttacaacaaatttgatgtttgtaatcccat
tccac

>chr8_75779245_75779445
ccaactgggtggagcccaccacagctcaaggaggcctgcccgcctctgtagactccacctctggg
ggcagggcacagacaaacgaaaagacagcagtaacctctgcagacttaaatgtccctgtctgaca
gctttgaagagagcagtggttctcccagcacgcagctggagatctgagaacgggcagactgcctc
ctcaa

>chr9_72715180_72715380
cagataatataattaacttgcccaaagtgacattgtttctaagtggcgtggccaagatttgaacc
aacatcaagttgatgtgatagccacattcaaccactctaccatactgtgtccacagcaggacctg
atccagggaggcagacagttaaggcattcagaaaattgagtgggaagatcctcgtttgagttcag
ggact

>chr5_132936901_132937101
agtgactttatgaccaggaccaaaggtcacataaatatcgattggcttaaaaatgcatcaaacct
ctattttgcttataaaattattaggcactgtaaggaagaaaagataaataagacaggctccatcc
atatgctaacgtaaaaataaaagagtaagaaaacaaaaagagaaaggaaggactaagaattcaga
aaaag

>chr4_146813550_146813750
actttcctcttctgaggcttggaagaaggagaaagaaaggaccattaaggaaaacaaaaaggtac
actgcatctggcattaattcacatacactgccatataaaaagcactaaaaatgatttgggttttt
ttcttgacaattccgagattttatccataagaaagttttagtactgcttttcttcaggtagaaac
ccata

>chr11_126458790_126458990
gggggaccaggggagacgagggcatcagtcactccagcagggagcttgctgcagggagaaagaga
gaacagcaatttgggccgtgaagcctggcactcattcttcaagggggctgcaagcccccaggatt
ttcctggaacccagggcacccgcacactagctgcctctcccggtcccttccagcaacctgaggaa
atgcc

>chr7_189517_189717
tgtgtggctgtgtgtgtgtctctatgtgtctgtgtgtctgtgtgtttctgtgtatctatgtgtgt
atgtgaatgtgtatctgtccatgtatctgtgtatatctgtgtctgtgtgtgtgtgtatgtctgtg
tctgtgtgtatatctgcgtctgtatgtatatatgcgtgtctgtgtgtatacgtgtctgtgtacct
gtgtc

>chr12_74936133_74936333
attcactgtagggatctactatcaattagctttcaaatacatctgcagcaaatacttttggttcc
tgttttgtaggtttggagtttaatatatttagtgctaagctaattagttttctaaaaagataatt
gcaagacccctgctttaccaaatacttgacaatgggactttgaaggttatttcttatgttttctg
actag

>chr3_172082306_172082506
ctgccccaagggcaccccctcttgctgcaatcctgccctgtgtggggcagcagcccactagtgcc
tcatagggagaactatacaaatgtgtagtctgatccaaggttttccatgacacggtggctcctgg
gcaaagaacaaaagctccccaagcacttttccaaatgaggttcctggcatgaggcatttaaatat
gccag

>chr4_30918902_30919102
gagctctttcagaattttcacttctgtattaatttggttcacactgccaacttaaatacttggcc
acaaaaaatttattattcaaaactgtcaaaatgaatctgaagagctagtgatataatgctgatac
catactatagcattctaattaaaatattttaaaatatgaataaaaatgataggtgtgatatatta
taatc

>chr14_67723647_67723847
tcattttgccaaggctaaggacgcggctgtgacacagcctcaggaggtcctgacgacaggtgccc
aaggtgatcggggtacagctttttttaaatacgttttagggagacatgagacatcaatatgtacg
ttggtttgatccggtaagacggaacaacttgaggcgggggctttcagttgataagagttagcggg
gatca

>chr17_41956074_41956274
tggccagcagagggacccctgcgctcagagcgccgcaaaccctccacctcctgggaaggcgcagc
aagacataggaaggaggatggggctggagttctgcaatgaggccttgaagcctctcctccaggcg
ctgcctgctccctgacgctgactcagccctccagccagccttcccgcctctggctttctcctacc
cttca

>chr7_105669964_105670164
agctggaaagttccctctcctccacctctgtctctctctctctctctctctctctctctttga
cagaaccttatctacccccctgggaagtgaagtgaaggaacttgaaggtggctccctccagctctt
ccctacccccacgaataaggctttgtcatggtccaagcttcatgaatttgttgcataattcaatg
gctcc

>chr1_55461212_55461412
agcaggtgcggaggccctgggatgcatgcgtgttccaggaacagcatggggccagggtggtggaa
tgggtgagtgagagggggcgtggcgggaggcaagctcagagggttgatatgcaggcagcgcgggc
caattatccagagcctttcaggtcatcttaaagcatttggcttttactctgatgagatggggagc
cattc

>chr1_179553177_179553377
taataggcagcagggtggaaaacacattggtctgacccaggagctctctggatgtggttaagtaa
ttttaggaacccaaggaatgaactcaacaaataacccattaagtctttttggtttgtataacaaa
aacaaatccaggccgggcagtgactcactcctataatcccagtgttttgggaggccaaggtggga
gcatt

>chr2_97075673_97075873
ctctaattacttgcccagcatctaccaagcaggccctgtcagcgggagtcctgcctgcactcctc
ccggggcggatgctctcctcttggctagcccagacaggtgcccttgggctccccgccaggcaggc
actgacctcccgcggtccagccacctcctgctctcagggatgccccagccggatctcagcccagc
aatcc

>chr2_121624930_121625130
cccgagtccagccagcctttccgttagccagccagccagcgagcgctcctaaaccctggccgccg
ccggttataaatgattcatgcagccccgcctccctcggcggccacttgatgttccctgcgctcca
gcccggcggtccaccgcctctcctgccgctgggcagtgccgccgctgggcagtgccctccccgca
ccgcc

>chr2_132302130_132302330
gggtgatctcttcctgctcgtgcaatcttgagcatggcaggacccccacacccactgcggttctc
cggcctgcacctcccgcccacaccccatgctctgtgttggggagaccacctgggactaccgggtg
gggattagtgggcatcgcgggggactgtgggaacagggcactgtgggtggaggtgtcaggaacgg
gaacc

>chr2_200274155_200274355
gtgctgggattacaggcgtaagccactgcgcccggccgcaaacctccaacattttctagagcaat
acttggctgtaagatattgcatagttaatacgacttcactgaaggaatagctttttccatggaca
agatggtgaattaacatttttatcatggctaaagtatactgcctactttatattttcaacgcctt
acttt

>chr3_57186160_57186360
gaactctctcggctgtgaccagagcaaacgagaccaagcaaaggcaagctgccccccgcccccga
ccaaagcataccctcacccgacctttgtgtccagcatgcattccagcccaggagctccggcccgc
tgggcagaggctgcacaccattcctgccagcatctaaatcgctgctttctggcctttcactgtca
ggcca

>chr5_113785501_113785701
gtgaaatgtacagatgctgcctcctcagatctgctaccttagattggatttagaatagagtaagc
atccaaacatacacacacacacacacacacacacacacacgcgcgcgctcgcgtgcgcgcactct
tcccttagaagagcccttaacccagaatgggggctttccctgcagggtattcccacaacatcacc
tcaca

>chr5_141066816_141067016
gccttctttatcctggagaactggggcagccctggggtcagggggagggaaggaacaaacacaga
acacctcccttcccctgcatcctcattcccatgactgggccctgtctccttccaccctaggctat

tgacatgagctgtatttacaagatttttaatgcaattttactgcaaaatgttttataaagggtat
ctagt

>chr6_799600_799800
ttgaggcttatttctctgatgttaaacatttcagctccttgaatgtgtggggcatcctctatttg
taggtagaatcctggaactggggaagtaatttgaggctatctggtcatcttcccacctaataaga
caagtgcttttatcatgctgattctgcatggaggacatggtctcattttcccaggaactaaaggc
tgcta

>chr7_72978864_72979064
agggtctcacctaactgcagggaagcctgggacatgtagtccagctttgtacctaggaaactgag
aagcggagttggggaacatagcaatctttgccactgtgggcaagtccctggcagtcatgacacgc
tcagtgaattaacgtggggttgaggaaatttggggtgcagcacatcaagaaattcacttttgccc
tgggt

>chr8_25139883_25140083
aaatctctggttttcgtgtcccatctcctttcacctgctgtatgctgttattgatgtttttgctt
tctgattctgtaagttccttcagtccatgtaaagataaatacagatttactagtatgtagcaat
acttgtattatttctctcttgcatttccccctgtagattacaacaccagacgctctgtttgatgt
cttta

>chr8_62825446_62825646
ttaaacattttaaagtcattcttcttaatgcttttccagtaggtgagttacgtctgatgagttga
aaaaggacaaaaaataaccttgatttcttccagcattgcaccactgcaactgttctctcaaggcc
attagtgccccaatgcttaatccactggacacttttctgtccttataattatcaggaatctctca
gttac

>chr8_80031045_80031245
tcagtacatatatataaagtatataataataaatatctcttaatgatataaacatctgcagagat
aataatttggtaactagaaatattttataatattttttcctaaaatcctattaatcaggtctga
aaagtctgttccttccaattgttcactttaaatcaaattgcctattttccttctaccttttttaa
aagtt

>chr9_120531179_120531379
gcatcactaatggattatagtacttttccactgaactaggaagtagcctcagaatcactctctcc
tcactgctcccagcagctgctatcaatcaattgcagctttgccactgtaggagctcttaaatttc
attgtttagctctgacattttcagaggatataaatgcaatgaatttgcctaactcctgaaattca
ttttc

>chr10_70860594_70860794
tgggatggcaccttgggcaggactctgttttgcccatggcctcagctcccaccagatgcctctag
tccctgggctcaggaaatagacaacctccttccactatcgctgtagcccaaggagggaagtattt
ttttctttctttctttctttttttttttttttttacagagtctcactcttgttgcccaggctgga
gtgca

>chr16_8858099_8858299
gctacagaaatctttctctctgagcttattcttccatgcagagtattttaaatttttcttttaaa
ggacagaagggattctttgtgtgcaggacaaaaggagctggggaaatgaatatgtgtcgcatgat
aggagccttgcatatgtcattcaatcctgcccccacttctcggttttagttactacaaacgatta
aagcc

>chr20_50013993_50014193
aggcctgagccgctggcttgccaaagtccccaagcccaagacacacattaaaaaggcatcgccct
tcgacaagttccctggggggcttacaacctcagcactgatattttacttgcattttttatcctcgg
atgcaaagtcctgaggacactcctgagggactccccaggagccgattcctcagatgcaaaaagtc
ctgag

>chr22_20231000_20231200
attgcaggagtccccggggggtcccagtgggggttccacccacagggcctaggaaacactgcaat
ggggctggggtccctctgggcacgtctatctcaaaaacgggcgcctggatgcccgagggcggggg
tggagggcaagtgtgtccagggcttaggaggagtcgcgtcccacaaccacccaggcagcctgctc
cctcc

>chr22_49928596_49928796
ctgaagcaccaatttaattcctttcttcctcttttttaatttggtctctccccccagccatgag
gtacccacgcaagcaggagcagtgatttacctgccagcctttcgctggtgcgaagaacatttcat
tttgctaacgatatttgctgtttgatagagctcctcaaggtaaacttctcaaaacaccaagtcct
tataa

>chr1_230653977_230654177
tcctcaccatcaaaactcttttgacaaattgaatcaatgaatagaatcttttctcttttcttttt
tcgcccatgctggcctcaaacatttgggctcaagagatcctcctactgaagcctcccatggagct
ggggctgtaggtacaagccactgcacctggcttcaactaatagaatctttatcctaatttcaggt
ccctt

>chr2_233134356_233134556
taaaagacttgagggtctagccaggctgcaaaggcaagcagactgctggtacaagcatgggaggg
atgagaaatggcagattgttccagggtgtgtggtgtagggtgcagagagagagaggagctagagg
agacacaggctaggccttggagtctgtaggaatgaaccagggcagctatggaatgattttaaata
tgtaa

>chr3_58023560_58023760
catgatcttaaatgaaaaccacgatcacttgccagaaagaacaggtaactaggctttgaaaaaat
aagttagaggagatagcataagaaaaaattaaaaaataaataaaatcaatgaaaacaacgtgtta
ctaaattcttgaaaagtttttttgaagactttgagcctgaggcctgttcttattgtttgtttgttt
gtttg

>chr5_54923443_54923643
ggaaataaagtggaagatgatgagagaaagaaaaagaaaatgatgcctgaggctaagggataatg
gagcttctagagtgagttctccaccagaacaaacagaaagggaagataatgagcatcttaactga
taccaaggggctgcatttgaatattactagttttaaatactcataggaatttcatacaattaaat
taaaa

>chr6_125754101_125754301
ctgagcaaagaagaattcgagaatccagcagctccctccacccccgacatccccaccaccgactc
cttgtgaaccagaatagattctgagaaactctgcactgccacgggtcagagaagatttatgggca
gaaaaaggaaagtgatgtacagaaaacagaagtgaggtacagaaacagttggattgttggattgg
ttaca

>chr6_155470708_155470908
atgtctgaatgtttaaccttgtttggaagtttttttcatctgtgtatatatgaagaattcagcatg
aggctgggcatggtggctgacgcctataatcccggcgctttgggaggccgaggtgggcagatcac
ctgaggtcaggagtttgagaccagcctggccaacgtggtgaaaccccatctctactaaaaataca
aaaat

>chr7_28428675_28428875
acagccatctctttaggattcatacaggcatagtttatcaacagcgtcagagctttctcacatgc
aaaagcctcacgttcattatttactgaatattggggaaataatttgaaaaatctgcacattagtt
atgtatgcattctgaaaacagtgaacactatttgcatcttaaaaagatctgagtaatgctgtata
atatt

>chr7_90954864_90955064
gttagatactttaattgactttcagtggcagtaataagtaactggagtcctggcttgtagaattt
cctttctaattgtcagtgttaaactactcattatagggataaatagcataccgctttatttaaac
aatttccttcatacatgtaaattccagcggaagttcccagtattttcaggcccttgggagagctg
tttgg

>chr11_41479624_41479824
tttgacccaggcaacgtagcctctgaataaatctattatgttacaatttgcatcttctttctata
catagacatcaacagctggagagaatcacttaggtatgctcttctctacctagttaacacacctg
tcatctcttccatgaacgtgtccacagtaacagttcaagttggctaacagtccatgaactaacac
tattc

>chr11_76780752_76780952
acccgttctgtctctgtccccatctattcaagtgttttgagtgccttcagagcccctcccaggca
cctctcaagtgccctcagagaactcttctgcctctccatccctggtccccgctcaaaactttcct

gggctcaccgtggtcctgaggcttggccttcctgctgcccacctctgagccctcctccaggctgg
tggag

>chr11_131788190_131788390
caatgcatttagctgacatttatcaccgtattctctcctgactgctggtttagtccgtaccacac
cttgcgttcttatgctttgactctcattccatgtgggacagctgtttgctgtgggtcaggtcctg
tgctgagcagtttaccagaagaacctcatttaatcctcacagatactccatcaggtggacacaat
gatta

>chr12_120885617_120885817
gcatgatctcagctcattgtaatgtccacctcccagactcaagagagcctcccatctcagcctcc
caagtacaggaccacaggcgcgtgccacttggcctggctaatttttttgtatttttgatagagacg
ggattttgccatgttccccaggcttgtctggactcctgagcaaaggtgatccacccatctcagcc
tccta

>chr13_44995200_44995400
aaatgtgatgtccaagaagcactcacctgctcacccgctcaatggagggccactttccttctcaa
agtgctcagacacaatcctcctagcccacccgctaggcctggcgtccccactctctctgctgaga
tggcctcttccactcagtcccccacatctttccacactcagttcaggtggcttgtttttgttttt
gagac

>chr14_77688047_77688247
caacctcctcaaatgtgagctcccgtccctcactccacccagacaaccctgaaggtcagcagtgg
ttcccccccaccgctaaatccaatggacttttctcagttcacatcttgcctgcgttctctgaaacg
tctgaccctgtagaccgtctttctctcttgcacaggtgctctccgttgcataggtgatgtcacat
tcttt

>chr15_80681745_80681945
cctggaatagtgcctaacacttagtaagcattcaataagtgatagctgtcagcaatactactcta
acttgttgtgatggctctgtatatgtaaattattgaataattagggtaataaattatacccataa
attatacccaggctttagttaaacaggtatttcaggaatgtgtgcctgtttggtctcaaacaatg
ggaaa

>chr16_55424899_55425099
aatcggagctgtctacaggtgggggccaattaccatctgaataatcacagtgccacacaagaata
gcatagccgctgagcgtgacatatttttatctctatgcatttcaatgaagtcaggctagtacata
aaaggttatcacctaggaaacatattttcctaagcacaagttaaacatgcaagtgagatcagcaa
agata

>chr17_48664001_48664201
tcccaggaggtagtgttaccctgtttaacagaagaggccactggggatctgagaggcaaagggaa
ttgcccaaggtcacataggaagaaagtctcagagttggtggcacctttctaacaccagtgtttgg
cacacagaaggcacttttagggctagttgatggagcagctatgaggcttggggagccccggtgct
gggaa

>chr17_55994801_55995001
ttggagagtctcaaccaggccttcactccacggagtctcttcctgactataattcttacagggaa
caaaaaccaaggataacaataatacttactgagcatgccatgtgggcctgacactaacacttgtc
tcattgatcctctcaaccatcccatgagagagaggcctttcttctccccattgactgatgtggag
acagg

>chr18_22761202_22761402
gttatgaaacatatttttctagtcctcccaggagcaaggccaggttcacaatttgacctcagaaa
catagcagtcctgcaatgccagacagcctgatagaactcagtattagtgctctctgcctggccca
cacaaaccataacatgcagcatggagcaatgttacttacgggaaatgacaagcaatggatcagtg
gggga

>chr20_24898800_24899000
cgagattctccacgccagaggcccggtgggtgggtgaggggcctgccaagcctgggagatttatt
tgcaggattttccccacggactcataactgaaatcgcagaaggccttgcggtttagaggccctgt
ctcatgccccaaattgcattttcccggaaagggctcagggcgactgaggtccccaccgccgctgc
ggagc

>chr20_56046194_56046394
cgtcttgtttccatttcccctaatctccagggcaggcacaattgtgctggccaagtgcccagtgg
gggtttggagtgggggtcgtttagcttctcctgccctggaaactaaactgcagtgggttgggagc
cggtaacaaggatttacgtttcggggttgccctcaccatttcctttaacacatgagtccaaggtg
gccgc

>chrx_133048134_133048334
ataaaaggcatttcaatggtgaggaatgtttgaggactattaagatgtgacaggattcactaggg
taaacatgtcttgcctcaggcttctgggagtctctaatccctataacaagcaaggtcctaaataa
gagaaataataatactagctactatttattgagcaagcctgtatcatgtgccctttacatgaatt
gtctc

>chr1_16443213_16443413
taccgtttcttccttcagatctcagcttggataccgcctcctccaggaagcctttctgactctcc
acaggctgagcacgttgttcagttcctgggtcccacagcccccacgcctgtcatatcacatttat
cttttgaatattctctccagcgactaggagttctgtgacggtgcggcctgggatctcccacccct
tgctg

>chr2_43941096_43941296
aaatatactatgtactatgtttttttctaaaatacgactgtgaaaatgttttcttagttactgatg
ggttagggaacaaaatatttgcacaatacttttccagtgaacacactgctactttaaatcacatt
cttactataattcataataatgagctatgctcttgtttcaaggacctcatatgcatatcaaaccc
tgaga

>chr2_159370554_159370754
tttgttcctgctagacagctctttcagcatcataatggccacaaaacctaacaccatctggtgta
taaatgtgttttcattgaaaacacagatatgttgtcatatcttccctcagtttgtttcatgctcc
cattgaacaaacacttttgaatacctactgtgtttcaggcactttctggatctgctgagaatgca
gtggc

>chr2_232316956_232317156
tcagaaactagagacaggtactagggaagactgggaatttgacttcagtccctaatcgcaagcct
caatgtctctttctggccctgcagggtaacccaccttacatctttttttttttttttttttttga
gacagtgttgtcgcccaggctggagtgcgatggcgcgatctcggctcactgcagcctctgcctcc
tgggt

>chr3_33467396_33467596
aactatgaatgctatgtggttcatctaaaggatggcattaacaacccctatctcggggtgggaag
tgggggagggcggcagggcagctctgaatatatatatatatatatatatatatatatatatatat
atatagcactttaaaaacttcttttaaaactttaaggtccagattctttctataaaaaaaaagca
agtca

>chr3_51129360_51129560
ggggagccatgaggtactgagcctgacgaactgtgcactctggcccagatagtgcgcttgtccca
tggtctttgcaacccgcagaccaggagattccctctggtgcctaacacatcagagacctgggttt
caagcacaaaactgggtagctgtttgagcaggcaccaaactagctgcaggagtttgtttttctcc
ataac

>chr4_154191750_154191950
tctacagttacctttgagagaaaactaatctcttgtgcttctgccagacattgaaaaacagaatc
atgagttgcaaataatttactttatttcaaaccattataagcaagagaaagcaatccagtaacta
ctgattcgtttacacaagagatggagcattaaataagaatgttaagttgaaaaatcttttaaaac
ttccc

>chr5_142658407_142658607
catcccatgctaattatccagcacttcatagacacaaatcatgttagttttccttttggggtggc
caaggtttcctcccatagttttaggcatttggatatatataccccaaaagtgttatgtcctaagt
gcctcagttctgcttgtatagtatcccacagaatacctacaaacactaaaaatacttttcaggat
ttgtt

>chr6_65970079_65970279
atctcgtgagaactcactcactatcagaacagcatgggggaaactgtctttatgatgcaattacc
tccacctggtctctcccttgacatgtgggaattataagaattataattcaagatgagatttgtgt

ggggagacaaagcttaaccatatcactcaatttcaaagacctgcctgcagatgcctgctctactt
tacca

>chr7_153869067_153869267
agatgtacacactgggatgcagagaggtgatctgacacaaggacagcccatggtcaagcccttt
gacttctgtctgctgttgaaaaggatgtgcctccctttcactgagcctcattgcaatacttgtat
agattctgtgcccctccctcccttccttccttcttccctgcctgcctgcctgccacaggtgttta
atgaa

>chr8_6945190_6945390
cccaggctagtcttgaacttctgtgtgcacccacctcagcctcctgcatttttaggaggcccctc
ttgtagggattttgatccagaggactgggtgcctcatgtctcctcccatctctctcttctttctg
tctctgtcctctctctctttctctttgccttatagctgccctgggaactagactctgccttaggc
atccc

>chr9_138189579_138189779
tgaggagagcaacactctccgtctctagcccctggactagaacctggatctgctgacaccattgg
ggcctgtcagacacctgccaatcctgacgttggatgagaaaaggctgctaagaccacggggtccc
accggtggagacagccatgcccacaggtgggaaggggcttgccaaggctaccaagcagatagcaa
cggcc

>chr10_107812810_107813010
aagccacttggtatcacttaggtgactgtagcactgattcctatgccattttgattagtaatgca
tattgatctccaggaccaggctgaacacggatcgaatgacatatgcacaatagcacaggactgcg
ctcatttagcaacaaatcgcctttggaaagtccctttatgtttattctcctcattttggttccat
ttgat

>chr11_120481590_120481790
ttgattggaaactctctgaccaggttctgtattggtgctcaggccttttacatgagaaccacaga
gtctcagaatgtcagcgcaggaagagccttgaaagaacgtccggcccatgtcctgtcgttttcaa
caggaaaccaaggcccagtgagcggacaagactttgatttggattgacagctcgaggcagaacca
ggaga

>chr15_83254945_83255145
aatgttaatctaatttgattggaaggttgaagcatttttttcagatacaaaaaaaaaaaaaagcc
actagctatcaattttcagtttatatcctttgccctctttaagaatgagttcttatactgtggct
tttcctttacatcaataagccagtacttattgtcctctagaaagagaaataccctaaaagaaaga
atact

>chr16_31646899_31647099
gcaccactcccccagcctcgctgccaccttgcagtttgatctcagactgctatgctagcaatcag
cgagactcagtgggcgtaggaccctctgagccaggtgtgggatataatctcctggtgcgccgttt
tttaagcccatcggaaaagcgcagtattggggtgggagtgacctgattttccaggtgctgtctgt
caccc

>chr17_34970487_34970687
aataaatgtgagcttttcaatgcgatgatgacgatgacaatgatgacgatgatgatgaaaagagg
cttttgaagctaaaaaaaaatgcccaagaacgcctcctcctcccctcaccctgctctctgggggg
aagggcaagtcccagctcattactggtgggggctgcgtagggggaggtgtcagattggaatcact
cctgg

>chr18_67889020_67889220
gaaagcaggtgctttgtagacaactggtcttaatgagcacacatcaataggtttggctgaaaacc
ttgtaataccaaagagcagaatacttaaatcaaatatgaaagtaccatagttgttcagtaaatta
aggcacactgcccggaaactgtgcacttttctgttgtcttaaaatgttgtgccaagtggcacaat
gctca

>chr19_37283160_37283360
gaggagtccttgagaagagcctctccagccaggaccaggtaagggaatctgtgcacttggccaga
cccagaacacacacagtggcagggacctgacagccacactcttaccccatagaatctccaccactga
cacacagatcaggatgcgtcaccatgagagatgacaccgcaaatctggctctcacggattgattc
catac

>chr20_53883793_53883993
gcagagaaagtctcctgggaagagtgtagccatgagctatgagcaccccacaggcctggaaattg
gaagttgatgcaccattctgatgcaggacaggcaacccctgggcttagcccaggaaagttcttag
cttcacccaggaaagaatgcaagggtgagcaggtggtgttagacagcaacttttattgatgcagc
agtgc

>chr1_149657176_149657376
ttgtaaaatagtatgataatgtaaagaacaagtttcaggccaaatttcttgttctcccaagaaaa
ttgtttctttttctttgagccatctcatttgaatttgctccaattgccttaaagacaccctagtg
gggagtcctttgggatgctcgcctttgtccccatgtctaacaaggatctctactacacccagaag
ttttt

>chr1_208271977_208272177
cagatcaatgggtaatgaatttgcagggaaactggagaatgactatgccctttacaataggcatt
gccatacgtttgctataaacataaagtctccttactggatttaaagtctgatcaatttacagata
cttgtgcaagcgtgaatggagcacacccacacaggtgcatgcactgtattccctctccccatgcc
cctgg

>chr2_168244154_168244354
acttgcatatttcattttaaatacacctatttactagtgaaatcatagaatgtgcttatttacgt
gcatttaatttaggttaaataactaagccatacaatactgtggtcatattttgtttggaaaagga
aaaaaaaatagttagtgttaatctctactgccagtcctcccacaaaaggctaaaaagttgcctta
ctttg

>chr2_237310861_237311061
tgatcaagtcacctcctggctcaagtgtccccaggggcttcccacagccccctgagaccacgggt
taccagctcactcagaggacacacctcctgcccctgctgactctcatctgtggtctattcccctg
tgaatccaccacgtctttttttcagctttgcatttcagctttgcatttttcagctttgcattttgc
tttga

>chr3_123052310_123052510
cagcaagagtgctgggggctccacgcggctgctgggcaggggacaaaggccctggcactggaggg
ccgaggacgctgcctctttctgctgctatcctccttctgtgctctaacggcaccctcccccagcc
caggaggaaggcaggggaggggcgtgaggcctgggtgtgtgcctcaggagggtgcgggacagggg
gccag

>chr4_55383843_55384043
tgagagcaacacattgttcactgaaaatctaccagtttagcttccctgccttccttgccacagag
agaaagaagggagggaaaaaagaatgatttgcaaagcagcattaatattattctgaggccaggtg
aattgggatgtgggtgagatcttgagatgaaagttcagctgcaagaaaatgaaaaagcagccata
atctc

>chr4_88855376_88855576
ttttctaggatatccacttggtataaaataaaatatttcctgtcatatgcaaaacaactttcatt
tcattcttgagtggtgcttacttctgatcacaattgtgtataacagagatgtccaatcttttagc
ttccctgggccacactgaaagaagagttgtcttgggccacacataaaacacactaacactaatga
tagct

>chr5_126629501_126629701
ccaggtgaacctggtaaagctgacgtcagatcacatttggagaaacactggcttacagcataaag
tctcacctccttaccgctatacacaagaccacctttgcagcacttctcagggttattttctgctt
ctctggtccccagctctccagctcccactgatcttgatcttgttactatctcaagaatacgctgt
gcact

>chr5_150053207_150053407
gattattatttacagctgagatgaagtttggacaggggagagcttgaaagggatacatttaccat
tctgcactgagggcctgaaaacaaaccataggaatcaagacaggagcttggcactgggggaaggc
cttgggctgaggagtgcatgttaactgcattggcttccttggtctggtgctatttttgaatatgc
tcctc

>chr6_160554210_160554410
gcctgtgacagcctctgctgctaggcaccgtcccacacatggtttggggggtctgacccggggcc
acatccacgctccaacaccacccgcagcccctccacccacgactcccctgacttccttttcccct

tttcatttttgttcccgttacacgtatcacgctctaacataggattgcattctcttatctgtgat
gtgga

>chr7_2505474_2505674
ttatctgtgctgattttcacttcttgttctgaccaggagacgcctggctcccatgatgcacaac
ttacttatttgttcaatcccagtgttcgttgtttcagaattgctaagatgtacccctgggagaac
caaacctactaactagagtccagtgtttgtacacagttggttttgtcctgagcttcaggatgtag
acaaa

>chr7_23148475_23148675
gtagcgctacttcatccttgtggtatatagtatcccattgaatgaatatagcacaatttatccat
tctatcattatgaacatttgggttatttctagtttggaactattaagactagtactactttgaac
atttttgtacactttgtacatgccttttggtatgcatatgtatgtattaatatttgtgttgggaa
atacc

>chr7_51424706_51424906
ttgcataaatcaacctcatctcttcccgcccccaggcatactaaatcacactgtgatggcgagtc
ttctcccttttgccgcctgtgctaactcaggtggaacctgccccttaatgtgccacactgggtta
ctcattccctgggcatggtgacctctgggttggaaactttctttggaaatgtgctgcccttcgta
cgctc

>chr8_90163684_90163884
gttagctggttgctcagctcaggcatttttttatgtggccattcttagtcttcataacccatagc
atctccctaagtcccttgatgaactccaactttctccctcaaacactcttatcaaaatatagctg
ttatttattgttttggtccttttctgtggggaaataattgtcaggacccctggtcagtcaccttt
ctgat

>chr9_126138579_126138779
atgtagaggctttgatggggaatcctgggagcctgtgactggagacaccagggaggtttgctggg
ctccccgaagcctgggccgaggctcgcagggacaagctgtcataacagcccctcggccccccctgc
caccctgcccccgcaggtgggccccagtggattctgaccttgctattgtggcagggggccgctca
gcctg

>chr9_139033979_139034179
ctacagcccagggcccgccctccctgcagaccggacagcaacgccacctctgggagactcgtggg
gacggggcccagggcaggctcacagtgggaggctggcagcacctacccggggcagggcctcctcc
tgccactaccacgtcaggcatcactgagaaggaggcaaggcctagagcagtcggcaccctctgca
gagct

>chr11_66307424_66307624
agcccgtgctgggtgacaagaacgcactggtaaactgggcagacgcagcccccctacttgggatac
ggcttcattcctgcatcacagacttggtgagcacctcctcagccctggaggcggtgccaggcact
agagggtgtctctgcccttggggagctcacagacattaaagaggccattcttggctggatgcggt
ggctc

>chr13_114271399_114271599
aacttggactagcacccgtgtgttctcaggagcgggtcctgctggaggcccagccacgtcaggca
cagtaggtgtgtgatttgtgcaacggaggcctctgataactgccagagtagatagcttttgtgtg
ggagctgcctcagcctgcattccaggagctcaggtttcctgagtctgaatggagtctggagagag
caatg

>chr14_76876047_76876247
agggccccccctaatttggtttgcaaatacattagccaaacaagctgagggggctcctttggggag
agggtttttgggggatggggatgggagatggtgggaaggctgaggaaatgaagaagggaagggtt
ttctgtgcaggtcctggagtataaaccgtacatgcgattgccaaggctgtgcatgtctcatggtt
ttacc

>chr15_86775396_86775596
atgttatgtcttttgcataagtccatagaagagcttaaaaggtttaagctaaagggatactacct
gtcctcgtcgcccccatccctgtccccttgggacggtcagacccagtaggttgggcgggccttgg
gctgagccgcagacagtaaagcatatcgttcctcttccggccatgtggtggtgctgttcttttag
ttatc

>chr15_97133396_97133596
cacaattacctttttaaaatgccaattaacccattcagtgctgtttggcatactgtgagcagtca
gtgacagcaatattgcacactgtagtcctacagtaaggcatacacatacagaagcacacacatgt
gcacatgcacacacatgattaggactggagtgagggagggagggagaaaggaagggaggtagggg
aggag

>chr15_99143477_99143677
gcccctgtatttcttgagctggagagaattgggcatacaggtatgtttacaaagactgagctcgc
ggaagaggttttaaaagaccaaaaaaaagtctcaatgggaaaagataccttctgccctgcctccg
ccacctcctgctcctcctcctctctctctcagctcatgcgcatgtacacacacacatacacacac
acaca

>chr17_76671205_76671405
aaatgaaacaggttatccggaacctctgtcccgataaccttcctcacagttttggttattaggta
tttagctcaaggaaaatgctgactttctactttaaagcccaacaagaacttggattctgaaggag
gaaaataacgtaagcgtccagtcagctctctcctctcctcggcgctccgcaggcacttgggatgg
aaaag

>chr18_55115202_55115402
gccacacagtggagtcatatggcactggccagggtgagaactgacagtaggcactgacccctgac
tctggcctttttcaatcagttgctttgctttatttctggttttattccagagaaaagagatagga
gagtccttttggtcaatgattgagcacaactcacatctctttgggtttcgagtaaaatggtcctt
gactt

>chrx_1474800_1475000
ccagtccttggaaaagctgaacgcctggtacttcagagaacaaaagggtggttctgaaggctgct
cccaggacagtggggctccgagggtgcaaccccaaggctcactcctcccagtgcccccaacgcgg
ctcagtcctgtgtctctgcctgtagagtttctttgttccctcttccttcctggtgtttttctctc
ccgct

>chrx_2161000_2161200
ggacccccttttcctgtagcatcttcaggtgcagccgtgaatcagagaagcagaaggggcttacct
tgaaaagcaaccagccgagaagcttcttcgccagacgggtggcccagaacacgtgcttgtagacg
tccgtgttgaccaccccggggtccaccacgttggcggtcacgtggcttccctcagccgccagcag
ccgct

>chr1_7945413_7945613
cccatgggtctgggaggtggacaatggcggcccaaacggaggtttcatgccagcctcagggagta
tgggatacttcctcccaggaaggacaggagatggccccggtataggccggccttaagacaggaca
aggcagggaaccgcgactcgggatccttaaggttacctccagcacaacccacccagcctccactc
ctgct

>chr2_205395555_205395755
cttaatgaagggttcttcagctaagagtgcttgaaacatattcttcccccaccgaggagagcatc
agccagcgagatgatgaagtcttctggtgaggacacctcctagggactagacaggctaacttctt
ggtcccatcctcttttccctcactctaccactcagagatttctgttaaccctttcttctcttagc
tttgc

>chr2_233470556_233470756
cgtaggttgcagtgaaccgagattgcgccactgcactccagcctggccgacagagcgagactcca
tatcaaaaaaaaaaaaaaaaaaaatcggttctgagcaaatttcgagtcactgacaaggcggcggt
atccagcagagggcgcgccaggcgccagcaccaggcccgaggacgcctggctcatccccaggccg
gcttc

>chr2_236923461_236923661
atggaaactaaaccgtgttggtagcctgcgaaatatatacagcagcagcaccaacactgaaggta
acgcctccgcacctccacccgcccacacctccacctgcctcttcccctctcatcccctgcccgcc
ccttcctttccaacacaggcatggaaatgcaatttgcccaaagccgcatgctcccttattgtgtt
tgcag

>chr3_107384710_107384910
gtcgttgagtctgtctgtatctggtgctgaaggcaagttgtttcttcctttctattgtggttcct
ctgcttgggacacctcaactccaggtgaatggtgagtgacagcctgacttctttccttgcacctt

catgtagtctttccattaactattgcagggaattgctaaaccactttcaaggagttatttgactc
cagat

>chr4_4398499_4398699
cagtaagcccctggtgtgtttcaagatgcatttcttaaatatttatgaagagtgatgccgtttcc
ttggcattcagcagtgggggtgggggttcttcccgtgggggtggtattggagaccctgaaggtgt
gtgtggacgccccctcacctttagctatgcgagcatcttgtaggatcctggggctgtggttgcag
agccc

>chr4_109838151_109838351
aaggaaaagtttaatggtaatatcaaatactgtctaagggctttacatctatttactcccttaac
tctcattgactctattaggtaggcactgccattgtctccctttacagatgagaaactgaggccag
agaattccaaggactttgccaaaattcacacagcctctgagtggaagactcatgctcttagcaac
ttact

>chr5_57246643_57246843
ttggatgcaattgtggcagaaagaaacaatgagtaacaactgtaactcaatgccagctgcagtgg
ctgaaattcatcctattaacctctcacttgtttttccttgagaaggaaggcccccaggaatcctg
gaggagctgttgcctgaatgtgtatgaagaaagaaacctgtgtggcacaaagggtgagctgtgat
agcaa

>chr7_51945506_51945706
atagttatgcaaatatgctgttttcatacacgcgtatacaaagccttggtaatttaggactcatg
atgagtgtgcaggctgcaggctctacttgagttttagagatgtactattcatctgcttttacaga
gtcaaaggaggagcagatcacagacgtgaagcccatctcatcactgagtaaagatgaagaccatg
ccagg

>chr7_107577164_107577364
cattcttgaatgtgaaaggatttgcagaagtattaataccttaactattcaaaagaaccccccttc
caagaaaaggccaggggattaaactactaactggagggaaaaaaaaaaaatcaggaaatggatac
cattctgatacaactactgccattcactctgtgggacctttgctatgtctcttaggttctctgtg
cccaa

>chr9_138949179_138949379
cagggatggctcagaggaagagaatgactcgaagggtggggagggacagctggggctggggtggc
tcctgttgagccccagatccgccctatggccccaccccagccagcagacccactgggtccacaca
ctgtgggatgctccctcacaccacaaaacattctagaaggtttccaaagaattaagctgtgtttc
gaaaa

>chr10_49525194_49525394
ttgaagatggttaaaaaacaatgaatgattttcttttaaagacagggtctccctctgttgccc
aggctggagtgcagtggtgtgatcgtagatcactgcagccttaaactcctgggttcaagtgaatc
ttcctgtctcagcctccagcctcccgagtagctgggactcggctggcgaattttaagtttttg
taaag

>chr12_19119933_19120133
tgccccaaggccccattttatagagacagactgcaggcccaggctaaatgagagattctaaaact
cacagggtagagccagcatactgcatttggtatccaggtaacaggtaacaatggctaacaatgtg
atcctgagaaagggacaaaagcctcctccccatttctaaccttgctgcttggagacctcagggtg
ggtac

>chr14_73447447_73447647
ttgtattttagtagagacagggtttcaccatagtctcgatctgctgacctcgtgatccacctgc
ctcggcctccccaagtgctgggattacaggcgtaagccaccgtgcccggccgagaatttattaaa
tttattaaaatgcaaactcccaggccctatctcagacctactgaattagaaactgtggaggggc
accca

>chr15_51115508_51115708
cctggatggggccagtttatgggtgtctccactgacagatttgagcagtttgaatttgatagggga
atggagattggatgactttccagagtaagcaagtgatattcgaaaaagcatgtttcagaataata
taagtagaaacctaaacattctcttatgatctttgcaattactgccagctctttcttatctacat
caagg

>chr15_101587477_101587677
ccatcgtgacagtagcagcctgtgctagcggggagggagatgggttaatagcgccagaagggtgc
actgattgaaggagtgggcgcaggcgctggtgctcctacgttcccattcctctgccctccctttg
aactctgtgtgccgctgtcttctcaagggagggtcatggagcactagcatcgtgggaggctgagc
tgaca

>chr16_7268999_7269199
tggtcacaagagaagaagaggtgagaagggctcggagccagacaaagcacgcctttgtaaatgaa
tgtggagttggccaccagcagtcttcattatccccgtcattgcaggcagctgagctggctttgtg
tcagccctgctcggagtagccttgttaattcacccccatgaaaatggaagtttcatacacattga
cataa

>chr17_73840805_73841005
gaagctgacctgggcccggggggggttctcaccccacttcctcgagtgcgtccagacgtggggcgg
ggcggggtggggtggggtgtggtggcgcccagggtgggggctcccaagaggcgtctccctggagg
acagccagttgctcagcctgcctggccccacaggtgacattccttggagatgacggggccaagct
gtgct

>chr21_34178530_34178730
ttctgtactgagtacagctcagctctagcacagtgaggaatgagggacagatggcactatttgac
cctttctcaagaacagactggggctaccccctggattttgctctctggtcatcaagcaacagggag
ccccatggccaaggtaagggacagtcagcatccagtgggggctggggacagaagcagatgaaagg
accat

>chrx_137369334_137369534
aattttgtggagagtaaatgagaagattcatgaatttagcactacacctggatcatagcagtaag
ggtttcaataaatgttagctccctctctctttcctgctccctctcccaggcatccatacagcctc
accacctttcaggcagccctgttaaaattgctggagcaaatatttcatttcaggctgtcttccct
cagca

>chr8_33343858_33344058
ccaggagttcgattccagcctaggcaataaagtgagactccatctctacaaaaaataaaaaatta
gctgggcgcggtggtgttcgcctgaggtcccagttacttcgaaggctgaggcagaaggactactt
gagccgaaaagtccgaggctgcagtgagctatgatggcaccactgcactccagcctgggtgacag
agaga

>chr9_133872379_133872579
cctccccgcttggcacttgcagttgcttcttcagttgtacccttcccacttcccctccggagacc
ctctccaacgccactcctctccttcccgaatcagctctctctgccggtgctaaggacagctccct
gaacccctgaaggaaatattcaggggtccctgctcccagagcaatcccagacacaggtgatcctg
gggta

>chr1_29609813_29610013
cccatagtcccttctcccatagcccctcccccatggtcccacccctcccatagccagccctcctg
accccaggctccttcccacagagcactaccccttcctgagccttctttctcaggctcctccttt
ctatccaggtccccagtctagtctctgccccttcctgaggccctgccttctatcttagatgcttc
tcttc

>chr4_120589152_120589352
aagcagatgagctggaggttaaagtggcagcgtgctgtaaccaagcctccaaatcaagcccttgg
agccgctataagcagagctagctgtgcaaagctgactccacatatgattgccctgccagccttgt
gaaggagacgaggcctgcctggccaaatcagacaccacagagtgcagttcaaggagaaagatgag
ccctt

>chr9_75829180_75829380
atagttcaagatctccgacactgtgtcatttcatagagctcacacactctgaccagaaggaaaga
tgggttccgggaagctgaggctactgaagattatctgaattctggttgaaaggccaaaagaaaat
gtgtattatggaaaagtgatgctcttaaagcaggaagtggtctctgggccatttacttgttgtgt
gactt

>chr7_54256106_54256306
aagaatttgaagtaagggatgatgattcctgttatacgtaatttatcctggctacaatgtacaag
gtaaagaggtcaagcaagatgcaggcagagaaaacaattaggagaatgcaataacagtcccaatg

atagatgaggcttacttgaacataaacagcactggtgaggaggaggcctcggaggaaaatatcta
agtac

>chr2_50449496_50449696
aatcattctatggagctcagtttcctaagaaataatgagaccaatttgcttttatatgaaaacac
tccagtttgtgatatcttcatacactgctggtgggagtgttcaacagtacaatgttcttagagga
caactcagcaatatatacattggttcggcttgtatgaatttgtacataaaaatatgcttttacac
atgtg

>chr10_43746194_43746394
aaagcacatgaggggcattgtggctccgctgttttcccacctgtcacctggtcaatggcggcttc
ccagctggaaggggcagagagggctggaggcttgggagtggtggtcctggggaacaaggctcaat
ggacccaggcactgacccgcaaccctgctgaagccagagacacctgggtggaggtggagacgtgc
aggca

>chr4_65714605_65714805
tggtgtgtggtggggaggcggggcggatggtttgggtttggggatgaaactgctcttcctcagat
tatcaggcattagattctcacaaggagcacacaacctagataacttgcatgcgcaattcacaata
aggttcatgctcctatgacaatctaatgtcactgctgatttgacaggaggtggagctcaggtggt
aatgt

>chr13_111894199_111894399
tttaacattgaaaaggacttttctgatacgtctgtgacagatcttatttcatcttatttagctca
agatctcattatgcttggctggcttcccctgttgttctggaggacaggcctggtttcgagtagcg
tctctggggtgtggtggctggcatcacggtttacctctgctcaccagacctttgacatttgtgtt
cgacg

>chr17_47953401_47953601
gctctcaggagtgtgcacttccacccctgatacagccagggatgagctgtgggctggggcagaca
gagcccacagatcaaagcactggcctcctaggtgagacgggacctcagctgtaggcaaagcggga
tgtttcccagttagaattcacagatgagcaatctgatccctcaccttttccctcccttttccctt
tattc

>chr1_42220013_42220213
acaagagcacaggagacaaggatcagattgcaatgctgttgggaggctatcacaacaatagtctt
tgcatgctagagatagcagcaagtgagccaaggtaatggcaatggaggtgagaagtggtcaactt
ttgagtacaatttgaaaatacgtttgggcttttctgcagagcgatgcctgcctgccgtacgtgtg
gctgc

>chr10_34151194_34151394
cttacggccgatatgctaaccttgaatatctaacagacttttaagaaattcttaggtttatgcag
actatcatagccccataacacataacacagccccataggccaacacgtcacctacattctgctca
tcaaataacactgatggagctatcggtcatgctggagccagcgtggactcctgctggtttccata
tcggc

>chr9_99018379_99018579
attagtgacaaaggccccactttattgaaatctgtctctgtggtttcagtcaaatgattcatact
gttattttctttaattgtggtaaaatacacataacaaaatctaccaccttaacaaaaatgtggaa
tactttacaaatttctgtgtcatccttgggaactcatgctaatgttctctgtatggttccaattt
tagta

>chr11_27761624_27761824
tatacaagatcatacaaccagtaggcagcagacctggtgttcaaagtcagggctttctaattccc
agatctgtttgcatgaccactcattcactagccatattgtgagcccagagagatgcattttgaga
aaaaaaaaatgccatgtcctctcccttactttatttctatatttctaacaccagtttaaatggag
gtctc

>chr11_73209552_73209752
ggaggggcgaccagcactgggttgcagtggcccactgtctaagccgtttgagctccttggggagg
ggcagcaaccagcattggaactcgcaactgcctgacatgctaagctccctgggcagggtaagggc
agcacccatttctatagctccaggctgtgtttttcccctgctggagcccgggaggctgaatggct
tggtc

>chrx_152532406_152532606
aggtgaattgagtgaaaatcagtgagggccccttctcgatgacagaagcgaaaaacacaggctcc
ttcaccagggcgggccattgtgttcctattgtctaccaaacggctctgctctccaggtcggagag
cggtgcaattgttcactaattgcatcctctactgatgcatgcaattagtgaacagaataaggggа
aaact

>chr7_17961275_17961475
acctggggaagtaggggggattttaaaatcccactacccagcttacgccacatacaaattaaatca
aaatagctaggtatgtgagccatgcatcagaatttccaatgttcaggaaagctgatataagagca
ttgttagtttgtgaagttccccagatgattgcaatatgcagttacttttgagaaccaccgatgta
tgcac

>chr20_24474800_24475000
gcatggccgaggactggatcactgagtgcagattgagaagcacagcagggcagtatgtgtgggac
ttgtggtgtaagggaagatagtgaaggatggaggattgagcgcacagtgtcagggaagctgctgg
gggcatgagcagggaggaatgctgagaagagtggccttagaaccacacagctagaggagctggcg
ggtcc

>chr1_204803577_204803777
tcatcccctcccggctcctgctgctctcttcctccaggaagcagctgtcttagcatctgccttct
cctccttcacctgctcctcctcactcctcccctcccgggctggcccagcccctcctgaaaggcct
ccctggcagcccctctccccgttattgtgatgctaatcatgttgttatttttttttgagatccag
aaaga

>chr4_189353606_189353806
gtgagaatatcacagcctattctaaaaactgctgaatcattgagtgcatatagttggagaagtgg
gtggagcagaacagcataagaagagagtaaagaagtgtgcagggtcagattacatgaggccttat
agggcgtgggacagagtttcacttcatcttaaatgcagtgagaagtcattggaggatttcaggtg
cggaa

>chr15_42373108_42373308
ccccacacaaagtgtccctctggtgcccactctgccccgctatggcccctgattgtctctgccac
ccagtcccccaggatcctcaccacgtagagccagagatgccactgaagtaggtcacacagtctag
gaggcccagcttctgcaaggccaataggtggccgtagagtgaggtcatggcccgggcacctcctc
ctgtg

>chr15_86407996_86408196
agaaaatctagaagctagtttttttttctggctcctcctcccgctctccttctaaggtagtccact
ccctcctctttctcactgtctctgatcgtgaccctcccacacctgctttcaaagctttgtctgtg
aaccaattagaggtttgaaaagctggtatcagaagtgcccagtatcatttttatgagacttcatg
aataa

>chr8_56822046_56822246
tatctagtcatccgctgatggacatttaggttggttgcatatctttgctattctgaatagtgctc
caataaacatacaagcacttgttctttttaatgtaatgatatcttttcttttgagtagataccca
gcagtgagattgctgcatcaatggtagttccagttttcattatttgagaaatctccatactgttt
tccac

>chrx_104195544_104195744
cgcccggctaattctggtgcgctgtttttttaagcccgtttgaaaagcgcagtatttgggtgggag
tgacctgattttccaggtgccgtctgtcacccctttctttgactaggaaagggaactccctgacc
ccttgcgcttcccgagtgaggcaatgcctcgccctgcttcggctcgctcagggtgcgtgcaccca
ctgac

>chr19_54675788_54675988
gggtctcggtaccgaagggtggcagcactgggcagctcgttcagcacagaagacagcgatgggcc
tggggaggagcagggggctgggaagacccgggagtctgggccctaattcctcctccctcagacca
ggaaaccaggtccccggcccctcctccctcagacccaggagtccaggcccccggctcctcctccc
tcaga

>chr6_83789481_83789681
aaaatcattgtcattaaatgaagaatgatagctacaaataaagatactttgagaaaggtactttc
tgatgaaaatttatgtttcaaataagctggaaagaaatacatggtgggatagttaacagttagtt

attcctcatcttgcgtttcacctctctaatcattctgaatagcaatgcattcatttttatcatta
ttctc

>chr2_191942955_191943155
tatttgcctgtttttctcacagttgcaggaatatttctatgccgatcattcagatactacctttc
aacatttctcagttgtgttgtacactttttttcattttcctttcttacttgaacttcttctacctt
ccttttcagattcagtttcccccatactttgtatggttaaattactcactttttcttctttgtta
agagc

>chr4_101467177_101467377
cacagggcagcgacagtgagagatgaaggcaacatcagtgtgcgccagagttttgcaactccaca
cgcagtccatcatggaccaacaactgcagcatcacctaggagctgttagaagtacagactctcag
atcccaccccagattcctgagttggaatctgtaatttgataagattctcaggtacattaaagttc
gaaaa

>chr1_57098812_57099012
ggaaatgagtatgggtcacttgggttccatagagaacagagagcagaattttgtaaaacatcagg
aaaaattcagttcctgcaacatttccatttgtcgccaaggctagcatggtctgggaaaaaaagca
taatttttcaaagtaaagttattattttatttgggatttagtttatgaaggacaatattgtgttc
ctcta

>chr12_39786733_39786933
aataaaataaagacaataaaataaaattcagattgtgggaaaatatatactataaatgagccata
ctagatgtttacataaaacaaggatccacattttaaaagaaaatgtttctgaaataaatagcatt
ctaaggaaattgctgtgtagggaacccagtttttggtcttctgaagatccttcagccctacctgc
atcac

>chr16_14805899_14806099
tctaaagccccagggttgctaagggagggcccagaccgaagaaggtttggtggaaagcagaacct
ttgtctccctctaattgctcctaagcctcacgctcccttgccctgcctgtcctgttgcttccctg
atcttctccgtgacctgtagctaaaccttccaccagcgcttgagaacttaatttgaaccggatcc
tttcc

>chr10_72834194_72834394
atgggcttttcccactgttggcccctaagctggttaacagagccacagcagaaactgtgcctgac
cctgcactgacaaattctggaaactccacagcctcggaaggtggtcaaacaacaaaaccactcct
tccagccccagggcattcaagatactatctctgtggctttctgctcatgattttcattacaaata
agcag

>chr13_114214799_114214999
tcgcacaggtctggacggggtcgcgaaggtctggacagggtcgtgggtctggacagggtcgcagg
tctggacggggttgcgcaggttttgagatgggcatttcctcccccagcatctcagggtgttcttg
gcagcccctaccccaggacaaacctagtttcctgtgttcctggtgtttgttttacaaaagcctaa
gcagc

>chr6_158470612_158470812
cggttagacaagtccaataatacaacttgtttaggggatggagggacccagcctgaggccctgag
cccagggaagcagctgtggcaagctgagcctggtcatggtgtccctgaggctctgcaggtatgga
catgcctctgctggtatgcatgcagctctgcaggtggggacacggctctgcaggtggggacatgg
ctctg

>chr3_8155600_8155800
cctgcacgtacacatccagatgatcggttcctgccttaactgatgacattccaccacaaaagaag
tgaaaatggcctgttcctgacttaactgatgacattgtcttgtgaaattccttctcctggctcat
cctggctcaaaagctcccccactgagcaccttgtgatccccactctgcccgccagagaacaaacc
ccacc

>chr9_94108779_94108979
tataataaactggtaatagtaaataaagcactttcctgagtttcgtgagatgctctagcaaatcc
cattcctgagaccaattatgacgtcttttttttctaacaccacttctgacaccaaatgtctgaggg
tttccacaccaattcttcaacatcaagcgggtatccaacaattcagttaaattctgccaccatcc
aaaat

>chr1_110040877_110041077
cgggcaggcgtgctgacagccggcagtttgcgtgggctgtgccatctgatgtctattcccagccc
tgggaggaaggggggagtcatttatattctgcaggaggaaggggccccagctgtcgcctttctgac
cagcaggcctggagggcaggggcacagagcagagaggagggcactggtggtctcctgcttagcct
ggtct

>chr3_13204400_13204600
ccccccttatccctttagctcccactcttgccagcccaagtgcaccatccccagctagtcccctc
acctgggcttcacccctgcacacagtccttctgggaccctctctctctccagttccccttctgag
catgcctctgcttcctgcgagaccctgcctctcactccctggactctggttccctggagcccaac
tccct

>chr1_14730813_14731013
tgcttatctgtgtctgcagctcgatttttcaggctgctctttgttagaaaagaaatgatttgggt
gctgctttttgttgaaagggaaattccactgcggactctgttgcccttactatctgcctaaataa
tttctttctatctcccgtatcagtgtgctggaacaacaaggtacctggcaggcctaaacaacaac
aaaaa

>chr10_88614820_88615020
ctgggattacaggcacctgcctccacgcccagctaattattgtatttttagtagagacagggttt
cacatcaggtctcgaactcctgacatcaggtgatcaacctgctgggattacaggcgtgagccacc
acaccctgcgtcgctaagtttttttaaccagagaggcttcaggtttcagacacacagaagcataat
tctgg

>chr13_62659799_62659999
agtttctttctcttaaaccacacctaagcacagattacattgttaatgcagtgatgaaacagaaa
aacatgattcccactacccctagaagaattttaagtatttctccttttaaagtaaatgtcaatct
tacactatttcctcgtttcctcttaaactttctttgaggtgtatgagttcatacggaactcttta
aggtt

II. 1,484 DNA Non-Enhancer Sites

>chrx_2970600_2970800
cagtcacatctgtaatcacaatacgttgggaggctgaggcaggaggatcacttgagtccaggagt
tgaggctgcagtgagctgtgatcacaccactgcactctagtgtgggtgacagtgagaccctgtct
caaaaaaaaaaaaaaaaagatacattcaaagaagtcaaaataaaacagtataaaacctatctccc
tgatt

>chrx_6179400_6179600
actttgaagaagtcagtcatcaagatgagagacccaactgtcaagctgctaaggatgacctagga
tgggggactggtcctcgtctggcagcaagaaatggccagaactctcagtgctacagccccaagta
acttaattctgcaacaacctggctaagcttagaaccagattcttcctttttttgaatctccagata
agaac

>chrx_11003079_11003279
tcggcctcccaaagtgctgggattataggcatgagctactgcacccggcccaaaatgtttataac
aatatagaaatacattatatttctaactcatgcaaaggtgccttatgagcaagcaacaatcctgc
cccaaggtctgcctggtctctggaaggccatggaggtaatggcaggagggcagctcttgaaatca
gatga

>chrx_22042679_22042879
tgggagctgtatcaatcatgtttttttattttctatattttatgatgtgttgacatcttggtgcct
tgcagacccagggaaggtctgtccctcccagggttagctaattcctagagatagtaactgacttg
cctgtgagcatgcctttgatatgcaaatcaaccaatccacgttcatataccccccacttcctttt
accag

>chrx_23280479_23280679
tacagcaaatagccttggcagatacagtgtttccctccagagcaaagggaggagccatgggagag
agggtggggtgttggagaagaacctcagaagagagagatttatgagaagggtctggaaagccaat
ctaaggaaagtaggactttttaagtacaatggatgcttcaaagttgacttttgcttattttgctt
ttaaa

>chrx_31634879_31635079

gtggaggagggtaaaaaatagtaagagtgtgaatgagagtggaggtaaggggctagtgagaaagg
aatggtcataaagaattagggaggcagaatctacagaccttaaaaatgaacttgaaagtgaggca
agtaagtgggagatttagaaatgactatatgatctgaactctctgactgaaaagatggtaccggg
atgaa

>chrx_33563079_33563279
aaaaaattggaagaagcaactgttataccagatgtgcagatatcaatgtaacataaaaaggaagg
aaatatgcaacaccaaataaatacgataattctccagcaacagagtccaataagaaaggtacgaa
atgcctagaaaatcattcaaaataatgatattaaggaagctcaatgagatgcaagggaacacaga
taaac

>chrx_35196679_35196879
tgaaatttggtatgcccaagtaaaaaatagtacctttccaagtttaatttagtctttctctaaac
gtttctaaataaacatttatttgctgtcatttgtctatgaaatgctatcctgagtctcagtatat
aatgaaataaaaccttgaacatggattcctaactaggtctactgtaatgtaggtatttctttttta
ctaca

>chrx_36223479_36223679
tacaaatttgttaaagagtggtaatctgataaagataataagtgcactttgaggaaggtgagctc
ttgctttgagagtgaacttggttatggaagttgtgctgaagttgaggcttaggctggctctaaaa
gaaaggaaaaatgttgatggctagaggttatcagaggagaagaatgtgttattggtgagtaactt
atctt

>chrx_36884279_36884479
gttctttcaccagagtagcaggaagaaattctcttttacaactcttaagatatgactgtcagcta
aaagaagatgggcacaatttgtgagtaattgattgttgttccagcaaacaagaggcattctcatt
aaagtatttcaggaaagtaaatctgaatgtataaaagttgaagaaaaaaatggcaaagtaagggct
caaaa

>chrx_37379685_37379885
aatgaatatttctgccaagtaaaaaatgtttaacattatcccagcttagtaaggggaaaggcagg
ggataagggtgggtagcaggtagaaccttcattagagtgagatggctccctgaggaaaacaactg
caactgtgtttttcgagttctattctcaatttttttgttttctttttgagaaagagcctggctct
gtgac

>chrx_42545456_42545656
gggaaatgttaatctaaatgaaatctttcattttgcaaaatcaacagaatttaattcagcaaata
tttattgaacaaacactatatgtcaaaaaaactgtttttgaagcccaggaaaaggatagagaagga
aatcaagatcctaagaggtgaagcaacttgaaagttgacacatcaaatgaatggactgagacaag
gctag

>chrx_53396675_53396875
aagaaagctgtagtcccactcccatccaccagcaaaggccaagagggaagcttagacttccacac
ttgctagctgtaagtggttgcccctctccctttctgctggggtggtgtcagagaaggttgcacgg
gcagctggaactcccactcccacctagcagtaactaggaaaccctcctggtgaggtatcaataga
gagtg

>chrx_55956475_55956675
cctcttttattttgctgagcagtggtttgtagttctccttcaagaggtccttcatatcccttgta
agttggattcctacgtttttttattatctttgtagcaattgtgaataggagtttcactcatgattt
ggctctctgtttgtctgttattgttgtataggaatgcttgtgatttttgcacattgatttttttat
cctga

>chrx_57973875_57974075
gattcttcctatccatgagcatggaatgttcttccatttgtttgtgtcctcttttatttcgttga
gcagtggtttgtagttctccttgaagaggtccttcacattccttgtaagttggattcctaggtat
tttattctctttgaagcacttgtgaatgggagttcactcatgatttggctttctgtttatctgtt
attgt

>chrx_58186875_58187075
ctggctagccatgtgcagaaaaatgaaactggactccttccttacaccttatacaaaaatcaact
caagatggatcaaagacttaaaagtaagacctaggaccataaaaattctagaagaaaacctaggc
aataccattcaggatacaggcatgggtaaaaacttcatgactaaaacaccaaaagcaatggcaat

gaaag

>chrx_62432075_62432275
tagcacctgcttgggtctggggaaatggtaaataataacccagtgtgaatccacagtccagtgca
ctgccctcaggggtctccacattgcggtccatgctattttcaggatttgggaggacagaggagct
ttcccactgtaaggatcttggcagttcacagcaagaatgaggactatcaaaatacttcaacttac
ccttt

>chrx_84579144_84579344
tgggtcaattttggaatatgtgccatgtgcagatgagaagaatgtatagtctgttgttttgaggt
ggagagttctgtagctatctgttaggtccattttgtcaagtgttgatttaaggttccgaatatct
ttgttagttttctgtcttgctgatatgtctaatactctcagtggagtgtagcagtctcccactgt
tattg

>chrx_88141744_88141944
cttcttgggaaggctgttcatgtatttgaagggatttgggtgttgtcatctaaattttcggtcac
tgtagccatatgtgcattagggatcaccccaagcccagaaacattgtgtttttttacagactcata
gaaatactgccttggtagttttaattaacatccagaataattctctggattaccaggcagagact
cttgt

>chrx_95273944_95274144
taaaactcccacaacaaaacataaggggaagtcttcatgacactggtcttggcaatgacttcttc
gatatgaaataaagcacaggcaactaaaacaaaaatagatcagtgccatttcatcatgaaaaaaa
gcctctgaacaccaaagaaaacaatcaacagagtgaaaagacaacctacagaatggaaaaaaatg
tttga

>chrx_106473344_106473544
ttcacccagttgctcaagctaaaaacctaaatcagccatcattcctctttcctttatacctcatg
tctaatccatcagcaagtattactggctgtaccttcaaaatatattctaaatccatctaattttc
attaccactgcaattgctatcatgttactaacgatcatcatctctatctggaccattctgacgag
tctct

>chrx_107912144_107912344
ttgtggtccataccagaagtattgctttggttctgtcatatgatcagcaaatctcaccaggcaca
attaaaaaaattatcttctataggaactgggcaatcaaatttatctccaacatcacagcatgatc
aaattgactagtgatctcttcataccaacctcatgtctatacctcatttgtcttgtgttcactgc
tctag

>chrx_110463944_110464144
cctcattttgtgcattcactttgaagaaaaaggtttctcaaagatgcacactccctcttcatagt
gttgtgtttgtttttaagttagagagtagtccctcttgcattcaaacctccttcaaaactcctta
cccaatgtgatgtttttcacttgcattgtcattagatgtccagaaaaaaaaaagatgtcaaaatg
ttttt

>chrx_121572119_121572319
ctttctgagttctttgtactgttttatagtttttcttgcatctcattgagtttctttaaaatcaa
tattttaaattatttatcttggattatttaaaacaaattattttaattattgtgggtacatagtc
agtgtatatatttatggagtacatgagatgttttgatacaggcactcaatgtataataatcacat
cataa

>chrx_125765719_125765919
caggatgcttttgctgattatctggaattttctacatagataatactgtcagctctgaacaagga
cagatttatttattccttcttgatctatataaatttgatttactttcttgtcttactgtcttag
ccaggacttaactaaaatgttgaataaaagtagtgagagcaaacaacctttctttttttcatgttc
ttggt

>chrx_128412519_128412719
ggtttttccaatgttattttctagaagttttatagtttcaggttttaaatttaagtccttaatcc
atcttgagttgatttttgtataagattagagatgaggacccagtttcattctcctacaagcggct
agccaattatcctagcatgatttgttgaaaagggtgtcctttccccactttatgtttttgtttgc
tttgt

>chrx_134773134_134773334

gctgctgccatattagtaagtgctggtggaacagttgcgatgacattatcgggtgctggttggcc
attttttgtcttctcttcatggacattgtgagtgacagtagcatctggaaacaaaatgaagaggt
taatttttagttaagaacattatgtgaagcccacaggtatatcccttttatgctcagtttctaga
aaact

>chrx_136699334_136699534
gtgcttactatgcctcctgttcataagtcaggtttgcaccaactgcatggcaaggcagtgattaa
gtactttcttctttttgaaactttagcaatagaacagcaagccacctgccttcctatttttcat
atatccatcatccatcataggaggcagcttggggcaatgcagctattctcaaacttttcttgtct
cagga

>chrx_137172334_137172534
tcatctcaatacacacagaaaaaacatttgacaaaatccagcatccctttatgattaaaaccctc
agcaaaaatcagcatacaagggacataccttaaggtaataaaaccggtctatgacgaacccacaa
tcaatattatactgaatggggaaaagttgaaagagttcaccttcaggactggaacaagacaagca
tgccc

>chrx_152839206_152839406
cagctgggccttcctcctgggtcctctttagtccgaccacccgacactgactcgccgcgcccagg
tcagttcagcttgctggtgttgggagcacaggtctaggctcacacggtcctgggcatgagtgtgc
acacaaggtgggaggcaattttcatgaaacttgccagctgcaagcagttctggcttgccaacaca
cacac

>chrx_154950006_154950206
gggcctccctggggcctgggggatctcatcctaccttgaagggaaggacacaggcctgtctggtt
ttgccacctgctgattgtagagacccatggtcttgagcaagcataggcattagccaggtagtagt
tatagcaggccttggtcaagaccccgtgctgtactggcttcaggtctgacccagtgcagtcacag
tggtg

>chr13_23321400_23321600
gaaagtttgttcttggtaattttctttcaacacaatgacttttaccccattcttaagcaatttaa
acacctaacaggattgcaaacactttctgtcatcttcactaggccattggttaatttctgaaagt
ctacagaaaaataaatttaaaaagcaatgcacttctaattataagatgtgagttctgccaggttc
acccc

>chr13_32440600_32440800
tttttaaatcccatagcctgatagtctcttactaataatctttattaatattctcttgaacttga
tattacagtattgcaaacagtgctttgtaagactttctaatggatcaaagacaacttttattagt
tgtttgatgtgtaattctagaagactgtaagtttccttagacctcaaggccctattcttcgaaca
tgctt

>chr13_34766000_34766200
aataataaaaaaaaggccaaaaaaaaaaaaaaagtcaatgttgtaaagaagaaactgttctagatt
aaaagaaagtaaggaaactgggtaaataaatgcaatgtatgatgctgaactggaccaagaagaaa
agtacatatttttattttgcttcagtggacatcagtgagataattggggaaacctgaatatggtt
tatag

>chr13_36005800_36006000
tttttgcagctctaattcagtattattaataatggataaaattgcatttttctcaggttgctgtg
ggcagcggggaattatttcaatgaatatttcagcacattattttaatcatttttccaccaaatcc
aagtgggcaatattttatacttcttaattcagcctactaagaaaccatggtcattaaattgtaaa
ctatt

>chr13_48780599_48780799
ctagaactagaaatatcgtgtgacccagcaatcctgttactggatatatacccaaaggattgtaa
atcatgctgctataaagacacatgcacatgtatgtttactgtggcactattcccaatggcaaaga
tttggaaccaacccaaatgtccatcaatgatagactggattaagaaaatgtggctcaaatactcc
atgga

>chr13_57229799_57229999
ctaccttggcctcccaaagtgctgggattacaggtgtgagtcaccgcgcccagtctgatcttttc
actgtttacaaaatcttgccttttccagagtgtcacatagttaaaatcctacagtatgcaacctt
tcagatctgcttattttacttagtaatatgcatttatgttatctctatatattttcctcacgcta

gctca

>chr13_60195799_60195999
tgacacgtcacatgaagagaatcaaggacaaaagccataaaatcatctcaatcgatgctgaaagc
atttcataaaatttaaccttccttcatcataaaatctctcaataagctagatatagaaggaaagt
acagcaacacaataaaggcacaactaatgtcagagcaaatgggggaaggctaaaagctttttctc
taaga

>chr13_69082799_69082999
tttttttcttactgagtttttgagttccttgtagattctgaaaagttctttagagaaataaaaca
cactagttggcaattttatatatgtgagtgtgtgtatatataacacacatatataattatatata
catatttaatatatgcaaattcataattgcatataattaggatataatctttccaagaaatgcat
aaaca

>chr13_76555399_76555599
tttacaaaatatttttatgaagtctggagtgaaatattgcaaatattagtggttatgcctgctgg
aatgggaagtgaagcttagtatggtgatttcaaaatttcttcattatgtatattttcattttaaa
atgtctacgatgaatatgagtaaaaattataaaaattcaagtattcaatcaaaacttggatgcaa
gtagt

>chr13_81662199_81662399
cacaacatggataatttggagatgataagtgaaataagccagaaactcaaagataatgcattatc
tcatgtatatgtggaatctgggaaacaaaaaaattcagatatacagagataaagaataaaaccat
ggttaccaggagtggaaggtgggaggaagggtgggaaaatgaggagatggaggtcaaaatagtag
atatg

>chr13_82726599_82726799
attcatattttccttattattcatccattgatggacacttaggttatttctacatattggctatt
gtgaataatgctgaaataaacatagaagtgcatagaaatgcaaacatatcttaaatgcttgattt
cgatttgttttgaatatatacccagtagctggattgctagttctattttgtatttttaaggaact
ttcta

>chr13_84798599_84798799
tctatttatcacagagcccttttctcccaccaccaagaactgtaggacataataaatatccttat
tgcttctctaagagactgggtagattttttttaagccttctatctctggtttcaaatggaccaac
ttctaagtcttccagttttcttattcttaatctcagccaagtatgaaagcatatattccacccct
gagtg

>chr13_88750799_88750999
tcttagaagcttttacttttatttaggatatgttttatagcacacgagaacatactataatgaac
tccctaatgtttatctcccagtttcaacaatttcaactgcctgttagatatcagatagttcagta
aacactaacataatttaaatattataagaatagtgctttttacttattatttatttaaactatag
tttct

>chr13_92325599_92325799
aaaaatttagttattctagtgactaaatattattttgtgactgattatcttttctgaacatctat
actaaccatcgatgacataacaactggttgttcactttgtgcttcatagtttgtgctatggcccc
tattagaagaatatattaaaaacagctcagaggaaacaagagctcgcccatccttatgcaaagag
agaga

>chr13_92764999_92765199
tgtatatatatgtatgtgttgtgaaaaatctgagcatagtataggttgaaaagccagtagggaaa
tgttttatattcaagtatagaaagtataattaagactattcctgatagctaaggagaaaggagaa
gggtttttacatgaaagcatttatgcctatttacataaaaggtgccgcaaaactgatttcacact
gatta

>chr13_104093399_104093599
ttggtatgcctgctgcgatatcttctgttaccctaaaaacactgctctgagagccacctgtgttg
gtgcctactcctccatgggagtttgggtcagagggcacattctgcttattcatgtgccaacataa
tgaaacctcaataggaaccctaatattcatgtcccaaagactgaactgaatctacagtggtatcc
ccaca

>chr13_108353599_108353799

caaatagataaagcaaatgtggtatatataatacaatgaaatactattcagcttttaaaaaggag
atcctgccatttgccacacatgactggacctagaagatatttgccacacatgactggacctagaa
gatattaagtgaaataagccagatatccaaagaaaatattgcataatctcacttcaatttggtat
catta

>chr13_109173199_109173399
tgcttctcttctgagtcagtagaggattggaattaattacaatttgggctaaggggcctcaaatg
agtctgtgcaatgggtgattcccatcttccacgtgttctttcctcccacgtccccatctctcttt
ttccttctttccttccttcctttctccctctttccctcctttgctcttcttctgtcacttcctcc
atttc

>chr13_112035799_112035999
ttcattctcgattttcagcctttccttctcggtttgttataggaggaacttttaccaaatgcctg
ggatttctgatattgagtccatgtctgatgcttggtttctgtgggtgaggtttgctggctggcgg
gtgattgtgtagggacttcactattttgtcagaagacccctaacctctcttgtctttccttgtgg
gcatc

>chr12_7669133_7669333
ggtgcagcacaccagcatggcacatgtatacatacgtaactaacctgcacattgtgcacatgtac
cctaaaacataaagtataataataataaaataaaataaaataaaataaaataaaaagatctttct
ccaaaagcataattgatcaatcatctcatagttaggttttgaggtccctcagtgctgggatgaac
ctgtt

>chr12_17599133_17599333
aggagccccagtgaaccctggactccaaatatatttctccttgttcctattgtgttgtagcaacc
caatttctccatggtaattttaacaagtcttctaagcaaacatagtaattgtttttctgcatttt
gtttcattggcaagatgagcttaacatggccatcagtcaatctcagcttccagtttacagtcaag
cataa

>chr12_25798933_25799133
tggatgaggacaaatcctgggttggttattgcattgtatctaataattctgatgataggattgaa
gtcctgtttgttggctattgacagtgtagaggtcaaaaataaaatcacaatacctgggttaattt
tttttaaataaatagtaaaccccaagctatgagggatggcattgttcaagatggtatggggtgga
agacg

>chr12_34217133_34217333
aattccacagtgctgaccatcaccctcctactaaagtctgaggcctactggagatttctgcttag
atgtttcataaatacctcaaactcagtgtgtctaaaatctaagccataattgcccctaaatagga
atttcactgacttatctgattttgccaatggtaactccagttcacccacaataggcaatttagag
taatc

>chr12_60761533_60761733
tacccaaagcaatttacagattcaatgcaatctctatcaaaataccaatgatgtttttcacagaa
atataaaaaaaatcttaaaatatgtatgagacacaaaaaaagcctcaatagctaaagcaattctg
agtaaaaagaccaaagctggaggcatcacattacctgactacaaatatattacaaggttatagta
aacaa

>chr12_74299133_74299333
aaagcatttaaccattctatttatgatggacatttggagtctttccagttgtgcattctatataa
cgctgcttggaacaatcttaaataaatctcttcatgcaaatagttctgttggaaacagacctagg
aatggaagtactgattcatattacatagatatactggtggctgggtgaattaatagtccactaaa
agcat

>chr12_82013469_82013669
tttagagtttctcccagttagataaaatgcaaacacattggaaaatgtaagctttagctactcag
ctgttaaggtttaatattcctgatactcagctgcaaaggtttaatattcacgatactaaatattc
acaatatttccttgtggtcacaaacaaaataaatctgaccatttaagaagaaaggcaggccaggc
gcggt

>chr12_82618069_82618269
acacttctcaaaagaagacatttatgcagccaaaaaacacatgaaaaagtgttcatcatcactgg
ccatcagagaaatgcaaatcaaaaccacaatgagataccatctcacaccagttagaatggcgatc
attaaaaagtcaggaaacaacaggtgctggagaggatgtggagaaataggaacacttttacactg

ttggt

>chr12_82738069_82738269
acatatgaaatcaaattaaaatgcagtaattagggtagaccctaattcaatgactggtgtcctaa
taaaaaagaaaatctgggcacagagacatacacacaggaagaataacatgtgaagactggagtta
tgatgccataagccaaaggaattaccagagccagaagagaatcctggaaccaatctttccctgtg
acgca

>chr12_83334869_83335069
gggaacatggtgtcacaaatgttaatatccagattaaaaagagccaagtgttagttgcattgtga
tatttaggagggagatctatctttcttatattttttttaaaagatgggatctcactgtgttgcca
ggctagatttgaactgctgggctcaagtgatcttcctgcctcagcctccccagtagctgggacta
taggc

>chr12_84686669_84686869
agtctgtatttgaaataaaaaagttaaaaataaaaaagttgagatttaaaaaaattaatacaatt
taatgttagcttttcaagaagaaggaataaatcatttgctcttcttccactacataataggtatg
caatgtgaatgctaatgataaaatagactagacaagtgtgactctaaaataaaatagaaagaaaa
aacag

>chr12_99985869_99986069
tactttaagactgtcattctgttcatactaatataatggtgactttattactagaaacaagattt
tctctatctgggtaattttagttctgttcattttagcaaaattgtaatgactagaaccatcttgg
tggttgatctaaagcacacgaaatgctctttattttgccagctacaaacatgttaatatgaaatg
cattc

>chr11_21024024_21024224
tattagaaagctttaatcagaaaaataaagatagatagatagatactgcagagcaagggttagctaca
cttagggacttcctcagccagaaaagtctccatgggcctgcattaaacctgcctctacattccat
ccaacaacagtctaccctgctccatccaaaaaatatggcagaaagaggttctcttgtctagggtt
ctgct

>chr11_21050624_21050824
gagaagtacacagattttgcttctttgtaaccgtcttgtttgggtggtaaggatgtgcttgctc
tgctatactttaatcgtattcattaaaggggaagagaaaggggttttgtcctcaacaaatgaca
caaggactcaaatacagaagtatggagttcttcttaggccatatttagttcactttaacaagctt
aaatt

>chr11_23761224_23761424
gtcaccttataatatacattgtacccattaagtattttctcattctttatccctcccactctcta
gcccttccaagtctcctgtacctatcattccacactctccatccatgtgtacttatttagctccc
atgataagtgagaacattgatatttgactttctgagttgtttcacttaagataatggcctccagt
ttcat

>chr11_26455824_26456024
cctttctcatttctgattgtgtttacatgaatcatctctcttttcttctttattagtctagctat
tggcctatgtacatcattaatttttcaaaaaacaaactcctggattcgttgatcttttgaatga
tttgttatctctcgttcttcagtttacctctggttttggttatttcttgtcttctgctagctttg
gggtt

>chr11_26616424_26616624
atacttccctcagggggccaggtttctccccccttgaagagtatattgtggcctggcgggactgca
gaagaatataagttgtttctttcttagcgtatgagggtcaaattggtcccaattctccagaatac
atcttaggggcatttttgccttggggggaatgtttcccatctgaaaaaagaacatagggatgcca
ggatc

>chr11_38876624_38876824
tattaaatatattatttaagaaatacaattttacacagagctaaatttcttttaacaaatatttg
cctaataggactcatatgcctttctctggggaagggcattattttattcattatttacctttgta
ttcctgcacaaaaatttggtcatactagtcagggaagctattatttgatgatatagaggattgca
taata

>chr11_84184952_84185152

atctaggtaaaactcatttatgttattcactattggtgagctatggattttcagctgtgtttctc
agagaggtggtaaaagtagttcattctcctatttatgtcatcctgccacaatgagcatgcagttg
ttcaattcataccttccatgtggggtatgactctgaaaacctcctctttcatacttatttgtttc
tctca

>chr11_97385790_97385990
ttgcatcctgatctatatcataccaaatgatgtgggctctgcctgcaggaaaattgctgaggttt
gcaggggaccaggagaagttttcaggtttgaaaagagaaggagtgtggagtaggtagacagagga
gattgtgggagtgagaatgaacaagtaaaacattttcctggctttaacaagggaccgtatagccg
catcc

>chr11_104854590_104854790
aggagatgggtacaatcctaccacattccaagaggcaggcccatgagatgagtacctttatttct
cccattttacagaagaaaatcagacttcaggctgtagaataacctctgaaatcacacaattggaa
agtagccaagctggaattttcatcaatgtgtctaagaaatgaaaacctatgctttttggcacagc
atttc

>chr11_127045790_127045990
atcaaaacccaaggaggggggtcgtgggaaccccgattcaatagctgtttggtcacaagtgtaggt
gacaagctggacgtggtcatgtgtctgtagtcccagctatttgggaggctgaggtgagatgatca
cttgagccccggagtttgacactagcctggacaacacagcaagaccctatctctaaaaaaaaaga
ataca

>chr10_44933994_44934194
gcccaaccttccctggaatgagtcttcggttgagggggcctgacgtcagcgccctgcagggttca
tctgtgtcacctaaaagtgtccggctgcttggcctggctatttctgctgaatgcacaaaaataca
ttttgccttttctccttccaagttgcagtttcttttctgcaaatgaagcagaagaaaagtgctgc
attga

>chr10_48880194_48880394
gatcactggacacctattttagccttgggatttatatcacaattccctaagacttcatatttcca
gaaaagaaattcaaactaaagctcacaattactattattatggtaatcctgataagatgtacatt
ttaactaaaagaagttcccactattttttttctttctacttttcaataatccttgggaccttagt
aggag

>chr10_57439794_57439994
gtaaaaagaactaattaaaattttattttaccaagtttaaactgagataccaatttagtcttcac
taatttactggtttttttatttatttcactgcaaaaggggttaaaaatccctacattgctccttca
tagaaagattgtacatgcccaactcaatatatagggaaatgtttttaggctgtataaaagcaaga
tacta

>chr10_58686594_58686794
tattctaatgtatagttatcaatatttaaagtcagtaaggcatttacaagttgattttataaaca
atatgattatagaatcatagatttttacaactattacaaaaagatgcttgggaaaaagaaagaga
ctatgtaaactacctctctataatacagacaacaagaacaatgaggaaaaagaaacaggaatgca
aggaa

>chr10_64659794_64659994
accttacccagaaacagttgaattagcagatggattttgacaggacctgcataattaaagtttcc
actgcagctttagaaactcctgttctgaaacccattttgtcagccaatgctaaagaatgtttttt
tggctctggaatataaaatccaaatagtcccagaggagaagctgcctctcagcctggacaccctc
aagaa

>chr10_84261420_84261620
ccatgtctttctctctttcctctctcagatctgtgcttaaaggttgaccatgcatcaagtgttgg
ttgaccatattttagtaactcaattctctaaccaccaaaaaatgggactttttagttctatgttct
gctttatcttactccatagctgatattattaatatttttatcctgcatatttcattatttatttg
cgcat

>chr10_100755010_100755210
cccaaaattgtatttaccatctttaagccttggtttccttacctttaaatgtagataataataat
tctaccttatgagtttgatgatgcctataaacaataagcaacgggtctgctacacatagtaagca
cacaacaagtattaccaattactatttttattattgctgtaatattaggcccattcctgatgtat

ccccc

>chr10_109008610_109008810
gagttttatttaaccgcatatatcatgacctactttccaatttgacttgactctagcataacatt
acgtgacaagcaagaaagtcaaaatattttaccccaaaacatgttcctttgccatattttgaaat
ggccctgcaaagctgtcctttgtgggaggaaatttgtatttgtaaagaatctctgttaacatagc
tagat

>chr10_110855210_110855410
catctgaagccttcttctctcagctcgtcaaagtcattctctgtccagctttgttccgttgctgg
tgaggacctgcattcctttggaggaggagaggtgctctgcttttttagagtttccagtttttctgc
tctgtttttttccccatctttgtggtttcatctacttttggtctttgatgttggtgatgtacagat
gggtt

>chr10_111289210_111289410
aaggaatgtcaaaatacaattgaaagctttatcaatggactggaccaagcagaagaaagaatttc
agagcttgaagactggtctttttaacccaatctaacaaaaataaagaaaaactgattataaaaat
gagcaaagtctttgagaaatatggtattatgtaaagtgaccaaacctatgaattattgacattcc
tgaga

>chr17_15064875_15065075
cagctcagtacctaacaggtcacagaccagtaccagtccgcagcccggggatcggggacccctgt
tgtataccttgattagcaacagtttgtacttatgtatacatgttgtgtttgaaacttaagacagg
ggttaccctttaccagatcatttcaaagaaccagtgggtgaactgggcctgggcagtcctgcctc
tgaag

>chr17_52105401_52105601
ttgcttaaataaagaccaaaaaatgcctatttcaaacaatattcttggaataatttaaacaagtc
aatatagagaaaaaatatagtatcgggcacatcataataactattatagaacattttagttggca
cttctcaaatataactgaccatttgtgtgtgtacacctcttaatagcccaaagaacatgactttg
tccaa

>chr17_52746801_52747001
ttagacaatcatggcctctggggaagttttgataaggtctttcaggggggttcttgggccaaaggt
gtccatcagagaagtcccatgtctcccaaggatgcgtctaccataggatccctgccaagctcagt
tattggctggaagcacccatggaaagtatggcctcagtacatatgtggcctcagtacatatgtgg
caata

>chr16_13749099_13749299
aacttaaactgtttccagtagtcttagcattaataatactattgtcatttttttattttgaattat
gcatacacatgcataaatataaaatgtatgaaatatgtatgtgaatgttattttgtgtctagatt
agcagcatgagaaaatagatgcaaatgtaaaaatcacaacaggtaagttacaataataattttta
tttaa

>chr16_20660099_20660299
tccacctttctggaccaaaccaatatacaccttacgtgtattaattgatgtcttatgtttcccta
aaaatataaaaccaagctgtagaccaatcaccttgggcacatgttctcaagatctgctgaggctg
tgtcacaggcatgtctttaaccttggcaaaataacctctaaattgattgagacttatctcagtat
tggtt

>chr16_34459299_34459499
tctcagctactcaggaggctgaggcaggagaatcacttgaacccgggagtcagaggttgcagtga
gccaagattgcaccactgcactccagcccaggcaacagagtgagactctgacttaaaaaaaaaaa
aagttcccagggattttctgcttcccaggtggtgcagatcaggcaggaccatcagccatcccctt
gcttg

>chr16_35139099_35139299
gatccagtgggggagtccttggactctcactgcattgggcatgcagagtatcaaagtgtaggggc
ctgcccactttggttgtagcttttcatgagggtcgggttggcagataaacatgtctgtgcctgca
agacagttatgtatagaggacaaagagatgactatagggagaggcatggcctcatttgctgcttc
atgaa

>chr16_51240299_51240499

aagcggggcatggtggctcacgcctgtgatcccagctctcagggaggcagaggtgggaggatagc
ttaggcccaggagtttgagacctgcctgcgcaatatagcgagaccccactctccacaaaaaggta
gaaaaaaaagccaaaaacaaaatcagcataacaaaatagttagaaaagaatatttgaaatgttcc
caaca

>chr16_53004699_53004899
aaacattgctgaagttgtttatcagctaggagattttgggcaaagactctggggttttctaggta
taatatcatattgcctacaaacagaaatagtttgacttcctctcaccctatttagatgcttttta
tttctttctcttgcctgattgttctggctaggacttccagagctatgttgaataggagtggtgaa
agtag

>chr16_63520499_63520699
aaataaagttttgtctttatttctcaaataaggaatgggaatataagcagtgaagtatttagaca
gggtcacaggcagcaactttaaatgacagctcatctcatttccactgaggttcacaaggcctgcc
caggaggccagagttcaatctgaattcatcttgcttctagcatagacaacagtgcatttcccca
catag

>chr16_80487299_80487499
ccatatacacgatgctttgcagtttgcataggaacttactagcctgagggagcaggattgaggtg
aggaaaccagatcattacaatggcaaaatcacacttataggagggtgaaggaacaggatgggatg
gagttgagggttatggtcagaaaggaccatttcaacccaattttaaaacattaacaggcagagct
cagtg

>chr15_24378707_24378907
aacatgggaagagtgtgtacaagattttgagtagaactggggaatacatgactcttagtagctgc
atgataagtttcctgattacagtgaaaatttagacacaccaaaaacaacaacaacaaaaaaaccc
aagagtatagaatcaggttatcctggaggaaaacatttcttttatagacctctaaaataaaatat
ttcag

>chr15_37578308_37578508
gccgaatatagacttcctggcccatacactccatttgctactgcagaataagatgattctaattc
aaaaaggataatgttggttttaaaaaagtataaaatagaaatgactgttttcacagtaccataaa
tgtaagcattgttatactaaaaatattactgttatgagaccaactcacgcacttccagccttaga
taaca

>chr15_55204908_55205108
ttcctctaaaatctggaacacaacaaggatgccagctatcattactgttattcaacatgttacca
gaagtcctagctggagcaatcagacaagacaaaattataaaggacatccaaattaggaaggtaga
agtcaaattatccttgtttgccgatgatatcatcttatatttggaaaaacctaaagactccacaa
gaaaa

>chr15_82893145_82893345
gcctcccaaagtgccgagattacaggtatgagccaccgtgctcagccagtttcaccccattttca
cccccaattctgttcttactctctgaggccacaagcccttttcgaggggctcttgtcacactttc
agctcaccaggagcctgtggttatctgtaacctccagatcttaccttcacaaacactgcacacct
ctggc

>chr14_30865049_30865249
tatggtcagtcttattaattttagccattttattgggtgcttattatagttttaatttgcatttc
cctaaagactgtgatgttgagcatcctttgtgtgtttaattaccattcatatctcttctttgatg
aagcatctgttagaatcatatgcccacttttaattggattgtttgtcttcctataattaagttgt
gagat

>chr14_46164450_46164650
gagagaggtaatgtattaatatacctgcaaatgtagtcatcatattctttaaatccataagcaga
gtggcttctacctagtagaaaataaggcagaaaagcaaagaatccttacagttttctctacacca
aataatgtatttgggtacaatcttattagatatgaagctattttgagcatgaaaaagtttctaat
ctttt

>chr14_83689447_83689647
atctacttgcgtagaacctcatggtcaacaggaggtgagacctgagggacttcaaagttctctac
tgtgtatgtccacaaccctgattataagtacaacttgacaattcaacatgactttctagattccc
aggaacattttgtacattttcaaagtctcctatggacatttaattcccacatcttcatttttaga

tttct

>chr19_6291200_6291400
ccaggccccccttcctctcttagcacgactgtaagcagcatctcaggtcttcttccccaccacgcc
aggagtcaacatagtcttgtgactacagggagcttttctctgtggggttacttttgtttccgtta
gggagtgtattagttcgttttcatgttgctgataaagacatacccaagcctgggaaggaaaatag
gttta

>chr19_32574560_32574760
atctcttgctagaaagtcattatctatagcttaaaagaaacaatttctctatgggtttgtaccaa
aaaggaaatattctatctgctttcttggcttcctctgatggggcttcacgaaaccagtttggtct
gggctgtacggtgaacacctgtcaaaacgtatggaccatgccatttatcaaataatctgctccaa
gaaca

>chr18_7218200_7218400
tccataaacacaggctgtcattcaatttatttgtaccctcttcaatttctttcattaatgtttta
tagttttcattacaaagctctttcacatctttggtcaaatttattgctaggaattttattttatt
ttttttgtagctattgtaaatgggattgttttcttgatttctttttcagagagtttgctgttgat
acata

>chr18_14785400_14785600
attccattgtatgacagtagcatagttaattgcacagtttttatgctgataagtaataaataaaa
atgaaaacatgcagatttccaagtctttaaaaagttagttttatgtgctaatttttttagttatta
gaaattaaaagtaaaatatttaaaatatttccttgtgcaatcatacattccactaagaatttgaa
ctgtg

>chr18_26622802_26623002
taagttttaaaatgtgattaaaactacatacctgcagaccccaaaatacagtaaaccctatcgca
ataaaacaagaataaaaccacaccaaacttgtcatcatcatattagttaaaaccagtgataagga
ggaaaatcttaaaagtagccagaggaaggatacattatgttcagaagaatacaaaagaggataaa
aacag

>chr18_35407802_35408002
catttattaaatagggaatcctttccccattgcttgtttttctcaggtttgtcaaagatcagatg
gttgcagatacgtggcattatttctgagggctctgttctattccattggtctgtatctctgtttt
ggtaccagtaccatgctgttttggttactgtagccttgtagtatagtttgaagtcaggtagcgtg
atgcc

>chr18_41815402_41815602
gttagttttctaccactgatgtaaaaaattaccacaaacttggcaacttagaatttctcttagag
ttttggggccagaagtacaaaatcagtttcactgggttgaaatcaatatattggcagggccaggc
ttcctctagtagctttaggagagaatatcattgtcttgccttttccagtttccagagctgctttc
cttac

>chr18_41918802_41919002
aatgttctttactttttgattgaagaactccttttagtgtttctcataggacaagtctggtgttg
ataaaatacctcagattttgtttgtcaggaagtctttatttctccttcatgattgaaggatattt
ttgccagatacattctgtgttaagagatgtttccctcagcaagttaaatatgtcatgccactctt
gcctg

>chr18_47940602_47940802
cccatgtaacaaacctgcacatgcacccccaaatctgaaataaaaggtggaaagagaaaaaaaag
gatattccgtctggatgggtcataaaatatgtaaaaaacattctgataattacacgcaagtctca
tcttttaagaaaagagtctctaaattaggcttcaagaaatactgaaattctgttactaattacgt
aataa

>chr18_52323602_52323802
atattttaatgtttaacttgtattaattacatttatttaaaatatttagtaccagaacattgcag
tttgaaattctgcaacactacatttaacattcttctcaccattacttcttattccacctggagaa
ctatgcttatgtgccatagcctgactcaaattacaactcctctttgatgtcttctatttttggta
tgtgc

>chr18_53382602_53382802

taaaataaaatctatatcattttgtaaattggtacttcaattttattcattagtttttaaataaa
cgcattgaaggtaattttatttttattttttttctggttttttttttcttttttttttttaaata
actttaagttttagggtacatgtgcacattgtgcaggttagttacatatgtatacatgtgccatg
ctggt

>chr18_54704802_54705002
ctggttttcattgattacgcagtagcatttagtgctgctggccagtccctggcactgcccgctct
ccttgccttcatggggccacaactttctgacttctcccgtttgcttttgcagacacctcctcttc
ctctagatattcttctccagagaggcaatctttaataacttgagagtcactgatacatataaaac
agact

>chr18_61904420_61904620
ataagagtatgttttatgtatgtatgtatctttcctatcagactaacctttttaagtgcagagat
tatgtataattcaattatgaatcactccctggcagatagaaagcactaaataattgttaattgaa
taatgagatcaaagaggtctatttcacttctaagtactttggcaaatatccactgcttattattc
tggaa

>chr18_66198220_66198420
ttttggaaatcatttctacaaaaattctctacactgtgtacagtatcaaaaatttttaaagtcat
gtctataattaggaaaatctttaaataatatgttgtaagccatactatgaaatatataaagggag
aattaaaatttattgtctgtaacaacagagggaactggaagtcattatgttaagtgggataggtc
agacg

>chr18_73013612_73013812
ctctaggaaaggtttcctaagggagatgattgtcacatattttggttacaattcttttcattaaa
atgagtctagattgaattcattgcaaaatcagctttaatatcaatatttcatgaaatgagcttca
taccccaaatgacactcctctcacttccggggaggggtgctgcgagttgctgcaggctgggtagt
gccac

>chr22_48727536_48727736
catggcctggtagatggagaagacacgccctctgctgctggtgcgtctgagggatgggaagagag
gaggtggctgagggcaaggggggcaccttcctcacctggagtccggatggagtggaccagcctggt
cagcggcggatgctctgcagcaggacacacgggcagggacgtccccaggggagcagtgccaggcg
agtcc

>chr20_18901200_18901400
gggggcagttttgtctcatggtttaacagcatccccccttggttgtcacgatagtgagttctcat
gaaatctggtcgtttcaaagagtgtgacacctctccccacttcctcctgctccagtcatatgatg
tgccagccccccttcaccatctgccatgattgtaagtttcctgaggctaccccagaagctgagcag
atgcc

>chr20_22647400_22647600
cacaaacacgtaagtaatctctaagaagttccagactccccccctacagctatcctgttcttctga
gccttcattagaattgccattaacatggccgggtacggtggctcacgcctgtaatcccagcactt
tgggaggctgaggtgggcagatcacgaggtcaggagatcaagaccatcctggataatatggtgaa
acccc

>chr20_31725139_31725339
gcctgacccatgttttctctgaaaatttcttatctgtaacccaactcctggtaccagtttctgtc
ttagttattggtttggttgcaaggagcagaaacttttctgacaaaaaagaaaacagtaaaaaaaa
aattgtgaaaagggaagtagggttatctctctgatatcaaagtttatacatcccctggacctccc
agggc

>chr20_41154186_41154386
ttcacttaaaaacacacactcgtcctctcccaacatatacgtaattgttacagaaggatttttttt
aggatacacggttttcaatattggccatagtgaaagtaagttgggtaatttccacatttccattt
ccctaattatctcttcaaatttctctgattacctcttcgagtctctataatatgaagagttgtga
ttttt

>chr20_51449393_51449593
tcttttgcctctaatgcttgctcatggctgtgttttctgcttgctagccacataaccttggctaa
gtcacttagcctcacgggggcttcagctgacttatttgtaaagtacagggtgtaacaaatgaatg
atttttggtgtttgtttttgtttttgagacagggtcttgttctgtcaccgaggctggagtgcag

tgctg

>chr21_15593129_15593329
tgtctggaacaaaccatcatgtatcttacaaagtccaacgtcaatgtcattcttatttacagtca
ttgatttagacaaaggttgattttaattcattgatatatatatagtactgtaaacaatctataaa
tatgatttgagaggtcagatatgcatatatcttctctgagtacagaattttaaaatcactactac
gtcct

>chr21_25239729_25239929
aggcaggagccagaggtgctgattttcattcagtgtgtggggtcagaaccaaccaatgaagattt
attccattgaacgtgctaatagtacacatgttgaaacatgcctatagacagaccatttcaactga
aaaatgtaaaagatatatattatccagagatgttaaggaatttgtccaggtcacctactgagtca
attgc

>chr21_41258130_41258330
agcccaccatactgcaaaagtggtagtagctctgcccaatgtcaaggaccattcagaaccttcta
tgatattaattttcaggaaactgacagcaacacaaaagtcgtcaccatgagtctttgttgagcca
agttgaataaacaagtggcttattttactaaaatacaaatctactaaatacactaatactagtaa
aatac

>chr7_8715875_8716075
tgcaataatgtaactagtttaaaaacagtgttcaaattttgtccaaatagtctatcaaagcaaag
gatggaattctggtgctctgtactttagttggcttgtgtgtattaagatgagtagctgtaaactg
aagtcatttgcataacactccacctgagggccccaaatcaggcctcatctatgcaatcgtgattt
ttatt

>chr7_11293675_11293875
aacctcgtctctactacaaaaaaaaaaaaacaaaaaaaaacgaaaattagctgggcatggggcgg
gcacctgtagtcccagctactcaggaagctgaggcaggagaattgcttgaatctgggaggcagag
gttgcagtgagccaagatcatgccactgcactccagcctgggcgacactagactagtgactcggt
ctcaa

>chr7_13413475_13413675
cgtattggtatgttgtgtttttgtttttatcttgttatttttaaaattctttttgatttactat
ttgacctaatgattggtcaagagtatattgtttagtttccataacagtttgaattttctcgtttt
cttgctgttaatgatttctagtttcattccattgtagttagaacaggtacttggtatgattttga
tttta

>chr7_13508275_13508475
tattctagtagtaaaatatgtttagaaaagcataatcaatattctcctttgagaagacacaacat
tatcacaatatcagtttatctcaagtaaatgtataaatataatactggttaagatcatcaaaaaa
tacattcatgaaatgtattagaataactttcaaatgtacctagaaggggggaaaaagctgcaaaag
catca

>chr7_21772075_21772275
tgggggacacaatattatcagcctgtaatattactccggtatgacttcatcctatctcattaca
tctgcagcacccctattcccaaataacatcacattctgaggtactgcagatgaggacttcaacat
agaaatttgaggaggagacaattcaacccctaacaatgaggaaatttattatttcacaaacaagg
aattc

>chr7_24586875_24587075
agatttgtttgttatcagtcaaatgaagaagctggagatgttgtgtggcaaatggaaagactgat
agaaacctgattcaggtaagactgaagaagagaccaggtgtatccaagtggaaggaaatgaggct
gggtgggcaggaggggccaagagataatgatgatgccttttatgacattctcaaaatgtttaagt
gcatc

>chr7_49083254_49083454
agtactggaagtcctaactagagcaatcacaagagaaagatataaagggcatccaaattggaatg
gaagaagtaaaattattcctgtttgctgatgatgtaattttacatttgggaaaacctaaagactc
cacaagacaactattagaatttataaacaaatacagtaaagttgcagaatacaaaatcaacatac
aaaaa

>chr7_49126054_49126254

cttccgtctatcttatttattctctccctctggatcaaatactggatttcctcaagcttctcttt
cgaccttgaattttcccagttttctttttaaagatgtttctcaaatttacttgactatttagat
ttttaaaaactttttgaatgcatttgtttcatatatatctatgttttctgttggtcatttattt
tactg

>chr7_53039506_53039706
gattatttcatttgcagtgattaatgatgttggcatttggttcataatataattgtattactttg
tatttctacagcattctttttctaagaacttaaaagtacttctcttttcttataccatcgagaag
tagtgtagtgtacaagtactgtataataattttagttttataatggtaaagtcactagctatttt
tataa

>chr7_79527864_79528064
tatggagaaaaccaaaaagatttgcttgtagtttggaggttgtttctctagaatttgatgatgga
tttgatgtgaggaataaagaaaaactcccaagcgttcagcctacgtttttcattgaattgtgata
ctgtttacacaagtgcaacagtagtgtttttataatggcaaacactaagagaaaaaaatattaaa
gatgt

>chr7_103860764_103860964
ctgcacagatagagccaatttactgagagctgagacagtggtattgcaatagagaaagaagtcaa
taattgcagggccagccaagcagaaagaagggagtttttactcaaatcagcctgcctgaaaattt
agaggctaggatttttcaaggatagtttggtgggcaggggctagaaaatgggaaatgctgcttt
attgg

>chr7_108897564_108897764
gtgatatttcattcatctgttggattaatgcaaaaaatgcaataaaactctcatacatatgagtc
atccactaagtaattcttcaccatgtgatggtttatcatagaagcctgatacattgtaattttta
aaatttaacctgggtaccaacctgagtccatgtacctttccacagggagactgaagctaaaagtc
tttga

>chr7_109685164_109685364
attccttaatccaacttaaatttgtaaatttggtgaattatgtatctgtttacttacatttcttc
tcaggtaagtaacaaattgcctcagaactatttatgtaaaatttaccctttttcccctataaatat
aaaatgctatctatcataaataatattttcatgtgtttgtagattaatgtttaggttttaaattt
tctca

>chr7_110796964_110797164
ctactccatgtgtcagtgatgagaagacgtgattttcaagaagtctgtcaagttcacttaaaagg
tgttcaatatcactgatcaacagagaaatgcaaatcaaaaccacaatgagataacatcacactct
agttcaaatggctttcatataaaacataaggcattaacaaatgctagtaaggatgtggagaaaac
agaat

>chr7_112801764_112801964
ccttacactgtgtttgttttcatcaaaacctttattaccatccgatgcattgtgagcacatagca
ggtgctcgataaatatattttggattaaaaagtttattaattaagaactcacaaatatctattta
caactgaggctgctctcctgggccacaagtcctagaagccatggggtgtagaagttaagatgatg
ggctc

>chr7_114310964_114311164
tactatttttgctgatgttttggatcattatgttgcattgcaaaactctaaacatgaaagctttt
tttttgcctcacatatgttttcatagatttttttaattaacaattttcagtcaactttatttttt
tctaacatttctggtcctcttacaattttttcttctgatttaattccattttttatttaatctctaa
acttt

>chr7_115090164_115090364
agatgcttttctagattctgtctgcactgcctgcttagtggaggggcagaggacatatggctttt
aatttgatatgtttttagcgggagtagccatggggcatgtccacagctgaggctagttttgtggc
ttgcagattctccccacttctcccaagtgttgccatctatctgggtccttgttctcagagcaaag
tcccc

>chr7_118397964_118398164
gcaggctggataggagaactgcaaccacttatagaaatacagtttatataccattttcacttagc
cctctccccccctagcaacctctatttaacccagaaagagggccgtcatccattctacagcctgca
ttccatgggatgggctgagaatttaagtgttcctcaaagataaagaatgaaaccttgtggtgggc

atttc

>chr7_153316867_153317067
gcttggccctgcagttagtgtttagaccccttaacagggtgatttggtaaaagttagtaagcagt
taagctgcatagctgaatgtgatttttaactgtactcttaatcgtggtggcattacaagaaggat
gtatggtagcacagggctggagggagaacaccaatggacttcctcttagccctgcacattcgctc
tgctc

>chr7_159065439_159065639
caagagctcgagatacagaagactgtcacactggccctctgcccttgcaataaggcagagtccat
tgagctgattaacacacaagccatctacagatggcaaagctgaaagggctttgtaacactggggt
tgcaggcaaccaaccagcccctagacactaccgcggggctgagagcctgaagcactcaccctggc
ctgtg

>chr6_22938021_22938221
agggattcaaccatggtatttacagtgtacttttcattggatacttctttatcatggcagatggc
ctaatgcttaagtgtacagatccgtgaccaggtgtccctctcataggatcttatttatactggct
gataccctagtggctcttgtgtgacttgtgtccagtttattcctaccaagacaactactttctaa
gagaa

>chr6_39708622_39708822
ccaacttacaagggatgtgaaggacctcttcaaggagaactacaaaccactgctcaatgaaataa
aagaggatacaaacaaatggaagaacattccatgctcatgggtaggaagaatcaatattgtgaaa
atggccatagagcccaaggtaatttatagattcaatgccatccccatcaagcaaccaatgacttt
cttca

>chr6_56031041_56031241
aagtataataatgtatttcacacactgacattcaaagcatcctacatttttctggagcaggtctc
atcttgctttacctgagcactggaatagtcatagtcatagacgacggggaaaaacattactgaca
gggcgtggtggctcacgcttgtaatcccagcactttagagggcttaggccggcgaatcacctgcg
gtcag

>chr6_70045879_70046079
agtttaacaagttttataatacacaaactatattaatatttttctactgaaattctttttctt
ttttcttttaacttttaagttcaggagtagaagtacaggctttttacacagataaacttgtgtc
tcagggatttgttgtacagattattttatactcaggtattaagcctagtacccattagtttacca
agaag

>chr6_92626079_92626279
aatagcaaagacatagaatcagcctagatgtccatcatagtagattggagaaagaaaatgtggta
catatataccatggaatactaggtacccataaaaaatgcaattatgtcctttgcagcaagaaggg
tggaactcagggtcattattctaagtgaattaacacaagaacggaaaaccaaatactgcacgttt
ttgct

>chr6_103419707_103419907
cctataaaatttactcacctcaaatcttcactataaaaaaaatcatgggccggacgtggtggctc
acgcctgtaatcccagcactttgggaggccaaggcaggcggatcacgaggtcaggagatcgagcc
catcctggctaacacggtgaaaccccatctctactaaaaaatacaaaaaattagccagatctggt
ggtga

>chr6_104564707_104564907
caaaaggaagaggaaaaatagaaatgaaaaaaggccctgactttccataaggagaaagtaatctt
ttggctcattagtgtcctttgattcaaattacttgaataatacatttttgaagaaaagagatac
ctacattttaagtaaaattgatgttgctgtcctatgaatcttttatcattgagttattgaatatt
ttgtc

>chr6_107487907_107488107
caaaggccttttctgcatctattgagataatcatgtggtttttgtctttggttctgtttatatgc
tggattatgtttattgatttgcatatgttgaaccagccttgcatcccagggaagaagcccacttg
atcatggtggataagctttttgatgtgctgctggatttggtttgccagtattttattgaggattt
ttgca

>chr6_113085307_113085507

aaattcaattgggcatcctgttttattttgtttttaatttgttaaaacctgacacctctacaaag
ccaatctctaacatcactaaaagaagattatgagtcttcactgtagatttcaacctgcagaaaat
aactgagaaatcctgttgtcccttaaatactaagacacatttctcatttcattatctctgaggct
gattg

>chr6_161095210_161095410
atgtaaatcaataaaatgtgactcatcacataaatgtgactaaagacaaaacccacatgattatc
ttgatagatgcagaaaaggcttttgaaaaaatgtaccaccgttcatgttgaaaactctcaataaa
ctaggtattgaaggaatatatctcaaaataataagagacatctatgacaaacccacagccagcat
catac

>chr6_164607610_164607810
tcaaaacagagcttcagagtttggtggggtaattttttttctcctgtaatttttttgataatacca
ctgtattacattcttgcatgtagtgtagctgctggcagactcatatcaagcagttttctgttgtt
tcctaagtagagatgcttgtcaacttaaggtggacttatctccctataagctcatattgagttga
aaata

>chr5_2122600_2122800
ataattcatagctcacaattcttctttaagtacttacaaaaatgctgggccactttctttttgct
ctaagttttggggtaaaaaagctgctgttattcaacttggggttcccttgtaggtcaggtgctgc
ttctttttggctgctttcaagatttttttctttgtttttcattttcagaagtttaattggggcgag
tttgt

>chr5_2940800_2941000
tgcttttattttggattgcagggtatgtttgcaggtttgttacatgggtatattgcaagatgccg
aggttcgaggtaggaaagatcccaacaaccaggtaaggattatggtactcaggagttcttcaacc
ctagcccagctccctccctcccccatctaatagtccttagtgcctactgttgtcatctttacttc
catga

>chr5_6975400_6975600
tggtgactataattcaacataagatttgggtggggagacagatccaaaccatatcattctaaccc
tgtcccctcccaaatctaatgtccttctcacattgaaaaatacaaagatcccctctcaacagttg
cccaaagtcttaaatcattccagcattaactcaaaaacccaaagtccaaagtcttatctgagata
aggca

>chr5_11839200_11839400
gaggcgtctagctctctccttaaatgaatctcagcctttttatgatttattagctgtccaagtaa
ggacagtggtacagatggacatttgaaaagtattgctgggatctggggtcatacaattggtgatc
tagtatctaattacccagagaactgaagcatattccatcgaatagccaatttggtctgctaggga
ccaat

>chr5_11840600_11840800
ttctatggttttatatttttcaaagaaataaatattaaatgggaagatgcccattgtttgtgatt
ttgactcaacttttaaaacttgcacattttgatctgaatgctacaaactggacccaccaaaataa
ctcgtatcacttcatgctatttaagaatcatgagtgtttacgtgtaggaattcatgctgtttaga
gtaaa

>chr5_13497800_13498000
ttacttgtgttgatcttttctattgatttctagtctctattacatttatttctgctccaatctt
tattatttccttctattaattttggacttagtttgttattcctcttctagttcattgagatatag
tactagattgtttatttggtatttttcttctgttttgatgtaggggttcattgctataagcttcc
ctctt

>chr5_35554843_35555043
ttctttattaacattaatttccagaagaaaattttccatctgctttttttaaactagggttatttt
ccaccggatgtgtttagtataagtagtcaggaggtatcatgttatttttctgaattgttctagtt
catatattcatatttttttaaagtatggttttgaaagaacccatttttatccaaaagaagttctgg
tagta

>chr5_43735443_43735643
aaaaaagcgaatttaatgatgagtcttggaattgaagttgggtaatgaaggaaaagaagacagga
gtagggtgagagacagtgtaaaagtgatagagtctgtagactggaggtcctgtattggtcaaaaa
attgttgatagggtattggatggggtgcataagaatcacttgagatgttgattggagaagggaat

ggtgg

>chr5_50712443_50712643
tttaaacattaatttaaaaatttatacaatacttttgattcatatttatcactatggggttttat
tttggtatctcctttttttccaggtttccccagaatcccagctctcagtgactctaggaatattag
aatgagattatataatgaaccatttgctttgtcctacaatgcacaaatggaagttttagattaac
aatat

>chr5_51269843_51270043
gcttaacatctaataagtgggttgaatgaaaaattgaatgatttcttagaggaaaaactctctga
atagcccatgtatgtatttaaggagaggttttattcctttgcttttctttcttttttttttttt
taaaaaaaaagaacaattcttacctttacaccaagttagtttaatctccattgtcaaaacaatgg
gtctt

>chr5_62959844_62960044
cataacaattaagttatataactaaggttaaataaaaactctagtactgatgtttccattaatag
agtcattctagttttggcattcttgtgcatacataatgctgtcatgaaaccatattgccttggag
ctaaagtgccattttatgccaaatatttatttgatagggaaaatgagaaccacaaaggataaatg
atatt

>chr5_75309844_75310044
gtcaaaaaatgcagcacttctaccagctaaatgccactgggggcctgcaaattattttaacagcc
aagaagaaggaaagaaagagaggatgaaagagaaagagagagagaaaggaatgaggaaaggaagg
aaggaaagaaggaaggaaggaagaaagaaagaaagaaagaaggaaagagaaagaaagaaaggaaa
agaaa

>chr5_83281044_83281244
ttaaaactgctcatgatcaatatgaggcattgcactagatttctgttctagcataatgatttctc
tagtttagcaagtcttggtgaggcataactgctggaaaaaaaatgtatatttttgtatcaaattt
tgtttggacaaattttttttaaagactctaattcttttctttgctcttgcttataaatatgaagtg
tttac

>chr5_84558044_84558244
agaaaactgtgggtccttgcattttcacgtacctgtgcaaaattactgcgagttccccaagtcac
tttatagatactaagaggagtaaaaggctatagttttttataatttgtcaaatatgtacaactgat
aaatgttgttcagttagtggtcttgtgatattattatttccctagactgctataatatgaagata
ataat

>chr5_101355901_101356101
aaattcaggaaatacagagaacacgacaaagatactcctcgagaagagcaaccccaagacacata
atcgtcagattcaccaaggttgaaatgaaagaaaaaatattaagggctaccagagagaaaggtcg
ggttaccaacaaagggaagcccatcagactaacagtggatctgtctgcagaaactctaccagcca
gaaga

>chr5_104438701_104438901
aacttttgtcaaattgcttaaatgcctcaagcctaaatttatttgaattatagagatgtactacc
taagaatatttgtgtaatgttcaagtaagagcatgtgtgtgagcttagaacagtaattaatattt
gataagcctcaataaattgtaatcactattatagaaaatcacaaatttcttccctgtatctaatg
aaagt

>chr5_117539501_117539701
aataaataagtgtccagcatataatcaaaaatcacctgtcatatgaagaaccaagaaaaatcaca
acttgaatgaaaaatgaccatcaatggatattaacttcatagtaaatcagatgttgaaattatct
aacaagaatcttaaaacaactgtcataaaaatagttcagtaatctgttataaatctctaggaact
aatga

>chr5_118165701_118165901
agtcttacatttacatctaggatccatttacagttaacttctgtataaaatatgaggtttacgta
gaagttacatgtgtgtattaaattgttctggcattatttgttgaaaagactatcctctcttactg
aattgcatttgtacctttttagaatcatcttttattttaaaacactttttttttttttgagat
ggagt

>chr5_120953301_120953501

acgcgccacctttaagagctgtaacacgcactgtgaaggtctgggcttcactcctgaagtcaagc
gagaccacgaacccaccagaacgaagaaactctggacatatctgaatatcggaaggaacaaacta
tagacacaccatctttaagaactgtaacactcaccacgagggtgcgcggcttcattcttgaagtc
agcga

>chr5_125521901_125522101
aagagcccaatttaaaaacgggcaaataatttgaatagccatttcttcaaagagatatacaaatg
gccaataagcccatgaaaagatattcaacatcattagtcattagggaaatgcaaaccaaaactgc
agtgttgctatttcacattgacaaggatggctgtaataaagagaaacagatagtaacaagttctg
gtgag

>chr5_130115501_130115701
tcaacacaaaacaatcaatgtatatgagtaaaattattggacttcaaaaatgaagaaagcacttt
ttggatgttcaggaaacaaaatcacaaaaccaaattattgagaacaaaaaaaaaatcaagcagaa
gttttagactgatccacagtaacattcaaaactagatgacatggtatctatccagaggaaaagaa
gttat

>chr5_137111701_137111901
gcgtagtatttcatggtgtatatacaccacattttctttatccagtccaccactgatgggcatct
aggtcgattccatgtctttgctattgtgaatagttgtgcggccactgtggaaaagtttggtagtt
cctcaaacattcagttaccatatgagctagcaacctcactcctagatatatagccaagaaaaatg
aaaac

>chr5_160446822_160447022
tatatcctattagttctgtccctctaagagaaccctgcctaacacacagcatatccctcatgaat
aaaggggaatactgtaaataaaaaacagctaaaggcaattataataaatgtatttctagcaat
gttaagacaattgttttaagataaaaactcaattctgagatagagcatgaaaagagttgagtaaa
tagtc

>chr4_6520099_6520299
gaaaagatcgataaaattaataagcctccagccaggctaacaaggaaaaaggagaaggacacaac
tgttaatactagaaatgcaagaggatacatcactacagatgccatggacattcaaaggataatca
agtaacactgtgaacaactctatgcccacaaatttaataacctagatgaaatgccaattccttaa
atgat

>chr4_25616102_25616302
tttcttgggtagaaaatatactcagcaataaaatgtcacataaaattaagaaactctttcaaata
aatgtgttagtgataacatagggctgggcgccatggctcacgcctgtaatcctagcactttggga
ggctgaagtgggcagattacaaggtcaagagatcgagaccattctggccaacttggtgaaacccc
gtttc

>chr4_29197302_29197502
gtgcagatatctctttgagatgctgaattcaattcttttggatatataccaaaaagtaggattgc
tggattatatggtagttctatatttagtttttgaggaactcccatattgttttctgtagttgct
gcactggtttacattgctaccaatagtgtgcaaggatacactttccttcatgtgtttgtcaaaat
ttgtt

>chr4_34453405_34453605
cttataatggaatattaagtacattcttctaacttggaaaggaaataaaaactagatataaagaa
aagaaaacaagccaaactcattatatttttgtaactcataaaccagccatgtacacacaatgttg
taagcctattaaatttcattgttttctgcctatataagcaagaacttaggagcactggctccatt
tctct

>chr4_54084843_54085043
gctggcgacgagctgcattcctttggagggggagaggcgctctgatttttagatttttcagcttt
tctgctctgctttttccccatctttgtggttttatctacctttgctctttgatgatggtgacata
cagatggggttttggtgtggatgtcctttctgtttgttagttttgcttctaacagtcaagaacca
cagct

>chr4_64994805_64995005
tttagttcttcagctgaacttatcatatccttgttattccttgtatctgactggatttcagatta
atagtatctctttagcccaatctcccatttcttagtctttcatctacttcactcttcatgtaatt
agttgccatatccactgatcatatcactgtaatacttgcaccctacttgcattttagtgtcata

cctta

>chr4_87321976_87322176
ctactgataaaatataattattcagtgaaatacaagtatgccattattcttttatttttaaaaga
attttattaaatccaaaaatacaagaagattttattgtgtgcttacataaacatggaaagtaaa
aataaatgacaaaatcaaatgtgggtcacgaggtcaattttctcagttaataaattgtaacttaa
aaaat

>chr4_91951977_91952177
taaatttaaattaatattccattctgaaagtctactttagggtttatgatcacacattactctgt
tagacctcttttggtatctggttagtcacagtttttagagcagtggtctaataccttcatggtca
caccttctgtcttcagaaagttcatttagtttcagactgttagccagacatcttacaaatatttc
ccatt

>chr4_96062777_96062977
ttcaatctctattgggaggagaaaacaatgacacatgaaatgccttgaagaaacttacaaagctt
ttcactcttcactctgaagaattcagcaaccactctgagacttcagagtcagaacaaaatatttt
catatgggaaaaataaatcctggcttagggctctctggtattaaattaaaataaaaaacctgcaa
taaag

>chr4_104197551_104197751
gtgactagtaggccacagtgatgttttgggtctccaggaattaatgtcagtttagagccagtgcc
caggagcccctaaaagttctgattattcccctttcctcaatgaacagttaccctgataactgtat
gtccctttggggaagggtgggagaaagattagctgtacaaatttttggcaatgtaccagggctct
tcctc

>chr4_104919951_104920151
aaaaaataaaaagacaaaatgtttacctggttctaggtacgaatcctggaagtggaaaggaaggg
gcatgaatttcggaaatggggaagaagaagaaacgacagcaactagatgtcataatttacagcag
cagatgtgaagcaagtttttggattatggccagtggaaagaaagcatgccagacagctaatggga
ggcaa

>chr4_117691752_117691952
ccacccgtctcggcctcccaaagtaccgggattacaggcgtgggccaccgcgcccggccctattt
ataatctttaaagaatttcaacttttattttagattcagagggtacatgcgcagatttgttacct
gggtatattgtgtgattctgaggtttggagtgtgattgatcccattaccaaggtactgaacataa
taccc

>chr4_120854752_120854952
cgtggctttgaggcatttcacctatttgtgcagccacctcctcctctgtgaaaaggtcactcttc
tactggaaagagaaccctgtttttatataaaatgtttttagcagaatactgtaagttctataata
ctgaagaagacaaacagatggtgagtccacatatgaaaagaagttcagcatcattaaccatcagg
gaaat

>chr4_130792350_130792550
acatacgtctgcatgtgtctttatagcagcatgatttataatcctttgggtatatacccagtaat
gggatggctgggtcaaatggtatttctagttctagatccctgaggaatcgccacactgacttcca
caatgtttgaactagtttacagtcccaccaacagtgtccaagtgttcctatttctccacatcctc
tccag

>chr4_131235950_131236150
gatctgctttccctcagagtacagatcactacttgacattatattatattatgcatttatttctt
cttactttctgtaatgttaggcttacaaagtagaaaatttccctgctgtgtttactttgtatcac
cactgactggtctcactctgccacccaggctgaagtgcagtggcacaatcatggttcactacagc
ctcca

>chr4_131537950_131538150
ttgacagcagtcacaaggctgattatgtactatgggaggggatataattctcacttgtcaatggg
aagagtaccaacatatctggggccatgttttaaaaccaccacagtcttgaaagacattgggaaca
tcactcacagggagcatgtgaacaaggttccaaaggtacataggcaaaacaggccaaagccacag
aaatg

>chr4_135490950_135491150

tgaaaatatttacagaaataaaaaatctttgcattaattagaagataaagcaacattttcttaag
ccactgatatttttacacatatttattattttaatattgatttatgatttttgtctctttatcat
tatagagttttaagttaatgtactgcttttttttcagaatatgtttaatgctccttaaccactcta
aagaa

>chr4_135671950_135672150
aacaaaataaaactcaacaattgtagtttgagctattcctaccaagcaatccatgacaaatccta
aaacaataaatgactatttgatactattatagtctttcaaatcctttgcaaaaatacttattcaa
tcttggcaacaatcacatgatgtaagccgtgttttccatcttatttaattgaagaaaaagctaag
aaact

>chr4_136887350_136887550
tgagggactcacttaatcttattggcttcacatctacacaaatgagacacttagaaagaataatt
ctaaaatccatacagctttaatattctatgatggtcagacatagaatattaaggttggatgaagt
atcttttatgcataaatatataattctatacattttaataaaagttgaagttttaaagctatgtg
tttgt

>chr4_153162550_153162750
atatataaagagctcttatgaatcgataagaaaaaaagccaacaattcatagaaaaatgggcaaa
gatcatgaaaaggtaactgacaaagaaagaaatgcaaatgactttccaacacagataagatactc
aatctcatttaaaattaaataaccaagttaaaacaataatgagctacaacttttaacctatcaag
ttgat

>chr4_156339550_156339750
gtctgtgcttttgaggtcttagccataaaatctttgctagactgatgtcttgaattatttcctct
acgtttttccacagtggttttatagtttgggatcttaatgttttacacactttctagttgtttac
atgcagcatgccgagatctaactctagttatgggtctagagacaacaagaagtagggaaatcaca
tttca

>chr4_160905550_160905750
gatacttaaacttttattactctgttatctagcacattaataaccaagttatattctatctagtt
taataggtgaaagagtgcctagattcaaccaataggctgtggggaaggtttagagtgggaaagag
aagctagtgcgaccctatgggtgcagctctttgggctaaatcagtgtactgttgttgtttacacc
tcaga

>chr4_171853425_171853625
tcaggcttccaagtcagttaacttgattataaatcagtaaaaattattcagtctgaaccaaaaag
aaaaaaagtaaataaattcaaaaaggatcagaaattcaggggtgtgcgggacaatagccaaaggt
ataacaattgtataattaactgcaggacaaaaaaaataaggtgtggaaaaaatatttgaaaaacta
acagc

>chr4_172133825_172134025
actctgcagaggcccacatctgcctgatggcatccacccgcaagggtggccaaatactaaaaggg
taattgctgcctcatagactctccttactacctatgtaacattgctaaaagtcctatgcagtaca
tagtgctaccttttctgttacaggatgaggagaatatgaggcagggaagtttacgactaaccct
ggtgt

>chr4_172695825_172696025
tttattttttcttcagaactaattgaatgaattctgaagacttgctgtttaaattatgcaaaca
taatattttctcacatttatggctgtcgtaatgtttctgttgctgcctcaggaactgcattgtac
ttaatgtagttacagataatacaactgtcataactgtcataaagaccacgtgacaagccaggcat
tccca

>chr3_2444000_2444200
tgaaattttggaaaggagatgtagatttaaacttagaattgtcaggttcaagtcttttcctcct
ccatctctgatttttatttagattttaagtattatgaattcatagtagtcgtacatattaaa
ggggtacatgggatattttgaaacaaacatacaatgtgtaattattaaatcagggtaattgggat
attca

>chr3_7796800_7797000
taaacagagggcaaagaaaggaaggctcatgtgtggactctgaaggaaatctcaatgagaattcc
acagagcggatggtctgggcagggtattttccacagagctgtctttggaaggctgcttaaatgta
tactatccccagagttgtttaactaaaactcagcacaggaaaacatcagggttggcaaggaccac

tccaa

>chr3_22790196_22790396
aagatcccctaggaggataatgcctgttgccgaggaaacacagcctcatttgggtttcagataat
gccaaaaatgaaacaaaaaaaattcacagaaaatacagaatgagttcagcatgtggactacattg
atgttgccttatactctgtgtcttcatcttccttctccttttctgtttcttcacttttgatacta
atagc

>chr3_35232196_35232396
gcctttgtttatgggatatgtgaaatcttcctaaacaaagaccagaaattcttgaggaatcccaa
tcttgctgtttcagattgtttcagtgtgatactggcttcttgcattgtgaactgtgtagtttgaa
agttgcaataaaagtcaaggcaattggggttgcaaaaagagggttttcagaaaaaggtctatttg
tttaa

>chr3_39787596_39787796
tggaattccatgcccacgagcaggtcagggactgaatgaggtctcatgtggtgaagcgagtgtgt
tttacaagcttatgccacctagaagtttccagtggctattttcaaactccatgcctaatgttcaa
tatatgtataatgtaatatttctgttagcaaactgaactggggtccacttgcctgacacagtaag
gcaaa

>chr3_42322996_42323196
acagaatagactctttatagcagtaggacacaaagttataaacaggatctgaggccattccaggt
aagggttaagtcactccctgcccctacacttaaagaataaactatgttttaactgccacaaggtt
tttccttttccctagcagctaaagaagcactgaccttgacataagcaatggtgaaacaatttaca
gctca

>chr3_68396310_68396510
agtgtgatttttgctattgagtttctgaatgattttgcttctctttaacatactccgcaaatact
agttgtctgctttaccagttcacagggatgccctgaatgatgttacaacacctcttccctctctc
tcttgctatccgctcccttgctttctcattctggatttaccaagactaggtattctccagggcaa
aggca

>chr3_70996510_70996710
aacataagtcattgaaatccacctagctgattcttactcatctttcacatcttaaacatattccc
tgattcctccttgtcagagcatagctctgcgtactgcaacttcttgtaacacatggactttcata
tttctctgtgaaactgattaatgcctggctcccagctagactgtaaaacacacaaagacaggctg
gttga

>chr3_87529110_87529310
tctttattgttacacatttatcacttgtgtgtacacacacacacacacagacatatgcaaaacat
atgtagaaaagtgtataaaactttttccttaccaataactttttctcaaccaatctaagtcccc
atcctccatacaggaagaagctatttctgttaaacagaaattttgataagccatgtggagtagta
tatta

>chr3_88989710_88989910
gtatatatgcaactacatgctccagaactatatcacatctgtatcaagaacagccctatgcttgt
gaaatcatgtatgacaaattagtatttaggagtggtatgaaatatttttccaatcaacacttcct
taactatcatttcagtgtacattcttcacagtgaacattttaccttagcttataaaacaagataa
tttta

>chr3_106278110_106278310
agatcagaaggaaagaacccaaattatcattatatctcaaatatgtgtgtcttcaaaaagaattc
aagagaatttatagacaaatatggaaaatgaaaagagagttcagcaggtttgcatgatataaacc
caatcttcaaaactgaatagcacttttaatatcagaaacaactaacttgaaaacattatacttt
tcaaa

>chr3_111948310_111948510
caattaaaacaacagcaataaaaattaccctcttgaccctaaatttacctttcatattagaataa
aatattagtttgaaaaggtaaaatttaataacacaatgtacaaaaagagattatcaagcaaaagg
gtgtatagattttttaaggctgagaaaattgtgtgcgtgtatgtgtgtgtgtataaagaattag
tggca

>chr3_117652310_117652510

aagctaatgaaggttatgtttcaatgaaggcactggcagaactctctcttcttgggaaatgccct
accaatatgttcagatttgtaaatttttcaaaagtaattttttgtattatttttcttaggagcag
gtcctcaaattacagaagccacagaaaactgactctgtccctgggtcaagataatagagcatggt
ggtat

>chr3_117862910_117863110
tggtcgatttgtgtatgcctcataaagttcttgcgctgtgtttttcagctccatcaggtcattta
tgttcatccctaactatttattacccatcttctgaagcctactttcgtcaattcgtccagttctg
cacccttgatggagagacattgtgaacgtttggaggagaacaggcactctagccttttgggtttt
cagca

>chr3_126967710_126967910
attggagaacatgacctgattggaagctctaaaaagacttttcaacattgtggtgattaattttt
agctttgataaaccccagcaagaagaaagagacttaacttaggatttgattttgaggacatttgc
caaagatgttaaaaggctgaaaacatttgatcaaaacagaataataatcattgtaaaataatagt
tattc

>chr3_157444506_157444706
taaatgtaaatggactaaatgctccaattaaaagacacagactggcaaattggataaagagtcaa
gacccatcagtgtgctatattcaggaaacccatctcacgtgcagagacacacataggctcaaaat
aaaaggatggaggaagatctaccaagcaaatggaaaacaaaaaaaaggcaggggttgcaatccta
gtctc

>chr3_158157706_158157906
tcaaaaattgtagtatttcaagagtaagtaatgccttccaaacctagagcacaaaaaaggcaagg
ggagggagagcactgggacaaatacctaatgcatgcggtgcttaaaatctggatgatgggttgat
aggtgcagcaaactaccatggcacgtgtatacctatgtaacaaacctgcacgttctactcatgtg
tccca

>chr3_162219706_162219906
tgaggaactgggaatctcacaatggaattacacaattatggagaccaagaagaagttccacaata
tgccttcagcaaagtggtatgtaattcagccctgttctgaaggcctgcaaatcaggggagctgat
gtaaaatcaagtccgagggcaggagaagatatttcccagctaaagtcatgagtaaggaataaagg
ggtat

>chr3_164991906_164992106
agtctcttttcattgatttttttttttttttttttgtcttaggacatagtaagggtttgaatttc
agttccctgctatataaataatgttttaatgattcatttataatgtagattattgcttctgcttc
agttcatctcatttccttcttggagacatttcttggagatatctataattctgtatgtgcgacgt
ggtta

>chr3_166042706_166042906
gttaatttttgtatacggtgaaataaaagggtccacttcaatcttctgaatatatctagacactt
atctcagcaccatttattgaatatgcagtcattatatcattgtttttgtcagtcttgtcaaagat
cagacggttgtaggtgtgcagctttatttctgagctttgtattacgttattttagtctatgtgtc
ttttt

>chr3_182370306_182370506
cataagctgtccaaaataacccattacttgataattcataagaatatccaaaagtaatctgatgt
aaagtatctacccttcacccctacaaacattcctttctgccctgatttgttcctcccatttatct
tttctaactcaccaaagtatataattatccatatagagaatttgtgaccccaaattgtcttctga
gaaat

>chr3_188687506_188687706
gaatataactgaataatttcaacaaatactaagtaagaaaatcagaactatgcatgtcttctctc
agctatggttcttctatgtagatttcttgtttttttcttttcttttttttccatccctttacttt
gactccgtaaacgtctttaccagttaggtgggtttctttatgtagcacatggttggatttttttaa
aaaat

>chr2_2467193_2467393
aaagaactacaactagttaaataccatctgagggcaacaagataatgtaatcagtaaagcaacaa
agaaaatataaatataataattgtaacctcaagatgaattgcagaaatgaggactgctgtaccta
tgcatattttcttttttgcttgatagacatatagctatagacatagacataggtagatatgtatt

tatat

>chr2_21087119_21087319
cttttcaggtgtttttttttttctccaacttttatttcaaatttatggcagtacatgtgcagatt
tgttacatgggcatattgtgtgacattgaggtttggagtatggatcctgtcacccaggtaataag
catagcacccaataagtagttttttaactcagcccctctccgctatcctcaagtagtccacgatg
tgtat

>chr2_32043096_32043296
atctcatggcagttttgattcacaaatctctggtgatcaatgacattgagcaccttttcacatgc
ctcttgccatttgtgtgtcttcttttgagatgggtctatttaaatatattgtcctttttttaaat
caaattattagattttttgtacagttgtttgagctccttatatattctggttattaatcctttgt
cagat

>chr2_33978696_33978896
atacttggcaaaaagtattaatagaataaaggcagggtgtcctaatttttagcctgttgttattc
gtgttgtactatattacatcatgactaactactgaaaaattatttttgaaacatgagagagaact
acatcatgataattctacctccatccccccccaaaaagagcctatccaaaaacctgagacttattc
ctaac

>chr2_52533696_52533896
tgggattgacatagatgtgctcaatttcactaaccatttgatgatttttttttggtggctatctc
aatcaacaatcccatcagcagtacatgaggtttcacatttgcactcagtctctctaacatcagat
tttctgttgtctgccattctgattgccaaaacagccatatcattcttttaatttgcatttctctg
cttat

>chr2_59747096_59747296
acttgtttcattataaaaattagacacgtgtaaaaactaaacctaagcaactaagcaaatactta
aaatagttttagtgcagcctatgatgaaagcgcataaggaaattacctaccaacttaaggtcaaa
gaaaaattaatattgacctatggaagaattatagtctcagaattttaggatttgttgttctattt
aaata

>chr2_77641492_77641692
gagcgaaatacttagtactatgtgaacagtttatttacttgtgtcaatcatccttcccctgccct
ctccccagaccttgtcttgataatggtagtgatcttcttctgtgttcccaatgagccctgttcat
acttctgtttatctcagattattttgtgtctttaattatttgccaaccacagacactaccagcaa
cagaa

>chr2_80037492_80037692
tactactaggagaacagtatggggaaaccgccccatgattcatttatctcccccctgggtccctc
ccacaacatgagatgagatttgggtggggacacagccataccatatcactatccttttgccattg
aattgccttttcaccttttcttttcttttctttttttttttttgttatactttaagttttagggt
acatg

>chr2_96275873_96276073
cttgaatcctttcctgggcaaagtcaaggactctcaaaggttaagccccattttgaggctcagct
tccctgcattgagcttaactcactttgccaaagtgttgtcaattatcaagcctagtgatgagagc
ttacaaagcttacatttattgagaacttatcatgtggtacgcatgtgttttaaatgcttcaaata
taaaa

>chr2_116534930_116535130
ttttctaatagaattatgttttgcttaccatagtcctagtcaaaacaagccagccatggaaagaa
taacctttctttaaagacctcttccacacttcacacagagacatcttacacagagggatgtgtgt
gtgcacacgcacacacacacacatatactcacacacagtgattgagagaggttatgaaaaaggat
gattt

>chr2_124803330_124803530
tggctttatgtgttggggaagaaggtagaatgaagtaatattgacagaaaggccattcctccact
tccccccttggcaggcctctcacagagagtcttgctatgggtgccacattaggacccttaaaatt
cacattctttgtttaagaggtgttgtgaatcccctgaaattgtgcactcatcactgtgtgtatat
ctgca

>chr2_125731730_125731930

cacaatagagtttaatgaaatatgataaaatacagtaacttatctactaaaaactggtaatagct
acctgatatcaatgtataataaataggcagtcgttgcactaggcatatttaccgaaaacatttca
cttaatcctcaaagtaatcattaggtttgatgttattgttaacatattaaagatgataaaactgg
aattt

>chr2_125898330_125898530
atagctagcctttgcactttctgtgtggcccagagaatctcaagactttagcatggtttaagagg
ataatcaatatgagactccaattcttttcaaatgtaatattcaacatataggtacatacagacac
atttacaaatcagtaccttggactctatcagcaagagagagcagaaataattaataaaaacagat
ccatt

>chr2_138303530_138303730
attttttaaattaattcaagacatcctttattgggataggaaagtggggtaatattgtcttatta
ctgtcatctgtactgagaaaaaaaacataaatggattcagaaatgagaggatttctaattcatat
ctatctgagaaatataagaaagactctaaattcattgagatttttactctccagaacgattcaaa
tctag

>chr2_164430354_164430554
acttgcctttccagcttttttcctggcaaggctagagctgacatactgttctaatattctatatca
cttccaatctatctgcattgtgcagtttcttttacatggcacttgacttgttaatatttttcaaa
ttatcaaaatcaaaagttatgtatcagtatatttatacagaatttcctttttcttctttggcttt
ttttt

>chr2_167736754_167736954
aagttgaatgcatttttttgtgttctgcttgagcaaaaatagaacaacagatggccataaatttag
ggaagcttaatgaattggcaagttatgacataaagtaagttggaattgggtaacttaaatggcta
ttaacaaaggaggaaaaatcttcaaaaatgttatttttttttggaaaatagaacacattataatt
tagat

>chr2_194022955_194023155
cttcatattttaaggactcatgtgattatcttgggctcacctggacaattcaggatactgtcccc
acctcaaaggtccttaacattaatcacatctgcaaagtgtcttttgccatgtaaagtgttctcag
attctgggaattagggtatagacatctttggggggaaggaggcattattttgcctaccaaaagat
acgac

>chr2_200497355_200497555
acctccttccctacttgcacaaccaaggactgagagagagaagttttctgggtacaccactttct
tccaaaactatcattgattggaagaatctgtttttttcccaactttaaccacattttaaaggctt
atacaaagtatttccccagtttctgttttaagtgatgtcattccccaagaacctaatattcaaag
aaaaa

>chr2_212193155_212193355
catctggggatctcgggatatctactccttcaaagacaaaaaccacaaaatttaaatctaggatt
gttggttaccttagatttgagcaataaactaaatatgatgccaaaatttgttagtgatatatgag
gtaaacagggttgctgttacaaattctactcattctgtatcacacaagagaaacaaaatccaaca
tcaac

>chr2_212872555_212872755
tttccaagttaaaaaaatttaaacataattttaaaaagtcaagggtcttccttattcaagaaaat
tggtttaattctttaacacaaaatatttcaccatatggtcataaccagcactattggcaaagggc
cttacatatattggatagtcaggaaatatttgatgaatgaatgaacagatgcatgaataagtgga
taaca

>chr2_213088555_213088755
attctctcattgaaccatcagtgtttgaatttagtcaaagcctggaaaatgaatcctgagggaat
aaatacttcgcatagattaccttccagccaacacaacgattaacagacataaggcttgtagttac
ataaaacatctgtaatagattgcaaaatttctgcatttatccagtagtattcaaaattttacaaa
atttt

>chr2_214831355_214831555
attttccaaatcttctatggtatacatatatgtctataataattagagcaataatccttattaaa
aatagtgatgcctttttttagaccttacaccgtgtttaaacaaattttactaattgcttggtggct
gctagcatgatcatttttttccacagtcttctgactctacttttgctgactctgccctgatatttc

atctt

>chr1_30955813_30956013
ataactcacgtacctgagcagtcagaggccatttgcttcatggtgatgttgacttttactggtga
agaggcaaaatttcccctactttctgcactgcacagtaatggggcataaattcacggggcttgag
agacctcaaggcccaggaggatccctccaggggacacccagcatgggcgttctgtgggtgccctc
cagga

>chr1_37544813_37545013
ctgtggcacaggtggcctggcttgatggctaagtgaaaagccaggtggacgtgagactcctgggg
tctaaaggctaaagtgttagctctcactttgaactccagtgccggcacaggttcaagcccagagg
aggcatcacataaaaggaagcagaaaagtgtggacttcctgggctccaatcttggctgtgccaca
cacta

>chr1_39169813_39170013
agagagtagcatgggcaaaagtcctcagcagaaaaaaaaaaatcataattgtgttcaggatttag
aaagaagctgggtcttacaggagtagatggatggaagtgagaatactaggggattaagttacgga
gagaggcagagaacaatcatatttggtcttatggattatggtgagatatttatattcattttaaa
tgtga

>chr1_47502813_47503013
agctatacagaactttgtaaaaaaaaatctttactttttaaatattatacaattatgatgaaaaa
gcaaaatgcaaagtgttagggaaaatattaaatgttaaatttattcaaaacttaaaaccttttca
atttttttttttttttttttttgagatggagtctctatcactcaggctggagcgcagtggtgtga
tctca

>chr1_55959012_55959212
cataggataatggcctccagctccatccatgttgctgcagaggacatgctttcactctgttttat
ggctgtgtagtatgccatatgtgtatcacatttactttatctaatcaactgttggtggacactta
gattggttacatgactttgctattgtagccagtacttaaaagcttcttttcatcatctgctctat
ccggg

>chr1_61183812_61184012
agtggttaaataaagcatggtgtattctttgtcataaaagaggaggacaaagttctgtatgtcct
ttatccttatgaggtacaatcatcaagatataattttctattaaaaagtacatagctttgattga
aacagaaacacaatagcacataatataggactcctcttacatggcattcaagaccaggagaaact
aaaag

>chr1_66248012_66248212
ataaagtgggggaatggacatcctattaaacaaatgagatggaataattggcaagccacatatag
aaaaatgaaactggaaatgaggatttctcatcttacacaaaaatcaactcaagatggatcaaaga
cttaaatctaagacctgaaaccataaaaattctagaagataacattggaaaaacccttctagaca
ctggc

>chr1_74402012_74402212
tcttcagatcacagacaattcaactatagtaatagaagtttattacggatggaaggttagatgtg
atggtaggagtttgatgaaattattttctgattgattaaatttttcccaattaaatagaaaacaa
gaccttaatctgatattgtagataaaggaatatgttaacagttttagaaaagaggaaattatata
aaata

>chr1_89133812_89134012
ccacttaccttatagagactttccaagtatggtctctgatgaaaagaaggggggacctctatttaa
atgtacctttgtgaggtcaagtaagaagtctaacctctcctttcttgtaaagcttttgaaaaaaa
taaaagtaaactcactttttttggcaatgttctcaactccatggtcataggaaaatagtatattt
acctg

>chr1_103170012_103170212
atatatgtatatataaaagtgcatttattagggagaattggctcataaaattacaagtcaaattc
ctgtgataggtcatctgcaagctagggaaagagaaaagcctggagtggctcagtctgagtccaaa
agcctccaaaccaaggaagcaaacgatgtagccctcagtctgaagctgaaggcctgagagccctc
gggag

>chr1_104441277_104441477

ttagggcttaaatatttaatgattgggaagtataccatcattaaggaaaggaagtagctttgct
ccaagcacactctgtcctgtgatatatctcagcttagaagactgcagactaattagacacacacc
atatgcctccctatggtttcatttactttatgttggtttctattcttcagtcagaactggtagat
gtgtt

>chr1_107567077_107567277
cattaaataggggtgacaatcactaacaccaccaaaacaactaaactgttctgttgtgattagct
ctggaggacagcattgtatgaaattctgcagcctcaagcagttctgttgatggcttccattcctg
gcaaaaccactgaggatgactctctaatagatgttatatctgcacatatgctgttcattgccctt
ccttc

>chr1_164506376_164506576
ttaaaaacctttttaaagcagtagttctcaacggaggggattttgtcttctagaccaaggtctaca
gatatttttgattgtcaaaggcagaagggatgtgcgggtcctactggtatttagtgggtagagtc
cagcaacagtgcaaaatgttttacaacgtataggagaacttcccacaacaaaaaaattattcaacc
caaaa

>chr1_188082377_188082577
gttgacaatatactgagatttaactacattaatccaaaagttaagccaagtttcaggctttattt
tttagagaactcaggttaagacataatcatatggtaaacatgattttatctcttgcttctttcat
tcaacattttgttgtcacattcttgatttgattactattagatattttatatcaatatactataa
taaat

>chr1_188445177_188445377
atgattagtgatattgagcatttattcatatatttgttgacagcatgtatgtcttctttcgagac
atgtctgttcacgtcttttgcctgtttttaacgggattgtttgtttttgcctattaaattattt
aagtcaggtggtgtgatgcctacagctttgctctttttgcttaggattcctttggatatttgggt
tcttt

>chr1_190372577_190372777
atcaaaatgcctggcaacattatgtattgaaaaagaggtgccaaaatagcattatcttagataaa
ataaagatatggaagccaaatggttttattctatcttcactacgtagcacagaacagggaatgta
gagcttgtccctgagtagatgaatggtttaatgatcgcatgaggaatgagtcaacacatcagtgt
attag

>chr1_193824777_193824977
gaaccactcctttacatcattattctttttcatattattgtgagctcaaactttctgtgtttct
acctgcattgataaaggaaacttcatatgaacatacagaaatagggggtgggatatgtgaaaggga
ttagttataaatctcacactgggcaacacaagcttgaggtttttctaatttatttaaatctcgta
ttatg

>chr1_209434777_209434977
acagagcgagactccgtctcaaaaaaaaaaaaaaaaaaaaaagaaagagttttgataacttattga
ttatttctggtttaacggtaattttggagggaaaagagctggattattatgtctattttctaaaa
ttaactgaggtttttctttgtgttttaacgtacagtaaatgtctacaaataaccaaaagcactag
gaaaa

>chr1_216546377_216546577
tcccatgtttataaagaaaactgctcttcatactctcactaaaatccaatcacttgtttgaaaat
ggtcctgttgtttgaatatgaaacaagcacattatgatatgctaagtgtgatgctacatatacta
cacataaggggtatataaagcacagaccttttcttcaaggaatttacaatctgtaatagaaatgg
cgtat

>chr1_216615977_216616177
tccagcctgggtaacaagagtaaaaactccatcttaaaaaaaaaaaagaagaagaataaaaagaa
ggaggaggaggaggggggagaagaagaaatggggggtaagcaaagaacaaagaacactaatggacac
caagatgtaataaagctagattaagatagtgtgacactgatgaaaagaatagaaaaatgcaccaa
agaaa

>chr1_218064777_218064977
aataacttgtagataaagggagctcacttgcttactattagagaaaaaagttgcaaataagcagg
tagaggtgcaggggtgggggtgtgggaaggctggaataattcccattgtggtggcttaaatttgg
agatgtcagcttgatttcttttaacatatataccaatagataaatatgaagtaaatgtagatatg

tgttt

>chr1_223032977_223033177
tgggagataaatttgagactatcttcccaaaagcagatttaaaaagactaagagatagaaaataa
agtggagatgtaaaataaaagaaaatacaattagaggaaaactatgctctccaatgtttgactag
tagagggagttccagatagagtttgggatgaattagttctcctagttgactctgtaacctagact
gtaaa

>chr9_19032000_19032200
atacatgatcttaactcttacaatagtcctatgaggtacgtattgttggtacagatgaggcgact
atgacctggagtttagttaccaatctaagatcccacaattaatgacagcccaggaaaggtgacct
cagaaccctcttgcctgttgctcctctgctgcccactccctgccccagccaagcagaaacccagc
tgccc

>chr9_24439000_24439200
ctgaactgaatatccgtttgctgtccatcagcatgaagccttgtgtgttgtattgatgacacgaa
atggaaagcatctcaagctatgaggactgaaaaagaagccaggaaccatgaagttcttcaggttg
gagcacattgtttcttctcattagggcaaattatgaaaagagtaggaattaattttccagcattg
tgtat

>chr9_24981000_24981200
cactccttctgatagcttattgaataaataagtgacaaaaaataaaccagacatgtggattgtct
tttttcattatgtatttttagaattaagaggtggcataaggtaatacaagtggcgttgaagata
cgaaagatgagtttaagtcaggctctgccacttaggggccatgtagcttaagttagtcacctcac
ttctc

>chr9_25052800_25053000
cttctgacactcttcattttctttttatgggtttttaattaattcattaatttcacctctcagca
tgaaggataatgctcattcagagtttgacatttctccatttggcaaattgttgttgcagaggatg
ttgtcggtttgatgttgaatttgataaaatatcctatttaaatattcaaattttagattcataga
gcttt

>chr9_39063800_39064000
taactgagaagtatgtgaagagagaaagcattcattgcatattcatctgagcagctttctagaac
tgatccagtcagactagaaaaattctaccaagctcaaaaatcatcacacaaaaaacaaccactta
gagtctctggaaactgtcctaacagaaaatgtttttgaaatggcattatattctgttggacctaa
ttaaa

>chr9_39825000_39825200
tctcgatctcctgacctcgtgatccgcccacctcggcctcccaaagtgttgggattacaggcgtg
agcctccacaccccgccggcaaataaattttaaagccctacattcttgggcaaattttaccctta
aacaaaaattcagccaattgtactaatccaataaagagccattttagggagcctaaagttttttt
atgaa

>chr9_42027400_42027600
ccattttaacaagtgttggaaattttttatttagcagaactaacaaattgtttgcgaactattg
aaatagaactattctattatggcctgcagatatttttctcaattataattcactttcatactgta
aaagtatctttgctttgtgtatatctttttcatataaaaacttttaatttggcagggaatagtgg
ctcat

>chr9_46945780_46945980
tcaaacaattattgtagagtgtagcacacctcctcatgggccacactttatacctcacctttagg
ggcccgctgagatgagtctagttctagatccagaagcaaagaaagatggtagacatggttcctga
gaatattcagcattttcttgatggcagcttcctcaggggaggccactaaaatttcatcagtcact
aagag

>chr9_68172580_68172780
tggatttcaaaatctaaaaatatcataacataacttcagggaagagtaagaagtactggtgtcat
ttttttccttttagtagattagttgaagaggtaaattgaacatggactatgtgtcagactttttt
ttccaaattatattcttaaatgtatagtaaaacatatgctagaggcagtttttttaataacaaaca
atatt

>chr9_78741580_78741780

ttaagtgaaacatgtcagacacggagacaaaatactgcatgttctcacttaaaagtgagagctaa
gtaatgtgtgcacatggatgtcgagtgtgggatgatggacaatggaggatgagaagtgggaggtg
gtgagaggggattagacgatgagagattacctaaggggtacagcatactttatttgggtgatcga
ttccc

>chr9_89122380_89122580
ggttaaaccctggccagtacacagagacctgggaacaaccttcccaaaacaaccctttaacaaat
actttctgctttagagggaggaggaccgagaggaagggaaggacacacacagcattaagtggctg
tgggtatttgatcaaacagttatgcaactgattagagcatttgacagaaaattaatgaaagattt
tgttt

>chr9_91209180_91209380
actgtgtggatataattttgtctctttttcatttaatttcccttgcatatacttttagaagtgga
acgactaggtcagagatttaaataatttttaatttcttgatacacattgttaaattctcattttc
agatatgttgtcccaattctcaatttcaacaatagtagagtgagtggagtgggcttcctatgcca
tcctt

>chr9_102258779_102258979
acacaattagctttaaatgagaagacatttttaagtgtctcttgtaatctgtagacttatataca
aatactaggtaatgatgctgtctgtgataaggctcgtacaatttcaaactaacactttaacttga
attatgactttgtcagctctattcaagatgtaatccttgtcagcttctacagaaactcaaaggca
atgtt

>chr9_105086179_105086379
aagaatataccttaagcttgcaatagagaaatatatttccgcacaaggtgataaacccagcacgg
gctatgtgagctctttatctccttgtaagaaagtgatttaggtccaaggtccatttgtacatagc
caagcaggcgtagaaagactgtgcatatgagaatgccttttggaacaggtatcacaaaaaggaac
taaaa

>chr9_105386779_105386979
cacagttccacatggctggtgaggcctcacaatcatggtggaaagtgaaggggagcagaacaca
tcttacatggcagcagagcaagcaagagcttgtgaaggggaaatcccatttataaaatcatcata
tcttgtgagacttatttactactataagaacagtataggggaactgcccccataattcaattat
ctcca

>chr9_105778179_105778379
tcaacttctaattcccattaaggctcaggtctggaaattatatatatatctaattgtatatatat
aatatgtaagtatatatacatacatatgtatgtgtatatgtatcaattatctatctatatggatt
ttgatagataattggtaattcacagtatcaattatatatacagatttagatagataattgatatt
atcta

>chr9_107303179_107303379
aacactctcctgcccacataactttagaaatagcagcagatgttcaccaaactttatgaaatgt
tagagctccctagtttaaaatatatctaccaaagcaaagttcgttcaaagtaagagattaataat
tcaaatcatgattactaaaatgtaatgctagaagtgaaaaatgatgtagatattatgaatgaaat
aaaat

>chr9_108613979_108614179
aatatggcacccatagcacaagcatcaaaaggaaaaatatagtttcatcaaaatgaaaaaccttt
gtgcctcaaaagttttcaaaggacaccatcaataaaatgaaaagataatcagttgaatggaagaa
aaatatttgcaaatcatatttctgtcatttgtatttagaacatataaagagctgttacaattcaa
taata

>chr8_15800429_15800629
tctctactgttttgcaagtttgacattccacttctctttttcttagtgcttgttcttaactttt
aacatgcatatgtgatgggtcattgaataagcaggaatcagaaatcaactttatatttgtctttt
ctgccaattaaacattgatgagctctcttccctggtgctgccaaccgaggtgcaagccatctcct
acttg

>chr8_47635835_47636035
gattccacccctatgacctaatttcctaccaaaggcccaacctcctaatagcattacattaggaa
ttagatttcaacatatgaatttgggggatgcaaacattcagtccatagccaacagtaaaccaaag
atagctcaaccacagaccccagccagtggcctatggcttagctgactcacttatatttacacttc

aggga

>chr8_51760847_51761047
tcactattgttctctgcaaggaaaatgtacatttgcttagcctcattttctgaaccatctatctt
ctgtgttttgaattgttttcctgtacatgtacatcatactgtatccaatctttatttctttcttc
tgtattttgaagttttactgaaacactcatgtttagtatcaaagcatttcatatactacagggac
atttt

>chr8_65357646_65357846
tgtgcatgttagagcttgaaaagaccattaaaatgatctcagtcaactttttttcttttaaaccct
agtgacctttcaaagttacacagcaagtaataagatgagagctggaatccaaattctacaatct
acacacatgccttcttcattgcaggctactctcttggaaattcttccttccatcctccttttctc
cttct

>chr8_69789246_69789446
tcatttggagttcctgttaacaatttgttctcattttcttttgtacataactatgcctcttaatt
tatttcatagagatatccaacatgtctagtttttgttttgtggatcatattttgaattaatagct
ctctatgcattaggaatacaatggaataattcaggctgacatttttcttggcggacaatggcact
ctcta

>chr8_75885245_75885445
tctttcattagagttctaaatactcaacttttagatgcaataaatacaaagttaattggctgaat
tttatttgtaaacttattattctatttagaaaaggaataatttggctataaaaacataggaaaga
acatagaaaatatccatgagttttgagtcctccattcttttgaatttgttaagttatattggtta
aacta

>chr8_76915245_76915445
agagggactttgtctttacccaacattgagaaaacatctttgaatcatattttcccagaaaatat
tatttcaggtacaactaagcatatcttctagcataaaaactgtaagttatgaaatcctctcatgt
cttaatatagtaaacatcaattacttctaaaaacaattatacttttatttaaagtaccacaaggg
gcact

>chr8_118157219_118157419
ggggataatctctctatgtatctgttaaaaaagaaatacatagtttgattacttttggcaaccat
cccactaccatggggaagatcagcttgaggttgaagaatagctgagagccacagagaaatgaatc
tggagacctcgtcaaaaagtatctgaaacctggcccctctagatgttttcagttgcagaaccaat
aattc

>chr8_129767418_129767618
tagaatagaatagcaggtagatttctaaggtttttgacttcaactcctgcctcccagacaaaaac
cctgaacccacctagagcataggggaatttgttgccctgaaaggaaggatataaacttggcttgc
tttaccacctgctgactgtagagctctagggctttgagtgaacacagatggtagtcaggtagtgg
ttaca

>chr8_136843618_136843818
tgccaagtgcatattgagtgtctgctatgtgccaggcattgtactggttaaggcaatgtaatgag
agagacacgatgtctcccagaagaaagctcacaaattaacgggtgagaaaaatatgtaagcagaa
aactactgtgattgtgtaataaaaaaaaaaggttagggttatgcctgagggccatgggaacacag
aggaa

>chrx_10705600_10705800
atgcaaaatgggaatgcatgagacaggtgggaaagagagggagaaagaagaaagtgaaaggggt
gggggagaggacaaagaaagaaagcagaggagagaaggaaagagaagagagaagaaggggggata
gagtggaaggaagggtgtcagagaggagggaggaaagtgaaggctggagaagaaaaaggagggaa
agggt

>chrx_26841679_26841879
tctgaaggggttaagtcaaagatatatggattcactagacctagagtcaagaatttttaagaaaa
cttctctaagttttggagtgtgaatataataggctaggttcctcctagaatatatactggaataa
ggatttgtatgaaagtagtttatttggaacataatcctgaaaacacccgatgctgaggagagata
tgaca

>chrx_29179079_29179279

caggagaatcgcttgagcccgggaggcggaggttgcagtgagcagagaagattgtaccactgccc
tccagcctgggtgcaaagcgagactctgtctcaaaaaaaaaaaaaaagtgtgataactgttttgat
acagcaagtggatggatggaagaagctctatgagtatacagcagggatacatggcctgggaggaa
aaagg

>chrx_40761256_40761456
aagtaaaaggatggaaaaagatgtataatacaagcattaatcacgacgaaataggaaaagctcta
ttatatcagataaagtagactgcaaagaaaagaaagttaccacagacagaaaaggacattatata
atgataaatgggtcagtacaccaaaaggacatagcactcctaaaatgtgtttgcactaagtaaca
gagca

>chrx_51857460_51857660
agcaagactaataaagaaaaaaagagagaagaatcaaatagatgcaataaaaaatgctacagggg
atatcaccaccgatcccacagaaacacaaactactatcacagaatactataaacacctctacaca
aataaactagaaaatctagaagagatggataaattcctggacacatacaccctcccaagactaaa
ccagg

>chrx_57739475_57739675
tatcatgaaaatggccatactgcccaaggtaaattatagattcaatgccatccccatcaagctac
caatgcctttcttcacagaattggagaaaactactttaaagttcatatggaaccaaaaaggagcc
cacattgccaagtcaatcctaagccaaaagaacaaagctggaggcatcatgctacctgacttcaa
actat

>chrx_74152875_74153075
ataaatctctattaaagaacttatctttgtgtattatatgtatttgggcatgtgtctgctcccac
attggactgagaatttctagctcttggagggtagggaccttgctgccttctctttatcttttcat
tctttaggtactgccaggcacttacatttgaagaataaaatgtcttaataagtgaatgagctgga
gattt

>chrx_78569744_78569944
atgacacagaatagccaaagctatcctaggcaaaaagaacaaaactggaggaatcatggtaactg
actctaaattatactacagagctatctatagtaaccaaaacagtatggtactggcatgagaaaca
gacacatataccaattgaacagaggaaataatccagagataagtccatacatctacagtaaactc
atttt

>chrx_78755344_78755544
cacaatagcaaagacttggaactaacccaaatgtccatcaatgatagactggattaagaaaatgc
agcacatatacaccatggaatactatgcagccataaaaaatgatgagttcatgtcctttgtaggg
acatggatgaagctggaaaccatcattctcggcaaactatcacaaggacagaaaaccaaacacca
catgt

>chrx_86297744_86297944
cgtggaaacattagcctgctgttaacaaaagaggggacaatgtaaggtggctaagaataaattat
gtccactatgtgggacagtcagaaaagagcattaatattattgagtcagtggcagcgataaagca
aaatttttctattcggttgccaggaaatattttagttcacaaaagattcctgtatctgcttttga
tattt

>chrx_89881344_89881544
cagcagcaatcaccaaagcagtatataaatgtcccctgattcattttcctcttctaaatttttca
ggcaacacgtaacagtcagtatcaaatttacttatataactttagtgtaaaaggggtcttgatat
agagtcattagttgtctagcctcagcatactagaaggaaacttaaatgggcatttagaccaaatt
ataca

>chrx_92465944_92466144
aaaagaaagaagtttacattttaacaagaagggagaaagacaaatctagaacaaatgatcacaa
ttgccccctgatttataaatagcctaaaatgtgtttaaagtactgacatgtccatgggagtacta
tgcttgttaaatgaagggaggatcatgaattattttaaccttacttgaagaatttctgaatgact
ccatt

>chrx_94767744_94767944
aactacactatgctctatgaacattttgtgttattatgtgtgcccttagacatcctgaattgcaa
tcaaaatggtaaccaaataaagttttctgtttggtgctttccagttgaaaaaaattacaaaatta
agaaaataacccatagtgtgttttgcacagtccagttttgctgataccatcagcaaaatctttag

aatag

>chrx_95212544_95212744
tgtgctgtagcctatacagccaaaaaggtggagcttcccaagccttgggaggccaccccttgcct
cagtgttgcccggatgtgatacatggagtcaaaggagaatatttcagagctttaagatttaatga
ctgtcctgctggcttttgtacttgcatggggcctgtgatcatcttttttttggccaatttctccca
tttgg

>chrx_96843344_96843544
atgcttagtgctaagacttccctttccaccccacactgcctgcactaggaagggaaaattaaccc
ggaatgcatgctttactaaacatcggtcagtaccttttaccatcaaaggacagactttttttttt
tttgaagtctcatgtgatttttatcattgaagtaaaactctctccaggtgatgtaatgacatacc
tccta

>chrx_103146544_103146744
attaggtatatctcccaatgctatccctccccccctccccctccccaccacagtccccagagtgt
gatattccccttcctgtgtccatgtgatctcattgttcaattcccacctatgagtgagaatatgc
ggtgtttggtttttttgttcttgcgatagtttactgagaatgatggtttccaatttcatccatctc
cctac

>chrx_114309944_114310144
cacccagtcttccacttgtgtatggccatctagatctccagaaatacatcagaacttttcaaaga
cacctttggacatctcattctccaaattttatgttttggggcctactgtttgtttaacccaact
gttgctgatgcctcaagaaactgtgatgtcaaataattgcttctaatttttaatggaaaatatc
ctgga

>chrx_138335134_138335334
cctttcaaatgagaggacctgtggtccagaaaaggcagactgaacctgcattgcatttcttcctc
tgtcctcccaaaagctggtgccaaatgtgggcatagtcatgggaaatgcacagtagtgtggggt
aaagacagccttagctactgagccagaggaccaaaaaagggaggaacagggaaccaaaagtacct
gtaag

>chrx_139282734_139282934
tcttgctttttcacttaataatacaccatggaaaccacctcattcctttaagtgctggcataaa
aggccatggtttagatatgcagttcactcagccatacccttctcacaaacatgccttccatttc
cagtgcttttatttctgtggagtagattcccatgagtggcattgctaggacgaaatgtagctata
tattt

>chrx_146723908_146724108
ataccagattatgtagatactccattctcaaggttttagaacatttcattccttcagtgtgagct
gtgtatagtgacttccttcctaagagtataatatacaattttaaaaactaaagatgagtagaact
acaaggagaaacctaccaaacactactctaatcccggtgatcaaggcaacatcagtagtaataca
tcatg

>chrx_150636942_150637142
ccttgtttttgaaagaaattttcactggatacagaattataggatgacagttatgtttttttactc
aagactttatgtttaaagcatcactgatgtcttgtttccaatgagaaatctgttgcctttgtttt
tctttattgtattttcttttttttttttcctttattttcttcctctgtacataacatgtcttttt
tgtcc

>chr13_19369400_19369600
gcagtgcagaaaaaatatatgggcttggaggacccacacaggaggttaccaacctccagacccca
gattcatagacgcatcagcagcttgcactctcagtatggaaaagctacaggcactcaacaccagc
ccagcccatgagagcaaccatggggctaaagccttcaaagccacaggtgcactgccctggtatag
aggtt

>chr13_19603200_19603400
ggtcaggctggtatcaaactcccaacctcaggtgatccacctgccttggcctctcaaagtgctgg
gatcataggcatacagtgacgagggtagttttttttaagaaactaaaaatactactcgggatagga
atgagagctgctgtgcccagccagcaggctccacttctaattctagttcacttgctatttccact
acatc

>chr13_44153400_44153600

ggttttaggagtaagttatctgtacccagaggtgttcaaacagaaataattatccacttgatgga
gatattagaaagaagatttatgtatcagaaatccaattacattagataacttttaacagctccct
ttctactcttgcagtgctaagctcccattgtgaactgaagctgagaagtacacagtacacttccc
agatg

>chr13_51140599_51140799
cctcagaggtctctgaggctctgatttttttttttagcctcacaactctttgtgttttattttaga
caaattctattcttaggtctttttttgtacaatatagaatctgacgttcatcccattcagtaaaat
ttttacttcaaatattatattttttttatccctggaattccactgggttcatctaaatttcttcca
tttct

>chr13_63360399_63360599
aacagattgaaagtaatcaacactttgcagaataatccccgctttttcactttacttattcatgg
tagacagtgaggttctaagaaaaacttagaaacttagatagttagaggttaataacacttgctct
gtgctgatcctggctaaaattgtgacttgggacagttattacttgtacttatctatcaaattttg
gttcc

>chr13_72034399_72034599
ccacatcttttcctgtgttgatttataaccactcaagcattaatttcataacattttcactgtgt
agataaatgttatcagtgtttttggaattagtcagttgagactcttgtggtgagtgtgaaattac
gagatgaggaaatgtagaaactaagctaaatactattcccagctcttgatcaaaatgagatttgt
tctat

>chr13_72496599_72496799
gtagcatctggatagcattcacaggctagcagttcattgtgtttcctactcacatgttgttcctg
atgaattattttaaagtatgactaaattattcattatacaagatcacaattatttatgcaactg
aggtttaccactaacattccaatatggattgttttaactatgggacttctataaagcttctaaga
aatag

>chr13_75010799_75010999
caaccaaggtaacatgaacactgagagatactggagaatttttttattattcaaaaatataatga
atacacgaatcatttttttgaagatacaagtgatagaactacagactactggaaatgaataagact
ttctctaaaagaaaatgaaaatgtcgaaacaaatcgaataatgtgaatatatctttaagaattta
gatga

>chr13_83420399_83420599
ccagtgcactccagcctggggtacaggagtgaaaccctgtctcaaaaaaaaagaaaaaaagaaag
aaagaaaaaaagagagagagagagagaaggaaggaaagaaggaagaaacaaaggtaggaaggaagga
aggaaggaaggaaggaaaagaagggaagggaaaggaagggagggagggaggaaggaaggaaagaa
gggag

>chr13_104237999_104238199
aaagataaattaggaggagatctcctttctaggatctgccaatcaacatagaacttatatagagt
gcactaagtatggtgatgctatagggtaaggacaaggccaccaactggaagtgggtgaatataag
tgaccagctttgtagaagatactaaattctgggaagcatgtaggaaatgatatagaggatagttt
ttcag

>chr13_107290399_107290599
ttcccatgtctgtgtactgagcagcaaaccaaattcaccatgctatccaattttctccatctttc
tccctttcccaaatcccttaataaggcttgcaattttaacttttcagtgtatgcagcacacctag
gatgtttgaaaattagaattttgtgtgaaagcatttatagtcactttctttttgaattatttcat
ccttc

>chr12_4899139_4899339
agcattttgagaggccgaggtgggagaatcacttgagcccagagtgcaagaccagcctgggcaac
acgacgaaactccgcctctacaaaaaaatgcaaaaattagccagatgtgttgcgcagtcctgtaa
tcccagctactcaggaggattgcctgagcctgggaggctgaggctgcagtgagctgagattgcac
cactg

>chr12_7668133_7668333
ataccaattcaaggttagacaggaaatggctggacagatgtccttgcagaagtatttttgtgtgt
gtatggatgtgatggccttgtgtttttttgcagtttttttgtgataatttttgttatcaggcattta
tgcatgagaaccctctcttcacagccttcccagctctatttgtcagggtttgttctgttttggtt

tggtt

>chr12_9727733_9727933
agtttttcaacaccagaatccttgcaatataacctgacagggctccacttgaacttgaaatacaa
gccacactaagcttccaggtctttttgttattgtcattgttgttcatgctaagactgactgttcc
aagctactgctgtggactttgacttaaaaagaggagtggggaatattctgaaatgctaacttaaa
aattt

>chr12_33496533_33496733
aaactgtcttttttttatttgaagaaacagtagaatactgtcttttacttattccacatacaac
tatgttataggaataaaggaaaatatacaattattttaagtattttccttccatttccactttca
ttgcatccctgaactccttctccctttttgctctgggtcacagtcattttttttgtgtctataagac
cctca

>chr12_40316933_40317133
tgtcctcaaaaattggaagaaaaaaatgaaggaagaagaattgaaaacagtgattcaagcaaata
cttgtatcctaatgatcattgcagaattatttacagtaaataaaagtggaaacaacacaagtgtc
caacaaatgaatgaataaacaaaatgtggtaatccatgcaatgtaatactattcacctatgaaaa
ggaac

>chr12_72129533_72129733
atttttgaaagacattttcactggtagataattactggttgaatattttttcttttagcacttta
aagataccatttgattgttttctggtctgcataacttctcatgagaaaattgttacttttaaaa
aatctttgttcctttggctacttttctcttccactggttttcaacaatttgaaactgatttcctt
ttatg

>chr12_73641933_73642133
gctcaagctgtaccttggttcatttagccatggctagagtggctgggacacaggacaccaacct
aggttgcacgcagcagggggggccctgggcccagcccatgaaaccatttttttttctcctaggcctc
caggcctgtgattggaggggctgccacaaaggtctctgacatgccctggagacttttttttaaat
cttct

>chr12_74290333_74290533
gggaaggctggattatcagtccctacaggacataagccatgatagataaattgtggtagacctgc
accatgctgccactagagcagagcatgagagaaactcataggagttccaggagtgtggagagata
tgtggaaataagtactcaagccccacctctcatcccaagaatgctgactgctgaagactactggc
tgaac

>chr12_74577533_74577733
ttggacagggaaagaagtattctgttttgtacagtgtattttaggatgtttttgtttccaggtga
atatgttacttggaagagtgatatatgatcctgaataatagaggaaaatgtgaagtcatcaactt
ataaatgtaatttttaaaatgcattttaaaagagattagagaaaaataagtgtgagtattgagct
ctaag

>chr12_88848669_88848869
caggaagagctctctctttcttcttcttctttttttttttttttttttttttttgagatggagtc
ttgctccatcaccaggctggggtgcagtggcacaatctcggctcactgcaacctctgttacccag
gttcaagcaattctcctgcttcagcctcctgagtagctgggtttacaggtgtgtgccaccacgcc
cagcc

>chr12_107998070_107998270
aaaattagctgggcatgctgatgcatgcctgtaatcccagctactcaggtggctgaggcatgaga
gtcacttgaacctgggaggcagaggctgcagtgagctgagatcgcaccactgcactccagcatgg
acaacagagagaaaaaaaaataatgagccaaggtcctaaacatgtcatttctcaaaagaagacat
ataaa

>chr12_117008217_117008417
gagcttgtttaaagtaggctaggtgatatggttgggctgtgtctccacccaaatcttgtcttgaa
ttgtagctcccatcattcccatgtgttgtgggggaactcaatgggaggtaattgaatcatggggg
cagtttcccccatactgttctggtggtagtgagtaagtctcacgagatctgatggttttataagg
ggttc

>chr12_118339417_118339617

acttttaaaaattaaaacgcaaaaaagaaaaaaaattatgacaaacaaagtattcagttttttta
aacgtggagtgtggttggttggctgttttaagaccttacattaccatgcaataagaaccgtcatt
tccagtcagcccattattcctagacctgcgaggccatcacatatgccgctgggtgggtttacgag
ggttc

>chr12_119438217_119438417
tcactggaaaagaaaggactcagaccaaggacccgtgaactttccaatacatttgaccccagtga
accctgtgtttctcgtgactggccctgtactaaagacctcagtgttcttggtctccatttaggca
aggtcagaccagagatttagtcagaattggtttaaactatttccaggtggaagaggcatctctgg
gaaaa

>chr12_119460617_119460817
atagaagtccagaggcagagttggttaattgagcaactcaactagacttcagatttggctctgcc
attctcagggtgttaatgatgtttcccctcaccactgcaagagggctgcagtgtctccagccatt
gcatgaagacacaatgagcagagcatctctttttgtgtctttttgagaacaaacaaagccctctc
ttgaa

>chr12_130436847_130437047
tgcagacaggaataatggcagaggtgatgttggggatgtggtgtgagaatgaggcatacatccca
gccgctgtgcagatggagtcctgatgcagtctgcctgcgggttgctggtgtgcaccctctgatcc
acagcagaatggagatgagaccacattttaaactttctggaccggagccacacaatctgcttttg
agtgt

>chr12_131109247_131109447
atttaattcactttaactaggattatatttgggtttctttgagacggagtcttgcgctgttgccc
agaatggagtgtcatggcactatctcagcttactgcaacctctgcttcccgggttcaagcaattc
tcctgcctcagcctcccaagtagttgggattacaggcacacgaccccacgcccagctaatttttg
tattt

>chr11_5993424_5993624
ctgggcacacatgcatgaagttctcagccacagaacacatgggtcccttgtatgcaataatatga
gattgctgagactgcagaagaggcttccagcaggaaactaggattcctaaatcttcatgcattcg
gtatcagagcccattgactttttcttctttttttcatggggattaatataagcatgaggagaaagt
ctcct

>chr11_14963824_14964024
ctttcattcattctgcaagaacagtttagctttggggaagggctattatttaaactataagctaa
attcctccccaaggctagtttgacctatgaccaggaatggataaggacaatttagaggttagagg
caagatggagttggttaggtctgatgtctttcactgttaggtctgatgtctttcactgtcataat
ttcct

>chr11_29928224_29928424
cacataaagaagtctagaccagctagaatcatgacaggatcaagtgtacatgtaataatactaac
tttaaatgtaaatgggataaatgctccaattaaaagacacagaatggcaagccagataaagagcc
aagacttatcagtatgctgtcttcaagagacccatctcacatgcaaaaacacacatatgctcaaa
atata

>chr11_32244224_32244424
gtctctaaagaaaaaaaattatattaatataatggccatcattctttgattgtcaggcaccgtgt
taagagattattataaacattattccagctagacctcacaaggaaagactcattcattcctttaa
acaaatgaggaaactgaggcagagaaaggctaaataccttggttcacactagttatactagttat
agagc

>chr11_37247624_37247824
tggacaacagtaaactatggagacaaagtttgtgtcttactgcattttgtatatgtaatacctaa
cttagaatgtgcttgaatacgtgcatgcataaatgaatgaatggataaacaaatgaaatacaatt
caattacaatacaatgtgtcgcctggatcttattctgcatttattcttaataaatgcatcagatg
atgat

>chr11_41321024_41321224
gattttctggtagtttctatatccagttatcaatctatatcctatagtcaaaaagtcaccagttt
gtttgatctgttataaatacatgaatgggaaaataggatcactaagtttaggagagagggtattt
tgtaaaataaagtgaatttatgatccatcatgaatttagaaatataaaagcattctaaattatgc

agtcg

>chr11_48600024_48600224
actgtaattctgcttgcttatcatcaaagtacatttggccactggtaaaattactattcatataa
ttaaatcattaatttataaggcatacaattttattgtgtgcctccaaaacgttttcctgaattac
aggtatcttatcccttttttttcctggagtatgtctgtaaatcttttcaagttgacaaagatctgc
atgat

>chr11_79586352_79586552
aggaatattagtgaggagtaatactttgaaaggaatctctttttctgaacagtagatctcaacag
tgggctcaaactattcagcaaaccatgctgtaaaacagatgtgctgtcattcagactttgttgct
gtatttatagagcacaggcagaatagagttagcaaaattcttacaggttcttggatttctggaac
ggtaa

>chr11_79731552_79731752
taggctgatcagcttaattgttatttcctctgcttgaatttctgtgatttaacttaatcttccat
ccttgcccttcaaacctatgtgctcttgatttaatcagcaggatcaaaactattgcacttccctt
tgcccctgagccactaacagaaaaatgattgacagttaatcatgtctgaagaggatctctttctc
ccttt

>chr11_81715752_81715952
tgtggtacaagggaaacatttctgggaaggaagtcaatatttcaaactggtttttcataaatata
gtctgtgcctgggctttctgataaatcatgctttctgccacattagtcatgcacctatttattgc
acgtaatggtggtggtgaaagctgaattactctaaatgtgataaagatgatactggggagatgat
ggggt

>chr11_91723752_91723952
caaaaactttggattggacttcatgccaagaaaccactacaaaagaatcatggacagtgcattag
aacactgcctcagagaagcccagtacctaatattgccattcttgagtcatcccttttttattattt
ttcacatgaaattcatcagcaaatcctgctagctctgcataaaaagatatccacaatccagctac
ttctc

>chr11_91926752_91926952
acattatttgaaagtaaaatatttaatatatacttaaataaattttattaaaagtaaaggcaaca
agtatgcaagacacatcacttactaattattttattatattttgctatcatctatgttatgtctc
ttaaatctgtagggcggaattttttttttttttttctcagacggagtctggctctgtcatcaggc
tggag

>chr11_92252752_92252952
ccacagactgggggggaagttagcaaatcacttatctgataaagactcttatccagaatatataa
agaactcttgaaattcaagaaaacaacccaaatttaaaaataggcaaaacatttgaacacttaac
ctacgataacttctttatcttagagaagatattcaggtgaaaaataagcacttttaaagatgctt
aacat

>chr11_102503990_102504190
ggggtgggggagaatagagagttgccatttaatgggaatataaagatcacatatgtgctgatgaa
accttaatttttatcgtcagctcagattttgttttaagatatattcaaaggtttcctgcaatctc
cgcttgaatctctccccacagggactatgaagtggccacctactatactgtctgcttagagtagc
cacac

>chr11_103374590_103374790
ctagaaaacttagaagaaatggataaattcctggacacatacaccctcctaaaactaaaccagga
agaaatcaaatccctgaatagaccaataacaagttcttaaattaatgcaataattaatagcctac
caaccaaaaaaagtccaggaccacacagattcacagctgaattctaccagaggtacaaaaaggag
ctggc

>chr11_115705590_115705790
ggctaagataaaaaaagagtgacagcatcacatactgacaaagatgtagagaaactggatcatgta
tacattgctgatgggaatgtaaaaggtagagtcactctggaaaataactgcatttcctttaaaat
gaaaaatgaacttaatgtgtgacccaactattgcacacttaggcatttatcacaaaatatgattt
tcaca

>chr11_127299990_127300190

tttcctatttctcaatagcagtttgtcaaatatcacctctcctgatacgtgtgctaccttcaaag
agcttttcctttctttcccatttcatcttctaatgttatacttctcatatactgtaggcagaga
ggttaggtcattaatcttattaaatgtgttggccatagtaataggtaaggcaaatcagtgagaga
ctaaa

>chr11_131078590_131078790
ttcagcatccccaggtgcaaggctgtattcctcaaaacaggctgtggggaacaacatagacaagt
atacccaaagcagagtgccggagagagggatagcagatgacaagtaccaagggccagtgctgggc
agagatgtctgcagatggctgcagaaatagaaaaaataaatatcgggacaatcatttgactctat
ggaag

>chr10_2390200_2390400
gagaagatgtttgcaacatgcactagaaacaaaaagctgattgctaggtagtcagccatttccta
tcatattcatctgctgttttccttgatgacactagtaaagtgcatggccaaagccaccttgccaa
tggcttccagatagattcaggcaatagacagaacgagtgtgagtttggaagagaagaggaaagga
ggaat

>chr10_18347594_18347794
cccagagaggtatactttaaaaaatattccaagtctgggtgcagtggctcacccacccagacctg
atcatgaggtcaggagttcgagaccagcctgaccaacgtggtgaaaccccgtctctactaaaaat
acaaaaattagccaagtgtggtggtgggcgcctgtagttccaactactcaggaggctgaggcagg
agaat

>chr10_35539594_35539794
agcctcttcctgacattctgccatttccatgaccatttggtgagaagtactgcacttagcttttt
cacacttctcaaagtaacaggtttatgtgtattcgtaaggttccttttcagtaaatgaaaataca
tctgggaagattcagggcttcgcattcatgttcacatctaacacctgattgagacaattgctgtg
atctt

>chr10_38903794_38903994
ttcttcctgataccccctgaaacttgcacagtacatgctataaaatgcagattcaatgagggatct
ttgcaatacaattttgagaaagaatcatgaaccaggatttagcgtctcttcccatcaaaccccag
gcccatagagcaattgcctttacctgtgatgcacacctcctacctgtcctccccgcagcccggca
tctct

>chr10_59026394_59026594
tttgaaatagcataaaaagaaagttgcatgttctcactaatacgtgggagctaaaaaaaattgat
ctcatggaagtagagatcaatttggttagaactgatgtctcgttggtgtctcttatgtgatctca
tgtgatcaacattttttagctctacaccaatgaatagaatgatggttaccagatggtgggaacggt
agtgg

>chr10_67406394_67406594
gcatgatttctagtcttttaaatttttaaagtttctccaaaaatagtatgtcttggttaatgtt
aaatgtatacttgagaagaatgtgtattttattgcacagtggtgtgtactataaatgtcagtgaa
caaaaaacaataaatttgtaaaaatcacttttagacgtatcttttttttttttttttttttttt
agaca

>chr10_68469794_68469994
aaggccaattgtagcgacatagaatgttgaaactgaaagggttcttagaagccatctggcaaaca
ctctcataaacagatgtgagaattgagatccagaaacatgaaaagatgtgtttagggcaacagca
gtcactggcaaatccagtgtggaagatggtgtcttcacacattccacttctttttctgcaggcat
ctcaa

>chr10_68483994_68484194
ccctattcatgcttttttaatcaattgatacttgtcctcctttctcacattgattaaataccagga
agtcctcacaagatagtgagatgtgaagttcccattgcagttaacaaatggtcctaagacccttа
gaaaacacatggttgtgcatctttatgcccacagggaatacgccattaatctgaactaataaaga
ggaac

>chr10_85793820_85794020
ttctttttatgcttctgattcacttgaatctgtgtattaagtcatttcaatcaattctggaaata
tatcacatattatgtcttcaaatatcacttctgcctgattttctctgcttttgggggggattctga
tctctatctcctccatgtcccttactctttcttctgaattttctatcttattttttcttctatgt

tatat

>chr10_89758420_89758620
aaaactgcaggtcaatatccctgatgaacttcaatgcaaaaatattcaataaagtactggcagac
tgaatctagcagctcatcaaaaggcttatccactacaatccagttggcttcatacccagatgcaa
ggctggttcaatatataaaaatcaataaatgtaattcatcacataaacagcactaaagacaaaaa
ccaca

>chr10_108355610_108355810
ctactccatgaaacaatcctgtatcattttgacaggatataactcatacctgctttaaatttcct
atacagctttagttgctaataataataacagtaaaaataaatattaaagctatgtttcagacact
atcctaagtgcattacctgaattaatcttcacacaatcccactggcagagatgctattaccatct
ctgag

>chr10_133213610_133213810
ttttttttgaaacctttaaaaaattgttttgttttgctgctgagagcaataaattgtttggaaag
tattaatgcaaatatgcagactcaccatatttttgctgtgtgagttctaatccctgctcttacac
atggataactgagggggagccagtaacttattttcttggagtctcagtttctttttcaataaaat
aggag

>chr17_12380475_12380675
ggaaggaaccaccattatatatgttggcatctatttgaacagactgtttccttgttttcatacac
acacacacacacacgcacacacttatatgtatatgcatatgaatatgtaaaacataatcatatat
accattcagtgatctttttttaacatttgacactagataaatttttatgtaaataaatcttgatc
agtat

>chr17_12735675_12735875
gtgccagctgatccactgagtgcagggtctgaaaaataccttaaacaccaatcttaggttttata
atactagtattatctgtaggagcaactggggagagtagtgatcctgtggcctcttagcttcatga
ctcctgagccataacttctaaccttctggctaatttgttagttttacaaaggcagtctggtccct
aggca

>chr17_49391001_49391201
tatatgatcaagttgtctataattaaagggaaaatataatggcctttctagagattgggtttgat
attaaaaaaaaaactcttatacactaaaaaattggttagaacaataaattttttttttttttttt
tgagacagagtctcactcttttgcccaggctggagtgtagtggcacaatcttggctcactgaaac
ctccg

>chr17_69483405_69483605
agatagccaatttcatttaaatctttaaggaagtaataagaatcattaattttattaaatctgga
tttggcatagatgtatttttaaataatctatgtgataaaatgggaggcacttataaagtactctg
actacctaaagagagggaaaaatacagctttacaattctctgagttgtcagcttaactactcact
tttta

>chr16_31846099_31846299
atatcaaaatgacaacgtaacattacaccccaaagaactaaaaaaaaaattagcagaaggaaatg
acaaagatcaaaacagaaacaaaccaaatagagaatggaaaagctatagaaaaatgaacaaaact
gggttgtttttaaaaaactaaaatcaacaaacacttaccaagactaataaagaaaaaatagaca
agaca

>chr16_51713699_51713899
ccaagaggtcatttggtttgctcaaggtctcttcattcatgaaaaataagaccacaccacacata
ttgtgttttgacttgattttctcacatatgaataggtcttgggaatatttctaagctagaacata
cctcactcagtattttaaatagttgcaagttcttctgtagaatggatgaaacataatttgttcaa
cttgt

>chr16_60146699_60146899
atgttctagcttggtttgtagccccaccaggggcaagtgtgaggttagtgagagttctagacttc
atgcaaagcaatgttgtcaggtagccattccacatgatagtcgtgtacagtgataaggagggata
ttacacataaaagcaagacaacattcagctgttaaatttattcctgctgatttttgttcttcact
acagc

>chr15_36348108_36348308

aagtatttttcaaaattaacaaaagacattaagctaattccattaagcacaattccattaagcta
aagattcaagaagagatataaccccgagaaggataaaatacataggaaaacattcctatcaatgt
cttagtagaactagtgaaaagcaaagacaaagacttaaaagcagttaaagggggttgggggaa
gcaca

>chr15_36385308_36385508
atggatcttcttcaagctcctcaaatttgatatgtctaaacaaaactggaaatcttcaaacatat
ttctccctttccctttgtaagtcaatcatactagcactctcagtggctaaaggctagaaaccatg
tggtcatcagtgatggcttcctttcagtcccttatttggtcagtcacctagtgccgtcacttcta
cctcc

>chr15_36549508_36549708
aagaactcctcagcaaatgtaaaagaagagaaattataacaaactgtctctcagaccacagtgca
atcaaactagaactcaggattaagaatctcactcaaaacctctcaactacatggaaactgaacaa
cctgctcctgaatgactactgggtgcataatgaaatgaaggcagaaataaagatgttctttgaaa
ccaat

>chr15_73498947_73499147
ttgttttgtttttttaattactcatgtattttctcatgtcctttactctatggaatatctctggt
cttgcttatctgctcatttgaaaactttttcctctgccagatttagctgtagattttctctatt
tttttgttttcaccaaaccactttctatcgtttaagaattcttcaacattttaattttaagaatg
gcatt

>chr15_87303796_87303996
ggacctatgtaaaaaccaaatgatcgataacttggccagccagctgctgccacaagaggtaaaga
aactgttgcctcctgttttcccctctgcaaaaagaaataacttggttcccacctagatttctttc
ctttggcagtgtgaaagacagctctttcttttcttcattatcataacttcctgacagataaggaa
agtga

>chr15_91638396_91638596
tctcaaccaagaacgctaaaagtaaatcaccagatagaaatgactttagacccttatcagcctgg
agacagcagcagaacatctacataacatgcttaactagcatttatctaccattagtttcctgtat
atttggcttcctacaatttcctaaattgtaggagactcaaagtccttttcttttgtcttgtcact
tctct

>chr15_92777996_92778196
ataattaaagacaaattctgggaccttgctagagatttagatatctaaatacaagaagctcaaat
aactcatgacagattcattgcaaaaaggtaatcaccaaggcatatagtcattggctatctaaact
caaaatgaaagaaagaattcaaagagctgtgagacaaaagcatcgggtaacctataaaggaaaat
gtatc

>chr14_28828049_28828249
tattgattggttccttttttatatattacaataatatgttaggaaatagatctccaacattttata
ttgctgggtaaattgcaagctgccacaaacagaaaaccttgtaatacaaatttcaagattctgcg
aatatattgtatttaggtagattttaaaaacaaggttgcaggggttaggcaggataaaatggggt
tctaa

>chr14_29722849_29723049
atcattgatggctgggtgcggtggctcacacctgtaatcccagcactttgggaggctgaggagga
cagattatgaggccaggggatcaagaccatcctggccaacatggtgaaaccccatcactactaaa
aatacaaaaaaattagctgggtgtggcagtgtgcacctctagtcccacctacttgagaggctgag
gcagg

>chr14_42794650_42794850
tcagtgcctctaagttaccttcttcatttattcattggttgccagataatacagaaaatgtaagc
cccaattctggggcttacaaagaagggctcttggcttctcttggaaaagaattcaagggtaagcc
agtggagttacacagcaatcttttattgagtgatatttctccttgcaaagcaggactaactcata
ggcag

>chr14_42813450_42813650
gattgggctgagcccactgattctttcgtaatgccataaatttttcttaaccaagaaatgaacag
caagtgataattctaattagttgattggtagctcactactgaaagcacaggtttttttgactcaaa
gtcttcattcctatctagatatgtaaagctgtcacatcttaagatattgattggtgagcatgtaa

ttagc

>chr14_44800650_44800850
ggtaatttataaaagagagagttttaattaactcacagttctgcatggctgcagagggctcagaa
aacttagaatcatggcggaaggtgaaggggaagcaaggcaccttcaacacaaggcagcaggaagg
agaatgaatgcagaaggaactaccaaacacttataaaaccatcagatctcatgagaactcactca
ctatc

>chr14_54382050_54382250
ttcattcctaaatgccgttatgacactgcctctctctgcaaataaaaaagatactgcaaagttaa
tctaaataactttctctcttacaatcatcacacttgacaatttatattcataagcaatattatca
cagatatttggcaccagatgtagaaccactagaaatattttgaccacgaggactttgagtgagca
atgcc

>chr14_79547247_79547447
cagaaggaaatatacattatataatccggtggtttatcttcttatccctcccccattaacttttt
tttgttttgttttggtttttgaatctctgcttcccttctccatcccctaagcaaaagcataacca
gcacgtttttagcatttagtagatacaagcctagcattgtgctaagtattttgcgtctattatc
tcact

>chr14_82013447_82013647
tcaagtcagcttcatccctgggatgcaaggctggttcaatatatgcaaatcaataaacataatcc
atcacataaacagaaccaatgacaaaatccacatgattatctcaatagatgcagaaaaggccttc
gataaaattcaacacttcttcatgctgaaaactctaaataaactaggtattgatggaatataata
agagg

>chr14_83822447_83822647
tgattcttgcaattagagaaggggtttgtttttattactgttcccagaacagaactccctattt
tgtgacaccgaccacaatggaaatccttgctcttatttttggaggggcttttgttgaaacttggc
aggggaataggctgcaaacaaagatgcgcttctctgtgtgtcagtggcagtttcacttctgcatc
ttcac

>chr14_85144247_85144447
ttgaatggtatggctctaataagtcattgagaaaattattattgatgctcatcattttatagagc
tgggttagctagacaatgtaaaagttcattggctctccatacctggaggacactagaatgaaaac
atgctgattcaaggttgcaatcaattcagaaggaattttcactatttatactgggtcatgaagag
tatac

>chr14_94135047_94135247
gggcataatggcacgcacctgtagtcccattgcttgggaggctgaggtgggagaattgcttagcc
ccaggaggtagaggttgcagtgaaccgtgactgtaccactgcactacagcctgggtgacagagtg
agactctgtgtccttcacaaaaaaaaaaaaaaaaattatcctctagtcctagctatttgcggggc
tgagg

>chr14_94337847_94338047
ctcatcttaactaattacatctacaagaaatctattcccaaacaaggttacattctggtactagg
gggtaggacttatgaattttggaggggacacaatttaacccataaaagaaggggatttggggaaa
cattgctcttagctttaaatgggtgtagtggtgacaagatgacaacggacaaccccagagctggc
aggga

>chr19_20928160_20928360
tctcaaactcctgacctcaggtgatccgcctgcttcaacttcccaaagtgctgaaattacaggaa
ggagccaccacacctggcaaagagctgtaattgctgttggtaagttgtaactggagcgaattttt
caaaatattgctatttttttttccaagtgcaacagctcatttcactttatttagctatgaataatt
gcgat

>chr19_31208960_31209160
cacgttccaccaaactggaggtatgtttccatagggaaggagaaaaattcatggtgtaatacaat
attaaaaatgtcacccagctgagcattccatgtagaagcttgtgtagagttttaatctcttcacc
tgttggtgctaagagaaaagagagagttggggtgggggagggagaccgggagggagaggaagggc
ttatg

>chr18_10650400_10650600

tcaaactgcaaggtggcagtgaggttgggggaggggagtctgccattgctgaggcttgagtgggt
aaataaagctgctgggaagtttgacccgggtggagcccaccgcagctcaaggaggcctgcctgcc
tctgtagactccacctctagggacagggcatagctgaacaaaaggcagcagaaacttaaacatcc
ctgtc

>chr18_31134202_31134402
cctctaactcattgattcaagttattgagtgtactggcttcatggaatatactgttttaaatggt
agaatccttgtaacactttgtaaagatatcagaagtaacattgaaaaccttttgtaccacacagc
agtttgaagatccaatagtgaagtactttaaaaagtcattgagcttaaagatgagttatatgctt
ctttt

>chr18_37413802_37414002
atccagcatcttggtgtggtgtggaaagtatggtatgacatcttagtatctatagttttaaagta
attcttggtacattaacactgataagaatattgaacagagatgaccaagtgtaaagttctatgtc
tcaacaaaatgatatctttccaggttgacaataacaattttaaatacttatatttttgttcaacc
atttt

>chr18_53520002_53520202
ttttccccattccacaggctggatatggacacagtgatgccccattttggggatgcagatgaggg
caaagtctccaggatggccaaacaacaagagagaaggagcctggggcctggactccatgtagccc
cacagcagacgagagtcctgatgacttcccagaggagagccacctcctagcaggcatgagagagt
gataa

>chr18_61412420_61412620
tgaaaaagaagtttttgtcaggaaaggctttgtagaggggttaggactttagctagttttgatgc
atgaggaggattaggaggtcaaacatagcaggacatcattaggacaggggatgatggtgttgagg
gcgtcagaccccaaatggaggatcttagggaaggagaaaaagggctcagagagatatgggcttat
catac

>chr18_62211220_62211420
gagttttcacaatttttttttttttttaattttgaaaacaggtatcttctggaactacctttcaac
tttatccttctgtacctggcttatctaacttaacatgtcttccaggtttcttcatgttgtaacag
atagaaagatttccttttcatgattacataacattttattgtatgtatctgtcatgttttcttta
tccct

>chr18_63617620_63617820
taaatattttttctgttgatattctttggtttttttttcataattctacaaatacattaatcat
ctatcactttaggtaaataacattcttgtagccctgttaaatcttcatgatttctattttccatt
gcaatctgttttggattatttttgttaatgtttgcttatgcttaattgctgaaccacatatgtat
aaggc

>chr18_70095620_70095820
ctccagtggttcagaagaaaatcttgttttgttttactagaattttacttttctctgagtttttt
tgctatgacaaaactcaaaaataatttaaaaatggaatagttattctgaatggctaatacgcaat
gacatatagagaacagacatttcatccctcactcttccaaggtctgatgtaaaaaaagttataaa
aaggt

>chr18_70811620_70811820
cccatcagatatatagtttacaaatatcagatagcttctcccattccacagtgttatgttttcaa
tttgttgattgcttcctttgctatgtggaaactttttttagattgacacaattccacttgtttatt
tttgttttcgttgcctgtgcttttagtgtcatatacaaaaatttattgccaagaccagtatcaag
gagct

>chr20_6125400_6125600
tttgtcaatttttgctttgttacaatagcttttggtgacttcatcatgaaatctttgcccattcc
tatgtcctgaatggtattacctaggtcatcttccagagtttttatacttttgggttttacattta
agtctttaatgcatcttgacttaatttttgtatatgttgtaaggaaggtgtccagtttcagtttt
ctgca

>chr20_6398600_6398800
tattgagtacttgaaatgtggctagtgcaattaaagactaaaattttaattttacatttaaatag
ccacatgtggctagttgctaccatattggaccccagagcgctagactataagctttgcgaaccta
gggaccacatgggccatcatcgcgtgcctaaataatagtaggcaatcaagggatattcgctatat

ttagg

>chr20_22852600_22852800
ctttttgatgtgctgctggattcggtttgccagtattttattgaggattttcgcatcgatgttca
tcagggatattggtctaaaattctcttttttttgttgtgtctcggccaggctttcatatcaggatg
atgctggcctcataaaatgagttagagaggattccctctttttctattgattgaaatattttcag
aagga

>chr20_54016393_54016593
tattctaagtcagccatcataaaaatgcttcgacaagccattacaaatatacttgggacaaatga
aaaaaatagaatgcctgagctcagaaacagaaactctcagcaaacacatagctccattgaattca
acagtagaatgtgtgggactaatgaagaaactggaagcctggaaaggagaacaataaaattacac
aatct

>chr21_24869329_24869529
aatctttgccactagagacttcaaatctcttcaggcttaagttggaaataaaattcaaatagcaa
tcaaacaaaaacaaattactattttcaaataaaataaatgatatttagtaaaagcaattcaaata
taacaaataaattttatcacagctttttttttttcaattttagcctgtaaatcaaggcttttgac
taatt

>chr21_40886130_40886330
tttggcttccaaaaacaaacccatctcataactcatagattatccatgcattttatttgaaaaga
cgattttagacagaacaaggcaatgaatggaactatgaaagaattctcatttcccatagttgaag
ttggagtcagaatctattattattattatttatttatttattttatttttttgagacagggtctca
ctctg

>chr7_14269275_14269475
ggggagaaaattactggggttaatctttagggatattgtttttttcaacaattacaaaatacactt
atgatcaatgaccatccttcattcccaagaaatctgtctctaagcataaagttgttaaataaaat
attttgttaagtgtaacaggataaattatgatatattccgtccatgccaagataaccaaatagtt
ttaac

>chr7_18223275_18223475
tttcattttctgtagagatggggggtctcgctatgttgcctaggctgatctcaaaatcttggcct
caagagatcctcctgctttggcctcccaaagtgttgtgattacagacatgggccatcacacctgg
ccattttaaacagtttgtaaactaaggtattaaagtaacaatacataaatggtaaagtatcctgg
gctgt

>chr7_35180275_35180475
aaggaaactcagagaagttccaaaaatgttctgggcaatggcagtgtcactgcaatgagtactta
acatcctcaagtgacttaggagaaaagaagtcattttttgtcataaaatgggatatgattaaagta
agtcttattactttatgatcattttttaaatatttacttgggcactgtcttaaaaagaaaaacttc
ctact

>chr7_35497075_35497275
tcctcacatttcaaaaccaatcatgccttcctaacagtcccccaaattcttaactcagttcagca
ttaactcaaatgcccataatccaaagtctcatctgagacaaggcaactcccttccacctatgaac
ctgtaaaatcaaaagcaagttagttacttcctagatacaatgggggtacaagcattgggtaaata
tagct

>chr7_39064675_39064875
aatggtaccagttcctccttgtacctctggtagaatttggctgtgaatccatctggtcctggact
gtttttggttggtaagctattgattattgctgcaatttcagatcctgttattggtctattcagag
attcaacttcttcctggtttagtcttgggagagtgtatgtgtcagggaatttatccatttcttct
agatt

>chr7_40456875_40457075
acattttaaaaatgtttacttggttccattcagtttttctttttttagtaatgatttttcagggca
tacatttttctataagcaagactttatctatatcctaatgtttggggtatgaagtagtctctttt
ttattgtcttgtaaatccttagtaatagcagttttctttttattttcagacctaaggattatttag
gaaaa

>chr7_51634306_51634506

gtcaaggtaggtggcaggaatgggcccctgcagagaagggcgtccctgtgcgttctggccttgaa
gagagcagatgccactcaaccaggagtctcgccagcagggagagagaaggtggatttgaacgcaa
ggcctccagtcagtgggggaagggtagatgttggatgccacccccatagggcagctcattctccc
ctctt

>chr7_68365864_68366064
tctgtctctactaaaaactataaaaattagctgggcatggtgtaatcatgtctgtaatcccagtt
cctggggaggctgaggcaggagaatcacttgaactggggaggcagagcttgcagtgagccaagat
cacatcactgcactcaagtgaaaccctgtctcaacaaaaagaaaaccaaaaaatcctcattctag
gttat

>chr7_71634464_71634664
cagctgattatttttttttttttttcagatacagaatctttctatgttgcccaggctggtcctga
actcctagcctcaggtgattttttttgcctcagcctctcaaagtgctgaggttacaggcttgagcc
accacgcagccttgcttcttgcttataccctcattctccctctaaggctttaattttttttgtta
ttttc

>chr7_84751664_84751864
cttatagaaatgaatcagaactggggaataaaattaaggactggctttgctcagatgtagaaata
aatgcaccacaagaatttaaactattttgttatttaacatttttttccttgactaaagttgttttt
gtgttttgtttaagcaaatatctgaagaaccaacttttgtcgaaacttgaattatttgatcagac
gagac

>chr7_89121864_89122064
catcataattacatttgttctgttcaattagggttgggtactagacagaaaatattttgtaaaag
gaaatacccaccttctttgaagcactgttatacataaattctttccaaaagtattttttaaaatt
acagagaaaatacaaaattgtaagtgattcatttttttattattataaaaagacacacttaggcca
gtagt

>chr7_113183964_113184164
atctttttcctagtcttcttggcaaaactccaaagctcgttaaatcaaactgttccccttcacag
tagcacttctagacagagtagtgcatattgactatttccaattgcttaccttctaatctgtattg
aaccattccaatctggctttatttttcattactctaccaaaataatccttatcaaggtcactggt
gatct

>chr7_118034764_118034964
ggcctgcaagggtcttggggattttataggcacaggataggggcacagcaggccagaggaggtct
tgggaaatacaacatttgggcaggaaaacaaaaacccttgtcctcacctaggtccatgggcacag
gccccgggtgtggagccctagccagggaccacgccctcctctactccacacttccctttctccct
tctgt

>chr7_123006364_123006564
aaatttttctttttaatatctccgaacaatttttttttttacacagggtcttgctctgtcacctag
gctgtagtagaatggggtggtcttggctcattgcagcctcaacctcctgtgctcaagcaatcctc
ccacctcagtctcccgagtagctgggaccacaggtgcacgccagcatgcccagctaatttttgta
ttttt

>chr7_142808678_142808878
ctttgcaaaaattacaacaattagaagattatggcagtgaaggggatgtaatctggccaaattcc
ttcttgcctttggtcttcaagttgaccttaattattcctgagcttaggctaagctaacttcggaa
gacatttagtttatagcttaaatgataatagcccttccctaaaactcaaccactgttgtaaagct
aatga

>chr7_144974467_144974667
ggttttaaaaaggtagtttccctgcacaggctcttttctctgtcaccttgtgagacatgtctttc
accttctgccatgattgtggggcctccccagccacgtagaactgtaagtccattcaacctctttc
ttttgtaaatttcccagtcttgggtatgtctttatcagcaatgtgaaaattgactaatacagcag
tgtat

>chr6_9464214_9464414
ccttttaatacacggaataggaaaaaggatgctaaacggagaatcatgagacttagatctccttc
tgatctcatagctcagaaaataacttgactgtagcacagtctttatgaatcttagttatttcata
attaatacgagaaataagattaatgggtgattcccaaacctgtggtatatcagatttttcagtaa

aactt

>chr6_9981614_9981814
aagcatccaattctttgcaatagacatcatccttttgccctttagtaggcagatcactgtgtttg
caagcaacaggtgcagggtgaagcttgtgatttatttgaggtatgtttctcaaaagttacatttc
attttaatttctgccatgtaggaattcatcaaggaacccctaagcagcacaacagattacatgat
ctact

>chr6_33439422_33439622
aaccatttcttcacacacacacaaaaaattgcaagggaaaaaagagatggattggaacctatagt
ttaaaagagtttaaaagaggcctggcatcgcttcttggccttttggctaagatcaagtgtaaaag
aggcctggcacagtggctcactcttgtaatcccaacactttaggaggataagcaagaggatgact
tgagc

>chr6_55189841_55190041
ttttactaaactcattgaaattcagtacttcctgtgctatctttgtcactaatagaagttacaga
tatttttatataacattttattagtggaagttctttcaaattaggatctgtgataatcactacac
tgaaacaacaatatttttagactcacttagatcttgatatttccttctgcaatgaggaagcatat
gtatt

>chr6_58678241_58678441
ctgctgtctctagttgatcaggatcaggataggaacattcagattcagtttattaaagctgaaaa
atgctgtcacacttcttccacttaatctggctgtgtaagcattttccaaaaagaaataaagttag
tattcagagatacgatcactctagacacagatctggtaagaagatcaagataggtgtgttttaat
tccag

>chr6_86743081_86743281
caacaacaacaacaaaaagaaaatttcaggtctatatacctgaggaacatcaatgcaaaaatcct
caataaaatactggcaaaccgaatgcaacagtatatcaaaaagcttatccaccacgatcaagtca
gcttcatccctaggatgcaaagttggttcaacatacacaaatcaataaacgtaatccatcacata
aacag

>chr6_89125681_89125881
ataccacgggttgcaagctcatccctcagacctgcgtaaggactcctagagctattcctgtttct
tctgtgacatataaagaaaagtcttgccccgttggcaagcttacaactggggtttgggttagggc
cttctttagggcctggaaagccacttctgtttcaggtgtccatcttactatatgggtattggctt
tctga

>chr6_93085679_93085879
gcccagcatatcttaatcaaaaaccccttatttgggaaaaaataccattggctaacccagcttca
aatgctgcaatatttgactaaagagcttcagaaaccataagaatctattttaaaatatatcaaaa
aattataccccctggaataagtaacattcccttaattcaacatgatgatgttgacatttttgatct
cataa

>chr6_101933287_101933487
accttccaagcataaatattttttctttataggaactttgtttcacttttaagaactactaaatca
ctaagtacaaaacatatttgtgacaattttggggaactttgtgatatttatataaaatatgttga
taagcttgtatgttagtcatttgtttaagtcattaattctgaaaagtaactcttacagaatttag
aaaag

>chr6_102368307_102368507
taatcgtatgtcagtggctcaaattgggttgaataacaagagcagatgagaagtacagttttagc
tgggcatcaatgttactactaaaacccaaggaggaaaacaaatactgagcgatagtcatcagtct
ctattatagatatcctccatgaagagagaatgaggaaagttactgaatggtagtactgggtagta
ttgaa

>chr6_113411107_113411307
ttgtatttataagtaaaatgacatacaagttcaccattgtgtgtgtgtgtgtatatgtgtacatg
ctctctctcacacatgcacactgtataaatgtatatacacacatgcacacacacatacataaaac
tcaacatgatatgaataaaagtgtaagttttattccaaatttttgctgatgtttgctgcatgctt
tttta

>chr6_142234507_142234707

cctatgaagttaatatgtttccttgattaaacccttttctcacgattgaatctgtaacacttaac
acttgggtgaagcccaggccttctcctccatagaggaagaatggaaatatcagatgggaagtctt
actctttcgacctctaaacctcaatgtatctgcacccaatttctcctgcattttaattgttacaa
aacac

>chr6_148369707_148369907
catgttgattaagaaaaatgacaaagaaatgaagcattataaaattgagaatgaaaaggaaatta
tatcaaactatgtaaaatttttaaaataataatgagtatattaagacttttgcatcaatacatta
gaaaagtaagcaaagcagacaatacaccatacccctccaccaaataatttaccaaatttgaatca
aggag

>chr6_153749107_153749307
actggttgctgcagattatgggtcaatgtcctgtttttatatatggtctagccattgtccattca
catatccagtttatcatccatgtattctagttagtggggacaaagcaccatttgatggccatcgc
tcaaaataggaaaatgcacctttgagaatagagcaaaaactttggaaagtgtcatccccaggcca
acatc

>chr6_156142508_156142708
gttctagtaataagagtatcccccaaggttttctttttttcctttgtattgtaagccaattactca
cgattcctgacactagggaagaagagactagtacgataggggcgtatgtgtagaagcggaaagat
atttgttttgtgttgccccagggtggctgagctaagtcacaagaaagtgaattcagctcagctaa
ggaag

>chr6_165102210_165102410
atttttccatttgagagggaaattaataatgaatgtgttttgttctcgttgcttgggttttgttt
tgctatgttgattttaaggaggttgattatttcttaataaatggtgtgaaattttggagactgcc
acctggtgagttgagagccctggtttcatgactaactggctagattttccaaacatttatgtaac
cgaga

>chr5_7219000_7219200
tgaactggccacatccttcatatagaaaattgaataatttaataaggaatggtctaactcagatt
ctagttctatctgtttatttgcctggtaattacagcataacaactcaaattggactgccagctga
gtgtttgcctggggccagagatttccttgtgcacatactggtgttcccttacaccaagtagagac
tgata

>chr5_18229843_18230043
gggagggatagcattaggagatatacgtaatgttaaatgatgagttaatgggtgcagcacaccaa
catggcacatgtgtacatatgtaacaaacctgcacattgtgcacatgtaccctaaaacttaaagt
ataataataataaaatttaaaaaaaagatctgaactttcagtagcattcaacacatataactact
cccct

>chr5_18445243_18445443
aacaaatttaaattcatttggggttcatttcattttagacattatgttcttagaaacatggatat
atattactaatctaaatggcttataattaaattgaagttaccaacatcatcaaaaataattattt
cttcttatacatcttgatcttctcattatgcaaatctggtctatcaattattatgtacacccttc
ggata

>chr5_29313043_29313243
tgtacgtaggttcaggtaatttctggtcccaaagcactgaaataggcagcctaacttctgagacc
aaacatatgtttttttttttgtttataattaaaacaaagtcactgttaaaaccaaaaagaataaa
aattacagtatctcactgaaaagcaaactatgatggtaaggtaacacattaagaactttaagatg
tttag

>chr5_51792843_51793043
cctccccaaactttcctatccccaaagctgctagctaaatcagtgatggtagcaactcttctgtt
atctggcattcatgggatcccacctcatacttaatctcaatgtggaaccatctgtacacatcttc
tgcgcacagcattgaccaaatggttccaaaactcattaagaaactattcaaaagaaaaataaatt
acatt

>chr5_57906043_57906243
tgtgtgtacatacgttttttatttctcttgggcaaatccctaggagtgggattgttaggtcatatg
gtaaatacaatttaacttcatgagaaactgctaaactgtttgtcagagaggctgtgccattttaa
acagtctcagcagcaatttatgagcattccagttgctccacatccttgccagcaattgttgtcag

gtttt

>chr5_63267244_63267444
aatgatcagtgatgttgagctttttttttataagtttgttggctacataaatctcttcttttgag
aagtgtctgttcatatcctttgcccacattttgatggggttgtttttttcttgtaaatttgttta
agctccttgtagattctggatattagacctttgtcagatgggtatattgcaaaatttttctccca
ttccg

>chr5_97134044_97134244
ttcattataccctgttgcactgacagtgtgatcctgcaaataaggattaactatgtgctttcatg
tggaactcctctttgcagcatgtttgctaaatgggttcttctaccttcaagttcactctacttat
atttttgcaaatgaaatgttggtgattttgtatttcattatgtactacggctcactcaagtaaat
ctgcc

>chr5_99218901_99219101
aaataaacaataaaaaaaattagtatatttatgtcccaaatattgcatgcatgcatttatgctaa
aaaagtgtattcattgtttatccaaaattcacattaaactgagtttcatgcatttttatatacca
aatttggcaaccctacatagaaatacatagtaaaaataaaaaacaaaacctccctgtggtcagaa
aagta

>chr5_103882501_103882701
tgaaatctgttgtaaaacgtgtgtttgaacaatatgaaatcagtgcatcctgaaaaagaacagaa
tagcagcgtttttttggggaacaagggaagataaccataaggtcttactgcctgcatggttgggaa
gtatagagccacagttttcttcttgcagaaagccagtacgagaaatttcactgaattcttttccc
aacaa

>chr5_117744701_117744901
aacaacatggcttaccaataaagcatcagcagaaaagacaggaaaagattgaggttcagaaagtc
aaggtgaagttaacgcccagcagcaaatgcagaagttctgtgcaacttcgtagcgcagctgagac
tgttttgccccttcagtattctggcttttctgagtggagttttactaatttgttttgctttgttt
tctcc

>chr5_122003901_122004101
tatgcaccagtttttcttatcaaaacgtatcagcgcttgtacaggaatacaagaagaacactgag
gagaataatcacagcctaagggaaacagcatggtagtcttgtagctacttccagcataaggacct
tgctgggcattatctgccaggctgtggtgcgggaaaccattatacagcatggaaaagtgaacttg
gcaaa

>chr5_125293901_125294101
ctgataatgagtgaggagcgtaaaattgtaaatcactaattgtatgacgtgtccaattatgctat
gttagacaacatagcctggaatgaaatataatggaacagaaagagaattttcaaccaaaatataa
tgtggctttacagttaatgaatatcttgctatgatacactttattggacagcagagacaagcatt
tcttg

>chr5_144161407_144161607
agagccattttacacttaagctattaatatatagtgcattcttttgccccatcctttgttatgga
ggcctgctatgagtattttcaggttgctaaggtatgtgggctcaggagcaagccaacgacgttgt
cacaggaaaaacaaagcatggagggcagcatagttccatgatgtggttctcatggtggctttctt
ctcca

>chr5_151530807_151531007
tagggatgtattaacattactcccaatgtcagggagaggtgaaggaacagactggctatgcatca
ctcagagtcctgaggctgtgcttctggcacagtttgactcagcgatctcccgtgagatcagactg
gctgggcctgccgtaacatcacacatttataggatcttaggttggaaaacactgtaggggtcatc
tagac

>chr5_155328822_155329022
aaacctttttacttcttaaccttattacttacctgtagctgatggtttctccccacttcatagat
caagaacagaaatgacagatggtaatggtttagtgggactctacccaaagtacagatttctcatt
cataactattcatagatttcctggaacttaccctgcttcactgtttgttcatgagttgatatttt
aaaaa

>chr5_155363622_155363822

atgggcggccatgggcaggcccagaaaaagcaccacaagttcccactctggtccacggaactggc
agtccggcccccaggttttaggccctgtccagcttgaaggtggggcttcactagggacgcgcccc
cttttacccaggagcctgtctgcctcttaccactgttccgtacagcccaggctgttcatgcccag
gggca

>chr5_165102222_165102422
ttttagtacctgttttatttttgttgttgttgttgttgggtatatacttaacagtgtaactacta
ggtaatatggtaatcctctgtttaactttttgagaaaacaccgaactgttttccacggtggctga
agcattttattttcccactgggaacgtatgaaggttctggttcctccatatccttgccaacactt
atttt

>chr4_18319502_18319702
ctttgactactgaacctttcaaatttgttgggcaccaggaatacaaaacaaataggatcagcctt
ctgatttggaggggaaatagactatagacaaataatggagccccatgtaaggggtcctgcagta
agttgcttatagaaggtagttgtatacagaaggtagaaaaaagggaaaggtcacctctggctatt
cctcc

>chr4_22789502_22789702
catttccaccaacagtgtacgagcgttccctttttccccacagcctcaccaacttctgttatttta
tgactttttactaatagccattatgactggtgtgagagggtatttcattgtggttttgatttgca
tttctctgatgattagtgacttggtttggctgtgtccctacccaaatctcagcttgaattgtagt
tccca

>chr4_29179702_29179902
cactatcaagaccaaggtgtaaattgtatagggcactcaatcccaaagttaaaaagccacctaaa
agataatactttgatttgcctatgtaattaggcacgacatgttccacttagtacctataatctag
gactgccttgggaacacttttctttatatatgccttctatttcccttctgtattatggaaagata
aatgg

>chr4_30546102_30546302
agagcaataactctggttactgttcctctggacatttattcaatccatcagtctaaaagccaaga
ttcaatttagtagtttgatctatcactaactaaaatattaatttcctttaaaatattaaatgact
tggaaactattggtcttgtgtgttataagaggaagacccaagggaactgaggaatgaagtgaaga
caaaa

>chr4_46748443_46748643
tctagaatcagggcattgaccaaaagtggagcatggagtacgctgtcaccagaggtaggcagaat
ggatattgaatggggcggaaacaaaagatgtccagtatacattgaaagtacagtggaaacagagg
tttattcattgagagagaccagtggactagattttctaaagtttcttcctcctctatgattttgt
ttcta

>chr4_58465843_58466043
tttacactttataatttatagcttaataaaatagtatatatcaggtggaatgtgagaaataagga
aattgatctattttgaatttgtaaagaatgcttctttgaaaaactaaactcaatttaataaaaat
attaatacattaagttgaagaaaacctggcttaaaaatggacaagacaaagtattttaaaaagaa
gatgt

>chr4_59820753_59820953
gatctcctgaattattattttcatcaaatttggctaatcattggataatcataatttatttttca
ttaaattttatgtctcctttctctatcctctccttaggaatccaaccacccatgttttagacca
actgaagttgtcctacagttcactgatactcttcgttttacatttgtttgctctctgtatcattt
caata

>chr4_61457605_61457805
atctcacaccagttagaatggcaatcattaaaaagtcaggaaacaacaggtgctggagaggatgt
ggagaaataggaacacttttacactgttggtgggactgtaaactagttcaaccattgtggaagtc
agtgtggcgattcctcagggatctagaactagaaataccatttgacccagccatcccattactgg
gtata

>chr4_67794005_67794205
aattcacttgcacctacagacagccagtctcctacccataacattcaggggtagactagctggcc
tggcatctagggaggttcattttcaaaaatttaaagccaagacgtctatcaacatattcagcaaa
cggcagtaggagcatctagtcattgatataaactgtctaggataagtctgctagggtaatatgat

tgctg

>chr4_68063405_68063605
gaatttgcacaacacattaagtttggtaattaatagaatcatgtatggtaatttgggctgttaag
agatttctgtttggttgtggatagtcaaaactacttgattaaaagatttcataagcttccatatg
tagcttctaaaacaaaagctgattatatatttagtacctactactgtatattcactgttttctac
tcata

>chr4_72538136_72538336
agtggagatctgaagaaagggagaagggagacatgtgaatgaacatgggaagaagggtccaacca
aaagaaactgcaagggcaaagactcgggagaagcatggaggcccaggttgctacagtgcaggtag
tgaaggggagagaaattcagatacacgttgggtatctgcataaatgcaaggaaatgaagtcagac
aaatg

>chr4_73847336_73847536
attgaattctctactgtctctcactcaatagcgaatagatttctatcatctatttacagatattg
acaaataattagaccaaaaaatcaaattcccaatcactgttgccactagaaaaaggaataaatta
ttggccctgctcaaatcaaaaccaaaaacagaggccgggcgcagtggctcacgcctgtaatccca
gcact

>chr4_112398551_112398751
tattatttgaaactatactaacctattttcagcccttcctacctctcgtcaccatctctcctcat
tttccaggtttgattactttaattaaggaaatgattaaaaagattatcattattaaaattttatt
tttgattatctattttctatttttaaatatatattttaaaatttgcaaatatatttaagaataaa
tttag

>chr4_119133352_119133552
ttaattgtttttataaataaagcagatagaggcagctacagtctggattaaaagtagagaaagg
ccaggcctcatgcctgtaatcccatcacttagtgggggcaaggtgggcagagagcttgagcagcc
cagaagtttgagaccagcctgagcaacatggcgaaaccccatttctacaaaaaaatacaataatt
agcca

>chr4_122204350_122204550
ccaggcaggccctccagatccaggtttcaggccaggatctgtggtcccaggcttctggtctaccc
caaagccaggcaggccccagtaggcccaggcccaaggcaagcccctgcagcctcatgttctaggt
ccacttaaggcttaagacctagccccagtgccaggccagtccccagagccccaggcttcaggacc
acctc

>chr4_136804950_136805150
attttaagagtactatcagtaagataaaagaatcttattaattagaataacaaaatgcgtaaggt
caagcaatcagagacaccatgttttgttggaatgcagcaaagcagagggagtgcaccataaacta
agaaatcagaattctaaagctaggattttaccaggatttgttttgttatgttaaacaaatgaagc
aaact

>chr4_153144750_153144950
acagcaacccatcaaaagggggcccagtcaactgagcttgtgataagggagccccgtctgctttaa
cccacacaaggagagtaacctgaagtgacctgatgttaaccaatctgctttttgcagtaggctat
ctccccgttcctgctcaagttgccttacagaaacccactcttctgcctcacccagtggagctcca
gtctg

>chr4_160002750_160002950
gatcacatgagcctaggaggtggaggttgcagtgagccatgtttgctctgcactccactgcactc
cagcctgggtgacagagtgagactctgtctccaaaaagagagagagagaaaaaaacaaaaagaaa
atgtgattttaaagctgtattttattctattgctttaacataatttatttaataagccctttctt
gttga

>chr4_164230350_164230550
tctctatgatttttctgtcaagtaattgtaattctaccttgttttcgaggtttggttcaggtgt
tttcaagaagcttccttcagtcctgtctgagctaagaacctctgagaatgctgtgaatatacatt
tagggttatgttcaccatatgctacatgcgaaatgatatatttctgtatattttccccactgaat
ttagt

>chr4_173442425_173442625

ttgcaaatagcctttcagcacttgactgacagaaaaattcttctgacagttggtataaaacattt
taccacaaacagaactttggcatctctagcacattgcagacatctgttacaatatgccataattt
ttagcacctcatcataaaggcaaatccagaactatgatctaaatataactagcatattttatatg
ggctt

>chr4_173475225_173475425
gattcttgccttggccttgttccaattgccattgccttcaacaagaggatttatcatttcccaat
tctaactcccaccccaactgtatattctaactgacccatggattctttcaatattggaaactatg
taagattaagtttacctctttgatatatttctcattaaagcattgcatgcatttcctcacattct
catga

>chr4_176416806_176417006
ccaagcaatggctagtgcccaaagtggggctcaagtacgggacagatttactgttatattggaaa
ggccctccgttttaccatgcaagctattctgtcgttattgagctagttgatgagcagtttgaagg
ctgtctctgcaggcctttcagttggatgtccctggctgccttgagcagagtttccgttaatgtct
ccaag

>chr4_180496006_180496206
gaaaaccttcactatcagtaggtttcccagtagaaaccatgcatgccaggagtcagtgggatgac
aaattcaatcagctgaaaggagaaaaaaaaagacaaaacagcaaccaagaatactatacctggca
aagttgtctttaaaaaatgaagatgagattaaaaaaactttccaaaataaatgaaagctggggaa
gttca

>chr4_182302206_182302406
aaagtttcttacctctgctccaatatggtatgaactattgtacacaagagcctatctgagaataa
tagcctcaaccagacttggaatccatatatctcattgcaaactagattgatttactaattggctg
ccagtagtgggcggatgttattcattccttcggatgtcctgacaaatcaccaaaagtctgctggc
ttaaa

>chr3_892200_892400
aactagatggggtttctaaaggttggcccccatgtttgagggccttggctgcaactgccaatgga
cggatccagttatcagataaaaaaatagccatgccacaactgctaggagccacagtcgtactggc
tgttcttgaggccccacatgctgtggtgactttaatgaataccaaaggacatcattggctaacaa
atgct

>chr3_1869400_1869600
aaagattattttgtcccaaaagtcaaagcttgaattttgtcaataatcctttatgtattaattac
acacgaagttctaagaaacataatcttgctttaagagatgtggcttgtcaatttagcccaattcc
agtgttagcaaatattatatagagattttttaaaagaaacaatgagcatatccattttgaagta
tgtag

>chr3_5717200_5717400
tcgctatagcatctggtacatagcaggttgcttaagacatcctggcaggttcccagaaatcttgg
tggttacatagaaggaggcttccagtaccaatccctccctacacttcagactcctttttttttt
tttttttttttgagagagggtcttgctctgtcacccagactggagtgcagtggcacaatcttgg
ctcac

>chr3_6181000_6181200
cttgcccccaaagcatacaattccttttctttaaaaaattacatgactcattcttataggaaaaa
gaaagagatagttctataaaaattaaaatgtcagaaaggaacaatttaaactcgttttatggata
gtcagtgtaatatagaagaatgagcactagttttgccgtctcacagacctgggtctaaatacaag
cttag

>chr3_16748996_16749196
tgccagggagatgggacagggaggggccgataaggtagaaccaatggggttgactaatcagacag
ctcacagattagttttgtggccctcctgtgtgctaggcatttgtctgggctctgaggacagtgta
gtgaaagagatgaacacagataattaactactagtaagcatgcacactactgccaggtaaaagga
atgca

>chr3_16766796_16766996
caaaattacatatgaaggatgcccactgatgcactgtttttaataaagaaaaaatggaaacaacc
ccagtgtgcattcatagagaagcagtaaataattgtaaattcatatagtataatacgaaatagca
atgtaactactgacacagaatgtggccttgatataaattaagggaaaacaacaagttgtaaagca

gtgtt

>chr3_19320996_19321196
ttgaaatgagattgccatggcatagatattggggcagatgaatgaaatgggatgccatagggcta
aaaaaaaaaaaaaaataaggaggaaatcatgactctaaaactattgggagaatacagaggaacga
gcatattttatattccatgtgtaacttatgagagcagcacatcatacatcatccaaccattcttt
taaac

>chr3_20254196_20254396
gataactttttttttttttggtatttttagtagagacaaggtttcgctacgttggccagctggt
cttgaactcttggccttatgtgatccaaccaccttggcatcctagagtgctgggattacagatgt
gagccactgtgcgcagccaccaatatctttctttaaagcctgtaaaaccctgagcattggcgaag
taaat

>chr3_22090196_22090396
tcaagattgctttaactattctgggtcttttgtgattccaagtgaattttaggattgtttttcc
atttaagtggaaaatgtcattggaattttgatagtgattgcatggaattagtagatcactttgga
tagtacggacaataataattattaattctttcaattcatgaacatggaatatcttttcatttgtc
tgtat

>chr3_29022796_29022996
atattctgtctgttctatcatattttaaaattttggtctcagtataattatgcctacccacatt
ctcatcccttaattccttctgtcattttctgtatacagttatgtttttttttatcattttattt
tttcacttgtcctcagtcacttttaatgagataatgcatgtgaaggtgcttagcacacataaata
ttagc

>chr3_84954110_84954310
gcaggagctgggaacaggtggtagccccaccctcttacaagttggaggggtgggagccccgccct
cacaggcacagctgcatccgcttagtcttggctgtggatctgtgtatccctgcactttgcgggga
tgggaggtgcaggaagccccccctgcccccgcagcctcagaagtgcctgttcctgctgcctggtct
ctccc

>chr3_86037510_86037710
caaggaagcagattttctgaataccttctgaaatatgtacataacatttatacagttttagtat
cagaactgcttttagcgaagctatgacaacactgtttcttaaataaagaaatatatctattaaat
gattgagaaaaacgactaagaaaaagaatttgccccagtgagtccacaggtttaaactattggaa
aaaac

>chr3_88514310_88514510
ctttttcaatctcatctacacttcaattttggaatagtctataatatttgagaattaacagaaat
attgcaagataaaagctagcaactgttatgtaaaagtatccattttccatggattcccgctaaaa
gactaacagttactaaatcatccccacaagccaactgcttttaaaaagacctctactacaacaca
agatg

>chr3_102490910_102491110
cttttctgctgttcatatgcacagagcagataaatcagacttttaggctccaaactcccttgccc
cattatggactcagttctcgcggaggtttgctcacattctgaggggggttgacctagagagttctt
cactatgatgagaatttccctcttttatttccattctaggaaggcttgtttccacattgctatcc
ttaga

>chr3_114450910_114451110
ctttcagtggatcatgatacattgttttatactttattgctttaataatccattctagactgttg
ctcaggactaacagcaagtagttgcatcatcataaattgcctttctatactttctgtaaatcaga
agatttccacatctctagttatctaacattgtttctcttctcctcaaacatggatggtagtaatt
gacta

>chr3_117549710_117549910
ttaaggtcctcagtggcaaaatctgatgttaggaaaaataatttaagagccctttgttttctccc
tggtcattttttactgatatttatcaatggaatattcacattcatagcacatcatgggtcaacttc
atatggaaaaacgaacctttcatgaataggacatatgctcaaaactatttccctagccctgtact
agaag

>chr3_117806910_117807110

tctgcagctgagagtctattgtttgaatgacaatgttatacgaatgaagtaataacctcaattat
gtgaaagttatatttctgctataacatggaaaagctgcattttatctttcatatttagtcctaaa
ataccaacaactcccttgaaagcttctttttctagaatgagctagatttaatctagataagtat
atgtg

>chr3_147318910_147319110
gaagcttatttattaatatctgcatgaagaaatttggtagcaatatagagaatagcaaaaataaa
tttcacaaagacaaactatttcaacaaatgatttctgatcattaatatatcataatttaaacaat
tagaagtttgtttctaatatttttgacatctcaaagacatagttaaaaatagagaggcacaaaat
tatta

>chr3_164188106_164188306
caacctaaaaatcatgaaaaccaatatacagttgttattttaagttgcacaggtttttttggctt
gctacgcagcaacagataaatgggaaaagagaacagtaagacacagggtacttatctaaggtaca
gtagtgaaagtgacaaaatctagaggcttggtcaaatcaggtagaaaggacgttgatataaggaa
tcttg

>chr3_173043506_173043706
aaattattatacattagatgttattatttttctttctgcagaaaagaaaacaaaaggctcagag
aggttaagtatctctaagtctaaggttgcacagctggtaagtagagtagctaggatttttgtcct
gtctatatcttctcaaacccatatgctttataattctgtaaaccaaatatagagctgcattttct
tcatt

>chr3_179768106_179768306
actggctcatttccattagcctaaaaacgtgtaatatctcccacctcaaaataacaacatacttc
catgccttctcgatattccccatctaccaagccttttctctggtcctttatagctaaactccttg
aaacttgactatttcagctgtctttactttccctcctcaatttctctgatgtcaaatctattcag
gtctt

>chr2_4263525_4263725
actgtctccaactgtatgatattgggaaggaaactttattttgaggatagttttagtttacaggt
atctatttctaggaggttttttttgtttttgtttttgtttttttgagatggtatcttgctctgccac
tcaggctggagtgcagtggcacaatctcagctcactgaaaccatctcccaggttcaagtgattct
catgc

>chr2_5263949_5264149
atcagagcttgctgattcatatctcttcagtctggcttccatgcacttattttctcaccaacctg
gccaatgttacaaactaataattcaacattgatgttcagataaaatcttgcaggtctggatattg
gtgtgaagttcatttccttaacgataattacagtaaaggtgttcctagaatgaatctgtttaata
caatt

>chr2_6849349_6849549
aagcaacctacatgtcttccaactgacaaatggataaacaaaatggggcatatccttgtgaaggg
ttattgctttgcccttaaaagaatgaagtactgacagatgcttcaatatgaatgaaccttcaaaa
catcatgctaagtgaaagaagtcagtcaaaaaagaccacatattttatgatatttttatgaaatg
tctag

>chr2_15217549_15217749
acaaatttccttggaatgaatcctctgtgtcagtcatttgctgggcactgagaatagagtgacaa
atgagacaccatgctttccatcgaagagattattgttcagttgggaacacagaaaagcaagcaag
taaatgcagcctattgtgtttcatgtaatatagggagatgtgaactactgatgtaataatgaagc
atggg

>chr2_76599292_76599492
tgttaagttcttttgtagttcagtgagttccgagttcttgtcccacaactgagaggaatgagata
tgcagacatcagagagtgggtaaggcagagtagaactgattgagtgacagaaaagctcctggcag
taagaggggacccgaaagtgggttgccggcaatgaggttgagtccaggggcttttatgggcttgg
catga

>chr2_78157492_78157692
ctgagacaaatgaaaacaaaaacacagcatgcaaaaacttctagaatttagcaaaagcaatgttc
agagaaaaaaattacagttataattatctccatttaaataaaaaaacctcaaattaagaacctaa
cttaacacatcaaggaactagaaaaagaaaagcaaattaaattcaaatataacagaaggaaggaa

aaagg

>chr2_81797889_81798089
ggatgtaaataaagtatcagctttaaaacacttatagcaaaataactgcaaggtgaagcattaag
ccctttaattacagtaccacacgaagttacttttctattcatactatattcccagcattcaaaga
ctgatgtaattaacaaagataaagttttgtaaataggtccagctgaggacccagagagagagaaa
ggggt

>chr2_84375689_84375889
ctgcagcttctccagctgattaaaattccattatttattttgtacatctggatggtaaatggaag
catccctagcattggtttctccaagctgtctccaaacaggttcaataagaggattaggagtttaa
ataggtttccaactgttaaaagtagttttctcatttttcatgttgacattctcaaaaccaatttg
tcttc

>chr2_107381568_107381768
actgcctagaaaaactttaataaccaagaactggcataaaatctgcctttacaaatctccatttc
tggtacagagtgacaggaaaatgagccccagcattcaaccaatcccctttgtgtattaataatcc
tcaaacacggctgtgaaaaattatgaagcatgcccaattgtggtgcacttagctgcgtgtgattg
gtttg

>chr2_108167968_108168168
tggctagagcactccggagtgaaagggtgggatatgttggctgactttcactaaacagtttatac
caatagcgctgggatgtgtcatcctcctctgatatcccttggagaaaggtggatatccaaactcg
tctggcattctgttagggaggaggcgatggggagtggagaagcaactgatggtgtccattcattt
gcgaa

>chr2_114844130_114844330
atttatccatgactaaaaaagttgataatgatattttaggtcgtatatggtgatcagttttgaat
gtacaggctatttggccatccccaatcatactacatggattactacatcgtgccacttccagcta
aaaaatgtaactactttggaggcctatggtgatatcctgggatgttaaagaaacagagagagaag
cagtt

>chr2_123981130_123981330
tgaaagctattggtggatctaccaatctgcaatctagagcatgatggccctcttttacagctcc
actaggcagttccccagtgtgaactctagtgggggcactgactccacatatcccttctgcactgc
tctaacagtggttctccttgaagtctccacccttgcagcataattctgcctggacatccaggaat
tttca

>chr2_125497330_125497530
caagtctctgagcctctgctcaaatgccatcatttgcataaaactgttcccacgacccctggtga
gaagtcatattttcttactctgggcttttctagaattttattaactgctctctttggcactgatc
tcactctgacttccattactcttagctgtgtgcgggacctccttcctataaatgttatgagctct
tttag

>chr2_126208930_126209130
acaatgatgcaggcaatttacaactctgccttagccttcacttcttgcttacacagatactcaag
atcaagtggaggtgaaagttttccatttttttctctttttttggatgtacacacaaccctgcacat
gtgaatgatcttctagattacaaaggatatgtcagagattttcctaggttccatggacatctcat
ttcct

>chr2_139732530_139732730
tgcctcattggaataaaaagtaaatcctttctggaggaaaataacatcaccgagttccttaaatt
acttgtatatgttcccagactttgtgtattcaatctaaaatactcaggtacataaaaaggtgaaa
gttaacaaacaaaagaatacaacgaagaggatccagatattagaactatcaatcacatcctttaa
ataag

>chr2_140284330_140284530
tgaacatgtaaatgaaaagcaaaagaaagaagcaaaggcagctaaacaatgactataggcttgag
agcttagattcagagaggaataaggaggaaaaataaaatatctgtcagagttcaagggcttcaat
acaagggccagtaagacagattctgaagtcatgaggctttccattggcttccaaagctacagctt
atgtt

>chr2_141370130_141370330

gtttaagcttggtatgcatcctctctcatctcctaccttttatttgtcagcattttcattatgg
tggaatttaatctttgtttgaaactcagtgattgagcgcttgatccgtctttaatcacacaatta
attaccatgctcagactatgatagctggcctaccaaacaatgaacaacaaagtcaacaattttaa
tactg

>chr2_164975754_164975954
aaagctctgcaatttgctgtctacttttctgccactcacttcctaaattaaacaaaaatctttct
ccctctaatatcaattagttattagattaaatattaggccctctcataccaattttatactgtag
tctaaattacaatctctaagcagtttccttccaaagccttttccttaacgccttggctcactact
tcctt

>chr2_178957754_178957954
tgacccacatatgtgaacaaaagattaatcttggactcagtagggaggctattttaactgagact
tatagattctgacctataagaaatttccaaaatgtgtctccactccttggcttctctgtagaaat
ccatactgtagaaacagtcgcatactgtttagggtcttctatggtaaaccaatgaactaccacca
aagac

>chr2_180766555_180766755
ggagataagtatctcacatggtgggagcaggagcaagtgcagggtgggggacatgctatgcactt
ttaaaacaatcagttgtttttttctagtatattattattatttattttaagttctgggaaacgtg
tgtagaacgtgcaggtttgttacataggtatacatgtgccatggtggtttcctgcacctatcaac
ccatc

>chr2_202428155_202428355
cctcccggacggggcggctggccgggcgggggctgacccccccacctccctcccggacggggcgg
ctggccgggcagaggggctcctcacttcccagtaggggcggccgggcagaggcacccctcacctc
ctggacggggaataattcttataaaaatcagtcaactcctttattttctaccatctttttgatac
ctttg

>chr2_211282355_211282555
acattttctttattcattcatctgttgatgaacactctggttgcttccatatcttggctattgtg
aatagtgctgcaataaatatcagagtgtagatatttgacatgctggtttctatttttggggaata
tacacctagcagtgggattgctggatcatatgaaaattctatttttacatttttgaggaacccccc
aaatt

>chr2_211378555_211378755
aatctttaatccatcttcagtggctttttgtgtatggtgaaaggtaggagtccagtgtcattttt
ctgcatatggctagtcagatatcccaggacaatttattgaataggaagttctttccccattgctt
gtttttgttgagtttgtcaaagatcagatggctgtacgtggcactgtttctgggctctctattct
gtttc

>chr2_229364756_229364956
ggcacttcttgtagtgcatatttcaggaaaagtttccttagtgttattttatctgaaaatattat
attttaccttgtattatataaaaataaaaataaaaatagttttgtttttcctgggtatagaatgc
tgggttaacagttataatcaatccatttgtcttgtgcgtgtgtgtgtgtgtgtgtgtgtgtgtgt
acata

>chr1_5146740_5146940
gatgtgatcaaataaacaagtctgtgtgtttcagctccctaaggacagaacacacttgcatggct
gtatttactgtttatatttagaacaatggttttaaagaaatgtaacagtgccaatacagtttgtt
gcaggtggggttttcagagagcagtcactgagactgagtttgggatactagatcccaaaggaagg
gatga

>chr1_59719812_59720012
acaaactaccatcagagaatactataaacacctctatgcaaatatactagaaaatctagaagaaa
tggataaattcctggacacatacaccttcccaagactaaaccaggaagaagttgaatctctgaat
agaccaataacaggctctgaaattgaggcaataattaatagcttaccaaccataaaaagtccagg
accag

>chr1_76881412_76881612
agaacgtcagtttcttttagtagttaagagagcataatattagcaccattgctgctcattgctac
tggattggtctttgtttcttgggctttacagaggacagaggtagtatgtaaacaatatatactgt
gtatattgtttaaagataaaatcgttatgagttcaaaccgaaactttcaattcaaatccagggct

atcag

>chr1_79719212_79719412
ttaaacaatttcaaattgggcactgccgctttaataacccacacctggttaagattccagttcca
ttgaattcacactgtggtgataagccctgaacatcaggcagagacattaatgagaacaatgcaat
taatcaccaaattgtgctcaatttcaataacataaaaatttctcttacatttttttctctccagaa
ataat

>chr1_80386612_80386812
ttcttctatgttatatatttgtcatgattgattaacttgaagatttgtgttacaaactattaatt
tcatttaaagcatattatttcattttatggtgccaatttcctgttgtgaattatttttgcttttg
acataattacctggttattttggcattgtgtaagtgtaggtctttctttgccagccaagttccct
ttctt

>chr1_82800612_82800812
gcatgctgagaaaacaaatgtatggcattttccctactaaaatagaaatatatgattccttagaa
tatgctaacatgtaatatgattatattacacaatataacatatattatgtgttggtatattacat
aatatgtatattatatattacatatataacatatgtatattataaggtagttacgtactataata
tatat

>chr1_91235812_91236012
gcatccctgaatgctcactagatgccagtggctctcacccccaatttgtgacaaccaaaaatgtc
tctaggcattgccaaatgtctcctggggacaaaattgctcctcattgagaatcactggtatactt
cttgtggtaggttctatgctaggaaactcttccagaggcaaagtagaagtatagagtgagttgga
gtgga

>chr1_103417012_103417212
ggaggccaaggcgggcggatcacgaggtcaggagattgagaccatcctggctaacacggtgaaac
cctgtctctaccaaatacaataaattagctgggcgtggtggcgtctctactaaaaacacaaaaaa
ttagccgggtgtggtggcaggtggtaggtgatagtcccagctactcgggaggctgaggcaggaga
atagc

>chr1_104744677_104744877
cagatacatgacaagaaaaatataaaatgccaacactactaaaactcagtgaaaacattgctcag
agaaaaaatttagagctgtaatcacttatattatcaaagaagaaatatttaaataaataacataa
tctttcgttttgagaaactataaaaagatgagaaaactaaacccaaaattagcagaaagaaataa
caaag

>chr1_164866176_164866376
caaagcgagactctgttaaaaaaaaaaaaaaaagaaaaaaagacaatcagctgctttcattctcc
aaaataagtgtagaaagataataagaaacgtttcactgaaactataactttgtctttgaccacgg
aacagaggtgggtggaaggcaattggggaaagaagccaatcagcaacctccagaaatttcctctt
ttttt

>chr1_176431177_176431377
acagggtagccactaagctgcaatataaatgacaaattatttatgtcttatttattaatgtaaaa
atgcccagaaattcctcctagagtctgtttcatccaccctcatccttcctgtatacctccctacc
tccattagtctggctgtaattgagaaatacttacacctagcctcaaccatagcaatgtagaaaga
gccat

>chr1_190265177_190265377
tgtttgtttgcttttgtggttttttttttgggggggggctgggcttttggtgttatatttaaaaa
atttttgccaaaaccagttttaaggatcttttcccctatattttattcttgaagttttacagttt
catgttatatttaagtctttaatattcctaggatgtacccacaaacattaaaaattaaaaaaaaa
catgg

>chr1_192378977_192379177
cgtctaggtatcatcctttacctctattgcatctgctactgatgtctctgatctgccatgacatc
cagctgacaggtccacattcaatatacctcttcttcctatggtgagacctcttctagatcactgg
tcttcacatctgctttcccattttcattgtaaatacactaacttttaaaatcccttcatcaacat
gcttg

>chr1_193931177_193931377

ttttcatgaggatttgaaaaagcaatctagatggctctgaagctatattcctacccattttgctg
agtgagccaagaatgggttcaacttgacgaggtagctcaacaaaacctaggccaatttcagctat
tggtttccctggctggctctaccttgtgaacaatgccatttcttattgctactttgtagtatgaa
agttc

>chr1_197382777_197382977
tgttgtgatcataacattcactagtgtgacttaaaaaggggaggcaccatgtgttaagcagggat
aggatctaagaataacaaatctgcctggtaagcagcacaaatgagtcttacactaagccacactg
atccagcttggagggaaatgagtcaaaaccaagtccccatactctctacctgcgagaaagaggtg
acatt

>chr1_216173177_216173377
attttacagataagaaaactgagtccagaagagataaatatgaacaagtattaactcttgataag
agagacctattagattttagctcagcacaatagtacaccttatacctcaggtatcaggaaatgga
caagaatcagtaataccagaaatcatcagaacatgcctatatgtgaaacttcctttctgacatca
actgc

>chr1_222926977_222927177
ctgtaccagggtgatttatgtaaccaagagaatatggcagaattaatgatatgccactccagaga
ttgttataaaagatactttggcttctgtcttgatctttctcactacttttctcttggatcattca
ttctgaaggaagccatctgccatgttatgaggacactcagccagtgcagcagactgaatatgttc
cccca

>chr1_239328777_239328977
gttatctgagaaacacagataaataaggcatggtcactgttcttaattagcttataatggatatt
atagagtcaaattatctgtttaaaattattatctggatctcagctgtctagttaaagcaaagcaa
ccccaaagctgagacaatttttaatacaaaatcacatgtcttaatttgatagggagcagataaag
gttta

>chr1_241472577_241472777
cgggaggctgaggcatgagaattgcttgaacccgggaggcagaggtagcagtgagcagagatcac
accattgcactccagcctgggcaacaagagcgaaactccaactcaaaaaaaaaaaaaaaaaaagga
aaaagaaaaagaaaatgacaagaataaacaacataaggtgtctatgagaaacagaaatcacccaa
ttgcc

>chr9_1139200_1139400
tccttcagtactttaaatatactatttcacttcctcctggcctgcaggatttctcctgagaaatc
tgctggtagttgtattagaatttccttatatgtaaatgtgttattatgtcttgctgttgtcggga
tttttctttgtcttttaattttgatagtttgattattatgtgtctttgtgaactcctctttgggt
tgatt

>chr9_7637400_7637600
tcttctttttctctacatttcgtgtttgttttctaaagtagtagtctccaaatagtggatcagct
agactgactgcatccagacatctggaaaactggttaaaaatttagatggattccctaagccctgg
ctatagagattctgattcagtaggtctaggatagggtctgggaaaacgtttcttaaaaaactttc
caggc

>chr9_7819200_7819400
taaataattatgctcagtgaaataagtcagaaaagactacatgttatataattccattttcacaa
aattccagaaaatggaaaatatagagatggaaaacaggtcagtggttatctggggaaagagataa
gggggtgatgagaaagggaagagattaaaagccctggaaaaaagcttttggggatgataaatttg
tattt

>chr9_9561800_9562000
actggttgtaccttctctcatttgttggttccttctccttccaagctcttattgttggaatggcc
catggctcagacttagacattttctctctatgtatacatgctgctttaaggatttttctctcatt
ttatggctttaaatgtcatctctgtgccaccaacattcactttttttaatcttctggactgacatt
tcttc

>chr9_10646000_10646200
attatgtggatctagctctagcagtgaaactacttgactttagatccaatttcaatttccttgtt
tctaaaataaaagtaattaatattcatttttcaaagaataattagaggattgaattagacaatgt
acaatatatgctcagcaaatgtaaacctatagtttgtattcatacataataacaaatgttcttaa

aattt

>chr9_10906400_10906600
tattcatcctattcttaaaattctcaatactatggtttcaatttatagtttatagttcaatttat
agtttaagattcaagctttctagcttgaaacacaaaggtgcttcatgatgagatcccagtctact
tttctgttctcatctcacacctctctcctctgacactttttttatgctccaggcgtaatgcactac
ttgtc

>chr9_11210400_11210600
aaataactctatgttcatatcaacaaaagattataattttacaagtacaataatattcagtagtt
ttcatgagttagatagttgaagttcagtattataaattggcttgaaaacttttgccactgctgat
catgcttaaaactaaatgaccaaatgtaagttgaatcctaactatgaatttcactaatagatttt
agtgc

>chr9_23183200_23183400
ataacatgagctgatgccaggtaaggttgtgtgcacctgtagtcccagctactcagaaggcttag
gcgagaggattgcttgagcccaagagttcaagaccagcttaggcaacatagtgagaccctgtgcc
aaaacaataataataatatgtactgagcccaaatatatcagtcacattaacaaatataaataggc
ttaga

>chr9_24732200_24732400
ctttcttaagctcaccttgcctcacccccttatttccactgtcagctaaccacaaatctttttca
aatcaacatctcagctctcaccaaattaattatttaggttcagataaaaattttatttacaccat
ttttgttcattcattcttcaggtgtttttaatctctcagtgtaaaacatttctaaacacagtaaa
gccca

>chr9_41650800_41651000
aaatgtggccaaggagtcagatatgtcaattctgcgtgaattaatctgtttgtgcaaatgatact
tatcattacctatgatcagcacttttagctttatcatatcataatttacccaatatgtataggtg
tcaacatgtgtgaagataggcaccagataagcaagactcattaagatgttgccatagattaatcc
tacag

>chr9_43547204_43547404
tcctcaaagacatggctaaatctaaactcttaggtgcctggaaattatttactaatagaagtaat
tagagaagaaacctggtgctttgttccactacagcgatagctgctgggaagagggcacctcttgg
atgcatagacatcagggagcagtgtctggagctgggttcttttttttctgagatggagtcttgctc
tgttg

>chr9_45129804_45130004
cacataaactacacatgcaaactatacacacacatataaaccccacacacaaagcacacacatgt
aaactacacacagaaatgatacacatttaaaccacacacacaaactacacatacataaactacac
acagaaactatacacacacatataaaccacacacacaaactacacatacataaactacacacaca
taaat

>chr9_66389180_66389380
acactctcagtttggaaaagcgatagacactcaataccagcccagcccataagggcagccatgga
ggctaaagcctgcaaagccacaggtgcactgccctggtagaggttttccatgagcctctacctct
gcagcaggctactccccccttcctactacccaccaccctccgaccaccctacagccagcatactgt
tcccc

>chr9_72228580_72228780
aaggcctagaaaggcagttaccctccaacataatgtgatttctacatgggtctgaattgctccat
gtaaccaggaaacttcccatgattttactaataaccacatattcccctgctcaagatatagtttt
cttgtttacatttctctataatacggtatacattcataatctttggacacacactcttcctgtgt
ctgat

>chr9_73678580_73678780
aagatggctgaggggggctgaggggagggtggctgtagggactattgcaagataaagctggaaatg
tagactgagaacacctgagaatgcagtataaaaagactggcattcctcttgctgataaagaaaca
ccgccaaaggtgtctgtggagaagtggacctttttttccaggtgacatttccagagttaaggtgt
aagaa

>chr9_76831180_76831380

gtactgccttgataattttggacaagatcccacagaattctctgggttaccaggcagagactctt
gaattattcacttactttctcccaaacaaacagaatctctctctcagttctgaactacctaaagc
taggagtggagtgaaacaagcacctctgtggccaccacaactatgattgcattggatcagacctg
aagga

>chr9_78208380_78208580
gctcagattcaccctactccttttgcttcacccattttcctcattgacatggtttttttgactgt
ctgtctgatgatctaatcttgctatccctctatccttatccatggagtaggctacagggagcctg
cggtctccatttgctatagtctgaatgtttgcatctcccccaaattcatatgttgaaacataatt
cccag

>chr9_87121180_87121380
ttggggattacaattcaacatgagatttgtgtggggacatagatccaaaccatatcacgctcaac
cagcatgaaaacagcctggcagtgaggagaggccagtgctcctaggaccagctctcaaaggaagt
ggacgcctgaaaataaatgctccagaagccctttgctcagatgggctattctgggagctctcaga
atcgg

>chr9_103440779_103440979
ttcctctctttctatttgggtgccttttatttgttcctcgtacctgattgctctggctaggactc
ccagtactaagatgaataggaaggcacaccacattttttgaaagagaaattatatcagatgaaaca
ctttctaaaagaacagtaatttagaggggttttgtccggtaacatattacaatgtgttacaaatc
agaat

>chr9_104483379_104483579
acccaaatctcaacttgaattgtatctgcctggattcccatgtgttgtgggagggacccagaggg
aggtaattgaatcatgggggccagttttcccattctattctagtgatagtgaatgagtcttacaa
gatctgatgagtttatcaggggtttccgcttttgcttcttcctcattttctctttctgccaccat
gtaag

>chr9_107244979_107245179
agctcactccggctcagcatcctaggtgtctagggcttgcttccccagagaactgcaatactgca
caggacccaatgcctagtgtctgatgagaagtccccaagagggagagaggacacaggggaacaac
cagactagaatgatttcctctgtgtcatgttcctccagtgccactacctctaaccctgattctcc
ttctc

>chr9_121132179_121132379
ccatataatatatgatctaccatttctatgaaatattcatagtaagtgaataaatagagacagaa
agcaggtttgttattgacagaggctggagggagtagagaatggggaggtactgcttaatgagtgt
ggggttagcttttcttatttatcagaacctcctacttattagctatgtgaccttggataatctgg
tgaac

>chr9_122207179_122207379
aataattacagctgacttatttatcaacataataactaacacttaccaggtgtccattgtgtgtc
agtttcaaatgcttaacacgggttaatcttcacaactactctatgcagtgatactaatattatcc
ccgtcttaccaataaggaagttgagatacagaaacattaaggtacttatccaaggtcatattgct
gataa

>chr8_5329192_5329392
atcttgatacaagcattctgtaaatatgtactttgctcttagaaaaggtgttagtgtacttcctt
gtagctatgtcatttgtatatcctggtaaggggttctgatgaggttacttttcttgttttccgca
cttcaaccaagtaaacgaatagaaaatatttcaggcttactaacttttcctttcccaatatggta
taatg

>chr8_52596047_52596247
tggggagaggggaatggggaattagtaagaagcagacataaagtctcaattatgcaagagggaag
agatccagagatctgctgtacaacattgcccctggaattagcaatgtggtattctacacttaaaa
ttttgttcataagtaagatcctggaccaaagaagctcccctaagaggtggtttttttcttgtcttc
tcctc

>chr8_53271847_53272047
actgtgaatctactaaatgccactgaattttacactttaaagctgttaactttagattatgtgaa
ctttacttcaattaaaaaaggaggaggaagaggcaaagaaagcgaacctgccataaccacaatga
acaaagttgatgggtttcctgctctaatagggcttaaatttgaatggcgtagacacataagaagc

aaaga

>chr8_85568845_85569045
tcaccacctcttccaggtccaacatttattatcatacttgaaaagttaatacaaacataccaag
ttacccaaaattgagtgtagatttcttgataattagagtaattgttaaagttaaagcattatcta
cctgagataaatatacttgaaatatatataaaatgagaaaaaatatattttaaaatgcattgggt
ttaag

>chr8_93643024_93643224
tccaacatatgaattttagagggtactcaattcaatccataacactgacctagttgaaaggtcag
gtaggctcagctaagaaaggcatcaacaaatagagtagcagggaagacattggtggccatatatg
catttgattttcacatatttgacatatgttataaatgcaacatgctagccgctggatatacagac
atgga

>chr8_98396624_98396824
cttcccacagctccatgaggcagtgccccactgagaactctgtgtaggggctccaacctcatatt
tcccctttatactgctctattagaggttttctgtggggcttctgcccctgcagcaggcttctgcc
tgagcacctaggccttctcatacatcctctgaaatctaggtggaggctgctaagcattcttcact
cttgc

>chr8_110188424_110188624
tccttcttcaaagggacctccctttcattgaaggagtttagggtctgtaaactggctcaaatctg
gaaattaattgagtgactatggctttgttcttatctcagtgtagactttttttcacttggactag
aacttttctgtttatacagatcaaataagaatgttagaggtttcttatctatttcacttctagga
acacc

>chr8_110948824_110949024
tgacatggaaatccatctattatatacagtcaatgggattgcaatacccaaatcttcctctcaag
gaaaatagatgaggagatttcttggtcataatcagagattgcaatggtgttgaataggcaacata
atatgaatatcctcttaaatataaaggccaaactaaactgatctgtttttatccatgcaactatt
tgtta

>chr8_111957424_111957624
ctccaatccacggttgatggacatctgggttgattccatgtcttttgctattgtgaatagtgctg
tgctaaacatatgaatgcaagtgtctctttgggagaatgatttattttcttttgggtaataaatt
cccagtaatgagattgcaggctgaaatggtaattcgatttttacttctttgagaaatcttcaaac
ggctt

>chr8_115346824_115347024
taattgtaaattgtggactgaattttgaattacttattttttaaactaggtttttttttctat
ttttcaaataccataaatacacaataagtaatacgtatatagtgtcaattgaatcttgttttcaa
gaagataaattcacaccattaatggttaatgaatgatatctagctgcataaagatatctgaaaaa
tgcta

>chr8_120335019_120335219
attccatgtctttgctattgtgaatagtatggtgaacacatgagtgcatgtctctttttggtaga
atgacctgtattccttaagagtatatacccagtaataggattactgggtcaaatagtagttctaa
gttcttcgagaaatcaccaaactgctttccacagtggctaaactaacttacattcccactaactg
tgtct

>chr8_121099019_121099219
ttcattatctctttaggttctctcttcaagctccactttgtccccacataataatccactgtcac
caaggaaatgtagccccgtccccaggtctcattgcttctctcatggagtaactgtgccaggttag
gctgactccactgtaggagctcagaaaatgacaccccaaaatacaatgctttggcttgttgagta
cttag

>chr8_121911819_121912019
cattctatgaagccagcatgaccataataccaaaaccaggaaagggcataaccaaaagagaaaac
tacacaccaatatccttgatgaatatagatgctaaaatctttaacaaaatactagctaaccgaat
ccaacaacatatcaaaacggtaatccaccatgatcaagtgggtttcataccagggatgcagggat
ggttt

>chr8_140536418_140536618

aacccagccatcttagggcataattggttttcaaatcaagtacatgaaaagggacaatatttaac
agtgaaatctcattagaaaatggaaagagattagatttgatcattccacagaattgtgggatcat
agttagagcttcccccagagttcatgagtttgaaataagcaagataaaaactttagggggaaagc
tgtca

>chrx_23051879_23052079
caccatgttggtcaggctggtcttgaactcctgacctcaggtgacccacctgcctcgtcttccca
aagtgctgggattacaagcgtgagccactgcgcccagcccgtatattcttatataatttttgagt
ggttgctataggaattataacatttatactaaacttattattatctacatagaatcaacgttcta
ccact

>chrx_28391879_28392079
agttagataattcgttgtcaaagctgatggaattctggataagtgttagcaacctttgaggaata
caagtgagattaatacagttaattggtagttgtcatttattcccattcttttctacatactttca
tataacacaatgtagaaagctaaaaactacattatctgactctcttcaagctaggcctctaaatg
gcagt

>chrx_31586079_31586279
gatgttttattttcattttatttagttctttgtgtttaaaaaatttttctttgcaacctcctcct
tgacctggggaaaatgatttaaaggtgtgctgcttcatttccaagtgttttgaggtttttctttt
gtctttctcttattgatttctagcttggtttcattatgttctgggaacatataagttgaattctc
taaat

>chrx_32524079_32524279
ggaaaaggtaaaactatgtaaactacaaaacaatcagtggtttctagagattaggagagaagtgt
agatgaacgggaaaagtattgaggatttttatggcagtgaaattacaccgcataacactctaaag
ataaatacatgtcattgtaaatttgtttaaactcacagaatgtgtggcaccaatagggaaagcta
atgta

>chrx_42083456_42083656
cttgttccgttcttagatgaaaggctttcagcttttccccattcagtataacattagctgtggtt
ttgttgtatgtggactttattattttgagatatgctccttctatgcctagtttgttgagagtttt
tatcataaagggatgttgaattttatcaaatgtttttttctccatctattgagatgatcatatgat
ttttg

>chrx_49408243_49408443
gtctttagacaataacttaactctttcaaccaattgccaatcagaaaatctttgactctatctgt
aacatggaaggacctccacacttggaattgtcctgtcttttctgaccgaaccaatgtacatctta
catgtactgattgatgtcttatgtctctctaaaatatatataaccaaactgtaccccaaccaccg
tgggc

>chrx_51392260_51392460
gctggaggcatcacactacctgacttcaaactatactacaaggctacagtaaccaaaacagcatg
gtactggtaccaaaacagagatatagatcaatggaacagaacagagccctcagaaataacgccgc
atatctacaactatctgatctttgacaaatctgagaaaaacaagcaatggggaaaggattcccta
tttaa

>chrx_51919260_51919460
aatgctcatcgtcactgcccatcagagaaatgcaaatcaaaaccacaatgagttaccatctcata
cctgttagaatggcgatcattaaaaagtcaggaaacaacaggtgctggagaggatgtggagaaat
aggaacacttttacacagttggtgggactgtaaactagttcaaccattgtggaagtcagtgtggc
aattc

>chrx_56194275_56194475
aagttttaaagcaaaatcaaacatatattacttagcagattttaaatgagcaaaccaaagtggat
aaattatttccatcccatattcagatatttttaatgctgaattttcatagcaaataccacagtat
acccataaatggagctatatctccacatatttttccaccagtggagatttttatcaatttgaaaa
gctgc

>chrx_62820075_62820275
ccttcagttctgctctgattttacttatttcttgccttctgctagctgttgaatgtgtttgctct
tgcttttctagttcttttaattgtgatgttagggtgtcaattttgaatctttcctgctttctctt
gtgggcatttagtgctataaatttccctctacacactgctttgaatgagtcccagagattctggt

atgtt

>chrx_67588275_67588475
catgaggctgaggcagagaatcacttgagcccaggaggcagaggctgcagtgaaccataatcaca
ccactgcactccagtctgggcaacacagtgagaacccccatctcaaaaaaaaacgcaaccatgta
tatggggaatttagaaagccacatgcatgccccagaaaacaagcatactcagaaaattcctgaga
ggact

>chrx_72268875_72269075
ataagcccctactttaattggaggaccgtaatgatgttttgggtcatttggagtcaatgtcagct
cagagctagtgtccactagtccccaaaaggtctgatcattcccctttccccagtgcagagttacc
ctggtaaaaggctgtaggtcctcttggggaaagatgggagaaagataaacattataaattgtcgg
tagta

>chrx_86582544_86582744
gctctgtatttctctagggagaaaatcccaaatacaacccaaaagctctctgccattgacactgc
agtggtactgcacttgctgcctttgggctggagaaagaacaaagaccctgatgactttgttggca
cctccagcatgctgcagccaccatacagagaggagcccagtctctcttccctgtgagtcccagac
ttcct

>chrx_90388144_90388344
tgggtggattatttgagatcaagagttcaagaacagcctggccaacatggtgaaatcccatctct
actaaaagtacataaaaaaaattagctcagcctgctggcacatgcctgtattcccagctacttgg
gaggctgaggcagaagaattgcttgagcctgagaggcagaggttgcagtgagctgagattgcacc
actgc

>chrx_92207344_92207544
atatactggggttagtatatattagcactatctgttgaaaggccttagaaacaatgagatgccag
taacaacaaacacatctagtgcccaggagttgttgtctaattccattcttcaacaaaaagaactg
aggatccttggagaagaggatggagctaggcctgaggcagaaaatatataggattggcctggaga
agttt

>chrx_94211544_94211744
tgaatgttctgcttaaaagatggcatctgcctgatacttatggttatcagaggctatgaagggtg
tatgtaagttggggagtggggatgaagagaggctgggtaatggatataaacatgtgattagatgg
aagaaatatgtcctagtgttcaatagcccagtagggtgagtatagttagcaacaatgtatgtaca
tttcc

>chrx_95364744_95364944
tagatttgaaggcctgagaatattcctctgtggcttgatgctctattctctgcatctatggctct
gcctttagagtaatttttttcttcactttgtcctatttctttctcaatcagtccaggctggcaac
atttttgttagtataaaattattttaaaatgtattggtctcctgtccaacttatggtgattcaa
gccat

>chrx_104496344_104496544
cactgggctccatgtttgaggacttggattctgatcaccatactaccattaacatggataagtaa
ttaaatatcactggacctcaatttccccacatataaaatatatgtaattaatagctgttatatac
cctagacatgttataaaagtttaatgattaaagtctttacttgagatttgctacaactatagaaa
tggaa

>chrx_109912744_109912944
gaaaaatcgatgtataaatctaatatttatcatttattttgaaagacatcttatggcaaatcagt
ttacaaagcaaataagaccttttttttcatttgtacttattaacacaggttttattagttttcttt
ggagtagggtacagagaaaatgtcctgaaaatctgcctagcctctgcatatttatgtattccctg
aactg

>chrx_118661572_118661772
atgtgtcactttcttttaaatattaatacctcatatatttttcttaaaaaaaaactttttaaaac
tagagacagggtctcaccatgttgcctaggctgctctcgaactcctgggctcaagcgatcctcct
gccttggcctcccaaagtgctagaattacaggtatgaaccaccctgcctggctcccacatttttc
ttaat

>chrx_124363119_124363319

ataatggagtcaaaagataactatgtcccaatccctggaacatgtgaattttaccttatgtggca
aaaaaaaaaaaaaagactttgcaaatgtgctttaaaatattgagatagggagactatcctggatta
tctcagtaggacctaattgtaatcacatgtacccttataagagggagacagaggaaaacgtaaca
tgcgg

>chrx_124389319_124389519
gtgacagcgtgctggcagtcctcacagccctcgctcgctctcggcacctcctctgcctgggctcc
cactttggcggcacttgaggagcccttcagcccaccgctgcactgtgggagcccctttctgggct
ggccaaggccagagccggctccctcagcttgcagggaggtgtggagggagaggcgcgagcgggaa
ccggg

>chrx_127070719_127070919
ggggaaatgaccacaggatgagtccagggacacactggacccagtgtaagtcacacctgagctaa
aactccattaattatctgacctctataagctcctactttaactgtaggaccacaatgatgttttg
gatcccctggaatcaacatcagctcaaagccagtgtccagtagtccccaaaaggtctgataattt
ccctt

>chrx_137766134_137766334
tgtccactgttgtaatcacgagcaggaaataactgttcatttccctaggtatatcccatctattt
caaatatgatcccctggctactcagtactaaagagcactcattagatctatattctacataacct
cattgctgtagattttattaaaaatttaataatgcatgcaaagttaagggatttattacttgcaa
aatag

>chrx_140465734_140465934
acacatggacacagagggggaccaacaaacaccagggtctcttatggggtgtggggcgaggggag
ggaacttagaggaggagtcaataggtgcagcaaaccacaatggcatatatatacgtatctaacaa
acctacacgttctgcacatgtatcctggaacttaaagttttaaaaagccattttaataagtgtgc
ggtag

>chrx_141127734_141127934
ctgaggccttcccagccgtgtggagctgtgagtcaattaagcctcttttctttttttctttttctt
tttttaataaataaacattttttaatttgagagagttgtagattagcctcttttctttatcaac
tacccagtcttggatatttcttcatagcagtgtgaaaatggactaatatagtaaactggtagcag
atagt

>chrx_146081708_146081908
aggagctgctaaattctacaacacctaatagctcctacctaacaagagctgcttaacatgtaaac
ggcagtcccaaggtgaacagatgtcttgtttggaaggctgctattctggttaaagggtcaagaaa
atcattttcttttgagttatttgggtgaagtatgtttttgtaaacaaatttacctttctctctga
gttct

>chrx_146478108_146478308
tgaagtctgtgcttttagtgtaactatcacctgaattgtgtacattgtacccattaactaatttt
ttatcccatgacccactccaaacttcataccgtctcattctctcacccttctgagtctccaagtt
ctattattgcactctctgtgaccatgtgtacaagttatttatctcccacttataagtgagaacat
gcagt

>chrx_151663944_151664144
agttaagtgtggttggagctgggtcacatggatgctgctggatctgtagtgaagtctacagttgg
taggccttttacttgaggctcagttgagcatgaatcctatgtggtccctgggaagacaggactgc
ctccaggaaattggtcagtggggttggtgctggaacaagggcctacaattggatctgcagttgga
tccac

>chr13_26298800_26299000
tgctgtctagtccttgggtcatttcctgggagtaaggaagagaaataaatcccttccagtaggaa
tatcttatggtaggcatggaaaagtagaagaaaatgaggtatctgtttttatgatgtcctaggcc
cattgaccacatcttgggcccacaaagggtgtagacaattgagtgagaacaatcagtataaagtc
tagat

>chr13_34158200_34158400
ccattttacactcccacaagcagtgcacaagggttcaatttctccatatctttgccaacacttgt
tattttattttgtcttcttttccttttgatagtagccctctgtattagttcattctcacattgc
tataaagaaatacctgagattggataatttataaattatgaagaaaagaggtttaattggctcat

ggggt

>chr13_38680400_38680600
actggaacatccatattcataaaacaagtttctagagacctataaatacacttagataaccacat
aataatagtggaagacttaaataccctactaatagtgttagatagatcaatgaggcagaaaacta
accaagatatctgggatctaaacaggaacatctgactaaaatgattaaacaaacatgtacagaat
tcact

>chr13_46997999_46998199
tttacgctataattcacaaatcagcccactctgaatgggattctctgcaacaagaggttctagaa
tctgcccaacttgaaattcaacaagcagtctcatgagtatcccctaaagatttccataaaggaga
acttctctgtttttaatctattgtcagatgaaatcacgctgaagaaaatcactttctcacagaac
aactg

>chr13_62539799_62539999
cttaagttcatttatcatgtcatggcctccataaatcatatacattttaccacattagatttgat
gtctaggatgcacataatcaagatgaatgctgctctcataattatagcttcaaacctgtgattca
atgggctgccataatcatctttacgaaagatcaccccacatatactgataattttatacaatttg
cttaa

>chr13_68063999_68064199
aaaaagaaagaaatgcattatatattgaaatgatatgataaaactcaaattattttaatcaaaat
aaacatatcttatataaactattaaaccaacagtcttatcaccatcaaaaccaccattgtcaaga
tcgtcaaatcttcagcctctcagttctgttggtttattctcaatcctcttcttgacttcttattc
aaggc

>chr13_69162399_69162599
catagaaccacaatggaccccaaaaacttgagaaaaatagagcaaaactgaaggcatcatgcttc
ctgattacaaaatgtattgcacaactacaccactacaataatgaaaacagtatgttactggcata
aacacaaacatatagaccaatgtaacagaatagagagctcagaaataaacccacgcatatacggt
cagct

>chr13_74985399_74985599
acctgaaagatgaaaaaaatattcttaatttgattcttgctaatttaaattctcaaccaaattaa
ctttagtgattaaatttcacacgtaaaaatgtttaacatcttatttcttagttggccttggaagt
gtttctagattattctggctcttctgcagaaggtctcaaaaagaaatgtggtaatttatcttctg
tagtc

>chr13_82489399_82489599
gaagaatgaaataaaatagaaaacatattacaatgtaaaatgaataagaaagaagtaaaatttca
gaatattttgaaatgctgacttcaatggagtagaggatgatatttactcttggccgaagtaaata
gtaaatgtattattttggagtgacaaaaagtttagttacaggcctggcacggtggctcatgcctg
taatc

>chr13_84044799_84044999
gtttgtattcgggtcctcatatgtcttgactggactattggaaatcttctgtttatttcaagatt
tcgattattttgctattctaatcgaatttatatcttaccagaattatcttcctaaattataaatt
tgaccttgttattccagttttcaaaaaccaccagctatcctgcatacatttaaaaaagattgtca
gcatt

>chr13_90359199_90359399
tttattcccatttatatccataaaatataatccttattttaattttaactaaataaaataatta
taattacttttacagtgctatttttccatttcacattggagaattataattctatatatttatgg
ggtgcaaagtgatgctattatatatatatgtatatgtatatctatatatatatatacacacacac
acaca

>chr13_105910799_105910999
gtgataaatgtgacctcttcttttaatttgtcttcacaaaatatgaaacatttccaaatagaaaa
tagtttttgactatctgttcctgcgtaacaaagtacccccaaatttactggcttaaataaaagca
atttgttcatgactctgagactaggaactcaggttaggcggcacagttctgcttctccaagtggg
gttca

>chr12_13484333_13484533

atgtaggtactagaagattcagtgtctggtgagaacacatttttgattcaaagggacaaatgag
ctttcttgggcttctttttagggcacaatcccattcatgagagcagagcctctcatgaataatc
accacctaaaaggtcccaccttgtaagaccactgcattgggggttaggatttcaacatatggatt
ttgaa

>chr12_14053333_14053533
aggaagcggaggttgcagtgagcagagatcacgccattgcactccagcctgggcaacaacagtga
aactccatctcaaaaaaaaaaaaaagttaataaaacatttatgcacattgttccaaattctgaagc
aaatacttccataactatcacccagattaaagaatccataagcgtccaaaaatgtccctgtgtgc
ccttc

>chr12_14968133_14968333
tgtgattctaaagaagggctaagtggggtcacttttctacttttgtcccagtggatcatcttgcc
cttgatgtatccagggccccaccacgtacggatgtgtggccagtgtatctgaactaggtatggat
gttaagtaggattttaatagatgtttggggaggatggtggtatagaggaaataatacagatagag
ggaac

>chr12_17903533_17903733
tagagtaaaactaacacatttttaaagtaacagatataagtttaatagatgtaatttactaagta
aaagagtcaaaattccattatcacaaaaatgaacaatgatttcctgaagtaaaagctgatgagag
gcactcatttgatgtccagcactttgcccgttgttcctcccattgtaaacgcaggcatacacaat
tatga

>chr12_23186333_23186533
caggagtctctttctttgggaccaaggaatgtaataatggttaggcagctgtccttggctcatga
cactccctgtcaaaggagaagaagcagagagcaagtgcagtccctcctcccaagtcctctctctc
tctctttctctcattggcttttgtttgacctgaggtgccatctctgaagaggagcccagaattca
cagga

>chr12_24278733_24278933
agctttcttttggggatatttttctcaatttttattctcttctccacccacaccattctggtttc
tgtaccatcatccttcccaggacgaagagtggagctaggcctctaataaccagtgaatccaagag
ggcctttcagatccgtatcttgcttggcatctcaatggcatttcaatggccatgggcacttcctt
tccta

>chr12_55663933_55664133
tagctcaagaacattaaaactgacccaaagtagcttgttgaaaaatggatcgtgtatattattta
tttgtaaactgacaaagacagtgcaaaaacaacaaaaaaatctcagagatcaatatataacatag
atgtgcctgccaaaattctaaataaaattttagtaaaaacttcaacaacaaaataaataaaataa
accaa

>chr12_71799533_71799733
ttgagcttgcagtgatccaagattgcaccactgcactccagcctgggcaacagagcgagactcta
tctcaaaaaaaaaatggataaattcctaaaaatatacaaactgtcaagtctgaaatatgaagaaa
taggaaatatgatcacaataatagcaagtaagaagattaaatcaacaattgaagacctcccagca
aagaa

>chr12_75471133_75471333
tatggttctgaatgactcatacaataattacgttccacacatggtcatttcagtaatataacttc
acaggctgtctgtgcaaattttggtttgtttcctaatgggatatttcacagttcctcaaaaccat
aattgagaaaaacaaggtaaagtatctgatacaaatcatttcttggcattactcatttttaatta
tttat

>chr12_83482669_83482869
tattagggacttagaagcagtatctcaggcatccatgagatataggatgcatgaatccaccctcc
agaaaataccttttttagttagcaaatatttagggcaaaggcttgtgcagctggtcatatctcata
cttccttgccaaaatcatgaccacgggggaaactaaataagcacctttataaactatgctaccag
cattg

>chr12_98495069_98495269
aggctcttttctgaccattgacatggtgctgggagcctggccttatactctctccagagttccag
ggaaaacctgtccagaggcagcttctggttcttcagatgcaaaatgcaaatgcattaaaacacca
caatagggaatatgttcaaagatttgttagtcacagatcctgggcagagagggcaccatgagtca

gaagg

>chr12_99534269_99534469
taacattgtttgccagctaaatggaacaggcactcttgtcaccatcatgattcattgcctactga
aagcacagagctggccttaatctaattcttaataaaaccttgcgaactaggtatactgtctcaat
ttgcacacggaaaacagatgttcacgaaggttgagggactggcccacgatctcacaccaagtagg
taaag

>chr11_24960224_24960424
aatgataccatattaatattttcacttatttgaacccttttatttgcttgtggcttggattttaa
tggtagtctgcaggagtacggtagcaatcaaacacagtcttaggggttgcccattcaattctggc
tattttgataacaatacttagctttagctacatggaatgaagatgctttgttggtgtttgacaat
ttgaa

>chr11_37350624_37350824
tctaaatgtgtgtatctccctttacaaatgtgaggagacatcatttctaccacactgttaatgag
gaagcattggagtagggcagaacaaaacaaaatgtgaactttgttttaggtgaagttgtttgatg
gcagaaggagagatatggtagctgaatggcagtttgcaagtgtgtcatattcagagttctcaaat
tcctt

>chr11_37762424_37762624
gatatcaaaagaagattccatgaaaacattccagttgttcccagctcctagcatctggaatgact
tacttggtcacttgtcccagtatcactttgtagttagaatttcatctgatttgtactctacttaa
atattaacttgtaaattttcaattgcttaacactgtgtatgttctagagctatttcaaatcctta
atgat

>chr11_41066824_41067024
agaccatggggcatagataatagttggcatcagaagagaatcactctttcctcttatttcctttt
tacatctcttcaagctgtttcgttcccataagaatatttataacctcctggcatttgaaagcaag
ttagtggaaagataattactcctcaacatttgctactaaatggatagagtcatccagaccatgga
gacag

>chr11_49764624_49764824
aggggcaatgtctgatttatatagggctcatagattggtttgatcaggtatgatgtttacataga
gtgcagagaaggctggtcaccccaccttaatcttattatgcaaataggctttccggttgattggc
accatcttgtctgctctttacagtatacatggctggcagagaagggaagaatggagctgccatct
tgaaa

>chr11_49890024_49890224
gagttgataccattctttctgaaactattccaatcaatagaaaaagagggaatcctccctaactc
attttatgaggccagcatcatcctgataccaaagcctggcagagacacaacaaaaaaagagaatt
ttagaccaatatccttgatgaacattgatgcaaaaatcctcaataaaatactggcaaaccaaatc
cagca

>chr11_80694152_80694352
ttaattaaaatattaaatgtgctgcctgtacttatgtttatcatctgtgaaacattcatttttca
tacttattcttttgcagtttttgagagtattgttatgcaatgattattcccaatgccaagctatc
aacaggagtattttgagataaacaatgtgtttacaataggaactgtagtggtgtcttttgttttg
gaaga

>chr11_81173152_81173352
ttgtgttcttaactactgtgttctcgctttcttctgcaagttctaacatcaaggattctccattc
ccttggtgacagcatgcatctgcttgaagctgtctgagggacggaaggcccctctgacggccaga
actccagcatattttccacttagagttctctcagtcctcttaccaaagaagaagagagacctgaa
gaaga

>chr11_85793152_85793352
cttgattactgtagctttatagtaagtcttgaaatcaggtagtgtcagtcttcttattttgttct
tctccttcaacggtgagttggctattctgggtcttttgcctctccatataaactttacaattagt
tcatcagtatccataaagaacatgctaggattttgattgggattgcattgggaagaaccagcatc
ttgac

>chr11_87230152_87230352

atgctaaatttctgagtccaaatggaaactcatttctgaagtcacatgtcatcttgaaggtgtgg
gaacaaggcattgaggctttatctgaataaaatgatatcttagaaaacccagagcttccagaagg
tatacgatgagcagagctgtggctggcacgtagcaggggcttcatcatactcatgtgttagatga
atgag

>chr11_88921552_88921752
caatcattctatgagagttcttttgattacatagtttgttagaaggtgctaagtgcacagataaa
gcagaaattagtggtgtacagtaaatgtttaataacttgttctcaatggaggagtgggaaagtag
ggagagatgattctgatttgccaattgttgtggtgtaaatactgacaatggtagatttccatcta
atgat

>chr11_90127752_90127952
gaattttgctatgagataatcgttaaaagcatgaactcggcacccaagcagactggttttgcca
atttttacttgtcttgtctagaacaagttatttaacctctctgtgttccattttccttctctgtt
atgtatataatgatcttaactaacttattgagttactatgaagattatattatttatacaatgtt
tggga

>chr11_90936952_90937152
caggataatttatatttttaaaatattatattttaattaaaaatggtataggtgtctctagactt
tttgcatacatttaacatgttataaacatttccataacagccttgcatgctttctattatgcttt
ttctatacatgatatgattgttcctattataaatagtacctgtctgcaatgtattgactacatga
tgcta

>chr11_91754952_91755152
tcactcagggatcatagccctaattggcttcttcttgtacaatgtcagaaaacagttattattat
tatttttaatactttaagttctggagtacatgtgcagaatgtgcagttttattacataggtatac
acgtgtcatggtggtttgctgcacccatcaacccatcacctacattaggtgtttctcctaacgtt
atccc

>chr11_97899790_97899990
cactttgtgcctcagttgccttatatttagtgcattcaacccatggttgttgtaatgataaagtt
aatcagcgtttgtataccacctaaattaatgtctggtacagagttaatgctatctctctctttgt
taaacacatctgcatgaaagaacacagtgattttgttagtctcttgaagttctaaaaacaacaaa
acagc

>chr11_99050190_99050390
aataaaaaataaacaaaaacaaacataaaaaacaacaaaaaaagaaatttaactagttttaccta
gttcatatagatatttctaaagagttaatgtaattttatggttacatttaccccactcctacaac
tcaggatgatgccatatgatccatatgcccaaaggctgtatcaaaggataaaataggattatttc
taatt

>chr11_109223190_109223390
aaaaatattcgttttcctcacttcccttgtggctagggtagaggcatgtagctttggtgcttgca
agcagagtactgtggccaaatattgaactggaaactagtgcacaaagaaaaagttagactgggaa
aaactaccaaggcaagggtggtggcagttgcatctgatttttgaagcaggaacatttgagcaacg
ttgga

>chr11_114964790_114964990
caacttattccaagtggctttggggaaagagacagaaggaacacctagaaataagagaaagctac
ctagaaacatgtttaaggagaatatgagaagatgtttctaatggttatggttgcctaaaattgta
aaagatttcctgggtaggtaatgatctccctgatacatggggaattcaaaccaaggctgggcagc
cacta

>chr10_25054194_25054394
ggagacactttccccgtggtcttggggattaatattaggctccttactacttatgcaaatttctg
cagctggcttaaatttctcctcagaaaatgggtttttcttttctactgcatcatcaggctgaaaa
ttttctgaacttttatgctgtgtttcccttttaaaacagaatgcttttaacagcatccaagtcac
ctttt

>chr10_36578394_36578594
tttatgacagcacaatgccattttggatactatagctctgtagtaaaatttgaagtcaggtaatg
tgattcatccagtttcggtggatgtttctgtttttgattttgttttgtgcttaggatgactttgg
ctatttgggtcttctgtcattctacatgtattttaggatttttttctatttctgtgaagaatgac

attgg

>chr10_37984594_37984794
tcactcaaaaccactcaacgacattgaaactgaacaacctgctcgtgaatgactactgggtacat
aacaaaatgaaggcagaaataaagatgttctttgaaatcagtgagaacaaagacacaacatacca
gaatctctgggacacatttaaagcagtgtgcagaggaaaatttatagcactaaatgcccacaaga
gaaag

>chr10_49012194_49012394
tctcaaaattttgatttagaaaatgaggtattttagtatgttaattataggcatctcaactgaga
agtctaatagcattgggccagaattgtcgcagcagtccaaagccatggataaacttaagttaaag
ggagcaaatactaacagccttccaaattaatctgtattggagaagatgataaacagatgaatggg
aacta

>chr10_57890794_57890994
tcacaggcatgagcaaagactttatgatgaaaataccaatagcaattacaacaaaagccaatatt
gacaaattggatctaattaaactaaacagctctgcacagcataagaaactatcatcagagtaaca
ggcaacctatataatgagagaacattttgcaatctactgatttggcaaaagtctaatatccagaa
tctac

>chr10_69203594_69203794
ttgcttttggtgtttcagacatgaagtctttgcccatgcctatgtcctgaatggtaatgcctagg
ttttcttctagggtttttatggttttaggtctaatgtttaagtctttaatccatcttgaattact
ttcatttcagaattggacagatcttccagacagaaaatcaacaaagaaatgtaatatttaatctg
tgcta

>chr10_100302410_100302610
ccaaggctcgagaactacgtgaagaatgcagaagcctcaggagccgatgcgatcaactggaagaa
agggtatcagcgatggaagatgaaatgaatgaaatgaagcgagaagggaagtttagagaaaaaag
aataaaaagaaacgagcaaagcctccaagaaacatgggactatgtgaaaagaccaaatctatgtc
tgatt

>chr10_109600610_109600810
attttgaggtaatatttcactggggttttatttgaatttccctgacaattcattatattgaaatt
ttttttcatatacctgctggtaatttgtacatcttctttgggaaaaatgtctattcaggttcttt
gctcagtttttaattaggctgtttgttgtcttgttttgttgctattgtgttacattaattcctta
tgtgt

>chr10_113814610_113814810
aaaatgatttcacatcacaaaaagcagccttcctctcttcagacacaagttacagattattagct
gttcccttgtaaaatgcccaactcagggccttcctctgcattcaagggcagcctcatatgggccc
agttccttcatgttttgatcctatttcccctcatgaggataccctccacatttagcaaggcccct
tcaca

>chr10_114114810_114115010
ttcatcccaacaaccaaacaaatacaagtcattcatttccctcattttcttgctgtaggtaaggt
aggctgccatacttgattctttctcattgacttgcattatcactgattaatttattatatacgcc
aaagaaaagcctactcaaacaattgatagttttctatttaaaaccaagcatttgtttcttcttct
tcttc

>chr10_130506210_130506410
tcaggagaaatattagccactgctaagtaatttacttttctttacaatggactcaacaaacttta
tctgtaaagagccagatagcctcggggcatattccaccctgccatggtaacgtgaaaacagccat
agataacccataaacgaagggccgtggtgttcgaacaaaactatattggtggacactgaaattgg
aactt

>chr10_132179610_132179810
ccatcttgaaatttgtaataattttgaacaaggaggtttcatttccttttttgtactgggatctgc
aaagtatgtagctcgtcctgaagctaatgtatttttggggtccacgtgctcaggggcccaaacct
tgtacctttagcaccctcagtttcccagtccagctctgggccctggaattgcatcagccttgctc
tcagg

>chr10_133174810_133175010

ccctgcgagtgaggttcactttgtgatgtgtcccatagtcccgccacatgggtctcaaactggcc
tgatctatgctttggaatctgtttggcctgcagttcttgaggaaggtaagttctcaccagggtca
cccagaagggtacctgattttgtctcttctggcctgctgctctagatggaaggggctacctggca
gctgc

>chr17_9491075_9491275
atacttttcagatgccaagtttgcaatatttgcttattttctgcaaggcgtatttccagaagtg
acaagggccctgtcccctctaatgttctctcttggcccgtctgtatttcagtatgcattgtctga
aaagcctctgagaaaaataccataaaagtggttttcaaagttggctgcatgttgaaatcacctgg
gcagc

>chr17_69851805_69852005
ttaccttctggaaacttccatgaaaataagccacttccctcattagcataattttctaaatatga
caaagcatctcattgaaaacaaaataaaacaaaagacaaagagtattcatctttttcaagtacaa
ttgccgcttctgagtggcttagtagaagtgttaaaatagcttccatttaatagtttataataaca
ataga

>chr16_61817299_61817499
aaaaagaaatgagcaaagcctccaagaaatatgggactatgtgaaaagaccaaatctacatctca
ttggtgtacctgaaagtgatggggagaatggaaccaagttggaaaacactctgcaggatattatc
caggagaacttccgcaatctagcaaggcaggccaacgttcagattcaggaaatacagagaacgcc
acaaa

>chr16_74051299_74051499
cattccaacgtttctaacttcctgaggagttgaatgcctagtgtggcatctagcacactcactca
cttggggctaccatattgaatccagaatctgtgctaagtcaatccatatcaacaaattcagccaa
atttaaaattatactctttccttgttcatttgccttgagaatccatttagcagatttttgcaaat
aaaaa

>chr15_39057108_39057308
aatcatttctgcataactagtggtaagtggagcactcttgaggaaacatggctccagggctttca
tgaagcaaccattttccttactctaaaacatccttactgtaaacatgatgtcaatatttctcaga
tttctccattaaagtactatacatggcagaagagaggagacatgtccccccttccctgggtggtct
atctc

>chr15_47377108_47377308
atcccatgcaaccaatttatgaacattttgcagaatcagatttccccgctgcttacaattttgta
ctttttggtaagctaagtcagttaccagccattcatggacttcctattttcaaaaatgttgctgt
tttccttttatatcacttcaataagccatttgccatcatgttatagtttcaggtgggaaaataag
tctct

>chr15_98250996_98251196
attttccagtgattaaggcttaatactttgagcagtcacacatacccgtttttttatatttttgg
atggaactcttgctaacagtgagatctgttattaacacatacttacatattagaagaattggaat
tgcctctaaggtatcattatactgaatattaaatttagccttagggtgcaataggagtgtctcca
ggaag

>chr15_98476996_98477196
aagacagcaaaaagaagcaagtccctggacccagccaatgcaaacatttattcctcctaggcctc
tgggcctgtgaagttagggctgccttgaagacctctgacattccctggagacattttggccattg
tctgggttattaacatttggctccttgttacttatgcaaatttctgcagccagcttgaattcctc
cttag

>chr14_19522000_19522200
aaagtcagcttaattaaaagggtaacatccagatgtgtgtgcatgtgtgcgcatgtttgtatttg
aaaggccttcatgtttttttgtttttttgtttgttttactctcctaagaccttgtctttttgttgt
tgttgagcaaatgtgtttatgtttttttttgtcttttttttctcagttgactgaattctgttttca
cttga

>chr14_25978760_25978960
ttgatgattcaggatgagtagaccaccataaattagccacctctccttgttctaacatcagagca
tctttaatgtagcattactagttcctggatttctcaaaggcgaggagacccacatacagtgctga
gcagttctcttgaaatattcctcacttggctcaccttgccctctgacagggggcaggacctcatg

aaaca

>chr14_26296960_26297160
agatacacagaatattgatgctaattattacatccatccaataagcagaaggaagcctacggtta
tcagtgaaggtctgtcccggattctggaagactagtaagaccttacctaactttctagtatcctt
ccttccttgtttttcccttcatctcccttaatcatcctttgagagaagagggctaagtaatgcaa
aaaac

>chr14_27813560_27813760
gtgactttcctcttccttcaccttctgccatgattgtcaggcctcctcagccatgtgaaactgtc
agtccattaaacttacttcttttataaattacccagtttcaggtatgtctttattagcagcgtga
gaatggactattacaagaacatatggagaaactaacaattttatttagaattttttttttctaat
gattg

>chr14_42833850_42834050
gtgaattcttatttctcaatatcctgtgaaagttatttcattgataaattctacctaatttaccc
caatgtattccctactgtgaaccaacaaggaagactaaagagtaaggtttcttgattgacaaaat
aatactcataaatatacttgcacattcaagcacacacacactcacacacatgcacattcatacag
ttcct

>chr14_44504050_44504250
cacaatctcccttctcccaaacacacattatttctatcgaattatcatttgttttttttcctc
aaggcacctattgcataacagtagaaaatatatctgttatttatacatttctctaattgaatgta
agctctaccgtgccaatgatttcatttttagtaccggatcataattgcctaggaaaaaaaccgga
atatg

>chr14_47418250_47418450
tttaaaacactttcaaactgattagcttatttgataatcctaaaattatgtgagccatgggaagc
agatttatcatctcccattttacagatagaggaagcaaggccacaaagaattaaatggcttgcac
agagctaatctgttagaagatgttaaaatcttccattcatgggagaactttctttacatactgac
caaga

>chr14_48431650_48431850
attaaaacaaaataatacgtaacaatcctaatatcaaagagggcaggtggataaggatgaattgt
catcttttgtgtctttatagtgatttaataaacacgttgaaaaaatacaaaatgttttaattcaa
tcaactaaaattatttagtaaaaataaatagctcaagaacatttagtcacctctgtgaaagccaa
tatag

>chr14_66967247_66967447
attttaaaattaaattaaaagagcatcatttgaatgttttaaaattatgggtttgttttttttt
taaccactacagtgagtttgaattcgtatatgacctccctccttttttttttttttcttgtttgt
tgttaactaaggcatggaataaagtagtttacttggtagctgaggttgtaacttcttaaaaagtg
aatta

>chr14_72834047_72834247
ggaatgtgtaagacccctacagagtgctctctagcctctgttttaacaatctgcaatgatggatg
tcatctctccttccatctttggacaactttgaccttgagaaactttttttttgtaataaaccta
ccagcctcctcttgcttcactgcattagtcccaattctacctctgtatcacaaagaatagaggaa
tggat

>chr14_98981047_98981247
ccactgggaaatggaaaacatcaacttttaaccctagtttttcaaccatagcactattgacaatt
aggcctaggtaattctttgttgccataggggtctgccctggacactgtaggatatgcgacagcat
ccctgaactccacccactaaatgccaatagcatctccatgcccttagtgtgatagccaagatgtg
tcttc

>chr14_107076355_107076555
cttagccaaattaattgtgttgtgtctcaaccattgtatatcactaaaaacaattttactcttct
taaacagtgccattttttacttattttatacccactctctaaattccttgaatatcctctatatgt
ttaccggactatagttttggctttgaaagaatttcaaataaatgacattatacattgtcattgaa
atgac

>chr19_4613400_4613600

ttttagcctataggttcctatccatcgctttctcttcttttttttttttcgtgtaatttatttgct
gatgatgcattattttattttaggtgtattcaaaagaacatacacatggattttgtttattcttt
gttgtttctatgtatgcaatctgacagcctatgtcttttaagcgatcagtttaattcatttatat
ttatt

>chr19_22553760_22553960
ctatttttagtagagacggggtttcaccttgttggccagtatggtctccatctcttgacctcatg
atctgcatgcctaggcctcccaaagtgctgggatcactgcaagctccgcctcccgggttcacacc
gttctcctgcctcagcctcccgagtagctgggactacaggcatgcgccatcacgcccagctaatt
ttttg

>chr18_4033600_4033800
gtagagcatatgcattttctatatctctgccaaaatatttctattatcaaatttaaattattttt
ttccaaataattcgggtgaaaaatggtatcttcctttattttgcacttccctgattaccactgaa
gtttagcaacttttttttttttttttgagacagagtctcgctctgtcacccaggctggagggca
atggt

>chr18_7522400_7522600
accagcagttactagtgtttaatagctgggcatggttaggggaaaaagagaaatagcgccctacc
caaaccactgtcattccagtgtaatttttgtaagttaagaaccactaggaaaaaagaaactttaa
agtacaatatggcaaaatttgccaggtgtggtggctcactcctgtaatccccacactttgggagg
ctgag

>chr18_27254602_27254802
aataagtctcacgagctctgacggtattataagggggagtttccctgcccaatctcattttcttt
ttgcctgctgccatctgtgtaagatgtgatttgctcctccttgccttctgccatgatcatgaggc
tttcccagctacgtggaactgtaagtccaattaaatctcttttgtaaattgcccagtttcaggta
tgtgt

>chr18_29839402_29839602
atgaatcactgatgtgtgataaaaacatgattatatgaaatttttttttttttttgagacagcgt
ctcactctatcacccaggctagagtgcggtggtgcaatctaggttcactgcagccttgatctctt
gggctcaagtgatcctcccgcctcagcctcctgagtagctgcaactacaaggcacatcaccacac
ccagc

>chr18_37906402_37906602
tacaaaaaaccttccttatcaatttttccacttcttttaccttcttttctcattctatgacaaaa
tccattctcttgtattctaagttttcagtctttctcttgatctgagatttgcatctcataaaatc
agaatggatagacagaaaatttaattaagttgtaatacatagagaaagggctaaaaaaaataaaa
taaga

>chr18_44114402_44114602
cacatcttctaactttggaaaggagataaggcactgaatgttagctgaaagatccaggaaagtat
ggccttggcaacatcctgcttccccagggcatccaccctgagtccctttgcatttcctcctgata
cgtctccactatccagccaggccagctcccattgtccttgacacacacgccatgcttatgcctca
tgtcc

>chr18_45237402_45237602
aaagtgattggttggtttgcccccctcagcccgcctcccctcaaagagagaagaagatcatctttg
ctccagcctgatcctttaatgtgcctaaaagctgttgcttctgtctaaagcagaccccaggccct
gagcagcgaataggtggagaggtcttaatggggattagtgtctgcccaagcaatgccagaacggg
gtcct

>chr18_49466802_49467002
gctctctctggatgctggaggaacttctgtgtgtgataccattctatgtgaactttgaagttagg
gaggataagatgaagaaaaggaagactggaagtcctagattattttttctgggcccggcctatat
tgctttttataccatcaacatatgacattgtcaaccaaaagaaattatgtcaagtttaagaagcc
tctga

>chr18_49545602_49545802
atgctagtggaaacccacgtactgagaaggcttcctggtttgcaaaagaaatcctcagcttcaag
gcatttctccaatttatacacagtacttctacaacttcagttgtgagggttcactctcagacttg
cctcacaacatcaaatttctttaccaagtgaacccactcataagatatatttgttacactctcaa

tctat

>chr18_60335820_60336020
agttaagggagattatacatctgtacctgacacattgtaatagttcaataaatgtggctcttctt
tttactctattgagaaaattgaggccattgtcaaaaatcgtttccttcaatttctcacccttcc
tactacacagtgttctaaaatctgtcttctccctttttggtttaaggtcaacttttctacctatg
ttctg

>chr18_61300220_61300420
atgataaattttattatcagaataaaggacaatgatatgattatctcaagagatgcagaaaaagc
gtttgaataaattcaacctgatttcatgataaaatttctcaataaattaggtacaggactgtatt
ttaacataataaaagccatctatgaccaacccacagctaaaatcatatgcaattatgaaaattta
aaact

>chr18_68280020_68280220
ctgtctctactaaaaatccaaaaattagctgggcgtggtggcacacgcctgtattcccagctgct
caggaggctgaggcaggagaattgcttgaaccaaggaggcagaggttgcagtgagccgagatcgc
gccactgcgctcctgcctggctacagagcaagactccgtctcaaattctgtgggattatatcccc
tcaaa

>chr18_73757612_73757812
ttgcatttgacttgtaattcatttctaaacttttgaagagcctggaatcagcaggagattggaca
ccagaagtaataagcaaattcatctttaagaaaagtttcttgaagcaggaaaaacattttcaaa
ctctcttccccttagagtcaaaaagcttctggttgctacttgctctgtgcctgcaagggctgctg
gtgac

>chr22_43847056_43847256
ccaccacacccagttaatttttgtatttttaatagagactgggtttcacgatgttggccaggatg
gtctcaatctcttgacctcgtgatccacccgcctcggcctctcaaaatgctgcaattaccggtgt
gagccgctgcgcccagctggtggtagctttaattactactattaatatcatccaaagaggacttc
cgctt

>chr20_7064200_7064400
ttaacatgtggatggatctttacacaaaatcaaatcatgatggaaatgtgctgcatgtttattac
tatctcaactccctcattattaaggcagtataaaaatgactcttatttcacaagtgtctatagaa
agcttgtggctaaaatgtaagtagactgaaaatgggaatatatcttaaagacaattcagagtcag
ttgaa

>chr21_18541129_18541329
ttgaacaatgagaacacatggacacaggaaggggaaaatcacactctggggactgtttggggtgg
ggggaggctggagggatggcattgggagatatacctaatgctagatgatgagttagtgggtgcag
cgcaccagcaaggcacatgtatacatatgtaactaacctgtacattgtgcacatgtaccctaaaa
cttaa

>chr21_24308329_24308529
aggggtaaaaaaactttcctagtgattgtttgtctgggacagatttaatttcttcttagcttatg
gggctctagtaagtgttttcaataatataaggagcataaatgaaagagcacatgcctaggtttga
atcctagctctaacacttatgactctggtaagtcatgtaatcacttgattcttttaaaatacatt
atctg

>chr7_13592075_13592275
ttggatgttgctctatttgttacaggactattaaaatgtatttattttatcttgaatgacaagta
taattacaaaattaaaatggagtctttttttaataataggaatttttgactatgatattatagttg
gaaaattaaacttcactcagtatcagagataattttgtaaagcatttagtagagtgcctgacagt
taata

>chr7_14829475_14829675
ttaagaaatggccaaaatgttttccagagtgctgaaaaaccattttgaattcccaccagcagagg
atgactgatacagtgtctccacaccctttccagtagttgatgtcgttaccaattttgtttagct
attctaatagctgaataatgatatctctttttggttttgattgcatttccctaatgggtaatgat
gttta

>chr7_35403475_35403675

aatggaaggccctccttaacaccaaaggaacagaggagctgagtggcatcagagggtgcacatct
gatgtgagcctggaagtgaagggtagagcctactcacaagagcatttaggaaagttaaaattaaa
agaaaaaaaaaaaagccatagagagaaacggtattattgtagagtccactatgactacccaatgag
gtaag

>chr7_48546054_48546254
atgactttggtctctcttttgcatttgccccaagagatggcttatagagttagcaggaaatgccc
acaggacttgggcatttctttgcaaagtgtgagaaaaacgactattaagagagtcagtacattga
ctgagcttaaaacagatgagtttgtcatgcaactgtacttctaagcagaaattcttctaagggaa
tgatt

>chr7_49107054_49107254
cataaataacccagcctcaggtatttctttacagtgatgccaaaacagactaagacatggttccg
tgtgtttgtgtactcctgtatcaatatcatactctgaaataagattttatggtaaattctgagat
actgtaaggcaagattccaaggtttttttctaaacacgggctattttattacttgttcttccata
tatat

>chr7_51482106_51482306
ccaggctggtcttgaacgcctgaccttatgatccacccgcctcggcctcccaaagtgctgggatt
acaggcatgagccaccgtgcccggctgagattgacttgtatgctcagaagtgcatttgcttttaa
aaacttgacattcagtatacttaaatatctaggttttttttgtactttttgtgtgtgtgtgagaca
gggcc

>chr7_56495706_56495906
ttgaacctgggaggtggaggttgcagtgagccgagatcaggctgctgcactcaggcagcctgggt
gactgagtgagactccgtctcaaaaaaaaaaaaaaaaaagatggcaccaacggatgtcagtcacac
aatatatatctgcagattactataatccaatacaaggcaatggggtctaatatctgttaacccag
ctgtc

>chr7_63935965_63936165
aaaggaactctgtgggtttgaaatttggaataatattttttccatgttgatgttacaatttgga
gaaatttttctcttattctatgtaaaattttttaggggggtgtaaagttcagtctacagtgttcatt
cttagtcacctaactgtagccaactcccttgtcattttctctggaaaaattttgggatcatggca
gcttt

>chr7_67873064_67873264
aattatatatacaattatatataattgcatatatataatatatattacatatattatgtaattta
tattatatatatatttatattatattattattacatatatataattatatatgtataacatata
tataatatgtaattatatatacaattatatataattatattatatattaatatatataatataatta
tatat

>chr7_71227464_71227664
tggacagcatcccacatggttggggaaaacaggcactcactatgctttcactttcccctgtggca
gaaactgcaggccaaggagttctcttggcactgagctgtgctgctttgggggtgtggtgacatga
gtaaagtgaaattgttcttcttacccttttcagtgcatctattttcagattttttgctccagtga
ggtgc

>chr7_88923864_88924064
cttgttactttttttttaaaaaaaatgcttctctatacctgctgggtctcctcaatgcttcccttc
tgttctgatcaattttctcctgttaattctttgataattattttcctgtctctgttatttccttt
tatcactcttaattcactgatattaaaattttagggccttagtttcatgtcttaattctttatac
tattt

>chr7_109307764_109307964
aggagatgctgcggtggaggaggtacaacacacctctccttactgcatgtgataagttccaagac
ccccactggatgcctgaaaccataattagtaaagaacccgattgtcataagttggaacacatttc
tgttcacatcctccacccagaaatttaaagaaggcttttccatcttaacttagcacttacttatc
acaca

>chr7_112938764_112938964
acacagctgacaaacttcccactctcatgggctttacagtctaatgaaaagacatagataataag
caagataaataagtaaaaatagtatggtagatgatcaaaagggctaaggagacaaggtacaaaaa
ggcaatggcaagtgttttaggggggttggaatttagaggagtcactaggaaaggacttgtcaatgg

gacaa

>chr7_114977564_114977764
agaattgtacatccccaaattgcaatagtgtcaaggttgaaaagtcctggtttagatagagacgt
gccaaataactcccagtttgggtgctgctttcctaggaacagaaggagctaataagttaataggt
aaggaaaggcccacatagagatttagttcctagctttaatggcacaagagctagctggcgagacc
cattt

>chr7_118365764_118365964
ccataatgaactttggacaagtcaagtgggattgatgaaaacttgcccagtttgtttccagagtt
cagaataggtcggtatagccaagtgaaatcaatggatggcacagttttctaatggtcttgaactg
tttttaggggcaatgatttttcacacagacactaaacaatttttcaggcaattaaaaatagcctc
cattt

>chr7_122441164_122441364
aagcacatttaaaactagaaatgctataagccaaagcaatactcaaaaatatgtacatataggga
gagatagagaccaaaacagaatgttcaactgtaagactaacataatccactaaccaaaatcagac
aaagacagcaccaaaagccaatgtacgttataagaaatgctgcaaaaatcctaaacctactaatg
tttat

>chr7_126914764_126914964
acctcaaaactacaggaagggtcatgttcaagcccagaatcacaaaaaacacaggcttcccttgg
tttgcatggggtcgcctctgtcatgttatgacaaaacataactcagtctgtttttatctatgata
atcctacttgagaggatgtgagaccagcactgaagaaagcacactggctttggtgaaaggcagac
ctggg

>chr7_146105467_146105667
acagaagggactgcctcagagacctctttaaattcttaaggcaaatttaaaatcttggcaatttc
ctcgaaggtgaacaactaatagtcctttgtagggacatggatgaagctggaaaccatcattctca
gcaaactgtcgccaaggacaaaaaaccaaacactgcatgttctcactcataggtggcaattgaac
aatga

>chr7_153380867_153381067
cttggtgtcaaattaaactttattcaaagggcaaaggaatgcagtattagccaaatgcaagaaag
tcaagtaatcctgtaaacaagcatggagttatagaacctagaagcagacattcctggttctggaa
aagatattcaaagtcttattcccagcccccacattttacagttacgtgtcttcaggattcacacc
cagga

>chr6_28026221_28026421
tgggtaataggatactaatgtcatattccatcttccaacttttaaaaagtttcctccttacaaat
aggaaaattgttttccttattagcactggatatgggaaagatgtagctgcaaggagtctttcatt
gattcccaagtgtatcctgctgatttaagtcaaaatgtttcctccctgctttcctacttggcatc
cagcc

>chr6_70238479_70238679
tcattttagtatcaccagactataattctgtattggaggtggaggaatcccctttattgcttttc
ctccaacatgagatgtgaggaaaaatttctaaggacaattacaatgaaatcttcataaaacatga
attctttttgccatgaggatttcccacacacatgcatgcataactgcccacaggatggatcactg
gctga

>chr6_75272880_75273080
aacaataaaataatgactacaacaattttgataggtaaacctatcagtatgttttacagcttttg
gttgatggggggagggaatctaaatgtagggtagaagctaaaatagaagactgacaacttctcta
agacaaattagagctataaaatttttatgaaagcaaagcataagggacctgctaagaatcatgtt
tatat

>chr6_85096081_85096281
aaaaaggaataaaataatggcatttacagcaacttgggtggaattggagactgttattctaagtg
aagtaactcaggaatggaaaaccaaacatcgtatgttcttactcttaattgggagctaagctatg
aggatgcaaaggcataaggataatacaatgaactttggggacttgggaagaaagaatgggaggtg
ggtta

>chr6_88577081_88577281

ttattcaccttgatcattatcctgagtgactttgtccttaaagtgactatctttttaacactccc
tgccctaccccaccaactcatttctctccccactcttctcttcccaggtaatagcactagagtcc
acccaaattagaaaccgcacagatatctctagttcctgtgtctctctttccactcagacagcctt
tcctg

>chr6_92937879_92938079
tctattttaaaccagtaccatgctgttttggtaactattgccttgtagtataacttgaagtcag
gtaatgtgatgcttccagatttgttctttttgcttagtactgctttggcaatgaaggctcttttt
tttggctccatatgaattttaggattttttttctagttctgtgaagaataatgatagtattttga
tggga

>chr6_94458479_94458679
aaagtgagatttattatgcaaatattaatagaataaaagccgattatggcatcagaaagaaaaaa
aagtggactatcatattttgataatagattggttttagctttttttctctgtcttaagtgtcttct
tatcttttgagaatctttgtctccagtttgtcagaagagactgtctgattgattcagtcgattaa
tggat

>chr6_98023479_98023679
atttatagtaatatactttgtgtggatgtaagatttcttttgaggcaatttcttgaactgtaact
attattgtacattatatctttttatcacttccaacattatttcaatattttattgctttacttca
acttgaaaatgtttcagtttcctgaatagtattctgctagcttcagaatgaaagcctgttatttt
ctgat

>chr6_100666879_100667079
ctgttagatgtctttcctggtttcaaaccataaaatcctactggatttcacgctgttgtatttta
aaaaatgttatgtcttaaagcttttctacaatttaaaaaaaagttgaatgttttcaacatttatt
ttatcaattaaatctgtgcctagactaaaataatgttatgttttaaggcttctctgcaattaaaa
aaaaa

>chr6_104456107_104456307
attattgtactgtatagaccttaaataaagttgaaacccattgagtataacattcgaagaaatgg
cttacaaaaactaaaaaccagaacaaatcttgaaacatagctgtcccagttggcaataagttaat
tgtgatttcagtggtatttgtttcccttaatatatttcaggggcaagtgtcaccaggagaggtat
tcagt

>chr6_109148907_109149107
aaagtattttcaaaatgatggtgggacaactggatatccacatttaagaaataaaccctgacctc
agataatacacaaaaattattttgagatgaattatagatataaacataaaggcaaaaactacgaa
gtatgtacaagaaaacacaggaaaatatctttacatctttggagttggcaaaaaattactcagac
aggat

>chr6_114050507_114050707
tctcactctccagttctaattaattggtaaagctctttcttcagattattccagattatacccat
ttctcactctgccactgccactttggcccacatctcccacacctccataaatataagtcagatca
cgtcactcttctgtatagaatcaaccaatgtttccattgcacttagactaaaagccgaagtgctt
acaat

>chr6_115195107_115195307
aaaactacctttttaaaatataaggttatcgttcaatttcttagaataaattatttcttctacat
cctaatatctttttttctagtaatttagtacaaatcttaacatcatttgccaagctagtgttagat
ctgagaaaatagggtcatagtttggacttcttttagaacatactattttgagaataaatccaaaa
gtgtc

>chr6_120026101_120026301
acttaactccaggtaagagataaatgacataaccaagaatgcatatggttcttgagaaactttta
tcattctcctttattcgcactttcttttttcagagaaaacaagatcgcagacttcaagttttttt
ctcatagctcctatggagcactctgcagtcagccattgtgtttatattaatgagaaggtagagga
agact

>chr6_120993101_120993301
ttaatgttgtttaaagcactgtaaatatgaggaagtaccagtgtactgggatctcaacagagaga
ttaaatttaaattatctagaagataaattgtgtgaaaatcctggttgagagaaggaatgattaac
ctttgtttagtgaggccaggcaggtggctatgtgacattcttccatcaaattagaacaagaaaat

ggtct

>chr6_121887701_121887901
taatgggatggctgggtcaaatggtatttctagttctagatccctgaggaatccccacactgact
tccacaatggttgaactagtttacagtcccaccaacagtgtaaaagtgttcctatttctccacat
cctctccagcacctgttgtttcctgactttttaatgatcaccattctaactggtgtgagatggta
tctca

>chr6_124011301_124011501
tggaactgaaaccagctataccatgtattaactctgtgaccttgggcgattttctcagtgactta
gtttctcatctctaaaatacgtaagattatttttgggttaaatttgttaacatatgaagaacaat
gccaggcactaagcaagtactcaaaaaataattgctattattatttctcttgtagatttcttacc
ctcaa

>chr6_141492307_141492507
ttattgccagtgaacatggctctcagtgggaaggggagttgaaaaggggacagctagggaaggta
atctttccctggagtccggccatccctggcaggactcctctcccaaacttcaccatcaagctgtc
cctctgaagtcaagctgcttctctcttacgtccaaccatagtctctgatgtcaggctgcttctcc
tctct

>chr6_154244507_154244707
caaatcagaagcgtttaggctctttttcatcaaatataaaaacccagcccagttcatggctcctt
tggcagcaaccctgagacgctttacagccctagaccctaaaaggtcaaaaggccgtcttattctc
aaaatacattttattacccaatctgctcccgacattaaataaaactccaaaaattggaatctggc
cctca

>chr6_165492210_165492410
tatttctaatataaaaatataaaagctaaactccttttgaggcttctcaagtcatttggaactgt
gagccaattaaatatctttcctttgtaatttatccagtctcggtcagctctttatagcagtatga
gaacagggtaataaagagaggacggaggtgagaataaatctttcttattaaattggaactgaagg
tatca

>chr5_4801200_4801400
attacactttctgctcaatctccttcacaggtgtctttatctctgcccaagtgtaaaattgagtt
gtgtctgaggctgtctattccacaggtttcctgggcaaagtccatccatggctttgacgaccatc
caaatgctgagcgccagacacatctctgcttcttgcctacagaacaaagacacctgtacgcctga
tatgg

>chr5_9579200_9579400
aatggaaatgcttggatttgaacatgggtaggtgagctttggagcactacaaatgctgtctatat
tttgaataaccatcagctgagctgcctaattcagccagtaagcatttcttaggtgacaggaggtt
atatatgaactggtacctttcttcctacaaaatttgatacatttagcaaatgtttagagaccagc
aaaat

>chr5_18201643_18201843
gtcttaagaattaccagcatacatttttgtttgcatttattaatcaagtaatttcatacgtatcc
ctgccaaatactataagttgtcaaaatttggcacaggggttacaaaattataaacccagcccaag
acagaatgatctttgcttgtgtaatctttaataacattgatattggtttaatgaaaatagctaca
ccctg

>chr5_25581443_25581643
agaatatgcaagacagggtaacttataaagaaaataagtgtatatggatcacaattatggaggtt
gggaagtccaagattgggcagcccatctgctgatgttctcactgtgtttaaactcatagtgaaag
cataaggaaggaaatgggcatctgcaaagaatccaagcaagagaaggagcaaccggcttgtgaac
tcact

>chr5_27448443_27448643
tatgtggactagagcattagggacccagccatactactgggtccagatggcatcacgatgctgta
gccctctgggtgattatggaggtatatcagtggggctccagggatgtggagatgcaggcactgtt
gggccccacggcaggatgtagtcttgtgagaactggactctcaaatggcagctgcttgggtcttg
ggggt

>chr5_33297243_33297443

aggaacccctcataggggcctgtgagtaccaccccaacaactagcatgaaaataaaggaaaatct
tgagttccctcaagggtaattccaggcacctagctatccttaagaagtaaataaacaacttgata
agtgggaaggtaatagtagcttaaaacaatagccaaggagttagaatcaccagaatgtttggttc
cctgt

>chr5_34372443_34372643
aaatagatataaattcaaaaaaatttggagtacttgaagtgtgataaaaaaaaaaaaacacagcc
aggtatagcataatcaaaccaatgaaaactaaaaaaaagaaaaatttgaagccagctagagaaaa
ttaagatatggtatagattgatggtaagaataattgctgaattctcacagaaacaatggaaacca
gaaga

>chr5_39748443_39748643
aggtgatttagaaagctaccatacatgcacagagaaaagcaaaggcccattaagagatctgagaa
gaggctaggtgcagtggctcatgcctataatcctcgcaccttggcaggccaaggtgggaagattg
cttaaggccaagaatgcaagaccagcttgggcagcagagtgagaaccagttactaaaaaaattaa
aagaa

>chr5_49565443_49565643
atctaaaagatttttgtttaaagtaaaaatagtaacaacacattgggtatttctggcatatggaa
aacataccagaatctctgggacacattcaaagcagtgtgtagaggaaaatttatagcactaaatg
cccataagagaaaggaggaaagatctaaaattgacactctaacatcacaataaaaagaactacag
gagca

>chr5_57844043_57844243
tattgctaaaagggctgtgtaagtcagataaatgccaaactcgtaattcacccagacacagcttt
tataccggctaggtcaatccaattatctcagagcagggacaatggggaaagtgcatcaggatgta
ttaggcagctttccactagcaaaatgaggttaaaaaatatggaaacttcccactaccacctatag
ggtga

>chr5_62867244_62867444
aaaaagcatttgctagactgtttttatttttaatctagagggaaaacgttagtattttatttgta
gtatttattttatgtataccttatataattattaatatagatgatttacacttattatcttacta
tttgattttatttggaccttgtgatttatgccacctttccctttctttttttgccttcattttaat
taatc

>chr5_65637244_65637444
ccaaccagcaaccttcacctagcaacctccatttaacccaaaacaaagggcctcaattccttata
cagcttgtattccaagggataggccaggggttcagatgtcctttatacataaggagtgagtcttt
acattggccactcctgaattccttagcttgagactctgaacacatattcttcttagaccatаggg
tcact

>chr5_81189044_81189244
gcagcccctggtctttcagtttaactatttcacacagattttccaagtgcctttattttctcaaa
gtctgttattcctcctctaaaattcagaaatgtgtaacttctatttatttttagccccttttcta
ctatatatgcatcattttgaatttttattccattcttaatactaggggttctcaagaattttcca
accca

>chr5_81346444_81346644
ataatagatgatataatcctttgaaggtggtataatcctctgtcacactgctgattgatagagac
tatttcaagtcaaaatcctacaaattcttggctgggtgtggtggcttacacctgtaatcccaaca
ctttgggaagttgaggcaggaggattgtttgaggccaggagtttgagaccagcctaggcaacata
gcaag

>chr5_84749644_84749844
tgtggaaattccagatgaacaactcaacactttaaaaaatgcatctaaaaatgagggctcccaga
tcaggcccatccagggatgcctattcatgtgcagaaacttctaaaaatttttcaaaaaactgtta
ttttctcttttaaaagactctttacaagaaacagatgaaaagcttaagcaactaattgataaga
aaaat

>chr5_103592501_103592701
cctctgactagataattttgaatgatctgtctttaaatttgctgattctttcttctgcttaatta
agagtttttagctcatttattgaattcttcagctccgaatttctatatggttctttttttataatt
tatatctctttgttgaatctctccttttgttcatatatcgttatctcgatttcttttggttgtcc

ttttg

>chr5_105873701_105873901
acactaggtcttatttcttctatataactctatagttgtttttaatacacctttttaaaactctt
ctattgagttaacagtgatttatactctacttttaaagtagtagaatattctgaatttgactaca
taattacctcgatcagtgagttttacacttccaaatatttttatattgttacttatccatttgtt
tcaac

>chr5_155714622_155714822
ttagttccgtttctttgtgtgtgtgtgtgtgtgtgtgtgtgtgtgtgtgtatgtgtgtatgggta
catgcacacttgcatgtatatatattcatcgatacacatactctttgctgacattgttttaaagc
ttagagataggctgggcatggtgtctctcacctgtaatcccagccctttgtgaggctaagctggg
aggat

>chr4_11658102_11658302
atgggatattggcattgagaggggtaagggtgattaggttttaatgagatggtaaggggtgcatg
atcggtcgctaaggagggagtagaggtgtcttaggtggggagatacaaggggaggatgtgaagga
ggctttgaactgggggaaaaggcagcaataaggtgtggctgtagcccaggaatagtcagggaagc
agatg

>chr4_12464102_12464302
caaactactccgagctaaaggaggaagttcaaaccaatggcaaagaagttaaaaactttgaaaaa
aaaattagacaaatagataactagaataaccaatgcagagaaggccttaaaggacgtgatggagc
tgaaaaccacggcacgagaactatgtgatgaatgcacaagcctcagcaaccgatgtgatcaactg
gaaga

>chr4_14644102_14644302
attcatgtggacggcaaacatatgaaaagatgttcaagttcacttataaacagggaacaacaagg
tattaccttatatgcccatctgctggcaagagttaaaaagggcaaaacactgattgctggcaatg
atgggggatgtggtcgcatatatcactggtagaaatgttgcctattataaccattttggaatcaa
aaagg

>chr4_20313102_20313302
agtagatggtaattctgattttttatttaatatagattgttatggaaatctttataaaattttgct
acaaaaatgtttacatatgtgaccttactggaagtgaatagtactactttggtttaaaagtttgt
tcttaagaataagaacattttagagttatagaaaatagtccagtcaatgataatagctgtgtgta
actat

>chr4_28327302_28327502
gtacccagcttatattatatgttaaacaattgtttgttgaattcaataaactgacatagattaca
ttggaaattgcagatttattgataccttaatgctatactaaatgtgactagtctttagtcttttc
tccttgggaaagagaaatctactacttccacctagtatgctacctttcctatgaaaaaggattag
cttat

>chr4_28417702_28417902
tgaacctctgacctcaaatgatcagcctgcctcggcctcccaatatgctgggattacatgggatt
acaagagtgagccaccgcgtccagcccatacctgagtttttgctaatgaggtgatggtgcaaaac
tttagaaggaagagggagttggccaacagaaaagatcaaactcatgaataaaatgggaattttca
cctcc

>chr4_31410302_31410502
atgtacattgtgtacacacgcacacacacacacacacagagacaccсctacacacaccatatcat
taaacttaagagttggtagttcattaactcaacaagcaactcaaggacatattggtcatccatct
ataccagaaatcctttcaagtcctagaaatagataggattaaattatattccctactctgcagtg
actgc

>chr4_32448102_32448302
tttagcaccccacattttgatgactctaatcatcctccatctttccagaactgtgaccatattaa
aatgttgagaccgtcggctttgtttgtgttggaaacttatggaaacaggaaaataaagtagatga
ctttgcagagtgttttgttatttttaaagatgcacgtgtcacaatgtattcatatttgaaagagt
aacag

>chr4_65697205_65697405

gaggaaattgaatctcagggaagtgaacttattttttttttaagattttatggtgtcagagcaa
agattagagtctagatttttctgagtatatcttgtgttacagtgaccctgttaacagagtgaatt
gacaacattatttccactggcagcagtgccctggagtagctgctatttctccatattatctgcag
gcagt

>chr4_68078805_68079005
tcacacaatctgacagtgacttttctgggttaagcttttgttatgtaaacccctgagacctagat
gctgtttgtttccacaatataacctagcctatcttgaccaatatactcctgagtaacaatatctg
ttattgagcactctcaagagcactgttttcatgtattaattcttattcacctcagaatctggatc
ccagt

>chr4_69475005_69475205
ttttcattggttctgtttatgttatggattatatttatttatttgcatatgttggaccagccttg
cctcccagctgacttaatcatggtggataagctttttgatgtgctgctggattcggtttgccagt
attttattgaggatgttcgcattgatgttcttcagggatattggtctaaaactctgtttttttgt
tgtga

>chr4_82300176_82300376
tatttcttgaaaaaaattcacatacaggtggacctgtgtagttcaaacctgtgtcgttcaagggt
cagctgtacatccaggtttgtgagttctgacagacagctcaccactgatgtgtgcaaaagccaaa
tttgccctgttgccatcatttccaggagctttgtttcatgcttcattccacaggctcaggggtct
tgggc

>chr4_95004177_95004377
ctctactgaaaatacaaaaattagacaggtatggtggcacatacttgtagtcccagttacttggg
aggctgaggcaaaagaatcgcctgaacccaggaggcggagtttacggtaagctgagaccatgcca
ctgcactccagcctgggtgacagagcaagactccatctcaaaaaaaaaaaaaaaaaaaatgagag
acaga

>chr4_113856151_113856351
agggaacaatgggggacaaagtcctcaggtgagaacgtattttggttgttccaggagcaccaaag
cattcactgtggctggagtaggtaagcaaggaggaagtgtaggaaccgaagtcagagaaagtgtg
tgtgtgtgtgtgtgtgtgtgtagggtgcagttgggtgctagaacacatagtgccttataagttct
tggct

>chr4_118824352_118824552
acatctttaaaactaccttcacagcaacatctaggctggtatttgacctaacaactgagcatcac
agcctagccaagttgacacacaaaattaactatcacagtgacatttcagaaaaacttggaaattt
gagaaagaataagccacgtgcctgttggagggaggggaggaaaagtttttaggcaggggacacag
cagtg

>chr4_125727550_125727750
tatgttattctcttggtatggagtactgctgactcaggaagactacctgagctttagtatctgga
gatttattgaggtttcattacataggcatcattgattaaatcattgtccaggtggttgagctcag
tctccagccttcctcccctccgcacagattgggctgacattggctggctcgaagcccaacctcta
attac

>chr4_126725750_126725950
cactttttgatggggttgtttgtttttctcttgtaaatttgtttgagttcattgtagattctgga
tattagccctttgtcagatgagtaggttgcgaaaattttctcccattttgtaggttgcctgttca
ctctgatggtagtttcttttgctgtgcagaagctctttagtttaataacatcccatttgtcaatt
ttggc

>chr4_131951750_131951950
attatgactaatttgaaagtaaaactataactcataccacacaaaatttaatataaacaaagctg
ctaaaactatattaatgttacaaaaggtagattttaaggcaataaatattattaaagacaaagaa
gtactcacactttaaaaaggggaaaatcaacaaatagtaaaactcattgtatagcttccatagat
ttaaa

>chr4_134352950_134353150
aagagtagctggcaattagtagcagctttaagaatttttgaattagtgattgtatctttgacacc
tgacttcataatgagctcatggtggccttccagggtaaatatgatagagtgctgaatttttaatt
atggaaataaaactacgttaactaaaacactttgacactatttcgtatttaaagcttcattttat

taccc

>chr4_139545950_139546150
cctaaggattgtggcctccaggcgcggtgtaagtgatattgtatgcctaatgcctgggatactcc
ctgggttactgtcgccttgaaagcggggccattgtcactctgtaagcctcgggaagtctgaatct
gggaattatttcatgaactagtatctttattatctctcgggccttttctttcctgcagtggaagg
cctct

>chr4_155293950_155294150
aaaatagagtatttcagaagtaatatcagtagagtagaagtgactcagttgaaaaaatattgaca
gtcttcacaaggataaatagagcagtagcaacgaacatcaatgtattttacaagaaaaattcttc
tactggcagataaaaatctcttagtaaccaccagcaaatgggttctcagcaattttcttctaaag
ggctt

>chr4_166689150_166689350
ttttgcctttaatcacttatattttttaatttgccttacattttaaaatttgatgtatatgtctc
tgcaaactgtcaactcctggaagacagagacagtgtcatgcttctttcagaacctataaataaca
aaaaacacaagagaattaaataaatatttgataaattacattcaacactggactgtaatcttcat
gaagg

>chr4_168339025_168339225
gctgttattgatgtggcaagtcagggcattctaagaaggaatgtccaggcagcaagacaaagcca
ccttggccctgtccagcctgtggcagaaaccactggagatggatctgcccctggaggcagagttc
actgggtgaagaatcagtctcacaaatggtccagcaggactgacaggtcctggggctcaaacccc
tggct

>chr4_168868825_168869025
agaattgagttcctgaagctgataacactgtaaacgtgtagagactatgactttgaaggagttca
tcaggtttacatttccagcaattgaaagtatatggattttacgttgatgatagaacaaggcttta
gaaatgtcactgtggtgtcttgtcaatactcaatacacagttgcctctacctccctttcccagtg
ccaca

>chr4_171432825_171433025
agatggaatctcgctctgtcacccaggctggagtgcagtgtcatgatcttggctccctgcaagct
tcacctcctgggttcacgccattctcatgcctcagcctcccgagtagctgggactacaggtgcct
gccaccatgcccagctacaaaagttctgggatgataggcgtgaaccaccatgcccagtcatgatt
tctat

>chr4_171476425_171476625
tctgtgtttagtgattttgaaaagaatactgagctaagagttatccaaaatgtctaagaacaact
gcccatgttttcatctagtgacagggaaagttctctacctaatgaacacatttgaagtaagcgct
gggcaaagaattaagctattttcttattacatctcataaaatacatgaggtttttttttctcttt
taact

>chr4_178734006_178734206
attttgcttagccttttcccgaaggattccccacgcagggttgttctgggttgggggtctgtctt
cctgcagctgaagtcagacctgaattgcagggcaatgtgtgtcattcttttcaaattcagaagat
gaaatcagtgagtctctgttcattctcaattttaagcaggacaaacaatgaaacaagaaggataa
cccaa

>chr4_178762806_178763006
ttttctccctgtcacaatgtgctttctggcttattttgtttggctttaagtgagacaaaaatatt
cttctacttataaataacatactggtggcaggtgattgaactccaacctagacatatattgctga
gagaaatgaacaaaagcacttgtggaaaggagaaaagcagaaattaagtttactattttatatgt
tgctt

>chr4_179428206_179428406
agaggtctgaagtcaaggtgttggcagagccctgcctcctttaaaggcatgagggaaggaccctt
ctttgcttctcttatcttctggtggtagttagctatcattggccttatttggcccatggcagcat
gactccagtctctgccactgtcttcaaataggccttttccttgtgtgtctatgtgtcttcacaca
atctt

>chr4_187888606_187888806

ggcaggcgacatgcgagtgctggagaggaaccctggacgccaccatttcacatggaagaggcagg
agacatacgagtgctgaggaggaaccctggacgccactatttcacatggaagaggcaggcgacat
gcgagtgctggagaggaaccccggacgccaccatttcacatgaagaggcaggcgacatgcgagtg
ctgag

>chr3_7632400_7632600
ccatgctgtgttcttctcaccactcgaagctcagcctacagacacatatcatggcttccttaact
ctcctctaccccagctaaagggagctccaccccagtcatgttctgtttgttcctattcctcagta
acttatgtcacttactggtttgttcacgtgtacctgtatctcctcattaaaatgaaagtacctta
aaggt

>chr3_20519596_20519796
tatatatttctatataaatatgatactttgagatggcagtttcttttatattttagttgaaagtc
gtattttgtttaatcactgtggtaaaaaaaattgtgtatctatttttttcatcaaattttagaaca
ctttttctttaaaaatataaatctgaattgatttcctttttttgttttcagagtatgtgagggc
tgctt

>chr3_22658796_22658996
tacaaggtgcaactaaaattaattttatacttaacatccgacagtcactttcctaattcccttac
ttaggaaagggcttttattttaactttcttattcccttttttgcagtttgggaaaatagaagccc
agagagaattaagttactcacccaaggttaccagctagacagtagtggctggcatttgaacctaa
atcag

>chr3_28148996_28149196
aactaactttactgagtttcaagctctgaaagctggcaaacattttcttctatatatccctgaga
ggcttcctgagtccattcctgattctgattaatccattggtcccgatattatttacttgcctatg
tatatctatgggtctgtctgggtgctccattcagtagcaagtttcttgaatcaggtgagccccag
gtgcc

>chr3_34862196_34862396
ttctacatggttatccacagtttgttgaacttaagcataaaaatgaacaatttcccctagatctt
cgaatcttcattctgaaggttgctgtgtgtacacattaaataaatttgtatgccttttctccaat
taatctgacttatgtgagttgatttttcagcagaccttcagaggatgaagggaaactttcccctt
ggcca

>chr3_35075796_35075996
agccattttgtgatgtaaggacccaggagacagatagaaagttgggaggctatttttagtcattt
cactattgattttacaaaatactgaaaactgaagcacaatcctttagcattccatagttaggaag
gagtgaagtcatcctaatacgtaaggaaagagaggaaaagagagggataagggatacatatctct
ctctc

>chr3_84734910_84735110
taattcatatcatctagaaatctattgtataaaataatacaaataacaacaatgatatactaaaa
aaatgttactttctcactatatactaagctacaagagtgaagatatacagatcagccacagtaag
catttattttaggaaaagggaagatgagacgcttctagtagtcaatgattcatagaaattctgaa
actta

>chr3_88850910_88851110
gcgtttgaagattcaccacactgttttccacaatggttgaactaatctacacatccctcaacagt
gtataagtgtcccttttttctccacaacctaaccagcatctgttatttttttgactttttcataata
gctattctgactggtctgtgatagtatctcattgtggttttgatttgcatttctcttaatgaatg
accag

>chr3_104850710_104850910
ttttatcattggctcattatggcacaatctacatgaaattcatcaatcaacaattatatcattag
atacactgaaactacatactatttttgagatcttttccttttattcaccaaatgcctactacaca
ttactaagtaatttatacactgtatctcataaaaccctcacaacactctgttaaaaagtatcatt
aaccc

>chr3_135218110_135218310
gacttttacagaagaatcataggtataatacaatataggtttccatttgatataaccaatcttcc
ctaaagaaattctgcactaaattaaaatagtgagcactataacataatataagcgcatagtctct
ggagcttagttactaaggttcaaacccagattccatcaccacttcctaaccgtaactttccttag

cctca

>chr3_137248910_137249110
ggaattagaagtgaggaattacagaacagtggatgaggttgggaaatcggactgctgtctttcag
tgccaattggctgaatgttctgacggtggcctcaatgattatgacaccagatctggctgataatc
ccagatgcacagcctagcccagaaaggtagtgaaagctctttctcaaggtgtgggagtggagaga
atctg

>chr3_146292510_146292710
caattgtcttagttttcatttgccttagttgtttttaatttattgcataagaagaaaatgttga
attttatttctctatttttatatctagatttatatttgtgtgtggcctaaaatcatattttaatt
tttcttaatggtcatattagataacaatattttcagcttcatgttacagtgtacctgaacacttg
tgact

>chr3_147717110_147717310
tcttatttatcttcttaattaagtttagttgatttccattttgtttttccctgtattccattat
cttgacatcctgtaaagctgtttctattgactctttttctcttggctttcattcatttgtttta
tctcagagtatataatttagttttcattgagagtcagtcatttatatgaaacaccgtagtgctaa
ttgaa

>chr3_154537306_154537506
atttcacaacttatgagtcaatgtttgtgcagagttctgagttcatagcgttaaagtatatagaa
ataattaccattaattataaagctttgcagtcctcactgtcaaggctatatttacagaagcagtc
ttaacatctctctgaagagctattagccaaactgactgaagaggagcatcctgacagcactataa
ttcat

>chr3_155376306_155376506
gaaagatgtggacttgcacattttactaaaataatcaaagctgatccatgtttgccctgacgaat
agagtttatccccacaaattggtgctctaaatttcttaccaaaaacctaattaaataactgattt
taaaaaaaaagaatattccttaaaaaataaaatttacttttaaaaaagtttatttctctagtcaa
tctct

>chr3_161616906_161617106
tttttgtctctcacctttctctcttggacactccaattacatgtttagtagactgcctgaaatt
gcctcacagctcactgttgttcttttcgttttactttctgtatttctttttttctatctatgttt
tattttgaatactttctattgctttgtttttaggttaactaatcttcttatctgtagtgtctaat
ctgct

>chr3_164110106_164110306
agctagatgttggactgcacaaaaggacttcaaagaacctataataaatatgttgcaaatactaa
aggaagctatatttaaataattaatggtataatactaaacagttattaaatagagaatatcatta
aagagttaggaactattttaaaatggaaattctaaagaagtaaaataaccaaagtaaaaaaaaat
tacta

>chr3_173236506_173236706
aaactggaacttatatttaaagaggaagcagagtgtaaaactttgaaaaatgtgcagcccagcta
cgtggtagaaaagaaaaatccattttcaggggagaaattcaagctggctgcagaaattcgcataa
ctaaaaggaaggcaaatgcttacacaatgggggaaaggcctcgatggcatttcagtgaccttcac
agcag

>chr3_180763306_180763506
ttccacttgatcgaatcggctactgaagcttgttcatgcatcatgtagttcttgtgccatggttt
tcagctccatcaggccatttaaggtcttctctatgctgtttattttagttagcccttcatctaat
ctttttcaaggtttttagcttcttcgagatgggtttgaacattctccttcagctctgagaagtt
tgtta

>chr2_31696496_31696696
ggtcgtggtggctggaagcgggtgatagctgaggaggtggttagcagagggatttgttgatgaat
aagatacagggtacaggaggagcaattcttaacacaaaagctagagtggcctgggtccatgaacc
tcttctcttctctctctacactcatacccatagagatatcacccaatttcatggatttcaatgcc
ataca

>chr2_41857696_41857896

tctgcaagcaggcaaattccaaagaagatccagaaggaccatctaagcatacttagatcttcttc
taaaatttaacaatcagataggtcattcctcctctcctgaaaggggcaagtgcaggaacaggagc
cagggattttcttctactggaggtctggccatagtggggaggatttgggagagtgtctctgctgc
cttaa

>chr2_59618096_59618296
aaaagagcctctgtgaagaatttccatggttctggtttgcatgagcactgatgaaaagaagtgga
ctttgtggataactctctgggcctaatagaagaacttattcggggtaaaaatataaaacaagtaa
agttagttgcccaagccctccattatccctttctgattcatttccctgacagctgtatgtctttc
tctga

>chr2_60068296_60068496
tggaactccagtcatcattcaattctataggaagagtggtccctagagttgtacaatgcaagtgt
tggtcaggaatcctttggagagtctaaagaaagtcaataaatcccagtggaatttacacaaaaaa
acctttgcatttaatgtcgagttttcagagatacaataaaacccacccactaaccccttaaaggc
tcagt

>chr2_68195696_68195896
atggaacatgcctttcaacagcatgttatatcctcctttgaccatataatggtttttatgttgga
atcaattttcttagaaattaagattggcccatctacctttcatggttatacattgcctggaataa
ttttatccatccttttattttcaatctttctatgttgtttcattttgtgtgttccttgaaaacat
caagt

>chr2_83970489_83970689
gttttctatctttctgttcttccaggtcattttaccccaattcctattattttaatgcctttgt
cccttcatgcactccattgtagcttacttcctactattctccaactccatttattttatcttacc
ttttccccttcctccttctaaaagggcctcctaaaaagttaggttgattaaataaaatcattaga
gttac

>chr2_103893768_103893968
tagaaggccatgtgcatgcacattgccattcatgcgcataggaaagacctgagaaggccctaatc
tctcacttctagctaacttcgattctctgtacaagcaagatgtaaagactaacacagagttttaa
accacccgcagaagcactaaaggcatgccccagcacacagacccacctcagcaaagattggggaa
tactg

>chr2_104757768_104757968
ccaataattaaagagtttggatgttatttccatccttacagtatgacaaaaaggctaacaaaata
aaaagcgactccccttagatccactggcgaactgaagtcataggacaaattgccaccttgaatgt
tgaagagacaggtggatcatagcttactggcagagaaatcaatgccagggccagaaactggtagg
aacac

>chr2_118506730_118506930
tctttgcctcctgtgtgtgtgtgtgatgggaatattttctttgttatgtccccagatactctgtg
agatgtttcggttctcaaggacacctgttcctcggctgggcaccatggccttggctcaccacctg
gccttcagggttcaggcagtggacatacactcctaagtaacctggtgggagacctgtcacactac
actgt

>chr2_123730130_123730330
tgccttaaaacagtatgtgtatattatttagaaatttaaaaatattttaaaggtcaaaatggaga
gtgatatagaacatagaaagaaaataaatccggaaccttcagaagtctataatctatatcctcag
tgaaaaatcagattagaagaaaaaaaagaattgctaactctgctctttgtgggacaatgtgccct
ctaaa

>chr2_125106530_125106730
aagaattcttgttaaattgatgcccttcccagaggtgtgtgttcttgccaatttttggcatcttt
aaaaaatgtttgtgaacttaactagtagagattgtgtagaagtgttgctttgattggacatgtct
ggcttaaaatatagtcatgtcactcacaacggaaatacattctgagaaatgtgtcattaggcaat
ttcat

>chr2_125285330_125285530
taaagcaaaaaggaggaaggaattacttctctcctaaaccatttcatttttcactgctgtgggtg
tcctttctaacacaggcagatgagcattggaccatctgggaagtctgcagcagaggttcccgaaa
tgcccaataacacacaactactcctgggtttggctggtttttgctcttactgcccctaccactgc

ttctc

>chr2_125541130_125541330
tattattcacaagtctttcacaacatttatttctaccaaacaaataaggaaattattttaatctg
ttaaccaaattcaagttatcactaaactcttttccagtttcctgagggggtttggcatttggttt
gaggtgtcaaaatttgtagtgaggattaaatcaatgaagcatgtagaaggtttgatattaaataa
attct

>chr2_142190530_142190730
tgtctttaggtagaagtgtaagttatctatgtgagacttctcttctttcttaatattgtataggt
atatacaactattctctctgagcactgcattagctgcataaattttggtgtttatgttttcattt
tcattcatctcaaagtattttctaactttacttgtgatatcttcctgatgcattggttattcagg
agtgt

>chr2_153841154_153841354
taacagataataaaatcatgaggtgagaaactgttgggaaactttataatggatggattaagctt
agcaatacatgaattcagtcaagaatctaaatatcacaaagagaaacaatcagccatttttttgtt
tcctgatacgatgctagggcaatacctaagataccctataaaaaactctatttctgaaaaaattc
aatct

>chr2_155575754_155575954
gcaactaggaaagccactagtatttattgaattaactactcaactgcaatagcttgacttaacct
aagccattggcatgtggtaaaactggaagagaacatagccctttaaatttctgaagtcttcggag
gtgaatctttgttaattcagactgtgccgatcaatgtagccaaatgaaaattaatgtgggtagaa
aacag

>chr2_184371155_184371355
aatataaataacaatacatgtaaaaatatctaatatattacctggatatgtttttactcttgatg
ttacttcagtttctctgtgatgcaacaggatgtgaattcatttattgtggatggtaccaggagta
cttttttttttttaatctgaggatgcatgtacttctttgcatttgaaaatttattacttattgca
tcttc

>chr2_189016155_189016355
cctatcagcctgcttcttgtctttttttcttgtaaagtgacttgactattctgagtatcatgtgtc
catcctccctttacattctacagaaggtataaatgttctttaaaagtaaacattcaggaaaagtc
ctggaggaactgattgctggatcatttcacttacattgaaaaagttttactacctctaacaaaat
agcca

>chr2_195757155_195757355
ctgttgatggacacagctgctttctaaacttatctgttgtgaacagtgctgcaccaaacctggga
gtgcagatacctatttgatatactgatttcctttctttggggtatatacccagcagtgggattgc
tggatcatatggcagctcaattttttagttttatgaggaacctcccaactgttttttgtagtggct
gtact

>chr2_196087155_196087355
ttgacatacagaaaaccgtacatatttaatgtatacaacttgacgagtttggaaataagaatata
tccatgaaaccatcgctatagtctatgccataaacctatgtatcaactccaaaagtttcttcctg
ccctctttgtgttgtgtatgtgtgtgataagaacacttgacgtaaaatctacactcttagaaaat
cttaa

>chr2_217811755_217811955
gcagatgaacttaaattttaatgatatgttttatttaacttactataacaagaatattatgctat
ttatatagaattattaatgagagatttacattattttaggacaaactttttgaaatctagtata
tattttacactgatagcacatttcattccagcccctctgtcatgctggtcacatttcaagggctc
gatga

>chr2_221890756_221890956
ttttttttttgagacggagtctcgctctgtcgcccaggctggagtgcagtggcgggatctcggct
cactgcaagctccgcctcccgggttcacgccattctcctgcctcagcctcccaagtagctgggac
tacaggcgcccgccactacgcccagctaatttttttgtatttttagtagagacggggtttcaccgt
tttag

>chr2_225287956_225288156

ttcccatcaaaattccaatgacattcttcacagaaatagaaaaaacaaccctaaaatttctatga
aactagaaaagaccatgaagattcaaaacaatactgagcaaaaaaacaaagctgaagacatcaca
ctatctgacttcaaaatatattaataatacaaagctatagtaagcaatgcagcatggtatcggta
taaaa

>chr2_225495356_225495556
tcgcaaggatcaatcaatccttcaacataaaattactgagcaattattttaaaggtggagccaat
aacatttcctgactgattggatgtggtgtaacagagaaaagggccaagaatgcctccaatgtttt
tgacctgggccactagatgtatggagttgccatccagtgagacaggggaatctgtggtaaggcag
gtttg

>chr2_228809356_228809556
ccatgttggccaggctgatcttgaactcctgacctcaagtgatttgcccaccttggcctcccaaa
gtgctgggattacaggcgtgagccactgcgcccagccagaattgatttgaacttaaagcaaaagt
atctgtttcaaaactacatttattcgagttaaataaattcagtaattcttatgtcaactcaatat
gatta

>chr1_48071813_48072013
gtcatattaataaatgtgaacaggctaaaatcacctattaaaagaaaaagactgtcaggaagaaa
gaaattctaagcagtataaaagagacatattatacctaaagcaaagccattcatatagatttaaa
ataaatattttggcaaaggtctatccatcaaatgcagacacgaatagagagatcataattagaca
aatag

>chr1_72008412_72008612
tttagcaatatgccagggcaggtggtatcataatgtcactcctatgataacctttgctcctcata
actccatgatgcaatgagagcaagtgactaaagattaagagctccacaagcaatttttcctgatc
caaccaattctggagcacatttaactttggaaaaaattgccactctcagactgacctgaacaagt
acaac

>chr1_76472212_76472412
tgtccaaaaagcaattctctaaaatctgtattgtgaagacctaacagaacaaaatatctataaag
tggaactttttttcatgatgaagccatgagtaggatataagtaaacatctcccttttttcttggta
aaagaggatgctggtaatgtgcaagctaagagattcattgagttttaaactaactgtccctagca
ttaga

>chr1_84860412_84860612
taatacttcaaaaagctattggaaataaataaataaagccagcatcctgactcccaggagtcctc
aaaggacaactgaactttgatgaagccagtagagaccaggttcttgagaaacccaactttatctc
aaccctcaaaaatcctcacagaatgtgaactcatagccaaaaatcaaaagtacatacaatagcaa
ggtat

>chr1_95806412_95806612
ctttgtcaggtgttaatagatattggataagaggggtcctacgggcaatggattcggagaatgtg
atataaacaaatttaagcatgtatctttacaaaggacttcttaaagactttaataagctcatctg
ctttgtacatattcagaaaaggatagatatgcaatatttctcacagttaattgacctcaatgagt
gtctc

>chr1_96612612_96612812
actgaggagtttcttgctcacaaaacaatatgttactgcaataagaatcaatttgtcttttctcc
ccactgttctctttgtctgtgtgtgtgtgtgtgtgcacgtgtgtgtgtgtttcagacagagtctt
gctgtgtctcccgggctgaaatgtagtggcacgaacttggctcattgcaaactccaccacttgga
ctcaa

>chr1_96843812_96844012
caacaaatagcatgatgaatggaatagtacctcacatctcaatactaacactgaatgtaaatagc
ctaaatgttccatttaaaacatatggaatggcaaaatgtataagaattcaccaaccaaactatct
gctgcattcaagagactcatctaacacataaggacgcaaataaacttaaggtaaaagagtggaaa
aagac

>chr1_106208477_106208677
gtaagtaaaactacaattttgaggctactgtatctgatatttacaccgaaaaataataatgaact
ctcatttgctatagccagaattatcaacacacctatcgaagactactcagacttccgtcattgcc
taaataaaatactggttattttagctatctcttttaatattggaacgagaacaatctttattctt

gctta

>chr1_108279477_108279677
ttaaagttcatatggaaacaaaaaagagcccgcattgccaagacaatcctaaacaaaaagaacaa
agctggaggcatcacgctagctgacttcaagctatcctacaaggctacagtaaccaaaacagcat
ggtactggtaccaaaacagagatatagaccaatggaacagaatagaggcctcagaaataacacca
cacat

>chr1_190751577_190751777
ttaggctgcccctcctccctatgcagagaacttgaggctgaggaggattctcagctccacactta
gtcacacctctgggtgcttggtggccactcgctggattctccctcagtactggtgtttgtgcctg
ccattgtaggacctgtaggtgggcttgcatggtccttcctcatccatcttctccccactcagtgt
ctgag

>chr1_195669177_195669377
gggggtagagagttcatcttcttttttcatatatgtgtttcatctaagtgtatagtaagtctta
aataaagggttcctcaataaagggtaaatgtataaaatatttatcaatgtataagtggtactatt
taaagtaagcctaattcctttgggctatcaaaatttataaaatatttatatttctttatacttca
gtggc

>chr1_238781977_238782177
tatactcaaagatccactgttattggatacagtctatgaactcactcactacaaagggtgacaat
gttcagcaggaagagtctgggtgacaggagtatgaattcatctgtgtgttatcacattgagtgct
tatgcagattatccacttttatctcagtttatgctggatttctgaacctggcatatacttcggga
ctgaa

>chr9_4331600_4331800
ctgtacttgagcgacagcggtcagtggtaaattcacccaattacacaatgatgatagagtatgat
gggttatgacgaagaaatatgatgaaggcaggcactggtcacagacttgagtgtgagccaaagtt
tcccaaagttcagaatgtctaaggtgagacctgaaggatgagcaggaaataaccagatgaacatc
tgggt

>chr9_9815400_9815600
aagctataaaactaaaaggaaacgtagagaaaagtctccttgaaattgatctgggaaatgatttt
tttagatgacaccgaaagcaaagtcaacaaaagcaaaaataaaccagtggtctatatcaaagtaa
aaatcttctacacagcaaaggaaacaataaacaaaatgtaaagaaatcctacagactgggaaaat
gcaat

>chr9_24437200_24437400
tgtgatcacctcccaaataaacaactcaagggtaccttgggggaatctaaagtaaaacatagaaa
atagaatatagaaatcaagaaagcattttaaaaaggaaaataagataaaattaaatttattttga
caataatttaaattctctcactggaaagatgttcagattggatcaaatttcacactgactggaag
agaca

>chr9_29623600_29623800
tttcattattctaatattggttaagcccctacctactgctcattagctgtcttcctcaagtctaa
gctccaaccatatcgaaacatttatagttccactggccagactgttttaaactaacaagcccatg
tagatgctggtgtcagccctaacagacctacagtttgacaaattggcaaacattgcttatcctcc
aggtt

>chr9_41562600_41562800
accatgccgggctgaaaattccattaaattttaactgtagagcttcttgctcaccccctaggagtt
agaaacttgagaaggcaagctcatccagagttggacaagtcccagctttaaatagagtctcaggg
aatgaggtcaagtctttgctgggggtcttgtagatgatacacatgcaggtcactattctgagtgg
agagt

>chr9_87963980_87964180
ggggggcgcataagaaatacgatgtgctgtatttttctttgcatgagcttagtttccattttgag
tccccattgtggtggctattggaaaggtaatctgattcaattcagggccagctttctccctaccc
cggtaggagcaggtacagtccataaggatgcgagggtgccagggaacggtgtgtggctgctgcct
gatgc

>chr9_108706579_108706779

gttaaaaccatagatacatttggaaagagacatcttaattatattgtcttctaatccatgaacat
ggtatacctctccatttatttagatctttgctaatttatctcagcaaaattttgttctttttgtt
ataggagtcttgtctcacttgttaaatttattcctaaagatattatatttatttcgtgctattat
aaatg

>chr9_109549379_109549579
gtagccataatgtatctactcctaaactaaatcggggtgttaagtcttcttagagctatttacta
attcatctatttcagaaacaaacaccataggcttattctatatcaaacatcaggttggatactgc
agcagcgacatagagcaatgaaacatatttttttatcctccaggagctcattgttgggtgagggaa
agaca

>chr9_122311979_122312179
ctttccctctgtcccgacattcatggagttccaagcaccaaattagggacatcttccaaatacat
tcccaaatcagcttccctttaaagcaaagctacaaaaagacatggcactctaaatgccaccccctt
cccatgctggtaagcagcttatttagcccagcgtgtggccatcttatgaacccagagaagagagg
ctgct

>chr8_800200_800400
tcttaacagtgtgtcaaaacagtgttttgaataatagaatattttaattttcataatgtccagtt
tatcacatttttaaatgcattgtgcttttggagtttatctcaaagcaatttttttgaaccttccac
ctgttgctacaagttaaaagttacgctgcctcttgaaagatttctgcactttgtgcagaaatttt
atttc

>chr8_1352593_1352793
agagtcgtgtattgagtgcttactgagcgccacctcctcatcctccagagtcgtgtattgagtgc
ttactgagcgctccctcctcgtcctccagagtcgtgtattgagtgcttactgagcgctccctcct
cgtcctccagagtcgtgtattgagtgcttactgagcgctccctcctcgtcctccagagtcgtgta
ttgag

>chr8_15801229_15801429
ggtactatcctagtgatagtgatatagaagatagaaagaaattatttaggcagatagtgagggca
aaagagtccgcggcaaaacttcccttttaacaaaaagcagccctagaaatcatttttttgtttcta
acaaagagcagctggaaagatcaagctgcaaacgtagataaggaagctggaagcttgcacagggg
aatga

>chr8_16647229_16647429
ggtgctggaacaattagatacctacatgcagagtaatgagcttcaacccttacctcacgccatac
acaaagagtaacttgaactatattgtatacttacaagtaagaactaaactaaactataaaattct
tagaagaaaacataggagtagtgatcttggattatgtaataattttacaaaagtaaacacaggaa
ataaa

>chr8_26960083_26960283
tcccaacactttgaggggccgaagcaggaggatcccaacttgtgcccgggagtttgagaccagcc
tgggcaaaataatgagactctgtctctacaaaaaatgtttaaaaatagcgtgccatggtggcgaa
cacctgtagttccaagtacctgggaggctgaggtaggaggatcacctaaacctagaggtcaaagg
ctgca

>chr8_35892458_35892658
ctatagagctgtaaaataataggtttgtgttaagtcactaaatctacaataatttttttatagtgg
caataggaaatagaaatccactactatggtgagtatctcttaatgattggtctgtttctcaggtt
tgctgggatgaaagaaagctcaaaaatccatgattagaaaagctctgaggtccttttagctctga
gatca

>chr8_40427843_40428043
ctgtcaaaacccatcaaattgtataactgaaatctgtgcatagcactgtattcaactgcagctta
atcggtttatttaaaagtaacttgtggggccacgtgcagtggctcatgcctataattccagcact
gtgggaggtcgaggcaggaggattgcttgagacaaggagtttgagaccagcccgggccacacagc
aaaac

>chr8_49397247_49397447
gtgcactttctttcttaatatgcatttagattactgtctcatgttctttcattttaacctgaagg
actccttttagtgtttcttgcagggcaggcctgctagtgacaaattctctctgtttttgtttatc
taagaatgccttaatttctccttccttcataaaggatagttttgctggatatagaatgcttaatt

gacat

>chr8_50925647_50925847
ggacacatttaacacaggcccaggcactttattcaataaatatttgttaaatgaatcaataaata
acggatttgcaaatctttatactgtagtcttgctagtgtttaatcttcatagaaacagtactatt
ctttgagtgacagagtgataaaaagtttattaaatgtgtagaagactgcaaagattaagaaggat
atctt

>chr8_75586645_75586845
tttaaagagctagagcaccataattataatgcaaaaaaatagggagaggaattttacataaaatt
aaacataatattgaaagacgtacatttctactaacaagttggatcctaatcagatgacttcattt
ttcctaccaaaaaataaagaatcatgagagaatttggctaagtttgtctttactcttccctactt
tctgt

>chr8_84378845_84379045
tgggaactagaacaaaggtgagtcttgctatactttttacaaagagactagtggctttttgcccct
gacctagagctctgtggaacttttaacttgagagagatgatttggagtatctggcagaggaaatt
tctaagcagcaaagtgttcaacatgtgacctggctactcttaaaagtgttcagtcttataccttc
acaaa

>chr8_96600224_96600424
ctgctatgggccccaatttcttttttttttttttttaattattattatactttaagttctagggt
acacgtgcacaacatgcaagtttgttatataggtatacatgtgccatgttggcttgctacaccta
tcaacccatcatctacattaagtatttctcctaatgctatccctccccccagccccacaccccccc
aacag

>chr8_98340224_98340424
tctatttcattctaaataggctgtaattctccttgttatttctcctttggccttggaatgtttag
aattatgtcttcaggtgtctaaataaataaaatttttgtcatattttcttttttattttttagct
taattatactatgattagagaatatgacctatgtgattaaaatcttttaaatgtgttgagccttg
ccttg

>chr8_108220624_108220824
gacagtcttccagtagatacaaaactgaagctggtgatcagcagcttcctcaggagatctcagga
gctgggtgagtgagctcaagtatttgcagtaagagtcaaaatggaggagttcaaccaatatatga
ccttcctctgggaatgcttgactgataagggaaaaatgcctcaagtgagcatgcacacaactaca
gtaaa

>chr8_112881424_112881624
aagatttattctagctaagaaattgcatggttggagttttactcttccttaaaattataataatt
ttttttctaaattaaaataagaataggacaatattgaatgataccttaaaagttttacatttaga
aggcttttggaaaatgtgactaacatgaactgtaggtattatgagactcttcttgcactggaagc
ctaat

>chr8_113885024_113885224
gaaaataagacatattaagaggtgagatagaatgatataaattattatgcacaatttatatagaa
agcctagcattctcaaggtcatccaggagggaaggagtgtggaagagagatgagaagtaagaaca
gaaagaattaagaagatgatctgactaagaagatgatgtgtagtgaagaagccagacaaaaacct
cagac

>chr8_117376419_117376619
tttacataaatatgtgtgctaaaaaggatgaaagtgtatttgatggatcctcactccttgatgca
aaattaaaagtagaaatttgctgggcatggtggctcatgtctgcagtcccagaggtttagaaggc
tgaggtgagagaatcatttgaggccaggagtttgagaccagcttaggcaatatagccagacccca
tctct

>chr8_121163819_121164019
agtgagctgagatggtgccactgcactccagcctgcacaacagagcaagactccgtctcaaaaaa
aaaaaaaaaaaaaaagcaaagtcagtgtggtaggcagaataatgtacctgcaaagatattcacat
ataatcttcagaactagtgaatatgttatatggcaaggggggaattaagatgtcagattgaattgt
ttgtt

>chr8_122768419_122768619

cttcattctctaataacagaaatttagctgaactttttgttcttatgtttgtagttttaaaat
taactttttggttcatctgtagttctcatttcttcttcttatggaaattctttctttctttcttt
cttttatcaatacccccaataaaggaaccaaccatcctgaaacctcagtctatatatttcaatta
agtac

>chr8_131624618_131624818
tgtttctctggagaaccctattacaagctccttgcccaggttggagggaggattataggagactt
tctggaaggaggaaaaaccatataaacccaacgttaccgaagtgaaggaaggaggatttcaggga
caagggagcattttacagtactacaattacagactggacaaatgggacgagtaatgaaaagtgtc
acttg

>chr8_136379818_136380018
taattaccctagggcccggtaccagaccactagagagcccctacactccagagcctgctgaaatt
attcaaaccagccaatcttaaacctgtttaccttgcttcacttattccattccttctcacagaac
ctacaacaaaggcatttgtcccccaaccttcccccaccaccaacttcctgaccaaccctgctgct
tccct

>chr8_142967693_142967893
taaaagaaacacaatttgttccaagcaggataaatgtaaacaaactcatgacaggacgtagctga
atgaaactgcaggatgtcaaagagaaaaactggtatctatgagagggaagaggtacaggcatatt
tctaatgggtgacaaggtctgctgggagacaatacagcaataaattcaaattgatgagggaaaat
catgt

>chrx_1880400_1880600
gtgctggggttacaggtgtggggtactgcacccagccaatttctgtttttttgtttttttgtttttt
ttttgagatggagtcttgccctgtcgccaggctagagtgcagtggtgcgatctcggctcactgca
acccccgcctcctgggtgcaagcgattctcctgcctcagcctcctgagtagctgggactacaggc
ccctg

>chrx_6309800_6310000
gcctcaggagcagtgagggatttgcaagaagggaagctacatagagaaacaataataggatcctc
attgtcttctgttctcctccttccatactcctctgtaactgagttcaacaagaatccgcatatcc
tcagaagagtagccttcacatttgtgaagtggaactcaagtgcctggtgaagctaaaatccaaat
gaaaa

>chrx_14204479_14204679
gtagacagctcagtgaatttttacatgtatggatacccatgaacttacccctcagatcaagaata
tttccagcatcccagaagattccttcctgccccttcctagtactactcttcccctgccaagtaac
cattattttaacttctatcaccataaactagtttgcctgcgctagaacttgatgcaaatggaatc
ataca

>chrx_15312879_15313079
ttgctaggggtttctatatttttactaaaaaatatttggtaggactagtttgatttgtgttttac
taagtttcatcctcaccctcagtctaggaagttgtcttcttatgcgatttcctataaatttctct
tagttcacgtcaacaaacatttattgagcatctactaggtgctaatgttttggaaacaatgataa
gacaa

>chrx_22615479_22615679
aatagcaagaattgggcctcactatgcaaatcttgtagaaacatatacagtctcagagaaaattc
aatatcttttgccattaattgcttccagtgacttttgggaacattaaaaatgtggcccagcagga
atgagtatcctgacagaaagttcagttggctcaccatacaggcgttctctaattaacatgtttaa
ttaga

>chrx_29632279_29632479
ctcatctggagtaattacatccgctgacaaatgcatatatagtgccaatgtcatacttgccttgt
tttctcaaccttccaattcctaggcaaattgtcagattctcaaggtaatagctgtcccacatgag
actctggtatgtgcagatctttattgtgacagttccttttggggcttctgtgacccaatgaagtg
aaaat

>chrx_36596679_36596879
ggagacattagggagaatcaataggtgttacagggtgttccacaaagcacttggtgcttaattta
ctgcaaagtgaaatattgactgcagagccagaggaggatggcgatatgtggtttagattagattc
aaacacttttagccctcagaatactattaatcaggctaatattgagtaaatatttattaagcac

ctact

>chrx_44185056_44185256
ctagataagccaatataaatgtcatttctattgccacaatagctaacaattgtagcactgttcta
agcactttagaagcattaaactttttttttttttttgagacagggtcttgctctgtcacccaggct
ggagtgcagtggcgtgatcacagctcactgcggccttgacttcctgggctcaagctatcctccca
cctca

>chrx_50803860_50804060
gaagatggaggcacagtataccaaaacctctaggataaagcaaaaacagtgctgaacatctacat
taaaataataggaaggtcacaactcaacaatctgatgtcactcatccagaataatgctcatcaat
aaaaaagaaacaaaccaaacctaaagctagcagaagaaataaaaaagatcagagcagaattaatt
gaaaa

>chrx_51800860_51801060
aatcccacgtggtcatgggcctttcatgtgttgttgaactcggtgtgctagacttttgttgaata
tgtatgcacaaacattcttttgggaagtgtgtgtgtgtgtgtgtgtgtgtgtgcgcgcgcgtttc
tggtcctagttcttctctaactgtttagtagaattctccactgaagccatctagtcctgcggttt
tctct

>chrx_54690275_54690475
tttgtatctttttgatgaaatgtctattcaaattctttgcccattttaaattgggtgatatgt
tttcattgttaaggtatgtgctttatatattctgtgtacaaattccttattatatatataatttg
caaatattttctcccattctgtgtattatcttttcactttcttgatggtatcctttgaatcacaa
acatt

>chrx_63529475_63529675
cttaattctatggctaggacttccaatactagttaagattttgaagtcacaaattatgaattaaa
ttactgtcaaaccttggagataatgtaggttcagttccaggccactgcaataaagcaattgtcac
agtaaagtgagtatcacaataaagccagtcacacaatttttggcatgtaattatgtttacaata
tactg

>chrx_67848875_67849075
aatacagcagtcggaataacgtggaaggacttgacttgctgagccttccagccttcatctctctc
ccatgctggatgcttcctgccctcgaacattggactccaagttctttggcttttggactctcgta
cttacaccagtggtttgccaggggctcttggtccttcagccacagactgaaggctgcactgtcgg
cttcc

>chrx_74048475_74048675
cctatgatagcttctggttttagtggtcttcttgcctaccaggcaaccgtgaaacaacaaattaa
gttgaaatcactcagggcaacaagtcactgcttgaaagcagtgttactttgaggttttgtcatcc
tagccttaaaaaagcaaagaaaatttcattttcactacagcctctgaaatcttcccttatctaat
ctctt

>chrx_74748275_74748475
ttatacttatttttatacattgggtttgatttgctagtatttttttgagaatttttgcatctatg
ttcattagagatattgatctatagttttcttctcttgtagtgtctttgcctggtttttggtatta
gggtgatgctggcctgatagaatgtgttaggaagtatttcctctgcttctataatttgaaagaga
ttgta

>chrx_82793544_82793744
cagtttgaaacataacagttttctcaggtactttcacattccttcgaaccaacaaattttaaagc
cattgaatgtaacggcatactttgaataaaatgaaactctgagatgaaagcaagccaacatgcac
agccctcttctctgtttttcttccagagtttaaattcagctaatcaaagtttttcctcatattag
agggc

>chrx_83641744_83641944
ctgtgttaatctagataatggctttccaatcttatttgtctttgcaaagaaacagtcccttcatt
tcacaatcttttatatggatattttcagctcaattttatttaattcttttctaatttagttatt
tattttcttctgctagctttggggttgatttgtttctttttttttccctagttcctttagttgcaa
agtta

>chrx_84707544_84707744

actttcttggttatttcagtggcaatgcaggaagaggttcatcaggtggcctggggctatttttc
tgttacgtgcctttaaatctccagagtggggagggaggatccaagagggaagaaagtgaggctct
atagtcacagctgcctttaagtgctaaagattttaggaagggcagtagaaggaattttactgacc
agcac

>chrx_84999744_84999944
accacctcacaccagtcaaaatggctattattaaaaagtcaaaaaataacagatgctgatgaggt
tgcagagaaaaggaaatcttattcactgctggcagggatataaattagttcagccaaggtggaaa
gcagtttggcaattctcaaacaccttaaaacagaagtaccattaaactcagcaattccattgttg
ggtat

>chrx_85847544_85847744
ttaccaatttcacaaactaattttctgggattttgattgggattgtgttgaatgtatacatcaag
ttatgtagaactgatatcttaattatattaattgcttacatccatgaatgtagaatatgcttgta
tttatttaggtctacatggatttctgttattagagttttgtagtttttctcctatatttcttata
aatac

>chrx_92283344_92283544
tgtccctgccttaactgatgacactgtcttgtgaaattccttctcctggctcatcctggctcaaa
agctcccctactgagcaccttgtgacccccactctgcctgccagagaacaaccccccctttgactg
taattttcctttacctacccaaatcctataaaaccgccccactcccatctcccttcactgactct
tttcg

>chrx_99094544_99094744
tgttcacataaaaacaaaaaccccctagaactgccttacaaaaaaatgctaaagggagttatttga
attgaaatgaaaggacactaagtaaaaacataaaaacatgaaagcataagactcactgataaagg
taaatatataatcaaatttagtatatttcaatatcataatggtgatgcaaaatcactactacaaa
agcta

>chrx_101375344_101375544
gcaatgacattcctcacagaattagaacaatctattttaaatttatatgaaaccaaaaaaggcc
tgaatagccaaagcaatgctaagcaaaaaaacaaagctgcaggtgtcatgttacctgatttcaaa
ctatactacagggctacagtaaccaaaatagcatgatattggtacaaaaacagacacatagtcca
atgga

>chrx_101421744_101421944
gcacaaatgggtccagaacaagaggatccacttatcatatcaaatactgcaccatccagaaggag
ctgacctcacagaacacttctgaaggcaaagataaagcaccaataaaagtaaaccctgaaaaaat
tgggtgccgttctccaggatgcagtgtatacattaaatcacagacctatacgttaggctttagat
ctaat

>chrx_101557944_101558144
gtgctgtgccttttactggtgatctgttcatctgtatgtaatgatagaaacgggggccagggcag
ataccaggtcttgaagtaggcttccatcctgtgactttgtatagatacttcctaaatccaacttg
aaatttcataataggctctttcataaatatgtagccccattttatatcctctatttacatgtgtc
ccata

>chrx_103242744_103242944
ttgtagagaaattatggtttttttttccctctgatgtgcagagttctgttttggaaagtgtgcac
atgcccgcagagatcctttggtttccaatagtttaagacaaattcaaggcaccacccagagggat
tgacttgcagttgaattgtggtagtttctcctggtctgcttaggaaaaaaaaaagtttttccaag
tcgtt

>chrx_116311372_116311572
ccatgggcagcaacttgtcacagttgctgggacttggggatataaggatggaagaagaaagaggg
atctctcacttttcctcacataccccaagtattttctaggaagagaagggaacctgggactcctc
gctcccctctttctagatgagtaggcattcatcttcagtctgtacccctttcaaatgcatcctga
acccc

>chrx_116368172_116368372
tatatgttcattttattttatgtaaactgtatgtatacacaatacacacaaacatatatacacat
atataaataatgtcttaacttttgcctttaagaaactaaataaagtgcctattaatctaatatta
tgcaatgtgttgggttccaaatactgtaagtgtatttattagtactaattagtaagggtggctct

actgt

>chrx_143422514_143422714
aagaagaaggctctctgccagtagagagggggggcctgaatgggttgcccttcatgaggttgggtt
ctggggattttatggcttgggaagtggaagaaagacgcgtagtccatgggctgttttggagaaag
catgatttagcttggtccaggaccttggcccaggaccaattaggagatgaagtaatgattcatag
gggct

>chrx_151625944_151626144
gccaggatggtctcagtctcctgacttcgtgatccgcccgtctcggccttccaaagtgcagggat
tacaggcgtgagccaccgcacccggccaacctgtgacctttcataagaaaaaaagacatctttaa
agtgctgaatttttaaaaactctatcaatacagaattttacatttatcaaaaatatcccttatt
aataa

>chrx_155099606_155099806
tctctcgcccaggttgaggatgcatgcccgtgacacagcctcgggaagtcctaaggacatgtgcc
caaggatgcagcttggttttatacattttagggaggcatgaaacattaatcaagtacatttaaga
aatacattggtttggtccagaaacgcgggacaacacaaagaagcgggtgggggatccaggccaca
ggtaa

>chr13_19166600_19166800
tctaggttatataattttgaggtgtacagtccatttcacctaggctgtctaactttggggtgttc
atagtatttcttcacatttctttctgtcgttagagacaagtataattaatacgtataaaatatgg
tattctcctatttccacaatgatcaagaacaaattcttcaattttatgtttttattatgtcctct
ctccc

>chr13_23181800_23182000
aggaactttaaatgcacattactaagtgaaagaagccaatgtgaaaaggctacatattgtatgat
tctactatatgacattgtgggaaagaccaaattatggagacagtaaaaaaatcagtggtttccaa
gaatgaagggagagagagggataaaaggggaagcacagaggatatttagggcagtgacgctattc
tgcat

>chr13_36758400_36758600
aaagaaaaaaatcacaagggatatgagaaaatattttgaactgaatgatagtggaaatatatcaa
agttgatgagatgcagctgaagcaatgcttagagaaaaactttataaaggaaaagttttagctt
taagtgcttatattagaaaagaagaaaaggttaaaatcaatgatggaagtttctacctacaaaga
ccttt

>chr13_48005599_48005799
ttattcatcactgattggaatataaattaattcatccattgtggaaagcagaaaatcatcatgtt
aaaatacctaataagagaatgggtctaaaaaattatttgcagtaattattaaaatcataaagcct
acggttttcataatgaatggatcaggctagtatcacctgaaccacctgatatggtttggatattt
tgtgt

>chr13_53805799_53805999
gaagttccaaattgtaaagggatcagaattaataatgctctgtcacgggtaacccgaacacacag
gagtttaaggaattcacatcttggagaagaaacctggaatattgctgttgaaaagagaagtctgg
tcatcagttggattgatctgtcttatgaataacttcaaattgactacaaataaccacaaaaatag
atttc

>chr13_54914199_54914399
gggctttgcaaagaagtggccacaaatcttaagtgttggttcaaacactagcttttttttttgtc
tactgtaatagtaattacccatacttaagtctcacagaagattaaggtagaatcttccttaggat
acttctttatttttttcttagacacaagattttctatgacagagggaattgtcagtccattaata
tcttc

>chr13_63898999_63899199
atttattaacctaattcaaaatgggagtatatttggataagatgaaactttgtcacttcaaaatt
agaatccttgcattatgtaatgatgaatatagccagttacaaaatcataaacctaagtaaaatgg
cagataacattaataaagaaaatttataaaatttaaatgcaaagactacagaattacccctaat
ctgca

>chr13_67316599_67316799

gcaacaaaagctaaaattgacaaatgggatctaattaaactaaagagcttctgcacagcaaaaga
aactaccatcagagtgaacaggtaacctacaacatgggagaaaattttcgcaacctactcatctg
acaaagggctaatatccagaatctacaatgaactcaaacaaatttacaagaaaaaaacaaacaac
cccat

>chr13_69723399_69723599
tgacttcttgctttgcatatatatgacttttagttggagctggtatctaaaatatgaaccttatt
ttcaacctttaattcttaagaaatatttgaataatgacagaaagtgaggtaaatgtttacaaaat
aaaacctttttttctaggagaaaatagaaagcataaaaatgcagttaaaattctaaagatgaggc
caggc

>chr13_70773399_70773599
gccatatttctatcaaaattcagtagggctaagtgccttgtactcaaagcttattctgtgggccc
cttgaaaagtaaaatttacaatatggtctttcaaatttttccgttaatgaaaccgctggacaata
tctaggatatacgcaatacgtattaaatgcttgatatcataaattatattttgggaaatgtacaa
gtatg

>chr13_75485599_75485799
ctaacccatgaattccagctccaatacaagcctgagagtaaactagattggatccaagtttcctt
caaaagttgcaaacacacatagggagaatatgttaactcttaaagtagaagaagaatgtgtgttt
ttggggagctcagagaggtgtatgaacaaataggaatatttaagctaccatgaggacttctgctc
tggac

>chr13_80000999_80001199
gtatataagaaagctctttattttctacctcactaccctactgaatttttctattcttatcttgg
attttctatgtactaatagtaatactaatagtcatagttttaaattctttccaattttgtacctc
aaatttatttctcttgtattatttattttttcagtagctagagtacaataataattatagcgatg
agagt

>chr13_81955399_81955599
tcagataactatgtatgcaaatatttaatcttcagagaaaacttgatttttctagagtttacaa
caactagtaaagctggagatgtttctgccagttgggttttataattcttatttaattgaatggat
gtttaaaatatgttacctagtttaaaaaacgcttttgataataattcaaaacctaatacatttag
ttaaa

>chr13_82664599_82664799
caagatcacaaagcttttctcctaagtgtgttatgattgtgttttatatttacttctgtaacaca
ttttgaattagtgatttatatggtgtgaagagaaggttgactgctgcttctactacttcttctcc
ctttccctccaccttcccctccccaccctttctttgtctttacaaaaaatatggatacacccttg
tccct

>chr13_85944599_85944799
ttacataatatttttcaagtttattcatacttattatgaataatgctactgagagcattagtgta
cagttctcatgtgaatatgttgggtattcacatacccaaagaaaattaaattgatacaccatata
gtaaaactaggtttaactttctaggaaactatcaaactgttttctaacatagtcatttagtgtgt
gtaaa

>chr13_87588399_87588599
aaatactatctcaaatgtaataatattaccctatggaaatccttttttaactgcaaatattcagca
ttgagtcagaatttatcgaaaactgctatttcagttagcatcccatcataaaaccaatagtctgt
tccaagtaaagtaattagtagaaaaatttaaggggtcatttatgaaagagtggatgaggtataga
agaac

>chr13_89381599_89381799
agcacaagtgctgacatatcctaggcttggaacaatgaggtcctcactatcctccttctgccctt
tcttttcattcagcctgagcaagagagcttactgtccccttagcctaaatctaggcccatcatct
ctcttttcttggaacaaccgtcattgtaaattatttctcctatcctttgtgatataaatctttaa
aaagt

>chr13_94134999_94135199
tgttacaggaaattatagaccccaaattgagatgtatgaaacaaagagttgtgtagcaagtacgt
ttgtaataatcaaatagtatttattttcattttacgcaatttaccatatggaggttttctaaatc
tatttaagatgtctttgttacgtgcatttttgaaacgtgctttgtggtctgtaagtctgcttgca

tttta

>chr13_105722199_105722399
agagattattggcttcaggtaatgaaatttctgtggagaaattagtacaccacatatttttgaag
atattttactagctatcttttttttaaaaaagcagtaggcccaggaccaaataatgaatttcatc
tttttttaaaaaatagatcttttctatttaaatcaattcactggatcttttctatttaaatcaat
ttcta

>chr13_107632999_107633199
cactgtgggaggctgaggccggtagatcacctgaggtcaggagtttgagaccagctggaccaaca
tggtgaaaccccgtctctattaaaaatacaaaaaaaattagccaggcgtggtggcaggcacctgt
aatcccagctactcgggaggctgaggcaggagaatcgcttgaaactgggaagcagaggttgcaat
gagct

>chr12_16406733_16406933
aaattgtgggtaacccttgtgcactgttggtggaaatgtaaaatggtgcagccattgtggaaagc
agtaaaaagagttctcaaaaactttaaagtagaattactactatatgattcagcaatcccacttc
tgagtatatatccataagaattcaaagtgagattctgaggaaatatttacataccctcattcatc
tcagc

>chr12_20966733_20966933
cacagtcacctaacactgtatctcttctaggaatgatggattaatatttgcaaattcagtgttcc
cagcaactttatggaacataattattgcaaaacgtgagaatcaattgtacatataacctatatca
tacatattatataatcagtatcctattacaaatcggatgcttatatagataacattgatatttac
atact

>chr12_41820133_41820333
tagtaagaatttactcacctttacatcccataggtaggacatgtctggtaaactacacaaggaaa
caatgactgattcaagaccacctctaccactgactcttcattcatggcatgcaggaccaaagatg
ctatgctaaatgaagtgggtcctcatgggaagagtatatcttattgaaaaaggcatgatcttaga
atcaa

>chr12_69630733_69630933
ttgaactcctgacctcaggcaatccacctgcctcagcctcccaaagtgctgagattataggcatg
agccactgtgcccggccacatatatttaattggagtcagggaaggagagaagagagtggaggcag
aaacaatatttgaagaggtaatggttgagaattttccagaactcatgaaacacattaatctatat
attca

>chr12_72841933_72842133
tttcttgctaattctgttgacacaacctaatggattaagttgtgcactgcacaactctagaaggc
tgccattcacatttgttgccatcatagatttgtgcatttattaagactgttttctggttgatggt
attacagtgccttgcgtaagaggtggctttttctaaattgtgtaatggcttcatttgggctagca
ataag

>chr12_72848533_72848733
gcaggttctgagattcttcagctctaacaaatcctcagatgatgccagtgctgttgattcatgaa
ccacaccttggagtaacaaggctagagtgtgatagttttctccctaaacacagattgttcatggt
cctgcactacttgagtaaaaacccataaagctatacaatatccttcaagatttatttgcttccat
cctaa

>chr12_73765133_73765333
attctgtttcatgttccaaatggcagctattctccaggggataaatatttgaaagatagagatgt
aacagcttaagtttgtccatcatttttctctcttggtcattcatttttcaacttgagttcaggag
ttaaacaaaaaagaagaaacagagaaactgatttaatgacatcagattatttcttcaagggctat
aatat

>chr12_75003133_75003333
gcataatagcaattccttatctatttcccatttccttcaaaatttctccatatagcactttactt
tctgcctcaatagttgcatcttttatttacctgttttcttactccaattttatttttgctcctag
atctgtactgaaactattctctaaagtagtgactctctaatttcgtgtgcataaaaaaacaggga
agccc

>chr12_75408933_75409133

acatccccattcaactcttccatttggcctgtacagaagatagatggatcttggacaatgacagt
ggattattgttagcttaaccaggttgtgactccaattgctgctgctgtacctgatgtgattttgt
ttcttgagcaaattaacacatctcctggtacctagtatgcagccattgacttgacaaatgccttt
ttctc

>chr12_83793869_83794069
gtcctacaaattacgtttggtaatgatgtttaatactgtaagaaaataaagcagcaaagactcag
cactctgttgaaacacggaattagttttgggctttttgatggaaagtcagctgtagtaattttat
gtgaaatggaagaaggcttaaagagaatattgtatgcaaatatcctttatcatctccggagaatg
gtttc

>chr12_85224269_85224469
ccaaaagcttaaagctgtcagataaaatatattgcatgtaaaaagtgttaaagacaattcatata
tttttctacttttaagataacttttatttgaaatatatttcaacctcactataattttgttag
ttcagaaggtcaaaatctttaggacagctcctcagaaatccaaactctttaggagtactcatcaa
aaggt

>chr12_98662469_98662669
aactcaaaatgtgaaaagaaggtgttagagtgcagtggttttgggttttatttttgttttttt
tttcagtttttctttgttttattcccttttatgatctaaaataaattgttatctctttaaag
taacttcttgtatctataagatcttttcgtaagcctcacagtaaccacaatgaaaaaacctataa
taaat

>chr12_101088069_101088269
tctctgtttcccttccttccctcttccgctctttctttcttttctttcttttgtttttgttttga
gacagggtctttgttgcccaggctggagtgcaatgctgtgaacctggctcactgcaacgtcaatc
tcctgggctcaagcaattctcctgcctcagcccctcaagtagctgggactacaggcactacaggc
acatg

>chr12_126104847_126105047
atatattcttgcttgggacaaagctgagtaggcagagctgaattggcctttcccaaactgtgtga
atttgaaggacagtggccagtcatgcctatgaaagttataaatcttcagcaagtgagcctccttc
cacaagttaatggaaggatggagagatcagatgttatagatcacccacaaaatatgcatattatc
acttc

>chr12_127141447_127141647
gattctaagtggctggtatttcccagagagaggagggttgcaattatggagagtgggcagattga
ggtggagtaactaaggggattggcccagatgatgtgaaagatgggttgtggatttcccccgtgtg
gacccagcatcctgagcaccctagaacatcaagcgggtggagctccgagcctgcgcaggcagctg
ggcat

>chr12_130030847_130031047
acaactttgattcattcctgaaaccctgttgctaaatgaaatgctattgaaaatacatgtctgcg
tgtgtatattattcatattaataggaaagattatgatggtttccatttcaaataggcagctccct
agacattagaaacctaagctacacctgcattctcctttagagcacgtatattaacattgtcaagc
cccag

>chr11_21638824_21639024
aaacaaggaaatggaagatgaaaaattatggtgggatcaaaaatgaatttaatacagtaatagtt
tactcttcacttgcattagatggatagcaaagttggctccaagctttctagcaagtagtggtcat
aaagaaacattattattcacatggctcacagtggccaaaatagaatatgaaccagttaggccagt
aaagc

>chr11_24075624_24075824
atatttatacgattgaacatgttccagagattttacactacttctagctgaaaattatacagaat
ggatcaataaagaaatataataaggatcatcctgattatatagatcatcctgctagaagtatatc
atttagcccagagtatattaagatctatatggacaagtaataaaatcttactattctcaactgga
aatct

>chr11_27827024_27827224
gctttcagaaggaagtaaccctgccgacaccttgatttcagacttctggattccagaactaacag
aaaagaaattcctgttgttttaagccatcaagtgtttgtactagaacactaattagaggggttaa
tgagattttgctattattgcactccctaagtagacaggaggccaaaaaccgggataaagggattc

taatc

>chr11_31210824_31211024
ctaaaatgtgttaatttcacacattggaaaagtagataatgtcccctatagtctaacaaatgtaa
cttctgcaaccatactgctattgaactaaacactccaatgtaaagtttaaactaccatccatctg
tgatctctgtttttttcataataccttcttatctcaggactgaaacatccactatattagtcttct
atggc

>chr11_40455224_40455424
acatatatgtgtagttttataatatgtaagtcgatgatatttatcatgttgcccacagaggatat
atgtgtaaaaggaagtagtccaaggacttaaagtttgggaagatgaggtttatgaggatctactg
gtgagaaaccagcagagcgaggtgtcctgcaaatgaggagaagaaagcatttaaagtaggaagga
aggac

>chr11_60199424_60199624
cccacttccaagaaaaatcttggagattttaagaactaccagcacattcactgcaaccccacctt
atgttcccatccaagagaacacactggattgcagtaagatggaatccaatcttaatgattataaa
ctgacttgaaatccctttgtcatgagatcaaagctgagtccacaatagctataaaaagaacatgg
atgag

>chr11_79581552_79581752
agccataaaaaaggatgggttcatgtcctttgcagggacatagatgaagctggaaaccatcattc
tcagcaaactatcacaagaacacaaaaccgaacaccacatgttctcactcataagtgagagttga
atgatgagaacacatggacacagggaggtgaacatcacacaccggggcctcttggcgggtggagg
gctag

>chr11_102809790_102809990
ggtatatgtaaccattttaaaacaagtaaacaaaaaataaataaaccttttagttgctaatttt
acatatttttggcgcaagagatgggacacagataagcacaaaagatggaacacagataaggatgc
ttgtgcaatgtcctttaatcctgcataaattatgaaaaaagagtgtcactatcttcacttataaa
aaact

>chr11_103366590_103366790
taggcacctcaccccctgcagaatgtttcccttatctaattatatgcttgcttagaagttccagg
aactgaaacttcaaacaatctggcaccaacagaattctcatccaccaggagattacctcaaggct
gcagttaattaacctgattgtgcctgggatggcatcagctcattcatcaaatgagacaataatgc
gtaag

>chr11_105620790_105620990
ttgtttgaataattatttgtatgctaagcaacaatagatgtcataagattcagccaagcaaaatt
ttgattcaagagtgttttcaaagctatattataatactatatatggaaataactgtattttaaaa
tttgaatcactggaccaaataaaatactgcccaaggtatcatactaacataataacattgaatat
ttgcc

>chr11_115987790_115987990
ctctggactggaatggaaaaggggtggttcttcaaggaaacttgggatgctgttaccaaaagaaa
gggaaatggatgctattaccaaaagaaagggaaatggatgctggggcaaacaaaacaaatactac
ttagactccatgaccattgggctcctcaatctttgaatctgtcattctcctgtgctagcttaact
gtggg

>chr10_1539800_1540000
tacacacacacaggcacaagcatcagttatgcttttatacactaatcctaaatctcttcaatct
gaaattatatttcataaagaacttgttctgaggaaataaaattatatataaatgtcacttctatt
ttccaatcatttataagttaattgaggggatctcacatacattcctgagatatctgcagaagtaa
ccact

>chr10_19170194_19170394
gagtcaagtttatttatttttcagtcatgttttatgctttgtatcctgtttaagaaagtcttcct
taccacagggtccccagaatactaacctatatgtttttaaaatggaaaaatctcctgtcacatt
tacatctttaatctagaattgatttttttgtatgatgtgagatagagttcccatttctttttatt
ttaat

>chr10_56437794_56437994

accaggatgctgaggatatactaggtggaaattttagaactgctgttgttcaggattttctcaaa
agagaaatattcagaaatatcaggtagttactttacaaagtaacatatataaatatatgtatata
cacacacacatatatttatatatgcacaaagggtttcttaaatgcacatgtacctttaaagtaag
aatgc

>chr10_59869794_59869994
aaaaagaatggagcaacatagagaaagaggcagacacattaagatcctgtggtccgagaaagtga
gaaagccacagggagagtagctgtcttaggtaactgatggcacagcaatccgcagtcacaatttg
gttgatatacctctaagtactttgtctccatgtggtttctgtaaccttttgtcaaacatcctttt
gtcaa

>chr10_61174794_61174994
tcaagacaatccaggaaaaacatgaccttaccaaatgaactaaatagggcaccagggacaaagcc
tggagaaacagagctatgtgacctttcagatatagaattcaaaatagctattttgagaaaactca
aagaaattgaagataacacagagaataaattcaaaattctatcagattaatttaacacagagatt
caaat

>chr10_67040794_67040994
tttaaaaattatttttaaaaaatgatttttataaagttattcagctaaaagactcagcatatttt
catgtttaacctaaatcaaaacaaagtgaaattcagaaaaccagatttttgttttgttttgtttt
gttttgtttgttggttgagacagggtctcactgtgtcacctaggctggagcgcagtggcacactt
atagc

>chr10_67569994_67570194
ccacaataagaatatttttgttgtacggttatttcccccaaacgtatatttgaatattgggaaga
agaacactggaggtgggaagagtagacaggtatattctaacaatatgccccagatgatttagaa
atatccatcatatattttctcccacacattgagaaacaataaaataagagtagagaggcatataa
agaag

>chr10_77607394_77607594
tacaattctataacaaaaagatgacctcattaaaaaatgggtgaaggacatgaatagaagttctc
caaagaagatatacaaatggctaacaagcacatgaaaatatcacatgattcaacatcactaatca
taaggggaaaatcaaagaaggtaccaataaatggaaagacatcctctaaaagattcatcactaat
cataa

>chr10_82628420_82628620
cattacatattttgaatattactctgtatcctaaaatatatacaattattacacgtcaagtaagc
acaaaaaataaaaattttcttgaacaaaaactgcataaatgttcttacaatttttgctactgctt
ggtattgtctgattatcagctcaaagtccagattctcataatctaagtcaggtccaggtgagtgt
gaggc

>chr10_83516420_83516620
gtattcacttaatttcattgataggttcttggtattgggactcagagaaaaatccaccaaaatga
aggcctcagaagcaaaactttttttctcttagcttcccccgctctcctgtctctcagtcccattg
tcccttgaggctttaatatagaactaggatttctcttccccaaggtaggtcatagaaaccagaac
ctctt

>chr10_89328620_89328820
cagaagctgatgccagcactgtctcctgtacagcctacagaactgtgagctaattaaacgtcttt
tttttttttaataaattacccagtctcaggtatttcttcatggcaacgtgagaactgcctaatag
attgggtgacagagtgagaccctgtctcaaaaagaaaaaaaaggagacagatagactccaataca
ataat

>chr10_90436220_90436420
caaagctgccctcaaagtactacacccctgtgtgtgagagacttcccaggacttaccactaccca
ttgccattacacagacctgtcccatatcccagatccccagcacactgtcctctcctttctgaagt
actaatacctgatataataagctaataattctaactgttcatggaatttctggtcttcttaaaca
atacc

>chr10_118292410_118292610
agggaagaatgtggctgttaagatataaaaggtggcaaagaattctaaatctcagagaccaactg
atgatcaagtaaatgcttcatgttcctgattgaatgttgaaagacatagtgaaaatggtaaattc
aatagaaatctctcttaacctctgcctacccaaatcactgttttcactcatgttccccattctta

cggtc

>chr10_119907210_119907410
gggttggatctgggcaggacaagaccacatgagtggtgcctgggtattccagagtgaccactgtt
gtgcaccacactttctgagcatttatttcactttaaggcaattacttgtttatttgtctttgagt
tcctcaaggacagggaccatgtttggcgggcttagcagtgtatctccagggcctggcacggtccc
tggca

>chr10_129314010_129314210
actatctcctctctaaaaagctaccttgaaaataaacttccacaaatacaaaagcacatatggtc
aagcttattaattgtgctgccttttgcaatagcaggtaattagaatcaatcccaatgccatgaac
aagagactagctgaataaatgacgacagagcctgcagccatgaaaaaacgtagtaggaagacctc
tctgg

>chr17_11284075_11284275
atcaccacgtcaggcagggctgcctcctctgaaggcttgactagggctggaggatctgcctccaa
gacgcttcactcacatggtgctggatgttggcaagaagcttcagttcctccccgtgacattctct
ccctgggtagctgagtgtcttcactgcatggtgcctggatttcctcagggtgggtaatcagagaa
ggaga

>chr16_6837599_6837799
tctgttctccatccactgtgaccttggaggccaggttttgaagacacgttattacaagatgaaag
agattgcatccctgagtgattcatgatcgtagcattccattgattactctaaaccaagctgcaaa
tgagcacagagcttctgctggcttaatccccaaaacctggagttgtaaaggacttagtcttcctc
cataa

>chr16_32463099_32463299
aaaattttaatgtttaaaattttcttatgtgtacacatgtttaatttatgtaatttcaaaacggg
gtatcatacatggaatttggtagttttctttccttttttgttcacttactctgtttttttaatgc
tttactgtaacccacaatgcagtgtccccaatatttattatgtataactgatattcacataaaac
atatt

>chr16_55932099_55932299
gcatgctacacattatacttcaattaaaaagtaaaagcaatagtgggtaattctctagttcatac
atgtaaaatcacatgtagatgcatcaagaaactctgaaacgtggccatgaggtgtctagggtggg
tagatctgcaagataggagtttgagtgattttatttttttcataattttctgcattgtttgaaa
ctttg

>chr16_60981099_60981299
actaaggcatctgcctacttcttaaactataggagccttctttcagttcattaaacaatatgctt
taaggaaaaaaaaaaaggaatccattgatactcttctaactggattacacttgcttctctattcat
ctcgttataacaattattccttaaattggcatttaattattcaggggggccttctataacctctag
ataag

>chr16_87187699_87187899
ggcatagaccctgaagctggggcagaggacaatggatgcttccagcatcatggctgccctcccgt
ccatggcacagaacacatttctcctcagaggtcgctcctggtctcatcctcgggtggtagcactc
acagcattataaccacgtctctccagccatccagggaaaacgccaaagatgttacacgatatggc
agttt

>chr15_54888908_54889108
gtttgctgccctccccatggaaatgagtgatttattgctctattagttcacatcagagctggtta
agagcatgggacctcccttttcttttgcaatgtgacacagctacttcccctttgccttctaccat
gaataaaaactcccagaggtctcaccagtagctgagcagctagtggtgccatgcttgtatagcct
gcaga

>chr15_91757196_91757396
caccatttctctatgtgattcagtgttattgaatggtatgttcaaacaccttttgaagtcatgtc
agataccatcaaaggtagcatcttccactttgggaaacacttaacttggaaatcagtcatatggg
aacaaaatatcagctcatgactactgggaagtggaccggttccggatctctagaggagggagctg
gcatg

>chr15_94688596_94688796

ttggctttcctccacagaaaattagaaaatgctttcatgaaaaaatccaagataaatatggagtg
ctgtctctgcttctctcttctcagggaccagagttgtataagccgacctctgttaatttatcttc
aattacttacaagagttgttccatacatagctgttagtggaaagtttagtcttagccaagccaat
ccata

>chr15_94823796_94823996
ttgtatgggtttttgtgtctcaatttgattcagttcaactccaactttgttatttttcttctgc
taacattgggtttgatttgctcatttttccagttcctctaagtgtgatgttagattgttaatttg
agatctttctaacttcttgatgtaggcatttagcattacacactttcatcttaaactccaactgt
gtccc

>chr15_101307477_101307677
gagggccagaaactggaaaccagatgcgctgtccccctcccttccctcatccttgtccccccac
ttccgtcctcctttccccttccctcccttctccttccctccctttctcctttctctcccttcct
ccctccatctttctctccttccttttctccctccctctctccatctcccctcccgcctgcccca
tttac

>chr14_25979760_25979960
aaaatatgcaaatgctgtatcttccaattagtttttcagtagaactgactgatatcaaagaagat
aaaaatttacaagatgaatttctcaaatatgaaattggtggttaggatttaaaaatgtgtcagga
ttcagtaagcatatccaaaggggggcttctgtcttgcctctgtgtatctttgtaaggtgtggaggt
gtgtt

>chr14_33466649_33466849
tccctcacccaagtctcatgttgcaagtcccacaattcccacgtattgttagaggtgattgaata
atgggggtgggtctttcctgtgctgttcttgggatagtgaatgggtctcatgagatctgatggtt
ttaaaaacgggagtttctctgcacaagctctctcttttcctgctgccatccatgtaagatgtgac
tggcg

>chr14_72890247_72890447
ggccattatcctaagcaaattaaagcagaggcacaaaactcaatactatgtgttctcacttataa
gtgggagctaaacattgggtcctcatggacacgaagataatagtaactgacactgaagactacta
gagggggtagggtgggagggggacgaggggtgaaaaacaaccagcaggtcctttgctcagtactt
gagtg

>chr14_84256047_84256247
aaacatttatagtttttctgaaccatagaaaattagcactcatatgatataatgagtatattaat
gaataagaaaatgtagactcagagaaatcaagtgaattgcagcagatcctcatgtatgaatcatc
tgctttcaatcactgcccattgcttttcactgtactgaaataggttgggtcccaaggagagcaaa
ctcgg

>chr14_90254847_90255047
agctactcaggaggctgaggcaggagaatcgcttgaacccaggaggcagaggttgcggtgagccg
agatcgcgccactgcgctccagcctgggcaggagaagcaaaactccatgtcccagacaaaaaaaa
aaaaaaagtgccgcaagtggtgctctacacgggtttttttgctaagtattccataacattattccg
aaaag

>chr14_94119247_94119447
ggttctctccttctctattttctgaagttattgtaagttcggcgttattttttcctgaagaatat
ttttgacctaaaactttgaaccagcatttcagggtagatgtggcacaatgaggtaataattggca
taattaggtaagcagtacttcttactctttatctatttacgttctttgattttctcttttttcct
tctgg

>chr14_97231847_97232047
agttcattgtagattctggatattagccctttgtcagatgagtagactgcaaaaattttctccca
ttctgtgggttgcctgttcactctgatggtagtttcttgtgctgtggagaagctctttagtttaa
ttagatcccatttgtcaattttggcttttgtcaccattgcctttggtgttttagacatgaagtcc
ttgcc

>chr14_105055155_105055355
agagctcaagcactccgggggctccgagacagcctgagccctggccctgctgcttggtgaatcat
gggggccaaaaggggctgctgcctgagggctaactaggaaaaggggggaccccgtggcgtgagatc
ggacatggggggcacagcaggcggccccgccacactagtcagcacagccctcctgtctcctgtgt

tgggt

>chr19_56529388_56529588
gacccattttacacagttcttcccatcttctgtatttctgtggtgtatatacacaatgggttat
tgaaggcatttcatttgcgtcccaaacgcaatccacttttacatacaatcaatttttcatacaat
aaccaaggcgatctaaaaatttacgcctgtaacttgattcccatgcttaaaaatccttaagtggt
tttta

>chr18_48670602_48670802
aagtttggaattattcctctttttctattctctggaaaagttgtgtaacagtagtgaaagggttt
ccctaaaagtaaaaacttaccactgaagctgtctggaagtgaagtgttctttgtggggaaggatt
taataagaaatttaatctccttgatatatatggactatttagattttctattcttgtgtcagttt
tgatt

>chr18_49551002_49551202
ggacatgtttttaagccacaggtctacagaaaaatacaatgtgagccaagatccctaattatacc
gaaaatcaagaaagtgctcaaagattaatgtagatctgtaaaaaagacaaaagaacgtgtttaag
catgctcctattgatcaaattcaggtcaatttaagcatcatagtgaataatcagtataatcaatt
gtaac

>chr18_53275202_53275402
caaatgtgctttgccctgctaatgctgataaaaactacaaatctgtctccttcactgttgatcta
caaatggaagctgcgtgccacatgcttcgtctcactgtaaccttaatggaaaagcagcagggctg
ttagcagaatttcctagaccacaaaaagataatattaattcaagcactttaggaaaattctcaaa
attta

>chr18_58849020_58849220
agaaagaagaatgcttatttacttctggtaatgtaaatgagttcagccactgtggaaagcagttt
agagatatatcaaagaacttaaaacagaactacatttgaccttgcaatcccattacttggtgtat
actgaaaggaatataaattttccaccaaaaagacacatgcacccatatatttatcacagcattat
tcaca

>chr18_76004212_76004412
cacacacacacacaaagggaatatgtggaagtgtggcttactgctgtttgaataatcctgaggct
gctaaaactgtaggtgagaggctgaaagagagggttgagggagctggctgtggactctctcttgc
cctagatggacttggccacacatgtgctgatgtgagaccccagggtcgtccccctcctccaacca
aaccc

>chr22_16262600_16262800
aattttaaaaatacatataattaccaggcaaaattgttaaaatgaactctgtcaaacactttta
agtgagaatcaatcaaacaatatagccaggataaactccattcattcatttaatacttatttatt
aggtagctacgtctgataggctaggcctttttctaagaagtaaggatatggtaatgaacaataaa
aaccc

>chr20_6340000_6340200
cagagatggccaactattcagaccatgttcacataaggcaaatgtggagccataaccaatctggc
tgtttctgtacttcgtttccattttctgtatgtaactttcctttttctgtccataaatcatcttc
aactatgcagcaatattgtagtccctcttaacctatcctgtttggaaggtggtgggggttgccag
ttcat

>chr21_20697529_20697729
aatgttatggccatgcactggctacactatgaatactatgtaatattgcacgagatacaaaaagt
tcagttagaaaatataagaaaaaaatttgacttaactagaattcttaactagaatttacttaactt
tacttaactagaattctttcttccccagtgatttgttactaatgagattaatgttttgtaaaaat
taata

>chr21_22307529_22307729
ctcatctggaaacagagagcaacatctttcatctaatgtctatagatctcctagcacttacctcc
cgggagaatgcactaaagaataatggctttctttctttccctttttttttttcttcaattttg
agatggagtctagctctgtctcccaggctggagttcaatgatgcaatctcagctcactgcaaact
ccacc

>chr21_23199729_23199929

aatgcactacatatttaattctgccagaaatttttttacagtgctgaagcaattatctctgggt
aaatccaactgggccacctgggtcatcaccttgacatgcagggtgttctctacagtatattctta
gaacatttgcaagttgcaaatgatgtaagctgtagttcttcataaatgacttcatagccaaagta
aagct

>chr21_25872929_25873129
aatttattttcttttgaatatttatccaataatgggagcagcagacatcagccctgggaaataat
ttatgattaattcctcaaaagcaattgtaacaacaactacaaatgacaagtaagacctaattaaa
ctaaagagcttctggctgagcatggtggttcacaccctgtaatcccagcacttcgggagtccaag
gcggg

>chr21_39561930_39562130
aacttacagataacttgtaaaaatcaattttaaaagataacctaattttaaaatgttcagaagtc
ttgatcttgatcaggcatattacaatatagcatgggccactgatcaagtctccagcagccaaatc
cacccccctgccccagcctgtttcttataaataaatttcggtggaacacagcctagcctattcatt
cacac

>chr21_46042372_46042572
tcgcagttcttagggccaggcacctgaaatgaagatatccacagggccatgctttctcttagctc
caagggagactctgtccttgctgcttctagtttctggtgctcccagcaaaccttggcgtcccttg
gcttatagctgcctcatgacaatctttgccttggctgtcatacgccgtcttccctttgtgtgtgt
gtttg

>chr7_21031675_21031875
gaagagcggatttctcagccagttaggaaattgagcacagacactatgctcagtcctttgaccat
agtaattcattagttttcaactggtctcccagatgatatagtgactgtcaaaatccagttttcca
cttcatctgacaactcataattttgtgtttgtgagtatttgttaacaaaataacttaatatggta
tgaat

>chr7_25200675_25200875
gtctctatttaaaaaagaaataatttgtttctctaaaagtccccatttggggctttaaaaatgt
ctcatataactcatttccacttaaaaatgttatttgattttatataaggaaaaaaacccagactt
acaaagaatcatttccatagacatcactttttttgttctgggtcaaataataaaattgttcaaca
ggaag

>chr7_31522275_31522475
aacggagtggctggaggaagaaagggtgagggaaaaattgagggtaattgattcttggttcttag
cttggtggcaggattgacggtgactatgtaagggaagactgaaaatagggagagggcagaatttg
cagtgtctctggaatacacgtggaaatacctgggaggccgctgagtggatgggtctggtgtgcag
aagag

>chr7_50040854_50041054
atgataaaatcagagacttcaaagaacctcaccttatgagttgtacggtatttgctatgacagga
acctaaaattttctcctggaggctttttaatgcttggagacaaaaactttaaacaatatagggaa
gaaataattgaacatcttttccttcttctttgcactcccatcagtttttcatttgtgatacatat
aggtg

>chr7_53415706_53415906
caaacctgcatgttgtgtacatgtaccctagaacttaaagtataatataaaaaaatcttattttc
ttaagccatcaactatgcactgagtatcctttcctttttgtcccatttttttaaaattgctcttat
tatttaagttgctctgtctccttcattggctctatttggttgaatcctcttcatattctgtttct
cttct

>chr7_71686264_71686464
cccagtgttttcacttacacaagtcttggcttctttttttttttttttttttttttgagacgggg
tatctatctgttgcccaggctggagtgcagtggcgtgattttggctcactgcaagctccgcctcc
tggattcaagcaactctcctgcctcagcctcccgagtagttgggattacaggcacccgccatcgt
gcctg

>chr7_78489064_78489264
ggcgttctctgtatttcctgaatttgaatgttggcctgcctcactaggttggggtagttctcctg
gatagtatcctgaagagtgttttccaagttggttccattcaccccatcactttcaggtacaccaa
tcagacttagatttggtcttttcacctagtcccatatttcttggaggttttgttcatttcttttt

actct

>chr7_79308464_79308664
atgaaaaaaactaccaattcacttctgggctttcagtgacaaggaaaactgaacagatcaaggtg
gtttaataaagattcaaaaccagatgcaaatatgatgaattcactcagtgagctttttggaaata
tgccagatcttaactccaagaggacaccaagtttgaattaattattttagcttgaataaagttat
tatgc

>chr7_82813064_82813264
tacattgctataaagtactacctgagactaagtaatttataaaaaaaaaaaaaagaggttcaattg
actcacagttctgtgagctgtacaggaagcatggctagggagccctctagaaacttacaatcatg
gaagaaggtgaaggagaagaaggcatgtcttacatggctgcagaaggaggaagagagtgaaaggg
gaggt

>chr7_86329864_86330064
tcttggaatgtgttttctaccttctttagcatgagcaaaaattgccccacactattattactgaa
gtcagacagttatcttagttatgtcccaatatctttacacagaaaacaattaaggaagccagaaa
gagaaatactatgccatatcaggatcctattcctgatgttttacagagaatacaagtcatttata
taaaa

>chr7_109982764_109982964
gctttcaggctatggttcagagagatcataaatccagtctttctcactttcttctttaggatacc
tcagtaaaggagatgaagcattcattccacaactaaagattaggattaaacctacataacctatg
ccaggtgggattcattggctgataatcatggtcaaaatcgcctagtccataaacataaaattcct
gttcc

>chr7_113543164_113543364
gtagagtagttcctatatatccatctaagaggataaagattgaggtgcctggattaatctttttc
actaggtaaaagtgtaaactttagattttgaaggaatgaataatttggattaaactaattacaat
aagaaagttcccttaagcaccaccagagtagtaagatgaatcacccgtgaaataggtatagaaat
agcta

>chr7_118173164_118173364
aaggagagagagagattgtcatgctgccatgaaatggatataaacaattttcagctgagactctgac
caaaaagtaagttttccatcaacaagaatttaaaacagtctttgatttatggcatccagcagaga
atgcaatcacagatagcacagccaggaaaagaacattcattattttagagagacaaagaattgtg
gaatt

>chr7_125323764_125323964
aggtgtagagtttttttgttttgttttgcttctttttttgtgtacatagtgttgttattatcagc
ataaaataatgggttatgagataggatttgcaagcctccatagtaacctgaaattgaaaaatgta
caatggatacagaaaatataaaaagcaagaaattaaatcataccaccaaagaaaatcgcattaat
taaac

>chr7_144428067_144428267
agttttatgtatatatacatatatttttttgagacagagtctctatcgcccaagctggagtgcagt
ggtacagtaatggctcactgcagcctcaacctcctgggttcaagtgatcctcccacctcagcctc
ccgagtagctgacactgcaggaacagaacaccatgctcagttcattttaaaatttttttttgcag
agatg

>chr6_12687214_12687414
agcaatatagctgtaaaacagacataagtagggtgaagtgtgaatgggaaaggagaaagaggaaa
gcacaagtatagataactctttcaagaaatagagctgtgaaggaaactgagagatatggcaatac
atagagagttacgtctagaaaggagagagaagtgtttaaatgctgcgggaagcagccagcagaga
gaggt

>chr6_19885221_19885421
ctcaatcccaggtcacaaaggttttctcctattttatgttgtagaacttttatagttttatgttt
tacacttaagtctataatccattttaggttaatctgtgtatatggtgaaagtatgaagttttttg
tttttattttgttttgcatgtgttctagtaccagtttttccaacaccatgtgttgaaaaactatc
ctttt

>chr6_22284021_22284221

tttaaaaatgaacatttcttatgtttcagccagacttacttattattagtgatactttataatgc
tatatgtagtaacatctcagttcctttgttcgggccatagggaaacctacatcataaaatgccgt
aagttaattggcagataggctgtcttcctatttttgccttttagtatcccatcaaggcttcaaaa
cttcg

>chr6_55549241_55549441
ttcattttaggtgcaatttccttcctcgaagtattgctttggtcattctcagtgatggtctggga
atggtaaccacttaaatttttactttctctaaatatgtctctactttacactcaatcttgaatgg
tagcttaactgagtagtcattttattctcctaacttaacagtatctctagaaaaggaaaagcgtt
taaga

>chr6_63883641_63883841
ttctcaattgtcatttttcaattatgtctctttcttgctcagatatcactggtggatgcccatgg
tctaaacgttaattccaaagtctttatcctaaaaaatgaaaggcagttctataataatcaatatt
tgtgttgtgcaaatagtgggctactctggtctcatctaggtgtatttgagcacttagaaaattag
aaaaa

>chr6_76300080_76300280
aggttcttgcgtctgccaagaagtgacaatttttattcactcactgtaaggctagaaactactaa
agccagacattttgtgcacactcttaaatacaacattttattcaaagacttggtaaaaggctagg
ggcattggctcacacctgtaatcccagaaacttgggaggccaaggcaggaggatcctggataact
tgagg

>chr6_77923881_77924081
gaacttgaagaaattttcagaagaaataaatgcatatctttaattgagaggcaaatgcacctcat
gggtttgcaggccaagccaagatgaattgagtattcctggttgcaggtgaggtgaatgggctttt
agacacaggggcttcccaggaatagtcattgcctataagcaagccacagagtgtttagaaaaggt
gttag

>chr6_78362281_78362481
ggtacaaaggtttggtatgcaattagtgcactttcttagaatataacttaatgttaaaggatagg
aaggaagatattggctatttttgaggagaccgttatccagtaatcatagcttccttatgtgccag
cagagtgcctagaatccagctggcttatctatccattcatgttaagttattcagctaagattttc
agagc

>chr6_88180881_88181081
gggctgaggtgggaggattgttttaggtggggagttcgagaactagcctgggcaacaaagtgaga
cgcccccacccacaatctctacaagaagaaaaaaagacaaaaaaaaaaaaaaagtaaaaacagct
ggtgcagacttgaaaacagaggcaggcttacaagtgccttttggaacaaattgagcatttagaaa
ttata

>chr6_92545879_92546079
tctgctatgcagctatttccaacaactaacagaatgacagaagttagaaaagggaaaatatgggt
ccatagttaaaaaaaaagaatatcatgaaaggggttgtgcactacagaggtatagcagaactcag
tagccccacacctatcagttacttcatagattaattcaaaggttctgtttaggcccttgactaag
gcatt

>chr6_95386079_95386279
tccttggaatgtagaatgaaatagccaccatggaaaatagtatggaggtcccttaaataaattaa
ggatctgtataatatatctgcatttccatgtttattacagctgtattcacaatagccaagatttg
gtaacaacccaaatgtctatcaacagactaatagacaaagaaaatgtgctatagacatagaatgg
gatat

>chr6_102266707_102266907
aactttgcttctgcttttattttccgcaaatatatttcatagggcatggcacttatttttcagaa
tttttctcattataattatattttaactttattttcaattttattgttttcatcttggaaaatt
aattttactttatgaatttccctaatgtaaccaaatggcaagtgctttttaaagccagaaatatt
ttcag

>chr6_103258107_103258307
aattcaatgaaatagaaatatttatttctgaatgattttctaatatatgttcatttatcttctta
tttatttaaattaagagtgcagaaaaatattacttgatagagatcattcttttaattgttagaaa
tattctatgtatagcaaactttatttaaaaaaataaaaactgcattcctcaccacaaaaagtttt

tttcc

>chr6_103650907_103651107
ggatttttgtgaatctataaactctgtaaactcattttgtgcaaggctcccaccacgttgtgtga
gaatcaagggcataaaggtaaattaaatttcatcatggcctcaaactccaatccagttatttaac
atgattgcccggtaaaacaacagtgatctttttaacccttaaagaaaagctgctgtgcagggtt
agttg

>chr6_117171707_117171907
cttcatggtaccccctcccctcacaggcctggaggccttaggaggaaaaactggttttgtgggcc
aggcccatggccctactgctctgtgcagccttggcacatggtgctctgtgtcccagctgctccag
ctccagctgtggctatgaggggccaaggaacagctcaggctgttgcttcagaaagtgcaagctcc
aagcc

>chr6_127183707_127183907
cttttggtttttagacatgaagtccttgcccatgccaatgtcctgaatggtattgcctaggtttt
cttctagggttttcatggttttaggtctaacatttaagtcttaagtctttaatccatcttgaatt
aatttttgtatacggtgtaaggaagggatccagtttcagctttctatatatggctagccagtttt
cccag

>chr6_142229907_142230107
gagttatatatgtatggtatcatagtaagccacaaactaaatgccttcccatgcttataataaaa
agaaagatacatacaagcttgtaagtgagcataaatctttgtcatctttatccaaccaacccaca
gaaattgaacagaactcttcgtttaggttaaccaaaaactgggcagtataaatttttctcatat
ttcag

>chr5_9692800_9693000
tctcccagatctgcaggcctaaaagagccaggcctccttggataagcatttatgttggcagagtc
catgactcagcatgatatcctttatgtgatgcggagctggatttgctgatctcctctttgtagat
aagcctcgtcctggccatatcttttttttccctcaaccctgatttcctcacttatttctttaaaa
attta

>chr5_18689843_18690043
cttccatatttcatattcagatttggagtacaattgtattaataggacaaaataaaaagccatct
gaaatgcattataaatattgtttatactcagatttggagtacaattgtattaagctgcctgaaca
tttacaatagagacgtatttaataaaacgctatttagccgggggtggtggcaggcacctgtagtc
ccagc

>chr5_20563243_20563443
aaaagcataaatgtctctgcaacaaataatatattttatttttcaattacttcacagttgaaaa
atagaggatggggcagagtgagaaaaagtatattggtgtcaacggaaatgttgacatatgtgaga
gctcataaatgaaagtatcatctaaacacaataacaaaagtctgaaatttacaagttaaaatgta
gacaa

>chr5_20658443_20658643
aagattacttcttccctgacaaaatttggccagagaatattatataaaaacactagccttctta
ttgctcagatctgtgttatcctggtcctgtactcagccttcactgacaccttgaccctgaattac
attatttccctttaagttcaggcttgaactcctttatattcctgggctgttcttccagattatag
acagt

>chr5_25492043_25492243
tatttcgtatacagaattatatattaactacaactcttattcttagtaacattacatgttgctga
tagaccagcaacaaatcctggtctatcaaatattagcattttatagatgagaaatcctacaattt
tggaaatgactcagacatcccaggagaaacaaaagtaattttaatatttttaaatttcatttatt
ccatt

>chr5_27108243_27108443
tatccaactcacacagcaccgaagtatgttactggtggcaaatccatatgggtctgaaggaacct
cagttcttctctcctcagaagaaagaatttgactgaggggcataaggcagaggagagaccaaggc
cagatttagagcaaaagtgaaagtttattaaaaagctttagagcaggaacaaaaggaagcaaagt
acact

>chr5_45081843_45082043

tttgttttttcttttgctgttgagttttatgtgttccttctctatgttgagtattattcttcta
ttcaatatatggtttgcaaatattattttcccatgttgtaggttgacttcgttttgatgattgtg
tcctttgctgtgcagaaatcttttcgtttgaggtagtcacaacttttttttttttttttttt
ttttt

>chr5_77600044_77600244
atttatttatttgagacagggtctcattctgtcacccaggctggagtgcagtggcatgatcacag
ctcactgaagcctctaactcctgggctcaagcaatcctcccatctcagcctcctgaatagctggg
accacaggtgcacaccaccatgtctggctatttttttaaaaatttttggtagagaaaatgtttca
ctctg

>chr5_83877044_83877244
ttaggcctactttcagagctaaaaggcctttgatatgaataatacggtaaaaactagaaaaaaaa
aacaatttggattaaaaattctcatttattttctattaaactcttcaactagagtaaatatttga
aataattcttctctccagataaaaacaaaagaatatcatatagcatttggggtgaagatagaata
taatc

>chr5_87399444_87399644
tatatttgttagtgcctgtaggcacccttaaatacatgtggatccttttatataataataacaga
tagaatggtaacataaaagtgtaaactgctttaaaaaaagcattatattcttgaactagttgagt
tgtaaagtgtttctaaagaaagaatttcacaaagattaaaatgtcattatttgctcataatcact
gcgta

>chr5_100480501_100480701
ctgtgtatggattatcaagtcatagaaagtaaatgtcattttaatggtgcccgttattgaaatag
agaatagctataacaatgtaaatctgtcaaaataaaatttactctggtaataaaagtttctgatt
gcttgactactatttaattcaggtagtgtgaaagatttattgtactttacaaattatttatctta
ctctc

>chr5_103798101_103798301
tcagttctttaaatgtttgatacaactcagtagtgaagtcattgggtcccaggcttttctttact
aggagacttttactaaggattcaatttcattacttattggtttgttcatgttttggatttcttc
atgattcaatattggtaggttgtatgtgtctaggaatttatccatttcttctatatttttcagtt
tcttg

>chr5_103918901_103919101
tgttttagcaccgtttcttgaaacagtattctttctacatcaagttttctttatatatatatatt
ttaaaaatcagttgactatatttgtgtggacctaattttggactcattatttcccatttactggt
ggaattcttcaactttatttcctttcttcagtattgtgttggctggttatcctagatatttttcc
tttct

>chr5_111103101_111103301
cagacatctttccctttgtctaagtgtccacagcacatttctacctgaaggacccacaagcatca
taaactcagctaatccaaaactgcttaccatctcccttgaatagctgggactattttctgttctc
tgtttatgcaaatggtctcacgtccacgtactccatcaaggtgaaaaccttcctcaactccctct
tccac

>chr5_116914501_116914701
tgttattatttttttgaccatactggtttatttatacaattttatactagatgcttctctcatttc
cttgataacacagctgcacacacacacacacacaaatgtctgtctattgctgaacaatgttaagtaa
tataatttgacacatgtacattgcaaatcaataaatgtgacattttcttttcctggtacattctg
tataa

>chr5_117760701_117760901
ctcagaaaaatcagatgacacaaacaaatggaaaaacattccatgctcatggatagaaagaatca
atgtcatgaaaatggccacactgcccaaagttatttatagaatcaatgctattctcattaaacta
ccattgatgttcttcacagaattagaaaaagctattttaaaattcatatggaatcacaaaagagc
ctgta

>chr5_121244701_121244901
aataatataaaataaattagtcaggcatggtggtgcatgcttgtagttccagccacttgggaggc
tgaagctgggggatcacttgaatccaaggggtcaaggctgcagtgagcaatgatcatgccactgc
acttcagcctggacaacaaagcgagaccctctccccactgttccccttaaaaaaaaaaaaagagac

aaatt

>chr5_125252501_125252701
agatgagaaattgctaggtttcagcaaaaactcagaagagaaatacaaggaaaaactcctaacac
tgtaagtggatgttacagtgtggaaaaccaggacattttaacagagaatgtccagaatggaaaaa
gaaagagaagataatccctgtgatgactatttatgaagattagggggtcaggggctccttttaa
gtagg

>chr5_128554101_128554301
ccagtgtgccccttttaatttacctattgacacttggggaaaaaacaggtgtattaggtagatga
tggtggatatctctaaattgattcaaacagtagttccaatcatagctgctatacttgacctaata
tatttactgaaataaatcaacaaagcctccgacatttggaatgtactatttgttaaatatattct
tctta

>chr5_133809301_133809501
attctaatagttggtggattctataggattttctatatacgagattatgccgtctgtgaaaagag
atcgttttatttcttcctttgtggtctggatgacctttatttctttttcttgcctaattgccctg
attagaatttccactacaatgttgagtatttgtggtaagagcagatattcttgtcttgttcctga
tctta

>chr5_143925007_143925207
ctttctatctgtattctatttttacccgcagatatcttagcaggaattatttgacttttctgaga
tcttccttatttcaatcactgatgaagactttccctattaaaatccacacaaaatttttataga
agtggggcaacaaagtgagtgagcttgtttatagtatatgttattgtgtattttataacatatta
caata

>chr5_161059822_161060022
ttgtaatacttggcatatccatattttcatgcagaataaaattgtttcataatctagcatgcatg
gttttccttacatctaagaaatgttgtcatcacagctattgctgatgcacagaagaaataaaaaa
catggagtaagttgactatatgaattataagaagttgaacactttggatagaatggtcttatgaa
ctaca

>chr5_165145622_165145822
cacttcttgctgtgggatctctcttccttcatttcctcatctgcaaagctgaggatataatagtg
cctatctgtattagtttgttctcacacggctataaagatattatccgagactgggtaatttataa
caaagagaggtttaattgactgacagttctgcatggctggggaggactcaggaaacttaaaatca
tggta

>chr5_165362822_165363022
cagaatatgcctataatgttgataatccttttgaaatgcattatccttcatatatacaaagctaa
ttaaaatgtcagtttctttatgtgatctggcttattttgtattttctctaataaaatgtaagcta
caaaatggcagaaacatttttctgtcttattcactgttcctcctcagattcctagaactatgct
tagca

>chr5_169553022_169553222
gtgtatacataggcctttactctatcttttatctttacccaaatctctggctggttcctgaacca
tgtttgtacaggatgggctctagacagatgagaataaaagaactgaacttggattttagctgcag
cccaagatgcaaaatttgcagtttgagccaaaccaggttaattgcccactaaagcaaacaaacaa
caaaa

>chr4_14252902_14253102
acccatctaggtcacggaatagctttgacattttcatctctctgaaacttagtttttacatattt
ttcagaagaaaaagaatcgaatagctgggccacagtgttataggatttatgcaggtaagagaaca
aagttttggaatatgattcttttgttattttccagttgtgtgatcttgggcaagtcactcagata
actta

>chr4_27591702_27591902
attgccttttaatttattcatcaggctctcatgcatgccgaaagcacattactgcacattgaaag
gaaaagaaaataattttgtctctgctttggttcatattttctcaattcacaattatatttaacct
cttaagaaactaattcaggcatgctcaaataaagctcatcaaagtagccacagcataaaagaaaa
atcct

>chr4_29333302_29333502

ctacagaatgggagaaaatatttgcaatgtatccatctgacagaggtctaatatcccggatctac
gaggaacttaaacaaatttaaaagaaaaaaacaaccccatcaaaaaggcaaaggatatgaataga
cacttctcaaaagaagaattaatgtggccaacaaacataggaaaaaaagctcatcatcactggtc
attag

>chr4_62230005_62230205
gcaccactgcactctagcctggtgacagagtgagactccgtctcaaaaaaaaaaaaaaataataa
taataataattttaaaaatcatgaaatatgagtatatgggcaatatatgtcgtgatttataaaaa
ttaatggtatcactgtattgaggatttggtttgttttgcttaaaaacatattgtacttcttcact
gcttt

>chr4_62867405_62867605
tctgtataatatgtattcaaaatggtatagtaaatgtgagagtgttcgctttaaagatataagta
tcatttatctggaaaactttttttagtatattggggaaagcattaaagttaatgtttacttgta
tttttcaaaaatgtttgtaaaatggtgaacaagacaggccccccttgttcaattcattaagaataa
aaaat

>chr4_63433805_63434005
gttattattttgaggtacgtttcatcgatacctagtttattgacagtttttagcatgaagtggtg
ttgagttttgacgaaggcctttttctacatctattcaaataattatgtagtttttgtcattggttc
tgttcatgtgatggattatgtttatttatttgtatatatttaaccagccttacatcccagggatg
aagcc

>chr4_63448005_63448205
atattctcgattttgcacttcagtatttcttgatctttattcctagggtccaagaagaactcctt
ccagatccccctctgctgggtattcgtccaatctcaaattatctgtgcaggtcatctaaagtaga
tctttttttcattgtgatctgatttaattgatataaattcccaaccacactggcaagatcaactat
atcat

>chr4_63526405_63526605
ttttggctaattttaatattatgttatctgaccccaattattgattcaggtaacatcaaaattaa
taagattcattgctttttacatttatatataagatgaaggactggaaagaaggcagagatctcaa
agattaggagacacttctggttgtaagaaccagaagtgggacttatgcaattaacttggcaattt
tcctc

>chr4_65270605_65270805
acatgaatgctgtgtcaaattcacatgtttcttgaaccggagtgcacttattcaacacatattgt
ttaaacactttatttgtgttaaacattgctattaatgctgaagatacaagtgtgtaaaacaaaca
aacaatctgatatttttgtgcttatatactaagggtaggccaaataatgaactaaagaaagaaaa
ataaa

>chr4_68836005_68836205
ctatttgatttttctctctttttttctttattagtcttgctagcagtctatcaattttgttgttc
ctttcaaaaaaccagctcctggattcactaatttttttgaagggtttttttgtgtctctatttcctt
cagttctgctctgattttagttatttcttgccttctgctagcttttgaatgtgtttgctcttgct
tttct

>chr4_82849576_82849776
atggaggaatacggagaagaaacagaattttgcaactcaaaaataaccttgtaaacagtgaagag
cctgctttcctccaaagaagaatctaaaatttgagtttaaaacattagttggagaatttgagtag
caaaaaatagaccctatgttttctcaaaatgttagaagacataaaggctatggccagtatttcca
cgctc

>chr4_83042376_83042576
atagaaatacagatagaaagatggatggatggaaggaaagagaaaggaaagggcgaaggaaggaa
ggcaggcaggcaggcaggcgagaaggaggaaggaattttttgattaactaaaaaatttaaattctt
tcagaaatataaaatgaaattaaaaggcaaacagcagtttgagaaaaatatttccaacataaata
aggga

>chr4_85952176_85952376
ttctgttgtcctctgacgtatactgctaaggaggaatgacaccctgttgcaacttagaaagaaga
atttttataatttgcagtgtttcatttaatattgacttgaaatgctgtgggcaactgcatttta
atgggttaaattcagagcaaagatctgtcttcatctactggaaaaatttcattagaattaaatgt

gcctc

>chr4_91215777_91215977
ctatatccttccatattttgtagtttttaaataggctatatatctcatactcccttattttgatt
tttaaagtcttaaatataaaagtaattcatgaattcattctgattgttgaagcatcgtattacat
ataacaccatgtccccgttgacgacaacctcaattcaaaggccctctttaagggtaatcactgtt
aatgc

>chr4_99175977_99176177
tgtgaggatctgagccaaagaggcttagattttacttctgctttttttacttattagttatagat
cctaagttaaattatttaacctctgagcatttttcattatttgtaaagctgacataataatacct
catacatagcactattatattatttaatagtaatatatgagaatttctgacacataaataattcc
acaca

>chr4_127203150_127203350
aatcaaggcatagttattttgtcacagagaaatgagacatatgtgtttttcctttccattgaatt
tttttagactcagaaagtatacttatatcaacatgttgtgtatcataggtcttcataaggcaaaa
catttttacaaacatgttacaggtgatgattgtttggaatgcagattgctccaaactatgtccca
taaaa

>chr4_131201750_131201950
tattatgacctaatagacttttgaacagaccacacaacctgtatgtttatattaaaggctcacat
agaacttatttgtatatatgggtgctttatctatttttttaatttttaaaaatttttatagattta
gggactacaagtgaacttttgttgcatggacatattagaggaagtctgggctgtcagtgtagcca
ttacc

>chr4_132237750_132237950
aaattctttgcctggggcaatgtctagaagagtatttcctaggtttcattctaaatttttttaaga
tttgaaatcttgaatcttgcacttaagtttttaatgcatcttcagttaatctttgcatatggtga
taagtaggagtctattttcattcttctccatgtggttagcgagtttgcccacaccgtttattaaa
taggg

>chr4_143665150_143665350
gcagatgatgactttaaatcaactatttaaacatattcaaaaaaccaaagaaaatgtgttcatag
aattaaaggaaaatatagtcttaatgaaagaacagatgatgactctcagaaaagaaatgaaatct
attttaaaaggcaaatagaaactctataatggaaagaacaataaatgaaatgtaaaattcactag
atgag

>chr4_161433150_161433350
tcactgcaaccttcgcctcctgggttcaagggattcttctgcctcagccttctgagtagctggga
ctacaggcatgtgccaccatgcctggctaatgttttgtatttttagtagagatgggatttcagca
tgttagccaggatggtctccatctggtaacctcttgattggtccacctcggcctcccaaagttct
gggat

>chr4_170729425_170729625
taatgtcctcagatgatctcccacattcaaatgtggtttttctcggctggaaggcgtgggctcct
tattcaatctcattctaactctcagacaattttagaggctggtgtttcttttcctaaccagaaca
tgctttctgctttggggccacaaaaatctatttttatttcataccagtgactgaaaactttgtga
tacaa

>chr4_190213206_190213406
ctgcagaattttgcataaataaagagaagccagatgctaatagccaagacagtggggaaaatgtc
tccagggcatttcagagatcttcagagcagcccctcccatcacaggcctggaggcctaggaagga
aaaacagtttcatgtgccaggcctagggctctggtgttctgtgaagcctcaggacatggcaccct
gtgtt

>chr3_4134800_4135000
ctctgagcctttaaatttctttgcctataaaatggagatatttagaatggctagctcaggaattg
gttatggggattaagttaaataaaattaaacaaggtaatacctagcacatagcaagtattcaaaa
agctttagtcataattgatatcactatcaactaacatttgtagataacttgttatatgtcaatca
ctctt

>chr3_26417796_26417996

aaaagggattaactcagtaagaactgaattgagttctcagtaagactcaacaacatccctttcta
attcctactcagcccccacaattgtatcttggcaggtgagagaaagaaatcatattgtgttcttg
cttccttgcccattctaaatgtgtttatgacatggaaaatcccagcatgaaagggagactggaag
gggaa

>chr3_35858396_35858596
ttttcgtcacttagatgagtacaggcttagaattaaatttactttgatttacataactagtgtgt
gtgagtgtgtgtgtgtgagtgtgtgtgtgtgtgtgtgtgtatgtgtgtgtgtagatagggtttca
ctgtgttgcccaggctggtcttgaacttctggtctcagggatcctccagcctcagcctcccaaag
tactg

>chr3_36230596_36230796
attaccatttcaatctcactgcttgttattggtctgttcagggtatctaattcttcctaatttaa
gctaggagggttgtatctttccaggaatttacacatctcttctaggttttctagtttatgtatgt
aaatgtgttcatagtagccttgaatgatcttttgtatttctgtggtgtcagttgtaatgcctcct
gtttt

>chr3_39900796_39900996
aaccggccatgactgaattatgaaaaatgacaaaatctgaacagaccaataatgaataaggagat
tgatttagtaatcaaaaacctgccaacaaagaaaagccccagactagatgactttaaaggtgaat
tctgccaagcatttaaagaactgttgccagtccttctcaaactcttctgaaaaactgaagatgag
ggagc

>chr3_81275910_81276110
aactttttgaacatacagtgatctatgggagaaaaacaaaaacagagttctaagactcaagcat
tggtatacgtaaaagctccaactgtagaaaaatatttatatatgaagagcaactgcaaccttgag
taggctccccaggctgccggttttaaatcaaaatgaatcaagtgttgaggaagattcttgatttt
atatc

>chr3_94879510_94879710
tcgcattgaacaatatgccaagcggattatctcagttccagctaaaaccctgattacaacatgcc
aaggcatcaaaaatgcaagccctttgaagacatgtcttattttcagaggactcattatcatcgct
tcccatttatatgcagttttacttgttgtaaagtgaagtaagctgattatgagatttttgggggg
aaaga

>chr3_104776910_104777110
tgaatatcaatgtaacctttcaatatgcagtgtacatttgattagatgtgtgtaatttctacata
gtaaagtatattttattgtttattgaaacaggtagcctataacatgttacgaaagaaaatgaatc
cataaaatcttaataagtattagtatcttatattaatgtataacatggtttattttatatttcta
tttgt

>chr3_105762110_105762310
ttccttccccttaaaccagaacttatacccatcagctcttttggttctctgacttttggatttga
actggaactacactattggccttcctgggtctctagcttgccaattgcagatcttgggatggatt
tcttagcctctataatcatgtgagccaattccttttaataaattttttctctctcaatcttactg
gtttg

>chr3_109981110_109981310
cttgaaatttataatagagatattgtgtcctcaccacgccccccaacacacacacacacactcag
acggtaactatgggtggtgattgatgtgttgattaatttgactgcagtaatcagtatatgatgta
gagaaatataaaatcaccttatataccttaaatatacacaattttttatttttcaattaaatatct
taaaa

>chr3_134967910_134968110
tcccttcagactgaacgctatgtcaacctgtctgtctgggttaactgaactgtgacagtgcccag
aggcagagacactggccacctggatgtagaagtcatgctgatttaggattccatgaagaagaaag
gttcagcccagggctccactataatcctcattccatcagggatgctgccttccatgaagtcagct
gtcag

>chr3_135052110_135052310
taatggtccacctgggatccacctttctgtatcaaggtagggaaagtgtccccagacagaaagta
aggtgaacctgagaccaactcctgttgagtttccttgtctcaagaattatagttctgctctgctt
attttccaatagctctaaacaattacatgtatttattcacttttatagatgatgatattaatgat

ttttt

>chr3_154543306_154543506
ttaagtctgcagaagtttctgctgccttttgttcagctatgctctgttcccagaggtggagtcta
cagaggcaggcaggcctccttgagctgcggtgggcttcacccagttcgagcttcccagctgcttt
atttacctactcaagcttcagcaatggcagacgccccttccccagcctcactgctgccttgcagt
ttgat

>chr3_156595906_156596106
tttcatttatttctgttctgatctttgttatttcttttttctgctgggtttgggtttggtttgt
tcctgtttctctagctccttgaggtgtgaccttagattgtttatttatcttctttcagactttt
gatataagcatttagtgctatgaactttcctgttaacactgtttttgctgtatcccagaggtttt
ggtaa

>chr3_166205506_166205706
agtgcaaacaaaacttcttctgcccttttcattttacccttcctatatttctgctccaaagtggt
aatacggccacatacttcatatgttctccacttgtgacatattttggaaacatttcttagatagc
taagttataattttaagttactttttcatttattaagggatgagataccttcagaattctttaat
attta

>chr3_180353306_180353506
atgttcctgttccaaattggagaagttggccaaaacagaggggccacaggctctatgcaagtcca
aaacctggcagggcactccttaaagctccagaataatctccttttactccatgtctcacatccag
ggcacactgatacatggggtgggctcccaaggctttgggtagctctgcccctgtggctttttagg
gtata

>chr3_181518706_181518906
cactttggaaggctgaggtgggtggatcacctgaggtcaagggttcgagaccaggctggccaaca
tggtgaaaccccagtaatattaactacttgagaggctgaggcaggagaatcacttaatcctggga
gggggaggtcgcaatgagccgagattgtgccactgcactccagcctgggtgacagagcaagactc
tgttt

>chr2_4447325_4447525
gcaggtgcctatagtcccagctactcgggaagttgaggcaagagaattgcttcaacctgagaggt
ggaggttgctgttagccaagatgacaccactgtgctccagcctgggtgacagtgtgagactgtct
aaaaaaaaaaaaagaaaagaaagcgtttagccaatcatttgttaattatgcattggggatttggc
tagtg

>chr2_16003949_16004149
ttaaacataaactttccttatcactcatcaattccactcctaggtaattacctaattacccagaa
agaataaaaacacatgtccccagaaaatatttgtgtgcaatattcatagtagctttattcataat
aattaaaatctgtgggaaaagcccataatgtttattaatactaagtgaataaacaaattgtgtta
tgttc

>chr2_16984719_16984919
gaacatggatgccttttttatttatgtcctgtttaaataattttcagcaacattttatagttttca
acatacaagtttttttgccttttttggttaaatttattcctaagtattttagcctttttgatgctac
tgtaaacgggcttattctcttaacttactttcagtttatttgctgttggtgtatagaaacacaac
ttatt

>chr2_22300295_22300495
tgtaagccactatgccaagctgctttattatgtctttgaagcacaagattttctcattttgatat
agttcaatttatctatttttccttatcacacttgggtgcatctaatgttgtattttaaaaaatg
cctaaaccaggatcttgaaaattacatttccgttcttcaaacagttgtattattttatctcttac
attaa

>chr2_41274096_41274296
tgagtaacaatacagacacccaaataatgtagaacttcggcaaatgctgaaaagtagacagcatt
gattttactttcagatcatcttgaattggagtcttcaaatgcaattttgaaattacagaattgtt
ctctaacaaacaactttttaattaaagtctacagaaacgtagtttatttagtttatttcttagag
agtaa

>chr2_59210896_59211096

ttggaaaataaaatattacatgtttttgttttgttttgttttgttttttttactagcactatca
caacttgcagtctcaaaatgttacaatgactactgggacccattattttttccaaacatctaggc
ttaaaaaagagtgaaagtttgagctttggaaggcaatgaatcgtgaatgagcttttgcataacct
tcaaa

>chr2_68779496_68779696
tttgaatagtttctccacttggtttaaatcttatccttaaccaataccaagaattgacaaatatc
ttcagggaagaaagcagcctgtgattgtcagcttacaaaggaaggactgtcattattaaatctct
ggaattttagataatctaaaattttcattgctctcacaattctgtcataaaagtgatttttacag
cctat

>chr2_77775292_77775492
cttttctccatgtgaagttgttggttatttagataggtccttaatgcttttcttgaatattttga
tacattaatcctttattctttatttgtggagaggttatcttcaaatttttctgcttatctatttt
agtttactgtactatcattgatattttgtgctttttttgtgttattttgtattttaatttgctttt
cgggg

>chr2_134672130_134672330
tttgcagacgacatgattgtttatctagaaaaccccatcgtctcagcccaaaatctccttaagct
gataagcaacttcagcaaagtctcatgatacaaaatcaatgtacaaaaatcacaagcattcttat
acaccaacaacagacaaacagagagccaaatcatgagtgaattcccattcacaattgcttcaaag
ataat

>chr2_139102530_139102730
tcttgcctactcctttgtactaattcctttaaagtcctctttctatgtctagtgtcacagggcac
agaatgaggagttagacagtcctcactcatgaacttccaccacgcctttcccaacatacccatgt
gcattcatttcataggtggatgctgccaggttagcaacttgccttcttctgtgtaactatctgtg
aaaac

>chr2_139489730_139489930
acattgctttgcaagtttggtgatctcatacacaatgtgtcaaaaggagggctctgtgattaagg
atgtaatcatcttgggcaatcaaagatgtttttttgaatccagtagagaaaactggcctatggaaa
gtcatagagaagaggagaaagtcgagaagctctcctggtaactggtcacacatccagcactggaa
gagta

>chr2_154696554_154696754
tttcttattgccaattaatgtgggtggcacattgtagttattgcttgtttattaacccacatttt
tctatgtaatgttgctctaatgcattgaagaactttgtttacaaatagaaaaataaacaaactaa
cacatagttcagtgtaaaattctttggcaatagcatgtaattgaacaggttttgaaacactgctt
ttccc

>chr2_168027154_168027354
agtgacgttcaatatttcagtcctactactgataatttctattttctcctctattttaagcaatc
ttgctagacgttagcaattttattaattaataaagcaactagttttgctttatctttatggtaca
tttctattatatggatttctaatcttatttttcctatttccattcttcaacttgctttcacttta
tttta

>chr2_200383555_200383755
gtagactctctctgaggacactttctcttgctactcataatccaggacttgcattgtttagcttc
tctcctagcttccttgcttttcttgctctcatctcatgtcctttgattcggtgctttcccatcat
tcctttactccttgctttgtctccttttgcactttttttcttatttctttggtttccctcttgac
tttct

>chr2_212786955_212787155
agaactgaaaagtgactaccagttacattataaatgtaaggaagttttaagaatagcttaaacag
tctattttgctcattattttgtattttcatgtatgaacaagagtgacgtgaaaaattcctgtcta
aatgattctaatatcaaaagtctctctgaatatgtaggaaaaaagtcctaagtaacaagaagaat
tacat

>chr2_215325955_215326155
tggggcctctcagcagcttgctcaggggccagaagtagcagcaatggcctggacagttgagtagg
tcctcatacccctgggcagtttgtgtggcataggcaatggcagtagcagtgataaaacaaccctc
aagttccaagggggaacactctagtgttaatggtggcggtgatgagctgggtaggccagtcctca

ggccc

>chr2_221126956_221127156
aaaattcatcttaattcaaccaactgatttcatgtaggcatcagcaggaatcaaactgagcatga
tatggtctttagatcatctagcagctgaccagaacaattgatttttttgtaactgcagcttttca
tttggcatcataaagatgcaacctaaataaagcattagcctctagaatgtgtttaaattgagttt
ctgga

>chr2_229450356_229450556
catcatgaagataggagaaagtttacttttccattgagtgagggcaaacagatggcttgtgattc
cccaacctcaactcttcaaaactcttggccgggcgtggtggctcacgccttgtaatcccagcact
ttgggaggccgaggcaggcgcatcatctgaggtcaggagatggagaccagcctggccaacatggt
gaaac

>chr2_229729156_229729356
ttaattctacactaaaaagttcagcatagagctgatcatactgtgtagacgtgatccttaattta
aaatgtaggcttggatagcctttgtggaaaaaataagagcaacaaggataaaacaaattacctat
gacccaagaaaatatttcctggcaaaaaaaaatttttaaaaagggagggattgtaaaatttccaa
aagat

>chr1_37586813_37587013
ggtgccactgccctccagcctggaggcagactgagaccccatccaaaaataaaataaaataaaaa
gcagtgacagtgcttggcacacagtaagcacccagtaaatgatgattattgtcctttttctgaca
actatgaacaaaataaaacacacacagccccttcccaacctctccttctgaaggttaaatgatgc
attga

>chr1_49737613_49737813
catgctgtgacagaaaacttggcctttgtgtgtacaatggatgtgaggtcactggtgagaactac
agttttggaagtggctggcagtggtacaatgaatgttatttgttacattgtcacactaatgttat
tactacaggaggccactcatttctttgtgcacttagttttaaaaacatgcagtttaaatacttag
aaaac

>chr1_57923012_57923212
caagagccattcacatattacatttgttagcaaaaactaggtctgaatagatctcttctgttaaa
ggatgattacataggcaaacagaatcaatgtggggagtgaaaaagtgctatagaacaaggaaaca
ggaatgatgtattcagaagacttctgaaaattatctgctgctagatctctattagaagtaatttt
ggaaa

>chr1_58204812_58205012
acacacaatgactttaaaggataaatattaatagatagtcatccacctctggtcctaaatctctg
tgggctctccatttctactgaataaagttcagaattccaggcagacaagttcagaacaatacct
gtgtgaacctgacttcacacctttgctcatctgaagtgctctcacatttctttctgttactatct
ttctc

>chr1_58846812_58847012
tctcaggggaaaagtggggagagagagcttcaagaagaatagctaatgaatgttgggcttaata
cccaggtgatggaatgatctgtgcagcaaaccaccatggcacacatttacctaagtaagaaatct
gcgcatgctacacatgtatccccgaacttaaaataaaagttgaaaaaaaaaagatgggagaagta
ggcca

>chr1_61217812_61218012
atgggcaaaaactgagagccacctatatgcatataataatgacacacataatatcaatcactttg
tgccaagcaccattctcaacatttttcaattataccactgatcccctaattatacttatttttca
gatgagaaaactttagcagtgagagtagctatcatgcccaaggtcacataggcagaaagtgtcag
aggct

>chr1_63502612_63502812
tatttttttaattttgttattattatactttaagttttagggtacatgtgcacaacgtgcaggt
ttgttacatatttatacatgtgccatgttagtgtgctgcacccattaactcgtcatttagcatta
ggtatatctcctaatgctatccctccctgctccccccacaccacaacaggccccagtgtgtggtg
ttccc

>chr1_73680612_73680812

tgtacaaggtaagtgccatctgcatcctaaattaaacatcttcatgcagagaatcagtctgccta
atgcactgatatataatacaatgacagcactcacttttaaatatattgccgaaaaggcaaaacag
cgcattattcttatagcattttgcctaccatgtatttttccacaatataaattgccagcttttca
aggca

>chr1_96640012_96640212
aaattaagaaatacaaatggctaataaacatattaaaaatgttcaatctcagtggtaataaagat
gctagtcaagataacattaagataatacatcacatttctttttttctttttttagttactatgtcc
agtgttggacaggattcaggaaaatagtcattcatgctcagctccaaggtgatttagcaatgtgt
gtcaa

>chr1_96697612_96697812
gccttcttcaagccttcagattacggcatcctcagccaacaccttgactgcagcctgatgagaga
ctctaagctagaaccacccagctaagcttcttccagattcttgatactcagaaactataagatat
aaatatttgtcatttttagccccaacactgatacaatcaagcatacactactatggaaaaagaat
tattt

>chr1_102883612_102883812
gcaatcaatacaagaacatgggtagatgataccccatttttgtaaattaattagctaaggatcaga
aaagataagtagctttattgtgttccaacattaaaaagcaacagacctgtaacatgatatttttt
gtttccggtttgaatggtctttcaattttactctgttccttcccctccaactcctttgatacagg
gatga

>chr1_103111012_103111212
aaaagaacccaaactcctagagaagatgaagtggagggggaggacccaggatgccaaccagacaga
agaaaagaacccaaactcctagagaagatgaagtggagggggaggaccctggagaagaggactgtt
gccctctagagaaaagagcaacatggtgtcaccacaattgggactgggaaaatttaccctctttt
tgcct

>chr1_107679077_107679277
actttacacggtgccatatactagaaagtacatgggtgctagaaaacatgttccctgcattggtc
actaactggcaatatcttataccatttgctaatttttgtgctccagtatcattaactcagtaaat
atgtatattttattacattaaacaatacgtcctttggtctgctgttagccttgagaggaactaaa
tgaga

>chr1_111476277_111476477
aggggaatcacccatcccagaggtcgaaacttgagttaatgcaaaccttgccatggagagctaca
gtattctgagttgcttagtaaacttgaaaggcagtccaggctataaggactacaactcacaggtg
agtccatgtgctgaactgggccaagaaacagtagacttgggggacatgtgacctatggagacacc
agctg

>chr1_117286877_117287077
gggaaagcaaaggagttcaagaaacttccagtggttggggggggaggggggttgctgagcctccaga
gaaagagtgcaagaaatctctagtaagagagagaatgagccacacacagtcaggaaactcagggaaa
tgcctacaacttccaggatgggaaataccctaagcaggacagggaatataaaggacaagacagac
aataa

>chr1_118989877_118990077
tgaaaatatgacaccaaaaattgtggattacagttaaagtagtacttagagtaaaatttacagct
ttaaacacatatattaaaaaataagaaaggttgaaagtcaataatttaaacttctaacttaggaa
gccaggaaaagaagagaaaatcaacatttaaaaagtagaaagaagaaaataaaaataagagttga
actaa

>chr1_163725976_163726176
cactgatatacatatatatacatatatacatatatatagcactgagaatagtgtctgagatataa
taagctatattattcctgtagagtccctttaaagatgaaaatttccatttggcaacttacctccc
aatcatcttttctccaggttaaatatcctcagatctttaacaataaaatgttgtttctacacctc
ctgcc

>chr1_187401377_187401577
gaagtcaaaaggttgttctttacattaatctacctttttttaaaaaattgtcttattttattgtca
ttactttctatccaaccaggaaaatgatgctatatttttatttttgcttttttcatttcaaggtg
ctattgctataaaaataataatatcactagtatcttcaataataataatcgtagttcacacttac

atggc

>chr1_187797177_187797377
aaggccaaggcagggggagggcttgagcccaggagtttgagaccaatctgggcaacataggaaga
tcctgtctctacaaaaatataaagacattagccaggtgtggtggcatatagccccctctgttgtc
ccagctactcggagtgctgggtgggagcatcacttgaacggaggatgtctaggagccatgattgt
gccac

>chr1_215928177_215928377
cacttggtgagtgtgatccatgtttttggtaaatcacatctattccttttagatttttggatggg
taggtaaagctttctagttactagtttcttcatgctgcagagatcattgctgtcacgtggctgtt
ttcccttcagcggcttgccattttccacaattaactcttctagtcttggcacgccactacacacc
cagtc

>chr1_216973177_216973377
aatttatctggaatgatagatgcacatgtctcattagtcagtaatttgggattatcacttatgta
gccttagaaccatgtaatgttcttgaggaaggggagtctccccctcaaaaaaaccccacatttat
ttgcaaataaaaatatccttacttattgctgtgagggggagaggaatctcagcactcaaataaaa
ttatg

>chr1_240207777_240207977
aaaattattaacagcataattggaaaaagtgcacaaaatctgctctgctagctgacgtatatttt
taaaaagctcttggactcaaattgaagaaggtaaaaatggtgaacaatctcaggactgcgcctga
tgcagggcattgagaaaagcttctttcttcttctcgaaataaaattctgatgagtcattttcttc
taaaa

>chr9_1394600_1394800
ttctaaccaaatgcaagggagctaagaaccttgataaaaggttacaggaactgctaactagaata
tccagtttagagaagaacataaatgacctgatggagctgaaaaacacagcatgataacttcgtga
aacatacacaagtaccaatagccaaattgatcaaatggaagaaaggatatcagagattgaagatc
gactt

>chr9_9627200_9627400
ggaggctgaagcaggagaatcgcttgaatctgagaagcagaggttgcactgagccgagattgggc
cacttcactacaacatgggcgacagagtgagactctgtctcaaaaaaattctattttgcagaagc
aataacaaagtccactgtagatgcttagtcaattctcctgcttaatttgtgtgtattcgtttgtc
atcta

>chr9_15784600_15784800
aggcacaacaactacaggaattgaattataaacttgaattgcactccagtgaggaagctgacaaa
aaccaaactcttggagaagctgttaaggtaagagaataaagggatatttgcataaagggatattt
tatctatgtgtggatcttagagggaattatgatatctcctgaataggaatagatcccaagatgta
aaatt

>chr9_17823400_17823600
ttgtagtatttatatatttcttaatcacttcccatatataaaactgtgctatttttaataggtat
ctatctttttttttgtacacgtcactgacaaagtttttgaatatatgacctctaacctaattttc
ctcatgggtcttacggttcataatgtgcagttttccatagcacagtggttttcaggaatgcatag
gtggc

>chr9_30501800_30502000
tccctctgaaatcaatgaagacagtagacaaaagaggtaagtataattttacactgtatagtttt
caaagttcttttcaattaaccatgaattttagatttctagactactttacaaataagaatttgaa
tgcttagactaagatacttagttgaattccttccagtctcccagatagcaatttctatataatat
ggact

>chr9_66399380_66399580
ttcttttgagaagtgtcttcattctcagtaaactatcgcaagaacaaaaaaccaaacaccacata
ttctcactcataggtgggaattgaacaatgagatcacatggacacaggaaggggaatatcacact
ctggggactgttgtggggtgggggagggggagggatagcattgggagatatacctaatgctag
atgac

>chr9_77076580_77076780

aaaaatataggacaaaaattgaaggtatataagaaaattatagaaagtttatatgacaaatcttg
ggaaaggaactttatgtgtgatcaagctggctaagatttgaaggaaattgtttacatgtttttga
aattgagcattactatcaaaagcaaactgacataaaactaaaatttggttctctctgttaaaaca
ataag

>chr9_85697180_85697380
aatgaagataatttcctaattagagaatgaacctgacctatctatcactcatggaagcccactac
cattggtatcccagggagaatgatggttctagctgaataacaacgcttgattagccctacatcac
ccatgggagctccttttatctcagcagatgaaatcagtagtacaaatctaactggttttatcgtt
tgcac

>chr9_99465979_99466179
tatttaaagtgtggtgaggaaaaaacctaccaaccgagaattctgtatcctgtgaaattatgctt
caaaattgaaggaaaaatgaacaaacaaaaattgagggaatttgttgccagtagacaagaggaaa
ccatgtcagataccaatctggattcactaaaagaatccagaaatcctaaatatgtagatatatgg
aaaat

>chr9_106931579_106931779
ggaaatattgttatatttgatataagaaatggaaacaagagctttaaattttatttaaaacctca
gtatgttaaaacaaactaaaaattgaacttgatcttctgttagtgcaacttataaatattaagga
ggtaggtactttttcactgttcctttttgcctagtctttcttctttagtttcttactttttta
tccat

>chr9_107341579_107341779
agagcggtcctctatgaactgccatcaccaagtcatttagaaaatgtaattagacaggctaaaga
agacttattccaatagagacatagagtcacaaactagtgagaaagggaatatgataacacatgta
ttttgtttggtcacatggaattaatgagatttgccacagtcctcacccttctatattgcttcaca
tccag

>chr9_108611779_108611979
tgagccatacagcatctgtgatagagaagactcactcaggagcaagagtttggaatgaaagataa
gatagtatgaacacacttctccactgcaggaaaccttaaaaatattgctaatggccttttggtat
gggaagcagcagagctgagccatcagcaccctccctgtgctctccttgcccactctgtggctctc
cgccc

>chr8_3327992_3328192
ggacattgacatggatacaacctaccagcctcgttaaaactttccctgttttattcgtattttgt
atgtacatttgtgcatgtagctctgcacaaatacgtcacatgtctcggctcctgaatccaccgcc
atagtcaatgcagggcagctccattccttccaaattctctggtgctcttcccttaaaatcacact
catgc

>chr8_4887192_4887392
tggtgggaggtaattggatcacaggagtgaatccttcgtgagtgatttaccatgattacattggt
gctattcttgtgatcgtgttgtcaagatacgtggttgcttaaaagtgtgcagcaactcccccctg
gctctctcttcctcctgctctggctatgtaagatgcccctgcttccccttttgccttccaccgtga
ttgga

>chr8_5809592_5809792
tcattggtaagttctaagagttcagtggctcagggcatacctgaatatcatttctaagataccag
ttgttccacctcccaaatcttatcctaaaaacagaaatggcatgtggtagacctctttggagttt
tattgtttttaatatactttacttttagatcagttttagatttggagaaaaattacacaaaatg
taaag

>chr8_12531229_12531429
tccccaacaagcaccttcccctgctccacggtgcccagtcccatcgaccacccaagggctgagga
gtgtgggcgcacagagagggactggcaggcagctccacctgtggctcctgtgtgggatccactgg
gtgaagccagctgtgctcctgagtctgatggagacttgtagaacctttatgtctagctaagggat
tgtaa

>chr8_14032229_14032429
gccttgccagggctgctgtaataaagaccacagtctgggtggcttaaacaatacaaacgtattgt
ctcacaattctggaggctagaagtctaaaacaaaggtgtcagcataaatgcttccatcttaaagg
ctctgagaaaaggatttgttctggacttcttttcttccaatatctcttgacatcatcttcccttt

tgatg

>chr8_19787320_19787520
gatgattatattatttcttcctctctcttaaagtagttttgtttttccatagtttttggaaaggg
gcagggcatgaacatccccaactcaggacaactaaaatctttaaaatgtgtccaggtcattctac
acttatggtttgaagcagtgctccccacatcccaccatatttccaggtccaagggctgccaatct
caact

>chr8_31742058_31742258
gtgaccactccctttttgccttcactttctctcctgacttgcatttgatcagctaaatttatgta
aaaaaatgaagcctacaaccctaagctgatctgaaacacacagcaaatggaggtagccacatata
tatttgataatttcccagtttatagcataacagttaaatcattgtaatttcttatggaatgtatg
gatca

>chr8_34497858_34498058
ccccaagatccagagtcagcatcagccagtttgtgtcacctacgctttctgtctttcctgccctg
actgccactgggttttccctgctgtttgtgagtctcctcacaggaggtctacaatggcaaagaca
ttgaaaatattcattgagaaaggccactctctgggctgctgcctcccacacacctcctccctcag
acttc

>chr8_34505258_34505458
tgtgggtgaccatcttcttctcgtattcccatatggtggagagcaaataaagagtaagcaagttt
cttttgtctcttcatacaaaggcacaaatctcatttattcataagggttttaccttcacagtcta
attacctacagaagaccccacctccaaacaccatcacgttggaaattagccttcaacatatatat
ttttg

>chr8_53943847_53944047
cgtgttacttctgatataaattacttaacaagagttcagaatgttgccatagaaaaggaatctct
agcatactagtgacaaatgaagttattatagacctatactcaaattacataatacccaaaacaag
tgagggacacattagactagaaagttgttggcaatatattgaaattattgaaatttctcatcttg
gtatc

>chr8_63591046_63591246
cataataaccaactatcgaatagcttcttagatcattacaatattaataaagttcattaattaaa
atggcccaagtcaccaagatgaatattttataggccagtcatttcaaacatattagtaccatcat
tattatatattgatgttatattgataatataacatcattatattatatcattacattgataatat
atcat

>chr8_63859046_63859246
tacctctgaaataactagtctctcctaaaagccttcattatggtggggtgctgcgaactcctctc
tgcagtctttgaagggcctctctgtgtgaactaagttgatgtcaagcctcttccccagctttatc
cttatgaatttatatcacggaacaagaaaatcacagtgtaatagcccccatatttgagttcttca
aatgt

>chr8_65217246_65217446
tacttcaaaaatatatcttaaatctattcattgttcttcatcgctagtcttagtcccatccaccg
tttcttgggtggataactgaaataggctcctaatgtatttcctggtttctcctcctgcctttata
atttaatcttcatacagcagacagagctatcttctttatagtctaataagacacattacccttct
gaaaa

>chr8_65411246_65411446
tgaacttcttacatgagtaggaaaaaaaatctttcttgaataagctactaccattctcagtctct
gtcacagaaaccaagcgcatgttaagatatcatctctctttttttgcaaaagtgctaacaccatgt
cattaagtttgcattgactaacatgccagagttaggagtatgatagggtataaagaattccacaa
ccttc

>chr8_69600846_69601046
aagaaaacgtggtacatatacaccatggaatactatgcagccataaaaaggaatgagatcatgtc
ctttgcagggacatagatgaagctggaagccgtcatactcagcaaactaacacaggagcagaaaa
ccaaacactgcatgttctcactcataagtgggagctgaacaacgagaacgtggacatagaaaggg
gaaca

>chr8_78351445_78351645

ttacattatatcatgtatcactatttcattctgtattattgtggattagtgtggaattaaaatac
cagtttctttatccattcatctgttaatggccccttaggtgtagtttcaggctgttgtaataaat
atgctctgaacatttgagcacaagtctttgcatagttgatagtttcattatatcttgggtaaata
actag

>chr8_92910024_92910224
ttttatagaaaatgtttgccaaaccctggttggaaaggaagaaaactgtctctaaaaattatatt
atcaatatttccaatcatttaaaactgtgctgtcattaaaggaattcataaaataaataaagcat
aaatatatttgtcttcgttcagctacaaagattgaaatattatacacaaaattttaacaatatgg
ctatt

>chr8_97424024_97424224
ttcttagcagctatgcctgagctccaaggagtaaacacagaaaaagcacacatcagagaaggaca
acagctagagagagagagaaaaatgacaaacaaagcaaccaggacagaagaagtactcatcaaaaat
tagagctattggaggagataagtctatgacccatacaaaaataaaaataatgagaaactgaggag
attta

>chr8_132040618_132040818
ggagactttgtagtcactgcacattgaccacttggggccatgaactcattctcaaatgaactacc
cgcctccaataggctgggaaggctcacccgatgaatgagggtgtgggagatttgactttagaaga
aaaccagcttgctcttagtgattttttttcttgaactaattagcagaaaaaaaaaaagaactaaa
aagac

>chr8_132121818_132122018
cctaatttcaagattgttgtgtctcagggaatagggaggcctgaggagaggaagagagatgtgtg
tgtgcagggagggagccaatcaatggagcagtcagaacacacacaacatttattgattaagttca
ccgtcttacatggatgcagttagcggcatcccaaaacaattacaatagtaacatcaaaggtcatt
gattg

>chr8_139065818_139066018
cttccatttgtttgtatcctattttatttccttgagcagtggtttgtagttctccttgaagaggt
ccttcacgtctcttgtaagttggattcctaggtattttattctctttgaagcaatgatttggctc
tctgtttgtctgttgttggcgtataagaatgcttgtgatttttgtacattgattttgtatcctga
gattc

>chr8_143243893_143244093
gaaagagagatgaagatgggggacagatgatggatggacgaatggaagatgggtgagtggatgga
ttgactgaattcaaggatggatggaggatgggtggatgaaagggagatggagatggaggatggat
gggcagaggatggatgggtggatgaattgatgaatggatgaagaatgaatggatgaaagagagat
ggaga

>chrx_7681200_7681400
aaccaggcccttcacaatgtgaccacattacagcttctctcaacagaaggcaggggatagttcac
ccctaaattttgagttcacctcattacttggtttagccaatgggtgtcaacagtggcagagattt
tgaaagtgcttgtgctttctcttgtgctcctgctgttggcccccatctttagatcatgcctgagc
tagct

>chrx_14744279_14744479
agtttcagtttatccatgactcaagctcatttcatataggaatcttgtcatattaccataacatt
ttattctgaacctgctagaaaggcaattagtcatttattataacacaaaaaaattcaaattggtt
gcagttatgttttcagaatcaaaaccatctcctttgatgtgaataccataaagttctatatttaa
aaggc

>chrx_19157479_19157679
cctgcctcagcctcctgaggagctgggattacaggtgcgtaccaccatgcctggctaatttttg
catttttagtagagacggggtttcaccatgttgcccaggctggtctttaactcttgacctcaggt
gatctgcccacctcagcctcccaaagtgctaggattacaggtgtgagccaccacgcccggccagc
ccttt

>chrx_27022079_27022279
aagatcactggtggaaatgtaaaatggtgcagccactttggagaagagtttgaccgtttctcaaa
aagtaaaagatgcaactaccacatgacccagcaactctgcttggacttgcatgtccaagttcata
gcagaattacttataatagtcccaaactggaaacaactgaggtgtttaatttttaaatggatcga

ccaaa

>chrx_27939879_27940079
tagaaaaacaaggggttgattaatcgaattttaggaggatattattctataagcaatttaagtaa
aatttctttacaaaatggtacttaggctacagaattaactgacaaaatgtcctataaaagaggaa
aataactagataaatatgtgtcaattacagcacaggcttaaaagggaaaattattgataggcaat
gtgca

>chrx_30290679_30290879
cagtcctatttttttctcaagtcctcaacttgggttccttatatctcagtgcccaggtaccttgcc
tactatagttatagggtctagtaccacctgtgcttctcctaaggacgattaagaacatgaacacg
agaggtgggtcagccatggttcaatcgaagaaacaatcactgataatgaggtagagtaaagaatt
tatta

>chrx_31787679_31787879
cacacataaaatacactaacactaatgatagctgatgagcaaaaaaaaagtcacaaaaagtatctc
atgttttaagaaagtttacaaatttgtgttgggccacattcaaagccatcctgggacgcatgtgg
ctcacaggccatgagttgaacaagcttgctttagatcataataattacctcctacaaatctcctt
tattt

>chrx_38527456_38527656
tggactaaatcaccaaagtttctcccggcccaaatactatgactggagaggggtgggagaggtga
tatttttatgttctaattctcaatttgcttgtggggcagaagtatcccatagctgagtataatgt
cataaaataggatatgtgtataaacaatggaatagttagttctggcctcttctggaggtggcttc
acacc

>chrx_46259856_46260056
agcaacatgacaaaactccatctctaccaaaaaaaaaaaaaaaaattagatggccttggtggcat
gtgcctgtagtcccagctactcaggaggctgaggtgggaggatcactaggcctgggaggtggaga
ttgcagtaaaccaagatcgcaccactgcactccagcctgggagacagatcaagacactgtctcaa
aaaaa

>chrx_53495275_53495475
ctacttctgtctagttacaaaacattttcatcaccccaaagaaaagccctatactcactaagcag
ttgctccccatttcttgcaccatgtacccgagaccggccccactcagcccctggcaatcattcat
gtgtgttcagtctttatggatttacctattcagggatcatacgatatgtgactttttttttttt
cagac

>chrx_63469875_63470075
tccaagttctctgattctttcttctgtctattagagtctgatgttgaaccactctagcaagtatt
tcaatgcagtttttgtatttttttagcccacaatttaaaaaaataatttctacctttcagtgattt
ttaaaattttgctcacatactgttttctttttttttaaaaaatttattattattatactttaagt
tttag

>chrx_66292275_66292475
tacaaggacaactacaaatcactattcaaagaaatcagagatgacacaaacaaatggaaagacat
tgcatgtcaatggataggaagaatcaatattaaaattaccttacttgtcaaatcaatatatagat
ttggtgctattcccaattaaactaccattgaaattcatcacagaactagaaaaaagatttaaaaa
tgtat

>chrx_74036875_74037075
cgtttgcactattagaatatggaagttcactggtgtgaaataaaaggacttgagagattcagatt
ttttttttaagtaaggcttctaacctagacaaaagccctatgtgaaatgaataattagtattgga
aatagcaagctaaacagaaattcaattcttgaatttgaatttgctattttatgccattggtcttt
agcta

>chrx_78957744_78957944
ttcactactatgtatttatcaaaaaaatgaaatcagcatatcaaagagatatctacactcccatg
tttattgcagcagaattgccaatagccaagatatggaatcaaataagttcccaccaacagaataa
tgggtaaagaaaatgtggtacgtatgtatacaatggaatactatttggcaataaaaacaaatgcg
atcat

>chrx_79693944_79694144

catttcatcatttcttctgtgtgtatgtatgcgtctgtgtgtgtatgtatgtatgtatgtatgta
tgtatataattttcaggactgtcggccatggggcatcattatattatttgtatataatatctatt
aaagaactttatttttgcttttattatcttgcagtttttctggttggtgtattactttattcagt
gataa

>chrx_81681744_81681944
aattcactaaggatggtcttcagctgcatccatgttgctgcaaaggacatgattttattctattt
tatggctggatagtattccatggtgtatatgtgccacatttttttatccagtccaccatcaatgg
gcatttaggttcattccgtgtctttgctattgtgaatagtgctgcaataaacatacacatgcatg
tatct

>chrx_89378544_89378744
catactgtacaagtccatttatatgaatagacaaaactacattatgttaatagaaatcaacagtc
attacctatgggtttcactgtctccctaggtgaggaaactttcaggcgtgttgtaaatgttctat
gtctaattttggttggtagttagatgtgtgtatgtaattgtcaaagcacatcaaacagcactcaa
gattt

>chrx_92245944_92246144
aaattacttagtctcaggtagttctttatagcaatgtgagaatggaccaatacagtcccaatttc
gcaggctttacaacagagcctacattccagaattcaaaaaagttaattggagataaagatctgaa
ctttatttaaaagtctatacaattattaggacaatatttaggataaatctttacaactttgtgag
atata

>chrx_95218344_95218544
ccctgaaatatctgattatttccctttctccaacgcacagttactctggtaaaaggccggaggtc
tcctggggaaggatgggagaaagattcactgcataaattggcagtaatgttgtggcatccttcct
caaggggacccagcctctccttcactcaagaggttctggactgtaaactggctcaagcctggaaa
ttgat

>chrx_97241944_97242144
agttatcctccacaaacctacatctactgcattccaagtattgattcaaaaatgtaacacaaaca
atgaaatataaaaagatcacctatttttaaaaagtaaagtagaagatataaaataaatttaaaa
taaaggaattaaaaataaaaatgaaaagaaataataaaatatgggaataaaaatataaagataat
aaaac

>chrx_101016544_101016744
tgtgaaacaacagacataaattgaaaccacatcaagccatatgctcatcttcttatatattatga
acattaactcaatgtctgtaatacacgtgcatcatttttcccattttatatttctttttatcag
cttttgcaatgcaaatgtttcaaattttcattttattattaaaagacaatgtggttaattataaa
tgttt

>chrx_104694544_104694744
gctagcctcagcctctcagttgtcctcattgagcatgtaataaatagttccagtgaagaactctg
ggaggaaggagtacaactatttgtattgaagtcttcagtatgtatttctggggatttttcaagag
ttgatgtttgcaagaaaatcaactttgagtaatatagaataataatctctttcattttagtaaat
ctcta

>chrx_109321544_109321744
tggctctttcttaaaatgtctatgttgtcttttaactcttggatcattttactggctttcttcga
ttgagtttcaactttctctggagtctcattgagcttcttgccatccagattctgaattctgtgta
tgtcctttcagacatttcaatctggttaggatccattgctgtggagctagtgtgatccctcggat
catca

>chrx_109846144_109846344
aaggacatgaactcatcatttttatggctgcatagtattccatggtgcatatgtgccaccttttt
cttaatccattctatcattgttggacatttgggttggttcccagtctttgctactgtgaatagtg
ctgcaataaacatacgtgtacatgtgtctttatagcagcatgttttataatcctttgggtatata
cccag

>chrx_119772372_119772572
actccctattcaataaatggtgctgggataactggctagccgtatgcagaaaattgaaactggat
attcttgtttataatagcaaaagtgggaaatagccattaataagggaatgtttgagttcattatg
gtacatccatataatggaatattaagcataaaatagagagcttatttcactaacctatactaaac

acttc

>chrx_126125119_126125319
caggagctgcctctaagcccataaaacagaaagtgctaccagaaagaaatcgacctcttgtactc
cagcgttgtgtttgggtgccccaaaatgatgaccctttctcagcaggaagtagccacaaagatta
tgatgccccatctccctacaatcttgtgataaatacatatacaaatattatagaaatcatgcaca
gattt

>chrx_126422919_126423119
tgaagtcaattacataattgacaataaagaaataattacagtctggtttgcaaagaccaatcata
ttggctacttgatcaggattttcattgacgataaacaggcagttgacgaaatgctttcttgatgt
gtgcctatctatttcccttgccaattactgtgaccatacctaccttgactttggcaattaagtgt
tgcca

>chrx_127231919_127232119
ctctggccataactttgcaggatgagatgcaacagcaaaactagcacaggcgtcaagattagaa
gtaaattccaatacaccttacatatctcatctttctttcttacatagcctctattttgactatga
aggtttagattttgttttgaacatcttccccatgaataatatttagagagtctttgtcttaccga
aaaaa

>chrx_128455919_128456119
tttttttatttattattattatactttaagttttagggtacatgtgcacaatgtgcaggttagt
tacatatgtatacatgtgccatgctggtgtgctgcacccattaactctaagtatataagacctta
ttatctatagtcaccatgctgtacattaggtctccagaacttattcgtcttataactgtgagttt
gtact

>chrx_136265734_136265934
tcagcttctgaaggcttcttgcattccttagcacttttcactgtcttcaaagctagcaaggttgc
atctttttggccatctttcatagccacatctccctttgactttttttctgcctccctcttccactt
ttaaggaccctcatgattacactggatctacctagataatccaggataatccccccatttaaaga
ccagt

>chrx_136805334_136805534
tagctcctagtcgtcgttcacagccccatttaaaattcccttcaatcaagaagtcttccctgaga
gttacattgagactgagattaaatccctctcttatacattcctctagcatctcttatttaagaca
ttcactgttgtaaaaaagtttcaaacatattcaaatgtaaggagaacagtataatgaggacccac
tgaag

>chrx_137182134_137182334
gaaagaaaatgaatagaaagcagatatttcacataaatgaaaccaaaagtgaacataaatagcta
cacttagataaaacagactaaatcaaaaatagtaaaaaaaaaaagacaaagaagatcattatatg
ataataatgagatcaatccagcaagaggatataacaattctgaatatatttgcatccaaaactgg
agcac

>chrx_138524134_138524334
tcactccaaaaaaaaaatcctgcacctcttagctgttaccccaagcacatcagtcatggccctttc
cctgcatccctgctaataatctactttaatctctatagattttcctattctggatatttcatat
aagtggtatcacaaaatatgtggtcttttgtgactagcatctttcatttagcataatgtttccaa
agtta

>chrx_141528934_141529134
gtcatgtggtcccactaccctgtaggaaaccctgggactgaggaaagtgcccagacccataggag
cccagtaaagggagggagaatccacttagcaggaagggtcatcctcatgccctgcctgtattaaa
aaaaggaagactgatgaaaaagcaaatgatccaacaaacaaaaacaaaagagagaagaaaagaaa
aggag

>chrx_143900105_143900305
gggtaggtatttaatgttctcactacacaacaataaatggtaagtatgtgagatgatgaacatgt
taattagcttgattgtggtggatatttcacagtgcatacatataacaaatcatcaagttttacaa
cttatatataatttttaactatcaactatacttacaaacagttggggaaaaataatccaaatgag
agaga

>chrx_146254308_146254508

gttgaaaaatgtttgttatcagacttaaagtctgtgttgatgttaatgccagaaaggtataatga
ggcatgtctgacccccactttccatcatggcatgaactagtctttcaggttaaatttttaaaaga
gccatggctaaggaggaagttcattcagatggttgggggccttaaaattttatttttggtttaca
ctagg

>chrx_151030344_151030544
cacctgcccaaagctttctggggccctcagccatctggtcaatttcctctggagtgctcagccta
tcacaactaactcatccccattcactagagagagctgttggggaatcaaatttgtttcttggtta
agggctttggagccacacagacccaggttcaaatctccactagactcgttattggctctgtgaca
cagga

>chrx_151090544_151090744
tcgaggtcacagagaggagagggctatggtctgaggggtggtacttcaggtccgcagagggagga
gtcccaggatctacaggacccaaggtgtgccacacttcacgaggaatggggatacctgtggctca
gaaagacgggaccccacagagtctggctgtcccctgttcttagctcagggggaccagaggaggg
atggc

>chrx_153488206_153488406
tcccccactgctgctgggacctgttctctcctttgccccttgtccctgcactgcccaatttgga
ccgcaagggttgccagggaagggcactggctgccttgttttcagaggtcgtagcacctagattgc
tccagccccttgcacttgcctgcaggccagagtgtcccaaaccctcccagtctcagctgctcttc
cccag

>chr13_29696600_29696800
aaccatcagatctcgtgagaactcactcactatcatgagaacagcatgaggataaccatccccat
gattcagttacctctcactggggccctcccatgacatgtgggaattgtgggaactacaattcaag
atgagatttgagtggggatacagccaaatcatgtcagatggggagagcattccactgggaccaag
tcact

>chr13_44212000_44212200
tatgggtttaatttgaaatccatcattacaaaaccatcctcatccggcaataggaaagactccca
ttataattttctgctgggagtcattagaaaaagccaaagcaatagaagggaaaagggtccgagag
cacctctaacttacaaaagtacaacccactttgttcaacaaataaaagcagagcctgctttgttc
aactc

>chr13_68662799_68662999
cccttataaaaccatcagatctcatgatattcactcactatcacaaagacagcatggggagaact
gcccccatgattaattacctcctcctggtcccaccattgacatgtggggattacaattcaagatg
agactttgggtggggatggagccaaaccatatcacatggctatacagtgatcctgaaattgtcta
agtta

>chr13_68845199_68845399
aaatctctgtgcaataatgttgattacataataaaaatcactcttttgcttcaaggttataaata
atgggaaaaaaggatttatgtgactagtcttagagtatagagaccctaatatgcttctggtactt
tgtgatgtgaatattcatattgtttgacccctttccttgcagaaatagcttgttgttgtttttgt
ctttg

>chr13_69173599_69173799
gtgtggtggctcacacctgtaatcccagcactttcagaggccaaggcgggtggatcatctgaggt
caggagttcaagaccagcctggccaacatggcgaaaccatgtctctactaaaagtataaaaatta
gctaggcatggtggtgggcacctgtaaatccagctactcaggaggctgaggcaggagaactgctt
gaacc

>chr13_72733799_72733999
cttagaacagatcccacaaacaagacttatccagcctcaaatgtcaataatgtcaaggttgagaa
gccctgctatagagtatgaatgctagagtgagaatctttttcccatcttcccttccctcagagtg
tgcaccataatgctctgcagacagtaatatattcaatatttggtaatgatcagccctgaaaactg
tctaa

>chr13_74132799_74132999
tctaattccttagctcagaatatgaggcctcagtctttagtatccattttgcttgacttctctgg
ggtgtttaatactactgtttttgaaactctctcattatttggtttctagaataccattctctttt
gctccctttacctctctgactttccagcttcctgtagtgggcttcatttccctaccgtcctcttc

ggtgc

>chr13_83267999_83268199
aggacatcctcacaagggcttttggtcacaaaacaatattacccagaaaatccatactgggcaga
ggagttggtagaaatggcagtggctgctcagcccacggtattggaattagagttgtggtagaatg
atatccatatgaggaagctgcagtctaccctgggatatcattgaataagtggagtaaggagatta
tcatt

>chr13_89844799_89844999
cactgtgcctggccatgtattgtatattttaagactgaatgtaagatcatcgtttttgatattt
cttcattgctattttaagttttaaagtatgttattaattgcttcgtttctcttttaggctttata
gcttgctatgtttccaactaagcactactttaactgcattgcacacattttcatatattatcttt
taatg

>chr13_98519399_98519599
aaaaaaaaaagaattgcaaagaatgacatttgaaacacaggctgggttttaatttagctttcctt
tatcacaaaataatgaaaagagacaagtccttgtaatgactctcgccccttccacacacactctt
ccttcaagtcacctctcatttaagacaggaaagtccgcacagcaagagtctcacccatatttgca
tgggg

>chr13_107789199_107789399
taagcaaggggataccagtttcctcaggcaagagaagaaaaggtaactatgatggcaaaagaaga
ctcaacagggtttggttttaagattctcattttcatcctggtccaaaatctcaaggttctgctca
tttctaatccctttctagacccacttaaataatttgacaaggctgtgaattcaatgtgcttaat
ataat

>chr12_25298533_25298733
ttttcatagcaaatggaaggaacttaggaaatattttttcttcccttttactaactatcaaaaa
taaagtctagttttttttaaatgtcacatagctagtcttttcagaggtaggaccagaacctaggc
ctcctaatttttattctggtaatttttctctaaaactcactgctatactaatgcaagtaacttaa
aatgt

>chr12_39887533_39887733
agaaatatttaaataaagaaaaattctcatctaccaaatttaatatatacatcatatgcattatt
ctatactgttatatgagtgtgtgtgtgtgtgtgtgtgtgtgtaaaataaacggttaaggtaaatt
ttggattaaggcaaaatttaatcctatgatttagattgcttatgaagatatcatacctatgaggg
tttac

>chr12_40702333_40702533
atactgggagtggtaaaaccaccttattgcagcaattaatgaaaaccaagaaatcagatcttgga
atgcaaagtgccacagttggcatagatgtgaaagactggcctatccaaataagagacaaaagaaa
gagagatctcgtcctaaatgtgtgggattttgcaggtatttctttctatagaattttaaaattca
ctttt

>chr12_63792733_63792933
tagctgaggtgctagtgctgcctggaccactgtcgcagtccaacctagtactgacaccatatggt
ttgaaactggatgtggacaaagtagccaattggatgaacttagagcaatatgggtggtgatcacc
aagtgttggggtgatcagacccaacactaggccttggaggctacgaagtctggcagagtcaaagg
aatga

>chr12_68466733_68466933
cttggctctactgacatttggggccagataattctttattgtggtgggccgtcctgtgactgtgg
gatgttgagtaggtctctacctacttaactggcagcaacccaagtagtgacaaccaaaaatgtct
ccagacattgccaagtgtcctctggatgcaaaattgcccctggtaaagaagcactattatagatc
ccaat

>chr12_73802733_73802933
aaatcctgagatttttggctgtgggagtggtagaaatgcttctaccttttattctgggtgcagat
gcagctatgcagctattttgttttttttgtttgtttgtttgtttgttttgtgagacagagttgccc
aggctgcagtgcactggtatgatctcggcttacggcaacctctgcctcccaggttcaagcgattc
tcatg

>chr12_83469869_83470069

ggtcagattgtcgcactgacctaaactgaatttacagtttttccaggcttggggtattgttattc
atcacaatctacatgcaatcatttttaaacaatatttttttcaatatatatttgtgtactctctgt
atatatgtgtgttttagattcacaaccagaaagatcaagggagttgaggcatagcaggaacgtaa
gtagc

>chr12_83643669_83643869
tctccctttggaatgagagcatttacccaatgcctgtaccctcattgtatcatggaagcaacta
acttgtttcttattttacaggctcataggcagaagggacttggccttgtctcaggtgagactttg
tacttggacatttgagttaatgctggaatgagttaagactttgggggaatgttggaaaggcataa
ttgca

>chr12_90612269_90612469
gactggttttgtgtggagagagagagaggaaaaaatgtaggaaaaaacctgaatggctctagaaa
gctgtaaaaggtttgtggagaagagagctcctgatcaccatgagctcctcaggttagttcatcta
cagacaattgggttgcctccaaaaattgggcaagaaagaatcaagagagtgcctgggttccacat
gtgcc

>chr12_103488870_103489070
ctctgttttaagctgccaccaactatcacctggattatggtaatagccccctgattggtcccttc
cctcatagtctatttcaacacagcagctgaagtgatcccattaaatcgtaagtcaggtcatgtca
taccctcattggaaacctctagtagccccatcaggttcagaataaagtctagtgctcttatactg
ctcta

>chr12_129052847_129053047
atcatagattttcatagcagcaaggatgcattctcctggcttcactccaacagctttattattta
tgctcataccttgttccacaaaggattttagacaatttacaatactgtctccaatataggaagcc
aaaacacatttcaaataaataatcatagcaaatacttaccctgtacttgccgtggactgggcatt
attct

>chr12_130108047_130108247
ccacttgtaagtgagaacatgtggtacttggttttccattcttcactcaggataatggtctccag
ttccatctaagttgctgcaaaagacattatttcattactttttttatggctgagtagtattccatg
gtgtgtgtgtgtatgtgtgtatatatatatacatatatatacacatatatatacatatatacata
tatat

>chr11_4545424_4545624
acccagatccttctgaatatctagaaagcctttccaagaagtatgggcacaaacgagcccagaca
gtgaagacaacaaatacctaactgttcaatgcccagatatctaatagatattagcaccaatatca
ttcaggaaaacatgacctcaccaaatgaactaaataaggcaccagagaccaatcctggagaaaca
tatgt

>chr11_20836224_20836424
attttctttatccagtccactgttggtgggcacctagattgattctatgtctttgctgttgtgac
tagtgctgtaataaacttgtgagctcatgtgtctttttgttagaaagatttgtttttgtttggat
atatacccagtaaagggattgctgagtcaaatggtagttctgttttaggtttttttgagaaatcgc
caaac

>chr11_23193824_23194024
tatgtgcagggtcttaacattaattgaggcagatctgaaattagaaaaagaatagccagtctctg
ttactgacctaaatattaacagtttatacagatcttatgccagatatattctcaccttctgtttt
ttcttaatattccatatacattctcaatttctgttctttccccagaaaacttatttgaagccttt
aacct

>chr11_24627224_24627424
tgaattcattgtaaaatgatatccacaatcaagataattaaaacatttgtatacttaagatatac
tctcttaggaaatttcaagtgtaccttacatttttattatcatgcaatatgttaggtctccagaa
cttatttgtcttataacttcaattgtgtgccctttgaccagcatatccccatttttttcaccctt
gacct

>chr11_25022224_25022424
cagaagccttttcctgtaaacaccgggtggcatctcttagtatccctgactggttagtgtaaaag
cagcagtcttcccctaagaaggtgcaaagtcctcctttctcagcagtgaggaggtctaggcctct
gtggttttggagagtcactgctgctaaagagtctatttgggattgtagttactatccttactgaa

tagat

>chr11_38843224_38843424
gtcagtctgcccctaccgggggtgcctcccagttaggctactcgggggtcagggacccacttga
ggaggcagtctccagcagtgtgctgggagaaccactactctcttcaaagctgtcagacagggaca
tttaagtctgcagaggtttctgctgccttttgtttggctatgccctgcctccagaggtggagtct
acaga

>chr11_39197424_39197624
tcaaaacaataactcatactggtataacttctgtgagtaacttctaggtctaccaaaaatacaag
caattgtaatctcatttgctgaatagttgttgcagataaaatatttacttataagattttgcctt
cactggggcaaagggacaagtctagaaatagagcctggcaataagacagatttatttatttattt
attta

>chr11_50108824_50109024
taattgctgttcagcaatatttgctgttctgcagcctcccctgctgatacccagacaaacagggt
ctgaagtggacctccagcaaacaccaacagacctgcagctgagtgtactgactgttggaaggaaa
actaacaaacagaaaagacatccacaccaaaaccccatctgtacatcaccatcatcaaagaccaa
aagta

>chr11_60328024_60328224
cagtgtcaagagaacttccatcactacaacatcaggatcaagaagaccttagcagctactaccac
cataaaccccaacaactcacactactgcaacaaacatccccactgttgcatgctgaggatccatg
taatctttgtcagtaccaaacccagctgacagagctgcaaagagactgcacagctgtgcactcag
tgatg

>chr11_81425152_81425352
gtagactgacctttagggtgatgttgtatattattctgctagaagataggctgtgtttaatattt
gctgtggcttctagaagcttcaaatttctctagtttcctcattttgtcaccccctgttgtcttta
gatttccataaaaactccttttaaaatatagactgtgctgtgcatgtctttcatctgtaatcat
gttat

>chr11_88897752_88897952
ctttttgtatttttagtagagatggggcttcaccatgtcttaaattgtgagctcaggttctatga
ctctataaaagtttatatcatttgcggctgtaactttcctcttctggtggatgtttttcccatct
aactcttcctgtaagagcagcctttttacattgccaagtttatgcaaggttttctatttcagctc
tcctt

>chr11_91790152_91790352
tattgatacaatttaaggtatgtgaggataatcaagggcataccatttttagcctaatatgcctt
catggaaattagtaacaaagaaatcatttatttgtttccatttcttaatacttacatattactct
tttctctctttctttctcttgagtaatacagtgcatatttatgctcaaaaataaacacatatccc
tgttt

>chr11_91878952_91879152
gtgttctttcagtcattgatttttcatgctccttgaagtctcctgctgcagtattcacagctgag
gaagcagtcacctcttccagtttttttgtttgtttgtttgttttggtttttgtttgactggctttg
ggagaaaaagactttcatcagtcagactgcctgggtattcctgtggtctttcaggatttttctat
ggatg

>chr11_99399190_99399390
tctgaaataccatggcagacaacctgagattttttgacaagatctatggaaaaccacagtaagtta
caactgcatatgcagtcaccaaaagagctgagatcttgagaaactttatctttcacaaatgcaca
cgtacaaatgggacatttctccatttattaaggaagcttcagtgttttcactttgagaaccagag
aagct

>chr11_111137590_111137790
cttcacccaccctattgccctcttacattatgattccagcccaaggatgggtgagctttggagtg
gtcactctgcttcaggccctcctgctcagctctcatgtcaccctccagatttcctgtgtgatggg
tggccccaattatgacttttgctccctctcgggaacacatttgtctccccaatcagccccacagt
gtgcc

>chr10_9415794_9415994

caggaataaaaggaagaaagacaaaaaaacagctaagacttagcctctgcattttctactctgta
gctgtttcagagactaaccaatatagacagtaccatttcaagttagatcttttggattgaaaatt
tagatcatattgatagctaaataaccccaagtattcaatatgctaccagaagacaaaagtgttac
agaat

>chr10_15551794_15551994
atggatccaggtcctcacagagggaagatgacgtgaggacacatgggaacacggtgtgatgaggg
agtatccatgtactgcgtttacaagccaaggaacacctgagactcccagaagctgggagagatgc
ctggaaacagatgcttccctagtgccttcagagagaacacagctcccccagtaccttgattttgg
gcttc

>chr10_44979594_44979794
gggctctgttctgttccattgatctatatctctgttttggtaccagtactatgctgttttggtta
ctgtaaccttgtagtatagtttgaagtcaggtagtgtgatgcctccagctttgttcttttggctt
aggattgacttggcgatgcgggctctttttttggttccatatgaactttaaagtagtttttttccaa
ttctg

>chr10_57733594_57733794
tgttgtgggtattgacagccaggcttctaaacctcttaaaactccccaactctagtgccaacttg
gacaatattcttttatgcactcctttttagttatccccacctgcccagttctcttattaggctga
gacattttaactaaattatctgctcccctgactattcctgggctacagccactcctcattgccac
ccttt

>chr10_58136594_58136794
ttggtcaggctggtctcgaagtcctgacctcaggtgatccgcccgcctcagcctcccaaagtgct
gggattacagacatgagccaccgcacccagccttcaatttcttgaggatctgtagtaatttatcc
tctcttatttctgttagtctctttgtctgtctctctctctctctttctcctttctttcctctctc
tgtct

>chr10_59683394_59683594
gtgtatatatatgtatatatatgtgtgtgtatatatatgtgtgtgtgtgtgtatatatatatatg
gtctatcccccaaaagatcatcaattctaagatgataacatcatatactgttgaaaactctttta
aatgaactgtaacagcagtaattgtgtttctctgggtcaactgcatggcaaatgttgcgtgaatc
aatac

>chr10_65919194_65919394
tcgttggaacagaggaatctctggagcaatatcttccaatttcttgactgaggagtatatgtttg
ccttccagagctctaggacctaagctgggagaaggtagctggagttgggtagactcggggtttgg
tttgtagatagtcaggcaatcctctttatttccaatgtagttctttactaccatcttatctgtgg
ctaag

>chr10_77905994_77906194
gggtatccttgttgactttctgtctcgttgatctgtctaatgttgacagtggggtgttaaagtct
cccattattaatgtgtgggagtctaagtctctttgtaggtcactcaggacttgctatatgaatct
gggtgctcctgtattgggtgcatatatatttaggatagttagctcttcttgttgaattgatccct
ttacc

>chr10_83204420_83204620
attttggctgaactaagggacaaaaaatcccgcatcaatgtgatcctattcatttttccttttgt
tgcctttgcttgtctttctgtgcctgggttatttcacttagcataatgatcttcagttccaccca
tgtcgcaaatgacaggatttcatttgtttttatggctgaaaaataaggactccattgtgtgtaag
tacca

>chr10_84686620_84686820
ttggacatccattgaacatgtctcaactgcactgtgtctctttgtctttattttctttatctttg
ggtcattcccccaattctaactgacctttgttatccttctggtctccagtttagttttctgggaa
gggctctccctggctcagttcctcagcaccatcactacttgagcagagcttttccacctgaagtg
ctcaa

>chr10_86460820_86461020
ggacagacacttcatctaggttttcttatttaatcttcctcagatccttggagggtggcattttt
atctctattttgctgacaaaggaacaggttcagagctaagtaacatgctaaaggttatactgctt
gggcctggagcccagatgcatatgactccaagtccagccccatgagtacaggatgtgaggtaggc

ccctt

>chr10_87312220_87312420
caagtaatcaaaactatgtactattggtaaggatcaaacatgtagatcaatgcaacagagtagtg
tccagaaattaacccacacaagtgtggccaatttatatttcacaaagctacaaaagcaattcaat
gaatacaagtatccttttcaacaaatgatgatagaacaatttgacatacacagacaagaaaataa
acctt

>chr10_90156820_90157020
cagaagatcaataaagaaatagaggacttgaacaacactataaaccaattaaacctgacatgcat
atacaaaatatttcactcaataacagcagaataatacattcttctcaagtgtacatgagacattc
tccaggatgaagcctgtgttaggccataaaaagaagtttcagtaaatttaaaagaattaaaatga
taaaa

>chr10_96618210_96618410
gaaatcccccaacctcttgtgcttcccaggtgaggcaatgacctgccctgctttggctcaccctc
catgggctgcatccactgtccaacaagtcccaatgagatgaaccaggtagttcagttggaaatgc
agaaatcacccatcttctggatcgatcatgctgggagctgcagaccatagctgttccattttggc
catct

>chr10_96692610_96692810
gaagtagtaccagaggaaattcttctattcttaactcattttatggggccagcattactctaata
ctcaaatcagacaaggacattgtaagaaagcaaaactatatatcagtatatctaatgaaaatagg
tgcaaacctcaataaaagattagcaaattgcatccaatcgtgtagaaaaactgtatacaccataa
ctaag

>chr17_11621475_11621675
gagccactacacccggcctgacaacatacaaactcccgtatctggcttctttcacttagcataat
gtttttgaggttcgtccatcctgtagcatgtttggtacttcattcctttatattgctgaagagta
ttacactctacgaattaccacattttgcttatccattcactgttgatagatatttgggttgtttc
tagct

>chr17_15070675_15070875
aagtatattgtggttgctaattatcaatttttttctagaaattgttcaaaaatattttagtatat
acgaggaatgtcttatggtctttttttttcaaactgttaaagcagtcattattattaatctattt
ttaagactgaaccatttctgggtttctgaaaaaacaaacaaacaaaaaccctacctcttcaaggt
agagt

>chr17_59355018_59355218
tataacccaattaaaaatgggcaaaggatctgaatagacagttcttcaaagaagatacacaatgg
ccaataatcacgtgaaaagatgcccaacatcattaaccatcagagatattcaactcaaaaccaca
agcagatactacttggcacccactaggatgactatgatcaaaaagatagataataacaagtgttg
ataat

>chr17_72067805_72068005
ataattcacataccatacaatttgccacttaaagcgtgcaattcagtccaggcatggtggctcac
acctgtaatcccagcactttgggaggccgaggtgggcagatcacttgaggtcaggagttcgagac
cagttggccaacatggcgaaaccccatctctactaaaaatgtacaaaaattacccgggcatggag
gtggg

>chr16_16999099_16999299
catcttccgccataattgaaagtttcctgaggcctctccacaccatgaggaactgtgagtcaatt
aaacctcttttctttataaattacccagtctcagatagttctttatagcagtgtggaaatgaact
aatacatgtcccttcaccttctaccacatatagaaccaacaacaacaaaaagaggaaagtacttt
gttag

>chr16_25598099_25598299
aggtctatttatgttcaccattgtattgctggcatgtagctccttttcctgcaataacacagctg
cctccttcccagtacaattttgtcccgtctcctgtatgcccaagaaccatacaaacaagtaaaga
tgtacggcagaatttgcagtcagagccttttgaaattccaagtgaattccctccagctgaaaatc
ccaac

>chr16_26182299_26182499

tggcattgctaagcattttacacttaattcatatcataggaagaacccaaatgggttgcagttgt
gcccatttcatagattaaaaatacctcaagcacagtaagttaaataacttctctccccaggatca
gactgtgatatagcctggatttaaacctgtatgacaccattaaagcctgtatgacaccatagtgt
gagct

>chr16_61873699_61873899
ctagatctgaaatagctcacagttcaaatgatgaggcaagtgtgagcaacagccgctaaaaggca
aatttatctcataccattttggaccttgctattgtgtctcacttcactccgttataacaattatc
acaccaaatttgttttctatatgtgcttttgttcctaatttttgcatctaccttgcctgcccctc
tttgt

>chr15_24044107_24044307
tgttatttttcacaaaaatagacaaaatatctaaaatttgtatggaaccacaaatgacctcaaat
acccaaagcaatgttgagcaaaaagaacaaggctggagacatcatacaaccggatttcaaaatat
gtacaaagctttagtaatcaaaacaacatggcactggccaaaaaaacagaaacatcaatcaatgg
aacag

>chr15_32525908_32526108
gcttctggtggctgcttgcatcccctgaagtgtggccgcatcactccaatcaccatttctctcat
cacattgccttctcctacccataatcaaagctacttctgcatcccttttataaggacacttgcaa
ttatatttcaggcactctggacaatctcagacaatctccccatcttttaaccacatgtgcaatat
ccctt

>chr15_34002908_34003108
agtgttctccactgttgcattttgcatacctacaggcttaacactacatggaaactgccaagagt
tatggcttgcattctccaaagtggcagcccaagttttagctgggcccatttgagccacagctaga
gttggagcagccaggatatatggggagcaatatcatgaggctgccctgggcctggcccacaagtt
aattc

>chr15_88743996_88744196
gggagaacagactaatacaaccaccaagctgcagccctccctccaagcctactggaagcgcctta
gcaatatgacagggtctctatcacaggctctgattgccacttcaatgagatgctgaaaatctggg
acaagagctctgcccaaatcccagctgctcagctaggctctaacaatgcagcgagcacaccatca
tcttt

>chr15_94705596_94705796
gtttttatctcatcaggctgttttgaagcataaataaataagcagatgttcacatatccaataca
tgcgtagtatagacataatactacatgtgcaagatctgtcagagtagatgaatgtgaattcaggt
gtagtttctgcttggggtgaaggaacgttgggaggctatactgtttcatgggaaaaaacacaggc
aatgt

>chr14_28076160_28076360
ctccaaccaccttgggcacataccatcaagacctcctgaggctgtgtgatgggcacatctttaac
cttggcaaaataaactttttaaattgattgagacccgtctcaaatacttttgggtttacaaattg
gtaaccacagaagaattctgagtagaggtgtccctgactttgaacaaatctcctattggtacttg
gtacc

>chr14_44848650_44848850
accattcaaatcccaagttcctcacaggaacaggaggtgccagactcctccctgctgaaaatgtt
gtgaaagttgtaatgctccacctcagtgggcaggctggctggagtttctctggggaacccctccc
atctggctgtctcatccctccctctaaggaagtacatctaactgccattagattaggatacagat
gaaga

>chr14_48350050_48350250
tggggctggttcataattggaacacaaaaatgatccagattttcatttaagaacaagtacaaata
aataggagcaaagcttttacatataatggtttaaatggcctgatagaatttctaatcacatttta
ttactattttactatacatgtattaaaggagacagcactaaaatacattatatcaacaataataa
ctggt

>chr14_88260047_88260247
acagcctcctgacaaattggctgtagctcttagtccctcaacgactactgtgcctgccccctca
cttagcctgcttctctcacttactgccaattccagacccgttaagacaatggattaccacaatgt
tcattttacaataacatacacaaggcatgaattcacagtaaccaagcctcagtacactctcaaac

agcta

>chr14_89393047_89393247
atctgatgattttataaatgggagttctcctgcacgaggcctcttgcctgccaccatgtaagatg
tgattttgctcctcattcaccttctgccatgaccgtgaggcctccccagccatgtggaactgtga
gtccattaaacctctttcttttataaattactcagtcttgggcatgtctttattagaaacatgag
aacag

>chr14_99479047_99479247
ccaagaggctggagccagcccatcaggagcagggagggcacagaccaggagcctgggatcagccc
caaggatccggtggcggtctccatgatgactgacctagaggtatgctcagccagagtgggactag
caatggagccggggaggcagcaatggccctgactgggaggcagagccgagcacagggcacatggt
cactg

>chr19_28388560_28388760
ttaaaaagttaataaaccccctagtaaggtccaactattccatgtttagacatagtcaaaaaaaat
taagcaactatattatatgtgccagaccctgtgctaggcattgagggtcaaaagaaataagcaca
gcttctgttccttagaacttagatcaaaactagaagtattcagggatttgtcaaaggtctcacag
ctaat

>chr19_33218560_33218760
ttcattttattttattttattttcttttgagacagagtctcactctgttacccaggccgga
gtgcagtggtgcaatttcggctcactgcaacctccacctcccaggttcaagtgattctcttggcg
tagcctcctgagtagctgggattacgggcatgtaccaccacacccggctaattttgtatttttag
tagag

>chr19_36992360_36992560
tttatatactttcatgttttcatgttactaataaatgactttttccttcagcttgaagaattctc
tttagcatttcttgtagggcagatataggagtgttaaacaccttcaacttttgtttgtctgagaa
agtcttaatttctccttcatttttgaaatacaggttatctgagtatagtattctcacatggcagt
tgttt

>chr18_6937400_6937600
gcgggcggatcacgaggtcaggagatcgagactatcctggctaacatggtgaaaccctgtctcta
ctaaaaataaataaataaataaaaaaggcgcctgtagtcccagctactccggaggctgaggcagg
agaatggcgtgaacccgggaggcggagcttgcagtgagccgagatcgcgccactgcactccagcc
tgggt

>chr18_7865600_7865800
attttcacatcaatgttcatcagggatattggcctgaaattttctttttttgttgtgtctctgcg
aggttttggtatcaggatgatgccggcctcataaaatgagttacggaggattccttctttttctc
ttgtttggaattattttcagaaagaataccagctccttttggtacttctgatagaattcggctgt
gaatc

>chr18_14328600_14328800
gtcctgtctccatcgaacagcctgtgctcttatgtgtgatagaggaaatgacctctgggtgggac
ttacattaaatgagtcccaacccttatattaaatgagaaacagaagtcaaaagtttggcaaattt
gcagcccggacatgtggtcaaaaagaaaagctgattttcaggggggaaaattgaggaaggcttcag
aaact

>chr18_26420402_26420602
gtgtcactttatctcctcagcatttagaactctgattaaattatagaaaagctcaataaatgtgt
gtagaataagtgatgtcaatgatagtagcaccatcgattgtcaaattgaaccctctcagtagaca
aggcttaaataattcaatattctgtactaaaaatcccccgtcacaacaataaatcctgttatttt
aaact

>chr18_26877802_26878002
gatggtgagtggtgccactttcactccttgactcctggacccatgaatcctggctatgggagaaa
gagtaccatatattggacgctgattcagagtatacacatcctcctggagaactttgccctagccc
tgcaaagtattgtcacctaattggcattgtaattgtgacttcaaaaggccacttcaccattttat
caagg

>chr18_50855202_50855402

tgctgagattacaggcatgagccactgcacctggccatgactgcagttctttctgacagtaatat
gaaatactcagaccattggttttatgaaacttctccttgttcatgatggtaaagctttgttgtct
acatttagggaacagatgtatccatttctgttacaaaaggagagtgtacattcagtacatcttaa
aagcc

>chr18_59018820_59019020
ttagtttgcctctatgttacttttggacagcaaaaataactgcaaagtctaattaaaaaaaaaaa
acaattttcaggtgatatggggatagttttaaaaagcagcacctttgggatgcctaaatctgtgt
acaagctctgtgatattagccaggtaacttgatgtctttctttaaaatatattcctactacctgt
gtcat

>chr18_60376220_60376420
tctacaatggggcggaaaaaagaaaagaatatccatgctgcttcttcacggtataattaatgaat
gtaagtatggaggaatcattatctggagaatacttttctctttttagtcacggaagaatcagaat
aaatgttttgggatggcgtaggaaaatagatttttgttagataggaatctttttttatcttttgtt
ttttt

>chr18_72556812_72557012
catccttgatgcgtctgccctaaataagctgaagagaaatcagcctaagaagagactaagggcac
ataggcatccaactaatgattgaaaagcaagttctgaaagtgaaataccagaaggaagaagagcc
aggtgatcctgattgatgattccggagtaaagcgtgtggctcaggccatctgtgacccacataaa
gagag

>chr22_16387400_16387600
acctaataatgcatttctcagaatgcatccccattgttaagtgatgcatcctgggctttgtgcat
taaaaaaatgccagaacactccatacttagtgcatagactttatcaaatgttgaataaaaacaga
aacaaaaaggagacaatcctacaatgaaaaggagacactcatgtctaccctagtgattcactgta
cgtgg

>chr22_35483000_35483200
taggtcttagtcctacactccttctaatttgctgtgtaaccttaccattaatctctctgggtctc
agttttctcatctgtattggaggtagcagtgctagctctgccttcaggcatgcaatatgccagaa
ctacagacaacagcccacaggatgcaaaagtgctttgccatcttaaaaatgccagatcactcaga
gccta

>chr20_53526193_53526393
cctcctcttcacaggttcaagcgattctcttgcctcagcctcttgagtagctgggaccacagaca
tgcgccaatacacctgcctaatttttgtatttttatagagatggggtttagccatgttgctca
ggctggtctcaaactcctgagcgcaaagcaacctgcccaccttggcttcccaaagtgctggtatt
acagg

>chr7_19297475_19297675
aatcattaataaaaaattctatctaacaaaatagccctggaccagaaagattctcagacaggttc
taccagacctacaaagtacagctggtaccagttctactgaaattattttttctaaaaaaattagg
tagagaggctcctccctaactcattttataaagccaagatctcccttacactaaaatctggcaaa
gacac

>chr7_28409875_28410075
tgaagccaggcctctaccactcactgctgagctgtgagttctgggaagaaatactcaacatctct
ctgcctcattttgtcatttgtaaagtgagaaaatacagagttgctgagtgaagtaaatgaaatct
tgtatgtgaaagagcttggtaattataaaacacctcataaaggtgaggtgtttgggaggataaaa
gtgac

>chr7_37875675_37875875
ttctaattaatgaatatgatacactttttggtttctttaattttttcagcaatactttgttgt
tttcagcaataagatatatttctcttttaaatttatgacatatttttatcttctttgatggtat
tgtaaattttaaagttaattttattttccaaaccttcgatgctagtttatggtatacaatcaatt
tttta

>chr7_46683475_46683675
ttagcatatttagtctatttacatttgatgtaactatagataaatcttttattctctgtcctttt
tgacattttcctggttttctttctgtctttagttttgtaacatgtcattctgatttatttaaatt
gttttaaattgtatttatttgtataaatttttactggtagctttaatttgtaatctatatcgact

tgaga

>chr7_48928854_48929054
ccaacaccatatgaaggagccatttcgttatgcaaagaatacaatctgaaatggcttaaggggac
ttgattggctaggagctgcccatggcaagcaaaacacatggaatatgaagatatggaactgctaa
gaggaccccccagacacgactttatgcagaggctccttcttagctgtgaaggatatgttcaaaaa
ccagc

>chr7_49732854_49733054
tgtgtctttaagtgtttttttgacgtcactgtttcagttttgttccttggaaattcaatttgtgc
atattttggttctccttttcctgcgttttatggtttttaaaaatttttctctttgttctttttat
ttgaaaatgttttattttgcttactcttctcatttctgtcctctccatcacctgctaggatttta
gtaag

>chr7_49970854_49971054
gaaaacgtggaagctttggaactaggtaacgggcagaggttgggacagtttggagggctcagaag
aagacaagaaaatgtgggaaagtttggaactttctagagatgtattgaatagttttgcccaaaat
tctgatagcaatatggacaatgaagtccaggttgaggtggtctcaaatggaaatgaggagcttgt
tggaa

>chr7_56554506_56554706
gatttgatttgaggtgatttgagggagctggagcagcggtagatcctgaccagcactcacctgga
acgcttgttgatatgtaaagataacacccactctctttatgaggtgtgatttttttttgttctaat
tattcctctgggccttctcttccaggtttgtgctatgtactaacatgcaatacaaatactgatga
aaaca

>chr7_83412664_83412864
atgtgtgtatgtatgtgcatgtgtgtgtgtacaccatttttttcaacagattgaaaatcatagtaa
aagggtacacttgggtttgatctcaaatgattacttaatcttaggccctaaattccagaagatta
ctcctgaactgtaataattgtaaaccaaagagctctgtgtttttctcaatatgatttaaaaaact
ccaaa

>chr7_88649864_88650064
ggcttaaatctattttgtaatccttgagctttctggatgtggatatacacctttcccaagacttg
gaagttttcagctattatttcatttaataggttttctatacctttcacttattttcttgtttag
gaattcccataatgtgcatatttgtttgcttaatggtgtcccataaatcctgtaggctttctttg
ttttt

>chr7_122823564_122823764
aaggttgcctatttgctccgcctttcaaatattgctttgtcattcaagaatcaacacttagattg
tgcatttggacttctcccctctctgcccctctgcctggtccatgaaccagcagcgtcagcataat
ctgggcacttgtgagaaatgtaggacatcaagcccttccccagacacactgaatcaatcagcatt
ttcag

>chr7_141939726_141939926
tcacagtaaacattagcccatgggtcctcttgtcctcatagaaccacagagaaaagtaaaaataa
ggaatacatcttttatttatcctccacatttaatatgaagttgtgagtagaggaggtgaagaaga
aaaagagttacattctcatagtcactaggagagaatctgacctcagaaacccctgaaggatgaga
gatga

>chr7_144349267_144349467
ccttatccttcacctatgggtgtgcaactaccagcagaagacaatggacctggctctcactgctt
gggcaaaggggtaacaaactagggcaaaggggactaatgttatgacacatctctctcagaaaatc
tgatgtgtgagttcagaataaaattaatagagacaaaatttggagaaaaaaatgcaaaccctgtc
cttta

>chr7_152393667_152393867
gggttgatttctaaatatataaagaactcctacaattcaatagcaaagcaaaaacagcaaataac
ctggttttacaaagtatatgggacatgaactcaaatatgaagaaattttttttttttttttgata
cgaagttttgctcttgtcgcccaggctggagtgcaatggcacgatctcggctcactgcaactcca
gcctg

>chr7_152973067_152973267

tacaaatatatttaaagaactgaaggaaataatgtatgatgacaatgcttaatcaaatagagaat
atcaatagccagaaattataaaagaaaaccaaataaaaattcagtatttgaaatgtacaatgact
gaaatgaagaattcacaaatgggctcaacaattgattacaatctatttcaactgacagaagaaag
aatta

>chr6_50144641_50144841
tcatattcccaccaacagtgtataagcattcacttttctctgcagcctcaccagcataagttgtt
ttttgacttttacaaaatagccattctgactggcatgagattgtatttcactggggttttaattc
atatttctctgatgattagtgatgctgagcatttgttcatatgtttgttggctgcttgtatgtct
tcttt

>chr6_65712679_65712879
tttattagttccccaaattaatacttttataatttcttatgcctgtctttactgcaatctctaaa
cataaattgtaaagatttcatggacaattatcacttccccaatcaatatcctagtgatttcctat
gcctgtctttacttcaatctcttaatcctgtcagctgaggaggatgtatatcgcctcaggaccct
gtaat

>chr6_72558279_72558479
ctctggtacattataggttagaatgggaaaagtcacatggggagaagtgatataaatacagtgta
acaagggctttaatagatgaaaaaactataagaagatcgggggaccactaactctgtcccaggt
agtcaaggggaatttcataaaggcagagaaaaaataaagctgcattttgaagagagaatagattt
ttttc

>chr6_78519481_78519681
accaaatctaaataatcataatattgcatgtgaagttatcagatgaaatagaatatctgagacat
gcttatattgaaaaaaaaatcattgtttatttgaaatttaaattatttgtgtttcttgtatttta
atttgctaagtctagcagctctagttgtgtggctcagtgagatttttagatttctttacagtcta
tgcta

>chr6_87011281_87011481
attagtttttctaattctgtgaaaaaagtcaatggtagcttgatggagatgacatggaatctat
aaattaccttgggcagtatggccattttcacgatattgattcttcctacccttgaacatgaaata
tctttccacttgtttgtgccctctcttatttccttgagcagtggtttgtagctctctttgaagag
gttct

>chr6_91656479_91656679
attattaatttcaatgtaatttcaatgatacattatctaatagctatgactatttatgacccaca
ctattctaatgggtattgagcacattttgatgaaatatactgtataccctgaaagtggtcttttg
tcctgttgtgggatagccatctatgacaggctggaattagcttgggatttttgttaggcacttta
cctgt

>chr6_102762707_102762907
ctttgataataaatttaatgtagatatttctcatcttaactaatgtattaaacctttgacatttt
ttcaataaatattataaaatctgcatttagtatttgatacaataattacaatgtttttgattta
gtggtattgttatttgattccatgttcaaataatatgctaaagtgttttaaaaaacatttggaat
gtgtt

>chr6_104343507_104343707
atagcaagtattgaaatttttagagaaaaactttagagtagctattatgatcatgcaaattaggc
aaaagaaacttcattaataatgaatcaaaagtgactacattttaacggagaaatagaaaacatta
aaaagaaccaaagtaaattaagaactgaaaaatgcatctcaaaacacacaaataacaacaccaga
taagc

>chr6_113354307_113354507
attgagcttgaaagatcaacttgcagttgtttcaaatctagccacaaagccgtatttgttgattc
tgtgctctcttgctctctttctctctcacgcacacataaacacaagttcctttaatctattctta
ttttccacttactgcctttgccttaaagttttgaagagttctacatattattttctaatgagtta
ccaaa

>chr6_116069307_116069507
aaagtggcagaaaggagaagtgccaacaggggaaatgccagatgcttataaaataatcagatctt
gtgagacttactcattatcacaggaacagcatgggggaaactcccatgatccaactacctccacc
tggtcccacccttgacagatggggattattacaattcaaggtgagatttgggtggggacacagag

ccaaa

>chr6_116197707_116197907
caatcatgccagttatagtcacactataagggaataaaaaagaggaaaggaatcaaacaagcata
cagcacatatgcaaaccattaggatatttccttttacatctaattgtttcaggattgtaccaatg
catccatattaaattataatgttaataggttgtccttttacctagagtaaaatagccaagaaaag
atgtg

>chr6_132758507_132758707
tagagctacagtttaatatagacattgatttatcttaatgccaaggtgagtcttaaagggaaatt
aaggtagtcaggctcattcaagactattaaggctagaatggccactgagggggaaaaaaagaaaa
ttgatttggaaggagattttaagaataaaactaaagaaatgttttgaaagatgaatgtctcagtc
cattt

>chr6_163493210_163493410
ttgatggaaaatcctcaacaaaatactagcaaactgaattcaacaacacattaaaaagatcattc
atcatgaccaagtgagatttatcccagggatgcaaggatgttacgatatatgcaaagcaatcaat
gttatacagcatgtcaacatcatcaacatatgaatgaaggacaaaaagcctatgatcctttcaat
ttgtc

>chr6_164645210_164645410
aaaatcaaaccacagtgaggtaacatgttgcgctcttatttgcaaaaattaaatatctgactgca
acaactatcgttgaggtttgcagtggaaactgtcattcaatgcttgtaagaatataaatttgaac
aagtattttgaagttcaatatggcatatttagtaaagtttaaaaaatacacatcctctgattgag
tcatt

>chr5_18718843_18719043
ttcacatccacaaataatgcttctgtgggatgttatacaggaatggaaaataaccttgttatgag
gtgtcacatacctcaatggcctttttagaataggattttgtctgtaatttaatgaaaggtcact
tgtgacctgggtgtgcaatctccgctcttcacatgatgaacctctttctgaagctatgtctgttc
agttg

>chr5_19168843_19169043
tccacagtcagtctctttaaacttaatcccaattttatttaaaatgcatttagaatttcagacgt
ctgctatattatatctggagagtcatcagtgtattcttcacataggaaaatcgtgcctttttttc
tcacattcagttgtctcaagatacaactgcaaagtaaacagtttcagatattgcgtgttaacttt
tccaa

>chr5_20503843_20504043
ataggcaagagaaagagttatggccaggtgcggtggctcacgcctgtaatgtcatcattttggga
ggctgaggcgggtggattatctgagctcatgagttcaagaccagcctgggcaacagggcgaaacc
ctgcctctactaaaaatataaaaattagccaggtgtggtggcaggcgcctgtagtcccagctact
cagga

>chr5_22851243_22851443
tgtactgtagtaagaacttaatacatatcattccgtgtgtggaagaaatgactcacactccctcc
ccctccccccaacaataatgcagctttctcttttttgtaggaattatctaaaatgatcacatcac
taagcagatcagtgagaggagtgagtttgagtagaaaataaaaaagcattcgtggcttaaaaact
tcccc

>chr5_25025443_25025643
gtgctggaattacaggcgtgagccaccgcgcccggccccagttttgatttctttagagtggtgaa
ggagaatttgttccatgcctcttttctagtatctagttatttattggcattctatagctttcttt
ggattgtagatctttgacttcatcttcacaaggcattctccctgtgtgaatatctatgtgcaaat
attct

>chr5_25123643_25123843
catttacatttgtatgcctcatatatgttatttcatattgctatattttcctgtaccctattatt
ttctggaaaaaatttaaatcaaattttaaatttaataatcttaatcactggttttgctttttgtt
taggtgatcagataattttaagacaaaattataagaatatatacattatatttggaaagttattg
ctttc

>chr5_32876843_32877043

aaaccatcccgcaacccttactcccatccccatccgcggaaaaattgtcttccatgaaactggtc
cctggtgccaaaaaggttgagaactgctgctctagaactctctgttttgcattttacttctgtga
gaactttcttttagcttccacttatgagtgagaacgtggtgtttagctttctgtttctggcttat
tttac

>chr5_34370843_34371043
gaaaagtcaataaaaggactttctcttctttagtgtcttagtactctttctttcttaatacctat
ttattgttctttagtttgaatgcaaagtttaatgatccatacattttaggcatttaatttaataa
aaaaagaaacgtaggatcttaaaaaatgactaatgacatcttaaaaatactcttaataaactaga
ttgcc

>chr5_35974443_35974643
attggcagaataatcatgctgcttctctgaagtgtggagtgtaagctagcatggttggaaagtcc
aggtggaagtcactaaaatgacctgtatctggcaaaacagaaaaccaaagcaatattgcactcta
cagagatttcaaagactaatgccacaattatggacttaaagattgcaagggtggtgattcccatc
acatc

>chr5_45532443_45532643
tttcaagataatattgatcattctagcagccttctatgtgggcattttttgttagtttactataac
tttagaaaagaatgtgatttccaacctttaagctattaaaaatttgttattgtaaaatatagcca
attataacataagaatcatttacagtcaatttttttaactaaattgagagaaattataattatgga
gcatg

>chr5_56945643_56945843
tcaatgaattcttcatttctagatgttctttgtgtgtgtgttttgttttaatgtctacctcttca
gcaatttttttcattgatgtcttgaattgtttttctaatttctttgtgttggttttcaattttctt
ttgtatctcattgagcttatttaaaatcaatattttgaattatttatctggtatttcaaagattt
ccttt

>chr5_62055044_62055244
agaatacagttgattcttgaacaacgacagtttaaactgtgggtccacttacactcagatgtttt
caatacagttggccctctaggttggagcgtttttcatccacaaacaaatgcagatcaaaatggag
tattctaaggatacaaagcccatatggagggctgacttttcgtatccacgggttctcaggaccac
ctgca

>chr5_65946244_65946444
ccaatttcaagccaccgatatgacatcactggaaagaacagcacagtagcatatcattgtataat
atttttcatcatacagctaaaatagacaacctcaaaagcataagtaatagtataatgtaacataa
gttatgaattttgagtatcattgttttgctataatttaattataagctactgtaatttttattag
tatgt

>chr5_68109844_68110044
tttctcctctgctttttcttctcttcctggtattccaattatacacgttacaccttctgaaattt
tacctacagattttgagtaatcagttctggggtttgtgtgagtttttcttttctttctttttttca
ctttgcacttcagtttggaaagtttctattgagctatcttcaaactcaacgatgctttccttggt
tatgt

>chr5_69290844_69291044
ctttattgctctatctgttttactatcgctcttcttcagtctcaaaacatatatggacaaaagca
atcacataaaggtctgtcctacttaatatggacatctttataaaagcaattacagaatcttctga
tagatctatacacacattcagatgtaagggatatgtttcttatacaggatactctcagtacattt
tgtta

>chr5_85368644_85368844
tcttatcaggcagtttagtgtaaaaaatttcagatacaatacttttttaaactaaacaagaaagca
gatcttgcccacttgaaataggcaattccaaacaacaaatagattgataagaatttcagtagatt
aatataaaacaagtaaaggaaaataataattttagaactttgtttcactagataaccatctttta
aatat

>chr5_86358244_86358444
cacacgaaaacatcatattaaaagaaatcatgaaaaatttcattagaagcccttaacttctaatg
agaatgtgactgctatcagtcaggaattctccctactcccaaacctactcttccaaaagtataca
aaaccaagaagaatgaaaaattggaattaaataaaaacattttcaatgaagctaaaatacatatc

atgtg

>chr5_91322644_91322844
tgcccacctttatacacctttgatctatagaaaatatagcaactttagccaaattcataatatga
aaatataaatgtgaaaggatcaggcaggattccaaagactttaaccttgggtgaacatccaggtc
tcaaatgttggtaactctgattaaaagattcaagcatatataaagaatcttggatctagagaaac
taaaa

>chr5_103160101_103160301
atggtggtggaattgccttctattatatttccaacccaaccagctgcctcaccattatgtgccat
caactacaagagctaagaaattatagatggcttggaacaagccacttcctctgcctgagaaatac
tttagaatgaattttgaagtttggtctttgcaattcatcagtattatattattttcatagataga
attag

>chr5_115979101_115979301
gatgtaaaaaaagtgtaatattaaatgagcctgggtaattgtattctcaattgcgtaggaaactt
tttgaatttcccttttgcatttgatgttctggctattgaacattcttttgtagaaatatctctag
atgtctatctgattccagagcatttacaaagattttggaatgcagctctttcttgtgatttgatt
ctgag

>chr5_121571301_121571501
agtcgagtttccacctgtggtgtttttttgtttttgtttttgttcgtttttttattattattatt
tttagtattattatttgttttatattgacagtctcactctattgcccaggctggagtgcagtggt
gcaatctcagctcactgcaacctcctgggttcaagtgattctcgtgcctcagcctcccaagtacc
atgtt

>chr5_155219822_155220022
attagttcagccattgtggaaagcagtttggagatttcttaaagaacttaaagcagaactaccat
ttgacctaacaatcctattactgggtatatacaaaaaggaaaataaatggttctaccaaaaagac
acatgcacttgtatgtttattgcagagctgttcacaatagcaaagtcatggaatcaacctaggtg
cccat

>chr5_157327822_157328022
ttcctgcaactgtgcttttgtcctcacaggccaggtggtgggagcacagcaagagaaacaaaaac
agcaggtgttctccccacgttgttgggactatagcccctgtgattggagaggatggttcaccccc
tcagaaggttagcccccgggcacctccactgctgttaccgtggctatcattgcaactgttgtgac
cattg

>chr5_164305822_164306022
acaccccagtgtcatcccttgcccgtaagtgcagactgtatgtgaggattctgttctgatgatca
gaatactgtgtgagtgatgggatgtcacttccaggatccggttgtataaagactatggaaccctt
atgggtactctttttttgttctgtcacacattccctctctgatgccttacactaagggatgaaaac
tgtta

>chr5_164909622_164909822
acaaggatgtggaaaaacaagaatcctgctcatgatagtagaaactggtgaaaaacactgaaact
ctttagcactttttactaatgctgatcatatccatgccttatgaccctatagctccactcctaag
cctataaactatggacatgcataagtatgtgcactggaagatatgaacatcaaggatttcatagt
ggcta

>chr4_18795702_18795902
ttatcaaggggaaaaagatgtaacagccaaagataactgaagattgatccctcttcccaccacac
acacacacgaatccatgatgatataatgttaggccttagtacacctgtgggggctttgtgtttta
gcatacagagagaagtttatttgggtagcaaattcctaacttttgatatttataggacatttatt
tttgc

>chr4_20174102_20174302
gaacaaattttagaaagcaaaagatgccagtagtatgtgcctttaggaagagtgttcttggctta
aatccaaaagctcatcagaagattcttggacacatccctcccactagatagatggttagctaaat
cctgccattagaatatcagtacatttgcctcaagtgaaacttttataaaagttactataggagtc
cctaa

>chr4_29760102_29760302

cttgtttcataataaaatattttgataaaataatatattaagatatcctaaaaataaattatctg
gacatcgtcacttcaaagtcatttcagcagaactgttaatcatggacttggactgcaaattcttt
aaaaatagtcttctgatttatgtttaaatatcttcattgatgtggatatgacttcttcttgagtt
agttc

>chr4_31010302_31010502
aaatggacagcattgtattattctaactcctatacccacagattgcctgccatgtttttttgtcc
attcttctctttattttctgcagtttagtaaagtttgttttaaaagaaatcccttcaaaaagtaa
tttaaaggagaaagttccccttaagctaacatagttcttatttcttctattttcaacttcagttc
ttttt

>chr4_45806443_45806643
ttttgccaccaggcctgccctaaaacagctcctgaaggaagcattacacatggaaaggaacaagt
ggtaccagccactgcaaaatcataccaaaatgtaaagaccatcgagactaggaagaaactgcatc
aactaactagcaaaataaccggctaacatcataatgacaggataaaattcacacataacaatatt
aactt

>chr4_58119843_58120043
ataactcctagggagacagagaggtggcaaccttttacaaacagagtgtcaaatttctaacctct
gatttctggtattttcaagagatagctgaggtcactttgttttgacttctgtgtacagggaagag
agaggaaatacggggatgataaaaggatgaatagtgaatcccaaactgtcagagggaaagtgtta
ttgat

>chr4_59719243_59719443
tgggatagtaaactagttcaaccattgtggaagttagtgtggccattcctgagggatatagaact
agaattaccgtttgacccagccatcccattactgggtatgtacccaaaggattataaatcatgct
gctataaagacacatgcacacgtatgtttattgcggcactattcacaatagcaaagacttggaac
caaca

>chr4_68067605_68067805
cgggatttcaccaggttagccaggatggcctcgatctcctgacctcatgatccgctgaccttggc
ctcccaaagtgctgggattacaggtgtgagccaccgtgcccagcccccatattttacttatattc
tttattaactctccctccctgttcagacgcaaactttatgagagtgaggatttttttgttttattg
tattc

>chr4_72917736_72917936
aaaagtccaggaccagatggattcacagccgaattctaccagaggtaaaaggaggaactggtacc
attccttctgaaactattccaatcaatagaaaaagagagaatcctccctaactcattttatgagg
ccagcatcattctgataccaaagccgggcagagacacaaccaaaaaagagaattttagaccaata
tcctt

>chr4_73630136_73630336
cacatattattatttctatgattcctattttcagtagtaattgcagcattaaaaagatgaccctta
agcacctcattttcttgcatttatttataatttttgatattttatttttctccttaattttaga
gtgcagaaactttggtttgttgtagtcctttggccacaaggcctccagcatttacaaccatgcaa
aggaa

>chr4_77267376_77267576
caaaaattaactcaagatggattaaagacttaaatgtaagacctaaaaccataaaaaccctagaa
gagaacctaggcaataccattcaggacataggcatgggcaaagacttcatgactaaaacaccaaa
agcaatgacaacaaaagccaaaattgacaaatgggatctaattaaactaaagagcttcttcacag
caaaa

>chr4_82151776_82151976
gaagatgtgggaaagtttggaacctcctagagacttgttgaatcgttgtaaccaaaattctgata
gtgatatggacaatgaagtccaggctgaggtggtcgcagatggagatgaggaacttattgggaac
tagagtaaaagtcactcatgctatgctttagcaaagagactaatgctttagcaaagtggcatttt
gtccc

>chr4_101748777_101748977
atttgcagatgaggatgtcttgtgaataaaatatagtcatgtttcataggttttaaaagaaaaaa
aggactatttagcacagtcctgtatccttccctttcagcataggctagaaaagagtaagtgtcct
agaatcaggaagacctaagttataaatccctatgcccctcttactgattatgtggcaattgccaa

gttta

>chr4_112011751_112011951
aacagtagtgaatgtagtagatattaatccaagtatataaataatcaatttaaaactcagtggtc
taaatacactaattgaaagacagattgtcagagcagatcagaaaagtgtgtacgtcgtgtataag
aaatccactttaaattaaatacagaaacagattaaaaccaaaggaatgaaaaaagttgtatcatg
caaac

>chr4_125923150_125923350
ttaacctccatgcaagggtatgttgtcccatttccttatggattattcattctacgtttttgtta
ctgtaatcgacagtgctcctaattttctcacccacaacactcctgtgtaaacctacataatttg
ggaaatacagttaagctgaagctacaagcaaatttgctgatcagaaaaaaaaattctctaaaagt
taaaa

>chr4_134655150_134655350
gttagttacatatgtatacatgtgacatgctggtgcgctgcacccactaactcgtcatctagcat
taggtatatctcccaatgctatccctccccctcccccaccccaccacagtccccagagtgtga
tattccccttcctgtgtccatgtgatctcattgttcaattcccacctatgagtgagaatatgcgg
tgttt

>chr4_141905150_141905350
cccaaagtgctgagattacaggtgtcagccccgtgcccagccgtgtgactgcattttgactgtga
cctgtcacatgaggtcagatgtagaattttccacttgcggtgttatgtccgtgttcaaaaatttt
aaattttgaatcattttgcattttggattttcagattagggacactcaacctgtattaatatctt
gagga

>chr4_144049150_144049350
aatggaatatatttactatgttgttctttacagaaaaggtttgccaaaccctgctctagaaatac
tggcttggcaaaactttatgaccatatccacaataaaaaatagtcttcataatgtggtcctcttt
acataatcatatatatttatataatataatctgaaacaaatgtttcatgaaataatacctctta
ctaca

>chr4_150383150_150383350
aaaatcaggtgcttttacaggctttatgtattacatatatgttattacctctatcaatatactgt
acttaaaaaggttttatatgtaatacatattgagtgatctatgaattatttggaaatttgttaat
gttctgtatctgtgctgcacaaatgcaggtcattatatagtaggtctggagtggagcctgagatt
ctgca

>chr4_161271950_161272150
ttctggctttaatgcttatcaaatttgttatcaaccaccataaaaataatgttggcattattaac
aaagtaccaataaaataactcaaatggtgaagtgtgtgtatgtatgggggtgagattacttgcat
aaattcacacagatgacaggagtgggtgtccttagtttagcaatgtacaattccagtagcagact
aagta

>chr4_172056025_172056225
agaaaagtgtatatgggactggagaggcatcacataaagtttggcaaaggagagtttatcaatat
aaaaaccttctgtaatatagaatttaacatccaacctagcaagaaaatctagaagactgcactta
aaaatatgttagaatgactattataagcttggagaattttccagcccatagtatatgatgcagaa
atacc

>chr4_176153012_176153212
agatatataaaacctctgtcattcctcatacatcgtttttccttattgaaaagttggttaaaaac
acattttaatgcttttgggaaagagagaaagagaaaagctccagctataatttgacagagaaggg
attgtaggaaacagacataactgatacagagtttttgttgtgggattgtaaggtataaaaataat
ttgtt

>chr4_180723206_180723406
tctattacagagactgttgttaaccttccattcccaacttgaacttacttaatggcgttgccaac
cagcagcggcacccctgagagcctccagcaaaataaaagctaacctaagtaacatcttaggtgtt
aatgtaggtttccaagaagaaaggtaatctgatcttttaacattctgtttctacttgcaaagtgt
agcac

>chr4_189259406_189259606

tttttctttttgtcagaaactgatgaatgctaattagtggtcattattgaggaataagtaaaata
tgcatttaattactcattcattcactcagcaaaatcaatgatcacttactactcaggtactggga
ttatggagataataaaacagctgattcctttcaagcatgtaccacggagtgaatgaaatggacac
ccaaa

>chr3_3348200_3348400
tgaagataaatggctcatctcccactttctttggttgaggtctctattctgcacaaaggccctgg
caaatctcttcatttttcttgtagctctatctgctctccagtgcaggggcatatatcacagccac
ctgtctgctggctgtgagacacctctacagaaacagacagcttaatgcaaaagagaaaagaccag
tctgg

>chr3_3686600_3686800
agacagaggaactgcagtggagaaagagtttaatacccacagagccggctaaaggggagactaga
gttttattattactcagctcagccttcctgaaaatgtggaggctaggagttttcagagagtttgg
caggtggggagctaggaaatgggaaacgccaattaattaggtcagggatgaaatcataggggggtt
ggagc

>chr3_7903000_7903200
tctctaatttgatgatcactcgaacccagattctggtgcttaccctgggaaaataacttagaccc
ctcaaaaatgctgttaacatgtttatgaaggaaaagtaaagtgttgtggtaacaatgtactgttc
gatggagaatgaatgttttggcgacaagaacaaaatgtccagaatacagactgttcactgagagt
aagca

>chr3_28996596_28996796
tgttttataataaacaggaagagtattatccattgtataggtactttaaaataggtgctttaaaa
tagttcttgattgctttcctaatctccttacacatatagagacctatgctgtgttagctgtataa
tctgaaagcaagagatcatttggtagaaacaaaatgaaatggcttaatgttggcagggtgttaac
tttca

>chr3_33393196_33393396
aaatggtaccgccagtttagaatacagcttggcagtttcttacaaagctaaccataaatgtatca
tttagtcaagcggtcgtatatttaagtcacaaaactggttttgtggatgttcgtaaaacctgctt
ggaaaagcaagctaggtgcagtggcttgtgcctataatcccagcaacacaggaggctgaagcaag
aggat

>chr3_34684596_34684796
tgataaaaataatatataaaaattacattttagaaattacctaattatttttcttacatattctt
ataaaatcataaaaataatatgaagcttatttctactttataggtcagaaaatagaggctttgct
aacaataacaaatgtcagtaaaatccaaaactgctgttttaaaaagatcaatacatttggaaaac
tgcta

>chr3_57721160_57721360
gatgtatgcattatcaaatgattaaatcaagctaattaacacatctatcacctcacattttcatc
attttttgtggtgtgaacatttaaaatttactctcagcaattttgaaatatacattacattacta
ttaattatagtcaccatgttgtgcaatagatcctaaaaacttattcctcctgtctagccaaaacg
ttcta

>chr3_74129910_74130110
gttctctcaggacagaaagccatctaccaacagaaatgggacaaacctgattcattttcttaata
atctttaaatctacggcatgttcttgggcccagaggtggagttaagttaagcacaatttttaaac
aaatgcattctgtattctgaagaggtgagctgcaggaaagctatttaagctgccaccatggctgc
attgt

>chr3_82326110_82326310
ccagttgatcccaaggcacataactgcgtatctattgctgtgttacaaacaatcccaaaattaat
aggtcatttcaataatattaacttatttttctttacaagtctgcaattctgacaaggcacaatgg
agaaagcttatttctgtatgatgtagcatagagtgactcaaataaactcccgtggcttgaggatc
tattt

>chr3_83208510_83208710
agttaaagctacagattacgtgatccactattatttatattaatagatctttattaatataaaca
cctattattagatctttatactttcattccagatatttatcagtctaaaattttaattaaaatat
tcacagtctgcatgcttcctacaacatactgtgttttagggaggtaatacataaatacattaata

tatga

>chr3_85559110_85559310
aatttagaatatgccctctcacaagaatctaggacagtctacaagtaataaacaaaaaaataaag
caatgtgggctcactgcccagatgatctcaaatatgtataatggaacaaagaaagaaaagaaata
cttataagcaaaatcactccatgaaggaagaaaaaatggaatagtaattggtcaaaattgtggta
gaaaa

>chr3_86857710_86857910
tactatgagaaactaaatgccaataaattagaaaaactggagaaataataaattcttatacacat
acaatccaccaagattgaactatgaaaaaatccaaaatgtaaacagcccaataacacataacaaa
atggaaaccacaacagtttcccagtaaagaaaagcccaggacttgatagattcactgctgaattc
cacca

>chr3_88468310_88468510
tgtaataataatagacataaagtgcacaataaatgtaatgtgcttgaatcatcctgaaactattc
tcccaccccctggtctgtggaaaaattgtcttctacaaaacttgtccctggtgccaaaagggcac
ctttattcccttttttgtgtccatgtgtactcaatgtttagctcccacttatgagaacatgcaat
atttg

>chr3_94588310_94588510
tgtagactgcccttcccaaattctgttgttatttggaaaaatgtgtttatcagcaggaaatcaat
tcaggctgcatgagttacaagctaatgttaattgatcctgctttttgttatctaaactgtgcaat
ttttttcctatgctggagaaatatataagaatacatccagaacactcattttacaaatatacaa
atata

>chr3_105698910_105699110
tgaaagaactgccgtttaaattttgactcatcaggcttatcgctcattctttcattacattttt
tcttgaaatctcatcaaagattagtgtaatcttatctaaagggtcacataacaaaggaaatagta
ataaccaacatttctaaagttggatgtttaggattaccaagcactgtaatacatactatttcctt
tgatc

>chr3_106340510_106340710
tagtaaagaaaccttgaatacaccaccttaaccatttattaagttaatatataatgggtcaagaaga
cctcgtatggctcctgataacctgctctgagaaggaggaaacatcagttccataagattcctgtc
taaaaggcatcgtctgagcctaactcaggcctatgttgtatgaagaaacatcacacaaacctcaa
ttgag

>chr3_116974510_116974710
ctcctttttttgtgagatttttttactcagctctagccaagggtctggatcctgagcatttctctt
ccttcccacttttcttccttaaatccttattttctgcttccattttctctcagtattcattttac
tcagcaaaaaggtgaatctaaaccataagacctaaagactcaaatgggtgcttttcatggttact
ctgtt

>chr3_138707110_138707310
agagggcccttggaagtcttccagtctgacctgcccatctgacagatggggataatgaggtccca
ggaggagaagggatttgcccagagtgaagcagctaaaggaagcacgatggctagttacttctatt
gttccagggtctgtgtgcctaagacttacttcttctccagccagaaagccttccttggaagctga
catca

>chr3_154433306_154433506
ttgaattttatctttttattctttaaagttttatttttatacatagaaatagctcctataa
tcccacagatttaaaatatatcttctgttatagaaaatttgcaaagtatagaaaaaactggcctt
acaaccaattttcaaataaagttggacttaatttttccattcttaaaagacagcatggtgtctat
ggata

>chr3_176331306_176331506
aacagtggaacaacaaggatatggaacttatatccagcaacttttttttgtccagaacaactagt
tcggagtttgcctatgagtcagtaatgtattttatggtgattactaatattttcttaggagttca
ataaatatttggacaaaaaacagacaaaagttacattaacttacctaaagtacagaactaaataa
aatat

>chr3_178129306_178129506

ggcctcttcctttgagaaactacacaaaacttccattactcatataatttgtgaaactcctattt
tggaattgcctttgagagcctgtagcaaattcttttgaataacttcagaggtagtaaattacaat
cttctgaggtgcacattttgaaatagtcaaaagtcattcaaagcaacttctgaaaagtaagatta
ggaga

>chr2_2343593_2343793
gatgcttcacagaggaaggtcacaccctgagagcctgggcaggtggagaaggcttctccggtgag
tgcaagaatggaggaggcgctggagctgttctgagggaacccccctcagcggcctggacattagaa
atggcctcagccctctgtcctcatcttccactcctgagcattcggcaagatgccagtatcttgtt
aataa

>chr2_4589125_4589325
ccttaaggtcaatggaagagacaagaaaaatcaataagatacataagaattggtgaggaagaaat
aaagctattttaatatgccgttgatgtaagagttttacctggaaaattctaaataatccataata
aaaataactcaataaaagttactatggtagcatgatataaaattactatacagaaattaaaagct
tcata

>chr2_7437349_7437549
tggtaatactaagagatgccctaatggatctcctgtattccacacatactcttccttatctccat
tgtggagtagtagattgatttcatcttgatattgcaggtcaatcaccccagccaacattgtaact
cccttcttcgcctgttttcttagaggcaggaggagcccaaagtgtccaggtggcaatcttaactt
ccagt

>chr2_19881919_19882119
ataccaagcacaattgctggatcatatgctaagaatatgtttacttctgtaagaaactgccaaag
tatctttcaaagtggctgtaccatttttgcacagcaaccagcaatgagagacttcctgttgctcc
acatcctcaccagcattcgatgctctcagtgttttggattttgaccactctgacctgtgtgtggt
ggtat

>chr2_22172095_22172295
ccagattgtaggctaaggactgtaaatatcctctgcagtgaaatgtgtctgttgggtcatggagg
acccaactttttcagaaggaagtcagactgcacatttaatactacagctttcttctgtctctgc
ctgggatttatttctcatccattttccaatcttgtttctctaaattcaaatagtttctcagttca
aacct

>chr2_34510096_34510296
ggtgaacatgtgtcatgggggtttgttgtacagattatttcgtcacccaggtattaagcctagta
cccattagttatttttcctgatcctctccctcctcccaccctccaccctccaataggccgcagta
tgtgttattcccctctatgtgtccatgtgttctcgtcatttagctcctacttataagcgagctat
acatg

>chr2_57966096_57966296
aaattcaaaaattgtttttaaagtcattgtaattaaagagataaagcttggtgggtagtaagtgg
gataagtgctcaaaaatggtatcatgattatcattttctagataaatcataattagaattttttt
ctaattttggagtcttctcatagacttcttaggtcaataaggtataatttactcaaagatatccc
caaat

>chr2_104108368_104108568
tggcattatgcaagagggaatgtaaaaatgaatggcccgtgcttagtgataagattagttcattg
attcaggatccatcaagtatccacctagcagatccatcccttacaaattttcaaacaagaatttc
tctaatggttttaggatatgtggattatcactacctagacttatgcattaagaatagcaaaatgg
tgaat

>chr2_116617730_116617930
tatagttttaataagttttgaaattggaaagtctgagttctccaagtctgttcctttttcatgat
tcttttggctattctgactggatcctctgaatttttcatatggaacttaagattagattatcagt
ttctacacaaaggcagatggaactttgacaaatattatattttgaattaataggtgaaatagact
aactg

>chr2_126961530_126961730
gcggaggttgcagtgagccgagatcacaccactgcactccaatctgggtgacagagcgagactgt
ctccaaaagagaataaactaatagcttccagggcttagggatggtggaggggaaggaagtgggtg
taactataaagcggtagaaggagaaagatccttgtgggatggaatgttctgtatgctgattgggg

cagtg

>chr2_129960730_129960930
ctttacagtggagaaacccacaaacaccacctcagccagtgatcaaggttaacatcaaagtgttg
ttatgtggataaacaggtactcttgatgtgaggtgagaatggtactttaccactgtgattttcct
tccaaaaacccatgtccacaatttcatcatgagaaaaatatcagaaaaatcccaattggaaggaa
attct

>chr2_140810930_140811130
aggtaaatttcattttgtattcataattgttgaaatgtgtattgggtaactaagagttcactcta
ctattcttcctacttttgtgtaaaatgaaatcttttcataacagaaaaggtaaaacataggtaaa
aagttaaaatgaaagaacaacttcctacctgcagatatattgctagatctgagattaataccacc
ataaa

>chr2_142481330_142481530
aattatatttcatgacctagtgtctcttctcaaattatcatcaccaggccatttcagaaatgtcg
taaaatataagtgaacactaaaatcaaaaggaaaaatatatatatatatattcttactcaatcca
cttaaaattcaccaaaaatgtcaacactaatcaggccaagtgatgcaataagtttatcttacttt
gtaat

>chr2_143241930_143242130
ttggtcccagatttcaagtaaacattctcataatccacagttgaaacacattgccttgtacagag
accacatgcaaaggaaaaattgtttttctaatttttattgtcttttggtgggggatggaaggacc
tgcacctttcttagtattgttggaattatctaaactaatttaccaacttcttaaaagtaagctaa
ggaga

>chr2_153878554_153878754
ctgtgatttggggatagagatgtgagtgggtaatgaactgaaaaggaacagggcagaaaaagtta
aaattcacaattttgcataatgtctgcctcactggaacacatctaatgtttgattttatgtatta
aagatgcttcaataattgactaaattttatggagtttttgtctggaggttttggtaatgatgtac
cttgt

>chr2_154860954_154861154
gatctgcctgcttcggcctcccaaagtgctgggattacagatgtgagccaccgcacccagccagt
gctaaatttaatacattgaaggagagattcatttctgacagaacatggaacttaaaatgaatatg
agtcagcctttcttactacatattgtagattgatctcttagtgttttgatcgacagctagtatct
cagtc

>chr2_164377354_164377554
gccagagaattctttgacatgccacgtattgataaatgcagtatatgttccctccccttgaggtc
agtaactatttgacccataagtgacactgtgctcgtttccagtaaaagaacttaagaaactggca
gctgctacttcctgattctcttgggatacacgatcttgaaacctaggaaccatgctgtgaggaag
cccaa

>chr2_167314554_167314754
tgagcaacctatttaggattggttaaatattttaccactgaagtacctgttatttattgtagtgc
attcagcagtcaggaataggtattataaagtacattgcaaatcccatctctactaaaaatacaaa
aaaaatagctggatgtggtggcatttgcctgtagtcccagctactcaggaggctgaggtgggagg
atcac

>chr2_195852955_195853155
ccaaatatgttttaagcaaattatattaaacatgattctcaatgcctccaaatgctttatctgta
ttataaacttattgtaactgtatatattgaaaaaaatccagtatgcatgtttcctagattgataa
aataagtcatagagggcagggacttgaccatagaatttctaagaaattcaaaattgtctaaatct
ttgta

>chr2_222586356_222586556
tacccagtagagacatttttcataatggttgtactactttacattcccgccagtagtatatgagc
attccagtttctctgcattcttacgagcaattgttatttttgtgtctttttttacaatagccatct
actgggataagatgatatttcattgtggttttgatttgcgtttccctgatgattagtgatgttga
ctatt

>chr2_222971556_222971756

tttagtagaaacagggtttcaccatgttagccaggctggtctcgaactcctgacctcaggtgatc
tgctcgccttgacctcccaaagtgctgggattacatgcgtgagccactgagcctggtctatatta
attcttatgggaagaaaatccaagtgcataagtctactcactataagctatattttcctggaatg
tgaac

>chr2_224382756_224382956
tgggagggacttatattcgcttggctattggccaaatgaaaatacatgtgaaagatctttgtaaa
ctgaagaatgctgtaatatcatcattattggaatgtcaatgttttgggaatcattggcacgagaa
ttacgtgtaaagctttttgctgatacacggtccttgagtttcttgaactcttgataaactcgtct
ccatc

>chr1_48422613_48422813
gggaggcacctcccactaggggctgactgacacctcatacaaccgggtgcccctctgagacaaag
cttccagaggaaggatcaggcggcaacatttgccattctgcaagatatgctgttctgtagcctct
gctggtgacacccaggcaaacagtgtctggagtggacctccagcaaactccaacagacctgcagc
tgaag

>chr1_56119412_56119612
aggctggtctcaaactcctggcctcaagtaatccacctgctttgacctctcaaaatgctgggatt
ataggcatgtgccattataaccggccaagaattagttttgaaagatgaagcaaaactctttaggt
gaggaatcagggtaactttagggaatataataccataagcaaatacatgaaggtgtgaacatgta
tgagt

>chr1_88616812_88617012
cctgattgaatcctggatgggaagacaatctacccacattcccagtgacaaaacttcactgaatc
caagtgtactaattcctactctgaagattatttccaaatcgggagcaacgttttaggaaatattg
gcaacaacaattaaacctatgatgctacgttgactatgatttgatagaactccttcatactcatc
ctcaa

>chr1_90677412_90677612
ttttgttctcttgatgaatagacccctttatcattacgaaacgtcccttttaatttctttactat
gtttgatactaatacagttactctaccttccttccttcattcctctccaccctctctctttttttt
ttaatgtttagtttacacagtctgtcatttgctgtctttttatttttagcctttttgtgtcttta
gaagc

>chr1_99551212_99551412
ttataaccaaggcaactaaatttctttccattatctctatgtgttgggataacttatattgttgc
cctggaaatgagtaatatctgtctaatggtgccaaatgggaaaactgtatccaaattagtgcaag
aggccataaaactgtcacactctgctcgaaatcatttgtatctatgtgacaaatttccaggagtt
aggtc

>chr1_99944412_99944612
ccaaaaagacacatgcactcttatgttcatcaatgcactatttataatagcaaagacatagaatc
aacctaggtgtccaacaatggtggattggataaagaaaatgtggtacatgtacaccatggaatat
tatgcagccataaaaaggaatgaaatcatttcctctgcagcaccatggatggagctaaaggccat
aatcc

>chr1_104870277_104870477
aaattattttttactatacatgcaaataaatgatatattattttcctttttttttttgctttact
aaaaatatatgtggacatttaagacaaaatccttgatcattatttttaagacacataaatgtttc
tgttgtgtatctataatttattccttttccttgttatatagattccattgtgagaataattattc
ttatt

>chr1_118855077_118855277
attttgaagctgttttagcttgggaaaaaacatccattttaatattgtcattatctcatgttggc
ataaaacttattgatcaaataactgtcaatgtgctagacattttgtaaattatttctgatccaat
ttgcagatgaggactaaagcttcagaataacttctgatggaagtattataaataccattgtcttc
agaat

>chr1_154073576_154073776
ggcagaggttgccgtgagccgagattgcgccattgcactccagcctgagcaacaagagtgaaact
ctgtctcaaaaaaaaaaaaaaaaaaaaatttggctgggggcggtggctcatgcctgtaatcccag
cactttgggaggctaaagcaggtggatcacctgaggtcagttcgagaccagcctggccaatatgg

tgaaa

>chr1_169468176_169468376
gggtacacaaagcctaatcatatcagataacaaagttcatgttgtgctcacagaaatcagctctt
cacatacagtggaatgcaaagttaatttgttaataatggatagaaaagaaagaataattttgaca
acactaagcttttttttttttttttttttgagatggagtttcactttgttgcccaggctagagtg
aagtg

>chr1_179908377_179908577
aatcctagttacctttcccaaccactatgaaccaataactcaaacaaggtggtgatttagctgtt
tgtcctatagttccactaaatcttcaagatgtatcctatacttcaaaaaaagatacataccttaa
aattagaattcctgctaatactaagtccacagggattaaattcgttattctagagtcacagggca
agtca

>chr1_186500377_186500577
ggctttttcagttttcagtgtttttacactgattttttctcatctttgtgaacttatctacctt
tgatctttgaggttgctgacctttgaatggggtttttgtggggtctttttttgttgatgttgttgt
agtagttttccatttgtttgctttttatttttttaacagtcaggccactcttctgtagggctgct
gcagc

>chr1_188030977_188031177
tctgtgcagaatttctttagttaatgaggtgccatttgtcagtttttgtgtttttttccaattgct
tttggagtttttgtcatggagcctttgccaagttctacatccagaatggtatctcttaggttatc
ttccagggtttttatagtgttaggttttacatttatctttaattcatcttgagtcaatgtttgtt
ttggt

>chr1_194391977_194392177
gttgacagagaggaaagccattcagctccagggtcccagtaaaaagttggttgactctgtagcca
tgatcagaactctcaaagtcatgtcacctatgtgagacttgcccatctgtcctatctatcctgac
gcttgcctcctgggtcctaacacctgtcagacaaacttcctcctgcctctcttctctgaggctag
tccca

>chr1_194978377_194978577
tatatgatgaagggaaataactgtttgcaaaacaaatttctttctatatgcacactttgagagt
aataaagcaactatgaaaactaaaaatatgaggaccttaggtcattaaaaaatgctgtgacaata
acataaacacatgttttcaataacccaaaattacaggcactatttcaaaatcaactttattaatg
tttca

>chr1_197426177_197426377
tcattgttcttcgaccctaaaaccctcatttcattctcctttgcctagtcaaatacatttgttct
cataatgttccagtaaagatttccttgcttccctcccccacacaatcagcatttggagtgtcttg
ttgccatacaatcctaagtacgcctctatcatagcatcgtataataatactaatagtggctaata
tttgt

>chr1_215271377_215271577
ttgtttttataatccatgatcatgtctatcttgggtctttgtcttcctagtagttttgtttttta
atctgttccttggcagttatagtgtcccttgcctatttgagttgtcctttgcagacatacattct
gcttagttaagactttgataagtgttcattaaaactgtgggcagtattccagaagaggagaatca
taatt

>chr1_216709177_216709377
tctctgttcactgcatcccttcaccacgaaaattcctgacaaaatggtgtacatgcactttgca
ctaaattcatctccattccactaaagtctggattgcatctcagctctatcatactgtaatcacca
atgacctaattgccccatcccatggcctgttcagatccgatttgatctctctggactattttgtc
cagac

>chr1_217332577_217332777
gaatgagccagcatgcccagcctaaatctgtttcttataagtatcatcatatgaggttttatctt
tgctccttacatagtagtgttcactttttttgttttccaccaaaggagtcaccaagggaaaccaga
tgaaactgtgttcattctttattaactttaagcactttaaatgcccctacttcccatacatactg
aagca

>chr1_219663977_219664177

gcatgacttcatactataaaatcataaatattaaaaatctaatctttattgaatgcttactatgt
gccaaatgactgctttctatgctttttctcatttacttcttctatttttattacataatcctg
tccattttacaaatacagaaactgaactttaggaagagaaaatagcttacataagtgaactagtg
ggtgg

>chr1_239048177_239048377
acagatccagtgataattttctcattcctcactttcctcagtgtggcaggcagacttttcacagg
ccaattctggagtgttttattggggttaaatgctgaagatcctacctggagctacttctctaact
tttccaatgtatttgtctaaggctactatctgctatattaaatacattttatctttttggaaat
gttat

>chr1_248392777_248392977
agcagtgggcgtgtcccagcaaagcagtagcggaaggcttcagctgggggagctataggtcagct
ggtgcatgtctgtagggccagtccgcaggagctctctgacaggagcagtctgtggcaaggtggtg
acgatgaaagcccccaggaagcacatatgttgggcatccaaagctgtgctgcaagcaggcatggc
caggc

>chr9_28178200_28178400
aagctacagatacccgcagctcccacagtgttgtgataaaatttaaattgtgacttagagacggt
agcgtatacattatattaaaatgtctgggctctcacatgtaggaagaggaagcttgaaaccgtag
tttacaatctcagtaaacttggaacatgaaatgacttatataaatgaaatatacattcaaaatat
acatc

>chr9_31418600_31418800
acacacaaggttgcttaaattcgctttcgtttttggttaaaccacgaaaacagtgagctgaagta
ggccaacttttatggaatgtaaggatctacattaactttcagttgtaatatttatgttgcatct
accctcaaaaataggtagtaatgagaaaataatatatttattaaacaaacatgccagggatatga
gtaac

>chr9_43183604_43183804
ttagggactttcagtaatgcaccattcatttgggacagtgatgtgacaattctcaggcaaatgtg
gaagtagatgtctatgtcatttgtgaatatgtagaaataaagagttcatttacatcaactgtccc
acctctgtcccatacctctccttccccatgaagaaaagagaggcagaagtagaatttaagtagaa
tttaa

>chr9_45976804_45977004
ggaaaaaaaaaaatgagttttacagaagtttttcagcagaagttgattggttttacacacacaca
cacacatacatgcacacatattttcacacatataaatatgtatattttacatatatatatgcaca
cgcatttgtttctatttaaaatgaaaaaagtatgttagtcatgtgtgcccaattttctgatacac
taaaa

>chr9_72044780_72044980
tgaaacaaaacaaaagcaaagccaaatcacacctcacatgaacacaaaatgctctttggaccaag
tgaaaactggctcagaattataatattactaaaacatctacactgaactgagacggaagcataaa
tatgaggcacctgatgaaaacacggaacaattccacaccagccaaaaatgctctcaggcccacaa
ggcag

>chr9_77268780_77268980
aaatcagagctgcagaaggtcacttgctattttagtaactgcaagctggtcctcattttataaca
tcacagttataaacaagacatcgtcaccttgcttgtcagtttacttctttttcaccaaaataagc
agatgtagccctttttctttctacatagaattaggtaatttggatgacttgtttccaaactttgc
ttaac

>chr9_90440980_90441180
gggtagacttcagggcatctgtaaaccctctaaacatgtaatgtgtatgtaacatgtatgtttat
ctgcatttttctgggaagtgtgttgataaatttcattagattccttatggggtctatgatcccta
atcagatgctgaagctaaaagtcagacgcagcctgtggcaaataaaacacacagatggctcatgg
tgaat

>chr9_96553379_96553579
tgaattattcactgctgatgtttgtgttttgatcttgtaccctgcaacttttcttaatttattag
ctctagtagctttcttcttaattctttgggattttctacatatagaatgatgccacttgtgaata
caggtagctttatttcttttttttttttttccaattttgaaatcttttagttctttttcttatct

aatag

>chr9_103754579_103754779
gctagaaaaataactgccctaaatgggagcaacattactttttagactttgtcatagatctggga
ttgggaattagatgttgtgtcaggaccttaattctcaaatcatgaccaaatatgagaagggccag
caacttacctaaatccaagtttcaaccccagataatcttatatccccatgaagaggggacaattt
tcatg

>chr9_109147779_109147979
agttcactaattcagttttggaaatttaaaccaaaatattaaaattaagaagataataaatctct
aacctccatatgttcagtcttttcacctcccaattaatctttttcatgttgatataatgctcctt
ctgaaatataactgaaattcagtagcttccctaaacgaaaccttcaatgactttataccatttca
taaat

>chr9_122080379_122080579
taagcctttaactggaaatgatctttagaaaactagctactctcgctcagctgcatgactgtgga
cagtcatgtcccctctcaagacctcagatttttatgtcttccatgtgaaaatgtggtgaggacac
aaacaatttgtcaactgtaaacccatctatatacatgattaaatatgacacctcatttgtctact
tttcc

>chr9_122377579_122377779
ttccctggacttaacaagggagaatatgatttccattaatatcagtggttcttaaacaggtggaa
tgttgtgaatcccttttaaaatatgaagcaccattatagattatgtcttccactaaatgcacaaa
acataaaacttagtacataattttaactattttaaagaatgccttcatgactcttctctataaat
cccac

>chr8_31749858_31750058
tttttgtttcatttctgttttagtaacctcttagaaaaaggtagtattaaaggagacctaggtct
cttttaccaatttaggataacttcacaatctccgcagataaattggtatctgtgtacacacacaa
aaataggaaattcttgtgagtagtttacaggggaagtgcttaatatataaacaaggcagtgctct
ggtaa

>chr8_34610858_34611058
aaaaaataggttgtttttcccagctatctcttatctccagtagcttcctttacttaaatcctttg
ctgtgagactttgccacgcctgccaacacataaagtatatgcccaggtgccgttgactttgggct
tgcccatgtggattacttagaccagtagaatatggttggaggtgacaatgtatcagcttggagtg
gaaga

>chr8_65245446_65245646
atttctgtttctctcaccaccattctcccagagctataaaaagggaaaccttcagagtcatcttt
tatttctttctttccattcctttctcccctccctcctgcactaaatcagtttcaaagttataggc
atgctatcctacatccatctcttcctctccattccaaactctgtaacatattcaggccttcactg
tccac

>chr8_65555646_65555846
tatatatacacatatatatacatatatatacatatatacatatatatacatatatacatatatac
atatatacacatatatacatatatatacatatatatatacacacacacacacacacacatataca
agcataccttgaagatactgcaggttcagttccagaccaccatcataatgtgaatgttgcaataa
agcaa

>chr8_74959446_74959646
tttctacataaaaagatttggtcctgaatttgtgaaagagcccgttttctgcactagagggacta
tttcttccttaatatgctttctggcacagtagcacagactccaacatcatctgacttccttgcac
agccaagaaggtgtggtggagtctgtttctctcactgtgcttctgtccccacaacttatgggtag
acgtg

>chr8_75997245_75997445
tgtcccaaatagagactgtttgagtttatagtagtcatctcagaaggagtggctcacgaatctcc
acttaagtttgtgacttggcttgaagcttggaaagaaaatgtgcctactttgggagcattcaggc
aatgggatcttggaaccaatttaattgcaactttcctcacacaaatatataactgttctttgcac
ataaa

>chr8_76461445_76461645

ttgatatatttctctttttacactaacagcatagtagcaagattaattagatggaaacaaatag
aagcatagttttatgtcaaaaatgatagcactgagattataaatgtttacaatatcttttagcat
ttttctaaagaataatcatgtaagtgaaattcataaacgcttagctaacatcttaatttttatc
tctgc

>chr8_76994645_76994845
cctcttaataccaccacaggggaagattaagtttgaacataaattttggagaggacacatattca
aaccatactattactcccccggcccccacaaattcatgttcttctcacattaaaaatgcatttat
tccatcccaataaccccaaaagtctaaacttacctagcatcaactttacagtctaagtccaaagt
ttcat

>chr8_91110224_91110424
ggtctgtgaagctctcagtgcagagggtagctcctctctgcctgcaggtcgtctctgcagctctc
ggcggagagggtgctcttctctgcagttagtcgtcccattgtctccagctatcagcagagagggt
acgtctcattgaagctggtcacccagtcccatgaactctctctactctctgggtcctctggccat
actct

>chr8_94040424_94040624
ttttaaatatccccagtaaagtccagatacacatatttcacctgtttaaagagttttgcaggagt
gttcaggtgcttgttggtaatttcattctagatctcagtatcttaactggaggaaggaatagcag
agaaaatacagtacaaaaagcaagactcaacttctgcacacaaagagaatacaaagaattgtctt
aggta

>chr8_94179624_94179824
tgaggacctactttgtgctaggaactttatgtaaatgtgttcttttagtctttatcactctgtaa
tatgttagtgtactcatttatcggtgtaattctatgtatataactcaataaactgataatacaaa
aagaacttttaaattggtatgcccacctcaccaaagctctttttatttagtcatgaaatttcata
actaa

>chr8_112761024_112761224
actatactttacttagctccttttggactgctgttggcttcaagtaaccagaatcacacaatcaa
aactgcagataagagggaactactgtaatcacctatttctctctctctctctctcacacacac
acacacacacacacacacacacaagatgtatcattaggtgacaagaaaacagctataaagagaat
aacat

>chr8_114757624_114757824
catgtatgcattttatttaaagataaatcttatttaaaaataaacttcttgcaatgctaatttat
ttagaatttctatctaagaaaccactatgtagctaggccattttaggctatagagtcatagatat
atcagggttcagtgatttttaatttaattctagggatttattttacaaacacatttttgacattc
ctaag

>chr8_134904418_134904618
aactctcatattaaataatcgttatgctgaatacagagctctaggctaccatacaagtgctcaaa
aatggatgcctactatttattgtttatattagattgttaatcatttctcctcagcaccttcctga
tgatacaccatctttcatgacatctgttgttggggaaaaaaggtctactgaacgtataattgcca
tccct

>chr8_137170818_137171018
aaggacaagcttccatccagataatccaaagggcgaggtcaataaaaaaaagcccaatcagaacc
caagaagtcagagtagtcagagtgaaggaaaaggaaatggagtggccaagagaagagccatagac
tagatttggatttatgctgtcctcattgcagtctttgattagctgtgatggcagtgaaatgggaa
tgttc
