# Supplementary material for: A machine learning technique for identifying DNA enhancer regions utilizing CIS-regulatory element patterns
Source: Sci Rep. 2022 Sep 7;12:15183. doi: 10.1038/s41598-022-19099-3 (PMC9452539; doi:10.1038/s41598-022-19099-3)
Supplement: Supplementary file 2 — Supplementary Information 2. [file 41598_2022_19099_MOESM2_ESM.pdf]

Online Supporting Information S2: The following are the sequences of **1,484** DNA Enhancer sites in the training dataset out of which **742** are DNA Strong Enhancer sites and **742** are DNA Weak Enhancer sites. See the text of the paper for further explanation.

I. 742 DNA Strong Enhancer Sites

```
>chrx_48897056_48897256
cacaatgtagaagcagagacacaggaaccaggcttggtgatggctctcaggggtcacagtctgat
ggggacacactggaggtcagtctggtggggagttttagcctttggtccttatggtgaagccta
gatttgagcctgttcacatattaagtggagatgctattgttcagctctgcaaggggggtttgtc
ctatt

>chr12_6444339_6444539
gccctcacattccctggcccatcccctccacctcaaatttacaaacacccggggttggcaagga
caaatggggaggagagtaaaagggaaaggttggggatcctgcatgggtagacgggggacaaagtt
gagacgggggcaggagtgggcaaaggtgtgcaggacataggcaaatagagctggccaaggctagc
gaccc

>chr12_6444939_6445139
gagcaggaggccagtcaccctgagtcagccacggggagacgctgcagctgtaagctgtgcaggcc
cggaagttcaagcctcagccgcaaacaggggaagccacacacagaaacacaacagcagcccccgc
aggtggaggcgtgtgcccctgctcagaggacaacaaccttcccctgcccgcaccagccaacaccg
tgcca

>chr12_6445139_6445339
cctctgctgagaacaggactggggcttccagggcaacaggaagggtttggggtggtgatgggaag
aacttcccagtcctgaaagtgtggttcctttccccgaagaagccactggagcacctaaggtcact
ggtactttgaatcccatccaggggggcttgaggcagagtcatgatgaagaactgggggacgccagc
cccag

>chr12_6445339_6445539
acagccttaaagggagcttttcagggacctctggccagtgggggatgagcctccctggagggagg
gagggagagaggctttctcaggccacgtgttcccttctctttccagcactctggggggttaagtca
gatccgggaaagggaaatcttttcccagcaactccgccccagtgactcaggccagagggagggag
accta

>chr12_6641339_6641539
tgcagtgcctaggctccagatcagtgcttctgaaccgggggcaattttgtctgccagaggacatc
tgacaacacctggggcctgttttgttgtcatagcctataggggaagaatgctaccagcatttgtg
ggaagaggccagggatgtggctcaacatcctgcagtgcacaggatggcccctcaacaaagaatca
cacgg

>chr12_6645339_6645539
gtggcatagtggggtggtgaataccatgtacaaagcttgtgcccagactgtgggtggcagtgccc
cacatggccgcttctcctggaagggcttcgtatgactgggggtgttgggcagccctggagccttc
agttgcagccatgccttaagccaggccagcctggcagggaagctcaagggagataaaattcaacc
tcttg

>chr12_7052339_7052539
cctgtcgtcccagcgacttgggaggttgaggcaggagaatcgcttgaacccgggaggtggaggtt
atggtgagccaagatccccccagctgcactccagcttggcgacagagcaagactccgtctcaaa
aacaaaaaacaaagccggttatgaagcgggggtggggtgggctagttttaataggtccaggcgat
tagta

>chr12_46780933_46781133
taaaaattttcccatttcatttatactccagtttcagttccgttgattcaaagttttgggactta
ggacaagtttgctagcttctctgagcctcagtttttaaaccgttaaataggaataacagcatgc
tgagtgccaagaattaaagaaaaatttatgtgaaaatatagattagaaagaaaactaatataaat
gcagg
```

>chr12_53772933_53773133
actctgcatattggtcagctcagtattaacttattcggtgagtgctgtcaccagatctcgtcccg
cctgcattcccagggcttgcagcgacattgaggcatctgcccgcctgtccgaccacccgggaggg
gggtaagatttgagaggtactttataggggcagttaaatgaagacgcaaacaagtcctagtgttg
atgcg

>chr12_109085071_109085271
ggcgaagatattcaacttggcaggagcagatgatttgctacatgtggttataaggtggcttctgg
gcaaaaccgtgactccagaaatgtgttcatgacaccttgagtttatttcagagtaatgtgtgttc
agatacctttctttaccggagatacagcagctttcccagactggggttaccatgtttacacactg
agggc

>chr12_109085871_109086071
tggctggcagctcgtgacataatttggaagtcattgttctgcacctggtgccctcccccctgcatc
ctccacccctcccagtcctgtcccccagcttttcacggcccatacacttggcagttgaaggtgct
ccaggcatcagacaatctatttcaggaggcagtgaatgggcatcccacgacatgtcaggacactg
gctgg

>chr12_122884047_122884247
aagaaaatcacatctctacacatcagaccctttccagtccattttgccccatccacaaaatgaaa
atgccactttatttactaaatctaaaaattctgttgggaggcaaaggagagttgaagaatttgca
atgaatttaacctgccaggagagggagtttaacaccaagcttccagaaacaggctgcgtggtagc
tacaa

>chr12_122884847_122885047
aaacaaaaaagtcaactatccaatgtcacggaaaagtaaccaaaaaaaaaaaaattatgggcctg
cttagccccaatttaaaaacatcacatctcctggctgaactgagcttaaggtccttggacgggct
tcatttcccacgtactggctgtttctagagccaggccccatacgctactgccacgcgcagcatac
tgcca

>chr11_6627824_6628024
atgctgccagaaggaaaaggggtggaattaatgaaactggaaggttgtggtgctggtttgaggag
taaagtatgggggccaaagttggctatatgctggatatgaagaggggttaattccttgcaggtc
ttcttgagatagaagtccaggccctgaggtggcaggcagcctgatagtgaacagaacccttgtgc
ccata

>chr11_9587224_9587424
ggcatttttaacctgtgtttcattttcatctgtgaaatgtgaataaaaataactctcttacaga
gttcccgtaaagattaaataagtatgtaaagcatctgggtcagtgcctatcatatagttggcacc
aaaatattctcccctctcccagctcctgcaaaggcaccccagctctttgcagcacttagggcctt
tctca

>chr11_46576424_46576624
atcaattctcaatcaccaaaagcttatccaagtagaagcaaaagacatggctaggatatgtacat
aagacaaatgccggtcaggcatggtgggaggccaaggtgggaaggttgcttgagtccaggaggtc
aagactacagtgagccatgattgcaccgctgcactcttcagcccaggtaatagagtgagaccctg
tctta

>chr11_47428824_47429024
ttgtatgggcccggtggggtgagatggacagaaagcttcagaagacaagctgggccaccagggag
aagttggcggtctaaggaaggccaacagtgcaacatgaagtgggacaggcaccagaggcagccct
gagagctgagtgtgctctcaagagccccagcagaggggaaagtgacagagtaaatggttggagct
gccat

>chr11_61738824_61739024
gccaaggagcacaggaggaaatgaaggtttcggggaaagcccttggggaaggcctgcttggtttt
ggactagatgtgctgagtttccaggcttccgggatgtccaggggggacaaagccagcatgaagctc
tcggacccactggagggaagtcggtctgctgcagggagtaggtttcctgaatcagtgttggctcc
atgct

>chr11_62320824_62321024
ctcccctccccagtggggagcagggagggaggggctgaggaagcggggaacaccacctcctcacc
cctttcttggaatctgcttttctcctgtcctttccaacttcctctgcctttcccccagcttcctg

gggaaagagagaccagcctgggtttccctctctcctctgcttacagttccctaagtccagttgaa
ggaga

>chr11_62321224_62321424
cgggtgcaggaagcctttagatgcctcgctctccctgcccctccacacttggtatgctgtgtcca
ccacccttatccctcagcatttgagggcagcatcctcctgctcagctttggagcagaagtgactc
agtgagtgactgcaaggattgtggaaaagctcggatgcacccttgataagtcacttcaactctct
gggct

>chr11_62647224_62647424
cccaactggcactgtttaggaacaaggagttcctgagagtgttggcaccccacggtgttgatgtc
cggtagttccctgctgtgaaccctgtctctgccccacttcaatccaaggaccctgtggagggaat
tctaggcttcttcaaagcctctgcagtaccagctgcctccaaggcctaacatagtgaagtgatag
ggaga

>chr11_65187024_65187224
gaaaccacagagctgacctggcttcagaacaagatgtggggctccaggcacccgggagaccagtg
accctgattggcaatagccgcaagaaagggcttggagacaggggccaggcgcggtggctacccct
gtaatcgcagcactttgggaggtcaaggcaggtggatcacttgaggtcaggagttggagaccagc
ctggc

>chr11_118783790_118783990
ctctgggaccctcgatgtggggacttccaggcttagggggccttcccatggccctggttcctagt
agctctccacacttcccccgactctgtgccaaaggagaccatgcaaatcagccatttttgcttgg
gaggttttccctgggagtttccagccccaccccacctgcagccctagggtaatgtcagacaaccc
cagct

>chr11_118783990_118784190
ccacaggaccagggaaactcctggtcccagacctttctagtctgggattgttgaacagggtactg
gggggtgatcggcggttttgcccattctggcccactgtcccttctggagcctgcacagacaagtg
gcattgcttggacaggcctgaggtcacctcacttctcttggctcctacagtcattgtcagtgtgg
ccggg

>chr10_74014594_74014794
tttgcataggggcattaccactggacttgggctcagagcaagtgtttatgagataaatgaggtgc
cgtctcagaatcacagagcactcctctacctttgaccttctgcaggctttcactggcttctaaga
gccaagaaccttctccgcccccacccagctctctctcccctgcaatctctccatgaacagcattg
tttta

>chr10_82219420_82219620
ccagctgttgggcctgtgctcctgtacactgcacggccttgagaggagttcggagccctaacatc
caggagagaggccccacagcagtggaaggaaatgggcctctcccgaatctcttgtttgtaccccg
aggtctgagtggtgatcctggggatgctatgggactctcagcagtaggagtgtgtctgtccccag
tctgg

>chr10_103911010_103911210
gggaccacaggtgcatgccactacacctggctaattttaaaaatttttttgtagagatagggggggt
ttcactgtgttgtccaggctggcctcttaactcctgggctcaagggatcctcccacctaggcctt
ccaaagtgttgagataacagttgtgagccactatgcctggccaattttatataggagaggggggt
gtgtt

>chr10_105667810_105668010
cgggaggcgggggttgcagtgagccaagatcacaccactgcactccagcgtgggccacagagcga
gactccatctcaaaaaaaaaaaaaaaaattagcacatgaacatttgtcctagagaatgcgaggtca
ttctggtcttgactgtagccttccaatattctaggtctaagctcgagttgctaccaagtgttctg
tgcac

>chr17_7478276_7478476
tgatctggttcccattgtgtaactgtgttgattgggaaggtagtttgtgagccatgaaatgcttg
gttcattggttgcttattgacctcattaacctaggacttgaatatcccaaagggtatgctcttta
ccacattcaactcctaatttatttgtttaggttatgatgtgattgctcaagcccaatctgggact
gggaa

```
>chr17_7482676_7482876
aactgccaagcaagtttcgctgagtttgacacatggatccctgtggatcaactgccctaggactc
cgtttgcacccatgtgacactgttgactttgccctgatgaagcagggccaacagtcccctaactt
aattacaaaaactaatgactaagagagaggtggctagagctgaggcccctgagtcaggctgtggg
tggga

>chr17_38269674_38269874
gtgggcggaacgcgctgaaagttttggcggggctagcagagggagaaaaatacccaaatttgagt
tgtgattttaatagggggaaaaaaaaaaggcaaggagggtgtgtcctaccctctgagaggcccc
cgcagaggggagcaggagagcagcacactagcatcctgctcgcggaagcctggaacgcaggacat
ggttt

>chr17_38270074_38270274
acccagatgaatcctctttccctggggagtggccaaaacaagtctcctctttttttctacctactt
ctgccccactgttagctggaaaacgtagctttatacagtcataaagctgcaagggaccttaaaga
tgatttatttccattttatttttttggtctgggaaaaccaagcccagtcaagtgaaaagtcttgct
gcatc

>chr17_42173274_42173474
ctgctgggttccaagaccttgggtccagtccccacatggacaatgggccgagtggctcagggagt
gcacgaagttccaatgggcttatgggagggctaggtctccacttctttgtacctacacacagttc
tcaggtcactgcatgtcactcctcaccactgccctgtggttgccaggacaactgggcaaacacca
cacca

>chr17_57923018_57923218
agggatcctatcccttcccaacaaggccaagagttcacctgaggtaatgcatttgccttggggc
gaggccttgaattgcagcaaataacctgtgtgtatttgcatatttaaccagtactcttaagaagg
attatacaaaaacctgttccagacagctgcttactcattgcctttaaaattgcgtggtgctggga
ttggc

>chr16_2301999_2302199
cccggcctcctgcccctttgggaacaggatcagagctgcaggtttagtcaccgaaatgggggtgg
gggcagcagaacccagcggtaagaacaggccacccgccttgctcagcacaagggcaagtgcacaa
aggccggaccgcagtggcacctttttatttgcagagaaatgccgaaagataagaatacagtctgt
tcccg

>chr16_9141899_9142099
attttcagacttcaaactcatcccagcaatgtagaggaagctgatgagggaaagctccctgcagc
gtgggctgtgcccccaggagcccctgagtttaaattcagtctctgctacttactaatcacaggat
gttggcaaatcgctgagcctgcatccccccatctgtcaaatgggggtgctaaggcctacctggca
agatt

>chr16_9142099_9142299
ccttcatctgatcagtgccaggcgctggggagatgatagtaaatgacacagacaaaattcctgcc
cccacaaagcttacattctggcgaggaaagacaataatgaacaaatctatatgtacaacgtaagg
ccagctggtggggagcatggtgaagaaaaattaaacgggtgaggtgtagaagtaagaggaggtaa
gagga

>chr16_21514099_21514299
gggaagggagtacccacgaccaccaaaccctgtcctctgcaatgtgaaatgtgctttgaagtcct
ctcaccctgaggcttgcccgccttctgctggtgaaagaagctgggggctaggcgcggtgggtcac
gcctgtaatcccagcactttgggaggccgaggtgggcggatcatctgaggtgaggagttccagac
cagcc

>chr16_53132499_53132699
gctttttctttgtctcttgtaaaccaacatcagcaatgagcaggccacctcaagtgtgactcaa
tcaacgcgaggcaggaggcatgcaaaacaaaacagaaaactcctggcagccttaatatagcgatt
tttctagcaatctacatgactatccaggcccaatgcaccccattacatatgcaaaacatctgacg
tttgt

>chr16_67275699_67275899
ggttgggtatgcactggccaacacaggctgaagcagcacagacccagaaaggtcaggcccaccac
gctgtcctgcctccttgcatgctgtaatcccaagagagcagagaatgtccctctccttccaggac
```

acctgcatccatttggggaagggtcctgaaggttaaatagtccaccttcttttttttttgatat
ggagt

>chr16_87421899_87422099
gaagacattctgaagactgcagtctattcagtgtcaacccctaaatcctcctgaccaaatccttt
ctgagaatagctccctgcactcctcttgagcaatacttgtttgtttacttgctctcaagagcaaa
gcgatgacaccacgatggataggacactgggcctatagtggtgcagacgacaaagccggtggcct
ctctc

>chr16_87812299_87812499
ccacaggcgtgtaccactacgcccggctaattttgtaatattttgtacagagatggggtctcatt
atgctgctcaggctggtgacaaactcctgggctcaagcgacccacccacctcggccccccaaagt
ggtgggatcacagacgtgagccagcgctccctgtcagcgtttcattccctcttcatatcacatca
cttgg

>chr16_87887299_87887499
cctagccggcttctggagagctttctggattcgcaagaggccggctgattgcatcactcgctcat
tcatgcccggagggaggcggggagtgggtgtggccaagccgcactcgaggctgcctctccccaaa
agggagagaggcacgctgccaccactgaactccagacaccagacaggcgctgcggagacactgag
cactg

>chr15_58624108_58624308
agcatctccctcctaattccctgtgatgtgataattctaagacccacttcctggagcaagaagaa
gctaagatccctcagcctggctgatgtggttttgaaagtggaagagggcaggaagtgttgtggtc
actcccacccagctgtgggcctcttcccagagccctcctcaccagatgctgcaaactcagactgc
aaatc

>chr15_89181596_89181796
gacccaaggaagtgtggccgggctggtggggcctccccgaaggcctgcttctccttcaccctcac
agagtcagccccactggaggcagagctcattgaggcatctggaacccacggaggcctgtaagggc
tccctcgctgctccttgccttccctgggttggaagtgggggctagccactcccaccacaagtttc
tcctg

>chr15_90943596_90943796
ctttgtcctattgtagtttatattttaattaattccttttccgtctccattcttgttaatagct
taaatgatctctttttagttcatgaagaagagtgtggcacaaaagatcctttcaaggtgaaaaaa
aggagcaggtagccaagatagttgggttacatagtttaccacttccctttataatccgtaggtgt
tgaaa

>chr14_61569847_61570047
tctgtgggtaatattgcactttgtaaacacttggtactcaataaattccagcacacatatggaag
caatggtgccaactagctggattgaaagtactcgatacccagtgtccagagcattggctaatgga
gaggaagaggttccctccacttctgactatgggtgtgcggcacaagcccctggcgggcctcacac
accct

>chr14_61570047_61570247
acttagcacctgtcaatctcctgtcctctgtgaaagagcctgcatttctccctgttgggcccgcc
acttctccaggatttttcagtgctgtctgtccaagtctttcttctgctggaagtgtgggaatgg
gaagtcagctctagccatggctacaaataattcacctttccagcagcctgcgggttttcttctta
ccggt

>chr14_61570247_61570447
acaacagtgagtctcattctcagaatctctgaggcctggccagtgaggcccctattcttcttccc
ctggcagaggccagctctgatggtgtccccacccaaagggcagacacccatgtgatttcagtggt
tcggaggaataggaacggaatggaagggtgaggtcttttctgctgaagctcaaagcactgtggat
acagg

>chr14_74208847_74209047
aactagaagtgcgtgtctgcagaagcaagacagagaaaggaagaatgtgcgccagcctggcaagc
ccacgtgcctccaggggctgcgagcccctccagtgggcgctcacacacactcacgcctcccttc
atcacagccctgtctccttgtgagccacagcacgggcttgaggcctggagcaagggaaggtccac
gtggc

>chr14_77422047_77422247
caaacaaaaccgagaaagtctctgccctcaagcttttattgaaggagggaaatagacaaaagaga
agcaaacaaacaaaaaaggtaaaatcagagggtggtaaaagctatgaaggaaataaagcagtata
actgcttaaggagcagggacctctgcaggtggatggcaagggaaggtctctctgcagagacgaca
ttgac

>chr19_4374400_4374600
cagggccagaaggacctctcctgtgctgacagcaaagtcaccagcgggagtccagcccacactgc
ctttctgatgaggcctccagctctggctgctgactttaaaccctaaggaaccctgtgcagggcct
gcatcccggaaggcctccaccagcccgaacctggcccgtccgccctagatggggcaatcggcgtt
tctcc

>chr19_4375000_4375200
cctggggctcccatcaccactaccagtctgcccaggaggcagcagagcactgagcccgacctgct
cccctcgggcacctacgagacccacctgcccctccatgcgtgggggagaagggggtgccctctgct
gtggaaagctgggacctactgtctgtggtgcgaggctgaccccacagagctccaggacaaccccg
agagg

>chr19_4375200_4375400
agtggacagagccagccgggccctccagcccccacctggcccagctgccgcctctgggcagatgt
ctccacttgataaacaggacagctaagggcctaggagagacatgcctggcccaaggccagacagc
aggagtggctaggaaggtcaagggatccccactgcaggccccaccaaccctggctctgcctggaa
tgcag

>chr19_6738600_6738800
ttgcccaactgcaatcagacattttctagctcagaagccaccagcaggtggggccaggtctcagc
agagacatcagtcccagtgtgggctcccaaggctctcctcctgggctcagctgtatctctctgtt
ctgggagcctgggtgctctgtgtaaggggaacgtatacccaagggccccccctgcaagacgcccag
ccttg

>chr19_13275200_13275400
tccagatggggactgcagggagggctgacccccctcccgcatacaccaggcgggcaaggtggagcg
cccctcctcggaaattgccccccgcccaagatgcgtcagtgacagctgtgcctcctcagcccgca
cccagccggtttccttttcctgtgcacactctcccgatgggctggccttcagcctggagagactc
cacgg

>chr19_13275400_13275600
ccccttggaaggggctccccagagggtcttcctggacgagggggaggggacaggctgaagaacaa
tggggcttctgtggctgcaggacgccaggcacttcctgttaaccctttcctcggggtgcagctgc
agggagcacctcccaggtcccgccttgtgggaaggggcagaggcagcctgaggggaaatcttgtt
gcggg

>chr19_14629600_14629800
ctacccagtcctttccccgggaggcgatgaacctgctcgcccttgtctcgtggggcctgcggtgg
gctcccgcccggtgacgccggacgaactacctaacctgtgtctgcctaacccaggggccccccttc
tatctacaacatggcaataagagaacctatcaggccgggcgcggagcctcatgcctgtaatccca
tcact

>chr19_18393600_18393800
gcatgcacccctccagctccccacctctgtctgaatggagcccacaccctcctttccctgacctc
cttcccctcactctctctgatccttggcctcccagttgcaggactaatcgtacctctgggccttt
gcacgtgctatgctctctgcttggtactcccttcccccagtgcaaagatgtgtcttcaggaagtg
cccca

>chr19_39173960_39174160
cccctgcctctcctctttctcgccccccgccctcctccagagaaagcaggtggtgagggctctct
aacccagacaggacttgctgttcagccccagcttgaaattgatttcctccagccctccttgagcc
aggccccgtgaacagaggaggggtctgagccaggcctcctaggccattggtgggagggagaatga
gacag

>chr19_39174360_39174560
tgaaatctgtcatcatgcccatataaggcaaactccttggattactttttaccttggcagaatca
caggaatgtcaaggtaaccaggccacctcagcatagctctgattctcacccggtcacctgacttg

cccgccctcccccatgactcacccagtggctcagcatggggcctgctgcactgtggctgctggaa
tctgc

>chr19_39174760_39174960
tcccccagcattcagagccttatctgtaaaattagagcacgcagaacaaaccctggtacacacta
agtgctcaataaatgtgagtcatttgtgtaattatagtagggtaggccttatgccccacccagtc
ataaaaatgcctcttgctgttgttggttcaggctcagccccttagtggctttcatggggcaggtt
gacat

>chr19_39175960_39176160
tttaggtaactcttaacaccacgaattagttgcctgctggcaaacttcaaactccaagcctgaaa
tttctggttggagtcacctcccagatggactcagggattcattcacccccctcatccagtgctcac
ggagcctctgtgaggtgcaggcctggagaggagccctgtggcctcagcaaagggcaggcaggctg
gtgag

>chr19_39177160_39177360
agcctctgctgctgccctaattttaaaactgccttttgggagtgtaagtttcctctgttaaaggt
agttatttcaaggtaggcctcaccatctcctcctcctggtgagaagctctgcctggagggctgag
cactgcctcccgctctgtgggccccacctgccttgggttgagacctatctcttcctggactctgt
gtggg

>chr19_39893160_39893360
cacacatactgttcacacaaccgggtacacatgacggctagcccaggcccgccgcccacgcagtc
gcaggccagggtacacacggccagcgccgcccttggccacgcccgctcaggcccaggcaatcgcg
ccaggcgcacacacggcgcgagcacacgtgccgcgggtacacgatccccagctcgcccgcgcact
cggcc

>chr19_39924960_39925160
ccttccccagttctgaccactttggatcagcttctggtattgggcccagtccttctccccaacta
ggctggcaggtccatgaaggcaagaccttaagtcatctggatcactgctatgtccccaaaactca
acagagggtacacatacaaatccttcaaatacagtgacagccaataacagtaacattcaacatct
gctac

>chr18_3596800_3597000
agcaaagccattcgctgtgatctctgattgtgcagtgtcatgtcctgtcaccagagccccctcgt
gtttgatgttggccaatgccgccagcatgatctagcaggccaaatcctaatctaccattctctga
caccagctggtcccctgggtcgtccacccgatgtcccccattctccccacttggcctcccccaca
ggctc

>chr18_3602800_3603000
aggtcaaagacgttattaagtatcgactctgaaaaagcaactataaatactgcaaatcgactaag
ccagaggacgcaaatatatcctgcgtgttgtgtgctggactgtggttgagacttcatggtcgtgc
tctgggctcatgggggcagctgactgctcataattaagattgctgttgccgctgcctgtgttcca
aagag

>chr18_3606200_3606400
tcattcctgagccttggtgccttgacgagtcaccacgctcaggaagaaagctgcaactcgccagg
atgaaactgggccccgcttcttagaacccttccccgcttccttcccctgccagaaccacactaaa
gaattcgctgctctcaaagacagacgttttcattacttccttacattatagagtttgggcttaga
taacc

>chr18_20839802_20840002
gcagcttcagccccttgtaagccctgcccccctctcaggactgccctgaggcctctccagtcctcc
ccacactcagagatctgtggggaagctccgcccagccacactccttgggataatactagccggtt
ctgcctgattccttttcccccggagccagcctagggggcccgggactcctctagtgagccttgac
tgtta

>chr18_20840202_20840402
tgcaaatgggttttgttttccttggcattttgttgttccgatcacagatttccctgccagggtgt
ctgtggttatcagctgcaggctcagcttggggcggccgcttcacacaagccactctgtaccacgt
gccctaccttagtgacgggagtaaggagcttccttcccctccatgtcattccttcctgttccctt
cattc

```
>chr22_30592400_30592600
aggtatcctgtcactgggtgctgagctgccggcacgcctcctatcaccattgccatggccagcgt
catcaccatcgcggtggccagcgtcagcgtttgacaaaggaagtactcagttacaattaccttcc
tgttggccagcagctcagataattgctttgcaatgtgtgtgagaacctcataaattaaaccaaga
aacag

>chr22_30592600_30592800
aggggccaggtctgggctgaagggaagtgtcaagtggaggaggcagtgatgcaaggctacctttg
aagtcactttgtttgtgaaaagtctgctgtgctctcttaaggaaactccactggaatgaggtttc
tgtgcatgtggggtttagggctattgtttttggctctgactttcacctcaagccctagctaggac
atgta

>chr22_35772600_35772800
agagtgctaggattacaggcgtgagccaccacaattggcccagtctatggatttttaaaaaataa
cttgggcttgtcttccttgctgaagaaggctgttttccagcctgtcacacagcagttaggctgta
gaccctcactggagcccctgccctgcagaatcgagcacatgttctttctgggtctgtgtggggtg
tgggt

>chr22_35773000_35773200
ctgcccaaaccacttctgtttcctgaaggcgccttgggaatgctgagtcgcgatttcctcatccc
ctcgtgcagctgcatttctgctgcgtcatgtttgggagggggactcgcggaaacaaagggaagg
cggattttgctagattttgctgagtcaccagtgcctcctcagcttctctttaggtgggaggtgaa
agggc

>chr22_36726454_36726654
ttggtctgttatcttgtgtggcctccaataaccttacaaggtattgtattgtaatgtacaaagca
ggtattatcatcattgttttgccatctttccacacagcggttcttctggatttgttcctgatctc
agaggcatctcttctgggcagtggggtggttagaattcttagcaaccagtgggctctttggccta
ctcag

>chr22_36727054_36727254
cttcccaaattcctgagcaagggacagtgggtggtggagacagggttcctcagagcacagtttga
caaccactagtccatgtgtctggagcagttctgcaaaggaggaagccacagccgagagcgctgtg
gccctgggttccgctcttccccacgcaatgcctggccccttgacctctctgggaatgaagggact
gctct

>chr22_36727254_36727454
gaggatggaaggaaatggtgttcctgtcgtacacggtgtgaggcagatgcccagacatgccgtcc
tcacccgcctgctgtctcggagctcccaactgagcaattccctggcaccgccacgagaaagtctt
tcataaaagttacaaattcaggaaccaggccattttctccctggatttcattttcaaccgaaaat
gtcct

>chr22_36727454_36727654
gaggccaagcccatgcagtgagggcatctgagctgatgtggccatctacctgtggacaggtgttt
ccagccagctcagcaggctgggcccaggagggaggaaggggaacctgaccgcaggtatttcatcc
caggtgcagacttctgctgggggcttaagcctcacttcctctgtgagaggagctgcagctcactc
ttgtc

>chr20_10492200_10492400
cagtaacagcaagcacaaatgactaatttaagaaaagcagtacagcagctggacgtgttccatct
tctttgatgctcaccacctccctgtgaggaaagtactcttactaccccagcttcgcagatgacac
ttggaaaacgtaaggacccacagttaggtggccagactgagagcagagtgctgagtcaccacacg
gtgct

>chr20_30160339_30160539
ggcaccgtcagtctgacagcagggctggctcctgcagtgtgaatgaagcggtcttgtgctcgact
gtgaagggggttaggacctgcttcccagggcgggtccagctttctgagggaaagccttcagagc
ctccatcaggccggcaccaggtaccagttaagcactgggcagggaacagaagtggtcaaacgccg
cttgc

>chr20_30160539_30160739
cgcgacttgaaactgctgaataaatggataatgtcaagcgctatggcttagggcagtagtacctc
cggttcctacgatcgtgtctggggtccctgaggagcaaggcaagagtcggccattcaggaggtca
```

gagttcagctgttcttgcacaagcagggaagctgagtcaccccaaacacgggcaagactagcggt
ggtat

>chr20_45946593_45946793
taacctctctgtgcctcagagagtttaccactttacaagtggtaccttgtaaagttatcgaagaa
gtcaatgagttaattcacgtaaagctcttagtataatacctggcaaatagtaagcacctattcaa
tggtaactaatgggtaactgtattttttttaaccaatcactcttcctccttttcttgcttctctt
ccacc

>chr20_45977593_45977793
tgaggtgctagaactttccaaacattcaggctgacactgaagtggtaagaagctgggcatttact
ccagaagcaattccatcagactgtcgttccacaggaagaaatgggtgcatcaccaattgatatgg
ctggaattttccatttggaagactcaacgccaggacaggaaggaggctggggaagagagaatgtc
ctgtc

>chr20_45977793_45977993
ctggggaagagagaatgttccgtggtctgggactgtgacaacgcaaaaggcccaaaaagacagcc
tcatgtctcgaaccctagtataaggagaagattttaggagagttccacgttaggaggagaggact
taggatgggagctagatagaatggaaaagattatagttcagagttaaattataggggcttggaga
cgaag

>chr20_45978793_45978993
taatcaatgctggagcagaagagagagggaaaaaaaagttaagctgttttatctttccaagtatt
acaagtgggcaccataatcacccaactttagcaatagcaccaccagctttagcacagcatttcaa
gcacaacaatttaagaaaaccatctttttcacagatcagcaaaccatcctttcaaaaggaacccg
tgcac

>chr20_45988593_45988793
actgcataatctgtgttttcttttaggaggtagaccagtttttttgctttttttgtttttttgataca
gtgtctcgctctgtcgcccaggcttgagtgcagtggcatcatcacaactcaatgctgcaacctga
agacccaccctttttgaggtgggttttcagtattgaatgatggcgctgaaggaaacttttagatta
gactc

>chr21_35320130_35320330
gcctccccccaaccctattgtaaaatgtcaagtgtttcagttttgagaaaaaagaaagctgaaga
aaccatgactcatgacatgggctgacaaacaacgatctgaaggtatgctggtgagtcacactgtg
gagctctgcctttgtgttgtggtgagggccggggctggaaggagtgtcatgggaccagccagtaa
aattc

>chr21_35320330_35320530
cactactctccgaagccgattgggaggcaatttaaaaaccctgggctttttccagccccagtgta
gtggatgactctgggtcccctcaggtatgtcgatctgtatgggaaatcaggaagaatgattatta
ataactggaaaaaaaaaacctgttagtatctttccatgcccaatgccatgctgaaccagccctgc
ctgct

>chr7_44679475_44679675
agcagctcagcagtaccggtatgaaccagttttttttctgactgcctccagcatgagctgaatttc
cgtctgtgcagttatcctcagccaattgaaaatcacctggagttgtattccaagcacaaaagaag
gtcagagagtggaggcccgatgatcatgatcgccctgtctccagggcctaggctggaaggagtcc
tgcag

>chr7_44679675_44679875
cctttgtggctcaggaccagagagctgaccttgaccctgaccttgtgatcccaggcatcagtggc
tggaaattcctttcattttattgttgagcccagaagcgcccagctctctttggcaaggttaagct
agggtaagaggcactgttactagagtgaccagagttctttaagcgtcgctctgctattactcagt
taacc

>chr7_104585764_104585964
ttccccagggactgggcctagctcaatcgggaaaagtcagcatccaaaagtcctcaatgacgcag
aggaagccaggagaaaacagtcaatggctagtcatcactgattcgtggtcacaggcgttagaaca
ggaaggatctgcacagatcagcctctcccacccctcactttcctgataaaggaactaaagtccgg
aaagt

>chr6_7146401_7146601
aataaaacactttgtggagcctgttgtgtgagaagcaggcagcggctgtgggcctcagccttgcc
atctgtttctagagcagtctgtggcccgcttaccatgccctctgtcatgtaacataggccccatt
cagcagcagcattcttgaatgtgcatttatgtctctgtgggaccgggggtagatggcctgagagg
gggaa

>chr6_26025821_26026021
ggagtccaagaactgagccagagtacaccagagtatgtagtggagtacagctttctcattcttta
gtagggctgtgtagagaaatactctattttatggatgtatagatcactctagtcctttctgatga
aaactttacagttgccactcatttactattacaaacaatgctgcactacattatgtttcagtttt
tagcc

>chr6_26026221_26026421
caaagatgctgaatatttggctgacttctaaaaatctggtcacctaactttgacacttcctaggt
cgtacagtttgaaactttcacaattaaatcagttttggaatttacaatttaaggcaggaatagaa
gactattgggttgacaggtacagtgaagatgcaagtcatctgagaatcttacatcagaggggct
tccat

>chr6_26122621_26122821
tgttctagacttgaataaaaagataggtagactacgtctaacccttcatcttaaaatctttacct
ggaaaagaccatgagtaaaatacttaagggaatgtggaatttcccaggccacaaagcggcctgca
gttgtctaggaagggagagtcctctaggagatacagtgtatgtgctaagtttaatgacgtctcct
cttca

>chr6_31706021_31706221
gattccatgcctctgtgtgggtgtgaattccctcatggtgaccctcaaaatctgcacacaggacc
ccttcccattgaggggaggggatcaaaacaactctacttctcagggtcctctcctgttccaactg
gtctgtgtccaagagaagccttaggtaaatggggccagcttgaagatcaaacaggtttggcagcc
tctcc

>chr6_35437822_35438022
cggggtcttagagagggtgctgcttggaggtcacttacatgattgattcaagtgcgtttctggcc
tgccacatttgaggtgtgccttggtgaccaggttctaagcaatgccaatggcttcctctctctat
cagccaagaagtatgatgcgtttttggcctcagagtctctgatcaagcagattccacgaatcctc
ggccc

>chr5_79542844_79543044
cagaacactaagaaaacactgggtctttacaattaggcaggtatggccacttaaagtactaaaaa
tagcttggtcaaagaattacatccaatggctagttctgtagacatatcagcttatatgaagactt
ccttttaacatcaagttatcacagatgatctggccacatactgctctgaaataaaagtagacaa
tgcca

>chr5_133838901_133839101
taggctttagatttttggtgctaaaaccagtacagtcccaggcaaaccaggacagaacagttgg
tcaccctaggtagtaatcattaacccagagagggccaggcttcaggagccagctggccagggttc
aggtgtggcccccagggccccctccaggcattgccttgtccttgtggtttgtggtagggccttgt
cccat

>chr5_179246594_179246794
gcttggccagcacctgtctgtaggccacagcctctgccagcacgcccctctgtgtcccctgcccc
tgtctgcaaggcagtggctccagcaggccctggggcattttccactctccaccgccggatgcagg
gagaggcctgaaccctctccacagggctgctctgggcagggtggaagccttgcccacttcggagc
cctcc

>chr5_179246994_179247194
cttgcctgggaggctccagagggtgaccactcgtcctgcccctctccttgccccagttctggcgg
acaggttactctggtggcataaagcagtgtttcttccttcctagctgaggaggctgttggctgac
ccccttggctgcccacaaggccaacgggcctgagcccccacagggccatgggcattacctgctga
attga

>chr5_179247194_179247394
ggagcccataaggagtcacttggaccacagtgaacacttggcgaccactgacactcaggagacct
tagctggtcctccagcacctctcaactccactcctactaaactgggaacttctctggtgctcagg

ccagagtcggggtccgtcaccgagtatgctatgcgctgcccatcaccgaggatgccatgcgctgt
aagag

>chr5_179247394_179247594
ggctgccaccgcggcaggctgaccatggcagggtcggaacagcaacctgagagccagcttgttct
ggccagcagtgcccactgggcgacctagcagcctcctgatatgggggctgtgtccccctctccct
gcactgggtacccccaactgaggatattgctgagtcatggccaggcccaagcctgggaggggcga
ggggc

>chr3_39192396_39192596
tactcctctggagaggcgggggaagtgcccctggttctcatgttcctctgttccacctcggaatc
cagaggctaagaggcagaggcaggagatgaaacaaaaagactgagccagagacaaggagaggcca
agagggaatgaaacatagtgagaatctaagaaaagtgtcagacagtgagtcagaggtgggggag
gggcc

>chr3_39192796_39192996
caggcagccctcctgccaccacacacaggcactgacaaccccccactcaactggccacagttatc
cacacgctcaggtcacactgtgcaaatccaggaaaggccacctcaccccctgggcaccaagtaacc
aaaggctgtttttgagaagtgggggcggggcttgaaaacgtcacagcaactcctctgtccacctc
agcag

>chr3_39192996_39193196
tcctaccagtgccccaggcctcaacccaagccagtcttagggagggggcacctttagctgcctcc
atccccccttcaggaatctgaagaatgtgtcctttaacctcagagccccaggccttggccagtccc
cccaaaacacttggggtcacatgacccctgtccctcaggaacttcctgggagaaggggagggcac
ctccc

>chr3_39193796_39193996
gggcacggcaagccacagaaggcttaagtagggagtgacaatgccagatttgcattttaaaaacg
tggctcagcaccgggagtggagggggtgtgaccggttggaggtgctgccagggccaggagagatg
acaggtcaagggcataggagaaaggcagataccaagatgtggagaaggaagagggcagggctgtc
aagga

>chr3_49066196_49066396
gtctgcagtgaagtcgatgtacccagggagaatgagaaagtcactgcgagggaagggagtgaatt
gggagtaaagctctcagcttagggcagcatgagactccatccccatgtaccaatcaacccgtaac
gcatgtgagcagagagtggcactgacagagtctgtgtgtcacaggcacagcacaaggtggaaatg
ggtac

>chr3_52028760_52028960
cacgcactcagacaaggccactccttaccctaagtgccactgccaagcaagggagatagccaaac
tgccctcaggaggcaggttacaccctgaccagagggttacacttcaggctgtggagaccaaggaa
agctttattatgggtctggtagggtagaaaaaggaattgcaggctttgggtgggccagttaggct
gtgct

>chr3_53303760_53303960
ctaattttgtatttttagtatagacgggtttttgtcatgttggccaggctgatcttgaactcct
gacctcaggtgatccgcctgtctgcatcaatattttaaataaagatgttaatgctccacaggttg
tgcgtctttccccacgtgagtccacattcttttctaccctaggattactactggcacagaggaag
ttgaa

>chr3_141086710_141086910
taattaccaagcctcatgccaggcactgagatataaatatcccctttaaagagtggtcatgtctt
aaatccaggaagagctagttcatgttggcacccagggtagggagcgaagcccagggccacagcac
agaggcagaggagaatagacactgaccaccaggagctgcccggggatggtgacaaaagtgccttc
cacct

>chr3_177077106_177077306
gcgcccagtgcccccaggctgcgacagccaagtcggtgggcgcgcatggcggggcttgaaggaaa
ggtccagagccggtgggaaaggccacgccccggggtagagcgcttccttccagatcccggccttt
aactgcaattccgttttaagggcaaccctgcccggttcacacctagccttgaagatgacagattg
cactg

>chr3_177077306_177077506
tgagcaatttgcagggtgagaacgggagcctacgactccgcacctgcactgccagagccataaat
gcagcaaatcatggcctataaaatgtcaccactgctgaagtgcaattttccaggggtcttctgca
gagggaactccaccacggagagcgaccctcggctctgcagcctaagaggaaggcccctgcagggt
ggggg

>chr2_43446296_43446496
gccctcacacacatccacacatacttacacacagacacacccatgtataccaacacacacgcccc
tgcgtacacaccaacccccacatcacaacagacacacccacacccaggcacaccccaaagatcca
catacctacatagcaacagatacacccaaacccacaccagcactcacacacatccacacacactt
acaca

>chr2_70370696_70370896
tgccccagcagttggagcagtttggtgagagagtggcacaaagctgacactggtacatggccctg
gccactagttgttaagttccacccatctccagcactgccctccccatgagtgacagtgaaaggtg
ggagggacgttacaggactgtatcaggcattagacctgctactccaagtgcagtctggtgcagtc
tgcag

>chr2_87785885_87786085
ctaaagagcttagctaggcatggtggctcacgcctgtaatcccagcactttggaaggtcgaagtg
ggatgattgcttgagcccagaagtttgagaccggcctgggcaacatagtgagacctcatctccac
acacacacatgcgcgtacacacacacgagcgcgcatgtgtgtgcacacacacacaaattagccag
gtgtg

>chr2_101620168_101620368
ctgcctgcacacttcaggcccctgctctttaagcatagatgaagtcaatagcattgaatgatctg
ttccaaaaacatgtcctgtcttaagacatgacatttaatcaaatttagtattttgattgaggact
tcagaaagtggattatgcatcttcttgtcctaagtgttatgctaattgctgggcattgcacactg
gtggg

>chr2_178029954_178030154
actccttcctcctaaccagcttcagctttcttcttccgaccacagactctccaagattttgcttt
tctcatcctatccttccctcttcttgtacaagcgaacactgtccttccagactcttccatggctg
cagaagctgtagggaggagccctcggccagccagacaaaatgcacgtaatattgctcactttagg
aaaag

>chr2_202022955_202023155
cctgcaccacacagctgtctgctcacagtcatgtgacaggggcttccccagccagaacctccagg
gagcacaaataaaggaaagagctggaaggcacagtacagcatttgttctgtccaagccttacata
tcagcatgggcagggagaatgtggtgaagttttctttcaaggactttgtttgataaggaaggaca
tttat

>chr1_8933213_8933413
ggcttggagtctacagacttttggtgatgataacgtgttcacgcatgttgaccactctagtgggg
atgctgacaatggggggctgcgcatgtgcagaggcagtgggtactcatgggtttatgtacctct
gcaccttccgcctgcttctgctgtgaacctaaaactgctctaaaaaagttgatgagacagggaga
gagag

>chr1_23882213_23882413
gaaggcaagaacatgagctttgaagctagaggaagctggggttcctaaactctgctctgctactt
tcttgctgtgtgaactgtttcttgctgtgtgccactttcttgctgggtgatcaagttattcagcc
tgagtctttttctataaaccaaggaatgttacccatcacggtggtttgtgaggattagaacaaa
aatgg

>chr1_23883213_23883413
agaacaggttaagaaccactgatcaggctgggagtttggttggcaatgccagaggttctcctcct
cccccatgcccctgttcaccccagcaagctcaccagggttaaggcactaaaggtgatgacgtgac
ctgtttgttatgaaatattgaagtaattcatgccagggaccaacacacagatgagatcataccat
aaaca

>chr1_27113613_27113813
atcctgagaaacaagaaatccaccaacggagaaggatttatcatctgtttcctttaaggagggtg
caaagttgttctaacagtttccgttacaagggtacaaactggccgctccccccaatagacggttt

tgttgagttcgcctcttttttttttcttcttttttttggccccacttcctatgagagaaactgcttg
ggtga

>chr1_36838813_36839013
ggtgtcaccaggtctttgctgggtgaagggtgggtgctccctgctccgtgctgaggccgcttccc
tgtggcaagtctctgcaggtatgtcttttttttcattatcatttgatgggcacccaccgcttgcca
gaaaccattcccagtgtttcccagattctctctttaactcgcaccactttctgatgtggattctc
ccctt

>chr1_36839213_36839413
ggaggcggatagagaggaggattagtaatccctcgatttcagccaggactccctgacgacaaaga
ttcatgcatgtgctgtcatcttcactgctccaaaacagagcgatgattttgttcatttttaaaac
gcaggacatgtatagctccaatttgtcaagaatacataatatcacatcatttaatgcataatatc
acatc

>chr1_36839813_36840013
cctcctcaccccactgccccgaggaagtccccactgggcttttctgtgacctcctttggaggctg
caaagaaagcctagaagctaaaggcccctggccaaggggggtgcatcccagagaaattctggaaga
aaagtctcagggctgtgcacacagaggtagagagaagctttctctgcaggctctcctggggaaaa
aatac

>chr1_36840613_36840813
gagaaagcagcaagcaaccgcagaacaactggttgattctaaaagccaccattctgtccactgtg
tgacctggacacatgcttctctgctctgcagtcttctcatctggaggacagggacagcgctactc
ccaaaaagggttcccttcaggattgccagggcccagtacataggaggcactagacgcttaactta
gaaat

>chr1_36853413_36853613
actcttgctggctctccaactctaggcttacggcagacggtgctgccataacaaagtgacgcctg
tcacaacagcatgagatggtggcttgacaaggtataggcagatctgcctatcacagaaggaggga
aagagaccttcaacccccaggggcttcctcaggcttctgaaccctcgtctccattactggttggc
tctgg

>chr1_37943213_37943413
tggggaggctggcccagccctgccttctgtcctggggcaggagcagcctagctggagtcttctac
acacacatcctatcagtgtctgcttagcccggctgggggaccctgaccttacaggcaaagagaac
tggaatgggggcagggatgtctggttttcctgtgccagcagcgcttcctccagtgcagtgaaaga
gacta

>chr1_45272213_45272413
gacctgccggtcgggcctcatcaatgctgggcaggcagcctcggggccgcaccggtgagggtttc
cgcccggagtctttggcattctcctcctcctggcgtcccgggggcagaggcctgctggccatgga
cctgctggctggaggaaccacactcagggtggccttcaccaggcatctgtctacccaccccagcc
tcttg

>chr1_45272813_45273013
tcatcttccacccagatctactctagggtggctggcacaaagatctcccctacacctggcccagg
ctgagggcagatgactcatgaagccccccttccaccttctgcctttggcctctgatcccacttcct
ccagaatcagttctgagatggctgccctctgctcccgaaactccctcaggcacctgagtcacccg
caggc

>chr1_45273213_45273413
gcccctgagtcatcagggagagcggccgacagggtcttgcagcttagaggcaaacttcctaagcg
caggcaacagaggggcagatgtagagcaaggctgtggtcagaaaggctttgtcctaccccaggac
tcttgagccagggcccattttctccccttgtggaatggccagatgactggccattcgtgaacaag
tcaca

>chr1_45274013_45274213
ctagggcaggctcagcttcgggggctaagaccctcgtatggcgcttaatgcccctctaagggctg
aagttttccctgggcctgcccgggagatgggtggagtctcttctcaggccagctccagtttccat
ggaaacccacaggcttcccaccccgtcggctgcctgggcagactcccagccagggcctctttcgc
cgccg

>chr1_45274813_45275013
gcccttccttctttggactttggcccctggcagaagaaaatgattctcagcccctgggatcctta
gagatggtgggtcatgattcccctcctccctccactcactcagcttggtattgaggcctcactgc
tagaagaagaggaacagatgacctgttctgggaactgaggcttcacaagaagaacaaagctccag
agatt

>chr1_150539776_150539976
agatacagcgacttccatcctgtttcatacctgaccccaactacccagctttgaagtggggcaag
gggcaaggaagcaggtagcccagcactcctccccgccatcaccaggctcctctgcagagctgttg
ttccttaaagctgacaacaagccacctcatttcaaaacacacacacacaacctcattttgacaca
aagtt

>chr1_150540176_150540376
ttcccatagcccttcacctgaggtgtgtgccccactttccttagatttgggctctcatgcaaaag
gaaataaacaaggtatcctcccctcaggaaagttcagtaacaaagcagtgaaaccgaacagctgc
tcccttgcagcaggccagctgtctcccctccccccatttccaccaagggccggagacggtgggaa
ctgct

>chr1_150540376_150540576
ccaggcaacctaaaggatggcgttgtgagctgagaaaggcaggcctgatagatctgggcatagga
gcttcaggaataaaatgcatgtgaatgcggtgtttcccagggagaattaaccgcattctcgcctt
caaatctgaatacctgagagtcataaccagcccagtagggtagactatgaaatgtatagcaaaat
tttca

>chr1_150540576_150540776
aaaccagctcctcctaccagacaagaaaattaacatttttttagggcacagaactgaaagcatttt
tactgaaatctaatccaggatgaaacctaacagttaaacattcttttccttctctgcttctttgg
ctctctatcccagcacaccttgtcccaggctaacttgagtaaacatacacatgtgaccacatgag
ggcgg

>chr1_156074376_156074576
tcgtccatcctccactcctggatccattcaccaaggggcctgagtgaggtcccacccccctaagcc
acacaggtcctctgcttctcccccgctacatctcactgggaccccagcctggcaacggctagtgc
gtcgtcggtgctcagttagccccaacatctctttcctttcctccaacaggaaacacaccttccac
ctcta

>chr1_156093776_156093976
acctggctttgtcttagggaaggacagctgggagcctagtggctctggtaggggatctgagaggc
ctcagaccctaggcatatttggctgtttggcaggtgtcacgcccaagggaagcgtgtggaagcag
agccatgcctgctgtgggtgcacatgcccgcgtgagggagtcggggtgtttcatcctggggcacc
tgtgg

>chr1_156096576_156096776
ctcaagaggccactgggatgcagccactcctgtgcttggggaacctggaggatgcaagggaaagg
actggcactctgctggcacagcacccggcctggggcaggacacgggcgaagccagggtctcccct
gtgagcactagaggatttcccgacccctgcccgggtattgtgtgcctgagcatgagtcacctgag
gggcc

>chr1_156717976_156718176
cttctcacttgatggaatttctaacccttcacccaaaagaggagaaatcccttctacagtgacac
ctctatccctcaagctacaaagaaaatgtcctttcttacatttggaagtggagggcagttcttcc
tagatgtagaggttatgaggtctcagggcagccctgtaccccaatacagacatctgtcttccagg
tggtg

>chr1_161089376_161089576
ttgtcacgtgctgctgaacagcaaaggtgagactttataaccctttagcctgcctcttcccatgc
tcttctacctagattctccagaattgtttctcaactcttatttccttgacccaaggatttagggg
tggtcctacttcagttcctagcctataaactatctcctccttgggaggagtaagcaaggcttcta
gaaca

>chr1_234747377_234747577
gattggtaggtggagagaaaaaaaaaaatcatgctgacgtttctctctagtcgttgctattgaaaa
taccaggttgcttcaaaggattaaccacaaggaagaggaaaaacacccttagagaaacagtggag

ggagctgcttaaaacacatgaaaaaacctgttcgaaaatggcacagaaaacgtaatgtcagacct
aaatg

>chr9_131903379_131903579
ttttgggaacctgggccaggggagggaaatgagggggataaaatgacaggtttttttgacttaa
atcttaaaagttttgtcacagttgcccttactagagaagtaagaggcctgccaggagtgggtcac
caggtggcagcccatggctcaagagtgggggctcctgagggctcttgtggtgaactgatgcccct
ttttg

>chr9_136215979_136216179
ttttagtgggtgtaaagtggtcgcagtccttaatttgtgtctcttagagacgggggcaatgatac
atgcttcttgctttcattgggagttgctgagcgagcattcagctcaatatggtagtggccttgaa
ttcagcttagccatctggaaacaagtacagtagcagtgtcgcagcgaggtactaggactgcaatt
ctgct

>chr13_99137399_99137599
agacagagccgactcatcaagacaggggaactgcagttgagaaagagtaattcacgcagagccag
ctgtgcaggagaccagagttttattattactgaaatcagtctctccgagcattcggggagaattt
ttaaggataagctggtggggtaggggaagccagtgagccagaagtgctgattggtaagagatgaa
atcac

>chr12_6446539_6446739
aggaggctttaaagtgtgacttcctgtgctggggctgcagagtggggaggcgacgctgagatcgg
ccttgtggtctgaccccgatccagccaccttgcagggacccaattagtcttcctttccccctcat
tcatgtgtctgttcatttaatgtcaaggtgttgggggaggtcgtggcagcttctcctccaagggc
ccagt

>chr12_6999739_6999939
tgtatgctagcatggaagggaattgtttcttcggttgagtgcctaaagtgcttgatggcctttca
gatggatcatgtcatacagaaaggaagtatcttaaaacactacaatctagtttaagacataaaac
agtagaatcgctgtcagcgtcacaagatacaaacgaagggctcagaactgggaccaagagaacat
agtct

>chr12_45626733_45626933
gagagggagcaggtacatctgttccctgcactccatcttttcctcttgaggaagggagaggcagg
gagctagaagccaacgggcctctggattctcagccagaaatggctctccagacttctactctact
ggctgtgggatgtgactcattcctgtctggaggaagagtttaacctgacttcggctgggagtcac
aacca

>chr12_45627933_45628133
aaatgtgttcttaagttggaagagaatgtgtgaggaaaattcactcctatctcatttacagaaa
ttcagattttaattgtcctgctgtaattgtgaaactgaactgcacattcattctgaggattattg
cactgatgagtttactgatgagtgtgacactggttcggagggaactgtttgcagactctccctat
gtata

>chr12_45628333_45628533
atttgttcaggccccttttccaagttcccagggaagttactgacactgatgttggtttcttttaa
gaatgactcatgatttttcgagcctggtttccactcttagtcatcttccctgcctgagttaaaac
aggctttaggctctcactgaattccagccttgacagaaccccccacccccacccctgggcttttg
attcc

>chr12_45628733_45628933
tctgtgttgctcttgtcagtgtttactatcagtccagcagtaacctttcagttctaataatttgg
cttgtccttttcctcctcagttgttcaaactcctatctcagaaacatgcctaggacctcagtctc
tttagttctttggattctgtgttgctcttgtcagtgtttcctaatcaccctcttgccctggaaat
aaagt

>chr12_45628933_45629133
cctctccaatgtggtgctacttctcctccttcctgtagcagactctggttgtggcctgtctgtgc
ctacagatttagtgacaggagctgtacttttctgttcccttctttagctgtcatactggtggtat
cttaaatcttagccctaaattgtgatgaaactgttgtattttcacaagtcttccttagtcatatt
gtgct

```
>chr12_45629333_45629533
acctagttgagtggcatgacatctgagcagaactagcaaggaggcttggtggtatgccgcctttt
aactccagcacctgtggctcttgagcattctcctcattcttcagtatggaaaatggatattttct
cagtcttgtgcaaaaacagcttcaagaaaagtcccctagtcaaaaattccagtatttggaagtgt
accag

>chr12_56520133_56520333
tggcacctgggcagcagaattgtcagtgggcggggacccaccctgtttacttgtgttggtaactg
cattgcagctgctgatcctgtggctcctgggccccagctgggacaagaacttcctgaggatgaag
ttttatcccctatataatttctgatgatctggatgaggtgaggaagggagaagtttggaaaagaa
cctcc

>chr12_76337933_76338133
tgaatggggggacctgctaagagcagagaaaatccccccagggatcattttaggccactgcttata
aaagaacaaaaataaaatgggaggaactccagaaccaaagtcctagagtccctatttgcttgtga
atgaaagagactagtatgtaaataagagctttgcataagccagacattctttcaagtccagttta
tctgc

>chr12_76338133_76338333
agtatgagtcacttccttttaaaagcaagaactaaaagagaaagaggcacatagtcattaccca
gcacaaaccttcttaacagaccttttcttcctattcaaattgcaagaaggtaaaagactcttgct
ctaacaaaacagcaacatcattttgcatatagtgtatacactgtttggccagttaataccctcct
tcagc

>chr12_109083471_109083671
ctcaaagaacagttaaaagatttccacattgttcaaacccctacctaatattcatctaagttttc
attttaggaaaaaatgagtatcaaaatcactttatgtgcccaaatttcaatcataacttattttt
ggctttgggtgatacatactgagaattcttcaactcagcaaggagaatgttaagtcacacatgtg
ctcag

>chr12_109086271_109086471
gatcacacagaaaagcactgtcaggagactgccattccctctcaagtagcccatgaaagaaggca
cttatcagcacggcgcctggcacttaaaggaagccctcaacaaatgtgaactgctgcgatttcat
cactgtcactgtgtcctcacaaggcatggtatagaccaagtactttccaaacctaagacaattct
ctgag

>chr12_120637017_120637217
gaagtttatgggagacaatcacctttcagcaccattctccaggaagagggagacgggcactgggg
agaaaggacaaaactgccaggggaactgacttctccagagctgggttgttttccaggtgccctcg
gatggccttgcgcatcatggtgttcttgcccatcagcaccacagccttcccgcgaagggacatgc
ggatc

>chr12_125402647_125402847
ttccccatttctccctcccaccagcccctggcatccaccattctactttctgtctctatgaattt
gacaactcatgtgacctcatataataagctttgtttaagggtaaaataaaatttaatagttgaaa
aagcactttttcaaagagtatttctaggaagctccaattcctgacaccaacctttaacaataac
aactc

>chr11_9385424_9385624
aaaggaaagaaagagaaaatacaaacaaaagctgagatttgctgagttcctaccatgtgctacgc
aatgccaacaacaaggtggcatggaataatcctcatgtctgttaagtgggtgggggtaggagtgg
gggtaggagtaggggagtgtcacacatctgcaaacggtaacgttgaacttccaaagtaggtccat
cgggc

>chr11_9587424_9587624
tattctgacctgtattaagcaaatgtgcagctggtaggataagatgcttgaacgtagtagacact
caatagaggactgccaagcagagttgatttctgtagttagaaactagttcatccttaccagtaat
ttttttttcttgtatacactcccaatcggcttatcggcttatcatgtcacacacaactggttac
tctct

>chr11_9587624_9587824
acccaactagatactggatgaggtcatctctcaatcctgtctgagctgaacaaggccttttatgg
aactttaaagttgactcaccactgagtcatcaaaacacagcaatacctaaacacgctcctgttgc
```

taacaaaagaccagcatgggatgtctgtcttacctcctttccctcccctaatgagacacggcccc
ttgac

>chr11_9587824_9588024
tcactggagaagagtcatcacgtcttttgtgctgcagtaatctgaacgaagccagtttaaataat
cacaatgtggccagcaaacaactggcagtccctgtggtcattcaggtcatttcctgtgtatgtca
ctaacccaaaaggagaggcaattaatcaatcacctgaatatttaataagtgcctgcattctgttg
ggcac

>chr11_9588024_9588224
tcacagttccctctgtttatgacatttctgtgaagtgaacttttcagttattgaaactcattaaa
aatatagagaacagtgttgcgggaagtcagggaccccaaacggagggaccacattagaacagcaa
cctttgtgcctctgtaaaggaaataaaaaaaggaatattggagaaaatgggactattcagttcca
ttcca

>chr11_47290224_47290424
agatctgggatgtgcacgaatgactgttctgtccccatattttctgttttcttggccggatggct
gaggcctggtggctgcctcctagaagtggaacagactgagaagggcaaacattcctgggagctgg
gcaaggagatcctcccgtggcattaaaagagagtcaaagggttgcgagttttgtggctactgagc
agtgg

>chr11_57093424_57093624
aacacaccaaggctacaaagaaagcatgaggacatggtttccaaagtcagttccctttattaaat
cattttcacagaccagaaatacaaaatgaaagtagaaataggccccaggtaggagctacaggcct
ggcctcacatgacccctgctccagcaacttgaacaggacaagcagcagctacatccttaaggtcg
ggaaa

>chr11_61582224_61582424
ttggtgcgtttttacagagcactgattggtgcattttacaaacctctagatacagaaaagttctc
taggtccccattcgacccatcaactccagctggcttcacctctcagggctatgagacttccgtcc
cttcttgcgggtctttcaataagtccctttcctctcttagctagggttctccaaaaaccacgaag
aggaa

>chr11_62320024_62320224
aagaaatgactgttaacccttcttagcaccatctgggatctcccagaccccgcctccaccccata
cacacaccagcccagggattagcatccctgccaaacaggcccttctccattcacttccgtcactt
cctaatctgtcattgtccaccccaccccccaaaagcaaatctatctggtgattctggaatccaaa
catta

>chr11_62433824_62434024
gtggcgacatagaccaagtgacaccctccaaccgtgcccacaggctcactggctgatagttgcct
ggcggaccgctgtctctgggatcggctgcatgcccagcctcgtttgggcactgtccccaccttcg
actggttctttggatacgacgaagtccaggggctcctactgccattgctgcaggaggcacaggct
gccag

>chr11_62434024_62434224
tcctctgcgagtgctggatgtgggctgtgggacttccagcctatgtacaggcctctacaccaaat
ctccacacccagtggatgtgctgggggtggacttttctcctgtggctgtggcccacatgaatagc
ctcctggagggtggcccaggccaaacacctctatgccctggacaccctgcctcaagcctccactt
catgc

>chr11_62650424_62650624
actcgtggttctccactcaggttgacactgtggccaccaaggtgaaggtgagtgttggagctgat
ggctggtggaagtcagatgctggggctgggacagtcctttcacagcaagccctgtagacccagcc
tgactccagctgagggtccctttgctccttaggaccaaggaggaatgcctcctcctatagccaaa
ggata

>chr11_65187224_65187424
caatgtggtaaaaccctgtctctactaaaaatacaaaaaaaaaaaaaaaaaattagccaatgtgg
tggtgtaagcctatagtcccagctactcgggaggctgaggcaggaaaatcacttgaacccaggag
ggagggtgcagtgagcagagatcacaccactgcactccagcctgggcaacagagtgagactccat
caaaa

>chr11_65187624_65187824
acttgttttgatctagagaaatctgctgacaccggccttcctgctctcaccctccagctgggcag
aggtatttttagacttatttgtagaggaagagagcagaacccaggcccaatgtgtaccctcctta
ctgtgggcacagctttggccccttaaggcaggacagagatatccatgtggttgtctaccctcagc
tcatc

>chr11_65256424_65256624
atggactgactgttcaggttcaaggggtcttgacctggctcctgaagttggggggctcccactgc
caggagagtggccacgactgtggggagcagtggagggaggtcagtgcaagtagggaaagtaggga
tcttgctgccacaaacagtgggggtgggggtgggggggcaggaacagaggaggctggactgcat
gaggt

>chr11_65259024_65259224
tgtgggaatgggaacttgctacctagaggtttattccactcaaacactgggtggctctgatccac
tattaccagttcaggagaaggccctgtcattctgcgggcttctgttttaatccggcattgttgag
aaacacaccccgacaaagtgcctgaaaccttgtggccgctaccccccttgtcccaggagatgtgtg
tctgt

>chr11_67055424_67055624
gagggttgagaaggcactgtcatggaattaagtgccagtggggagctgtgttcaagatggcagaa
atcaggagaggctggaaccaagaaagtcctgtcggggttgagtttggagagggctcagggaaggg
accgcctctgagaaaaggggtggccagggagggctgcagtagtggtgagcaggtgagctctgggc
attca

>chr11_67121624_67121824
gtcccaagtcgtgtccggcccgccccaccagtcaggaagattccatactgagtaggaaggcagag
caagggtctggtccccccaccacaatgtgtttggatccctagttcttcactggcaaaatctgccc
tgtctctcacaataatgtgagtctgagcagaaaatattaataataaacctaacactcattgcacg
tacca

>chr11_71814552_71814752
gtctctaaactggcagtagtggccatcgtgaattgtgttatgcgttctcgtggccactaggccac
ttttctgggccattgagcgaactcgtaccaaatgccctcgtatgcccttgtgatatactcttcta
aatgccatgcgaggggcgactgggatccaggcctgatgctccagatcgtacggtctgggtgcaca
cacac

>chr11_118783390_118783590
ctgctcctagctgcccccacgttgggagcctctgcttggctcaccagctggcccagcagtgtcac
tgcctagctcaaaaccaccccaaagaggcagtgaggaaatgttttggagggttcatgctcctgag
ctgccagggccccctgtgatgacgtgaaagcccaggaccctgctggggacaacagggcgaacgag
atcct

>chr10_105668010_105668210
tctgggcactgtcaccaacccccggacttcccagtttgtgacctctcccagcagaccctgtgcct
tggtgcccagaggtctggctgctggaatgcccctgaatggaggcacccttgcagtgctgcacaat
gtcctcagcagctacacagtaccgcctgggccgctggccttgtttcagctgcgggatgagtcagt
gcgag

>chr17_7486476_7486676
cctcgccaggcctcggctccccggagactcttgggggtctgggccccaagggtgattcaggtgct
gcccttccccgacctgggctcgcctcccccacctcctcctctgcttcactgtcccgggcccagca
gttgccggcgcactcatgctccgaggcctgagccaaccggaggcgagacaagcacagggccctgc
gcgca

>chr17_17740675_17740875
gaccaggatccaagttataggggtcccaggcctgctgtgttgtgagcaggcctgatgtaactgct
gggccagttacatcactctgcctcaattttcctcatttgtaacatgggcctaatgatgctttctc
acgggcaggctgggaggctagtgacaatgtggaggggagcatgacctgtgagatcagcctgcccc
ctctc

>chr17_38267874_38268074
agaggtggggagtgagttctcagtgccctgagtcccttttagagagaaaggttgaggctggactc
attggcttgtgaaggacttgcagcttcagagcacatgacttctgggtcacccttctgctcagggt

catccctgatgacccaaggtgtcctctggaggcttgttaactcaatctccctccttacgcagggg
ctgtg

>chr17_38268274_38268474
cctcaacattctagtttgatcgcttttgggctgtttcccttgaaaacacgtgccccagcaaggat
tgaaaaactacctattggtactatgctcactacctgggtgacaggttcagtcataccccaaactt
caccattacacaatatacccttgtaacaaacctgcatatgtaccccctgaatctaaaataaaagt
tgaaa

>chr17_38269874_38270074
ctgccagaaagagctgagccaggccaggctgcggagctggaagcctctgtggctccacgctggct
tcccatcccccatcctcccggagaaggcaaaggtggcctctccaactgtggtgtcctttcagtgt
cacaggactggggtaataccagtcacacaactccagtctctatttacctttccgagagcttcagg
aaacc

>chr17_42173474_42173674
gcagggagccccaagcccagcccaagccccacaaagtctccagccaggaaggggaaggcaggata
ccactgcctgggaaggcggaagtgagagaggcaggccaacccattcctgtttctcttctacttct
ttctccaaagaaagccctcactctcctcgctacagcccagggaggtcacgaggggctgggaagac
tcctg

>chr17_42173674_42173874
tggcaaagtggcccactccagcccaggcctgagaaaaaaggaccccgaaatccttctggctacca
gtatcttctgccttcatgagctggcctctctccagaccacccccccagcattaagaggcgagagag
ggaaaaaaaaaacaaaaaacaagttttggttttttttttgttttttttttttttggagatggagtt
ttgct

>chr17_57914418_57914618
ccaggcatggtgacaagcgcctgtaatcccagctacttgggaggctgaggcaggagaatcacttg
aaccctggaggtagaggttgcagtgagccgagatcaggccattgcactccagcctgggggacaga
gtgagactctgtctcaaaaaaaaaaaaagtgaagtgaagtgaatttgagtcagtgtacttaatagg
acaag

>chr17_57919418_57919618
cggtgtgatacccttctttttcagctgttcgtgccttcctttcttgtatccaccaaagtggagac
aaatacatgatctcaaagatacacagtacctacttaattccagctgatgggagaccaaagaattt
gcaagtggatggtttggtatcactgtaaataaaaagagggcctgggaattcttgcgattccatct
ctact

>chr16_14595499_14595699
aatgtgccaccatgttattaaagccatcatggtgagagttcctgggcaggcagcccgctggaatg
ctgagtcatctgttcccccgctcacagctggccctcctccccagcacatgctctgcccacaagca
cactctttggggaatgcattaggaagcagctacctgaatgtatcctgaaaatcacagacaaggca
ctaaa

>chr16_14595699_14595899
cacacacccattggagcctgttctgtcacaactcaagccagagtgcaggcataaatcactgccgt
ttctggaaagagcacaggcacaccttgcactcagctctctagaggccttagctaaatgctgttta
gattatttttaaaaaatgtggttttgactggttcaggatttaaaagagttaacaaacatcatatg
agcag

>chr16_87422099_87422299
ttccaatcagaaagaggagaggagggagaggccgaggaggcctgaggcctggagcaaattctccg
cgtgacttgccgtccatattgagtgtggcaaaaaataaccccgatgctttctgtaggtcaggtta
atgttttgtgattcatttgacaggttcttacacacagctagacagcatgatttacagccacttac
atcac

>chr16_87812499_87812699
gtctgtggtccaggtgcgctgggggcagcgcaggccgggttcaggagagctcggctcagcagcag
ccgatggagctttcctgggggctcctacctggccgccagctccagaagccaagccaggggctctt
cgagacatggtcccaccagaggagagaccgagcctcggagaaatgaacgtcctaggccatgggac
tcgga

>chr16_87812899_87813099
accgcagtatgcgaaggttgcatcaccctcgcggggtctaagaggaaggatggggaccagcccgc
aggcgggcctgagcctcctggcaaagccctttgtcaacgtcaacagaagcatctggcgtcctttc
tggtcgcggacgtcagagctggctgctccggcactcagccgggtggttggtccccaggcagccgc
tgggg

>chr15_93374396_93374596
aagcttttggactgagatatgcgtaagcaaggcaaaaagatcccatagtccaggaatatcaacca
gtccagtttcccaaggaaactaaaagtaactgaaaatgagaagggtgccatataggaaaattgaa
ggtggggcataattattaatacactgctttggaaatgctggtatggaagattcatatatggactt
caaag

>chr14_74208447_74208647
cactttcacccagcacccagaaggggagtaatttcctcctccatttccttcctcagctcctgggc
cttgaagagagtaagagaccctcctcgtgtgcagcctttgtcttttcatatatgaagctggaggg
agggagaggcacagagactaggagggcatccaagtcaccctcacccccagcaagaggggagtggg
gggat

>chr19_4374800_4375000
caccctgcctggcccagctccacatgggtgtgggggaggctgcgggctgcgcctggtggccgtat
ggggcctaggctggccagtgctgcccaatggtgggggctccctcaccgcccgtctggcagaagcc
atgggccacacgctgtcccagcacatggtggcagggcggtgacctgggctcggctctaatgaccg
gcagc

>chr19_4375600_4375800
ggctcctctcccaggaacaaaccacacccagagctccagcccgagcgaacacatctacatgaaaa
tcagcctcatgcatcatgcattgtaatttctcatcttggggaccaggcaggagggctgtgtgaca
tacaactggtaaaatcagtttccgatgaaaacttataaaaggaaaaagccaacccacaaacaaat
gaggc

>chr19_4375800_4376000
gataaatcccctgaggctccgcctggcagggaaacaccagctctgcagcactgagcacaggctgg
tgttcccactgggggcagcatggaggatctgcgtccagggggcagagtgtgatgacatcacccac
atgcacaggaacgataaggctttcttaaaaaacgatcttttctcaacagctcctcaaaaggagct
gaggc

>chr19_8067000_8067200
ctcagccttcttgtccctaaagaacccacgtggttctttgaacacacgatgttctctgaacactg
cgatgttcccacctccgggcttggctgtatcctaaaaagccccaccctattgtctttgcctggtt
agctcctccccaccgctactgcctcctctcctccaggaagcattctgggctctcctgtgcccacc
caggt

>chr19_13276000_13276200
ctaccaagaggacacggcagtctaggagctccctaaggggcaggagtgggctgggagccttggaa
acggtttgtttctcctgggagccctgagaagtcatgttgggggaattaactgctgtgttaattga
ggcttcaagccaagtcccctctgtttacacagagagtctgagaaatgccctcggaagattccgag
agcct

>chr19_13957200_13957400
tctttgggatggctttggggccaggcgattacacttcctaagcctcagttttgtcggctgaaaaa
tggggctgaaaatgcgcaatttgcagttgttaccaggctttgaggtgagacgtgtgatgtgctga
tcgcaggtagaaagagggtaagtgatacaccaggtacacctggatcaagggaccctgacaggccc
agccg

>chr19_13958800_13959000
gtaggggtgccacagagcctaggctgcgctgggggactggggaagcgcatcccatgagaaaggcc
cttagctgggtcaccaggaacccagggaaccatagtgtccaaccacccccaccaatcccgtgggg
gagggggttcaagttctgagagttgagggactgcagccaacatgcctgggttccaatcgagggtc
accca

>chr19_18475400_18475600
gtgacagagcgagactctgtctcaaaacaattggacttaagatggcatgtctttgttttagggcc
aaaaatgaggccccccttgcttaaggcatggggtccgtggccaggcaacggggtgggactgctgcc

ccagatgtggccatgggggtcgtggtcctttcaacaccaagctatgagctgtcgccatggagacc
agggc

>chr19_18475800_18476000
atgtcccggcatgtctgagcaggaggaattccgggcggccaaagggttttatgtagattgctttt
ggacactcgcccaggagtcaggagttgattttatctcacttcctggtaaccttgaacttaccag
gcagggatgagatgacgtaaacttcctcagatgatgtaagttcagccacatcttgctgcccatta
gggag

>chr19_18476000_18476200
agaggacgacccccacagtgaaagtaattgagtgaaatgcacgttctggaaatcagtttctattt
tggcctggaggaatgtggctatactgcttccctgagtgtgacacagttggaatctaatcttgttg
gtttcccaacatctaggtttttgtttgtttgtttgtttttatgatagggtcttgttctgttgacc
aggct

>chr19_18484800_18485000
tgggtgaggctgtcacctctcccttccctgtatggtgctgataagggcgtggggaggcagagaca
ggcagagatagacatgatgagagacaggcagaaatggagggagagccagggagagagagacacaa
agagaaaaagtggaaattctccagatattttgtttagggtgaggacacggagacacagagaggga
cttag

>chr19_39175560_39175760
cttaacccacccaaatggctttttgtttgtttgtttgtttgtttgagacggaatctcgctcttta
gccagggtggaaggcagtggcgtgatctcagctcactgcaacctctgcctcctgggttcaagcga
ttctcctgcctcagactcccaagtagctgtgattacaggcacatgccaccaagcccagctaattt
ttgta

>chr19_39175760_39175960
tttttagtagagatagggtttcaccatgttgaccgggatggtctcgaactcctgacctcgtgatc
cacccaccttggcctcccaaagtgctaggattacaggtgtgagccaccgtgcttggccaacccac
ccaaattgtgtaccctacaccatcctagtgcttgggagccaacacccactcacaggaggctaagg
gcaag

>chr19_39176560_39176760
tcgcatattcacccttccaccagcagggcccagtttacggatcatttgaatactagcaaaaaaac
aagccatctgctggagagattgaacagagggagaaacaggagtgtttgccctgcttccagacacc
cctttcctgtggcagttcctgcacctggcaagatctactggggaattcccggggtcccactagct
cagga

>chr19_39176960_39177160
acttggctatctttttgaaagatcaaagtttaaaccagatgatctgtagtcccccacccccaccc
tgaaacctgagcttagctgtaagcattgaaagtaaatggggtgtttgtagctcaccctccctgtt
ctccaggtgaagggccccgtgtgcctgatcatttcatagcaaaatgctagatggggccagaggag
gcccc

>chr19_46221360_46221560
ggtaccccacctgtccagtcgcatccctccccactcctccgccccgcaggcaataccccagtgca
ccctggtgggcacctgatctggcatacatagaatactcaggacccatctgatccccggtaggagt
gatcgtctccacggtatggatgaagaaactgacgcagggcaggtctcctgaccacaaggtcacac
agagc

>chr19_46221560_46221760
tggagtgtgagctggagcagggatatgaagccagggaatctgatttcctagtcactgtgccctac
tgtttcactatatattccctgggtttcagctcgaccacccctcctctaagaagctgacacccccc
aatccccctaggccaggtcaggcttcacccagggcgtccatgcggtgggctcattgctgtctatt
cctca

>chr19_47611360_47611560
ctgctgaataaataccaattgccagaaatctcaggagtcatcctttttttcttagctgtgctgcca
aggcacaggttaagcgcctggctcaattcagctactttgcttgctattcactctgtcactcaact
ctcggtgtgaatcggacccagttgctctggcactagaattactgttcgtggggaccaaaattaag
ttctc

```
>chr19_47612360_47612560
tgcaacatccattaatttgatttttaactaatactggtacatcagtctgggggaaaaaaggcag
tactgtgacatttcaaaaacagattaccaaaatacagtttttgacttaattactggggcttctag
gtcttaatggatgagatttctttgagaatggaaaacaataaaccgttgtgttgacatgcctgtga
gcttt

>chr19_47612960_47613160
tgcagtttcctcatctacatgtgcgtgaccttttttccttgtaagaaggaaaaaaacaaaacggt
tcttttggagagggccagggttctctaagtatgtcagggatcaaaagtgggaaactgggaaaatg
caacttcaacgtgttttgtcttttaacggaacttatgcttgtaaaacacatcagaagtacagtgt
ttggt

>chr19_49120588_49120788
agaggcggccggttggtgcgactcataaacaacctcttcaacacaacctggttgaatgtggagtt
ggttcttctggccagaaacctgtataactggagggacgggaagacagtgagaagctgggacagcc
tgctcccccgcagccttccagtgaaggggcaaacacatgatgatctggactaaaataatctttat
ccctt

>chr18_3603000_3603200
aacacaagaggacagagctaaggtgtaggaatgcggaagaacttgcagaaactagagctagtctt
cggattgaaatgaattaccaataaagggttgggaaatggatcaaggagagggaagcgttttgatg
gtccctgctccgaggttcagcccatttgcagttgctgataaccttgtcaggtcaccagataaagg
caagt

>chr18_3606000_3606200
gtcgggacattaagaaaaaaaaaggtctcgctgtggtttgctattagatggagagatattcagat
ttcattttaaaatgaaagtgaaagattaaagaattttggcatcctgaccctttgtagaggccat
tcctcagaggcacagtgtgcagacaaaactatcctagaggtgccaaccagaacaagctctaatgt
ctgtc

>chr22_36726054_36726254
aatgtgaacatacatgtatgtacatagccatgcacacacacggatgtatgcacaggcacacacac
ttcaaattccgaaaccccagagccaaattcagtgtctcctctccatccacccgagcgtccctagc
tctcccacttctccagccttccccgtccaattcctgaagccaatttaccactgtcaacacagcct
aacca

>chr22_36726854_36727054
aacgggaggaggccgccagggacaagcctcttctgggacacgagccccctgccagatgaaagtct
agccgtggtaggcgcctgaacccactgccccagcccctctcttggtgagggtggggtagagatgg
ggttatgagtcactctgcagtagttttcagacttttcaaaaagcagcggcagtccttcctcagat
aaaat

>chr22_36767254_36767454
cagaatgtgactggtatttgaggacagggccttggaagaggtaattaagttaaaatgaggtcctt
cgggtaggccctaatcccatggctggtgtccttataagacgaggaagagacaccaggcgtgtgcg
tgcacagagcgaagaccatgtgaggacacagttggcagacggccatctatgagccaaggagagag
ggctc

>chr22_38576654_38576854
ttccactcctttcaattctgcctatgacccaacagtaaaatcatgtatttgtacagggccatact
ggttataaagctttaccttctacattttctcattcaaattttgaacctcatggcagacttacgag
ggcaggaggtacagttcccctgtcacagatgaggaaactgagacttggacagaaaacaggttcca
aactc

>chr20_10492000_10492200
ccatgccatggggctcacactgccctagggatactgtcagggatgttgaactccatgagactgtc
tgttcacctgactcccaggcagacagtaaggcgatcaggcttttccttaagttgagctgaaagct
gaaatctgcctccttgaaacctacctccagctgttttaattttgccagcagagtatgtccagctc
ttcac

>chr20_45946793_45946993
tcctccccgactaccgccccccactccaaaaacacagcttgctttctggaaggagcatgttaaat
caattccacaagttcaaagctatggaaaacaccggcctgtgtagactggagaccagaatcagccc
```

aggacaggatttctgcatttcaatcagactgaaaatgccaggtttcccccagtaactgtgaaggc
agggt

>chr20_45977993_45978193
aaaggtagttgggtttaaatgagaatattaacacatttagccatctgattttgaaccccgttaac
aatttcatttacaccgcatctgtaaagaaagcctgtgacacaaatcaccaaagaaacaactgaca
tgttgtcacaaatggttttcatcaggagaaagcagcctgctaaatttaaacacatcgcttatatg
caata

>chr20_45978593_45978793
aaaaaacccacttgaaggatttctgcagcgatgaaaacattcaaaggtgtattgatcatggagga
aggctgcattcaagggaaccattatttggcttgtaacagcctagaggagctgaatacttcaggca
ggaatgctgctgagagacgaggaaaatgcaaactagttatgtggtggctatttgcttctgagttt
taaga

>chr20_45988193_45988393
ctggggcaggcctacgaagggctggtcctgggcttcatggaatgtggctgataagcaggaaataa
aaccaaatgttgcaggtctcaatctagaaatccattcagcagctcctcttaaagccccactgcag
tgagtatcccagcagttacttctaaattagtgctttacatagactgattcctgaagtaggtggca
ttgtt

>chr20_45988793_45988993
attcaaatgcccctcattacagattgaggcccagaggaagcgaccctctccctcccaacctccag
caagatgcaagatgtcaaggggaacactttgccatctctaaaattaggcttttatattccctga
aaccagtggttcaaccatgggggatttgcccttcagggggcatttggcaatgcttggagacgtg
tgggt

>chr20_52239793_52239993
caatgggtggttattactaacaccagtaggaaaaaatatcacaggagcttcatgacaaatcctta
ggcagggtttaggtctcactaatttagaatatgcctggtaagcactggttaaaaattgatgatcc
cgggaagtctttctcaaacgatttttgtaaaatacctaggagaccttgaactgtctgttacggag
gtttc

>chr21_35319930_35320130
gatacatagcccaggggtattcccattcctccacgacacaaatgacaacttccttgagctctgat
ggcttgccaggtggctgatggcgtggagaaccttgctcatgtttacctgttcttgcatccattac
tcacctctgcccacgtgcccccacacttgcacacaaagccacagagtggcttaaaaaaaaaaaat
gaaaa

>chr7_1083274_1083474
ggcgtgggccgtggtgagcctctccctgccctcccgctggaatagaaagctgatcccgacgtccc
actccgacccacggtcaggaagccgtcctgtccaggaaggtggctgaggtacatccctgaacaca
gtaggccacagcatcatgcagcggagggccagtggccgctattcttcctgctggtgttccactcg
gcccg

>chr7_44678475_44678675
tgggtggtcaatgactcagcctagtcttgatttatcccccaggatggggacagacagcaggggag
agatctcaggttgagatgagtgtacagtaagggctgggagtatcagaaggggaaacgctctccca
gatagtgacaaagctaagcgaccatgtgaagggagaggacaaacatcccatggaaagaagggtag
ctgcc

>chr7_44678675_44678875
aggcctgcgggaaggaaggagcatgatgctggggcatgggagcaagtggacagggagggctctgc
aggtcccctggaacctaaagccaggggttatggaaggcagatccagattcaggtgttcagaggat
gaggcaggggtgggtagtttaaggagctggagaagaggtatctccttggggagtggaagatgtta
gagag

>chr7_100143464_100143664
ggaggaagggggctctcgtttctaaattgcttcttttggccagcagtggcctctctgtgattcag
aaagaaaaactctgactcataggccatgtaactaggccccccttcctaggctgcagtgttgccttg
tagagaattctccctgcccccagactgtggctctggttgtacaaaatccagtctacagacatcaa
ggtca

>chr7_104585964_104586164
acttgctcagagtcctactgccagtgggtgtgaaagccagaaccaccaccctcattgccttctgt
atacatagctttaacagtttgcaaacactcttatatgctttaatttcatctgatcctggactgag
agcaaatatttgtatttcaaccaaaaggccaaatgctcagtttcctcatctgagaggataataag
agtac

>chr6_7146801_7147001
ttaaaagccagtttgggtctgatataagttctgcttcgtgtttgtgtccattcactccgtaaaca
taccatatatagtcatgtttgagctcactataacacacattgcaagggagccgctcatagtgcac
acatcctttggctgttagaagcagactcacataggctatttctggtgtgtcgtggctttcttctt
tcttt

>chr6_7147001_7147201
tttttttttggtggggaggaggtggtgggggagggagaggaaaaggagctctttttccttccat
cttcacactggttggaagatgtttctgattctagtttgtctggcactctgaaagctaaggaactg
tattgtcatggcagaagaaacaattctctgtgtgcatctgttttagaatgaaaccgtacttttct
tagta

>chr6_26026021_26026221
atctcatctggtctcagcatttatttatctgctgatgactcacacatttttatctccagctcaga
cctctcacccgaactcacttattcaactgcctattcactatatcctcctgtcaggagaagcttgt
tagcaaagtaaccagaaaacaaagttgattttttttgctgaagtagccatcaatatatttgttac
taaat

>chr6_29932421_29932621
ggacacaaaacgtccccaaagcattcaagatccaacaacaagaagaggagactccctctgcattt
ctgcagaggctcagggatcaagtgaaaaaatattcaggattaaatccagaggacccagtagggca
aggccttttaaaggttaattttgtaactaaaagctgatgtaatattactaagaaactgcaaaaga
ttaac

>chr6_35438022_35438222
aggtttaaataaggcaggaaagttcccttccctgctcacacacaacgaaaacatggtggccaaag
tggatgaggtgaagtccacaatcaagttccaaatgaagaaggtgagtgggtctggcgggttgcta
tgggtgaaggtgttggcagggtctaaatcttatccaagtctctaaatatgccagtaagagcaccc
accag

>chr6_43141422_43141622
tccagagatcctgataggacaccctgccctcagagtcattagagacgaaggtcattatgggaatg
ggggaatgacatcactgtataattctttttccaacttctcaaggaaggtagagataaaaagtttg
ctgacctgcccatctccctcctcacccctcaggcctatgagctgtccacgctgacagggacacag
gtgct

>chr5_172191995_172192195
acacatgaatgaatgaatgaatgaacgaatgagtgaatgaatgcgtgggcctcctgcctagcttt
aaaagccacattcaaggaaaaggtggctatgagcttatcttccttggcagagcagttttgtccac
tgtggcgctagcagagagcacaccctttgttctccacggggtggaagacacctcctcggtgggca
gaggg

>chr5_179246794_179246994
gggaaggatcattcacacctgtggaccagcccctgctgtgcgcacacccacacatcaccttcgca
cctgactggccccatccagccactcttgcctccctctgggtttcctcccctgggaggtttctcca
gctcctgcaagccctgggctgaaatggcatgagttggacccagcaggttctgacctcctactcac
aggac

>chr5_180668994_180669194
cctcagcaatgctgagtaacttatttaaagtgagggaggccaaacatcttctgtaggtgtgctct
gctttccagttcccaaatggactggatctcccctgggaaaccagccaattgcatccacctcactt
ctgcccagattcttcccaaggccccagagctaagtgagcagctacttgcgttgtgagatcccaga
ggcgc

>chr4_38673805_38674005
ttcagagggaaagggagatgattgtggaactcctactataagctaagtgctcttatgttagctcc
ttaggcttgacataaaaacttcattcagggaacaagcccgatctcctgtggggagtggggaggga

gccctctcaggaaggctgaggcctgcctgtctgtccttcccagtactggtggcggtgtgggacgc
accac

>chr3_39193396_39193596
gaaactgcacctgacaatgactgcttcctccacctaagtggggccttcttccctctctcattcat
tcattcaacaaatacacactaagctcacatatgtgccaaacactgtggcaggcactcgcgcatgc
agggagcaagacttatacaactgctgccctcttgaagctctgaattgagcagggaggacaaatag
agtgg

>chr3_48507396_48507596
tggcaccactgccctggtccttccagctgcctgtcactggtatgatggccccggtgcattgtgcc
accagcaggccacagctgtggatcttggaaggcctctggggtcccccgggagcaggggagtgggt
gtggggggaacggatggtggtgagagggacagaccaggcaggctgacgagcagggcgggcctgg
ctcac

>chr3_52028560_52028760
agtttgtgattcactccacggaacagagagcaaaagtaaagggaaggaacagactggggacatgc
tcagctctcccctagaccctccagccttctcagctaatccttagaacccccctgacacaggaagag
aagctgaccccaactccagcactgggtcagatggaggtcataaggctggtcagtgtgggaatgtg
ccctg

>chr3_53304160_53304360
aggcctggggctggagaggcctggaactcctcggaggtaagtgccagtgtgaggccagccaagcc
ctgccgggcctgctttccttgtgcataaagaaaacatgccgagtcattgtgacttgcacttttttg
actagtgcggtcaggctctgtgtggcactgccgtaggcctgggtacctcctagtgtgaggagcag
gagct

>chr3_171855906_171856106
cctttaagactttaggcctctttcttccaagagctctctcagaccactgcctgacttttccctga
attctggaggagcaggtagaggtagcactacacagtctggttatatgcgtaaacctttccttagc
tctcgaattagatgtgaattcagttgtgacaggaaacagtgtatcttcaccctgctccccagcca
cacag

>chr2_43446496_43446696
cagacacactcacatccaaacacagccatacccacagaccctcccccacatagcctccacatgc
agaagcacacagatccacacaccagtactcccctagggcgacatcacacatgcagacccctgaac
acctgcagacggagcaacaccacaaacctctgcacacccacagacagaaaaggacgcagaaacac
gtact

>chr2_87785085_87785285
ctagaacgtgacctgaatgtgaactagacattcgttttcctaaaccccatgagacccaggtattc
ttgacatgtgtagagaggattgaaggggcccgaggctgtctgtcctctctccgtgggcctggctc
tccttggaccaaatcgaatgcatgggaacacaccccccagttgtctttttccttcggtttatata
attcc

>chr2_101620368_101620568
aaaaaaggattgttactgcctagtttaagggttggtttaggctggttttaggaggacataccatc
tacagtaagtcttcactgagctttaagcacactggcctactgtatgctgtaaaaacttggttttg
ttacataagacattgcttaaagtcagtttccaggagcctatcatcgacattgaggatttctcaca
tgtac

>chr2_112221329_112221529
tcactatgttgcccaggccggtctcaaacttctgggctcaagcaatcatcccacttcgaccttcc
aaagtgctgggattacaggcgtgagccaccatgcctagctaagctctttagaaatgatttactcc
catttttgaagtttgccatgtcacagaaaatgagaccccactgagagattcaaatgtgcgggaa
gaaag

>chr2_112221729_112221929
acccacacaggctgggtttcagtttcaccagggagcaaacaccattatgcaaattattttccttg
ctcaactccccatcccccatcactggcttacatattcattcttcactctgaagttaatgtacaca
agaggccaactaaggatttgcatcctatttactttaaaatgcccaggtgcaattttgaaacaaga
ttctc

>chr2_219270156_219270356
atatgctaggcccaccagctaagtgtgtgtgggggtctctacgccagctcatcagtgcctcctt
gcccatccttcaccggtgcctttgggggatctgtaggaggtgggaccttctgtggggtttgggga
tctccaggaagcccgaccaagctgtccccttcccctgtgccaacccatctcctacagccccctgc
ctgat

>chr2_219270756_219270956
cacacctagagggctgttggtggagggaggggctgcccggccctcgacacttcaggtgggaaggg
cagcgtcagagcacaaatttgagcctccaggctgtgctcgtctacgtcttcccgcctcgggtatg
tggtctgcaaaatggagatgtgccctattggcaggactaattaagtgcctggacacagacgacag
gatac

>chr1_8773013_8773213
atggaggactatagtttattcttcccagccaaggattaatgtccctagggaaaaggccaaaaagg
tactcatataactagaaaagcacattatcctaaaataagacttgttataaaatctgaatcataaa
tcttacaagatacctttcaacattaaataggaactaatttagtaactggaggaggaacagtttgt
ctttt

>chr1_8933413_8933613
agatgaaaggttctgagcaagtccatcatttcatgtttttttgaaaaggattaagacacacaagag
aaagttccattcatttcaatttcaaaatgtccaactcccaacaatcttctagcatctggggtctt
ttgttgcttaaagttcattaatatccaagtagaaagttccacccattgactaagcaaaccactct
tactg

>chr1_24307213_24307413
cccggttggtccaagttaatgtcctgcttttattttgccttaaatttcccgtttggtccaggtta
tgccctgacctgcgaaagaaggatttacagcttgtattgagtgtgtaaaggaaggaggtgtgtgc
ccttgcgcagagctgggcctgtctctccagtgttttcagcctgcctttgcccctcacccacaccc
tagcc

>chr1_27049613_27049813
tggctggggagagaagggacaatgagaatgatttatgctggtcattttatgtaccagggtacata
gggtgatttttttggtggaagcctagtctgtcctaagatattactcctgagatctaagtgagcca
cgtgcagctggcaggaagctgaaagctgttggttgggagaggccttaatttttacacttgcccct
gcctg

>chr1_27050013_27050213
tctcggctctttctgggttatcaggcgccagtccaagtcctcccaggcagccagcttcatggcca
gagattacgagcactaaccttcctggcaggggcggtgtggcttcgagtttatgcagattagccat
gtgtttctcacttcaaggcgttccacaggaaaagccttttcctgtgtttggctctcagtgggcgg
ttccc

>chr1_27113213_27113413
tgccacgttactcacaggcctctggagctgagaacaagtgcgtggcactgaaaaacctgggggaa
gggccatgaaactccatttttttccccttctccaacacacaacgactaaatatgatttgcctttt
ctccaccttgcacacaacagatatctgaaggcaatttaggtctcagggcctgaaatgagttacag
agtca

>chr1_28835413_28835613
gtaggtaaactgatttttttttttaacagggagggtttgacaatctttggcagacttggagcaaa
agattgaggtgcatttcatgcctccttttgagagtcttgctctgtcgcccaggctgtagtgcagt
ggcgcaatcttggctgcaacctcagcctcccaagtagctgggattacaaacataagccaccacgc
ccagc

>chr1_36839413_36839613
tccaaatgctgcactttattgccttgggggttataagtgaactggctgcttctaaccagagctct
ctttgtataggatgaccccacccccctgaggcctgctctgctcccagcctgtgggagagtacctgc
tgcagccagagaggaagtgactcagcccagtgggaagtgggaggggcggtcagcaagagggccct
gcgcc

>chr1_36852413_36852613
tgtctttccagctgtcccctgggtcactctatatccatgtgtgggcacacgtgtctaagcttgtg
tgcactgctgggttttcctagggtgtgtggccacttgtgtgtatgtgcatttgtcatctccacat

aggcagtgccaggacacaagagctgatatgagtgaagtctggctgcctctacctgtgtcctttgt
ctctg

>chr1_36852613_36852813
aatgcctggtgcccactgagctgctgatagggctgactaacaagagtaatcaggacagctcccac
cgttgtgccacactctgcatggagcacttgagacagtattaggtaggaggctgtttacagctgag
gaagccagtgctcagagaaggaaattaatgtgcccaaacacacacagctaataatggcagggcta
ggatt

>chr1_37943813_37944013
gggacctgacctattgtggcaggtcagggaaggcttctcagaggcagtgacatctcacaagggtt
ctgaaggaagagcaggagtttgccagcagcattgtttctcccgttcgccacctgtctctcataac
acactttgttcaaactcaattttgggagctttgttctcccaaataaattctggatcttctgttag
agaca

>chr1_38465213_38465413
tgccagttcagagtaggtggggatggggaaggggtgcttctgggaaaccaggaatcccacatgtt
tctttctgtatttaactcggggtaggaagaggtcaggaaaaaaaccaggaagcagaagttgtttt
cttatctaggccctagcagatctagggggaggggctttttcctgggtcagtgactcatttcctgt
cccag

>chr1_53791212_53791412
aggcaggagggaaagatgtgctagagacatagactcagtcagtctagttctgccacgcacaagac
acaaagccaactttgggttaggtctgaaagtgaacgggagggtgactggcaaacagtcaaaaacc
aaaacgattcaacagagagggtgcctgtccaagtgtcccgtgacccttcctgtgcagtgtggggc
tgtgg

>chr1_150536376_150536576
gctgtgctgagaggcctgggaagagccctgtgctgggggaggaaggctaggttgggtcggcatgt
ttgtgtgctgtactcttctgagccttacctgaatctcttgagatagctccactgaactttgagtg
aagaggaaaagaaactgggggaaagagggtgggattataaaaaaaggagcagactgtacccctgg
gatcc

>chr1_150541176_150541376
cagtgacctaccttaggtgatgctcttaaaagagcaactttattcaaaccaggcctgccaatgcc
aactccttcagtctagatccaaagatggctatgatcctgagattgtgtgtttagggtcaccactt
taacatgatacaggaagcaattgagtcttttctacttcctaaacacagtccatccagttgtgtgt
cattt

>chr1_156074976_156075176
tgctcctctggggcgggcctgtgggctggcttagggatagcagacaggggaatctggggagttgg
actaatgtggatctagaagggaggtggttgggctgcacgatgccttaagactcctcccagctctg
aagttctacttctaggcaatgaatgatgaaacgctggcccaagtgcagtcccttccccatcccac
agggc

>chr1_156092576_156092776
acggtgaccttgggctgcccaccttctacccttccagcacccatactggctcccccaacctgcgg
ctgggctgggaggaggtcttggcccctaccaatcccttaaggaaggggaaagagtttgggaaggg
gagtcctcccttcacccctgcctcccccaagttgtgagagaggaagccggaatcctgcctgctga
agcca

>chr1_156093376_156093576
actatcttcttaggccatccctccctcctccaggcactacttcttttgctctatccccaagcccc
accccctgcatttttgtgacaacaccggaatgatttctagagagagaggccaggaagaaggaaagt
ggcacttggcaggagaccttgcagggggcggctggtgaggaagccagccgcccattgtccaggac
cccag

>chr1_156099776_156099976
agggcacccagactgggtcctgagaaaggaaagggtcaatattgtgcctggtcatccttgtctga
ggtccctctgagctctaaccagactttccttccccacagtcccacatgtgtaaaaggactagga
gaggtgaccagtacctttggggctcagatcgagaagtgctagggacatgtgggccatgagcttag
ttgtc

>chr1_156718176_156718376
cctttgtatccaggctgtgactcaaaacaagtgagtcaaggctaactgggtcagggtggggccaa
atagtgctggggaccccacggtacccctccactagccagcaataggggaactcggctacctctt
aaccattccagatccaggcaagttacttattcttaggccttgcatttgcccgattccatcagaca
ggcct

>chr1_161089176_161089376
aggtatcagggaaatagcgagggagaggtagaatctttgttggacagtgtccctgggttgccaga
tatgagggtagagccttgagaagtcagtgaagagttgtcagtgtcccttccccccagggaatgtg
gactctggctgtccttgggtggtttccatgagcgtggccaagactgggagcagactcagaaaatc
tacaa

>chr1_234747577_234747777
tttttacaggctaaccttgaatgtcttttggggatttccatagaaggtgcgcctgttttaaaggt
ctctgcatctgttcctggatcttgccctgcaattgcaatcctgcctcaccctctccaagctggga
gaatggggcctgtccagatccttcccgggagtagctggtgccagcgggtttggtcactcccaggc
cctgc

>chr1_234859577_234859777
gcttcataaggaagcacgagctggggcagttggaaaccacatccctgtgagtgctgattacagcc
tactaaggaaaatgagatttttatctcacaggaattcagttataaaaataaaatacagcgtctgg
cttgattcagctaaatgtacaagcactagggtgtttttaatttaacagagaagctggacaagaag
aatga

>chr1_234859977_234860177
cagttggaggagagaggagaaaaacaactcggagctgtgctaatcctgtttgctgccctgaaggg
aatggaaggcggccaaggcagggagttctgggaaaccatataagggtgccaaacaagcattgccg
ccaacattaggaactgacagctttgttctttgtaacttggctgcccgccagaggcctcatttcag
ctctg

>chr9_91932380_91932580
ctgctagaggctgtgtagtgaactgagacccaccaggacagaaatctgaaaaaactggagctgca
cctgccttacttgcttgaaatacttcgtattcaatggtagagttaacgtctgtgcaagggacaga
ggaaacctacaatttgcctcaacctaatccccattcctcaaatagcctgttttgctgacattttc
tgttc

>chr9_131903179_131903379
ctgccctcctggaggagagagcctggggtgattctgagtgaagcgagtggtccaagacaaggccc
ccagggaccagagtgggctttgtctctgttgggtgcctcgggctcttgcctggctgtggtggagc
ctgggagagaagaggaggggagggaggagcctgttgtccttgctcctgaacatgggtgtttggat
gggct

>chr9_131903579_131903779
gagagcgtttctggcttatcttttctccttcaaaaaataacccagtctcccctgacacccgcatg
tttctagaaccttccaagtctctgtttctttgccagattcagcctctttgcagagtcttccttct
gcagcatgggtctggggtacagagccctgttcccctgtaaatgctgcctatgagctggggggctg
tgtcg

>chr9_131903779_131903979
aggggaattatgaactgcttggttaataaatacaaatgtgctggacttgcggtgtcaggggggata
tgctcaggcacacaggcgtccctctgtggtttctgacgtcatgggaacttcatgctggctgcaga
atctttctcttccgtcctggagggtcagactctttggcaagagggaggaccaggaatggagccgt
ccttg

>chr9_136008579_136008779
cctcagggcctaatcctggggctcctcctttcggagccagggtgggcagggctacccggggcagt
gggcactgagcttgctggaaaggccatcgagggactgctgacacagtcaggctgcaggaggagca
gcaccagctgccacgcagaaatgcaggagtcccgaggaggtggtgctggcaggaggggagcagca
gggcc

>chr8_95449024_95449224
tggcttcacaagctttagcaaatcaagcttttcggataaggtaaaattggaagatctagcctttc
ctaccattgttcttttcaaattaagtaggcagtttctgaaaccaccatgcagccttcagctctgc

tcagctcttttcacaaacagaaaaagatcaggcttaactaggctagcctcatttaggcccacgcc
tacag

>chr8_103800824_103801024
gccacttgctaacggtgtgccctaagggtagttccataacctctctgagcttcgattttgtcttc
tgtgcaaaatagggctgaagcacctcacaagattatcacagagatactctgctactggactgtct
tccttcccttctcagctctctaatgaacgctctggcccacattttgggttgaggcctcccaggca
ggccc

>chr8_103801024_103801224
acccgctttgttcctgccctcttccctccctgttcacctagaagggacccaagagcagctcccag
gaaggcctgccccatcagagctggctagcaaggggcctgcggggttgctgagaagacaccactgc
attctcacccagtgacctttccccagggctcctagggagtgggtgctcacatctccccttcccag
ggtac

>chr8_103801624_103801824
ggagacattgctcaaagagaggcccagggccttttcccagccccgggggccgccctggagccag
gcctcagaggtctcagctctggagtccctgccagctgcttctccagaatttgcttctctgccttc
tcctactggggtggagatgaagagtatctaggatggccagttggagcagataacaatacaggatg
accag

>chr13_99136999_99137199
cctgatccttctggggacgtgcaagcaacaacagaacatccagagctctctacccagagcaaacc
ccgcctcaggaccaaggccgcctccttcctggagaggaagatggagaaataacacagccttcccg
cttccctccaactcattcctggtagcagacgcgctgggtggcaaggctacccagacacaaggctc
accag

>chr13_99137199_99137399
agggcactcaggaaaccagctcaagtccttattgaggtcactgcgtctcaaatacccgggctggg
cctgtgactcacacaccagcaccaacaaccaggggggtgggatattgcatcaggaaaagctccact
ctgggtggaacctgcttttcaacctcattgggtgtgtcaccgcccagtgggttcaccttgcccgc
tgcct

>chr12_14410733_14410933
agagaaaatccagcatggacttcgcagaaagaactggatttggcctggactcgctggagcctaaa
gctgctgccagtagtggcaaagtcagatgaaatataaggcctggagcgtggacagtggcctcttc
cagaaagagaaatcacacagcactgtctctgtgccataatccagtccgcattttctgtcttgctg
ctcct

>chr12_31901933_31902133
aatatggtggttcaaaattagtatttttcctctttgtaggggaaatcacccaaaagtaacaatca
gaactgttaccacctgactggttcttgcccactgcacagataaaaccaaaacactgagccagcag
gagttgcagcagagaaagagtttgattattgcaaggcaatagagtgaggaggacgggatacattt
cccag

>chr12_46776533_46776733
tttgttgtgtaaccatttgtcacttctacatggatgaggtcaacgaaatcctcaaagttgctgtc
tcaggaataaattttgttcgtggttgggaaaacgaagtggagaatgttatgggatagagaaaata
gcttattttctcagtggttaagtgggagtgggtaaaacacaattggccttgcatgtgctttgttt
aaacg

>chr12_109085271_109085471
ttctgctggcagcaattgtcagcctgcttagcacagactggcacgctccacaaacccagtaaaga
gttaaacgttctttgcacttgactcagtggcttctcttcaccacacactcccagatcccaccatt
ctctgagccacccgtgcagccaatcaaagcaaaggaaggaaattatcctggccggctgacagcca
cagcg

>chr12_109085471_109085671
ctctgatggccatatatggtaaaaaccactgtcagagccctgtcaggcctgcgggccggctgctg
tggacatatcagtgaactgcagcagcaggagaagctggccaaggaaacgcacaaaggctgcctgc
tcatctccaggggagggggtggggtggaaaccatcatctgccagggcacacattcagtgagtcac
agcca

>chr12_109085671_109085871
ctcactgtcatcaaaaatagccccggggtacattgggaagttccttctgccaggactttgtgctc
cattgttaactgggggtgagcagggtgcccaccccacccctcccagcaaaactgtggataacaca
gtcactaggagaaaatgaaaaggcccttctgtggcacttcatgtggtaatgaggccaggccgtgc
tcgtg

>chr12_109086071_109086271
ctctgaacaaaatcctctgcccctaacaccaaagctaccaagtgccaaatgtccacatctgtaca
aagggctaccggcactgccactgttgtctaaaacagagtaccacaatgtgtagaaatggaaattg
cctgtcagcaatgcacagtaaggcagacatgcagccaatgcaaatccagcaggtgctggcaaaga
ctgtg

>chr12_109232071_109232271
cgtgttttccagaacagaagtggccctgtttcgtgcccagagcagaagagaacgatgaagagctc
tgctctcccaggtcttcctggtctgtgtgtgtccaggttttgagggcctctcacatacacggctc
tggaccacgtaagatctaattttagcattttcctgctcggagaccacatgtttggaacagcaggg
gctga

>chr12_122883847_122884047
gtcctgttttgggtatcaaatccaaatcctgtaagcaaaaggtcaaggattcagaagtgaaagga
aagactgtggctctaactttgccaaagtgaaaagtacagacctctgccatcagtgaagaaagaac
ccagatctgataagagcttcaaacaggctcagcacctcctggcacacatagagcggcatcacgtt
gcccc

>chr12_122884247_122884447
cgtccagaaacccaggtttcaacgcggccctgcttcaagacccaaaggaaattcagcaccaggcg
ggtgaagtgacttacctgtgcacagctgcactcgcagacttcggagcccccacctaattctaaaa
tgctgtatgtccatgaacttaataacagaaggtcaggggagagacgttctgagagcaacggaaat
ctatc

>chr12_122884447_122884647
ttaagataaagatgtttctgaggctgatttttcaagtcaaacttgagagagaaattcaaaggcta
tgttctcatatcatattcctgggtttgggaggttcattcacagcttaatcctgtcccctgaagtc
acggttacgtaagtatgctgtgtcacttgctgtccccacccccaacacaggagatgcagtgtaac
ctgag

>chr12_125402847_125403047
cctgcggtattttttttccttgtcatgcactagtagaaatatttggctttgtttctttgtttttt
gaaacagtatctcgctctgtcatgcagtggtatgatcacggctcactctgcagcctggatgttcc
ggatcaggtgatcctcccacctcagccttctgtgtaggtaggactacaggtatgcgccaccacac
ctaat

>chr12_125403047_125403247
ttttgcatttttttgtagagaggagggtttcgcactttgggaggctgaggccggtggatcacctga
ggtcaggtgttcgagaccaacctgtccaacatgctgaaactccatctctactaaaaatacaaaaa
ttatcaggtgtggtagctcacgcctgtaatcccagctactccagaggctgaggcaggagaatcac
ttgag

>chr11_355600_355800
accgtctggaaatgctgcccagtggatttcagcctcattttacccagctcccaatcaagatggaa
ttgctctggttcaaacgcctagcttcgccagctagggggtgaaccggccacagatccccggaggc
caacagaagacagagcccctgggtcagaggcaaggacggttgacttctcaggcagggaggtggcc
aaggg

>chr11_46576224_46576424
tgtaaagaacaaagggagctaagtcaaactgaagtcacacagggctgctgatgaggagaaaatgc
cagtcaattgtgtttttcattcctcttttaaaaaggaagaagaaacaagtttcccaatctgtccc
ttaaagtaaagcagactgagacaaaggttaccaacaatagtctttgtgataccgtcacacagtcc
ccaaa

>chr11_47290424_47290624
agccctcgctaacactgtgctgtgtctgaagatcatgctgaccccacaaacggatgggcctgggg
gccactttgcacagggttctccagagccctgcccatcctgcctccaccacttcctgtttttccca

caggggccccaagaaaaattctccactgtcactctgtggtatggccataaatgcctcagcgccgcc
tcact

>chr11_57093224_57093424
taactgggagaaacttcctaatcagatcagctctcatttctagactctcaggtggcccatccggg
cagaggcgggtgaaaaccaatgctcgtccagccgggaacagaacaggatgtacctcgagacttgc
ttcccggctggccaggaaggccctgcccagtctgaccagagcagtaaacaccaccaataaaagtg
ggagc

>chr11_57093624_57093824
gtaagatgaggatttggatcctgcattgccctgcctcccaccctatctctccccaaattataaac
agccatccttgggaagcagcagagttaagacgtctccccactgccctagtgacatacacaccaac
aggagagcatgttcagatggcacagaatccagggactgcatttcatgaggagaaactggtaccaa
aatat

>chr11_62320424_62320624
ttcaagcccaaccagaacccaagttaaagctggttagagaacaagagacatgaggctgtctccag
agtaccgtctgggaggaaagaaggcaaagaaagactgtccaggcaggtggaagggaaagaaattg
ggcagggagagtggggcaggtggacacgatgggtcatggctggatttcaggcagagttaggggtg
tggga

>chr11_62320624_62320824
atgctgtggggcaataagaggagatgcagacctgagattaaggaaaagaagcgacaagacatgag
gctgtgaaggaaggagggagggagagagagaaaacagaagagctcagcactgagctaaggactgg
gcgggctgagttgtaaacaggagccgagggagggagttggagtaaacggagcagccaacgcccca
gccag

>chr11_62323824_62324024
aaggatccagcaagccatggagagcccagcggggatgggaagggcaagagcccttgctgcagtga
ctcaggcctccctggggcagggcgggggacagaaagaactccagatgtgcttcccctttcctgcc
tcacatctgccgttagaaacttccactgttaacctctcccctacccaccacagcagagaagccct
tgcat

>chr11_62650224_62650424
atctgtttcaatggaatgaattttgcccgggttcaaattcaagcttttgtaccaagtaatacata
ggaaacactcagtgacttgcatttgtgatttccttattttcccttaactcatagactgtctcatg
attgcgtcttcctccgttgttttaggcatccgtgtcattctggaccttactcccaactaccgggg
tgaga

>chr11_65259224_65259424
gggatgtgggccacacttgcatgaggaagaatgaatatacgtgtgtgtgtgtgtgtgtgtgtgtg
agcatgctcatggaggatgtttaatcagcttcatgctgggtgtgccaggccaatacaaacatctg
ccagggacctgaagcatgactgaaaagatcagggacacaagaacgcggctgcgactccctgagag
cttca

>chr11_71751152_71751352
ccgtctggtccaagtccaaagagtttcctgaggtcggggcaaaccagtttggtccatctaaaggg
actagcttgggctgaagcctcctcttcacacagaccactccatctttgtagcctagagtttggca
cagaaccccaaagccctggagaggaacaagggagggattacagaggaaaccaagcttctctggtt
tcttc

>chr11_118783190_118783390
gctcccccaccccgagtgacgcgagggtaggagctagcagcctaagggttgctaagggttaaagt
ggacttgatcctagctgggggagggagctgaggaaggctcagatgcccagagcctccaggtatga
cacaccggctctgctccacacacaactaccacaggtagggcaggcgttcacacatccagtggcaa
cctgc

>chr10_74014794_74014994
gcttcctctgctctgggtcctgggaaggggcttggcctccagaaggggccagatgccctggcctc
tcaccccaggcctggaagttagcctggcctgctagcgggcagcccaaaagagccagcagagaacc
agggcacgctcccaggaacaccagggctggtagcaagccgacctcctcccgcggaggctggggtt
tggcc

>chr10_103911610_103911810
tcttctgcagcagaggcaggagtctggaggctgggatggtatcagaaagggcctccctgggtatt
ggaggcagtgaagatgaagggcgagaatccattggccgtgccagtacgccatgggggcggttggg
agatcttgtagaacaactgttcgagcccgcttgtccactcattactctggaatccgagtcccagc
agggg

>chr17_2299850_2300050
gcaccccaccacccgctcagcatccctgctgaaagcccagcaggaaggcaaggcccactcaggag
ctggctgccaacaggggaaggggaggcacctccatctcccgcttgcctcgtcctctcccaggcgc
caccattctggttccctggctacggggctggcccaggcccttggagtctgcagactccaggccag
ggagc

>chr17_7486676_7486876
acccggcacctaaggaggcctgcccggtgcagactctcctgctcccaccggcgcccttccctcta
gagacgctgagagaacgggagctagtagcgcccccacccaacgccacctcggagactccggctcc
ttctctctcaacttcgaacaatacaaagtgtgctaggagaagacaagatggcgcccagcaggagg
agcgg

>chr17_17740475_17740675
agtcattcattttatccagcctcgcgcacaatggcagcggcctctggccggctgctgcgggaacg
cgggcaagccgagccccctctctgggcctctgtttctccagcaccaagcaaggaagccgagtgga
agccccaggcctgggcccatcccaaacttcatttcctcatttcctagatttgactctttagaaca
agaca

>chr17_17744275_17744475
agatgacgctcgtggtctcattccagcttgggctctgtggtgagctgggcgagttctttccagcc
ctctctgagcctccatggccagctttgcccctggaggctcacacccatgtgggcacctgcaggct
tatcatgagtcactgaacacaccctctaccaaagagctacttgaaccccaaagattgctgcacag
tgagg

>chr17_27135474_27135674
actgtaaactactaagggttaaactgattgagagaaaatttgcctaagcaaccattctttaggaa
gagttcagtaactcaccaacaataaattctacatcctcatctgagtggtaaacagattacagact
ccatcaagtcctctactttcaggattcagactcatagcaaacaacttcctcaaaagattcagctt
ccaag

>chr17_27135674_27135874
agtcaaaccctggggggaggggattgtgcttaaacacacaaaatattgtctctgacacctgactga
aaaagctgaaaagctaaaaactaccatcacccacatctccatatgaatgcatccattgttctaaa
taagtctctgacaattgacagagcattctcagagtctcctgtgcctctaatattttctcacctag
agtaa

>chr17_33390687_33390887
tcatctcaggaaccaagagcagcggctttcatgtgaagtgaatggtgccgcctggggatgtatct
ttggtaagcaagattgcaacacagcagggagtttacccagggcagtgctgactcacgctgggcaa
aatttgtacttcccaaacaactcagcctgaaccaaccgcagtcaactgcaacctaggaaagaaga
ggcag

>chr17_33390887_33391087
ctcactgattgttttaaatcaagtgctgattactgggggcaggtaaacctcattcccccactact
ggtaacttggccacctccagagcccttacccaaaaagttccagtggccagtttctaaaatttcat
ctgagaacaggctggggctggggccagcaagattactgaacatagcaggttcacagctctgaaga
tttcc

>chr17_33391087_33391287
ccaggaggctgataaataagtcactttgcatttacaaattagaacatctgggtcagtggactcag
ggaacttgcccagtcatcattagccagtcaaatcattagggtgaatttttgtcagttctaaccag
gctcattgttctttttttcccactaggcctcctctgacctctctatttcttcagacaactcaagtt
ctctc

>chr17_38270274_38270474
ctctttccacgacataggcattcccttcaaagaatgtaggtcatagaaatggaattctgccattc
tataagccttagaggggtggtgaaagtattggcttttaactctttccatactcttagccagaagg

caaaaagaatggtcattatctcattagggacagaattgcttagggaggcccgacactgtggctca
atccg

>chr17_42172674_42172874
tgcccgctcccatttctcaacaccgtgccccatgcaaagcctcatactagaagagaaactgaggc
acagaggttggttctaggactgacagtgcccttggtaaccccaggagaggctggtggagaccttc
tggatatctctaccctctgtccccttctggggcctcatccctatctggggcagactgggcaggag
ggaga

>chr17_57864418_57864618
caaaattaagcaattttaagtggtcttgatggattctgttacaggatattttgactattcgcaca
gttaggtgtcggtaccaaaggtcacttacttgcactttgttcatcaagaaaaggtagcacttaga
gtaattctggagttgtgatgtctaagcacacttagcatgatttatagtggaattcttcgtgcccc
ctttt

>chr17_57864618_57864818
acttgctttctctagaagcatttactttattttgaactcttttgccacctcactcgcagacttta
atctttctccctttttaatttcttctatttctttaactcattcccatattatcaacaaggaaagat
agttgcaatgtgatgcaagctgttaaatgaatggccagtgggaaacttgtcttctcaacatacat
tccca

>chr17_57923218_57923418
aggtgccaactgtttgctcattctctttgacccactagccttgcagtttgctgtgtgggtgttga
tactcctaaattaaagcagggaaggggtggttttagatggcttttaacacctgctctccacccaa
gcaccctggattcatttgatatctggattctaattctgtagtcaggagactggcccaaactgtgg
cccag

>chr17_57924018_57924218
ggaggatctttctgtgaacttgctttgatgacctccacccaaagccctttgaggaacagttttgt
ttattcaaggagcccttggcaggtcactcctcccctaccacaaatctaatccaaatgcgacattt
tcctttgaggccatctatcccttttggggggacaaaacacatcagcttttcccttcagaccacttt
tgact

>chr16_2202999_2203199
cccatgagcgtgtggtgaagagggaggacggggagaagctggccaaggtgagtcagggcagggggg
gtggtgagggggtgcccctggaggctgcgagcctaggctgtccccgccaggccaccacctggctg
gcagcagctgtttacaggagtatggactgcccttcatggagaccagcgccaagacgggcctcaac
gtgga

>chr16_4665999_4666199
tatagctagcctcctagctctcctcactcaatctctcatcgcccactcgtggcatgggtccttcc
tagcctggctctgggcctgccccactgtagccccatgaccctctggacggcgcctgggacaggct
ggcggggccagcagcctctggtgcttcggggtccccctcccagcagttctcccgctttcccacgc
aaaat

>chr16_20877499_20877699
gcgagctgaattatctcaaaacaaggactgtaccatgtggagggcctggtttccagatttgtgtt
cctaaagactagaatgctgaaagaaacaaaaggcccaaacttacacaaaccccacatgctcacag
actggcaactctaattcccaggcacgctgactcaccagggtttactgctcagctcctgctgctga
gtgag

>chr16_56965299_56965499
acgcgttttgaggggggactccagtgtgagcaggactgtggctgacttgtcaatcatcattagggt
gatgtcccaaacgttcaagtctgaggcccagggaaattggcaacagctgcctggatgcctccatc
ctcccagcccaaggaaaatctggccttgctccttcgttgtctttcttaggagcacgtggagttct
cctag

>chr16_56965499_56965699
gtgggcttgcactcggggtcgcccagccgttctttgagggttcgagtgggtaggcttcccctgta
ggacgggcttccccgtaggtttccacgtggggccacagaggcaggaaaaggggaagtgggcactg
acacctggggtgaaaaagggtgggggtgcggctttgtgaggaaagaatttgcagagactgctaaa
ctggg

>chr16_87421699_87421899
catggtggaacctcagcccgttccacctggtgttacacactcatttaggaaacagctgcgggtcg
cgtgactgatttgtcagtgctttaccacgcgttctactttgcagattcttgaagctgaacaggcc
agacttccctggggaggaagacactgaacatacagcctgctctagctacttattccctggattct
ccagg

>chr16_87886899_87887099
gccgcctgcctatctctcagaaattaaccaccccgctacaattctgtggatgccagagcggagac
ggctactacacaccaggaaagtctggttgaaaaatgtctgaaagtcagtgttttcgctgctattc
ctaaaataaaatacttgaaattccggaacttccactcctcacccaaactgaagaggtgattcttc
acctc

>chr16_87887099_87887299
ttcccagggctcccagggacatggagatgttaccagtccagagctctccagtccaaccctcaggg
ctcagcagggtcaggtcagtgggtctaggcagctccttgtccgctgcacagggcaaggggcaggt
cctggcttgccagtcccatttccttatggttggggggagcacgaccataaaagaaactgagcctc
gggga

>chr15_90943796_90943996
tagatgtggccatgaaaaaccagtgttaatcgggcagctgagtgtatggattgtccaccagtcaa
ggttggggaaaaaatctggctgggctaagtgtggcccgttagtttgacctcatttggcatgtatt
tcttctggtcttacaggtcagagcaactgtttttatatattttaatgtccctaatgacaaaatag
taaaa

>chr15_90943996_90944196
atacatttgaggacccaagttggcatgttctaggaatttcattttccggcttagtgtggtgacct
gcctctgattgctgtacaattccttctgcttcacagtgtcacatctggggataccagttggtagt
ggaacctgttttggacatttttaagccttgtggtatctgatggactcttggttgtttctagctgg
tgcta

>chr15_90944196_90944396
aataaaccccttgacaagaagtatttgtgtaagaaagcgctggtaggcaaagtgagaacatccgg
aagtgattcatattactgtggttgtttgcttaactcaggacttggttgcctagagaccatatcgt
tgagttagccgagtgaagtgccagtggaacctggagggcctgtggcctaccggatcaaatcaggg
ctctg

>chr14_23476760_23476960
gggccccgaggattcccaggcaaaatcaacagagtttagttttgctttgctttggaaggccaagc
caacgatatatatgtgcgaggaattctctcctttcttaggaagaaaaagcaagaacagtggatat
gaccctacgttcctggatgtagaaatcaaatagcctaggaaatcagggatacgggctctgcactg
ttcag

>chr14_61569647_61569847
agcacaaagcaaatagggcttgggtgggctcagggacaactaaaggggttggatgactaactccc
ttttctgtgaccatccctgcctcttgtttactccactgaaaaagggcgtagggaggtcagttgcc
gctgctgatctgcacaaaggtcaaaggcagagccaccgaaagtagtctgctcctgtttttgaagc
tttct

>chr14_74208647_74208847
ttggacaagaagtgcagagctgcgggctgcagaaacaacctttctcttgcttttaaacccgcatt
ccctgacagccaacctaattcagagcaggaacccaaaagctaaaccctgcactgaaaatcaccct
gggcacccccatgaagctgcagggtgggactgtctggctccgcctctccgagggaacatcaagcc
cacag

>chr14_77422247_77422447
attcatgctgagacctgggccagccatgcaaagaagggggtgtaggcagaagtgccgcagtggg
aacaggctagaagttgggcctggtgctgggacagtcccagttcaggcatgtcctcctggtgtcgt
ttttaatagctcatcctttcacaatcaagagtggctcatgccggagatgagttacccgcaatgag
gggct

>chr14_77422447_77422647
ggatttattctaagggcagagggaagccatcacaggaaatgatgtgattgatgttttgaaaagaa
ctcgctggctgttccgtgaagaataaattagaggggcaggagggaagaagtctgcctggggcggc

tgcagcaacccaggcgtgctgcgaggtgccctttgactagattccagtgggggctttcgggcgag
cagaa

>chr14_105147555_105147755
aaagcaggggtagggaagggccctcccagcgccccactgtaataggggcctcatcaatgccccat
gctcactgaataaagcactgccagcgaaaggtgaaaagaggaacaaagaacattctcctggacgc
cacccacagaaagccacgtgcaggcttggccctcaccttggggaccttggacacggagctggtta
tgtca

>chr14_105147755_105147955
catctggctctcagagctggggcagcgtctaggaggcctgatgtagaaagcactcagctaagccc
tagttaccggcacacgggcaccagcgcccctctcagcaaactccacgtcttatgaaattagcac
tggatttccacttcaattggaaaatatggccaaggtaagagatcaggaacgacaaggccaagctg
gggta

>chr19_4375400_4375600
tcagggcaggtggaaggggaggggcaacgccccagggctgggcgaggaatctgggagcaggtggg
aagctcatgctaccttcccaggagacgggaggacaggcctgccaaccaggggggctcagctatgtg
ggacacacgctcccacccttgcccttgcacagggtttcatccaaacatggagatgccagtgagtg
cctgg

>chr19_4376200_4376400
tcccttccatttctgaaggacttccatttctttgtgactgtgggccaccatgggcatgagtcacc
tgtcaccagagcctggtgtccctgtgcagctgtgccaggtgggcagaggagacaggtgaaggcac
attcctcagcgcccgctcacagctctgcccaccgtgcattctgtagagaacctcagcgccctcct
ccctg

>chr19_13275600_13275800
aattaactgttgtgttaattgaggcttttgtgagtgtacttaggaggggtgaggtgaatcacacc
ttttctcacttgatggagttacagggctttctcctctattgctgagggctgtgcagtcagggacg
gggcagggctagcctgggagaagcctgtgcactgagctcagagacgttcaaggcttttccccaga
taggt

>chr19_18393400_18393600
ccaactccaggggctgggctgatgaggcaggaatgccgggtataattattacatgcttgttacca
ctatcatggctgttagggcttaatgagccactattaagactggctgggaaggggggtgtgagggag
gagagatggacccaccccaccaatactcacaagggatccttaggacatctggatctgacctacag
cctct

>chr19_18476200_18476400
ggagtacagtagtgtgatcatggatcactgcagcctctacctcctgggctcaagtgatcctccca
tctcagcctcctgagtagctgggactacaggcatgcaccaccacacctggctaattttttaaattt
tttatacagacagggtttcaccatgttgcccaggctggtctcgaactcctggactcaaactgatc
ctcct

>chr19_18485000_18485200
ccagctgaggatgacgatttgggatgttaggtcacccaggtgctgtgctgtgtaactcaggcaca
gagctgccctgttccgagcctcagtttacccctgtgactcagagcagatctccataccccccatca
cagggctgtgtatccgagctcagccagagcctggcatgcagtaggtgcttcataaatacacactg
tggac

>chr19_34662760_34662960
acagaaaccctggttcacggaattgatccaagctacatactgataattgggattagtgagttaca
atggcgttgcaagccatgaaactggttcagaccggctgaatgagcgggacaggaaggggctgaaa
cacttgagtccttgtgagagccccagctccaggggcagcctcgggggaagctccccgggcgatga
ctgcg

>chr19_39155960_39156160
ccttctttgaaaaggggcatcatcctatcctcagtttgaggattagacgtgatagtgcgcaggaa
gcactctggtgcctggtacattgtaagtgctcaataaatgcagcttcccaaaatgatttcaatgg
cacatggtcctgcatccaggtgggatctgctacttgagtctccccctcaaaggggcctgctgaaa
tacgc

>chr19_39156560_39156760
caggctgccctctcttcagttggtgtgtgatgtagaagcaggaagggcttcccactgataaccct
aaaaataactgcccagaaatgctgtcagatggggccaagaggtgcacaggagcctgtgcccagct
gccgacctggaacgtgggactttctagagggcttggaggagccctgccccttccaggcctcttcc
caaca

>chr19_39157160_39157360
gcatctccagttgtgggatccacgtgccaggagaacacgaagtacacacaggtccgcagtgggag
cccgctcggagacacctccccgccatcacggctgggactgtgcatatccaaacacttcttgcctc
tgagatgagcagttttcttccccttgactgtgccagggttcaattagcctgggcattaattaaaa
cttgt

>chr19_39174160_39174360
ccatttggcccacaggccaccaactttttccccccttctctaaaagacacacaatggcagttggg
ctgcacctttgtgagtcaccggtgatagcccatcataaattgttatttcctatatttccagagca
gctttatcgggcgttgcctgtgcagtccgggaaccgatttggtatggggtcagttaacatgctgt
cttgt

>chr19_39174560_39174760
caaggacctcctgggactgcccttggttttgtgtcttccttagagtagatcaaatgagaggaacc
atgtgaagtagctcacacagggcctaacagagcaaacacaagacgtggaaagcagacttggtgag
gcttgagtttagtttggggggttgcgggggtcccagctctgctgcttaacagccctgtggccatgg
gtata

>chr19_39175160_39175360
tgtcctttgacgtcatacccacaccgctcctggagacagccaccccttcatgccagccccaggaa
ggcttgtaggtggggcgagccagggtgagagtgtagccctgttcctcggccagggcagatgtttc
accatttttaatggagcaattatgagtcagaggtttcagtctctactggttcctcccgatcctat
aatta

>chr19_39176160_39176360
cacccaacctctggatcgaggggactcaattatgcggataaaggcccctaatcctctcagtgctc
caaactcactctgcttctgaaagggatcttcgtaccctgggttcagagagtaggaagcccgcagg
gaccgagtatgcatgaaacaagcccaagtctgtgggggctgtacatggtcaagatcacagatttc
cagac

>chr19_39176360_39176560
caagccagctccaccctagctgggatgcccaccctgagacacccctctctgctttcactggaata
ggatggccctgacttggccgggtggactgttgaatcagagcctctcaaaacaggaaaaaggaaaa
ggatcaccaagcaggagtttcagttgtcaaagacaaaacccttaccacgattaaagattactcca
gggcc

>chr19_39176760_39176960
cgggtgtgtagcacttgcctaaataacaatcccatcagtgccctgtctgaccttttctccccaaa
tgccaaaggcctcttgtctggatgaacaaatccctgggcggatttgtccagactaaaacgttaat
tctaaagactgggccccttaggcactcgctaagattccaggccacactgtctcaaggccagaatt
ggctt

>chr19_39892760_39892960
cacaggggagggcagtcattccctcacccaggcacctgcgcggagttacacacacttagtcactg
cagatcccacagtggctcagagctacgcacagatatgcaggtggggatgaaaatctcatgttctt
aagaatgccgcctcagagacaaattgcagtacagttacaacagagcatccaccccacacccagag
atggc

>chr19_39892960_39893160
caaagtacacacggtacagtcgatcacacaggcggtttcacacattgattcacacagccaagtca
tgcgggattgtatgcacacagccttctcacaggaaactgggcacactcgtgtccctcacgttccc
ccactgcatacccaggcacacaggctctcgcagccccagatgccaaggcgcacacctttgcaccc
gcaag

>chr19_47611960_47612160
aaaccacagcagtggcttactaaaatagctggctctacttagagaaaactatggcttataaagaa
atcaaaaatttaaaccttcagagcagagcaaaattgtacctgggggagaggacaggccatgaatt

agtcattttggggaaccgcatttttttcttttaaagagcaaatttgaggggaattaaaaaaataa
aggag

>chr18_3596600_3596800
ggaagaattacaagatcaggcactgctgtctgtctgttccacggatgtaaccacagcacacgcgt
ggctcacggtactagtgtgataaatgcttgttacatgaaggcgtgaacagggatgagaagagact
tcctggagaaacaaaaggactaacaatcaggaaggggaggtgatcggggcaggagtaaagtggac
acctc

>chr18_3603200_3603400
cttgtcaccctcaggcacctggctgcgaagtaataaaacggttgccttatcatctaaaggtggag
tgagtcaaacctcagcccagggtaattatagtaaactgaaattgcctgtcctttacaaaaaaaaa
ggcggagggagagagaaaggaaaagggcttaaggcaggaaattgtgtttgtctttctccttggat
ttaaa

>chr18_20840002_20840202
ggtaagagacaggaagcagacaagccaagaggttgctgcagctgcccccaggaggaaacgggcag
cagggagtgtggcccagcccccactgtacccctccaggggcccgagcccttgccagcccaatgac
accttgaagtcaccacttttcctttcttgcccgctaataaaacctatttaaacaggaaattcata
ccaaa

>chr22_30592000_30592200
aaacaaacagagagactgccctttctgtaaactcaccgaatgtcaaatattgttcactcgcatgg
cattggcttactaagccactacagagccacttccctcacgtaatcccctcaatccaaaggcactt
caacttgaagaggatcaactgaaataatacacatttcctctgatgattggacaagaggcagccga
tggaa

>chr20_30160139_30160339
tcgttgtaacaacactttgaggtacctacatcattcttacgatggggaaactgaggcagagaccg
taagggcgggcccagggggcatgtgaacccagagtgtctggcttccaagactcgtattcaccgtg
gtgccgtttgacatgatcaggataatggatccctggttccttttgggggcagcattggagaaggg
ggttt

>chr20_30307139_30307339
actcctacccttaaggatctcccagccgcagtctccaggataattacccgatgccaccacagtag
ccagtcggtgcagcttggaacaggataatacctgagaggaagggggcgggtaccttctacccagg
ctcaaaggatcctgagtcaaatgttctttactccctggccttagggagagaatgtgtgctcagaa
atggt

>chr20_30307739_30307939
gtccctcctgtaatcagctgaacaaatatttgccccaaaagagaatcaaagcaggagggaacagg
agaggtcactgcacaaaccaacaggaactatttaatcatttatcccccctagagagttcatttaca
gaatggctttcaagtctagtcagctagctcaagagaacccaccacctttacctcccctgacccca
cccca

>chr20_45978193_45978393
atacaacggtgatttattgaaggttatttcagcactaatgctttctattccaacttaaggagaaa
acattctcaactcagaaacactctgcctgaaacatacacaggttgtccaactggatgctgttgat
gggttctggcttggggtgactggaaatacatcttaacaaaaagccccatgcagccctcggagatg
ggtag

>chr20_45987993_45988193
cacttgatcacttgaaatgttttctggggcaaagagcaggaagctgctggcttattctggcagct
ctgctctgtttgaggaatgctgttggcattcgggtaggtctgaggttatctggaaaacagcaatt
cggctggggacagacacaaaagtatggtggcagatggaatcctgggaggaacacagattgaaaag
aaagc

>chr20_48782993_48783193
aggaccacattttgcttgtttgttcatgtgatcgatgatgatggacacttaggttgtttccactt
tttagctatcgtaaacactgttgctaatggaatatgggtgtacaaatatttcttcaagatcttgc
tttcaattcttttgggtatatacccagaagtggaactgctgcatcatatggtaatttctgtgttt
aattt

>chr20_49434993_49435193
tctctctctctcactcaaccccaagtgcttcctgaggcggccatcttgatctcgtccttccctcg
agatggtttttattccgcagaggagccaggagtggaccacaaggtggcactctcgggcaccgatg
cagggagccgttcagcggggacgctagactatatttaactttcctcagctctgcaccgtcccctg
tgcaa

>chr21_35319730_35319930
cttttttcttatcttacgtcgcagtgtactttgtctcttgtaactcctgcaggatttttcttaa
agccttacatggtgctgttatcacgtggcacaaaatgaagccgacctctttgtgtgtgggaatag
agggagcattacactgctgggatgcaacagatggagggtggaaagctgtccagcaccttgtctct
tccaa

>chr21_35320530_35320730
tctgcaagccagcgaggctgctggattttctcgaagggtattgagttgctaagcaacagcactgg
cggcgcctatcatgcccagcgatgtcaatgcctgagtgtggcctgcagagcacatttgctcccct
ccccctcgcctcctctcgaccccttccccccactacatgagaacattaatttgcatctcagggac
aggag

>chr21_35320930_35321130
cttgaagtgtttttttcctgtctgcccatccagcatcttatttatgccccattctgtccctctct
ctcgttgcctttgttctgtggtctcctgggtgatggagttattgtttgtgtgtagggatgtgtgg
ataaaagggagctgatttctagaggtgagatggccctgtggttaacggttggaggctgctgttga
caagg

>chr7_44679275_44679475
gggcttgctgttgttgggtgtgggggctcacccagtcctggagacagaatgggctctttgtctga
aggtggggcagggtgctcccccagtggtgtgggaactgagccaactggagagtgaagtgggatgc
atggtgtaagatgaataagggaaaggctgcagaagttgcattcccagcattttccttcccgggga
ttccc

>chr7_100143264_100143464
ctggttagaaataccagaatgaatttgctgtgattacctcttttgggaggaacctgagagttatg
ttggggagctgggtgcaagtgggagagctgggcctgtttaattccaggccagtgactcacatctg
gtccgctgcatgaccctggtgtgtacccagtgaaatgcagcacacctaggccacatggttttagc
tacca

>chr6_7147201_7147401
tcttaacatcacatgcattttgtagtttatggtctccagtctccagctgtttttggagcaccttc
taactttgagagggtgagctctagcctgtaaaatggactgtgggtggctcgtggagaaggtgccc
tggtgtgcttttctgtgtcctctctggattctccctgagctgtccacctctgaagcctgcttcac
cttca

>chr6_26025621_26025821
agcaaactgatagaatttggagaggtggttaggcatcccgggaatgagaaacagcccgaagctgc
cactatcacaggctttggcattgctagaagttaacgtggcacttacagctaggccgtggtgttct
gttgaacaaactatttgacagagcacagagcatgtaagtggtgaggccagttgagttagccaaga
aaaga

>chr6_29932221_29932421
gaaaaagacgggaataattagaagggcagccataatcatttgggagagacagcagcatcctcctg
ggtaaggagtcctgccagctaagcagaaattcccaaatgcagatcctggatgggataataatgac
cccagggatcgggtccaaatgcaagaccatagggagctaataattagagggattatgcagtccac
tcata

>chr6_30582221_30582421
gaactaatcaagtgcagaacgtgatacagcactgaatacagtttatccccaaactgagaggtggg
aatgagggcgatttagaagaaagtcctaaaagtacccaccttcccccgattctcattacacaaag
cgaccaaatgcaggaggcccactggttcctaagcagaaatggcacacttcagtgtcattaggccc
gttta

>chr6_33558022_33558222
cagggtgtgggtggagcctggagagggctgaaggctttctcctggacttcctggccagagcccat
ggtgtccttgactcagaccccgagctggggccgggtggaagggcccatctggactgcccccctga

gtcagccaggaggcagccttgtgaccagatgactcacctgtgccctaattgccctgttaatcatt
tcctc

>chr6_34625022_34625222
ctgcaaaatatgttttggagggaagaaactatttgtgcactcaggacaattgccaattctcatcc
ccagcttgtgtcatatttcaccctttggctctgccatcaggataggactttaagaccactgcagt
aggaacactagtaagtgggaaagtcaagctacccaacttaggcagatccattcctatcctcaaat
ctcct

>chr6_34625622_34625822
caacaagtaattcttcatgaaatggagctggctaaggtgatgggaaagggaaaacattgacccag
cattcagtgcagtctgaaagtttaaacctccagattccttcagtttttctgctcaaaaaagtttt
aaatctaatttcagatggttaaaagtaccctgaagaacaaacagaggcaacgttaacagtgataa
aaagc

>chr6_44205022_44205222
gaggtcaggttggcagtgtccccccaaacccttggggaccaactgtttcctggggcaggttggca
ctgagggtggaaatgggcagcgcccaactccctctggccccaacctcatcaatggccttatgtcc
gggctggacccctccagcccatctcggtatgacctggcttcctgtgtagatgctctcggggagag
gacgt

>chr6_74289279_74289479
ggggatttgccagaggcggtgcctcccttccccagcagggcatgtgctccagatcttgtgccact
tgacatgcagagcctgggtgcaacctgcagaaaccatgtgggctgtgccccagccttggggtggg
tgtggacctggtctccctggggatactggaagggcctctgggagatatttagggcgcagcctgga
catct

>chr5_180669194_180669394
agggttccatcccaggagcctgagagggcaaactggccatctgaggagataaccacatcactaac
aaagtgggagtgaccccgcagagcacgctgtggaattccatagttggtctcatccctggtcagtt
tccacatgatgatggtcttatctaaaggaggtaaaacagaagaaaaatcagtgaggaacccgcag
cccgt

>chr3_39192196_39192396
aggctcagatacgcactgcggctctgcctaagggatcgccacagaaagagaacttaaggtgagga
ggttatgacctccaagcctacagacagggaaacagagacacagggaagctacggtcatgctgcag
gttctagtgatgaacaatcccctccaaatctgcagagcgggaatgaaatgagaaattacctcctc
cctga

>chr3_53303960_53304160
ccactgtccccctttggttggatgtttgcaatacttctgcttttatactgcaacaaacaatactt
gtgctttccctccatctagcactgtgcctccaggagagagggtttttttctggtcgcccctgcag
acatgaccctgcttgtccctggccaatcccagggccagactggattgttggccctctgactcacc
ctaga

>chr3_69101910_69102110
aaagcacaacctctctctcaaaatagtagtaaactcattgctgcagtcaaaatacccctaatact
gtggtctgtcggtgtcccctgcacaaattcctatgttgaaacctaatctccaatgggacagtatt
aaggggagcctttaggagatgattagatgatgatggagccctcataaatggggttagtgccctta
taaga

>chr3_133291510_133291710
tcaaggccccaagggtatttaaatgggctttaaacccatttaaacctggctttcgcctgggcatt
ttggtctccacccctatgataagacagtcccctccttttatcagggatagaagctaaggataaac
tgtatcatcatctagactcaagtcagggcgttaagcaatgtgctgagcagctaataatgttgact
ctctt

>chr2_26981296_26981496
tggcagggacgtggagcctcactgtaggtcacaccctggcgtgaagggctgatcagccaccccat
ggtgtccacatggttgtctctgcctggggtgaccctgctttctgcacctggagcaatcctgctcc
tccttcaagtccctactcaggtctcctctgaattccagttctacagggctactgtgaaaaccaag
gccac

>chr2_70313096_70313296
actgtggggacccaaattatcccttgatactgccccccctattatccactattaagctttaaaaag
gcgagagattaaatattgtgcccacattttacagtcattcgcgttcccgtttcaaattaaaggga
ggaaaacgcgttaaaattaggcctccgaccttcagaccagcctgtgggctttttttaagtcataaa
actcg

>chr2_87784885_87785085
ctaacaaatttatttatcatgacaggaaaggaaagcatagctttgtgcaccccatcccacaccct
ccaccccctacaaattttactaacactgtttctgggttcagaatttttttgcctgagttggtacc
tcaaatcacttgagttaaactgcaaatgaatcacacataatgcagataaaatgacttcatgccct
aattt

>chr2_112222129_112222329
gagagtggacagacagcctcgggccccttcaatcctcgctacacatgtcaagaatacctgggtct
catggggtttaggaaaacgaatgtctagttcacattcaggtcacgttctagaaattagggcatga
agtcattttatctgcattatgtgtgattcatttgcagtttaactcaagtgatttgaggtaccaac
tcagg

>chr2_202022755_202022955
gctttagccatttttcttctgttcttttaggtaaggattttctgccccagacctcagattcccat
ttccttcctagcttctctgctgttatagctcagtctcttccaagaccatgcaagattcagaccct
agtaggaggagatggaggaactaggacttaaacccacatcttgtgattccaaggctggttcctct
tttgc

>chr2_216980155_216980355
ggatgcttgtctaggcggcagatacctgcccagccagtatgctacgcatgcttgaatccagcagc
aggcacccagacaccgttagtccatgtacaactgggaattgttgtgttgtttacagactaaagga
ttaaacagaccctacaaaaatgaagaatgacagaattccattatctctccatacagcactcttcc
ccatc

>chr2_219270356_219270556
cccctgctggctgggggcagctcccaggatatcctgccttccaactgtttctgaagcccctcctc
ctaacatggcgattccggaggtcaaggccttgggctctccccagggtctaacggttaaggggacc
cacataccagtgccaagggggatgtcaagtggtgatgtcgttgtgctcccctcccccagagcggg
tgggc

>chr1_8772413_8772613
taacttctaacgtttacatcatctgattaattccaacaataaaatctaaacactgatcatcccct
ccaatcttcagagcaagctgactctacagtgaagaatacgagagccctatcagctgactctggta
tgtttaaacgattataatgtaaagcatgttcattctcttaagtgaaaggaaatgactgccaaatg
cttcc

>chr1_8772613_8772813
ttcacaatctctccagaatagggcaggtcctttaatgtgagattttcctgacattccaattgtag
cagacattgaacaaacagagacgcattaaaagccgacctggttctattatcttttattgaaggcc
tagcaaagatcagacatgagccctgtgctatttacatttttttggtgcattaatcacttttatact
caagg

>chr1_23881813_23882013
gacatgaatttgaattctggctttgttacttagaagccgtgtggccaccggcaagttacttcgcc
tgttctgagctagttcatttataaaataaagacacctcccaaatgtctgtatttcacagccgatg
cgatcagctctgaatccgatcgcatgcgacaatttgcacaagacactcagcagtgaatcttgtag
ttaag

>chr1_23882613_23882813
attagcttaataatacctcgtaggtgtgtgatgcatttcaaagaactttcccaccattatcttct
gtcctgggaggcagcagggcacagaaggagcatggctcagagaggtggaatgcttgctcaaggtc
actcggcagatgaccaccgcagcccttttttagccttccacggtactgaaacttccgagcaaacgg
gaggc

>chr1_27113413_27113613
agagatacagtcccaggcccccacagactgatgtgggctggtcttcgcctggagaggtagcaacc
atctctttgttcccacccacatgagtagccgttgcagaagataaacccctccctgagccaggctg

tggccagaggctaacccggcccctggcttcgtttggagggcagataactgaaaccacataaggct
ttgga

>chr1_36839013_36839213
ctattattgaaaaagcaaagcctaaagactgtggcccaggttgaggctctggtgagggcttttcc
actgagtctgaatgacagccatccctgaagaaggcatgcaccctctccccgggagcttggtggct
atgagcaatctccttaggaacccattggaagccatttgctttgtgctcaggacagatgctgacag
gctgg

>chr1_36852813_36853013
caaatcaagtctgatggattccaaaaaacttggctttgaatccctgctgcatcacttacaagctg
ggtgactatgtgcaaattgcccaatcttccccttctgtccaaacagggataacgcttggggatc
actgtgaggactcggagttatagaaacagctttttgcaagacccagcgagcactcatactcatct
cagtt

>chr1_38465013_38465213
ctgtctgtctggatgtacttgcgtccatacagggactcgttgcattttgcacagtcaaatgactc
gctcatggtggcaaggggagagaaccctgttaggagcacaaggtggaactggggtcacacagaga
ggctgtggcttggctcctggcaaagaggcctctgcattcaaaacacagccaagagacccggcata
ggaaa

>chr1_45273013_45273213
tttggcttctgagttctgtcctggctgcacagccctggccccgctggcctgaagggcaagtggga
ggggaaagacctgtgttgtggagttgccatgggttgctagagcaaacactcctccctcccaggat
ccctccctccagtctggctctggcttaacctgagagcattgttgctgtgagcatgtgctgagcat
ggggt

>chr1_45273813_45274013
gacaactcatgtgactcctcctctggcagggatgggactgtcccctcccaaacaagccaggtcgg
ggccctgacccatctatgggggcgaggattttccctgttgagtgggggtttccccggcctggcta
accggatcctgaccctgtgtgcctgtggaggagggaagggttaggagggcaaggagtttgaactc
tgaac

>chr1_110546277_110546477
gactggcatgtgtttatataatattggtggtgggactgagaaaagaagtatggacaggtcaggca
actaggtaggtagtagatagctaaataggcattgaaacagtctgggtgtgtgttacattctgttg
caaattgtggtgttcaaaagattgggaagcccttttctgtcctacttactgcctttttttctaact
tctta

>chr1_150536176_150536376
actcaagagggtgtttcagggctgagtctgaaggaagaaaggatttgagggaggaatgtataaga
ttagtccaaaacaaggtatgcacgagatatggcagtcagatgccagggagccatcagctctgcca
gctgaaggggaggtgcagagttggctggaagctggccagagcagagcatttctcctgtgagcact
ggaga

>chr1_156073976_156074176
caagaggtgcgtgtatggaggggtatagctcagcctcccagctcgggtggggagcggtggctcag
gcctgtgtaggctggcttttgttggggaggagcctggaagggcctgcagctactggcctccctcc
tccttcctccttgcttagcaactgttgtcgtctggtaaatatttgccccaacaggatctggggct
ggagc

>chr1_156075576_156075776
ttaggtaggatgaacaactgtccctattttagcattaaaagtcccttatctggccaggcacggtg
gctcacgcctgtaatcccagcactttgggaggccgaggcaggcggatcacgaggccaggagaccg
agaccatcctggctaacacggtgaaaccccgtctctacttaaaaaaaaacagaaaacaaaaaaca
aaaaa

>chr1_156093176_156093376
gactgcctaggtcctccctcacttcttctcctgaccttggggtgtggctcccactctctcccagt
gtcctcagggttaataactatgtgccaccagatagagagttaaggggctgctgaattggcttctt
gtgaagggaatcccctaaatgtccctcgttttggtcactggcctccctcccgccccccttcaggac
attct

```
>chr1_156093976_156094176
gcttttgaggtgtatgatattcagaacttcacaggttggggtttggggaaggctcaaggggcttc
taagtccctggaacagctgcccccctcagttcctctctctctctctcttttttttttgagatggag
tctcgctctgttgcccaggctagaatgcagtggcgcgatcttggctcactgcaaactccgcctcc
tgggt

>chr1_156095976_156096176
cctgtgtgctgcctggcaatggggaactctgagggctggtgagcagggctgctgaggagtgggtc
taaggagtccctgcagggctgggccagctcctccacctcccctttgtcttcccctcccacttgtt
atttttagctacagtgtctgtccctcttgcttctcccccagattgggagaggaaacggaggcctc
tccct

>chr1_156099576_156099776
cgactggttatagctaaagctttacccactttgaggagcagggaggcttaaagctggggcccaga
tggacctggaggcctgggatccacatctggaaccagatgctgaggctatggtagatgggtagggc
tcagccttctcccagggcacggatgaggcaggagggagggaggcagggacccctctgttcagtgc
agatc

>chr1_156717776_156717976
gaatgggattaaaaatagcatgaggcacaccagatatgccagccctgctgacttctagctacata
aaggatgggtctagagggaagagagaggaagacaggggcctgagcagataggcatctccccccaa
agcatcatcccacctggctccctcacctagagtataggcacttgtggggaagggccaggggaggt
gtgct

>chr1_161171376_161171576
gaaagacgttagggaaaagaggctgacgcaagacggaagtggggtgtgaggcatctctaagggcg
cttcctgggtctgtgattctgtaggttcgggaagcctggaggatgtcagggtcacccacagagga
cctccactcccccatctgggatgaggtggctcctacacaccccgacactcccctccgcaccacca
atgcc

>chr1_234859377_234859577
tgtgtggaaccgtaggggcttccctggacaagaacgtgtgtccatgtgtgtgtgcatacatgtat
aatggtctttgtgtgcacatcctctgtgtgtgtgtgtgtgtgtgtgtgtgtgtgtgtgtgtgtat
gagtgagcaatgtgtgaccggcctcgggccctttaaacctgaacaggagccaagttcatttctca
gcgga

>chr1_234860177_234860377
cggcatgattaccctggacggccgccagccccgcgctgagagctccttttcttctctccgagctt
tttttttttttcctctttccctctgccaaacatgcagcagttaccatggcagtggaaagtaaagt
tttaaaaaaacacaacaaagtagagaggcacgcctttatccagatgtgccacatgagcagataga
aaagg

>chr1_234860377_234860577
cagttcacctattcatcggggcctttgactgcagaagaaaacactggggcactggctgtcaagtt
cgggagggaggtgactccaggataaacagagcagcgagcagccctggctgcagacagagaggggc
tggctcccaggccctattttccttccctctctctctcccttcctggggcattgtggagtgcgtaa
tgtga

>chr1_234860577_234860777
aacggaaaatgtcatgatttccactgggaacgtgtccacggttttgagaaagctccagaaactca
ggtccataaagcactgaggggaggaggaagggagggaggagaatgggcggtcacagtgccctctg
gtcaccaaggtgaacccagcgagcaggagccagtacgtggctgtggcccaagcctctgcctgctg
ctgga

>chr9_99182979_99183179
actgggcagaaggtgattccaggagcaaagctgaggtcataccagatctttgcttgtttggctcc
attttcatgcaatgaagaagctgtctgaagtgtatggatcatattcctgatgtctttgttctgtc
tcagatattaacagcccataatgtcagaggcctgtgaaccagagcaactccattttaaaaaggag
ctggg

>chr9_132176379_132176579
tccttggtttccaggcgctgtaggccaggagaggagggggcaggaaccccggcccagcggtccca
gacgttttcgaagcgacagaccctgggcccagcgcggggacgcggaggggacacgggctagcagc
```

cctgccctccggagctcaggcccagtccgggcttcccaggccccggggctgccctcccggagccc
gacct

>chr9_132176579_132176779
cccctggggggtgggggtgctgccccggggccccccttcctgggtttcctggtccttttgttcacgt
atccccctccccggattgaaccagcaaagagcacaaagcagaaaatacatccttgccgcccgcag
gctccctcatcccgcagaaaactctgaaacagtccacacccgtcccaaagttgagagaaacctgg
acgtt

>chr9_136008779_136008979
atgcacccctcctcccacccacgtggtccctggccctgcctctgccgccagcctgggggagagca
gggtctttgttccctgctggccaggccgccacgtgatgcgccacatcactgtcactgcctggaac
gaagactgccatggacagccgaggcgctggggcctgggggtgaggccagttggaggagctccacc
tgcag

>chr8_95448824_95449024
gattgggtctctttttgctgcaatagctcctgtttggcatattctttgagccttttataaagggt
gcgtaggccctgtgctgttcgaggagggcggtctactccaattgcattatagttaactgctatga
tatcccaacatctattcttttctactattactgaatgtttattagtgtgttcttcgagaattttc
acata

>chr8_95449224_95449424
gggagggtttattaaaattcctccagcttaagctgacgcaggttcaggaaatctgcttaagccaa
gcctgacctacataaagcttctcactgaagaagacaggaaggcacaagcaaaacacacttgtgta
taaagggaacagctcgagacaggatttaatacacaaaggtctagagataagcatggaacacagct
aaaaa

>chr12_6444539_6444739
agcttggatgactgtcaggtcagagctgagggcgtgctggggagtcctggagccccctgcacacc
atggcattctggaaggtgtgctttgcagtctcaggccaaccccacagcagctaaatggcttgtcc
tgttccggcatcagtgccaggaaagcaaagccaggagtgaatatttagctctaacttggactttg
atctc

>chr12_6446939_6447139
aaaaaatgtcttggtcagaaagtgtcttatcctcccctggtgtcctctcagctcccagaaccaga
cctccagactgggagcagataaatatggatcaactgatcgatctaatgatacatctgtcttccac
tctagtagaagccagtgattcccaaagagggaggtgaggagacagcacccctggtaggaatatat
ttatc

>chr12_45628533_45628733
cacttcctagctcagctacattccagtctctggaaccaattctgactccaggcaggcaagatgag
acaattcagcatttactatcagtccagcagtaacctttcagttctaataatttggcttgtccttt
tcctcctcagttgttcagactcctttctcagaaacatgcctaggacctcagtctctttagttctt
tggat

>chr12_45629533_45629733
gagtccaaaaagttgagaactactcctggctttttccttccctccctcacactgacggcattcct
gaactagcatgcctgtctgtctctacatttctgggtgactgcctcatcccaggtggccttcctcc
agctgtgagctgggtgtgttcccaggtatttaatccacccttggggcaagttcttccaggcagcc
tccag

>chr12_56520333_56520533
ataggtcaccctctccattcccccattctcaggaacctccttgcctactccagtgcttggcaaat
ttcagtttatagagaggtttctctctaaggttcatccatgttccttctgtagttctgctttatca
gttggtgagcaggtcactcataattcctccaatttccttgtttccctttttctacctgcttacctc
tggga

>chr12_56694733_56694933
tgctttgtgagctgtcagttctgtgtgtaatggcaggaaatccaagtcttaactgcgcccttact
atgaaggctttccgagccagccacagcttctgggtcatgggcattagtctcactgccactctggt
cacgtaaccccacacagcagctcaccccactttagccacagattaggagccgcagactagcccct
catcg

>chr12_76337733_76337933
ctgctttgttgaactctgtgaaggaaatcataggatgtaaatatagctccatctagacagacaag
aagacatgagataccagtggtgggatgtctgagattctgaggagttagaaatgaaggagaggaaa
tttttataccctctctctggtccctggtcttatatttatgaaattcctactttcagcaaagaaaa
aagtc

>chr12_109083271_109083471
ggattcagactgctttctacacggtttcccttatctaatccaactcaggcagctgtctcacttcc
agtacttttccatctcctgatctgcagtcctctatgcctattatccagactggttacaccacccg
aagatgtaagggcctaagagcccccaccccatcctggggaaggcaaacaaagatactttacatga
cctga

>chr12_109084671_109084871
aaactggttgtctaagccctcgtagtaagcaaatgacattggagattgatctactgaaaccctct
gtgaacgctgaagagctccagggctaacccttcccattgcagctctgcattggtttaatccacag
gtctcagaatgaaaatccaccctccttggatggtggagatgataaatagaaaaatattacaactt
ttttt

>chr12_109084871_109085071
ggggggtgggaggggctaatgccttgggctccatggctgagtcagttccacacaagcaagaggac
tgcagtatagactcaattcagtcagccagaaatgaaacccaagtcaccgttcctccaagaaagca
gacatgtgtcccatcacatgccctctcagctcattcttccccagactcaactcaggtcccacccc
taatg

>chr12_109086471_109086671
ctgacagaatgaaagccctgtcaagttactgtttttgttattaaactctctaatcccagagaaag
aatgcaaaggattcagtgtccattagacccacagcttttttttttttttttttttgagacagagt
cttgctctgtcgcccaggctggagtgcaatggtgctatctcagctcactgcaacctctgcctcct
gtgtt

>chr12_118558417_118558617
gaatttggggaaataaaggattactagggaaatgaatgggtcaggaactagaagagaaattaac
ttgtaaatagatgtctttgaaatgtgaatgagcctgacattatcttatgaaaggatctgttcagg
tgtggttaccttctgtgtcttcctttctgcaataaagagataacagagagggaaagaaagaacaa
ttgtt

>chr11_355400_355600
gggaacagatggctcagaacacaggaagttaatttaactgcagtccagggaggatggcattctgg
aagccgagtcggcctgaggtctaaggggcggagggaactccaagcttggcctggagctcagggaa
gggagctcgcgtggacttcaggagcccggtgggtgcttcctgtgcacaaacgggcagggtctgag
ctccc

>chr11_355800_356000
aaccgcttctgcggcggcttcccaagccccagctcccgcagaaggcctggaggggcccggggacc
cgtgccacgcagtgagtggcggcaggacacgagcccgggggcgcagcttcctggacactctgccc
ccagacactggctgccacgcgggacggtgaccagcgctgccctcggcgcccgggaggcctcccag
ggctg

>chr11_6628024_6628224
cttttgccccccgagtttggcctgccctggcgccacaggcagtactggaagctgataagtcaggg
cacaaattaaaggtctaggaggcaagaccaactgataaacagggggcgtaatgcttcctggggat
aatcccatctcaaggaaaagcttttggctatttctgtgttagcagatgcgagcaagttgtggagg
aaaat

>chr11_6628424_6628624
tttccttttatggccccaggctgtcaggcaggcccagctccaaaccaattgttttgttttgtttg
tatactttactttataaaatacatatttcttggaaaagaaaaaaatcaggtatagttttaatccc
aaaacagccattctcattttcttctgttttctatgcacaggattgctaagtaggggaatcctgct
ttgca

>chr11_6628624_6628824
tggtggtttgttatctacctcatcagtagtcaaaagcatttctccttttcaacataaacccttcc
aaaacatcatttaaattccattccatttttagatgttagtttcagtaccagagccaaatagtaag

tttgttccctcttctataagttgtggcctgccttctccaagacacccttctcccccactaacatg
cactg

>chr11_6628824_6629024
aacagctgctgtcaatccattccctcaagaaacttaaaggtctaacagacaagacagtccattaa
cagatttttacaatccagtgcaacaagtgctgaagtaggggacatataagatgtggtggaaaac
agaaaggaccaatagatgttgcagaaggggtgcaagagaaaccaggggaagagcactaccacta
aggaa

>chr11_47429024_47429224
gggaggctgatgagggtcagagacttaagagacctgtactcagtcgctatctctgcccaagtgag
tacccacatgccccttcctgaatcttctcctgtactggctgatttccagagaagaatacaaactc
gaaacgccaagccagactcccactgctgttcaggacctagacgtgagaagtggtatcacatactc
tggct

>chr11_62321024_62321224
tcaggctgagcagggaaggaagggcctaggctgcccagagcctgagtcaccacatagttgaggtt
cctgtcactcgaagtcccgcctctgttacactccgtgccctgtgcccgccccaggacttggatcg
ctacatctgaccctggccccagggctggctccacttccacgctccttaccaagctacccttacca
agcta

>chr11_62324024_62324224
cccacccccatctagaggccaacggcccagccttcctggggccacaaccctctcgtcttgaggct
cctcctcctgcttgtgggtactggccctagcccaagtgcaagcactgcttcaaacaacaacctgg
ccacccttactctgcaccgcgtggcgagggctggaggcaggatgtgcagatgactcactcttcct
gcagg

>chr11_65186824_65187024
cagctccagaactagacccgtggagaatggagggctggggccctgcacattctcaagcagtagta
gcaacactgtcattgtcctgcccttggtataagcctgaagaaccaccctttcttacccggcttct
gctccccttttccaggggctcctgtgggaaatcccgggtacttcctgggactggtctgtctatat
gtttg

>chr11_65420624_65420824
aaagcatcggtgggccgggtagatctttacctggcaccacacattccccaagagaagcacacact
ccctgggccacccaaggaatttggccccatcacatctttcgaacatcgtttctcttacctctgac
gctggctagtccctctttgacatccactctcagctgatctaagacctggagctaatggggcctct
tggaa

>chr11_65420824_65421024
gactcctgaaactgctccatccccacagccagcgggctctactaggctctcagtgtgctaggact
cttgccttagctgcccgttttcatccactggacggtaactcggggtttccaagggcattctcaga
ctgcaagctctcagaggacagtgcacgcattcacccagctctctaccaccaatgtctggcctgat
gtgac

>chr11_67055624_67055824
gaagccttcgggctgctaccaaccccaggacctcacagattgacaccatagcgtggggtggcaaa
gtgaccccagatggcacaggcgccatgtgactacagggagaaggcagggtaaaaatggcaatcag
gccttccagcccagagtctcagaactgaggacgccctacagcaggaagtgatgctcaagctagct
ggccc

>chr11_71750952_71751152
ccgagatctcacctggcaggttacccagaggggccaagccaccttgccctaggctaataagctat
aaaaacagcataggaaatactttgctggtacatcccctgcctctgagaagataaaaggcaagagg
accagtggctccaagagacgccatacagagaaaaacaatctttctcaattcctggggagtcctgg
gtaga

>chr11_118783590_118783790
tggcctctggctcagggagatacagcgcccgagacgacagggtcccaaatcccctttctcctggg
tgtcaggacccctcagtcctcagaagctctctccaccactcaggtcacttcctcttcctgaggtg
gtgactcagcgggcagggaggtcaaggattcgtagcccagcaggaaatctgctcaaggaccaagg
tgctg

>chr10_74081194_74081394
tcctcccaaaagagtcagagacccctgcaccctcagccgaccccaggcatgacagaccccgcctc
atcccacagtgcacaaggagatcacactcagatctgggccccagtctggcgtctgccccaggcca
gctgcatgaccttgggcaggtgattgaatgtttttgggtctccatttttccacttgtgaaatggg
gatag

>chr10_74081394_74081594
cgatgcctactttgtaaagctgttgtgaggctggggaaaatgagaacgatatcaagtgttcaca
gtagaggccagcactgttgtgagcggcagctcctctccctcccctcccctccccctttccctatc
acaggtggggccaggtggaatgtcacagtaagtccttgggatagaaaggtctactgacatattca
aagtg

>chr10_103911410_103911610
gtgaactctgatcccggaggaagaagggttattttcaacaatctcttcagcttgaatagattgtt
gctgtgtgtgattcaggtttggtgcatgacacctttcctcttcatggagctgacctaaggcggaa
tataagggagatgggaggggcagcttgggaaatcaacacctcccaccctcacgacggataacccg
aagga

>chr10_105668210_105668410
caggaagcacccccagcacaggcctgcggtgagtcaggggaagcagaacccagaacagcagggag
cggccacctggcagatggcccaaaagccatattgtgcctgaggacagcaaggagaggggcagttc
tccacaagaattaaagttgcaaaagcctctctactctcaataatttcagcagtcagatgcttttt
atttt

>chr10_105668410_105668610
cccaaagcccacagatgtttcatgctcctcagacactcactcagagggaaatgcttactggagaa
gaggcaatcatagtgccttaatctttttataatgggaaaagaggttaagtgaagctcctttagac
acaggcttaatatctagcctggttctagggccaggattttaaagtcctcaaacagtctcatttat
ttgat

>chr17_27073273_27073473
gctgggtgagccattgctctgcctgagccatgccaggcatgtgtgactggccctggctggggtca
gcaagggcatgtcgacagaaagaagcatgccaacaggctccttgggtgctgacggccaggtaggg
tttggtgcaggagaaggcgggcagctagcaggaggggacggtcaccaggcttcagaagcccactg
agcag

>chr17_38267474_38267674
agcttctgaaataggtgctttccctctctcccactataagccacagtgtttaggatgaccactgc
ttccagattgtgtgatttgccctggcaaggaaatcctattgtgggggggaggaaatgaggcagtcg
tgacacaaagtgatggatgagccttttgcttagcagagagagagagagaaagccggcaggctgtgca
gaact

>chr17_41560874_41561074
taaaacaagagaaggcaatctactgcctactggcttgtcaagggtcacttgagatcggacgacag
gaaccacaaagaagtgaggccgggaatttgcggatccacagtaaagcaagtttgtctttttccag
acgtgcattaagattccttttttcaagtgttctaaagcgcaggcgcaggctgttttgcggttgcct
cccgg

>chr17_57914618_57914818
cattaggcagtgttgcaagtacatatcggaatctctttggctggctctaagaaagagtttgaact
tatttacctccttagccctatgtaacaggtaagaaactaaaaggtacagaaaatagagatgtttg
atttttctaagttgccccaagctaccgtttttaaaaacgcctgcaagcatgtctaaaacaggagc
ctgtt

>chr17_57923818_57924018
tgactcagaggacctagagggagggcttgaacacactccagcactgtttctacaatttagccttt
atttgcattggaaaccacattcctgaattcttgaggggcaggctctggcttattctgggcaact
gacttcaagtgggacccctgagctacctatgtaagcaaagagtcagccatctctggagagttacc
aggga

>chr17_57924218_57924418
gaagcagtgatcaacattgcttaattgtgttccagtctgtttccagcggggaaaaaatgctttag
gggagtgaggcagttagggatttgaaaacagggatggtgccgcctgaaggtggggaatgacttag

atcactgtcttcctttggctcctcaccatgttgtgtggaggaaacgaaaagcagctcaagttcac
tcctc

>chr16_21513899_21514099
ccctggggtgcgcgcgcacacaggcgcgcccttggattcacgtgtgccagttttcccactttcta
cggggcggggtggaaagtgaaaatagtcagatcgacaggtggatatccaccttcccaacctccac
ctgaaatgtgcccatcgagtcctagcaccttttaccatttcttcccaatgaaaaaaactaaacga
tggaa

>chr16_29607099_29607299
aaaatagtaagatcgagaggtggatatccaccttcccaacctccacctgaaatgtgcccatcgag
tcctagcaccttttaccatttcttcccaatgaaaaaaactaaacgatgtaagggaagggagtacc
cacgaccaccaaaccctgtcctctgcaatgtgaaatgtgctttgaagtcctctcaccctgaggct
tgccc

>chr15_58624308_58624508
cagaagcctgcagccttccggggattggctccctgcgaagataaccctgtccttcaacaaaccct
cgcttcctatttgcattcctaattggcatcttctattgcttttcctggtgacttcatttttcact
cttggctaaaaatgggtctctgatgatttattctatcctgggtgttgacaagctgaagaagttgt
gtggg

>chr15_89181396_89181596
atctggggtaggaggcaagcgtgggtaacgctcagcttattgttggatctcaccacagtgaaaga
caagcacttccctcattgaaagttgatttctttttgccccagttgtaactcatttgacattcac
taaataccaagccctccctatctggcaggcactgttctaagagctcaaagtcctagcctgggaga
gtgtg

>chr15_93460796_93460996
caaacaagcaaaaagaaaaaaaaggtgtgaccagtcagtcttctttgcttttccatcttaagaca
catagaagtgactgagaaggaatagttatgcaatgacatcggtttaataatcagttcaactgaag
aatgtatgtaaataagggcagtgatacctaacgctgatagttgatagtgtggattgcataattca
gactg

>chr15_93460996_93461196
tggggtgatatcttacacacgcacatacacacacacactcttgggctttgtcttctttataggta
aggattaggagaattttgacatatgcctgacacaaaagtgctccgtaaatattagctattgtatc
actggcaggaagttagcctggaaataagttttatggaattccagtgtagattgctttgtgattgt
tttgc

>chr14_23789760_23789960
gttttgagactttacctcgccagcaaagggggccagtctgttagcggtgcagattggaggggtg
acattggaagctgtccaggaaaaagaaaatggaactggggagcagaaggcctacgcaagagggcg
ggacagacaggacttgtgactagtagctctggactgaggaatcctccctgctttctggtgcggga
gagct

>chr14_55570247_55570447
taaaggtgaatgcagccccagtgccagcacataaggccagtcagtgctatacagggtcagcttag
ccctgggctgacccttaagatcaggaaaaagttatggggcagtgacttctcagcaagtcttttcc
atgtttctggtgtcttcgaattcctagctgaacagaatttatcaaagcagaaggtagctgtttat
acccc

>chr14_55570447_55570647
agtttacccaggcaaagagaaatgctttggaattgagatattttcagggcatgagtggtttagat
ctctgaaactggaagacgaaaatccaggttattagagatcagcagcacctgagaggcaggcttcc
tggcacctgaagactcacagacaagccttggtagcctggcttgttttccttgcagttcaaaacca
tttct

>chr14_75725447_75725647
cggggaggtcaccctgacaattatggccaactgacggtggagggagtggtctcggtttggccctc
agtctggagtcacagtctggattcacaggcagcgcagagagatgttggctcaggacctgaggctg
aggcgtgctgggacagcctagggggtgtcctggctttccaggggctggggaaccctctctggccc
ggggg

>chr19_1270600_1270800
tactaaaacaaatttaaaaaaattatccaggcgtggtggtgtacacctacagtcccagtttcttg
ggaggctgaggtaggaggattgcttgagcccaggaggtggaggctgcagtgacccctgcactcca
gtctgggtgacagttagacacactgcccccgaccccaaaaaaataagtcctaggtatgagtttga
gataa

>chr19_1415400_1415600
tttttttttgagaactgcttgaactttaattccaaaggattgtgtaaatgagcatttttccggt
cggttccagccaggcttgggggcccttccttcatggggtgggggctgctgctggctcctgctggg
tgccgggccctgctggcagggatgtgggcggggctggccctgactcagcgcgggcacagcctatg
gagag

>chr19_3983600_3983800
aaagcctccagagactccgaccctcccctcctcacttctgcccctgctccaggggaaatgaagag
ccttagccagagtccctcaccaatgtaccaacccaggacaaaaagcagttggggtcaccgtactc
tgcgctccccctactcatatcggggccagaaactgacatggaatgaacatcatccagtgcaacac
aggag

>chr19_5968200_5968400
cctctctacacggaagcttacaggaaggaaatccaagaactacagaatttatgctgaggcagcag
agcaaaactgatgatactggagagcgcgggctctggtctttgtgtgtgttcaaacctgccctggc
cacttacttgcggttgggatcccagataagacaatgtacctctggcacaatgtcttcatttgcat
atcag

>chr19_5968400_5968600
ggctgttatttcaccccaggcagcttcggcaaggatgaaatgaaatgctgcacatgccgtgtttg
gggaagtggttggcacacaggatgattccacactggcactggtagcattattatgctcggaagat
tgcaaacatattgatgccaccttaaagcataggccttggattcaaggacagctgccagtgcccgg
caacg

>chr19_6738000_6738200
gacggacttcctcttcttgagccagggatgtggctcccagccagagctgaggcttttggtaaaca
cacgcatctgttcaggcctgacctgttttcctaatcttcctggtccacagagaggcctaaagctg
gcctcaggttaaagtttgaccctaggatgacctatgaccccaggctgagaccccagccttaggct
aagcc

>chr19_6738400_6738600
ctgactccagattggacctgaccccttaccgggttgctaatccttctggttaagcctctggcctc
tatcccctatccccacggtggtgctgacctggactgaaccccaattccagtttgagtctctgagc
agaggtgaactcagaactttgctaggctgagcccccatttgagactagccacagaactcactggg
ttcaa

>chr19_6738800_6739000
tctgaggctgcagagaaagcgccaaagcccggagagaatgggagtgggaatccccagtgcctcct
ctcctctttcattccagcacctcaaagggatttacaaggtgtgaacagacttttatgggagaggt
gacagacttcccttctccagggcccagatggcccagatgtctgggggaggggcggagtcggaatc
tcagg

>chr19_8066800_8067000
cctcaagcggttcagccattacggggccacctcagtactgcaacagcctgtgactggtttctcct
tgtctccccccagcctctccaccagggtgagcagtgaactaaaactgctcagtagccacccctgg
cctacctgcagggtaacagccaagccctctgcccagtggacacctgagcactcctgccaccacta
cccat

>chr19_8067200_8067400
ccctttctgggcttccacatcagccagggcataattctcacagcactcatcagagtgggctgag
atgacctacacacatcatgcacactctccagagcctagcgggggcccaggacacagcagcggagc
agggagagtcgttcagaggagggagcgagcctcacttccctcccacaggctggcttccgttggtc
ttccc

>chr19_11253600_11253800
ccgtctcttccttggggaaactgaggcccaggttgctggggtggaggggatgtggagtgacaggg
cagggcaatggctcctcggccccctccgggaacaaagccaggtcattcctgtggggagggagcgc

caaccccagggttgggggggcacgggccctgggtcaggggtacagataagcctgggctcccaga
accct

>chr19_11253800_11254000
tgtcacagcggcacccctgccccatgcctgctttctctccagggcactcagagctgcagtttgca
ggacgagagggagacgtgcccaatccccccatcccaggacgctcagccgtgcgtgggctgggccc
actccactcccggcgcccacagttgccccagtgaaccggccagtggaggcatcctgtcccgtgtg
cggga

>chr19_11254000_11254200
aaggaatgaccgccaaggtgacagcagcgcttagccaaccctggcgctgacacctgacttgcaaa
ctgctccctgcctgaggcttcaaggcaggcgagaggctttgtgtgtgtgacttcctccccgaagg
gacactggcaacagctggagacatttcctgttgttaaggctgagggggagctgctggcatttggt
gggtg

>chr19_13275800_13276000
tacaccaggatctccagggcatggacagggatggggtcggggaacccaggcatcctaggtgaatc
caaggaacagccatgtctgcaaaccactgttctagctgccttgctgggcttacctaggggccacg
atccctgagtcagttcccttggcagcctgggtctcagtttcccttggcttgtagaagttctttca
ggggc

>chr19_13957400_13957600
gcccacaggtcaccccagctcacatgcagggctgcctgctgggccccatggcaacgccacacaca
gagacatcaaacaggccctgccgaggaagtcccacgtcactgcggttagagtggctcctcccagc
ccagcccccggctggcggcagtgacccccatgccagtcagggcccctgcctcctgttgccctggc
gacca

>chr19_13957600_13957800
gctgtgttatggactgagccgggatcccaccacatccctgccccctcaccagggcaccagccact
ccccacatccggagaaggacattacacggtggccccagctcaccgtgacaaactcaattgcctgg
tgtctggagcaccaggaaagactttgtctacctacataatggttcagaagggcagagaggtccca
gggat

>chr19_18393200_18393400
ggctcagagaggtggagtgaccatcccccgagagttagctaggagtcacagcagggcccgggctg
tgactaggcctgagctcttgtccacccttagaaccctagaacctgggagtccaggcccctgcgca
gccccttccttccgccgcccacgttttaaccccaggtctgccagggctgtgatgtcctgggcgag
ggtgc

>chr19_18484400_18484600
cctacaggaaataggatttctgtactgggcatttgaaccccattcccttgtttctgctgaccccc
agcagtctcaatatcccctacttcctcttcccagaaccaccctccccaaagcccttatcgatgct
tggaggattcctatgcatccctcaaaatccaaggtttgggaaagatgctgcagtggtctcaagac
atcct

>chr19_18484600_18484800
cagtgtgatgacagcctggggtgggggtaagggggcaaagtacagcaccagaaatcccaactgag
tccccccacacgtcttccaccttcccaccctctctttcgggttctggctccctgcctccaggcaa
tcaccagtttttaccaggagtgcctcagacctgcccggacatgtccaagtcccctagaagcaggg
agggg

>chr19_18485200_18485400
agtagaattatgatgggagcgagcgtgtgaactgggttgctaatgtggagcgtgaagagggaggc
ccagggttactcggccaatgggttccgggcagcaagcacgtgggggaagcctcagctgaccactt
cctggccagtttatgcaggaactcgcacagccggtccctgaggctgagcccaccacttccgcccc
ccatg

>chr19_34662560_34662760
ggatatattgtgtatacataacggagaggtgtagtcagaacagatgtggaagcaagacagggcaa
ggctgactcacggcttttagtctgagcagctgagtaaaaatggtaccgtttacatcaaataggga
acggtggtgaaggagcaggttagaggggaaacagggagcatggctttggacccttaacgtttgag
attcg

>chr19_39156360_39156560
cagagttacaggccctgaacaattttgtgttgggatgtgccataggcagtgccaggaaggttctt
tcatgagtaatttaacttgttactaagtttgctatcagccggggggctctttccccgggttccct
tctttccctgggtccccttccccgcaaagcaaagtccaactcaagttagaaacatcccacagcct
ggagt

>chr19_39173560_39173760
agtcacattactccgtgttatggtacccatccctgtctccttagcttgtttttgtctgtattggc
tcttccactagactgtaagttgcatgagggcagggatgtctgtttaatcccagtgctcaggatag
tgtatggctcgtgatagatgcctagtacattttaaaatgagaacgaatgaagtttgggagaggct
cagag

>chr19_39173760_39173960
cagtgagtctccccccttgttgggggactggggagtgcctgggaggggctatctggtgccagcggt
ttggagtggctgggatgactctggaatcctgtgaggcccagtcagtttctttggttctcatgcag
tgcagtgccctcagacctaaccattttgttccgtgctggcttgaaagggtctgcctccctcccag
tgcag

>chr19_39174960_39175160
ggtacccagagtcctcctcggtcctcttcccatggtgtagtgagagcactcaggaccacctaggc
ctttcctagaaaactgaacccacaccttcccagtgctgccccaccctggtcccccaccccctgca
ggacaaaccactcctcccttgttttggggccaggagtcagatctgcccctgagagcagcaggggc
ccctt

>chr19_39175360_39175560
ctctttggctatagaatcctattttgatctcttcttttcttttctcttctttctctctctgtggt
catggtcaggtttttctttttttaaatcctcccaagacactgctaatgttgtctgtctcatgcat
ccaaggaatctgagatggactgaatattgtcaagggaaaaaaaaagagacccccaaatccagagt
gattt

>chr19_39888760_39888960
gttaatgtgtttctccccatggtcctatttctctcactctgacctctctctcttagtcccctta
gctgtcttctatccccagctcctaactgggactctgtgtctatgcaggggggccagcacccctggg
ttatctggggctaagggaagggacttcatttccaggggccacagccaagcccagagtcccccagc
ggctc

>chr19_45959160_45959360
ctgggagaaaccccagccacatacctggccgctgacatcacccggccagggcacccccggcagcc
tagacaagctgactgaatcacaggcggaattcagccaccccgggcacgtggcctgctgtgacccc
ccgcaacaccccccgagtggccgtctggctgcgggggttgggccgggcacacaggggtcagtgagg
gggca

>chr19_45959360_45959560
tggggcctgagtcagggacagggtggctacagccagagaccacccagccacaggcgtccatgtgg
ggcaggaaggagaaagtttgggaaggagagcctgtggggaggccctggcgggtgaggaggaagca
cgtgtgggtgtgacggggaggctgcggcttgtgggcagcggctgggcgacccacaggggtgggat
ggggt

>chr19_47611560_47611760
tagaatctggtttgacttgatagctatccacggagacctggaaatagtttcattagcaattttca
ccagaggtatttactgtaactggatctgacctctatggagagatgtttctacaggcccaatagcc
ataaaaatgtaacaaaaggaagtgaaggaccctcagacagccatttctctcccatcagggaagaa
ggtaa

>chr19_47612760_47612960
ttcaaataagtgcaattcattttgggctgacccagtgccctggataatgtgttttgttcggttcg
tgcttcagacatttcttgcaggatatcgtagtttggagatttcttcctcccgtaactgattttac
atacctaggatcaccactctacggcagttgtgaacatattaagaggaagaaaacaagtggcctat
tgaat

>chr22_30591800_30592000
ctgtctggaaatataaaggctcacattggctttggccaccaaagagaggaaacattctgcaatcc
ttaaaaactcatggccgaagggggtggggagaactgtttgagccttgtacttacaaagtcaaaag

ggaaaacatgtgtttcacatttgtcttgggatgggaaagaaaaacttttcctttcacttaatctt
ggttt

>chr22_30592200_30592400
tcaggtttccaaacctataacccctggtttcagccggagacttgaggcaatacagcttcagtcat
gactcaggattttgcgagtgaaagcaagcacgtcaagtgatgtaagaaaattcccccattcctcc
aacccactggcttcctcctccctggagaattcagtcagacagcattgtgttgggggtgggggaga
aacag

>chr22_35772800_35773000
gtggtcaggacaccgtctgtgaccttatttactggggacgtcccctgcttgaggagaatatccag
gcaaggtctcctcactctccttagccaccatccagcttggccaagaggcctccccatgattcctc
ccacgccagggcctcgcctttgtttttcactgttaggggctggcgagtcactgacccgcccccct
ccctg

>chr22_35773200_35773400
agctttaatggtaggcaggaggaagtgaaacttctagaaaacggcagaagcctcttgtttgtctt
ttctaagtctgtgcctttcccctgctgggtcccccagctcccaccctgtgaagtccacctcaatc
tgcccctgatttaatgttgcaatccaccgaggccttcttatgcttcacatctctctgacttctgc
atctg

>chr22_36725054_36725254
cttctgcccactggtcattcaggagggaaaaggaagcccagacagaaagaagaaaaaacaaacct
cacacccaacaccaatggcagctgcccctgccctgggccgcagcttcctcccagcccccctaagca
tcaacggggtgggggcgcagaggggctgggtcactctcattcacaaacacaccccacaccctgct
ctctg

>chr22_36725254_36725454
atgtggaaactactcaggaggcaagctctaggggaggcaagccttactccccccttgcctttctca
aaaaacacgagtccttcaagcctgcacagcgaggacagagattagagacctgcatcctcgactag
acagacgggaagacagaagggagggaggcggcaggaaccacagcctggggaagacagagcaattt
cattg

>chr22_36725654_36725854
taaaaggaaacattccccaacccttccagaggaaccgttttcccagggctgtccctaggatggct
ggaattcctctaattgtgtgaggaggagtcagaggctctgctgctgaacaacaggaagcagagcc
cccaaacacactcgagggcatgcacacagaggcgcacgcacgcacaagggcatggacacacacat
agaca

>chr22_36725854_36726054
cgcaagcatctgtgcatacacagtcacatatggaggtaggtgtacagaggtacgtgtgcacagaa
atacatgcgcacacacgtgtgcaaagccatatacatagatccacacaaggcacacagctgcacac
gcattcacagatgcatatccacaagcacaagtgcaaacacaggcacgcaaggcacatgtattcac
aggtg

>chr22_36726254_36726454
taccttaaagaaatctggatttgttctgccaggaacattcatctggacaaatgctttctgaaagg
caaactgctgggaggcctcctcattgcatctcttaccgcactgcttagtatcctaaatccaccgg
ccggatgcaccacgaatcgtaccatggccccatgcaaccgaaaaggtctttccaatgctcaggaa
gggtt

>chr20_30307939_30308139
gttatctgacctctattgacttgaccccccttgccttttacaagagctggcacagacgagttgaaa
ttgcaaagaaatgcaaaatgagagaggggggtggggttccagggcaaactcaggaggtctcttgct
cagtggtaaagtgaagtgcctgccacaccctaggcccagcctatagacagttgcattcttgacat
tcctg

>chr20_45946393_45946593
gagtagggcaaccaaccatgtgcttagctgggtcccgggcaaaccaggacacataggtcaaggca
tatgctctcatgggccaggttgcaaacgacagagaggcaggatgccacagtggtggggaatcagc
tttctggcgtcaaaatgcatgcagaacctggctgtgctctcccctgcagtgtgaccgtaaaaagg
caact

>chr20_45946993_45947193
cagaagcagaggcctgctctacagcccttttcgtaatcaccagtcctccccttggcctacttcaa
acatgaccacacactgctgctcccccagaagcagcaaagcagagcatggttaatcacttcaaagc
gtttcccaaagacacagagcaggtacccaaacaggcctcccttccccaggaatatttggggccct
cccca

>chr20_45947193_45947393
accagtaattacatcataaagagacaaaagtccggaatttcctcctggcagagcatagaagtaag
aaatcaggagcaaaggttggggggtggggaggaagcaaaattgtcctcgcagcagagaatgcctaa
gccaggaggaacttgtaaatgaaaaccgctcgcattagcaaatgattatttcctccaacgctgac
cgtct

>chr20_45947393_45947593
cttagacccacagaaactcgaggaccggtctgcaccctggctgacttgggcctgccagagctgcc
gggctgagccctctcctccagaggactgcactggcctgaactgtgcttataaagaatctctgcaa
atcaacaggaacttctcatccagagccgggatagggagggctggaatggaaatgaggctgtggaa
tgaaa

>chr20_45978393_45978593
acagttaacttcaaaggaacaaatagcaaccttgatcttttctagcaactagaattctgtttccc
tctctagcatgaagtatgtgaaattctaaacttcttgaaaccgatcccatattttgagtaataga
gaaaacgctaatctgtgatccaactgaaatctacagtggtgagggaaacaattatgcagaactga
gccaa

>chr20_45987793_45987993
ctgtttgtttctgcgggtgctgctttgaggggaacaaacaggactgtggggttggtggcagatct
cccatgcagctaagtccacgaaccaatgaggaaataaaatatgccgctggtgtgcgtggtggatt
ttaacctgggattaaagcttgttgcataagcagctacagcatcctccccgcctccctccctctct
ctgat

>chr20_48782193_48782393
tgattttgactcccaggggacatttggcaatgtctggagaaatttttggttgtcacaactggagg
tggtgatttactggcatctattgggtagaagccacggatgctgctaaacatcctacagaggacgg
ttcccccgctgcctgacaacaaagaatgacccagtccaggatgtcaatggggccaaggttagaaa
tcctg

>chr20_48782393_48782593
acctgtaagtaaactgaagaggcatgcctttttctgttgcaatggagctcccgtggatctacaat
tcaccccagcaattccatttccccccacctatgtcacaaaatcccacctctgtaggcacagggag
gtccagccaagtgtgtttaggatcgctggaaactggaaccaacctgcatgcccgttaggaggaga
cagat

>chr20_49434793_49434993
aagagttttgcagcctggctgacctggtgagtggctgcctggaaggcgtgggtttaggcccaggc
cagacttcaggcccttgctggagttttctgcggaagccattttggtggtgagttagcaaccagga
ggaatggggcagcggccagcgctgagaggaaagccacgtgcaaacactccctcctctctctctct
ctcgc

>chr21_45148172_45148372
ttgatcagggtgaagcagtcacatgatctctcctgtcctctttcctcattctggtttcctaaagt
ggtcagaggtctctgcaaaataagctaaagggagggttctgctgggtgggtggcttcggcttctg
tgcagcgctcaccctgctctcccctggaacgcctggaactagggtggtcaggctggggtggagga
gctcc

>chr7_44679875_44680075
ttattaataccctgcctggtgactgctgtgtgtaaattctgcctgagcccacatctctccagtgg
gaatatgattgacattccaggtgctctaagtaagttggaaaagcccacagagagcctcagtaaaa
gttagctatacctatccctgctgctattggtgggtttttttttgttttgttttgttttactatttt
aattg

>chr7_100143064_100143264
acacatacaagtggcttcagctgccaaataattcagaatacttcatatgccctttaggtgccctg
tgaaaaagaaactgaagtggataaagacaacctgactggctggaggcttgagtcctagtaataat

gtactttccattggaaaaacttactctattctgagaaaataaattgatctctgttcctccccagc
acttc

>chr6_7146601_7146801
agcaggagatgggaggggaggaggcgaggtggggacggagaaggaagtgtgtgcacacgcagaca
gcctgggagggcagtgaggtggagtcactgcaactcggtgccagcaagtcaggagacattactgc
tggaagtatccacagttagctctgacttaggggacgtcttgtggccataaatgtggaggaaccga
gctct

>chr6_7147401_7147601
gactgccagggcaagacatgcagcttctgcagaactcatggcagccgttttccacttggccgagc
tgggtctgtgaagcagagaggaatcagtaataggaaagaaatgtagttgtttttttccccccttag
aatacctaccatactgcatttcagcttggagtgcgcagcatgaggcatttgtggttcagaaaaga
ggtct

>chr6_34625222_34625422
ttcacacctttccaacttatcccacagtcaaagcttatttgtatgtgtattatacaggtaattca
gccagagaaagattaagaaacattttttaaaccccaaactgaaactgaaagcagcgaactggaatc
ctggcaggaagctggagaggatgacaaagcaaattcatttcccaagtacaaggcagaaagtggac
tttag

>chr6_34625422_34625622
cccgagtgatctaacccatcaagcaggtacacagctacaaatgtggggtacacagcttctcagag
ttcagagaatttaaattactggcccactaggctgagcaacagcaggcatagaccaaacagctcca
attcagtgtcagaaagtagacatgatagcaggtattcctgttcaaaacaacatactcagctatta
ttagc

>chr5_180668794_180668994
gaagagccaagtgacagagaatcccttcagaattgcaggacatgtcctcactcccacttggggat
tggctgacaatgccatctgtcatcaccaggaccctctaattccacctgccaattacctgagcttt
caaggtaaaacatgttcaaactgctgcccactcagaacttttgcacctggcaaaaatgttcagcc
acact

>chr3_141086910_141087110
ttccgcatctctttcaataccactgcaactccttgagtaaggtgggcaccacacagaagaagggg
gatggcagaggggcagtgagaggtccccagggaaacaccctcttcttcttccctgcttctctgac
agagcacggccatgcctctgccatgcaggagggtgatgtcctttggggaggccttgttgaatctg
tctgg

>chr3_156807106_156807306
gtttcttggcagcacgttggggacactgcgccatgagaacagagccacagaatgttaaaggcaca
gcctccttcgagggcatttgcataatgcctcccgtttacagatgaggaaattgaggcccaaagag
gtgatgggaaaggcccaaagctgcatttctggcttctggcagctgggaccagatccagctcgtat
tgaca

>chr3_171858506_171858706
catgaggctgggttacgtcaataccaaactatgttgctttcctaggaggttttaagtccctctca
agggaggtactcagggtatatgctgcttcctgcctttctccttcttcctgtctcagttatcttga
tttttatgtttattcttatatttacataagaacggaatgttaaacccttagataaaactgtcacc
caatg

>chr3_177078106_177078306
tatcgatacatgcttcattttgccttcgggtgcacttataaatcatccttaagtgacctctattt
ctaccaaagacatccacttggtctttaagaagcattcttagtgatcctcacaaaggaaaaaaaaa
aaaaggagatatcttctgtgacaggaagccaaaggctgattttcttttttttttttttgagactc
ttgga

>chr2_26981096_26981296
gcctagtaggtctcagtcttattttacccagcccctattcaagatagagttgctctcgttcaaat
gcttctgacgattcctctgtattttttttcagaattcctgtctctttgggccctgataccttagcc
tggggctcactgctacctggaagcactaatgcaatcctagttttctcccctaaagcccattctag
ccagg

>chr2_43448296_43448496
ttggcctcgggcttcctctcccctgctgctgaggccttcagtcaggggctggcatctgggagaac
cttgaaactgtttcccatcccctttggcagcaagcaacaagcaccaaaacgctccagttaacacc
agtgcaatcatcgttaacgtgaaacagctgcccgaggcctggcgtatccaccagaccgccccctc
ccctc

>chr2_70312896_70313096
tctagactaaaaatgaacacttaattgtggtaaactattaataagattcaccggccaactgtcct
atgaaaaagtcagaggcactgtagtctcttcgtagctctcgttctctccagcacaacagcccatg
ctaggaagtctagaaaagcagtaacttatctcattccttaaattaaacagaggtttctaaatgct
ttggt

>chr2_70313296_70313496
cggaggtggaaggccccggggaggaagaggggtgcattctagagctttcgggccgaccccaattt
ctcgttggcgacgaatgctaaccacgtgtcgccattttgtgttcaggaaacatggcggccgcccc
aagggtaaggaacaggggaggcggagtagcgccacgttagccgctttccctgagagattgtgaag
cacgt

>chr2_70370496_70370696
ggctcagagtcatgcccaaggccgcgcataaaagagtggcagactggggtttcaaacccaggctc
taaactgtgaacctgcaagatggtgggagtggggaagacagtgaaaagttgtgcagggaattcac
agaactacagaacccactttgtacttctggaactgctgagtggggaactagttctaaggaacaag
gttag

>chr2_87785685_87785885
ggcgtctggccaggagcaggggctggggacagcaagtgtgaaaccagctgaagcacctgcagctc
aggcgggctgcaggctccctgctctcccccctgtgaagaagcacacattgttctttcttcccgcac
atttgaatctctcagtggggtctcattttctgtgacatggcaaacctcaaaaaatgggagtaaat
cattt

>chr1_10447813_10448013
tgcatagagggctgttcttgtagaacttgtgctcatgctttgatttgggatttggggagttaggg
caagccagaaagtttttctggtggataataatgtgggttgactttcttaagcattttaagccaag
cacttgagtttctaacaactaaaaagctaagtcagcctgacacagctctagcgcgccctggcttg
attct

>chr1_23882413_23882613
aagataaaatagtctggaacctggcaggcacttccataggtgattatgacagaagacactatcct
ctctgggagttatctgacctagggccaaattgctttcactctctaatgactaatggattggcatg
ctggtctttcttttgtcctggccacctgaacaactcagtcctgggagtcctgacaggcaatttcc
ttgga

>chr1_23882813_23883013
aacatgctgtggcagaaaagggtataagacgttgagctcaatgcctggctctactgcagttcatg
gtgtaatgtggggcctaggacctcagaaccgctgccttagaatcagcttcctttacagatgcaga
aactggggctcaagagaggcaaagtccctagcctgaagttatacaggtaagaggtgacagagcag
ggatt

>chr1_23883013_23883213
tatgtccctttatggtcgtctctaaagccagcacagcacacctaaaaaacaatccaagatagacc
caacccaggccttgtaggtggtttggggcaagggttcctcacatgaggattgaagtctgctattg
ctctgcactgtgtgtgtatttttttaagagagtgttcatatcttccttcagatcagagtgaccag
gacac

>chr1_27113013_27113213
agatttttgagagaatttgatgtggacagagggaaccctgagaatgcatgtctttttttccccttg
tgaatttacagagcaaggacagttcatttctcagttttgtctttgcagagagagtgctgcttgga
gcctcatggtggacttctagagattcaagtcacagccttaggccccagtcacaagtgtgtcccca
ggctt

>chr1_28835213_28835413
ctacagcttcccagagtcctgtggacaatgactggggagacaaaccatgcaggaaacatatctag
tatactagattttaagttgaagtaggatcttcaggagtctaatcattatttcttttcttttagga

gagaagacgatctgcacttcgcattttggcattgacatttaattttagggtcctttatatagaag
ggaga

>chr1_32644813_32645013
tttggaagaaggacattgaggcccagagagagaacagaacgtccagccacacagcaaatccgtga
tgaagttgggactggagtatgggtctcctgagtctcagcccaggactctatccctcttcccgagt
cctcggagttcccggatggagtcacatttgttcacggccagggaggaaggtttgatggaggcctg
cagga

>chr1_36840413_36840613
tggcctgctctggctcatttggactagaagaggagattgcaacaagggaggtcctggcatgggca
tgtgactaagagtcccttcagctccaaccatcagttgttgctttgtgcagtgactcagtcctgga
ataaggcctgccatggggtcccatgtggagttcaagttcttaccacccatgatagcccaaatcag
gcaag

>chr1_37943613_37943813
cagtgctccaagatactagcctaggatcatgtgcccggagggaggcaggtgggcggatacatgcg
cacactgcatacctgagacaaggagccaggcctgggatgacccagctgccatggttttgagaggc
aggccagaaggcccaaggaagggggtgatgtgatgggggaagcactgggcattgtgaagacaca
gacca

>chr1_38465413_38465613
ccagcgtctagcctccactgtcccgactgtccccagcgtgggagaagcaggggcagggagccacc
ccactctactccttatgagcagtactcccaccctctcctgactcccaggaagggactttaggaat
atggataaaggaacccttaaccagagctctggagtacagaacttagagacgggaatgggctgtgc
ctgcc

>chr1_38465813_38466013
ctcatccccccacccccgctgtgtcacaccagcttggacagccttggcctgtaacatctgcagca
gctgtggcggcacagatggccagatgtcaggttttgccagcaatggggagaaaaaaggaaaatga
agtgtcgggggaggcagaggggaactggagacagactgggagtgcagtgtacccccctgccacctc
ctgct

>chr1_38466013_38466213
aacatctggcaaaaccccaggatctttccatcccctggggtcatgatttccctcctactgcctct
gggagagaaggtctctcccaccttttccgatttctcagggtttgggactagggagctgaaaggag
agagagcaagagccactgaagacattttctccccaatcaggccccatcttgggtggggagtagga
caagg

>chr1_45272413_45272613
ctcagtgatgtcaagggtttttaccttctaccccttcttacctgtgttttagaacaagtggatc
agatagtcccaggctgcctgggttcctaggtgctgagaaggttaaaggctgttagacacatgagc
aggggacaggaggctgctaggactgggatcctcggctagtccttgccgactgagctccttctctg
ggcac

>chr1_45273613_45273813
gaggcccactggctcagctggtaggcaccaggccttataaagtggcagtgactcggcagtgaacc
ccgagtccagcctcgtgacctttgctgacccaagtgtcctggggcttgtccctagctggctacac
ctctagtacctaggtttccccaaagttacatttcagagatggcagaggctgtgacacctctgcca
gggct

>chr1_90372412_90372612
ccaggctgcatatagtttgcttttcctgttcgttgcattccaacctgtcaacggctattttctga
aggtctgtgggggctcatgtagaaaaggaataggtctgggtggaggccaagaatctgaatttgta
tcatgtaccctagctcatcttaattcaagtgttagtctgtaagaaacagaagaatgggtaactca
actcc

>chr1_110546677_110546877
ctggtattgcctcacctcttctcgccgcgagcattgacagaagatctagttgagtcctactttga
aaggagtattgcttctgactgtggaattggattctagctgtgtgtgtgttatatcctgttactgt
tgagacacagaggcgggagtcggcgggggaagatatgtgctgagattagtcagcctgccctccct
ctccc

```
>chr1_145455843_145456043
caaaaatagctgtaatcatttttttgtaaataaggacaagatcaaggtcaagtgtgggaaagacag
gacctatgtccttgtgaacatgtgtgaacagacacaatcatgtccttgtggcctgaggaccagag
gctcagaagtcagctgtactgcagggggcgtaggctgttctctacaatttactttgcattgccct
acaga

>chr1_150536576_150536776
ctaggctaaaaggccctccccttgatgagacaggcagagatcatctcccctggaacaaggtcacc
cttcctctctcccatcgctacattcctctcctccttactgttgactattggttgcctgcctcttt
catcacaaaatgagactcctctttagctttagtagagggttgggcaaactttttctctacaggag
tagat

>chr1_150540976_150541176
atagcaatagttgagtctcccacgggaacaggatcagggagagtggaagccaccattcagtatct
tagggttccagttaaataacttttggaagctcttattaaaattctgttctgcagacagcatagtg
ctcttaggcaaaaccttggttcctttccctgactatgtaaaacctagagggtgaaggaccagtga
ggctc

>chr1_150541376_150541576
atacccagtccagacttatttagaccaaagagcctttttttcctatcagtgttttccaaatttta
tcttaagaggcagggtcttgttatgttgcccatgctggagagcagtggctattcacaggtgcaat
tctactactgaggagcacaggagttttaaccagctctatttccaacctaggcaggttcacccccta
cttgg

>chr1_154943576_154943776
cagaggcagctgggggtggaaaagggaggagatctcagagctcagcatttcccttcctgtcctgg
ttggacactcgaattcccccaccctctctatgcccgaaagctggctggccccagcccaggccacc
cccaaccgctaccccatacagtaagcctgggccattagcatcctctgctacagcagctgtggact
ggagc

>chr1_154943976_154944176
ctcatagccaagcactgtcctgtctcccctttccacagggcttgttaacacagcacactgtcaag
ggtgagaatgaggcaatcaggtccccactcccactccaaaccaccccaattcacatccctcctct
tacagggccctaagccccaccaaagagcacagcatccttcacaccagggaaggagcctggctgga
ggagg

>chr1_155953376_155953576
tacgaccctctctgctgtttggagaaggcctctgggcctaaagccaataaggactgaatttcctg
ggatttcatcagccccatcccatgagctaatcccaaacccagagccaggaggatgtgtcagctag
agattttcaggaccacaaagcagagcctgatatcttaaagggaccattacttgcttacacatcca
cactg

>chr1_156075176_156075376
ggagaccccagccatccctactctacttggcagccccccctccccactcctcctgagagtgcccct
tgctccaccccagcggccaggaaatctcctgcagattcaccaccaccctcctggctgggagttca
ctttcctagttgacctcctggcctgagggccagaggagagctttcaacggggaccttgaggagtg
tgagg

>chr1_156092776_156092976
ggaataattctggctgagatcccaggcccggcaggggcgctgagtcatggtagagggcagagtgg
agagtggacaggagaccctaagcttgtccagtcagaaaagcagaggctgaggggtggccttttct
tgagaactacattcaagttgcagcaagaaggacagtggtctgaatttgacggggacaaatggaag
ggaga

>chr1_156096376_156096576
ctggggagccggacttccttgtcccaccaggcacagctcttcagacccctgccttgggtcacatt
tgcaagtgccaactctcatttctaccttattcttttcctctctgttcccctccccacccccctctc
ttccctctttctgagatcagatttgccagtgatgggaagagttagaaacaggatgcccagccctt
ctcgc

>chr1_161171576_161171776
ggcggccttggcatcctattctttttttgttttggtttggcgggagctctcagacccgcccagccc
cacttaggctcctttctccaatctcaatttatgacatctggaaattagctggctttcccaactcc
```

tgtcttttggattcagtgatgggaaagtaattggcaaagcctggggctaccctataagggcaggg
ctcag

>chr9_35728600_35728800
atctcaatcacacagcccccacacagatcccatcacgactgcattatcttctcctagggagccca
tcctatgcacacagcttttgcacagataacctccataacaagcacacccggcacccagtgtctct
cctcccattatgtagacattctccattcacccagcaagcattccttgagggccagctgtggggaa
gaccc

>chr9_130834379_130834579
ttacctgtaaaggagcacaagttagtcctgtggggaacgtttgaagtcttggagagaagatctgc
ctttttgtaaataactgggcaacaggaagacatcctgcctcttgtacccccataccacttcctgt
tgaattagcattagtattaactgcctacagaaaaagcagtgctgaggctttgcagaactagcagg
agccg

>chr9_131901579_131901779
gaaatctcaaagtagccactaggtggcaggagaggcacactgaacttggagagggtttggtgaca
tttatttgaggcagcagaaggaacagggaggggagggcgtgcctagagttgttggctgttccgca
ccttctccacaggtccgggttttcactttgggtctaggctcttgggcatggtgttcaacagtaga
cccta

>chr9_131901779_131901979
ggaggagtgtgcccaggagccggggtggctgcagcaagggcccatcttgccacgtggccgctggt
tgcagcacacgttgtgttggttctccagagcgcccaccctcttccacctcggagcagtgagcagc
attttgcagtccctagttggtgagtggcctggcctagctcactggggacctggaggcttgcatgg
agttc

>chr9_136216179_136216379
gtacttcgtggcaccttggcttcttgttagatgaggaaaagcatcgtgctctttgttctcaggtg
tttgtgtgcagatgatgtaaaagaatatttgctatctgagagatggtgatgacattttaaaccac
caagatcgctgatgcaccaacacccttcctagtggccccagacatgaacttgacatggaatttga
gcctc

>chr12_6444739_6444939
tgccagcttcatttctggttttccctgggcttcattttttttttctactataagagacactttgt
gtaaagataagagggtgtcctcaatgtctgttaaacactggggagcaactgactcaccagatcac
agaaaccctagggcagggaaggagggtcaggcagcccacagccacccagcctcaccaactccaca
tttcg

>chr12_6446739_6446939
tgagatcccatctccctcccgtgaagccaccattgacgatgtctgtttcctccaccccggctcct
tagacccatagtccctgtgaactcgaagcacgtgaactgaccctatctacttttggctcttatca
taatatggttatgttgggtgacagatatgatctccccaaagtgaccacgaaccttataagatgag
agatc

>chr12_6641139_6641339
tgtctttgtttcatctttcacattagcccagtttcatgcagcagagagagggttatcagtgcaga
gagagatgagtgagcccagagtcctagggcctgtcccgggatggcagatgagcttcctgccccgt
cactgccacctttcccctctcaacctctggaccctgcacagtgaccagacagcctctctggggag
aatta

>chr12_6641539_6641739
cccacaatgtcaatagcgtcacagttgagaaaacctgctctagaccaagggttgctttctgccgt
gtgcctcaccccacccccactcgtgttccctaatcccatctccaaaggttggcagcagaccggcc
caggctcgtggaagttcagatcatgatcccctccagctctgcaggagacaagacctgtctcccag
cattc

>chr12_31902133_31902333
atccacctcaccaagaattcagaggctaaggtttttaaggatgatttggcaggtagggcattagg
aaacagatgttgtggattggttgggggatgaaatcataggagtgttgaaactgtcttcatgtgct
gagtcatttcctgcaggggagtggtcagaggaccggttgagtcagttcctttgttgcagtcacgg
tcact

```
>chr12_45628133_45628333
aggagttcatgtgtaataatttatttctccagaaggtcacctttttttcctactgatttttttttta
ctggtaaaaatattgcaaacagatattgttagtctcgtttggtaactgaaagagctctgagaacg
gaaaagttagagtgacctatccaaatgaaggagcagttcagtgttggtgcaggatattagattcc
aaaat

>chr12_45629133_45629333
gtctgttctaagtagggctgtgaataagagtggggtacagtttcctgcaataagagatgggaggc
ttaacttcagcttggcacgtaagaaagatcaaatgtttactgcaatgtgggaaaaccacctcctg
ggcataatagaaatccaggcctggcagtaaacaattctattcaaaagcagcttctcagggctgag
gaatg

>chr12_46776333_46776533
gactgattaaggcacctgcctccggttccatgtcacttcgtcttgggacacgtggcgctaatgcc
acttttcaaccccccaccccattcagcgacattaacccgggcgacagtccagggctaaacagttg
ctgaaacagttaaatgtgtgttttattgaggtgaaccacgtacacaacaaccccctttgagtgaa
acaat

>chr12_76338333_76338533
agagggaaatgaacaagtgagattaacaggcagatgagatgatacaaagcaagcacaggtccagc
cagggttaacctgacacaaaggaatctgaataacaagaagcctttgaatgactcaggtaaggttt
atctcctccaaaaggaagttaacaccatgttactcacagtagcaaaattactcatgaactttgga
ggaag

>chr12_76338533_76338733
tctcagagaaggctatggtcagaaaatggaaaaggaaacaagccacaggctcatcttcagatgag
gactctgcacagggcttgacctttcaatcgaagaacaaccacagtagtattctgagatggccaag
tgtatgggaaagtgactgcttctctcccactccaatgtctactgtaagtcttgttcagccccgag
acaga

>chr12_109231871_109232071
gttatttttaaatctccacttaattcgattttggtaaaacacgacctgtaatttttctttatcg
gtaggtaataaaagcttcagatgattttactgatcactggtatgggcatatttcatgactttgcc
ctttcatctcttgcatagttttaccctcaccaagcaagaccttccctgcctcagcactgtttgcc
ctctt

>chr12_122884647_122884847
ctcctcggcttgagccactgaaaacacttacacaacgctgggacctttccaaattgacttgtttc
cccttaagaaagggcaagctatttgatttcttccttccacttcattcacattcatatttctgta
tatacagaagagactgaaaaagataaataagattgaagggcatttgcaaattttcttcaattctt
gagta

>chr12_125402447_125402647
taacctatttgggctttgtttgtgtcgtcgtttggttttgtcttaaaattgtggtaaaatacaca
taaaatttaccattcttaagtgtacatttctgtagcatgaagtacattcagtattgtgcatcagc
catcaccaccaagccatttctggagctcttttcgtcttgcaaaacaaactctgtccccatgaaaa
actga

>chr11_6627624_6627824
ccattaggttctacactatttctctttaatactttgcaaagtcatttgttccatttttcatataa
ttctcaaagggtaccctgataaagacagtaagcactgttactgttttgttggtgaggaaatagag
gcttaggcagttgcccagtgggcctagcatctaggaagactctagctgagtctaggaagactcta
ggaag

>chr11_46575824_46576024
taaaaaataaaaaaaaccgaccttccgacctcctgttactcaagaggaacaataaaatacaggtc
acaagtactgtatgactcagaaaactgtgatgcaacctcagattcctacaggattcagaaagggg
aaagagtagctacaaatatcaataccaagaaaaaaatcaaaggatcaggattaccaaacaacatt
tctat

>chr11_46576024_46576224
caatttccagggacctaaacacactaacagctcaaacaagagaaagctttactgatgggaagaag
tcagagggagtaaaacaattacacaatctctctttcatctgaagaaaaatatttgctgttgcttt
```

aaaaggataagggacttctctcctgatatgctcttaccagccacctgagaagagattttactctg
ttccc

>chr11_61739024_61739224
tggctaaacatgcatgtagctgcagtgaacatcgcagcaagactgagggacagcttgtgctgtag
aaggtcattgggaggaggctggctgggaacaatggaagggaaatctattggtgggggagtgtgag
tgtgtgtgtgtgtttatgattgtttatatgattgcttgtgtccgttccctactttctggtaactt
gaggt

>chr11_61739224_61739424
gtcagaactgcaatgtctaagcctttagattcttccgaagctgtggcctggtcttgggacctttg
ttgaccagggttctacctcccccaccctccagctcagctcctcgtgacttggaggcagcagagag
tttggctggtctcccaggacagccgcctcccagggccaggagtggcgggagggacctcagatgag
agaag

>chr11_62320224_62320424
tttcctaagcaattctcaagccccaaacccttaaccctagccgaaaatataaagccaagtaaaat
catccttccccagctcaattcaaatcccagcctgtatttaaacttcaagcttcaaataaggagtt
cctgaatttcttagatcagagagctttgatacacagaaactctccaattacactcagtcctaatg
aaatc

>chr11_62389824_62390024
atcttcaccttattcatccatccagtcagtcacgccacccaatagctgttgctcaatacttatta
cacgaatgaaatctaaggcccaccacatgatcctctgggtgtctctcctggaatcctcactataa
tactttgagatagataagaaaccaaagttgacaaaaattatgtcattgccacagggcagttttgt
gtcct

>chr11_65187824_65188024
tctccttaaatacattcctttcccgagaaaaacgagctgtgtggaacttggaggccagcaaagct
tctacataaggaccctctgaaacagtgcccttttgtccttgaaaacccagggtggcaaaggcagc
tccagatacaagggcacaaaggagacttggttccctttgtgacagggaagcctatgctttacaga
atcct

>chr11_65255224_65255424
cctgaccagggtggaataaccaggaggcaggcgcacgtgaccgtttccatggtgacctcccttag
caaccctgactggtcctgtttatgaacgctggaaaattccaaccagcctctgtcctgattttttt
acctactctgcctccgcctgctccaatgcaggggtgaagtcactgccaacgtttaaccctttccc
cacgt

>chr11_65255424_65255624
gggcctggctccagtcagatcgagggtctgggagggctgcagggatctctcagtcgccaatggtt
gcagacaatccagtaagccacagctgttacttgctgcactgaaccctgcctgcgctgagtaccga
aagcaggagggaaggtagccacatctcttgagcccggcgctgtcctaagctcctgacatccccac
aggcc

>chr11_65255624_65255824
tgagaaaggggcaccacaaacaggaaccaggctgagactgagcctgtggaccctggtagcaaggg
cgccctctctctaagaagccctctgtcatgctaccgatgttgcagaacacctggggcggagtagg
gagtgccaaagcgcaggggatgagtgcacagcagggagacttctatggtctgcaggtgagctgaa
ggcag

>chr11_65255824_65256024
gcccctgaggaggtggacatttaggctgaatggaggacagggaggtagcaggacctgctgtctcc
aggacccctaagtttctaggccagagctgacggctggtgggtgcagtcagccaggctccagggaa
ggctgctccaggcagggctgagtcttcacggttcagcttttagaactggccaggttggaattctg
gctgg

>chr11_65256024_65256224
gggcagacagggagggcaaaccacaaacacttctgcctgatctggctggggaatccacagagacc
accagctgggactggggtgagcctctcagagcccctagcaccagcccaaggctgcatgggctgac
tgtgtgggcggatgctgcctaagtcatgtgcagagccgtcctgagctgaggcagaactataagac
atgaa

>chr11_65256224_65256424
ctctgttttcaagacaggtgacaatgacaaacagcaataaaagccacacaccatagctcaggata
acaagggctgagatgcgtttggacaatgagaagaaaataaaaattaaaaaaaacagctgattcac
caaatgaagggcacagtcccaggcgtgctggtgacgtcactgggcagggaaggctgcaggaatct
gcagc

>chr11_65257024_65257224
ggagggcaaagaggaggagtaacagcctcctccgtcacagcagtactcgagtcacatcagctcat
caccaatgtgcctcccccagagtaagggcagcaggggcggaagaagcccaggggcacctaaatat
tgtgggttcagcacttctgctctccagaaatgatccaggtccactgttttctcaggggggagtatt
ttata

>chr11_65257224_65257424
tgtctatctctagaagcttccaaggggaaaaagtgacttcaaggggaaagaggagctgggaagac
cttggcagctccttccccttgcccggccttcctttccctactctgggctccaggctccaccccac
ctcctgttaggccttcctgaacagcagcagcagctgggaatgcaaaatgacatccctatgttgtc
cctgc

>chr11_65257424_65257624
cctttgtccagggtgagagggacccttcctcggtggagttgacacagacctgcctctctctcatg
tctgtactgggagtgagcctgaggcctccctgccaaccggactggaaactctggaatccccttttg
tcccctttgatcctcagaggagcgagggagccctgttaacaaacacatagtcatccgagaaaatg
taggc

>chr11_71814752_71814952
agatgatccagatgaccagtacctagtcggggaacacagagggtgtatcgcccagctgggctggg
ggtgaatacacagagggtgtattctccacctgggctggggatctgcgacttacaggaagaaggga
ccttatactaccttcacatgctgacgcgcagcggagataaatcctggtagaggagatagaagcca
cctca

>chr11_118782990_118783190
gaggtacgagaaagcaaagggatggggctctgggaacacgtcaccgtgcctccctcttaggggggt
taggtggggaggggtagcaagctcacaaacagggagcaggtgcgagtcacctgagtggaaggcca
ggcgcataccaggagaatgaatcggaggagcggaagaggcggcttgacaaagggcctgagatatc
catcc

>chr10_76969594_76969794
gggcctaatgcaggaggtcagtctaaactaacggtctcccatatacattttatctaatagctctg
tcacccttcctgcacacccggggaactcgggtgcggcattctaacgcctgctcagcatgattcca
catttctccaggcccataatccagaggaggttgggcccggagcaggcttgcctcgccaagctcc
ggctc

>chr10_103911210_103911410
cccatgaggatccttatctttcctgagcggcattagtgggaggtagccaaggggtagtgggtagg
attgaagggattgctaactgctaatatctggaatgtggcagaacttggaattaacttggtaggtc
tgagcattgatactggagttactcatgacaaaggcattgatactggagttactcatgacaaaggc
agagt

>chr17_7482476_7482676
gctctgtgaatgacaatgctgactggagtgctgcccctctgtaaagggctgggtgtggatggtca
caagcccctcacatgcctcagccaagaggaagtagtacaggggtcagcccagaggtccaggggaa
aggagtggaaaccgatttccccaccaagggaggggcctgtacctcagctgttcccatagctactt
gccac

>chr17_17656075_17656275
tccttctttccggccgattcctcacccccctcaggaggctctccacacagaccgtgacctgccctg
cctggcactcctctgcccagcgtttatatctctttttgtgcactctccgccctcatcacacgtgt
gcctcagggcagggccctgtctgtggcctggcacacagtaggtgctcggtttgtgttgttgaatg
cctcc

>chr17_17744075_17744275
agccccccctgcccccctgccccccctgcctggccctgcatatccttcactgctgtgggagccaagc
tgggacctgcccaggttatggcagaggggccaggctgtgggaagagactgctcagagcctgagtc

tcccctcccccacttctccatctgtatataatggggtcagatgactgttgacatgggtgattctg
gtcct

>chr17_27073473_27073673
tgcccagagtcttagctgtgcttgctggacattttccttgccccagcttctcttccttgtgtgt
gtggtgtagtagagcctgtctgagctgtggagtcagacacacatagccaggtgctgcttcacctg
taagcctcttcgtttcatctgtaaaatgggggcattccacccccttctcagaggcaggtggcaagg
gccgg

>chr17_27135274_27135474
ccaaccatttgatctaggagagaaattaataacaatgccagcccaggttaccaaaagctaaattg
tcctggatgaagagagagaaccatacatcattccagtcctttattttgagccactgaacaacacaca
tgcatatccaacaaggattcttaaaggacagtatcattagaaagacagctctggccttcactgag
aacag

>chr17_38267674_38267874
gagtggctcttgggtccaaagggcttccccttggccaagtcccaccccaaagcccaggaagtcca
cagcttggcgcccagtcaaggcaaaaacattcctcaggactctgacaggctggggatggttccc
accacgaggatgggggaggggaaggaaacacgcgggggaggaaatgaccacaaagaagggggtga
gggta

>chr17_38268074_38268274
gttgagcttgggagcccctgagtggctaaaagctggaattgcagttgtgctgggggaggagccca
tcgggtctccctctgcctctccacctccgcccctccctttccctctccctaagcccaggactctg
gaaatagcacctggtaatgggagcgctgacagatgctgagttataggagagcaaatgtcctggct
agagt

>chr17_62224468_62224668
gcgttcactacatggacattcgatgaatgaaggtgtcccgaattaacattatactagacaatgaa
gggtgtaaactgcagcccaggagcaccactgccctacccccctcagttacgaactttcactggatt
ctttaaatgtgggctgaagataatggcacatagtttgaaggatgtagtgaggatggctttgtagc
cttag

>chr17_62224668_62224868
gactgggcagtgatcaacgcagtaaatgaaagctgcgattgccaggtgagggccccggtgctctg
tactcattttattgtgcccctatctgaacaggattccgtagaaatgacttagatgcttatctagt
atttgacattgagatatttattttgtgaaacaatgcaagcgattttaaatccccacatactattt
gatta

>chr16_2202799_2202999
ctggtctgctgcctcccacaggcctggctgaccgagatccacgagtacgcccagcacgacgtggc
gctcatgctgctggggaacaaggtgggaggcccggctgtcctcacctgggccacagggcagggca
ggtgagggggcaggggccaaccatgggccagctttcaccaagaccctgtgcctgggccaggtgga
ctctg

>chr16_4665799_4665999
ctagggaccctgaacccctctgtgattggcatcgctctgtgttaaatagtaggcacgcccttaag
agaggggtctctaggaattttggcccaaatcggctggtctaggctgaaaggcccggcacactgta
ggcgccgacaaatactcgttcagtgaatgaatgaatgagccacttaaaagctccgccgcacgtca
ccgcc

>chr16_14595299_14595499
aaaaaaaaaaaaaaaaaacagaaagaaaaagagaatgaatgttaaaatgtaggcagaggcatgaa
aaatagaaaagaaagtcagcttgcagagccaagtttatatcttaccccaccctgatgtggttggc
caacagctgatagagggcttttcggtaaagtaggtagaaacaaaccccaccaaccctcaggttac
caggc

>chr16_53132699_53132899
gtaacaagcccttaacccctggctgttattcttatttccaaactggggtgcatcattagataaat
gcagaaatcaagaaccgtcgctcctagtcaagcgtctgtggctcagttctcagagccaggccaaa
ggagtaattatagctttcctctatagtgtacggggcaggaggctgatttaataaaggaagcctac
tgtct

>chr16_53132899_53133099
gtctccagcctggctctgactcatggaacgagggagtcccagagcactacccacagagggtgggt
ggtgagcagctgagggtgggaggtgagggtggccgctagcaaggcctgggcccagagccctgaga
atgaggaaactccctgcttggccaggaggaacctgcagctggagccagaggaagtgaggctcaga
cagag

>chr16_87421499_87421699
actgctcaaaagaactagtcacactaactggaaagaagggaaaatattcaaactttgaacaagag
gattgcagtttaaaataagcgggctgatggagcctccacagttgcacacgttaacagtcccagaa
tggagcaaaagctataaacacagcagtcctacagaatttgtttagaattaaacaaacaggcaaac
aaaaa

>chr16_87812699_87812899
cccctcaccccacccgccacacctgccaacttcccggcctgcagggtggcaggaagcctgaggca
ttattcagcttccttagggcccgttggctgatgcaacacagctcagaagttgagtccaagcacca
agcccgcaatgtctgcgtcggccgccaggggtcactcctcccgcgcagaaggcggccttggagcc
tcgca

>chr16_87886699_87886899
cgtgggctcctcttctctctttccccttgggagtaacgctcagaaaggtctggccagccaaggcc
ttgaggactccagtggcttttccccagctgaacaaatcctgtggaatcttacaatgtaagcaaca
gaccccgaggaaagacatccatgctaattaactaatgacaccggaaacgtcctgaggtgcgggac
gggct

>chr15_41575508_41575708
agcattaaatttttttccctaggtgttgtgctacccctggattaaaagcctgggcgcggtggctca
tgcctgtaatcccagcacttcgggagaccgaggcgggtggatcacctgaggtcaggagttcgaca
ccagtctggccaacgtagtgaaatctcgtctctactaaaaatacaaaaattagctgggtgtggtg
gcatg

>chr15_44092308_44092508
ggagacgtccggaagagaacactgacctccaggttgcggtaaatgcaaatgccctgtgaaggagc
tttgtccttagggcctcaacactgcggccactcaggctgttctctccagattagggggcagtttg
tctatctggccccctgtggattagcattctttattggtttaggattagacgtcattcaggttgaa
cggag

>chr15_66124946_66125146
tggttaacatttaactaataatactgacgtatgttggtttagcccagatgctggcagcttttaat
tctccaaactcctggcctgaatactgatcatgtttcctcaggctaaaagccagcaaaaagaatca
cctggccaaacatcccctttcagtctgctttccttagaatggcctcccaggggagctctctgggc
aaaca

>chr15_90944396_90944596
ctccttaatttttctcctgaagtctcctagcaatctgattgatctctgtggtttgacagtgcgtg
aattaaaaaaatcttaaacatattaatgtttaagaaagatctggttaatggtttattttgaagag
taagagtttacttgctgaagtttgaatgttgggttaaaatttttaaaaaaatagttgtatgttg
ggaag

>chr15_90944596_90944796
aattagttatttcaataaggcattttattctagaaacctcagtactttaaggcagctaaaggaaa
gccaaaataatcccccaaaacaaaaaaccaaaaacctgcaatttgcaaggcagtcataatctaaa
atattttctgtctgtcccaaagtcagtgtattccccttgcctctccccctcttcctcattgtcat
gatta

>chr14_61569247_61569447
gggaagggaagtgagctcctttagcagttcaaggtttccctcccactctgggacagggtggtaga
ggtatgagagtagagactgctgagagaatatggtctctttcattgtgctctgttttctcctttg
gcctcatggggaggcatggacgggtgtcatcaaagggtgacttgaggaagtaggagagcaaatgg
cttct

>chr14_61569447_61569647
ccctggttctcagaagtctaggtccctcacctggctcttatccacaatgcctgtgctgttggtaa
tagttcacgtgcacatgtctcctccagttaggttgaaaaaaatcctgctggctcgcactccaaca

tgggctgctttgtatgtctccctggcaaggcacacagcaaatattggttgttgcctttggaggcc
aggtt

>chr14_61570847_61571047
ccatgcagcagggaagcgggtgtctgctgcaaatctccccgtgtgggggggatgggaagaacaatg
aggctgaacagacacaaagggagctgtcctgtgcccagacagccccagtgacatcttcaggccca
gccaggatcctcccttcccaaaatggagcctgtttgctcccagtattagtagggctctttcgatt
gcaaa

>chr14_75725247_75725447
aagttctgctgcttttaaagtataaaagataaattcaaagttccttatgcttctcctttctggga
gaattctgggttttactccaacagtgggaaagttttctaagctggttttgctcctgtctcatggg
caaggcagtttggtcggtggctgttctgtgggctgctgtctggaggagacggtcggtggagacgg
tcggt

>chr14_77421847_77422047
tcttatccatagatcctgcacaggaacacccccacgcaggccctggccctctccttgttatgtgt
ccctgaggctgtcctcccaggacttggttcactttcaggtctgccacccattggtgcattcattt
ctgtatttaatgttactgagcaccaactatgtcccacacactcttccaggtactggggatacagc
agagc

>chr19_1265000_1265200
gaggtgggggctccggagcccccagaccttttcccagtttcagaaactaggtggctgcatcccca
gcgtccacgcaggcgagcctaagcctcggtcagctttagggtgtccgtgggccatgcagggtctt
gggcctttcacagacacctgatctgcacgtcacagacacgcatgggtgcacgtcactacacgtcc
gccac

>chr19_1265200_1265400
atgtgcttggctggtctgcgccccagcagaaggggtcacctatgtctctgcagtatcctgtgtac
tgaaagagcaaacaggtgtgtgaccccagggcaggtcggggtccgtcctgtccccagcctgtcta
ctgcgtgtgcctgtggcaggcagggtctccggatgccgagtgcatggtgtatgactttgtgtcca
gtggg

>chr19_3983800_3984000
cttcccagcgtcccaagcactagaacaccctcccctcccaggtgtgacagccaaacctctccaga
tattgtaggagtgggggcaagtcccccagggagggagaaccacggagttaagccccctcctcaag
aaaaccttccagctcgcagtaccccgaatccctgtgcctctccatcctgtctcgctggactgaac
ctcac

>chr19_4374600_4374800
cgggacagcctcctccctggcctcctggccctcgtctgcattgagaggcttggcctctggtccgc
atgctgcccttcccgtgctgtggccttgcagcccggcctctcccctgctgtccccccttggctctg
gcctggcccctgggcccctgagtcacccctgaggtgactcagcagtccttggaaacgcatgccga
ggacg

>chr19_4376000_4376200
aatgcatagccctgcaggaagagactggaaatggtcttggagagagaactgcggggcatggtgac
agcggtgagaggctgcaggtgcacagatctcaacgagggccgtgagttccctcactccctgaga
gctgaggagacttgcccgaaagcccagagcccttcctgagacacagaaaccatcttcaccatctc
taaga

>chr19_6738200_6738400
ttttattccagagtgaggcaattggagccccacccccagattccaggctagaccggactccgggc
tgaactctgaccccaggtgaactccctctctgcaagttgagtcctaacttcagcctgaaactctg
acacccccctcgtcccccacaccacacactgaggcttaatctctggtttaggccaccgatctggac
tagcc

>chr19_13957000_13957200
agtagctgggattacaggcgtgcaccaccatgctcggctaatttttctatttttagtagagacag
ggtttcaccatattggccaggctgatcttgaactcctgacttcaagtgatccgcccgcctcagcc
tcccaaagtgctgggatgacaggtgtgagccacagcgccaggccctacaccccagctttctagct
ggatg

```
>chr19_13957800_13958000
ctgtggggactcccagccaggtgcctgaaatccccggggaagggtcccagtattgctcaagggca
ctggtggggcaggtcagacaggaatagaccccaggcctggcccagcccccacctcaggaatgact
caccacccacccagctcccgatgacatcaaggcctggcctgccaccctgacctggccccaggcca
gaact

>chr19_18485400_18485600
gtcccagccctgctgactcatactcagctgccacggtccgctttccctgctgagcacgggggtgg
gggcgggggggaagagggggagggaagggcacctgaccgaggccagaggcccagaaaggacagaa
ggtcatggctggggagaagcaggcaggagaaccgagtcttggccttaactggctggcaacgtaaa
acctg

>chr19_39156160_39156360
agctcagtgggaaaccaaaacatacacccctttcctccagtgctccccaggaagcgccaagtcct
gagcccagccagcaacttcgaagaagtgccctccaacaagtggcagtgggcacttgagcaagtga
cttaacgcagccctctccccagcccctgcatccatctgggcctcattatctgggaggtggcttct
tgttt

>chr19_39177360_39177560
gagtgcaggctcttccccttggggagaacccagttctttgacgtataatctgagtggtttgggtt
ggttggttggttggttggttggtttcccatgtgtgggatggctccggaagtctgtttgagaacag
aggcaggctcagatgggagcagctcctaccccgggccgcctatcccccttaccctggggtttct
tttag

>chr19_39925160_39925360
acaaaggaaatctcagaatttttattaaattctaggccaggcatggtgatcacacctgtaatccc
agtccaggcagaaggatcacatgagaccaaggaccagcctgggcaataaagtgagaccccacgtc
tacagaaaaatatttctcaaatgagccaggcgtggtgatgtgcctgtagtcttggcaaccgaaag
gctga

>chr19_47611760_47611960
aagatctaagagcaagaagcagaggtgtaggcaccgttaaataaattactaaccaggcccaatat
gaaattgctctacattcaactgtatggaaacgcaagagtcaaaaccgttcatctttaacttgcca
ggcagttgaacagccaggtccacaggtacaaaacaattctcccctgggatcagccttcctggctg
tggaa

>chr22_36725454_36725654
ctggcccctaacgacagtgtggcaaccgataagggtttggaacaaacatttttgtcatcccagcc
ccctgccctcccccactgcgtctccctgtggtctgccccagctgtgggcatgtttagtcaccggc
tctactagcaggcaagaatgtacctgcgagtgctggcaagtcgtgagcagccaacggcccaatgc
tacca

>chr22_36767054_36767254
tcaaagcaattaaacaaccggtagagcaccgtcctggagggctcctcccccagatctgaaggcat
ccacagaatgtccctggatcagagaaccctctgctgacacaaacacactcccctactcccttctt
ccctgttacagattgaattgtgtccctcccaaaaatatccattatgttggagccctaactcccag
taccg

>chr22_38576854_38577054
caggcagaaactctaacaaaaacaatgcattcaaaaacaatgcattcagcccaagggtgcagcct
caacgttgccaaaccgaacctgcctgacatcctgttgggcgggtaagaggcaggtgagggaaaga
cccagaagctagggggaggtattcgttggggtcatgtgggtagcccattaaaaatacggccacag
gtccc

>chr20_30307339_30307539
tctgatataactggctcttccacacacatcccctctgttcaccaagaggaagtgacatgtggtat
gttgtccaacaggctctgatgattcttaagcaaagagatggaagatggaatttcaaccccatgga
gatctaataaacttacccagagttgctgtgtctaagacctctttcatatgcaccactgggcagct
ggcaa

>chr20_30307539_30307739
ttctctggtaggggtagccttctgcagaggctggctagcagaccaggcacctctcccagactgcc
ttttgtttatagctggcaacaacccacctggtattaggaccactggccaaagacaatagtcaggc
```

aaagtgggcaagcagcctcaatccctacttggagatgcctcagagggtgtgtctgcagtactgga
atcct

>chr20_45988393_45988593
agaaacctggaactaagcccagagaggtgaagtgagctgcccaaggtcacacagctgggaagtgc
cagagatcagatgtccaccccgttagcacttcacatccctaaactctgtaccttctcctggctct
aaagaaagaagcaaaagcaaagccgaaccaagcacagcagaaacaatgaggctgaaacctacgaa
cagca

>chr20_45988993_45989193
tgtcacagttagggaggttgtattaccggtatccagtgggtagaggccaaggatgatgcaaaaca
tcctataagtacacaagaccagccccgccacaacaaggaagtatctgatccaaaatgtccaaggt
tgggaaaccctggctttaagccctttaaggaaagggaagaaaacagtccaatcattctgcctctg
gctag

>chr20_49435193_49435393
acatggagacagccaggaaatggcttcaggcctgcgaggcctaggaggagcctcttgcccaggcc
acagcccgtggtggagttgggcatcgagttccttcctgaatccttctgttctctctgcttttacc
tcctactctgtctggttggcccaaatctactagcctggctgaggaggaagatggaagacaatggc
ccaga

>chr21_35320730_35320930
ctgtgccaggtgattctgtgtataaaacagcgcagtgcagcccctgaagcacaaatattgttttg
ggttgcaaggcggtgcatgtgacgtagttgaaaatacagactcatttgtgtatactccccacgta
aggagggtaggcttcatctaaaagcctgcgtgtcaaatactcctttctaatacatcctggtgtgc
tctgc

>chr21_45147572_45147772
ggagggagccagcactaggtggagctctgccagccggggcacagggaggaacagcatccaggagg
agggtgcagcgggtgtgaggccctgtggagcagactctggagctagaataaggtggcatttttgc
tgcacgctgtgggggtggacggggcccgaggtcacaccagggagcccatattgggtcaggaatgc
tgctt

>chr6_30582421_30582621
tctccaagttactcttgcaagcccttgtgtctttcccatctccctctacacacatatatacatac
acacacgctcacacacatcctcaaagcttcccagtcttaggtttgcctgtttttcacccctggc
agctgaagtggggaaaaattacaagcagttgtgatgagtgaaggaaagtgaaaataaaaactggt
tctat

>chr6_52382641_52382841
gtccaccctaatctcagccaccctagactgggagaatgtaacggtgggcagtggtgagggggtgg
gaggaggtggcgaggacagagagaacatgatcttggaaatcagacaaactttcctttttcctctc
cctcttcctttccacaaagacctattctgtgagaggcactgggctaactgttgaaacatggagat
aaaag

>chr6_52382841_52383041
acaccacccccggttggaaaacatacagtgcagcagggagactaacagacagatgtacgattaca
ctacagacgggcagcgctcctgagaagcacatacagtgagccacagcaggcgagggccccactct
ggctagggtggaggttgtgggtaaactgttcactggtcagggtgaacagaacggcattggaactc
gggga

>chr6_74289479_74289679
ataccatgcactggcaaacatacaaacaacaaaaaaacagggtattctatatattctattgattt
ttaaatgttggctgcagctgctacattcctcgagagtaactgagtcccacacaagaaaccctaag
ctcaacagattctctattagatgattgcatttttttcagaaacaatctcctaaccaccaggcctct
ggcca

>chr6_74289679_74289879
atttcttttctgagttcctgttcctgccagaaagggcaaattcattatcctgtgaggtccaagct
ggcctcttggaggtacagcaaagacttttacccttggcctggaagcaaaggctagccatgtggcg
cccttgggcttttttttttttttctagaaagttttctggtgagttaaaaaaacgtgctcagagt
cctac

>chr5_133839101_133839301
tggggctggccctctgcaaggagatgccccctcctccatgcaggaaaacctctgtggtctgggag
ctctcaaaaggcaggaagttgaatcatcaactccggtaacgtctctgtcagcctgtgggtctgcc
cagggagctcagggctccaggcccagggggctggctctgcgctttgtttgctgctttctctgaac
aggaa

>chr5_172192195_172192395
gagcagggctgctggtctgcacctacaggctgttctcaaacaggctcaccccgcagagctctgga
aacgaggtgagcagccctcccacctgctctccagaaagcaacctgcagagaaaaacagcccgggt
tgacctggatccacccggcgggcgcggggaggggccgcagcggcattgtttgcctctgtgtatgg
caacc

>chr5_179246394_179246594
atggcgagaaagcaaaggagctccttcttggggggtgagtggggcgccttgagcgcttcctcaaag
ctatgttcccagagccacaggccttccttgtgtccctcaccctgctcagaccgggccatagccgg
gggctggggcaggaaagccggccctcggcgggggccacgtggctctcaggcgcctgggctgctga
gtcac

>chr3_39192596_39192796
ctcagcagctacccaggaatcaagatcctgaccatggcagtgttcccagggtgagcacagccccc
tccctagggtcctggaggaaggaaaagcagttcccaggagctggctcagacagaaataccacacc
gccgaggaaccccagagacaggccctgaatctgagagccaggcttcttctgggatctgagccctg
acaat

>chr3_39193196_39193396
agcacctctagcccccactgaaaaaccagcctgggtggccctgagagggcagaccgaaagccttg
gcacctcctccccaaccatccctctgaaggagagctgggggaggggacggctcctcggctttggc
ctccaggacctggcactggataggtgccaaatgtttgctggctacatgaggcacttgttcctgca
gaagg

>chr3_39193596_39193796
ggtgctgggatggagggtctgaagggtgggacagggtacggctggggtgaaggtaggtctctccg
aggaaggaatgtaggcagagttccagatagggggctccgcatgggtaaggaacaggaggagggacc
agcacagtgtgatcggaggaggggcagcccgcgaggctgagcctgggctccagggcaggctttac
ctcga

>chr3_52086760_52086960
ctttcaggaaggataaaggtacccactagccaggcccagcctggcgaagcctgcttccctctctc
tattcccctccctcgttgggggagttcaaatgctgaggcagcacccctagccctcttgacccagg
gggtggtgggcagggctgagaagctccaggcagccccagttgcagcccctcccccaggcctcagc
ttcct

>chr3_53304360_53304560
gctttccagcaacgccctacagcccctgtcaactcctgcccaggggcgctggacaggctgggctg
ggcaggggcggtgctggtgacagacaggacactcttaggggctcctcctgcacagtctgcaggac
gcagtagggagagatcttcatccttgtccccttttctggtacctcttgggtttccctgagcaagg
ctggg

>chr3_133291710_133291910
ccctccatcccccctatcttgtaagaaatagtatacccactttacagattagggacttgacgatag
gatttattgaacttgaccttggtaaacataatccagagggtggagttcagacaaaatccctttct
acctaccaaccagcctatgaaagatcttttagtaaatgggacctaaacttcaggctcactgctcc
tctca

>chr3_171858306_171858506
acattccatacgctgacctgagaaaccccttttacagacagcagcttgctgctgagtaactggaag
tttaccagcaggtggtttccaccagggccggaaaatactgacgggcccagagcagaaagtccagg
ttatggaaggaatgagagcaaaggctgtgattgtacgtgcaactgtcatcttgctgggattgtgt
aatgt

>chr3_177077906_177078106
tactttccagagtacctggcattatctaagagaggagcactccggctgcccaagaaagggcctga
actgggagcttggcggcctcggggaggggcggctgctcagagccaggcctttccctagtggggag

ggcatctgcaaggcacaaattagaaacagactatgagcttttgcttcactaaatgcagatgcact
ttaga

>chr3_183903106_183903306
agaccaattggaggaatattcacgttaaatataaagcagtagcatcattttggaggaaattccta
acttcccgagggaatcggtgaaaggaaatagccttttattggcatttattgagtgcagagaagtc
tgctcagggtgtaggtagggaataatttatacaggccacgttgtcctggttttcttgccctttgg
cggtg

>chr2_27603896_27604096
cgcgacgcccgccaggccggcctaccagcagctcgctctgggctctcctatacacgcgcaggaca
cacacgagcacacgcacacactcacatggggctcgccacatgctacagatgggaccaagtcggg
ggccacggcagcagaaaaaaaaggacacgatgcgtacgattaggttttggcctttagtctgaaaa
agtgt

>chr2_43446096_43446296
gcaacacttacagctgctcacccccacagagccacacacctacccacacacagcagcagctaca
cacaggcacacccaaacccactcccatagatcacacacacacacacacacacacacacacacc
cagactcagacataccccaaggacccacatacctacctacacagcaacagacatacccaaacccc
cacca

>chr2_43448096_43448296
aatgaatgtggcttacccccaacattccagctgatcaaaagtacttgtttagaaaaataagttta
ggaatgaagtggaaacaaggcctttgaaatgcagcttaacctaaatagcacattttagacttttc
ctccccactgcacattctttatggtgtaggggccagggagcctgcagtcttcacccctcctgagc
cctcc

>chr2_70359496_70359696
gcttcagcacagcctggggaaggaagccctgccagcaggcagcgccaggccaagtgtaccctctt
tccttatccctgacttagaaaaacaaaaccgataggcaaatccactcatcggcatttctgaatcc
agttgttaaccaatcctcttcctgccctttactctccttttcttccttttccagaaaatcctgg
aaagc

>chr2_112221529_112221729
aacaatgtgtgcttcttcacaggggagagcagggagcctgcagcccgcctgagctgcaggtgct
tcagctggtttcacacttgctgtccccagcccctgctcctggccagacgccggcctctgtgggcg
gcccggagggcaccgtgcgttggtgcaggccttcgtgggcagcagcgctgacgtttggaaacaag
gcccc

>chr2_112221929_112222129
ctttcagaagggcaatcacgaatataaagtagagaattagtagcagtgtcaagagtaaagccccg
aggctaataccaaacttctaacataaacggagtgttgaaaggcacatgccaggaattatataaac
cgaaggaaaaagacaactgggggtgtgttcccatgcattcgatttggtccaaggagagccaggc
ccacg

>chr2_219270556_219270756
gggggtgaatatggttggcctgcatcaggtggccttcccatttaagtgccttctctgtgactga
gagccctagtgtgatgagaactaaagagaaagccagacccctatcctgcttctgtggttattgcg
ggggacttcagcaagtggggtgtgtgccttgcacctgcggctgccgtgggcccccccccccccgc
ttcag

>chr1_8772813_8773013
gctttagaaaaattcaatggattttttttttaatgaaaaacaaaataaaaaaaaaacacaggat
gtaaccacagctatattggtctttttctcaggaaggtcagaaaagctttaaaaacaggaagtctg
tgctataaggttgagaaatgtttgttttaacagcctaaggcgtcatggttgcagtttgcagtcag
ctgct

>chr1_23881613_23881813
gcagagtggagcccttggaagcagcgctgcgggaaagagcgcggcaggaggttctcaggtttagc
ctggggcatggggaagagtcgtggccacgaggggtgatgaggccgggaagacagataattgaagg
ttgtccggcgcgtccacagctagagagctataactggctttctgaaaagggcaccatttctggtg
tcaca

>chr1_23882013_23882213
gaagcctcgtggatactttatgcgccttatagtttatccgaacaacacacatggaagggaggtac
cgacatccccctttcacagatgagggggagtcctaggtctttcattccacaagcgctccctggga
caagcacccccctggggaccaagcatgtgccaggctttgggaatggcgagggaaaagagccaattt
gaaga

>chr1_27049813_27050013
gaatgccctatcacaggggagggtcccatactggaactggatcaacagctgtcgcctgtccctgg
aatcacaggaagcggaggacggggtgcctttgtatagggagctctgaagccagacaatagtttgt
gctggcggggccatgtggcgagtcatgtgacctggggctgttttctgctggggtccttacccatc
tgttc

>chr1_36839613_36839813
cagcctgctcccccagcacatcctctgccaccgctgcctttcctcagcaggactccaatgtgctg
agcacagcaggcctgggggccaggcagggcctgaaagtccagggtctgcagcttggtgacctagc
tctagtctcctccaggaagcaaggttcatgagttagccccacataaggagactgcgtaatcagtg
acatc

>chr1_36840013_36840213
atggagttagagaccggtccccatagccctgaaccagaccggctgctcctgggagcaaagggctt
caccagcagtcacactgagagccaccagagcagaatccagggcagcattgccagccctgccacca
gctctcccctcccaagggcctccggaagcagctgatgtgacaacaaataatctgtcactgtgtgt
gggtg

>chr1_36840213_36840413
tcagaggaggaagggacagacttacaagctctttctctgggccgtactggcttggcctctccatc
cagagaggtagcctgggaaagcagccaagcaccctggcttagggatgtttgcggtggccccttgg
ctcttcagggtgccttctgccatgcgcagtgacggcagactgagtcactggcaggtcttccctgt
catac

>chr1_36853013_36853213
gaagggcttcaatgaggtgggtgaccaaaagacaggcataaagaaggccccagcccagcaagctc
cactctctgacaaaacaaaaagcctagggattccggaagccctgactctgacaaaaatgagttct
gggttttccagagctctaattatcagctagatgaatcactctgcgctgacaccaccattcctacc
cccag

>chr1_36853213_36853413
gagactctcagctgattgaaaatccaggattttaacaaaggagaggaggaggaacagaaagctag
gaggagaagaagtaatgaatagaaaccaaaggtgccagccagaggaaatgagaaactgtcatcag
gacactaccatgaggagaacgctgggacacacacacacacacacacacacactcacactcacgct
gacat

>chr1_36853613_36853813
cttctctttgtcaccaaatcgcaaggcctaaatctggcaaattttgagtgtcagacctacaccct
gtggctaggtaccaccactgtggtgaacaaaggcacctcttatactctgtcatcagtatgatgcc
aagagtgctgggaaacagaatagcacagcagttaagatcattgagttggagtcacacagacctga
gttca

>chr1_37943413_37943613
aagctagctgtagggggcactccaggcctcatgtgctctaccaggaggagggctctgatagcgc
ccactgatcagtttttccatctgctgagggaatattcaggtgctagggctgctgagaaccgactt
gtcccagccaagattttcagtgcccacaggcagtgcactgccctgagagccctggcatcccatta
gttcc

>chr1_38465613_38465813
cctcctgatccagaaaacagtggaatgctagagaggtgctggactaatgaatggtagaagaggcc
agtgtgagaggttgggccttccctcccggggaatcagccaagcaatgggaacttcagaggctcag
cttaggttcaagcaggaaggaaatcctgcaagctggacacagccagtcttccctagctagaccct
ctgcc

>chr1_38466813_38467013
ccagggcatgaggccaccaggggccagggtctttagagctggaactcctgggctgggggctatgg
tgtagagaagatactcctctttctcagccccccttcctgccactcaggcccaggtttcctctggcc

caacatctgaggattgcctgggctgtcagccacggggcagcagggggccacacaggtggaatgcg
tccac

>chr1_45272613_45272813
tatggaagttagccaggtacgctcggagtcttagagactgccatttctcccttccacccgacttc
tctggaaggagaggcaggcctctgcctcagcctggactgtggaggcagagcctccaacccctaac
caggaaggactgacagactcagctccttcaaccccacagatccttccagccctgggggtgttgaa
tccag

>chr1_45273413_45273613
gaggcctaggttagggggagatcttgaggctgcgggggtggtggctcaggaatccagagtctggg
cccacaggaaggaacacgtcatggctctgttgtctccccatcggcctcgacaaggacaaacattt
ccgctcccggcgcagagttagggtggggcttggatgacttcctcagttcagggctatgtgcgggg
gaggg

>chr1_53791412_53791612
gcctgtccacctgtcgccctcctccctcagaccccgtcagtgctccctggctggcagggctggcc
tgtcttggagggcgccccagcgcctggcacgcagcagatgctcccgaatgatgaatgaatgactg
cccgggtctatgtctctaggagagccctgtttttctgttcccggagctacatttcttccaccgtg
acaac

>chr1_90372212_90372412
tggaaaaacctgcctgtgtgttgtgccaagttgtgttagccactccatgttattcttagatgggc
tctggagtatgaccagcctgtgtgcatgcatttgtagtaggcactggataagaactggagtaatg
actacagaagtgaacggtcacagctctaacctgctgagtcatctcagctcagccttgctagcaag
gcctc

>chr1_110546477_110546677
tgtttacttctagttggcagttgcaggcctcgattactcattgactggagcactttaggagataa
tagtgaagtgaagtcagtgaagtgtttagtcttacaggaagagaggggagtagctcacaagggtc
aaactactctttggcagagtttttcaagctgtgtgttccttaccttttaagtgactcagcagttt
ggggc

>chr1_110546877_110547077
ccaagaaactcaggccccatctggggagagtgagaacttaagaaacttgagacagggaaggagag
ccgggagcagccaaaacctgctaagtctcagaagactggtttctgcacaagggaatggaagaagt
gactggaggtggctgtggattcaactctggcctgagattgattcccttgagataagttgttttct
ggtgg

>chr1_145455643_145455843
acaaagggaagaaccaaaagagaaactgcaactcagccacacactggtttctgtaactggagaac
aggatatttagggggacttgctctgcacagacatacctttcaaagtggcatctccagagtctgga
agaggagggagtatggtgaataagtcagctcctcctccctcctcatacctggttttcttgccttt
cccca

>chr1_145456043_145456243
tgaggccttgaatattccttggctgggctcgattgtgaatatgtgtcccaccatgggggtagaag
ggaagggacaaaggacaagcgggctgtagggctgtaaccctccagtgtgtcacaccagacccggc
ttagttaagcatttccttcattcctccagccctctgtcaccaggcctcacccacaacagagagac
agata

>chr1_150539976_150540176
gtttccctctttctgtttctctgctttgcctcatgagggatttctgagaaatgaaaaaaagtcac
ctcccacagccgcagcctgcagttttgtttttggttttgtttttacagatgaagaagagggggg
tggaggaaaggagtataccgcttaagcatctagtattaatccaaaactctgcaaagtaagagaaa
tgctc

>chr1_154943376_154943576
tagggctatccaagcccctctcccctgccctatacaaacaactagtaatctggggagaagtaaag
tgggggtgggagggaaggatggaagagtgggacggaaaggaaggagataggaggtctgggtcact
caaaaccggacacacgcaggccaaagggggcaggaagccaggcaggagtacaggggaatgaggga
ggaaa

>chr1_154943776_154943976
tagcgaagtggggatgggaagtaagtggtcccaatggatgcagagcagggccaagaggaagagca
aagctggtgaaaaaaggggaataaagttcaacctggatgatacatcaggaagctaccaggcaaga
agggaacaagaggacatgctggtcacggcttgttctctacgtatgccatctaccacaccaagacc
gatgg

>chr1_154944176_154944376
tggaagaaacagttctccaagagaagtagaaactacaggccaggaagggacagagagatcagaag
acacatctgcgtcaataaggacaggagaaaaataagtacaggagaaaggggtgtcaggagaagat
gggttgaagctggacacagggaacagtcccaaacccattcctgccacccacggcttcctgccact
gctct

>chr1_156074576_156074776
aaccttagctccaccaagctccaaggggagagaagagagggcatatgggaatgttttgctggacc
ccatactgcactcccaggccaggaagctctgcatcaggaagccagcaccattttcacctcctctg
gggtaggactgaggggaccatggccaagaggaaacagatgccccccttagctcctccctgggtagc
ctgag

>chr1_156074776_156074976
cgggccagggcctgagagcatgccagttttagcctgtcttcctgtccttcccagccagacccctc
tcatcctcctcccccagtggttttctcattacctgtcactgacggagagcccctcagaggtcaag
gccaaagtgaggtgggctcttggcatgctgtgcaagcttggagctcacccggcaaagagggccaa
tgggg

>chr1_156075376_156075576
gctgcaggggctgtggactggagtggattcaccagggagcaaaggaaagtgaagtttcagggcct
ctcagttgcacgggccctttcaagacccgaagaggagccctgccaaactttccacatgatcccat
gtccataacatttgcaaaaagatcattttgtatttcttaaagagggcctccccacaaaattgtgt
gcact

>chr1_156092376_156092576
aaagggcaactcgttttcgatgcctctcccttctggacggtggaaagggctgtgtcatagagtag
gaacgggagatgcggcacaggaatggctcccattgacccgggttgggggctagggcgaaggccta
ggagaggcagaactgttaccttagagctggccaggattagagaacagtgcctggaaccgggggga
ggggc

>chr1_156092976_156093176
taggacacatgagttcctttaggtctggctcaggggagctagacttcatttcaaggggtctaggt
tctgggcagttgagaaggaggctatttggggtcaccaaggctcccctttcttcccaaagctctaa
cactgccaccttctgctggctaggagagagctgtgtcttctgaggctagagctggaatgcagtga
gacca

>chr1_156093576_156093776
tgccctggcctccggcctcaggcttctcctgcctctgtacaatgccacgttgatacgcccagcag
ctgtgactcaggcctggccccctgccaggcccagcacttctactggagttgcgtctgaacatgtc
aacaggcttcctatccctctctcagcaccagttctccccacttcagcccctccctctgcctggaa
ttaaa

>chr1_156096176_156096376
ccgggcctagcctgttgcccccagcaaccgggcccaaacaggcctgtggccggccctggcttcca
tatctggcatcagagttgggctgagcagggtgactcagagggtgggtcagcgcctggcccggtgc
ccacctagcccctttgctgtgctggtgcctttcttccccaaacagccccaagggcccgggcctgc
tgcag

>chr1_156099976_156100176
aggctcctcagagggagggaagcttggccaaagggaagtgagtagagtccagggagaaggctaag
taaggccctgtgtgggaaggggcaggagacaaaggtacccctgtctctttgggaaagaatgggag
gagagagagggaaaagcattcatatcacggggtagagctctgcccttggccccaggcacgttcct
gagcc

>chr1_156717576_156717776
gtgggtgaaagttcagaaataaggagaaatgaggttctttgcagaagcacagactgcaaagtggc
tacctggccaccgtctcgtgccaccccagcttcccatggaaagcacactcagaggcagatagctg

aatggtgtggcaggggacccaggaggccaccagctgcccagccccagcagtgggggagaaacaaa
agaac

>chr1_234859777_234859977
cctaaaacattgcatttaaagaaagtcagaggcgagggggaggctacaggaagtgagtgggagta
gagatgtgtgtgtgagagagagagagagagagagaaatggagggagagggagggaggaagggaggag
ggaggtggagagagagagagaaaaaaaccaccctgctggcaaatgaatgcagcagggatgatgtcat
tggaa

>chr9_35073000_35073200
gtattagtctagtcctattgtggtaacccatacacccatcttctcccaaaacctggagatcttct
aagacgccatcaccaccctgacacttcttgctccttctcaacgctgcctccaaccacccctactc
tgaactttctgactagcaccccactcctaccccattgtcactgcaccctctggtaaccccaatcc
cacag

>chr9_35073200_35073400
cctttgcagtttctcagcgcttgtgcctttcttcctcccgtcattaatattaataccttataata
aggcactcaaatccagagaagtcaaagggcaaccacagaacacacagccaattcagtagaaccca
aattagcaagaaaactgcatatgctgggacttgaaaatccttgggccccaaataacaggggtccc
tgtta

>chr9_91932580_91932780
cttaaatccacttgcactttcctccaagcctttgagtgagaggaaagttcctcctccatctattg
aaatccactctgcattcttccctgtacttttggtcaacctcctatccctgccgcactttatacat
ttctctcaatatttaacgaatttgctctgtgtagaactttttttttctcgtgaaaattatactat
tccat

>chr9_99183179_99183379
taaaatgaggctgaaacctactgggctgcattcccagacggttaaggcattctaagtcacagcat
tagataggaggtcagcacaaaatacaggtcataaacacattgctgataaaacaggttgcagtaaa
ggaaccggccaaaactcaccaaaaccaaaatggccatgagagtgacctgtggtcctcctcaactg
ctaca

>chr9_136008979_136009179
cctccccaccgcaactctggctgccttcaggagctgggcctagtgagggccatacatggcacaaa
catttccagctactgtgcatgaagcttacgacgaccctgaccccaggcttctcccaggacacacg
acctaccagtggtcccacgatgctctgcccagcacgccgggtggcctgggataatgcacctggcc
acaca

>chr8_103801224_103801424
ctttgggtggtgcacaaataaccagttactcagctcatgtccctgaattggcaggacagccttgt
aggccccagggacattcagtgacctcagtctcagagaatgtgggggaattccatgagaggcagga
ctgcacagcccagcctgaagggaaggtcagggcaggctggggagttgacagagccagctgaggtg
tgggg

>chr8_103801424_103801624
tggtgtactgtaccccctgggtgtcagggtggcccccacaagcccagggaagacagccagctgcgt
ggtctgcaggccaggcgcacttgcggatgttgccacactgagcctgcctctcccgcactggggac
tgcccccagaagcctgaattgttttcaaggcttcctactgcagctgctgggccccgcctgtttcc
tcccg

II. 742 DNA Weak Enhancer Sites

>chr2_87930085_87930285
aaacaaacaaaaaagaaggtaatcagtgtggatgaggatgtggaaaagttggaaacttcagacat
tgccgttgagaatggaaaatgatacagctgttttggaaaacagtttaatgtttccttaaaaagtt
gaatagagaattagcatatgacccagcaattccatttttatgtttgtaccccaaagaattgaaag
caagg

>chr2_88301085_88301285
attcaaaataaaataatgagtacacgttttggaataacgtcctattaatgaaaagaatgggatcc
attttgctgggataacattttgcctagttgaggttccaatgtaaaatcttctcagatcgagggca
gtaaaaaagctggatttgatctgcaggtggtagtgtgccggccctccaagccctatctatggggt

ggagg

>chr3_126780310_126780510
acatccttgccagcatttggtgtttttttttttttaattagcatactgttcttgttttatagctg
tagcatttctctcatttctcgaaaactattaattagaatttttggtaaatttccttgtgtttcct
gctgtaatgttggtttctttaatttccttctctatgtttactttgttttctgtctctcaagctgg
ggctt

>chr3_171979306_171979506
tggcttttattttaaatttaatttgctcaaccagacacacagtgtagcagacgctattaccgtat
ttcagtttttggtgctgtagtgagactttgaaattctaggaattgagtagataacacaagtgtga
tgtgggaaaggtccctgggaaacaacagtgattctattaggctggtgcacaaataattgtgggtt
ttgcc

>chr3_176244106_176244306
gcctctttcttggatcaagttcatgatgccagatacttttctcttttgtgataaggtctcttttc
ctcattcctcatgtaaatgggcctttggcaagtctgtccctccggatcaccagatatgtcttaat
cctgcttagactcaaattcatatttaaactgggactcaagccagatgcatttttcaatgtcctta
ttcat

>chr4_114130551_114130751
agatgcaaatctcccccacaggacagctttgcagggctacttctttttgtaggccctctgaacgg
ccatctcaaaatatgtcaaagaagaatatttgttatcgaaccaaactggggcccactcgccctgt
atagcaataccagatatccacaccaaggttttcagcgggagaaaggaaggtgtttattggcaggg
tgcct

>chr5_172834994_172835194
ctttctggttgttagttttccttctaacagacaggaccctcagctgcaggtctgttggaataccc
tgccgtgtgaggtgtcagtgtgcccctgctgggggtgcctcccagttaggctgctcgggggtca
ggggtcagggacccacttgaggaggcagtctgcccgttctcagatctccagctgcgtgctgggag
aacca

>chr7_70005464_70005664
cggggaagcacacagtcgtctctgggtttctggtgaaagtccattgtgcctgctcagggctcct
tggtgcaggagcagtcaagccttcctggaggttgaagatcacgtgctgtgtttggtggctcccca
cttgggtttccatcgattcatggtattggtttgggaggtgctcacatcctcctgcaagtaccctg
tagtg

>chr7_107615764_107615964
caccgacactggccagcaatgagaccagtagaaaaatcagtatagctgtcacaaattccctcatt
ttgagagccagctgggtcacacgtacatcctgagaagaatgcaaagcaccaggttaaaatcagga
ggaaactcaatgtatctttgtataaatacatatattcccttcctccctctaaatctcacaaaagt
cgact

>chr7_152109267_152109467
cttgaggcaagagtttgagaccagtctggccaacatggtgaaacccgatctctactaaaaatcca
aaaattagccgggcatcgtggcaggaacctgtaatcccagctactcaggaggctgaggcaggaga
atggcttgaacccgggaggcggaggttgcagtgagctgagatcctgccactgcactccagcctgg
gcgac

>chr9_132698579_132698779
ttttattccctttcctctaagaatgcactcttttgcccacagacaggatgcctgtaatcattatt
cagtgagcagcaacctgcagcagctcctcctgactggcagatgggcctggcggccacccagaggc
tggggacacagcaagaatccagcacagcaccgatcccgattccctcctccccaaactacctgagc
catgg

>chr12_45048333_45048533
aggatgtcagttcctattggaatgcagctattccacaaccagattcctgacccacagacacagta
aggaaacactcttcaagcagtcagtgtggctgtgtctcaccccaacagaagcacacaggagagat
gaggcttctgatttggggaagaaactctatctcagggcccaaaaccatgacttgtcatgaatgt
gacac

>chr13_35509600_35509800

atgggtttcactgtgctagccaggatggtctcaatctcctgacctcgtgatccgcctgcctcggc
ctccaaaagtgctgggattacaggcgtaagccaccgcgccttgccttagatcactttcatctggg
tgggaacatgaatatattcatgaaaacggcacttccactggcaagaacttcagtaggggagatga
gaagg

>chr16_88291899_88292099
acagaggaagggatttcaagaccacctgctgacatttgaggattttgctcaaggaaggttcctgg
aagggaggggccactcccatgagacagcacagctcagcagagctgaatggctcacccccccaccc
ccccagcttaccccctacattgccgatggccttctctgccagggataggggccctggagccatcg
gctaa

>chr17_18312675_18312875
tatacgaggaggtcatgttaagtattccacaacaaattaaagagatgatgtccatgaagttatct
atgctactctatataacagatggaaaaagcttgggtaatattcagtgcggatactgacttgtgtt
cctgactcataggcggtgctcagacacatttaacccactgtctgacagaataaatgaacgtgtcc
tgtaa

>chr17_71087405_71087605
aaaacacaaaaattacttgggcgtcgtggtgtgtgcctgtagtcccagtgcctgtagtcccagct
actcgggaggctgaggcaggagtaatcgcttgaacccgggaggcggaggttgcagtgagccgaga
tcgcgccactgcactccagtccgggtgacagagcaaggctctgtctcaaaaaaaaataaaaataa
ataaa

>chr22_28076200_28076400
tacaatggaagctccccacagggtaggaactttttccacccgttcactactatctcagagcttag
cccactgcctggcacatagtagatgctcaataagtacttgctcaatgagctgtattcgctggggt
aacattcgatgctctaacaggtaagccctaagttctcagtggcttaacacaatcattttgttctc
tgcct

>chrx_137749134_137749334
aatagtcataataacgagtaaacagcaggtttgaaacatggtatgcatagttattccaaatgtac
atatatatctttatgtaatatatatgtattttatatatgtacacatatatgtgtatacatatatt
atgctatatcatatattaggttatatatgtatattatgtatttatacatatatatataaaatacgta
tatat

>chr1_17662413_17662613
ctgaaggggaacaggtgtggtcagaggccccagctctgctgtccgaactgtagccagacttcctg
ggggccgtggaggagggatgtcttgaacctgtgtctcctctgaaggacgggaagaggggctcaca
ctatgggtgcacacagtcccctggggaaacgacctgcccattcagggccaggctgggtgccccaa
ccccg

>chr1_19679413_19679613
ctggcctaccaccagccgcctacactggggcacgagtgggcacagggcatagcctgatgtgccca
cggaagggacccctgcgaggtgccagcaagccaaagctggcggccttctccagatgggccgtggg
aaacaggaagacaagggtgcgcccggggccacacacagggaagggcagctgccgctgctgccggg
gtgag

>chr1_44018613_44018813
tgtctaggcctttgggactgctgctggtatgtgggggctgggagagagggaggagtctcggttcc
tggccggagccccggggtggatggtggtgccatcactgagatggagagcaggggagggacaact
ctcagggagagctggagctcttcccagcagctctccagcacgcctttttcctggagcttggaatt
gatgt

>chr1_55279212_55279412
aatggatgaaaaggagctgggcagtagggtgtggcaggtgcatggtgcaagagactaaccccgcc
ccgacccgctggttggggaaagactgtctgtttccaccagcccttgtcagggggccttgaggct
ggcccggcctccggcctccgggatgtctgcacagcaggatagtaagtgatgccccattgcttttc
tctgg

>chr1_226661977_226662177
tgcagatacacccagaaacagtgcttcaccagccatccaggcatccctcaatccaggcaagttaa
cacctaaaattaaccatcacagttactaatatgggaaaattggaggactactaataaaactcaga
ttctcagcttcttttgatgattggaagatctgacatcactgtgcccagctagctgaatgataata

gttgt

>chr4_688200_688400
cttaaatatctgctagccataataaataaatgaatgtacttttttttcttagctcccacaagtta
gcctaaatatttgccctggcatacttatactggtcccagcaagcattaggtcacagtctgttcct
cttccttatttgaaggtgtttttacctttctcagcattccacaaattacttcctccttcctttgt
tctcc

>chr4_81383176_81383376
ttaccaatctaaatcctttggccatcctgggattcccaaagggcagtaaccatgctaatttaatc
caaccttggatccccaacacttagtgcagtacaagacaaatgggcattcaaaaccatagtctcaa
atgagaagataaatgcgttttctctcttgtattttcaacatcttccttttcactgacactttccc
cttag

>chr5_115200301_115200501
tcctgctgaagaaaaccgtgtctatatgttgagcatgacttcacaggatttatgacacaaccaat
caaagaaatcatggaagagattgtgaatgtagcaaaaaaggtgggagaatgaagggtttcaagat
aagggtttcagagaaatctaagagcgcatagcacaacagagtaattagcagaacacattttgatg
gagat

>chr5_129492101_129492301
tttgcattttcaaagtgttttgtctactattaattagcatttcttcgcagcatacctgtgagatg
ggtcaatatcaccattttacagatcagaaaagtggaatgacaaaggctgagtgatttgaatgaag
gactataacaagtcaaaggcagccaaggatggatttgtggcaccagcctgggagttcaaacatat
catgc

>chr5_139021216_139021416
ggatcaggggatatgaagggggtgtcccctctttagtatttcaagggaagtagatagttacagcc
ctaccattccccattaggccatttgaaagggaaaaaagttacttccaacaataaacagttccatc
taaccacagccccactgctgcactctcccttgctgtagtctctgggcctctggacacagttgcag
tctct

>chr6_9086201_9086401
attcaagttttattcaaccagttcttaaaaatgagttctaaacaaatagtgattttgcattagc
ctgcaggaggtcatctcttacaatggattcatagtgctttgccggtgatgagaattctcacctcc
actgggttgtgcatcaggaatgacctcgtcactctgctgtatcacagatgcacccccataatggg
agaac

>chr6_143377107_143377307
ttgctggatgtgttgggtttggatacatgcacacgaagagagagcattccaagcttgatgaggag
cactggtaaatgcacttctagtactagagagtgacggagatgttttgcatctgtatgagatcatg
caagactcacgactcatctagaccctaatttttctaacacttttcaatgaccctccatagaatcc
tcggt

>chr7_15821475_15821675
taatagacatgaagccagccatgtcacctcaaatcagtctccttgaaggcatgtagttagcggtt
ttacaaagggcacttgctgctgataggtaggatggagacaaaatcataagttgttctcttgagta
tagttgtttctgggtggggccacggcaatgggagtcagcaagtacagatggagtcactgtgtcca
catac

>chr7_30394075_30394275
ttccttcttaatgtgcagtctcaaatgaataattaaagggaagaaccacttcttatagaatgaat
ttttctgcagggtactcttacagataatgtacacaatcaaatttgttggttggtttttatаggag
gaatgggcatatcaaacaaatacttaactttagaaaatgtccctgcctttctgtttctttcagaa
tttac

>chr8_7441190_7441390
cctgaaattacacgtctactttctttcccaggctggcgctgagatgggcaggtgctgcagcagcc
cggctggaagcgatgcagcatccaggacgacggaggaaggggcagagagggacctccgctttcca
ggctgccttttatactgcctctggtcacctgacatggaacgtaccctaacctaatcagttacctg
tacct

>chr9_33415400_33415600

tctactcccgccctgcacgcccctacccacaaccctggcccctagttgagcctctcactcctttc
acagcagctgaggcaacccagccaagagcctcagtgagatgggtttgggtaaggtggaaccttaa
cccccagactcccaacctcatcctgttttcagcacccatgagactggctctagcaccccctgctg
gcagc

>chr9_132268579_132268779
ttctgcgagatgttaccattggggaaactgagtgaaggcgggtacctgggatccctctgttttct
tttttgttgttgttgttattttgagatgaagtttccctcttgttgcccaggctgggtgatccacc
tgcctcggcctcccaaagtgctgggattacacgcgtgagccactgtgcccagcctctctgtttta
tttct

>chr10_48431194_48431394
agctcactccggtggtagcggtggtgtatctgcagcatctggggttgaccgatggatggggtgaa
agttcaccaataatgttgcaccccccatccccaagctagaaaatgccctaggaaccaagctccc
aggactcagtggcgccacgccctgaaccttgctttccccatctgcctcacagggacaacagcctc
catct

>chr12_1953139_1953339
agagcagggcagccccactgacttagtgagtactgcagcccctaatgaaaccacccgtctgggtg
gtttatgaagctttcacagacatgcaggataaatagaagaatcttagagatggactcttctgatc
tcatatttggaattggggctagaatcccatgctgggtttttaaactccaactttgatggctggct
aggga

>chr14_67665647_67665847
taaacactgagctattacctctaactcttaatacattcgagaagctgctcagctgatttcacaga
ggggtgacaagggaggacaacagacacttcaaaaggcaagaagaggatggcaggagctcttttgc
catggctaccaggccgatctccgagcggaaccaggatgccactgtgtacgtgggggcctgaatg
agaag

>chr15_40530308_40530508
gcggaagactgaagactcacagcctggacttttaaccaaacaccacatggcttcgctacagaaga
aagttagaaagttttcaaaaataaaatgtagagccaagtgccaccttggatataagggactgggg
gacgggaagaggcaagaactcccaggcttccctctggctgtcaatagctagtgggtcctggcagt
gggag

>chr16_19328299_19328499
ccagagtcatcactatttgctttacaatagaaagaatccaccagtcacatgggcctgtcaaagcc
aagacttctggggagcattggtctgcattaataatacaatagatccactagggtgagtctccagc
tgcgctgtatgtgtggatgccagggagagattgggagaagccctgcaggagccctcagagactcc
atatg

>chr16_84592099_84592299
gtttggaaatttagcacaagtgttatcactgttccccgatcactttgtaagctgccttttatacc
tgcctttatgttctggggtttaatagagccatcacagcttgtcttgcctgtgcgtgcacgcagtg
gtgtggcctgtcctccgcagtgacaccggacatgggaactgctggggatgaaccaaggagcaaag
aattc

>chr17_77025805_77026005
gacgaacaaagcccctcactgctaggaaacgggttctctctggctcgcctaattccagcctggat
gaagtgccaccctgctgggctccctaccacctgtgctgttcctggtcctaacacatcccatacca
tagctgccagggactctactgcccaagggaaagggcatcttgttccgttcgtcccaagaatccag
ctccc

>chr19_10196200_10196400
gttgccatctctgtgtccccaggctgggccagagccccaccacctaagggctccctgatgtgccc
tgtactgggttagaattgtccgtcccacggggttcttaaccattgcagattccctggatccctga
gacagccggattctaccgggctactagagaccaagaatctgtatttttctcaagcaccgctcccc
ccaac

>chr22_26966000_26966200
aaagaaagacagaaagaaagaaagaaagaaggaaagaaagaaagaaagaaaagaaagagagaaaa
aaattaattaatcacactttgtcaaatgctgaggaatggtgttgctggctggggtatgctacccg
ccccactccaccccacacacttttttttcccggctgctgcagaggaacacacaagactctttgca

ttcat

>chr2_85683889_85684089
tgaccaggggcagacttagctgtgtaaggctggtccagactcctccagcagtcacatagatcagc
tttaatgtgcccacgaatccctgggcttcttgtttaaatgcacattccagtgggtgagcctggga
taagcgggcgatctgtgtttctaacaagctcccaggtgatactgatgaccacactttgagtaacg
aggat

>chr2_135583330_135583530
aacggcgcaccacgagattatatcccgcacctggcttggagggtcctatgcccacggagtcttgc
tgattgctagcacagcagtctgagatcaaactgcaaggctgcagcgaggctgggggaggggcgcc
cgccattgcccaggcttgcttaggtaaacaaagcagcctggaagctcaaactaggcggagcccac
cacag

>chr3_183544706_183544906
taggaataattctgggtgccttcaggaggtttagtgtggtagtgagggacggaaggaagtgtaat
tgaactgacaaggccaggtcagcatgagagggaggcaggctggggaaagctggactcgccacctg
ttgtgagggagggtggttgggccaggtgggaatgtaggtcacgggggaggagggttcagctgagg
tagca

>chr5_714800_715000
acactgagccatcttgtttcaatcactaatcaagctctgccctgggagcttgctctttgctgtgg
tttggacatctgacccttccaaaccttatgttgaaatttgatctccattgttggagatggggcct
cataggacgtgtttgtgtcatggggctggacccccctatgaacagattaatgccctccctctgaag
ggagt

>chr5_128734901_128735101
cctccacaaagagctgtcacatgactttggcacctcccaattcttcccctgtcactcagctttt
agacagaagctggctttttctgtagtcctttcttcacctagcgcatgcctgaagcctggaagct
ttgctgcaggcttaaattcttaagtatgtaagaggttatagccattcgaggaaaaataccaaata
ggcaa

>chr6_15208221_15208421
gaggatcactagagccagagagattgaggctgcagtgagctatgacctcaccactgcactccagc
ctgggctacagaacaagacccagtctcaaaaaaaaaaaaaaaaagagaaaagaaaaaaaagagga
ctgagtcagccttttcacttattatccctctggccctcattcctccttttggtttgagaactaag
gtttt

>chr6_73839479_73839679
cttagtaatgtgctgctctatttccctcactcctagtaagttctgtagtaataagcagaagctct
tcagaatgtacacctcacggaagcagaggtacccagcatccgggctattgacatgagatttgaga
atgccaactaacctcaggtgcatgaccaggttacaccgccaccccacctgagccctgttcagaga
ctcac

>chr6_133289107_133289307
agtagtgacacatggaaagataaagctatttattctttttagaataatggttagcaaactacagc
ctggccaaaatccagccattcccctggtttcataaataaacttttattggagcacagtcatgttc
atttgaaaacatagtgtttatgactttcatgattcaatggcagaggtgagtatttgaggcagaga
ccgtt

>chr6_142469707_142469907
cgctgtgactacatttgctgcatttttggagcttacatacaatggaggtagagagtttcaacaat
atagagtctatcatacatagagtattaaaagcttatgaagacaaatagagcaaggaaaaggaata
agagtatcaggagtagtggtggcagggagtgcagatgggatttcagtttcatagtggtcaggaac
acttc

>chr7_139654931_139655131
ttagtacctggctgtgttctggttgaccacagctgcctgaggctcgaaggtggaagaggctgtgc
cctcctctcctcccaggtctttgtttagacgccaggtcttcgtgtctcctcccaccctctgccgt
cactgctcagagtgccccatccaggagcccatctccctccacctccccccagatctgcagaagct
cctct

>chr10_83660820_83661020

catggtgaagtcgggagctccagatagaaatccaaagagagagaaagacaggggcagggaaggta
cagtgtggaataatgtggactgtctcacatgacacttaagaggtcatgtgatgctagtgactttg
ctgatgtgtcaaggctagggctgagaggctgtgtccagcactgtcatctccactacctgaggcag
aagca

>chr12_117556417_117556617
ctgggcgtcaagagtgaaacttcatctaaaaaaaaaaaacaaagagttctagagttctaaaactg
tgatgtgcataacattcccctggagcctctggtgtcgcaggaagggttggggactctggggtcag
tacctgttagaaacccccaggtgatggtgaggttggggtgagcaggatggcaaaatactctgccc
cccac

>chr12_131912647_131912847
gcataacaaggcacatcttctggaggtgtaacacggcatagccacagcggctctatgggcagaga
accctcagaccctgcagcaaaggacactgtgctctgcctggattccctcagcacctgggtgagct
acccacccttggtctcccctccagtgggcctcacaccagcgcccatccctgtctaggcacagacc
atgtg

>chr13_108215199_108215399
aaaaaattaccttccaggcatttcccagttcccatgaggagtttcctctgtgttccacttcctaa
ctgccaaagttgacttttgaaatgttggagctgagtccttcttaaaacttacatgagtaggcttt
ttgaattcagaggttggaaggcttcttcagatgaacaagttaatgtaagtaaaataggtttaatg
agttt

>chr14_78121247_78121447
gctggctctgagccatcctggagatataggctggcttaaacctggaagggcctcctggctggccc
tgggataacataaagcaggctaagggacttaaaggcatgtgggaacacagggagcttgggaggtt
ttgctctttgcttttgaggtttctaagccaggaacaacaggatcagatctgtgttctggaaagca
ggttc

>chr16_52344499_52344699
ctatcactgcaggacagtgcccctaaacttagtggcttaaaacaacaaacacgtattttctcaga
gtttcttaggcactaggatctgggcacaacttaattggatgtctttgtctcagggcctttcacag
gactgcatcaaagtgtggcaggctgggactgcagtcatcttcaggttggatgggacaagatggac
ttcca

>chr17_35969487_35969687
caggccccacctcgccgcggaccgggcgatggggcggggcctcgcgcccacgtgactcccccggc
cctcggggaggcgtggcgcgcccacggcacgtgacccggcgccgcaggggtcccccagattcaat
tctagctctcgggctggtggggcggtcgcgggcgcctctccaagggagctgattcattgtctggt
aggcc

>chr17_55859601_55859801
tttggaaagaggaaacatccggctcctcctccctcacatctctgcttctgggtctgggcttgtgg
gtcccagcttgggctaaggagggccatgctccgggtgggagcagctgtgtccccacccccctcctt
cccatgcgacttgaggccctctggtcaggactggagctgtcacatgcagccagcagctgtggtgg
cgcca

>chr19_709000_709200
gcccccgcgccgcgcagccccggccgccaggccttagcccgccccggcccccgccaggccgcgtc
cccctcccctcccctcccccgcgcgccacccgcgcccgcccccgcccggcaccgcggacccaccc
ggacctcggcggggagatggagtgagtaggcgcgctcgggccgcggggctgggggcccggagctc
cggga

>chr20_12576800_12577000
actcaagctgatttagtaaataggaaatatataggcttgcaggagctaaaaattcaggctgccag
tctgtctttagtcactatgaactccatcagctcaaatgagatcccgagggtccagcttttctctc
tttctcttgcagctcatttcccctgtgttgatatttctctcatggccacaaatggattctgatgc
tccag

>chr21_33286729_33286929
aaaaaaaaaaaaaaaaaaaagacccggccctagcccgcagtgaatgaatgctagccctttgttct
tacattttactcttctgcagggaatcccgtagaattgtaggatgctcagatggtctattagcct
gctcaggctgccacaacaaaataccatggagtgggtggcttaaaacaacagaaattcatgtttca

cagtc

>chr21_38164930_38165130
atgaggcacctgctcagagaactgaagttactcatccagggtcacatggctgtcaagaggcatgc
ccagtacctatgacaaacattcatccccaagtaacgaagccaacaacctgtgtctcactccagaa
ccacagagctgttacaacacgtggtgcctcctgagccagcagccaggggcaagaacgagaggatg
caggg

>chr22_49079288_49079488
gaggccaagaggctctcttggccaaagtgagtttacctgaagggacgggctgaggccagagccag
gtcgttgggccacagaccgtgcactcgctggttcctccacggctcaggaacagcctcccactgcc
aggacagcctaggaggctgggccagcaccttcattcagttcaacgctttctatcacagaactgtt
ttgaa

>chrx_78621744_78621944
tagtttaaattacaacactggacctgcttcagatctctaaatagagggaaggggatgttgttcaa
caagaaaaaaatttaaaaataattgaaaagtatcctcaaatttattttacaagagaagagtgtt
ttctttaggaaataccactctaaaaatatacaaggctcttgcagtaaaagggcacagtgaacacc
caaca

>chr1_59168412_59168612
tatttaaaattccctctaatcctgcaaccctaggacttgttgaaagactcatttctcaccaagta
gtttgaaagcagaatgaataattcagaattagtgtttcatgaaacttgtagcattcctactgtgc
ttggtactgatgcattgaatgagattgactcagtgtctgccctcataaaatcaatcaaatgtgca
atttc

>chr1_180209177_180209377
aaacaaaaagaacacttcatccagaaggcagttaaaaagcaaagggagttaaaaagcaaaagcct
cacaggaggcacatcaagctggcacatcaaagtacaaactttacaaaatattaatctggtctcag
agcatcgttaaataaacattattccctttttaaaattattataatagaggtaggggctcactatgt
tgccc

>chr2_84540689_84540889
gtctcaccgggctgtccttgatagaggtagcctgtttgtcatgttaatgaagagaaaatttccag
ttctgagttccacaaagactgttctcccccacctacagctaaagagggagaaaaatgtcagcagg
taaggggtagaattggagtgaaccacttccttacctttgggagcagctggcctaatgtcaatgtc
aaggg

>chr3_57193160_57193360
ttttttaaagacacatttacatttatgcactcaggacttcaagtgtgaattctgttcaacagact
aaattctgagcaagataatgggccatctcaaagattccccccttggatttgtttcttcaagactg
gccctagctttaatctgcagtaagactatctgtttcaataggatgtgatcacttgagggcttgtg
tcttt

>chr4_3305802_3306002
gctccatgaggccggaattgaggcatttaatgtgtgtgtccctgatgctcagcctcttggtttct
ttggatttgaaatgttaaaacctcaagaagtgacatttattaaaaacccacgaaaaactatgcat
gatgacaaaatgctgacctgggacattttatgtattgtacttttcagttcagcctaatgtattat
tcaaa

>chr6_42151622_42151822
agtccctttaagtacagctgaccctttcaaaggcagaaagaccacagcctgcagcgtatttgctg
ccatttttcctgttgacaccactggtgaggatgccttggggaggaggaggccagggtcctcccca
tgaacgcccctcagcatcccggaagttatagaattattgctgccccctgccccccaaaactcctg
ccttt

>chr7_6413875_6414075
gcgccgagctgacaggttcctcttaaagccctgagtcccagggaagggacctgcaggcccggcca
cgcccaggccgctttagcgcgccaagatggcggctgccacgccccgcggccggagccgagtgggc
ccgagcgcttccgagcattcccgaagtccagagaaactccgggagcggcgcgggcgggagcggcg
cgggc

>chr7_21579075_21579275

gtttgggtggacatgagtcccattaaaacttggaacaggggtaaatgtcgtatttccaagagaag
gaaaaggtgaatggacactggggacagttagcagtttctgctacatcatcttcttttaaaaaaat
ctagctattatcacagccctatgtgatttttgtctcatatttcttctttgtcctcatctcttgct
attct

>chr9_99835179_99835379
gagatgtgagctggggtctgcagggtgcgtgtgtgtgagcgatgtgagctggggtctgcacagtg
tgtgtatgtgagctatggaaactggagtctgcagggtgtgtgtatatgaacaatggaagctgggg
tctgcagggtgtgtttatgtgagtgatgaaagtgccaccctgatgtgtgtttctgctctagaata
gcttc

>chr9_102485179_102485379
gaggcaggggggaagtagcatgtcagaagaaactgtgaaaacagtgggataaatcgggaatattac
aaaaagaatagacccaccctactatgggactgtggttcagagctagctctccaagtgtgaaacca
caaaaggcagctatcagagagtgattcttcacatgcaccccctctgtgcacagatgtggaggggc
agtga

>chr10_47694794_47694994
agttcattccctaaaacaagctcggatgaagcttccaacttggctgctgctacatctgcttttaa
atgattagaatattccctaactactgggtctaattgcagactggaatatgcagtgggcctatgtt
tcccccatgttcttgagtaggaacttctaatacctgattgttgtgttcatgaacaaccaaggtaa
aaggt

>chr11_12482824_12483024
ttgtatctacatttcctatgtaattagccagcttaccaggtcaagttctatgttctccccaaggc
cgttacaaatgctcagggcatgtggacacacacagatgcacacccacgcacaaaggcatggcact
tcttctctaagtaggagcagacttttaaaccaaggaaatggttctcattctcaatctaaaaagat
aacag

>chr11_122394790_122394990
tatagaaacaccatgtcacaaagcttttcagacaaagacgtagtgggggtgagttcattgggtgc
agaatagaatattttgatgggttgcatccaacccatttcccaccccccaactcctacatgctctg
ataaaagtcagatctgtaaagaacttcaaaggctccccagtctgacaaacagtaataaaacaaga
aatcg

>chr12_97855669_97855869
cttctggggaaggagagttccatctcttattttaactaaatgacaagctactttttgtttttcta
ttccttgtcttctagtattgctaaacaatcaaatgcaaggtattttgtcttctttcactccttaa
aagaaaatcaatcaattaatacacaaagataaaagtgagaaacctactgtaatctactaaggtcc
aggaa

>chr15_41058908_41059108
caggagctactggggctcagccttgttgctacagaactgcaggacagccagggcagtgaggggcc
tggaggcttattttttttttgaaatggagtctctgtcgcccaggctggagtgcggtggtgcaatca
tggctcactgcagcctcagcctcctcctacctggaagcctgggtggggagcaagcccagggaggg
cggca

>chr17_41996474_41996674
gacacctctccaggcctctgacttagctagatctccaccatgtgactccaccatagactccatgc
cttcttcttttgcaaaccctcggacacccaaacacctaccaatttcagccctttctgtgcaggtg
ccggagcccaggaagcacacatcaaggctcgcttgccagcagggtgctgccaataaaatgtagtc
acatg

>chr2_85683689_85683889
ctcagctgcatcatcagtcctggagcacctgacgggtgctggagcacacagcataatccccacaa
acaacccttgacttgacttccctttccactctcctctcaccgcccagtccccagctctgtcagc
ctcactccccgctgatggccaagcttgtctcagagttgcaggtaaccttggcatttgtgccaaac
gttgc

>chr2_184375955_184376155
gatgcctgtgtgtttcccatctgaaagacaaaactgcctgtggttttggtttgtttccccccctgc
ccaaggacctgcaacagtccctggaccctgctgataggaatggttgtgctcactgatgcagcagc
agaaatacctctcactcaagaaccctcaatggtccctggaccctgctgattgaagtagttgcact

cacca

>chr3_78946510_78946710
acacatcaaaagcaatttccaggcaggtagcatgtttagaagtcaggacttggaatcgctcttat
atatccctacatatcccaagtagttagcatctttgtcattcggtagatatgttcatcagttgagt
ttacagaactttaaaaaacagttaattacaatcttaaattaatgagataccttttgaaaggactt
gtgtg

>chr5_126664501_126664701
tcacacttcgttgaagggaaatacttctcacatagataaagcaatgagacaaggaaataattgcc
atgctcatttgttacagccaaatatattaaagatttttttgatggatgaggaaaacactagctgt
atattacacggcttcaactgtaaaacagggacaataaaaactactacttcttagttaagttgctg
taagg

>chr5_141122816_141123016
ttctccctggtagcagcatggcagattctgaagtcacaaaaactcctcgctcatcctgagagtag
agaactctacccaagttactgatctacaggccaagccacgtcgatcttctgtccccactgtcagt
gcttatgcaccctgggcagaggcctacactgccttttcggtaaaggcagctggcccctagccctc
cggat

>chr6_24693421_24693621
aaattgtcatccattaagattgtctgtttttctttgtgcttctgtagacagtcttcttttggggg
tggctcacgtgtttatgtacacagattttctcataccatatgtcacactcctgtgcttgaggccc
attacagacacccttactgagccagagacatacattcctttatctgtttagcagaggcagtggag
gcagt

>chr7_100241064_100241264
ctctgaagcccctccccacctcatcctcccctcctcggtctgtcccctttcatattcagagggag
agtatcctcttcatatcattcaatcaataattcattcattcaacaagtattattgaacagcagtc
tctgccctgagctaggccctgttcctgagctgggggggatggcacgcacagccccaggccaggcaa
cctgc

>chr10_14372194_14372394
cagccaaaatccagacttagggagcaaaaggctcaaggggaagataggccacctttcacacaggg
tggggaggaggtcagggaagacagggtgggaactgtgcttagggaggaaaaaaaaaaaaagaaga
aggaaaataggaagctgcaggttatttaaaagtgagccctccctagcttcgcaaagcggcacaga
caggg

>chr11_6496824_6497024
cttcattatatgctagcctctctgcctgggatgctcttccctttaccctgcctgtttgtcatcta
ctcacccttcaggtctcagctttttttttttttttttgagatggagtcccgctctgttgcccag
gctggagtgcagtggcgcaatcttggctccactgcaagctccgcctcccgggttcacaccattct
cctgc

>chr11_113731790_113731990
agatcgcaccactgcattccactccagcctgggcgacaaagcgagactacacctcaaaaaaaaaa
aaaaaaccacctctgtctggacaacagagatggttgctaacctttccaacctccataaaacaagg
aatggccctcaaaagccttggtatttttttcagtttttcttccagcaacaatgccctgctgatcca
attta

>chr13_40153000_40153200
tcatggataagtcaacattcaaaccaggccttaaaaggcaaataggtttggggtctgtgaagaga
ggagagaaaggtgaagggaacagcgttcagcaaaggcacaacaatgttattctaatcttatgtaa
cctctcgtgtaactagtgcacaagaaacatcagaagattcagtgtggctaaagtagaataaacaa
gaccg

>chr14_74881447_74881647
atgctcgtgttgactctagattcctggtaatagaataacaacaggatagagggcctcctttttca
gctggggcagactaagtcatgtcacgccaaaccgccaataacagctagaaaagtggtgtgtggtg
ggtgggtaatatgaaggcttcaaggaatcaccaagggagtaagaactggagggtcagaatgccaa
gatcc

>chr15_50350308_50350508

tcctggggcatccctaatgatctgtttgcctcctatctctgctggctagagctcatctatgcctc
ctccttttaaatcaactggggatctagggagtcagccctcagctttggcccacgaattcgtctta
tcctgagctttagggatcttgtatgatcagagtgggcatttctttgagttgaggtgggggctggc
ttgtt

>chr17_32297287_32297487
gaaccagtggggtagttggatacctttcctcattctcgaagcccaggcagcaccccacttaaact
tctggagctgtggatgcatcttgaccaggccacgctctccttggtctctgcctagtgggatgctg
cacttggaacacatcccagagacaggcagtgcccacacccagctgtgaaacacactgctcccctg
ctgag

>chr17_77834205_77834405
gataccatgctctggcagctgagggtaattacgggggctgcgtccttgaggacgggtctgcgcat
gtgcttctcgcggggctggtggcccagccggggcggcctcggtgccgtcagctgctcggtgagat
gtgaagggccggggctccgcgtggggccttccgtgctgcagcagaggcctgggattggaatgcga
aatcc

>chr18_10087200_10087400
gggacacagaggctgttttcacaagacttacagggtggcatgggaatgcagctttgtaaacaggt
gatttcagcacccaccgtgagtctcaatagagaccatgaatctgcacagggcaaggaagcgctca
ctcagcatacgtatgttaggggcaagttatagaatggtttttagagttacaatccttatttaatg
gtgaa

>chr18_57246820_57247020
atttatgacaacggttctacatcagacaacacacaattccagagggccctcggcggttcctacat
gctctatggtggcagccacctgcctggagcatgccaacagaacggaatgttaggctcttactgcg
ttagtgtctctgtccctgagatctgtcctggactgtccctgaacagcagcattcaattccttgtc
tcagg

>chrx_21858879_21859079
aaatgagacttttccttctatcatttaaacttgtaaagtgcaggaggttacatagcgtagtcctt
agatccagggctgggcttctagccaatgtgaccttgggaaaattgctatctgtgccttggtttct
catctttaaatagagataatagtaaggatctcctaagattacagagagaattacattagtgtatc
cacat

>chr9_115610379_115610579
aaagtctcactgtgcccttcgccttttttgatgtgtcatatcaatgggatgcagatccctataaag
ttctgtgagaagttcagatgatgactctcttctgcttgtcactcaatgatggaggggcctctctg
cagacgccggcttggtctcttggcctctttaccccactgccggctcgggcctcatctgacagcca
taggc

>chr15_35312708_35312908
agttgcttcagtcacaatcaatggtcatttaactgcgtctaaggaggtcaggtcttaggtgcagg
ccttttgtttggctgatttgctttgcagatttgggtccaaggcatgcaaatgtccctgtttagga
tcacaaaggagtcactggggagccaccagcattctctccactacatttcacatcttctccagact
taata

>chr9_129109179_129109379
gaacttggtggcaagctcagaagcaaggttagagtggacctgcaaggtaagtgggaggtgacaac
gagaggttgagagtgcagatggcctttgaagaggggaggccagggcatagcgggagctagaggat
gccatgaccccagctagaggatgccatgactccctttgttaaaggtaggaaagtgtcgaatgtgt
ctagt

>chr19_7445600_7445800
acactgattttgtttagccaagacacattactgccctctgctggaaaacctgtcataatgtctca
tatacaagcccagagtgtcaaggacaggtgggctcttagacggggtacctggtgtatgcctgtac
tcggtagcactggggtaggtgagtgggagaagaggctgccgacccaggtatctgtcccctctgat
cctgc

>chr12_117539417_117539617
gtctgatgtgggggataggaagtggcattctctggacatcagcccctcctgcttggcacagttgc
tctggtggggacaaaagccctgggacaaggagaagggcacatggcttcacattgtcccttggtga
tctcaaggaagcccctggtatctagtatgaagggagtctgtcctacccgatcacctgtctaccct

gaccc

>chr4_76994976_76995176
ttttactcaaaattttgtttgtgagatttatccacattgttatatatggtcagagtttgttcgtt
ttcattgctatataaaatttcacttacagtctaaccattctactgttgatggatatttggtttcc
agtttggggggttattatgaatagtgctactatcaacattctattaatgtattttggtgtatgtat
gcgtg

>chr17_14227075_14227275
atttagcaaacccaggaaaacattgtctttggagaggtttgagcttgattaatctccaaaacgtg
ctaatggggcaatagagagctcagctcttgttctctccttcaggtggtgtttgcctgtgagctta
gtgccctatgttcccagcccagggaattggaaattgattcatttgggaaagtaacagcttaaagg
tcttt

>chr19_38984760_38984960
atagccccacagcaccctagcctgggcaacagagcaagacactgtctctaaaagaaaaaaaggaa
aacaatctgctagaatctgcctgctcccagcaggtggagggcgcaggtggtagtaactgggaaaa
cttctggaacaggggggccccttccacattgttctggtccaaggccccatgtgccgacctgccctg
catgg

>chr6_79290681_79290881
cagctattaaacaatgttctccctccaacaaacctagtcctctgcactgtgctgtgtgatgctgg
ggccatggcttaccaaattccactgatcctttgacaaggaggcttcctgttaggttctgccacca
taaaggtactagagaggggactgcaaggctggaggaggaactgaaatactccttttttgctttgct
tcctg

>chr14_50275850_50276050
ctaaaaatactcttaccatgtgattcaacaatattgatgtttggtatttaccaaaatgaactgaa
aacttatatctgtacaaaaacctgcacagatgtttatagtagctttattcatattgccaaaacat
gggggcaaccaaattcagtagattaatggataaactgtggttcatccagacaactgaatattatt
caggg

>chr1_197780977_197781177
catgcttattcaatttgtttcttctttctctcagactggttaaaggccaaaacttgcccttccta
tttgtgtcctcatgttctatgtatttatttgaatcatccaaagaatgttaagtatcatgctctcc
tcctatcctgtactttcttacctctgggccttcttgggtaacaattaactcatactgaattgtac
tatat

>chr2_61808696_61808896
attgcaccaacttaaatcccaccagcagtgtataacatatccttaccagcatgtgttgttatatg
atttttttcctttttgactatctgaaggttataaagtggtgtttccttttggtttaaattttca
gttctctgattactaataaagttgtgtgcatttttttcatatgtttattgaccattcaagtccctt
ctatg

>chr20_25768600_25768800
acgttgcaaatgtttgttctaaatcccacactctgaagtcagaaagacctgggttagccctcagc
cactgacggttgcttgctatgtgaccttggacaagtcactgcccttctctgagcctcaacaccca
catttacacaggggaagaataatacctccctcttgggctgctgtgaagcagagctaatgcgtaca
ttgcc

>chr21_28642129_28642329
ttttcagacttgtctccttccctccctctcccttgagtcactagtcctggagcttcttgaggttg
ctgaatcaccacttatctgcaggcctacttgacctcagcatccttattgacagtattcctctgct
tgaggatcctttcttttccatgtcatgtacccatgcatattcatctttcaatatctgcatctaat
aggtt

>chr22_50111996_50112196
ttgaaccagaagcccagccctgcacctcccctccacttccctttcttctcctttttcttcctttc
tccttcctggcaaactgcagaaataccttgctatgagctaggcctggactcctatctgtgcagcc
actcacagtggtcagttgcatccgtcagccactgctgcataacaagcaacccacggtaagtggcc
tgaag

>chr10_35814794_35814994

cttctgtttaaatatttatgttcagactgtggccaggtaatgtgtaaggtcttgaatatgctcat
ggacactgaacctctgcattctatttttacagtgtcgctcttgcaatatattatcacatcaaaaa
caggtatgtggatggttgtgtgctaaaaacatcatacgttatcttctgttatgttgttcctattt
tttca

>chr2_78193492_78193692
cctccttcactatgggtaagcttccaccttccattcctccttcttctcccttagcctgtgttctc
aaaaacttaaaacctcttcaactcacacctgacctaaaacttaaatgacttattttcttctgcaa
tgctgcttgaccccaatacaaactcgacagtagttccaaatagccaggaaatggcactttcaatt
tttcc

>chr3_62905560_62905760
taggacagaagttctcaaaatgtggtgccccaaccagggttatcagcatcacctgggaaccgaat
agagatgcaaattcttgagccacatcccaggcctacttttagaaactctggaggcgggctcacct
tagtcctacaagcactcctggtgatttcaatgcactcatctttgagaaccactgttctggggtgt
tctga

>chr11_13025224_13025424
tcaaagcccatatcaattcagagagccacatttcaagtattcaacagccccatatgcctagtggc
tactgtattggacagagcagccctaaggattaagactgcaaaagatctatgatgtgctgaaaggc
atcttctctatttatattaaagtgattaagcaaccacagtctgaatttttaagtacctccttcac
cctcc

>chr18_45502202_45502402
gcctgacctcaggcaattgcagagacgttaccaggcaggccccaagggaaaagctgctctggcac
accagagttgcagtacagcctggaggaagttgcagtcaaggactcagccccgagggttaagtagc
acccgcattgttactcttctcttagatttcagagatgttttccctgccctgcttcctatcttatc
ttgat

>chr19_29481560_29481760
ggccagacagtgtcaatagcaccagagcactctaacacctggcaagcttcaccctgagccagggc
ccccaccagccagcgggagaatctgtcatcgtgacaagccaacctgccttccgcctgtgctgctt
cactggcctgatggttagggaaggaaatttgatctctccttcctgcccagttcctcttaactgca
ggccc

>chr4_188952406_188952606
tagggaaagggaactcacaatgagtcatagacacaaatgttaaaataaaactagtaaacatctag
aaaaaaaaaacgaaagaaaaggctgggtgcggtggctcatgcctgtaataccaacattttgggag
gccaaggtgggcagatcacttgaggtcaggagtgcgagaccagcctggccaacatggtgaaaccc
catct

>chr13_84505399_84505599
atatgctaaacaaggggtgtattattcatgcctccccattttagacatatagggtaacttcctga
cattgccatggcatttgtaaagtgtcatggcactggtgagagtgtagcagtgaggacggccagag
gttattctcatcgccattttggttttggtgggatttagccaacttctttactgcaacctgtttta
tcagc

>chr6_32983222_32983422
cctttggctgggaactgctcacttcccttagaactttcccctcccgtctcctgacttctctaaat
gccagagttccaacctctgtctcctgggaaattctaagctaaaatcactcttcctttatatctgc
agatagtttgaaatttatacatcaaaaaaagtaactttaaaaatatataactggtctcattacac
tctgg

>chr2_51000696_51000896
aaacattggtgcagtgcactagctaagttcagtgaactcccaagctaatcctgtgttctcagaag
ctataagcgccaatctcttcacctttgctcaggggatgagctcaccacagaaactggcctgttt
tttgacctcaaaatccagttgggacagggttgggggatggcagaagcaatgaaagggaaggtttc
caggc

>chr2_212528755_212528955
tgaaccaatgaattggcaattaccacttccaaggtgaatttgaatttgaatggcagaatattact
ttacctttgaggatctgtcacttcacagagtgagttaagcttatttcttgacctgcaggaaatt
gtagcaacatctgcaaattgatgttttcttggcaggaaatgatgtcgcttgcttaatttcttcag

tatcc

>chr8_22638855_22639055
cggggctgggagtggggaggagggacacgggggtgggctccaggcttttccccacacttcctcag
ggatcgctttacataaccagggctgcttagtcaccgcttttttacttttttttttggaaacaatttt
ctcagcatcacagctcccgtctccatcccttactagtcaccggtgctagggaagtccctttttctc
ccgtt

>chr1_71054612_71054812
attttaaatcaggtaagcggcctctgtttactctcttctccaacctccctcactatccctcaacc
tctttctcctttcaatcttggtgccacacttcaatctctcccttctcttaatttcaattcctttc
attttctggtagagacagaggagacaggttttatccgtggacccaaaactccggcgccggtcacg
gacta

>chr10_1463400_1463600
ttacagtcaaagcacagcctgtgcacctgcccgtcggttccatagtgtttgtctgctgcttggtt
tgtgggacaggtggctggtcaggatctgcaggacctaaccccacccacgctcagctgtgaggaga
ttaaagggtctcagtgtctcagggtcaggttccaggctggcttcttgcgatttcacagaagtcac
taaat

>chr19_24221760_24221960
tctcttgtccaaatgcctatgcatgtggcaagggtgcctttggcttgcttttctgccctcctaaa
cctaaatctgcagttccaaattttgagtccagctaatagggttctccattctgtgttggagcagt
cttcatgtggggatttctcctctacttttcctctcctcactgtaacacaatatggagccaagtta
atggt

>chr11_22360424_22360624
gtttttgtcctttactacacaggatttatcttctttacagttaagaaatggccagcccctgctca
aacagcgtttcttttggtgcttatagataccccgaattaaattcctttgatgcagacaaatgaat
ataaaacacaaccatctaaaaaaataaccaagaaattccaaagagtaatggtttatatatatata
tatgt

>chr1_160780576_160780776
gacttgagatatgattcaccctgaggcaaaattcttcttcagttgtgaacctgcaaaaccagaga
agttatctgcctccaaaattcaatggtggaacaggtatggaatagacattgccattccaaaagag
agacattgggaagaagaaaaaagtcacaggtcccaaacaagtccaaaacctaacagggtaaattc
catta

>chr20_61075205_61075405
aacggctgtgtgtgtgtgatgggccggagcccgggggagtcaggagctcagactcaagtgaagct
cagagtgaacaggaagggtcacagccgcaccgtcccccaacccccaggcggtcagaatccagagc
gtgggcccagggctgcccctgcccgtcggtccccggagaaaacgcccggtgtgtgcggagcttca
ccacc

>chr12_67966533_67966733
ctggatctctgccactctgacttggccactccatttagaggcctcttgttgcagggctttggtca
ggagctcttttataggcctgcccatgcaaagagtatagttatcatgtgaagaaagttttctctga
cccctgagggtacagaaaagaaaaggcttgtgtgagtactgagtgccaatgaacctgttaaaaag
atgtt

>chr2_35641096_35641296
aattgtcttatgagttgtatggagttctatattttgtacatgctagctcgttgagttgcagctaa
atgtgctgcctgtgaggcactgtgctggcagtggtgaggaaataaatcattgctcagtgggaatg
atgaaggtacctgtgtcacaaggaataagattcatcccttttatgaatgctttataagttgtaaa
gttct

>chr4_123617950_123618150
ggcaagaggcttatttagcttttaaaaagatttacatacatctcaatgggggcaaagaaagaatt
cacaattacaagtgttcttaagtaatagaaagcagaaggatgattaccagaggctgagaagtata
gtgggggaagggggttagttaatgagtattaaaaaaaaatgaataagacctagtatttgataac
acaac

>chr14_55141050_55141250

tttgcatgaggttctctaaaaaagtaagactgtaaaagcagcttgttcaaaacttgacctcttcg
gcatgcccaagaatttgtgaaacaaaagagaaggcaatggataattgattctgttctctaaaggc
ccttctgacactggtcaggtactggtgtggtttctactggtgtagaaactggtgtagatactggt
gtaga

>chr11_117009990_117010190
gctggaattacttgcgtgagccactgcgcccgggcgcctcttttttttgaggtgaagtttcgct
cttcttgcttaggctggagagcaatggtggatctcggctcactgcaacttttgccttccaggttc
aaacgattctcctgcctcaacctccgaagtagctgggattacaggcatgtgccaccacgcccagc
taatg

>chr17_48154001_48154201
atgcgggcagccttcgtgtcagagcagcagcagaagctgagcaggtggctgtgggccgccggccg
cgttaatcggccaagggtgggacggggcctcattaactggcagggtgggggcggggcctcatggc
aaggcgagcccagggacagggctttagcgggaacaggtgggatggtcagaaacggggctttctcc
tccag

>chr5_129061501_129061701
tcttggctgaggggaggggtccattcaattagttgggggacttagaattttattgttggtttaca
tggtgaaattatctcatgtgtacccaagtacagagaagattgtactttccttaggtagatttaat
tgatgaatatcactacggcaatggaacaaattttgctttcagagaccttggggcttattttttgg
tgggg

>chr2_47905696_47905896
cccagtcctgagaaaacaagaaattcaaaatctaagctgttggaagaataaattattgtgagctt
taaagaatgattaaaagcctgagtcccatgacaggcagctataacctaggcagttgcaaacttta
ttttctctgattatggattaagccttcttccttgcctacattgttttgtaaaatgctgtaaatgg
ctgaa

>chr5_78984844_78985044
tttgagcagtgggaagccattgaggggttttaaccacaacttgatccggggaaaggtttggggca
tttgggccagtttcagacaaaacttagctttttgctaacgtgggggaaattgctcctcctcaagg
tttaagatgagtggagataaagtttcctggctcaataacagggaccactgagcaagaaaatacac
agtat

>chr14_82863247_82863447
taatccccagacagttccaagtttttttggttcattatcacatttcatttggagtctcctcccata
aggcattctcacccaacactagctgcacagtttttctcaaaatatctttcacaagaggcttcaga
gctctgataacttcacacaggagacagttgggtgttgcagttaaaaacggtgcccatggacttca
agggg

>chr1_242375177_242375377
gcttaaagctagtggagcctatgtttggcaggctcaattaagggacctaatgcaaactgttgtat
cccaaaacccatggttccctgaagaaggcatgctagacgtaaagctctggaacaagtggggagaa
atcttaaacattgcatgcaagggcaaggtcccagtatcatctttaatgctatgggccttagttag
ggcag

>chr1_228107977_228108177
aactacacacatctatctcgatttagtggtttcaaacaactaagcagatcagaggcagggttagg
agggaatatgtcagggacggaaggcggggccctcccagactggggcttggccgagagcggggagc
tgcagccggggaggtggcagggatggagaaaataataattattatattagttaaataatcataat
aattt

>chr8_5423192_5423392
gcttttacgtatgttactctgagaacaccctcttttatttgtggattagcatagagtttccataa
tccatggggcctcctgtttgtgtggaagtggttagtgtgtcctctgatttacaatcccctgtggt
ttctgtagccctgttttcctactgttgccttctttgccataagcctcccacgatcacctcccctt
ccaag

>chr2_188430755_188430955
taaggtggggcagggcatattcacttcttttgtgattcttcagttacttcaggccatctgggcgt
atatgtgcaggtcacaggggatgcgatggcttggcttgggctcagaggcctgacagaaagcataa
aatgagaactatagaaaaagtacatagtgtagaaataaaataaaatttcaggactatctaaattt

agtat

>chr2_194212955_194213155
cacagaggctgcagctcatggagggtgcagtgggcgggctttttgtagtctccttttctctcatg
gagtcatttagaagttttccttagtgctgttctgatatttaggaagagaaagatataaagatgct
tcctaggatgaaaataaactgaacaggtttcctgtagtctcacctggacctctcctgcaatccaa
aacca

>chr3_150820710_150820910
taagtgtaagtcaattaactggacttcagaggacagaaaccaaggcttatgtttgtgttctctgc
cctcagatcacctagcacactagcttgttaagtactctgtgaaaaatgatttaaaggaaaaattt
gttatgtaagattgcaatgcaagaactatatggcatacctcagcaataagggatctaaagagaaa
actat

>chr4_135545550_135545750
actaatcttccttccaaaggtttactgctcaaggctacacttaagaaccttcctcctcaaagtga
gttaccattaaattcctgtgcaactgtttgggagattgttttggttggaattacagtggtataat
gactgcagtgctcattgcctctgtgcataacacttgcaagttctttagtccttattagctctcaa
acact

>chr5_6143200_6143400
gtttccaaacaagatcacattccgaggtgccagggattaagactgcagcatatgaatggggtttg
agggaacacaattcagcctgtagcagtaggtattcttattgctattttttgtataaagaaatcaa
cagtatcagccagggcccacttaggaaaacagaaatcacaccaattattataacggagagaattt
agtat

>chr6_136827507_136827707
caaaccacacttgaaagtcacctgtagggaagggaagagaaatgtggggagggaagggcattggt
aggagtttttagaggacacaatccacagttttagataattcaggatattttaaggtttataagct
ttgggaaatttctggaaacaataaaagattatttatcaggggggcatctgctcttccagcctcagt
actgg

>chr6_151423107_151423307
agtttcctagaaaactgtgacaagcaaagcaataacacacgtcgagaaatatctgatcaagcggg
aaatcttctgactgtcggggatctctagtaagatctcttggaatgaagtgcactgtgtatccaaa
actattttccagcgccagtgaagttgctcttacctaaaacaaatgggtttatgctagtttccacc
aagga

>chr7_29322475_29322675
aaataggaatcgtgaaagaagcaacagagaaagatgaaaaagattggagaagaagaagaaaatga
aaacagagaaaaggaacaatatcagcaggtggtggatggcccgagtggtagaggaagtgaaagcc
aaatagcaaaagtggcagcaaagaggacagctggccagtaggagagaaaagagaagccacctctc
ctgga

>chr9_27354000_27354200
tggatgatctgtcacgtctgtgtctgtgtcagtgtgccaggctccccctccctgctggagaccaa
gtgaaatggcatgtgtttgcctgggttcccaggctgccagccatagcggctgaacagcaggtccc
aatgggagccatgaggcaagctctgggggtggatggatgtgctggtcttcaccaggctagccagg
gtgac

>chr10_77298594_77298794
aaacatccccaggcaatgaccttctgctagcctcatgtgatattcacattctacagtactgaatg
ccaaattttctgtcttggaatagagtggtacaggttgcatgttcaagtttagaatcagcagggtg
ccatccctatattagtgttgcttgcatgcaattgagtgaaagctttttagaggatttcctggcag
aatgg

>chr11_2024424_2024624
cacgatccctgttcctgctggaggccctgttgggagggtctccctctggaccttgggctcaagtt
ctttggggtccaagtcatgaccactgcagaacagagatttctctttgcctccaagtatccactgt
cctacgtgctatgggagctgtcatcggtggtgtccctgggattaggctcccagccatgcatgggc
tccta

>chr11_68868624_68868824

gtgagatgaggtggaagtgagcagagggggctcctggcagggctgcgagcctcctacctctgggt
cccacatcggtgctgggtcccaatgcccaccttgtctcaggggtgacgtcatcaccttttgaaga
agccccagatggtgttgaatagcgatcgccacccccagtgtgcaccagggaaggagcatcgtaga
cctag

>chr14_96448247_96448447
ggcgagggggataaatggtccccacctcaaagaccatgaagggttaaaggaaatggtatttgtg
agctccttagcagagtaacaggcacatcaaaagtgctcagaaaatctgtactcaaaccccaata
cccacagcctgaggagccaattactctcctcttgttctctctaaacccccttcaatcaggctga
cacct

>chr15_74556147_74556347
tgcaacagtgtggttatatgtgtgtctggatgaatctggatggtggtgtatgccttgcagtgcac
aagtatgtatagatcgtgtgtgtggctctgtgtgtatgcatgcatgtatttatgtggctggtggg
aagttagaggttatgatgagtggacctgagctgggatcctgccctgggctctacatacttggggt
aacag

>chr15_90011796_90011996
atccagaaacagccagtttagcaagatacctagttaacttaagacaatttcagcaggctttacaa
aagtttggaactcaaaggccccaccataccgggaactaaccagcaacccaaaatcaggctaaggg
ataaggtacttgttaaaacatagaaggagggatcacctgctcaacaattgcaaccccaatggaag
ggacc

>chr17_3875851_3876051
atgtcagtggtgaaggggaagtggcgggggcagcgtcttcccgcagagagaacagaaaacagtgg
cggggttgccctgctgctgccccactttggtttgtaaattggtttgagtcaaaatgcaaacagcc
ttgcaggcttgggaacctcaccttcctcttcttcggggtcacctgggcctggtggacaggtgtcc
caggt

>chr17_35118487_35118687
tggatgtgtgagtaggtgtgtatagtgagtgtgctggtgtgtgacaaagtgtggatgtgtgaata
ggtgcgtgtagtgagtgctggtgtgtggagtgtgagtgtggatgtgtgagtaggtgtgtatagtg
agtgtgccagtgtgtgacggagtgtgagtatggatgtgtgaatagatgtgtatagcgagtgagtg
tatat

>chr20_50214993_50215193
gctcccaggtgacagtgtcaatggcgctttatgtaagttctttccagattgatgaaccaagaaaa
aggtcaactgaatccttatgcttaaggtttacaaactcaggagagtttcttgtatagtaactcta
gtttcttgtttgcttatttctttgtcttccccaccacaactaagatgacctccgagaagtaccca
ggcgc

>chr20_60140805_60141005
cctggcagaggagaagcctccttccacgtagggaacagcatctgcctgcagagggctgctgccag
gagccagccgaggctacatgcctgcctggacgctgtcatgaggttgctgccggggttgctcagct
gcctggggtgagggtgtctgcatccacccccctctgtcctcatggggttctccagccccatgtaca
ctgtt

>chr20_61865355_61865555
ccccggtgcactgcgggcagactgggaaccaggcagcaaatccacccagcttgaggcccacgtcc
tcctgaaagggccccaggcccacacccggggttttgctgaagtgaccaaaggtaaccgctgtgct
gagggggcggtggaggacagggagggggcgaaggcgaggtgggcgctggaggacgtgggggtgaa
ggcga

>chr22_46241536_46241736
tagaggggaaggcttctcagcttgatactgagtccaagtatttgcagagtgattatttttttaata
ccttaacaatggatgtgaagatttcaaaaatacttatttttgcaatcttaaaacagtcggctctc
tgtatccacagattgaaccaactggggattgaaaatactcaggaaaaataaaatagtagtacaac
aacaa

>chrx_8672400_8672600
agattgtcttcagctgcccaacctccccttcccaagtgtaaaccctcctgccctgccccacacca
actctccttctttcctgcaattcaatgctccccataccattggcttatacacacccatcaaaatg
agaagaaagccaatagccacagagcctattttgaattgggaattaatatcaatacattgcgttga

gcaag

>chrx_45763456_45763656
ccatgacacaggcactgaggagcaggagtcaacccgtgaaagctggcaccaggacgggtctgtcc
ttcaagggaaaagacatagcccctgtgggagaagccatccaaggcagggaggggtggtctagctt
tcccctccgaaccattccttccattggctgcacccaggcagctgacacaggagcttactcagaaa
actgg

>chr2_181010755_181010955
agtcttctcttacctaggatacaaggatgtatatccatggtttttctcactgaattcaaattaat
tgcctgtgatataagtttatatttatgccaagatacacaaggttactttttatcctgcttaact
ctagaaaataattaagattatctaagataaaataattatataataaagccgctgctataaaaata
gatac

>chr3_23787796_23787996
ataagactccaacgctcaatagcccacccagctaataataataataatgataataaaaagtctat
gtggaaacctgtgataatccaaataactaattctagccattaatttcagccttctccctcactgg
gagattcgagagctgcgaaacaccaagaaataaatcactgagcttcgcttctcacagtctaccgt
ctggt

>chr3_191640106_191640306
atttgtcctcctgcccatttttgattgcattgcttaataaggattgagtttggagagtcctgtat
ctattatgggtaacgtccttcaccaggtgtatgttctctgaaaatggtggttgtaagcattgacc
tgctaggtggctaattaagggattggcttgggagcttgcctctgttttgcccacctcagtgcttt
ctcat

>chr5_169691222_169691422
ttcagacatactgaagaccctgaagaccaattgccccaaattgcaattctttcttcctaaataaa
acaaatttagagatttcttctacgtttttattttgactttgatatggtccatgtgggctgacaa
ttgttagttaactcgcctttctttcccttccttccacacccaggcacagttagtgccctatggcg
aatgg

>chr6_18485421_18485621
ctctcagggctaagatacatacctcattggaatgggcatagctgtggctcaccagatagtggagg
agtccctgaaataccttgatgaacttgtaggaaatctttcctctgaggtgctgagaaagccattg
acagggacatgctgtgctttgcaattcattgcaatgttacctgagggtttgttggggaaggtatt
ggcca

>chr6_34109022_34109222
gctgtccaggctctcctgggactcaccatcatcaccaccaatgggcgaccagtgccttctcggga
gaactgagagccttagctagacaccacggcctgcctgtcagcatggctcggccccaaatggcgtg
ggctggcattatgggctggcatttcccaattaccacagataatatctaccttaattgaatggggt
ccaga

>chr6_129517307_129517507
ttgccaagaatacctatagtctagtttaagatgcacattaggagagtctgctgacataatagcat
ctggcttctaaaagtaatctctggtaaactcttttgttttgttgttcttgcttgactttccattc
tacttcacggatctcaaattaaagctatagatttgaagaaggaaactgatatttgagatttgtag
gacag

>chr8_129476018_129476218
gtttccaaacagctagaaacatccttttccctgaataatatccatgttaggctttcagagctgag
tgagtaataagatactagggagagtcctggataacaggtaagtggagttggaaccacttccagag
caaaggataaatgctcttacttggctcataggcctgacatgacatgaacaaaaaaccagagtttt
ctaat

>chr9_2213600_2213800
agtgggcatatttgcttataagctgttaagcagggtattggaaggcctctatggaacttcactta
tcagtgatattttgcttaacctaactacatgccaggtatgtagttcagtgacacttaaggacaaa
ggtggagtcactccccaaacttaacatcaaaacaactctctaagaagcaagcctgtggacataag
aaacc

>chr9_102062179_102062379

atctccacattatcagcatgacttctctgagtcatgttagcccctgcatcctccccttcaggcag
caggatgtcctcacaaaacagggccattgacaacacaggtgtgcaatttcaattcagtacaattt
gattcaaaaagatgtgccctttatgctggcgctggacagagaaggggagaagaaactttcttggc
tgggc

>chr13_20260800_20261000
aaaattcaaaaatatagtttatgtataaatagtatttgaaggctgttgatttttattaggtattc
tttctaaagtgcaacagtgtaataaagattatttctagatctcaaggaggatctgaatgatggct
ataatgaagggcctatttaggtaacttttaagtttgaggaataagagaatggggttagtccactt
agggt

>chr14_48734050_48734250
ttgagatctagaacagaataatgggttgtggagggaggtattgaggataggagagtatatgggtt
cggcaccacggggtgtataggcaaaacaatttagttgataaggcacagatcctgaactaatctgt
aagacttgtccagtttttggacaggtaaaatgggggaattgtaaggagagtttataggctttaaa
agacc

>chr14_70758247_70758447
ctcaacctagcagagccaaagagatacttgtatgatgtaagtcagattatgtcattcttctgctc
aaaactctccaataaatagctcaccattcccttcagagaaaagccaaaggacttaaaatggccaa
caaagcctatgatctgcaccccccctcctccctctacctcacttcatctttctccctgactcactt
ggttt

>chr15_60412708_60412908
agattctcttcataacccacttttgagctaagaaagccattcatcccaataacaatagctggcct
ttattgagtgattgccatgcaccaagctctgtgtgtgtcacttacaaatacaggcagccttgctc
tcaagttcacacacttaatactaccctagaaaaattctcttaacataaaatgcaagtcaatctgc
agagc

>chr16_710199_710399
aagcacagaggccccccgcagggctccccagcacctcctcagggttgcttttggtcgccaaaggt
gatggctctggcccttgggcgcctgtcccctggaggcaacttcatcccacacccccacccctgcc
ctccgctggcctccagacgggactctgtggtggcctcctgctgacctgccttcccctctcaccca
gcagc

>chr16_3048399_3048599
gacccctggctctgccccacacctccttctgcctctcagcccttctagcctctgcccccagtgtt
gccacactcatctctgctttccctctccaggacacggccatgaaaatgggccctggccaggtgag
gccactgggaccaggggtgggggcccaggaggaagggagagggaggctgctggaagacggagggg
cctcc

>chr16_29923299_29923499
agctgctgctcctcccggggagatgtcaccatggggatgaggcacagcggggacagcgtctccct
tttttgctggggaagaagcggaaggtggttaacagcacaaccaaaggccacgtactcccccactt
aaaagccttcaaaagctggttgggaacaactccagactcctcagtggtccgcagggctctgcagg
ctcca

>chr22_42777256_42777456
tggcaaggcccctgctcccagaagctggtttcccaggatgattttctcaagcaagtgctgacaga
gcaggagtcaagcgggggtggggttggaggctgaaacccattgaaggggtcaacttcctagaggc
aggcagggatgcctgggggtgccagacgtggctgagatgtttctgcagaggggaggttgggaaga
cctag

>chrx_62975075_62975275
ccactgacatacccgccgaccaataacagatgccgcagtagcacgttcgctgcccaggggcggga
cttgcgctggcgtgctagccgcgaccgccaatcagagcctcaaggctcccaagcaagctcgctct
ccccagaccggcgagagtcaccagccaccagttctggtttgggggtggggagcttcttgggggcg
gatag

>chr1_3270340_3270540
ctgcctctgtgtcttgtcaccaggccttaccctggggacccctgctcccagcggagccagtagtg
atgacaggcgcagctgggagcagcttggtagacctagggggtctttctagaagccaagggggccc
ttggcacacacatgtggatgcagggctgcccacccaacactgctgagcccacacaggcccaagag

aaaag

>chr1_12125613_12125813
gcagacctttataaatgcatgtccatatttatattgttttaatagacatatatatagttttccat
atactataccctacagtacatatggttcttcatttggctttataatgtctatgaggcatcttggt
gagctttccatgctgaaacttacagatcctctgcctatcttttaaaatgctatgtgatcttttgc
caaat

>chr1_114566077_114566277
agtggaaagcccatttgcataataagattagagtggggtggccagtgtcccctcctgctatgtaa
acctcacacttggtccaaccaatctgtgggccatatgtaaatcagacaccgtctcctcaagcctg
tctataaaatccggtgcactccagtgcactctgctgcaggccggaagtcccatttgggcacccct
tactc

>chr3_191389306_191389506
cacctttgaaacatgggtgaataatcagagaggtgtccctgcaatgattaaacaccaagggaagg
ctgccttcccagtccgtgaccggcgccggagttttgggtccacggataaaacgtgtctcctttgt
ctctcctagaaaatgaaaggaattgaaattaagagaacggagagattgaagagtggtgccaagat
tgaaa

>chr5_34545243_34545443
tgttactggcagcaaatccatacggatctgcggtaacctcaattcttgcctcctcagaagaaaga
attcaccaagaagagaccaaggcaagttttagagcaggagtgaaagtttattaaaaagctttaga
gcaggaaggaaagtacacttgtaagcaggcaacttgagagatcaatgtgccatttgacttttgac
ttgga

>chr5_143116607_143116807
tgactgcagcgtccaccagaattcctggagggcttgttaaaactgattactgggttccatcttca
aagattctggtcagtagatctggggtgaggcctgagaatttgcatttctaagacactccccacga
atgctaatgcagtttgagcatcacagcactagagttagctgttggatcctttcctctggggagtt
ggatg

>chr5_176202194_176202394
ctcgtggttcgtttgaggcccgtcttcccaccaacaccacacagagctttttgagggctgagaat
aaacccaagtcatctacgtctctccaggaacacgtgtttattaaatgaatgaatgaatgaatgaa
tgaacgaacaaaccagaagctgggtgacggtcgctgtcatggagtagaaagaaagactcttgggt
taggg

>chr5_176369594_176369794
aaattagctgggcatggtggcaggcacctgtagtcccagctactcgggaggctgaggcaggagaa
tggtgtgaacccaggaggcggagctttcagtgagccaagatcgcaccactgcactccagcctggg
cgacagagcgagactccgtcttaaaaaaaaaaaaaaaaaaaaaaggttgggccaggtgcagtggg
tcaca

>chr6_168733551_168733751
taaagttacgggagaggatgtcaaaaataacatgaaatagaggaaatctgtggccactcaatgca
aggggtagagctcagtaatgtgtgaggtcactagcaatagaaaattagcaccaaaatcttgttca
ttgtgaggaccctaagtgattgagaggaaattaaaatacagaaggaataatttctttacatgtga
attat

>chr7_137888860_137889060
gtaacaaaattgcacttgtaccttataaattttacaagcaaaaaaataaaataaaataaaatata
aaataaaacaaaataaatgaatgtgatgatggcacctgagtcacgtgaaagacagctgtaaccta
ggcagccgtaaactttgtttctctgtttatagattagccttttaccttgcctatattgttttgta
aaatg

>chr9_45734404_45734604
cccctttaaagaacgccaccgctgagaactgaccctcacatctcccttcctgaatttctttaggg
aaagcaacaaccatccctgtaagccatgggttttcatgcggaggaaggagagggctagttgtctg
agagggaaactgcttaaggctgcagactaggaaatgggattccaggtttaagatagctcctttct
ggacc

>chr9_128191379_128191579

ccacacggcctcctccgagaacagacgcctctgccaggccatgtggagcgcaggtgtgcggggcc
ggccgctcttggattaaagtcacacacacacacacacacacacacacgccaggtgcatacactta
aactcacgtgcatgtacacacatgcagaaactcacaacacatacactcacacatggttacatgca
cacac

>chr10_104951210_104951410
ccccataaaacaggagaaatagcctagagaaagattaatgagcacctctgtgcctaacattctat
tgtaatctggctttctttaggaagatgtgatgtcttctggttaagggtggcaggtactataaggg
aatactatctatataaatcttcttacaattacaaacttctgaaaacttgtagctgatggtaaatt
ttcat

>chr11_94391152_94391352
tcagtgagtctactttagctagttttgatccttgttcaaaaacagatacatattttaggttgtt
gctatttgtttatacttgaatggggataaattctatgtgctttatcaagagctttagatatttaa
agagcaagacagtctgagtgcccagcgagaaagcctggttatgttcagctcctcggtctctcgct
ctggt

>chr11_126906790_126906990
catattttcactgacttgctctatgctaaacagctttttctagtacaatttgagccatgagtgg
aggtttcaagcaagtcagtgaagagtttttgaaacaggctggagtgcttcagtcctttcttttca
ttttacttctggtgtaatagagctcttcaagagtttcatttcaggggatgctgagtattaggaga
gggct

>chr12_77018069_77018269
ctcctgaaatccaagttcccagatgccagccaagggccggctttgtaagcaggcctttccaagga
taagcagttagggctgctacctcaacttttgttgcacagacctgcacagggtttgtctgtgcag
gtttctttgttttgttttatttgtattttacttagttgccatttttaaatattaagatatttca
ctcag

>chr12_79268269_79268469
tcaataagatagttccccaggaatgaaaatgcaataatgtttgcaaattgtctggtgccctgtta
ctatatggtagatttgaccacctttgctcctctttctcttttttatttcagaaattctttcac
ttgtaaaaatgccacctgactcaaaatgcttaagcaagaaaagggattttggtgctcatgtgacc
ataaa

>chr13_73109199_73109399
cggatggtgcttccaggactcacagcttcccttccctctggcccttctttatgccctggtgtttt
tcagattcagtatctctattaaaacttcctactcctctttcctggccttgtctccctctcttaac
ccctacaaaggactgaaactggctgtcagctctggggcttgccacagtcatagcagcctttgtta
acaag

>chr14_69096047_69096247
ctccaggggggtttgcaaaataagaaccccaaaacccacctctctttcccattttcttctttaaag
gtagagaaagcactgcatttcacattgggtcttggaaacccaaagctagacacaaaatgtgggcg
ttagcagctctgaaaagtgtgtgcttggtcttaacgtttattcttaaaaaggaggtgttcatacc
ccagc

>chr16_1299199_1299399
ctcttcctcccccgacctctgacccttggcccctgacctgcatgagcccctccagctccctcctc
ccagaggggtggagggcgatggccacagcctaggcctggaggcagaccccgctccaggcctgcac
agccccggctcgcaccccagtgcagcctcttctccccgtggggaaactgaggcagagagggcgcc
gtgac

>chr16_8981099_8981299
tattgacccattattgccccaacctacagatgcggaagctgaggctgagtaactagatcagcgct
ggagacataggtctgtcctcagggttcagcatcagggaggaaggcacagaggtgaggggtgcatg
tgtgtgtggctgaaggcctggaaggaagggtgtgtgtttatgtgtgtacgcgcatgtgtgtggct
gaagg

>chr17_35200087_35200287
atttggcatctggttcaagtttggtttatttagagcccaggtcggcgggcttttacatattaaag
gtgcattttaaaagagcattttctattctaatttgggggagccaagctctttagggctttcggga
gcggtgtccacctgatttacatttcccccattggagccgagccacagacgcacattagaatgtaa

ataat

>chr18_33072202_33072402
aaaacagcaaaaattcaaaatgcaacctcaatatccaataataaagtcaattaaatatgagtagt
taattccttggaatataatgcagtcaatagtcataaaagactatttgacttttaaaagtctaatt
gagaagactatcaacacatagaaatgttcacaaaataatgctaagtgaaaaacggggcacgtaat
actat

>chr20_60091005_60091205
tccctcacccctcctgcctggtttcctggctgctccagggtagaatgcagaacttctccagcccc
atcttcccctgggctctggaacgcccccccaatacgattccaccgcaggcaggcagctccctcac
cacaggggctcgcctcgtgctctgcgttgcactggcagaggggcagcacaggacttaattctgca
gccag

>chr21_31232529_31232729
aggctatctactctaagccttttgactgctggaaacctcctagctgcagcttgtgagccctgggc
agagttacagcctgctgctgttactgccaacctgggggagtgacagcataattggatgcctctct
gtcttttggctcaagacaagggcaaggaagaatgcccacctgcagggaggaagtagagtccagaa
gacag

>chr22_30281000_30281200
gaccagcacagtggtacagccccgcttgtcgttaatgaccagccataaaaaaccctgagtttctc
agctcataggccaggttcttcagtggttgtctagtttaaggctggtcattagaaatattatggac
tacctaaattcctcgaactcatgcaggacctggtgaattaaaatctctgaggatagagcctgggg
atctg

>chrx_92084144_92084344
cccactcctgcccgccagagaacaaacccccctttgactgtaattttcctttacctacccaaatcc
tataaaatggccccaccgttatctccctttgctgactctcttttcggactcagcccacctgcacc
caggtgaaataaacagccatgttgctcacacaaagcctgtttggtggtctcttcacacggacgcg
catga

>chr1_164562176_164562376
cacacaatccttagattttttttttttttttaatttgagacagagtcttgctctgtagcccaggc
tggagtgcagtgccgtgatcttggctcactgcaacctctgtctcctgggttcaagggattctcat
gcctcagcctcccaagtaactggcattacaggtgtgtgccaccacacccagctaatttttgtatt
tttaa

>chr2_114430330_114430530
tcacatgaatattgatatccgaaactccctaggcctaagcatccatttgcccagtcaagaatttc
aattctcacagtttttctcctctgctccacatattcctgggaatcaccaggatgtgtcttacatg
tgccaacagtgagcctgggctgcctctcaccctgagtctgtgttgctgtggaggcaccactctgc
agcct

>chr2_131268730_131268930
ctctccagctgcatcccatttctctgttcccctgtagagcaaaatggctccaaagcgtggtctcg
tcttaccttgaacacccctcctccctctcgctatgtgagctactccagttcagctctccacctct
gcactaccctgaacctgctcctgccggcagcaatggcctatggagaagtgcagcccccagctgta
agaca

>chr3_19034596_19034796
aataaggctataattgtttcaaaaggttgacacacacttaaactaggtatcttggatttctttga
gtttaaagccctagtagaacagacaactgaatgtttttaagtatgtggtgcatttgctgtttttc
tttcattattgttagggtttgatggcaatgttattgtcagtatggtccaacagaactctctacaa
tgatg

>chr4_92488377_92488577
tgcactatgatctgagagtatgtttggtgtgattacatttcttttacatttgttgaggattgttt
tatgttcaattatgtggttgattttaaagtatgtgccatgtggtgatgagaagaatgtatatttt
gttgttttaaggtggagagttatgtaaaggtctatccaatccatttggtccaatgttgaattcag
gtctt

>chr4_159182550_159182750

agggatttttttgcaaacaccaaagactctacatgctgtactattttatgtaaatatgtacctt
ttctatggctattgcaaaaattctaaatacctaatcattgcttatcttcgatgctttgtaagact
gatttacctcctgaaaagatgattcaagatcataaataaaaggaagaaaatgtcattttccttaa
ccttt

>chr5_89600644_89600844
ccttatttgtagataatattaatggtgataagaatagccaggcttacaagaatcatttccaaact
gcactcaaactgaactccaagtctgcaaaagcaatattgtaccttccccacccccacagaaaaat
aacaatagatcacaaccaaagataccagcaggtgtgcaacatgcaaatggcaagcatgaaaactg
aagtg

>chr6_144965507_144965707
aataccagggtattccaaatgaatgatttttatctgactcattaagaagtgaaaataaatatttt
attatacatgtaatatgatttagttttacaaactgttaatatcataataatctcaagaagttcta
aaaatggagataattatgacagccataaaatataaagtatatggaattatatatttggctatata
ttagt

>chr7_27473475_27473675
ggattaaaaggaatgccattcagttgaggcagcatcttaagacctggggacgtctgtccccatgt
aaaacttctggtgaatgatttaaactatctctactaggccaacatccttttaagctaagaaaacc
acattctaatctagacatttgatataaaaattagatttcatgaaatagcataaacgtattgtcgt
tagct

>chr7_143583067_143583267
cggcttctctacaaatatgatttctccctgtggagaactttgttgctagttctgatccatgttat
ttgagtggaacacgagacttgaccatggagcaggaactggctttatgcctgaccaggtcctgcac
tcgcatctactcatctaacctggagagctcaactgtaaaacctggaggaaccactcatctctttt
tatgg

>chr10_60775594_60775794
agctcacttaacttaaaagttcaaggtggtattatctttaggcaagcaggatccaggagtttgaa
taattccattgcagatgctgtgttctctattgctagtctctgctttatcctccattagccccgtt
tttttcaggttttcctttgtcatgacaaaatgtttgtcaatagatctaaattagtcttagacatt
ccaat

>chr11_132202190_132202390
caggttccgggggtggtgagcgcatgtacctgtggggtgggctgcgcagactggatatggtcaca
tagtgccagccactcaccagcctgagatggagatgatgatgatggagagcttcagaggggctgct
caggcttttcccagatatactacaagcaagagaatggctggcaggcacttggtggcctttttctg
tcttc

>chr13_54707199_54707399
aatgtcatcagttaaggcaggaactggccatctggatgtgtacgtgcaggtcacaggggatatga
tggcttagcttgggctcagaggcctgacagttttcccttagggcttctgtcaggccagcatttta
atatgcttgacatttttgactttccagtaaatgtttaaaagaaattgtaaactaaatttagctga
tgaat

>chr14_103813847_103814047
gcacaaaggccgtcacaaccttccacaaataatacttctacaaggacatctacccagcaactgcc
tgtccaaccttggaccggcgtcggccttgttattgaccttcatggccaaggctgattatctcaaa
acaattatgtaatcctcctcacttttcctttaaaagctattctctccctttaccttcctaaagta
cacac

>chr15_84145796_84145996
agtcacctctgctaatgccttacttggccatcacacaccaaactcttctatccagatattatagt
actggggaagaatgactgtggtatgtttgcttggatggggactctcatttacttggagggaggaa
ctcaagaagcatttttgggttgcaggttgtctggcattttgctgtgcatttcattgtaatttctc
accag

>chr16_76920099_76920299
aagtactcacagataaaagggtgccatagctgcaacttgctctataaatggccaaatgactcgga
aaataaagcatgtgtgtgtgtgaagagagagagattgaaagagaaaggaaggggaacagggagagag
agaataaggcagatggaactactactaaaataggctctatacatatgtatatatagtggtgcttg

gaaca

>chr17_27923074_27923274
gccgccacagaaacagctcggctttcctcaagcactcaggagccaggtttccctggtcttggctt
ggcatagctgcctcttcttcactccaagaaagccccgcagaggccctccgtgggcaggcttggca
gtgcagctggctgaggagggggccagcatggccaccttcagagcaggttaatgggctttgctcag
cccca

>chr20_32262139_32262339
gcagccccgcgcacgccatggcgccgccacccgctcggctcggctgcggttgctgccgaccctg
gacgccgcggcggactcggtgtggctagaggccgccccttggcgccggcgccgacgcgcgggctc
aggccccgcccccgccccgcccccgcggacgccgggttccctcgcctcaaggtccaactccagcg
ccgcg

>chr21_35231530_35231730
atcagcagttcctcaggatcactccgggaagctggccctaggctggtcgtattttcttccaactt
tagtttgcagaggcagctccagggccaggagtcaagagacctgggtttggattctgacttactca
ctgcctttcctttttgagccttagtttccccatctgtaaaatgaaagggttggattcgatcatca
caaag

>chrx_89032944_89033144
cagaccaaaaaggggaagctacctatattactgagacaaatatcatgtatgtttttatatgtgct
ctgaagaggatccttattgcctgtcactgcttgactgaggctgctgaaaatcaaatacaaaacct
catcctttgagtgtgtgaattacaatacaggttgaactcaaagcctcacagcatgtctactgtta
aagta

>chrx_115947772_115947972
taaagtcccttctgttatcagactgtattgaggtgggaaggctaaactgaggaattatgtctgac
agaagggaagaaatgactgcggtggccttctcagaacctgtaggaaaggcctctacctatccagt
gaaagtatctacctagactaagaggtattttagttatctgactcagggcatgttgagtaaagcta
atttg

>chr1_57822612_57822812
ctttaagagacttactttaatccctttgtgcttcaactatacattggaaacaataatgaccctat
cctatagggtcactgcatggattaactaagttcatcctgtgaagtcattagaacagtgcctggca
cacagtacatgttcaacacatgttatattttatcaccaatacactctagcacataataggtgtcc
aaaca

>chr2_150172354_150172554
ttctgaaaaccctgtgaaatggactggttcgaacgggcagtttataacatgatacttttagattg
gaaggcccagtgaggtgaggatcttgaagtactcactccctgagaagttattctcaaaatgaaaa
cagtttaaataggtggacaggaacaggtatttatgaagcaaagagttaggctaatttttgcttgg
tctca

>chr2_207096955_207097155
gataaatgtttgagatgatggatatgctaattatcccgatctaatcactctacatatgtattgca
acatcactatgtaccccataaatatatacaattattatttgtcaattaacaaaataaaatttagt
ttacaaaaacacagaaaaacaaaaatccttcatggcttctctctttttcctgtctctcactttac
ccacc

>chr2_236666061_236666261
agccttgtggcttattagggcttcctctgttcacacgctgtgtacatcgtcctggtggatgggtg
tttatctggtagagtagttcctggtctaggcagatccatgtcagtcctttgacctcattgcccct
ggctttttatgtttgtgctgcagttaacattctggaaagcacttttatagaagccattccatttg
tcttt

>chr2_236757061_236757261
aacactgggagctattcagctgcctgtaccatatcagaacatgcttaagaaaaagagggaaataa
ttattgtctcaacaaagatttgggtttagcaaaaactggaaataattcacagtgaaactaataat
ccattgattgtgatgtccatggtgagatcattactctcgagaataatactggtaatttagtttgc
tcgga

>chr3_115833910_115834110

ttaaaatggaatttttagtctagctcaggttttatgaaattataaaatgaaaactaaatatggct
tacatttggggaatttatttttttcttgaaataagccaatacatttgcattactttcaatgcagaa
tgaagacagcaaagctgaaaggggctttaataataaagggtaaaggccagaatttgttgagcgtc
tattt

>chr3_195537003_195537203
tggggaggtgtagggaggtggtggcagtttcctttgccctcagcctagtcctgaaacatcccgag
gtggaagggtccgcttcaccagcatggcttctcttgcagcctttaaacggcactcgccttctgac
tcactcccaggaagctcctcaaatatgcgcccaagaaatattttccctcggcgactgttcttagc
ctgct

>chr4_91654377_91654577
taacaaggatgagatgatatctctttgtagttttgatttgcatttctctgatgattagtgatgtt
gagcaccttttgatatatgtgtttcccatttgtatatattctttagataaatgtctattcagatg
ttttggccatttttaaattgcattgttaatttttttgtcctatagagttgtttgagctccttatat
attct

>chr4_183004606_183004806
agaaatgtgttattgatcacctaatttttttatttttttttcagtcttgcatttgctttttcaactg
tagaagtgcaagaagtggtaaatgtgccgcattttagacgttaaaggcattgttaacagatttga
cacaaaagataatgttatgagatgcaaacgacagaaatttacatgtcaaatcacatacttaatat
ttatt

>chr5_72779844_72780044
ttctgccagctgttgaatatatctattcttattacacatttggaaactggggaaatgatcacagg
agggtttaacaatccactgtgagatgaacctgagttaaaactccattgagccaggcgtcgtggct
cacgcctttaaccccagcactttgggaggctgaggcgagcagatcacctgaggtcaggagttcga
ggcca

>chr6_11993414_11993614
aactaaaaagaggggagttgagcttacacactaaatgcaaaaacccttcatatttcttaaccttt
tctaggctggaataccgtttgctaagtacacacactcttgtgagtgcatacacacgcatacacag
acacatatgccctaggcagacaactagaagtcttctccagggcatttgacaagcgcaatagaaaa
gatct

>chr7_13789275_13789475
ttgtttgtggtgttgttgttgttgttgtttttagttctggaaaatacctttagaaatgtatacat
atgccagacacctaggacttctggaacctggcaaaccaccttttttttaggaacaatgccatcata
acagatgggtgttagtgtaggcatgccagggaatcaaaatgtacatcacgttccactacaagggg
gcaga

>chr7_127657564_127657764
ggacaagtcaggaaaagattgcctagctgactcttccctggtctcagttccgaatgaaaagcctg
ccatgcggccttcagcatctgctactccccgtgccacacctgatcccatctcaagtcatttcaca
ccagcagagggaagccctgttccaactccagcagcagcaccactcggggaggggggcaaactct
ttacc

>chr9_8750600_8750800
ggcatgtaagcctgaacctgaatgacaagaagaaatgcagaaaagcttcccatgtgaaggcacag
actgcccagggcagaggctgggatgcaggaatgagcttggttgtggctggagcccagagattccc
agcagagaatgataccaggcagactgggaaaataaagtgggcccaggttgcacagggcctgtaag
caaca

>chr9_80639980_80640180
atggacatttatttttgtttttaaagcccaaattcatgaccacctaggatgtgttaactcatttc
atcagtatgcctaggtccttttactacatgttagatgagaaatcagacagatttttacatatcgg
taatgtaacaaatcaaaggtgattgttgaacaacttcctataattttatttttttacatatcggta
atgta

>chr9_87771380_87771580
ttatccctttaaagcagggtgggtgactggacatgggaagcagcagagatctgaccagtacacct
gccttcctcacttggattcctaagcccacctgagttgtcatccagtcccctgttagaacccttgt
agccgcagctgtgttctataactacagcaatgtctgaggatacttcgctttatgtgcataaagga

gagag

>chr14_33634249_33634449
agtttagcagagtgtaagtcaggtttggggcagaaggaaaaatcagtcaggataaatgggtcagc
attcatcatttgttcaacaaatacttattgtactaggtgccaggcattggactcggcctgtcgga
aagcactgggtgatttatttttgttgggttgactccctggtgggattattcacaaacaacctttc
gatgt

>chr15_70923546_70923746
tggaaagaggtttgcggccagtatttatccagggtaggtatttctagttagctcttagcaactcc
ggggaaaggggcatttttctctgccaatatttgcatgtccattcagaaaagaactccaactggcc
ttgcttggatgttcacacccttaactagtccctgtggccagggacagggcatcctggtttggagt
gagtg

>chr22_32607800_32608000
gctgaaactcctggagtcagggctaggttaatgggaaggttcccaggacactgcactctagggta
accagcttctccctggagggagagggtactgtctgcatcaccccatggtatcaccaagcaatcag
tgttgagtcaactcccccagggagaccaggtatccctgtccatgaaaattcctcctagtggcata
aatgc

>chr5_118739101_118739301
ctgtaatgtagtacaatatcaaaaccaggaattgacactggtatgttggtgctagactacagact
tccttagttttcactgttgtataaatggaaccatacagttgtaatcttttcagattggcttttttt
ttactaagcataatacccttgagacatagccaggttgttgcatgtatcagtaattgattcctttt
tattg

>chr2_188423155_188423355
gtgttgaagtgttggggcagtgaaaatttttgggggggtgatatggcgagagaatgggtgatgttt
atcagggctgcttcaagcgggattaggggcggcgtgggaacctagagtgggagagattaagctga
agggaggtcttgtggtaaggggtgatattgtggggatgttagaagaaacatttgtcgtatagaat
gattg

>chr16_70694299_70694499
ggacctggggacctgaaggtggatggggacacagctcctggcttctcctggtgctgccctcactg
tccccccgcctaaaggggggtactgagcctcctgtggcccgcagcagtgagggcacagctgtgggt
tgcaggggagacagccagcacggcgtggccattctatgacccccccagcctggcagactggggagc
tgggg

>chr5_162236222_162236422
cagaaaatgacagacaatcccctgacaaaatacatcagcctcaatttctaaaaattatatggtga
tcaaataaaacatttctgtgacctagatctaactcagagtcaagaagataaaatcctgacctaat
gttcagccattttattttattttatagatagcaaaccaataccatcttgttggggggaagtcaggg
acccc

>chr4_152474350_152474550
atcaatgattaatgacctgcatttttatttataatatcacatggttttatatttcataaacaatt
acaagaaaagtacatataattaaatcttaatttgctaatttaaattgtataaaaacagggaatat
atacaactaaatttgtgggtattccttgtattattgatcagtatctgccatatgaaagggaagaa
acaaa

>chr18_34768602_34768802
tccagaactgtctccttcagtaagtttatgcctcaccacagtcctcaccacaggtttccctaatt
cttttgtctttttcctttggatagaaatatttttgtgaacagcaaaatgtttctactgctgtatc
aatgttaggtcacatggcattgacttttattcaaactggaagatttcatagcaggattataacat
tatat

>chr19_17958000_17958200
ccagccccagtcgagtttaaaataaggcttcagtggcagtggcgccccactgcaatcccagctac
tcagggggctgaggcagcaggatagcttgaggccaggggttagagacctgcctgaacaacatagc
aagactcccatctccaaaacaacaaaaaaaatcttaacagaatattattattctgtaatactgtaa
caaaa

>chr7_155017267_155017467

```
tgtgagtgactacggaagacaaggtggactccaaacccgacgctatgatccactcgagcctcgtc
cagggaatttatggagcacgtgggacctccctgccatccagaagcacagaggaggtgcgtcccca
caacaatgccaggaacacacagcggggaattctctgacatcgagaggcttccagtgaggggcact
ggctg

>chr2_9533749_9533949
cctcccgcagaagaagcctgcgccggggtaagccacccccagccagctcggccatccgtgctcct
gccctctgacctcacctgcccagggctctgcaggaatgtttgcaatccccagggctcctactcca
gcgtgggcgccacataactaattcatcggggctgaagggtcttaaaaataactggaggattttta
gactg

>chr4_105613351_105613551
ctgctcgggggtcaggggtcagggacccacttgaggaggcagtctgcgggttctcagacctccag
ctgcgtgctgggagaaccactgctctcttcaaagctgtcagacagggacatttaagtctgcagag
gttactgctgtctttttgtttgtctgtgccctgcccccagaggtggagcctacagaggcaggcag
gcctc

>chr1_10879813_10880013
ggctgctctcgggctggtcagggcccagttaggaccccagagtcaagctccttccagggggcact
gggaggggcggggatagagggactcgtgggtgagctgccaggcgtgggccaggctggccagcgga
aggagaggtacagacctcccacgccgagggtgcccacacaggccccaactctggtcctcggcagc
cagcc

>chr16_12322699_12322899
ggcgagtcaagagccagagggacttcattgcaaagctggtgatgcggggcaggctgcctccctgc
agtgaccacacgagggagcctccaccgagtcttctgactcctgctgctctccctaagtgagtgtc
tagaatgcgcttttctcccccactcccaaatgccttgcagggcttgcttgccctcattccttcac
acaga

>chr13_89894999_89895199
ccttgtgatttcctaggcctgtctttactttaatcccctaatcctgtcatctcgtaagcctagga
ggatgtatgtcacctcaggaccctgtgatgattgcgttaactgcacaaactgtagagcatgtgtg
tttgaacaatatgaaatctgggcaccttgaaaaagaacaggataacagcaatgtttagggaaca
agaga

>chr14_63863647_63863847
caataaaagtaataatatgaataatagctaatacacatatggcacttactgtgatcatcaggcaa
ttttccaagcaatctacatattaatcctcccaatgagatagttttgtccccttttacagaagag
gaaactggggcacatagggttaagtaacgtgcccacggtctcacagctaataggtggtaaagcta
gaatt

>chr9_124710179_124710379
atagctacaaagcctggttatgctggtgccgtgcttttggaggatggaaggagattgtaaattga
aaaggaataaagaaaactaccatttactgactgtctactctgcaactaggcactgagctagacac
tctcagacctggcactagaattgacacttactgaataactattacatgtcaagccctattccagg
ccctt

>chr6_56658041_56658241
tcttagagttgaaaatggtattatgattatgtaaagaggatgtccttattctgacaacacaaata
cttaattatttagagacaaaaatgtcatgatgtctaaacttaaattttaatggttcagtaaaaaa
aactgagaaagcacaaattcagcaaaatgttaacactcagtgaactaaggtgacaggtatacata
gggtt

>chr9_139642979_139643179
ctccaggccctggagacccgtttatatggctctgaatttctccgtgtgggtggcactaactgcca
ggaaccctcctggcctcctgcaaaattacccaggggagcactgagcagtggcagcctctgagccc
agatcagaccagctgcacagccaggagagggtctggagtgtgtggatcaaccatactatactgat
cccct

>chr17_41440874_41441074
cagcaggcagcaccggcccgctcccggagccctccatcctgcgctttcccaaacccccatcaaaa
caccaacccagtctcctccctctcaagccattttccaacctgcctgggagccactctgatctccc
agaacacctcattttgtgaattataaaccttttcacaccttctcggtccctgtgtgatgtcacca
```

gtctc

>chr12_67948933_67949133
actgggaggtgtctcccagtcaggatacatgggggccagggaaccacttgaggagtcagtctgac
ccttagcagagcttgagcgctgtgctgggagatctgctctcttcttagccatcaggcagggacgt
ttaagtctgctgaagctgcacccacagccgccccttcccccaggtactctgtcccagggagatgg
gggtt

>chr2_61867296_61867496
gtgaaaaaccccagggtatataccccagacaatgcaactgcttcactattgtcattgtacccatc
ttacctcacttaagagttagtgtcacacttacacacacttaagagttgtgtaaaaacagaaggtg
ttgggcaccataaagacactaaatactgactggggagagaacacaggaagtataaggcttttctt
cctcc

>chr6_34106022_34106222
cccgagccccttggggttcagcaaaaagagccaggccaagagctccctcccaccttcctctcagc
tcccaaccttccctgtgttcccaccagcacttccttcatctccattcacctttgtctctgcaggg
cccacccagcccaggtgctcaggagcgctctagaattgtgaatggctgtgtggagtctggtttca
attcc

>chr7_148096667_148096867
tccaaggtcatactaagataaccatttattaaactctttggctgtttttttcctaaactgtaataa
actccccctctccaacattcaaaataatagtaaataattctctctgaaattaacacagtgttcct
attattgctttcacaacactaaaaagaaatagaactaacggagtcgaactgtggggcattctggc
aggca

>chr7_97000464_97000664
agtggtcagggaaggtgacatttgagtagagacttgattgaaaggtaaggcaagtcctgtgaaac
tctggggctaagagtgtttcatgcagagaaaaggaaatgtaaagacctcgagcacaactgagctt
tgcacatttagggggcagcaaggccagctagggggagtgatagagaaggggtagggtcagagaat
caagg

>chr5_492200_492400
agggaggtcagggccgggctgtgagggaggggagggaggtcagggccgggctgtgagggagggga
gggaggtgtggggactcgtgggggaggggagggatgtcggggcagggcctgttggggagggaggt
cagcggggactcgtggaggaagggaggaaggtgtggggactcacaggggaggggagggaggtcgg
ggtgg

>chr13_52001199_52001399
catttactcctgaaaaacagcaccaacagaacagatataatatacaatgttggataacatgactc
ttaagcaaaggatatcatacaggtggaattatacaactccataaaaagtaataggtcataccaac
accagccacaagccaaacaggaattatgtacacatatctctaatgtggaaattaatgacacattc
ataca

>chr6_37789422_37789622
gtaactcattgtaagacatatgtcctaaagaaagtcgctgccagtggcagctaatgctcaggatg
tatcaggtctctttcctggcaacacgtttatgtccttcggactgttaccatgaatggaatcttta
tcgaaagacttatatcaaggcttagaaactaaggaaatggaatagccttttacctttatttattt
attta

>chr4_183215006_183215206
actaattcgcactgaggtatgaatgaggcttaaccctcccaggggacatttgcattttaaaagag
gagcttgttaacccaaatgcctcgacaatgacaattagtggcatgtgtggggagatatattttgg
gaggttgggtctttcctgcgctccctgcgtcctaggccatcacaggcccgggtaaacttcaggat
ttcct

>chr20_61848555_61848755
cagtgacagcagggacggcaatggagtcccaagacggctccaagcctggacatccgcatcagtgg
ggtagcagacagcctctcacactgctgcaggaggaggtggaaactgcccccagcaggccggtcag
ccacagagaggactgctgctccaagttgctccaggtctccctggagccctgcctcgcctaaagct
gggga

>chr12_22850333_22850533

gtcttcttagaatctagcatcccagcagcttttgaagtcccaccagtgtatagtcttaaaaatgt
gtgtccataacattgaaacaagcattaaacgattttatctctctagatgagtttcagccaaagcc
tataattgtttttctcagactattgcatggaagtcaataaaacccaagccaagcattattgactt
ttgat

>chr2_184896155_184896355
taaaattagagtgcgttttaatattgtaaaaacccaacagaagttacatgggatataatattcta
agcctgagctcttaagtggtgtacgtctttcttcagtaaggtcaattggcagagaacaaaaatgc
agagctcaggagaatgaattcgtagctttttctcccatctggacatggagttacagctcagtcct
ttgct

>chr9_135358979_135359179
ggggccacactgtgaaatgggatgcatccagtttcatggtttcagacatgcttccccagctccca
ccatactgtcagaacagcgagactcagatggaaacggccttcactatccttcccagcccagggcc
cactccccaccccatgcccacctcagcacagtctcagaagagagcacagtctcctcaagactgag
ccacg

>chr8_126429618_126429818
gagtagctgggactacaggcgtgtgccaccacgccctgctaattttaattttttttgtagcaacg
ggggaggggggggtctcactatgttgcccaggttggtcttaaactcctggacttgagggatcctc
tcgcctcagcctcccaaagtgctgggattacagtatgagccactgcgtccggcccactggctccc
agtct

>chr17_25817073_25817273
gatggtaaggtgtcgggcaagtgtgtggactctgggatggattgcccaggtccattcctctctac
ctgttatcagcatcctggtgggaccttacagaaataacttctaagtctccgtttctgcatttgta
caagggggctgttgagaggatcaaatgggattatccttaaaaagttaagcatatagtaaatgtta
tgtaa

>chr12_45165333_45165533
atggctgtaattactatggctttaacgatttggccttggcattcctgcctttggattttatcctc
tctgtttaactccagcacactattttaatggtccaggcaggaaattaagcccctcctcccaatgc
tttgtttatgtagattcaactaaagaaaaggcttggtttcttctgacttctttcccagaacaaca
accat

>chr2_139406130_139406330
aaaaccagttttatttgtttctgggtcttgtcttataattgtaactgtaataggttcattgtttg
atgcacacagcaagtcaatatgtggagacaccgagttgcagtaaagagatttaatcatagggcca
ctgcatgaggagaggggaggaaacctcaagtccatctctctgaggaattttgggttagggattta
agggc

>chr13_100805599_100805799
gagtttagattagaggagattcatcctaaattgaagtacttgggaaagatccagtggtagatgtg
aaacttggagtccatcttctataataggtggtatctgaggacaataaaacaagtgtggatttaag
tttaaacaaagacttaaatgtattctttttaaaggattgtgaagtactcaatttctttcttacag
aagaa

>chr19_7865200_7865400
cacacagaccaagcggagaaaggaatttcttgaccctctttttgtgcctgtttgaagtcagccct
tattttttcccagtctttcaaatccatggagctgtgttccgggaaccaaggagaatattgttcta
ttagctgaaacagggacagaagattttgggtgctcacgtttaaccacagtggcacaataactgcc
gcacc

>chr2_15783349_15783549
ttttctgcctcaccaaccatggggaatgtgagctgggggggaaaagtctgttaaagctggtcatt
catttcagggggccaaggagcgattctgaaggagcaaagtctcctcatgctcttggccttttgat
ctactcaggctctgtggcaggaagacagggaggaatccctgatcacccacagccctccccgctgc
tgtta

>chr6_88641881_88642081
aggtagagaaattggggtctaaatcctaagactcatgctcccttctgaaaaaattgtatctgcaa
agatcagcagccaagtgaagaatactaaccatatttattaacatctcagcaagcttccaattaca
acaatactaccccacttgccctgtgaaaagtgtctctggttaatttgaaacctagcatgtggctt

aggct

>chr2_200848355_200848555
ccatctccttccaatctcccttcaacagtgctggattcaattaactgttgactttgttgattaaa
gtaattcatattcaaatttgaggaggtattgaataataccgttggtagctgtgttgtagaggagt
gtagaattccctaagaccttctggcatatctgaagaagggaagggttgaaataggagcagctctc
tctgc

>chr2_206148755_206148955
ttaaactatgcaattaaatcaagaaagcagattattctagtagctgacattaacgtttgaaaaga
gacaggattagctgtacttagtcacatataaactgcacaaagcgaacaaaggggaaaaaaattat
ttcaccaagcttcccttgaaagaaaatcgttccatcttgcacctctaatttttcagcttaattct
gttac

>chr2_208274755_208274955
gacttggcatggcaatggcgagaagtgaacagattcaagcaataatgaggaaacagaatccctgc
cttccttcaaccgtgggaatagaaatgatgcacagcttggggggaggaggagtgagacatttcat
tttttgcctaatattcttcaaacacaaattatcattccacattttcccttagaaaatcaccatct
cccat

>chr3_46422196_46422396
tgtgtgctaataaattcacaaaagccaacatattggagtcactcagggagttttacaaatagtga
ggttaaatccaacctcaaatagttctgattcgatctgcctgcattgctgccctgtggttccccac
tgtagaagctccccaggtgattctaagtgtagccaagtctgagaaatactgcctaaagcctgttg
gactg

>chr3_56155960_56156160
atggatcccacctacagctcagccccactccagggcagagacacactactgcacaactggcaact
gacaaaggacttcaccaagactattttggctttgccaaaatggatgaaaggtcacatagtaacat
agtctggttcttaaaaataagtttgtaatggcagaaacattggaaacaacctgtttatctatagg
ggaat

>chr4_13649102_13649302
tgaaataatttcctatatcctcttttaactcacttctaataggataacacatctatttttctca
ttctgaattctggatgattttattgttgagggaagacagtagatattgcatatctactttctttt
ttctgttttgtgtgtgtgtgtgtgtgtgtgtgtgtgtgtgtgtgtgtttacagttttatttaaacac
aaaac

>chr4_87370176_87370376
gctcaggcggtaatgcaagcaactgtaaatacagaggaggatcgctggctcgccacccccaccgc
accacccccacctcacgaccctgctcacctcctgctgtgcagcctagttcctgtaccagtctgtg
gccccaggggttggggacccgtgttctaactgactgtggtttgttccttgttttccaagtgtcat
tttta

>chr4_134017750_134017950
caggaaatgtccacacaaaaaagcaagaatagctaggaaaagtctatttctaacatcaataagag
gagaatcaggatgttggtaagcagtaagcaagttctgcaaaaacagttattgtaataataaatat
tcatgtgtacctgttccctcaggtagtatacgctcaccccacggtagacaaaactaagtcccatc
tagtt

>chr4_148547550_148547750
ttcactgtctcagtcataattttgcaaaggcagtttcactaataaatgaggaataaggtggaagt
tacaagccaaggtaaatggaggtagagtgatcagtgtagacatacaacataagaatgggtgcatt
gcctcctatgcagtacaggaccatttgtacaacgtcctgacaaattttgtctctatttacaccct
tgaaa

>chr6_84927681_84927881
ccaagtctaatggaacaagagacgtggcaaagaaaacagtaaccattggaggttaccagagaagc
agcagtattcacagagcaaagttaaatgtgaattacaactctgctttgaaggaattatataagat
taattggggggtataccaagacaggaaaatccaagaatatttccatgaggaagtaacatttctg
ctaag

>chr6_107424507_107424707

tcacaagctctgctcctcctgggtggacacccagcacccagatgcctagcatgccacagacattc
ctctcgcctacctgactataggctacgtaggcgctgccacaactacccaaccaccacagcaccca
cctcctaaaggctgctgtgagcattccctttgcctcctggactctggagaacttctcattctgac
attct

>chr8_38889243_38889443
gggcgctctgattttagaattttcagcttttctgctctggtttctccccatctttgtggtttta
tctacctttggtctttgatgctgatgacctacagatggggttttggtgtggatgtccttttttgtt
gatgttgatgctattcctttctgtttgttggttttccttctgacagtcaggtccctcagctgcag
atctg

>chr9_107696779_107696979
ctaagaacatgcacccaaggtggttggggtgcagcttggtttttagggagatatgagacttcaat
caaataagtttaagaaatacattggttcagttcaaaaaggcagggcaacttgaagtgggggcttc
taggttatagatagatttaaaatttttctggttgacaattggttgagtttatctaaagacctggg
atcaa

>chr10_34703394_34703594
acatcctcgggtcaaaaccctaaggtttctataaaagctgaatctactgaccacagttaacaaca
atctggctgggcgtgggctcacacctgtaatcccagcattttgggaggccaagatgggccaatca
cttgagcccaggagttcaagaccagcctgggcaacacagcgagacccagtctctataaaaaaaaa
aatta

>chr11_133004590_133004790
tgcaaaggagcccagcccacatcaagcgagatgagccaccatcctgctacgaccaccaggtggtg
ctgttctccctgcacccaaagtcattcatgccaggagggcgcagagagctccctgcaccccagct
acaaggcagcaggaaacaccagtaaggttccttggctcagagctgttgtgcataattacagccac
tttct

>chr12_45525333_45525533
ctaactggaaggcataattaggaatgacttcactacagtctttcagtaggtgggactctggctgg
tcttagagaatggcttcattgtggaaggcagcagatttctgcacagctgagtagaaacagtgcag
gagggaccagggcttccatcacagtcactctagtgacatggttggggaaggaggaagtcagagtc
tcctg

>chr12_116719017_116719217
aaagcttagacaaatgtagagagaagtaggactctcatacactgctggtgggaatataaattaac
accatagctttggatgtccgttcaactagcatcaaaatctataatgcacacagcctttgacccag
ctgttcgacttctagaagtttatccaacagagatacttgtacgattgtataaagacatgcagtgc
aggga

>chr17_55989201_55989401
ttcttaagtgattcctcattaaccaggaggcattcctcatgtcccaggtgaactgaatctgtttc
attctcaggcaccccagtgacattcacaaacagacttctaattattcactcgctcattcactggc
agtcaaaaaacacttactgagcacctactgtatgtcaggatgcgcaagataaaaacctcttttct
tcaca

>chr18_59470220_59470420
taaaaaacaaaccatgagctaatagttcaatcttcttatgtaaatatgagcacttaatttatagg
ctggttggttggtttgctttgggccagcctttgattatcacttcaaaccagaaacgaaaggagta
agtcaaaatagtgaattatcattcaaggccagtattcccccaactgtgttctgctagacatcaat
agaca

>chr19_38326960_38327160
aagaatatcttaacgcaatgtatctgcctgatattactgctacctactctacatcaaatccttct
ccttcctcttcccccctccttccatccgaatgaaattctgatttggcttagggcgatcatggaccc
agaaacttcagcaacatagagtactactatagtactccaaggctggcatggccctgtggctctct
gagct

>chr20_16349200_16349400
gctggtatctttataaaggtctgagaacttgtggtaaaaatatcccagtccattgtttccttctg
ttctgatatttaccagtgactccgatgagctcatttttttagcatccaactcttcctcaccttgc
tgctcactataaaactcagacgccagtacgctctgcggggcactgcaggatgctctctcctcatg

ggaaa

>chrx_153298806_153299006
atgcggagggaagagcaaggaaaaaggtgtggtccaggcagagccatacatccccttcctgccgg
tggagaagagcaccctacctattctagtgtgggaggcttccccgttcttttctggtgaccgaagc
agctccttctctacacatcactttggcccggtggggtctcgtcagaaatggccaccgtgaggccc
cacac

>chr1_19332213_19332413
tagtgttctcgtgatagtgaataagtctcacgagatctgatgattttataagaagaaacccctttt
ggcttggttttcattttgtcttgtctgctaccatgtaagacatgccttttgccttctgccatgat
tgtgaggcctccccagccacatagaactgtgagtccattaaacctctttttctttataaattacc
cagtc

>chr1_56840812_56841012
atctgggtaaaaccactaagtaatagaactgcacatgggttatttaaatccataatactcacttt
gagttagatgaacagaaaggagcttgccttctctgcttaattcttgcttcctcctcccccaaccc
cagcccatcctgtcaaaaaaaaaaaaaaagaaagaaagaaaaagaaaaagaaaagatactgcctg
ctcct

>chr1_64410012_64410212
aagaatcccagctggttaaatttagggtgcggaaagagatccaatcggcagataatccctggcca
tgggcccacagcattttcatttgattgtgcattggatacattttcattcattttaagcccttgcc
tttcagtattttgttccttggagacaggcaaactagtatcaatagctctcttagcctatgtcaaa
tggtt

>chr1_247550977_247551177
tagaggaattgcagtggagaaagtgtaattcacgcagagccagctgtgcaggggaacggagtttc
gttgttactcaaatcagtctccccaaacatttgggcagcagagtttttaaggacagcttggtggg
tgtgaggaagccagtgagccaggagtgctgattggtcagagattaaatcatagggaattgaagct
gtcct

>chr2_106886968_106887168
ggccaggagactctcaggaatgtgagtcctgctaacccttaaagggacacttacagagcaagagt
agcaacagtgagaagagggtggggtgtcccgtcacagcccttagcacatgtccgcagagcaaagc
taggtggctcccagccctcggggcagcaacacaggcccgcacctccactgccacagctatgctct
cagct

>chr2_240362063_240362263
aaggagctggtggtcagaattcctcagcaggtgaacagaaagccaggaaaacatcttcccaggga
ccttgcccagccccaggtgcagctgttgttctggaaaattactgcactgcatttcagaagcatct
ctaatccccaaatctgtcatcgccttgcccaccctggcccagcttttgtaaaagggcaaagagaa
gagca

>chr3_116868310_116868510
ctaacactaattcagtggtaattctccgcattgcacaaatgagaaagctgaagctcagagaaatt
catttacttgcccaaatttctactttctacttccctatgaatctgtaaccagaaaaaatgaatag
tttgtacttattttattctcattgtccatagtttaaaaaaaaaaatgtgtaatctaccagctgcc
ctgag

>chr4_149036550_149036750
aatcaactagaattttatttctaggtgataaagacctaatgaaaattcaccttgggatattaaca
ttctgattttgaaactgctcttactttcacttcttaattacctatttatttgctaatttcccata
actggtgtaaataattatttactttggtcatcagcacttggagacttggagatggtgctgagtat
tgtgt

>chr4_150105350_150105550
ctgattttatagctgcttgctcagaagcactggtgacgactaagacttgcaagtggcatctgaag
tggggggcagtattgtggagcctgagtccttaactcaggatttgatgctatctccaggtagatag
tgttagaagtgagttaaattataggacaccggccgggcgtggtggctcatgcctgtaatcccagc
acttt

>chr5_167155022_167155222

ctggccccaagtaacatgccaggtaacagtttcaagcctgggttacttgaaaagaaggctgagta
ggttggtgggaactgggaccagtcttatgatggaggaatatgctgttgctattcttgaaattctt
ttcctccaccccacttcatcccctaccccttgttgttggcaagatttatcttcaggtatccaggg
aagat

>chr7_100805080_100805280
tagctgggcataatggtgcacacttgtagtcccagctactcaggaggttggggtaggaggatcac
ttgagcccaggaagtcgaggttgcagtgagctgtgattgcaccactgcactccagcctgggcaac
atagtgagaccctgtctcaaaaaaaaaaaaaaaaaaaaagttaagaagtggctttagccagcatg
tctct

>chr7_146110867_146111067
aagggggactttcaaaataagcctttaaaaggaaagcacttttttacctatatttttcttttcttt
tttttctttttaaaagttgagcttataagatcatgcctggtgaagctgtgttcatccactcctc
tcagcattagtggaagaattagagtattaacgaccttatagaccaggtattttgtagcctccttt
acagt

>chr9_27671600_27671800
tcttgggtaaatgagctgtttcttgggacattataaatcatatttggaatcaagtttattttcaa
gctgcttttgaagtctcgatgctgtgctaactgaacagaaaatgttaaatgtttgagggagtagt
gcaagctctcaaatgctaacggcatgagctggacttaaaaaaacaaatgggatttataccctctg
aggca

>chr11_44978024_44978224
taaggcctgccactcctggaccacccccaccccagtgaagccatacagtttggggacagtaagtc
acctgcggaccgacaggaacatctgacaaatagattctagtttataaatctctttagagtgtatg
agtctcaaagcctacttgggggcaggaaaatgttagaaacacaaattctcatttccacctcagac
aactg

>chr11_88162152_88162352
tatttgcaactgaagcaactgtgctttatgtcagataagataaagattaaaaaggagaaaaagtg
acatgttctcacagagctgttctaaatgtggtctgaagaacaacagcaatagcagtactgccttg
gaactggatagaaaagaaaattcttgggccccatctgagacctgctgaatctctgcgggggcaag
ctagg

>chr14_57325447_57325647
gcacgaagccctgtgcttgttactctatgtgcctattgcatagtgaccaagctgtcggggcgtgg
gagaaaggtatgaggtgaggctacagagttttactggccttgaagatcatggtaaggggttgagg
ttatattctttttcttccccgcaaaagacaccattgttccaatttgttattcagtatagtcaga
gttag

>chr14_73401447_73401647
tctgtctcccatattaaacaatcggtgcctccaagccaggtagcacagcttattatctctgccca
ctccccagtgcctcacatggagtgggcgctttgtaaatgtgtgtggagtagtaagtggaaataac
ccagagcagaaagaagctacctgggacttaaatgtaaagtagccacaggttttgttttgctttgc
ttcag

>chr18_52882602_52882802
atgctaatgggggcaccaaatgaaatatttctcctggaaagttccctgggcaagttgggaagaga
atctgcttttcctcctggctatgccaagtgctgtgtggcctccaggaattcagaaagagaaactt
ccacatcgtgttaggtgctggcagagaggataaatgaacacatcttcattaattcgtttctgcct
attgg

>chr19_7211800_7212000
accacaggcaagagagggaacgaaaacacttcttctgccaaatagagctttggagggaatatcggtt
tgtctggggcaggactgggtcacgggctacacgccttctccttgtattctcacgaattcaacacc
tggtaatgccggggccgtttcccaagtcacagactcagactctggggtggataattcccccatca
ctgtt

>chr20_4031000_4031200
ttctcttccaactcactctgctcctgatgtgattctcaacacagcagccagattaatcctttaga
ggctgagaccatgttcctcctctgctggaaaccctccaatgttccaacagctcccagcccactct
aagtataagccacagtccttacaatgtctgtgaggccctaggtgatctccatcatacttccctcc

tatca

>chr22_42813856_42814056
cggtgtctgggagggcccccgccccgtccccgggcttggctgtttgttttgagtcttcaactcct
ctcctgtgcctccccacccccacagaactgcaagcgcagacatctcatctgggcagcgccgtgga
tccctgcttagtctgcccaccctgctttacaggagactgtgttttggggggtgacacaccctcca
tccct

>chr1_81717612_81717812
aagaggatgacctcccctgatagaggaagaccattattcctgtcaagagtagctttgctcccctg
caagaacctgcaaacagctcaaaaaaaaaaaaaaaaaaaaaaaacccaaaaccaataaaagtctat
aatgtaaaggactccaggttaagtaatttcatttctcttgtctttgtctaaaattgtaaaaatta
cctga

>chr1_181561577_181561777
cctctgcttgtcctgcctcactacagctcagctccctcctccagtgaccggtgggggtgggggg
tggggaaacaatgaaaagtaggggaaaggtaatttatttcagtgtttacagagggtttgcgtgta
attagcagtttatttctgtgacacagctggcctcctcagcttggaacctgactttgaaatgctct
taaag

>chr2_15268349_15268549
gcctttaattatccttttaatgctaatatccttacttttgcagcagaattattaatgtgattgac
agcgggggactactccagacctactggggctgttacatgatatggaatagatgcaccataatacc
tttccaagatctgacaaattctaaattccaaagcacatctgttctccagggttttggctaaggga
ttggg

>chr2_118309530_118309730
atcttaccttggtccctccctctaagaggcatgtaaatggaacagggactgcaaaggacttctct
ttgttcttagctctcccctgagggcaagcacatttttgcaacacaggttaggctctcttttgcct
ttccaggaatgtgcacatacacgtgcatatacacacacatacactcacacatttgtgactttcta
tttac

>chr4_38123605_38123805
tcaagcacattcctgcattgtgcacccaaatgatctcccgatttaagacccccctgtgtctcacag
aagcttctggggctgaactttctccggccttggagggttggacgctttgaatgggaggagtggtg
gtgagtggagcatctctggcagcaggcatttgggagtctctggcaggaatcaatcagcgtagtct
ccaaa

>chr4_102248977_102249177
acatttcaataaatatatattaacaaaatgagaactgtcaacaaaacactctagttgtatgaacg
ttactcaaatttttcatctacatggtactggacacctttcataaacaacaaccaaaaaaaatata
tttgaagttcagggtccacaaaaatatcaccagggaaaaaaagaagaaaaagaaaatatgaacct
aagct

>chr4_123746750_123746950
tcttggagacttaagaacaaatgcacttatgcatggccttttgaaacctaatgtttctacaagga
ggcacgtccatacttgcaactccttctagaagcttccccaaatctcctgcctccccacgctgagt
tatccgatgtctgaaatgtcacagcacttagtcttactcttctatggcctactttctactgctat
ttgtg

>chr5_54987643_54987843
aaagtaggcctgcatagtttgaaaaagccccccaaatgacttgacatatattattcccctataac
aacaagctccttcctccacctaagtttagttagaccccagttccactaaaaggataagcgaacca
tcatactttcagtccaaagcagttattaaaattatgtgatttttattgccacctagtggaagaga
tagaa

>chr8_10195190_10195390
gaaccttatactcctcctctctcggcagctggtgcttccctgttcttcctcagccatgctggggc
tcactgcagcctcctctgctcaactcgcctgggtgcagccatcttcaagaaaacattattaaagc
aatattattagtatgcagcaaggtgccttctttctgctaaatcagtttgtaaagtggtcctcaca
aatgc

>chr9_77102580_77102780

gtagctgcagttagctgtatagtttttatttcaaggaaagaaatgtggcgataaactccaaggat
gagctctcaccaaattgtagaatctcaagggaaaacacagattcccatctactcctttaagcaat
agaaaagataaaaacgttttgtactaagaaatgatctgattttggtgagtgtgttgaaaagaatg
tagta

>chr12_52685533_52685733
aaagtgtcttgccagcagggcattccttcatctgcaggcattggggagggaaggggaaggcagag
gagaggacgagaaggaggagtagagcctggcgaggggaagagaagggaggccaggtgctgggaga
aactgggaggaggagacctgtgtgttaagtgtgatgtggggagaggggtttcaggaaaggctgag
gcatt

>chr12_111471017_111471217
agataataacaacagaaatccctattccaaaggacctgaggccccgccctcggcccccgcccgta
aagaaccctgagctactactcagacgaagccccctccccgccaataataataatataatggcgc
ggaaactgtgggcggtggtggtgacatttcccacgactcagtggcgcccccgggcggctcccacc
ctccc

>chr13_54784999_54785199
ttgtggttggcaacactttgcacatattttcacatattgttggtaagagaatggaatgtgcccca
gggtgactttgctggggaacaccactgaaagcttgcacctgatttcttctaacctttccctatgt
gccttttccctttgctgatttgaaacagttccctttcattgtatacaccataatcataagtacaa
caatt

>chr14_99610047_99610247
cttcaccatgttggccatggttggccaggctagtctcgaactcctgacctcgggtgatctgccta
ccttggcctcccaaagtgctgggattataggcatgagccaccgtgcctagccaaaaatttgcatc
tctaacaagcttcaagctgtggccttggccaggggatcaggcattcagactcactaacctgaatg
ttcct

>chr15_49683508_49683708
caagcatgaaaataagggaaaaggcttgagtcccttcaagagaaattccaggcatatagctagcc
tcgagaagtaaatcagcaactcaataagcaagaaggacaagatgtttggttccctatagaaacta
aagatgacacatgtccctgagttgtttttcagaaacccagacccccatcagatggaaaaggccaa
ccaca

>chr16_52535899_52536099
tccctgcctccgcaagtaaaacttgagccatgccaatgagggaaaaagtggccgcgatttcccag
gtggcttagtaatacacagttttctgacacactgcaactccattctttcctaaaagttccttcca
agcctgtaggcactgtaagacaaacagctaagcacttcctgagtgcctactatttgccaggctgt
atgca

>chr18_35105602_35105802
ttagggttccttggtgactggctgatgggtctacagtatttcctcaaacttccatgacaagagaa
tgggattcatgaacatttacattgatcagacattatgctgaagggaggaaatggtactttctccc
tttaaagaaggatcctgcattttacaaaatcgaaaataattattaaaaacccctttcacaagatc
ccttg

>chr18_43185602_43185802
aatattaaagatttggttattaattaaacttgaccaaggttaaacagctggtaggcagcagagct
aaatttagatcccaggtctcctcactcccaatgcaatgatttttcttctgatttgccatgtcaga
gaacacctggtccctccattttatcctctcccagaaagataggttatcaggagcctagcatggaa
cagca

>chr19_5689800_5690000
tgcccaccaccacgcctggctaatttttttgtatttttagtagagacggggtttcaccgtattagg
ctggtctcgagctcctgacctcgtgatccacatgcctcgacctcccaaagtgctgagattacagg
cgtgagccaccgcgcccggcctaatgggcgttttaacagttcatacagtttggtattattattcc
cgctc

>chr20_57987605_57987805
ctcatcagtaaacaccagcaagccaccagctgctaccaccgtgtcctgcaagagtccgaccccaa
attcctctccctggagcccctccttcctgaagcccctttcagagggatagaatgttttctttacc
agttagcactcaggagtaaagtgcctgggaacacaaatgaaagggttactctctttgcagcattt

tgctg

>chr1_53056212_53056412
gaatcatacatcagttcattcctcaaattctcactgcgctccaaccaagtaccaggccccatact
agataccggggacttagagatgaataaaaccctacctcattcctcaaagaactcacagcatagca
ggatggacagatttcttcattcatccaagaaatatgtattgagtgcccatcaatagtacaatggt
taaac

>chr2_36523696_36523896
tcagggagatggtcaaatagtgttttcagtagtcttttaacagtcattacattactcacgttagc
gaagtgcctcggtttaaaataaatagctgcacaacctgatcataaagaagtactatatgatcatt
tgcagaggaggcaatctggtgagtacacctttcactaatgtttcaggcaacaaattttacctaaa
tacaa

>chr3_132911110_132911310
gtattgaaactagtttactcctcttattttggacacattgagtaggagatttgagcagtactttt
ctgaggagttgtccagcaggcatgtgggaagctcagggaggtgtccgtagttagaggcatcacta
atgcagaggtaactaagatttgagagttttgaagtaattgcctagggagagtgtttagaggaaga
aaaga

>chr3_148721510_148721710
ccatgctgctggaacccgggccacagtttgaggagcactattataggggggaacattcctttggct
gagatgctggggctcatgtctaaaccagaattaactcactgccctgttatccacagtagacaagc
cagctgccagcaaactgtggaagagcacgcaaagtacacaagcattgaacaaatgcacagattta
aaaag

>chr4_5786899_5787099
cccattcttccttcttagaacgcatgggaggcctggaggtgcatcagcctttctctgccctagag
gcagcctgcgtgagcctggtgggctccagatggtggagcagtctcagacagccctgagcccctgg
cagtgctgttaagccattaccgcagccctgaaccgcctgcccctacatgcttattgtcagaaaca
agccc

>chr5_155351222_155351422
atctagtcatagcaaacctaggaggaaatactcatcactaatgcacaaaagaagacagaacaagg
aggaggaggacctgaatgggggacaaaggcacctgaattcatcaaatagtttattgtcagcacag
aggaagggatgtcttgctatagcaggagaactttcatcacaatggatcatcagcctcttggcacc
tgttt

>chr5_174973994_174974194
gtagagacggggtttcaccgcgttagccaggatggtctcgatctcctgacctcgtgatccacccg
cctcggcctcccaaagtgctgggattacaggcgtaagccaccgcacccggccccaggtgtttata
tctattgggagacggcctttccctggcgccggctgccaccaagtattattttagagacacagttc
acaac

>chr8_34895858_34896058
atccagctatcatgctccaaaatatttgcccacactaaaacctgcactcaaatgtgtatagcagc
tttattcataatcaccaaacacaggaagcaaccaaaatgtgctccaatatatgaatagataagca
agatgtaatacatttaaacaattgaatgtttttcagcattaaaaggaaacaagctatcgaaccac
aagaa

>chr8_56044446_56044646
tgttacagtgagcatgctgtttacacatggcctgtgcacagtcttaatgagagccattctgggga
taaaatgatcagatgtctgccctagaggagcagtttcatatggcaggtgtagcgcagagcccgaa
gggtgtggcactaccagatcattggtcaggccatgggaatgtccaggtaaccagcagtcagcagt
catta

>chr9_109521179_109521379
aggcaagtcatagaatacaaggaaatacttactaatcataaggaactgacattcacaatacatga
agaactcttaaactcaacaacaacaaaaaggcaaacaacctgattttaaaataggcaaagggctt
gaatcaacatttctcccaagaagatatacacatggccaataagcacatgaaaaaaatgctcagta
ccatt

>chr9_138993979_138994179

ccagagggaaagtggccagcagggctgcaggtgaggccctccgtccctctgggctacgtgggcgg
gccggcacccctgactcccaggagtgaccaggaaggcatttggcccctttcagaacaagtcactg
gcccctgctcatctggggctcctgttctagtagcaggggtggcaggctggttaaaaaagaaatcc
aggtc

>chr10_88626620_88626820
cagcaagttatttatctccttgaacctatcttttctctgtaagtaggaatgattttacttactgt
aaggatcacagagatgggataatgcacgtaaaactaatttgtaagttataaaaatgacagtgaca
atgggatgctaagcaggtaccattaaataccaccacatatctgagtttctctgatttaatctgca
caaaa

>chr12_40500533_40500733
tccactcatgtaacgggtaattcccagaaatagaaagaaaactcaagaccctgatttgtgattcg
gcaggcagtctccatttccccgccccaacatgctaaccaaaaggtaggatagcttctctcctgag
aatccccactgttgccctccatgaggcactgttttaacagcctcatttacttgaaacaaagcaaa
gataa

>chr12_113950017_113950217
cagagctaggggtggggcttgaatggtggggactctgcctggggaggaggcaagacccttgaatg
caggaagttcactctcactttcccctctggctggtgaccaggctttcttctctctgggcctcagt
ttccctatctgcaagtagggaggcagtttaaatgcctgtaaggtggagagggagggagttactgt
gactt

>chr13_108867199_108867399
ctgacgcctcccagacccccccacctgacgcccctcaggccccccgcctgacgtcccagacaccc
ccacgaccggatccccagacccccaatctgacgccccacttcccctacttgactccccacacat
cccccgcctgacgacccccccgacccccgcctgacgtcccagacaccacccatctgatcccccaga
ccccc

>chr13_115070298_115070498
tcgcttccgagcgcgagagtgtggcggaaacaggaggatctgcaaggcagaaggttcggggactg
gtcctgagaagagggaagaggcagagtgagtcactgcacgcacctggcctccatggacgagcaag
ggcatcccagaaacgtgtaaatgaccccgagtgtgactgggaaggagaacttattccttaccagg
aaact

>chr14_102411647_102411847
cttgcaaatacgaatcccgcctctatccctttgagatgtatatctatctccttcagctcaggagt
gtctttctttaggacctgaaagccgtccttttgaaatgtaatcatgaggaaggttagactcctcc
agtctctgtgggaagacagaatcctaacttctataattgacagctgggctaatcacatgacattg
accaa

>chr20_30511939_30512139
cattcctctcacctccaccccgggcctggccaccatcctcttcctcctagacaaccacgcccgcc
ccctcactggtgcctgccgctgctctgcccacttcactccaccccaacgtggcagccagagagga
gacacccttccatggcttcccatggattgtgagacaaaccccaaaactgcatgggacctgacct
ctgcc

>chr20_38724786_38724986
cctggactacagctatatcttgttgccacccttcttcccaatccaaagcctcctttgcatcctcc
tcttgtatcccccaccttaacccacaagtataagatacctctactccctccttggcgacccttac
catctcattaaaacctaatcacctttaccccactcaacaccaatatcccatcccacagtgcgctt
taaaa

>chr1_82130812_82131012
tctgcatggcattttcaaaaaaatcttcaaagacagatggataaactgattatttagaattagta
gagtttgccaagtaagcatgtttatgaagaccatttgaggtagagaacatgcaaagatacattat
acaaacagcgtaagtacctggaaatcacagaggaagtgtcctgatatcagttgcattttccttta
aaaat

>chr1_110031277_110031477
ttccagattcccccatcccaaaggagggttttggccccatgggggaatgaaacaaagacacatga
acacagctgcaaatatgcccacctctctccctgccccccaacttgcccgggccaaagcaccctgc
cgtccctcaccccttctcagttcatacctactccccagctctccactctgctgtattctagatcc

actca

>chr1_164056176_164056376
tctttccctgatatctctgggcatctgtgaggaagaatttcatgttacacagactcagtctgatt
ttcatccataacctcataggacagattggcacctaactggtgctcagctcctgcaatcacagcct
ctcattgcataatgagttgctgttgctcttcacattctgttttacccaaaatgataacataattc
tgctt

>chr1_248056977_248057177
gttctcagatctccagctgcatgctgggagaaccactgctctcttcaaagctgtcagacagggac
atttaagtctgcagaggttactgctgtctttttgtttgtctgtgccctgcccccagaggtggagc
ctacagaggcaggcaggcctccttgagctgtggtgggctccacccagttcgagcttccaggctgc
tttgt

>chr2_80109692_80109892
caggggaaaagcatggcctggagctatagaaatggatgtcacccttacccctcccaggaagctta
gtgtgttaggcagttgtacctcccagggctggctgctgcccctccgccaaggagcataaatggct
tagacagcaggcagccacagctgtggtgctggtcactactccccctgggagcttggcaggcttaa
gcagg

>chr3_65376360_65376560
atggttaaaaaaaattaccagtatttttaaaaatcaagagaccacttaaaaagcatttaatttct
tctcattagcccaaaatcccagtcaaacatgtttaagttctgttgcatatcagatttgtgctcag
agcacatcacaatctccactcggagaattaatgagacagcagctcttatctaccattccttttaa
ctgaa

>chr4_140774150_140774350
tttataaataggaacacacagtacacactttactactaccctcttgtgttacagacaagacctga
cattacagaatgactaggtgaccagaaccagaaagtgattcagttacagaaccacagtcagccct
agtatccacacattccagggaactctacacttctcatttctgcttcccttatacatacagactaa
ggaca

>chr6_38443822_38444022
ccatttggcaggctggaaaaaattaatttggggttgccattgtgtttttcactctgtataattta
accgttaaatatcaaaggttcactgaagtaaatgaaagtagtttttaagcttaacggttagacac
tttaaaaattgttttcaaggcagcacagcacaaaggccagaatcatctgatcacaaattcctccc
aggga

>chr6_41540422_41540622
ccagccagaggcagacaaaggaagtttggagtttgtttgttttggctgcccctcaccacaatcca
aattaaaccagaatttgctagtacctcctccatcctgcccagccagcgcttggtttctcttccct
gtgcagagcccagtttcaggtctggtacattcaggttctgcttttgccactgtgtcccgttgggc
aaatc

>chr7_89778864_89779064
tcctttttctggcctagagcataatacttcagccctgggggccccactcacctggtattctatgt
atatggtgccccctgcagcagccctgggctcattacaagtaacactgattcttaggtgttctatg
ccttttaccaatctctttagaggaagcagatgaacatttagaattttaacagggaatgtttacc
tgagg

>chr10_1563600_1563800
ccaaaaataacattctaaggccccctaagcttctgaatgcacttcctccccagccagggctcttt
taaaatttaacctgagagactgtttgaggcaatgttgggaagtcggggtcagacatgcctcatga
tgcctctccggcattaacagcaacacagaccttaagtctgataagaaacatttacagcctcttct
ctctg

>chr12_64453733_64453933
aagtcctttttctgcttggggaaatagcacccccaaaagacatggttgcaggcccatttacttg
atgattgttttagggcattgcttcttagacttttaatgtgcatatgaatcacctgtggatccttt
ttaatcttcagattctgaaataggtcggggtggaactcaggatcctgcatttctttttttttctt
tttcc

>chr12_81243469_81243669

ctttctccacccccaccattcctcaaacatattgaactctccaagttacctaggtctctgcaca
tgccgtttactctgctaggaatgcccttccacctggcagactcttattcatccaaatttaatatg
atgataaaaggtcaaattcaaatattgtctcctctattaaatgttttctgcctccctcaagcaga
gtgag

>chr13_53480999_53481199
ttctcatccagcttaaaaagagttaagtgagaaaagcaaggtgcaggaaagcgtgtataagaaga
tctcagttttgtaaaacagaccatgacaagctgctcaaactccgcatgtgtacgtaaagggctat
actggattacacacagatgaaaatctgggacacaggctgagttcttaaaacttactgccgggagc
ggtgg

>chr13_113273999_113274199
aaaagaggcccacgtgtggcaggagaacagggaatgagggtagccaagggataaggcagaagcaa
aagaacagcaggtgcagccagttctgggcaggatcaggcagcactcaggccacagcctcactcct
gaaataacgagactgtctccacttcagcctctgattggccatgagctgcctccacttcagcctct
gattg

>chr16_52295899_52296099
tgaggtgctgcctgcaaggacacagaagggtcagaagaaacaggtataaaataagaaccaacttc
attggcccttccctagcacaacaatcatgtcagtagactggaccagtgattccaacacaatggga
aaggttgggattaagaagcgttctgttcccatttagttcttttcctgtgtctctcagcctcagtt
gttcg

>chr18_37227602_37227802
acaggttgtttgttctaatcctatggataagtctttccctcattttagaatgttgcttcacatta
atgtgttgaccagtacttagctgaatacttgagtgcctcttgtcttagtccatttgtgttgctgt
aaagaaatgcttcaggctaggtaatttactaagagcaaggtttatttggtttacaattctgcagg
cagta

>chr18_46509602_46509802
tccatggcaggaaaaacatctgccaaagacattcccttaagatgtcaccataaaaaggcttaagg
tattagttctctctagggcattttcagacagttcttggggaatagctgtttgtaaaaagagctca
ccatttactggccttttttttttttttttttgatcacaccttctaaagtataagctgttggtaga
aaagc

>chr22_18808600_18808800
gcttgaaagctctatctgtgcctgcacttcttggtccaaaaggcgcaggggggatgggggagaggc
accggggtcatggtgcaggctcagctgttgagcctcattccccaaacacaatgtgacaggtatca
atagttctggagttgatcatttcacagaaaggaaactgctgtcaacctgcccaaggccacacagc
tagtc

>chrx_104878144_104878344
aaatcaattaatcatattgactgactttcctagttcaaaaagagaaagataagtgatagtatctg
aaaagagtacaaaaaggaaaggtacattttcccattctaataatcaggcctacaattgctctcat
taactctaagactaagccccctgcccacttacctccagtcaccacaatccctaagtaaacaattt
gccat

>chr12_105051670_105051870
agcctggacccgaataaagacacgctttatttccccttcagttgacggaggggggattttgaaggt
agcaggggaaacagagtgagcagtgttcagggacgcagcccaccttcctgggtagttttccactg
caacagacccgccgaggccagccagccaaggatactcagagttttacaagcatgctgtgtggact
ggggc

>chr2_67556696_67556896
gataattgaaaaatgaaatggagaacctcttaaaatggcaatgtatttggaatatccagggagtt
cttgaaatgtgaattctaaggaaatgggtagtacttaagaagaaaagattaactcaaactcacta
tttcaagtgataaaactgcaagtgaatcacaaagggatggagctgccaatgcctgcagaggaaaa
ccaaa

>chr15_67841546_67841746
taggtgttctatgcttattcaattaagctgtctcagtatactaattttcatgtgttttcaacttt
atagatttatttaagcattgatgtcagattatatacccttttttagatttttgttgtaggctagat
taactttatttcggtgtcattaagtatgttttctgttgcttaagagtaaaggttgataagaactg

tttgt

>chr12_131433047_131433247
caaggcagcgctcttctttctttcgcctattaaactttctgctcttaaacctactccttgtgtgt
ccatgtctttgatttccttagcaggagacaacaaaccccggggatttccccagacaaacgatgct
gcttcattgggactaacttacgcgagtgtctcgttcttggttctctgagagcagagcccagtgcc
aacac

>chr3_193902106_193902306
agagtgagggaagtctatctccctgacaattccctaaagtgatttattgtggaggtcacaatagt
ccctgtttggacctgttacagtttgctaagctccactaagcttaggtagtaggagatccttcatt
ggaacacttgtgttgaagggaggaggaggaggagaggaagaagccgccactgctttgtttttagc
tctag

>chr6_6169801_6170001
aaggttggtcatcctagactagtagttgtcaataggaggcaatgttatccccaggggacatttgg
caatgtctggagaactttttgattgtcacaactggggtggagggtgggttactactggaatctgg
tggataggggccaaaaatgcagttaaacatcctacaatgcacaggatagtcccacaacaaagtat
ccagc

>chr7_74899664_74899864
tatgtcacacctcaagcagatttgcagtcccttgacagcagcctcatctggcacataaaggacct
ctgcctctctgcatggcttcccagggccccggagaacagtgaggctgccttggcctccacctgct
gctccagagggcagcacagccccggactccatctccccagcccctgtgggaaggggggacgttcct
ggagt

>chr12_8680133_8680333
ccctggctccgcaggggaaaagcacagcctgaagctacagaaatgggtgcttcccttccgccgcc
cagggagctttgcatgttaagcagctgtgagtcccagtgctggctactgcccctccctcaaagag
cgcaaacggcttagacagcaggcagccacagccagtgctggtcgccctccgcccgcgctcccccca
ccccc

>chr8_145690992_145691192
tcgggaggagctcggggaccagaaaacgctctgtttgggtgggtgcgtgcgtgggcatagtggtt
tggaaggagcgtgatgaggggagaagggccggggcggggctgcgaggggcggggtctgggcgggg
ctgcgaggggcggggggtctgggcggggctgagggagataaggtttaggcggggctgagagcggc
gggtc

>chr1_113067277_113067477
ggggttgggcatctttgaagcaatgttggataacaagaaagagatgcttccttttcactctttgc
cctccctgtcagcctgagcacaaccatgaggttacacacacacacacagaggtgtacatatacag
acacatagagaacttctctcaggctgcataggagttctgctcatcctcctctccccaacaattaa
aaaaa

>chr14_92803247_92803447
gcatcacctcccgtacctggaggaacgcttgctagcaaccactgtgtcttgctcattggctgtcc
aaggggaaagagaaaataaacacctcctaagattaccaggtacaatagtaagtaattaggctttg
gggatttatctaggagccagtgctcaactagggctagggctggatcagattacttggattttttt
tttta

>chr12_119712417_119712617
gaaagagcacactgtaccacacgcccactggggcttcgggagctataaacattcaaccctagatg
ctgctgtggggtcggagcccgcgctccccacgacctgctcatctgcatgctccctctaggggttt
aagctgtggggcactgaagaagagagccacaccccccatcgcacaccccgagaggggggataaggga
atttt

>chr1_65862012_65862212
tctgctcaaaatgcctccccattttactcagcatgaaaaccaaagtccttgaaatggcctgtgta
gcctctgtatgctctagcatctcagtgcccgtgacctcatttcccattctttcccactttactct
ctgcattccagctgtactggcctctttgctgttgcactggctgtttcctctgcctcatactcttc
cccag

>chr8_17770720_17770920

taattataggttggatggtgccttcctgtccatctggtactaaacagtgtaaaattgtaacactc
tgataaacttcagatgccatgcctattccaattaaacccatagaagctctctgtttgggccatgt
ttttggccactgatccagggctgtaacagaaatattagcgcctgtatcgacaagtccttcaaaga
gtttt

>chr11_113755190_113755390
cccaagagacggaggttgcagtgagccaagattgcatcattgcattccagcctgggaaacagagc
gagactcctggggggtggcactgcagctgagttgacaaagatagggaaagctgaggggaaaaatgg
gagttttagcactgctaagtttagcttaaagctgcctccttacatactttaagttcagcccaaag
gtttc

>chr17_75966005_75966205
actagaatgctttagttaatggggtaagctagaagtaagctgcctttgataactcatgtaagagc
agcatatgaatggatgataactgtctttcttccatttaattcagctacttcctattcaatctcaa
atctcagttggaaagcaatttccctcacttgacccatttttcttctgcattatatctttcttctg
gttct

>chr15_68585746_68585946
acactgcttatttataaaatgagaataatgattatatgtacatattcagacatcaaaatttaatg
acattctattttgagaagggaagacaatgctgaagaaagtaaaactgttgattgaaatgctgaca
gggtggagaacgaatttgaagacagtggaataaactatgaactatgctaatggtaattagatttt
ttgtt

>chr3_75683510_75683710
ctgactggggcttctgcctggggttttgcaaagagctacttatgaatatagtctctccagattcc
ttgtttcaaaggaagtgagcatgagctagcaagtgtagcaaccccacagctgataaacaactttg
tcttggttttaaaccatcacatcttcatttcacattggaataaagtaagtgaaacctgctacccc
agcct

>chr9_72437380_72437580
aaactacactgggaaacagaacatcctcttttagcctcatatagggcttacctcatagctctcaa
gcaaaacacttagctcttctgtgactctgttaccccacagagatttgagtcataccataaatcac
cactagaagttattgttaaagagaaagacttttgcaaagggacagtgcttctgctctctgctgc
accac

>chr1_4131140_4131340
ctggattttgatgaccttgatagttgtttggcatactggccagatattttgtagaatttccgtca
atgagggtttgtctgatgtttttctcatgattagactggcgctatgtagttttggaaggaagact
gcaggggtgaagtgcccttctcattgcattgcatccaggatgcctattctcaacatgcctcatta
ctact

>chr16_22627899_22628099
ctataaaatggggactaaagtcctgttgagacatacaacgaatggtaacagagtaagagtcattc
ccctgagcctagtggcagtcatggcttatgctggaattgagacacacaaaggagtccagacacat
gatccaaggtcactttaaggagggccccaatcccatgctcctgtttctaaatgccaattagtgaa
atttc

>chr4_25216902_25217102
tccaggtgcatgatacaagatgtcagtggatctaccattctggggtctgaaggatggtggccctc
ttctcacagctccacttgacagtgccctaatggggactctgtgggggctccaacctcacatttcc
cctctgcattgccctaggagaggctctccgtgagagctccattcctgcagcagacttttgcctag
acatt

>chr6_106901707_106901907
gaggccagttggtatacctgggagaggcggaggcttgaagccgcagatgaactgggatcccagtg
tagggatggagccggagcagggagaagaagggcaggaacttgagagagcaaggagaggcagagtg
tggttcaggcgaccctgtctctaagtgtcactggaatggggtcagccaggcgctgtctctactct
ctact

>chr22_48583336_48583536
gaggcctgtctccttggcagtgctattttagtccttgtaaacacagccaccagggaacaaaggcc
atcagagcgctcagcctggccccacggaaacctctccaggaggccattctgcctgctcactccaa
gggctctgccttcaggtctgccgcctgtgccgccccctgcccgctcctcctgtgcttgaaatctc

tgttc

>chr17_9124875_9125075
tggtcttctgtgtctgcatcatactgcggggatttgacccgagtccacgtggctcaaagggaagc
tctctgggcatcagacaagggctgggagctccccggtgcatttccagtgttccgccaggctgtag
cggggccgggagtctgcgctgtaaacggctcaccactcattcccatgcaccggaagttccgctgc
ccaga

>chr2_8364749_8364949
gtgtcactgtgtgacacctgtgtgagtgtctaaaccccgccatccctgctacgcctgccttcagt
tctctgttccctctccggatggtaagctctgtgagggcaggtgtattagttccccagaaaagcag
ggcaaataggatgggagggagagagaggggaaagaaaagagagagagagagaggttttaaggaat
tggct

>chrx_108427544_108427744
aaaactagtttttatctcatgttatttccccgttaaccatttttgcagcatgtgaatgttagaca
ttctttcaatcacctattccattgccccaagaaattgctagctaggaaaccctgaatttgtactt
tcaaagggatgccttcccagatgaaacaagttaaataatttgtagaactcagatctaaacactgt
tattt

>chr7_114465164_114465364
gtctaccttgaggaagtctgggatcagtttacagagtaggcagagaaaatgaacatgaagggatg
aacttgtatctaaacaggttgttctgaccctgtgagctctggagcaccttggattttgcagttct
aatgcttgtcccactagtttttgttgtagtattctgaagcattggctctacttgtaacacaaata
aataa

>chr2_178012354_178012554
agcaaagatatttttaagaaatacctctgtgactactacacaatttccatatactccaagctaca
tttgtgctaaagatgtttttgttggaccatcgcgtatccctgaagagataaacctggggagattt
ttagaattccatactgaaccccctcccattattatgatagcagaggatggaaatgggaataagaa
tgctg

>chr2_122444130_122444330
gaaatgtagcaggacgagccacagacgaaactcctcagacaccgaattaaagaaggaagaggttt
ttattcggccgggagcatcagcagactcatgtcttaagagccgagctctccgaaaaagaaattct
tggccttttaaaggcttacaactttaaggggtccatgtgaaagggtcgtgataaatcgagcaag
cgtgg

>chr2_21512295_21512495
tgggcatgtcccagttaccccttggggactttcttaatgttatcatggcaacatacaattaagtc
agattacatttttatatgcagggagaggtgctaagatactattctaaattattcatcagttcac
ttttcacaaaacaatcccagatatgactttaagatgtgcaatattaaaaaaaaaaaaaccagata
atttc

>chr13_60118999_60119199
gctatcagagagtcaggaagttgcaagaacttcagaaaatgtcagagatgactatagctatctga
agcatcatagcagaggatttgcaaagaaacaccctgaggctgctggaaaatttcatttttgctga
aatctgcaaaattgcctactgctgctaaaggcaataatatagtttgtgcctttcttatgccttcc
aaatc

>chr1_117492077_117492277
aaaactctggctactattactgccacgtgtccctgtgggcacccggacacaacaggagctggcac
aaagtggcagaggccgtgtcttccccagctggtgtgggtgtgacctggctaggtgagtggtttgg
agaatgactcttaacctcttcagcttaacccctcctcggagttatctgaaggagttatctcagga
ggctc

>chr3_20435996_20436196
tcaccacccccctgtgctccctcccatgaggggtggagcatggtggacctaagcaaacagtgtttg
ctcctgacagcaggtttctgcacttcctcactgtagttcttgcactcgttccctttttgtgaagga
ttgagcagggcaggctgagtacatggggcaccgctgtcatgaatcccacaaaggggtcaagaaaa
tatcc

>chr7_121128164_121128364

ccagtagaagaagtgaccctcaaaattatggtttacaactgcattgggcattaaccagtttcgca
tttaacccagcaatgttatcctcctgttaatatctagcttttcaaatgatctttgcaccggtcat
gacatctgactttgacatgtattcattttctgtttccataagtcataattttgcctttgtgaaaa
tgatt

>chr17_14293075_14293275
tgcctctccccctcccccaacgcattacagccatccccttctctcctctcaagtatgtgacgagt
gactggcctcccatgtttataaggctgctctgcaatgcgattgctcctccaggactttattaatg
ccatttacgattcccgtaataactaaaccacactgttctccaaacaccaggccttggagaagtca
ttacc

>chr1_241800377_241800577
gaagagcatactcacctgaaagtctttaggctaaagcaacaatactgctaaactttctaatattc
atcattgcacagttttgagctctggttttcactactgttaaacctactgaaatataaatcagtac
aagctgtacctgtctagcccaagatgactaataatcctggatactggaaaaacaagccattcaat
tggaa

>chr2_171537154_171537354
gtaataggttgcaaatgagtttggttgaaatgtgagaatagctttgtaactgaaaaacagaagta
ataccagcaatgtggagaagctcacataagaagctcagacattagcaatagcaaaatcagagcgt
gaaattcagattagccctccccaggctgtgagaggaaggggggtattgcaagacttggcatggcta
aaggt

>chr20_21919200_21919400
acctttggcatactggtgactaacagagaactgaaaggaaatgtaactatctgaccatggttggc
tcatccagtgaactaatatgaataagagaggacatggtaggtttcccttgttgttcatgtttttt
agaatgccgttgccttctttctgtgtttggagtaagttccggtttggatgaaaagggtggcttct
acagg

>chr7_41995675_41995875
atcccctgccagctggctgcctccctttcctctgggaactcagtgaatcacacctccgcagcgcg
tttggctcttgcaagccctgaagggctggaggcttgtgcctgccagctccgaggagctgcagccc
aacttctgagcatggatcatgtcgggaccctggaatttctgagcacaggaggctttccagctgca
gggga

>chr18_3048200_3048400
attgccctccgtcctctccctcccgtccctcttgctctgtttggagtctaaagtagaagcatagg
aagtgggctagaaggcagcacatcccagggccgtctgcgacttctctgcatgcagagacagatgg
cagtctctgcaattactccccatcatttcctccagcgatgctcccagtcagactccaggaagatg
ccaga

>chr11_8890824_8891024
ccttctggcccaattaggggataacatttcccatacatacacttgaacaattttttcttcccccta
tcttggggagtttttccaaaggtaatatttgccagatagcctgggcccagtggctcacgcctgta
attccagcactttgggaggcccaggcgggtggatcacctcaaatcaggactttgagaccagcctg
gccaa

>chr12_50275933_50276133
ctgtgtccagctctgctcagctgcagggctgggagtggcccccactgtgctgagaattccccaaa
ggctacggtgggagagggcaggatgggcagacgagagaccagggggtgctgggtgctagttatgg
gataggctggagctaggggaccacagagtccgcaggcctgagggtcaggacgaggtcagcatgtt
tctga

>chr5_49845643_49845843
catattatgaagttctggtttggtatatgcaagattttgactgtttgttcactctatcattatga
agtatttccctttgtcacttttaatgtattccatccctgacttcaccaatgattccaagttctgt
atacagggtttggattcttggctcctctttctatgcaggtgctgaggccttagccaccatgaatt
tgcta

>chr2_169391154_169391354
cagtaggatcactcctgtatagggttcctgacaacccctgttggacggtctcaccctgttgggtg
gcatggggaacaacaggacccgtttaacaaagcactttgtcccttggtggaaggggtgtgcctca
ctgtggggaaacacagtcatctgggctgcccagattcctcagaactaccaggaggaaaggctaag

tctgc

>chr11_76158552_76158752
atgtaagccatgctcactttttcaaattatattttactatcaataataaaacacattttatgctt
gtaatttattatttagtgtttcttttcccttacagcagcctttctcaaccagcattgagggaaag
aattaaaccctaatgctaaggtatccattgtatgtaaagaactaacttcgccctgtgcatctaga
gtgct

>chr4_92191177_92191377
attatactttgccgcagagatattaatgtctttgattacaccagctccagatgtagctaagggt
gctttttgtaaattctgaataattctgaattctgaaaaatcacttggcctcaagcttttggataa
agagttacttaagaattttcttatgtttatatttccattccatgtctaacactgttttgcttttt
gatgt

>chr4_180067206_180067406
agcaatggaatgctatgcaccctcaaaaaaaagaagaaatcctgtaatttgtgacaacatgatta
aacctgaaagacattatactaagtgaagacaggagaaataataaaagttcaagagactcagtgta
caacatagtgactaaatcaagtgaattaacatatgtattacctcacatacttatcattgttttgt
ggtga

>chr4_184722606_184722806
atgggacatgccatcttttggggtattctgacataagaaattgagagtgtgctaaatgctttgag
gactctttgttttgagtgaacgagaagcagttacttctgtcttgtgagctggtaggggaggtttc
ccaggtatgaggcttgaacttgatcttgtgcagatggagatgggggtggggcgggagttcctgca
gagca

>chr6_20881421_20881621
tggaccccatcctcagagtctgcgattcagtaggtctggagtagagcttgagaatttgcatttct
gtcaggttccaagataatatgatgttgctggtctgggactacactccaagaaacactgaaaacag
actttgctttcacctttgatttatgaatccctctttgccatatataatcatagcatatattttgt
atatg

>chr6_108026507_108026707
gcagagcgttgccatcaatctcctggtgggataggatcagaagaaagataaacccagaagatggt
cagagtcatcgaacagtggctaaggttaaggtgtggtcaaggggtgtgctcagggcatatggatt
gatttaggcagcatattctcttggtcctggggcaaggtcttttaaaatattaaagcaaacaaata
ctgtg

>chr10_99628210_99628410
caggcctgttcccagagaggggctcatcagactgtggggacaacggctccagcctcttaggtggg
ggctgggcagtcccctctgggtggttctcatgtttgttgtcactgccgctaagggctgcagtgag
ctgtgtgcagcctggactcactccctctgctggaacctgggccgtgtgtgggttgccacaagtga
gcgtg

>chr11_95026552_95026752
aggagtatgggagaggaaactgatagacagttggggccagtttttggtttttttttttttttt
gagagagagttttgctcttgttgcctaggctggagtgcaatggtgccatctcaactcacggcaac
ctccgcctcccgggttcaagcaattctctcgcctcagcctccctggtaccttggattacaggtat
gcgcc

>chr16_4238999_4239199
ggaggaggagaacactttctggtgaagccaaggggaggtggcagccttaccattcaggaaacctt
tgtgccctcctccactggagccctcagttatctaaggcagctgcgtggatgcccaggggaatgag
gcattgaggtgctgaggagagagcagttctttaagtttcagaggacaccacgccttcctgggcgg
ctgag

>chr16_11759699_11759899
cacaacgccacggccagctaacttttctttttagagataggagtctcactacatttccagggct
gttctcaaactcctgagctcaagccatccccctgccttggcattccccaaagtgctgagattaca
ggtgtgagccaccgcacccggcctcatctccatttctcagacgaaaaaactgaggcacagaaacg
ttcgg

>chr16_49573299_49573499

ctcatcggctgcattcagctatagctttctggtggcacagcacatgaacagaagtgcagcctcgg
agtcagattgcacaggttcaaatctcccaccaccatccactagcagtgggatcctggaaccccac
ctccccgggccaatgttccccaccatgtaaaatgagaatgattgtagtgcctacatcataactgt
tggga

>chr16_61973699_61973899
attgtaaatagctgtcactaacggactatctgccatttccagggacataagcattatttgtcaca
tggcacagataagaagctgagactcaaagaatgtaagaaacccatactagagcacatagactcca
agtgaaggggacaggattttagctagaaatacctggctcacaaaacatcaaaccacttccaacac
atctc

>chr17_7614475_7614675
aagatggttgctttgtccaccagaggtcaagttcacctctctggtgctgtagttcccagctcctt
cctgattttctaatcgctccttctggggaacaggaagttgatattgccatggtggcggggtatg
ccgtcacctcagtagttttactgtaaaagggaaatttgaacaacaaaaaccaaaaaaaataaaaa
taaaa

>chr19_55652188_55652388
acaaaatgggcatccggctgaagaagtaacatttgggctctcagggcaggggaggcacccaccgc
cagcttagcctgacgttcgcgttccttctcagttctgaagcgctgttgctcggctctctctgacc
ggcgccgctcctgggaaacggagaagcataaggggtgcagggacgtacccccagtcttccttcc
ccaga

>chr22_17943000_17943200
gtgtcctgaaagccagagaagaaattattttaagatagaatcaacttaaatgctgcttttaaact
tagtatgatggaggctgagaaccatttgttaaatttggcttggttgcagtacaatggtgggaatg
aaaatttgtttgcatcactaatactggcattaagattttgtatagaagtttttgttaccatctct
gaggc

>chr22_40640254_40640454
gcgtgtttcacacctagacagaggcacacagtctgactccaaagaaatccagcataagacctaag
gaaatttcagtaaaatctcctccttggttgtgtggattttgcatattaggcttttatatctttat
acccagaggtgcagaaggagaaaggctaatatttgaatgcagacaaagtcagtgacaacatgtag
gtaaa

>chr1_10899813_10900013
tcaggcccgtagggattgctcagtggcacagagcaggtaacagtccccaggcctcgcctaacaca
gaaagtgctcagccagctctttcgtcaatagatgagcaaagcgtggatggggcaaaatgagcgtc
ggctgcccagtctgctcagatcttcaggtggggtctcagtgatagggtcctcagcactgctgaga
tggca

>chr1_108067277_108067477
cgctctggcaggaaaccgttccttcctcatgtgcacagggaccacttcccagtccactcttgtta
acgttataacaaaagcttatgtgtgaccacttttaagggaggggacatattctttcaggacagtg
atttgttaggtatgaaataaataattattgatcaactgcttaaccacccaagtaagacatttgta
atctt

>chr5_68090044_68090244
atcacaatttgcagttcatgttggaaaatgaatttcacttaccaaggagaagtgcaaaattggta
ttatttaagatagttctaccctcacaaagcggggagtcaccctcactcccccacagccaggacag
ccaatgtgcctttagatttagaaatgggctggtctgtcacacgtgcactttctttctcttgtcag
caaag

>chr6_2908401_2908601
aggagatgagccttgaaacgtatgttgaggtcaaatcgaaaaggcctgtatgcaaacctttgaag
cccagagctgactcccttccatgatcacctggtttcagctataatgggcttggcactagcctccc
atcctctgcccatcttcttgttctcaaataatcttggttctacagaagaggatgtagagaggaga
agggg

>chr6_71469479_71469679
atagtgaattgattggtgtctatattggtaaatccttgacgttcgacacagtgaagatataatac
tggaaacaacagctgtaataactttactgcttaataagtgtttgccctatgccaagccctgacca
tggcattttatgtacattatctttttatttctcaaaataactcggtgacatagagcataatatt

ttctt

>chr6_71707879_71708079
gagccaagtggtctggctcagcaggtcccacccccatggagcccagcaaattaagatccactggc
ttgaaattcttgctgccagcacagcagcggtctgagattgatcttcacagcagcggtctgagatt
gacctgggatgctcgagcttggtgggggaaggggtgtccatcactgttgaggcttgagtaggcag
tttta

>chr6_151936707_151936907
aggctaaggagaataaagaaagggccagaaacatgatagaagtggtaaccagtgaaatgaagaca
ctaaaaaaatctctggaagaagcagaaaagagagaaaagcaggtgggtctaaatctggtgatgaa
accttgtttaggaaatgacaggacaattcaagagcttgatattaatacttattcatgcatttctt
tttaa

>chr6_157193708_157193908
cctcccaaagtgctgagccacctcccaaggtgcgagccaccactcctggcctaacccatcggttt
tcagtctgtttggtttattggcttgctaagctttctttaataatttctattcagtaggacagagc
cttccagggaccagaaaaaggggaaataggtatctgagaaataacaaggtctcttcagcaccctt
ttcca

>chr7_30489875_30490075
agagtcactggctgagagtacagagaacagactccaggttttccaattcccaggctagggcagcc
ttcgacccccctgcaccccatgtccccatgcaattatttacactctgacactggtgtgacaggcac
tgggagtcagttccaaatccctactgctttttagaaatgcatgagtcaagtaatggcaaatttaa
ccccc

>chr7_139672531_139672731
gttattaacacaccactcattttgggaggggaatatcatcaagtcccagccaggatgtaaacccc
acgactaggaagcctcgtccgctgggtaccttggtaggtcccagggccgagcacagtgcctggca
cgtgagaaataggtgttgagaaaagttatttcactcttccttttctaactggaaaccaactccaa
gcaca

>chr8_144368825_144369025
gtggtgctctgtccaccttcgggggtgtccctggtgcccagcgcagcaggagtgaatgtcctgag
cctgagccagttctccttgactgcacagcaccaaggtgccagtcagtgctggtcaggctggccgc
taggtcttggtgcacccgccacttcctaccacccttgcattttctcctcttagggccactggtgt
gtctc

>chr9_117954379_117954579
cacagagatgttgcatatttgaagccagctatgcatttgaatgttctctttggaactgttatccg
ttatcattgtgagtttggagcaatgccacttgcctggatttcttcctttgtttcctacctggcaa
acctaatggtattccctaaactccaaatatctgcacatcatttccctgtttataacatttcattc
tatcc

>chr10_88183820_88184020
taccttagcccaaactcctcagagaaatgcatttgaactttctttccatctcctcgttcggcaac
cctataattaaacctctttatctgctgcaacccagagaatattaacattctcagggtattaactt
gctgggtacatcaggtaacagacctgttaacggtttcaatcccactgttgggtatctacccaaaa
gaaag

>chr11_116331190_116331390
ccttgggagtggccttggggctgttccagcagggaattccaagtgccaggtaggaggtaggccag
agcggggcctctaaagcacaaccccagagcaaggaccctgttacctaggtcttcaagtggtagca
gcctaaagagcaggtgtttactggtgttcatacagcactggcaaatagggagagacacagactga
cagag

>chr11_133584190_133584390
gcacaggtgtccatcattggccacataactagcgctgccattggttgcacactcatctctcctgc
agtgcaggagcatctcaaggcagtggacgtgccttactcatggctccaccacagcacccagcaca
gacctggcaaggggtaggctcacagctgaattttgaatgccgatgcctttcgtctcatggctttg
tattc

>chr12_118553617_118553817

agttttagaacaggaatgcaaggaaataaagtacacgtggaagagggccagtagggaccttgaga
gattcaagtgcatggtttgacctttgacttgtggttttatatgttggcagtctgcatgtacaatg
gcctgccagcacttgggaggggctgcatgtgagtgtgtttactgaagctatatgtgtgctcactt
gaggt

>chr13_94212999_94213199
cagagcgagactccgcctccaaaaaaaaaaaaaaaaagaattttgctcaatatccagcataaccct
gttaattttgtgggaacataataaaagctactttgataacaaagcagaaaaatcagctgtttgtc
ttcttgcgcatgtcaaaccttaaatttggctgtgttttctgctctcttccccagtaacttcattt
ttaag

>chr13_111179799_111179999
aggagtcagaagaatgtgccctgctgccagtctgagggtcaaagtgcgagagctacccgggtcac
ctccctcttctgggaaaagccagcctgggaatgctgttgtttgggcgacagagccaccctgttgg
caagcagcttgtgctctggaattcagcacctgaagggcaacattcctcccttccgcacgcctacc
agggg

>chr18_53019402_53019602
ttcacaaggcagtcaccaactaaaacaaagggtccttgttttaaattaagacagtctttttgatt
cctaaatagctcaacaaagtggcatgtcttctagtgaaagcagttggtcaaatatgacccataag
ctggaaaatcaaaaattcaaacctcccctttccaccgccatatagctaaatatcataaagagaac
ctctc

>chr19_51556588_51556788
gcaaccagagtgataatccgatttcagatattcctcccctgttgacactctccaggagctccccc
actacaatgggagaaagaccacctccctgactacagctaaaaagattccgaatatttggctccag
ccaaatcctccagtctcattccctacccagtcactcgttcatcattactgtacagtcatgctggc
ctgcc

>chr22_35159600_35159800
ccccttgtaaggaagagcatgtctatgtctctatacagttccccatttcacccagaatcaaagcc
acagcctggaagtggtgcagagagcctcacaggctgtgcccacccccatttcctcttaatctcct
ctcttactctctccttcactcactctactctagccacagggagctccttgctgcgtctcaaaaca
cacca

>chr1_65326812_65327012
tcttggcactcaacggtgaatggctaaatcggcattattcctgcttaggagaaaagaaacagagg
cagtgtggttcatctcatagcagacttgctctagaaggtgaaactcccggattttaatctaggaa
taatcccaggcccagtgaagagtttgctagattaagcctagggagctctgagagttggagagagc
agagt

>chr1_91604012_91604212
agcagggtcttaggctgggattgaaacaagggtggccactggaccagactagtttggggcagcca
gcaaaggcagctaatcctgggattggacacatgtatgagatggtcctatctgaggagctctgccc
gggagactacttcaagcaagtttgaatgaggagccagaggaagagccagctgtcagtagggagaa
gaaag

>chr1_205407777_205407977
ccagctgcagtgctggccccaacctcagccctgggtgaagaaggggctgccggcttggaggcaga
ggacaactggacctccactcagaacactttgaccttgggaccccacccatgttcccaaagccagg
gctcaagatatctagtatccctctggagccaagggagttctcccgggggaggaagctctctggat
gggct

>chr2_1771393_1771593
ggcacgtgccgcaccggatccccagagtccgagcccgccccaccccccacgccctccgtggggct
ggtgcggcctcccaggtgctggtcccccagcacttaccactaagccgccgcacctgcttccttct
tattcaccctggccctccctctgcctctccacaccaccaggcttgagttaactcacacctccttc
ctctg

>chr2_97612273_97612473
gtggccacaggagatgctctggagttagaagagccagagctaagctgacaggggactcaagagct
tgtttctgatccttcctaatggggagcgcatcagagacaacgtggcaagaagcaaaggagagtgg
ggcatcccctctgcctcagcggaggagacacataccactgcagggagcaggacaagggaagcctc

tgttc

>chr2_189318555_189318755
aaggtttccagcaggaaaagatattctaggttacatgtgtgaattttgctagttctgtagatgct
aatgcttcaggtgcttttggcctttctttctttccactgcactcttggctccccagcctggacct
ggtcactccatctggtaatgatgcagggtttttctcagccactttgccacctggggacccgtatg
gctgg

>chr4_80475976_80476176
gagttaagagtggcagtttggggatagcaccaggagatatcagctgtgatggcttggaaaaacag
tgtaaactggcagtgtaaacaagagcagggcatgtatgagtagttgagaatggtgaataggagta
tgactagacagaagatagtagggatgacaagttttctggggcacagtccaagttggtctggtgtc
tggaa

>chr4_120518752_120518952
ttacatgcttaatctaataatagcatcactgctccaaaaactacgcagttggaattcccttcaga
gaatatggaatatccacactggaaaagctcatcttttgatgagctttgaaggtcatttcttttta
aaaaaataaaaaataaattagtcacagtaaagctgtgaatgaagtgattacactagattccatca
attgg

>chr5_67486644_67486844
ccctccacccacacacattcacttaccctacttatatgtgaatattgcaattaagatggcaaaag
gcaggcagcagcgcgagcacatgtggccatgatgaatgcaaatgttaaagattaaccttttttaa
aaaaagcaggtttacttaattttgaaaagtatcatatttgcaaagctaatttagtatttttaatt
cccac

>chr5_175302794_175302994
ggatcaggcagcaggatttttgggcagactcgggaatttcagataaagaatcccagcaggctgaa
gaagagctacttagagccacagggatgtaagcattgagaagactcagtggtgagtagaggtactt
tgcaaactggtggaggggtggcagtggtaaacaatgtctgatgaatgcatgctggcaagcagagg
tggcc

>chr6_160663010_160663210
tttacttccatcacctggcttccctaagctgtttctatggacctgtttgtgctgttccacaacaa
tctcccctgtagccattgggtaatccctgtcttgcaattcaattttctgttctcaccatggcctc
tgtattttaccccagcctgtaagtgtgagaggtgaactgctgggcctggctggacctttacgtgt
tctca

>chr7_43641075_43641275
taaggtagaattggaaattgatccagaaaagaccacctgaaatttctgtctccaaaatctcctca
ttcttgcaaaacctgctcttcagcctttcatcctgggacccatttcagttcttccagtcttcacc
cggcttctgactttttcttcccttttctttccttctcctctttagtagttaattcaattcctct
cttac

>chr7_148999267_148999467
tagactggccgtcaagagagtcagggatgagctcacaggagcatgacgcttgtgctcggtctcaa
agaatgagtaaatgtcttcagggactctccaaggcgtgaatcccgtccaaaggcctcagccaccc
acctccgccgccacctctgctaccatcctgcctcaaacatcatgctccacagccaggccttcccc
tggct

>chr9_10331400_10331600
cttccatctgaaagcccagacactgtggagcagaggcaaaccatttccccattccctgtctgaat
tccctgattcacagtttttttgtgttttaactagtcttgaagtggttagttaaatagtaagagat
ggttcataaagaatttggtgctgaaagtggagcagtcagagttgcatctgctttcctgctcttgt
gttat

>chr9_126126379_126126579
agagatggcagctgatgaagagagatttgggaagctggttccatgcatctttctcatcgttccag
tgtctcagcgtctgggtgtattagtgtacatgccagtgcaggtcacattcgtccatgtgtctcct
agttccaagttctgttttgcatctgtaaagatctgccagtacctgggggtgtctgcagtctgagt
gcatg

>chr11_44212824_44213024

aagaggtgagcaaggcattttggccaaaggccagcacattgtcagaataatacacacttattctt
ttggtatctcctcctacctttgagtgaacaggttcatccagtgtatttgaaaattgcattgacag
ccaaggttttgaaggcatagctgcgatagatgagctttgcctcttctccagctctgttgctggat
gcctg

>chr11_70020952_70021152
agctccctgtcccatggcagtaaagaaaagagtgccatcccattccagcaggtacagctaaaaaa
agaaaagaaaaaagaaaagaaaagaaaagaaaagaagagtgcagcccacactgagtcctcacaaa
ggcctgatgctcaggcagccgacagtaccccacccccgcccgccacctgccacatcacagtgcgc
gtgtg

>chr15_60852308_60852508
ttttgctccctatgagattgtattaaatttagatctctccagaacatttgactcccttcttaagg
agatttatgttgaaaattctcacatagccacaaaacgcagtccattttatattaaaatgtgagtg
tatttctaaaatttttcttttgttttgtttgtgttctcaagaatgctttctaaaacaaactcat
gtgac

>chr15_76400745_76400945
ttaaagatcattcatcccagtgcttttcaaactgtaacgtcttataaatctcctggggatcctgt
taagctacagattcctgattcactgggcctgggatggggtctgaggatttgcatttctaatgagc
tcccaggtgatgccagtgctgctgctggacccgggaccgcactttgaaaagcaaggatctaagaa
atcaa

>chr17_17527075_17527275
ttaggtgggacgtgctgggggctaggccgtaggagcgggatggtgagaagaggcgatctggagg
gatcatggaggcatcgcccccaggatttgcagcgactgggccagggggggatgagggattgcaag
gagtggggaggactcgggtttcaggcctgagcaagtgggacaatgggggtgccaatgtgaggtgg
tggag

>chr17_19078607_19078807
tccgggggccggctctgggccttcacacatgctgttctctccacctggaacacacttcgctttct
caaacgccacctcccatttatttttcagcatacagttaaaaaataaactcctgggaaacctgcct
ttatccagctggtaaagttctcctgtaggttcccacagcacccgctgcctcagtctcctccccat
cctgt

>chr18_44352602_44352802
agtcagaggtggcgctgtgtgaggaggccccaggaccctactgggatgacactggtaactccgag
gacaagcttggctaggagagcaagagagagcacaggccatatggcatgaaaagaacatgtgacca
gagacaggacctggatttttggttcagtttacactgtcagcatgctgtgtggccatgaacaagtt
gcttt

>chr19_28658760_28658960
aggtccattttattgtcctctctgcacctggtgcctagcacagagcccagcatattcacatgtgc
tgaagaattatctagcttcttaagcatcatataaaataagcattattagtcatagaaaaagtata
agctccaattcactggtttagcgatgcctattttcctaggtttcttttctgattcctagatgtga
ttgac

>chr1_100900612_100900812
cctgtgtctgtggaactatccgggtgatgccaatacacactaaagttttagaggccctggtctag
agagagagagtatggagtgagaagaattttggtctagagttgaaccttgagaaggtttgactttt
aaagactgggaagtgaaggatgactttgcaaaggaggttggaagtagtgtttagagggtagaagg
aaaat

>chr1_113369877_113370077
acagtgttactatgacaccaggaaaacttagaactttgtgtgaaatagactggccagcattagag
gtgggttggccatcagaaggaagcctggacaggtcctttgtttcaaaggtatggcacaaggtaag
ccagggcacccagaccagttcctgtacatagacacttggttacagctggttttagatgcccccca
cagtg

>chr1_117553077_117553277
gtgtgtggttttcccaaggccttgtccgcagctctccataggttgctagtagcaacagcattcaa
caaagctgtgagagttctccctgtgttgtctctgcttgcaggcagaacagtaatttgacaagaaa
gctggtaccataggtgataagtcattaacttagaaatgataattctcaaactaccattctctagc

atatt

>chr1_208351977_208352177
tgggaacccaatttggaaggtatcgaatgagggacaatgtaaacacctgtatccaggaggatgcg
aggagaagaagagttccaataactcacccttaggggagtggtttgcaaaatgtggtactcagatc
aacagcatcaacatcaactggaaacttacaaaatcagcatttggaaaatacaaattctcaggctc
catct

>chr2_125083930_125084130
gcatttgtgtgtgtgcctgtgccaacatgtgatagtcgtgggcagaaactccaaaagtaaagcat
aaagcttttctcatatgtacccccaactatgattaaaaagcctgtgcttgacagagaaaaaaaca
aaataatatttattagaaagtaagtttattaaagcagctaagaacatgggctctggaggcagat
tcctt

>chr3_71095510_71095710
gttctgatgagtgtagaactaatttgctcaaaaatcaacagatgaatgcaaaaagaaaaaaaaag
gaaactaaacaaagttttccttttataagagaaagtggttgatttctatcgtttacagaaaaat
aaacctgaatttatccctagatattaaaacatggcatttataaagcattactaagatttaaaata
gcaca

>chr3_72745110_72745310
cttagaatgttgacaatttcctgctaagattctggaggcagcaacagaaagttgacagtgtgagt
tttggagatagcctgtctgcctcagctctcagccctcagcagccaggggagggggtgggaggag
tgggtaacttatgccctgtgcctcggtttcctcatacctaaaacggagagggcagcagaacctcc
ctccc

>chr4_62254805_62255005
tggatttgtatattagaaagataactctggaagcagcatgcatgatcatgtgactggaggcaggt
ggattacccaggaaatatttgcaataactcaggtgaggtatgagaaagatccagtttaacacaac
aacaatggggattgagtgggagtaacacatttaagattatttagaaggcagttgataggtcttag
tgact

>chr6_114649907_114650107
aattactacttgtcagtgtaatctattttagggaactctactttgaatgataaaagatgggcttg
atattgagagggtgggataggcaaggtgactgcttacttccccagatcagaaactcactctttct
tatgatgaatgagtacctttattgtgatgatgtcttatctgaattattctgtatttctttagtgc
atgag

>chr6_144258107_144258307
gttattgctccttcataaaacactgagtagaaaggaggcttcttaggacagaggggacttgttcc
ttttttatttttaacaaaaaaaagaacctctgagaatcctgtcccctacgatgtggtcaaaact
aagccatggtgtggtgtgcccatatccataaacttgtgtatctccatacctaacaacactgctgg
gctca

>chr7_25946875_25947075
ggaaagccattgcagctccatccacacacaaagtgggcagtgagcctgccctgccaagaggcagc
tgctgtctgttttggggaaggctcccgcatctttgctcagcagggagtcaccttctctcttttgc
ctcccactggccacctctgcaacctgctcacacaaccaattgaaactttctggcaagacatgaaa
tgtgc

>chr8_7616790_7616990
ggtgcagtgtcaaaggggacgcgactggttccaaagctcgagaagaccatggggtcacttgggct
acatgagaaaacgccccagtgtgctggttcatcattccgactcctgcctgtctcttcccgtccaa
ggaacatggaccctaagtcgtgcaggtgcggatgaccatgggcagaattaggggccgtggcacaa
aagtt

>chr10_73336994_73337194
ctgttagttccagctcctgccccttctccatcagtgctgcagagagaatgagtaggggtgtgggc
agagccattccaagcaggaattgccaggcacagtgctgtgtgggccatggttcctagggccatgg
cggcctcctgagttgccaccgtcatcccccgagatacctgctttcatgatttatgctttcatccc
taaag

>chr11_11684024_11684224

atgccgtgcaccctatatttgcaggtgaaaatcagacttgtcccctgcaacactcccaggattcc
aaggacagaatgcctggagtcagtcgtaccaggctttctataccctctagcctcgccttcctctt
gcaacaaaaagactcacttggaattgatctacactgttgccttgcaaagtaactagcagcccata
atgtt

>chr11_45394224_45394424
gcccatgcttgccctggctctgccgggaagacagtaggtgctttgcttcctgagaaaggtcagga
agaggcgtggctcctccagctgtacagacgaccaggccagatccacatggccccgtttggtgctt
ggatgtctggtaaactgcctgccaaggaggaacgcaggtaggacaagaaaactgtgggtggtgaa
acgca

>chr11_45676224_45676424
cctggcctccctctacctctgcttagtaaaaatccaagcaaaccttggacacaggagaccacgat
ctatcatttctgttagaaagcaggcaagttggtgagctttcactcccaggagaaagctcagattt
cattatcagtgaacttgagatgagggcacagactgggagcagtgaacagctctatattggaaaac
tgtaa

>chr13_74547199_74547399
acagcaatatacaaaaagcagaaatgtaaaacccatttagctttctgaatatgttttgccccagg
aaatgcataaaataactttgttatttaacactgcagatgcacctgtgggcaccaaagtactctta
gttatttaaaataattaccctgtggagaaatcttaaggtctttaataaataaaaatctgtgcaat
agggc

>chr14_95723047_95723247
tctaggctgggatgggagagaaattgccaaatgtagggccctggtccacattgcaccgggaaaac
agacatggggaaactggggatggcaggagaagagctgtcagtggacagggccactaagcctcctg
ggtcaggggcagaattggagcaggagcttgaaggccccatgcggtgttagcagatagggaaaagg
agagg

>chr1_34944213_34944413
aagccgccctacttcctgctcctttgatgttgggctgagccacgtgacttgctttacccagtggt
gtcccactgggggactcactccacatctgagcagagcctttcaatgtgctttctggttgagctc
tttctcccttgagcttctgccctctgccacaagaacagcatgctgcatatgtgggctgccccttc
agctg

>chr2_52248896_52249096
ggagtgggaagctagaaagtcatgtgtcctgatcatgcctttgagccataatattagccttaaac
tgacttgtccctaacattttgtggtatggggcaatttagcccctaccttttaaaagcacaatagg
agaacttcctgttatttgaaacagatgtagtcctatcaagcaaccagcaatctgtaatgtgctac
aataa

>chr2_118607530_118607730
gctgatggttcagttacaggctctggggctaaatcccctggtcctggctaccctgaacttcacgc
agctctgagtaggggagtaaattagcctgcaggggggaatgtgggtaattctcgacctcattacag
ccctcctttccaatttcgccttcctacgagcagcttaaaatgcagaagagatcagctgagacaca
cactc

>chr2_154260954_154261154
aagcaagttgtatttcctgttctttgctgttagaagctttctaatttcacctaattgtgattttt
ttcaaaagatttttttttttactttagaaaaaaaatctaggaaaagtatttcccaattttctgcta
ttattaagtaaaattaaggtattagtctacatcatttctctctgataataaatttggaatccagg
aagga

>chr2_213617555_213617755
cagattaagagacaaatcatcattgaatacacagtttattacagttccaaagaggaaggagcaca
ccatgtcatgcagggccacaccagggtcgatcaagagggagaaagagcaagggaaaagcatgagc
aggagcctttattgtaggttccttggaaaaggcaaagcaaggcagagtaggcaggtttaggattg
gctaa

>chr3_14133199_14133399
ctcttttactaatcctcctcagcacagaccctttacgggtgtcgggctgggggacggtcaggtct
ttcccttcccacgaggccatatttcagactatcacatggggagaaaccttggacaatacctggct
ttcctaggcagaggtccctgcggccttccgcagtgtttgtgtccctgggtacttgagattaggga

ggggt

>chr4_79843376_79843576
atcctcatgtttacaacgcaggttttcccttgtatttccattcttatttaaagctcagtggtgaa
aaaccaagtacacaaaggaatgcctgaggatctgtgggatggattgtttaggccagggtttctca
ccctttcttctttcccaacacacgtgacgagtggtacaatcccttctctgattcaccgtggaagt
gattc

>chr4_139792150_139792350
ttagcacatgttcgatgtaaaggaacatccttctccattgaaagagaaattttatttctctggga
gaaaaaattacaacgtgtagtcactaggatgcattatgaacctgttattgcgtgtgggatttatg
atcaaggtctcttctcttccgcaaggattcttcagctaagggcggaattacagatttcagcgccc
acaaa

>chr6_138437307_138437507
aatcagcataattttttttttttttttttttgagacagggtctcactttcttacccaggctggag
tgcagtggtgcaatcacggctgactgcagtgactgcagcctcgacctcctagggctcaagtgatc
ctcccttctcagcctcctgagtagctgggactacaggtgtgcaccaccacgtcctgctaattttt
atatt

>chr8_3896792_3896992
tccaaattccttcatgatgtgtactttctatgtaaaccaacgatatcaaatgtgtttgttttgga
ctatttgctgctgaaagctcttcaccccactctctctcctggctttgggaaggattagaaaaatc
tggttatataattttcaggatcacattaaaaaatcggatttcctagtcttgaaaccttacatttc
ttctc

>chr8_54230447_54230647
tgtgtatatatgtgtgtgtgcatttatctttatccagaccatagcatttttaaatgagaaaatac
tctttataaaggaataagattgttaaagtaaatgaaaatggaaacacgacctgaagaatccctga
gcatacaaagccagccgggcctcctcgtgacctcaaccttgcttgatttgcaaatataagtgaaa
cttaa

>chr10_26055194_26055394
tgaaatatctgtttcacaagtaaagcaattttaccccaccctactcaccctagatgaatgcctg
atcatctttctgttaaagtgtagtcccctgcacatggatgctcttaccagaacaccagtgctgtc
atttacattccatgaggaacccaggaatttaaccaagccttcgcggaggctctgaagccagccct
accat

>chr10_50135194_50135394
aacctctcgttaagtacttctgctgttgccaaacagcagccgtctctatgtgctggtcaaatgat
gcctcccccgaaccctccccctgcttccacacacatcctcagacccatgctaagaggtacatca
tgcccgtgtcttcataaagtgacctctcctttgccatttcagtcatccgcaacactctcctttgt
ggtag

>chr11_84101552_84101752
taggggagaggtcaggaaaaactgtatgagtttcgaatggcaaggagaccgcaggtggtaaacag
gaatgacctaaacctgagcagcatctatggaatgtgaagcctgggcccaaaggggggagcacttgg
ataaattatagagtctggtgatcacctggtcttagaggctgtgagaaagagaagttaaagctaaa
attcc

>chr11_127639390_127639590
attttgtggtaaggggtgatattgtggggttttaagaagaaatatttgtcatttagaattactgg
tgatggcctggatacagttttgtatgaattgaaaaactaaacggaataagagaaggagaaaaaca
ggtgttaaaggactaagaattgggagtacctaggacatctaattagagagtacctaaggaggttc
agcat

>chr12_125281847_125282047
aagcagcgtcctgttgagtcccccgtccccatgccctcccccagtttcaggagcagaaaagtggc
cacacggaagcagcttccaacacacgttcgtttttcaaggctaaaggccaacaggcagcaagctg
atctcctgatccccctccccaacgtgcccagctcaaaaaacaaagaaataaaggccacttcctct
ccccg

>chr14_65048047_65048247

tttagaacaatgaatgcagttagagttcagtcctcagtctccccttatctgaggtctacatgcca
gtggacccatttggtgggggtctgagtttctgaaaaacaacgcagggacatatgttaaaatgtta
tttttactttatatagggaaccaaacatcttgtgactctaacttccttggctgtcttttaagcta
ctgtt

>chr15_61436508_61436708
gtcccaaactggtaaaaaaggagctgagccttttagtttttggatgtagggtattccgaggaaag
gggcgtgacttggccaggggagtgtgttcagcgaaggctgagttccttctgctacccacatgcac
agcaactgaggactgagaacttgggtcctacagagggatctgggaagctcagcacagctgccatg
actgt

>chr16_84170299_84170499
gcccccactagacacccctcacctcttgtcaggggagcgccatcgtgtgcaagtaccttaaacgt
gtgttaatattattgttgctatgtatattttaaaaatcacagagcatcaggggggctgatggcat
tcaaggtctggtcctcagccttgatggatagaaacccaagttcttcattccttagaggcagctca
agaca

>chr18_25358802_25359002
atatttaagcatctgcaaatacctagagcatgtttctgtatcatgaataaaaattataaattctt
agtataggacatgggctgtctgtaaggggtgcaataaaaacagaaaagaaccacgaatggcccca
atagcctagtcaaattaaatcagaatgtgagctttttgttgttattgtttaccatcaacttactt
ttgaa

>chr22_28605800_28606000
gatctgaaaacacacagactgcctcctcaagtgggttcctaacccctgagtagtctaactgagag
acacctcccagtaggggccgactgacacctcatacagacaggtgcccctctgagacgaagcttcc
agaggaaggatcaggcagcaacatttgctgtactgcaatatttgctgttcggcagcctccaatgg
agata

>chr22_46515336_46515536
ggctttgggtagcaccccgcaggcccagcagtagagctcatatggcctggggtcacctccggggg
gaggcaccctccagtgcccagccaggggcaggctgagtgcgggcaccggagaggccagctaacag
caggaagcccagccctcgagtgattgccccctgcagccctaatacatgacagaggggcctcgggg
tggca

>chr12_128643647_128643847
aaacaaatgttttataaagtcagtaaatatagaaatagcattataagcatattatagtgtcctgg
aggaaaacacccgccagaagaatcaaaagctccaaagtctaaaagaggctgcagaagcacagatg
tgaacttgaagagggtggaatgacgacttttgaatttcatcataaccttagatttaattagttgc
cacat

>chr19_10198000_10198200
aggaccgggtcacgggagggagagcaagagggggggacccgacccgttcatggtgactgaccccccc
tctgaaacaggagggaccaggcattgtcggtccataactcatcacccaatctcactttttcacct
ctccatcatttgtccggccagggagcctgcaaacagtaggcgctcattatgtgctgattgtattc
aatgg

>chr3_47118196_47118396
actcattcaaaagaaaaaaaagtattccaagtaaggagaacagtatgttcaaggacatgaagagg
tgaatatgtatgagaagactgatgtgcctcatgcactgagtgtgtaaagagaaactgcctgctag
gcaagaagtttagacagatacggaattactttctctatcatacactaaaggctttgttcttagag
taatg

>chr17_71511805_71512005
cttcttgggaagaaggggctaagctgggggaccccaccctgcagccctgctcccgaggaaccacc
ttttcaagaatctggcttgaagatcccagtgggagaagataaattgggtttacatcccatttcca
tgctaactagagtttgaccttatgcaaattcacctcctgggccttacctgtctcatcagtgaaac
agatg

>chr12_32240333_32240533
aaaagtctcaggtgctcattaaagagaaagcacttgtagcagctgccccaagactgggctttctg
attagcatgctgggtggcttcctgacggtagaggggaatccctaagaaggacatttgacccaccc
acacccgcatattgcaaaacacacagcttctgaaatggtaaatggctggctcaatatctttgcag

tccca

>chr7_119393364_119393564
tggtgaggttacagtcctagtcattcagttaagcactaatttcagtgttcctatgaagaattttg
cagatgtcactaaggcacaacagcttctgcctgggcccagggagctccatcctgctcccgatcgc
ttcttgaatactgtgaacaatagctttctctaatgcccatatgttctatcctgtctatgatcttt
ggtcc

>chr2_58724496_58724696
ttctgcggtgaactctttctctctgtaatccttccccttttacctggtcacctcttacttgacc
ttagtcagcagttcattcttcacttcccctgggaagcctttgattcctcaaaggcacataatgct
cttcgatggtctcacatgacatcttgaaccacagtttgtagaatgcctttcttggtttaattagt
tgatc

>chr5_120277901_120278101
tcaagtttagggaaattgctcagaatcaatcttagctaattcttgacttattttactctaatttg
ttcttcttatatttacactcttcactctgtagtttttgctttatattatttagttcttttgaatt
aggtcctgccgagttgaagctcagtcatttgatcacagatgtcactctctgctcatcacagtgat
cttga

>chr17_79899309_79899509
gcagccgtcgcgcatagcagggtggccagcatggcgaaggcggcggtgaagttgccccaggagag
ccgcaggcagccgtggagccgtgtgaactcacaggccaccaccagcgcagagacggcgaagcaga
agccccaggcggccatgcagaaggtgccctggacgcccgcaaagccaccccggtgggccaccagg
ctgaa

>chr15_83376146_83376346
ccccagtctcattccagacaccagaccaacttggactgtgccccagaaaacttgtcatccctact
atcttctgactagtcatactcctattcaccatactcaactactcatacatgccctgctcttgttt
acactgccggtttacactgtttctccaaaccattacagctgatatctcctggtgctatccccaaa
ctgcc

>chr3_39619996_39620196
atgaattgaaaaactaaatggaataagagaaggagaaaaacaggtattaaaggtctaagaattgg
gaggacctacgacatctaattagagtgcctaaggaaattcagcatagtcctgtcagcaaagatta
tttatttacttcaagagttaagagtggcagtttggggatagcaccaggagatatcagctgtgatg
gcttg

>chr15_93824396_93824596
ctagggattttctcctaagaaataattaggcaaagagccaagagttatgcacaaggatgtgtgcc
tcaggagtgtttataatacagctcaattggaaacacttttctctctgtccaggattaaaaaaatg
aaggcatgttatctcatggaatagtatagggtcattgtaaatgatgatgtagatttatatttgtg
tatgg

>chr14_96362047_96362247
ccttgggaaggtgtttccggcccatttttttcttgctttgaaccacagcacgtgctgttgccccc
actggaacactctcccgcttctcttcctccttcagatctcagcccgctgtcgccttctctctgac
cctggtcccttccactgcaggctgttgctccggtgtctctgggctgctccaccacgtggtgcctg
cccat

>chr19_12249400_12249600
acagaacacaaatattttaaaaaaagttatccatgaccttgtgaaaagatgataaaccactaaaa
tactgcatctatgtgaaagaaaggatgaagaaatatactctacaataaagaaagttgatacagga
ggctgaggcaggagaatcacttgaacccgggaggcagaggttgcaatgagccaagatcatgccat
tgcat

>chr21_20926729_20926929
gaaaaatgctaataagactcatgctgagaaaagctctcactcatcagggtgcatcattgcagaca
ttaagctgagactgtgaattgctattagccaaaactggagaaagactaacgagaatagtgccaga
aaatacttcgtggcactgtttgaattgctgtctgaggcaggaggtttgttgtattgtttttgaaa
tgatt

>chr8_728600_728800

tgccctgtgtcagtgtacccctgttggggggtgcctcccagttaggctgctcaggggtcaggggt
caggcacccacttgaggaggcagtctgccccttctcagatctccagctgcgtactgggagaacca
ctgctctcttcaaagctgtcagacagggacatttaagtctgcagaggttactgctgtctttttgt
ttgtt

>chr14_104320447_104320647
cttcttctgcagcatttccccagatcgcccagggatgacgcatctcaccacattcctccagactg
cccagacctgggtcacgcatcacaatctgccaccttggagctgtctatgtacctgttgccccagg
ggccagcaggctccatgaacaggtgtcgcctggtttcttccatgtacccacccaggtccaggccc
tggtg

>chr13_79856399_79856599
tcctctgctttgcaccaaactgaaaacactcattttcatgtaatttatggattccttcagctttt
tactcccgttaaacttaattactcgaagaaaaatttctcatgtaaaaacattcctttgggagatc
ttcaaatcccaaataaaagaagtaggtgtctaagcaggtaagtccaatatgaaaaattgaggaaa
gatta

>chr2_231769756_231769956
acaaacatgtcccaccctgagccggtcctctggagccctccttaaccactccaggcccactggcc
tcaaggcttctccactgccctcagggaaacccctccacccactcccagaactgtgctttggctgg
gggctcacacagcgatcactgggtcattggacatcagttttccccagtcagggtcattccgtctg
aaatg

>chr2_54612096_54612296
agatggggacggcactctctgccagaaaaccagtctcagcaaacttgctgagttaaaactatcag
atttaagctaaaaacaactatatttccaaaatatgcagcctgtgcaccatgggaaaaggaagact
atccgtacctagggagtagataagtaataaatagtctcatactcttttaaggagagcaatgcccc
aggct

>chr13_56227399_56227599
ttgtccatactttagatgaactgaatcaagcctactatactcactgacccagctaaatgtatact
ttgcaaaggagtaataagaaatgtcctgtgtggtgtgtgtggccatgtgccaatgtgaataaatg
gtgtgttgagcgtagagtggtagctagactttagaatttgcttgagggtattagtttgaataaat
gttta

>chr14_65434647_65434847
tctccctgcttcaagtcttaaccccttcttccagccaacctcattagtaccctactcaaacccc
tcctaccattgctaaggctcatgttccacctccttggccaggctctcaatgccctctgccaactg
gtccctgcattcattcattaatttaacaacaggcattgaatctgcctggccctgtactagggact
ataaa

>chr2_1240049_1240249
tagtctcccagcattttgaagtgtaaaaattaatttattatagcagacatccaggaccaaaggcc
ccacgggaagcgatggatcctgagggacaggtgctccccaggaggaccaggaaaggtgatcagaa
agggctgtgcgaaggtggaggcagagctgcctgccctccccatgcaggggacctgcttcctctgg
cttct

>chr12_7403733_7403933
gccaagatggccgaataggaacagctccggtctacagctcccagcgtgagtgacgcagaagacgg
gtgatttctgcatttccatctgaggtaccgggttcatctcactagggagtgccagacagtggggg
caggacagtgggtgcagcacaccgtgtgccagccaaagcagggtgaggctttgcctcactgggaa
tcgca

>chr21_18840329_18840529
tttacagaaaaagcttatctagcctcttacatgaggaccactatttgtttttcttaaacagagag
taacaacttcagggctgcaaaatacctttgagattacctaattcaccctcttcattttacagata
tgcacccaaggtctggtacgcttttactaggtcagtgtttgggctgaagaaaacagggttgggtt
gctga

>chr12_96380469_96380669
ctaccaattcgacagtttttcttccccaattttctagtcgggaccaactctaatcctactccttt
ttacaggactcctaagtcctgtaaaaagattcctaagacagcacagagcttgacacacccagatc
cctgggtgctctcaggcaagtcactcaacttccgggctgtgtgtatctgtaaattgcagttcttt

caaag

>chr11_61991824_61992024
agcttgtattgctatctcatggctaaggttccaagctactgaatcttcatttatgtgtgtgtata
catgtccagatgtgtttatttgtatgtacacttattgttacatgttgtgtctaccaattggctta
taagggcatcataaattaagtaaataagtctaagtaattttcaagctcacatgacctaagtatga
cttta

>chr1_162369176_162369376
aagggtacccctgtaagggcttacgtctgtacctacaggcgggctttgctatcttaagggactat
cggaagtggacttctgatgggaatgcaaaaatgcaattaaaaaaaattccaactgttattttaag
ttcaggggtacatgtgcaggatgtacaggtttgttacataggtaaacgtgtgccgtagtggtttg
ctgca

>chr18_56436820_56437020
attcagaaacgggaacaaggctatttgaaaactttgcaatgttatggcagaaataatctttgcag
gatgtaaatgaaaagttccaagcaattacgacaaatttttgagcaattaatgcactgggtcttta
gccttcaatgagctggaaagctgagcaagacagaacttgaaccccctaataattataacagctaat
actta

>chr11_94165952_94166152
cagagcgagactccgtctcaaaaaacaaaacaaaacaaaacgaaacaaaacaaaacaaaaaccct
tatctgagttctgaattcacacattcaattgcctataacttcccttcatgtaagtttctaggcat
ctcaaacttttcacatctaaagtggaacttttaattatatgcctatcattccctacaacacatct
gtcct

>chr12_43865133_43865333
tcctacgcccacggagtctcgctgtttgctagcacagcagtctgagatcaaactgcaaggcggca
gcgaggctgggggaggggcgcccgccattgcccaggcttgcttaggtaaacaaaggagccaggaa
gctcgaactgggtggagcccaccacagctcaaggaggcctgcctgcctctgtaggctccacctct
ggggg

>chr8_10405990_10406190
cgatgggctgtggcaccgcggggctgcactgggtcctgggagctgggagacggtttccctcactc
tgaggctggggtcactttggaccctttccttcctgacacggtgagccaggaatctgggccaacac
cttagccccggccattccccctgcctgcaagtctcctgcggaagcagacgaggagctgttgggac
agaag

>chr7_128678364_128678564
atggacaaagcaaaaaataacagatgctggtgaggttgcagagaaaaagaaatgcttacacactg
ttggtgggagtgtaaactagttcagccattgtggaaagcaatgtggcgattccttgaagagctaa
aaacagaactaccattcgacccagcaatcccattactggatgtatacccaaaggaatataaattg
ttcta

>chr13_110969599_110969799
ttgcaccacactcacacatacacacagatgcacacacacacgcacatacacatagatgcaaatac
acagagacacacacttgcacccacacacacacagaaacacatgtacacacacataaacacacatg
ctcatgcgtgtacacacacacacacttagccggaggacgctcctggtttctttcttcatgggagc
agcca

>chr5_174967394_174967594
tctctggctggtcccacctgggctcactcatgtagctgcattcagttgccagcagttccgagatg
gcctcagccacatgcttggtagtaggtgctggctgttggctggggcacctcagtgctcctcccca
cggcctctcctcccgcaaaaggccaaactggctttcttgcacagtggcctcaggacagcaataca
ccagg

>chr21_44167331_44167531
gagagccctgtagcaatgtggggaccctgggaccagtctcagttaagctggaacttccccggatg
actggggagaggagctgcaaaaggcagggtgggggtgggggggccttcccgagatggcagcccagc
cagaggccagcgttcttgcctctgggaagagaataaaggccccagacagggtggagctagctcag
ccccg

>chr8_27509681_27509881

gctgagaccaaggaatctggggggtcctgcaggtgagacccagggatctgaggtttggttcttct
catacacattagggtttgtagtagttgacctaggtcacagcacacagccagaggcaagtcacttc
tgccttccagatgctgacagattgtggtaaacttgttacaacaaatttgatgtttgtaatcccat
tccac

>chr8_75779245_75779445
ccaactgggtggagcccaccacagctcaaggaggcctgcccgcctctgtagactccacctctggg
ggcagggcacagacaaacgaaaagacagcagtaacctctgcagacttaaatgtccctgtctgaca
gctttgaagagagcagtggttctcccagcacgcagctggagatctgagaacgggcagactgcctc
ctcaa

>chr9_72715180_72715380
cagataatataattaacttgcccaaagtgacattgtttctaagtggcgtggccaagatttgaacc
aacatcaagttgatgtgatagccacattcaaccactctaccatactgtgtccacagcaggacctg
atccagggaggcagacagttaaggcattcagaaaattgagtgggaagatcctcgtttgagttcag
ggact

>chr5_132936901_132937101
agtgactttatgaccaggaccaaaggtcacataaatatcgattggcttaaaaatgcatcaaacct
ctattttgcttataaaattattaggcactgtaaggaagaaaagataaataagacaggctccatcc
atatgctaacgtaaaaataaaagagtaagaaaacaaaaagagaaaggaaggactaagaattcaga
aaaag

>chr4_146813550_146813750
actttcctcttctgaggcttggaagaaggagaaagaaaggaccattaaggaaaacaaaaaggtac
actgcatctggcattaattcacatacactgccatataaaaagcactaaaaatgatttgggttttt
ttcttgacaattccgagattttatccataagaaagttttagtactgcttttcttcaggtagaaac
ccata

>chr11_126458790_126458990
gggggaccaggggagacgagggcatcagtcactccagcagggagcttgctgcagggagaaagaga
gaacagcaatttgggccgtgaagcctggcactcattcttcaaggggggctgcaagcccccaggatt
ttcctggaacccagggcacccgcacactagctgcctctcccggtcccttccagcaacctgaggaa
atgcc

>chr7_189517_189717
tgtgtggctgtgtgtgtgtctctatgtgtctgtgtgtctgtgtgtttctgtgtatctatgtgtgt
atgtgaatgtgtatctgtccatgtatctgtgtatatctgtgtctgtgtgtgtgtgtatgtctgtg
tctgtgtgtatatctgcgtctgtatgtatatatgcgtgtctgtgtgtatacgtgtctgtgtacct
gtgtc

>chr12_74936133_74936333
attcactgtagggatctactatcaattagctttcaaatacatctgcagcaaatacttttggttcc
tgttttgtaggtttggagtttaatatatttagtgctaagctaattagttttctaaaaagataatt
gcaagacccctgctttaccaaatacttgacaatgggactttgaaggttatttcttatgttttctg
actag

>chr3_172082306_172082506
ctgccccaagggcacccccctcttgctgcaatcctgccctgtgtggggcagcagcccactagtgcc
tcatagggagaactatacaaatgtgtagtctgatccaaggttttccatgacacggtggctcctgg
gcaaagaacaaaagctccccaagcacttttccaaatgaggttcctggcatgaggcatttaaatat
gccag

>chr4_30918902_30919102
gagctctttcagaattttcacttctgtattaatttggttcacactgccaacttaaatacttggcc
acaaaaaatttattattcaaaactgtcaaaatgaatctgaagagctagtgatataatgctgatac
catactatagcattctaattaaaatattttaaaatatgaataaaaatgataggtgtgatatatta
taatc

>chr14_67723647_67723847
tcattttgccaaggctaaggacgcggctgtgacacagcctcaggaggtcctgacgacaggtgccc
aaggtgatcggggtacagctttttttaaatacgttttagggagacatgagacatcaatatgtacg
ttggtttgatccggtaagacggaacaacttgaggcgggggctttcagttgataagagttagcggg

gatca

>chr17_41956074_41956274
tggccagcagagggacccctgcgctcagagcgccgcaaaccctccacctcctgggaaggcgcagc
aagacataggaaggaggatggggctggagttctgcaatgaggccttgaagcctctcctccaggcg
ctgcctgctccctgacgctgactcagccctccagccagccttcccgcctctggctttctcctacc
cttca

>chr7_105669964_105670164
agctggaaagttccctctcctccacctctgtctctctctctctctctctctctctctctttga
cagaaccttatctacccccctgggaagtgaagtgaaggaacttgaaggtggctccctccagctctt
ccctacccccacgaataaggctttgtcatggtccaagcttcatgaatttgttgcataattcaatg
gctcc

>chr1_55461212_55461412
agcaggtgcggaggccctgggatgcatgcgtgttccaggaacagcatggggccagggtggtggaa
tgggtgagtgagagggggcgtggcgggaggcaagctcagagggttgatatgcaggcagcgcgggc
caattatccagagcctttcaggtcatcttaaagcatttggcttttactctgatgagatggggagc
cattc

>chr1_179553177_179553377
taataggcagcagggtggaaaacacattggtctgacccaggagctctctggatgtggttaagtaa
ttttaggaacccaaggaatgaactcaacaaataacccattaagtctttttggtttgtataacaaa
aacaaatccaggccgggcagtgactcactcctataatcccagtgttttgggaggccaaggtggga
gcatt

>chr2_97075673_97075873
ctctaattacttgcccagcatctaccaagcaggccctgtcagcgggagtcctgcctgcactcctc
ccggggcggatgctctcctcttggctagcccagacaggtgcccttgggctccccgccaggcaggc
actgacctcccgcggtccagccacctcctgctctcagggatgccccagccggatctcagcccagc
aatcc

>chr2_121624930_121625130
cccgagtccagccagcctttccgttagccagccagccagcgagcgctcctaaaccctggccgccg
ccggttataaatgattcatgcagccccgcctccctcggcggccacttgatgttccctgcgctcca
gcccggcggtccaccgcctctcctgccgctgggcagtgccgccgctgggcagtgccctccccgca
ccgcc

>chr2_132302130_132302330
gggtgatctcttcctgctcgtgcaatcttgagcatggcaggacccccacacccactgcggttctc
cggcctgcacctcccgcccacaccccatgctctgtgttggggagaccacctgggactaccgggtg
gggattagtgggcatcgcgggggactgtgggaacagggcactgtgggtggaggtgtcaggaacgg
gaacc

>chr2_200274155_200274355
gtgctgggattacaggcgtaagccactgcgcccggccgcaaacctccaacattttctagagcaat
acttggctgtaagatattgcatagttaatacgacttcactgaaggaatagctttttccatggaca
agatggtgaattaacatttttatcatggctaaagtatactgcctactttatattttcaacgcctt
acttt

>chr3_57186160_57186360
gaactctctcggctgtgaccagagcaaacgagaccaagcaaaggcaagctgccccccgcccccga
ccaaagcataccctcacccgacctttgtgtccagcatgcattccagcccaggagctccggcccgc
tgggcagaggctgcacaccattcctgccagcatctaaatcgctgctttctggcctttcactgtca
ggcca

>chr5_113785501_113785701
gtgaaatgtacagatgctgcctcctcagatctgctaccttagattggatttagaatagagtaagc
atccaaacatacacacacacacacacacacacacacacacgcgcgcgctcgcgtgcgcgcactct
tcccttagaagagcccttaacccagaatgggggctttccctgcagggtattcccacaacatcacc
tcaca

>chr5_141066816_141067016

gccttctttatcctggagaactggggcagccctggggtcagggggagggaaggaacaaacacaga
acacctcccttcccctgcatcctcattcccatgactgggccctgtctccttccaccctaggctat
tgacatgagctgtatttacaagattttaatgcaattttactgcaaaatgttttataaagggtat
ctagt

>chr6_799600_799800
ttgaggcttatttctctgatgttaaacatttcagctccttgaatgtgtggggcatcctctatttg
taggtagaatcctggaactggggaagtaatttgaggctatctggtcatcttcccacctaataaga
caagtgcttttatcatgctgattctgcatggaggacatggtctcattttcccaggaactaaaggc
tgcta

>chr7_72978864_72979064
agggtctcacctaactgcagggaagcctgggacatgtagtccagctttgtacctaggaaactgag
aagcggagttggggaacatagcaatctttgccactgtgggcaagtccctggcagtcatgacacgc
tcagtgaattaacgtggggttgaggaaatttggggtgcagcacatcaagaaattcacttttgccc
tgggt

>chr8_25139883_25140083
aaatctctggttttcgtgtcccatctcctttcacctgctgtatgctgttattgatgtttttgctt
tctgattctgtaagttccttcagtccatgtaaagataaatacagattttactagtatgtagcaat
acttgtattatttctctcttgcatttcccccctgtagattacaacaccagacgctctgtttgatgt
cttta

>chr8_62825446_62825646
ttaaacattttaaagtcattcttcttaatgcttttccagtaggtgagttacgtctgatgagttga
aaaaggacaaaaaataaccttgatttcttccagcattgcaccactgcaactgttctctcaaggcc
attagtgccccaatgcttaatccactggacacttttctgtccttataattatcaggaatctctca
gttac

>chr8_80031045_80031245
tcagtacatatatataaagtatataataataaatatctcttaatgatataaacatctgcagagat
aataatttggtaactagaaatattttataatattttttcctaaaatcctattaatcaggtctga
aaagtctgttccttccaattgttcactttaaatcaaattgcctattttccttctacctttttaa
aagtt

>chr9_120531179_120531379
gcatcactaatggattatagtacttttccactgaactaggaagtagcctcagaatcactctctcc
tcactgctcccagcagctgctatcaatcaattgcagctttgccactgtaggagctcttaaatttc
attgtttagctctgacattttcagaggatataaatgcaatgaatttgcctaactcctgaaattca
ttttc

>chr10_70860594_70860794
tgggatggcaccttgggcaggactctgttttgcccatggcctcagctcccaccagatgcctctag
tccctgggctcaggaaatagacaacctccttccactatcgctgtagcccaaggagggaagtattt
ttttctttctttctttcttttttttttttttttacagagtctcactcttgttgcccaggctgga
gtgca

>chr16_8858099_8858299
gctacagaaatctttctctctgagcttattcttccatgcagagtattttaaatttttcttttaaa
ggacagaagggattctttgtgtgcaggacaaaaggagctggggaaatgaatatgtgtcgcatgat
aggagccttgcatatgtcattcaatcctgcccccacttctcggttttagttactacaaacgatta
aagcc

>chr20_50013993_50014193
aggcctgagccgctggcttgccaaagtccccaagcccaagacacacattaaaaaggcatcgccct
tcgacaagttccctgggggcttacaacctcagcactgatattttacttgcatttttatcctcgg
atgcaaagtcctgaggacactcctgagggactccccaggagccgattcctcagatgcaaaaagtc
ctgag

>chr22_20231000_20231200
attgcaggagtccccgggggtcccagtgggggttccacccacagggcctaggaaacactgcaat
ggggctggggtccctctgggcacgtctatctcaaaaacgggcgcctggatgcccgagggcggggg
tggagggcaagtgtgtccagggcttaggaggagtcgcgtcccacaaccacccaggcagcctgctc

cctcc

>chr22_49928596_49928796
ctgaagcaccaatttaattcctttcttcctctttttttaatttggtctctccccccagccatgag
gtacccacgcaagcaggagcagtgatttacctgccagcctttcgctggtgcgaagaacatttcat
tttgctaacgatatttgctgtttgatagagctcctcaaggtaaacttctcaaaacaccaagtcct
tataa

>chr1_230653977_230654177
tcctcaccatcaaaactcttttgacaaattgaatcaatgaatagaatcttttctcttttctttt
tcgcccatgctggcctcaaacatttgggctcaagagatcctcctactgaagcctcccatggagct
ggggctgtaggtacaagccactgcacctggcttcaactaatagaatctttatcctaatttcaggt
ccctt

>chr2_233134356_233134556
taaaagacttgagggtctagccaggctgcaaaggcaagcagactgctggtacaagcatgggaggg
atgagaaatggcagattgttccagggtgtgtggtgtagggtgcagagagagagaggagctagagg
agacacaggctaggccttggagtctgtaggaatgaaccagggcagctatggaatgattttaaata
tgtaa

>chr3_58023560_58023760
catgatcttaaatgaaaaccacgatcacttgccagaaagaacaggtaactaggctttgaaaaaat
aagttagaggagatagcataagaaaaaattaaaaaataaataaaatcaatgaaaacaacgtgtta
ctaaattcttgaaaagtttttttgaagactttgagcctgaggcctgttcttattgtttgtttgttt
gtttg

>chr5_54923443_54923643
ggaaataaagtggaagatgatgagagaaagaaaaagaaaatgatgcctgaggctaagggataatg
gagcttctagagtgagttctccaccagaacaaacagaaagggaagataatgagcatcttaactga
taccaaggggctgcatttgaatattactagttttaaatactcataggaatttcatacaattaaat
taaaa

>chr6_125754101_125754301
ctgagcaaagaagaattcgagaatccagcagctccctccacccccgacatccccaccaccgactc
cttgtgaaccagaatagattctgagaaactctgcactgccacgggtcagagaagatttatgggca
gaaaaaggaaagtgatgtacagaaaacagaagtgaggtacagaaacagttggattgttggattgg
ttaca

>chr6_155470708_155470908
atgtctgaatgtttaaccttgtttggaagtttttcatctgtgtatatatgaagaattcagcatg
aggctgggcatggtggctgacgcctataatcccggcgctttgggaggccgaggtgggcagatcac
ctgaggtcaggagtttgagaccagcctggccaacgtggtgaaaccccatctctactaaaaataca
aaaat

>chr7_28428675_28428875
acagccatctctttaggattcatacaggcatagtttatcaacagcgtcagagctttctcacatgc
aaaagcctcacgttcattatttactgaatattggggaaataatttgaaaaatctgcacattagtt
atgtatgcattctgaaaacagtgaacactatttgcatcttaaaaagatctgagtaatgctgtata
atatt

>chr7_90954864_90955064
gttagatactttaattgactttcagtggcagtaataagtaactggagtcctggcttgtagaattt
cctttctaattgtcagtgttaaactactcattatagggataaatagcataccgctttatttaaac
aatttccttcatacatgtaaattccagcggaagttcccagtattttcaggcccttgggagagctg
tttgg

>chr11_41479624_41479824
tttgacccaggcaacgtagcctctgaataaatctattatgttacaatttgcatcttctttctata
catagacatcaacagctggagagaatcacttaggtatgctcttctctacctagttaacacacctg
tcatctcttccatgaacgtgtccacagtaacagttcaagttggctaacagtccatgaactaacac
tattc

>chr11_76780752_76780952

acccgttctgtctctgtccccatctattcaagtgttttgagtgccttcagagcccctcccaggca
cctctcaagtgccctcagagaactcttctgcctctccatccctggtccccgctcaaaactttcct
gggctcaccgtggtcctgaggcttggccttcctgctgcccacctctgagccctcctccaggctgg
tggag

>chr11_131788190_131788390
caatgcatttagctgacatttatcaccgtattctctcctgactgctggtttagtccgtaccacac
cttgcgttcttatgctttgactctcattccatgtgggacagctgtttgctgtgggtcaggtcctg
tgctgagcagtttaccagaagaacctcatttaatcctcacagatactccatcaggtggacacaat
gatta

>chr12_120885617_120885817
gcatgatctcagctcattgtaatgtccacctcccagactcaagagagcctcccatctcagcctcc
caagtacaggaccacaggcgcgtgccacttggcctggctaatttttgtatttttgatagagacg
ggattttgccatgttccccaggcttgtctggactcctgagcaaaggtgatccacccatctcagcc
tccta

>chr13_44995200_44995400
aaatgtgatgtccaagaagcactcacctgctcacccgctcaatggagggccactttccttctcaa
agtgctcagacacaatcctcctagcccacccgctaggcctggcgtccccactctctctgctgaga
tggcctcttccactcagtcccccacatctttccacactcagttcaggtggcttgtttttgttttt
gagac

>chr14_77688047_77688247
caacctcctcaaatgtgagctcccgtccctcactccacccagacaaccctgaaggtcagcagtgg
ttccccccaccgctaaatccaatggacttttctcagttcacatcttgcctgcgttctctgaaacg
tctgaccctgtagaccgtctttctctcttgcacaggtgctctccgttgcataggtgatgtcacat
tcttt

>chr15_80681745_80681945
cctggaatagtgcctaacacttagtaagcattcaataagtgatagctgtcagcaatactactcta
acttgttgtgatggctctgtatatgtaaattattgaataattagggtaataaattatacccataa
attatacccaggctttagttaaacaggtatttcaggaatgtgtgcctgtttggtctcaaacaatg
ggaaa

>chr16_55424899_55425099
aatcggagctgtctacaggtgggggccaattaccatctgaataatcacagtgccacacaagaata
gcatagccgctgagcgtgacatatttttatctctatgcatttcaatgaagtcaggctagtacata
aaaggttatcacctaggaaacatattttcctaagcacaagttaaacatgcaagtgagatcagcaa
agata

>chr17_48664001_48664201
tcccaggaggtagtgttaccctgtttaacagaagaggccactggggatctgagaggcaaagggaa
ttgcccaaggtcacataggaagaaagtctcagagttggtggcacctttctaacaccagtgtttgg
cacacagaaggcacttttagggctagttgatggagcagctatgaggcttggggagccccggtgct
gggaa

>chr17_55994801_55995001
ttggagagtctcaaccaggccttcactccacggagtctcttcctgactataattcttacagggaa
caaaaaccaaggataacaataatacttactgagcatgccatgtgggcctgacactaacacttgtc
tcattgatcctctcaaccatcccatgagagagaggcctttcttctccccattgactgatgtggag
acagg

>chr18_22761202_22761402
gttatgaaacatatttttctagtcctcccaggagcaaggccaggttcacaatttgacctcagaaa
catagcagtcctgcaatgccagacagcctgatagaactcagtattagtgctctctgcctggccca
cacaaaccataacatgcagcatggagcaatgttacttacgggaaatgacaagcaatggatcagtg
gggga

>chr20_24898800_24899000
cgagattctccacgccagaggcccggtgggtgggtgaggggcctgccaagcctgggagatttatt
tgcaggattttccccacggactcataactgaaatcgcagaaggccttgcggtttagaggccctgt
ctcatgccccaaattgcattttcccggaaagggctcagggcgactgaggtccccaccgccgctgc

ggagc

>chr20_56046194_56046394
cgtcttgtttccatttcccctaatctccagggcaggcacaattgtgctggccaagtgcccagtgg
gggtttggagtgggggtcgtttagcttctcctgccctggaaactaaactgcagtgggttgggagc
cggtaacaaggatttacgtttcggggttgccctcaccatttcctttaacacatgagtccaaggtg
gccgc

>chrx_133048134_133048334
ataaaaggcatttcaatggtgaggaatgtttgaggactattaagatgtgacaggattcactaggg
taaacatgtcttgcctcaggcttctgggagtctctaatccctataacaagcaaggtcctaaataa
gagaaataataatactagctactatttattgagcaagcctgtatcatgtgccctttacatgaatt
gtctc

>chr1_16443213_16443413
taccgtttcttccttcagatctcagcttggataccgcctcctccaggaagcctttctgactctcc
acaggctgagcacgttgttcagttcctgggtcccacagcccccacgcctgtcatatcacatttat
cttttgaatattctctccagcgactaggagttctgtgacggtgcggcctgggatctcccacccct
tgctg

>chr2_43941096_43941296
aaatatactatgtactatgttttttctaaaatacgactgtgaaaatgttttcttagttactgatg
ggttagggaacaaaatatttgcacaatacttttccagtgaacacactgctactttaaatcacatt
cttactataattcataataatgagctatgctcttgtttcaaggacctcatatgcatatcaaaccc
tgaga

>chr2_159370554_159370754
tttgttcctgctagacagctctttcagcatcataatggccacaaaacctaacaccatctggtgta
taaatgtgttttcattgaaaacacagatatgttgtcatatcttccctcagtttgtttcatgctcc
cattgaacaaacacttttgaatacctactgtgtttcaggcactttctggatctgctgagaatgca
gtggc

>chr2_232316956_232317156
tcagaaactagagacaggtactagggaagactgggaatttgacttcagtccctaatcgcaagcct
caatgtctctttctggccctgcagggtaacccaccttacatcttttttttttttttttttttga
gacagtgttgtcgcccaggctggagtgcgatggcgcgatctcggctcactgcagcctctgcctcc
tgggt

>chr3_33467396_33467596
aactatgaatgctatgtggttcatctaaaggatggcattaacaacccctatctcggggtgggaag
tgggggagggcggcagggcagctctgaatatatatatatatatatatatatatatatatatatat
atatagcactttaaaaacttcttttaaaactttaaggtccagattctttctataaaaaaaaagca
agtca

>chr3_51129360_51129560
ggggagccatgaggtactgagcctgacgaactgtgcactctggcccagatagtgcgcttgtccca
tggtctttgcaacccgcagaccaggagattccctctggtgcctaacacatcagagacctgggttt
caagcacaaaactgggtagctgtttgagcaggcaccaaactagctgcaggagtttgtttttctcc
ataac

>chr4_154191750_154191950
tctacagttacctttgagagaaaactaatctcttgtgcttctgccagacattgaaaaacagaatc
atgagttgcaaataatttactttatttcaaaccattataagcaagagaaagcaatccagtaacta
ctgattcgtttacacaagagatggagcattaaataagaatgttaagttgaaaaatcttttaaaac
ttccc

>chr5_142658407_142658607
catcccatgctaattatccagcacttcatagacacaaatcatgttagttttcctttggggtggc
caaggtttcctcccatagttttaggcatttggatatatataccccaaaagtgttatgtcctaagt
gcctcagttctgcttgtatagtatcccacagaatacctacaaacactaaaaatacttttcaggat
ttgtt

>chr6_65970079_65970279

atctcgtgagaactcactcactatcagaacagcatggggaaactgtctttatgatgcaattacc
tccacctggtctctcccttgacatgtgggaattataagaattataattcaagatgagatttgtgt
ggggagacaaagcttaaccatatcactcaatttcaaagacctgcctgcagatgcctgctctactt
tacca

>chr7_153869067_153869267
agatgtacacactgggatgcagagaggtgatctgacacaaggacagcccatggtcaagcccctttt
gacttctgtctgctgttgaaaaggatgtgcctccctttcactgagcctcattgcaatacttgtat
agattctgtgcccctccctcccttccttccttcttccctgcctgcctgcctgccacaggtgttta
atgaa

>chr8_6945190_6945390
cccaggctagtcttgaacttctgtgtgcacccacctcagcctcctgcatttttaggaggcccctc
ttgtagggattttgatccagaggactgggtgcctcatgtctcctcccatctctctcttctttctg
tctctgtcctctctctctttctctttgccttatagctgccctgggaactagactctgccttaggc
atccc

>chr9_138189579_138189779
tgaggagagcaacactctccgtctctagcccctggactagaacctggatctgctgacaccattgg
ggcctgtcagacacctgccaatcctgacgttggatgagaaaaggctgctaagaccacggggtccc
accggtggagacagccatgcccacaggtgggaaggggcttgccaaggctaccaagcagatagcaa
cggcc

>chr10_107812810_107813010
aagccacttggtatcacttaggtgactgtagcactgattcctatgccattttgattagtaatgca
tattgatctccaggaccaggctgaacacggatcgaatgacatatgcacaatagcacaggactgcg
ctcatttagcaacaaatcgcctttggaaagtccctttatgtttattctcctcattttggttccat
ttgat

>chr11_120481590_120481790
ttgattggaaactctctgaccaggttctgtattggtgctcaggccttttacatgagaaccacaga
gtctcagaatgtcagcgcaggaagagccttgaaagaacgtccggcccatgtcctgtcgttttcaa
caggaaaccaaggcccagtgagcggacaagactttgatttggattgacagctcgaggcagaacca
ggaga

>chr15_83254945_83255145
aatgttaatctaatttgattggaaggttgaagcatttttttcagatacaaaaaaaaaaaaaagcc
actagctatcaattttcagtttatatcctttgccctctttaagaatgagttcttatactgtggct
tttcctttacatcaataagccagtacttattgtcctctagaaagagaaataccctaaaagaaaga
atact

>chr16_31646899_31647099
gcaccactcccccagcctcgctgccaccttgcagtttgatctcagactgctatgctagcaatcag
cgagactcagtgggcgtaggaccctctgagccaggtgtgggatataatctcctggtgcgccgttt
tttaagcccatcggaaaagcgcagtattggggtgggagtgacctgattttccaggtgctgtctgt
caccc

>chr17_34970487_34970687
aataaatgtgagcttttcaatgcgatgatgacgatgacaatgatgacgatgatgatgaaaagagg
cttttgaagctaaaaaaaaatgcccaagaacgcctcctcctcccctcaccctgctctctgggggg
aagggcaagtcccagctcattactggtgggggctgcgtagggggaggtgtcagattggaatcact
cctgg

>chr18_67889020_67889220
gaaagcaggtgctttgtagacaactggtcttaatgagcacacatcaataggtttggctgaaaacc
ttgtaataccaaagagcagaatacttaaatcaaatatgaaagtaccatagttgttcagtaaatta
aggcacactgcccggaaactgtgcacttttctgttgtcttaaaatgttgtgccaagtggcacaat
gctca

>chr19_37283160_37283360
gaggagtccttgagaagagcctctccagccaggaccaggtaagggaatctgtgcacttggccaga
cccagaacacacagtggcagggacctgacagccacactcttaccccatagaatctccaccactga
cacacagatcaggatgcgtcaccatgagagatgacaccgcaaatctggctctcacggattgattc

catac

>chr20_53883793_53883993
gcagagaaagtctcctgggaagagtgtagccatgagctatgagcaccccacaggcctggaaattg
gaagttgatgcaccattctgatgcaggacaggcaacccctgggcttagcccaggaaagttcttag
cttcacccaggaaagaatgcaagggtgagcaggtggtgttagacagcaacttttattgatgcagc
agtgc

>chr1_149657176_149657376
ttgtaaaatagtatgataatgtaaagaacaagtttcaggccaaatttcttgttctcccaagaaaa
ttgtttctttttctttgagccatctcatttgaatttgctccaattgccttaaagacaccctagtg
gggagtcctttgggatgctcgcctttgtccccatgtctaacaaggatctctactacacccagaag
ttttt

>chr1_208271977_208272177
cagatcaatgggtaatgaatttgcagggaaactggagaatgactatgccctttacaataggcatt
gccatacgtttgctataaacataaagtctccttactggatttaaagtctgatcaatttacagata
cttgtgcaagcgtgaatggagcacacccacacaggtgcatgcactgtattccctctccccatgcc
cctgg

>chr2_168244154_168244354
acttgcatatttcattttaaatacacctatttactagtgaaatcatagaatgtgcttatttacgt
gcatttaatttaggttaaataactaagccatacaatactgtggtcatattttgtttggaaaagga
aaaaaaaatagttagtgttaatctctactgccagtcctcccacaaaaggctaaaaagttgcctta
ctttg

>chr2_237310861_237311061
tgatcaagtcacctcctggctcaagtgtccccaggggcttcccacagccccctgagaccacgggt
taccagctcactcagaggacacacctcctgcccctgctgactctcatctgtggtctattcccctg
tgaatccaccacgtctttttttcagctttgcatttcagctttgcatttttcagctttgcattttgc
tttga

>chr3_123052310_123052510
cagcaagagtgctgggggctccacgcggctgctgggcaggggacaaaggccctggcactggaggg
ccgaggacgctgcctctttctgctgctatcctccttctgtgctctaacggcaccctcccccagcc
caggaggaaggcaggggaggggcgtgaggcctgggtgtgtgcctcaggagggtgcgggacagggg
gccag

>chr4_55383843_55384043
tgagagcaacacattgttcactgaaaatctaccagtttagcttccctgccttccttgccacagag
agaaagaagggagggaaaaaagaatgatttgcaaagcagcattaatattattctgaggccaggtg
aattgggatgtgggtgagatcttgagatgaaagttcagctgcaagaaaatgaaaaagcagccata
atctc

>chr4_88855376_88855576
ttttctaggatatccacttggtataaaataaaatatttcctgtcatatgcaaaacaactttcatt
tcattcttgagtggtgcttacttctgatcacaattgtgtataacagagatgtccaatcttttagc
ttccctgggccacactgaaagaagagttgtcttgggccacacataaaacacactaacactaatga
tagct

>chr5_126629501_126629701
ccaggtgaacctggtaaagctgacgtcagatcacatttggagaaacactggcttacagcataaag
tctcacctccttaccgctatacacaagaccacctttgcagcacttctcagggttattttctgctt
ctctggtccccagctctccagctcccactgatcttgatcttgttactatctcaagaatacgctgt
gcact

>chr5_150053207_150053407
gattattatttacagctgagatgaagtttggacaggggagagcttgaaagggatacatttaccat
tctgcactgagggcctgaaaacaaaccataggaatcaagacaggagcttggcactgggggaaggc
cttgggctgaggagtgcatgttaactgcattggcttccttggtctggtgctatttttgaatatgc
tcctc

>chr6_160554210_160554410

gcctgtgacagcctctgctgctaggcaccgtcccacacatggtttgggggtctgacccggggcc
acatccacgctccaacaccacccgcagcccctccacccacgactcccctgacttccttttcccct
tttcatttttgttcccgttacacgtatcacgctctaacataggattgcattctcttatctgtgat
gtgga

>chr7_2505474_2505674
ttatctgtgctgatttttcacttcttgttctgaccaggagacgcctggctcccatgatgcacaac
ttacttatttgttcaatcccagtgttcgttgtttcagaattgctaagatgtacccctgggagaac
caaacctactaactagagtccagtgtttgtacacagttggttttgtcctgagcttcaggatgtag
acaaa

>chr7_23148475_23148675
gtagcgctacttcatccttgtggtatatagtatcccattgaatgaatatagcacaatttatccat
tctatcattatgaacatttgggttatttctagtttggaactattaagactagtactactttgaac
atttttgtacactttgtacatgccttttggtatgcatatgtatgtattaatatttgtgttgggaa
atacc

>chr7_51424706_51424906
ttgcataaatcaacctcatctcttcccgcccccaggcatactaaatcacactgtgatggcgagtc
ttctccctttttgccgcctgtgctaactcaggtggaacctgccccttaatgtgccacactgggtta
ctcattccctgggcatggtgacctctgggttggaaactttctttggaaatgtgctgcccttcgta
cgctc

>chr8_90163684_90163884
gttagctggttgctcagctcaggcatttttttatgtggccattcttagtcttcataacccatagc
atctccctaagtcccttgatgaactccaactttctccctcaaacactcttatcaaaatatagctg
ttatttattgttttggtccttttctgtggggaaataattgtcaggacccctggtcagtcaccttt
ctgat

>chr9_126138579_126138779
atgtagaggctttgatggggaatcctgggagcctgtgactggagacaccagggaggtttgctggg
ctccccgaagcctgggccgaggctcgcagggacaagctgtcataacagcccctcggccccccctgc
caccctgcccccgcaggtgggccccagtggattctgaccttgctattgtggcagggggccgctca
gcctg

>chr9_139033979_139034179
ctacagcccagggcccgccctccctgcagaccggacagcaacgccacctctgggagactcgtggg
gacggggcccagggcaggctcacagtgggaggctggcagcacctacccggggcagggcctcctcc
tgccactaccacgtcaggcatcactgagaaggaggcaaggcctagagcagtcggcaccctctgca
gagct

>chr11_66307424_66307624
agcccgtgctgggtgacaagaacgcactggtaaactgggcagacgcagccccctacttgggatac
ggcttcattcctgcatcacagacttggtgagcacctcctcagccctggaggcggtgccaggcact
agagggtgtctctgcccttggggagctcacagacattaaagaggccattcttggctggatgcggt
ggctc

>chr13_114271399_114271599
aacttggactagcacccgtgtgttctcaggagcgggtcctgctggaggcccagccacgtcaggca
cagtaggtgtgtgatttgtgcaacggaggcctctgataactgccagagtagatagcttttgtgtg
ggagctgcctcagcctgcattccaggagctcaggtttcctgagtctgaatggagtctggagagag
caatg

>chr14_76876047_76876247
agggccccccctaatttggtttgcaaatacattagccaaacaagctgagggggctcctttggggag
agggtttttgggggatggggatgggagatggtgggaaggctgaggaaatgaagaagggaagggtt
ttctgtgcaggtcctggagtataaaccgtacatgcgattgccaaggctgtgcatgtctcatggtt
ttacc

>chr15_86775396_86775596
atgttatgtcttttgcataagtccatagaagagcttaaaaggtttaagctaaagggatactacct
gtcctcgtcgcccccatccctgtccccttgggacggtcagacccagtaggttgggcggccttgg
gctgagccgcagacagtaaagcatatcgttcctcttccggccatgtggtggtgctgttcttttag

ttatc

>chr15_97133396_97133596
cacaattaccttttttaaaatgccaattaacccattcagtgctgtttggcatactgtgagcagtca
gtgacagcaatattgcacactgtagtcctacagtaaggcatacacatacagaagcacacacatgt
gcacatgcacacacatgattaggactggagtgagggagggagggagaaaggaagggaggtagggg
aggag

>chr15_99143477_99143677
gcccctgtatttcttgagctggagagaattgggcatacaggtatgtttacaaagactgagctcgc
ggaagaggttttaaaagaccaaaaaaaagtctcaatgggaaaagataccttctgccctgcctccg
ccacctcctgctcctcctcctctctctctcagctcatgcgcatgtacacacacacatacacacac
acaca

>chr17_76671205_76671405
aaatgaaacaggttatccggaacctctgtcccgataaccttcctcacagttttggttattaggta
tttagctcaaggaaaatgctgactttctactttaaagcccaacaagaacttggattctgaaggag
gaaaataacgtaagcgtccagtcagctctctcctctcctcggcgctccgcaggcacttgggatgg
aaaag

>chr18_55115202_55115402
gccacacagtggagtcatatggcactggccagggtgagaactgacagtaggcactgacccctgac
tctggcctttttcaatcagttgctttgctttatttctggttttattccagagaaaagagatagga
gagtccttttggtcaatgattgagcacaactcacatctctttgggtttcgagtaaaatggtcctt
gactt

>chrx_1474800_1475000
ccagtccttggaaaagctgaacgcctggtacttcagagaacaaaagggtggttctgaaggctgct
cccaggacagtggggctccgagggtgcaaccccaaggctcactcctcccagtgcccccaacgcgg
ctcagtcctgtgtctctgcctgtagagtttctttgttccctcttccttcctggtgtttttctctc
ccgct

>chrx_2161000_2161200
ggaccccctttcctgtagcatcttcaggtgcagccgtgaatcagagaagcagaaggggcttacct
tgaaaagcaaccagccgagaagcttcttcgccagacgggtggcccagaacacgtgcttgtagacg
tccgtgttgaccaccccggggtccaccacgttggcggtcacgtggcttccctcagccgccagcag
ccgct

>chr1_7945413_7945613
cccatgggtctgggaggtggacaatggcggcccaaacggaggtttcatgccagcctcagggagta
tgggatacttcctcccaggaaggacaggagatggccccggtataggccggccttaagacaggaca
aggcagggaaccgcgactcgggatccttaaggttacctccagcacaacccacccagcctccactc
ctgct

>chr2_205395555_205395755
cttaatgaagggttcttcagctaagagtgcttgaaacatattcttcccccaccgaggagagcatc
agccagcgagatgatgaagtcttctggtgaggacacctcctagggactagacaggctaacttctt
ggtcccatcctcttttccctcactctaccactcagagatttctgttaaccctttcttctcttagc
tttgc

>chr2_233470556_233470756
cgtaggttgcagtgaaccgagattgcgccactgcactccagcctggccgacagagcgagactcca
tatcaaaaaaaaaaaaaaaaaaaatcggttctgagcaaatttcgagtcactgacaaggcggcggt
atccagcagagggcgcgccaggcgccagcaccaggcccgaggacgcctggctcatccccaggccg
gcttc

>chr2_236923461_236923661
atggaaactaaaccgtgttggtagcctgcgaaatatatacagcagcagcaccaacactgaaggta
acgcctccgcacctccacccgcccacacctccacctgcctcttcccctctcatcccctgcccgcc
ccttcctttccaacacaggcatggaaatgcaatttgcccaaagccgcatgctcccttattgtgtt
tgcag

>chr3_107384710_107384910

gtcgttgagtctgtctgtatctggtgctgaaggcaagttgtttcttcctttctattgtggttcct
ctgcttgggacacctcaactccaggtgaatggtgagtgacagcctgacttctttccttgcacctt
catgtagtctttccattaactattgcagggaattgctaaaccactttcaaggagttatttgactc
cagat

>chr4_4398499_4398699
cagtaagcccctggtgtgtttcaagatgcatttcttaaatatttatgaagagtgatgccgtttcc
ttggcattcagcagtgggggtgggggttcttcccgtgggggtggtattggagaccctgaaggtgt
gtgtggacgccccctcacctttagctatgcgagcatcttgtaggatcctggggctgtggttgcag
agccc

>chr4_109838151_109838351
aaggaaaagtttaatggtaatatcaaatactgtctaagggctttacatctatttactcccttaac
tctcattgactctattaggtaggcactgccattgtctccctttacagatgagaaactgaggccag
agaattccaaggactttgccaaaattcacacagcctctgagtggaagactcatgctcttagcaac
ttact

>chr5_57246643_57246843
ttggatgcaattgtggcagaaagaaacaatgagtaacaactgtaactcaatgccagctgcagtgg
ctgaaattcatcctattaacctctcacttgtttttccttgagaaggaaggcccccaggaatcctg
gaggagctgttgcctgaatgtgtatgaagaaagaaacctgtgtggcacaaagggtgagctgtgat
agcaa

>chr7_51945506_51945706
atagttatgcaaatatgctgttttcatacacgcgtatacaaagccttggtaatttaggactcatg
atgagtgtgcaggctgcaggctctacttgagttttagagatgtactattcatctgcttttacaga
gtcaaaggaggagcagatcacagacgtgaagcccatctcatcactgagtaaagatgaagaccatg
ccagg

>chr7_107577164_107577364
cattcttgaatgtgaaaggatttgcagaagtattaataccttaactattcaaaagaaccccccttc
caagaaaaggccaggggattaaactactaactggagggaaaaaaaaaaaatcaggaaatggatac
cattctgatacaactactgccattcactctgtgggacctttgctatgtctcttaggttctctgtg
cccaa

>chr9_138949179_138949379
cagggatggctcagaggaagagaatgactcgaagggtggggagggacagctggggctggggtggc
tcctgttgagccccagatccgccctatggccccaccccagccagcagacccactgggtccacaca
ctgtgggatgctccctcacaccacaaaacattctagaaggtttccaaagaattaagctgtgtttc
gaaaa

>chr10_49525194_49525394
ttgaagatggttaaaaaacaatgaatgatttttctttttaaagacagggtctccctctgttgccc
aggctggagtgcagtggtgtgatcgtagatcactgcagccttaaactcctgggttcaagtgaatc
ttcctgtctcagcctccagcctcccgagtagctgggactcggctggcgaatttttaagtttttttg
taaag

>chr12_19119933_19120133
tgccccaaggccccattttatagagacagactgcaggcccaggctaaatgagagattctaaaact
cacagggtagagccagcatactgcatttggtatccaggtaacaggtaacaatggctaacaatgtg
atcctgagaaagggacaaaagcctcctccccatttctaaccttgctgcttggagacctcagggtg
ggtac

>chr14_73447447_73447647
ttgtattttagtagagacagggtttcaccatagtctcgatctgctgacctcgtgatccacctgc
ctcggcctccccaagtgctgggattacaggcgtaagccaccgtgcccggccgagaatttattaaa
tttattaaaatgcaaactcccaggccctatctcagacctactgaattagaaactgtggagggggc
accca

>chr15_51115508_51115708
cctggatggggccagtttatgggtgtctccactgacagatttgagcagtttgaatttgataggga
atggagattggatgactttccagagtaagcaagtgatattcgaaaaagcatgtttcagaataata
taagtagaaacctaaacattctcttatgatctttgcaattactgccagctctttcttatctacat

caagg

>chr15_101587477_101587677
ccatcgtgacagtagcagcctgtgctagcggggagggagatgggttaatagcgccagaagggtgc
actgattgaaggagtgggcgcaggcgctggtgctcctacgttcccattcctctgccctccctttg
aactctgtgtgccgctgtcttctcaagggagggtcatggagcactagcatcgtgggaggctgagc
tgaca

>chr16_7268999_7269199
tggtcacaagagaagaagaggtgagaagggctcggagccagacaaagcacgcctttgtaaatgaa
tgtggagttggccaccagcagtcttcattatccccgtcattgcaggcagctgagctggctttgtg
tcagccctgctcggagtagccttgttaattcacccccatgaaaatggaagtttcatacacattga
cataa

>chr17_73840805_73841005
gaagctgacctgggcccgggggggttctcaccccacttcctcgagtgcgtccagacgtggggcgg
ggcggggtggggtggggtgtggtggcgcccagggtgggggctcccaagaggcgtctccctggagg
acagccagttgctcagcctgcctggccccacaggtgacattccttggagatgacggggccaagct
gtgct

>chr21_34178530_34178730
ttctgtactgagtacagctcagctctagcacagtgaggaatgagggacagatggcactatttgac
cctttctcaagaacagactggggctacccctggattttgctctctggtcatcaagcaacagggag
ccccatggccaaggtaagggacagtcagcatccagtgggggctggggacagaagcagatgaaagg
accat

>chrx_137369334_137369534
aattttgtggagagtaaatgagaagattcatgaatttagcactacacctggatcatagcagtaag
ggtttcaataaatgttagctccctctctctttcctgctccctctcccaggcatccatacagcctc
accacctttcaggcagccctgttaaaattgctggagcaaatatttcatttcaggctgtcttccct
cagca

>chr8_33343858_33344058
ccaggagttcgattccagcctaggcaataaagtgagactccatctctacaaaaaataaaaaatta
gctgggcgcggtggtgttcgcctgaggtcccagttacttcgaaggctgaggcagaaggactactt
gagccgaaaagtccgaggctgcagtgagctatgatggcaccactgcactccagcctgggtgacag
agaga

>chr9_133872379_133872579
cctccccgcttggcacttgcagttgcttcttcagttgtacccttcccacttcccctccggagacc
ctctccaacgccactcctctccttcccgaatcagctctctctgccggtgctaaggacagctccct
gaacccctgaaggaaatattcaggggtccctgctcccagagcaatcccagacacaggtgatcctg
gggta

>chr1_29609813_29610013
cccatagtcccttctcccatagcccctcccccatggtcccacccctcccatagccagccctcctg
accccaggctccttcccacagagcactacccccttcctgagccttctttctcaggctcctccttt
ctatccaggtccccagtctagtctctgccccttcctgaggccctgccttctatcttagatgcttc
tcttc

>chr4_120589152_120589352
aagcagatgagctggaggttaaagtggcagcgtgctgtaaccaagcctccaaatcaagcccttgg
agccgctataagcagagctagctgtgcaaagctgactccacatatgattgccctgccagccttgt
gaaggagacgaggcctgcctggccaaatcagacaccacagagtgcagttcaaggagaaagatgag
ccctt

>chr9_75829180_75829380
atagttcaagatctccgacactgtgtcatttcatagagctcacacactctgaccagaaggaaaga
tgggttccgggaagctgaggctactgaagattatctgaattctggttgaaaggccaaaagaaaat
gtgtattatggaaaagtgatgctcttaaagcaggaagtggtctctgggccatttacttgttgtgt
gactt

>chr7_54256106_54256306

aagaatttgaagtaagggatgatgattcctgttatacgtaatttatcctggctacaatgtacaag
gtaaagaggtcaagcaagatgcaggcagagaaaacaattaggagaatgcaataacagtcccaatg
atagatgaggcttacttgaacataaacagcactggtgaggaggaggcctcggaggaaaatatcta
agtac

>chr2_50449496_50449696
aatcattctatggagctcagtttcctaagaaataatgagaccaatttgcttttatatgaaaacac
tccagtttgtgatatcttcatacactgctggtgggagtgttcaacagtacaatgttcttagagga
caactcagcaatatatacattggttcggcttgtatgaatttgtacataaaaatatgcttttacac
atgtg

>chr10_43746194_43746394
aaagcacatgaggggcattgtggctccgctgttttcccacctgtcacctggtcaatggcggcttc
ccagctggaaggggcagagagggctggaggcttgggagtggtggtcctggggaacaaggctcaat
ggacccaggcactgacccgcaaccctgctgaagccagagacacctgggtggaggtggagacgtgc
aggca

>chr4_65714605_65714805
tggtgtgtggtggggaggcggggcggatggtttgggtttggggatgaaactgctcttcctcagat
tatcaggcattagattctcacaaggagcacacaacctagataacttgcatgcgcaattcacaata
aggttcatgctcctatgacaatctaatgtcactgctgatttgacaggaggtggagctcaggtggt
aatgt

>chr13_111894199_111894399
tttaacattgaaaaggacttttctgatacgtctgtgacagatcttatttcatcttatttagctca
agatctcattatgcttggctggcttcccctgttgttctggaggacaggcctggtttcgagtagcg
tctctggggtgtggtggctggcatcacggtttacctctgctcaccagacctttgacatttgtgtt
cgacg

>chr17_47953401_47953601
gctctcaggagtgtgcacttccacccctgatacagccagggatgagctgtgggctggggcagaca
gagcccacagatcaaagcactggcctcctaggtgagacgggacctcagctgtaggcaaagcggga
tgtttcccagttagaattcacagatgagcaatctgatccctcacctttccctcccttttccctt
tattc

>chr1_42220013_42220213
acaagagcacaggagacaaggatcagattgcaatgctgttgggaggctatcacaacaatagtctt
tgcatgctagagatagcagcaagtgagccaaggtaatggcaatggaggtgagaagtggtcaactt
ttgagtacaatttgaaaatacgtttgggcttttctgcagagcgatgcctgcctgccgtacgtgtg
gctgc

>chr10_34151194_34151394
cttacggccgatatgctaaccttgaatatctaacagacttttaagaaattcttaggtttatgcag
actatcatagccccataacacataacacagccccataggccaacacgtcacctacattctgctca
tcaaataacactgatggagctatcggtcatgctggagccagcgtggactcctgctggtttccata
tcggc

>chr9_99018379_99018579
attagtgacaaaggccccactttattgaaatctgtctctgtggtttcagtcaaatgattcatact
gttattttctttaattgtggtaaaatacacataacaaaatctaccaccttaacaaaaatgtggaa
tactttacaaatttctgtgtcatccttgggaactcatgctaatgttctctgtatggttccaattt
tagta

>chr11_27761624_27761824
tatacaagatcatacaaccagtaggcagcagacctggtgttcaaagtcagggctttctaattccc
agatctgtttgcatgaccactcattcactagccatattgtgagcccagagagatgcattttgaga
aaaaaaaaatgccatgtcctctcccttactttatttctatatttctaacaccagtttaaatggag
gtctc

>chr11_73209552_73209752
ggaggggcgaccagcactgggttgcagtggcccactgtctaagccgtttgagctccttggggagg
ggcagcaaccagcattggaactcgcaactgcctgacatgctaagctccctgggcagggtaagggc
agcacccatttctatagctccaggctgtgtttttcccctgctggagcccgggaggctgaatggct

tggtc

>chrx_152532406_152532606
aggtgaattgagtgaaaatcagtgagggccccttctcgatgacagaagcgaaaaacacaggctcc
ttcaccagggcgggccattgtgttcctattgtctaccaaacggctctgctctccaggtcggagag
cggtgcaattgttcactaattgcatcctctactgatgcatgcaattagtgaacagaataaggggga
aaact

>chr7_17961275_17961475
acctggggaagtaggggggattttaaaatcccactacccagcttacgccacatacaaattaaatca
aaatagctaggtatgtgagccatgcatcagaatttccaatgttcaggaaagctgatataagagca
ttgttagtttgtgaagttccccagatgattgcaatatgcagttacttttgagaaccaccgatgta
tgcac

>chr20_24474800_24475000
gcatggccgaggactggatcactgagtgcagattgagaagcacagcagggcagtatgtgtgggac
ttgtggtgtaagggaagatagtgaaggatggaggattgagcgcacagtgtcagggaagctgctgg
gggcatgagcagggaggaatgctgagaagagtggccttagaaccacacagctagaggagctggcg
ggtcc

>chr1_204803577_204803777
tcatcccctcccggctcctgctgctctcttcctccaggaagcagctgtcttagcatctgccttct
cctccttcacctgctcctcctcactcctcccctcccgggctggcccagcccctcctgaaaggcct
ccctggcagcccctctccccgttattgtgatgctaatcatgttgttattttttttttgagatccag
aaaga

>chr4_189353606_189353806
gtgagaatatcacagcctattctaaaaactgctgaatcattgagtgcatatagttggagaagtgg
gtggagcagaacagcataagaagagagtaaagaagtgtgcagggtcagattacatgaggccttat
agggcgtgggacagagtttcacttcatcttaaatgcagtgagaagtcattggaggatttcaggtg
cggaa

>chr15_42373108_42373308
ccccacacaaagtgtccctctggtgcccactctgccccgctatggcccctgattgtctctgccac
ccagtcccccaggatcctcaccacgtagagccagagatgccactgaagtaggtcacacagtctag
gaggcccagcttctgcaaggccaataggtggccgtagagtgaggtcatggcccgggcacctcctc
ctgtg

>chr15_86407996_86408196
agaaaatctagaagctagtttttttttctggctcctcctcccgctctccttctaaggtagtccact
ccctcctctttctcactgtctctgatcgtgaccctcccacacctgctttcaaagctttgtctgtg
aaccaattagaggtttgaaaagctggtatcagaagtgcccagtatcatttttatgagacttcatg
aataa

>chr8_56822046_56822246
tatctagtcatccgctgatggacatttaggttggttgcatatctttgctattctgaatagtgctc
caataaacatacaagcacttgttctttttaatgtaatgatatcttttcttttgagtagataccca
gcagtgagattgctgcatcaatggtagttccagttttcattatttgagaaatctccatactgttt
tccac

>chrx_104195544_104195744
cgcccggctaattctggtgcgctgtttttttaagcccgtttgaaaagcgcagtatttgggtgggag
tgacctgattttccaggtgccgtctgtcacccctttctttgactaggaaagggaactccctgacc
ccttgcgcttcccgagtgaggcaatgcctcgccctgcttcggctcgctcagggtgcgtgcaccca
ctgac

>chr19_54675788_54675988
gggtctcggtaccgaagggtggcagcactgggcagctcgttcagcacagaagacagcgatgggcc
tggggaggagcagggggctgggaagacccgggagtctgggccctaattcctcctccctcagacca
ggaaaccaggtccccggcccctcctccctcagacccaggagtccaggcccccggctcctcctccc
tcaga

>chr6_83789481_83789681

aaaatcattgtcattaaatgaagaatgatagctacaaataaagatactttgagaaaggtactttc
tgatgaaaatttatgtttcaaataagctggaaagaaatacatggtgggatagttaacagttagtt
attcctcatcttgcgtttcacctctctaatcattctgaatagcaatgcattcatttttatcatta
ttctc

>chr2_191942955_191943155
tatttgcctgtttttctcacagttgcaggaatatttctatgccgatcattcagatactacctttc
aacatttctcagttgtgttgtacactttttcattttcctttcttacttgaacttcttctacctt
ccttttcagattcagtttcccccatactttgtatggttaaattactcactttttcttctttgtta
agagc

>chr4_101467177_101467377
cacagggcagcgacagtgagagatgaaggcaacatcagtgtgcgccagagttttgcaactccaca
cgcagtccatcatggaccaacaactgcagcatcacctaggagctgttagaagtacagactctcag
atcccaccccagattcctgagttggaatctgtaatttgataagattctcaggtacattaaagttc
gaaaa

>chr1_57098812_57099012
ggaaatgagtatgggtcacttgggttccatagagaacagagagcagaattttgtaaaacatcagg
aaaaattcagttcctgcaacatttccatttgtcgccaaggctagcatggtctgggaaaaaaagca
taatttttcaaagtaaagttattatttatttgggatttagtttatgaaggacaatattgtgttc
ctcta

>chr12_39786733_39786933
aataaaataaagacaataaaataaaattcagattgtgggaaaatatatactataaatgagccata
ctagatgtttacataaaacaaggatccacattttaaaagaaaatgtttctgaaataaatagcatt
ctaaggaaattgctgtgtagggaacccagtttttggtcttctgaagatccttcagccctacctgc
atcac

>chr16_14805899_14806099
tctaaagccccagggttgctaagggagggcccagaccgaagaaggtttggtggaaagcagaacct
ttgtctccctctaattgctcctaagcctcacgctcccttgccctgcctgtcctgttgcttccctg
atcttctccgtgacctgtagctaaaccttccaccagcgcttgagaacttaatttgaaccggatcc
tttcc

>chr10_72834194_72834394
atgggcttttcccactgttggcccctaagctggttaacagagccacagcagaaactgtgcctgac
cctgcactgacaaattctggaaactccacagcctcggaaggtggtcaaacaacaaaaccactcct
tccagccccagggcattcaagatactatctctgtggctttctgctcatgattttcattacaaata
agcag

>chr13_114214799_114214999
tcgcacaggtctggacggggtcgcgaaggtctggacagggtcgtgggtctggacagggtcgcagg
tctggacggggttgcgcaggttttgagatgggcatttcctcccccagcatctcagggtgttcttg
gcagcccctaccccaggacaaacctagtttcctgtgttcctggtgtttgttttacaaaagcctaa
gcagc

>chr6_158470612_158470812
cggttagacaagtccaataatacaacttgtttaggggatggagggacccagcctgaggccctgag
cccagggaagcagctgtggcaagctgagcctggtcatggtgtccctgaggctctgcaggtatgga
catgcctctgctggtatgcatgcagctctgcaggtggggacacggctctgcaggtggggacatgg
ctctg

>chr3_8155600_8155800
cctgcacgtacacatccagatgatcggttcctgccttaactgatgacattccaccacaaaagaag
tgaaaatggcctgttcctgacttaactgatgacattgtcttgtgaaattccttctcctggctcat
cctggctcaaaagctcccccactgagcaccttgtgatccccactctgcccgccagagaacaaacc
ccacc

>chr9_94108779_94108979
tataataaactggtaatagtaaataaagcactttcctgagtttcgtgagatgctctagcaaatcc
cattcctgagaccaattatgacgtctttttttctaacaccacttctgacaccaaatgtctgaggg
tttccacaccaattcttcaacatcaagcgggtatccaacaattcagttaaattctgccaccatcc

aaaat

>chr1_110040877_110041077
cgggcaggcgtgctgacagccggcagtttgcgtgggctgtgccatctgatgtctattcccagccc
tgggaggaagggggagtcatttatattctgcaggaggaaggggccccagctgtcgcctttctgac
cagcaggcctggagggcaggggcacagagcagagaggagggcactggtggtctcctgcttagcct
ggtct

>chr3_13204400_13204600
cccccctatcccctttagctcccactcttgccagcccaagtgcaccatccccagctagtcccctc
acctgggcttcacccctgcacacagtccttctgggaccctctctctctccagttccccttctgag
catgcctctgcttcctgcgagaccctgcctctcactccctggactctggttccctggagcccaac
tccct

>chr1_14730813_14731013
tgcttatctgtgtctgcagctcgatttttcaggctgctctttgttagaaaagaaatgatttgggt
gctgctttttgttgaaagggaaattccactgcggactctgttgcccttactatctgcctaaataa
tttctttctatctcccgtatcagtgtgctggaacaacaaggtacctggcaggcctaaacaacaac
aaaaa

>chr10_88614820_88615020
ctgggattacaggcacctgcctccacgcccagctaattattgtatttttagtagagacagggttt
cacatcaggtctcgaactcctgacatcaggtgatcaacctgctgggattacaggcgtgagccacc
acaccctgcgtcgctaagtttttaaccagagaggcttcaggtttcagacacacagaagcataat
tctgg

>chr13_62659799_62659999
agtttctttctcttaaaccacacctaagcacagattacattgttaatgcagtgatgaaacagaaa
aacatgattcccactacccctagaagaattttaagtatttctccttttaaagtaaatgtcaatct
tacactatttcctcgtttcctcttaaactttctttgaggtgtatgagttcatacggaactcttta
aggtt
