# Supplementary material for: A machine learning technique for identifying DNA enhancer regions utilizing CIS-regulatory element patterns
Source: Sci Rep. 2022 Sep 7;12:15183. doi: 10.1038/s41598-022-19099-3 (PMC9452539; doi:10.1038/s41598-022-19099-3)
Supplement: Supplementary file 3 — Supplementary Information 3. [file 41598_2022_19099_MOESM3_ESM.pdf]

Online Supporting Information S3: The independent dataset contains **400** DNA sample sites out of which are **100** DNA Strong Enhancer sites, **100** DNA Weak Enhancer sites and **200** DNA Non-Enhancer Sites. See the text of the paper for further explanation.

I. 100 DNA Strong Enhancer Sites

```
>chr11_6627824_6628024
atgctgccagaaggaaaaggggtggaattaatgaaactggaaggttgtggtgctggtttgaggag
taaagtatgggggccaaagttggctatatgctggatatgaagaggggttaattccttgcaggtc
ttcttgagatagaagtccaggccctgaggtggcaggcagcctgatagtgaacagaacccttgtgc
ccata

>chr11_9587224_9587424
ggcatttttaacctgtgtttcattttcatctgtgaaatgtgaataaaaataactctcttacaga
gttcccgtaaagattaaataagtatgtaaagcatctgggtcagtgcctatcatatagttggcacc
aaaatattctcccctctcccagctcctgcaaaggcaccccagctctttgcagcacttagggcctt
tctca

>chr11_65187024_65187224
gaaaccacagagctgacctggcttcagaacaagatgtggggctccaggcacccgggagaccagtg
accctgattggcaatagccgcaagaaagggcttggagacaggggccaggcgcggtggctacccct
gtaatcgcagcactttgggaggtcaaggcaggtggatcacttgaggtcaggagttggagaccagc
ctggc

>chr10_74014594_74014794
tttgcataggggcattaccactggacttgggctcagagcaagtgtttatgagataaatgaggtgc
cgtctcagaatcacagagcactcctctacctttgaccttctgcaggctttcactggcttctaaga
gccaagaaccttctccgcccccacccagctctctctcccctgcaatctctccatgaacagcattg
tttta

>chr10_105667810_105668010
cgggaggcgggggttgcagtgagccaagatcacaccactgcactccagcgtgggccacagagcga
gactccatctcaaaaaaaaaaaaaaaattagcacatgaacatttgtcctagagaatgcgaggtca
ttctggtcttgactgtagccttccaatattctaggtctaagctcgagttgctaccaagtgttctg
tgcac

>chr16_87812299_87812499
ccacaggcgtgtaccactacgcccggctaattttgtaatattttgtacagagatggggtctcatt
atgctgctcaggctggtgacaaactcctgggctcaagcgacccacccacctcggcccccaaagt
ggtgggatcacagacgtgagccagcgctccctgtcagcgtttcattccctcttcatatcacatca
cttgg

>chr14_61570047_61570247
acttagcacctgtcaatctcctgtcctctgtgaaagagcctgcatttctccctgttgggcccgcc
acttctccaggatttttcagtgctgtctgtccaagtctttcttctgctggaagtgtgggaatgg
gaagtcagctctagccatggctacaaataattcacctttccagcagcctgcgggttttcttctta
ccggt

>chr19_39177160_39177360
agcctctgctgctgccctaattttaaaactgccttttgggagtgtaagtttcctctgttaaaggt
agttatttcaaggtaggcctcaccatctcctcctcctggtgagaagctctgcctggagggctgag
cactgcctcccgctctgtgggccccacctgccttgggttgagacctatctcttcctggactctgt
gtggg

>chr22_36726454_36726654
ttggtctgttatcttgtgtggcctccaataaccttacaaggtattgtattgtaatgtacaaagca
ggtattatcatcattgttttgccatctttccacacagcggttcttctggatttgttcctgatctc
agaggcatctcttctgggcagtggggtggttagaattcttagcaaccagtgggctctttggccta
ctcag
```

```
>chr20_30160339_30160539
ggcaccgtcagtctgacagcagggctggctcctgcagtgtgaatgaagcggtcttgtgctcgact
gtgaagggggttaggacctgcttcccagggcgggtccagctttctgagggaaagccttcagagc
ctccatcaggccggcaccaggtaccagttaagcactgggcagggaacagaagtggtcaaacgccg
cttgc

>chr20_30160539_30160739
cgcgacttgaaactgctgaataaatggataatgtcaagcgctatggcttagggcagtagtacctc
cggttcctacgatcgtgtctggggtccctgaggagcaaggcaagagtcggccattcaggaggtca
gagttcagctgttcttgcacaagcagggaagctgagtcaccccaaacacgggcaagactagcggt
ggtat

>chr20_45977793_45977993
ctggggaagagagaatgttccgtggtctgggactgtgacaacgcaaaaggcccaaaaagacagcc
tcatgtctcgaaccctagtataaggagaagattttaggagagttccacgttaggaggagaggact
taggatgggagctagatagaatggaaaagattatagttcagagttaaattataggggcttggaga
cgaag

>chr7_104585764_104585964
ttccccagggactgggcctagctcaatcgggaaaagtcagcatccaaaagtcctcaatgacgcag
aggaagccaggagaaaacagtcaatggctagtcatcactgattcgtggtcacaggcgttagaaca
ggaaggatctgcacagatcagcctctcccacccctcactttcctgataaaggaactaaagtccgg
aaagt

>chr6_26122621_26122821
tgttctagacttgaataaaaagataggtagactacgtctaacccttcatcttaaaatctttacct
ggaaaagaccatgagtaaaatacttaagggaatgtggaatttcccaggccacaaagcggcctgca
gttgtctaggaagggagagtcctctaggagatacagtgtatgtgctaagtttaatgacgtctcct
cttca

>chr3_177077306_177077506
tgagcaatttgcagggtgagaacgggagcctacgactccgcacctgcactgccagagccataaat
gcagcaaatcatggcctataaaatgtcaccactgctgaagtgcaattttccaggggtcttctgca
gagggaactccaccacggagagcgaccctcggctctgcagcctaagaggaaggcccctgcagggt
ggggg

>chr1_150540176_150540376
ttcccatagcccttcacctgaggtgtgtgccccactttccttagatttgggctctcatgcaaaag
gaaataaacaaggtatcctcccctcaggaaagttcagtaacaaagcagtgaaaccgaacagctgc
tcccttgcagcaggccagctgtctcccctccccccatttccaccaagggccggagacggtgggaa
ctgct

>chr1_150540576_150540776
aaaccagctcctcctaccagacaagaaaattaacatttttttagggcacagaactgaaagcatttt
tactgaaatctaatccaggatgaaacctaacagttaaacattcttttccttctctgcttctttgg
ctctctatcccagcacaccttgtcccaggctaacttgagtaaacatacacatgtgaccacatgag
ggcgg

>chr1_156717976_156718176
cttctcacttgatggaatttctaacccttcacccaaaagaggagaaatcccttctacagtgacac
ctctatccctcaagctacaaagaaaatgtcctttcttacatttggaagtggagggcagttcttcc
tagatgtagaggttatgaggtctcagggcagccctgtaccccaatacagacatctgtcttccagg
tggtg

>chr9_131903379_131903579
ttttgggaacctgggccaggggagggaaatgagggggataaaatgacaggtttttttttgacttaa
atcttaaaagttttgtcacagttgcccttactagagaagtaagaggcctgccaggagtgggtcac
caggtggcagcccatggctcaagagtgggggctcctgagggctcttgtggtgaactgatgcccct
ttttg

>chr12_6999739_6999939
tgtatgctagcatggaagggaattgtttcttcggttgagtgcctaaagtgcttgatggcctttca
gatggatcatgtcatacagaaaggaagtatcttaaaacactacaatctagtttaagacataaaac
```

agtagaatcgctgtcagcgtcacaagatacaaacgaagggctcagaactgggaccaagagaacat
agtct

>chr12_45628333_45628533
atttgttcaggccccttttccaagttcccagggaagttactgacactgatgttggtttcttttaa
gaatgactcatgatttttcgagcctggtttccactcttagtcatcttccctgcctgagttaaaac
aggctttaggctctcactgaattccagccttgacagaacccccccacccccacccctgggcttttg
attcc

>chr11_9587824_9588024
tcactggagaagagtcatcacgtcttttgtgctgcagtaatctgaacgaagccagtttaaataat
cacaatgtggccagcaaacaactggcagtccctgtggtcattcaggtcatttcctgtgtatgtca
ctaacccaaaaggagaggcaattaatcaatcacctgaatatttaataagtgcctgcattctgttg
ggcac

>chr11_61582224_61582424
ttggtgcgtttttacagagcactgattggtgcattttacaaacctctagatacagaaaagttctc
taggtccccattcgacccatcaactccagctggcttcacctctcagggctatgagacttccgtcc
cttcttgcgggtctttcaataagtccctttcctctcttagctagggttctccaaaaaccacgaag
aggaa

>chr11_65187224_65187424
caatgtggtaaaaccctgtctctactaaaaatacaaaaaaaaaaaaaaaaaattagccaatgtgg
tggtgtaagcctatagtcccagctactcgggaggctgaggcaggaaaatcacttgaacccaggag
ggagggtgcagtgagcagagatcacaccactgcactccagcctgggcaacagagtgagactccat
caaaa

>chr11_65187624_65187824
acttgttttgatctagagaaatctgctgacaccggccttcctgctctcaccctccagctgggcag
aggtatttttagacttatttgtagaggaagagagcagaacccaggcccaatgtgtaccctcctta
ctgtgggcacagctttggccccttaaggcaggacagagatatccatgtggttgtctaccctcagc
tcatc

>chr10_105668010_105668210
tctgggcactgtcaccaacccccggacttcccagtttgtgacctctcccagcagaccctgtgcct
tggtgcccagaggtctggctgctggaatgcccctgaatggaggcacccttgcagtgctgcacaat
gtcctcagcagctacacagtaccgcctgggccgctggccttgtttcagctgcgggatgagtcagt
gcgag

>chr17_38268274_38268474
cctcaacattctagtttgatcgcttttgggctgtttcccttgaaaacacgtgccccagcaaggat
tgaaaaactacctattggtactatgctcactacctgggtgacaggttcagtcataccccaaactt
caccattacacaatatacccttgtaacaaacctgcatatgtaccccctgaatctaaaataaaagt
tgaaa

>chr19_18475800_18476000
atgtcccggcatgtctgagcaggaggaattccgggcggccaaagggttttatgtagattgctttt
ggacactcgcccaggagtcaggagttgatttttatctcacttcctggtaaccttgaacttaccag
gcagggatgagatgacgtaaacttcctcagatgatgtaagttcagccacatcttgctgcccatta
gggag

>chr19_18476000_18476200
agaggacgacccccacagtgaaagtaattgagtgaaatgcacgttctggaaatcagtttctattt
tggcctggaggaatgtggctatactgcttccctgagtgtgacacagttggaatctaatcttgttg
gtttcccaacatctaggtttttgtttgtttgtttgtttttatgatagggtcttgttctgttgacc
aggct

>chr19_39175560_39175760
cttaacccacccaaatggctttttgtttgtttgtttgtttgtttgagacggaatctcgctcttta
gccagggtggaaggcagtggcgtgatctcagctcactgcaacctctgcctcctgggttcaagcga
ttctcctgcctcagactcccaagtagctgtgattacaggcacatgccaccaagcccagctaattt
ttgta

>chr19_39176960_39177160
acttggctatctttttgaaagatcaaagtttaaaccagatgatctgtagtcccccacccccaccc
tgaaacctgagcttagctgtaagcattgaaagtaaatggggtgtttgtagctcaccctccctgtt
ctccaggtgaagggccccgtgtgcctgatcatttcatagcaaaatgctagatggggccagaggag
gcccc

>chr19_47612360_47612560
tgcaacatccattaatttgatttttaactaatactggtacatcagtctgggggaaaaaaggcag
tactgtgacatttcaaaaacagattaccaaaatacagtttttgacttaattactggggcttctag
gtcttaatggatgagatttctttgagaatggaaaacaataaaccgttgtgttgacatgcctgtga
gcttt

>chr6_43141422_43141622
tccagagatcctgataggacaccctgccctcagagtcattagagacgaaggtcattatgggaatg
ggggaatgacatcactgtataattctttttccaacttctcaaggaaggtagagataaaaagtttg
ctgacctgcccatctccctcctcacccctcaggcctatgagctgtccacgctgacagggacacag
gtgct

>chr2_87785085_87785285
ctagaacgtgacctgaatgtgaactagacattcgttttcctaaaccccatgagacccaggtattc
ttgacatgtgtagagaggattgaaggggcccgaggctgtctgtcctctctccgtgggcctggctc
tccttggaccaaatcgaatgcatgggaacacaccccccagttgtctttttccttcggtttatata
attcc

>chr1_161089176_161089376
aggtatcagggaaatagcgagggagaggtagaatctttgttggacagtgtccctgggttgccaga
tatgagggtagagccttgagaagtcagtgaagagttgtcagtgtcccttccccccagggaatgtg
gactctggctgtccttgggtggtttccatgagcgtggccaagactgggagcagactcagaaaatc
tacaa

>chr9_131903179_131903379
ctgccctcctggaggagagagcctggggtgattctgagtgaagcgagtggtccaagacaaggccc
ccagggaccagagtgggctttgtctctgttgggtgcctcgggctcttgcctggctgtggtggagc
ctgggagagaagaggaggggagggaggagcctgttgtccttgctcctgaacatgggtgtttggat
gggct

>chr13_99136999_99137199
cctgatccttctggggacgtgcaagcaacaacagaacatccagagctctctacccagagcaaacc
ccgcctcaggaccaaggccgcctccttcctggagaggaagatggagaaataacacagccttcccg
cttccctccaactcattcctggtagcagacgcgctgggtggcaaggctacccagacacaaggctc
accag

>chr12_31901933_31902133
aatatggtggttcaaaattagtatttttcctctttgtaggggaaatcacccaaaagtaacaatca
gaactgttaccacctgactggttcttgcccactgcacagataaaaccaaaacactgagccagcag
gagttgcagcagagaaagagtttgattattgcaaggcaatagagtgaggaggacgggatacattt
cccag

>chr12_109085671_109085871
ctcactgtcatcaaaaatagccccggggtacattgggaagttccttctgccaggactttgtgctc
cattgttaactgggggtgagcagggtgcccaccccacccctcccagcaaaactgtggataacaca
gtcactaggagaaaatgaaaaggcccttctgtggcacttcatgtggtaatgaggccaggccgtgc
tcgtg

>chr12_125402847_125403047
cctgcggtatttttttttccttgtcatgcactagtagaaatatttggctttgtttctttgtttttt
gaaacagtatctcgctctgtcatgcagtggtatgatcacggctcactctgcagcctggatgttcc
ggatcaggtgatcctcccacctcagccttctgtgtaggtaggactacaggtatgcgccaccacac
ctaat

>chr11_46576224_46576424
tgtaaagaacaaagggagctaagtcaaactgaagtcacacagggctgctgatgaggagaaaatgc
cagtcaattgtgtttttcattcctcttttaaaaggaagaagaaacaagtttcccaatctgtccc

ttaaagtaaagcagactgagacaaaggttaccaacaatagtctttgtgataccgtcacacagtcc
ccaaa

>chr11_47290424_47290624
agccctcgctaacactgtgctgtgtctgaagatcatgctgaccccacaaacggatgggcctgggg
gccactttgcacagggttctccagagccctgcccatcctgcctccaccacttcctgtttttccca
cagggccccaagaaaaattctccactgtcactctgtggtatggccataaatgcctcagcgccgcc
tcact

>chr11_62320424_62320624
ttcaagcccaaccagaacccaagttaaagctggttagagaacaagagacatgaggctgtctccag
agtaccgtctgggaggaaagaaggcaaagaaagactgtccaggcaggtggaagggaaagaaattg
ggcagggagagtggggcaggtggacacgatgggtcatggctggatttcaggcagagttaggggtg
tggga

>chr11_62323824_62324024
aaggatccagcaagccatggagagcccagcggggatgggaagggcaagagcccttgctgcagtga
ctcaggcctccctggggcagggcgggggacagaaagaactccagatgtgcttcccctttcctgcc
tcacatctgccgttagaaacttccactgttaacctctcccctacccaccacagcagagaagccct
tgcat

>chr10_103911610_103911810
tcttctgcagcagaggcaggagtctggaggctgggatggtatcagaaagggcctccctgggtatt
ggaggcagtgaagatgaagggcgagaatccattggccgtgccagtacgccatgggggcggttggg
agatcttgtagaacaactgttcgagcccgcttgtccactcattactctggaatccgagtcccagc
agggg

>chr17_7486676_7486876
acccggcacctaaggaggcctgcccggtgcagactctcctgctcccaccggcgcccttccctcta
gagacgctgagagaacgggagctagtagcgcccccacccaacgccacctcggagactccggctcc
ttctctctcaacttcgaacaatacaaagtgtgctaggagaagacaagatggcgcccagcaggagg
agcgg

>chr17_33390687_33390887
tcatctcaggaaccaagagcagcggctttcatgtgaagtgaatggtgccgcctggggatgtatct
ttggtaagcaagattgcaacacagcagggagtttacccagggcagtgctgactcacgctgggcaa
aatttgtacttcccaaacaactcagcctgaaccaaccgcagtcaactgcaacctaggaaagaaga
ggcag

>chr17_38270274_38270474
ctctttccacgacataggcattcccttcaaagaatgtaggtcatagaaatggaattctgccattc
tataagccttagaggggtggtgaaagtattggcttttaactctttccatactcttagccagaagg
caaaaagaatggtcattatctcattagggacagaattgcttagggaggcccgacactgtggctca
atccg

>chr17_42172674_42172874
tgcccgctcccatttctcaacaccgtgccccatgcaaagcctcatactagaagagaaactgaggc
acagaggttggttctaggactgacagtgcccttggtaaccccaggagaggctggtggagaccttc
tggatatctctaccctctgtccccttctggggcctcatccctatctggggcagactgggcaggag
ggaga

>chr17_57923218_57923418
aggtgccaactgtttgctcattctctttgacccactagccttgcagtttgctgtgtgggtgttga
tactcctaaattaaagcagggaaggggtggttttagatggcttttaacacctgctctccacccaa
gcaccctggattcatttgatatctggattctaattctgtagtcaggagactggcccaaactgtgg
cccag

>chr17_57924018_57924218
ggaggatctttctgtgaacttgctttgatgacctccacccaaagccctttgaggaacagttttgt
ttattcaaggagcccttggcaggtcactcctcccctaccacaaatctaatccaaatgcgacattt
tcctttgaggccatctatcccttttgggggacaaaacacatcagcttttcccttcagaccacttt
tgact

>chr14_23476760_23476960
gggccccgaggattcccaggcaaaatcaacagagtttagttttgctttgctttggaaggccaagc
caacgatatatatgtgcgaggaattctctcctttcttaggaagaaaaagcaagaacagtggatat
gaccctacgttcctggatgtagaaatcaaatagcctaggaaatcagggatacgggctctgcactg
ttcag

>chr19_18476200_18476400
ggagtacagtagtgtgatcatggatcactgcagcctctacctcctgggctcaagtgatcctccca
tctcagcctcctgagtagctgggactacaggcatgcaccaccacacctggctaatttttaaattt
tttatacagacagggtttcaccatgttgcccaggctggtctcgaactcctggactcaaactgatc
ctcct

>chr18_3603200_3603400
cttgtcaccctcaggcacctggctgcgaagtaataaaacggttgccttatcatctaaaggtggag
tgagtcaaacctcagcccagggtaattatagtaaactgaaattgcctgtcctttacaaaaaaaaa
ggcggagggagagagaaaggaaaagggcttaaggcaggaaattgtgtttgtctttctccttggat
ttaaa

>chr22_30592000_30592200
aaacaaacagagagactgccctttctgtaaactcaccgaatgtcaaatattgttcactcgcatgg
cattggcttactaagccactacagagccacttccctcacgtaatcccctcaatccaaaggcactt
caacttgaagaggatcaactgaaataatacacatttcctctgatgattggacaagaggcagccga
tggaa

>chr6_7147201_7147401
tcttaacatcacatgcattttgtagtttatggtctccagtctccagctgtttttggagcaccttc
taactttgagagggtgagctctagcctgtaaaatggactgtgggtggctcgtggagaaggtgccc
tggtgtgcttttctgtgtcctctctggattctccctgagctgtccacctctgaagcctgcttcac
cttca

>chr3_39192196_39192396
aggctcagatacgcactgcggctctgcctaagggatcgccacagaaagagaacttaaggtgagga
ggttatgacctccaagcctacagacagggaaacagagacacagggaagctacggtcatgctgcag
gttctagtgatgaacaatcccctccaaatctgcagagcgggaatgaaatgagaaattacctcctc
cctga

>chr3_133291510_133291710
tcaaggccccaagggtatttaaatgggctttaaacccatttaaacctggctttcgcctgggcatt
ttggtctccacccctatgataagacagtcccctccttttatcagggatagaagctaaggataaac
tgtatcatcatctagactcaagtcagggcgttaagcaatgtgctgagcagctaataatgttgact
ctctt

>chr2_70313096_70313296
actgtggggacccaaattatcccttgatactgccccccctattatccactattaagctttaaaaag
gcgagagattaaatattgtgcccacattttacagtcattcgcgttcccgtttcaaattaaaggga
ggaaaacgcgttaaaattaggcctccgaccttcagaccagcctgtgggctttttttaagtcataaa
actcg

>chr2_216980155_216980355
ggatgcttgtctaggcggcagatacctgcccagccagtatgctacgcatgcttgaatccagcagc
aggcacccagacaccgttagtccatgtacaactgggaattgttgtgttgtttacagactaaagga
ttaaacagaccctacaaaaatgaagaatgacagaattccattatctctccatacagcactcttcc
ccatc

>chr1_23881813_23882013
gacatgaatttgaattctggctttgttacttagaagccgtgtggccaccggcaagttacttcgcc
tgttctgagctagttcatttataaaataaagacacctcccaaatgtctgtatttcacagccgatg
cgatcagctctgaatccgatcgcatgcgacaatttgcacaagacactcagcagtgaatcttgtag
ttaag

>chr1_36852813_36853013
caaatcaagtctgatggattccaaaaaacttggctttgaatccctgctgcatcacttacaagctg
ggtgactatgtgcaaattgcccaatcttccccttctgtccaaacagggataacgcttgggggatc

actgtgaggactcggagttatagaaacagctttttgcaagacccagcgagcactcatactcatct
cagtt

>chr1_156075576_156075776
ttaggtaggatgaacaactgtccctattttagcattaaaagtcccttatctggccaggcacggtg
gctcacgcctgtaatcccagcactttgggaggccgaggcaggcggatcacgaggccaggagaccg
agaccatcctggctaacacggtgaaaccccgtctctacttaaaaaaaaacagaaaacaaaaaaca
aaaaa

>chr1_156093176_156093376
gactgcctaggtcctccctcacttcttctcctgaccttggggtgtggctcccactctctcccagt
gtcctcagggttaataactatgtgccaccagatagagagttaaggggctgctgaattggcttctt
gtgaagggaatcccctaaatgtccctcgttttggtcactggcctccctcccgccccccttcaggac
attct

>chr9_99182979_99183179
actgggcagaaggtgattccaggagcaaagctgaggtcataccagatctttgcttgtttggctcc
attttcatgcaatgaagaagctgtctgaagtgtatggatcatattcctgatgtctttgttctgtc
tcagatattaacagcccataatgtcagaggcctgtgaaccagagcaactccattttaaaaaggag
ctggg

>chr9_136008779_136008979
atgcacccctcctcccacccacgtggtccctggccctgcctctgccgccagcctgggggagagca
gggtctttgttccctgctggccaggccgccacgtgatgcgccacatcactgtcactgcctggaac
gaagactgccatggacagccgaggcgctggggcctgggggtgaggccagttggaggagctccacc
tgcag

>chr12_56520333_56520533
ataggtcaccctctccattcccccattctcaggaacctccttgcctactccagtgcttggcaaat
ttcagtttatagagaggtttctctctaaggttcatccatgttccttctgtagttctgctttatca
gttggtgagcaggtcactcataattcctccaatttccttgtttccccttttctacctgcttacctc
tggga

>chr11_47429024_47429224
gggaggctgatgagggtcagagacttaagagacctgtactcagtcgctatctctgcccaagtgag
tacccacatgccccttcctgaatcttctcctgtactggctgatttccagagaagaatacaaactc
gaaacgccaagccagactcccactgctgttcaggacctagacgtgagaagtggtatcacatactc
tggct

>chr11_62324024_62324224
cccacccccatctagaggccaacggcccagccttcctggggccacaaccctctcgtcttgaggct
cctcctcctgcttgtgggtactggccctagcccaagtgcaagcactgcttcaaacaacaacctgg
ccacccttactctgcaccgcgtggcgagggctggaggcaggatgtgcagatgactcactcttcct
gcagg

>chr16_21513899_21514099
ccctggggtgcgcgcgcacacaggcgcgcccttggattcacgtgtgccagttttcccactttcta
cggggcggggtggaaagtgaaaatagtcagatcgacaggtggatatccaccttcccaacctccac
ctgaaatgtgcccatcgagtcctagcaccttttaccatttcttcccaatgaaaaaaactaaacga
tggaa

>chr15_89181396_89181596
atctggggtaggaggcaagcgtgggtaacgctcagcttattgttggatctcaccacagtgaaaga
caagcacttccctcattgaaagttgatttcttttttgccccagttgtaactcatttgacattcac
taaataccaagccctccctatctggcaggcactgttctaagagctcaaagtcctagcctgggaga
gtgtg

>chr15_93460796_93460996
caaacaagcaaaaagaaaaaaaaggtgtgaccagtcagtcttctttgcttttccatcttaagaca
catagaagtgactgagaaggaatagttatgcaatgacatcggtttaataatcagttcaactgaag
aatgtatgtaaataagggcagtgatacctaacgctgatagttgatagtgtggattgcataattca
gactg

>chr19_11253600_11253800
ccgtctcttccttggggaaactgaggcccaggttgctggggtggaggggatgtggagtgacaggg
cagggcaatggctcctcggccccctccgggaacaaagccaggtcattcctgtggggagggagcgc
caaccccagggttgggggggcacgggccctgggtcaggggtacagataagcctgggctcccaga
accct

>chr19_11254000_11254200
aaggaatgaccgccaaggtgacagcagcgcttagccaaccctggcgctgacacctgacttgcaaa
ctgctccctgcctgaggcttcaaggcaggcgagaggctttgtgtgtgtgacttcctccccgaagg
gacactggcaacagctggagacatttcctgttgttaaggctgaggggggagctgctggcatttggt
gggtg

>chr19_39173560_39173760
agtcacattactccgtgttatggtacccatccctgtctccttagcttgtttttgtctgtattggc
tcttccactagactgtaagttgcatgagggcagggatgtctgtttaatcccagtgctcaggatag
tgtatggctcgtgatagatgcctagtacattttaaaatgagaacgaatgaagtttgggagaggct
cagag

>chr19_47611560_47611760
tagaatctggtttgacttgatagctatccacggagacctggaaatagtttcattagcaattttca
ccagaggtatttactgtaactggatctgacctctatggagagatgtttctacaggcccaatagcc
ataaaaatgtaacaaaaggaagtgaaggaccctcagacagccatttctctcccatcagggaagaa
ggtaa

>chr21_45148172_45148372
ttgatcagggtgaagcagtcacatgatctctcctgtcctctttcctcattctggtttcctaaagt
ggtcagaggtctctgcaaaataagctaaagggagggttctgctgggtgggtggcttcggcttctg
tgcagcgctcaccctgctctcccctggaacgcctggaactagggtggtcaggctggggtggagga
gctcc

>chr3_141086910_141087110
ttccgcatctctttcaataccactgcaactccttgagtaaggtgggcaccacacagaagaagggg
gatggcagaggggcagtgagaggtccccagggaaacaccctcttcttcttccctgcttctctgac
agagcacggccatgcctctgccatgcaggagggtgatgtcctttggggaggccttgttgaatctg
tctgg

>chr2_43448296_43448496
ttggcctcgggcttcctctcccctgctgctgaggccttcagtcaggggctggcatctgggagaac
cttgaaactgtttcccatcccctttggcagcaagcaacaagcaccaaaacgctccagttaacacc
agtgcaatcatcgttaacgtgaaacagctgcccgaggcctggcgtatccaccagaccgcccctc
ccctc

>chr1_28835213_28835413
ctacagcttcccagagtcctgtggacaatgactggggagacaaaccatgcaggaaacatatctag
tatactagattttaagttgaagtaggatcttcaggagtctaatcattatttcttttcttttagga
gagaagacgatctgcacttcgcattttggcattgacatttaattttagggtcctttatatagaag
ggaga

>chr1_38465813_38466013
ctcatccccccacccccgctgtgtcacaccagcttggacagccttggcctgtaacatctgcagca
gctgtggcggcacagatggccagatgtcaggttttgccagcaatggggagaaaaaaggaaaatga
agtgtcgggggaggcagaggggaactggagacagactgggagtgcagtgtacccccctgccacctc
ctgct

>chr1_110546677_110546877
ctggtattgcctcacctcttctcgccgcgagcattgacagaagatctagttgagtcctactttga
aaggagtattgcttctgactgtggaattggattctagctgtgtgtgtgttatatcctgttactgt
tgagacacagaggcgggagtcggcgggggaagatatgtgctgagattagtcagcctgccctccct
ctccc

>chr12_76338533_76338733
tctcagagaaggctatggtcagaaaatggaaaaggaaacaagccacaggctcatcttcagatgag
gactctgcacagggcttgacctttcaatcgaagaacaaccacagtagtattctgagatggccaag

tgtatgggaaagtgactgcttctctcccactccaatgtctactgtaagtcttgttcagccccgag
acaga

>chr11_62389824_62390024
atcttcaccttattcatccatccagtcagtcacgccacccaatagctgttgctcaatacttatta
cacgaatgaaatctaaggcccaccacatgatcctctgggtgtctctcctggaatcctcactataa
tactttgagatagataagaaaccaaagttgacaaaaattatgtcattgccacagggcagttttgt
gtcct

>chr11_65255824_65256024
gcccctgaggaggtggacatttaggctgaatggaggacagggaggtagcaggacctgctgtctcc
aggacccctaagtttctaggccagagctgacggctggtgggtgcagtcagccaggctccagggaa
ggctgctccaggcagggctgagtcttcacggttcagcttttagaactggccaggttggaattctg
gctgg

>chr17_38267674_38267874
gagtggctcttgggtccaaagggcttccccttggccaagtcccaccccaaagcccaggaagtcca
cagcttggcgcccagtcaaggcaaaaacattcctcaggactctgacaggctggggatggttccc
accacgaggatgggggaggggaaggaaacacgcgggggaggaaatgaccacaaagaagggggtga
gggta

>chr16_87812699_87812899
cccctcaccccacccgccacacctgccaacttcccggcctgcagggtggcaggaagcctgaggca
ttattcagcttccttagggcccgttggctgatgcaacacagctcagaagttgagtccaagcacca
agcccgcaatgtctgcgtcggccgccaggggtcactcctcccgcgcagaaggcggccttggagcc
tcgca

>chr19_3983800_3984000
cttcccagcgtcccaagcactagaacaccctcccctcccaggtgtgacagccaaacctctccaga
tattgtaggagtgggggcaagtcccccagggagggagaaccacggagttaagccccctcctcaag
aaaaccttccagctcgcagtaccccgaatccctgtgcctctccatcctgtctcgctggactgaac
ctcac

>chr19_18485400_18485600
gtcccagccctgctgactcatactcagctgccacggtccgctttccctgctgagcacgggggtgg
gggcgggggggaagagggggagggaagggcacctgaccgaggccagaggcccagaaaggacagaa
ggtcatggctggggagaagcaggcaggagaaccgagtcttggccttaactggctggcaacgtaaa
acctg

>chr19_39177360_39177560
gagtgcaggctcttccccttggggagaacccagttctttgacgtataatctgagtggtttgggtt
ggttggttggttggttggttggtttcccatgtgtgggatggctccggaagtctgtttgagaacag
aggcaggctcagatgggagcagctcctaccccgggccgcctatcccccttaccctggggtttct
tttag

>chr22_36767054_36767254
tcaaagcaattaaacaaccggtagagcaccgtcctggagggctcctcccccagatctgaaggcat
ccacagaatgtccctggatcagagaaccctctgctgacacaaacacactcccctactcccttctt
ccctgttacagattgaattgtgtccctcccaaaaatatccattatgttggagccctaactcccag
taccg

>chr20_30307539_30307739
ttctctggtaggggtagccttctgcagaggctggctagcagaccaggcacctctcccagactgcc
ttttgtttatagctggcaacaacccacctggtattaggaccactggccaaagacaatagtcaggc
aaagtgggcaagcagcctcaatccctacttggagatgcctcagagggtgtgtctgcagtactgga
atcct

>chr20_45988393_45988593
agaaacctggaactaagcccagagaggtgaagtgagctgcccaaggtcacacagctgggaagtgc
cagagatcagatgtccaccccgttagcacttcacatccctaaactctgtaccttctcctggctct
aaagaaagaagcaaaagcaaagccgaaccaagcacagcagaaacaatgaggctgaaacctacgaa
cagca

>chr3_133291710_133291910
ccctccatccccctatcttgtaagaaatagtatacccactttacagattagggacttgacgatag
gatttattgaacttgaccttggtaaacataatccagagggtggagttcagacaaaatccctttct
acctaccaaccagcctatgaaagatcttttagtaaatgggacctaaacttcaggctcactgctcc
tctca

>chr2_43448096_43448296
aatgaatgtggcttacccccaacattccagctgatcaaaagtacttgtttagaaaaataagttta
ggaatgaagtggaaacaaggcctttgaaatgcagcttaacctaaatagcacattttagactttc
ctccccactgcacattctttatggtgtaggggccagggagcctgcagtcttcacccctcctgagc
cctcc

>chr1_38465613_38465813
cctcctgatccagaaaacagtggaatgctagagaggtgctggactaatgaatggtagaagaggcc
agtgtgagaggttgggccttccctcccggggaatcagccaagcaatgggaacttcagaggctcag
cttaggttcaagcaggaaggaaatcctgcaagctggacacagccagtcttccctagctagaccct
ctgcc

>chr1_38466813_38467013
ccagggcatgaggccaccaggggccagggtctttagagctggaactcctgggctgggggctatgg
tgtagagaagatactcctctttctcagccccttcctgccactcaggcccaggtttcctctggcc
caacatctgaggattgcctgggctgtcagccacggggcagcagggggccacacaggtggaatgcg
tccac

>chr1_90372212_90372412
tggaaaaacctgcctgtgtgttgtgccaagttgtgttagccactccatgttattcttagatgggc
tctggagtatgaccagcctgtgtgcatgcatttgtagtaggcactggataagaactggagtaatg
actacagaagtgaacggtcacagctctaacctgctgagtcatctcagctcagccttgctagcaag
gcctc

>chr1_145455643_145455843
acaaagggaagaaccaaaagagaaactgcaactcagccacacactggtttctgtaactggagaac
aggatatttaggggacttgctctgcacagacatacctttcaaagtggcatctccagagtctgga
agaggagggagtatggtgaataagtcagctcctcctccctcctcatacctggttttcttgccttt
cccca

>chr1_156093576_156093776
tgccctggcctccggcctcaggcttctcctgcctctgtacaatgccacgttgatacgcccagcag
ctgtgactcaggcctggccccctgccaggcccagcacttctactggagttgcgtctgaacatgtc
aacaggcttcctatccctctctcagcaccagttctccccacttcagcccctccctctgcctggaa
ttaaa

II. 100 DNA Weak Enhancer Sites

>hg19_ct_usertrack_3545_11005 range=chr11:132363991-132364190 5'pad=03'pad=0
strand=+ repeatmasking=none_0
ttatggtcaccttcgaccccagaaataatggtctctgttgtcagattccattcaaatccaagaag
ttgttgtcaataatacttttgtaaatgcaagaaggacaatgtcagttgaaacgtgttgggtttgt
cttcccatactgctgtgaatcatgcatatgcctcatctgctgctacttataataaaactgcgtaa
caatt

>hg19_ct_usertrack_3545_8529 range=chr11:112506991-112507990 5'pad=03'pad=0
strand=+ repeatmasking=none_0
catccaggcttggtcctggttgttccttgctgttataccagcctggcaaggactcctgagctgct
gctaagagtggttccttgcctgactgttcacgccatggcttcttgttgcagaaccctgtgctctt
aatgctgcagagctgcaaatctgtcactgccacctctgagtagatcactattaaggaccttggt
cagca

>hg19_ct_usertrack_3545_7245 range=chr10:99167611-99168610 5'pad=03'pad=0
strand=+ repeatmasking=none_2
ttgtttttttctgttttgagacggagtttcgctcttgttgcccaggctggagtacgatggtgcaa
tctcggctcactgcaacctccacctcccgggttcaagcgattctcctgcctcaggcttcctgagt
agttgggattacaggcatgtgccaccaagcccggccaattttgtatttttagtagagacagagtt

tctcc

>hg19_ct_usertrack_3545_12669 range=chr3:179421107-179421306 5'pad=03'pad=0
strand=+ repeatmasking=none_0
actgttaaatagcaaaaattattgagctcaaaccatctaaccaggtgattcttccttcatcttct
gagtgttttagcacagaagaatttcattttcagagagtgactctataggttctaccagaaagtta
aagcacctctgggtgatgctgggttcacagagacatagcactgtgagctcggcaggggtcttaga
gcttg

>hg19_ct_usertrack_3545_5404 range=chr11:68037625-68038224 5'pad=03'pad=0
strand=+ repeatmasking=none_0
gagaattaagtttgtattaagttggagaccagggcagatggaaagacggaaggttaggctggcac
tgggagctcccttcttcaggggtcagctccccataggatctgcaaggaaggtgcaacaggatact
cccttgagccatgtgacactccatgacaaaggcacccccaactatgcccctgggtgtcaacctcc
ctatc

>hg19_ct_usertrack_3545_6136 range=chr12:81601870-81604269 5'pad=03'pad=0
strand=+ repeatmasking=none_4
cctcaacactccctttccccagaagaaataccttttagtgaatagaatatattcgataaatact
tattgaatgactgtcctttgaatatcattcttggaataagaaaatgttgtaaaggaatcataatg
gtaacatatagcttttatcttcctggagtcctctatttttttctctctggttcattttgggatta
cattc

>hg19_ct_usertrack_3545_9048 range=chr7:122581965-122582564 5'pad=03'pad=0
strand=+ repeatmasking=none_0
ttttatcatacgtgcttgtttttaagccattatcacttagtttcaaacacactcttgtatttttc
cttgtcttagtaggtctggggctccgcaaactacatttccactttttcagctgccttcctgttag
gcttccacatagtgaattctgaaggaagactggagtcctggaggaagaaaaaaatgacatattct
cctgt

>hg19_ct_usertrack_3545_1030 range=chr6:10594615-10595814 5'pad=03'pad=0
strand=+ repeatmasking=none_0
gacttcatgaaagtggtatgcaaataactgatatttgggatgtactcatattaaggatatattat
gggctctggagtgagacaaatgcaatattgaattttagctttatctccgttagtttgatgacctt
gtgtactctgacctaaaactcttcattttgtagtcacaataatgatggctacttagtaaggctgt
tttga

>hg19_ct_usertrack_3545_2146 range=chr21:47373973-47374172 5'pad=03'pad=0
strand=+ repeatmasking=none_0
aggattaaacaagttttactggggtctgaagaaactccccagacctccacaaacaagcttccttg
gaaactaaaggagctccctcaacctccgtgatttagcagagggcaagataagggtgatcacccca
gcacctggacccagttagattcagtaaatccactgaggctccagaggaaggtcttcaggactcag
gcctt

>hg19_ct_usertrack_3545_6017 range=chr5:109173902-109174701 5'pad=03'pad=0
strand=+ repeatmasking=none_1
ttcatgcccaggtggagccactttagtaaaaaaaatagcatttttgctgccatgtgatgcacagg
taaaacagaccactggatactgctggtttgcattcctgcttcctgggaagttctcccttccattt
catgagcctctttgggaaaggtacaaacttagggaattactgacacagaataggaacaattggca
atcct

>hg19_ct_usertrack_3545_7241 range=chr9:124708580-124710379 5'pad=03'pad=0
strand=+ repeatmasking=none_3
ccccattcattagatcacctgcttggccctgacagcacttgggacccctgacctctgccgccttc
tctggtcctcactatccctcatggtgtggtcatgtttagctcaatttatacatagaaactcagat
taaagatcctgttccaggtagcccagctctcaagcagaaaaatggggactaaaaccaggtatgtc
caggt

>hg19_ct_usertrack_3545_3021 range=chr13:55147000-55147399 5'pad=03'pad=0
strand=+ repeatmasking=none_1
acaaacagaaaggacatccacaccaaaaacccatctgtacatcaccatcatcaaggataagtggt
ctacccaataacaagcaataatatgccagatacagtggctcatgcctgtaatcccagcactttgg
gaggccaagaggggcagatcacttgaggtcaggagttcgagaccaaactgaccaacatggtgaaa

cccca

>hg19_ct_usertrack_3545_2621 range=chr10:33269995-33270194 5'pad=03'pad=0
strand=+ repeatmasking=none_0
gagtagataaaatgtgttatgccagtgccctgggaatccgatacagctggcccaggaggggcctg
gggttctgctttgttttgtcttcagatagtgtctagctctcttgtcaggctggagtgcagtggcg
agcctagctcactgcagcctctacctccccggctctagtgatcttcctgcctcagcctcccgatt
agctg

>hg19_ct_usertrack_3545_6150 range=chr6:66276880-66277679 5'pad=03'pad=0
strand=+ repeatmasking=none_3
ggattatttaggtttctaggaaatgtatcattattttatttttcatatttgctattaatgaaagc
cttacacttggtgaagttatatgttaacctctagttttcagaccaattaaaataaattttggtag
taaaaaagataaaccctagcggatttatagttatgcagggttttaaccagtttcttttctaagct
agtta

>hg19_ct_usertrack_3545_1282 range=chr10:14922595-14922994 5'pad=03'pad=0
strand=+ repeatmasking=none_1
taaaagtttggctttaaaaacagtacagtccataagtattacaaaatatcgatatactccatttt
tttaataactggagtttattcaccttggtgataatatattgcagtatgggtagaataataagtgg
aattaaagcttcatgcaacatatcaaagagaaaactaacatataatgagtttgtaaccgtagaaa
tatat

>hg19_ct_usertrack_3545_2483 range=chr7:28520676-28521475 5'pad=03'pad=0
strand=+ repeatmasking=none_1
acgcaatcccaaaggggggctgtgattcaggttgtaggattctgagcttttgggataccaggggat
ggcaaatatctttgaagaacagggatattcttctggtttgaacagaatagtaatatttaaccgca
gtgttatgtgtcaagttcaggccttctgggggagactactggctctaaactgcccaggaacacac
cctct

>hg19_ct_usertrack_3545_5595 range=chr9:103116780-103118179 5'pad=03'pad=0
strand=+ repeatmasking=none_4
ttaatgggtgcagcacaccagcatggcacatgtatacatatgtaactaacctgcacattgtgcac
atgtaccctaaaacttaaagtataataataataaaataaaaaagaacatatcgccttctaatata
ccatgtaatttacttatttatcataatgattatttgattctgtccttcatccaccactaatatgt
aagtt

>hg19_ct_usertrack_3545_2904 range=chr22:39864855-39865854 5'pad=03'pad=0
strand=+ repeatmasking=none_0
cagctcagccgaatcctccaggaacaggcacggagattgaagggtgagattacacagaaacttct
gtgcctccttgagggacttggactttctcctgagggcagcggttccaagctgggaggggcagcat
cagatgtggctttcgcttggctcatgggggccgggttgggggcccattgaggaagctgctgccat
tccgg

>hg19_ct_usertrack_3545_9202 range=chr10:123874411-1238748105'pad=03'pad=0
strand=+ repeatmasking=none_0
gaaacgtgcttgaacatttaaaatattttagcaagaaggaaagttaaagatcaaggtttcgtttt
acagatacgaaaagagagactcaaagaagtccagggtcttggccagggccacacatcaaggtagg
actccaaccccagaccgcagacacttgccctggtgttctctgcgccaagtcctgcggcctcgttg
gtttt

>hg19_ct_usertrack_3545_9648 range=chr11:120971191-1209715905'pad=03'pad=0
strand=+ repeatmasking=none_0
agttttgctcattccacatctactggatgcttggaagcatctaaccgagcacgttttcctcttat
tcacatgccttttaaactatttacttactgcaaatttttttatcagaaggggtttatctcccccct
ctgcctccagtgaccctcactgggtgttagggaaggtttccaggccacagtttgagatttccctt
gtgga

>hg19_ct_usertrack_3545_5052 range=chr19:51910989-51911588 5'pad=03'pad=0
strand=+ repeatmasking=none_0
ccttgcagcatccttcccactctaagcttaaaggagactgtgtaaccagcagtgctcacccatcc
tgggcctgagtttgaatcccagctctggagttcagctgtgtgacatgcaagaccttgtccctctc
tgaagtccttctgtaaaatgaggtggctgcattgggtgatcccattccacaccatgagtctatag

tccta

>hg19_ct_usertrack_3545_695 range=chr22:24190001-24191000 5'pad=03'pad=0
strand=+ repeatmasking=none_2
cacccccttccaagaggccctgcttagaggcccccctccgccccaaggaatgcaggcatcagtgct
tcagactctggcctaaccaccgtccccacctcccaggcccccaccctttccctccagatgtgggc
acagctgtatttttagaacagccgaccacgaggacattctttccttctaattaagatggcttcaa
atctc

>hg19_ct_usertrack_3545_10029 range=chr7:133635861-1336360605'pad=0 3'pad=0
strand=+ repeatmasking=none_0
cagctcccattcctgcaaaacaaatgaggaggggtgcttttcttgaatgctgtgcctggactttc
tctcccttgacccagccctacccatggaataccaaggcaacctcaacatctgttacactcctgcc
ttaattctattcctcagcaccagcataactcaatagaggagtatgggcttgtccttggacaaggt
acttg

>hg19_ct_usertrack_3545_8904 range=chr8:132997819-132998218 5'pad=03'pad=0
strand=+ repeatmasking=none_1
cagttcattggaggagccagtccacacaagtgcctggagaccttgtacacaggctgcaaaaggta
atgcagcaggtggaggatggtgatttgtaaagcgatttccaaaatagtgcatttgagaaaataga
tattcttgctttattataataggcttaatcaaatgtaatgtgtcatcatttttttgctttatatat
tattt

>hg19_ct_usertrack_3545_10628 range=chr5:168884623-1688852225'pad=0 3'pad=0
strand=+ repeatmasking=none_1
gggaactaagatatattggtaacagaagatggttgtgaactctactgaagtggggtagagggaaa
taagagttctaatttaatcacattggaaacctttcaggtcaagaaaaaaaattatgcttcctata
gcaagctatcaaaacttacatactttaaaatataagaccatttatcttttctcatgggactcaga
gttcc

>hg19_ct_usertrack_3545_5887 range=chr14:100030048-1000312475'pad=0 3'pad=0
strand=+ repeatmasking=none_2
ctccatagaggctggacctcactcccattagccatggaagagagcactgtctctccacagaagtt
tcttgttttgttgtttgttctgcgcagtgaaggggacagacaggtggagacctcttcgcacccac
tgctgcgtccttgctgctctctggtcctgataaccaagaagcaccagggtcttgcactctcctcc
ttgca

>hg19_ct_usertrack_3545_8816 range=chr7:121096565-121097564 5'pad=03'pad=0
strand=+ repeatmasking=none_3
gagctagtgcattcccttgtctgcccctagctggtgtgtctctaggttgcattcacctcaagttg
actggctcctatcccagcacagcactaggacttgcccagaaattgtggttcttgtggcctagact
gccttccaaatttatttaggaccccagagtactttagcctgcagtgatggggctagccagaactc
aggtt

>hg19_ct_usertrack_3545_6900 range=chr6:83779082-83779881 5'pad=03'pad=0
strand=+ repeatmasking=none_2
gtggagtgtagttattgattttattcaaaatggcattttgaggttccaacaagtgaagacttgtt
gataatggcatcacttttgctgtgatgatttctccttatagtcatgcattcctaaaatgaaatta
tggtagaagccacccaaaatgtgcttactgtgtaataactacctattttaagagcttatatcatg
gaaaa

>hg19_ct_usertrack_3545_232 range=chr11:1330825-1331024 5'pad=03'pad=0 strand=+
repeatmasking=none_0
cctcgaggccgccgccgccacagtcagctgacagccgccgcgccccgcccccggcaccgccccac
tgcgcaggcgccgcgccaggcaccgccccttccggctctcgcgcgcacgcgcgctccagggtagg
gcaggcgcagcctctcgcccggcgcctgcgcacctgctctctcctcgagccctgcagcccggcga
aatgg

>hg19_ct_usertrack_3545_6082 range=chr10:84519421-84520020 5'pad=03'pad=0
strand=+ repeatmasking=none_1
agaaacagcggaaggaaggatggtaggtaacagtcggcacaagacacacaaagaggaaactctat
gggttctgcatgcgtgagttgctgaaaatgagctttaaggttaaagccaggagtttggaattaat
gtgtagcatgtgaaatataaaaaactccattcaatgccctcaattttatcagaggattagggtaa

cctcc

>hg19_ct_usertrack_3545_2631 range=chr12:29224734-29225333 5'pad=03'pad=0 strand=+ repeatmasking=none_1
gttaactaaccaccattaatcacccaaagcatagatcaaatgcaaacccattatagaaaaaagag
aatgaggaacagtgtgtgtccttttggtcaggagtgtgctaacacaggaatagataggatcctaa
aatgcaacaatcaatggcaagtggtctttttaatttagtgggcaaccagaggtgacatgatttga
tgcct

>hg19_ct_usertrack_3545_2008 range=chr8:23313456-23314255 5'pad=03'pad=0 strand=+ repeatmasking=none_3
catttcctattagaggtagaagaatataagccatagattcaacatgcaacaactacaagacctct
gttaaggtaaacagagaaataaacgattcaactacacgaattagtgttccattttataattctac
tatgtcaaacagaattaacaaacctgatacaaaaacatcaaatatatggtaacacaaataatata
actct

>hg19_ct_usertrack_3545_3152 range=chr19:35631561-35632360 5'pad=03'pad=0 strand=+ repeatmasking=none_0
tgggggagagcctggctttgctggtcctttgattccccctcgccctcccccagagtcccagtatt
gatatctctgtcattctccttccctctatttgtccttcctctctgattccacctgtctgcatct
tttcctgtctgtgtctatctgtgtcactgtctatgtgatacctctctggttctctttctcttgcc
tgcgt

>hg19_ct_usertrack_3545_1589 range=chr22:30000801-30001000 5'pad=03'pad=0 strand=+ repeatmasking=none_0
caggaccacatagtaaatattttcaactttgtagactagagaatctctgttggagttattcaact
cttcaactctaccattgtaacacaaaagcagccttagataagatcagcttccagcatgacagctt
aaggaacccattcctctgtaaaactggtgaaaattctttttgttttgtttcgttttgagacaga
atctc

>hg19_ct_usertrack_3545_3843 range=chr19:42381761-42382760 5'pad=03'pad=0 strand=+ repeatmasking=none_0
cagggctgggggcaggaaggggacttaggggtagcagcattcagcgtctgtcaaggggagaaaaa
gctttctctgccttaaacctcaggtgcctctctctgttgggagtcccttctcagcactgggggaa
tgggtgtctcatggactccccctcacctgctcaaggacagctggcaggggctgtggccacgctaa
cccag

>hg19_ct_usertrack_3545_12977 range=chr2:189327556-1893277555'pad=0 3'pad=0 strand=+ repeatmasking=none_0
aacccatcattttaagctgataacaacttaacatcattttcataaacaaacaaacaaggaattaa
aacttataaagacttgatgccataacttcatacccgttttcttttaactttttttgttactactta
tatcttattgttccatttttcttgaaaagttgttttagttattatttttgattggatcattgttt
agttt

>hg19_ct_usertrack_3545_12625 range=chr2:183364756-1833661555'pad=0 3'pad=0 strand=+ repeatmasking=none_5
atcatcagaaaaaggaacaattaattgcttgctgagcatgtgcaaatttacaaaacaatttggct
tttcagatggaatttgaaggcctgaattatttaagtcaactgaaactactactactgtgaataga
ggcacaacgacccttatatagtgacataatcatcacatccttctggttaataacatatttcacag
cagtc

>hg19_ct_usertrack_3545_1738 range=chr10:21583995-21586194 5'pad=03'pad=0 strand=+ repeatmasking=none_9
gcctggagtgcagtggggagatctccgctcactgcaacctccgcctcccagcttcaagtgattct
cctgcctcagcctccagagtagctgaaattacaggcgcctgccaccacacctggctaattcttgt
atttttagtagagacagggtttcaccatgttggccaggctggtcttgaactcctggcctccagta
agtaa

>hg19_ct_usertrack_3545_7734 range=chr9:128453580-128454179 5'pad=03'pad=0 strand=+ repeatmasking=none_1
tgagagcccaggagaaaggtctgagcaaacttgggagacatcaacatgtaagtgacaggtaaatc
catagatgtgaattttgcccagggagaatatgtaagaagaacagtaacctagggataaaaccaag
gaacagcaatagttaaagggtagcctgaaggaaaaacaaaaaacagaagctggaaaaaaaaaaag

tcaat

>hg19_ct_usertrack_3545_1143 range=chr12:8716934-8717733 5'pad=03'pad=0
strand=+ repeatmasking=none_2
ggaaggggctccccctgccccgcaggtgccgccccgaggaggtggaggaggaggagccgggcact
gactggggacccaggtgagtctagcaaatgacaaggcccaacggtcaaaaccgcctcagtaatgc
gctcgtccaaagcacgcacttaacgctcaatcctggtgtcgcgtcccgttgccaggcaacgaaag
caagc

>hg19_ct_usertrack_3545_764 range=chr7:6297476-62982755'pad=03'pad=0 strand=+
repeatmasking=none_0
tgtggaggtagggaggttcctgggtgggtgaaattcagcaacagcaccatctagaatcccgctgg
tcatcctcagggtgtgggcttggaaatggacaggcttaattaagagccattgtgaatgcaagctg
gctactcatccaggtccaacaaagtctagcataagaaatgcaacagggctgggcacggtggctca
ctcct

>hg19_ct_usertrack_3545_6687 range=chr9:119065780-119066779 5'pad=03'pad=0
strand=+ repeatmasking=none_2
ttatttcttcattcacttttttgtttatgtttacatcactaaagcctttattcatttgcatttatt
tttgaatgccctcaggagagtggaaacaaatggtaacatggcatttcctgatgcttaacaatgta
tagtgccagcctgctgaatgaatgaatgaatgaatgagtgcacagatagatgaatggctagggtt
catcc

>hg19_ct_usertrack_3545_7969 range=chr12:110072818-1100734175'pad=0 3'pad=0
strand=+ repeatmasking=none_0
ccaccaccaccatcaccaccaccaccaccatcaccatcaccaccaccaccaccaccaccatcacc
accaccatcatcatcatcatcatcaccaccaccatcaccaccatcaccaccaccaccaccaccat
cgccatcatgatcaccaccacaatcatcaccaccactgtcaccaccaccaccgtcaccaccatta
ccacc

>hg19_ct_usertrack_3545_1396 range=chr4:16078903-16079902 5'pad=03'pad=0
strand=+ repeatmasking=none_3
aaacatccccctctccacctcaccccgaatccaaagtagaagggcatggcctcaagtctagggac
cacaccgtttgaggctggacacatgtgcaacactggaacctgtctttctaataagcttaccaagt
aatcctgatggaaatcttcaccctaagtcaccacctctacaactaggggtctcagagatgaaagc
caggg

>hg19_ct_usertrack_3545_3715 range=chr16:49782100-49783499 5'pad=03'pad=0
strand=+ repeatmasking=none_2
tagctggttgctaagaaatcacaccgggtgacatcacctgatcacttactacaggcaagacatgg
ccagggtcagagttcaaaacagtttctggaaatctttccagcgaagccccttttccgtgcaggga
tgggaggatgggaaagtatcttgatgggtacccacctgtgacccccctgccctatgacagaggtac
tatta

>hg19_ct_usertrack_3545_11269 range=chr5:175065595-1750679945'pad=0 3'pad=0
strand=+ repeatmasking=none_5
tcctctgggggtccccaacaaggggcagggagcagtgcaggggagtgcctcagacctcagacccc
aggctacgcgacctggatccaggtcccagctctgcctctcggtagcagggcaacttgggcaagtt
gttaacctctctgggcctcagtttccctgtgagcaaagtaaagatgctgctagtaatcacaccta
tctca

>hg19_ct_usertrack_3545_968 range=chr4:8281901-82821005'pad=03'pad=0 strand=+
repeatmasking=none_0
ggagaggtaatatggtcccacctgggggctgtgcccgtgccctggcgcttctcctcaggcgaggc
acatggggcgccagcctcaggccccagggcacacaggggagcagcagaccggctgagtcctccct
cctggagggaggagggcctgggcctgggggttgccctgggatccctctgggtcgtggaaattgtg
ggtct

>hg19_ct_usertrack_3545_4850 range=chr7:64735166-64736365 5'pad=03'pad=0
strand=+ repeatmasking=none_2
tgttcatatatggttgtttattaaactatttatcatgaaagtatgtaagtggcatgcttaatgtc
tgaaagagatagagacttcagcttttcctgttgaggtataaaatttaagtgccttactatttcct
tacaatattctataaacactgtgtttgagtgattttgctgttttctttaaacactgggcattagt

tttaa

>hg19_ct_usertrack_3545_1633 range=chr5:29524844-29525043 5'pad=03'pad=0
strand=+ repeatmasking=none_0
aggttggtcattcattttatttcagcaaatgagtagtaagtaggtcgaggtagtaaaaagaaata
cctcattttagtgaaagagtgtcttaagcaattgcaatataaaactcacattacaaagacaatgg
aagtttgacctaccagtttttggagtgtcagaactgtgcaaacagagcatacaagacagagttta
cacta

>hg19_ct_usertrack_3545_2583 range=chr22:38367855-38368654 5'pad=03'pad=0
strand=+ repeatmasking=none_2
tgaggaagcaccagtacagggataagagatgaagagacaggccaggtcaggctcaccaagcaggt
aaccggaacctttaattttattatgtggaatgcttaatgcagagttaataggggctagagtggct
aggagaggggactactgagataaataacaggagacagtaatgagttacatgtggatttggggggc
tgcag

>hg19_ct_usertrack_3545_14460 range=chr2:210443956-2104445555'pad=0 3'pad=0
strand=+ repeatmasking=none_1
ctaaatcgtaagtgagggctgcatcctattgtcagtgaatgatggcggagctgagtggcttgtca
tcattctcgaaaattctgagactagtgagctctctccctctctcccactcgccttattttcctgt
tcgccactgcggggacttctaatttgcagcttccctttccccggcacacacagacacgagctggt
ggctt

>hg19_ct_usertrack_3545_4451 range=chr17:42174275-42175474 5'pad=03'pad=0
strand=+ repeatmasking=none_2
ggcatggttgggaacattcctctgtggttttcccgccagccatactgacgactctgtggccacaa
ggccagagccacatgtgcagaagtggccagatgtggccagagggtggggttacacatactcatct
tcattcccaacccatgtcctgccccagcaccaggagaaagggtccttggaaactgcagaaggcct
tcctg

>hg19_ct_usertrack_3545_11356 range=chr3:155243307-1552449065'pad=0 3'pad=0
strand=+ repeatmasking=none_3
aaaccaggctctgaggctaagaggccaaggcgactgcagtaggcagcctctaaataatagtacca
atgtctcctgtctcagtattcacacccttctgcaatcccctccctttgagcgtggcctggaccta
gtgcccgacatctaatgaactgagtacagcaaaagtgatgggaggttacttatcagatcaggtta
tagaa

>hg19_ct_usertrack_3545_7141 range=chr3:98442511-98442710 5'pad=03'pad=0
strand=+ repeatmasking=none_0
tccatctcaagaaggtaacagagctttggtgttctgttggctcagtgatttttggttacagaat
aaaaactaaaagtcaccagatatccagaagacaactcaacttgtgtttcttttgtttagaaatct
tagactgattgagtcagagaagcaggagtccactgttgtgcttcatgttaggggaatgagcgtgg
cgagg

>hg19_ct_usertrack_3545_1613 range=chr16:13262700-13263099 5'pad=03'pad=0
strand=+ repeatmasking=none_1
ttgcttttggatttccaactctctgtatacaatgacccctacggaagacctcacacctgtttcag
agaagagaataatacccgcccctgggtttggccatttcctttgtcttacagttgtcatgggatct
tcacccctcccagctccattggaaggtcagttgcagtgcagcccttttccggggtaataagtttt
taatg

>hg19_ct_usertrack_3545_6105 range=chr14:101123848-1011252475'pad=0 3'pad=0
strand=+ repeatmasking=none_5
agcagaggctgcagccaggagggcaaaggggaggtggctaggccatggaggggaggaggagcatg
gggagagcagcattgggggagctccagaaggcccctggtgtcagaacagagggcagacctgctcc
tcctcctccctccctctctgcagctgcagccggaagggccttgtccctgggagggctgccttgac
tcctc

>hg19_ct_usertrack_3545_379 range=chr22:20244001-20244600 5'pad=03'pad=0
strand=+ repeatmasking=none_1
gcctgcaggaagccatgccccgtcccgctcactcctccactggctgcttctccgaccccgatcat
ggctgcagcaggcctggaggcccggtcacccccaggcccagtcacatggccctactcaggcacca
cctgtgcttcttggctgggtttctcatctgagaaatgggaaggctgggtctgggtgaggcacttc

tgtgg

>hg19_ct_usertrack_3545_258 range=chr15:26373508-26374907 5'pad=03'pad=0
strand=+ repeatmasking=none_0
aacttcaaatcaatgaagagaacaggaatgaaaaaataatctacttgatctctgggaaggtgtgt
ggttgattagaaatgatgacaatttttttgaccattgggacatggcagtcaaattctgggatgaac
ttagaaacttgtttgacccataaattgccgtggaagtgaagctatgtgacttacgaggctaggtt
tctaa

>hg19_ct_usertrack_3545_7846 range=chr7:104999165-104999564 5'pad=03'pad=0
strand=+ repeatmasking=none_1
ctaagaacacagagagagagagagagagagagaaagcaagagtggaggtggaggtgcctcttttcaa
caaccagttctctggaaactaagagtgagaagcaagaatgacacctcaagccattcaagagggat
ccaccccctacaatccaaacaccaggccccaccaccaacactggggtatcaaatttcaacatgag
gcttg

>hg19_ct_usertrack_3545_7989 range=chr1:68831813-68832012 5'pad=03'pad=0
strand=+ repeatmasking=none_0
tcaagatctcatgagttgagacacagccgctccctaggggagcacaggacctggtttctgtgccc
attccctgcccctctttaggctgacttctctttattgaagtcataatggggttaaaatgaccaat
ctgttcaaccttgcaagagacaaggaaaggaggaagggaaagagagcctttcacatttgtgtgtg
tgtgt

>hg19_ct_usertrack_3545_6961 range=chr8:101693225-101693824 5'pad=03'pad=0
strand=+ repeatmasking=none_1
actgcacaggaacatttagtcctagttttgcataaagacccacaatgcactcatgcactctttgg
cattctttaaatcttgccccattgtgacgtggagacatggatcttaagaagaaaaaatggtcttt
gttcatcttaggtatttctttcagtggtacctagaggccattggcaaatatgttcaaattataaa
ccaaa

>hg19_ct_usertrack_3545_16446 range=chr1:206079578-2060801775'pad=0 3'pad=0
strand=+ repeatmasking=none_0
tccttggaccccttcctcgggccccacgcctacccgtatgaccacatcctcactattctcccta
aacacaaatcgagggacatacaaagcaagcactgtacttacttcttagccaatccgtcccctccc
ccatccccaacacacagggagactttatgttagccttgtaaataagaaacaccattccacagtta
ttttt

>hg19_ct_usertrack_3545_10361 range=chr5:166802623-1668028225'pad=0 3'pad=0
strand=+ repeatmasking=none_0
ggaagtaaaccagcaaccctccagcacacaagaaatgagaagagaatttaaaaatcacattctga
tgataacacagcattcaggtgtagtcagttggtcttattagcaagtaaattacattgcaaaagag
gcggcttgataaactatgacctatacctagaaaatgtcctctctccacaagtttctaggtctttg
tattg

>hg19_ct_usertrack_3545_2881 range=chr15:61201309-61201508 5'pad=03'pad=0
strand=+ repeatmasking=none_0
tagagaaatggtggcaaaggaattcttgctgtggtcccagcggtgagcataacagagttacctct
ctccaccccaaatattcagactgcaaggacactggctcccaagcttcgacctgcttggagttcca
ttccaggtactctgctgatgtctcggccagcctcagccacagactcccagctgagctgagctgtt
ctgag

>hg19_ct_usertrack_3545_804 range=chr3:12468401-12469200 5'pad=03'pad=0
strand=+ repeatmasking=none_0
actcagctggcagtgtgcacccttggccacacattagaatcacccaagagctattaaaaatcctg
aaggtcataccccaggcaagtgaatcagaatctctagggatgggtcccaggcattcgtttgcttt
tttagtctccccagataattttaatatgtagccaaaattgataatccctgcactagaccaagctc
cgatt

>hg19_ct_usertrack_3545_4134 range=chr11:58826025-58826624 5'pad=03'pad=0
strand=+ repeatmasking=none_1
cattcccacaagatgcaagtaaagaagcaccgcggacaccgggggagggtcacagagtgacagga
ggactggttgaccagctgactggcctcacttctgacaaatgcaaattacccagccacagtccaac
caagggtatcaaaacaggatctctgcagatggagctcagtgttatgtgttttggatgctcgcaat

aagat

>hg19_ct_usertrack_3545_15835 range=chr2:232233357-2322337565'pad=0 3'pad=0
strand=+ repeatmasking=none_0
acagggtcacactttgttgcccaagctggagggccctggcgtgaacaaagctcactgcagccttg
acctcccaggcacaagtgatcttcctgcctagcctcccaagtagctgggattacaggcacatacc
accatgccccgctaatttttttgtatttttagtagagatgggttttcaccatgccggccaggctgg
tcgcg

>hg19_ct_usertrack_3545_6801 range=chr9:120106380-120107579 5'pad=03'pad=0
strand=+ repeatmasking=none_0
ggaggtgggaaaggagatctctccagcacagcatgcctgaaaaacgatgccagaactacacagcc
ttccacaaaagcacgtggagaaaaaaaaacccatccccaaaagcagcattaacaggagaatatta
aaccctctaccatggcacagtgaataagacttcaaagccctctacgtatggaaatgaatcctgca
ggcag

>hg19_ct_usertrack_3545_1820 range=chr21:44846573-44847372 5'pad=03'pad=0
strand=+ repeatmasking=none_2
gccgccgctgctgctgcctccggggccgcgccgcatgtcagcccggagccgccgcgctccgccgc
acgcggcgcccggccccgccccggcccgccccggcccgccccccgtagtccccgccccctgcccc
gcccccgccgcccgggcgggggccgcccattgacgtcgctttgacgccagagcgacgcgcggccg
agcgc

>hg19_ct_usertrack_3545_2158 range=chrx:66783676-66783875 5'pad=03'pad=0
strand=+ repeatmasking=none_0
caaggaacagaatgactcaatgcaaattatgggacctctttgagtttggggcccctacatttaaa
ctagtaactccgttgcacatattggcacccttcccccaacaaaattactgggcaggaattttctt
gaatccttccgtggcctggaatgatctcccttctcatccttgtgatccacacagctggcaaatgg
caggc

>hg19_ct_usertrack_3545_4904 range=chr8:68880447-68880646 5'pad=03'pad=0
strand=+ repeatmasking=none_0
tggaagcatctgccagcgactggggaggggaaaggtcacttgaggactgtggaaaatattccctt
tctccttagcatcctggaacgtcggctcttgactaaattgttaatagaatctgggagctcggatt
ctggtgctgacatttttttcagggttagttctagaaatgatatttcttaagcagccttttgcgga
gcttc

>hg19_ct_usertrack_3545_17094 range=chr1:214327578-2143285775'pad=0 3'pad=0
strand=+ repeatmasking=none_1
tgatgttagaaagtcattacctatccacaggcgggcatagccatccagagagagatgagcccagc
agagaccccagccctgggtcatcttagtggctctgggtgcaaacagagccacagaatgggagcaa
acactataaacaaagtcaaaggcaaatacctaaatcagtacccccacctcaacctgccagacttt
caact

>hg19_ct_usertrack_3545_6087 range=chr11:73250153-73251552 5'pad=03'pad=0
strand=+ repeatmasking=none_0
ctatgcaacttaggtcccttcccctatctgggtcttcattttctcaattcaacagttaaatggta
caacatttaacttaaaaaatcaattgaattaaatgatctcaaacactccaggaatataggataca
caatcaagactctttatttttatttatttattttttatttttattttttgagacggagtctcat
tctgt

>hg19_ct_usertrack_3545_2515 range=chr8:29374882-29375281 5'pad=03'pad=0
strand=+ repeatmasking=none_0
tcctttgcaaaaactctcccagaagagttacaaacctctgcaactaagtgaattggatatcttga
ttagacttgttcctttttctttgacagggcaaaatccctctctgaggaagaagggaggagcctgc
attgattaacatattgccattcctgaatgaatttacattcatgaattataatattggaccattcc
tctga

>hg19_ct_usertrack_3545_438 range=chr22:21057401-21057800 5'pad=03'pad=0
strand=+ repeatmasking=none_0
gacgctgcaggtgcttgagcgggaccctgaggtctttagtaggggcggagcagcagcgtgagcgg
ggccgtgaccacctgggggtgtggcttaaggcaggccctgaaggcgtgggcggggcggggatgtg
ggcggggcataatcgcctgggggcggggccctgagggctagatacgggcggagcgcggaggggggc

ggggc

>hg19_ct_usertrack_3545_3792 range=chr5:72989645-72989844 5'pad=03'pad=0 strand=+ repeatmasking=none_0
ccttttcctatcctctctgaatggccttgcttttatctctttgccttgtctcctgttttccttct
gagggggcaaacaaattccaaagcagcagttctagccatagagcaaagagaggaccatggtaact
gttttacccagcctggccagcagccacgagatgtcagttcctgggaagtctgaagctccttagct
gctgc

>hg19_ct_usertrack_3545_13421range=chr1:165628977-165629176 5'pad=0 3'pad=0 strand=+ repeatmasking=none_0
tcttcaaggagtgtggccctgacaacttcttggttttggccaagtgaaaccattttgggacttct
gacttccagaactgtaagataataagtttgtgttgttttaagctgctaagttggtggtaatttgt
tacagcaagcataggaaactaatatactgggttcagttctaggaaacagagagggtagcagaaag
cacag

>hg19_ct_usertrack_3545_5006 range=chr14:91827848-91828447 5'pad=03'pad=0 strand=+ repeatmasking=none_2
gagcctgtccgtcacacagctccctcccagcctcaaaactacactccaggctgggtgcagaggct
cacgcctgcaatcctagcactctgggaggccaaggtgggcggatcacctgaggtcaggggttcaa
gaccagcctggccaacatggtgaaatcctgtctctactaaaaatacaaaaattagccaggcctgg
tgacg

>hg19_ct_usertrack_3545_4014 range=chr12:52195934-52196133 5'pad=03'pad=0 strand=+ repeatmasking=none_0
ctagttacaggtctagaaacaaacaggggtgttgggggtgggttcagaagagaatcagcattgtc
ttgcttcaggggagaccactgttgcctcaagcattgcagggttaatctcagacaaatgtggaaag
gctggtggcagcctgggtgggggaagggatattgccaccactgggggtgaggaggctgttttctc
tggca

>hg19_ct_usertrack_3545_1105 range=chr11:10714825-10715224 5'pad=03'pad=0 strand=+ repeatmasking=none_1
gttccctccgcttcccagccgaagaggcaggttattctaggatataggggaggcttacctaaaac
tgagttattctcctttcctcctctggccagcctctcttcactctggggagcttttaccatttaag
gtcaatgttacatctggctccggagctcagagccgagaagcctctggctgcagaacctcggccgc
acgcc

>hg19_ct_usertrack_3545_6560 range=chr11:77851953-77852152 5'pad=03'pad=0 strand=+ repeatmasking=none_0
acccatgtgaatatgattcacagatttctagctcagcactcttctctcactttcagaccctggtc
tccagttggctacctgacctcacgtagatgtttttgttttgtttttgagagacagggtctcattc
tgtggcccaggctggagggcaatagtgcaaacacagctcactgcagcctcgatctcctgggctca
agcag

>hg19_ct_usertrack_3545_3072 range=chr14:68809048-68810247 5'pad=03'pad=0 strand=+ repeatmasking=none_1
tgttgctatgtacccatgccttctctctctctctttctctctctctccctctctctctctgtctc
tctctttctaatgaagagtttagctttggagttaatgagcaaaagatttccttaccataaagtga
agcccatcaagtgaaagcaagtagaaaggcgcagaaaggtttatccttgtcaccatgtggaagga
cactt

>hg19_ct_usertrack_3545_104 range=chr2:314201-3144005'pad=03'pad=0 strand=+ repeatmasking=none_0
ccgcagggaaagcggtgaagcgggcagggcgagctggagcacctgtgtggcatcatcagctccag
cctgaagttggtcttgtggtggtgttttcgttgctgccttcatctgatagccctgaagaaagtgg
cggcctctgggaggcgaacggggaggcctcagtggccaggggctcggctttgtgacccatgcagg
agata

>hg19_ct_usertrack_3545_3772 range=chr18:51912403-51912802 5'pad=03'pad=0 strand=+ repeatmasking=none_0
agagctctttttacagacatcatacacacaacacatacagctacgcagacaggtaggagaaaact
caatcccctggtggagccctgtaagagagcaggctaggaaaacatacagatatcaaaccagaaag
gacttattccctaaggcaggattgctaaacaaagccttgccgcacccccaggatgtagaacaaga

tggag

>hg19_ct_usertrack_3545_5330 range=chr13:107332600-1073331995'pad=0 3'pad=0
strand=+ repeatmasking=none_1
gaatgaatgagttccgctctagcaaaaagtgcctgtgatctgaaataattagtcatcacaatcag
agagaatgctatgtgactagtaaatcatacaattgaggaattgcatttaaaaattagagttatca
ggaacacctaaaaagctgtctattcaagtaagcccctggagctttgataattatcaggaggcaga
atgta

>hg19_ct_usertrack_3545_3927 range=chr2:47982497-47983496 5'pad=03'pad=0
strand=+ repeatmasking=none_4
aacttgtgagaatcaaagagctgcagtattgtggtgggattataggcagggaggctgatgttctg
cggcaggagtaggtagcaaggtagattgctccaaggatcttcactttgctttacctgcttccaac
ctgaagagctttcagattttttttaacttttattttttgtctattcattttttttttttttttg
gacac

>hg19_ct_usertrack_3545_11710 range=chr2:170932155-1709337545'pad=0 3'pad=0
strand=+ repeatmasking=none_0
cccttgtgtccctgctccactcttgtgttcctaaccctgatcctgaccctaggtcaaccagattc
tctttctcaggaatgttaaatctgtatagcgagacttagcagggtaaatggtgacaggagctgta
tagtccaacagtactacccttgaaaaactgtctgtgaattactacttctgaattttttttatcagt
taaga

>hg19_ct_usertrack_3545_1424 range=chr3:18384997-18385596 5'pad=03'pad=0
strand=+ repeatmasking=none_1
ctgaagcttttaattctttaatccaccatggaatgttgcttaattacatattttattatgcataa
ctgaaaattatgtactttagtttcaacaatgatatgaaagcatgtattgtttataaatagagact
ggtttgtctcttttttttatattacaaatatttgtggaattctcacatgttctccttctaaaactt
ggctt

>hg19_ct_usertrack_3545_12013 range=chr3:169777107-1697777065'pad=0 3'pad=0
strand=+ repeatmasking=none_2
gatggggaggtttgttacacagggaaacgggtgccatggtggtttgctgtacagattatcccatc
acctaggcattaagcccagcatccattagctgtttttcctgatgcttttcttccccccactcccc
cctctagcaggcccaagtgtgtggtgtttcccaccatgtgtccacgtgggaaccagattcttgct
gtggg

>hg19_ct_usertrack_3545_1361 range=chr10:16401995-16402794 5'pad=03'pad=0
strand=+ repeatmasking=none_0
ctcccacccccagaagatacatgacccaagccagttagagccacatttatgtatgtacttaagaa
agcaaaacagatcaaattacacatttcaaaacattagctctgtacactataatcttatgtggggt
gataatgatgacaatgggaatctgagaatgggggaggggttgagagaggagaaaagaagaaactg
gtcag

>hg19_ct_usertrack_3545_5147 range=chr11:66579425-66579824 5'pad=03'pad=0
strand=+ repeatmasking=none_0
tggggagagagagaaaacacttttttaaaatgtaaaaaacaatttctaatccaaaaatagaaaaga
ggaaaaaggaaacaaagaatacaagcaagaaatggaaaaaagatgatggatttaaactccactat
agctatcttcatattacaagtaaatgatttaaacaacccaattataagacaactgcacatcttgg
ctcct

>hg19_ct_usertrack_3545_324 range=chr14:23648161-23648960 5'pad=03'pad=0
strand=+ repeatmasking=none_3
gtaatcccagcactgataggccgaggtgggaggatttcttgagcccaggagttggagaccagcct
gggcgatacagtgagaccctgtttctacaagaaaaaatttaaaatagctaggtgtcgtggtgtat
gcctgtggtcacagctactgaggaagctgaggtggaaggatcgcttgagcccaggagtttgaggc
tgcag

>hg19_ct_usertrack_3545_181 range=chr3:3122201-31226005'pad=03'pad=0 strand=+
repeatmasking=none_0
tctctgtgcttcactgtgggaagccctgttctattgtttgtatccccgtttctgataaggctaag
tgtgcaggcagcttctctaattaaaggtggctgctttgcaaactaccgtttctcctgacattccc
caaactctaggtgggactgaagccctttattaaaaacctatggctatactatttatttatttata

tgaga

>hg19_ct_usertrack_3545_199 range=chr17:1393251-1393650 5'pad=03'pad=0 strand=+ repeatmasking=none_1
gctctcctgcctccactcctcacaccaagtagtcacgacctgctaaaacctcacctccaggccgg
gcgcggtggctcaggcctgtaatcccagcactttggtaggcctaggcaggtggatcacaaggtca
ggagattgagaccatcctggctaacacggtgaaaccccgtctctactaaaaaaaaaaaaaaatta
gctgg

>hg19_ct_usertrack_3545_5022 range=chr9:97742380-97743179 5'pad=03'pad=0 strand=+ repeatmasking=none_0
ctgtggaaatcacctggcctggagctcctctccccgtccactctcctctggccctgtgcctccca
gagaccattggacagcccaggccccttcgccaaatcaaatgctggccatggcaagcctaggccct
gggaagtctgagggtgggatcccagatgcgcggtcagaggaccctgcaggctgggagctctctca
gtctg

>hg19_ct_usertrack_3545_6986 range=chr12:97645870-97646069 5'pad=03'pad=0 strand=+ repeatmasking=none_0
atgatggtgacagggtctctgctgtcattctcattttattgagttgacagtgtgaaaagataata
aatcttgagaccccccaaatcactaagctaaagggaaaagtcaagctgagaactgcttagggcaaa
cttccattctattcagtcatcgctctgctcattgagataaatgcatatgtgactgcttcctttgg
aaagg

>hg19_ct_usertrack_3545_1526 range=chr20:20421201-20422000 5'pad=03'pad=0 strand=+ repeatmasking=none_0
tacctgctctgaatagaagcacttttggcccccccttgtcaaagactagggaagaaagcccgcgct
ctggttggcagcagtcttggattctcgcccccggccatactgtgcaatcacgctgtttattccac
tgtaacaatcagacagcgagagcagagcacctggtactccccggcccgggccttccttggcagct
gacgg

>hg19_ct_usertrack_3545_16441 range=chr2:237568062-2375698615'pad=0 3'pad=0 strand=+ repeatmasking=none_6
gtgaccctaatgtttcaagtcaagctacaaagctaccttatttttgttcttactgtcactaaaga
tacatctaaaattataaaataatttcatgacagtaggtaaagtagaatcagttattttgggagac
attatcaatattcagctgcaataaaaaattaatgggtaaaagcaattctgatgtataataaagat
taact

>hg19_ct_usertrack_3545_11721 range=chr3:162438307-1624393065'pad=0 3'pad=0 strand=+ repeatmasking=none_4
agtttcctgaccaaatgttgttcaagagaaaagctagaaatcagatactgaggtcacaaatgttt
tcagcatttctaactgtatatttgatgtcctataacctagaccacacatttttaactgctaaaac
ttaggcaatttttattacgggataatactgacaggataaaaagcagtttgaacagcaagctgtca
ttagc

>hg19_ct_usertrack_3545_4858 range=chr15:75929146-75930545 5'pad=03'pad=0 strand=+ repeatmasking=none_0
tttatttacttatttatttactctgtcgcccaggctggagtgcagtggcatgatctcggctcact
gcaacctctgcctcccaggttcaagctattcttctgcctcagcctcagctttgcaaacctagccc
aagtagctgggtttacaaacatgtgccatcacgcccgactaatttttatatttttgctagagatg
gggtt

III. 200 DNA Non-Enhancer Sites

>hg19_ct_usertrack_3545_158 range=chr12:78319870-78393869 5'pad=03'pad=0 strand=+ repeatmasking=none_323
aattttctcatttctcataaagtttaacagttgtttatttgagtcagaattcaaataagcttct
gtacattacaattggttttaagttcttataagactctataggttttcccttcataatttttcttg
caatttatttgttaaagaaattgggtcatttgtcctattgagtgctccactgtctgtttttatta
ttgta

>hg19_ct_usertrack_3545_57 range=chr7:10106276-10452075 5'pad=03'pad=0 strand=+ repeatmasking=none_1151
actggttatcttttaggactagttaatataacccattctctaaccaacagataactcaaccaggt

tcagcacctgatgggttactcttcaaggactcccttctaaatctcactttgctgtgtccacaatt
ctaaattgctatacaatagcattttctcactctcattcagtattttacacagaaagatatgcctt
gaacc

>hg19_ct_usertrack_3545_762 range=chr2:59404297-59409096 5'pad=03'pad=0
strand=+ repeatmasking=none_15
atgcatatgttcttcagtaaacagagcagccactggtaccacaggacagccagtgctgaggagga
gagggtgagaggggactctgcaggaggaaagaacagggctcagcagcaagagctgagaaaaagga
tgagggaagctaagaccttgaaagaaagcagcgctgggctctgctccaagcctaaatttatgaaa
acatt

>hg19_ct_usertrack_3545_78 range=chr11:21614425-21696024 5'pad=03'pad=0
strand=+ repeatmasking=none_133
ctgctctcctcgctctataaaagtcagagtgcctaagctgttaatttgcaaaccccttcttaatc
taccctctattcatagtttatatccagaactatggtttaatataatcgtaaggccattgactttt
gaatacgtagctccagtcttagtctcactggactaggtcctatatctaaccactaccacaaagtc
tccta

>hg19_ct_usertrack_3545_9 range=chr17:51184002-51429801 5'pad=03'pad=0 strand=+
repeatmasking=none_60
gcttgggtatatattgtccaatatagcaggcctcatgtgctccttaaaccagggctacttcaaat
agagttgccctttacagttggataatggttaactttaaatagcaagatgtatctagcaagagggt
ttctgaaaacccctgggtttctttcttatattgtcctctatttcctaaatttcatttttttaactt
acttt

>hg19_ct_usertrack_3545_997 range=chr2:95475274-95522073 5'pad=03'pad=0
strand=+ repeatmasking=none_60
tagagggcactcctttttttgttcgtccctctttagagttactacataggagctcttcctcagggc
aagcagtaattctggagttttcaaaactttcaccaatattcagctcgaacttgtttgtaatgaat
tttaaagaaagtcgtgaatatacagacagattattccctttatcacaattcttacccagttctgg
ttctt

>hg19_ct_usertrack_3545_6 range=chr13:49222200-49305199 5'pad=03'pad=0 strand=+
repeatmasking=none_159
tgccaatttaccagaagggtttccaatgtgggagttacaggtggcagagacagtgggcaaatagt
atactcagaaggtcactgagtagcaactagggaggcttccgcatggcaggtctaccagttttccc
cctctgtctcaggactgctctacacagaagcctaccattatttccttgtaaagatgatgcaaagt
gcacc

>hg19_ct_usertrack_3545_419 range=chrx:113089936-113172135 5'pad=03'pad=0
strand=+ repeatmasking=none_410
ctgattgtgtattttcatataatctgtcttcaagctcactagttctttcttctgcttgatcagtt
ctggtgttggcagactgatgcatttttcagtttgtccactgaatttcttagctccagaatttctg
cttaatttttaaaaattatttcaatctctttgttaaatttctctgatagaattctgaatttcttc
tctgc

>hg19_ct_usertrack_3545_270 range=chr4:96916178-97273377 5'pad=03'pad=0
strand=+ repeatmasking=none_941
cctagggaatagaaaggagcactttctgtcttggcaaagctacactggttgtaacagtggttctc
aaacccaagattattaaaatgtaagttctgattcagtagttctggggcaaggcccaagcttctgc
atttgtaagaaggccccaaataatgtacatgtcgttgcccacataccatacttttaagacaagaa
gttag

>hg19_ct_usertrack_3545_165 range=chr5:63555645-63680644 5'pad=03'pad=0
strand=+ repeatmasking=none_476
ggatgtccactttaaccactgctattcaacattgtgctggaaactctagctggaacaatttgatt
taaaaataaataaataaataaaaggcatccaaattggaaaagaagtagtaaaactatctgtattc
tgatgtcctaatcctgtatacagaaaatcccaaagaattcagaagaaagctactagagctaatta
attta

>hg19_ct_usertrack_3545_667 range=chr7:34174276-34205675 5'pad=03'pad=0
strand=+ repeatmasking=none_52
aaccaggaacctaagcctgtgccctatgccctgatgtctttcttcaattggccttgtgtaccttt

taaaactccctaggtgattctgacctgcagccagtgttgaaacccctgagttagtttgtgtgttt
acttgggtggctgtggggaggtaggatgatggatttttgggggttgctgaagcttcctcagctgc
ccctt

>hg19_ct_usertrack_3545_54 range=chr13:31810801-31876600 5'pad=03'pad=0
strand=+ repeatmasking=none_278
tctgaaatttttttcttgtgtctctgccaggttttagtatcagaatgaggctggccttataaaa
tgagttagggaggattccctctctttttattgtttggaatagtttcagaaggagtggtaccagct
cctctttgtatctctagtggaattcggctgtgagtctgtctggttctgggctttgtttggttggt
aggct

>hg19_ct_usertrack_3545_788 range=chrx:24524680-24558879 5'pad=03'pad=0
strand=+ repeatmasking=none_142
agaaaaaggtggtaagttctatgttatgtatagcttatcacaataaaaaatgttaacattataaa
ggaaaagttatcaatcacagaacaagtttcactgttttatggtaataaggaaaataatctgtagt
tttcttctgactatgtacccttaagcttcaactctactagcaacagtggggttctgcttcattta
actta

>hg19_ct_usertrack_3545_61 range=chr1:251538-267537 5'pad=0 3'pad=0strand=+
repeatmasking=none_34
gtttagagaataacatttgatggaatcatgcttttactttctgcttacgactcaattgtttgtac
tgacattaacatcccaaatccttagcatggcctacaaggccctgagcaatgtggcacctgctgaa
gcctgctgcctcatttaataactctttgtctctttcccagatccagccactctaacatttttttag
ctcct

>hg19_ct_usertrack_3545_1210 range=chr12:110663018-1106832175'pad=0 3'pad=0
strand=+ repeatmasking=none_33
tgtctctgaccttctccatcttttttgttcatatatatttttcctccctgtttatgagtgttat
actgttcaaatgttttctgattctctctcacacaaattttcttgtcagtggtaggttcacttttg
aagcatctttcaaaaatatattttcattttaaaaaatatatgccgtaggtatgattgcataactc
tgaag

>hg19_ct_usertrack_3545_1825 range=chr1:198655578-198668377 5'pad=03'pad=0
strand=+ repeatmasking=none_25
cataggtccttttcccccaacctttcttttggggtatactcttcctacgtaggacatcatctctg
tctgttttgctttggttttggtctcctgattttcctgcccccacctttgctctttaataatccat
tttccatttcctgaaaggttgatctaaaattcaaatgtttctcctttaggcctccctttaaagac
atccc

>hg19_ct_usertrack_3545_321 range=chr9:5818601-58320005'pad=03'pad=0 strand=+
repeatmasking=none_43
aaaagaagtgcttcaattaggccatccactaaaaggatttaggtgatagtctatttcaggggtgt
ccaatcttttggcttccatgggccacattaagagaagaactgtcttgggccacatataaaagaca
ctaacactaacaatcactgatgagctaaaaaataaaataaaaattgaaaaaaaaatctcacgttt
tgaga

>hg19_ct_usertrack_3545_248 range=chr2:135763731-135800930 5'pad=03'pad=0
strand=+ repeatmasking=none_16
agttttagatgcttatgggtcatttgaatttttctttttttagtattatatatagtttttgtgtag
atccttagtctagaacactctaagagacctcaaaacacatttctaccatttcatttaattacagt
gattcagtgaaatttttatgctgagctttgagaaaacagcagccttcatttggaagtgttagtca
tacag

>hg19_ct_usertrack_3545_435 range=chr4:176458407-176691406 5'pad=03'pad=0
strand=+ repeatmasking=none_441
ctctctaaaacatgtagaacaaaaataatgagctcatgtctgtaagagtgccataaaatagaaaa
aaatattagaactctttttataattggtctttaataaattatcactgcattttagaagcaaactt
acttcaatgctgtggtcatgtaactcttttaattgataactagtttaataaacatttattgcttg
ctatg

>hg19_ct_usertrack_3545_292 range=chr5:147101408-147202207 5'pad=03'pad=0
strand=+ repeatmasking=none_170
acaaataatgaatgactaggggtttctgagcattccatgatactaggatataagtcaattaaaca

cacaccctttgaagagccacaatgaaaattgaaatcatttaatttattctgcatatatatgtata
tcctgtattgctctaacaaaaagaaacacaggttatttgcaaatatccatgtgaaagcatgagaa
ataaa

>hg19_ct_usertrack_3545_770 range=chr8:48146853-48172047 5'pad=03'pad=0
strand=+ repeatmasking=none_61
acaggtgatctgcccacctcagcctcccaaagtgctggaattacacgcgtaagccaccacaccca
gcctccaattttattattttaggtgctattataaataaattattctcttaatcttctttctgggt
tgtatagaagtataaatgacatttgtatgttgatcttatagcttacaactttctgaatttgttta
ttagt

>hg19_ct_usertrack_3545_1451 range=chr1:153240977-153250376 5'pad=03'pad=0
strand=+ repeatmasking=none_10
taacaaattattgtagctattattatttttaatagcttagtcttttaactttcatactaaagata
taaggaatttacacatcaccattacagcattaaagcattttaaatttgactatataatactttta
ccctgtgagttttatattttctatgtttttgtgttgctaaccagtgcccttttctttcatcttaa
agaat

>hg19_ct_usertrack_3545_121 range=chr10:56851795-57019594 5'pad=03'pad=0
strand=+ repeatmasking=none_3
tgagaagaaaacaaattaatgacaaacagactagaatgcaccaataaaaacttacaccaagaact
gttttagaaaacatgcttattgctctgtttgtttctattctctttgtctcatattgtatttcaat
tttgaaagtaagtttatgtaatataaataagttgatcaattaacaaaatacataatctagactca
catat

>hg19_ct_usertrack_3545_612 range=chr6:30182622-30186821 5'pad=03'pad=0
strand=+ repeatmasking=none_14
atttaaaatactttgtattccaaatttggtgacttttaaatgtctattagctcaaaagttagtga
aaatatattgtataatatataatgacaaattcaacttaaaaaaaatttttttttttttttgaaa
cagggtctcactctgtcacccaggctagagtgcaatggtgcaatcatggctcactacagcctcga
cctcc

>hg19_ct_usertrack_3545_55 range=chr6:2750602-27612015'pad=03'pad=0 strand=+
repeatmasking=none_44
tgctgtggcacaattatagctcactgtggccttgaattcctggcctcaagtgatcctctcgagta
gtgaggaccactggtgcacaataccacgccaagctaattgtttaaaaaatttttgtagagatgga
ggtctcactttttttgcccaggttggtcttgaactcctgggttcaagtgatcctcctgccttggcc
tcgca

>hg19_ct_usertrack_3545_54 range=chr14:42080651-42293650 5'pad=03'pad=0
strand=+ repeatmasking=none_995
gcttatcatccatagtaaaatcagaattaccctcaatatgacagccaaggtattgttgactgtct
cattttcctatcagatccagataaaataggagaatagtttttcattgacttacgcaatatttaca
tggccattaaaaagaaagatacaggttaaaattattttcctaaattctgaggtagagtgaaaatg
aaata

>hg19_ct_usertrack_3545_75 range=chr17:63290739-63354738 5'pad=03'pad=0
strand=+ repeatmasking=none_1
ccctactaccacatagaggtgttgggctatttaagtgagagtccacatgaatagtgcctgccgcc
tagtaagcactcagtacatctgagttatttcttattaattcgacaagatgttcaggagaagctgg
tgcaaacttgcgtggctcctgccccacttgcccgctcctcaccccccccttaaagaagcaagagga
ggctg

>hg19_ct_usertrack_3545_376 range=chr4:149516351-149746750 5'pad=03'pad=0
strand=+ repeatmasking=none_106
aaacagtaatcatacctgaagaaattaaaatagagccacatagcaaaatgtgacctaaatgcagt
acataagccaaaaacataaattcataactagataaatcaatggatagactttatgctaccagcaa
taatgtagtaattgggcctaacttaccctcccacagtaaaaaaaaaaaaaaaaaaaaaaaaacaa
acgga

>hg19_ct_usertrack_3545_138 range=chr13:86526400-86760199 5'pad=03'pad=0
strand=+ repeatmasking=none_492
tggaaaaaatgccagataagaagcaagactagcttgcagctcccactcagacagaacagtgtgtg

aaggctcatcttgtgaacttttgctccaagaactgccacagaaatgtttgagaaaaactgaaaga
attcacagaccctttgaaaaaagctgcttactgctgcaaactctgattgatagacaaccaaaaag
cacac

>hg19_ct_usertrack_3545_1548 range=chr1:160847577-160854376 5'pad=03'pad=0
strand=+ repeatmasking=none_9
tagctaagagggaccttttcccaaccccagagatgaagagctggtcctcagagagggctcctcat
ctgccagaggcttgtgaagagaaattcaactctgcaggaagatcaccactgaacatctccaaggg
ctgagttgcagaaattaacagatgtattcactctgttcctgtttacatttttcttttctccttta
tttta

>hg19_ct_usertrack_3545_143 range=chr8:43392244-43759643 5'pad=03'pad=0
strand=+ repeatmasking=none_1590
agaatctgtgaaggaacatttgtaagcccattgagtttgatagagaaaaacggattaccaacata
taaaaacttaaaagaaactatctgtgaaactgctttgtgatgtgtggattcatctcacagagtta
agcctttcttttcactctgcaggttggaaacactccttttccagactctactaagggatatttgg
gagtt

>hg19_ct_usertrack_3545_101 range=chrx:4500201-51864005'pad=03'pad=0 strand=+
repeatmasking=none_1231
ggtgattgaaaaactacatacctggtactatgctaattacatgggtggtgaaataatctgaacac
caaacacccgcgacacataatttacccatgtatcaaacctgcacatgtaccactgaacctaaaat
aaaagttaaaaaaaaaagatctgatagccaaactataccatggtggttacacaattatgtacata
ttttt

>hg19_ct_usertrack_3545_310 range=chr5:154640808-154832207 5'pad=03'pad=0
strand=+ repeatmasking=none_453
caataagaaaacaagcaataagaaaccatattggccatgtggataacttctttggtaaggtgtct
tttaaggataaagatcttaaaaggataaaggataaagaccttttaaggataaagaccttaaaaga
caccttgccaaagaagttatccacatggcaaataaacatattaataaatgctccaaataatgtgt
tataa

>hg19_ct_usertrack_3545_187 range=chr1:104092478-104271877 5'pad=03'pad=0
strand=+ repeatmasking=none_107
gtttttttgtatgcaattctcgatctttaaagagatgacaacaaattttggttttctactgttac
gtgagaacattaggccccagcaatatatcattgtgtatggaaaaataaaagtgctgccagaacca
aaaaaacattaatttctaaaaggtcatttagatgatttccatgagagactttttaatgttcttct
cctgt

>hg19_ct_usertrack_3545_98 range=chr13:69502000-69861199 5'pad=03'pad=0
strand=+ repeatmasking=none_395
aaaataagaactttcagaaaactgagaattaaattaacatatataaacaacaaacataagattta
ttctttacctgcaataaaatttgcaccataaaaatatttcacaaatattttatatattataatct
tttaaaaattatatttttgtagaaaaatactaatttttttcattagtactaaatctaaattttatt
tttca

>hg19_ct_usertrack_3545_232 range=chr7:125237165-125310964 5'pad=03'pad=0
strand=+ repeatmasking=none_195
aaatacaaaagactgaagtttatttatataaaattctggaagatgaaaaatctagtgacagaaaa
ttattggttgccttaagatgaaagtcaggagagagaatccactgcaaaggaccgaaggaaacttt
ttcaggtaagaaattaatttgatatcttacttggctttacacaattactaaaatcaaatttcaaa
cttaa

>hg19_ct_usertrack_3545_11 range=chr8:13585630-13777229 5'pad=03'pad=0 strand=+
repeatmasking=none_747
agtcaatccatgtaatgtgaacaagaaatggttctcgatacagttgtcattgttttaagccactg
gtattttggtatcaatttttactgtaacaaaatctatctaataaacatctccaagatataaagta
cccaaactagtcaacctattgttagatggccagttagtaccttccagggaataaaaccaaattgg
gaggt

>hg19_ct_usertrack_3545_1209 range=chr9:128891580-128906979 5'pad=03'pad=0
strand=+ repeatmasking=none_5
tgtgggtggtcccggctggtcattccagctttgactttggagggagggagctggaagaggaaatg

ggctagagggtgatggacacccatccattctatggggcctgggtctctcaatgacccacagatc
actgtggcaaatatgacactattggccaaagttttgtaattggaacgggagtcagtctacagcag
tggtt

>hg19_ct_usertrack_3545_130 range=chr1:71151413-71210812 5'pad=03'pad=0
strand=+ repeatmasking=none_292
ttttgttgtggtagtagttactattctttcatttccatgtttagaaccctcttaaagacctctct
ggatcttaaggacctcagcttgtctggaaaggattttatttctcttttgtttatgatgcttagtt
tggtgggatatgaaattcttggttgacatttattttctttaaggatgttaaaaatagctttctaa
ctttt

>hg19_ct_usertrack_3545_388 range=chr4:157598751-157812350 5'pad=03'pad=0
strand=+ repeatmasking=none_919
agagtaaataaaaatatacgttttgcttaccttttaaatgttctttctcctcttttctatgccag
tccagatgcttggatatagttcctacctgccagtagatggagatagtaaaattgagtcttttggt
gacctcaaatcctaatctaggtggttcagaatcttcaatttcagattttggaagttaaaaaaatt
tgaag

>hg19_ct_usertrack_3545_333 range=chr1:238402378-238546577 5'pad=03'pad=0
strand=+ repeatmasking=none_275
ttaatgtagtgaagatgatgaagagaaccaggtttaacactgttagagaaggtatttacaaacaa
ggctaaaaagaatcatgaagttttggattaaaattggaggtattgataggaactcattgttaaaa
gtatatacctatagatatttagttgtggaattattattattactattattttgagaaggagtttc
actct

>hg19_ct_usertrack_3545_264 range=chr7:144206668-144382667 5'pad=03'pad=0
strand=+ repeatmasking=none_749
actcactatcacaggaacagcacgggggaaccgcccccacggtccaattacctcctcctggtccc
acccttgacacgtggggattatggggattacaactcgaggtgagatttgcgtaatgacacagagc
caaacagtatcaagatccttgaataacgtcctttcattcaaggtcatttccttataaggttgatg
aggaa

>hg19_ct_usertrack_3545_1010 range=chr11:83382753-83392152 5'pad=03'pad=0
strand=+ repeatmasking=none_19
tgattttgttcaaaatacaataattcttgagcctggtaacatatattttaaagttcacattgatt
gactccaaatctgtatccacattgtaaagaatcagaacatttgactgtggtttagcttataattc
aacggtcatttttcaacaacaaatttttgtgagactgatactttaggttttctgttccttattct
actgg

>hg19_ct_usertrack_3545_231 range=chr9:119906980-120009579 5'pad=03'pad=0
strand=+ repeatmasking=none_15
agttaagtattaataatttctgttcagagatgaggatagcaagactcagaaatgttacatggttt
ttccaggtcatgcagcttagggacgatagaactgggatttggactcaggttatggtgactccaat
gctctggccttatctgttgctgcactctataagtactccctctctggttggctggagatgggtat
gttaa

>hg19_ct_usertrack_3545_280 range=chr7:152557268-152591267 5'pad=03'pad=0
strand=+ repeatmasking=none_146
gtggacaatggtcttggatgtctgacacctcatacgtcaggagctttttttgattgttagctgcta
gcttctgcgtgatttttgtgtcttttaaatttttattttgtttttgtttcctttacttttggggt
catattcaagaaatcattgcccagaccaatgccatggagtttttttcactgttttcttctagtag
atgtc

>hg19_ct_usertrack_3545_541 range=chr13:70099200-70111599 5'pad=03'pad=0
strand=+ repeatmasking=none_27
tactgcaccactaaccagtcattccctcatctctctccctctccttgggcatctatattccctga
gacacaacaatactgaaattaggccaagtaataaccctacaatcacctttaagttttacaatgtg
ttgacttaaagacaaaaataaaggcaaaattaatataagtggatagtttacttgagccaaatata
aggac

>hg19_ct_usertrack_3545_92 range=chr11:29454625-29641824 5'pad=03'pad=0
strand=+ repeatmasking=none_659
acaatgcttagagttcaaataataatctagattgataagcattttcatctaaaatgctccttgac

aatcacatgtatgttacaaatagcttttatttttaaaacaacatgggcttagaagtcaggaaaa
tttaaatacatgtttattttctttgtattatacaagtatgtgtatgtgtgggcacatacacacaa
acaca

>hg19_ct_usertrack_3545_106 range=chr8:24435111-24542910 5'pad=03'pad=0
strand=+ repeatmasking=none_274
aaatctggttgtgtctttatgtcacactcagcgcctcttctcctcctcactccctgcttgagaac
ttctgtttttatccaggtgtctcaagcaagttttggaaattcagtattccttcaaccactgatta
gtgatgggaccttatgaaatgtcaaagagcatttattttgtgaaagtaagaataacaataaagag
tagta

>hg19_ct_usertrack_3545_1512 range=chr4:127513551-127525350 5'pad=03'pad=0
strand=+ repeatmasking=none_27
ttcctgggagtagcatcagcataggtagcaaactgtaatataccctaaatcagattactgccatt
gaggttatcacttaaaatatcatagttgcttaaatttaaggcaaatgattcttcagttatttttt
ctataaattcaatgtaataagttatgaatcatattttttattttttttatccctggagaaaaaaattc
atacg

>hg19_ct_usertrack_3545_1202 range=chr10:100551011-1006124105'pad=0 3'pad=0
strand=+ repeatmasking=none_241
agagaacatcaacaatgtttaagacaacatattaaattaaagaaaggggatacaaagataaccag
tcctccaaatcctcacaatttagtaggagaaataggagatacacataaatagttagaataaaagg
tacacaggtcaagtgaccaagcctgattcatttttatattcctcacaggaacacagtgctaggtg
ttcaa

>hg19_ct_usertrack_3545_63 range=chr17:51430002-51594001 5'pad=03'pad=0
strand=+ repeatmasking=none_659
aatagctttaatctgtttgtaatcatctcttattaatgtgatacattggatatactgagaatata
gatttttccaagaaatatattgacaaagtgaaattcttctggaattgtttctacctacatatccc
accaacaactgaagtcaacaggcagtgacaccagcacttactctacaaagcttgcaatattgaat
ataat

>hg19_ct_usertrack_3545_213 range=chr7:118015365-118343564 5'pad=03'pad=0
strand=+ repeatmasking=none_1557
ttggtttcatcacaatgccataggtataaaattaaattagttaaaatcactaaaatggtagattt
taagtggccactccatgaaaaatgagaagtatatgaggtaatgaatatattaattagtttgttct
agccattccactgcaatgtacatatattttaaaatatcatgtatatgacaaatatacacaatttt
tattt

>hg19_ct_usertrack_3545_1268 range=chr8:114184425-114214424 5'pad=03'pad=0
strand=+ repeatmasking=none_51
gaataggttgatacatataaggaaacacagaatgtaacttcagaaaataatcttagggttgcact
ttggaaagacttgaaattagctgttttagggtcaatgctaagctgctgctgatgctgccaaaaaa
aaaaacaaaacaacaaaaacaaaaaaacaacaaaaacaaaaacaaaaaaaaactcacaacgaattg
cttaa

>hg19_ct_usertrack_3545_1451 range=chrx:101860545-101905944 5'pad=03'pad=0
strand=+ repeatmasking=none_225
tgttatgacctcctgcgggtcctgacagggaaaggtcctcttttgggggcaactgtggggacggt
gcaatgccaacgtgagccaccatttgaaatgagaaacagcggtgtggcagaaagaaatgagtctg
gtgcagaccagtggttggtttcctagggttagaggtctaggtctaccttcttttagaagaggggc
agtgg

>hg19_ct_usertrack_3545_273 range=chr13:29395601-29484800 5'pad=03'pad=0
strand=+ repeatmasking=none_25
cttattagtctttttactctgtttactgtagacccattgtaccagtatgttcagttcccatagaa
aaacaaaagagtgtttacacacagtcattatagataagccagcagtgtgtgtgattagggccaaa
ccagtattgatgatggcctgatactgactttagaaagtttgactcaccttgcagaaatgaaaagc
caaca

>hg19_ct_usertrack_3545_235 range=chr9:120932780-121147779 5'pad=03'pad=0
strand=+ repeatmasking=none_873
aattacatagagtatgtgataccctgcttaatatatgcatatatagtaaaagtgtgcaaagaaag

catcagaagacacatacgtaattcaggaaaatgatcagggtgaagcaggagagagaaggaggtgt
gtccaagataaaacaaagagttaacttcagtgatgttagaattgttttgctttaagtcaggtgaa
atgct

>hg19_ct_usertrack_3545_233 range=chr7:125342365-125764764 5'pad=03'pad=0
strand=+ repeatmasking=none_1248
ctgtggaaccatgaattaaacctcgtttcttcataaattacccagtctctcaggtagtcttcata
aattacccagtctcaggtagttctttgtagcagtgtaagaacagacgaatacaactacttaatag
attcttgatcttaggtaacttacaacttatttaaatttcagtgttcttgtattatatgtaagcta
taata

>hg19_ct_usertrack_3545_221 range=chr5:101313902-101559501 5'pad=03'pad=0
strand=+ repeatmasking=none_30
gaatatgttgagtacttaatatgtgttggaaataaaagcaaatgcatgaataagaaagaaagtct
ggataattaaagagtttgttaattatactatgttcccaaagttcaattaaagtattgggccagaa
ttcattagatagcatgccttgttgaatacgagttgtgttggctgactctcacataaatgagttca
tagag

>hg19_ct_usertrack_3545_383 range=chr4:156363951-156395750 5'pad=03'pad=0
strand=+ repeatmasking=none_15
aatataaataatccttattgccattttaagtatattctaaattttttcttttttgatctcctgcaaa
cctaaacagtatattatagatgtccaaaaatgtatttatttgcaaacactttttataacttggga
actttttttctgagataaacctctgaaattttttaatttttaatagtaaatatgatttgatgtgtgt
ttgct

>hg19_ct_usertrack_3545_100 range=chrx:3881201-41810005'pad=03'pad=0 strand=+
repeatmasking=none_882
ggtaaaaattgggatgttagcattgtctttctggagactttggtgggctggagtctttgcttagt
gatggctgttgaggttgcttcattaggggtaggttaatttggtaatgagcattcaaagaccccaa
aaagacaccctactcccctgtcagagcatgcaggaaccaagaggactgcaaacttaatgacatgg
gcatg

>hg19_ct_usertrack_3545_64 range=chr18:29080803-29133802 5'pad=03'pad=0
strand=+ repeatmasking=none_112
atatacaaaaaattagctgggtgtggtggcgggcacctgtagtcccagctactcgggaggctgag
gcaggagaatggtgtgaacccaggggacagagcttgcagtgagctgagatcgcgccactgcactc
cagcctgggcaacagagcaagactccatatccaaaaaaaagaaaagaaacatgatttaattctgc
ccaaa

>hg19_ct_usertrack_3545_1098 range=chr7:81630065-81646664 5'pad=03'pad=0
strand=+ repeatmasking=none_25
gcaaatttcatcttactgtaaaacgtggtgtcagtcttttaatatcttccaaagacacatctact
cccatcacaccaagaatcagctggttctataataagagggcaaaaaatggcattttatgtcaaaa
atttacgtatttttacacaattatcatatgatcatcttttgcttgcattatggttatattccaca
ttagt

>hg19_ct_usertrack_3545_102 range=chr4:19226903-19415302 5'pad=03'pad=0
strand=+ repeatmasking=none_759
cgatccattatgtggagtgattatttaccatacttctctggcttgattccttttgatactgatct
tatttataagtgctttcagaaatttcttctggaacaaggaaggcagatccatctacttcctcatc
tacttcgtggcctttttttctctgtctccttatgttctctgaatcctggctctgcctgagaacgcg
tcttc

>hg19_ct_usertrack_3545_274 range=chr3:139847711-139935110 5'pad=03'pad=0
strand=+ repeatmasking=none_360
tgtgttaagccttccccagtacaccccctccctatccaagttgacacatcaccaaaggaccttga
tagcataaccttgaaactgcttgttcccctaggagtctcctctcccaaaggggcatcttgaagtc
agggaggttgacctcaatcctagcacagaacagaactgtctgttaaatacaaatctacataaatg
agcat

>hg19_ct_usertrack_3545_73 range=chr16:33141100-33205499 5'pad=03'pad=0
strand=+ repeatmasking=none_209
gttgccctgctacactcccatcactaacatagtaaaaataatgatcaataaatactgagggaact

cagaggccagcgctggtgcgagtcctctgtatgctgagcaccggtcccctgggtccactgttctt
tctctatactttgtttcagtgtcttatttcttttctcagtctctcctcccacctgacgagaaata
cccac

>hg19_ct_usertrack_3545_1587 range=chr2:172366755-172372354 5'pad=03'pad=0
strand=+ repeatmasking=none_24
tggggcccccaaatcactcagctaaagggaaaagtcaagctgggaactgcttagggcaaacctgc
ctctctattctatccagtcatccctctgctcactgagataaatgcatatctgattgcctcctttg
aaaaggctaatcagaaactttaaaaaatgcaaatttctataatctggaagcccctccccattttt
agttg

>hg19_ct_usertrack_3545_583 range=chr7:26721876-26725875 5'pad=03'pad=0
strand=+ repeatmasking=none_4
gagtataatagctatatgccactcactcctataatctgaacttgcaagtgaaatctaatcaacgt
tttagttttatacacaattgcagctaaattggttttaaaaagactgtgagctatataaatttagg
aatgattccaagaaagtttcttgacaggagctgaaactaatcagtttgatttctttattaagtag
ttaac

>hg19_ct_usertrack_3545_10 range=chr16:25850500-26383699 5'pad=03'pad=0
strand=+ repeatmasking=none_598
acggagcttctcctcatggctggtgttggctcctcacagcatgctggtttcagggtcatcagaag
tcttacaaggtggccgacttccctcaaggcacaaaagcagatggtgccaagccttcaaaatgtgt
gatcactgacatcccagaatgtcacgttcaccacattgtatcgtaaccataataagttcattgca
caatg

>hg19_ct_usertrack_3545_519 range=chr10:38946395-39047594 5'pad=03'pad=0
strand=+ repeatmasking=none_98
atcctgtgtagaaatgcctttggtgctcctgccagaatccatttaccaggtcaatgaacacatct
cccagtgcttcttgttggctgctattacctgcaggtttctagaacccttgtcctgctgacaagca
cctacttgggaatactagggaggttgtgcacccctctattttccctccagacagcatctgccaat
gacgg

>hg19_ct_usertrack_3545_109 range=chr6:50160842-50226841 5'pad=03'pad=0
strand=+ repeatmasking=none_170
cctgcctttttttgttctccatttgcttggtagatcttcctccatccctttattttgagcctaca
tgtgtctctgcacatgagatgggtttcctgaatacagcacactgatgggtcttgaatctttatcc
aatttgcgagtctgtgtcttttaattggagcatttagcccatttacatttaaagttaatattgtt
atgtg

>hg19_ct_usertrack_3545_148 range=chr21:26743530-26758129 5'pad=03'pad=0
strand=+ repeatmasking=none_33
tttgtctgtggactagtttaaccagtgggttttatacttttttgaaaagttttcttctgcacatta
gtgtatttttctttcagatcgaagaacacattttaccatttcttgtaagatgggtatgctggtga
cacattctctcggcttttgtttgtctgggaaagactttatctctcctttatatttgaaggatagt
tttgt

>hg19_ct_usertrack_3545_71 range=chr10:26047195-26164394 5'pad=03'pad=0
strand=+ repeatmasking=none_336
tgcccagggccttcatacacattaactaatttaatccacaaacaatccgatcaggtaggtaccgt
aattaatgggtgccataattatccccattattaactagttccagaaatataaggtataagtcagg
ggtctaactttttttctcaaatgaaatgatcatttgtttcagcacactttattaaataatggttt
atttt

>hg19_ct_usertrack_3545_1536 range=chr2:165336355-165352154 5'pad=03'pad=0
strand=+ repeatmasking=none_2
tatcagagggacaaactcttcagaaagaatgccttttgtatgaaattttatattcttaaaacttc
atcatcaaatcaattaaaacttatttacaaaaggaaaaatcatttctccaaatcttccagaccat
gcatttaagatagaaataatttatgattctttataaataaaaatcagatgcccggaaaaactaag
atcaa

>hg19_ct_usertrack_3545_9 range=chr13:54983200-56951599 5'pad=03'pad=0 strand=+
repeatmasking=none_2890
atgtttgtgatatatgtatgtgtgtatgtgtatatacacatacatcttaggacttgtgaatcatc

ttagggcttcctacctgtaggcagaaagaaatgataactcagtaaggcatattatcctaaagtaa
tactatacgtgtgtatatcaatgaatttatacatctattatggtgtgtagcacagtttaaattcc
ttgat

>hg19_ct_usertrack_3545_1177 range=chr6:117602508-117638507 5'pad=03'pad=0
strand=+ repeatmasking=none_107
tggacaagtcattctaccttcttgacaatgctgggtctcccatgtcaaagtgagggtcctccact
caatgatctctttgtaattctcccaaactaggggtgactgagcagttggctttggtggtacccat
ctcttctaattgaagcctacagaggaggtttccaaacccagatatccagtatcttcaccctcccg
cctct

>hg19_ct_usertrack_3545_405 range=chrx:108116545-108297144 5'pad=03'pad=0
strand=+ repeatmasking=none_11
acatgaactcccctgaaggagtgaggacaaaagggaatagacagggggcaagaactgaactttgg
gagacagtcagagggaataagtgggaggaaggagagctggaacagccaaagacagagacatttaa
aaaaacacacacacaaaaggagaagcaatgtgacagaagcctactgacttttggactaattcatt
ggtcc

>hg19_ct_usertrack_3545_44 range=chr12:7708734-77413335'pad=03'pad=0 strand=+
repeatmasking=none_89
cctagtaagtaccacagtcaatgccactagcttcagatatatacctttgttgctacatttattta
aatatttttcagcaattttcttttattacatttacatgagtccaaacagaaaaaaaattgatgat
actgtcaatgtgtataggtctcataaaattacagttaagacaacacacacattaattgtgccgaa
aatga

>hg19_ct_usertrack_3545_900 range=chr14:107173356-107183155 5'pad=03'pad=0
strand=+ repeatmasking=none_19
tatctgtttcattttgcattcctgttagcaatgttttagcctctagcaacctggtatgctcacca
gcattgatgttatctgtatttcttcttaatttcagccattttaaaaagtgtacagtggtgtctct
ttgcgggcttgatttgaatttctctaacggaaaatcctgttgagatcctgtttatatgcttcagt
gtcat

>hg19_ct_usertrack_3545_772 range=chr9:75815981-75884380 5'pad=03'pad=0
strand=+ repeatmasking=none_153
gattctcttccctagtagtgtcttgaaaacatttgatttttatttgctttctctcttgaatcatg
gttttcccggaattattctagtttttaacagctaaaaactaataagaaattgcaactaactggca
gcctactggcatatttttttccagttggtagaattgtggctagtgctaaaacaaagaagcaagcga
ttctc

>hg19_ct_usertrack_3545_2168 range=chr2:239748454-239750253 5'pad=03'pad=0
strand=+ repeatmasking=none_7
cgtagtggctcacacctgtaatcccagcaccttgggggccgaggcaaatggattacttgaggtc
aggagtttgagaccagtctggccaacatggtgaaaccccatctctactaaagatacaaaaattag
ctgggcatggtggtgagcatgtgtgatcctagctactcaggaggctgaggcaggagaattgcttg
aaccc

>hg19_ct_usertrack_3545_208 range=chr6:103098308-103297707 5'pad=03'pad=0
strand=+ repeatmasking=none_479
tgatgtgaattcctaagatgtattctttttttaaaaatgtgaagataattgaaacatacaaggaat
gtaggcacaatgaaacaaagctatttaaaaattaacctagaacctacaatcaaaaagcaaaacaa
agcaaaaccaaaccaaaaaagccattgctctagtggttgctgtggtgtcctgtcaggaatctccc
ttcag

>hg19_ct_usertrack_3545_95 range=chr16:60011300-60193699 5'pad=03'pad=0
strand=+ repeatmasking=none_336
gcaaaatgcatggctgaaagtggtatgagatatctgataagtaggaaagtaactttagatctaat
tatggatggccttgaatgctagatgaaacgttctggacttcattcactaggttacagtgaactga
aatgtggaatgctgtgaccctctggcagcagcgtgaatcatgcattgaagaggaatcagagatag
gaaca

>hg19_ct_usertrack_3545_53 range=chr2:185476556-185694555 5'pad=03'pad=0
strand=+ repeatmasking=none_624
gcagcaaaccaccacggcacacgtatacctatgtaagaaacctgcacgttctgcacatgtatccc

gaacttaaattaaaaaaagaaaaaggaaaaaagaaaatatgtgttctagtttatttgtttgtttt
taacttaacagtggttaattcgttttctaattaggtgatctcatttataaataataagtttttc
tcttc

>hg19_ct_usertrack_3545_1569 range=chr7:138134661-138144260 5'pad=03'pad=0
strand=+ repeatmasking=none_19
gcagtgagggaaagaaatatctcctataattatctgtcctgagccttccatctctctctctctct
tttttttttttttttgagacagagtcttgctctgtctcccaaattggagcgcaatggcacgatct
cggctcactgcaatctccacctcccaggttcaagcgattctcgtgcctcagcctcccgagtagct
gggat

>hg19_ct_usertrack_3545_296 range=chr18:18775603-18818002 5'pad=03'pad=0
strand=+ repeatmasking=none_58
aagccatataggaaactataattatttagtcttttctgtatgactctttattttctctagtatta
attattgtggaatgtctttcatttgccttgttatctatatataaatatttttttcactaagtcaac
ccccaaatctccactaattgtctaaatctcttaatacattcacatgtatcaggtgtttcatcaat
ttaat

>hg19_ct_usertrack_3545_225 range=chr20:17103401-17165400 5'pad=03'pad=0
strand=+ repeatmasking=none_232
atagagaggccttctctaaacttctaacacaaaataatcccctgccggcactctctaggcacata
gcctgctttttttattcttagcacttaacactacctgatgttcaggcatcttctttatttgttat
ctgtctcctcccatgaaattttagcctttacgagagaagagactgtttttgttcactgctatttc
tccag

>hg19_ct_usertrack_3545_680 range=chr7:35230276-35248275 5'pad=03'pad=0
strand=+ repeatmasking=none_75
tcatagtaaatttggtgttgtagaaagcttatatacccacatagatgaaggcattttataatata
tagtatgactatgactatgaggacttaaggatatgcctaaaaggaaaattaactctgcaactatt
taagttacatctctattctcaaattattttagagtaagaataaagtaccacctcttatagtcaaa
ataat

>hg19_ct_usertrack_3545_1724 range=chrx:130812120-130902719 5'pad=03'pad=0
strand=+ repeatmasking=none_287
agactttatgggctgtgtgtggttgtgtttgtgtgtgtctctgagagttttataatgtcttttag
gtataagaattaactagtaaattaaagaataatgcttgacaaagattaagaaaaatccatgtatt
aataatgaagaaaaccactaccatacaactagtcaaggcaacttaagaacttttggattatttcc
aggaa

>hg19_ct_usertrack_3545_1080 range=chr9:111524580-111535979 5'pad=03'pad=0
strand=+ repeatmasking=none_12
tataatacatataacatacaaaatatgtgctgacggtttatactgccaggaaggcttccagtcaa
cagtaggctactggcagttaaatttggggagagtcaaaagttgtacatggattttcgactacacg
ggagacagcacccccctaaatcccatgtgttcaaggatcaactgtaatacctttatgccactctag
atctg

>hg19_ct_usertrack_3545_1849 range=chr2:202423756-202483755 5'pad=03'pad=0
strand=+ repeatmasking=none_89
gagaggggttacttaaacataattctatatgataagctgagtcccgataggactgagtacttctc
gcatctctcagggatggctggatcttttaaaccacgttaacctttggtatccctgctcaatatct
atagtcatctctgctgtaatgagtaggtccacaattttccagaaactttttgagtgccaaaatga
cacag

>hg19_ct_usertrack_3545_47 range=chrx:87868745-88604944 5'pad=03'pad=0 strand=+
repeatmasking=none_2314
atcctttaatattacatgaaaatcttgttcaagagagaaagccaaatttcatccttgcattattg
tactattaatgtcaaccccaatctttaataaaaccttataggcaaatccatcaatcttaatcagt
ttgaccatgaggtgaaattttaataaactttttataaccctttacaagtttttgtaaagagcaga
tcagt

>hg19_ct_usertrack_3545_16 range=chr16:62795100-63031699 5'pad=03'pad=0
strand=+ repeatmasking=none_483
acatttattcatgtgcctatatttcattaatgtccctcaccttcattagggtgttgcatgtggac

agaaactgtatctttgtttttgtccccattgtatttccagagccaagaaaaggaccaggcaagta
gtgagatttgactgacaggtttaaatagatggacaaataaaggaagcatatgattttcaacagac
gataa

>hg19_ct_usertrack_3545_290 range=chr8:111381825-111667824 5'pad=03'pad=0
strand=+ repeatmasking=none_1152
tgcatatgtttatgaatatgtttatgaatgtaaaattcaaaaacatagttgaatatatacaaatg
aaatacacatatgtatatatgtgtatttcacatatatacatatacacacatatacatatatacat
atatacatacatatacacatatataaacatacatatacacatacatattacacgtatgtatatat
gtgta

>hg19_ct_usertrack_3545_1176 range=chr7:90104865-90121464 5'pad=03'pad=0
strand=+ repeatmasking=none_58
ttggatgcatgggtctgacaaaggagatctcaataagttaccaaaggcatctactacaaataata
ataataatggttattatttattgaatacttattgagttaatactaaaaacttcctgggattgtgg
ttattatacttggacaccacttttatttttctctttccaaatatagtgtgtttggtgtagtgaag
aaaaa

>hg19_ct_usertrack_3545_8 range=chr10:9973395-106649945'pad=03'pad=0 strand=+
repeatmasking=none_1139
tgacccaattatgagtaaccaatagcccatgatacagacacaggatgctgtgaacatgtgtccca
gatggtcgggcacatgttcacagctatgaatgtataaatgtataaagctttggtttatacatttt
aggggggattcaagacatcaaccaacacatgtaagatgtacttgggttcagtctggaaaggtggga
caatt

>hg19_ct_usertrack_3545_2 range=chr7:108829165-109291564 5'pad=03'pad=0
strand=+ repeatmasking=none_54
gtcatcaaataatttcttgtgggaatagtttacacagatatttcatatagaaactatttgaaaca
aaaccataacatttttatcatatgtaaagtaacaataattccttagtatcatcaaatatgagtac
tcatattcccctgaatatcttgtaacaattttgtataatttcttttaatttgaattcaaataata
tttat

>hg19_ct_usertrack_3545_989 range=chr3:90117311-90140710 5'pad=03'pad=0
strand=+ repeatmasking=none_46
ctatagattgttttaatctattcattatactcaggaaaacatttatatattttttttctttatcc
tgtgttaacttgagaaaatcatcacttgccacttggccacagaagccatatgctcataacaactt
aggccaccaatatctataaaataatttcaggttattgcaaaaatcctatcagtgtgagaagtgga
ttcca

>hg19_ct_usertrack_3545_125 range=chr13:79553000-79630799 5'pad=03'pad=0
strand=+ repeatmasking=none_348
agtgctaggatggtgatactcacagggttctgtgggtgcatatacgacaaatctttagggagacg
acacacagctgagacttagaagagggagagataaaggaggggaacactcaccaatgagtctggag
aagcaggcgagcaccacatcataaaaggcctttcacatcatgccaaggactgaatcctctctgca
cttag

>hg19_ct_usertrack_3545_127 range=chr15:98298797-98442196 5'pad=03'pad=0
strand=+ repeatmasking=none_112
tagtcaggaaggtgaaagatctttgcaatgacgattacaaaacactgctcaaagaaatcagagat
gacataaacaaggagaaaaacattccatgctcatagatagtaggaacaaatattgttaaaatggc
catactgttcaaagcaatttacagattcaatgctatccctctcaaactaccaatggcattcttca
tggaa

>hg19_ct_usertrack_3545_133 range=chr8:36265645-36392642 5'pad=03'pad=0
strand=+ repeatmasking=none_103
taccgcagtcgacatcgccctctaacatctggctcttcactatattttaacttcattatctgaca
cacaatctcactctctttgtcccagtcacacaggaatttttgtagtttcttgaatatgtctgttt
tttcactttctgttctattttcctatggtttcccttcagctatctacatccatacggatcacttc
ctcct

>hg19_ct_usertrack_3545_3range=chr4:33402806-336376055'pad=03'pad=0 strand=+
repeatmasking=none_62
aaattcaagctattcaattcctcaaggcacagggactatagtgaaagaggcgggtgtgtaacatg

gtaagggccaattttgaaagataaaataagtttgatttccctataaattaatcattaatgtcaaa
ggcaaaccgatgcaaggccagcatatgggcctctgtgttagattaacaaggttttcctaaagtat
taact

>hg19_ct_usertrack_3545_22 range=chr13:68878800-69367599 5'pad=03'pad=0
strand=+ repeatmasking=none_2080
atattatacaattttttgatatttcctcttatactttaaaacttaaatagtatatatgttccat
ttgagctatataatttgaaagaataattccattttaaaataagactattctaataccacctaat
aaaaagtccatctttccttttgacttgtttttgaaatgaactttataaatagctacggcagctt
cataa

>hg19_ct_usertrack_3545_171 range=chr1:99732213-99791812 5'pad=03'pad=0
strand=+ repeatmasking=none_146
agacagccaaatgaacaatttcataaagctatgctagagggaaggataagatgctaagggagcca
catagacggagatgtgattcaatttagggtacagtaagaggtttcaaagaaaacttactgaggga
aatcatgcctaaatcatactgagttccttcattgatctcacaggtcctaaaatcctgcagcaaag
tgcca

>hg19_ct_usertrack_3545_474 range=chr3:22605997-22661796 5'pad=03'pad=0
strand=+ repeatmasking=none_196
tattcactagaacagtgtagtaatctcaagtatccttccccagccctccttattttaccattcca
taccctaactatttccttcatcctccattaggatctgccctttgacacatctaatggattgcttc
atgtccaaacatttttctcattcatccttgcatcacaattcatgttgtcttcctcattcatttgg
atttc

>hg19_ct_usertrack_3545_74 range=chr4:179197207-179501806 5'pad=03'pad=0
strand=+ repeatmasking=none_906
tgatttgcttttgagtctgtttcaatcaagatgcctctttcagaaaaaattgtctacttggcttt
attagagtcaagctctaaaacaaagaccaccaggcttatgaaaaactaagggtgtatcttatggg
tgtatatttatctctagaaattctcagttctaagggagaagattttgtgacattttaagactatc
tcaga

>hg19_ct_usertrack_3545_213 range=chr15:25454308-25513307 5'pad=03'pad=0
strand=+ repeatmasking=none_20
gcgggttggtggccatggcccatgcccagtggtcacagggcaaccctgggatcatgcaggggagc
tggtgagggcagtggaggtgctctccggaacggaagtatggctcagcccaggtatctggcccaaa
cccaatgaccagcctgccttgtttctcagggtttggtgatggggagggccaagggtgccctgcat
ctgtg

>hg19_ct_usertrack_3545_846 range=chr6:63914842-63955841 5'pad=03'pad=0
strand=+ repeatmasking=none_33
gctcggcgtcggccgccgccatgggagtgcacgtggaaaccatctccccaggagactggcgcacc
ttcccgaagcgcagccagacctgcgtgatgcactacaccgggatgcttgaagatggaaagaaatt
tgattcctcccgggacagaaacaagccctttaagtttatgctaggcaagcaggaggtgatccgag
gctgg

>hg19_ct_usertrack_3545_177 range=chr3:85625711-85946310 5'pad=03'pad=0
strand=+ repeatmasking=none_945
caatttcctctaattcctttatatgggttctaatatcagagatgctctttatttctaactcagat
tttagctattcaatcacattttaatttccttgcatttctttttacttcatacatttttatcat
gtatttcttatttattatttttacttgatttttcatttctctcctcctatttcattgagtccac
ctatc

>hg19_ct_usertrack_3545_197 range=chr1:106250478-106564677 5'pad=03'pad=0
strand=+ repeatmasking=none_233
agttaaaaaaggctctaatagtttatttgcttggttaggtgaaatatggattaaaagagagccac
tgtgagtgagctagaaatgccttggtttcatgtagaggaatcattcataattcagttgttaacaa
atactactatagctattataattatcaatttaatatagggaattcaattattaataaattgttgg
agggt

>hg19_ct_usertrack_3545_132 range=chr18:58756221-58964620 5'pad=03'pad=0
strand=+ repeatmasking=none_18
tggtcctggtcctttaccattttggagtcacagattagattcttaagaatctgacaaacccacag

aattcaataatgtttaaacaaggccacagcaacaacaagaccacagcagcaacaacacaattaat
gtagtctcagggggttcatcggtcataggtttaaacatcatgctctagagtttattggccattaa
acact

>hg19_ct_usertrack_3545_99 range=chrx:3881001-40140005'pad=03'pad=0 strand=+
repeatmasking=none_545
ttggtgattaagatcttgtgaagaatgacgcagctgtttgctaggaagaagtgatacaattgcaa
ccattgctggtgcaaaatgtacatgccaccatgcatgtactaaaaatgtggctttttcttgtgtg
ctggaaaattactataaaaatttaacattttaagtgttttgcaacatctttgatactttaattgc
aactt

>hg19_ct_usertrack_3545_46 range=chr3:161278507-161569706 5'pad=03'pad=0
strand=+ repeatmasking=none_276
gaaatagaaccagtagaagactaatattagaaaatgtatgtcaaagatttggctcatgtggttgt
ggaggctggctagacacatcttaaatctgcaggacaggcagtcaagaagagcatgctagcaagct
tggacattagttgaagatactatccataagcagaatttttttcttcttcaggggaagcctcatttc
tgttc

>hg19_ct_usertrack_3545_650 range=chr19:28733561-28780760 5'pad=03'pad=0
strand=+ repeatmasking=none_61
ttcaaacagagagagagtaaaatcttcctcatcagagtgccctggaatggaggagcctgtgcacccc
tgggccaagaaactaacacccatctgcccatccttcttttacactagctccaccccaacctagga
gatgctccaagtctaaagatgtcttcatcatacttcctccccatcccaagagtcctccttttctc
cagag

>hg19_ct_usertrack_3545_89 range=chr15:61459909-61487308 5'pad=03'pad=0
strand=+ repeatmasking=none_69
aatgtgacaatctgggatgcttatgggacgtcaaagagggtttgaaattcatgctctgaaattaa
tcagactttgttgccaccttttcagtgacatgcccatcagcaaagaacaagcattgtcattagca
gaacaaagaaaaaaaaagtgtatgcctaggattagctttggcaaaaaaaaaaaaaaaaaaaaaagg
ataaa

>hg19_ct_usertrack_3545_75 range=chr5:18954444-19638843 5'pad=03'pad=0 strand=+
repeatmasking=none_3308
ctgaagggcatctacttgcttccaagtttcagtgattagaaagaaaactgcataaacatctacat
atgggtttttgtataaacacaaattttcaactcatttgggtaaacacaaataagtgtggtttctg
gattgtataatgaatttatctgaaattgtgaacagtgttgaaataagttaggggaaaatggcaaa
cattt

>hg19_ct_usertrack_3545_23 range=chr2:77310893-78182092 5'pad=03'pad=0 strand=+
repeatmasking=none_3069
catagtcatataccccacagtagaatctgcacaaaataatttggctttttttagaaacagagttta
taaagcattgcaaaattcaagaacctatagaatatacaataccaagagtgaagactaatgtaaaa
tatggactttgggtgatgatgtgtcaacttagactcattagttgtaacaaacgtaccactctagt
gggag

>hg19_ct_usertrack_3545_938 range=chr8:73166047-73195046 5'pad=03'pad=0
strand=+ repeatmasking=none_126
cacagcaacactatttaaaatagccaagaaatggaagcaactcaagttttatttcaagatgaata
aaaagagtgtgtgtgtgtgtgtgtgtgtgtgtgtgtgtgtgtgtgtgtgtgtatgcaatggaata
ttattcaacttcagaaaacaaaaatattctggcacatgctacaacatggatgaaatttgaagaca
ctatg

>hg19_ct_usertrack_3545_22 range=chr13:68878800-69367599 5'pad=03'pad=0
strand=+ repeatmasking=none_1869
aggcagatattaaaatataattgaaattctggatttttaaattattcagaaattaataatacat
ttatttttactatatattttaatattgaatcttccttgaattaaatttattagtgatattaccac
aatgcatttgatagttattggaataatttccctttttattcttatatctaccacatactcatgatt
atgat

>hg19_ct_usertrack_3545_1355 range=chrx:80259545-80298944 5'pad=03'pad=0
strand=+ repeatmasking=none_105
agagtaaataaacaacctgcagaatgggagaaaatgcaaactatgcatgtgagaaaggtctaata

ccttgaatttattattatttttaagcaaatcaaggaaaaaaacaaacttctttattaaaaagtgg
gcaaaggacatgaccagacacttttccaaagaatgtatacatgtagtcaagaagtatatgaaaaa
atatt

>hg19_ct_usertrack_3545_89 range=chr18:38197403-38515402 5'pad=03'pad=0
strand=+ repeatmasking=none_1402
cgcacatgtagaatggatcaaggagcagaaagtttaactggcaagaaagaagagaacagctcccc
catactgggggaggagggctccaaacagaataaccccacttgtaacagaaagcagtaggttatat
tgagagtcttaaggaggtggtgtctgatttacatagggcccaggggactggtttgaccaggtgtg
tcatt

>hg19_ct_usertrack_3545_235 range=chr11:101034391-101299590 5'pad=03'pad=0
strand=+ repeatmasking=none_231
tatctcaattataaaggcaagttcagctttacacatttgtatggtaagttgaagattcaggtaga
ggtattaagatgtgtggcatttacttaaacagtcacattttccttctacgccccccttgcctgt
aaaacagctacaaaatttcactggtgtcatccatccttaagtttcagaccgccctccctccttca
tggac

>hg19_ct_usertrack_3545_972 range=chr3:87448911-87553110 5'pad=03'pad=0
strand=+ repeatmasking=none_279
tgatttgctagtattttgttaaggattttgggtctatgttcatcaggtgtattggcttgtagtt
ttcttttcctcactctgtctttgccagctttgccagggcgatgctggctttgtagaatgagttag
gcaggagtccctcccattcgatttttggaataattttagtagaattggtactagctcttccttg
tatgt

>hg19_ct_usertrack_3545_324 range=chr2:185050356-185204155 5'pad=03'pad=0
strand=+ repeatmasking=none_133
acatctatttatgtattttgcctacttttggatggggttattttatttatttatttttttcctggt
gatttgtttaaattccttatagattctggatactagtcctttgttcgatacatagcttgcaaata
ttttcctccattctgtagggtgtactttcatttggttgattattttttttcattgtacagaagctt
tttag

>hg19_ct_usertrack_3545_278 range=chr6:155851709-156063708 5'pad=03'pad=0
strand=+ repeatmasking=none_924
tattatgtgatttaaaaaggactcagaaaggtaagtgagtttaattaatgtcatacaactcgtaa
gtggcaacatatggatataaaatgaaaaattcctaatttgacagtcattgtcttccctttagatt
gcgccaccttcttctgtggacaaatagattccaaatctcctggcttctggttctttgtggcacac
tttag

>hg19_ct_usertrack_3545_344 range=chr1:247650778-247739777 5'pad=03'pad=0
strand=+ repeatmasking=none_422
atttcttggaggctttgtttgttccttttcattttttttctctaacattgtcttcatgctttat
ttcattaagttgatcttctatgtctgacatcctttcttctgcccgatcaatttggctattgatac
ttgtgtatgcttcacgaagttttcgtgctatgtttttcagctccatcaggtcatttatgtttttc
tctaa

>hg19_ct_usertrack_3545_166 range=chr7:88464865-88939064 5'pad=03'pad=0
strand=+ repeatmasking=none_1350
cactgacaccaactgggtaggggaaaactatctcaacccatgtttgcctccactctcacacaact
ataaccatcatcaacacagaagacttatgtaaccaaaggtacggggctttcgtctcacacagcaa
gcaacacacacgtgttgggtgttccccaattcagttttgtcagtatttacctggaaatagtttct
gatct

>hg19_ct_usertrack_3545_64 range=chr5:10988401-11182800 5'pad=03'pad=0 strand=+
repeatmasking=none_75
tagtacttacattcacaagcaagactacttcgctggggacctatttttcagatgaaagtcaaaac
agaaatcattgccttaggtaaccagctaagacattaatgctcacgtatcagaagcctgattgttc
aaaattgtaccagttttccagtcatttttgagtggaagaaacctattaatgtaatcacattaagc
agaga

>hg19_ct_usertrack_3545_371 range=chrx:94086745-94260744 5'pad=03'pad=0
strand=+ repeatmasking=none_762
cctagggtcttgagagaacataggtggcagtcaggtagtggatatatgccttaggtgagttagat

agtggttataggccttgggtaagacctagtgctgtgctggcttcaggtctgacccagtgcagtcc
cagtggtggtggccacaggggtctttgtattaccacacccccaattccaggtgactcagcacaga
gagag

>hg19_ct_usertrack_3545_32 range=chr12:99492470-100013669 5'pad=03'pad=0
strand=+ repeatmasking=none_2004
agttaacactccttcatcaaggggaggcaagaattaaattccctccacttaaatgtaggcaggct
tttgactacaatggaagtgatgttgccttctgatgtctcattttcttagaatgtttgcttagaat
cccaacacagaccacgagaaagtccaagtaggccatggcagcgatatgagtatggatcttgagaa
taaaa

>hg19_ct_usertrack_3545_839 range=chr6:58288442-58296841 5'pad=03'pad=0
strand=+ repeatmasking=none_1
cgtgagccatcgcaaatacaaatattcattaaacgtataggctgtctacttactctatgaccttt
ctacctagactaaagccaaaaaactctgacaccctaaaagattcataaattatttactacataag
gtttagatgcatcattaccaatattgcacatagaaatttaatgttgttagttgagcaaacagatg
ccatg

>hg19_ct_usertrack_3545_715 range=chr5:50223244-50254643 5'pad=03'pad=0
strand=+ repeatmasking=none_131
gtcctctcctctcctctccccttctcttctttctttttgagtcagggtctcgctctgtcaccctg
gctggagtccagtggcctgatcatgtcttacgtcgaaccctggttgatcatgatcttctaagaat
ggtcttattcaactttctttccattttccccattcctcaagcacactaagaaaaggctgtattac
atgga

>hg19_ct_usertrack_3545_36 range=chr15:26874508-27016307 5'pad=03'pad=0
strand=+ repeatmasking=none_697
cacaaaacaaaattttctttgacaagtatcaggtcaactaatttttaaaaaaaaagaatgtacga
aaagaaacattaaaagatttagaaaaaaattggaaatgcataactccactatccatgaagcaagc
tatggcaaaaccaatgctaaatattaaaacattcaaatcccttcctagattgtttccacaaaaca
ccaaa

>hg19_ct_usertrack_3545_295 range=chr4:107820152-107954551 5'pad=03'pad=0
strand=+ repeatmasking=none_553
gctgggaagggtagagcaaaggaaaaatgaaatttttatttcagaataaatttatgaaatcatact
tcaacatgggaacttgcagctgggggagggcaaagggacagcaatatttttcaagtaagtcactt
taataaaatacacaagccctcactttgacacagcattggaagggacattcttgaacaagtatcaa
agtgg

>hg19_ct_usertrack_3545_34 range=chr21:21332130-21815929 5'pad=03'pad=0
strand=+ repeatmasking=none_555
ttattaaaatttatgatcaattactgattattttaagaatgtggaaatagaagttcatccaaag
aaatactccaccaacaataccaatagtcatttagccaatagctaaggattctcttataacaaata
atttacagggactctttacatttgcaaaactgacaatagctatcttctgttagtttttatattaa
agttt

>hg19_ct_usertrack_3545_181 range=chr1:103436413-103571812 5'pad=03'pad=0
strand=+ repeatmasking=none_520
gtgtattttaaattataaaaatgagctaaaatcatacaaaaaatgtaggctcatttattaagcag
ccagttaacagatgactacttactcgttacccaacagtactaggtacccaaaaggacacaagcag
cataaaatgcacagtagaattagctgtctgtgctgataaggaagtgcccatcctttgcttcactt
tgtgt

>hg19_ct_usertrack_3545_719 range=chr14:88930048-88958447 5'pad=03'pad=0
strand=+ repeatmasking=none_95
cctgcagagctcaccaaacaatactgatgggaaggaggagggaaaaacctagcatgccaggagag
tttccagaaggtacaaaggcttgaatgtgatggtgaggggatgtgggggtgctgggaggctccca
gagggccgctttaacagagtggcaaagacagaggagtgggcgggtaaccagcaagcagaagcagc
acaca

>hg19_ct_usertrack_3545_478 range=chr6:18627022-18717221 5'pad=03'pad=0
strand=+ repeatmasking=none_433
ttcaagtgtttgtataaaaatattagataattaagcaaagatagatatggaacataggaactttg

tctgcagagaggggagacacgaggagaaaggggtagaagacggaaaatggaggagaaaaaggag
gggaaatgtcaagggaagaggagcaagcaaagctgtttgagagagtggggaccaagacagatgtg
acccc

>hg19_ct_usertrack_3545_286 range=chr7:153846668-153937067 5'pad=03'pad=0
strand=+ repeatmasking=none_105
ttattatcctccgaatctgcctggggttttgatagaacctggctgccaacggcttgccctggcct
ttctgaaacctcaccatttccacaatgaaacaggacgcaaggactgtgcccctggtatgggaagg
gaaggaggagaagcttgcgtttacaagtgagatatccacagacagggaacatactacatttttt
cttct

>hg19_ct_usertrack_3545_2041 range=chr2:226535357-226600156 5'pad=03'pad=0
strand=+ repeatmasking=none_90
aacttgttctgatctcacatataataagtgataaaaaccacatcatcttatccattttattcatc
ttatactgtctcctcagaaaatagaaaatcaactttttttttgtctgtctatttttgagatggagt
cttgctctgacacccagggtggagtgcagtggcacgatcttggctcattgcaacctctgcctcct
gggct

>hg19_ct_usertrack_3545_941 range=chr6:80694882-80713081 5'pad=03'pad=0
strand=+ repeatmasking=none_63
agcagtttttagattttattttcacattgaaaatcagtcagatttgcttcagcctcaaaaagcat
gtttatgtaaaattaaatgagtgatggcaggcaactgtagtttttttttcctaaatgggaaaaggg
ttaaagatgtcactccattgttgcccagcttacattgtttctggcaagaagtctactgttcttat
gattg

>hg19_ct_usertrack_3545_1286 range=chr3:131226511-131245310 5'pad=03'pad=0
strand=+ repeatmasking=none_81
tcctgggcaacactagagaaattatttcagagcttgaagccatatttactttttaggaaaaagca
tttttgttccatcaacaaggctggagttttagaatcctgctatgttctcaggagtcacaaacag
gaatggaatggcagcctgatgcacctttatgaatttacactggggttttaaaattagaaaacact
gtaca

>hg19_ct_usertrack_3545_223 range=chr9:113871580-114061579 5'pad=03'pad=0
strand=+ repeatmasking=none_458
atcatgagtgaactcccattcacaattgcttcaaagagaataaaatacctagcaatccaacttac
aagggatgtgaaggacctcttcaaggagaactacaaaccactgctcaaggaaataaaagaggata
caaacaaatggaagaacattccatgctcatgggtaggaagaatcaatatcgtgaaaatggccata
ctgcc

>hg19_ct_usertrack_3545_1846 range=chr4:182064807-182160206 5'pad=03'pad=0
strand=+ repeatmasking=none_416
caggtctgttacacagaatagattgtacaggtgcagtttggccaattattgtaactgttttcttc
agagtcttttctgctttactcatcaagctcactaggaaacagtcattcctttttcactgtttctt
ttatacccaggcttccatatattaccttgaaatcctaaatacgttccaaggctccccccatgtcc
agctg

>hg19_ct_usertrack_3545_34 range=chr16:10008900-10125299 5'pad=03'pad=0
strand=+ repeatmasking=none_55
gccactgcacaccccaccagagcaaatattttcttccaaccttaaaataacatctacaaattaat
tcagtctgtcaaggggaactctccttgggtaattcaggaagtttttgcctttgggtaaggcactc
taggcacaaacaactcaaattctcacctgtaagttgaaccaagaaggaggtttatgatctacagc
tacat

>hg19_ct_usertrack_3545_7 range=chr16:9391700-96780995'pad=03'pad=0 strand=+
repeatmasking=none_92
ttagcctcttttataaggacactaatctcattcatgaggactccaccctcatgatgtaatcacca
cccacctccagataacctcccactaagacttaggttttaaaatatgaattttggtgtgacacaga
aatttagtccattgcagggagcaccttgagctgtagaacaacttgatggctatagaagaggcttt
tctgt

>hg19_ct_usertrack_3545_605 range=chr5:36379044-36398243 5'pad=03'pad=0
strand=+ repeatmasking=none_24
tctctgaattcacatttaagtgggcattgtgtctttatctggcaacctttttctatgggtgctgcc

acaacttcctccggggaccactgtttccctaaggaactgcccccacaggtggcctggagacctca
aaatgccacagcaccaggatgttcaatgagaaggttgccctgaacacctctcttccccttttgtt
ttact

>hg19_ct_usertrack_3545_27 range=chr3:84287111-85007510 5'pad=03'pad=0 strand=+
repeatmasking=none_2919
ttcttgcccactgcacagaattacccaatacgcagagacagcaggtgtttgcagtaaagaaagag
tttaataattacagattcagctaaatgagaacatgggagatagttctcaattctgccttcctaag
aattcagaagttaaagtttttttttagggatattttgacaggctagggaatggggaatgctgac
tggtt

>hg19_ct_usertrack_3545_64 range=chr9:10856201-11319400 5'pad=03'pad=0 strand=+
repeatmasking=none_1881
tatgtttgggattttaattatctactaggtgtaaagcctaggacctagacagaagtgcagataag
gtttgacttattccaatattaactccatgtttcctaggtgttacctagctgcaaagcaggcaaat
tgtacagctaagagttaaagtggcattttataaagcattcaggagcctaatcacttttaaattgt
ataaa

>hg19_ct_usertrack_3545_323 range=chr8:127751419-127822018 5'pad=03'pad=0
strand=+ repeatmasking=none_191
acatcctctccagcacctgttgtttcctgacgttttaatgattgccattctaactggtgtgagat
ggtatctcattgtggttttgatttgcatttctctgatggccagtgatgatgagcagccataaaaa
atgatgagttcatgtcctttgtagggacatggatgaaattggaaatcatcattctcagtaaacta
tcgca

>hg19_ct_usertrack_3545_785 range=chr3:59244761-59294360 5'pad=03'pad=0
strand=+ repeatmasking=none_143
actcttcattctttctccttcccatgccccaaccctgctcctcttctagcctttctcccctcaac
aaagggtaacctatccacccattcattgttcaagtcagtaactagatgtcatccttaattccact
ttctaccccattgtcctccctaccctcctttccaatccagaagtctgtcagctctgtctccaaaa
tactt

>hg19_ct_usertrack_3545_77 range=chr2:16643320-16668919 5'pad=03'pad=0 strand=+
repeatmasking=none_59
tgctgtgacaagagagggcagagttttgtgatattgcttttgaagacaatgtcatcaagatacaa
aagccatgggaatagtgtgtatggtgtgtgtatacaagggaaatggggaaatggtgtgggatgtg
aacacaagtgtgtgtttgtaaggggtggaaggagggagagagaaaatgaaaaccatcacgtaggg
agtgc

>hg19_ct_usertrack_3545_694 range=chr13:98227600-98241199 5'pad=03'pad=0
strand=+ repeatmasking=none_25
aatgtcagtctatctcagattctccctacctccttattctttgatttttctccataatacttatc
tgatatgctgtatattttatttctatttccctcaatagagtggcagctccctgagaccagggact
ttggctcttttattcagtcctccatccccagcaccttgaatggtgtccggcacatgttagacagg
gggac

>hg19_ct_usertrack_3545_148 range=chr7:74573665-74591264 5'pad=03'pad=0
strand=+ repeatmasking=none_10
cacatccagctatttttaaatttttttttgtagagacagaggtctccctacattgcccaggttggt
cttgaacttgtagccttgagtgatcctcccacctcagcttcccaaagtgttgggattacaggcat
gagacactgcacctgactgcaagaaagctttaaaagataaaggaatgataaaaagaaagtaatct
ttttt

>hg19_ct_usertrack_3545_203 range=chr11:88720553-88830952 5'pad=03'pad=0
strand=+ repeatmasking=none_416
catagcctgggacataaagtccacttttaagatacttggaaatttgaaaactactttctagtct
agtagagctcatctctttgtaaatatatttaaattataagaccaggtcttgtagtaattcttcat
caatacttttggaaaagatagtaaaatttttcacttacaataaaaaataggttaagtaaaatggc
agcag

>hg19_ct_usertrack_3545_915 range=chr8:70855247-70858246 5'pad=03'pad=0
strand=+ repeatmasking=none_6
gcagacatactgtcaacatatgggactgtagttactattacaatcatgcctccttttatctttga

acttttttttttttttttgtaggcagagtcccactctgtcacccaggctagagtgcaatgctgcaa
tctcggctcactgcaacctctgcctcccaggttcaagcgattcttctgcctcagccccccaagta
gctga

>hg19_ct_usertrack_3545_145 range=chr18:63533821-63698820 5'pad=03'pad=0
strand=+ repeatmasking=none_694
tttacagaaagctgattggcccattttgacagggtgctgattggtgcatttacaatcccttagct
agacataaaggttctccaagtccccaccagtttagctagataaagagtgctgattggtgcattta
caaaccttgagctacacacaaggtgctgattggtgtgtttacaaaccctgagctagacacagagt
gctga

>hg19_ct_usertrack_3545_174 range=chr5:75182445-75312044 5'pad=03'pad=0
strand=+ repeatmasking=none_383
tttcattgtactgcactgctaggctagccccaccatttctttgtcctgtatgcttcttttatttg
gttttacctccattttgcttgagtacactcacaacgaattccttagaaagagtgtatgggaggtt
aatattataagactctgcatatctaaacatgtattctggcctcccttatgattctggtttgtcct
tgtat

>hg19_ct_usertrack_3545_163 range=chrx:27638480-27701879 5'pad=03'pad=0
strand=+ repeatmasking=none_231
aactcagaaaaacctgcctgtaacacagcatatttgtttttcaatattattttgtagaatgtgct
atataagcaattgaaagattataattttttttgaattctggtttgaaagataattttgttttctat
gcttctgtgatgtaaggcaaaaaccctggacaatttgtaacagaattgttttcatcatgtgccat
ctttt

>hg19_ct_usertrack_3545_47 range=chr3:163487507-164524706 5'pad=03'pad=0
strand=+ repeatmasking=none_2874
tttactgagcctaatgggtgtcagggtcagtctaagtgaaagaaaggaggggctaggatgaaggg
tgaaaaggaataataaagacagcatgtttgagatctagaacagaataatgggttgtagagggagg
tattgaggataggagagtatatgggtttggcaccatggggtggataggcaaaacaatttggttga
taagg

>hg19_ct_usertrack_3545_635 range=chr16:62229100-62247699 5'pad=03'pad=0
strand=+ repeatmasking=none_69
ttgtgtttttatctttgaggaatcgccacactgtcttctacaatggttgaatgaatttacattcc
cactaacagtgtatgtgtttgtttttctctgcagccttgccagcatccgttattttttttttga
atagccgttctgactgctgtgagatggtatctcattgtggttttgagttgcatttccttaataat
cagtg

>hg19_ct_usertrack_3545_1309 range=chr4:103061778-103102977 5'pad=03'pad=0
strand=+ repeatmasking=none_47
agctgctcagtaggaaaagggtattcgttttacaattgaaggcttagaaagaatacaacacactt
accaatgaaaatataacacatataactagtcatgagaagatatgaaatagagacatgttcttggc
tcgaaaaccaaaacaacgtaagtgcctgactcatagtaggtgcttaataacatttgtattgagtg
aatcc

>hg19_ct_usertrack_3545_29 range=chr13:86910400-87219399 5'pad=03'pad=0
strand=+ repeatmasking=none_1366
ttactatttgagataatggatatgttcattggattgatgtgattatttggcactgtgctcataaa
tcataacatcacttgtaccccataaatatatataactttaatttattgatttaccattaaaacta
aaaattaaagagaattataaaaggaaattttatgcataggaatttttttgtcattcccagtatgag
gaaaa

>hg19_ct_usertrack_3545_102 range=chrx:5031601-51864005'pad=03'pad=0 strand=+
repeatmasking=none_347
aggatcgggaaaaataactattgggtgctaggcttaatacctaggtaatgaaataatctatacat
cgaactcccatgacacaagtttacctatgtaacaaaccagcacatgcatccctgaacttaaaagt
taaaaaaaaaaaaagtataactgaagaagactatgagaatcttggaaagtcccagaaggaaatatt
aaaga

>hg19_ct_usertrack_3545_222 range=chr10:129362611-129492810 5'pad=03'pad=0
strand=+ repeatmasking=none_288
tgtagccatttgaattttatctgggtaacgaaaaatttgcaaagcaaattaaaaatgtaaataag

attagagagggaatgtttaagctactcaagagaacaaagtgttttcatgcattcatttacataaa
aaatggataggcaaattaaaatatcttctcattattagagccacttattgcacatatttatttat
tgcct

>hg19_ct_usertrack_3545_84 range=chr10:37319795-37415994 5'pad=03'pad=0
strand=+ repeatmasking=none_303
ttggattcatatatgtagttgttttgatgaaggaaaataaatttacatcgttttaggaaaaaaat
agattcgttaaatgtcctttgtgcagctataagaaccaatgaagtaatatttaagctacattacc
tcaggagaaggtgttgaccatgtcctgaacaatccattttatagttgggaaatattaccagcatg
tatgt

>hg19_ct_usertrack_3545_492 range=chr13:53775600-53824399 5'pad=03'pad=0
strand=+ repeatmasking=none_152
ttttatgttagtggggcttatgtatttcaacaaaccagtcaagtctttctcttaagagtggaata
aatacagtatttttctcccccatgaatcatttaaacccatacatatccatgttttatttctactc
cttaaaggctctacctgtttatttaattcagagcttcttggagatgatgcaccttagaagcacca
atctt

>hg19_ct_usertrack_3545_113 range=chr7:52986107-53254906 5'pad=03'pad=0
strand=+ repeatmasking=none_288
attacaaaacctacagcttaccttattctcaagaaatattgaaacatttttcctgtaagcaagaa
aaagataaggataccattattgaaattaaatatatatatttttgatatctcaacaaattaggaaca
aaggggaatttcctagacttggtaaaaaaattacaaaaaccatacagctaacactgcaatgctga
gaaac

>hg19_ct_usertrack_3545_1790 range=chr4:171181226-171295825 5'pad=03'pad=0
strand=+ repeatmasking=none_157
ctacaatttactaaaaagctattattattgtgttgtagcagtatttcttcaagagttgaaatgat
agctggctttagtatagaaagcctgataaaactgttataaatcactagtggtttgggaaagaaga
ctagattcttatcacttatgatttctacagtttctcagagaattgcttaggacttttgagtctct
ttctg

>hg19_ct_usertrack_3545_1530 range=chr6:158635613-158641812 5'pad=03'pad=0
strand=+ repeatmasking=none_21
aataagacaatttttatgtgaattcctaatttatccatccattcactcatccattcatctgttaa
tttgtttatttaccatccattcaattattcatcacatactcactacctgctgctgggctcggcat
gggaatagggaaaaaacagtaaacatgaaacttgctctttttttctcctaaggcccaaaatgttcc
tgatg

>hg19_ct_usertrack_3545_306 range=chrx:76140007-76380809 5'pad=03'pad=0
strand=+ repeatmasking=none_1033
ttctaggacatccctgaagggcagcagtgaagggacatcttcccagtgggcagaacttcgagcag
tgtacctggttgtgcactttgcatggaagcagaaatggccagatgtgctattatatattgattca
cgggctgtagccaatggtttggctggttgataagggacttggaggaagcatgattggaaaatcgg
tgaca

>hg19_ct_usertrack_3545_449 range=chr11:16692025-16759024 5'pad=03'pad=0
strand=+ repeatmasking=none_162
gaagaaactacctaaaatatatatacatatatataattattcatatatatatataaaatccttca
tatgtatatatatataaattcttccccacacatatctttaaggaatacatttgccttgaacattt
agcaaatgaataaatgaataaaacaattattgtaagatgacaaatggtaggaaatttgaacttac
tacag

>hg19_ct_usertrack_3545_231 range=chr10:132366211-132858410 5'pad=03'pad=0
strand=+ repeatmasking=none_741
tggccaacctgtgggcttgttcttagacactccaggtcactgggcactcaggaaaggacagtggt
gtgtccccaatgtggacttggtgctttctgtgtgggcttgatgctgatgtacaagtaaggattgc
tgttccctgtccccaaagggcctgccagcagctggacaccatctttcacgaagtgttcctgtggg
cagca

>hg19_ct_usertrack_3545_287 range=chr4:105416952-105613551 5'pad=03'pad=0
strand=+ repeatmasking=none_264
attgcaagtaccctaaacgtagcttattgccttcctttcctgtttcctgacttctctgggtcgtt

tcattcgcacctttcttttaagccatctgggatcctctgtacaaccgagctttaatgctggacac
ctggtctgacaatgtttgcttctctctttcacctgccagccctttgttcctccccaagattgaat
gagcc

>hg19_ct_usertrack_3545_1074 range=chr19:53239189-53251588 5'pad=03'pad=0
strand=+ repeatmasking=none_39
cccagctacttgggaagctgaggcaggagaatcacctgaatgcaggaggcagaggttggagtgag
tcaagatcctgccattgcactccagcctgggccacagagcaagacttcatctcaaaagaaaaaaa
gaacctcaaaagatcgcaaccatttgtgcctcacttatctgtgacctggaagccccctccctgct
tcaag

>hg19_ct_usertrack_3545_57 range=chr19:22328361-22728560 5'pad=03'pad=0
strand=+ repeatmasking=none_1464
tattacaaagatgcaattgtaggggattaatgaacaggctgcttggttgaaatgagatcatttat
ctagctaattatttaatctattgaattttagttggttgttcatggggaccctggctaagaagcat
ggtttcaacattttttttttttttttctgagacaaagtcgcactctgtagtccaggctggagtgc
agtgg

>hg19_ct_usertrack_3545_13 range=chr5:23978444-24504443 5'pad=03'pad=0 strand=+
repeatmasking=none_2041
gatctccacaattgtacaaaagtaatgcaaacttatttctcaaattttgaataaacctcccaat
gaaatagagatccatgtgtcctatcattcctagttccctcaaaactttaatgaaatcactttaac
tgcctcactttaaattggaaatcttttaatgtctgtagagtctatacacccaataactgacatat
cagca

>hg19_ct_usertrack_3545_61 range=chr3:3238201-33810005'pad=03'pad=0 strand=+
repeatmasking=none_191
ggaaagactggcctagtaccttgtctacatgtttcccttacaaggttcctgacctgtggtaagta
aagaatgccatttttttgataggcccgagaacatcaagatatttggggaactcaagaagtggggaa
ctcacccagttcatgcaggtattgcatggtgaatctttggcttgactttctagtctcaaggcttt
taaaa

>hg19_ct_usertrack_3545_123 range=chr8:33857459-34313458 5'pad=03'pad=0
strand=+ repeatmasking=none_527
ttctttctttaaaatactcactgaattatactattaccatgtttctgttattccagattgaattc
ctttcttataattcttaacgaagactcctgtctaatctctctgcttttcaagtcctcagtcagaa
tctactgtagagagaactgatgtatcagacttccccactttccactttaatcatgccactgcctt
ttctt

>hg19_ct_usertrack_3545_25 range=chr16:6734400-69749995'pad=03'pad=0 strand=+
repeatmasking=none_121
aacaccttttaagccaacagctgagcgatttgctatgacaaatctgccactatgcattcacttaa
tttccaattaatcaaacattcaataaatcattagtataattacacctgttcccttcttgccctag
cccattagctcaactcaggcgtcccttcgactggctgtggggaaggtgttcatttgtctgccctg
gctgg

>hg19_ct_usertrack_3545_1682 range=chr1:176918578-176923377 5'pad=03'pad=0
strand=+ repeatmasking=none_23
aaagctcagagcagggaggctgaagtcctgccagccaagcctgctccaatccccactgccatctc
ccatggctgctccctccaatgatgtccctggcataatcctagaaacctgtgaagatatcacctca
catggcaaaagcgactttgcagatgtgattaaattaaggatcttgaaatggagacattatcctgg
attat

>hg19_ct_usertrack_3545_1824 range=chr3:192490707-192507106 5'pad=03'pad=0
strand=+ repeatmasking=none_28
atcaacacaatgtgttggtaagaatgtgagacaactggaaatctcatacattcatggtggaagta
taaagtgatacaacactttagatacctggaatgatctactatcctccgagtcaaaaattctattt
ctaggtatatacctaaaaggaacaactgcatatgtctaacaaaaaagtgtgcgaaatgtgcatgt
cagca

>hg19_ct_usertrack_3545_21 range=chr8:51375448-51762047 5'pad=03'pad=0 strand=+
repeatmasking=none_510
tgtgtatagcaatcttcttccagcgttaagaagggaaagtggaaaatatacaactttggagtgga

caaacctaacaaacgctactgcagctgggtggttaacgtcaactgtgatgtcaaattaataatat
gtacccttgatatgatgtaacaaaaatggccctttgcctctgtgattttcgtccccaaatccata
atcct

>hg19_ct_usertrack_3545_424 range=chr19:13324001-13345200 5'pad=03'pad=0
strand=+ repeatmasking=none_35
ggaggcggagattgcagtgaactgagatcacactattatactccagccttggcaacaagagtgaa
attctgtctcaagaaaaaaaaaaaaatatcaagtcagctgacctcaaaatacagagatgatcaggg
taggcctgccccaatcaggtgagcccttgaaaagcacagtattgtctctgctgggtggcagatgg
ggaag

>hg19_ct_usertrack_3545_1727 range=chr4:163863151-163959350 5'pad=03'pad=0
strand=+ repeatmasking=none_18
caaaatttccttgaaatcttcagtgagccaagtacataggtttcaagtgaatccttttaattact
gctgggctcactaaagaatatttccaccttcacgaacaggccacaccagcatttccaatactacc
tggcaccatcacaccttcacctctgcatttcagccacacagctgctttctgtctcccatatggcc
aatgc

>hg19_ct_usertrack_3545_247 range=chr11:106190591-106592190 5'pad=03'pad=0
strand=+ repeatmasking=none_672
tgacaattcagctattaattttattaaggctctcttttatgtagtaaggttttctcctactgctg
tttttaagattttctctttgtctttttgccatcttggatacgatatgtcataggcgttgatctc
tttgaatttattctatctggagttcattaaactttttgaatgtatagattcctattttctttaaa
tttag

>hg19_ct_usertrack_3545_24 range=chr1:106903278-107031677 5'pad=03'pad=0
strand=+ repeatmasking=none_155
acattcacttcagaaattagaaaataaagaaatatagaaaataaaagttgctgtctaaatatcat
cagctatggatttccctctgacagtaaactggaagcaataatgtaattgactagtttttcctctt
atgaaaccctatttctatcagaaatatgtaagagtagaaaaacaaaaaagtttaaaatctcacaa
taaca

>hg19_ct_usertrack_3545_925 range=chr3:77236311-77304310 5'pad=03'pad=0
strand=+ repeatmasking=none_217
caccaaatggaaccattgttagggcatattacttttctattagtttacttttctattagaatcat
tcaagcaaatctcaaagtttttatctgaaaattgttttgtttcaaatttctttaatgaaaatact
gtaaggttggcttccacattaactacacggaaactaaagttactagaaaagacaggttagttatt
aaatt

>hg19_ct_usertrack_3545_17 range=chr1:77592413-77609212 5'pad=03'pad=0 strand=+
repeatmasking=none_62
caaagccattctaactcaaaactctaggtttctatattagtacatgttaggccgtcctgccttcc
acattcattcggcacttgagttttagcgtctatgtgtttttttcccaagagatttccctactgttg
agcaaagtggagcttcctctgttgcaagccactgttcagcaataggctcctgcgaatactttctg
ctact

>hg19_ct_usertrack_3545_23 range=chr2:77310893-78182092 5'pad=03'pad=0 strand=+
repeatmasking=none_4005
aatgctacaataatgttatctttcaaaacttcttagaattattctggggaaaactccttcacatt
tacacagacgtttaacagttcattttaacagttcatttccacaagtctcataggtactattttac
tttcatggtatgcttattttgatcaaaggtaggtcctgtatcctacaaaacaaagacatgactga
aggaa

>hg19_ct_usertrack_3545_315 range=chr5:155550023-155696422 5'pad=03'pad=0
strand=+ repeatmasking=none_721
ttccactgtgtcacatttcatcttaagtcttcctgttgttgagactgtgaaatttacactattct
cattccaagacttctcttaaaatctctttatcacttgtttttttatttcctggtaatgtagtattt
caaagttgtttcctggttctgaaggtttcagttccaccctaattatccaaaaaagaaaaatgttg
aattt

>hg19_ct_usertrack_3545_35 range=chrx:65967276-66215075 5'pad=03'pad=0 strand=+
repeatmasking=none_674
ttcccactacccagagctccccacatcatgagactggaagcagtctgcttggcaggcagagtggc

tcaagcagatggcagcaatctgatagcatgcggaggattccagataaattagctgatgaatggaa
aagatggttgttatcctcataaagtgggggaactttatgaggaaagaggtgctaatcttcatccc
agctg

>hg19_ct_usertrack_3545_58 range=chr4:136292751-136339550 5'pad=03'pad=0
strand=+ repeatmasking=none_108
tgtataattatatttaacctgaagcaaatgtttagacaatgtatttatgctgagagtaatataaa
acatttaggacctgaattatatcttaaaatattggctactttaaacagtagcctttctttatctt
atctcttataatactgttcaagtactctttatattttattttgtttggcaatatttcgtatcatt
ttgct

>hg19_ct_usertrack_3545_2132 range=chr2:235412462-235453461 5'pad=03'pad=0
strand=+ repeatmasking=none_202
caggaatgtgaggagtcagggaggtgtgtctggggcagagggcatggcacgcaggaaggcaggga
aggatgggcccgcagggttcaggaacagcagcggggtgcagaccagagagtgcccggaaagcacg
aggaatttgcttgaaacgtaatggaagccccttttcctctgtttgctcaccctcaccttcctggaa
ggtgc

>hg19_ct_usertrack_3545_1056 range=chr7:75535865-75543264 5'pad=03'pad=0
strand=+ repeatmasking=none_3
atctccagagagagagattctgcgcttctacactttggtgatgttctcatccctgaccctgtagggt
tatctcaacccaggaaatctgcaaactcacttgccttgcaccctcatggacgcctctcaagggga
ataaaccatcctgtccaaaggtcatggtgtgatactggagacacatgagacacggtgtcagaggc
cgctg

>hg19_ct_usertrack_3545_455 range=chr14:57767448-57770447 5'pad=03'pad=0
strand=+ repeatmasking=none_7
cttatgacaatcttggaattatttttatttaatgttaaatattttctggcatttttactttaatt
atttttagctattcacccacaagtgcagtaaggctgacataattacctcctttaattcacaaaag
acatgtatttacctatttgttataatgcttcctatgctagcatgctgttactatgacaagactac
tctga

>hg19_ct_usertrack_3545_313 range=chr3:163065707-163487306 5'pad=03'pad=0
strand=+ repeatmasking=none_1595
tcttaggtttaagtccatcttcatttgattttttgtataagatgagaaaagaggatccagtttcat
tctactacatgtggctagccaattatcccagcaccatttgttgaaaagggtgtcctttctccact
ttatgtttttgtttgctttgtcaaagatcagctggctgtaagtatttgggtttacttctggatgc
tctat

>hg19_ct_usertrack_3545_1044 range=chr10:89053199-89053398 5'pad=03'pad=0
strand=+ repeatmasking=none_0
tagatttgcccttgaactgatcctccagtgcaaaatactttaattgctaacgtaaagtttcataa
aagcacataaatctcatttattatagttctaatatgaaaatctggatctctttagaaccagcttt
attaggatctcttggctgcatatgtcagaatctcaactcaaactctcttatgaaaaaaattcagc
agatg

>hg19_ct_usertrack_3545_354 range=chr18:29267803-29290802 5'pad=03'pad=0
strand=+ repeatmasking=none_94
gaaaagaaaaaaaagaacatcatgtaacttctaaagactctcttcatttccataattttatggtt
aatgttttatagcaaaatgtaacattaataaccagtaacaatgtattccagcctgatttttgttt
aagatccccagctgaaagagcttaaatagactaaatcagaaacagcaatagcagtgtcataaaga
taatt

>hg19_ct_usertrack_3545_456 range=chrx:126012720-126480719 5'pad=03'pad=0
strand=+ repeatmasking=none_1006
tagaatgtatttatgaacttctggaagacaaatatttcttaaacaaggcagaaaaaataaccata
aaagaatataatatatatttaattacatgaagaatgtaaatcaatataaagataccaataagaaa
attaaataacaactcacagagtgaaaaaagatatgcatacctctgagcaaaaaaaaaaaaagtac
tcaga

>hg19_ct_usertrack_3545_104 range=chr16:62442500-62794899 5'pad=03'pad=0
strand=+ repeatmasking=none_229
atatgccattttagcataaggattattttgagctgaaggcaatgtagaaaaaaaaaaagacacag

aagtaccctctacccttcccttatctgcctgaaagcagggcataaattttctttgtgaatgtgtt
tccctctcttgtatcagaaagggaagaatttttatcggtagagatgggaacagtaccgagatgag
actgc
